# A transcriptomic study of Williams-Beuren syndrome associated genes in mouse embryonic stem cells

**DOI:** 10.1038/s41597-019-0281-5

**Published:** 2019-11-06

**Authors:** Rossella De Cegli, Simona Iacobacci, Anthony Fedele, Andrea Ballabio, Diego di Bernardo

**Affiliations:** 1 Telethon Institute of Genetics and Medicine (TIGEM), via Campi Flegrei, 34, 80078 Pozzuoli, Naples Italy; 2 Lysosomal Diseases Research Unit, South Australian Health and Medical Research Institute, Adelaide, SA Australia; 3 Medical Genetics Unit, Department of Medical and Translational Science, Federico II University, Via Pansini 5, 80131 Naples, Italy; 4 Jan and Dan Duncan Neurological Research Institute, Houston, TX 77030 USA; 5 Department of Molecular and Human Genetics, Baylor College of Medicine, Houston, Texas 77030 USA; 6 Department of Chemical, Materials and Industrial Engineering, University of Naples ‘Federico II’, 80125 Naples, Italy

**Keywords:** Embryonic stem cells, Expression systems, Transcriptomics, Microarray analysis

## Abstract

Williams-Beuren syndrome (WBS) is a relatively rare disease caused by the deletion of 1.5 to 1.8 Mb on chromosome 7 which contains approximately 28 genes. This multisystem disorder is mainly characterized by supravalvular aortic stenosis, mental retardation, and distinctive facial features. We generated mouse embryonic stem (ES) cells clones expressing each of the 4 human WBS genes (WBSCR1, GTF2I, GTF2IRD1 and GTF2IRD2) found in the specific delated region 7q11.23 causative of the WBS. We generated at least three stable clones for each gene with stable integration in the ROSA26 locus of a tetracycline-inducible upstream of the coding sequence of the genet tagged with a 3xFLAG epitope. Three clones for each gene were transcriptionally profiled in inducing versus non-inducing conditions for a total of 24 profiles. This small collection of human WBS-ES cell clones represents a resource to facilitate the study of the function of these genes during differentiation.

## Abstract (summary)

**Table:** 

Measurement(s)	transcription profiling assay • regulation of transcription, DNA-templated
Technology Type(s)	microarray assay • gene overexpression
Factor Type(s)	WBSCR1, GTF2I, GTF2IRD1 and GTF2IRD2
Sample Characteristic - Organism	Homo sapiens

Machine-accessible metadata file describing the reported data: 10.6084/m9.figshare.10003127

## Background & Summary

Williams-Beuren Syndrome (WBS) is a neurodevelopmental disorder caused by a hemizygous deletion of 1.5 Mb segment occurring in approximately 95% of cases and a larger 1.84 Mb deletion observed in about 1 of 20 cases^1,2^. Clinical main features comprise, distinctive facial features (elfin face)^3,4^, supravalvular aortic stenosis, connective tissue anomalies, hypertension, infantile hypercalcemia^5^, dental, kidney and thyroid abnormalities, premature ageing of the skin^6^, impaired glucose tolerance and silent diabetes^2,7^. The cognitive hallmark includes mental retardation, hypersensitivity to sound due to the absence of acoustic reflexes and hypersociability^8,9^. While the primary cause of WBS is well understood^10^, we still know little about the molecular basis of the phenotype. The first genome-wide transcription study performed in primary fibroblasts from eight individuals with WBS resulted in set of candidate pathways mis-regulated in WBS possibly involved in associated phenotypes^2^.

To facilitate the study of genes involved in WBS, we generated and transcriptionally profiled of mouse embryonic stem (ES) cells^11,12^ with inducible expression of the three GTF-transcription factors (*GTF2IRD1*, *GTF2IRD2* and *GTF2I*) together with the translation initiation factor *Eif4h* (the human homolog is known as *WBSCR1*^13,14^. The ES properties to self-renew^15^ and to differentiate in the three germ layers^16,17^ have made these cells a unique *in vitro* system for studying the molecular mechanisms that regulate lineage specification. The three GTF-family members are all highly expressed in the brain. Mouse hemizygote models for *GTF2I* and *GTF2IRD1* present cognitive and behavioural phenotypes associated with WBS^3,4^, moreover *GTF2I* deletion is known to be associated with increased sociability while the *GTF2I* duplication results in increased separation anxiety^18,19^. Targeted *Gtf2IRD1* knockout mouse is known to cause the up-regulation of growth factors and other genes involved in brain development and cellular proliferation which may be linked with the extreme thickening of the epidermis observed in the mouse model^20^. Moreover it has been reported that the transgenic expression of each of the three family members in skeletal muscle causes significant fiber type shifts^21^. Finally, *WBSCR1*, the human homolog of *Eif4h*, is known to contribute to neuroanatomical WBS deficits^22^: in *vivo* studies on knockout mice displayed growth retardation, a smaller brain volume, a reduction in both the number and complexity of neurons and severe impairments of fear-related associative learning and memory formation^22^.

**Fig. 1:**
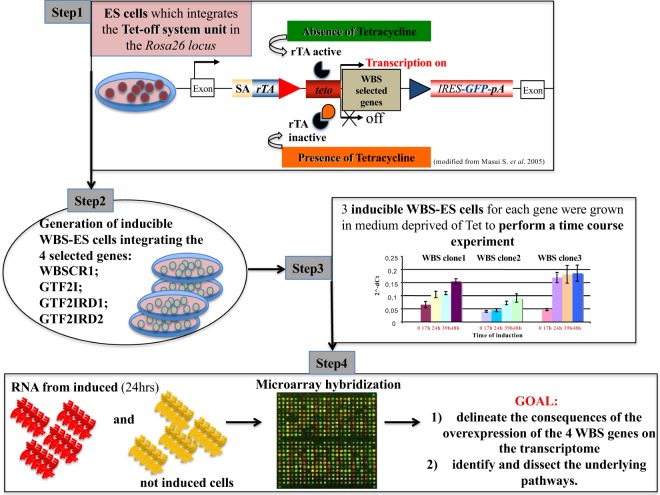
Flowchart of experimental design of this study.

In a previous study on Down Syndrome, we generated a collection of mouse ES clones capable of the inducible expression of 32 mouse genes (orthologs of human chromosome 21 genes) under the control of the tetracycline-response element (tetO)^14^. Here we used the same approach exploiting the ROSA-TET system^23^ to generate 12 mouse ES clones carrying the 4 Open Reading Frames (ORFs) of the GTF-transcription factors (*GTF2IRD1*, *GTF2IRD2* and *GTF2I*) together with the translation initiation factor *Eif4h* (Fig. 1). Three positive clones (Supplementary Fig. 1) for each gene were selected and grown in medium deprived of tetracycline (Tc) to perform an induction time course. RNA was extracted (Supplementary Fig. 2) from each clone at the time-point of maximal expression (24 hrs, Supplementary Fig. 3) and total RNA extracted from un-induced clones used as control. Total RNA was profiled by Affimetrix microarrays (the whole set of results is available in the GEO database [GSE96701^24^])^25,26^. This analysis was performed to detect differentially expressed genes (that is, in induced versus non-induced cells, Supplementary Fig. 4) in ES cells modeling the WBS.

## Methods

### Generation of recombinant WBS-ES clones

The generation of recombinant WBS-ES clones started with the modification of the cell line EBRTcH3 (EB3) as described in^14^. The cells were cultured in ES media supplemented with the leukemia inhibitory factor (LIF), at 37 °C in 5% CO_2_. The ES media contained DMEM high glucose (Invitrogen, Catalog No. 11995–065) supplemented with 15% fetal bovine serum defined (hyClone, Catalog No. SH30070.03), 0.1 mM non-essential aminoacids (Gibco-Brl, Catalog No. 11140–050), 0.1 mM 2-mercaptoethanol (Sigma, Catalog No. M6250) and 1,000 U/ml ESGRO-LIF (Millipore, Catalog No. ESG1107). The basal expression of the transgenes in each stable clone was assured by the growth of the cells in this ES media +LIF supplemented with 1 μg/ml Tetracyclin (Tc) (Sigma, Catalog No. T7660). The selection of positive recombinant clones was assured by the growth of the cells the ES media (+LIF and +Tc) supplemented with 1.5 μg/ml of Puromycin (Puro, Sigma, Catalog No. P9620). After trypsinization (Trypsin-EDTA solution 10x, Sigma, Catalog No. T4174) the cells were plated 1 day before the nucleofection on a layer of 0.1% Gelatin (Gelatin Type I from porcine skin, Sigma) in 100-mm dishes (Nunc, Catalog No. 150350) in ES media (+LIF and +Tc). For Nucleofection protocol 2 × 10^6^ cells were counted for each sample. Plasmids were prepared using Qiagen plasmid Midi-kit (Catalog No. 12145): 5–6 μg of pPthC vector in which the ORF of interest were cloned were incubated with 3 μg of pCAGGS-Cre vector^27^ and 100 μl of Mouse ES Cell Nucleofector Kit (Amaxa, Catalog No. VPH-1001) was added to the plasmid mix as described in^14^. Cells were then incubated for 15 minutes at room temperature in complete medium and then plated. The day after, the cells were washed twice with PBS (Dulbecco Phosphate buffered Saline 1x, Gibco, Catalog No. 14190), and switched to selection media (+LIF +Tc +1.5 μg/ml Puro). The colonies were grown for one week before they were individually trypsinized and transferred to 96-well U-bottom plates (Nunc, Catalog No. 163320), then each clone was equally distributed among two gelatin-coated 48-well plates for selection in “selection media” (ES media +LIF and +150 μg/ml Hygromicin B in PBS, (Invitrogen, Catalog No. 10687–010)): the clones resistant to selection media and in parallel dead in selection media were isolated, replicated in 12-well plates (Nunc, Catalog No. 150628) and then in 6-well plates (Nunc, Catalog No. 140675) to extract the genomic DNA using standard conditions.

### Cloning strategy

Each human coding sequence was cloned from the ATG to the stop codon without the 5′ and 3′ UTRs. For the 4 WBS ORFs, we cloned the longest annotated coding sequence (NM_001368300 for GTF2IRD2; NM_001199207 for GTF2IRD1; NM_032999 for GTF2I; NM_022170 for WBSCR1). The exchange vector pPthC-*Oct-3/4*was modified as described in^14^ and the epitope 3xFLAG was designed to be in frame with the stop codon of each ORF. The cDNAs were amplified using the plasmids as templates by PCR in standard conditions: the forward and reverse primers were designed to include in the sequence the restriction sites recognized by the enzymes AscI and PacI at the 5′ and 3′ ends, respectively (Supplementary File 1). After digestion with specific restriction enzymes, the cDNA fragments were cloned into pTOPO-bluntII (Invitrogen, Catalog No. K2875J10), and then the cDNAs was cleaved by AscI-PacI. The fragments obtained by digestion were separated from pTOPO-bluntII as described in^14^, the purified cDNA fragments were then inserted into the appropriately digested and purified pPthC vector^23^. The *Escherichia coli* positive clones were selected by enzymatic digestions and then sequenced by using the universal M13Fw primer and, for longer sequences, internal forward primers specific to the gene of interest.

### Induction of transgene expression

The induction of the 4 transgenes’ expression to Tc was verified on three positive clones for each WBS gene of interest. The complete removal of Tc results in sufficient induction of the Tet-off system as decribed in^28^. Cells to be induced were grown in medium deprived of Tc to perform a time course of induction (17, 24, 39 and 48 hours), by using the growth in Tc as control, time 0. Total RNA was extract at each time point of the time course and at time 0 and then 1 μg of each reverse-transcribed as described in^14^. The levels of each transcript was measured by Real-time RT-PCR experiments by using Light Cycler 480 Syber Green I Mastermix (Roche, Catalog No. 04887352001) for cDNA amplification and in LightCycler 480 II (Roche) for signal detection. RT-PCR results were analyzed using the comparative Ct method normalized against the housekeeping gene *Actin B* (refer to Supplementary File 2). All primer pair sequences used for RT-PCR are available in Supplementary File 2. For the time course of induction of the GTF2I clones (named C6, B3, A1), of the GTF2IRD1 clones (named D1, D2, D4), of the GTF2IRD2 clones (named A3, A4, A5) and of the WBSCR1 clones (named A3, A4, A1) refer to Supplementary File 3, Supplementary Fig. 3 and Online-only Table 1.

### Microarray hybridization, data processing and statistical analysis

The preparation of the RNA’ samples for the microarray hybridization on the Affymetrix GeneChip Mouse Genome 430_2 array was described in^14^. Low-level analysis was performed by robust multiarray average (RMA) implemented using the RMA function of the Affymetrix package of the Bioconductor project^29,30^ in the R programming language^31^. The low-level analysis for the BAMarray tool (v3.0) was performed using the MAS5 method as described in^14^ and implemented using the corresponding function of the same Bioconductor package. For each gene, a *t*-test was used on RMA normalized data to determine the differentially expressed genes (induced versus uninduced). *P*-value adjustment for multiple comparisons was done with the FDR of Benjamini-Hochberg^32^ (threshold FDR <0.05, refer to Supplementary File 4 and Supplementary Fig. 4).

### Accession codes

The whole set of results is available in the GEO database^25,26^ as “A transcriptomic study of Williams-Beuren syndrome associated genes in mouse embryonic stem”, SuperSerie code GSE96701^24^ (Supplementary File 4, Supplementary Fig. 4 and Online-only Table 1). The title of the SuprSeries is “Expression data from inducible ES stable cell line overexpressing the human GTF2IRD1, GTF2IRD2, WBSCR1, or GTF2I”. In details: 1) GSE95267 refers to expression data from inducible ES stable cell line overexpressing specifically the human gene GTF2IRD1; 2) GSE95268 refers to expression data from inducible ES stable cell line overexpressing specifically the human gene GTF2IRD2; 3) GSE95269 refers to expression data from inducible ES stable cell line overexpressing specifically the human gene WBSCR1; 4) GSE95270 refers to expression data from inducible ES stable cell line overexpressing specifically the human gene GTF2I Fig. 1.

## Data Records

The whole set of results is available in the GEO database^25,26^ as “A transcriptomic study of Williams-Beuren syndrome associated genes in mouse embryonic stem”, SuperSerie code GSE96701^24^.

## Technical Validation

The overexpression of the 4 selected WBS genes was based on the inducible expression by means of a tetracycline-repressible promoter (tet-off system). The first validation of the system was based on the cloning of the *luciferase* (*Luc*) into the exchange vector as described in^14^, the second was the establishment of the expression of the *YFP* reporter gene, which is separated from the *Luc* gene in the recombinant locus by an *IRES* sequence, by detecting a comparable level of the *YFP* expression and protein accumulation following induction^14^. The study of the growth properties of our mES line (EB3) compared to the parental line (E14) (data not shown) and the ability of these cells to differentiate in the three main germ layers was also performed in^14^: in details the down-regulation of the pluripotens’ marker Oct3/4 was also confermed in the EB3 as well as a farther induction of the mesodermal (Brachyury), ectodermal (Gfap) and endodermal (Afp) markers during mES differentiation. Collectively these data suggest that the system we chose allows the efficient and long-term overexpression of the transgene in a dose and time-dependent manner. It is therefore suitable for systematic expression of WBS cDNAs. The positive clones overexpressing the 4 selected WBS genes were identified by PCR using the primer pair used in previous studies^13,14^: 5′-GCATCAAGTCGCTAAAGAAGAAAG-3′ and 5′-GAGTGCTGGGGCGTCGGTTTCC-3′ (Supplementary Fig. 1).

## Supplementary information

Supplementary Files.

## Data Availability

Codes that were used for data processing are included in the Methods and available as supplementary material (Supplementary File 1 includes the sequences Asc1-Pac1 of the 4WBS ORFs; Supplementary File 2 the Primers used for RT-PCR_WBS). The whole set of results is available in the GEO database^25,26^ as “A transcriptomic study of Williams-Beuren syndrome associated genes in mouse embryonic stem”, SuperSerie code GSE96701^24^ (Supplementary File 4).

## Online-only Table

**Online-only Table 1:** Overview of samples and procedures used in the study.

Source: inducible ES stable cell line overexpressing specifically 4 human WBS genes	Protocol 1: Induction of transgene expression	Protocol 2: RNA extraction	Samples	Microarray hybridization	Data: results available in the GEO database
WBSCR1 clone A3	T0 (CTRL, in presence of Tc)	RNA extraction	Biological replicate A3	Microarray (Affymetrix GeneChip Mouse Genome 430_2 array)	GSE95269
WBSCR1 clone A3	after 24 hrs in medium deprived of Tc	RNA extraction	Biological replicate A3	Microarray (Affymetrix GeneChip Mouse Genome 430_2 array)	GSE95269
WBSCR1 clone A4	T0 (CTRL, in presence of Tc)	RNA extraction	Biological replicate A4	Microarray (Affymetrix GeneChip Mouse Genome 430_2 array)	GSE95269
WBSCR1 clone A4	after 24hrs in medium deprived of Tc	RNA extraction	Biological replicate A4	Microarray (Affymetrix GeneChip Mouse Genome 430_2 array)	GSE95269
WBSCR1 clone A1	T0 (CTRL, in presence of Tc)	RNA extraction	Biological replicate A1	Microarray (Affymetrix GeneChip Mouse Genome 430_2 array)	GSE95269
WBSCR1 clone A1	after 24 hrs in medium deprived of Tc	RNA extraction	Biological replicate A1	Microarray (Affymetrix GeneChip Mouse Genome 430_2 array)	GSE95269
GTF2i clone C6	T0 (CTRL, in presence of Tc)	RNA extraction	Biological replicate C6	Microarray (Affymetrix GeneChip Mouse Genome 430_2 array)	GSE95270
GTF2i clone C6	after 24 hrs in medium deprived of Tc	RNA extraction	Biological replicate C6	Microarray (Affymetrix GeneChip Mouse Genome 430_2 array)	GSE95270
GTF2i clone B3	T0 (CTRL, in presence of Tc)	RNA extraction	Biological replicate B3	Microarray (Affymetrix GeneChip Mouse Genome 430_2 array)	GSE95270
GTF2i clone B3	after 24 hrs in medium deprived of Tc	RNA extraction	Biological replicate B3	Microarray (Affymetrix GeneChip Mouse Genome 430_2 array)	GSE95270
GTF2i clone A1	T0 (CTRL, in presence of Tc)	RNA extraction	Biological replicate A1	Microarray (Affymetrix GeneChip Mouse Genome 430_2 array)	GSE95270
GTF2i clone A1	after 24 hrs in medium deprived of Tc	RNA extraction	Biological replicate A1	Microarray (Affymetrix GeneChip Mouse Genome 430_2 array)	GSE95270
GTF2IRD1 clone D1	T0 (CTRL, in presence of Tc)	RNA extraction	Biological replicate D1	Microarray (Affymetrix GeneChip Mouse Genome 430_2 array)	GSE95267
GTF2IRD1 clone D1	after 24 hrs in medium deprived of Tc	RNA extraction	Biological replicate D1	Microarray (Affymetrix GeneChip Mouse Genome 430_2 array)	GSE95267
GTF2IRD1 clone D2	T0 (CTRL, in presence of Tc)	RNA extraction	Biological replicate D2	Microarray (Affymetrix GeneChip Mouse Genome 430_2 array)	GSE95267
GTF2IRD1 clone D2	after 24 hrs in medium deprived of Tc	RNA extraction	Biological replicate D2	Microarray (Affymetrix GeneChip Mouse Genome 430_2 array)	GSE95267
GTF2IRD1 clone D4	T0 (CTRL, in presence of Tc)	RNA extraction	Biological replicate D4	Microarray (Affymetrix GeneChip Mouse Genome 430_2 array)	GSE95267
GTF2IRD1 clone D4	after 24 hrs in medium deprived of Tc	RNA extraction	Biological replicate D4	Microarray (Affymetrix GeneChip Mouse Genome 430_2 array)	GSE95267
GTF2IRD2 clone A3	T0 (CTRL, in presence of Tc)	RNA extraction	Biological replicate A3	Microarray (Affymetrix GeneChip Mouse Genome 430_2 array)	GSE95268
GTF2IRD2 clone A3	after 24 hrs in medium deprived of Tc	RNA extraction	Biological replicate A3	Microarray (Affymetrix GeneChip Mouse Genome 430_2 array)	GSE95268
GTF2IRD2 clone A4	T0 (CTRL, in presence of Tc)	RNA extraction	Biological replicate A4	Microarray (Affymetrix GeneChip Mouse Genome 430_2 array)	GSE95268
GTF2IRD2 clone A4	after 24 hrs in medium deprived of Tc	RNA extraction	Biological replicate A4	Microarray (Affymetrix GeneChip Mouse Genome 430_2 array)	GSE95268
GTF2IRD2 clone A5	T0 (CTRL, in presence of Tc)	RNA extraction	Biological replicate A5	Microarray (Affymetrix GeneChip Mouse Genome 430_2 array)	GSE95268
GTF2IRD2 clone A5	after 24 hrs in medium deprived of Tc	RNA extraction	Biological replicate A5	Microarray (Affymetrix GeneChip Mouse Genome 430_2 array)	GSE95268
