# Supplementary material for: A transcriptomic study of Williams-Beuren syndrome associated genes in mouse embryonic stem cells
Source: Sci Data. 2019 Nov 6;6:262. doi: 10.1038/s41597-019-0281-5 (PMC6834640; doi:10.1038/s41597-019-0281-5)
Supplement: Supplementary file 1 — Supplementary Files. [file 41597_2019_281_MOESM1_ESM.pdf]

Supplementary Fig 1: Identification via PCR of 18 WBSCR1, 4 GTF2IRD1, 7 GTF2IRD2 and 3 GTF2I positive clones. In red the ones on which the farther experiments have been performed. **PAGE 1**

Supplementary Fig 2: quality control of the RNA samples in the time course of induction (17, 24, 39 and 48hrs). In red the RNA samples (T0 and T24hrs of induction) on which the Microarray experiments were performed. **PAGE 2**

Supplementary Fig 3: Average induction, expressed as fold change of induction (2^-ddCt) of all 4genes: for the microarray experiment we chose 24 hrs after tetracycline removal. **PAGE 3**

Supplementary Fig 4: Scatter plot of RNA levels comparing all detected genes pre and post-induction samples. **PAGE 4**

Supplementary File 1: sequences Asc1-Pac1 4WBS ORFs. **PAGE 5**

Supplementary File 2: primer pairs used in RT-PCR. **PAGE 8**

Supplementary File 3: time course of induction of WBS clones **PAGE 9**

Supplementary File 4: includes the differential expression data from inducible ES stable cell line overexpressing the human WBS genes. **PAGE 14**

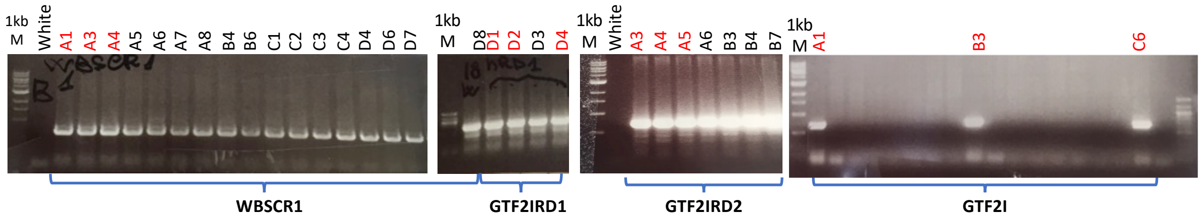

Supplementary Fig. 1

Identification via PCR of 18 WBSCR1, 4 GTF2IRD1, 7 GTF2IRD2 and 3 GTF2I positive clones.
In red the ones on which the farther experiments have been performed.

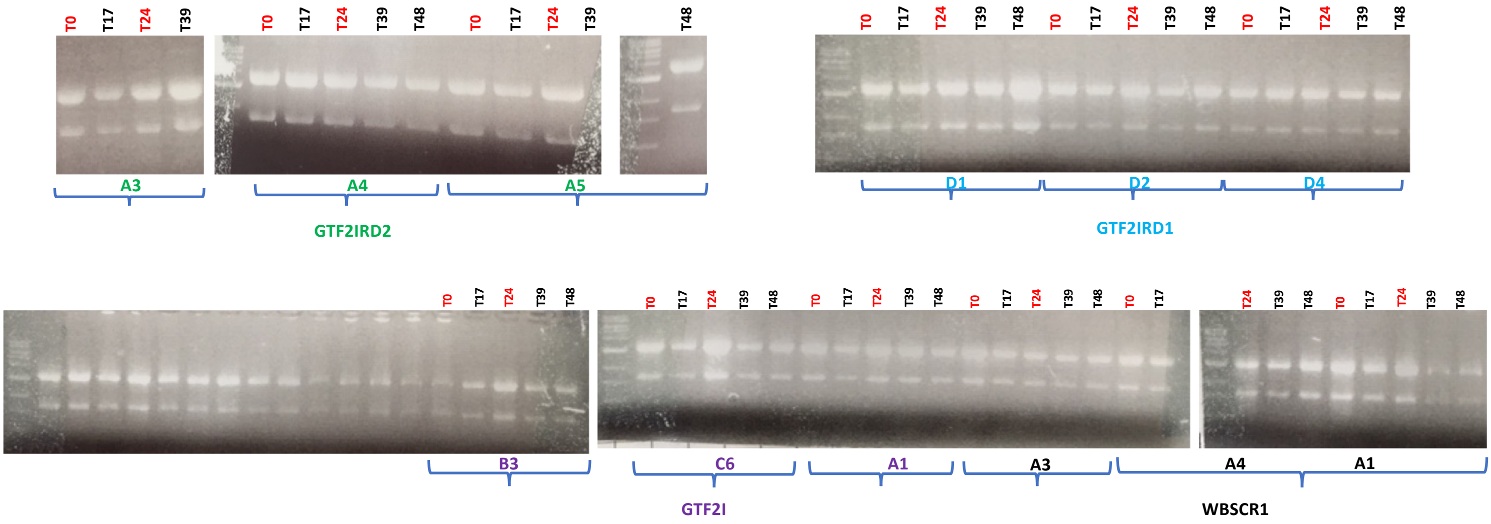

Supplementary Fig. 2

quality control of the RNA samples in the time course of induction (17, 24, 39 and 48hrs).
In red the RNA samples (T0 and T24hrs of induction) on which the Microarray experiments were performed.

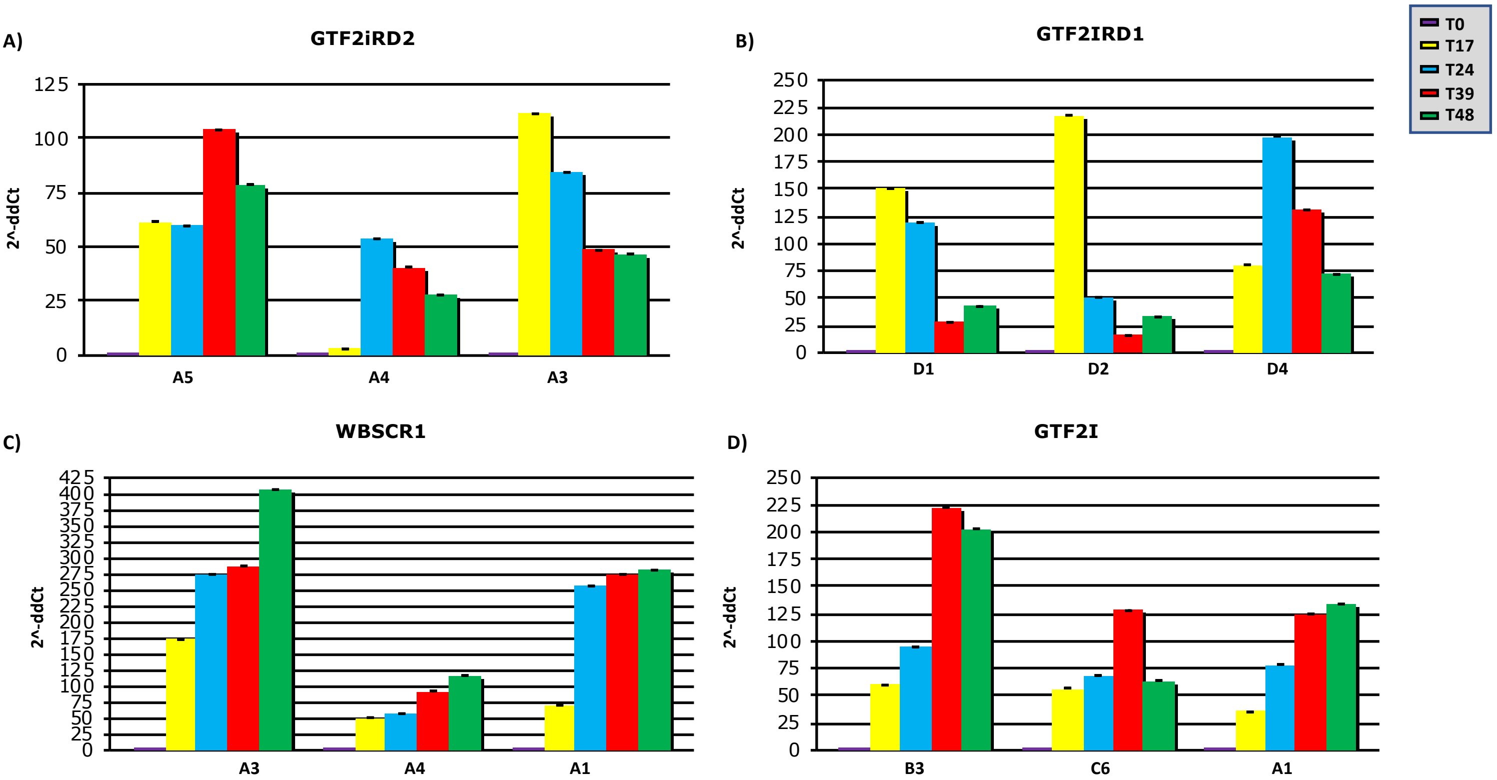

Supplementary Fig. 3

Average induction, expressed as fold change of induction (2^-ddCt) of all 4genes: for the microarray experiment we chose 24 hrs after tetracycline removal.

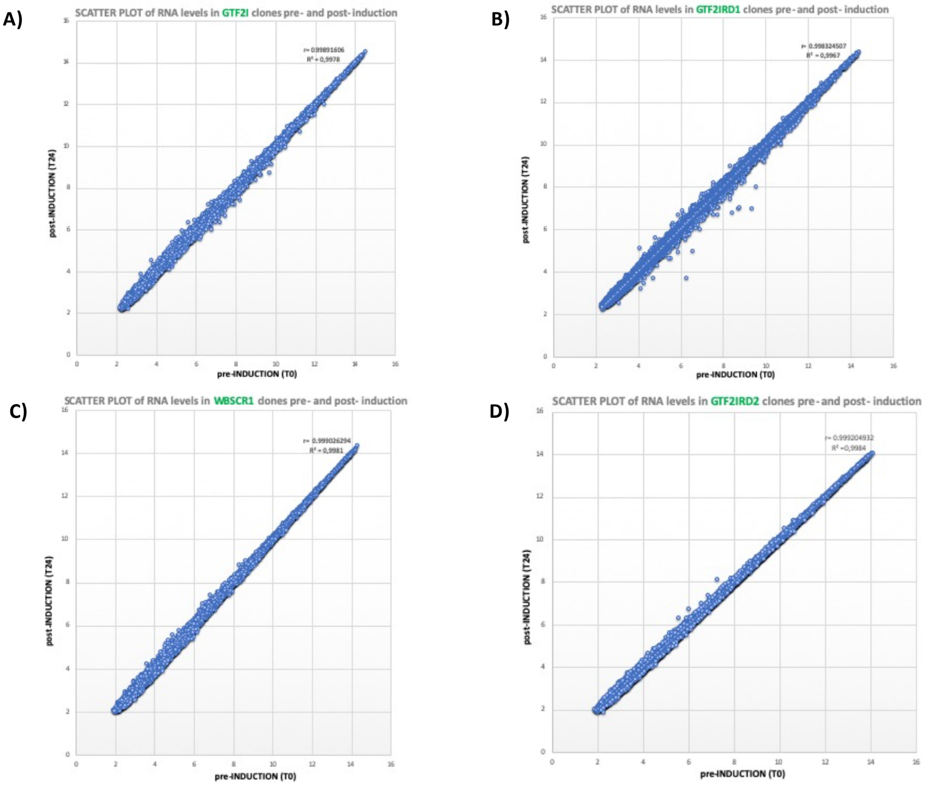

Supplementary Fig. 4

Scatter plot of RNA levels comparing all detected genes pre and post-induction samples.

Supplementary File 1: sequences Asc1-Pac1 4WBS ORFs.

AscI-WBSCR1-PacI
GGCGCGCCATGGCGGACTTCGACACCTACGACGATCGGGCCTACAGCAGCTTCGGCGGCGGCAGAG
GGTCCCGCGGCAGTGCTGGTGGCCATGGTTCCCGTAGCCAGAAGGAGTTGCCCACAGAGCCCCCCTA
CACAGCATACGTAGGAAATCTACCTTTCAATACGGTTCAGGGCGACATAGATGCTATCTTTAAGGATCTC
AGCATAAGGAGTGTACGGCTAGTCAGAGACAAAGACACAGATAAATTTAAAGGATTCTGCTATGTAGAA
TTCGATGAAGTGGATTCCCTTAAGGAAGCCTTGACATACGATGGTGCACTGTTGGGCGATCGGTCACTT
CGTGTGGACATTGCAGAAGGCAGAAAACAAGATAAAGGTGGCTTTGGATTCAGAAAAGGTGGACCAGA
TGACAGAGGCTTCAGGGATGACTTCTTAGGGGGCAGGGGAGGTAGTCGCCCAGGCGACCGGCGAACA
GGCCCCCCCATGGGCAGCCGCTTCAGAGATGGCCCTCCCCTCCGTGGATCCAACATGGATTTCAGAGA
ACCCACAGAAGAGGAAAGAGCACAGAGACCACGACTCCAGCTTAAACCTCGAACAGTCGCGACGCCCC
TCAATCAAGTAGCCAATCCCAACTCTGCTATCTTCGGGGGTGCCAGGCCTAGAGAGGAAGTCGTTCAAA
AGGAGCAAGAATTAATTAA

AscI-GTF2i-PacI
ggcgcgccATGGCCCAAGTTGCAATGTCCACCCTCCCCGTTGAAGATGAGGAGTCCTCGGAGAGCAGGAT
GGTGGTGACATTCCTCATGTCAGCTCTCGAGTCCATGTGTAAAGAACTGGCCAAGTCCAAAGCCGAAGT
GGCCTGCATTGCAGTGTATGAAACAGACGTGTTTGTCGTCGGAACTGAAAGAGGACGTGCTTTTGTCAA
TACCAGAAAGGATTTTCAAAAAGATTTTGTAAAATATTGTGTTGAAGAAGAAGAAAAAGCTGCAGAGATG
CATAAAATGAAATCTACAACCCAGGCAAATCGGATGAGTGTAGATGCTGTAGAAATTGAAACACTCAGAA
AAACAGTTGAGGACTATTTCTGCTTTTGCTATGGGAAAGCTTTAGGCAAATCCACAGTGGTACCTGTACC
ATATGAGAAGATGCTGCGAGACCAGTCGGCTGTGGTAGTGCAGGGGCTTCCGGAAGGTGTTGCCTTTA
AACACCCCGAGAACTATGATCTTGCAACCCTGAAATGGATTTTGGAGAACAAAGCAGGGATTTCATTCAT
CATTAAGAGACCTTTTTTAGAGCCAAAGAAGCATGTAGGTGGTCGTGTGATGGTAACAGATGCTGACAG
GTCAATACTATCTCCAGGTGGAAGTTGTGGCCCCATCAAAGTGAAAACTGAACCCACAGAAGATTCTGG
CATTTCCCTGGAAATGGCAGCTGTGACAGTAAAGGAAGAATCAGAAGATCCTGATTATTATCAATATAAC
ATTCAAGGCCCTTCTGAAACTGATGATGTTGATGAAAAACAGCCCCTATCGAAGCCTTTGCAAGGAAGC
CACCATTCTTCAGAGGGCAATGAAGGCACAGAAATGGAAGTACCAGCAGAAGATGATGATTATTCTCCA
CCGTCTAAGAGACCAAAGGCCAATGAGCTACCGCAGCCACCAGTCCCGGAACCCGCCAATGCTGGGA
AGCGGAAAGTGAGGGAGTTCAACTTCGAGAAATGGAATGCTCGCATCACTGATCTACGTAAACAAGTTG
AAGAATTGTTTGAAAGGAAATATGCTCAAGCCATAAAAGCCAAAGGTCCGGTGACGATCCCGTACCCTC
TTTTCCAGTCTCATGTTGAAGATCTTTATGTAGAAGGACTTCCTGAAGGAATTCCTTTTAGAAGGCCATC
TACTTACGGAATTCCTCGCCTGGAGAGGATATTACTTGCAAAGGAAAGGATTCGTTTTGTGATTAAGAAA
CATGAGCTTCTGAATTCAACACGTGAAGATTTACAGCTTGATAAGCCAGCTTCAGGAGTAAAGGAAGAA
TGGTATGCCAGAATCACTAAATTAAGAAAGATGGTGGATCAGCTTTTCTGCAAAAAATTTGCGGAAGCCT
TGGGGAGCACTGAAGCCAAGGCTGTACCGTACCAAAAATTTGAGGCACACCCGAATGATCTGTACGTG
GAAGGACTGCCAGAAAACATTCCTTTCCGAAGTCCCTCATGGTATGGAATCCCAAGGCTGGAAAAAATC
ATTCAAGTGGGCAATCGAATTAAATTTGTTATTAAAAGACCAGAACTTCTGACTCACAGTACCACTGAAG
TTACTCAGCCAAGAACGAATACACCAGTCAAAGAAGATTGGAATGTCAGAATTACCAAGCTACGGAAGC
AAGTGGAAGAGATTTTTAATTTGAAATTTGCTCAAGCTCTTGGACTCACCGAGGCAGTAAAAGTACCATA
TCCTGTGTTTGAATCAAACCCGGAGTTCTTGTATGTGGAAGGCTTGCCAGAGGGGATTCCCTTCCGAAG
CCCTACCTGGTTTGGAATTCCACGACTTGAAAGGATCGTCCGCGGGAGTAATAAAATCAAGTTCGTTGT
TAAAAAACCTGAACTAGTTATTTCCTACTTGCCTCCTGGGATGGCTAGTAAAATAAACACTAAAGCTTTG
CAGTCCCCCAAAAGACCACGAAGTCCTGGGAGTAATTCAAAGGTTCCTGAAATTGAGGTCACCGTGGAA
GGCCCTAATAACAACAATCCTCAAACCTCAGCTGTTCGAACCCCGACCCAGACTAACGGTTCTAACGTT
CCCTTCAAGCCACGAGGGAGAGAGTTTTCCTTTGAGGCCTGGAATGCCAAAATCACGGACCTAAAACA
GAAAGTTGAAAATCTCTTCAATGAGAAATGTGGGGAAGCTCTTGGCCTTAAACAAGCTGTGAAGGTGCC
GTTCGCGTTATTTGAGTCTTTCCCGGAAGACTTTTATGTGGAAGGCTTACCTGAGGGTGTGCCATTCCG
AAGACCATCGACTTTTGGCATTCCGAGGCTGGAGAAGATACTCAGAAACAAAGCCAAAATTAAGTTCAT
CATTAAAAAGCCCGAAATGTTTGAGACGGCGATTAAGGAGAGCACCTCCTCTAAGAGCCCTCCCAGAAA
AATAAATTCATCACCCAATGTTAATACTACTGCATCAGGTGTTGAAGACCTTAACATCATTCAGGTGACA
ATTCCAGATGATGATAATGAAAGACTCTCGAAAGTTGAAAAAGCTAGACAGCTAAGAGAACAAGTGAAT
GACCTCTTTAGTCGGAAATTTGGTGAAGCTATTGGTATGGGTTTTCCTGTGAAAGTTCCCTACAGGAAAA
TCACAATTAACCGTGGCTGTGTGGTGGTTGATGGCATGCCCCCGGGGGTGTCCTTCAAAGCCCCCAGC
TACCTGGAAATCAGCTCCATGAGAAGGATCTTAGACTCTGCCGAGTTTATCAAATTCACGGTCATTAGAC

CATTTCCAGGACTTGTGGTTAATAACCAGCTGGTTGATCAGAGTGAGTCAGAAGGCCCCGTGATACAAG
AATCAGCTGAACCAAGCCAGTTGGAAGTTCCAGCCACAGAAGAAATAAAAGAGACTGATGGAAGCTCTC
AGATCAAGCAAGAACCAGACCCCACGTGGttaattaa

AscI-GTF2iRD1-PacI

ggcgcgccATGGCCTTGCTGGGTAAGCGCTGTGACGTCCCCACCAACGGCTGCGGACCCGACCGCTGGA
ACTCCGCGTTCACCCGCAAAGACGAGATCATCACCAGCCTCGTGTCTGCCTTAGACTCCATGTGCTCAG
CGCTGTCCAAACTGAACGCCGAGGTGGCCTGTGTCGCCGTGCACGATGAGAGCGCCTTTGTGGTGGG
CACAGAGAAGGGGAGAATGTTCCTGAATGCCCGGAAGGAGCTACAGTCAGACTTCCTCAGGTTCTGCC
GAGGGCCCCCGTGGAAGGATCCGGAGGCAGAGCACCCCAAGAAGGTGCAGCGGGGCGAGGGTGGAG
GCCGTAGCCTCCCTCGGTCCTCCCTGGAACATGGCTCAGATGTGTACCTTCTGCGGAAGATGGTAGAG
GAGGTGTTTGATGTTCTTTATAGCGAGGCCCTGGGAAGGGCCAGTGTGGTGCCACTGCCCTATGAGAG
GCTGCTCAGGGAGCCAGGGCTGCTGGCCGTGCAGGGGCTGCCCGAGGGCCTGGCCTTCCGAAGGCC
AGCCGAGTATGACCCCAAGGCCCTCATGGCCATCCTGGAACACAGCCACCGCATCCGCTTCAAGCTCA
AGAGGCCACTTGAGGATGGCGGGCGGGACTCGAAGGCCCTGGTGGAGCTGAACGGTGTCTCCCTGAT
TCCCAAGGGGTCACGGGACTGTGGCCTGCATGGCCAGGCCCCCAAGGTGCCACCCCAGGACCTGCCC
CCAACCGCCACCTCCTCCTCCATGGCCAGCTTCCTGTACAGCACGGCGCTCCCCAACCACGCCATCCG
AGAGCTCAAGCAGGAAGCACCTTCCTGCCCCCTTGCCCCCAGCGACCTGGGCCTGAGTCGGCCCATG
CCAGAGCCCAAGGCCACCGGTGCCCAAGACTTCTCCGACTGTTGTGGACAGAAGCCCACTGGGCCTG
GTGGGCCTCTCATCCAGAACGTCCATGCCTCCAAGCGCATTCTCTTCTCCATCGTCCATGACAAGTCAG
AGAAGTGGGACGCCTTCATAAAGGAAACCGAGGACATCAACACGCTCCGGGAGTGTGTGCAGATCCTG
TTTAACAGCAGATATGCGGAAGCCCTGGGCCTGGACCACATGGTCCCCGTGCCCTACCGGAAGATTGC
CTGTGACCCGGAGGCTGTGGAGATCGTGGGCATCCCGGACAAGATCCCCTTCAAGCGCCCCTGCACTT
ACGGAGTCCCCAAGCTGAAGCGGATCCTGGAGGAGCGCCATAGTATCCACTTCATCATTAAGAGGATG
TTTGATGAGCGAATTTTCACAGGGAACAAGTTTACCAAAGACACCACGAAGCTGGAGCCAGCCAGCCC
GCCAGAGGACACCTCTGCAGAGGTCTCTAGGGCCACCGTCCTTGACCTTGCTGGGAATGCTCGGTCAG
ACAAGGGCAGCATGTCTGAAGACTGTGGGCCAGGAACCTCCGGGGAGCTGGGCGGGCTGAGGCCGA
TCAAAATTGAGCCAGAGGATCTGGACATCATTCAGGTCACCGTCCCAGACCCCTCGCCAACTGAGGCC
GATCAAAATTGAGCCAGAGGATCTGGACATCATTCAGGTCACCGTCCCAGACCCCTCGCCAACCTCTGA
GGAAATGACAGACTCGATGCCTGGGCACCTGCCATCGGAGGATTCTGGTTATGGGATGGAGATGCTGA
CAGACAAAGGTCTGAGTGAGGACGCGCGGCCCGAGGAGAGGCCCGTGGAGGACAGCCACGGTGACG
TGATCCGGCCCCTGCGGAAGCAGGTGGAGCTGCTCTTCAACACACGATACGCCAAGGCCATTGGCATC
TCGGAGCCCGTCAAGGTGCCGTACTCCAAGTTTCTGATGCACCCGGAGGAGCTGTTTGTGGTGGGACT
GCCTGAAGGCATCTCCCTCCGCAGGCCCAACTGCTTCGGGATCGCCAAGCTCCGGAAGATTCTGGAG
GCCAGCAACAGCATCCAGTTTGTCATCAAGAGGCCCGAGCTGCTCACTGAGGGAGTCAAAGAGCCCAT
CGTGGATAGTCAAGGAACTGCCTCCTCACTTGGCTTCTCTCCCCCTGCCCTGCCCCCAGAGAGGGATT
CCGGGGACCCTCTGGTGGACGAGAGCCTGAAGAGACAGGGCTTTCAAGAAAATTATGACGCGAGGCT
CTCACGGATCGACATCGCCAACACACTAAGGGAGCAGGTCCAGGACCTTTTCAATAAGAAATACGGGG
AAGCCTTGGGCATCAAGTACCCGGTCCAGGTCCCCTACAAGCGGATCAAGAGTAACCCCGGCTCCGTG
ATCATCGAGGGGCTGCCCCCAGGAATCCCGTTCCGAAAGCCCTGTACCTTCGGCTCCCAGAACCTGGA
GAGGATTCTTGCTGTGGCTGACAAGATCAAGTTCACAGTCACCAGGCCTTTCCAAGGACTCATCCCAAA
GCCTGATGAAGATGACGCCAACAGACTCGGGGAGAAGGTGATCCTGCGGGAGCAGGTGAAGGAACTC
TTCAACGAGAAATACGGTGAGGCCCTGGGCCTGAACCGGCCGGTGCTGGTCCCTTATAAACTAATCCG
GGACAGCCCAGACGCCGTGGAGGTCACGGGTCTGCCTGATGACATCCCCTTCCGGAACCCCAACACG
TACGACATCCACCGGCTGGAGAAGATCCTGAAGGCCCGAGAGCATGTCCGCATGGTCATCATTAACCA
GCTCCAACCCTTTGCAGAAATCTGCAATGATGCCAAGGTGCCAGCCAAAGACAGCAGCATTCCCAAGC
GCAAGAGAAAGCGGGTCTCGGAAGGAAATTCCGTCTCCTCTTCCTCCTCGTCTTCCTCTTCCTCGTCCT
CTAACCCGGATTCAGTGGCATCGGCCAACCAGATCTCACTCGTGCAATGGCCAATGTACATGGTGGAC
TATGCCGGCCTGAACGTGCAGCTCCCGGGACCTCT TAATTACttaattaa

AscI-GTF2iRD2-PacI

ggcgcgccATGGCCCAGGTAGCAGTGTCCACCCTGCCTGTTGAAGAAGAGTCCTCCTCAGAGACCAGGAT
GGTGGTGACATTCCTCGTGTCTGCCCTCGAATCCATGTGTAAAGAACTGGCCAAGTCCAAGGCAGAAG
TGGCCTGCATCGCAGTGTACGAAACAGACGTGTTTGTCGTCGGAACCGAGAGAGGATGCGCTTTTGTT

AATGCCAGGACGGATTTTCAGAAAGATTTTGCAAAATACTGTAGGTGTTTTAATTTTATCCTTTGTATTCC
CAATCTCAAAA GGATCGCAGGCAAGACTTCCACAGTATTTTCTTCTAAGCTATCAttaattaa

Supplementary File 2: primer pairs used in RT-PCR.

Actb RT up:
5'-ACG TTG ACA TCC GTA AAG ACC T-3'
Actb RT dw:
5'-GCA GTA ATC TCC TTC TGC ATC C-3'

GTF2I RT up:
5'-AATGTCCACCCTCCCCGTTGAA-3'
hGTF2I RT down:
5'-TCCTCTTTCAGTTCCGACGACA-3'

GTF2IRD1 RT up:
5'-GAGGCAGAGCACCCCAAGAA-3'
GTF2IRD2 RT dw:
5'-CCCGCCCGCCATCCTCAAGT-3'

GTF2IRD2 RT up:
5'-GTCCACCCTGCCTGTTGAAG-3'
hGTF2IRD2 RT dw:
5'-GCGCATCCTCTCTCGGTTCCG-3'

WBSCR1 (EIF4H) RT up:
5'- TCTTAGGGGGCAGGGGAGGTAG-3'
WBSCR1 (EIF4H) RT dw:
5'- TCTTGCTCCTTTTGAACGACTT-3'

Supplementary File 3: time course of induction of WBS clones								
A) time course of induction of GTF2i clones (named C6, B3, A1)								
Threshold Cycle	Ct	Identifier	Average	SEM	dCT	SE dCt	2^-dCt	SE2^-dCt
16.9		b3/T0	16.86667	0.033333333				
16.9		b3/T0						
16.8		b3/T0						
15.6		b3/T17	15.5	0.057735027				
15.4		b3/T17						
15.5		b3/T17						
16.2		b3/T24	16.3	0.057735027				
16.4		b3/T24						
16.3		b3/T24						
17.6		b3/T39	18	0.4				
17.6		b3/T39						
18.8		b3/T39						
17.4		b3/T48	17.43333	0.08819171				
17.6		b3/T48						
17.3		b3/T48						
17		c6/T0	17.03333	0.033333333				
17.1		c6/T0						
17		c6/T0						
16.9		c6/T17	16.86667	0.033333333				
16.8		c6/T17						
16.9		c6/T17						
16		c6/T24	16.23333	0.120185043				
16.3		c6/T24						
16.4		c6/T24						
16		c6/T39	16.13333	0.08819171				
16.3		c6/T39						
16.1		c6/T39						
21.1		c6/T48	21.33333	0.120185043				
21.5		c6/T48						
21.4		c6/T48						
15.8		a1/T0	15.96667	0.08819171				
16.1		a1/T0						
16		a1/T0						
15.8		a1/T17	15.8	1.25607E-15				
15.8		a1/T17						
15.8		a1/T17						
16.5		a1/T24	15.63333	0.437162568				
15.3		a1/T24						
15.1		a1/T24						
16.7		a1/T39	16.76667	0.066666667				
16.7		a1/T39						
16.9		a1/T39						
16.3		a1/T48	16.16667	0.08819171				
16		a1/T48						
16.2		a1/T48						
27.7		b3/T0/g	27.8	0.057735027	10.93333	0.066667	0.000511	2.36E-05
27.8		b3/T0/g						
27.9		b3/T0/g						
20.7		b3/T17/g	20.53333	0.08819171	5.033333	0.105409	0.030536	0.002231
20.5		b3/T17/g						
20.4		b3/T17/g						
20.6		b3/T24/g	20.66667	0.066666667	4.366667	0.088192	0.048473	0.002963
20.6		b3/T24/g						
20.8		b3/T24/g						
21.1		b3/T39/g	21.13333	0.08819171	3.133333	0.409607	0.113965	0.032357
21.3		b3/T39/g						
21		b3/T39/g						
20.9		b3/T48/g	20.7	0.1	3.266667	0.133333	0.103905	0.009603
20.6		b3/T48/g						
20.6		b3/T48/g						
28.2		c6/T0/g	28.23333	0.202758751	11.2	0.20548	0.000425	6.05E-05
27.9		c6/T0/g						
28.6		c6/T0/g						
22.2		c6/T17/g	22.23333	0.033333333	5.366667	0.04714	0.024237	0.000792
22.2		c6/T17/g						
22.3		c6/T17/g						
21.3		c6/T24/g	21.33333	0.033333333	5.1	0.124722	0.029157	0.002521
21.4		c6/T24/g						
21.3		c6/T24/g						
20.4		c6/T39/g	20.33333	0.066666667	4.2	0.110554	0.054409	0.004169
20.4		c6/T39/g						
20.2		c6/T39/g						
26.7		c6/T48/g	26.53333	0.08819171	5.2	0.149071	0.027205	0.002811

26.5	c6/T48/g						
26.4	c6/T48/g						
27.1	a1/T0/g	27.16667	0.066666667	11.2	0.110554	0.000425	3.26E-05
27.1	a1/T0/g						
27.3	a1/T0/g						
21.7	a1/T17/g	21.86667	0.08819171	6.066667	0.088192	0.014919	0.000912
21.9	a1/T17/g						
22	a1/T17/g						
20.6	a1/T24/g	20.53333	0.033333333	4.9	0.438432	0.033493	0.010178
20.5	a1/T24/g						
20.5	a1/T24/g						
20.8	a1/T39/g	21	0.1	4.233333	0.120185	0.053167	0.004429
21.1	a1/T39/g						
21.1	a1/T39/g						
20.1	a1/T48/g	20.3	0.115470054	4.133333	0.145297	0.056983	0.005739
20.3	a1/T48/g						
20.5	a1/T48/g						

B) time course of induction of GTF2iRD1 clones (named D1, D2, D4)

Threshold Cycle Ct	Identifier	Average	SEM	dCT	SE dCt	2^-dCt	SE2^-dCt
14.9	1/d1/t0	14.76667	0.08819171				
14.8	1/d1/t0						
14.6	1/d1/t0						
14.6	1/d1/t17	15.03333	0.338296386				
14.8	1/d1/t17						
15.7	1/d1/t17						
14.2	1/d1/t24	14.33333	0.08819171				
14.5	1/d1/t24						
14.3	1/d1/t24						
17.2	1/d1/t39	17.4	0.115470054				
17.4	1/d1/t39						
17.6	1/d1/t39						
18.2	1/d1/t48	18.2	0.057735027				
18.3	1/d1/t48						
18.1	1/d1/t48						
15.2	1/d2/t0	15.3	0.152752523				
15.6	1/d2/t0						
15.1	1/d2/t0						
15.4	1/d2/t17	16.23333	0.935117343				
15.2	1/d2/t17						
18.1	1/d2/t17						
15.7	1/d2/t24	15.96667	0.133333333				
16.1	1/d2/t24						
16.1	1/d2/t24						
21.2	1/d2/t39	21.16667	0.033333333				
21.2	1/d2/t39						
21.1	1/d2/t39						
16.4	1/d2/t48	16.3	0.1				
16.1	1/d2/t48						
16.4	1/d2/t48						
14.6	1/d4/t0	14.83333	0.120185043				
14.9	1/d4/t0						
15	1/d4/t0						
17	1/d4/t17	17.1	0.057735027				
17.2	1/d4/t17						
17.1	1/d4/t17						
15.3	1/d4/t24	15.1	0.1				
15	1/d4/t24						
15	1/d4/t24						
16.8	1/d4/t39	16.83333	0.08819171				
17	1/d4/t39						
16.7	1/d4/t39						
17.5	1/d4/t48	17.33333	0.08819171				
17.2	1/d4/t48						
17.3	1/d4/t48						
24.9	1/d1/t0/1	25.23333	0.202758751	10.46667	0.221108	0.000707	0.000108
25.6	1/d1/t0/1						
25.2	1/d1/t0/1						
18.8	1/d1/t17/1	18.26667	0.290593263	3.233333	0.44597	0.106333	0.03287
18.2	1/d1/t17/1						
17.8	1/d1/t17/1						
17.7	1/d1/t24/1	17.9	0.2	3.566667	0.218581	0.084397	0.012787
N/A	1/d1/t24/1						
18.1	1/d1/t24/1						
23.1	1/d1/t39/1	23.06667	0.145296631	5.666667	0.185592	0.019686	0.002532
22.8	1/d1/t39/1						

23.3	1/d1/t39/1						
23.7	1/d1/t48/1	23.26667	0.218581284	5.066667	0.226078	0.029839	0.004676
23	1/d1/t48/1						
23.1	1/d1/t48/1						
25.9	1/d2/t0/1	26.3	0.2081666	11	0.258199	0.000488	8.74E-05
26.6	1/d2/t0/1						
26.4	1/d2/t0/1						
19.4	1/d2/t17/1	19.46667	0.233333333	3.233333	0.963789	0.106333	0.071036
19.1	1/d2/t17/1						
19.9	1/d2/t17/1						
20.8	1/d2/t24/1	21.3	0.450924975	5.333333	0.470225	0.024803	0.008084
22.2	1/d2/t24/1						
20.9	1/d2/t24/1						
28.3	1/d2/t39/1	28.2	0.1	7.033333	0.105409	0.007634	0.000558
28	1/d2/t39/1						
28.3	1/d2/t39/1						
22.1	1/d2/t48/1	22.26667	0.08819171	5.966667	0.133333	0.01599	0.001478
22.4	1/d2/t48/1						
22.3	1/d2/t48/1						
26.9	1/d4/t0/1	26.36667	0.352766841	11.53333	0.372678	0.000337	8.72E-05
25.7	1/d4/t0/1						
26.5	1/d4/t0/1						
22	1/d4/t17/1	22.3	0.152752523	5.2	0.163299	0.027205	0.003079
22.5	1/d4/t17/1						
22.4	1/d4/t17/1						
18.7	1/d4/t24/1	19	0.173205081	3.9	0.2	0.066986	0.009286
19.3	1/d4/t24/1						
19	1/d4/t24/1						
21.6	1/d4/t39/1	21.33333	0.218581284	4.5	0.235702	0.044194	0.00722
21.5	1/d4/t39/1						
20.9	1/d4/t39/1						
22.2	1/d4/t48/1	22.7	0.288675135	5.366667	0.301846	0.024237	0.005071
23.2	1/d4/t48/1						
22.7	1/d4/t48/1						

C) time course of induction of GTF2iRD2 clones (named A3, A4, A5)

Threshold Cycle Ct	Identifier	Average	SEM	dCT	SE dCt	2^-dCt	SE2^-dCt
15.8	a5 t0	15.8	0				
15.8	a5 t0						
16.2	a5 t17	16.1	0.1				
16	a5 t17						
15.2	a5 t24	15.2	0				
15.2	a5 t24						
16.5	a5 t39	16.65	0.15				
16.8	a5 t39						
15.4	a5 t48	15.5	0.1				
15.6	a5 t48						
15.4	a4 t0	15.7	0.3				
16	a4 t0						
17.4	a4 t17	17.4	0				
17.4	a4 t17						
17.4	a4 t24	17.4	0				
17.4	a4 t24						
17.2	a4 t39	17.05	0.15				
16.9	a4 t39						
16.9	a4 t48	16.95	0.05				
17	a4 t48						
16.8	a3 t0	16.8	0				
16.8	a3 t0						
16.7	a3 t17	17.05	0.35				
17.4	a3 t17						
16.8	a3 t24	17.25	0.45				
17.7	a3 t24						
16.2	a3 t39	16.2	0				
16.2	a3 t39						
16.6	a3 t48	16.8	0.2				
17	a3 t48						
27.1	a5 t0 2	27.25	0.15	11.45	0.15	0.000357	3.72E-05
27.4	a5 t0 2						
21.5	a5 t17 2	21.6	0.1	5.5	0.141421	0.022097	0.002166
21.7	a5 t17 2						
20.9	a5 t24 2	20.75	0.15	5.55	0.15	0.021344	0.002219
20.6	a5 t24 2						
21.3	a5 t39 2	21.4	0.1	4.75	0.180278	0.037163	0.004644
21.5	a5 t39 2						
20.6	a5 t48 2	20.65	0.05	5.15	0.111803	0.028164	0.002183

20.7	a5 t48 2						
26.9	a4 t0 2	26.75	0.15	11.05	0.33541	0.000472	0.00011
26.6	a4 t0 2						
26.7	a4 t17 2	26.85	0.15	9.45	0.15	0.00143	0.000149
27	a4 t17 2						
22.7	a4 t24 2	22.7	0	5.3	0	0.025383	0
22.7	a4 t24 2						
22.7	a4 t39 2	22.75	0.05	5.7	0.158114	0.019237	0.002108
22.8	a4 t39 2						
23.2	a4 t48 2	23.2	0	6.25	0.05	0.013139	0.000455
23.2	a4 t48 2						
26.5	a3 t0 2	26.85	0.35	10.05	0.35	0.000943	0.000229
27.2	a3 t0 2						
19.9	a3 t17 2	20.3	0.4	3.25	0.531507	0.105112	0.038725
20.7	a3 t17 2						
21	a3 t24 2	20.9	0.1	3.65	0.460977	0.07966	0.025453
20.8	a3 t24 2						
20.7	a3 t39 2	20.65	0.05	4.45	0.05	0.045753	0.001586
20.6	a3 t39 2						
21.2	a3 t48 2	21.3	0.1	4.5	0.223607	0.044194	0.00685
21.4	a3 t48 2						

D) time course of induction of WBSCR1 clones (named A1, A3, A4)

Threshold Cycle Ct	Identifier	Average	SEM	dCT	SE dCt	2^-dCt	SE2^-dCt
16.2	a3/T0	16.13333	0.033333333				
16.1	a3/T0						
16.1	a3/T0						
16.2	a3/T17	16.63333	0.260341656				
17.1	a3/T17						
16.6	a3/T17						
14.4	a3/T24	14.6	0.115470054				
14.8	a3/T24						
14.6	a3/T24						
17.7	a3/T39	17.8	0.057735027				
17.9	a3/T39						
17.8	a3/T39						
15.9	a3/T48	15.93333	0.08819171				
15.8	a3/T48						
16.1	a3/T48						
14.3	a4/T0	14.36667	0.066666667				
14.5	a4/T0						
14.3	a4/T0						
15	a4/T17	15.23333	0.120185043				
15.4	a4/T17						
15.3	a4/T17						
14.9	a4/T24	14.5	0.2				
14.3	a4/T24						
14.3	a4/T24						
16.8	a4/T39	17	0.152752523				
16.9	a4/T39						
17.3	a4/T39						
16.4	a4/T48	16	0.230940108				
16	a4/T48						
15.6	a4/T48						
14.7	a1/T0	14.7	0.173205081				
14.4	a1/T0						
15	a1/T0						
14.9	a1/T17	14.8	0.1				
14.9	a1/T17						
14.6	a1/T17						
14.5	a1/T24	14.2	0.152752523				
14.1	a1/T24						
14	a1/T24						
14.9	a1/T39	14.63333	0.176383421				
14.7	a1/T39						
14.3	a1/T39						
14.9	a1/T48	15.1	0.115470054				
15.3	a1/T48						
15.1	a1/T48						
27.5	a3/T0/w	27.5	0	11.36667	0.033333	0.000379	8.75E-06
27.5	a3/T0/w						
27.5	a3/T0/w						
20.6	a3/T17/w	20.56667	0.08819171	3.933333	0.274874	0.065456	0.012471
20.4	a3/T17/w						
20.7	a3/T17/w						
17.8	a3/T24/w	17.86667	0.120185043	3.266667	0.166667	0.103905	0.012004

17.7	a3/T24/w						
18.1	a3/T24/w						
21	a3/T39/w	21	0.057735027	3.2	0.08165	0.108819	0.006159
21.1	a3/T39/w						
20.9	a3/T39/w						
18.7	a3/T48/w	18.63333	0.033333333	2.7	0.094281	0.153893	0.010057
18.6	a3/T48/w						
18.6	a3/T48/w						
25	a4/T0/w	24.7	0.152752523	10.33333	0.166667	0.000775	8.95E-05
24.6	a4/T0/w						
24.5	a4/T0/w						
19.9	a4/T17/w	19.9	0	4.666667	0.120185	0.039373	0.00328
19.9	a4/T17/w						
19.9	a4/T17/w						
18.9	a4/T24/w	19	0.057735027	4.5	0.208167	0.044194	0.006377
19.1	a4/T24/w						
19	a4/T24/w						
20.8	a4/T39/w	20.8	0	3.8	0.152753	0.071794	0.007602
20.8	a4/T39/w						
20.8	a4/T39/w						
19.3	a4/T48/w	19.46667	0.120185043	3.466667	0.260342	0.090454	0.016323
19.7	a4/T48/w						
19.4	a4/T48/w						
25.3	a1/T0/w	25.26667	0.033333333	10.56667	0.176383	0.000659	8.06E-05
25.2	a1/T0/w						
25.3	a1/T0/w						
19.1	a1/T17/w	19.23333	0.08819171	4.433333	0.133333	0.046284	0.004278
19.2	a1/T17/w						
19.4	a1/T17/w						
16.7	a1/T24/w	16.76667	0.066666667	2.566667	0.166667	0.168794	0.0195
16.7	a1/T24/w						
16.9	a1/T24/w						
17.2	a1/T39/w	17.1	0.2081666	2.466667	0.272845	0.180909	0.034214
17.4	a1/T39/w						
16.7	a1/T39/w						
17.1	a1/T48/w	17.53333	0.218581284	2.433333	0.247207	0.185137	0.031723
17.7	a1/T48/w						
17.8	a1/T48/w						

Supplementary File 4: includes the differential expression data from inducible ES stable cell line overexpressing the human WBS genes

A) includes the differential expression data from inducible ES stable cell line overexpressing the human GTF2IRD1

Probe Set ID	Gene Title	Gene Symbol	fdr	signed_ratio	foldChange
1425427_at	hypothetical protein LOC639910	LOC639910	7.78E-06	-5.71425662	-2.5174873
1443961_at	predicted gene 4340	Gm4340	1.16E-05	-5.0350893	-2.3527131
1451899_a_at	general transcription factor II I repeat domain-containing 1	Gtf2ird1	1.67E-05	-2.85120289	-1.5128101
1427479_at	expressed sequence BB287469 /// eukaryotic translation initiatio	BB287469 /// Eif1a /// Gn	2.58E-05	-3.30133394	-1.7306859
1444529_at	predicted gene 8300	Gm8300	0.0004	-2.97064061	-1.5872636
1425220_x_at	cDNA sequence AF067061	AF067061	0.00044	-3.24322894	-1.7330596
1455886_at	Casitas B-lineage lymphoma	Cbl	0.0025	-2.08211094	-1.0631514
1445849_at	cDNA sequence BC080696	BC080696	0.00584	2.08605896	1.05326437
1452972_at	tetratricopeptide repeat domain 32	Ttc32	0.00803	-2.87249412	-1.5906217
1425926_a_at	orthodenticle homolog 2 (Drosophila)	Otx2	0.0085	1.79278541	0.8392493
1436619_at	RIKEN cDNA D630045M09 gene	D630045M09Rik	0.00851	-1.58712483	-0.6681766
1439040_at	centromere protein E	Cenpe	0.00868	-1.95244792	-0.9717232
1427275_at	structural maintenance of chromosomes 4	Smc4	0.00877	-1.60344216	-0.6843715
1444142_at	predicted gene 11544	Gm11544	0.00926	-1.92728873	-0.9539331
1416077_at	adrenomedullin	Adm	0.01513	1.78518061	0.83177569
1448123_s_at	transforming growth factor, beta induced	Tgfbi	0.01737	1.57277957	0.6531082
1419106_at	transcription elongation factor B (SIII), polypeptide 2 pseudogene	2210409E12Rik /// Coil	0.01786	-1.84078336	-0.8960134
1420517_at	chromatin modifying protein 4C	Chmp4c	0.01802	-1.87348628	-0.9170856
1423201_at	nuclear receptor co-repressor 1	Ncor1	0.02622	-1.52460747	-0.613368
1425565_at	RE1-silencing transcription factor	Rest	0.02633	-1.62722778	-0.7168854
1438737_at	zinc finger protein of the cerebellum 3	Zic3	0.02697	-1.50291781	-0.5923679
1424784_at	predicted gene 13139	Gm13139	0.02723	-1.48889457	-0.5752382
1422396_s_at	achaete-scute complex homolog 2 (Drosophila)	Ascl2	0.02887	1.56457694	0.64273366
1430309_at	Nipped-B homolog (Drosophila)	Nipbl	0.03148	-1.59953739	-0.6878834
1424800_at	enabled homolog (Drosophila)	Enah	0.03334	-1.346179	-0.4304033
1434286_at	trichorhinophalangeal syndrome I (human)	Trps1	0.03618	-1.72209802	-0.7961177
1428750_at	CDC42 effector protein (Rho GTPase binding) 2	Cdc42ep2	0.03634	1.53698895	0.61684376
1449534_at	synaptonemal complex protein 3	Sycp3	0.03714	-1.58823852	-0.6805553
1427445_a_at	titin	Ttn	0.04023	-1.74498588	-0.828102
1437110_at	RIKEN cDNA 2810474O19 gene	2810474O19Rik	0.04125	-1.66137728	-0.753647
1459989_at	---	---	0.04222	-1.71122989	-0.787717
1442939_at	Rap1 interacting factor 1 homolog (yeast)	Rif1	0.04542	-1.7887097	-0.8713648
1448182_a_at	CD24a antigen	Cd24a	0.04646	1.41527293	0.50091608
1436050_x_at	hairy and enhancer of split 6 (Drosophila)	Hes6	0.04919	1.45872314	0.5431403
1437633_at	ankyrin repeat domain 11	Ankrd11	0.05015	-1.61881514	-0.7150114
1450194_a_at	myeloblastosis oncogene	Myb	0.05106	1.58002311	0.65559166
1421317_x_at	myeloblastosis oncogene	Myb	0.0519	1.49450115	0.5765869
1428604_at	RIKEN cDNA 2610305D13 gene	2610305D13Rik	0.05234	-1.38535024	-0.4746665
1432556_a_at	family with sequence similarity 183, member B	Fam183b	0.05331	1.54774419	0.62805249
1460454_at	glyoxalase domain containing 5	Glod5	0.05434	1.49150466	0.57608288
1417513_at	ecotropic viral integration site 5	Evi5	0.05437	-1.46285725	-0.5565668
1436505_at	peptidyl-prolyl isomerase G (cyclophilin G)	Ppig	0.05458	-1.5116347	-0.6083258
1426243_at	cystathionase (cystathionine gamma-lyase)	Cth	0.05471	-1.30744962	-0.3883288
1435349_at	neuropilin 2	Nrp2	0.0553	1.50006254	0.58282554
1437250_at	melanoregulin	Mreg	0.05569	-1.40601137	-0.4980602
1456239_at	fibroblast growth factor 17	Fgf17	0.05999	-1.41796409	-0.5057607
1427446_s_at	titin	Ttn	0.06086	-1.4512794	-0.5378549
1436746_at	WNK lysine deficient protein kinase 1	Wnk1	0.06106	-1.52005917	-0.6131513
1438688_at	serine/arginine repetitive matrix 2	Srrm2	0.06196	-1.41133104	-0.5035542
1429448_s_at	tet oncogene 1	Tet1	0.06302	-1.40942429	-0.5024964
1452609_at	RIKEN cDNA 1190005I06 gene	1190005I06Rik	0.06499	-1.49623692	-0.5934086
1419647_a_at	immediate early response 3	Ier3	0.07103	1.38363055	0.46629814
1433735_a_at	transmembrane protein 64	Tmem64	0.07395	-1.65107487	-0.7552383
1456250_x_at	transforming growth factor, beta induced	Tgfbi	0.07893	1.41842981	0.50190406
1460364_at	general transcription factor II I repeat domain-containing 1	Gtf2ird1	0.08007	-1.42337081	-0.5145105
1445843_at	chromodomain helicase DNA binding protein 2	Chd2	0.08222	-1.50513407	-0.5971
1452713_a_at	small nuclear ribonucleoprotein 40 (U5)	Snrnp40	0.0858	-1.40427298	-0.5001961
1421399_at	insulinoma-associated 1	Insm1	0.0871	1.82965501	0.83319155
1448107_x_at	kallikrein 1	Klk1	0.08777	1.48628253	0.56859518
1418872_at	ATP-binding cassette, sub-family B (MDR/TAP), member 1B	Abcb1b	0.09495	-1.42414897	-0.5133767
1420366_at	sperm equatorial segment protein 1	Spesp1	0.09553	-1.49291797	-0.5802841
1437791_s_at	echinoderm microtubule associated protein like 5	Eml5	0.09597	-1.55997039	-0.6553931
1428227_at	RE1-silencing transcription factor	Rest	0.09611	-1.39102135	-0.4867723
1428647_at	pre B-cell leukemia transcription factor 1	Pbx1	0.0963	-1.39225761	-0.4774726
1450976_at	N-myc downstream regulated gene 1	Ndrg1	0.09655	1.33338165	0.41454401
1454709_at	transmembrane protein 64	Tmem64	0.09687	-1.33480143	-0.4187605
1419021_at	mcf.2 transforming sequence	Mcf2	0.09724	-1.44909614	-0.5487144
1418430_at	kinesin family member 5B	Kif5b	0.09761	-1.35643028	-0.4452185
1421523_at	fibroblast growth factor 17	Fgf17	0.10084	-1.36091037	-0.4488335
1420729_at	2-cell-stage, variable group, member 1	Tcstv1	0.10299	-1.42212421	-0.5154612
1449848_at	guanine nucleotide binding protein, alpha 14	Gna14	0.10897	1.43935722	0.52124967
1460514_s_at	achaete-scute complex homolog 2 (Drosophila)	Ascl2	0.11111	1.40745033	0.48879997
1416034_at	CD24a antigen	Cd24a	0.11828	1.38912775	0.46678657
1438035_at	family with sequence similarity 82, member A1	Fam82a1	0.12075	-1.49685078	-0.5923944
1427046_at	grainyhead-like 2 (Drosophila)	Grhl2	0.12165	-1.30727982	-0.3868219
1449530_at	trichorhinophalangeal syndrome I (human)	Trps1	0.12399	-1.47512673	-0.5760915
1419562_at	baculoviral IAP repeat-containing 6	Birc6	0.12464	-1.41204171	-0.5095523
1429490_at	Rap1 interacting factor 1 homolog (yeast)	Rif1	0.12469	-1.51906534	-0.6287479
1423756_s_at	insulin-like growth factor binding protein 4	Igfbp4	0.12667	1.40881868	0.49401251
1440929_at	gametogenetin binding protein 2	Ggnbp2	0.13058	-1.39839025	-0.4883638
1449544_a_at	potassium voltage-gated channel, subfamily H (eag-related), mer	Kcnh2	0.131	1.36835575	0.4515857
1420760_s_at	N-myc downstream regulated gene 1	Ndrg1	0.13236	1.34002808	0.41839261
1452021_a_at	hairy and enhancer of split 6 (Drosophila)	Hes6	0.1326	1.39434716	0.47690133
1434151_at	methyltransferase like 7A1	Mettl7a1	0.13268	1.33878268	0.41999561
1416630_at	inhibitor of DNA binding 3	Id3	0.13271	1.42633633	0.50293893
1457445_at	trichorhinophalangeal syndrome I (human)	Trps1	0.13279	-1.45877673	-0.5549499
1428187_at	CD47 antigen (Rh-related antigen, integrin-associated signal trans	Cd47	0.13295	1.56081281	0.62122033
1447807_s_at	pleckstrin homology domain containing, family H (with MyTH4 do	Plekhh1	0.13429	-1.30387749	-0.3834577
1435610_at	family with sequence similarity 19, member A4	Fam19a4	0.13464	1.45825395	0.53553309
1436789_at	cyclin J-like	Ccnjl	0.1368	1.38193564	0.46594604
1416701_at	Rho family GTPase 3	Rnd3	0.13886	1.43654324	0.51580665

1436983_at	CREB binding protein	Crebbp	0.14258	-1.53888491	-0.650216
1421217_a_at	lectin, galactose binding, soluble 9	Lgals9	0.14435	1.33105787	0.40953491
1422884_at	small nuclear ribonucleoprotein D3	Snrpd3	0.14455	-1.25358234	-0.326729
1416432_at	6-phosphofructo-2-kinase/fructose-2,6-biphosphatase 3	Pfkfb3	0.14525	1.38491168	0.46309146
1418648_at	EGL nine homolog 3 (C. elegans)	Egln3	0.14536	1.59854913	0.64034831
1433585_at	transportin 1	Tnpo1	0.14556	-1.24453593	-0.3157914
1451594_s_at	serine (or cysteine) peptidase inhibitor, clade B, member 6c	Serpinb6c	0.1466	-1.39804785	-0.4924393
1425019_at	UBX domain protein 2A	Ubxn2a	0.14671	-1.43069783	-0.5332687
1439824_at	choroidermia	Chm	0.14708	-1.30632223	-0.3870385
1451191_at	cellular retinoic acid binding protein II	Crabp2	0.14712	1.31090785	0.39024514
1454896_at	recombination signal binding protein for immunoglobulin kappa J	Rbpj	0.14722	-1.25223387	-0.324948
1444004_at	THO complex 2	Thoc2	0.14786	-1.33387793	-0.421215
1438214_at	trichorhinophalangeal syndrome I (human)	Trps1	0.14816	-1.49481425	-0.6014166
1434178_at	myeloid/lymphoid or mixed-lineage leukemia 3	Mll3	0.14821	-1.30436153	-0.3855283
1421323_a_at	GTPase activating protein (SH3 domain) binding protein 2	G3bp2	0.14841	-1.22504267	-0.2941619
1422557_s_at	metallothionein 1	Mt1	0.14842	1.24175877	0.30861192
1456599_at	nuclear transport factor 2-like export factor 2	Nxt2	0.14931	-1.37685181	-0.4676571
1436538_at	ankyrin repeat domain 37	Ankrd37	0.14953	1.32130976	0.39890046
1450644_at	zinc finger protein 36, C3H type-like 1	Zfp36l1	0.14977	-1.27454835	-0.3521499
1448908_at	phosphatidic acid phosphatase type 2B	Ppap2b	0.14984	1.36098697	0.44144515
1460005_at	biorientation of chromosomes in cell division 1-like	Bod1l	0.15058	-1.49210625	-0.5988033
1438736_at	THO complex 2	Thoc2	0.15074	-1.32383647	-0.4089321
1451071_a_at	ATPase, Na+/K+ transporting, alpha 1 polypeptide	Atp1a1	0.15074	1.24767804	0.31886277
1438349_at	zinc finger protein	Zfp229	0.15111	-1.36019024	-0.4501261
1428923_at	protein phosphatase 1, regulatory (inhibitor) subunit 3G	Ppp1r3g	0.1518	1.41122055	0.49656545
1455151_at	A kinase (PRKA) anchor protein (yotiao) 9	Akap9	0.15248	-1.41884133	-0.5088038
1434905_at	NADH dehydrogenase (ubiquinone) 1 alpha subcomplex, 4-like 2	Ndufa4l2	0.1526	1.30175084	0.37857495
1423025_a_at	IQ motif containing J-schwannomin interacting protein 1 read-thro	Iqcj-schip1 /// Schip1	0.15266	-1.25561226	-0.328787
1416048_at	polyhomeotic-like 2 (Drosophila)	Phc2	0.153	1.26779893	0.34157884
1442369_at	RIKEN cDNA 4832406H04 gene	4832406H04Rik	0.153	-1.39127931	-0.4829588
1460409_at	carnitine palmitoyltransferase 1a, liver	Cpt1a	0.15304	1.25511077	0.32720462
1429037_at	RIKEN cDNA 1700019A02 gene	1700019A02Rik	0.15306	-1.41319275	-0.4997001
1426759_at	mitogen-activated protein kinase kinase kinase kinase 3	Map4k3	0.15322	-1.25828653	-0.331625
1415837_at	kallikrein 1	Klk1	0.15531	1.67306567	0.70406745
1452517_at	pleckstrin homology domain containing, family H (with MyTH4 do	Plekhh1	0.15964	-1.35735049	-0.4430557
1444058_at	DAZ interacting protein 3, zinc finger	Dzip3	0.15975	-1.39441135	-0.4826134
1418114_at	recombination signal binding protein for immunoglobulin kappa J	Rbpj	0.15997	-1.28338748	-0.3648718
1418566_s_at	NudC domain containing 2	Nudcd2	0.16008	-1.23934078	-0.3107285
1436214_at	RIKEN cDNA 1110028C15 gene	1110028C15Rik	0.16044	-1.34803954	-0.4311918
1422530_at	peripherin	Prph	0.16237	1.31194886	0.39123263
1436759_x_at	calponin 3, acidic	Cnn3	0.16342	-1.27019071	-0.3483189
1415949_at	carboxypeptidase E	Cpe	0.16393	-1.3817344	-0.4712461
1448005_at	SAM and SH3 domain containing 1	Sash1	0.16405	-1.34457064	-0.4355409
1449507_a_at	CD47 antigen (Rh-related antigen, integrin-associated signal trans	Cd47	0.16463	1.33436527	0.41409559
1457033_at	zinc finger and SCAN domain containing 4A	Zscan4a	0.16492	1.31361738	0.38979152
1452077_at	DEAD (Asp-Glu-Ala-Asp) box polypeptide 3, Y-linked	Ddx3y	0.16609	-1.33777163	-0.4267468
1431176_at	chromatin modifying protein 4C	Chmp4c	0.16626	-1.41360402	-0.5070289
1416700_at	Rho family GTPase 3	Rnd3	0.16676	1.38775456	0.46679721
1436794_at	NLR family, pyrin domain containing 4F	Nlrp4f	0.16738	1.39959711	0.4811581
1437694_at	Zinc finger protein 809	Zfp809	0.17015	-1.30095779	-0.3798004
1449279_at	glutathione peroxidase 2	Gpx2	0.17298	1.28860923	0.36532265
1448992_at	internexin neuronal intermediate filament protein, alpha	Ina	0.17815	1.28184955	0.35554675
1424171_a_at	hydroxyacyl glutathione hydrolase	Hagh	0.1783	1.27452546	0.34652289
1451299_at	protein kinase, X-linked	Prkx	0.17844	-1.38138233	-0.4745885
1435176_a_at	inhibitor of DNA binding 2	Id2	0.17882	1.39122178	0.46845076
1418428_at	kinesin family member 5B	Kif5b	0.17943	-1.32365897	-0.4114421
1448244_at	lysophospholipase 1	Lypla1	0.17956	-1.24179599	-0.3128823
1418222_at	RIKEN cDNA 2610024G14 gene	2610024G14Rik	0.18006	1.31198649	0.38766715
1438506_s_at	abl-interactor 1	Abi1	0.18011	-1.37723957	-0.4759195
1417176_at	casein kinase 1, epsilon	Csnk1e	0.18042	1.24309376	0.31232943
1449661_at	Suppressor of zeste 12 homolog (Drosophila)	Suz12	0.18044	-1.48893173	-0.6006734
1424297_at	zinc finger protein 282	Zfp282	0.18055	1.36631544	0.44619182
1454858_x_at	methyltransferase like 7A1	Mettl7a1	0.18081	1.31514619	0.39180714
1455794_at	smoothelin-like 2	Smtnl2	0.18127	1.25103331	0.32199053
1427334_s_at	RIKEN cDNA 2810474O19 gene	2810474O19Rik	0.18127	-1.58738845	-0.7215123
1427347_s_at	tubulin, beta 2A	Tubb2a	0.18129	-1.29940204	-0.3824208
1422524_at	ATP-binding cassette, sub-family B (MDR/TAP), member 6	Abcb6	0.1814	1.27584534	0.35137581
1450457_at	Casitas B-lineage lymphoma	Cbl	0.19177	-1.48830487	-0.6000134
1449140_at	NudC domain containing 2	Nudcd2	0.19272	-1.25574663	-0.3310806
1420043_s_at	THO complex 1	Thoc1	0.19464	-1.38639759	-0.4812476
1425020_at	UBX domain protein 2A	Ubxn2a	0.19528	-1.29144651	-0.3730976
1454708_at	actin-binding LIM protein 1	Ablim1	0.1954	1.33021705	0.40855749
1440984_at	bromodomain adjacent to zinc finger domain, 2B	Baz2b	0.19944	-1.41508327	-0.5146806
1455961_at	---	---	0.19952	-1.30021092	-0.3807643
1415857_at	embigin	Emb	0.19991	-1.27787042	-0.3615105
1454992_at	solute carrier family 7 (cationic amino acid transporter, y+ system	Slc7a1	0.20019	-1.27451793	-0.3520789
1417625_s_at	chemokine (C-X-C motif) receptor 7	Cxcr7	0.20032	1.43254185	0.49930516
1427131_s_at	leucine rich repeat containing 58	Lrrc58	0.2004	-1.26437457	-0.3420209
1419917_s_at	transmembrane emp24 protein transport domain containing 7	Tmed7	0.20042	-1.29628452	-0.3833919
1449164_at	CD68 antigen	Cd68	0.20062	-1.29991838	-0.384829
1435076_at	family with sequence similarity 57, member A	Fam57a	0.20076	1.34117744	0.420964
1437224_at	reticulon 4	Rtn4	0.2008	-1.35124853	-0.4472399
1456174_x_at	N-myc downstream regulated gene 1	Ndrg1	0.20098	1.39013968	0.45771993
1434705_at	C-terminal binding protein 2	Ctbp2	0.20104	-1.21540272	-0.2844634
1433794_at	senataxin	Setx	0.20127	-1.23250088	-0.3021027
1416131_s_at	EFR3 homolog A (S. cerevisiae)	Efr3a	0.20134	-1.32974763	-0.4251234
1427150_at	myeloid/lymphoid or mixed-lineage leukemia 3	Mll3	0.20138	-1.31035151	-0.3945315
1425303_at	glucokinase	Gck	0.20179	1.36777394	0.44604303
1435783_at	family with sequence similarity 169, member A	Fam169a	0.20189	-1.27828019	-0.361567
1438992_x_at	activating transcription factor 4	Atf4	0.20192	-1.3756675	-0.4815245
1420908_at	CD2-associated protein	Cd2ap	0.20193	-1.32639052	-0.4159505
1451536_at	mitochondrial fission regulator 1	Mtfr1	0.20206	-1.25109341	-0.3237871
1418546_a_at	STAM binding protein like 1	Stambpl1	0.20323	-1.32772528	-0.4117201
1451461_a_at	aldolase C, fructose-bisphosphate	Aldoc	0.20425	1.28667874	0.36093565

1449625_at	---	---	0.20504	-1.31368798	-0.3960599
1448797_at	ELK3, member of ETS oncogene family	Elk3	0.20518	1.49466977	0.55223369
1455240_x_at	predicted gene 7969	Gm7969	0.20582	-1.47255435	-0.5777451
1450645_at	metallothionein 4	Mt4	0.20789	1.30143794	0.37894169
1415996_at	thioredoxin interacting protein	Txnip	0.21082	1.24151794	0.3104078
1418515_at	metal response element binding transcription factor 2	Mtf2	0.21123	-1.23340095	-0.3049962
1457248_x_at	hydroxysteroid (17-beta) dehydrogenase 7	Hsd17b7	0.21158	1.30087238	0.37848708
1423424_at	zinc finger protein of the cerebellum 3	Zic3	0.21592	-1.29352891	-0.3806711
1448957_at	recombination signal binding protein for immunoglobulin kappa J	Rbpj	0.21627	-1.23817739	-0.3114414
1426236_a_at	glutamate-ammonia ligase (glutamine synthetase)	Glul	0.21679	-1.24009063	-0.3137403
1437475_at	cDNA sequence BC028454	BC028454	0.21732	-1.31809449	-0.399198
1460570_at	piggyBac transposable element derived 5	Pgbd5	0.21941	1.32136451	0.40134914
1437884_at	ADP-ribosylation factor-like 5B	Arl5b	0.21963	-1.47587862	-0.6013959
1455054_a_at	DCN1, defective in cullin neddylation 1, domain containing 1 (S. ce	Dcun1d1	0.22007	-1.30083008	-0.3809372
1434566_a_at	PRAME family member 8	Pramef8	0.22031	-1.29432957	-0.3781449
1424975_at	sialic acid binding Ig-like lectin 5	Siglec5	0.22334	1.46309169	0.53113257
1428718_at	secernin 1	Scrn1	0.23006	1.35418843	0.43363702
1448842_at	cysteine dioxygenase 1, cytosolic	Cdo1	0.23243	1.37754173	0.45192063
1417216_at	proviral integration site 2	Pim2	0.23339	1.25951051	0.33143083
1437262_x_at	breast carcinoma amplified sequence 2	Bcas2	0.23829	-1.58344383	-0.7296279
1435053_s_at	pleckstrin homology domain containing, family H (with MyTH4 do	Plekhh1	0.23882	-1.30202871	-0.3886865
1455865_at	insulinoma-associated 1	Insm1	0.23961	1.38994134	0.46604557
1450977_s_at	N-myc downstream regulated gene 1	Ndrg1	0.23994	1.23063097	0.29898843
1455129_at	metadherin	Mtdh	0.24122	-1.29400032	-0.3829447
1441807_s_at	---	---	0.24219	-1.36922164	-0.4547251
1444496_at	expressed sequence AA645497	AA645497	0.24225	-1.40043774	-0.4977764
1440193_at	ankyrin repeat domain 12	Ankrd12	0.24303	-1.41033903	-0.5148061
1437267_x_at	Heterogeneous nuclear ribonucleoprotein H1	Hnrnph1	0.24396	-1.36997138	-0.471373
1426840_at	YTH domain family 3	Ythdf3	0.2442	-1.2380401	-0.3118119
1416895_at	ephrin A1	Efna1	0.24451	1.34935218	0.42819591
1448239_at	heme oxygenase (decycling) 1	Hmox1	0.2446	1.26101332	0.33334696
1417508_at	ring finger protein 19A	Rnf19a	0.24492	1.23183139	0.29972064
1455241_at	cDNA sequence BC037703	BC037703	0.24493	1.38393157	0.45840442
1448470_at	fructose bisphosphatase 1	Fbp1	0.24509	1.35210378	0.4282183
1449887_at	chromatin modifying protein 4C	Chmp4c	0.24545	-1.47318158	-0.5906997
1417135_at	serine/arginine-rich protein specific kinase 2	Srpk2	0.24549	-1.2711777	-0.3461874
1434307_at	transmembrane protein 64	Tmem64	0.24557	-1.26946519	-0.347228
1417091_at	conserved helix-loop-helix ubiquitous kinase	Chuk	0.24571	-1.20784487	-0.2725155
1426425_at	SGT1, suppressor of G2 allele of SKP1 (S. cerevisiae)	Sugt1	0.24572	-1.18247994	-0.2424861
1446037_at	---	---	0.24576	-1.29955737	-0.3861033
1449344_s_at	transcription elongation factor B (SIII), polypeptide 2 pseudogene	2210409E12Rik	0.24581	-1.44330455	-0.5582016
1433718_a_at	chromobox homolog 1 (Drosophila HP1 beta)	Cbx1	0.24583	-1.27010163	-0.3537259
1448334_a_at	cyclin I	Ccni	0.24613	-1.28318666	-0.3699012
1417311_at	cysteine rich protein 2	Crip2	0.24625	1.23817367	0.30595134
1417403_at	ELOVL family member 6, elongation of long chain fatty acids (yea	Elovl6	0.24634	-1.25960719	-0.339489
1416965_at	proprotein convertase subtilisin/kexin type 1 inhibitor	Pcsk1n	0.24637	1.31473024	0.38549915
1426438_at	DEAD (Asp-Glu-Ala-Asp) box polypeptide 3, Y-linked	Ddx3y	0.24652	-1.24573532	-0.3194866
1423680_at	fatty acid desaturase 1	Fads1	0.24676	1.26561224	0.3369019
1429206_at	Rho-related BTB domain containing 1	Rhobtb1	0.24728	1.39331896	0.46398058
1418847_at	arginase type II	Arg2	0.24808	-1.38744219	-0.4832884
1440314_at	---	---	0.24832	-1.41141734	-0.5160865
1434528_at	alanine and arginine rich domain containing protein	Aard	0.2493	1.23103703	0.29741316
1438368_a_at	matrin 3	Matr3	0.25024	-1.19643194	-0.2613354
1419415_a_at	retinoic acid receptor, gamma	Rarg	0.25025	-1.214925	-0.2821415
1423653_at	ATPase, Na+/K+ transporting, alpha 1 polypeptide	Atp1a1	0.25078	1.22146035	0.28736067
1437306_at	family with sequence similarity 190, member A	Fam190a	0.25645	-1.31038453	-0.3923769
1439958_at	REX1, RNA exonuclease 1 homolog pseudogene /// REX1, RNA ex	Gm1995 /// Gm7104	0.25684	-1.42069872	-0.5294354
1448029_at	T-box 3	Tbx3	0.25741	-1.22790978	-0.2979911
1427165_at	interleukin 13 receptor, alpha 1	Il13ra1	0.25761	-1.38772415	-0.4872283
1418709_at	cytochrome c oxidase, subunit VIIa 1	Cox7a1	0.25822	1.29546163	0.36595158
1436841_at	family with sequence similarity 63, member B	Fam63b	0.25855	1.36029764	0.43219292
1435083_at	cortexin 1	Ctxn1	0.25861	1.27680998	0.35004712
1433779_at	cancer susceptibility candidate 4	Casc4	0.25875	-1.23890059	-0.3091423
1437020_at	E1A binding protein p400	Ep400	0.25902	-1.37882224	-0.4839285
1428607_at	v-raf murine sarcoma 3611 viral oncogene homolog	Araf	0.25912	-1.30997684	-0.3900376
1433856_at	diphosphoinositol pentakisphosphate kinase 2	Ppip5k2	0.25919	-1.22023638	-0.2876476
1418025_at	basic helix-loop-helix family, member e40	Bhlhe40	0.25932	1.27850757	0.349551
1460223_a_at	erythrocyte protein band 4.9	Epb4.9	0.25964	-1.22982897	-0.301298
1433454_at	ankyrin repeat and BTB (POZ) domain containing 2	Abtb2	0.25967	1.32658604	0.4054256
1433575_at	SRY-box containing gene 4	Sox4	0.26002	-1.20219961	-0.2660708
1448352_at	leucine zipper protein 1	Luzp1	0.26014	-1.29092226	-0.369544
1451190_a_at	SH3-binding kinase 1	Sbk1	0.26021	-1.31600393	-0.4071898
1458510_at	expressed sequence AU019823	AU019823	0.26042	-1.30052158	-0.3821965
1447839_x_at	adrenomedullin	Adm	0.26143	1.29290471	0.36849686
1438036_x_at	family with sequence similarity 82, member A1	Fam82a1	0.26598	-1.30762369	-0.3873725
1429958_x_at	hydroxyacylglutathione hydrolase-like	Haghl	0.26705	1.26725599	0.34146359
1446565_at	---	---	0.27173	-1.33031896	-0.4144539
1427647_at	zyg-11 homolog A (C. elegans)	Zyg11a	0.27207	1.32502014	0.4038688
1449482_at	histone cluster 3, H2ba	Hist3h2ba	0.27237	1.32918197	0.40691497
1448433_a_at	procollagen C-endopeptidase enhancer protein	Pcolce	0.27294	1.24447491	0.31054912
1426270_at	structural maintenance of chromosomes 5	Smc5	0.27399	-1.24298043	-0.3159736
1434279_at	---	---	0.2765	-1.15758917	-0.2126669
1439719_at	thymocyte selection associated	Themis	0.27766	1.28187192	0.35574686
1437463_x_at	transforming growth factor, beta induced	Tgfbi	0.27796	1.33457597	0.40670945
1460597_at	additional sex combs like 2 (Drosophila)	Asxl2	0.28065	-1.25270627	-0.3253388
1435702_s_at	tyrosine 3-monooxygenase/tryptophan 5-monooxygenase activa	Ywhae	0.28216	-1.23226526	-0.3055599
1438833_at	cancer susceptibility candidate 5	Casc5	0.28217	-1.68655583	-0.8646269
1436167_at	Src homology 2 domain containing F	Shf	0.28414	1.31858065	0.39075737
1428410_at	N(alpha)-acetyltransferase 50, NatE catalytic subunit	Naa50	0.28469	-1.23225681	-0.3015867
1460682_s_at	carcinoembryonic antigen-related cell adhesion molecule 1 /// ca	Ceacam1 /// Ceacam2	0.28475	1.30756302	0.37444598
1416326_at	cysteine-rich protein 1 (intestinal)	Crip1	0.28493	1.27775588	0.34254392
1430325_at	expressed sequence AV039307	AV039307	0.28499	1.36925135	0.44173505
1416714_at	interferon regulatory factor 8	Irf8	0.28508	1.46422831	0.52331283
1459854_s_at	dynein light chain Tctex-type 3	Dynlt3	0.28511	-1.30721976	-0.3980422

1449322_at	protein tyrosine phosphatase 4a1-like /// protein tyrosine phospł	Gm13363 /// Ptp4a1	0.28511	-1.20610699	-0.2716954
1436799_at	ecto-NOX disulfide-thiol exchanger 1	Enox1	0.28535	-1.26034226	-0.3431149
1420575_at	metallothionein 3	Mt3	0.28548	1.3196804	0.39070549
1439759_x_at	sulfotransferase family, cytosolic, 6B, member 1	Sult6b1	0.28556	-1.36286819	-0.4629556
1425537_at	protein phosphatase 1A, magnesium dependent, alpha isoform	Ppm1a	0.28578	-1.25251563	-0.3286575
1418304_at	cadherin-related family member 1	Cdhr1	0.28586	1.29606548	0.37300051
1439450_x_at	RIKEN cDNA A230046K03 gene	A230046K03Rik	0.28596	-1.27881173	-0.362117
1460471_at	oocyte expressed protein homolog (dog)	Ooep	0.28601	-1.30963649	-0.4084045
1435245_at	glutaminase 2 (liver, mitochondrial)	Gls2	0.28609	1.23864248	0.30762654
1431644_a_at	islet cell autoantigen 1	Ica1	0.28623	1.27255334	0.34644823
1453285_at	transmembrane protein 88	Tmem88	0.28637	-1.25550648	-0.333132
1455475_at	RIKEN cDNA 3110057O12 gene	3110057O12Rik	0.2865	1.30355522	0.38213332
1422734_a_at	myeloblastosis oncogene	Myb	0.28656	1.50981785	0.56055248
1423462_at	TGF-beta activated kinase 1/MAP3K7 binding protein 2	Tab2	0.28663	-1.20768142	-0.2729351
1426858_at	inhibin beta-B	Inhbb	0.28672	-1.28018325	-0.3697856
1441011_at	---	---	0.28673	1.36662572	0.44106256
1439947_at	cytochrome P450, family 11, subfamily a, polypeptide 1	Cyp11a1	0.28687	1.27733187	0.35190794
1449090_a_at	Yamaguchi sarcoma viral (v-yes) oncogene homolog 1	Yes1	0.2869	-1.23539303	-0.3091768
1460181_at	stathmin-like 3	Stmn3	0.28733	1.27577602	0.34853953
1417385_at	aminopeptidase puromycin sensitive	Npepps	0.28781	-1.29074514	-0.3830917
1435113_x_at	stathmin-like 3	Stmn3	0.28886	1.22579505	0.29300293
1418649_at	EGL nine homolog 3 (C. elegans)	Egln3	0.28942	1.68282773	0.67188788
1454701_at	RIKEN cDNA 4930503L19 gene	4930503L19Rik	0.28962	-1.29437518	-0.3768657
1426440_at	dehydrogenase/reductase (SDR family) member 7	Dhrs7	0.28963	1.26245987	0.33168206
1436247_at	integrator complex subunit 2	Ints2	0.29768	-1.25615472	-0.3326641
1420901_a_at	hexokinase 1	Hk1	0.2992	1.23452225	0.30297655
1422135_at	zinc finger protein 146	Zfp146	0.29983	1.39356297	0.46236068
1422008_a_at	aquaporin 3	Aqp3	0.30133	-1.44765033	-0.5750652
1425415_a_at	solute carrier family 1 (neuronal/epithelial high affinity glutamate	Slc1a1	0.30156	-1.33810267	-0.4267717
1456789_at	zinc finger protein 462	Zfp462	0.3017	-1.2003517	-0.2649166
1452661_at	transferrin receptor	Tfrc	0.30208	1.19524329	0.25377228
1415871_at	transforming growth factor, beta induced	Tgfbi	0.30229	1.33260464	0.40542455
1419772_at	---	---	0.30258	1.27249492	0.34740681
1426419_at	RNA binding motif protein 26	Rbm26	0.30433	-1.27341997	-0.3613268
1434367_s_at	nuclear transport factor 2 /// nuclear transport factor 2, pseudoge	Nutf2 /// Nutf2-ps1	0.30463	-1.12013239	-0.1637584
1452217_at	AHNAK nucleoprotein (desmoyokin)	Ahnak	0.30501	-1.22806945	-0.2969082
1450989_at	teratocarcinoma-derived growth factor 1	Tdgf1	0.30526	-1.15938503	-0.2156493
1438321_x_at	family with sequence similarity 63, member A	Fam63a	0.30549	-1.27227259	-0.3578795
1448688_at	podocalyxin-like	Podxl	0.30682	1.24670712	0.3178759
1427174_at	predicted gene 14325 /// predicted gene 14326 /// predicted ger	Gm14325 /// Gm14326 //	0.30702	-1.21835679	-0.2863169
1438619_x_at	zinc finger, DHHC domain containing 14	Zdhhc14	0.30705	1.32117616	0.39979357
1423924_s_at	tetraspanin 14	Tspan14	0.30708	1.24258419	0.31120273
1452360_a_at	lysine (K)-specific demethylase 5A	Kdm5a	0.30716	-1.52637383	-0.672069
1440177_at	glutamate receptor, ionotropic, kainate 3	Grik3	0.30732	-1.40732209	-0.5267706
1446230_at	---	---	0.30783	-1.28440124	-0.3633385
1418825_at	immunity-related GTPase family M member 1	Irgm1	0.30794	-1.32173207	-0.4101069
1418467_at	SWI/SNF related, matrix associated, actin dependent regulator of	Smarcd3	0.30818	1.27360308	0.34209728
1425675_s_at	carcinoembryonic antigen-related cell adhesion molecule 1	Ceacam1	0.30819	1.42676362	0.4818979
1439367_x_at	ADP-ribosylation factor 4	Arf4	0.30859	-1.22629602	-0.2997736
1456730_x_at	actin-like 6A	Actl6a	0.30868	-1.25318905	-0.3365429
1439972_at	ethanolamine kinase 1	Etnk1	0.3088	-1.34260129	-0.4378295
1457119_at	hypothetical LOC100503442	LOC100503442	0.31039	1.29147428	0.36643715
1448977_at	transcription factor AP-2, gamma	Tcfap2c	0.31047	1.26531621	0.33539804
1415759_a_at	hepatitis B virus x interacting protein	Hbxip	0.31112	1.20581769	0.26836485
1437921_x_at	zinc finger protein 516	Zfp516	0.31117	-1.2328638	-0.3050187
1427256_at	versican	Vcan	0.31139	1.26540175	0.33779252
1448830_at	dual specificity phosphatase 1	Dusp1	0.31158	1.22866924	0.29245894
1426649_at	transmembrane protein with EGF-like and two follistatin-like dom	Tmeff1	0.31205	-1.20417293	-0.268874
1423198_a_at	SMEK homolog 2, suppressor of mek1 (Dictyostelium)	Smek2	0.31299	-1.31977672	-0.4205075
1428877_at	signal recognition particle 72	Srp72	0.3132	-1.35547578	-0.4737852
1456898_at	---	---	0.31327	-1.22552395	-0.295259
1431442_at	hypothetical protein LOC100049077	LOC100049077	0.31353	1.4162885	0.48321738
1448265_x_at	myelin protein zero-like 2	Mpzl2	0.31391	-1.21351574	-0.2814247
1454268_a_at	cytochrome b-245, alpha polypeptide	Cyba	0.31392	1.23380702	0.30062807
1436932_at	grainyhead-like 3 (Drosophila)	Grhl3	0.31394	1.2776293	0.34857979
1454783_at	interleukin 13 receptor, alpha 1	Il13ra1	0.31405	-1.39920343	-0.5045552
1454740_at	mindbomb homolog 1 (Drosophila)	Mib1	0.31417	-1.30704329	-0.4029677
1436386_x_at	RIKEN cDNA 1700029I01 gene /// predicted gene 13139 /// pred	1700029I01Rik /// Gm131	0.31471	-1.43002254	-0.556462
1419083_at	tumor necrosis factor (ligand) superfamily, member 11	Tnfsf11	0.31608	1.31048336	0.38068659
1416444_at	elongation of very long chain fatty acids (FEN1/Elo2, SUR4/Elo3,	Elovl2	0.31616	1.28884491	0.36220095
1436448_a_at	prostaglandin-endoperoxide synthase 1	Ptgs1	0.319	1.36153582	0.4321185
1439264_x_at	LIM and SH3 protein 1	Lasp1	0.31903	-1.22121817	-0.2934198
1442849_at	low density lipoprotein receptor-related protein 1	Lrp1	0.31919	1.3144731	0.39152906
1434329_s_at	adiponectin receptor 2	Adipor2	0.31961	1.1892432	0.24968547
1428630_x_at	hydroxyacylglutathione hydrolase-like	Haghl	0.3198	1.26078683	0.33400708
1459843_s_at	MAD homolog 1 (Drosophila)	Smad1	0.32017	-1.23575491	-0.3078593
1423072_at	RIKEN cDNA 6720475J19 gene	6720475J19Rik	0.32025	-1.28738287	-0.365467
1455046_a_at	pogo transposable element with ZNF domain	Pogz	0.32063	-1.26261826	-0.348366
1415697_at	GTPase activating protein (SH3 domain) binding protein 2	G3bp2	0.32082	-1.19488572	-0.2592821
1447258_at	---	---	0.32082	-1.34386072	-0.4438652
1456402_at	RIKEN cDNA A330076H08 gene	A330076H08Rik	0.32087	1.18003977	0.23809225
1448767_s_at	gap junction protein, beta 1	Gjb1	0.32089	1.25374157	0.32590676
1435588_at	WD repeat and FYVE domain containing 1	Wdfy1	0.32095	-1.25178229	-0.326025
1450994_at	Rho-associated coiled-coil containing protein kinase 1	Rock1	0.32101	-1.19142834	-0.2535712
1428083_at	nuclear paraspeckle assembly transcript 1 (non-protein coding)	Neat1	0.32147	1.21912095	0.28218632
1436519_a_at	RIKEN cDNA 1110057K04 gene	1110057K04Rik	0.32161	-1.31670147	-0.4154699
1436574_at	RIKEN cDNA 1700029I01 gene	1700029I01Rik	0.32171	-1.26125188	-0.3454897
1423135_at	thymus cell antigen 1, theta	Thy1	0.32208	1.25347638	0.32418955
1442027_at	neurobeachin like 1	Nbeal1	0.32227	-1.25310635	-0.3293511
1456147_at	ST8 alpha-N-acetyl-neuraminide alpha-2,8-sialyltransferase 6	St8sia6	0.3224	1.36301003	0.43552001
1434567_at	PRAME family member 8	Pramef8	0.32265	-1.21483264	-0.2812666
1438633_x_at	LIM and SH3 protein 1	Lasp1	0.3228	-1.19867291	-0.2633645
1430700_a_at	phospholipase A2, group VII (platelet-activating factor acetylhydr	Pla2g7	0.32299	1.25270942	0.32462677
1424020_at	ADP-ribosylation factor-like 6 interacting protein 6	Arl6ip6	0.32307	-1.20780878	-0.2754555

1429183_at	plakophilin 2	Pkp2	0.3231	1.19774194	0.25967503
1425149_a_at	phosducin-like	Pdcl	0.32322	-1.21990643	-0.2912139
1452722_a_at	cullin 5	Cul5	0.32329	-1.27473103	-0.3526988
1429624_at	SAFB-like, transcription modulator	Sltm	0.32353	-1.37647744	-0.4935075
1436696_x_at	carcinoembryonic antigen-related cell adhesion molecule 11	Ceacam11	0.32382	1.22623294	0.29163077
1436763_a_at	Kruppel-like factor 9	Klf9	0.32386	-1.32476156	-0.4292155
1418756_at	thyrotropin releasing hormone	Trh	0.3245	1.35870484	0.41507257
1434642_at	hydroxysteroid (17-beta) dehydrogenase 11	Hsd17b11	0.32454	1.23029132	0.29407833
1448289_at	collapsin response mediator protein 1	Crmp1	0.32459	1.22064987	0.28494071
1418189_s_at	metastasis associated lung adenocarcinoma transcript 1 (non-coc	Malat1	0.32483	-1.37721217	-0.4970964
1435994_at	potassium voltage-gated channel, subfamily H (eag-related), mer	Kcnh1	0.32503	1.27806844	0.35306931
1455223_at	insulin-like growth factor 2 mRNA binding protein 1	Igf2bp1	0.32524	-1.19466968	-0.2581811
1432757_at	RIKEN cDNA 2900011L18 gene	2900011L18Rik	0.32552	1.30738342	0.38243989
1426980_s_at	RIKEN cDNA E130012A19 gene	E130012A19Rik	0.32638	-1.33280313	-0.4432115
1448997_at	cytohesin 1	Cyth1	0.32686	1.30161383	0.37753656
1434481_at	male-specific lethal 1 homolog (Drosophila)	Msl1	0.32834	1.26864454	0.33793388
1433679_at	far upstream element (FUSE) binding protein 3	Fubp3	0.32853	-1.19263106	-0.2550907
1442003_at	diaphanous homolog 2 (Drosophila)	Diap2	0.32943	-1.2499036	-0.3232611
1439998_at	jumonji domain containing 1C	Jmjd1c	0.32951	-1.25507317	-0.3278917
1418125_at	INO80 homolog (S. cerevisiae)	Ino80	0.32971	-1.21688252	-0.283484
1418761_at	insulin-like growth factor 2 mRNA binding protein 1	Igf2bp1	0.32975	-1.21942409	-0.2928552
1459041_at	---	---	0.33458	1.20796712	0.26905394
1448458_at	topoisomerase (DNA) II beta	Top2b	0.33499	-1.3897522	-0.5005655
1438316_a_at	coiled-coil domain containing 102A	Ccdc102a	0.335	1.2899266	0.35820481
1455908_a_at	serine carboxypeptidase 1	Scpep1	0.3352	-1.25112001	-0.3362497
1459289_at	---	---	0.33649	-1.27509364	-0.3519371
1457683_at	glutamate receptor, ionotropic, kainate 2 (beta 2)	Grik2	0.33651	1.266194	0.3402761
1425035_s_at	DNA (cytosine-5-)-methyltransferase 3-like	Dnmt3l	0.33652	-1.18662349	-0.2516273
1424369_at	proteasome (prosome, macropain) inhibitor subunit 1	Psmf1	0.33723	1.25892167	0.33106301
1431429_a_at	ADP-ribosylation factor-like 4A	Arl4a	0.33886	1.19906245	0.26019722
1426528_at	neuropilin 2	Nrp2	0.33991	1.30535988	0.37722546
1431804_a_at	trans-acting transcription factor 3	Sp3	0.34009	-1.23327592	-0.3105643
1439464_s_at	testis expressed gene 10	Tex10	0.34052	-1.1874655	-0.2527792
1417388_at	brain expressed X-linked 2	Bex2	0.34105	1.21850644	0.28028762
1428210_s_at	conserved helix-loop-helix ubiquitous kinase	Chuk	0.34122	-1.29774425	-0.3953136
1450380_at	ependymin related protein 1 (zebrafish)	Epdr1	0.3413	1.21938468	0.28606829
1427319_at	RIKEN cDNA A230046K03 gene	A230046K03Rik	0.34137	-1.28701221	-0.3812179
1450140_a_at	cyclin-dependent kinase inhibitor 2A	Cdkn2a	0.34151	1.42042162	0.48376803
1423758_at	GTPase activating protein (SH3 domain) binding protein 2	G3bp2	0.34154	-1.16566618	-0.2227045
1437638_at	serine/arginine repetitive matrix 2	Srrm2	0.34158	-1.34115895	-0.4438686
1437761_at	LUC7-like 2 (S. cerevisiae)	Luc7l2	0.34165	-1.19504552	-0.2593401
1447329_at	---	---	0.34171	1.42917654	0.48987537
1452123_s_at	FERM domain containing 4B	Frmd4b	0.34195	1.24105381	0.31083554
1457094_at	src homology 2 domain-containing transforming protein E	She	0.34215	1.34232649	0.40642175
1423508_at	MYST histone acetyltransferase monocytic leukemia 4	Myst4	0.34264	-1.18952709	-0.2524339
1427991_s_at	ubiquitin specific petidase 45	Usp45	0.34279	-1.23068423	-0.3025745
1439906_at	---	---	0.34306	-1.23622976	-0.3065285
1436072_at	zinc finger protein 826	Zfp826	0.34318	-1.28253079	-0.360329
1440121_at	---	---	0.34347	1.34952724	0.4204999
1452679_at	tubulin, beta 2B	Tubb2b	0.34364	-1.26609464	-0.3581559
1415856_at	embigin	Emb	0.34396	-1.30717027	-0.4071502
1446045_at	---	---	0.34424	-1.26390108	-0.3386531
1457220_at	Ras suppressor protein 1	Rsu1	0.34474	-1.30052253	-0.3817179
1456179_at	predicted gene 13212	Gm13212	0.34501	-1.21437215	-0.2857745
1436038_a_at	phosphatidylinositol glycan anchor biosynthesis, class P	Pigp	0.34531	1.23827513	0.3052563
1431043_at	kelch repeat and BTB (POZ) domain containing 5	Kbtbd5	0.34579	1.27654402	0.35197628
1452256_at	RIKEN cDNA 1110002N22 gene	1110002N22Rik	0.34644	1.19313752	0.25395353
1437305_at	zinc finger protein 770	Zfp770	0.34654	-1.32296052	-0.4151734
1457214_at	---	---	0.34654	-1.27804254	-0.3618889
1459846_x_at	canopy 2 homolog (zebrafish)	Cnpy2	0.34658	-1.18678398	-0.2485507
1418792_at	SH3-domain GRB2-like 2	Sh3gl2	0.3468	-1.22466205	-0.2967149
1450051_at	alpha thalassemia/mental retardation syndrome X-linked homolo	Atrx	0.34711	-1.48159818	-0.6309966
1435800_a_at	cold shock domain protein A	Csda	0.34711	-1.16169476	-0.2178917
1425075_at	GATA zinc finger domain containing 2B	Gatad2b	0.34723	-1.46790664	-0.6178293
1455470_x_at	LIM and SH3 protein 1	Lasp1	0.34731	-1.22006083	-0.295534
1418777_at	chemokine (C-C motif) ligand 25	Ccl25	0.34738	-1.2559315	-0.3302274
1422993_s_at	RNA and export factor binding protein 2 /// THO complex 4	Refbp2 /// Thoc4	0.34744	1.2372733	0.2980706
1421070_at	family with sequence similarity 48, member A	Fam48a	0.34756	-1.27706031	-0.3566888
1429115_at	RIKEN cDNA 2010003O02 gene /// hypothetical LOC100504309	2010003O02Rik /// LOC1(	0.34769	1.29380161	0.36035601
1431800_at	RIKEN cDNA 1300010F03 gene	1300010F03Rik	0.34783	1.36537744	0.43431379
1452300_at	family with sequence similarity 82, member B	Fam82b	0.348	1.29461822	0.36563893
1423590_at	napsin A aspartic peptidase	Napsa	0.34822	1.26430552	0.33171566
1417065_at	early growth response 1	Egr1	0.34863	-1.42584015	-0.570844
1415834_at	dual specificity phosphatase 6	Dusp6	0.34863	1.2581814	0.32160575
1432018_at	achaete-scute complex homolog 2 (Drosophila)	Ascl2	0.34897	1.33016641	0.40199817
1455642_a_at	tetraspanin 17	Tspan17	0.34929	1.2501225	0.31592691
1423551_at	cadherin 13	Cdh13	0.34989	1.27308915	0.34791263
1454231_a_at	retinitis pigmentosa GTPase regulator interacting protein 1	Rpgrip1	0.34999	-1.31858051	-0.4075333
1460588_at	---	---	0.35036	-1.28595603	-0.3631274
1452207_at	Cbp/p300-interacting transactivator, with Glu/Asp-rich carboxy-te	Cited2	0.35659	-1.22367223	-0.2996737
1418645_at	histidine ammonia lyase	Hal	0.35706	1.26656417	0.33946767
1423405_at	tissue inhibitor of metalloproteinase 4	Timp4	0.35738	1.27836867	0.35371833
1452016_at	arachidonate 5-lipoxygenase activating protein	Alox5ap	0.35812	1.2114162	0.27621986
1448794_s_at	DnaJ (Hsp40) homolog, subfamily C, member 2	Dnajc2	0.3605	-1.27132081	-0.3641945
1427689_a_at	TNFAIP3 interacting protein 1	Tnip1	0.36083	1.1855953	0.24501916
1457424_at	eyes absent 1 homolog (Drosophila)	Eya1	0.36102	1.30355828	0.37500656
1416007_at	special AT-rich sequence binding protein 1	Satb1	0.36111	1.32805824	0.38727087
1435135_at	arylacetamide deacetylase-like 1	Nceh1	0.36145	-1.27185306	-0.3487765
1423839_a_at	basic transcription factor 3	Btf3	0.36263	-1.23190978	-0.3149039
1416397_at	mesoderm development candidate 1	Mesdc1	0.36309	1.2203916	0.28373848
1426896_at	zinc finger protein 191	Zfp191	0.36405	1.23893454	0.30590737
1425025_at	transmembrane protein 106A	Tmem106a	0.36419	1.20554886	0.26890053
1455831_at	fusion, derived from t(12;16) malignant liposarcoma (human)	Fus	0.36492	-1.23218001	-0.3012278
1416625_at	serine (or cysteine) peptidase inhibitor, clade G, member 1	Serping1	0.36506	1.23143215	0.29883239

1424349_a_at	lysophosphatidylglycerol acyltransferase 1	Lpgat1	0.36509	-1.2403354	-0.3136098
1431899_at	NK6 homeobox 3	Nkx6-3	0.3653	1.22733372	0.29294117
1448293_at	early B-cell factor 1	Ebf1	0.36565	1.35491125	0.42375429
1424172_at	hydroxyacyl glutathione hydrolase	Hagh	0.36622	1.19093592	0.25074518
1451179_a_at	quaking	Qk	0.36776	-1.18928868	-0.2534835
1449088_at	fructose bisphosphatase 2	Fbp2	0.37263	1.25973251	0.33053562
1435217_at	predicted gene 7969	Gm7969	0.373	-1.40113328	-0.523767
1454787_at	zinc finger, DHHC domain containing 9	Zdhhc9	0.37312	1.24005101	0.30711016
1446358_at	---	---	0.37324	1.24165549	0.31017153
1439216_at	---	---	0.37345	-1.29885898	-0.3875931
1434909_at	Ras-related GTP binding D	Rragd	0.37355	1.22446927	0.29108503
1455692_x_at	RIKEN cDNA 1700097N02 gene	1700097N02Rik	0.37355	1.23633418	0.29702075
1419513_a_at	ect2 oncogene	Ect2	0.37358	-1.17588315	-0.2340931
1456587_x_at	upregulator of cell proliferation	Urgcp	0.37385	1.23938011	0.30941591
1421077_at	SERTA domain containing 3	Sertad3	0.374	1.22995157	0.2955948
1420768_a_at	DEXH (Asp-Glu-X-His) box polypeptide 58	Dhx58	0.374	1.2498334	0.31737192
1426206_at	roundabout homolog 4 (Drosophila)	Robo4	0.37402	-1.26318985	-0.3429448
1452785_at	RIKEN cDNA 1700034H14 gene	1700034H14Rik	0.37427	1.21540898	0.27898947
1457049_at	ATPase, H+ transporting, lysosomal V1 subunit E1	Atp6v1e1	0.3772	1.28833521	0.35852994
1442704_at	---	---	0.37724	1.33412548	0.40157434
1437225_x_at	guanine nucleotide binding protein (G protein), alpha inhibiting 3	Gnai3	0.37742	-1.24640914	-0.3242608
1436684_a_at	RIO kinase 2 (yeast)	Riok2	0.37749	-1.28989911	-0.3932276
1460681_at	carcinoembryonic antigen-related cell adhesion molecule 1	Ceacam1	0.3777	1.2585905	0.32092723
1426317_at	StAR-related lipid transfer (START) domain containing 6	Stard6	0.37783	-1.25311718	-0.3270993
1450891_at	signal recognition particle 19	Srp19	0.37795	-1.1892306	-0.2542769
1459652_at	expressed sequence C79356	C79356	0.37946	1.27396853	0.34912799
1450102_a_at	autocrine motility factor receptor	Amfr	0.37978	1.21101342	0.27561272
1433855_at	4-aminobutyrate aminotransferase	Abat	0.37998	1.29807933	0.37091927
1424672_at	Dmx-like 1	Dmxl1	0.38053	-1.24612546	-0.3196109
1434697_at	DEAD (Asp-Glu-Ala-Asp) box polypeptide 6	Ddx6	0.3808	-1.18958103	-0.251324
1459511_at	---	---	0.38122	-1.25141135	-0.3236127
1427569_a_at	utrophin	Utrn	0.3814	1.26225107	0.32996145
1430808_at	TBC1 domain family, member 5	Tbc1d5	0.38195	1.28245497	0.35592044
1415859_at	eukaryotic translation initiation factor 3, subunit C	Eif3c	0.38318	-1.18403752	-0.2455942
1449310_at	prostaglandin E receptor 2 (subtype EP2)	Ptger2	0.38358	1.3436562	0.4132352
1416488_at	cyclin G2	Ccng2	0.38386	1.2136993	0.27659496
1437239_x_at	polyhomeotic-like 2 (Drosophila)	Phc2	0.3846	1.20585294	0.2698019
1448154_at	N-myc downstream regulated gene 2	Ndrg2	0.38474	1.2275814	0.29143488
1460605_at	Crx opposite strand transcript 1	Crxos1	0.38479	-1.29609154	-0.3822444
1450037_at	ubiquitin specific peptidase 9, X chromosome	Usp9x	0.38505	-1.22882257	-0.3004816
1421307_at	carbonic anhydrase 13	Car13	0.38516	-1.30549496	-0.4008911
1438175_x_at	myomesin 2	Myom2	0.38601	1.28022405	0.35362263
1453146_at	GTPase activating protein and VPS9 domains 1	Gapvd1	0.38631	-1.20816651	-0.275841
1449869_at	pre-B lymphocyte gene 1	Vpreb1	0.38644	-1.29613009	-0.3854373
1437774_at	---	---	0.38646	-1.24550133	-0.3212291
1435492_at	suppressor of cytokine signaling 6	Socs6	0.3865	-1.23982111	-0.3108222
1420609_at	membrane-associated ring finger (C3HC4) 7	Mar-07	0.38667	-1.26956174	-0.364351
1433766_at	N(alpha)-acetyltransferase 25, NatB auxiliary subunit	Naa25	0.38696	-1.20358409	-0.2707505
1452004_at	calcitonin/calcitonin-related polypeptide, alpha	Calca	0.38699	-1.21105021	-0.2814057
1454009_at	RIKEN cDNA 1700008O03 gene	1700008O03Rik	0.38702	1.27589907	0.34911686
1437303_at	interleukin 6 signal transducer	Il6st	0.38704	1.23838763	0.30528384
1429436_at	PRP40 pre-mRNA processing factor 40 homolog A (yeast)	Prpf40a	0.38729	-1.26508188	-0.3536486
1442883_s_at	family with sequence similarity 108, member A	Fam108a	0.38773	1.19540161	0.25404134
1448007_at	bromodomain adjacent to zinc finger domain, 2B	Baz2b	0.38907	-1.25640313	-0.3294398
1437539_at	protein kinase, AMP-activated, alpha 1 catalytic subunit	Prkaa1	0.38944	-1.22473054	-0.2936781
1451451_at	grancalcin	Gca	0.3895	-1.27612284	-0.3519615
1436609_a_at	low density lipoprotein receptor-related protein associated protei	Lrpap1	0.38982	-1.25038682	-0.3388276
1459420_at	---	---	0.39008	-1.29074038	-0.3736569
1423260_at	inhibitor of DNA binding 4	Id4	0.3905	1.23086435	0.29821643
1434666_at	polycomb group ring finger 5	Pcgf5	0.39067	1.25654542	0.32735314
1448460_at	activin A receptor, type 1	Acvr1	0.3907	1.23958178	0.3088824
1429003_at	SNW domain containing 1	Snw1	0.3909	-1.18520642	-0.2483423
1429776_a_at	DnaJ (Hsp40) homolog, subfamily B, member 6	Dnajb6	0.39091	-1.18548647	-0.2466909
1426439_at	DEAD (Asp-Glu-Ala-Asp) box polypeptide 3, Y-linked	Ddx3y	0.39119	-1.26651881	-0.3564683
1447550_at	predicted gene 8350	Gm8350	0.39137	1.3061737	0.37925201
1450896_at	Rho GTPase activating protein 5	Arhgap5	0.39138	-1.29439226	-0.3885071
1436761_s_at	family with sequence similarity 13, member C	Fam13c	0.39153	-1.46870213	-0.6280405
1437500_at	nucleolar complex associated 3 homolog (S. cerevisiae)	Noc3l	0.39163	-1.21041986	-0.2801981
1419642_at	purine rich element binding protein B	Purb	0.39188	-1.24607622	-0.3301372
1422229_at	deubiquitinating enzyme 2a /// ubiquitin specific peptidase, pseu	Dub2a /// Usp-ps /// Usp1	0.39189	-1.26561656	-0.3451104
1438246_at	casein kinase 1, gamma 1	Csnk1g1	0.39205	-1.38359776	-0.5124086
1449899_at	glutamate receptor, ionotropic, NMDA3B	Grin3b	0.39206	-1.28265595	-0.3644656
1431946_a_at	N-terminal EF-hand calcium binding protein 3	Necab3	0.39228	1.28471289	0.35536061
1446114_at	---	---	0.39238	-1.21741655	-0.286928
1450981_at	calponin 2	Cnn2	0.39255	-1.22298408	-0.2973909
1425658_at	CD109 antigen	Cd109	0.39257	1.29187147	0.36457433
1424801_at	enabled homolog (Drosophila)	Enah	0.39257	-1.16592277	-0.2230926
1438444_at	serine peptidase inhibitor, Kazal type 10	Spink10	0.39259	-1.26834717	-0.3442123
1416552_at	developmental pluripotency associated 5A	Dppa5a	0.39271	-1.08237497	-0.114791
1443771_x_at	MAD homolog 7 (Drosophila)	Smad7	0.39276	-1.21587362	-0.2876608
1455141_at	trinucleotide repeat containing 6a	Tnrc6a	0.39277	-1.18024035	-0.2396437
1438180_x_at	HCLS1 associated X-1	Hax1	0.39281	-1.13488605	-0.183061
1416617_at	acyl-CoA synthetase short-chain family member 1	Acss1	0.39282	1.2669534	0.33672975
1459009_at	---	---	0.39295	1.46248063	0.51032057
1452242_at	centrosomal protein 55	Cep55	0.39297	-1.16949301	-0.2261884
1422716_a_at	acid phosphatase 1, soluble	Acp1	0.39461	-1.41728899	-0.5642547
1418402_at	a disintegrin and metallopeptidase domain 19 (meltrin beta)	Adam19	0.39463	1.21128902	0.27325256
1424598_at	DEAD (Asp-Glu-Ala-Asp) box polypeptide 6	Ddx6	0.39472	-1.25692222	-0.3419505
1453293_a_at	RIKEN cDNA 2810408A11 gene	2810408A11Rik	0.39474	1.22334944	0.29065627
1418820_s_at	zinc finger, CCHC domain containing 10	Zcchc10	0.3948	-1.26426808	-0.3568787
1420361_at	solute carrier family 11 (proton-coupled divalent metal ion transp	Slc11a1	0.39527	1.21769649	0.28351634
1445597_s_at	phospholipase A2, group XVI	Pla2g16	0.39556	1.18737531	0.24689913
1436353_at	RIKEN cDNA A230046K03 gene	A230046K03Rik	0.39574	-1.19834935	-0.264668
1451941_a_at	Fc receptor, IgG, low affinity IIb	Fcgr2b	0.39588	1.23424938	0.30308086

1421106_at	jagged 1	Jag1	0.396	1.24138039	0.31125806
1453255_at	solute carrier family 43, member 1	Slc43a1	0.39606	1.23912644	0.30518332
1425245_a_at	regulator of G-protein signaling 11	Rgs11	0.39622	1.25204055	0.32114524
1452807_s_at	family with sequence similarity 57, member B	Fam57b	0.39626	1.35547514	0.41896472
1424445_at	transmembrane 4 superfamily member 5	Tm4sf5	0.3963	1.22207344	0.28799271
1458133_at	---	---	0.39643	-1.26518984	-0.3401455
1455795_at	dermatan sulfate epimerase	Dse	0.39643	-1.31050202	-0.4206187
1441135_at	predicted gene 9900	Gm9900	0.3965	-1.26558888	-0.3407383
1424649_a_at	tetraspanin 8	Tspan8	0.39652	-1.2666258	-0.3456692
1433634_at	interferon regulatory factor 2 binding protein 2	Irf2bp2	0.39658	1.19530716	0.25615464
1452448_at	aquarius	Aqr	0.39662	-1.20872622	-0.2776878
1420773_at	deubiquitinating enzyme 1	Dub1	0.39662	-1.27839731	-0.3643763
1459107_at	potassium voltage-gated channel, subfamily H (eag-related), mer	Kcnh3	0.39668	1.23557863	0.30116607
1448364_at	cyclin G2	Ccng2	0.39676	1.26543346	0.32837265
1454731_at	myosin X	Myo10	0.39685	1.17092601	0.22725988
1437759_at	Phosphofructokinase, platelet	Pfkp	0.39687	-1.26105044	-0.3468903
1417136_s_at	serine/arginine-rich protein specific kinase 2	Srpk2	0.3969	-1.22627112	-0.2944418
1424008_a_at	RNA binding protein with multiple splicing 2	Rbpms2	0.3969	-1.16428007	-0.2197372
1426350_at	mannoside acetylglucosaminyltransferase 2	Mgat2	0.3969	-1.22881317	-0.3038355
1422210_at	forkhead box D3	Foxd3	0.3969	-1.19434954	-0.256441
1429726_at	solute carrier family 16 (monocarboxylic acid transporters), memb	Slc16a9	0.39709	1.2622136	0.33460834
1422910_s_at	structural maintenance of chromosomes 6	Smc6	0.3971	-1.50806048	-0.6900894
1452436_at	lysyl oxidase-like 2	Loxl2	0.39716	1.3718209	0.43299001
1455425_at	tet oncogene 1	Tet1	0.39722	-1.18450517	-0.2483605
1449686_s_at	sterol carrier protein 2, liver	Scp2	0.39734	-1.21719622	-0.2911347
1453015_at	RIKEN cDNA 4933407C03 gene	4933407C03Rik	0.39739	-1.25103544	-0.338264
1433293_at	succinate dehydrogenase complex, subunit A, flavoprotein (Fp)	Sdha	0.39749	-1.2380278	-0.3084293
1422470_at	BCL2/adenovirus E1B interacting protein 3	Bnip3	0.3976	1.17279739	0.22941384
1450343_at	vomeronasal 1 receptor Vmn1r187 /// vomeronasal 1 receptor 6	Vmn1r187 /// Vmn1r63	0.39778	1.22401872	0.29067318
1436124_at	phosphate cytidylyltransferase 1, choline, beta isoform	Pcyt1b	0.39779	1.17735242	0.2355224
1450436_s_at	DnaJ (Hsp40) homolog, subfamily B, member 5	Dnajb5	0.39791	-1.2103384	-0.2760067
1428167_a_at	myelin protein zero-like 1	Mpzl1	0.39852	-1.21946308	-0.2922524
1427004_at	F-box protein 2	Fbxo2	0.3987	1.18862401	0.24626212
1437785_at	a disintegrin-like and metallopeptidase (reprolysin type) with thro	Adamts9	0.4027	1.24691691	0.31694455
1417533_a_at	integrin beta 5	Itgb5	0.40297	1.22249536	0.28500798
1421701_at	vomeronasal 2, receptor 10	Vmn2r10	0.40314	1.25807457	0.32173325
1460462_at	mediator of RNA polymerase II transcription, subunit 18 homolog	Med18	0.40399	-1.17259223	-0.2314243
1419315_at	SLAM family member 9	Slamf9	0.40419	-1.25834546	-0.3383794
1440351_at	---	---	0.40452	-1.25545654	-0.3315128
1439246_x_at	trinucleotide repeat containing 6a	Tnrc6a	0.40481	-1.23325727	-0.3041587
1455406_at	calcium/calmodulin-dependent serine protein kinase (MAGUK fan	Cask	0.40605	-1.30742817	-0.4041615
1427238_at	F-box protein 15	Fbxo15	0.40607	-1.18287885	-0.245918
1452222_at	utrophin	Utrn	0.408	1.19673975	0.25568081
1418824_at	ADP-ribosylation factor 6	Arf6	0.40836	1.28316406	0.35404301
1434452_x_at	eukaryotic translation initiation factor 2a	Eif2a	0.40918	-1.38070592	-0.525165
1449454_at	bone marrow stromal cell antigen 1	Bst1	0.40919	1.23935489	0.30623577
1452259_at	PHD finger protein 20	Phf20	0.40925	-1.31576164	-0.4328164
1416916_at	E74-like factor 3	Elf3	0.40927	1.19460236	0.25603636
1423180_at	potassium voltage gated channel, Shab-related subfamily, memb	Kcnb1	0.40948	1.26309319	0.33612552
1421852_at	potassium channel, subfamily K, member 5	Kcnk5	0.4096	1.18945878	0.24982722
1436298_x_at	phosphoribosylaminoimidazole carboxylase, phosphoribosylamin	Paics	0.40976	-1.35508511	-0.4891482
1438324_at	RIKEN cDNA 9330182L06 gene	9330182L06Rik	0.40983	-1.1973657	-0.2601473
1441498_at	---	---	0.40995	-1.30660045	-0.4038687
1455944_at	zinc finger protein 516	Zfp516	0.41008	-1.23396097	-0.3050861
1422573_at	adenosine monophosphate deaminase 3	Ampd3	0.41013	1.21210605	0.27685876
1448996_at	rod outer segment membrane protein 1	Rom1	0.41018	1.26278717	0.33346355
1437461_s_at	RNA-binding region (RNP1, RRM) containing 3	Rnpc3	0.41036	-1.25051733	-0.335179
1447181_s_at	solute carrier family 7 (cationic amino acid transporter, y+ system	Slc7a7	0.41316	-1.17016489	-0.2277215
1443018_at	---	---	0.41354	-1.27327733	-0.3515618
1453243_at	RIKEN cDNA 0610030E20 gene	0610030E20Rik	0.41381	-1.24899405	-0.3239142
1420385_at	guanine nucleotide binding protein, alpha 14	Gna14	0.41402	1.25650067	0.32791932
1418752_at	aldehyde dehydrogenase family 3, subfamily A1	Aldh3a1	0.41406	1.29940871	0.37074098
1426033_at	regulator of G-protein signaling 9	Rgs9	0.41443	-1.25806951	-0.3363932
1434475_at	peptidyl-prolyl isomerase G (cyclophilin G)	Ppig	0.41471	-1.2908125	-0.38731
1445207_at	---	---	0.41492	1.26732857	0.3344531
1439245_at	trinucleotide repeat containing 6a	Tnrc6a	0.41509	-1.26502026	-0.3431875
1420907_at	CD2-associated protein	Cd2ap	0.41526	-1.16992892	-0.2285103
1417196_s_at	WW, C2 and coiled-coil domain containing 2	Wwc2	0.41535	-1.21158365	-0.2770704
1448235_s_at	RIKEN cDNA 4932431P20 gene /// high-mobility group (nonhistor	4932431P20Rik /// Hmg1	0.41554	-1.36925303	-0.5081967
1416041_at	serum/glucocorticoid regulated kinase 1	Sgk1	0.41587	-1.16331929	-0.2195815
1448452_at	interferon regulatory factor 8	Irf8	0.41626	1.2695945	0.34146136
1451120_at	polymerase (RNA) I polypeptide D	Polr1d	0.4166	1.15437817	0.20654819
1458599_at	---	---	0.41675	-1.26740626	-0.3499598
1443825_x_at	sperm acrosome associated 3	Spaca3	0.41679	-1.26442993	-0.346926
1449600_at	predicted gene 3716	Gm3716	0.41685	1.25959893	0.33007244
1438735_at	remodeling and spacing factor 1	Rsf1	0.41702	-1.21056983	-0.2772799
1455432_at	TAO kinase 1	Taok1	0.41784	-1.19879455	-0.2655271
1416569_at	actin-like 6A	Actl6a	0.41799	-1.1696893	-0.2281773
1427382_a_at	suppressor of variegation 3-9 homolog 1 (Drosophila)	Suv39h1	0.41838	1.21321135	0.27802138
1417234_at	matrix metallopeptidase 11	Mmp11	0.41852	1.20294446	0.26398889
1436848_x_at	inositol (myo)-1(or 4)-monophosphatase 1	Impa1	0.41859	-1.24252984	-0.326687
1416481_s_at	HIG1 domain family, member 1A	Higd1a	0.41887	1.22918748	0.29535257
1419595_a_at	gamma-glutamyl hydrolase	Ggh	0.41901	-1.21346332	-0.2799964
1438076_at	---	---	0.41911	-1.24504606	-0.3188293
1428262_s_at	predicted gene 11847 /// heterogeneous nuclear ribonucleoprote	Gm11847 /// Hnrnpa3	0.4192	-1.14562052	-0.1972084
1448443_at	serine (or cysteine) peptidase inhibitor, clade I, member 1	Serpini1	0.41961	1.23927963	0.30399064
1452008_at	tetratricopeptide repeat domain 39B	Ttc39b	0.41996	-1.23193316	-0.3075157
1428169_at	autophagy-related 16-like 1 (yeast)	Atg16l1	0.42014	1.19943485	0.25779603
1437147_at	gamma-aminobutyric acid (GABA) A receptor, subunit gamma 2	Gabrg2	0.42035	1.19149768	0.25128796
1438237_at	predicted gene 13138 /// predicted gene 13242 /// reduced expr	Gm13138 /// Gm13242 //	0.42042	-1.11996048	-0.1647379
1450475_at	distal-less homeobox 3	Dlx3	0.42043	-1.25831564	-0.3353065
1438695_at	RIKEN cDNA C230091D08 gene	C230091D08Rik	0.42044	-1.20003166	-0.2687752
1449363_at	activating transcription factor 3	Atf3	0.42061	-1.22399522	-0.2948509
1450861_at	Fanconi anemia, complementation group C	Fancc	0.42067	1.24076784	0.30559003

1440553_at	mitochondrial trans-2-enoyl-CoA reductase	Mecr	0.42073	-1.2942117	-0.3850761
1436600_at	TOX high mobility group box family member 3	Tox3	0.42088	1.25373679	0.32134236
1453072_at	G protein-coupled receptor 160	Gpr160	0.42089	-1.21062251	-0.2762618
1424671_at	pleckstrin homology domain containing, family F (with FYVE dom	Plekhf1	0.42093	1.19373009	0.25488616
1449204_at	gap junction protein, beta 5	Gjb5	0.421	1.17526535	0.23242098
1454254_s_at	RIKEN cDNA 1600029D21 gene	1600029D21Rik	0.42115	1.21382753	0.276894
1439198_at	---	---	0.42116	1.24585329	0.31336594
1427812_at	iduronate 2-sulfatase	Ids	0.42125	-1.24095394	-0.3115615
1419304_at	brachyury	T	0.42132	1.25018144	0.32065889
1451967_x_at	karyopherin (importin) beta 1	Kpnb1	0.42149	-1.22287691	-0.3071107
1437405_a_at	insulin-like growth factor binding protein 4	Igfbp4	0.4217	1.21146747	0.27472684
1456200_at	inositol polyphosphate multikinase	Ipmk	0.42181	-1.2118924	-0.283119
1456112_at	translocated promoter region	Tpr	0.42196	-1.22653365	-0.3092561
1426696_at	low density lipoprotein receptor-related protein associated protei	Lrpap1	0.42208	-1.2424823	-0.3288665
1417632_at	ATPase, H+ transporting, lysosomal V0 subunit A1	Atp6v0a1	0.42225	1.20459422	0.26438214
1435884_at	intersectin 1 (SH3 domain protein 1A)	Itsn1	0.42237	-1.26421551	-0.3490889
1426782_at	G protein-coupled receptor 125	Gpr125	0.42279	-1.20753614	-0.2793764
1439357_at	interleukin 17 receptor E	Il17re	0.4228	-1.24440924	-0.3160665
1447788_s_at	TSPY-like 3	Tspyl3	0.42316	1.27617414	0.34412167
1449434_at	carbonic anhydrase 3	Car3	0.42525	1.24585698	0.31289937
1425319_s_at	small nuclear ribonucleoprotein 48 (U11/U12)	Snrnp48	0.42554	-1.18917674	-0.2524984
1422728_at	inhibin alpha	Inha	0.42563	1.24793993	0.31880614
1436339_at	RIKEN cDNA 1810058I24 gene	1810058I24Rik	0.42599	1.1832574	0.23819442
1415902_at	aldehyde dehydrogenase family 7, member A1	Aldh7a1	0.42616	-1.23900468	-0.3119521
1452937_s_at	coiled coil domain containing 28B	Ccdc28b	0.42802	1.23652838	0.30177618
1416422_a_at	Sjogren syndrome antigen B	Ssb	0.42834	-1.25018997	-0.3388803
1449071_at	myosin, light polypeptide 7, regulatory	Myl7	0.42838	1.27065264	0.33503827
1421339_at	exostoses (multiple)-like 3	Extl3	0.42962	1.27370236	0.34276365
1422492_at	coproporphyrinogen oxidase	Cpox	0.42984	-1.22570503	-0.3044022
1427242_at	DEAD (Asp-Glu-Ala-Asp) box polypeptide 4	Ddx4	0.42995	-1.20615934	-0.2747068
1451335_at	placenta-specific 8	Plac8	0.43029	-1.21339776	-0.2809694
1423809_at	transcription factor 19	Tcf19	0.43173	1.1930928	0.25378354
1437478_s_at	EF hand domain containing 2	Efhd2	0.43183	-1.20944187	-0.2773184
1421816_at	glutathione reductase	Gsr	0.43224	1.18510428	0.24168384
1453681_at	ATPase inhibitory factor 1	Atpif1	0.43234	-1.23889322	-0.3158763
1443504_at	hypothetical protein E330022O07	E330022O07	0.43252	-1.23831876	-0.3104196
1458165_at	importin 7	Ipo7	0.43286	1.29555077	0.36366878
1456778_at	---	---	0.43327	1.23846268	0.30793457
1416267_at	short coiled-coil protein	Scoc	0.43338	-1.21685611	-0.2908896
1419896_at	tet oncogene 1	Tet1	0.43339	-1.26308949	-0.3552719
1428584_a_at	hydroxyacylglutathione hydrolase-like	Haghl	0.4349	1.19074825	0.25115142
1416715_at	gap junction protein, beta 3	Gjb3	0.43547	1.22318674	0.28226627
1448318_at	perilipin 2	Plin2	0.43603	1.20149779	0.25990529
1443239_at	---	---	0.43606	1.19831057	0.25862111
1458447_at	centromere protein F	Cenpf	0.43606	-1.29194556	-0.3827529
1454903_at	nerve growth factor receptor (TNFR superfamily, member 16)	Ngfr	0.43634	1.20024242	0.25804728
1459239_at	---	---	0.4364	1.18507831	0.24403662
1423005_a_at	espin	Espn	0.43656	1.31052743	0.37715841
1456842_at	Bol, boule-like (Drosophila)	Boll	0.43656	-1.18272857	-0.2439389
1425347_a_at	zinc finger protein 318	Zfp318	0.43657	-1.30563961	-0.4113255
1447861_x_at	Meis homeobox 2	Meis2	0.43675	-1.2952939	-0.3820895
1437082_at	A kinase (PRKA) anchor protein (yotiao) 9	Akap9	0.43685	-1.39323999	-0.5294621
1450886_at	germ cell-specific gene 2	Gsg2	0.43694	-1.1970013	-0.2612485
1452666_a_at	transmembrane and coiled-coil domains 2	Tmcc2	0.43698	1.22015385	0.27163486
1458830_at	---	---	0.43807	1.20757855	0.26915924
1428693_at	RIKEN cDNA 2610044O15 gene	2610044O15Rik	0.43813	-1.2794266	-0.3692318
1450774_at	lymphocyte antigen 6 complex, locus G6D	Ly6g6d	0.43821	1.29162862	0.35704254
1425523_at	RNA binding motif protein 25	Rbm25	0.43848	-1.18447053	-0.2484649
1437974_a_at	hexokinase 1	Hk1	0.43849	1.15763857	0.21042116
1426924_at	ring finger and CCCH-type zinc finger domains 2	Rc3h2	0.43852	-1.16934222	-0.2262099
1427936_at	threonine synthase-like 1 (bacterial)	Thnsl1	0.43855	1.22989809	0.29491056
1434084_at	RIKEN cDNA 5730601F06 gene	5730601F06Rik	0.43859	-1.17356545	-0.2326236
1417206_at	uroporphyrinogen decarboxylase	Urod	0.43864	1.17105086	0.22515984
1419554_at	CD47 antigen (Rh-related antigen, integrin-associated signal trans	Cd47	0.43865	1.32589275	0.38565131
1437471_at	leucine rich repeat containing 45	Lrrc45	0.43865	1.20657923	0.27009585
1418496_at	forkhead box A1	Foxa1	0.43872	1.24404856	0.31276852
1433934_at	Sec24 related gene family, member A (S. cerevisiae)	Sec24a	0.43894	-1.17963358	-0.2409297
1454201_a_at	cyclin Y	Ccny	0.43896	1.23175806	0.29762454
1430568_at	zinc finger CCCH type containing 13	Zc3h13	0.43897	-1.24442027	-0.3176685
1448473_at	budding uninhibited by benzimidazoles 3 homolog (S. cerevisiae)	Bub3	0.43901	1.168859	0.22502617
1435493_at	desmoplakin	Dsp	0.43907	-1.30974313	-0.4177168
1422264_s_at	Kruppel-like factor 9	Klf9	0.43909	-1.30080722	-0.4126834
1427315_at	transmembrane emp24 protein transport domain containing 7	Tmed7	0.43913	-1.19886397	-0.2621328
1424287_at	protein kinase, X-linked	Prkx	0.43921	-1.30202236	-0.4010698
1423810_at	protein phosphatase methylesterase 1	Ppme1	0.43924	1.1685807	0.22249071
1448905_at	mitochondrial ribosomal protein S34 /// non-metastatic cells 3, pr	Mrps34 /// Nme3	0.43926	1.20854081	0.26806226
1426599_a_at	solute carrier family 2 (facilitated glucose transporter), member 1	Slc2a1	0.43927	1.15518456	0.20474501
1452843_at	interleukin 6 signal transducer	Il6st	0.43937	1.17612536	0.23399235
1439852_at	---	---	0.43938	1.21967989	0.28582506
1440248_at	cancer susceptibility candidate 4	Casc4	0.43941	-1.20613764	-0.2729609
1434888_a_at	matrin 3	Matr3	0.43945	-1.15559925	-0.2100486
1446295_at	tripartite motif-containing 24	Trim24	0.43956	-1.23158025	-0.3011706
1452150_at	expressed sequence AU040320	AU040320	0.43959	1.22884729	0.29504319
1424292_at	DEP domain containing 1a	Depdc1a	0.43962	-1.20601931	-0.2733298
1428660_s_at	torsin family 3, member A	Tor3a	0.43963	1.20506362	0.26641415
1418860_a_at	LETM1 domain containing 1	Letmd1	0.43964	1.23869946	0.30848773
1416590_a_at	RAB34, member of RAS oncogene family	Rab34	0.43966	1.18511302	0.24309348
1437982_x_at	COX15 homolog, cytochrome c oxidase assembly protein (yeast)	Cox15	0.43966	-1.26865225	-0.3663988
1417864_at	phosphoglycerate kinase 1	Pgk1	0.43967	1.10741372	0.14584881
1430216_at	zinc finger protein 292	Zfp292	0.43969	-1.25637151	-0.335156
1437995_x_at	septin 7	Sep-07	0.43975	-1.23018607	-0.3168166
1458486_at	---	---	0.43977	1.2104501	0.2753582
1422622_at	nitric oxide synthase 3, endothelial cell	Nos3	0.4398	1.19183754	0.25139736
1431260_at	RIKEN cDNA 4833417C18 gene	4833417C18Rik	0.43995	-1.23593624	-0.3059235

1456311_x_at	establishment of cohesion 1 homolog 2 (S. cerevisiae)	Esco2	0.43995	-1.22024458	-0.2874144
1438009_at	predicted gene 11276 /// histone cluster 1, H2ao	Gm11276 /// Hist1h2ao	0.43998	1.09670374	0.13086902
1441494_at	---	---	0.43998	1.25282642	0.31928028
1429135_at	RIKEN cDNA 1110059M19 gene	1110059M19Rik	0.44001	-1.24907277	-0.3227698
1416447_at	transmembrane protein 30A	Tmem30a	0.44003	-1.17657491	-0.2353696
1427889_at	spectrin alpha 2	Spna2	0.44008	-1.16314583	-0.2193768
1447914_x_at	proline rich 5 like	Prr5l	0.44009	1.23143813	0.29811366
1447908_x_at	tetratricopeptide repeat domain 3	Ttc3	0.44011	-1.18696283	-0.248241
1429833_at	lymphocyte antigen 6 complex, locus G6E	Ly6g6e	0.44015	1.22198936	0.28723698
1439415_x_at	ribosomal protein S21	Rps21	0.44021	-1.25099254	-0.3437027
1452707_at	kelch-like 30 (Drosophila)	Klhl30	0.44021	1.23992681	0.30907434
1429628_at	RIKEN cDNA 6330407J23 gene	6330407J23Rik	0.44023	-1.19239113	-0.2548994
1448754_at	retinol binding protein 1, cellular	Rbp1	0.44025	1.24773513	0.30811274
1434819_at	beta galactoside alpha 2,6 sialyltransferase 2	St6gal2	0.44027	1.19902197	0.26070066
1432171_at	RIKEN cDNA 4933409F18 gene	4933409F18Rik	0.44027	1.23923946	0.30718822
1419280_at	phosphatidylinositol-5-phosphate 4-kinase, type II, alpha	Pip4k2a	0.44029	-1.24652519	-0.3224707
1455905_at	RIKEN cDNA 2610507B11 gene	2610507B11Rik	0.4403	-1.23476055	-0.3098674
1417461_at	CAP, adenylate cyclase-associated protein 1 (yeast)	Cap1	0.44033	1.14843001	0.1995523
1455885_at	archaelysin family metallopeptidase 1	Amz1	0.44035	-1.27271346	-0.3667421
1429656_at	Rho-related BTB domain containing 1	Rhobtb1	0.44044	1.26511605	0.33501752
1454672_at	neurofilament, light polypeptide	Nefl	0.44048	1.22513966	0.29056998
1425182_x_at	kallikrein 1-related peptidase b22 /// kallikrein 1-related peptidas	Klk1b22 /// Klk1b9	0.4405	1.22113467	0.28660374
1451425_a_at	makorin, ring finger protein, 1	Mkrn1	0.44052	-1.15312841	-0.2063175
1424398_at	DEAH (Asp-Glu-Ala-His) box polypeptide 36	Dhx36	0.44054	-1.22634794	-0.3086929
1450992_a_at	Meis homeobox 1	Meis1	0.4406	1.31133696	0.37811042
1442166_at	copine V	Cpne5	0.44061	1.17251417	0.22871096
1458354_x_at	keratin 28	Krt28	0.44062	1.22456502	0.29067222
1426600_at	solute carrier family 2 (facilitated glucose transporter), member 1	Slc2a1	0.44063	1.20239109	0.25488091
1452514_a_at	kit oncogene	Kit	0.44068	1.22165293	0.27845549
1454180_at	armadillo repeat containing 2	Armc2	0.44071	-1.28709733	-0.3797362
1448991_a_at	internexin neuronal intermediate filament protein, alpha	Ina	0.44074	1.17600571	0.23250812
1427641_at	---	---	0.44077	-1.27676293	-0.3674878
1453013_at	zinc finger protein 740	Zfp740	0.44078	-1.15123233	-0.2032164
1438916_x_at	RIKEN cDNA 6720401G13 gene	6720401G13Rik	0.44083	1.29059652	0.35812612
1415708_at	taurine upregulated gene 1	Tug1	0.44086	-1.1829237	-0.2486791
1434025_at	---	---	0.44091	-1.14500422	-0.1977895
1430341_at	nudix (nucleoside diphosphate linked moiety X)-type motif 5	Nudt5	0.44097	-1.27658452	-0.3625465
1454159_a_at	insulin-like growth factor binding protein 2	Igfbp2	0.44097	1.14892408	0.19790125
1437880_at	SKI family transcriptional corepressor 1	Skor1	0.44101	-1.28767197	-0.3806298
1416144_a_at	DEAH (Asp-Glu-Ala-His) box polypeptide 15	Dhx15	0.44104	-1.14073477	-0.1918802
1438977_x_at	RAN, member RAS oncogene family	Ran	0.44105	-1.15023111	-0.2091435
1425312_s_at	cleavage and polyadenylation specific factor 4-like	Cpsf4l	0.44106	1.21827808	0.27633086
1428332_at	phosphoinositide-3-kinase interacting protein 1	Pik3ip1	0.44109	1.23761567	0.30300617
1452936_at	cartilage acidic protein 1	Crtac1	0.44114	1.25380832	0.32393233
1423142_a_at	GTP binding protein 4	Gtpbp4	0.44115	-1.34001602	-0.4732491
1435128_at	brain-specific angiogenesis inhibitor 1-associated protein 2	Baiap2	0.44119	1.23112859	0.29453255
1417695_a_at	sterol O-acyltransferase 1	Soat1	0.4412	-1.22287324	-0.2910296
1442577_at	RIKEN cDNA C330006K01 gene	C330006K01Rik	0.44128	1.25746075	0.32866288
1422537_a_at	inhibitor of DNA binding 2	Id2	0.44135	1.24923646	0.31232912
1420463_at	cytokine-dependent hematopoietic cell linker	Clnk	0.44136	1.242289	0.31271729
1443897_at	DNA-damage inducible transcript 3	Ddit3	0.44136	-1.26246986	-0.3463885
1451770_s_at	DEAH (Asp-Glu-Ala-His) box polypeptide 9	Dhx9	0.44148	-1.16264261	-0.2198681
1449519_at	growth arrest and DNA-damage-inducible 45 alpha	Gadd45a	0.44151	-1.16855739	-0.226426
1454793_x_at	DEAD (Asp-Glu-Ala-Asp) box polypeptide 5	Ddx5	0.44154	-1.23434527	-0.3241914
1417394_at	Kruppel-like factor 4 (gut)	Klf4	0.44158	-1.15392961	-0.2066234
1456702_x_at	methionine adenosyltransferase II, alpha	Mat2a	0.44172	-1.19154203	-0.2603637
1429514_at	phosphatidic acid phosphatase type 2B	Ppap2b	0.4418	1.21210932	0.27352467
1426037_a_at	regulator of G-protein signaling 16	Rgs16	0.44182	1.26523863	0.33563682
1436708_x_at	minichromosome maintenance deficient 4 homolog (S. cerevisiae	Mcm4	0.44183	-1.20501202	-0.2810468
1435914_at	nuclear receptor co-repressor 1	Ncor1	0.44243	-1.24744154	-0.3322581
1456386_at	---	---	0.44253	-1.23519135	-0.3058338
1441321_at	hypothetical LOC552911	LOC552911	0.44262	-1.2357735	-0.3105277
1437850_a_at	cellular nucleic acid binding protein	Cnbp	0.4428	-1.34018236	-0.478761
1447783_x_at	solute carrier family 25, member 39	Slc25a39	0.44304	-1.16448372	-0.2199787
1448951_at	tumor necrosis factor receptor superfamily, member 1b	Tnfrsf1b	0.44413	-1.19154469	-0.2534555
1456225_x_at	tribbles homolog 3 (Drosophila)	Trib3	0.44556	-1.27401334	-0.3786377
1416897_at	poly (ADP-ribose) polymerase family, member 9	Parp9	0.44572	1.20113177	0.26403429
1431211_s_at	thioesterase superfamily member 5	Them5	0.44575	1.28977963	0.35648118
1434832_at	forkhead box O3	Foxo3	0.44576	-1.19247428	-0.2575116
1454843_at	phosphoribosyl pyrophosphate synthetase 2	Prps2	0.44586	-1.17554422	-0.2363875
1438487_s_at	zinc finger, ZZ domain containing 3	Zzz3	0.44601	-1.20474861	-0.2767146
1440007_at	RIKEN cDNA D930003E18 gene	D930003E18Rik	0.44601	1.2395335	0.3089465
1425564_at	RE1-silencing transcription factor	Rest	0.44606	-1.2337035	-0.3101562
1434108_at	F-box protein 11	Fbxo11	0.44612	-1.17514924	-0.2331263
1436920_at	protocadherin 17	Pcdh17	0.44626	1.21100721	0.27617494
1420189_at	---	---	0.44628	1.23288214	0.30044309
1437935_at	meiosis-specific, MEI4 homolog (S. cerevisiae)	Mei4	0.44632	-1.26209669	-0.348739
1422536_at	troponin I, cardiac 3	Tnni3	0.44645	1.20735077	0.27046824
1441788_s_at	dyskeratosis congenita 1, dyskerin homolog (human)	Dkc1	0.44654	-1.16690905	-0.2274318
1423792_a_at	CKLF-like MARVEL transmembrane domain containing 6	Cmtm6	0.4466	-1.18134324	-0.2431296
1450248_at	a disintegrin and metallopeptidase domain 11	Adam11	0.44678	1.29278584	0.36135638
1416416_x_at	glutathione S-transferase, mu 1	Gstm1	0.44699	1.16656892	0.22171833
1426269_at	vesicle-associated membrane protein 7	Vamp7	0.44731	-1.18439802	-0.2447176
1428383_a_at	RIKEN cDNA 2310021P13 gene	2310021P13Rik	0.44748	1.34545353	0.40176088
1437983_at	sal-like 1 (Drosophila)	Sall1	0.44779	-1.16474759	-0.2212901
1450942_at	zinc finger protein 830	Zfp830	0.44788	-1.21339656	-0.2803966
1444275_at	---	---	0.44795	1.18357541	0.24300282
1429521_at	alkB, alkylation repair homolog 8 (E. coli)	Alkbh8	0.44823	-1.24540893	-0.3333167
1424868_at	glycine-N-acyltransferase	Glyat	0.44824	1.25409089	0.32403407
1455214_at	microphthalmia-associated transcription factor	Mitf	0.44838	1.18376283	0.24247946
1419368_a_at	ring finger protein 138	Rnf138	0.4484	-1.19386269	-0.2616985
1426276_at	interferon induced with helicase C domain 1	Ifih1	0.44847	-1.28654389	-0.3811005
1436236_x_at	coactosin-like 1 (Dictyostelium)	Cotl1	0.44848	1.15871343	0.21235753
1458350_at	optic atrophy 3 (human)	Opa3	0.44856	-1.23683305	-0.3088766

1426695_at	RIKEN cDNA 9030624J02 gene	9030624J02Rik	0.44875	-1.25817226	-0.3371566
1452678_a_at	cysteine conjugate-beta lyase 1	Ccbl1	0.44884	1.24332142	0.30907813
1427477_at	transmembrane protease, serine 13	Tmprss13	0.44885	-1.23576516	-0.3079802
1432160_at	G protein-coupled receptor kinase-interactor 2	Git2	0.44908	-1.23361446	-0.3038915
1429177_x_at	SRY-box containing gene 17	Sox17	0.44917	-1.22202798	-0.2897946
1429795_at	RIKEN cDNA 1700001L05 gene	1700001L05Rik	0.4495	1.21403378	0.27475749
1451149_at	phosphoglucomutase 2	Pgm2	0.44954	1.15502898	0.20697704
1434687_at	hypothetical protein C730026J16 /// zinc finger and BTB domain c	C730026J16 /// Zbtb38	0.45004	-1.24338416	-0.3209243
1436569_at	phosphatidylinositol-3,4,5-trisphosphate-dependent Rac exchang	Prex2	0.45007	-1.28267724	-0.375956
1429063_s_at	kinesin family member 16B	Kif16b	0.45013	-1.21184624	-0.2797975
1429634_at	zinc finger protein 580	Zfp580	0.45013	1.15797923	0.20360565
1421624_a_at	enabled homolog (Drosophila)	Enah	0.45028	-1.18992869	-0.2554576
1454622_at	solute carrier family 38, member 5	Slc38a5	0.45056	-1.24010011	-0.317273
1419145_at	smoothelin-like 1	Smtnl1	0.45074	1.22906899	0.29368118
1418712_at	CDC42 effector protein (Rho GTPase binding) 5	Cdc42ep5	0.45076	1.17342462	0.22898409
1426628_at	transmembrane protein 184C	Tmem184c	0.45078	-1.17445749	-0.2337
1441852_x_at	autophagy-related 16-like 1 (yeast)	Atg16l1	0.45101	1.18086352	0.23954266
1435758_at	UDP-Gal:betaGlcNAc beta 1,4-galactosyltransferase, polypeptide	B4galt6	0.45119	-1.18490699	-0.2507196
1438510_a_at	histidyl-tRNA synthetase	Hars	0.45148	-1.14642342	-0.1978273
1436786_at	RIKEN cDNA 1110069O07 gene	1110069O07Rik	0.45192	-1.24099769	-0.3139165
1450054_at	adducin 1 (alpha)	Add1	0.45272	1.15141654	0.20307176
1456430_at	tetratricopeptide repeat domain 14	Ttc14	0.45273	1.20289977	0.25453252
1452741_s_at	glycerol phosphate dehydrogenase 2, mitochondrial	Gpd2	0.45284	-1.28579554	-0.3931132
1439427_at	claudin 9	Cldn9	0.45309	1.25754086	0.32507016
1451486_at	solute carrier family 46, member 3	Slc46a3	0.45343	1.29272372	0.35943416
1429009_at	small nuclear ribonucleoprotein 70 (U1)	Snrnp70	0.45374	-1.20762712	-0.2752978
1439065_x_at	predicted gene 13152	Gm13152	0.45457	-1.18144795	-0.2489251
1459601_at	Salt inducible kinase 1	Sik1	0.45465	-1.19473838	-0.2601291
1437525_a_at	polymerase (RNA) III (DNA directed) polypeptide A	Polr3a	0.45471	-1.18393629	-0.2509235
1454674_at	fasciculation and elongation protein zeta 1 (zygin I)	Fez1	0.45476	-1.21755793	-0.288075
1440305_at	---	---	0.45552	-1.23216623	-0.3033642
1458353_at	NACHT and WD repeat domain containing 1	Nwd1	0.45602	-1.17741736	-0.2387887
1415929_at	microtubule-associated protein 1 light chain 3 beta	Map1lc3b	0.45618	1.17828495	0.2324418
1445678_at	---	---	0.45631	1.18587166	0.24379727
1426594_at	FERM domain containing 4B	Frmd4b	0.45654	1.33530677	0.39659795
1428939_s_at	guanine nucleotide binding protein, alpha q polypeptide	Gnaq	0.45673	-1.24414881	-0.3284769
1450418_a_at	Yip1 domain family, member 4	Yipf4	0.45681	-1.26921691	-0.3670661
1429318_a_at	quaking	Qk	0.45859	-1.20872996	-0.2855883
1434278_at	X-linked myotubular myopathy gene 1	Mtm1	0.45882	-1.09589898	-0.1340653
1428599_at	kinase non-catalytic C-lobe domain (KIND) containing 1	Kndc1	0.45882	1.20506041	0.26830427
1416173_at	pescadillo homolog 1, containing BRCT domain (zebrafish)	Pes1	0.45893	1.22858724	0.2923955
1445299_at	---	---	0.45904	-1.22792571	-0.2989303
1438908_at	mitogen-activated protein kinase kinase kinase 12	Map3k12	0.45921	-1.39752676	-0.5573236
1450649_at	guanine nucleotide binding protein (G protein), gamma 10	Gng10	0.4593	-1.15752591	-0.2133657
1450394_at	golgi phosphoprotein 3	Golph3	0.45938	-1.18132201	-0.244901
1432192_at	RIKEN cDNA 4930483K19 gene	4930483K19Rik	0.45948	1.24853145	0.31515749
1458220_at	deleted in liver cancer 1	Dlc1	0.45952	1.23236623	0.29904426
1426137_at	predicted gene 13138 /// reduced expression 2	Gm13138 /// Rex2	0.45955	-1.23916436	-0.3219988
1439670_at	upstream binding transcription factor, RNA polymerase I-like 1	Ubtfl1	0.45968	-1.23845416	-0.3176663
1441879_x_at	makorin, ring finger protein, 1	Mkrn1	0.45969	-1.23965767	-0.3281808
1428236_at	acyl-Coenzyme A binding domain containing 5	Acbd5	0.4597	-1.17496521	-0.2329602
1425128_at	UDP-GlcNAc:betaGal beta-1,3-N-acetylglucosaminyltransferase 8	B3gnt8	0.45979	1.24178145	0.31115169
1418516_at	metal response element binding transcription factor 2	Mtf2	0.45987	-1.1389763	-0.1895764
1421821_at	low density lipoprotein receptor	Ldlr	0.45989	1.1792715	0.23575769
1428862_at	tetratricopeptide repeat domain 17	Ttc17	0.4599	1.21912606	0.28335459
1456341_a_at	Kruppel-like factor 9	Klf9	0.45997	-1.29507783	-0.4218855
1428384_at	DNA segment, Chr 4, Brigham & Women's Genetics 0951 express	D4Bwg0951e	0.4601	1.27413532	0.33753171
1428046_a_at	zinc finger protein X-linked	Zfx	0.46026	-1.2653092	-0.3539006
1424953_at	cDNA sequence BC021614	BC021614	0.46034	1.21149629	0.27513588
1426065_a_at	tribbles homolog 3 (Drosophila)	Trib3	0.46046	-1.25216618	-0.3478821
1443161_at	---	---	0.46071	-1.27804832	-0.3743839
1459861_s_at	lysine (K)-specific demethylase 2B	Kdm2b	0.46077	-1.16509177	-0.2240646
1428739_at	energy homeostasis associated	Enho	0.46098	1.19506309	0.25678459
1452841_at	phosphoglucomutase 2-like 1	Pgm2l1	0.46102	-1.27110069	-0.3636302
1451324_s_at	forty-two-three domain containing 1	Fyttd1	0.46137	-1.19812495	-0.267195
1418835_at	pleckstrin homology-like domain, family A, member 1	Phlda1	0.46143	1.31243032	0.36431157
1427885_at	polymerase (DNA-directed), delta 4	Pold4	0.4618	1.20817567	0.26820652
1437093_at	dynein, axonemal, intermediate chain 1	Dnaic1	0.46255	1.21400716	0.27947072
1421694_a_at	versican	Vcan	0.46262	1.29686002	0.35868702
1431536_at	RIKEN cDNA 4933432K03 gene	4933432K03Rik	0.46267	-1.21568779	-0.2831544
1438967_x_at	anti-Mullerian hormone type 2 receptor	Amhr2	0.46291	1.18629706	0.24398968
1430159_at	RIKEN cDNA 5830408C22 gene	5830408C22Rik	0.46454	1.23697339	0.30156087
1441068_at	RIKEN cDNA A130001G05 gene	A130001G05Rik	0.46464	-1.24433236	-0.3192307
1441971_at	---	---	0.46481	-1.20673842	-0.2778819
1458878_at	Yamaguchi sarcoma viral (v-yes) oncogene homolog 1	Yes1	0.46505	-1.2330127	-0.3039758
1430542_a_at	solute carrier family 25 (mitochondrial carrier, adenine nucleotide	Slc25a5	0.46506	1.10217683	0.14017492
1430233_a_at	Na+/H+ exchanger domain containing 1	Nhedc1	0.46523	-1.16309656	-0.2199166
1456865_x_at	RRS1 ribosome biogenesis regulator homolog (S. cerevisiae)	Rrs1	0.46532	-1.16400484	-0.2224579
1440609_at	mitogen-activated protein kinase kinase kinase kinase 4	Map4k4	0.46544	-1.21026031	-0.2780089
1438045_at	early endosome antigen 1	Eea1	0.46549	-1.21827754	-0.2895179
1433207_at	RIKEN cDNA 5033430J17 gene	5033430J17Rik	0.46561	-1.22971515	-0.2983499
1426062_a_at	caspase 7	Casp7	0.46565	1.25100428	0.31635607
1438224_at	zinc finger, SWIM domain containing 5	Zswim5	0.46568	1.25666164	0.31958139
1415736_at	prefoldin 5	Pfdn5	0.4657	-1.15524375	-0.210845
1423626_at	dystonin	Dst	0.46573	-1.19149572	-0.2591168
1451285_at	fusion, derived from t(12;16) malignant liposarcoma (human)	Fus	0.46573	-1.13155534	-0.1793933
1416187_s_at	proline-rich nuclear receptor coactivator 2	Pnrc2	0.46577	-1.21429252	-0.2973349
1419758_at	ATP-binding cassette, sub-family B (MDR/TAP), member 1A	Abcb1a	0.4658	-1.19277712	-0.2608884
1453179_at	alkaline ceramidase 3	Acer3	0.46582	-1.23385285	-0.3059528
1441828_at	StAR-related lipid transfer (START) domain containing 5	Stard5	0.46591	1.21478034	0.27800125
1451199_at	queuine tRNA-ribosyltransferase domain containing 1	Qtrtd1	0.46593	-1.20385714	-0.2793099
1435477_s_at	Fc receptor, IgG, low affinity IIb	Fcgr2b	0.46605	1.21017816	0.27100381
1438276_at	---	---	0.46609	-1.24761372	-0.3259613
1423674_at	ubiquitin specific peptidase 1	Usp1	0.46619	-1.16367803	-0.2191951

1433829_a_at	heterogeneous nuclear ribonucleoprotein A2/B1	Hnrnpa2b1	0.46644	-1.20094965	-0.2802247
AFFX-r2-Ec-bioB-3	---	---	0.4666	1.17911444	0.23585092
1457218_at	spermatid perinuclear RNA binding protein	Strbp	0.46686	-1.21320298	-0.2826595
1433540_x_at	protein phosphatase 1, catalytic subunit, beta isoform	Ppp1cb	0.46688	-1.22924473	-0.3172826
1446902_at	expressed sequence AU022538	AU022538	0.4669	1.19433276	0.25584132
1441588_at	KCNQ1 overlapping transcript 1	Kcnq1ot1	0.46784	-1.2302067	-0.2994563
1418629_a_at	KH domain containing, RNA binding, signal transduction associated	Khdrbs1	0.46852	-1.15711838	-0.2108909
1427310_at	bromodomain PHD finger transcription factor	Bptf	0.46893	-1.1639397	-0.2227604
1440934_at	RIKEN cDNA 6230409E13 gene	6230409E13Rik	0.46899	-1.22209583	-0.2897682
1442031_at	coiled-coil domain containing 109A	Ccdc109a	0.46935	1.29475603	0.35535772
1460116_s_at	sprouty protein with EVH-1 domain 1, related sequence	Spred1	0.46945	-1.16574841	-0.2215935
1429434_at	phosphatidylinositol 3-kinase, catalytic, alpha polypeptide	Pik3ca	0.46946	-1.2376402	-0.309438
1427473_at	glutathione S-transferase, mu 3	Gstm3	0.46954	1.21777961	0.28200356
1460125_at	coiled-coil domain containing 141	Ccdc141	0.46968	1.31875801	0.37571595
1426957_at	transformation related protein 53 binding protein 1	Trp53bp1	0.46973	1.17406789	0.22840771
1431612_at	RIKEN cDNA 1700007J10 gene	1700007J10Rik	0.46975	-1.23829796	-0.312092
1417928_at	PDZ and LIM domain 4	Pdlim4	0.46976	1.23799175	0.30370094
1425986_a_at	DCN1, defective in cullin neddylation 1, domain containing 1 (S. ce	Dcun1d1	0.46982	1.24090027	0.30802734
1419608_a_at	melanoma inhibitory activity 1	Mia1	0.46989	1.28206848	0.3386245
1451857_a_at	notum pectinacetylesterase homolog (Drosophila)	Notum	0.46997	1.17663716	0.23168813
1437479_x_at	T-box 3	Tbx3	0.47	-1.22570509	-0.3093326
1437869_at	protein phosphatase 2, regulatory subunit B'', alpha	Ppp2r3a	0.47004	-1.28414237	-0.3828424
1419704_at	cytochrome P450, family 3, subfamily a, polypeptide 41A /// cyto	Cyp3a41a /// Cyp3a41b	0.4708	1.17644594	0.23327191
1421160_a_at	RFNG O-fucosylpeptide 3-beta-N-acetylglucosaminyltransferase	Rfng	0.4717	1.18127513	0.23635604
1418310_a_at	retinaldehyde binding protein 1	Rlbp1	0.47195	1.20401559	0.26745921
1453091_s_at	LETM1 domain containing 1	Letmd1	0.47216	1.23893122	0.30274641
1449168_a_at	A kinase (PRKA) anchor protein 2 /// paralemmin 2	Akap2 /// Palm2	0.4722	-1.31392385	-0.4373235
1448213_at	annexin A1	Anxa1	0.47232	1.23597451	0.29661352
1418208_at	paired box gene 8	Pax8	0.47245	-1.2871393	-0.3858599
1419018_at	reproductive homeobox 6	Rhox6	0.47276	1.20264651	0.26437302
1423090_x_at	SEC61, gamma subunit	Sec61g	0.47278	1.15271834	0.20059694
1443340_at	calcium binding atopy-related autoantigen 1	Cbara1	0.47281	1.24191758	0.30881626
1450035_a_at	PRP40 pre-mRNA processing factor 40 homolog A (yeast)	Prpf40a	0.47283	-1.21285117	-0.2916073
1416656_at	chloride intracellular channel 1	Clic1	0.47284	1.16706861	0.22263262
1419918_at	Transmembrane emp24 protein transport domain containing 7	Tmed7	0.47299	-1.2664203	-0.361387
1458364_s_at	torsin A interacting protein 1	Tor1aip1	0.473	1.28562302	0.3218991
1443672_at	leucyl-tRNA synthetase, mitochondrial	Lars2	0.47318	1.19987422	0.26039486
1458235_at	---	---	0.4732	-1.21070279	-0.2762129
1434039_at	amyloid beta precursor protein (cytoplasmic tail) binding protein 2	Appbp2	0.47321	-1.17523417	-0.235309
1430591_at	dopa decarboxylase	Ddc	0.47322	-1.21589777	-0.2835534
1456019_at	CWF19-like 2, cell cycle control (S. pombe)	Cwf19l2	0.47325	-1.17030477	-0.2285228
1450928_at	inhibitor of DNA binding 4	Id4	0.47359	1.35568769	0.40899633
1427037_at	eukaryotic translation initiation factor 4, gamma 1	Eif4g1	0.47359	-1.15414967	-0.2070538
1438403_s_at	metastasis associated lung adenocarcinoma transcript 1 (non-cod	Malat1	0.47376	-1.31012335	-0.4253797
1415822_at	stearoyl-Coenzyme A desaturase 2	Scd2	0.47378	1.12463555	0.1693917
1419967_at	SEH1-like (S. cerevisiae	Seh1l	0.47418	-1.2587377	-0.3541315
1450029_s_at	integrin alpha 9	Itga9	0.47561	-1.22536396	-0.3004284
1427113_s_at	tubulin tyrosine ligase	Ttl	0.47574	1.19199266	0.25204443
1454725_at	transformer 2 alpha homolog (Drosophila)	Tra2a	0.47607	-1.21027551	-0.2906205
1439864_at	coiled-coil domain containing 40	Ccdc40	0.47643	1.21053159	0.2712803
1416666_at	serine (or cysteine) peptidase inhibitor, clade E, member 2	Serpine2	0.47649	-1.19008414	-0.2567071
1429107_at	ubiquitin protein ligase E3 component n-recognin 3	Ubr3	0.47654	1.2079678	0.2651787
1423557_at	interferon gamma receptor 2	Ifngr2	0.47673	1.24814083	0.31365646
1442288_at	annexin A6	Anxa6	0.47683	1.21509956	0.27871323
1418130_at	24-dehydrocholesterol reductase	Dhcr24	0.47702	-1.21249375	-0.2856096
1436620_at	coiled-coil domain containing 45	Ccdc45	0.47801	1.23253558	0.29801294
1424775_at	2'-5' oligoadenylate synthetase 1A	Oas1a	0.47813	-1.183149	-0.2466598
1424383_at	transmembrane protein 51	Tmem51	0.47926	1.17694692	0.23060882
1436116_x_at	adaptor protein, phosphotyrosine interaction, PH domain and leuc	Appl1	0.47927	-1.28217214	-0.3756911
1458334_at	wingless-related MMTV integration site 7A	Wnt7a	0.47935	1.34698571	0.40486526
1455845_at	WSC domain containing 1	Wscd1	0.47946	1.24307567	0.30666003
1424516_at	RIKEN cDNA B230354K17 gene	B230354K17Rik	0.47948	-1.15923859	-0.215368
1444972_at	expressed sequence C77626	C77626	0.47958	-1.20146384	-0.2660276
1426930_at	CUGBP, Elav-like family member 4	Celf4	0.47969	-1.24725108	-0.330521
1432850_at	RIKEN cDNA 5430434G16 gene	5430434G16Rik	0.47982	1.23799514	0.30301221
1456929_at	cDNA sequence BC042782	BC042782	0.47997	-1.22000495	-0.2900919
1422440_at	cyclin-dependent kinase 4	Cdk4	0.48004	1.13750529	0.1851417
1435181_at	lin-54 homolog (C. elegans)	Lin54	0.48005	-1.16567647	-0.2223782
1452336_at	zinc finger protein 395	Zfp395	0.48022	1.1875166	0.24641685
1441927_at	synaptotagmin VII	Syt7	0.48041	-1.22830744	-0.3002367
1447540_at	tigger transposable element derived 3	Tigd3	0.48077	1.20060087	0.26311819
1449608_a_at	---	---	0.48098	-1.24685958	-0.3270695
1415997_at	thioredoxin interacting protein	Txnip	0.48098	1.21492859	0.26923551
1425528_at	paired related homeobox 1	Prrx1	0.48102	1.21742196	0.28000776
1448682_at	dynein light chain LC8-type 1	Dynll1	0.48108	1.11685903	0.15837661
1441674_at	---	---	0.48111	-1.21388809	-0.2806756
1419577_at	FIG4 homolog (S. cerevisiae)	Fig4	0.48113	-1.15335413	-0.206073
1429870_at	TRAF2 and NCK interacting kinase	Tnik	0.48119	1.25285265	0.3188504
1434831_a_at	forkhead box O3	Foxo3	0.48119	-1.1668902	-0.2243263
1425048_a_at	high mobility group box 1	Hmgb1	0.48135	-1.10584524	-0.146085
1451920_a_at	replication factor C (activator 1) 1	Rfc1	0.48168	-1.29265366	-0.4071825
1453427_at	casein kinase 2, alpha 1 polypeptide	Csnk2a1	0.4817	-1.2256427	-0.2936749
1428016_a_at	Ras interacting protein 1	Rasip1	0.4818	1.22693517	0.28594862
1426407_at	CUGBP, Elav-like family member 1	Celf1	0.48377	-1.16855414	-0.2319642
1446758_at	brain and reproductive organ-expressed protein	Bre	0.48381	-1.21345503	-0.2794223
1425323_a_at	family with sequence similarity 173, member A	Fam173a	0.48403	1.18605441	0.24082921
1424433_at	methionine sulfoxide reductase B2	Msrb2	0.48424	1.19327729	0.25001844
1438207_at	golgi-specific brefeldin A-resistance factor 1	Gbf1	0.48493	-1.32523761	-0.4467831
1436400_at	fragile X mental retardation gene 1, autosomal homolog	Fxr1	0.48668	-1.18745528	-0.252077
1429865_at	RIKEN cDNA 4931417G12 gene	4931417G12Rik	0.48672	1.25397171	0.31713038
1420594_at	BRCA1 associated RING domain 1	Bard1	0.48683	1.20038626	0.26235841
1456577_x_at	pitrilysin metallepetidase 1	Pitrm1	0.48743	-1.21771348	-0.2947748
1435457_at	PDZ domain containing 4	Pdzd4	0.48752	1.24763862	0.3014884
1435127_a_at	O-sialoglycoprotein endopeptidase-like 1	Osgepl1	0.48764	1.19202359	0.24919582

1426128_a_at	potassium voltage-gated channel, subfamily Q, member 2	Kcnq2	0.48851	1.18679814	0.24693805
1451382_at	ChaC, cation transport regulator-like 1 (E. coli)	Chac1	0.4892	-1.20360942	-0.2818037
1424077_at	glycerophosphodiester phosphodiesterase domain containing 1	Gdpd1	0.48946	1.24594006	0.30997707
1438663_at	HLA-B associated transcript 2-like 2	Bat2l2	0.48983	-1.18466776	-0.2536821
1416290_a_at	proteasome (prosome, macropain) 26S subunit, ATPase, 4	Psmc4	0.48987	-1.23427408	-0.3329939
1416236_a_at	myelin protein zero-like 2	Mpzl2	0.48999	-1.16465344	-0.2203416
1455235_x_at	lactate dehydrogenase B	Ldhb	0.49004	1.17014907	0.22169812
1455938_x_at	RAD21 homolog (S. pombe)	Rad21	0.49012	-1.26357094	-0.3709015
1438715_at	coiled-coil domain containing 102A	Ccdc102a	0.49012	1.23259221	0.2955123
1428914_at	SH3 and PX domains 2A	Sh3pxd2a	0.49018	1.20892457	0.26899192
1426373_at	ski sarcoma viral oncogene homolog (avian)	Ski	0.49024	1.14647664	0.19652288
1439515_at	SET domain containing 5	Setd5	0.49043	-1.18701313	-0.2475212
1429417_at	chondroitin sulfate synthase 3	Chsy3	0.49058	1.16823901	0.22276579
1417424_at	immediate early response 3 interacting protein 1	Ier3ip1	0.49084	-1.16281246	-0.2196916
1435080_x_at	serine/arginine-rich splicing factor 18	Sfrs18	0.49104	-1.15710825	-0.2106462
1447825_x_at	protocadherin 8	Pcdh8	0.49113	1.19966447	0.25194689
1454448_at	RIKEN cDNA 2900018E21 gene	2900018E21Rik	0.4912	-1.18345698	-0.2444196
1432087_at	RIKEN cDNA 4933406G16 gene	4933406G16Rik	0.49292	-1.24249692	-0.3204419
1418729_at	steroidogenic acute regulatory protein	Star	0.49294	-1.20392375	-0.2704185
1453421_at	serine racemase	Srr	0.49301	-1.21676433	-0.2842825
1439696_at	nuclear receptor subfamily 2, group C, member 2	Nr2c2	0.49333	1.2010042	0.26204846
1419091_a_at	annexin A2	Anxa2	0.49338	1.15645976	0.20876146
1442719_at	protein arginine N-methyltransferase 3	Prmt3	0.49421	-1.21328891	-0.2793088
1435545_at	cDNA sequence BC032203	BC032203	0.49519	-1.27628495	-0.3917291
1415977_at	myo-inositol 1-phosphate synthase A1	Isyna1	0.49539	1.15133448	0.20231717
1434028_at	aryl hydrocarbon receptor nuclear translocator 2	Arnt2	0.49558	1.21575469	0.27770199
1426558_x_at	RIKEN cDNA 0610010B08 gene /// predicted gene 14295 /// simi	0610010B08Rik /// Gm14	0.49576	-1.19178421	-0.2598503
1453282_at	coxsackie virus and adenovirus receptor	Cxadr	0.49584	-1.22374764	-0.2998088
1447408_at	---	---	0.49586	-1.43622937	-0.6292274
1441419_at	---	---	0.49734	-1.22470181	-0.3000742
1428870_at	nucleolar and coiled-body phosphoprotein 1	Nolc1	0.49737	1.15818194	0.21171749
1419322_at	FYVE, RhoGEF and PH domain containing 6	Fgd6	0.49747	1.23067434	0.29241541
1433789_at	small nucleolar RNA host gene (non-protein coding) 3	Snhg3	0.49756	1.32016942	0.36474391
1441492_at	---	---	0.49772	1.22493001	0.29252921
1438371_x_at	DEAD (Asp-Glu-Ala-Asp) box polypeptide 5	Ddx5	0.49788	-1.19641297	-0.2764293
1434336_s_at	REST corepressor 1	Rcor1	0.49845	-1.19299218	-0.2638137
1450743_s_at	synaptotagmin binding, cytoplasmic RNA interacting protein	Syncrip	0.49944	-1.14832162	-0.2003566
1448804_at	cytochrome P450, family 11, subfamily a, polypeptide 1	Cyp11a1	0.49957	1.2025471	0.26605011
1446800_at	RIKEN cDNA 3010026O09 gene	3010026O09Rik	0.4997	-1.24493327	-0.321264
1455902_x_at	Ras homolog gene family, member f	Rhof	0.49995	-1.22287162	-0.291481
1426259_at	pantothenate kinase 3	Pank3	0.5001	-1.25252466	-0.3377275
1445445_s_at	prostaglandin E receptor 1 (subtype EP1)	Ptger1	0.50014	1.17196689	0.22839223
1452249_at	prickle homolog 1 (Drosophila)	Prickle1	0.50051	1.24048409	0.30542724
1432950_at	RIKEN cDNA 5330421F21 gene	5330421F21Rik	0.50057	1.19472801	0.25547429
1426655_a_at	family with sequence similarity 63, member A	Fam63a	0.50067	-1.18388687	-0.248848
1456609_at	calcium/calmodulin-dependent protein kinase II inhibitor 1	Camk2n1	0.50078	1.19421111	0.25587311
1418431_at	kinesin family member 5B	Kif5b	0.50079	-1.20537259	-0.2758644
1432870_at	RIKEN cDNA 4932429P19 gene	4932429P19Rik	0.50086	1.24701962	0.31059129
1429006_s_at	alpha tubulin acetyltransferase 1	Atat1	0.50109	1.25437475	0.31821404
1433342_at	RIKEN cDNA 5730416F02 gene	5730416F02Rik	0.50154	1.20986117	0.27420156
1451255_at	lipolysis stimulated lipoprotein receptor	Lsr	0.50168	1.15512475	0.20762886
1455648_at	predicted gene 7072	Gm7072	0.50176	-1.23417279	-0.3130265
1416708_a_at	GRAM domain containing 1A	Gramd1a	0.50201	1.17396527	0.23062227
1422597_at	matrix metallopeptidase 15	Mmp15	0.50264	1.22872741	0.29004096
1422584_at	superkiller viralicidic activity 2-like (S. cerevisiae)	Skiv2l	0.50281	1.21301487	0.27171956
1421054_at	exportin 4	Xpo4	0.50299	-1.1939398	-0.2644617
1460729_at	Rho-associated coiled-coil containing protein kinase 1	Rock1	0.50302	-1.28346014	-0.3948352
1435660_at	similar to Sp110 nuclear body protein	LOC664787	0.50305	-1.22516178	-0.2954958
1433673_at	RIKEN cDNA E130309D14 gene	E130309D14Rik	0.50327	1.22818816	0.29077457
1426593_a_at	F-box protein 22	Fbxo22	0.50352	-1.17315051	-0.2384029
1419416_a_at	retinoic acid receptor, gamma	Rarg	0.50379	-1.21884351	-0.3055445
1436944_x_at	phosphatidylserine decarboxylase, pseudogene 1 /// phosphatidy	Pisd-ps1 /// Pisd-ps3	0.50391	-1.19275013	-0.2610623
1424107_at	kinesin family member 18A	Kif18a	0.50407	-1.15374031	-0.2081133
1416967_at	SRY-box containing gene 2	Sox2	0.50421	-1.13300214	-0.182659
1427284_a_at	tocopherol (alpha) transfer protein	Ttpa	0.50422	-1.19374698	-0.2584911
1435275_at	cytochrome c oxidase subunit VIb polypeptide 2	Cox6b2	0.50423	1.16114014	0.21322067
1446304_at	---	---	0.50435	-1.26570807	-0.3623807
1438939_x_at	necdin	Ndn	0.50441	-1.21156546	-0.2809951
1453240_a_at	granule cell antiserum positive 14	Gcap14	0.50443	-1.18999204	-0.2529397
1434657_at	glutaminase	Gls	0.50445	1.16871988	0.22474317
1448130_at	farnesyl diphosphate farnesyl transferase 1	Fdft1	0.50452	1.14969607	0.20011081
1439775_at	bromodomain and WD repeat domain containing 3 /// predicted	Brwd3 /// Gm10452	0.50453	-1.2228498	-0.2981704
1428944_at	ubiquitin-like modifier activating enzyme 6	Uba6	0.50455	-1.18739971	-0.2566181
1416476_a_at	ubiquitin-conjugating enzyme E2D 2	Ube2d2	0.50456	-1.20123193	-0.2817618
1443800_at	---	---	0.50457	-1.20585346	-0.2726218
1460141_at	Ferric-chelate reductase 1	Frrs1	0.50461	-1.25917319	-0.3549738
1417663_a_at	N-myc downstream regulated gene 3	Ndrg3	0.50469	1.18799308	0.24608988
1428552_at	RIKEN cDNA 2610001J05 gene	2610001J05Rik	0.50472	1.151658	0.20282596
1425096_a_at	pentatricopeptide repeat domain 1	Ptcd1	0.50472	1.18552329	0.24388486
1438993_a_at	ATPase, H+ transporting, lysosomal V1 subunit D	Atp6v1d	0.50484	-1.15716098	-0.2125235
1433499_at	upregulator of cell proliferation	Urgcp	0.50488	1.21736647	0.27910252
1443675_at	---	---	0.50503	-1.27003534	-0.3628463
1428853_at	patched homolog 1	Ptch1	0.50525	1.14339319	0.19252438
1452753_at	forkhead box K2	Foxk2	0.50667	1.16779352	0.22199865
1426705_s_at	isoleucine-tRNA synthetase	Iars	0.50683	-1.14462271	-0.1960775
1451978_at	lysyl oxidase-like 1	Loxl1	0.5069	1.21596073	0.2784783
1416382_at	cathepsin C	Ctsc	0.50703	-1.18259337	-0.2420821
1425652_s_at	RNA binding protein gene with multiple splicing	Rbpms	0.50708	-1.22832064	-0.3209231
1429383_at	casein kinase 1, gamma 3	Csnk1g3	0.50726	-1.15959946	-0.2148811
1448529_at	thrombomodulin	Thbd	0.50771	1.21753553	0.28033805
1425139_at	sestrin 2	Sesn2	0.50772	-1.18209513	-0.2466941
1416072_at	CD34 antigen	Cd34	0.50883	1.23885302	0.30145733
1423748_at	pyruvate dehydrogenase kinase, isoenzyme 1	Pdk1	0.50965	1.15221519	0.20166632
1454867_at	meningioma 1	Mn1	0.50966	1.30446114	0.36207423

1438922_x_at	solute carrier family 25 (mitochondrial carrier, adenine nucleotide	Slc25a5	0.50971	-1.19450267	-0.2709934
1438310_at	---	---	0.50972	-1.19929855	-0.2710125
1442060_at	prolyl endopeptidase-like	Prepl	0.50987	-1.24491773	-0.3273486
1421810_at	DiGeorge syndrome critical region gene 2	Dgcr2	0.50995	1.16066183	0.2137552
1421076_at	SERTA domain containing 3	Sertad3	0.50996	1.17688097	0.23368296
1437297_at	chromodomain helicase DNA binding protein 8	Chd8	0.50999	-1.18025434	-0.2454797
1417509_at	ring finger protein 19A	Rnf19a	0.51001	1.26585518	0.32168251
1439088_at	PDZ domain containing 8	Pdzd8	0.5101	-1.26201893	-0.351659
1424220_a_at	porcupine homolog (Drosophila)	Porcn	0.51011	-1.19778126	-0.2713483
1437916_at	---	---	0.51011	-1.19623385	-0.2589407
1424219_at	mitochondrial rRNA methyltransferase 1 homolog (S. cerevisiae)	Mrm1	0.51011	1.21466267	0.27441029
1437526_x_at	heterogeneous nuclear ribonucleoprotein R	Hnrnpr	0.51012	-1.19374568	-0.2702119
1424511_at	aurora kinase A	Aurka	0.51023	1.13595536	0.18316439
1417303_at	mevalonate (diphospho) decarboxylase	Mvd	0.51024	1.15696334	0.20904435
1426406_at	SET domain containing (lysine methyltransferase) 8	Setd8	0.51049	-1.20933546	-0.2919924
1460078_at	zinc finger protein 771	Zfp771	0.5106	-1.24249958	-0.3208192
1444843_at	---	---	0.51081	1.22986483	0.28899177
1424066_at	dihydrouridine synthase 3-like (S. cerevisiae)	Dus3l	0.51082	1.14590096	0.19631232
1437200_at	FCH domain only 2	Fcho2	0.51089	-1.18298348	-0.2446424
1444952_a_at	nuclear casein kinase and cyclin-dependent kinase substrate 1	Nucks1	0.51094	-1.25320629	-0.3612486
1456925_at	purinergic receptor P2X, ligand-gated ion channel, 6	P2rx6	0.51101	-1.21749372	-0.2846222
1453174_at	neurobeachin like 1	Nbeal1	0.51102	-1.17814921	-0.2420187
1454064_a_at	ring finger protein 138	Rnf138	0.51113	-1.14622791	-0.1987523
1433887_at	DnaJ (Hsp40) homolog, subfamily C, member 3	Dnajc3	0.51116	-1.20588087	-0.2721382
1419277_at	ubiquitin specific peptidase 48	Usp48	0.5113	-1.1765075	-0.2387238
1437981_x_at	RIKEN cDNA 1110057K04 gene	1110057K04Rik	0.51135	-1.21943458	-0.2867965
1441252_at	RIKEN cDNA 2010001A14 gene	2010001A14Rik	0.51159	-1.21102908	-0.2775758
1427872_at	platelet-activating factor receptor	Ptafr	0.51174	-1.27232057	-0.3735675
1458178_at	hypothetical LOC100504030	LOC100504030	0.51205	-1.22756114	-0.3017013
1456610_at	KDM1 lysine (K)-specific demethylase 6B	Kdm6b	0.51207	-1.15542607	-0.210129
1415918_a_at	triosephosphate isomerase 1	Tpi1	0.51211	1.11186181	0.15238726
1457858_at	THO complex 4 pseudogene	Gm4850	0.51216	-1.21606831	-0.2833723
1423785_at	EGL nine homolog 1 (C. elegans)	Egln1	0.51226	1.17207473	0.22658323
1448259_at	follistatin-like 1	Fstl1	0.51237	-1.16462357	-0.2218442
1431263_at	protein kinase, AMP-activated, gamma 2 non-catalytic subunit	Prkag2	0.51243	1.1892808	0.2495161
1452918_at	DNA segment, Chr 19, ERATO Doi 737, expressed	D19Ertd737e	0.51255	-1.1643025	-0.2254699
1417501_at	F-box protein 6	Fbxo6	0.51259	1.16109826	0.2112877
1456434_x_at	heat shock protein 8	Hspb8	0.51264	1.22662568	0.28757734
1455083_at	ATPase, class VI, type 11C	Atp11c	0.51269	-1.17957341	-0.2383675
1435679_at	optineurin	Optn	0.51272	-1.25430911	-0.3471767
1434572_at	histone deacetylase 9	Hdac9	0.51275	-1.18613888	-0.2485836
1449426_a_at	annexin A10	Anxa10	0.51285	1.30540239	0.3614963
1439456_x_at	ATPase, H+ transporting, lysosomal accessory protein 2	Atp6ap2	0.51289	-1.14429172	-0.1951752
1422845_at	calnexin	Canx	0.51291	-1.15528834	-0.2133345
1436827_at	family with sequence similarity 59, member A	Fam59a	0.51301	1.17561724	0.23253599
1459976_s_at	superoxide dismutase 1, soluble	Sod1	0.51302	-1.12310662	-0.1686314
1449839_at	caspase 3	Casp3	0.51305	1.14244825	0.19208872
1448317_at	transmembrane protein 128	Tmem128	0.51305	1.17353302	0.22717666
1456405_at	death inducer-obliterator 1	Dido1	0.51309	1.1416961	0.19034118
1451458_at	transmembrane protein 2	Tmem2	0.5131	1.20602288	0.25912737
1428795_at	RIKEN cDNA 1110021L09 gene	1110021L09Rik	0.51313	1.17644147	0.23334555
1449575_a_at	glutathione S-transferase, pi 1	Gstp1	0.51323	1.13358618	0.17852957
1421676_at	RIKEN cDNA 4930542N07 gene	4930542N07Rik	0.51323	1.18878395	0.24511642
1459130_at	HECT domain and ankyrin repeat containing, E3 ubiquitin protein	Hace1	0.5133	-1.20759697	-0.2743392
1456628_x_at	ribosomal protein S24	Rps24	0.5133	-1.1265986	-0.177787
1430798_x_at	mitochondrial ribosomal protein L15	Mrpl15	0.51345	-1.18257632	-0.2483722
1426777_a_at	Wiskott-Aldrich syndrome-like (human)	Wasl	0.51346	-1.26437928	-0.372179
1459098_at	---	---	0.5135	1.22732513	0.29311376
1442398_at	---	---	0.51352	-1.22083942	-0.2902857
1453154_at	RIKEN cDNA 1700029M20 gene	1700029M20Rik	0.51353	1.23980111	0.30270099
1415777_at	pancreatic lipase related protein 1	Pnliprp1	0.51355	-1.24212563	-0.3239807
1443345_at	---	---	0.51366	1.20813224	0.26564368
1459118_at	hypothetical protein D230038C21	D230038C21	0.51373	1.22193208	0.27085813
1449345_at	coiled-coil domain containing 34	Ccdc34	0.51386	-1.18893465	-0.2507563
1428289_at	Kruppel-like factor 9	Klf9	0.51387	-1.16177382	-0.2218186
1422220_at	POU domain, class 1, transcription factor 1	Pou1f1	0.5139	-1.21663847	-0.2838734
1426711_at	transmembrane and coiled-coil domains 3	Tmco3	0.51391	1.22082013	0.28096271
1443654_at	ankyrin repeat and FYVE domain containing 1	Ankfy1	0.51402	-1.19688013	-0.2623592
1436287_at	predicted gene 10664	Gm10664	0.5141	1.17607887	0.22693994
1434127_a_at	H3 histone, family 3A /// hypothetical LOC100503721	H3f3a /// LOC100503721	0.51417	-1.11598802	-0.1618923
1459897_a_at	suprabasin	Sbsn	0.51417	-1.21102781	-0.2767311
1433960_at	interferon stimulated exonuclease gene 20-like 2	Isg20l2	0.51419	-1.15976236	-0.2189247
1436838_x_at	coactosin-like 1 (Dictyostelium)	Cotl1	0.51441	1.16123259	0.21335913
1442223_at	Enabled homolog (Drosophila)	Enah	0.51459	-1.19120841	-0.2567333
1427920_at	PHD finger protein 19	Phf19	0.51492	1.18443348	0.24380415
1455356_at	calmodulin regulated spectrin-associated protein 1	Camsap1	0.51511	-1.1975756	-0.2735448
1418332_a_at	ATP/GTP binding protein 1	Agtpbp1	0.51532	-1.17402819	-0.2354673
1416728_at	casein kinase 2, beta polypeptide	Csnk2b	0.51534	1.13551448	0.18328904
1442812_at	predicted gene 10482	Gm10482	0.51556	1.25738428	0.31932194
1437154_at	centrosomal protein 170	Cep170	0.5156	-1.20944538	-0.2816353
1456982_at	RIKEN cDNA 6430562O15 gene	6430562O15Rik	0.51562	1.23817096	0.30373633
1439561_at	RIKEN cDNA 2010012O05 gene	2010012O05Rik	0.51567	-1.23824764	-0.3177435
1457252_x_at	phospholipase D2	Pld2	0.5157	1.18894618	0.24711309
1455901_at	choline phosphotransferase 1	Chpt1	0.51588	-1.24214593	-0.3258707
1442103_at	Nipped-B homolog (Drosophila)	Nipbl	0.516	1.21509998	0.27846699
1443238_at	---	---	0.51608	1.2106575	0.27151276
1423895_a_at	CUGBP, Elav-like family member 2	Celf2	0.51719	-1.19565163	-0.2626715
1444062_at	RIKEN cDNA 2900056L01 gene	2900056L01Rik	0.51895	-1.19123765	-0.2538941
1429491_s_at	Rap1 interacting factor 1 homolog (yeast)	Rif1	0.51911	-1.14242656	-0.1932374
1426910_at	PRKC, apoptosis, WT1, regulator	Pawr	0.51925	-1.16447448	-0.2253401
1449021_at	ribonuclease P 21 subunit (human)	Rpp21	0.51935	1.1818759	0.23638466
1438956_x_at	proviral integration site 3	Pim3	0.51938	-1.27193432	-0.3719506
1417946_at	abhydrolase domain containing 3	Abhd3	0.51941	1.20508037	0.26900992
1437288_at	inositol monophosphatase domain containing 1	Impad1	0.51944	-1.23568889	-0.3309809

1419609_at	chemokine (C-C motif) receptor 1	Ccr1	0.51947	-1.21026791	-0.2791154
1434331_at	eukaryotic translation initiation factor 2C, 1	Eif2c1	0.51963	1.17347668	0.2280923
1446087_at	---	---	0.51966	-1.23567446	-0.3160444
1420553_x_at	serine (or cysteine) peptidase inhibitor, clade A, member 1A	Serpina1a	0.51976	1.16693353	0.21695094
1426442_at	glycoprotein m6a	Gpm6a	0.51998	1.29637323	0.35471418
1433519_at	nuclear casein kinase and cyclin-dependent kinase substrate 1	Nucks1	0.52	-1.12746372	-0.1769472
1445082_at	---	---	0.52001	-1.19107506	-0.2533
1455267_at	estrogen-related receptor gamma	Esrrg	0.52008	1.19959046	0.26250585
1421574_at	RAS related protein 2a	Rap2a	0.5202	-1.27648315	-0.373527
1423633_at	small nuclear ribonucleoprotein 48 (U11/U12)	Snrnp48	0.52029	-1.13554356	-0.1834732
1448615_at	copper chaperone for superoxide dismutase	Ccs	0.52039	1.16854569	0.21985778
1418564_s_at	serpine1 mRNA binding protein 1	Serbp1	0.52042	-1.14465843	-0.2042111
1425522_at	RNA binding motif protein 25	Rbm25	0.52047	-1.23379949	-0.3130524
1438250_s_at	predicted gene 12372 /// TAF9 RNA polymerase II, TATA box bin	Gm12372 /// Taf9	0.52051	-1.15656676	-0.2156655
1417009_at	complement component 1, r subcomponent A	C1ra	0.52056	1.19166411	0.25198759
1448466_at	cell division cycle associated 5	Cdca5	0.52057	1.14183974	0.19046138
1417550_a_at	germ cell-specific gene 1	Gsg1	0.5206	-1.19157211	-0.2540499
1442379_at	family with sequence similarity 196, member B	Fam196b	0.52061	-1.24132582	-0.3215021
1428381_a_at	pancreatic progenitor cell differentiation and proliferation factor h	Ppdpf	0.52062	1.15539581	0.20474924
1420917_at	PRP40 pre-mRNA processing factor 40 homolog A (yeast)	Prpf40a	0.52064	-1.20647608	-0.2836919
1449381_a_at	protein kinase C and casein kinase substrate in neurons 1	Pacsin1	0.52067	1.17388729	0.23102701
1448656_at	calcium channel, voltage-dependent, beta 3 subunit	Cacnb3	0.52071	1.20826351	0.26330738
1423719_at	cDNA sequence U46068	U46068	0.52073	1.22319352	0.28256825
1433534_a_at	chaperonin containing Tcp1, subunit 2 (beta)	Cct2	0.52079	-1.15920717	-0.2230175
1433515_s_at	ethanolamine kinase 1	Etnk1	0.5208	-1.22079602	-0.3049413
1434280_at	---	---	0.52082	-1.12732777	-0.177516
1450982_at	solute carrier family 9 (sodium/hydrogen exchanger), member 3 r	Slc9a3r1	0.52091	1.15621367	0.20743
1452187_at	RNA binding motif protein 5	Rbm5	0.52094	1.18221254	0.24044078
1450770_at	RIKEN cDNA 3632451O06 gene	3632451O06Rik	0.52105	1.18957983	0.24993509
1428526_at	LSM domain containing 1	Lsmd1	0.52109	1.18240272	0.23228021
1428573_at	chimerin (chimaerin) 2	Chn2	0.52115	-1.21623374	-0.2840694
1435633_at	PWWP domain containing 2A	Pwwp2a	0.52128	-1.15592087	-0.2186365
1433259_at	RIKEN cDNA 4930518C04 gene	4930518C04Rik	0.52129	1.18066633	0.23890832
1429658_a_at	structural maintenance of chromosomes 2	Smc2	0.52131	-1.3171957	-0.4529247
1416633_a_at	family with sequence similarity 96, member A	Fam96a	0.52134	-1.18241738	-0.2541142
1455686_at	ligand dependent nuclear receptor corepressor-like	Lcorl	0.52158	-1.25944345	-0.3548872
1456462_x_at	protein phosphatase 1, catalytic subunit, beta isoform	Ppp1cb	0.52158	-1.18953092	-0.257331
1448559_at	flotillin 1	Flot1	0.52172	1.16803318	0.22207742
1418514_at	metal response element binding transcription factor 2	Mtf2	0.52187	-1.19640521	-0.2757863
1431461_at	RIKEN cDNA 4933417E11 gene	4933417E11Rik	0.52213	1.19196371	0.25172035
1431971_at	predicted gene 14318	Gm14318	0.52227	-1.17681408	-0.2361785
1429741_at	potassium channel, subfamily V, member 1	Kcnv1	0.5224	1.16056036	0.21405344
1447791_s_at	guanine nucleotide binding protein, alpha 14	Gna14	0.52249	1.24862589	0.31296487
1459741_x_at	uncoupling protein 2 (mitochondrial, proton carrier)	Ucp2	0.52294	-1.15828474	-0.2165271
1451485_at	LUC7-like 3 (S. cerevisiae)	Luc7l3	0.52303	-1.215833	-0.301318
1437152_at	mex3 homolog B (C. elegans)	Mex3b	0.52339	1.25226934	0.31362919
1425806_a_at	mediator complex subunit 21	Med21	0.52381	1.16813636	0.22106542
1456803_at	Polymerase (RNA) III (DNA directed) polypeptide C	Polr3c	0.52386	1.20738422	0.27074546
1447444_at	UBX domain protein 11	Ubxn11	0.52432	-1.25032887	-0.3349955
1445422_at	hypothetical protein LOC621549	LOC621549	0.5247	-1.20289975	-0.2758603
1435494_s_at	desmoplakin	Dsp	0.52478	-1.18760937	-0.2542634
1432543_a_at	Kruppel-like factor 13	Klf13	0.52481	1.24559378	0.29635333
1428131_a_at	CDC42 small effector 1	Cdc42se1	0.52491	1.13136887	0.17806936
1437719_x_at	RIKEN cDNA A230046K03 gene	A230046K03Rik	0.52499	-1.20685615	-0.2788473
1416289_at	procollagen-lysine, 2-oxoglutarate 5-dioxygenase 1	Plod1	0.52509	1.15787636	0.20992471
1457193_at	myeloid/lymphoid or mixed-lineage leukemia 3	Mll3	0.52525	-1.20599287	-0.2747528
1444066_at	GTPase activating protein and VPS9 domains 1	Gapvd1	0.52547	-1.21523069	-0.2832287
1423982_at	serine/arginine-rich splicing factor 10	Srsf10	0.52551	-1.14288819	-0.1941506
1418373_at	phosphoglycerate mutase 2	Pgam2	0.52569	1.18994928	0.24944307
1437559_at	regulator of G-protein signalling 7 binding protein	Rgs7bp	0.52572	1.17946177	0.23571421
1427151_at	glutamine and serine rich 1	Qser1	0.52574	-1.26762475	-0.3758802
1459740_s_at	uncoupling protein 2 (mitochondrial, proton carrier)	Ucp2	0.52581	-1.16931393	-0.2303093
1435285_at	metallophosphoesterase domain containing 2	Mpped2	0.52584	1.22309147	0.2844636
1427983_at	zinc finger protein 280C	Zfp280c	0.52586	1.19707253	0.25193566
1430569_at	tetratricopeptide repeat domain 9C	Ttc9c	0.52599	-1.20209649	-0.2711953
1428897_at	RIKEN cDNA 2610029I01 gene	2610029I01Rik	0.5261	1.16308947	0.21608627
1455087_at	DNA segment, Chr 7, ERATO Doi 715, expressed	D7Ertd715e	0.52615	-1.15606284	-0.2102246
1429098_s_at	nonhomologous end-joining factor 1	Nhej1	0.52625	1.20171257	0.26060928
1438081_at	mutated in colorectal cancers	Mcc	0.52633	1.17950222	0.23786236
1433281_at	prune homolog (Drosophila)	Prune	0.5264	-1.22525735	-0.2967461
1427990_at	ubiquitin specific petidase 45	Usp45	0.52654	-1.2218215	-0.3080039
1436678_at	sarcoglycan, beta (dystrophin-associated glycoprotein)	Sgcb	0.5267	1.19649948	0.25878242
1431917_at	phosphatidylinositol glycan anchor biosynthesis, class Q	Pigq	0.52678	-1.20071998	-0.2647688
1429298_at	dimethylarginine dimethylaminohydrolase 1	Ddah1	0.52692	1.15629879	0.20745963
1448027_at	nuclear receptor coactivator 3	Ncoa3	0.52695	-1.22232066	-0.3109012
1419556_at	E74-like factor 5	Elf5	0.52696	-1.26144262	-0.3547178
1437627_at	mex3 homolog D (C. elegans)	Mex3d	0.52707	1.17009734	0.22110253
1452660_s_at	kelch-like 7 (Drosophila)	Klhl7	0.52715	-1.18611623	-0.2505576
1429571_a_at	sperm acrosome associated 1	Spaca1	0.52727	1.24830987	0.30526059
1449146_at	Notch gene homolog 4 (Drosophila)	Notch4	0.5276	1.15894097	0.21215951
1437667_a_at	BTB and CNC homology 2	Bach2	0.52771	1.33453842	0.38771407
1451181_at	transmembrane protein 121	Tmem121	0.52792	1.20699717	0.26377264
1422867_at	granzyme G	Gzmg	0.52798	-1.20735142	-0.280466
1426473_at	DnaJ (Hsp40) homolog, subfamily C, member 9	Dnajc9	0.52798	1.14847703	0.19750327
1437354_at	RIKEN cDNA C230091D08 gene	C230091D08Rik	0.52816	-1.18724449	-0.2516174
1421870_at	tripartite motif-containing 44	Trim44	0.52828	-1.14562757	-0.1986387
1458456_x_at	RIKEN cDNA 6430571L13 gene	6430571L13Rik	0.52839	-1.20100423	-0.2642963
1422871_at	potassium inwardly-rectifying channel, subfamily J, member 12	Kcnj12	0.52859	-1.22069152	-0.2926222
1451287_s_at	allograft inflammatory factor 1-like	Aif1l	0.52861	1.2062134	0.2687568
1460015_at	spermatogenesis and oogenesis specific basic helix-loop-helix 1	Sohlh1	0.52882	-1.23833817	-0.3195144
1441178_at	DTW domain containing 2	Dtwd2	0.52896	-1.21228091	-0.2907127
1460308_a_at	immature colon carcinoma transcript 1	Ict1	0.52906	1.14362733	0.19253452
1425642_at	centrosomal protein 290	Cep290	0.52919	-1.2045651	-0.2756014
1459890_s_at	RIKEN cDNA 1110008P14 gene	1110008P14Rik	0.52922	1.1915254	0.24371174

1446031_at	---	---	0.52924	-1.22639029	-0.3062105
1424221_at	sushi domain containing 4	Susd4	0.52929	-1.20720516	-0.2790552
1433043_at	versican	Vcan	0.52935	1.18409655	0.24321656
1456102_a_at	cullin 5	Cul5	0.52949	-1.20365876	-0.272106
1434343_at	RIKEN cDNA 5730403M16 gene	5730403M16Rik	0.52958	1.18881757	0.24728768
1428301_at	predicted gene, ENSMUSG00000068790 /// alpha-takusan pseu	ENSMUSG00000068790 /	0.53056	1.15143333	0.20140246
1446104_at	---	---	0.53062	1.2408879	0.30573977
1452309_at	cingulin-like 1	Cgnl1	0.53064	1.26295113	0.31833907
1452146_a_at	COX15 homolog, cytochrome c oxidase assembly protein (yeast)	Cox15	0.53079	-1.18910291	-0.2592872
1431483_at	RIKEN cDNA 4930544M13 gene	4930544M13Rik	0.53094	-1.21063615	-0.2763065
1421975_a_at	adducin 2 (beta)	Add2	0.53103	1.20866061	0.2726219
1419463_at	chloride channel calcium activated 2	Clca2	0.53199	-1.21413575	-0.292036
1418123_at	unc-119 homolog (C. elegans)	Unc119	0.53214	1.15662384	0.20757762
1439438_a_at	SAP domain containing ribonucleoprotein	Sarnp	0.53219	-1.14762677	-0.2040314
1457155_at	aldehyde dehydrogenase 2, mitochondrial	Aldh2	0.53224	-1.23003915	-0.3077682
1435386_at	Von Willebrand factor homolog	Vwf	0.53249	-1.21362496	-0.2841936
1459154_at	---	---	0.53253	1.22092731	0.28115862
1456351_at	bromodomain containing 8	Brd8	0.53255	1.19417464	0.25178282
1438634_x_at	LIM and SH3 protein 1	Lasp1	0.53265	-1.17382713	-0.2432218
1449591_at	caspase 4, apoptosis-related cysteine peptidase	Casp4	0.53292	-1.20668332	-0.2786671
1422679_s_at	Ctr9, Paf1/RNA polymerase II complex component, homolog (S. c	Ctr9	0.53334	1.14806307	0.1983021
1427219_at	hypothetical LOC100504481 /// hypothetical LOC100504581	LOC100504481 /// LOC10	0.53414	-1.24249395	-0.3321076
1422528_a_at	zinc finger protein 36, C3H type-like 1	Zfp36l1	0.53426	-1.23540031	-0.3347746
1453762_at	vacuolar protein sorting 26 homolog B (yeast)	Vps26b	0.53454	1.16297647	0.21751401
1454991_at	solute carrier family 7 (cationic amino acid transporter, y+ system	Slc7a1	0.5346	-1.20884929	-0.2958551
1442220_at	---	---	0.53461	1.15569674	0.20733508
1421410_a_at	proline-serine-threonine phosphatase-interacting protein 2	Pstpip2	0.53462	1.2706398	0.33140307
1416370_at	zinc finger and SCAN domain containing 21	Zscan21	0.53464	1.15213338	0.20413271
1432077_at	protection of telomeres 1A	Pot1a	0.53469	1.17774257	0.23036987
1425160_at	cDNA sequence AF067063	AF067063	0.53469	-1.22565639	-0.3024504
1451409_at	RIKEN cDNA 2210021J22 gene	2210021J22Rik	0.53489	1.15343151	0.20486959
1424138_at	rhomboid family 1 (Drosophila)	Rhbdf1	0.53489	1.15356336	0.20507028
1424628_a_at	NADH dehydrogenase (ubiquinone) flavoprotein 3	Ndufv3	0.5349	1.16127397	0.2102406
1451380_at	zinc finger, FYVE domain containing 19	Zfyve19	0.53494	1.16867295	0.2232563
1434613_at	RIKEN cDNA 1810013L24 gene /// hypothetical protein LOC1005(	1810013L24Rik /// LOC10	0.53499	-1.16288451	-0.2204018
1454764_s_at	solute carrier family 38, member 1	Slc38a1	0.53505	-1.15903056	-0.2157198
1440920_at	matrix metallopeptidase 14 (membrane-inserted)	Mmp14	0.53514	1.16988504	0.22552762
1459011_at	---	---	0.53526	-1.2198558	-0.291953
1454831_at	forkhead box N2	Foxn2	0.53535	-1.15854613	-0.2178982
1457434_s_at	protein tyrosine phosphatase-like (proline instead of catalytic arg	Ptpla	0.53541	1.15817423	0.20909343
1433905_at	A kinase (PRKA) anchor protein 7	Akap7	0.53552	-1.15998681	-0.2153011
1449292_at	RB1-inducible coiled-coil 1	Rb1cc1	0.53561	-1.27747771	-0.3840349
1458738_at	---	---	0.53572	-1.22480412	-0.2996686
1418746_at	paroxysmal nonkinesiogenic dyskinesia	Pnkd	0.53572	1.16939846	0.22250516
1438278_a_at	CWC22 spliceosome-associated protein homolog (S. cerevisiae)	Cwc22	0.53572	-1.14679681	-0.1987406
1460537_at	RIKEN cDNA 9430085L16 gene	9430085L16Rik	0.53587	1.17225067	0.2209879
1452857_at	CREB/ATF bZIP transcription factor	Crebzf	0.53595	-1.1682695	-0.2316413
1424632_a_at	REV3-like, catalytic subunit of DNA polymerase zeta RAD54 like (S	Rev3l	0.53597	-1.152079	-0.2082695
1437070_at	CDC14 cell division cycle 14 homolog B (S. cerevisiae)	Cdc14b	0.53604	-1.19572454	-0.2641056
1438976_x_at	methionine adenosyltransferase II, alpha	Mat2a	0.53617	-1.21805951	-0.3092767
1450536_s_at	keratin associated protein 12-1	Krtap12-1	0.53655	-1.19605646	-0.2600274
1433747_at	leucyl/cystinyl aminopeptidase	Lnpep	0.53657	-1.14808879	-0.2011089
1457998_at	---	---	0.5367	-1.20294266	-0.2678831
1422799_at	HLA-B associated transcript 2	Bat2	0.53696	-1.14641809	-0.2006553
1436898_at	---	---	0.53699	-1.17235205	-0.2380241
1445734_at	---	---	0.53793	-1.18314756	-0.2574366
1434882_at	metadherin	Mtdh	0.53801	-1.15991131	-0.2200776
1431195_at	ring finger protein 170	Rnf170	0.53811	1.22627125	0.28836695
1458499_at	phosphodiesterase 10A	Pde10a	0.53816	1.23285384	0.29658879
1428624_at	magnesium transporter 1	Magt1	0.5383	-1.15494435	-0.2094856
1454766_at	antagonist of mitotic exit network 1 homolog (S. cerevisiae)	Amn1	0.53868	1.18950915	0.24922049
1416260_a_at	sorting nexin 1	Snx1	0.53885	1.15747547	0.20839427
1426671_a_at	RNA binding motif protein 39	Rbm39	0.53891	-1.13536794	-0.1835701
1435869_s_at	adaptor protein complex AP-2, alpha 2 subunit	Ap2a2	0.53898	-1.15422865	-0.2082166
1453259_at	inscuteable homolog (Drosophila)	Insc	0.53905	-1.18773783	-0.2503346
1455532_at	ataxin 7-like 2	Atxn7l2	0.53934	1.16332833	0.21779047
1423891_at	glutathione S-transferase, theta 3	Gstt3	0.53959	-1.2061207	-0.2748177
1417624_at	Ngfi-A binding protein 1	Nab1	0.5396	-1.16553897	-0.2226299
1428196_a_at	family with sequence similarity 82, member A2	Fam82a2	0.53987	1.14407153	0.19391999
1424093_x_at	CD151 antigen	Cd151	0.53989	1.14802565	0.19768024
1456110_at	ankyrin repeat domain 11	Ankrd11	0.53998	-1.20457338	-0.2818436
1455010_at	RIKEN cDNA 1500012F01 gene	1500012F01Rik	0.54012	1.17677864	0.22730995
1449877_s_at	kinesin family member C1	Kifc1	0.54021	1.18417489	0.2351328
1437553_at	BRCA1/BRCA2-containing complex, subunit 3	Brcc3	0.5403	-1.18630152	-0.2495984
1430719_at	RIKEN cDNA 4833447P13 gene	4833447P13Rik	0.54037	1.21937377	0.28305517
1456916_at	Nuclear receptor-binding SET-domain protein 1	Nsd1	0.54039	-1.24166892	-0.3239274
1417706_at	alpha-N-acetylglucosaminidase (Sanfilippo disease IIIB)	Naglu	0.54042	1.20882687	0.27109803
1437936_at	coiled-coil domain containing 160	Ccdc160	0.5405	1.169401	0.22131439
1459825_x_at	insulin-like growth factor 2 mRNA binding protein 1	Igf2bp1	0.54052	-1.15931234	-0.2187409
1439886_at	---	---	0.54053	-1.1923723	-0.2590155
1428091_at	kelch-like 7 (Drosophila)	Klhl7	0.54062	-1.15166684	-0.2048094
1460474_at	RIKEN cDNA 2610028L16 gene	2610028L16Rik	0.54075	1.19864932	0.25307916
1456382_at	ATPase family, AAA domain containing 1	Atad1	0.54077	-1.22248094	-0.2977791
1434434_s_at	transcription elongation regulator 1 (CA150)	Tcerg1	0.54097	-1.12866494	-0.1749885
1431835_at	transcription elongation regulator 1-like	Tcerg1l	0.54121	1.21069225	0.27343891
1458848_at	---	---	0.5426	-1.19880707	-0.2637531
1421193_a_at	pre B-cell leukemia transcription factor 3	Pbx3	0.5433	1.1766084	0.23397477
1438746_at	RIKEN cDNA A530058N18 gene	A530058N18Rik	0.54333	1.16311175	0.21493648
1430122_at	RIKEN cDNA 9430002A10 gene	9430002A10Rik	0.54356	1.26174618	0.32427226
1451189_at	zinc finger, SWIM domain containing 1	Zswim1	0.54358	-1.13781754	-0.1868633
1441501_at	serpine1 mRNA binding protein 1	Serbp1	0.54359	-1.17213752	-0.2308907
1431210_at	ATP/GTP binding protein-like 3	Agbl3	0.54359	1.150985	0.20186423
1426340_at	solute carrier family 1 (glial high affinity glutamate transporter), n	Slc1a3	0.54363	-1.23462251	-0.3253656
1428639_at	lin-9 homolog (C. elegans)	Lin9	0.54377	-1.26394809	-0.3746715

1434884_at	metadherin	Mtdh	0.54385	-1.24115157	-0.3462387
1437238_x_at	NMD3 homolog (S. cerevisiae)	Nmd3	0.54416	-1.14005317	-0.1901964
1426294_at	hyaluronan and proteoglycan link protein 1	Hapln1	0.54423	1.19572815	0.24817526
1426235_a_at	glutamate-ammonia ligase (glutamine synthetase)	Glul	0.54423	-1.14594621	-0.19957
1434179_at	myeloid/lymphoid or mixed-lineage leukemia 3	Mll3	0.54423	-1.16872841	-0.2295697
1421882_a_at	ELAV (embryonic lethal, abnormal vision, Drosophila)-like 2 (Hu a	Elavl2	0.54424	1.13136556	0.17776028
1437130_at	predicted gene 5465	Gm5465	0.54427	-1.18415561	-0.2494369
1447266_at	UTP18, small subunit (SSU) processome component, homolog (ye	Utp18	0.54432	1.15842582	0.20484143
1446430_at	HECT domain containing 2	Hectd2	0.54435	-1.26120812	-0.3634805
1452895_at	F-box protein 45	Fbxo45	0.54437	1.16808356	0.22403002
1426377_at	zinc finger protein 281	Zfp281	0.54444	-1.13810228	-0.1887106
1458327_x_at	solute carrier family 26 (sulfate transporter), member 1	Slc26a1	0.54444	1.1200492	0.16337778
1435634_at	protein-L-isoaspartate (D-aspartate) O-methyltransferase domai	Pcmtd1	0.54446	-1.16226504	-0.2227864
1442643_at	KDM1 lysine (K)-specific demethylase 6B	Kdm6b	0.54448	1.22970825	0.28786228
1449791_x_at	solute carrier family 38, member 9	Slc38a9	0.54451	1.29226291	0.34830698
1421059_a_at	asparagine-linked glycosylation 2 homolog (yeast, alpha-1,3-mar	Alg2	0.54451	1.15574224	0.20833274
AFFX-r2-Ec-bioC-!	---	---	0.54451	1.19354904	0.24486581
1440679_at	---	---	0.54452	-1.22420445	-0.2986455
1458059_at	Nucleolar protein 7	Nol7	0.54453	-1.21802585	-0.290346
1448357_at	small nuclear ribonucleoprotein polypeptide G	Snrpg	0.54457	1.12704003	0.16941862
1457021_x_at	anti-Mullerian hormone type 2 receptor	Amhr2	0.54476	1.17610812	0.2291046
1437457_a_at	myotrophin	Mtpn	0.54487	-1.13485113	-0.1827826
1428543_at	phosphoribosyl pyrophosphate amidotransferase	Ppat	0.54493	-1.15437793	-0.2101257
1427272_at	zinc finger and BTB domain containing 44	Zbtb44	0.54499	-1.18036331	-0.2398866
1452786_at	tRNA 5-methylaminomethyl-2-thiouridylate methyltransferase	Trmu	0.545	1.17297793	0.22607007
1451888_a_at	odd Oz/ten-m homolog 4 (Drosophila)	Odz4	0.54515	-1.29028442	-0.4131463
1422702_at	antizyme inhibitor 1	Azin1	0.54516	-1.13501506	-0.1828344
1457245_at	disrupted in renal carcinoma 2 (human)	Dirc2	0.54531	1.19806949	0.25971025
1417124_at	destrin	Dstn	0.54533	-1.11053671	-0.1515609
1427459_at	carboxypeptidase N, polypeptide 2	Cpn2	0.54545	1.21917805	0.28077074
1432115_a_at	phosphatidylinositol glycan anchor biosynthesis, class N	Pign	0.54572	-1.18574817	-0.2475949
1445001_at	---	---	0.54577	-1.19288005	-0.2575095
1455293_at	Leo1, Paf1/RNA polymerase II complex component, homolog (S.	Leo1	0.54586	1.14605525	0.19514621
1433691_at	protein phosphatase 1, regulatory (inhibitor) subunit 3C	Ppp1r3c	0.54593	1.20275703	0.26613838
1456244_x_at	glutaredoxin 3	Glrx3	0.54599	-1.27062077	-0.3983912
1433226_at	RIKEN cDNA 6330403N20 gene	6330403N20Rik	0.54604	1.1955195	0.25541227
1426371_at	fatty acyl CoA reductase 1	Far1	0.54606	-1.14666925	-0.2010863
1447222_at	heat shock protein 12A	Hspa12a	0.54606	-1.22052761	-0.2968194
1441339_at	chromodomain helicase DNA binding protein 9	Chd9	0.54609	1.23744342	0.29696404
1426366_at	eukaryotic translation initiation factor 2C, 2	Eif2c2	0.54611	-1.19177811	-0.2686757
1422698_s_at	jumonji, AT rich interactive domain 2	Jarid2	0.54617	-1.13258779	-0.1842443
1460248_at	carboxypeptidase X 2 (M14 family)	Cpxm2	0.54628	1.25838915	0.31950008
1448104_at	aldehyde dehydrogenase family 6, subfamily A1	Aldh6a1	0.5464	-1.14890135	-0.2010614
1441623_at	---	---	0.54658	1.18156213	0.23217837
1451677_at	nuclear prelamin A recognition factor	Narf	0.54662	1.18169146	0.23946975
1429807_at	IZUMO family member 3	Izumo3	0.5467	-1.18249677	-0.2427852
1457480_at	---	---	0.54708	1.33764133	0.38553603
1426981_at	proprotein convertase subtilisin/kexin type 6	Pcsk6	0.54719	1.14884036	0.19986669
1456230_at	RIKEN cDNA 4933422H20 gene	4933422H20Rik	0.54721	-1.2084426	-0.2758593
1439265_at	---	---	0.54724	1.18991013	0.24383841
1418444_a_at	glycerophosphodiester phosphodiesterase 1	Gde1	0.54729	1.15832302	0.20892965
1456853_at	predicted gene 4924	Gm4924	0.54733	-1.17785352	-0.2387995
1417851_at	chemokine (C-X-C motif) ligand 13	Cxcl13	0.54737	-1.20433065	-0.2749088
1444149_at	zinc finger and SCAN domain containing 12	Zscan12	0.54746	-1.19298076	-0.2563825
1446738_at	---	---	0.54751	1.21409188	0.27463052
1449084_s_at	SH3 domain protein D19	Sh3d19	0.54769	-1.15422666	-0.2070572
1420669_at	aryl hydrocarbon receptor nuclear translocator 2	Arnt2	0.54781	1.246351	0.30698143
1452438_s_at	TAF4A RNA polymerase II, TATA box binding protein (TBP)-associ	Taf4a	0.54782	-1.1505397	-0.2031344
1418185_at	NADH dehydrogenase (ubiquinone) 1 alpha subcomplex, assemb	Ndufaf3	0.54822	1.20702859	0.25876043
1455332_x_at	Fc receptor, IgG, low affinity IIb	Fcgr2b	0.54829	1.18944373	0.2471785
1418624_at	Y box protein 1	Ybx1	0.54851	-1.0833683	-0.1157486
1448961_at	phospholipid scramblase 2	Plscr2	0.54856	1.13391261	0.17933663
1447677_x_at	D-tyrosyl-tRNA deacylase 1 homolog (S. cerevisiae)	Dtd1	0.5486	-1.24042242	-0.3308041
1443807_x_at	cyclin F	Ccnf	0.54867	-1.19017125	-0.2675686
1456022_at	homeodomain interacting protein kinase 2	Hipk2	0.54868	-1.19986306	-0.2636961
1422849_a_at	poly(A) binding protein, nuclear 1	Pabpn1	0.54895	-1.09169879	-0.1288889
1422179_at	gap junction protein, beta 4	Gjb4	0.54936	1.18441567	0.24301041
1430001_at	interleukin 31	Il31	0.54997	1.17017554	0.22474468
1442442_at	---	---	0.55003	-1.20974386	-0.2761363
1435727_s_at	LIM domain and actin binding 1	Lima1	0.55013	1.13839694	0.18666979
1427021_s_at	ferritin heavy chain 1	Fth1	0.55019	1.08055868	0.11125197
1428105_at	TPX2, microtubule-associated protein homolog (Xenopus laevis)	Tpx2	0.55034	1.14764014	0.19791096
1447411_at	---	---	0.55039	1.22894085	0.29070799
1435079_at	serine/arginine-rich splicing factor 18	Sfrs18	0.55049	-1.17180153	-0.236014
1458584_at	RIKEN cDNA 4832406H04 gene	4832406H04Rik	0.55054	-1.21283583	-0.2835558
1415888_at	hepatoma-derived growth factor	Hdgf	0.55079	-1.23844737	-0.3473135
1439439_x_at	eukaryotic translation elongation factor 1 delta (guanine nucleoti	Eef1d	0.55098	-1.13207673	-0.1831268
1419685_at	UPF1 regulator of nonsense transcripts homolog (yeast)	Upf1	0.55106	-1.162599	-0.2177326
1429351_at	kelch-like 24 (Drosophila)	Klhl24	0.5511	-1.1542091	-0.2088309
1437495_at	membrane-bound transcription factor peptidase, site 2 /// Yy2 tra	Mbtps2 /// Yy2	0.5512	-1.17452265	-0.2347371
1431143_x_at	apoptosis-inducing factor, mitochondrion-associated 2	Aifm2	0.55121	1.16948734	0.22449226
1427055_at	thioesterase superfamily member 4	Them4	0.55151	1.24733464	0.30119389
1418555_x_at	Spi-C transcription factor (Spi-1/PU.1 related)	Spic	0.55154	1.23822265	0.29748095
1424847_at	neurofilament, heavy polypeptide	Nefh	0.55155	1.15735486	0.20825324
1426719_at	amyloid beta (A4) precursor protein-binding, family B, member 2	Apbb2	0.55176	1.16520845	0.22018386
1457473_at	chromodomain helicase DNA binding protein 1	Chd1	0.55186	-1.20318409	-0.2846009
1446918_at	---	---	0.55187	-1.20208739	-0.2685457
1457020_at	---	---	0.5519	-1.19185912	-0.2542339
1446901_at	expressed sequence AU022077	AU022077	0.55202	1.18382175	0.23863509
1433903_at	predicted gene 5081	Gm5081	0.55231	-1.1288031	-0.1752114
1432175_at	RIKEN cDNA 5430434I15 gene	5430434I15Rik	0.55253	-1.19930294	-0.2627411
1435448_at	BCL2-like 11 (apoptosis facilitator)	Bcl2l11	0.55267	1.15534571	0.20749548
1442466_a_at	diphosphoinositol pentakisphosphate kinase 1	Ppip5k1	0.55307	-1.22635196	-0.3024328
1437537_at	caspase 9	Casp9	0.5532	1.18648863	0.24470925

AFFX-r2-Ec-bioB-5	---	---	0.55347	1.18168318	0.2331335
1421341_at	axin2	Axin2	0.55348	1.20127481	0.26298245
1436222_at	growth arrest specific 5	Gas5	0.55363	-1.15774105	-0.2130066
1434681_at	taxilin gamma	Txlng	0.55435	1.14098634	0.18989727
1417823_at	glycine C-acetyltransferase (2-amino-3-ketobutyrate-coenzyme A	Gcat	0.5544	1.12835251	0.17342826
1424760_a_at	SET and MYND domain containing 2	Smyd2	0.55448	1.15352778	0.20342638
1426955_at	collagen, type XVIII, alpha 1	Col18a1	0.55473	1.13886082	0.18560673
1452985_at	uveal autoantigen with coiled-coil domains and ankyrin repeats	Uaca	0.55497	1.1827451	0.23448444
1452094_at	procollagen-proline, 2-oxoglutarate 4-dioxygenase (proline 4-hyd	P4ha1	0.555	1.14772158	0.19778056
1418011_a_at	SH3-domain GRB2-like B1 (endophilin)	Sh3glb1	0.555	1.17254932	0.22838176
1418997_at	LYR motif containing 5	Lyrm5	0.55508	-1.28973716	-0.4086647
1430460_at	family with sequence similarity 188, member A	Fam188a	0.55516	1.19496188	0.25611535
1426834_s_at	RIKEN cDNA D930015E06 gene	D930015E06Rik	0.55522	1.16379024	0.21607867
1439122_at	DEAD (Asp-Glu-Ala-Asp) box polypeptide 6	Ddx6	0.55543	-1.20216294	-0.2777873
1456550_at	BCL2-like 12 (proline rich)	Bcl2l12	0.55543	-1.24373941	-0.332851
1420161_at	expressed sequence AA409749	AA409749	0.55547	1.29928817	0.3511557
1457828_at	signal transducing adaptor molecule (SH3 domain and ITAM moti	Stam	0.55559	1.23907928	0.30049868
1459680_at	---	---	0.5557	1.23703454	0.297432
1437359_at	ribonucleic acid binding protein S1	Rnps1	0.55572	-1.1989754	-0.268972
1420056_s_at	jumonji domain containing 6	Jmjd6	0.55573	1.14427534	0.19349535
1454990_at	AT rich interactive domain 2 (ARID, RFX-like)	Arid2	0.55583	-1.14663363	-0.2005203
1421210_at	class II transactivator	Ciita	0.55593	1.21899249	0.28250653
1434292_at	small nucleolar RNA host gene 11	Snhg11	0.55604	1.21294503	0.2702539
1439304_at	RIKEN cDNA B230216N24 gene	B230216N24Rik	0.55605	1.18916314	0.24866669
1450401_at	trimethylguanosine synthase homolog (S. cerevisiae)	Tgs1	0.55607	-1.18982617	-0.2649393
1455389_s_at	RIKEN cDNA 2310051F07 gene	2310051F07Rik	0.55613	-1.1360824	-0.1862939
1448176_a_at	heterogeneous nuclear ribonucleoprotein K	Hnrnpk	0.55623	-1.09653162	-0.1329593
1456669_at	RIKEN cDNA 1110018N20 gene	1110018N20Rik	0.55625	1.19338432	0.25336067
1415973_at	myristoylated alanine rich protein kinase C substrate	Marcks	0.55626	1.16677117	0.21950238
1453447_at	RIKEN cDNA 1700109H08 gene	1700109H08Rik	0.5563	1.19417294	0.25414996
1441333_at	---	---	0.5563	-1.26402031	-0.375275
1417951_at	enolase 3, beta muscle	Eno3	0.55634	1.13339951	0.17918741
1457579_at	DNA segment, Chr 11, ERATO Doi 717, expressed	D11Ertd717e	0.55638	1.22035712	0.28096529
1449653_at	---	---	0.55643	-1.1963829	-0.2659064
1433648_at	sperm associated antigen 9	Spag9	0.55651	1.16625635	0.21812651
1454160_at	RIKEN cDNA 4930483C13 gene	4930483C13Rik	0.55664	1.18219393	0.23973076
1433920_at	sema domain, immunoglobulin domain (Ig), transmembrane dom	Sema4c	0.55664	1.23181611	0.29244997
1457474_at	---	---	0.55713	1.2379563	0.30071711
1420906_at	CD2-associated protein	Cd2ap	0.55717	-1.17148727	-0.2328799
1459981_s_at	rosbin, round spermatid basic protein 1	Rsbn1	0.55729	-1.16027487	-0.2158932
1452600_at	TAF6-like RNA polymerase II, p300/CBP-associated factor (PCAF)	Taf6l	0.55745	1.1471748	0.19794718
1454665_at	interferon regulatory factor 2 binding protein 2	Irf2bp2	0.55751	1.22865883	0.28855182
1429762_a_at	Bardet-Biedl syndrome 5 (human)	Bbs5	0.55755	1.16738911	0.22283396
1453313_at	sestrin 3	Sesn3	0.5576	1.24100671	0.29357543
1422798_at	contactin associated protein-like 2	Cntnap2	0.5581	1.21232667	0.26977579
1438786_a_at	RIKEN cDNA 2610021A01 gene	2610021A01Rik	0.55821	-1.20771231	-0.2896163
1439129_at	dedicator of cytokinesis 5	Dock5	0.55822	-1.18202038	-0.2413763
1437094_x_at	dynein, axonemal, intermediate chain 1	Dnaic1	0.55834	1.17597259	0.231659
1458058_at	RIKEN cDNA 7030407E18 gene	7030407E18Rik	0.5584	1.21658699	0.2793182
1428942_at	metallothionein 2	Mt2	0.55855	1.08665794	0.11934743
1438041_at	phosphodiesterase 7A	Pde7a	0.55872	1.16707386	0.22135485
1425656_a_at	brain-specific angiogenesis inhibitor 1-associated protein 2	Baiap2	0.55879	1.15560174	0.2085675
1434318_a_at	transcription factor E3	Tcfe3	0.55883	1.15222276	0.20082922
1430628_at	RIKEN cDNA A230083N12 gene	A230083N12Rik	0.55895	-1.19905123	-0.2667897
1440227_at	solute carrier family 5 (inositol transporters), member 3	Slc5a3	0.55899	-1.20072871	-0.2669889
1438344_at	RIKEN cDNA 4833424O15 gene	4833424O15Rik	0.5591	1.15931094	0.21003304
1420006_at	Bone morphogenetic protein 15	Bmp15	0.55946	-1.20138305	-0.2778776
1443622_at	---	---	0.55956	-1.20055713	-0.2675027
1416124_at	cyclin D2	Ccnd2	0.55974	-1.24956243	-0.3517657
1448181_at	Kruppel-like factor 15	Klf15	0.55974	-1.15209584	-0.2052336
1449456_a_at	chymase 1, mast cell	Cma1	0.5599	-1.2261823	-0.3081625
1416948_at	mitochondrial ribosomal protein L23	Mrpl23	0.56012	1.16821631	0.21619346
1444894_at	Protein tyrosine phosphatase, non-receptor type 18	Ptpn18	0.56119	-1.22290944	-0.3021249
1416901_at	Niemann Pick type C2	Npc2	0.56122	1.13170244	0.17736747
1426774_at	poly (ADP-ribose) polymerase family, member 12	Parp12	0.5614	1.20985156	0.2635804
1417062_at	armadillo repeat containing 10	Armc10	0.56146	1.13613158	0.1839816
1460707_at	protein tyrosine phosphatase 4a2	Ptp4a2	0.5616	-1.12872796	-0.1756995
1416423_x_at	Sjogren syndrome antigen B	Ssb	0.56161	-1.14233058	-0.1927254
1438955_x_at	peptidylprolyl isomerase F (cyclophilin F)	Ppif	0.56167	-1.19081892	-0.2621493
1421351_at	glutamate receptor, ionotropic, AMPA4 (alpha 4)	Gria4	0.56169	1.20355347	0.26434018
1429556_at	TEA domain family member 1	Tead1	0.5617	-1.15412216	-0.2096766
1439455_x_at	capping protein (actin filament) muscle Z-line, alpha 1	Capza1	0.56173	-1.17023862	-0.2394813
1455993_at	odd Oz/ten-m homolog 4 (Drosophila)	Odz4	0.56173	-1.20610808	-0.2786828
1424040_at	microtubule-associated protein 7 domain containing 1	Mtap7d1	0.56174	1.15650333	0.20592057
1449115_at	metal response element binding transcription factor 2	Mtf2	0.56183	-1.17537303	-0.2486849
1455375_at	---	---	0.5619	-1.15991285	-0.2164798
1416736_at	cancer susceptibility candidate 3	Casc3	0.5619	1.20479654	0.2544993
1450150_a_at	ribosomal protein L13	Rpl13	0.56195	1.06543405	0.09131647
1425046_at	cDNA sequence BC018465	BC018465	0.56204	-1.20602914	-0.2752354
1444234_at	N-ethylmaleimide sensitive fusion protein attachment protein bet	Napb	0.56208	1.19479567	0.2557043
1428437_at	LSM14 homolog A (SCD6, S. cerevisiae)	Lsm14a	0.56213	-1.13112877	-0.1786871
1449976_a_at	G protein-coupled receptor 35	Gpr35	0.56213	1.21087068	0.27272838
1426606_at	cartilage acidic protein 1	Crtac1	0.56215	1.20149361	0.26040914
1441743_at	paired box gene 3	Pax3	0.56215	-1.17432382	-0.238407
1431044_at	THO complex 1	Thoc1	0.56215	-1.21873025	-0.2949946
1436818_a_at	Musashi homolog 2 (Drosophila)	Msi2	0.56218	-1.13544331	-0.1856407
1430149_at	NADH dehydrogenase (ubiquinone) 1 alpha subcomplex, 3	Ndufa3	0.56218	1.22001449	0.2836468
1429007_at	solute carrier family 35, member B2	Slc35b2	0.56219	1.15607841	0.20680263
1435529_at	predicted gene 14446	Gm14446	0.56222	-1.20881814	-0.2770746
1447513_at	potassium voltage-gated channel, Shal-related family, member 3	Kcnd3	0.56223	1.19306943	0.24673148
1424752_x_at	zinc finger protein 71, related sequence /// zinc finger protein 738	Zfp71-rs1 /// Zfp738	0.5623	-1.17522142	-0.2410672
1455573_at	keratin 14	Krt14	0.56235	-1.19282215	-0.2546352
1450093_s_at	zinc finger and BTB domain containing 7a	Zbtb7a	0.56236	-1.2077895	-0.2823507
1434531_at	mannoside acetylglucosaminyltransferase 5, isoenzyme B	Mgat5b	0.56238	1.21033422	0.26617447

1423821_at	transmembrane protein 168	Tmem168	0.56239	-1.19430818	-0.2682154
1432126_at	RIKEN cDNA 1700072H12 gene	1700072H12Rik	0.56243	1.20391708	0.26385871
1422927_at	Yip1 domain family, member 7	Yipf7	0.56246	-1.19511261	-0.2671853
1440508_at	hypothetical LOC100503381	LOC100503381	0.56249	-1.19729888	-0.2611966
1450069_a_at	CUGBP, Elav-like family member 2	Celf2	0.56266	-1.17092012	-0.2417393
1419411_at	tachykinin 2	Tac2	0.56268	1.23856923	0.2997794
1456433_at	regulator of chromosome condensation (RCC1) and BTB (POZ) do	Rcbtb1	0.56269	1.14796123	0.196805
1439403_x_at	ring finger protein, LIM domain interacting	Rlim	0.56271	-1.19252057	-0.2624576
1439884_at	nudix (nucleoside diphosphate linked moiety X)-type motif 16	Nudt16	0.56277	-1.19207269	-0.2560809
1456664_x_at	heterogeneous nuclear ribonucleoprotein F /// hypothetical LOC1	Hnrnpf /// LOC100503154	0.56297	-1.10887984	-0.1509492
1448848_at	torsin family 1, member B	Tor1b	0.56297	1.15538302	0.20551434
1426401_at	protein phosphatase 3, catalytic subunit, alpha isoform	Ppp3ca	0.56301	-1.17628332	-0.2402733
1426956_a_at	transformation related protein 53 binding protein 1	Trp53bp1	0.56304	1.14860601	0.19847825
1452020_a_at	SIVA1, apoptosis-inducing factor	Siva1	0.56311	1.14962734	0.19530161
1415792_at	RanBP-type and C3HC4-type zinc finger containing 1	Rbck1	0.56329	1.13494162	0.18190047
1452983_at	centrosomal protein 57	Cep57	0.5633	-1.15977435	-0.2175436
1417937_at	dapper homolog 1, antagonist of beta-catenin (xenopus)	Dact1	0.56331	1.17687723	0.2322645
1437280_s_at	serpine1 mRNA binding protein 1	Serbp1	0.56331	-1.07915208	-0.110527
1421108_at	camello-like 2	Cml2	0.56332	-1.18038031	-0.2398384
1416591_at	RAB34, member of RAS oncogene family	Rab34	0.56345	1.16800264	0.2198938
1449072_a_at	N-6 adenine-specific DNA methyltransferase 2 (putative)	N6amt2	0.56348	1.15442013	0.20221857
1428405_at	host cell factor C1 regulator 1 (XPO1-dependent)	Hcfc1r1	0.56349	1.16661847	0.21529719
1439055_at	transcription elongation factor B (SIII), polypeptide 2 pseudogene	Gm15698	0.56356	-1.2476336	-0.3422391
1446319_at	ankyrin repeat and SOCS box-containing 7	Asb7	0.56359	1.20827389	0.26950529
1427254_at	zinc finger protein 445	Zfp445	0.56363	-1.2037152	-0.280579
1447936_at	RIKEN cDNA 2410006H16 gene	2410006H16Rik	0.56375	1.16866139	0.21704067
1417476_at	F-box and WD-40 domain protein 5	Fbxw5	0.56377	1.17342982	0.22849408
1417175_at	casein kinase 1, epsilon	Csnk1e	0.56459	1.167799	0.21669755
1459237_at	---	---	0.56471	-1.27425725	-0.3887603
1444599_at	hect domain and RLD 4	Herc4	0.56478	-1.23736773	-0.328058
1428792_at	breast carcinoma amplified sequence 1	Bcas1	0.56497	1.2416383	0.29786762
1449144_at	guanine nucleotide binding protein, alpha 11	Gna11	0.56502	1.15551769	0.20440783
1440244_at	avian erythroblastosis virus E-26 (v-ets) oncogene related	Erg	0.56504	1.22658918	0.28610529
1444437_at	ubiquitin specific peptidase 34	Usp34	0.56522	-1.22722039	-0.3104776
1454832_at	phosphatase and actin regulator 1	Phactr1	0.56525	1.23449522	0.29322091
1438322_x_at	farnesyl diphosphate farnesyl transferase 1	Fdft1	0.56531	1.15867432	0.20593229
1418470_at	Yamaguchi sarcoma viral (v-yes) oncogene homolog 1	Yes1	0.56533	-1.18357691	-0.2517719
1456877_at	dual oxidase 1	Duox1	0.56579	1.19507221	0.25550682
1447614_at	schlafen 1	Slfn1	0.56585	-1.22335032	-0.306832
1423942_a_at	calcium/calmodulin-dependent protein kinase II gamma	Camk2g	0.56594	1.16447887	0.21582021
1453855_at	matrix-remodelling associated 7	Mxra7	0.56604	-1.20738727	-0.2829231
1457481_at	---	---	0.56724	-1.19479203	-0.2583366
1449528_at	c-fos induced growth factor	Figf	0.5674	1.21752342	0.27737021
1426534_a_at	ADP-ribosylation factor GTPase activating protein 3	Arfgap3	0.5675	1.16030975	0.21330308
1455571_x_at	calmodulin 1	Calm1	0.56758	-1.12185397	-0.1680846
1446155_at	RIKEN cDNA 2700089E24 gene	2700089E24Rik	0.5677	-1.18791469	-0.2490951
1459798_x_at	TRM2 tRNA methyltransferase 2 homolog B (S. cerevisiae)	Trmt2b	0.56772	1.14183531	0.18683901
1457084_at	NLR family, pyrin domain containing 9B	Nlrp9b	0.56773	1.17006315	0.2262732
1424420_at	cell cycle progression 1	Ccpg1	0.56782	-1.15075727	-0.202978
1420937_at	cleavage and polyadenylation specific factor 2	Cpsf2	0.56793	-1.17496037	-0.2446159
1453229_s_at	ubiquinol-cytochrome c reductase hinge protein	Uqcrh	0.56797	1.12501034	0.16678956
1437885_at	RIKEN cDNA D030029J20 gene /// hypothetical LOC100502854	D030029J20Rik /// LOC10	0.56798	-1.18577974	-0.2498553
1440801_s_at	adrenergic receptor kinase, beta 2	Adrbk2	0.56802	-1.18220743	-0.2415407
1449799_s_at	plakophilin 2	Pkp2	0.56802	1.13670248	0.18270352
1421097_at	endonuclease G	Endog	0.56803	1.16543836	0.21951139
1457931_at	---	---	0.56803	-1.18056442	-0.241619
1435772_at	kinesin family member 21B	Kif21b	0.56807	1.15383136	0.20491577
1427893_a_at	phosphomevalonate kinase	Pmvk	0.56808	1.13737796	0.18471864
1428082_at	acyl-CoA synthetase long-chain family member 5	Acsl5	0.5681	1.16333621	0.21678702
1425031_at	fukutin	Fktn	0.56811	-1.25598061	-0.3602487
1451739_at	Kruppel-like factor 5	Klf5	0.56813	-1.19674099	-0.2755122
1421207_at	leukemia inhibitory factor	Lif	0.56813	1.17761544	0.23517631
1456958_at	RIKEN cDNA C230072F16 gene	C230072F16Rik	0.56815	-1.22750194	-0.3100084
1453003_at	sortilin-related receptor, LDLR class A repeats-containing	Sorl1	0.56815	1.17494852	0.22904005
1430208_at	keratin 42	Krt42	0.56826	1.18778264	0.23884605
1448237_x_at	lactate dehydrogenase B	Ldhb	0.5683	1.17999801	0.22762967
1433082_at	glyceraldehyde-3-phosphate dehydrogenase pseudogene	4930448K20Rik	0.56831	-1.193957	-0.2595384
1421858_at	a disintegrin and metallopeptidase domain 17	Adam17	0.56831	1.16134616	0.21300593
1419555_at	E74-like factor 5	Elf5	0.56837	1.24231464	0.30008426
1417728_at	methyl-CpG binding domain protein 3	Mbd3	0.56838	1.19695712	0.24438386
1443586_at	FIP1 like 1 (S. cerevisiae)	Fip1l1	0.56842	-1.20811989	-0.2778901
1427558_s_at	asparagine-linked glycosylation 12 homolog (yeast, alpha-1,6-ma	Alg12	0.56842	1.16582506	0.21932205
1435574_at	---	---	0.56844	-1.22657724	-0.3110318
1428162_at	RIKEN cDNA 4933421E11 gene	4933421E11Rik	0.56845	-1.20930607	-0.2960346
1449414_at	zinc finger protein 53	Zfp53	0.5685	-1.16538112	-0.2217744
1448727_at	transducin-like enhancer of split 6, homolog of Drosophila E(spl)	Tle6	0.56853	1.16554718	0.21827653
1447544_at	---	---	0.56854	1.16287678	0.21675725
1432881_at	RIKEN cDNA 4932437C15 gene	4932437C15Rik	0.56854	-1.19053089	-0.252101
1449857_at	RIKEN cDNA 1200011I18 gene	1200011I18Rik	0.56854	-1.16225142	-0.2195109
1433744_at	leucine-rich repeats and transmembrane domains 2	Lrtm2	0.56858	-1.20441544	-0.2722136
1416799_at	transient receptor potential cation channel, subfamily M, membe	Trpm7	0.56858	-1.12978378	-0.1773521
1449236_at	delta-like 3 (Drosophila)	Dll3	0.56859	1.20126851	0.25842533
1454927_at	zinc finger protein 41	Zfp41	0.56866	1.14231107	0.1914166
1433977_at	heparan sulfate (glucosamine) 3-O-sulfotransferase 3B1	Hs3st3b1	0.56875	1.25857214	0.31543008
1423977_at	RIKEN cDNA 4930453N24 gene	4930453N24Rik	0.5688	-1.2198798	-0.2978873
1454297_at	RIKEN cDNA 4631402F24 gene	4631402F24Rik	0.56883	-1.1889932	-0.2499511
1416206_at	signal-induced proliferation associated gene 1	Sipa1	0.5689	1.15579611	0.20852935
1442452_at	STE20-related kinase adaptor beta	Stradb	0.56909	-1.1795886	-0.2385414
1455697_at	family with sequence similarity 124, member A	Fam124a	0.56921	-1.22655722	-0.3150859
1432997_at	RIKEN cDNA 5830462P14 gene	5830462P14Rik	0.56948	-1.19074927	-0.2533899
1418057_at	T-cell lymphoma invasion and metastasis 1	Tiam1	0.56954	1.19758586	0.24921553
1452246_at	osteoclast stimulating factor 1	Ostf1	0.56961	1.1618671	0.21233456
1448930_at	RIKEN cDNA 3010026O09 gene	3010026O09Rik	0.56966	1.17568782	0.22951685
1458374_at	expressed sequence C79407	C79407	0.56966	-1.1639986	-0.2275083

1429689_at	RIKEN cDNA 4932433N03 gene	4932433N03Rik	0.56966	-1.1678679	-0.2264672
1428586_at	transmembrane protein 41B	Tmem41b	0.5698	-1.20789989	-0.2973339
1415801_at	gap junction protein, alpha 1	Gja1	0.56994	1.16515849	0.21481775
1435182_at	coiled-coil domain containing 61	Ccdc61	0.56995	1.17605586	0.22811556
1417541_at	helicase, lymphoid specific	Hells	0.57004	-1.12003566	-0.1649183
1426114_at	heterogeneous nuclear ribonucleoprotein A/B	Hnrnpab	0.57005	1.10954741	0.14779372
1459666_at	trafficking protein, kinesin binding 1	Trak1	0.57066	1.15740518	0.20981411
1451318_a_at	Yamaguchi sarcoma viral (v-yes-1) oncogene homolog	Lyn	0.57069	1.17552499	0.23059839
1453451_at	RIKEN cDNA 6820402I19 gene	6820402I19Rik	0.57098	-1.2218598	-0.302777
1422158_at	calsyntenin 2	Clstn2	0.57104	-1.17794022	-0.2389778
1418653_at	cytochrome P450, family 2, subfamily c, polypeptide 50	Cyp2c50	0.57104	1.24431395	0.29957459
1446858_at	expressed sequence AU022245	AU022245	0.5713	-1.16117934	-0.2167987
1450136_at	CD38 antigen	Cd38	0.57141	1.2284868	0.28643139
1438266_at	hypothetical LOC100503741	LOC100503741	0.57206	-1.1987017	-0.2674811
1428858_at	WD repeat domain 70	Wdr70	0.5721	1.17440297	0.22597455
1416696_at	DNA segment, Chr 17, Wayne State University 104, expressed	D17Wsu104e	0.57211	1.16196912	0.21189187
1446737_a_at	hook homolog 3 (Drosophila)	Hook3	0.57216	1.19797783	0.25607876
1453160_at	mediator complex subunit 13	Med13	0.57221	-1.16066534	-0.2188214
1452535_at	Immunoglobulin heavy chain 6 (heavy chain of IgM)	Igh-6	0.57231	1.18591913	0.24565789
1428304_at	establishment of cohesion 1 homolog 2 (S. cerevisiae)	Esco2	0.57234	-1.1257791	-0.1718786
1425241_a_at	WD repeat and SOCS box-containing 1	Wsb1	0.57238	-1.16917286	-0.2313408
1441462_at	dedicator of cytokinesis 4	Dock4	0.57243	-1.20496591	-0.2739293
1447176_at	---	---	0.57249	1.23188874	0.29048586
1439773_at	lymphocyte antigen 6 complex, locus E	Ly6e	0.57258	1.17023202	0.22637275
1427467_a_at	retinitis pigmentosa GTPase regulator	Rpgr	0.57301	-1.1626168	-0.2177586
1454182_at	RIKEN cDNA 5430417C01 gene	5430417C01Rik	0.57332	1.18753425	0.24619862
1438902_a_at	heat shock protein 90, alpha (cytosolic), class A member 1	Hsp90aa1	0.5734	-1.08171494	-0.1158737
1422732_at	polymerase (DNA-directed), delta interacting protein 2	Poldip2	0.57381	1.1303011	0.17554146
1423954_at	complement component 3	C3	0.57385	1.23442025	0.2957874
1457561_at	integrin alpha 5 (fibronectin receptor alpha)	Itga5	0.57392	-1.26994904	-0.3921114
1452599_s_at	expressed sequence AI413582	AI413582	0.57396	1.19827179	0.25347468
1432963_at	RIKEN cDNA 2410049M19 gene	2410049M19Rik	0.57396	1.1883638	0.24829847
1426400_a_at	calpain, small subunit 1	Capns1	0.57402	1.11879334	0.16117758
1449167_at	erythrocyte protein band 4.1-like 4a	Epb4.1l4a	0.57407	-1.13637141	-0.1872927
1439704_at	histone deacetylase 2	Hdac2	0.57409	-1.23531048	-0.3240478
1418553_at	rho/rac guanine nucleotide exchange factor (GEF) 18	Arhgef18	0.5742	1.14705562	0.19590726
1424325_at	establishment of cohesion 1 homolog 1 (S. cerevisiae)	Esco1	0.5742	-1.19540827	-0.2643196
1450954_at	YME1-like 1 (S. cerevisiae)	Yme1l1	0.57422	-1.17539518	-0.2449431
1423427_at	adenylate cyclase activating polypeptide 1	Adcyap1	0.57435	-1.23163096	-0.3165096
1423840_at	coiled-coil domain containing 56	Ccdc56	0.57437	1.14031729	0.18900215
1426334_a_at	BCL2-like 11 (apoptosis facilitator)	Bcl2l11	0.57437	1.17452965	0.2311557
1422742_at	human immunodeficiency virus type I enhancer binding protein 1	Hivep1	0.57438	-1.18006502	-0.2426728
1433354_at	RIKEN cDNA D530015H24 gene	D530015H24Rik	0.57442	1.17381566	0.23087731
1430285_at	Ppp1r14c pseudogene	Gm14057	0.57443	1.23140251	0.289979
1434620_s_at	family with sequence similarity 13, member B	Fam13b	0.57445	-1.12170864	-0.1714234
1429823_at	sarcoglycan, gamma (dystrophin-associated glycoprotein)	Sgcg	0.57446	-1.21413282	-0.2940999
1460547_a_at	heterogeneous nuclear ribonucleoprotein K	Hnrnpk	0.5745	-1.13587146	-0.1930403
1450474_at	serine (or cysteine) peptidase inhibitor, clade B, member 9c	Serpinb9c	0.5745	1.13906473	0.18706185
1456716_s_at	RIKEN cDNA 3110002H16 gene	3110002H16Rik	0.57452	1.16049529	0.21251334
1450389_s_at	phosphatidylinositol-4-phosphate 5-kinase, type 1 beta	Pip5k1b	0.57452	1.21147822	0.27097262
1453301_a_at	alkB, alkylation repair homolog 4 (E. coli)	Alkbh4	0.57456	1.18521288	0.23847676
1438227_at	src homology 2 domain-containing transforming protein E	She	0.57457	1.33500022	0.37752566
1416233_at	eukaryotic translation initiation factor 3, subunit I	Eif3i	0.57458	1.10363848	0.14166416
1423951_at	TM2 domain containing 3	Tm2d3	0.57459	1.1469723	0.19376348
1427888_a_at	spectrin alpha 2	Spna2	0.5746	-1.14531816	-0.2021629
1442618_at	---	---	0.57464	1.21506077	0.27236715
1450575_at	cholinergic receptor, muscarinic 4	Chrm4	0.57466	1.21082394	0.27298829
1436892_at	sprouty-related, EVH1 domain containing 2	Spred2	0.57468	-1.29091137	-0.4337816
1422331_at	POU domain, class 3, transcription factor 3	Pou3f3	0.57469	1.23652065	0.29579793
1453709_at	Rho GTPase activating protein 32	Arhgap32	0.5747	1.19867906	0.26022567
1455440_at	chloride channel 6	Clcn6	0.5747	1.15259941	0.20281904
1438360_x_at	solute carrier family 25 (mitochondrial carrier, adenine nucleotide	Slc25a5	0.57472	-1.25869831	-0.3845306
1458387_at	phosphatidylserine synthase 2	Ptdss2	0.57475	-1.20298608	-0.2682084
1423333_at	endoplasmic reticulum-golgi intermediate compartment (ERGIC) 1	Ergic1	0.57477	1.13821609	0.18489693
1444650_at	rho/rac guanine nucleotide exchange factor (GEF) 18	Arhgef18	0.57477	1.24778307	0.30364549
1429794_a_at	purinergic receptor P2X, ligand-gated ion channel, 1	P2rx1	0.57477	-1.2104986	-0.2862641
1448270_at	DEAD (Asp-Glu-Ala-Asp) box polypeptide 21	Ddx21	0.57478	-1.15949476	-0.2261309
1425759_at	NOBOX oogenesis homeobox	Nobox	0.57481	1.21370182	0.27310872
1450869_at	fibroblast growth factor 1	Fgf1	0.57482	-1.21351049	-0.28692
1449357_at	RIKEN cDNA 2310030G06 gene	2310030G06Rik	0.57482	1.17994934	0.23277871
1447242_at	---	---	0.57483	-1.1916711	-0.2539759
1428908_at	RNA binding motif protein 25	Rbm25	0.57484	-1.13842616	-0.190253
1432933_at	RIKEN cDNA 4930429N05 gene	4930429N05Rik	0.57487	1.1845154	0.24224619
1437593_x_at	apoptosis inhibitor 5	Api5	0.57489	-1.11394158	-0.1578051
1439138_at	---	---	0.5749	-1.18759868	-0.2501415
1435753_a_at	nuclear casein kinase and cyclin-dependent kinase substrate 1	Nucks1	0.57491	-1.15800511	-0.2199754
1435373_at	casein kinase 1, epsilon	Csnk1e	0.57491	1.17463061	0.2309194
1425009_at	t-complex-associated testis expressed 2	Tcte2	0.57496	1.1935874	0.25400469
1447696_x_at	adenylate cyclase 5	Adcy5	0.57497	-1.15629309	-0.2095687
1455026_at	sno, strawberry notch homolog 1 (Drosophila)	Sbno1	0.57497	-1.24301605	-0.3568315
1460027_at	cDNA sequence BC061194	BC061194	0.57498	1.12830793	0.17331955
1417430_at	cerebellar degeneration-related 2	Cdr2	0.575	1.1502139	0.20042803
1447234_s_at	sorting nexin 6	Snx6	0.57504	-1.15556278	-0.2151374
1445088_at	---	---	0.57504	1.19674999	0.25319853
1434224_at	transducin (beta)-like 2	Tbl2	0.57514	1.13941782	0.18778288
1443749_x_at	solute carrier family 1 (glial high affinity glutamate transporter), m	Slc1a3	0.57515	-1.18263009	-0.2449114
1423801_a_at	adenine phosphoribosyl transferase	Aprt	0.57517	1.1376256	0.18340266
1439316_at	RNA binding motif protein 39	Rbm39	0.57521	-1.19904829	-0.267183
1420730_a_at	t-complex protein 11	Tcp11	0.57526	1.16826442	0.22158095
1419946_s_at	---	---	0.57527	-1.21561879	-0.2961533
1434353_at	Scm-like with four mbt domains 2	Sfmbt2	0.57528	1.17799162	0.2265745
1426851_a_at	nephroblastoma overexpressed gene	Nov	0.57529	1.18130884	0.23704387
1439082_at	DEAD (Asp-Glu-Ala-Asp) box polypeptide 50	Ddx50	0.57533	-1.18103397	-0.2408709
1416178_a_at	pleckstrin homology domain containing, family B (evectins) mem	Plekhb1	0.57537	-1.15034529	-0.2029656

1416885_at	RIKEN cDNA 1110038F14 gene	1110038F14Rik	0.57537	1.16933487	0.21890625
1445400_at	---	---	0.57541	-1.18601929	-0.246404
1433866_x_at	peroxiredoxin 1	Prdx1	0.57542	-1.19882951	-0.2908033
1434499_a_at	lactate dehydrogenase B	Ldhb	0.57544	1.14509953	0.19004165
1423339_s_at	zinc finger protein 830	Zfp830	0.57544	-1.20676113	-0.2785486
1428327_at	trafficking protein, kinesin binding 1	Trak1	0.57546	1.18480477	0.23660107
1459681_at	---	---	0.57553	1.19719062	0.25418003
1432892_at	dehydrogenase/reductase (SDR family) member 7	Dhrs7	0.57558	1.2181143	0.27842614
1418507_s_at	suppressor of cytokine signaling 2	Socs2	0.57559	-1.12529826	-0.1710335
1448088_at	expressed sequence C78228	C78228	0.5756	-1.20605235	-0.2736858
1420947_at	alpha thalassemia/mental retardation syndrome X-linked homolo	Atrx	0.57564	-1.21424387	-0.2937444
1442569_at	---	---	0.57565	-1.18654999	-0.2520528
1444036_at	---	---	0.57566	-1.20912	-0.2784871
1422125_at	5-hydroxytryptamine (serotonin) receptor 2B	Htr2b	0.57567	-1.1865672	-0.2501921
1458640_at	---	---	0.57568	1.17557477	0.23316704
1437618_x_at	G protein-coupled receptor 85	Gpr85	0.57568	-1.15942362	-0.2191902
1444411_at	---	---	0.57571	-1.18391808	-0.2437547
1430969_at	mitochondrial carrier homolog 2 (C. elegans)	Mtch2	0.57579	-1.20445559	-0.2732507
1431261_at	chloride intracellular channel 5	Clic5	0.57585	1.29682584	0.34454667
1448112_at	cytochrome c oxidase, subunit VIIc	Cox7c	0.57585	1.0915108	0.12578264
1459232_at	---	---	0.57587	1.22757249	0.28490371
1429185_at	RIKEN cDNA 4631416L12 gene	4631416L12Rik	0.5759	-1.17578634	-0.2355657
1427349_x_at	RIKEN cDNA 2810021G02 gene	2810021G02Rik	0.57592	-1.14769484	-0.2014447
1420023_at	eukaryotic translation termination factor 1	Etf1	0.57593	-1.1519978	-0.211032
1426035_at	serine incorporator 4	Serinc4	0.57593	-1.19285661	-0.2612191
1451611_at	phospholipase A2, group XVI	Pla2g16	0.57595	1.1939946	0.24369631
1448632_at	proteasome (prosome, macropain) subunit, beta type 10	Psmb10	0.57595	1.15702822	0.20583097
1420022_s_at	---	---	0.57599	-1.20524412	-0.2773533
1425828_at	NK6 homeobox 1	Nkx6-1	0.57599	-1.20538062	-0.2759986
1437607_at	Glucosaminyl (N-acetyl) transferase 2, I-branching enzyme	Gcnt2	0.576	-1.1308001	-0.1774897
1434788_at	frizzled homolog 3 (Drosophila)	Fzd3	0.57602	1.17734148	0.233933
1436332_at	heat shock protein, alpha-crystallin-related, B6	Hspb6	0.57604	-1.18424996	-0.2549507
1445161_at	USP6 N-terminal like	Usp6nl	0.57608	-1.17595593	-0.2354905
1425157_x_at	tetraspanin 33	Tspan33	0.57617	1.19054	0.25038668
1453312_at	DDB1 and CUL4 associated factor 6	Dcaf6	0.57624	-1.20609539	-0.2881526
1433569_x_at	RAN, member RAS oncogene family	Ran	0.57624	-1.06625499	-0.0926983
1419206_at	CD37 antigen	Cd37	0.57625	1.16764755	0.21842299
1423911_at	protein phosphatase 2, regulatory subunit B (B56), alpha isoform	Ppp2r5a	0.57628	-1.13680105	-0.1882368
1427041_at	cDNA sequence BC013712	BC013712	0.57629	1.18697229	0.24539132
1417839_at	claudin 5	Cldn5	0.57632	1.17676944	0.23260697
1440251_s_at	zinc finger protein 64-like /// zinc finger protein 64	LOC100502777 /// Zfp64	0.57638	1.18976757	0.25001549
1429947_a_at	Z-DNA binding protein 1	Zbp1	0.57647	1.19034901	0.24879657
1454233_at	RIKEN cDNA 2310006M14 gene	2310006M14Rik	0.57661	-1.18086712	-0.2409859
1434285_at	FERM domain containing 4A	Frmd4a	0.57664	-1.16584465	-0.2260067
1452218_at	coiled-coil domain containing 117	Ccdc117	0.57673	1.16096393	0.2093093
1421278_s_at	spectrin alpha 1	Spna1	0.57673	-1.22463633	-0.3051218
1440408_at	RIKEN cDNA B830008J18 gene	B830008J18Rik	0.57689	1.20037703	0.26236089
1422652_at	crystallin, gamma A	Cryga	0.57693	1.18430889	0.24276918
1422686_s_at	exocyst complex component 4	Exoc4	0.57694	-1.14336115	-0.1937337
1439195_at	---	---	0.57698	1.17280731	0.22955529
1436982_at	trinucleotide repeat containing 6b	Tnrc6b	0.57699	-1.17105502	-0.2413139
1432619_at	RIKEN cDNA 3110037L02 gene	3110037L02Rik	0.57703	1.21543172	0.27428977
1421302_a_at	guanine nucleotide binding protein, alpha 15	Gna15	0.57716	1.16591989	0.21819082
1455682_at	v-abl Abelson murine leukemia viral oncogene homolog 2 (arg, A	Abl2	0.57717	-1.17674511	-0.2387364
1417410_s_at	protein kinase C, iota	Prkci	0.57723	1.11233485	0.15354566
1418181_at	protein tyrosine phosphatase 4a3	Ptp4a3	0.57724	1.15910571	0.20676666
1447846_x_at	membrane bound O-acyltransferase domain containing 7	Mboat7	0.57742	1.19308974	0.24808648
1448595_a_at	brain expressed gene 1	Bex1	0.57762	-1.07537785	-0.1063009
1418512_at	serine/threonine kinase 3 (Ste20, yeast homolog)	Stk3	0.57775	-1.14957127	-0.2015996
1454463_at	RIKEN cDNA 5430402O13 gene	5430402O13Rik	0.57786	-1.22212336	-0.3009332
1436704_x_at	methylenetetrahydrofolate dehydrogenase (NADP+ dependent),	Mthfd1	0.57789	-1.18149722	-0.2561697
1433811_at	myeloid/lymphoid or mixed-lineage leukemia (trithorax homolog,	Mllt6	0.57798	-1.14780921	-0.2003088
1418217_at	non-metastatic cells 7, protein expressed in (nucleoside-diphosph	Nme7	0.57799	1.12786046	0.17311735
1441554_at	chondroitin sulfate synthase 3	Chsy3	0.57813	-1.20701705	-0.2851094
1448752_at	carbonic anhydrase 2	Car2	0.57821	1.14500916	0.19278556
1457754_at	RIKEN cDNA 4930430F08 gene	4930430F08Rik	0.57822	-1.16392806	-0.2282529
1455570_x_at	calponin 3, acidic	Cnn3	0.57838	-1.11918572	-0.1635537
1437690_x_at	casein kinase 1, delta	Csnk1d	0.57841	1.13382675	0.18027163
1458721_at	---	---	0.57844	1.24450675	0.29908641
1433520_at	SREBF chaperone	Scap	0.57847	1.12387973	0.16801286
1460417_at	cDNA sequence AB041803	AB041803	0.57849	1.20226451	0.26280201
1435756_at	sterile alpha motif domain containing 10	Samd10	0.57853	1.1829131	0.23879931
1458560_at	asp (abnormal spindle)-like, microcephaly associated (Drosophila	Aspm	0.5786	-1.25383967	-0.358016
1424687_at	HEAT repeat containing 6	Heatr6	0.57862	-1.17371091	-0.2352589
1416551_at	ATPase, Ca++ transporting, cardiac muscle, slow twitch 2	Atp2a2	0.57862	1.14141673	0.19050352
1435263_at	transmembrane protein 106A	Tmem106a	0.57864	1.19013706	0.24605891
1431150_at	RIKEN cDNA 1700110M21 gene	1700110M21Rik	0.57865	-1.19167566	-0.2546835
1418499_a_at	potassium voltage-gated channel, Isk-related subfamily, gene 3	Kcne3	0.57867	-1.14480774	-0.1953314
1436604_at	tubulin tyrosine ligase-like family, member 3	Ttll3	0.57869	-1.22284197	-0.3112359
1441878_s_at	RIKEN cDNA 1810049H13 gene	1810049H13Rik	0.57869	1.1546873	0.20717303
1428489_at	zinc finger CCHC-type and RNA binding motif 1	Zcrb1	0.57876	1.12030916	0.16388966
1450840_a_at	60S ribosomal protein L39-like /// ribosomal protein L39	LOC100503815 /// Rpl39	0.57876	-1.06514432	-0.0922594
1432882_at	RIKEN cDNA 4932431P20 gene	4932431P20Rik	0.57892	-1.18428071	-0.2441912
1441643_at	membrane-associated ring finger (C3HC4) 3	Mar-03	0.57894	-1.18174211	-0.2414727
1417896_at	tight junction protein 3	Tjp3	0.57899	1.19990591	0.25469382
1436811_at	potassium channel tetramerisation domain containing 3	Kctd3	0.57907	1.14690735	0.19599736
1455205_a_at	ubiquitin specific peptidase 19	Usp19	0.57916	1.14978506	0.19928973
1424470_a_at	Rap guanine nucleotide exchange factor (GEF) 3	Rapgef3	0.5794	-1.1940881	-0.2670089
1422554_at	necdin-like 2	Ndnl2	0.57946	1.13072717	0.17529751
1415738_at	thioredoxin domain containing 12 (endoplasmic reticulum)	Txndc12	0.57959	1.13600754	0.18251477
1459757_x_at	CCR4-NOT transcription complex, subunit 10	Cnot10	0.57969	-1.16120691	-0.2201466
1420293_at	lysophosphatidylcholine acyltransferase 1	Lpcat1	0.57972	-1.22374471	-0.3072986
1436590_at	protein phosphatase 1, regulatory (inhibitor) subunit 3B	Ppp1r3b	0.57979	1.17332411	0.23014918
1427401_at	cholinergic receptor, nicotinic, alpha polypeptide 5	Chrna5	0.57986	1.22163559	0.28044446

1422718_at	adaptor-related protein complex 3, sigma 2 subunit	Ap3s2	0.57987	1.12726584	0.17282344
1419932_s_at	---	---	0.57992	1.18106869	0.23771581
1436941_at	family with sequence similarity 55, member C	Fam55c	0.58001	-1.18279515	-0.2450628
1423797_at	acetoacetyl-CoA synthetase	Aacs	0.58003	1.14732719	0.19577229
1420032_at	Checkpoint kinase 1 homolog (S. pombe)	Chek1	0.58004	-1.20937299	-0.2834896
1428306_at	DNA-damage-inducible transcript 4	Ddit4	0.58008	1.16243595	0.20776504
1431665_a_at	translocase of inner mitochondrial membrane 8 homolog b (yeast	Timm8b	0.5801	1.15293149	0.19814065
1434047_x_at	heterogeneous nuclear ribonucleoprotein A2/B1	Hnrnpa2b1	0.5801	-1.10755605	-0.1483788
1427267_at	trinucleotide repeat containing 18	Tnrc18	0.58012	1.27321493	0.3233937
1436194_at	PRELI domain containing 2	Prelid2	0.58015	1.15842864	0.20645185
1419624_a_at	RIKEN cDNA 1700010I14 gene	1700010I14Rik	0.58028	1.1715397	0.22567127
1452148_at	low density lipoprotein receptor-related protein associated protei	Lrpap1	0.58032	-1.17621285	-0.239831
1446157_at	---	---	0.58035	1.18554069	0.2405599
1455119_at	phosphatidic acid phosphatase type 2 domain containing 1A	Ppapdc1a	0.58053	1.19502782	0.25578946
1447967_at	transmembrane protein 69	Tmem69	0.58053	-1.15466496	-0.2085942
1423428_at	receptor tyrosine kinase-like orphan receptor 2	Ror2	0.58054	1.15866595	0.210219
1459693_x_at	tyrosine 3-monooxygenase/tryptophan 5-monooxygenase activa	Ywhag	0.58054	-1.17503268	-0.2329068
1444051_at	RIKEN cDNA 1700019D03 gene	1700019D03Rik	0.5806	-1.14001636	-0.1927975
1440379_at	solute carrier family 1 (neutral amino acid transporter), member 5	Slc1a5	0.58064	-1.19325859	-0.2601426
1457491_at	Pleckstrin homology domain containing, family A (phosphoinositi	Plekha1	0.58069	-1.20875186	-0.289759
1445373_at	---	---	0.58076	-1.17584912	-0.2355868
1435269_at	N-6 adenine-specific DNA methyltransferase 2 (putative)	N6amt2	0.58083	-1.1740524	-0.2353198
1459926_at	expressed sequence C77068	C77068	0.58123	-1.16676486	-0.2225815
1451434_s_at	G patch domain containing 8	Gpatch8	0.58144	-1.17249086	-0.2348257
1419858_at	---	---	0.58145	1.16708114	0.21865
1450300_at	gamma-aminobutyric acid (GABA) C receptor, subunit rho 1	Gabrr1	0.58157	-1.19381562	-0.2596408
1416183_a_at	lactate dehydrogenase B	Ldhb	0.58165	1.18782717	0.23333381
1436100_at	SH2 domain containing 5	Sh2d5	0.58191	1.17429181	0.22119111
1458876_at	---	---	0.58201	-1.19025025	-0.2586891
1440582_at	itchy, E3 ubiquitin protein ligase	Itch	0.58203	1.21379235	0.27341844
1422769_at	synaptotagmin binding, cytoplasmic RNA interacting protein	Syncrip	0.58208	-1.13566919	-0.1888617
1421692_at	calcium channel, voltage-dependent, R type, alpha 1E subunit	Cacna1e	0.58226	-1.2055281	-0.2769388
1417680_at	potassium voltage-gated channel, shaker-related subfamily, men	Kcna5	0.58226	1.17488118	0.22839728
1426583_at	activating transcription factor 2	Atf2	0.58234	-1.1318288	-0.1815065
1450047_at	heparan sulfate 6-O-sulfotransferase 2	Hs6st2	0.58307	1.18523828	0.24182108
1422887_a_at	C-terminal binding protein 2	Ctbp2	0.58323	-1.12526042	-0.1746436
1436052_at	RIKEN cDNA 1700020O03 gene	1700020O03Rik	0.58328	1.18788164	0.23988234
1456336_at	cysteine-serine-rich nuclear protein 3	Csrnp3	0.58331	1.19066676	0.2515996
1429388_at	Nanog homeobox	Nanog	0.58336	-1.10201245	-0.140543
1449670_x_at	G protein-coupled receptor 137B	Gpr137b	0.58348	-1.19607554	-0.2624786
1436844_at	---	---	0.58348	-1.22262173	-0.3153301
1429771_at	RIKEN cDNA 3110073H01 gene	3110073H01Rik	0.5835	-1.13950628	-0.1891745
1421992_a_at	insulin-like growth factor binding protein 4	Igfbp4	0.58362	1.17930824	0.23701692
1426948_at	translocated promoter region	Tpr	0.58365	1.12172974	0.16560598
1454613_at	dihydropyrimidinase-like 3	Dpysl3	0.58368	1.15845608	0.20897233
1448188_at	uncoupling protein 2 (mitochondrial, proton carrier)	Ucp2	0.5837	-1.12108628	-0.1655861
1422507_at	cystatin B	Cstb	0.58378	1.167987	0.21458526
AFFX-BioC-3_at	---	---	0.58379	1.16055267	0.21050042
1434405_at	folliculin interacting protein 1	Fnip1	0.58391	-1.21779835	-0.316046
1449405_at	tensin 1	Tns1	0.58391	1.22097252	0.27494561
1448521_at	bromodomain containing 7	Brd7	0.58395	1.12441203	0.16893322
1436583_at	defective in sister chromatid cohesion 1 homolog (S. cerevisiae)	Dscc1	0.58396	1.19339387	0.25391738
1460265_at	Proliferation-associated 2G4	Pa2g4	0.58406	-1.1964997	-0.2680378
1451385_at	family with sequence similarity 162, member A	Fam162a	0.58406	1.11472924	0.15612757
1442312_at	transducin (beta)-like 1X-linked receptor 1	Tbl1xr1	0.58421	-1.21253406	-0.2954437
1420196_s_at	TBC1 domain family, member 14	Tbc1d14	0.58422	1.14475611	0.19309784
1449110_at	ras homolog gene family, member B	Rhob	0.58437	1.13812965	0.18631759
1427388_at	leucine rich repeat containing 2	Lrrc2	0.58444	-1.15308092	-0.2056614
1452814_at	copine III	Cpne3	0.58445	-1.23509616	-0.3392346
1459728_at	ISY1 splicing factor homolog (S. cerevisiae)	Isy1	0.58445	-1.19843315	-0.2678281
1422565_s_at	nuclear factor I/C	Nfic	0.58451	1.30614682	0.35511523
1455481_at	iduronate 2-sulfatase	Ids	0.58454	-1.16956285	-0.233307
1438476_a_at	chromodomain helicase DNA binding protein 4	Chd4	0.58456	-1.17734394	-0.2395808
1437182_at	death inducer-obliterator 1	Dido1	0.58456	1.2080289	0.26246899
1436814_at	RIKEN cDNA 1810010D01 gene	1810010D01Rik	0.58466	1.23325431	0.28889769
1427420_at	NK6 homeobox 2	Nkx6-2	0.58467	1.22423226	0.28048811
1449542_at	pre B-cell leukemia transcription factor 1	Pbx1	0.58474	-1.17680862	-0.2406608
1426867_at	doublesex and mab-3 related transcription factor 2	Dmrt2	0.58475	1.22738425	0.28121014
1445085_at	---	---	0.58485	1.19301694	0.24963066
1458367_at	predicted gene 94	Gm94	0.58489	-1.16475833	-0.2202294
1425466_at	SUMO/sentrin specific peptidase 2	Senp2	0.58495	1.13405577	0.17906129
1434743_x_at	RUN and SH3 domain containing 1	Rusc1	0.58499	1.17186255	0.22759672
1448782_at	thioredoxin domain containing 11	Txndc11	0.58504	1.18046808	0.23456412
1432741_at	RIKEN cDNA 9530071P10 gene	9530071P10Rik	0.58514	1.18785617	0.23825656
1455227_at	arylacetamide deacetylase-like 1	Nceh1	0.58526	1.19063554	0.24484038
1442334_at	predicted gene 13040 /// predicted gene 13043 /// predicted ger	Gm13040 /// Gm13043 ///	0.58529	1.20614761	0.26309991
1416410_at	platelet-activating factor acetylhydrolase, isoform 1b, subunit 3	Pafah1b3	0.58534	1.14722479	0.19320666
1440732_at	nuclear receptor coactivator 2 pseudogene	D030018L15Rik	0.58536	-1.15790899	-0.2119717
1427413_a_at	CUGBP, Elav-like family member 1	Celf1	0.58536	-1.13721938	-0.1906043
1428402_at	zinc finger, CCHC domain containing 3	Zcchc3	0.58546	-1.1905193	-0.2740086
1457357_at	tousled-like kinase 2 (Arabidopsis)	Tlk2	0.58547	-1.2106269	-0.2890877
1455142_at	suppressor of cytokine signaling 4	Socs4	0.58552	-1.13453815	-0.1836774
1417392_a_at	solute carrier family 7 (cationic amino acid transporter, y+ system	Slc7a7	0.58552	-1.13558821	-0.1880489
1417587_at	timeless homolog (Drosophila)	Timeless	0.58552	-1.15484709	-0.2156107
1449678_at	Transportin 3	Tnpo3	0.58561	-1.16161664	-0.216761
1447853_x_at	Kinesin family member 13A	Kif13a	0.58563	-1.20326467	-0.2701831
1435240_at	bromodomain adjacent to zinc finger domain, 2B	Baz2b	0.58563	1.17963881	0.23180608
1425536_at	syntaxin 3	Stx3	0.58564	-1.14694722	-0.203559
1436419_a_at	RIKEN cDNA 1700097N02 gene	1700097N02Rik	0.58567	1.20955084	0.25514445
1448330_at	glutathione S-transferase, mu 1	Gstm1	0.58574	1.14058954	0.18904013
1434236_at	zinc finger, DHHC domain containing 20	Zdhhc20	0.58575	-1.1686876	-0.2314033
1424698_s_at	grancalcin	Gca	0.58581	-1.19569145	-0.2610551
1420152_at	---	---	0.58582	-1.16215461	-0.2172011
1438384_at	SPANX family, member N4	Spanxn4	0.58583	1.2022125	0.25638381

1437829_s_at	eukaryotic elongation factor-2 kinase	Eef2k	0.58583	1.14829936	0.19861053
1415698_at	golgi membrane protein 1	Golm1	0.5859	1.1332244	0.17730728
1422365_at	olfactory receptor 17	Olfr17	0.58592	-1.19877462	-0.2688325
1426652_at	minichromosome maintenance deficient 3 (S. cerevisiae)	Mcm3	0.58597	1.11849963	0.16003107
1425495_at	zinc finger protein 62	Zfp62	0.58597	-1.26818341	-0.3991043
1427172_at	oral-facial-digital syndrome 1 gene homolog (human)	Ofd1	0.58599	-1.14684152	-0.1978605
1421569_at	glutamate receptor, ionotropic, delta 1	Grid1	0.58601	1.18205843	0.23930063
1447868_x_at	glutaredoxin 3	Glrx3	0.58603	-1.2334834	-0.3263809
1423922_s_at	integrator complex subunit 3	Ints3	0.58607	1.13407998	0.18089016
1432326_at	RIKEN cDNA 1700042B14 gene	1700042B14Rik	0.58614	1.1757629	0.23046535
1456223_at	---	---	0.58615	1.2490794	0.30508416
1422146_at	sema domain, seven thrombospondin repeats (type 1 and type 1	Sema5b	0.58616	1.18489355	0.23686461
1454933_at	family with sequence similarity 176, member B	Fam176b	0.58623	1.19900031	0.24874595
1451969_s_at	poly (ADP-ribose) polymerase family, member 3	Parp3	0.58629	1.27918426	0.33149163
1422908_at	ATPase, (Na+)/K+ transporting, beta 4 polypeptide	Atp1b4	0.58629	-1.16198338	-0.2231523
1428139_at	transmembrane protein 180	Tmem180	0.58635	1.15870456	0.21081834
1453634_a_at	endoplasmic reticulum protein 29	Erp29	0.58638	1.13442917	0.18013393
1456407_a_at	tousled-like kinase 1	Tlk1	0.5864	-1.20306178	-0.2862691
1424257_at	cyclin-dependent kinase 7	Cdk7	0.58641	1.17665431	0.23289786
1424208_at	prostaglandin E receptor 4 (subtype EP4)	Ptger4	0.58645	-1.20482325	-0.2784354
1448398_s_at	ribosomal protein L22	Rpl22	0.58648	1.1199785	0.16324801
1454508_at	RIKEN cDNA 4930485E13 gene	4930485E13Rik	0.58649	1.15449086	0.20546406
1457779_at	RIKEN cDNA 1110046J04 gene	1110046J04Rik	0.58652	1.1850017	0.24151138
1452233_at	ATP-binding cassette, sub-family C (CFTR/MRP), member 1	Abcc1	0.58654	1.14410947	0.19327029
1452584_at	RIKEN cDNA 1500032L24 gene	1500032L24Rik	0.58658	1.1412313	0.18647265
1443088_at	RIKEN cDNA 9930031P18 gene	9930031P18Rik	0.58661	1.20474505	0.25967251
1456103_at	promyelocytic leukemia	Pml	0.58667	1.13122443	0.17757087
1447838_x_at	echinoderm microtubule associated protein like 4	Eml4	0.58669	-1.17804502	-0.2371212
1454417_at	RIKEN cDNA 4933408M05 gene	4933408M05Rik	0.58669	1.17757649	0.23466279
1449746_s_at	GLI pathogenesis-related 1 (glioma)	Glipr1	0.58675	-1.17880586	-0.2466722
1438113_at	zinc finger, matrin type 4	Zmat4	0.58675	-1.19194121	-0.2552788
1421392_a_at	baculoviral IAP repeat-containing 3	Birc3	0.58675	1.16374726	0.21827156
1443880_at	zinc finger and BTB domain containing 39	Zbtb39	0.58678	1.14755701	0.19831055
1451307_at	mitochondrial ribosomal protein L14	Mrpl14	0.5868	1.17398846	0.22243307
1425111_at	sortilin-related VPS10 domain containing receptor 3	Sorcs3	0.58681	1.20107198	0.25858734
1420979_at	p21 protein (Cdc42/Rac)-activated kinase 1	Pak1	0.58682	1.1715722	0.22493854
1417227_at	methylcrotonoyl-Coenzyme A carboxylase 1 (alpha)	Mccc1	0.58684	1.14431508	0.19289036
1451639_at	CCAAT/enhancer binding protein (C/EBP), gamma	Cebpg	0.58684	-1.16439023	-0.2210627
1438197_at	vezatin, adherens junctions transmembrane protein	Vezt	0.58684	1.16909484	0.22322088
1428768_at	PAN3 polyA specific ribonuclease subunit homolog (S. cerevisiae)	Pan3	0.58689	-1.13924076	-0.1899224
1444102_at	hypothetical LOC100503664	LOC100503664	0.5869	-1.16537843	-0.2212345
1421815_at	ependymin related protein 1 (zebrafish)	Epdr1	0.58691	1.15278282	0.20444765
1453244_at	RIKEN cDNA 5830416P10 gene	5830416P10Rik	0.58693	-1.17194703	-0.2297554
1450839_at	DNA segment, human D4S114	D0H4S114	0.58693	1.17238416	0.22749388
1418311_at	fructosamine 3 kinase	Fn3k	0.58695	1.1880984	0.2485749
1437286_x_at	RIKEN cDNA 1110020G09 gene	1110020G09Rik	0.58697	-1.19231052	-0.2613554
1434810_a_at	cancer antigen 1	Cage1	0.58701	1.15771303	0.20842436
1451630_at	tubulin tyrosine ligase	Ttl	0.58702	1.16685278	0.2215143
1429926_at	apolipoprotein O-like	Apool	0.58703	-1.17682379	-0.237006
1429984_at	RIKEN cDNA 5730455O13 gene	5730455O13Rik	0.58704	-1.18513273	-0.2590264
1429760_at	ribosomal protein S6 kinase polypeptide 6	Rps6ka6	0.58704	-1.16435211	-0.2199675
1420880_a_at	tyrosine 3-monooxygenase/tryptophan 5-monooxygenase activa	Ywhab	0.58705	-1.10511991	-0.1450856
1436423_at	thymocyte selection associated	Themis	0.58709	1.20592122	0.26276113
1435108_at	Rho GTPase activating protein 22	Arhgap22	0.58711	1.19740033	0.25184048
1450815_s_at	coiled-coil-helix-coiled-coil-helix domain containing 2 /// SCAN do	Chchd2 /// Scand3	0.58715	1.09601635	0.13176333
1431426_at	regulation of nuclear pre-mRNA domain containing 2	Rprd2	0.58726	-1.22973979	-0.3210905
1422632_at	cathepsin W	Ctsw	0.58732	1.15903393	0.21101166
1455661_at	predicted gene 9958	Gm9958	0.58735	-1.18792246	-0.2500152
1438692_at	general transcription factor IIIC, polypeptide 4	Gtf3c4	0.58736	1.17303102	0.22457562
1458639_at	---	---	0.58739	-1.18863087	-0.2538999
1437497_a_at	heat shock protein 90, alpha (cytosolic), class A member 1	Hsp90aa1	0.5875	-1.07078809	-0.0989401
1419314_at	tubulointerstitial nephritis antigen	Tinag	0.5875	1.16803063	0.22384381
1443388_at	---	---	0.58752	-1.21765901	-0.3010721
1454885_at	zinc finger protein 141	Zfp141	0.58754	-1.18147844	-0.2566513
1418054_at	neurogenic differentiation 4	Neurod4	0.58769	1.14960143	0.1986415
1423786_at	RIKEN cDNA 8430410A17 gene	8430410A17Rik	0.58779	1.11194922	0.1528575
1417596_at	B9 protein domain 1	B9d1	0.58781	1.14362489	0.19019678
1420042_at	THO complex 1	Thoc1	0.58781	-1.27869776	-0.4164037
1428872_at	male-specific lethal 1 homolog (Drosophila)	Msl1	0.58787	1.23857479	0.28466894
1457361_at	zinc finger protein 804A	Zfp804a	0.588	-1.17561601	-0.2449835
1458248_at	---	---	0.58805	-1.19340518	-0.2601584
1452659_at	DEK oncogene (DNA binding)	Dek	0.58815	-1.09475376	-0.1308509
1448147_at	tumor necrosis factor receptor superfamily, member 19	Tnfrsf19	0.58826	1.20729075	0.26407774
1449842_at	transmembrane protein 179B	Tmem179b	0.58827	1.18163689	0.23115051
1448016_at	spindle assembly 6 homolog (C. elegans)	Sass6	0.58831	1.15118414	0.20272132
1421654_a_at	lamin A	Lmna	0.5884	1.14148731	0.18742098
1457239_at	Spleen tyrosine kinase	Sykb	0.5884	-1.20681715	-0.2778228
1455918_at	adrenergic receptor, beta 3	Adrb3	0.5884	1.16583267	0.2182823
1453810_at	aftiphilin	Aftph	0.58843	-1.18684571	-0.2519156
1445395_at	---	---	0.58846	-1.18009124	-0.2396929
1434847_at	cyclin M4	Cnnm4	0.58847	1.24675148	0.29834651
1453467_s_at	ribosomal protein S15A	Rps15a	0.58855	-1.06990221	-0.0986319
1442543_at	---	---	0.58859	-1.17633497	-0.2378524
1458947_at	---	---	0.58861	-1.24630727	-0.3497299
1429906_at	RIKEN cDNA A930035E12 gene	A930035E12Rik	0.58861	1.2225198	0.27720636
1417886_at	RIKEN cDNA 1810009A15 gene	1810009A15Rik	0.58862	1.1233224	0.16570402
1449688_at	DNA segment, Chr 10, ERATO Doi 610, expressed	D10Ertd610e	0.58866	-1.17400483	-0.2347558
1426434_at	transmembrane protein 43	Tmem43	0.58867	1.1518588	0.20358298
1437474_at	GATA zinc finger domain containing 2B	Gatad2b	0.58867	-1.1917327	-0.2735236
1454336_at	cyclin C	Ccnc	0.58867	-1.17596377	-0.2345839
1425264_s_at	myelin basic protein	Mbp	0.58869	1.24935423	0.30185134
1450965_at	testis expressed gene 261	Tex261	0.58869	1.14903237	0.1978686
1454070_a_at	DDHD domain containing 1	Ddhd1	0.58873	1.16191787	0.21581216
1460231_at	interferon regulatory factor 5	Irf5	0.58882	-1.18047415	-0.2469974

1426697_a_at	low density lipoprotein receptor-related protein associated protei	Lrpap1	0.58888	-1.16750705	-0.2332628
1441562_at	RIKEN cDNA A230048O21 gene	A230048O21Rik	0.58889	-1.16421797	-0.2198386
1453365_at	RAB GTPase activating protein 1-like	Rabgap1l	0.58889	1.18825742	0.24371338
1417801_a_at	PTPRF interacting protein, binding protein 2 (liprin beta 2)	Ppfibp2	0.5889	1.19005411	0.24105644
1448928_at	histone deacetylase 6	Hdac6	0.5889	1.12147119	0.16494499
1453133_at	solute carrier family 25 (mitochondrial carrier; adenine nucleotide	Slc25a31	0.58891	-1.19435887	-0.2625501
1448766_at	gap junction protein, beta 1	Gjb1	0.58892	1.24389661	0.29772515
1456256_at	eukaryotic translation initiation factor 5	Eif5	0.58892	-1.17664613	-0.2433521
1441201_at	---	---	0.58893	1.19163068	0.23918931
1456535_at	vacuolar protein sorting 13C (yeast)	Vps13c	0.58893	-1.18334244	-0.2467841
1417326_a_at	anaphase promoting complex subunit 11	Anapc11	0.58894	1.13008611	0.17347309
1416707_a_at	polyamine-modulated factor 1	Pmf1	0.58896	1.14877517	0.19601178
1434229_a_at	polymerase (DNA directed), beta	Polb	0.58896	-1.1533693	-0.2087867
1417377_at	cell adhesion molecule 1	Cadm1	0.58897	-1.24085224	-0.3595188
1441191_at	RIKEN cDNA 2810454H06 gene	2810454H06Rik	0.589	1.20884947	0.26482184
1450354_a_at	phosphatidylserine synthase 2	Ptdss2	0.58901	1.18468643	0.23351504
1447272_s_at	ATPase, class V, type 10A	Atp10a	0.58902	1.33838664	0.3764864
1417952_at	cytochrome P450, family 2, subfamily j, polypeptide 6	Cyp2j6	0.58903	-1.17311502	-0.2307467
1455774_at	predicted gene 11175	Gm11175	0.58904	1.18297427	0.23754805
1453871_at	solute carrier family 6 (neurotransmitter transporter, GABA), mem	Slc6a11	0.58905	1.19709788	0.24873717
1426242_at	polymerase (RNA) II (DNA directed) polypeptide A	Polr2a	0.58909	1.16423872	0.20995027
1441872_x_at	---	---	0.58914	-1.16490451	-0.2227868
1429527_a_at	phospholipid scramblase 1	Plscr1	0.58914	-1.18298371	-0.2587792
1416681_at	ubiquitin protein ligase E3A	Ube3a	0.58915	-1.19507348	-0.2815551
1450618_a_at	small proline-rich protein 2A1 /// small proline-rich protein 2A2	Sprr2a1 /// Sprr2a2	0.58915	-1.16479134	-0.220852
1415773_at	nucleolin	Ncl	0.58915	-1.10985722	-0.1536389
1454852_at	trans-acting transcription factor 1	Sp1	0.58916	-1.12655687	-0.1748573
1428712_at	MON1 homolog b (yeast)	Mon1b	0.58917	-1.18006597	-0.2406033
1434504_at	zinc finger, FYVE domain containing 28	Zfyve28	0.58919	1.20431366	0.26189544
1424641_a_at	THO complex 1	Thoc1	0.5892	-1.18651819	-0.2702649
1459840_s_at	coiled coil domain containing 28B	Ccdc28b	0.5892	1.17290801	0.22810506
1440497_at	FtsJ methyltransferase domain containing 2	Ftsjd2	0.58922	1.18186218	0.23793531
1439629_at	neurexin-3-alpha-like /// neurexin III	LOC100505240 /// Nrxn3	0.58926	-1.13889369	-0.1895396
1429112_at	talin 2	Tln2	0.58928	-1.1969041	-0.2691474
1420809_a_at	RIKEN cDNA 1500003O03 gene	1500003O03Rik	0.5893	-1.14582272	-0.1998646
1448560_at	BH3 interacting domain death agonist	Bid	0.58933	1.13409203	0.18085898
1434899_s_at	trinucleotide repeat containing 6a	Tnrc6a	0.5894	-1.14606456	-0.2000511
1422640_at	protocadherin beta 9	Pcdhb9	0.58941	1.1333519	0.17985618
1449731_s_at	nuclear factor of kappa light polypeptide gene enhancer in B-cells	Nfkbia	0.58942	-1.15158676	-0.2140174
1423567_a_at	proteasome (prosome, macropain) subunit, alpha type 7	Psma7	0.58942	-1.11415121	-0.1564238
1457366_at	cell division cycle 40 homolog (yeast)	Cdc40	0.58945	1.20341413	0.26126832
1455504_a_at	makorin, ring finger protein, 1	Mkrn1	0.58947	-1.09009136	-0.1245781
1436834_x_at	malate dehydrogenase 1, NAD (soluble)	Mdh1	0.5895	-1.1980832	-0.2919672
1424471_at	Rap guanine nucleotide exchange factor (GEF) 3	Rapgef3	0.58951	-1.15079493	-0.205351
1427461_at	cDNA sequence BC005561	BC005561	0.58952	-1.22368215	-0.3147215
1435237_at	RIKEN cDNA 2310009A05 gene	2310009A05Rik	0.58952	1.15410646	0.20278938
1416408_at	acyl-Coenzyme A oxidase 1, palmitoyl	Acox1	0.58954	1.15660627	0.20826203
1419375_at	WW domain binding protein 4	Wbp4	0.58955	1.1377404	0.18396498
1421031_a_at	RIKEN cDNA 2310016C08 gene	2310016C08Rik	0.58956	1.15020049	0.2011433
1420973_at	AT rich interactive domain 5B (MRF1-like)	Arid5b	0.58958	-1.16722629	-0.2270329
1429532_at	microrchidia 2A	Morc2a	0.58961	1.12057924	0.16403967
1438539_at	---	---	0.58962	1.18860594	0.24612002
1444747_at	---	---	0.58966	1.17186724	0.22748855
1440959_s_at	myoneurin	Mynn	0.58969	-1.18387196	-0.2480717
1453163_at	protein phosphatase 1, regulatory (inhibitor) subunit 12A	Ppp1r12a	0.58969	-1.15508975	-0.214646
1445123_at	Predicted gene 11517	Gm11517	0.58982	-1.18321999	-0.2517915
1416755_at	DnaJ (Hsp40) homolog, subfamily B, member 1	Dnajb1	0.58983	1.11776034	0.16024978
1457256_x_at	patched homolog 2	Ptch2	0.58988	-1.13246928	-0.1824497
1416945_at	prostate tumor over expressed gene 1	Ptov1	0.59007	1.14642535	0.19183255
1448180_a_at	hematological and neurological expressed sequence 1	Hn1	0.59008	1.12239399	0.16611474
1450517_at	T-cell acute lymphocytic leukemia 2	Tal2	0.59031	1.22448154	0.28027595
1451355_at	alkaline ceramidase 2	Acer2	0.59038	1.25090797	0.30261688
1436004_at	ubiquitin specific peptidase 27, X chromosome	Usp27x	0.59124	1.18277259	0.24037202
1452344_at	synaptojanin 2	Synj2	0.59126	1.20152173	0.25424945
1428946_at	ubiquitin-like modifier activating enzyme 6	Uba6	0.59127	-1.14162606	-0.1938314
1420811_a_at	catenin (cadherin associated protein), beta 1	Ctnnb1	0.5913	-1.09558413	-0.1340914
1418671_at	calpain 5	Capn5	0.59145	1.15046325	0.20214594
1437127_at	RIKEN cDNA A630033E08 gene	A630033E08Rik	0.59146	-1.22769438	-0.3274076
1435829_at	zinc finger, matrin type 1	Zmat1	0.59147	1.22252218	0.27943745
1438847_at	Max dimerization protein 3	Mxd3	0.59151	-1.15826619	-0.2154563
1433450_at	cyclin-dependent kinase 5, regulatory subunit 1 (p35)	Cdk5r1	0.59155	1.23156838	0.28674959
1427114_at	tetratricopeptide repeat domain 19	Ttc19	0.59162	1.15103842	0.20179827
1446614_at	Diacylglycerol kinase zeta	Dgkz	0.5917	-1.1954043	-0.2663204
1438978_x_at	necdin	Ndn	0.5917	1.20451431	0.25892599
1434199_at	transmembrane protein 151B	Tmem151b	0.59175	1.15215394	0.20305706
1439087_a_at	phosphoinositide-3-kinase interacting protein 1	Pik3ip1	0.59178	1.19613007	0.25300753
1417058_a_at	keratinocyte associated protein 2	Krtcap2	0.59185	1.21263659	0.25828017
1438953_at	c-fos induced growth factor	Figf	0.59188	1.19728277	0.25593616
1416371_at	apolipoprotein D	Apod	0.59191	1.18381274	0.23423106
1459902_at	RIKEN cDNA 2700007P21 gene	2700007P21Rik	0.59192	-1.15043332	-0.2067609
1428056_at	killer cell lectin-like receptor, subfamily A, member 1	Klra1	0.59197	1.17618516	0.2335975
1436822_x_at	predicted pseudogene 10166 /// predicted gene 10268 /// predic	Gm10166 /// Gm10268 //	0.59198	-1.0834724	-0.1189873
1443617_at	---	---	0.592	1.18377737	0.24026149
1431896_at	RIKEN cDNA 4930447C04 gene	4930447C04Rik	0.592	-1.19172115	-0.2595732
1434052_at	expressed sequence AI593442	AI593442	0.59204	1.16305402	0.21636474
1416944_a_at	tousled-like kinase 2 (Arabidopsis)	Tlk2	0.59205	-1.12286109	-0.1675633
1458035_at	---	---	0.59205	1.17136722	0.22110194
1423437_at	glutathione S-transferase, alpha 3	Gsta3	0.59207	1.17122866	0.22785334
1451214_at	kelch repeat and BTB (POZ) domain containing 2	Kbtbd2	0.59214	1.13384694	0.18109859
1452001_at	nuclear factor, erythroid derived 2	Nfe2	0.59215	1.1860585	0.24385151
1425573_a_at	ArfGAP with SH# domain, ankyrin repeat and PH domain1	Asap1	0.59227	1.18743699	0.24486936
1420568_at	stimulated by retinoic acid gene 8	Stra8	0.59232	1.1300496	0.17481775
1449875_s_at	histocompatibility 2, T region locus 10 /// histocompatibility 2, T re	H2-T10 /// H2-T22 /// H2-	0.59238	1.22348381	0.28222872
1452492_a_at	solute carrier family 37 (glycerol-3-phosphate transporter), memb	Slc37a2	0.59239	-1.19159861	-0.2575557

1459749_s_at	FAT tumor suppressor homolog 4 (Drosophila)	Fat4	0.59239	-1.13501081	-0.1841687
1455103_at	DEAD (Asp-Glu-Ala-Asp) box polypeptide 46	Ddx46	0.59241	1.12856759	0.17186023
1444063_at	RIKEN cDNA 5430435G22 gene	5430435G22Rik	0.59241	-1.18822347	-0.2497092
1431893_a_at	prenyl (solanesyl) diphosphate synthase, subunit 1	Pdss1	0.59247	1.14488679	0.19308401
1452365_at	chondroitin sulfate N-acetylgalactosaminyltransferase 1	Csgalnact1	0.59253	1.1444318	0.1885098
1417492_at	cathepsin B	Ctsb	0.59254	1.22016143	0.27579645
1422851_at	high mobility group AT-hook 2	Hmga2	0.59254	-1.17587965	-0.2402049
1439168_at	calcium/calmodulin-dependent protein kinase II, delta	Camk2d	0.59262	-1.21632165	-0.3057443
1431017_at	regulator of chromosome condensation (RCC1) and BTB (POZ) dor	Rcbtb2	0.59273	1.18741925	0.24248771
1449561_at	NFKB activating protein-like	Nkapl	0.59276	1.20811846	0.26648328
1417057_a_at	lysosomal-associated membrane protein 3 /// peptidylprolyl ison	Lamp3 /// Ppid	0.59278	-1.10992986	-0.1529021
1434778_at	wings apart-like homolog (Drosophila)	Wapal	0.59282	-1.12127029	-0.1660065
1452198_at	lysine (K)-specific demethylase 2B	Kdm2b	0.59288	-1.13390077	-0.1863521
1447115_at	Solute carrier family 26, member 10	Slc26a10	0.59289	1.17622949	0.23408037
1429410_at	enhancer of yellow 2 homolog (Drosophila)	Eny2	0.59291	1.14986981	0.20060465
1422212_at	forkhead box H1	Foxh1	0.59291	1.17493499	0.22386395
1457472_at	GRB10 interacting GYF protein 2	Gigyf2	0.59297	1.18003024	0.23470472
1448979_at	muted	Muted	0.59297	1.15775726	0.20882721
1417772_at	glyoxylate reductase/hydroxypyruvate reductase	Grhpr	0.59307	1.14800264	0.19311147
1456413_at	phosphodiesterase 4D interacting protein (myomegalin)	Pde4dip	0.59309	1.18297401	0.23970802
1447067_at	---	---	0.59309	-1.17056117	-0.2302619
1450224_at	collagen, type IV, alpha 3	Col4a3	0.59312	-1.20343209	-0.2752439
1428766_at	RNA methyltransferase like 1	Rnmtl1	0.59313	1.13076724	0.17611656
1421105_at	jagged 1	Jag1	0.59315	1.1754654	0.22822843
1430872_at	RIKEN cDNA 4930412O13 gene	4930412O13Rik	0.59318	-1.16292184	-0.2189913
1418386_at	N-6 adenine-specific DNA methyltransferase 2 (putative)	N6amt2	0.5932	1.14624209	0.19132373
1435469_at	quiescin Q6 sulfhydryl oxidase 2	Qsox2	0.59327	1.14752516	0.19750614
1440300_at	vav 1 oncogene	Vav1	0.59327	-1.21680535	-0.3021882
1424402_at	RUN and FYVE domain containing 3	Rufy3	0.59331	-1.21291453	-0.2935747
1458365_at	golgin, RAB6-interacting	Gorab	0.59331	-1.16071027	-0.2187755
1422621_at	RAN binding protein 2	Ranbp2	0.59351	-1.13723967	-0.1888977
1459043_at	---	---	0.59404	1.35107727	0.38803055
1440144_x_at	hypothetical protein C330046E03	C330046E03	0.59455	1.21265694	0.26801564
1453589_a_at	cadherin 11 pseudogene /// cadherin 11 pseudogene	2610005L07Rik /// 68204	0.59458	-1.15443624	-0.2104803
1415908_at	testis-specific protein, Y-encoded-like 1	Tspyl1	0.5946	-1.12776545	-0.1738919
1420823_at	sema domain, immunoglobulin domain (Ig), transmembrane dom	Sema4d	0.59465	1.16071716	0.21463727
1431997_at	glyceraldehyde-3-phosphate dehydrogenase pseudogene	3000002C10Rik	0.59465	-1.11594579	-0.1596036
1419938_s_at	Rho guanine nucleotide exchange factor (GEF) 17	Arhgef17	0.59467	1.17694878	0.23201593
1428748_at	zinc finger protein 826	Zfp826	0.5947	-1.19084783	-0.2593049
1420782_at	tumor necrosis factor receptor superfamily, member 17	Tnfrsf17	0.59472	-1.18175091	-0.2458686
1445494_at	---	---	0.59479	-1.2352326	-0.3377994
1420091_s_at	microrchidia 3	Morc3	0.59493	-1.13327183	-0.1850508
1456792_at	TBC1 domain family, member 2	Tbc1d2	0.59496	1.16862138	0.22157987
1460054_at	trafficking protein particle complex 6A	Trappc6a	0.59497	1.17464271	0.22938134
1445239_at	GATA zinc finger domain containing 2A	Gatad2a	0.59508	1.1819392	0.23457466
1422175_at	matrix metallopeptidase 1a (interstitial collagenase)	Mmp1a	0.59509	1.17893614	0.23354672
1457255_x_at	kinesin family member 5C	Kif5c	0.59548	1.14093379	0.18560186
1436181_at	ArfGAP with SH3 domain, ankyrin repeat and PH domain 2	Asap2	0.59561	1.1673213	0.21466949
1420613_at	protein tyrosine phosphatase 4a2	Ptp4a2	0.59591	-1.13522107	-0.1889791
1431894_at	inositol 1,4,5-triphosphate receptor interacting protein	Itprip	0.59599	1.19354089	0.25159679
1439901_at	---	---	0.59613	-1.18459715	-0.2469064
1438657_x_at	protein tyrosine phosphatase 4a1-like /// protein tyrosine phosph	Gm13363 /// Ptp4a1	0.59615	-1.09631523	-0.1328438
1419951_at	Lectin, mannose-binding, 1	Lman1	0.59615	-1.21491849	-0.3004536
1428806_at	casein kinase 1, gamma 1	Csnk1g1	0.59623	-1.1522657	-0.2058065
1417491_at	cathepsin B	Ctsb	0.59626	-1.22612563	-0.3173945
1435145_at	cell adhesion molecule 2	Cadm2	0.59638	1.13846081	0.18480473
1419560_at	lipase, hepatic	Lipc	0.59641	1.20062145	0.25762862
1458469_at	Casitas B-lineage lymphoma b	Cblb	0.59651	1.2639665	0.31467926
1420114_s_at	RIKEN cDNA 2410022L05 gene	2410022L05Rik	0.59664	-1.18844026	-0.2531693
1428133_at	Smad nuclear interacting protein 1	Snip1	0.59675	1.12354033	0.16605938
1446421_at	---	---	0.59742	-1.21236803	-0.2974791
1455492_at	RIKEN cDNA B330016D10 gene	B330016D10Rik	0.59768	-1.178916	-0.2384333
1444390_at	PR domain containing 14	Prdm14	0.59769	-1.1463705	-0.2006106
1456037_x_at	prolactin regulatory element binding	Preb	0.5977	1.13823448	0.18203545
1423413_at	N-myc downstream regulated gene 1	Ndrg1	0.59771	1.14434675	0.19244367
1426814_at	SEC16 homolog A (S. cerevisiae)	Sec16a	0.5978	1.1398016	0.18721828
1424608_a_at	basic leucine zipper and W2 domains 2 /// predicted gene 4354 /.	Bzw2 /// Gm4354 /// Gm	0.59788	1.16442897	0.21468335
1452992_at	cell division cycle 26	Cdc26	0.59792	1.19544053	0.24553009
1424203_at	nicalin homolog (zebrafish)	Ncln	0.59793	1.17557406	0.22161691
1426171_x_at	killer cell lectin-like receptor, subfamily A, member 7	Klra7	0.59793	1.15202017	0.19966035
1437953_at	glycerophosphocholine phosphodiesterase GDE1 homolog (S. cer	Gpcpd1	0.59863	1.21791315	0.27319971
1423249_at	natural killer tumor recognition sequence	Nktr	0.59879	-1.24814209	-0.3588281
1423533_a_at	ras homolog gene family, member T1	Rhot1	0.59898	1.13879542	0.18257639
1426027_a_at	Rho GTPase activating protein 10	Arhgap10	0.59905	1.15741242	0.21085007
1449228_at	SH3-domain GRB2-like 2	Sh3gl2	0.60025	-1.16838334	-0.230629
1456211_at	NLR family, pyrin domain containing 10	Nlrp10	0.60033	1.22860229	0.27492784
1419783_at	---	---	0.60143	-1.19086829	-0.2630693
1452453_a_at	calcium/calmodulin-dependent protein kinase II alpha	Camk2a	0.60154	-1.14824572	-0.2091542
1415839_a_at	nucleophosmin 1	Npm1	0.60157	-1.0631338	-0.0883275
1457542_at	nucleoporin 133	Nup133	0.60164	-1.1916793	-0.2631724
1424521_at	zinc finger, AN1 type domain 2B	Zfand2b	0.60189	1.19450864	0.24507184
1432390_at	RIKEN cDNA 4933400F21 gene	4933400F21Rik	0.6019	-1.18071625	-0.2424213
1423421_at	ankyrin repeat domain 49	Ankrd49	0.60206	-1.1521738	-0.2095395
1437191_at	zinc finger, B-box domain containing	Zbbx	0.60207	1.19807741	0.25585479
1455713_x_at	prohibitin 2	Phb2	0.60208	1.11059739	0.15081924
1448965_at	INO80 homolog (S. cerevisiae)	Ino80	0.60214	-1.16054208	-0.2212788
1434848_at	---	---	0.60218	1.19050127	0.24329313
1452669_at	family with sequence similarity 53, member C	Fam53c	0.60222	1.14839718	0.19629497
1417654_at	syndecan 4	Sdc4	0.60222	-1.17971308	-0.2479786
1451031_at	secreted frizzled-related protein 4	Sfrp4	0.60222	1.16049038	0.21424674
1448187_at	polymerase (DNA directed), delta 1, catalytic subunit	Pold1	0.60224	1.11674909	0.15894898
1433079_at	RIKEN cDNA 4933436P19 gene	4933436P19Rik	0.60225	-1.18055258	-0.2405666
1439244_a_at	trinucleotide repeat containing 6a	Tnrc6a	0.60227	-1.13022626	-0.1806025
1427927_at	HscB iron-sulfur cluster co-chaperone homolog (E. coli)	Hscb	0.60229	1.14612338	0.19418971

1455521_at	Kruppel-like factor 12	Klf12	0.6023	-1.17822393	-0.2422053
1416123_at	cyclin D2	Ccnd2	0.60232	-1.18985499	-0.2634256
1459549_at	---	---	0.60233	-1.16325474	-0.2200911
1434193_at	predicted gene 12942	Gm12942	0.60236	1.1573094	0.21075135
1435048_at	expressed sequence AI854703	AI854703	0.60241	1.26702024	0.3170937
1438007_at	family with sequence similarity 19, member A2	Fam19a2	0.60241	1.17012894	0.22613099
1459481_at	---	---	0.60241	-1.12656193	-0.1725246
1450062_a_at	melanoma antigen, family D, 1	Maged1	0.60247	1.11625088	0.1583766
1419399_at	microsomal triglyceride transfer protein	Mttp	0.60248	1.2300462	0.28038248
1438297_at	EST AA545190	AA545190	0.6025	-1.15833213	-0.2131273
1416355_at	RNA binding motif protein, X chromosome	Rbmx	0.6025	-1.17399088	-0.2374164
1434800_at	synaptic vesicle glycoprotein 2 b	Sv2b	0.60254	1.19865961	0.25540616
1446621_at	---	---	0.6026	1.22593551	0.27957036
1452418_at	RIKEN cDNA 1200016E24 gene	1200016E24Rik	0.60265	-1.16300085	-0.2221883
1439166_at	nucleolar protein 11	Nol11	0.60266	1.23615962	0.28681847
1435557_at	formin homology 2 domain containing 1	Fhod1	0.60269	1.15999219	0.21372901
1453529_at	RIKEN cDNA 6330418B08 gene	6330418B08Rik	0.60275	-1.14349696	-0.1964497
1456573_x_at	nicotinamide nucleotide transhydrogenase	Nnt	0.60276	-1.13984796	-0.1910497
1416995_at	protein kinase C and casein kinase substrate in neurons 3	Pacsin3	0.60285	1.14692236	0.19631984
1433505_a_at	leucine rich repeat containing 8D	Lrrc8d	0.60285	-1.13526642	-0.1885498
1435368_a_at	poly (ADP-ribose) polymerase family, member 1	Parp1	0.6029	-1.15185686	-0.215256
1448108_at	serine incorporator 1	Serinc1	0.603	-1.13807338	-0.1867853
1442198_at	---	---	0.60302	1.19848673	0.2566026
1419774_at	DNA segment, Chr 2, ERATO Doi 239, expressed	D2Ertd239e	0.60317	-1.17738686	-0.2357996
1450619_x_at	---	---	0.60323	1.16301068	0.21686664
1417115_at	mitogen-activated protein kinase kinase kinase 12	Map3k12	0.60329	-1.1490334	-0.2006574
1455757_at	DNA segment, Chr 3, ERATO Doi 254, expressed	D3Ertd254e	0.60334	-1.16444146	-0.2270593
1429761_at	reticulon 1	Rtn1	0.60342	1.18778513	0.2386547
1424056_at	ubiquitin specific peptidase 48	Usp48	0.60342	-1.12103632	-0.1657813
1460704_at	RFNG O-fucosylpeptide 3-beta-N-acetylglucosaminyltransferase	Rfng	0.60348	1.1267383	0.17154214
1454769_at	TatD DNase domain containing 2	Tatdn2	0.6035	1.19088187	0.23763492
1445984_at	---	---	0.60353	-1.15820613	-0.2142888
1457414_at	hypothetical LOC100504349	LOC100504349	0.60356	-1.15602465	-0.2098554
1437873_at	zinc finger protein 799	Zfp799	0.60361	-1.1357646	-0.1840118
1434541_x_at	KH domain containing, RNA binding, signal transduction associate	Khdrbs1	0.60369	-1.11154566	-0.1526469
1428546_at	synaptotagmin binding, cytoplasmic RNA interacting protein	Syncrip	0.60389	-1.1217098	-0.1679104
1416335_at	macrophage migration inhibitory factor	Mif	0.60392	1.07295797	0.10040932
1419403_at	cDNA sequence BC017612	BC017612	0.60402	1.15321583	0.20302074
1433053_at	integrin beta 2-like	Itgb2l	0.60404	-1.21515699	-0.3046576
1415963_at	heterogeneous nuclear ribonucleoprotein H2	Hnrnph2	0.60406	-1.12265422	-0.1707922
1418530_at	nucleoporin 160	Nup160	0.60407	-1.12366785	-0.1694057
1427126_at	heat shock protein 1B	Hspa1b	0.60411	1.1775925	0.22752769
1424805_a_at	transmembrane protein 214	Tmem214	0.60422	1.14565987	0.19151593
1429703_at	RIKEN cDNA 2900072G11 gene	2900072G11Rik	0.60424	-1.17421157	-0.2357685
1452615_s_at	tRNA phosphotransferase 1	Trpt1	0.60427	1.17016606	0.22601113
1430586_at	RIKEN cDNA 2700007P21 gene	2700007P21Rik	0.60433	1.16981582	0.22452595
1434164_s_at	KH domain containing 1B	Khdc1b	0.60443	-1.17148848	-0.2412775
1444193_at	alcohol dehydrogenase, iron containing, 1	Adhfe1	0.60444	1.1997491	0.25800301
1443814_x_at	cathepsin H	Ctsh	0.60444	1.19967609	0.25613662
1456564_at	RIKEN cDNA C030046I01 gene	C030046I01Rik	0.60449	-1.18477398	-0.2541896
1424806_s_at	transmembrane protein 214	Tmem214	0.60485	1.13278609	0.1789456
1438816_at	AT hook containing transcription factor 1	Ahctf1	0.60502	1.27047374	0.31257524
1454114_a_at	Na+/H+ exchanger domain containing 1	Nhedc1	0.60503	-1.12744926	-0.1738363
1460530_at	RIKEN cDNA 4933416A02 gene	4933416A02Rik	0.60521	-1.17133287	-0.2283517
1429478_at	RIKEN cDNA 6720463M24 gene	6720463M24Rik	0.60538	1.16418367	0.21167504
1447083_at	---	---	0.60543	1.17849997	0.2342307
1458711_at	---	---	0.60551	-1.18448822	-0.248469
1427081_at	RIKEN cDNA A630072M18 gene	A630072M18Rik	0.60699	1.18735159	0.24030856
1431897_at	carbohydrate (N-acetylgalactosamine 4-0) sulfotransferase 9	Chst9	0.60699	1.15168591	0.2010761
1460644_at	branched chain ketoacid dehydrogenase kinase	Bckdk	0.60714	1.13216833	0.17887531
1452184_at	NADH dehydrogenase (ubiquinone) 1 beta subcomplex, 9	Ndufb9	0.60716	1.1240273	0.16585877
1459631_at	predicted gene 10063 /// ribosomal protein S12 /// ribosomal pro	Gm10063 /// Rps12 /// Rp	0.60716	-1.18554135	-0.2514504
1424167_a_at	phosphomannomutase 1	Pmm1	0.60719	1.17942375	0.22337753
1458951_at	vaccinia related kinase 1	Vrk1	0.6072	1.15377898	0.20455488
1448619_at	7-dehydrocholesterol reductase	Dhcr7	0.60736	1.12165536	0.16517931
1426361_at	zinc finger CCCH type containing 11A	Zc3h11a	0.60744	-1.15041778	-0.2099637
1442886_at	---	---	0.60751	-1.2242242	-0.3203982
1433818_at	1-acylglycerol-3-phosphate O-acyltransferase 3	Agpat3	0.60753	1.19387779	0.25008303
1452564_at	oncofetal antigen	Ofa	0.60757	1.19809623	0.25406996
1456822_at	RAD23b homolog (S. cerevisiae)	Rad23b	0.60771	-1.22538984	-0.3156963
1448146_at	WW domain containing E3 ubiquitin protein ligase 2	Wwp2	0.60771	1.16183425	0.21510293
1421156_a_at	desmocollin 2	Dsc2	0.60775	1.19353288	0.25007894
1419078_at	ninein	Nin	0.60782	1.1698643	0.22238074
1438131_at	F-box and WD-40 domain protein 2	Fbxw2	0.60786	-1.22365748	-0.3156592
1441943_x_at	---	---	0.60788	-1.12079675	-0.1650837
1452195_s_at	Sfi1 homolog, spindle assembly associated (yeast)	Sfi1	0.60789	1.17149726	0.22679203
1452736_at	R3H domain and coiled-coil containing 1	R3hcc1	0.60793	1.16724005	0.21404777
1423771_at	protein kinase C, delta binding protein	Prkcdbp	0.60793	1.18302613	0.23830378
1444078_at	CD8 antigen, alpha chain	Cd8a	0.60797	-1.18269008	-0.2454077
1418665_at	inositol (myo)-1(or 4)-monophosphatase 2	Impa2	0.60805	1.13568042	0.18084341
1436343_at	chromodomain helicase DNA binding protein 4	Chd4	0.60807	-1.16093574	-0.2156537
1415753_at	family with sequence similarity 108, member A	Fam108a	0.60812	1.12026586	0.16220687
1425214_at	pyrimidinergic receptor P2Y, G-protein coupled, 6	P2ry6	0.60813	1.20945109	0.26578272
1425148_a_at	sorting nexin 6	Snx6	0.60813	-1.12199749	-0.1662184
1442906_at	---	---	0.60815	1.17265687	0.22732375
1449151_at	cyclin-dependent kinase 18	Cdk18	0.60821	1.15147746	0.20234342
1419924_at	Folliculin interacting protein 1	Fnip1	0.60823	-1.26021154	-0.3773814
1431009_at	RIKEN cDNA B230219D22 gene	B230219D22Rik	0.60823	-1.17721698	-0.239826
1453486_a_at	signal peptide, CUB domain, EGF-like 2	Scube2	0.60831	1.18731494	0.2442819
1454060_a_at	neuroblastoma ras oncogene	Nras	0.60837	-1.1247772	-0.1730193
1433018_at	RIKEN cDNA 4930560O18 gene	4930560O18Rik	0.60843	1.17831085	0.23279874
1441294_at	---	---	0.60848	1.1304765	0.17661461
1442201_at	---	---	0.60848	-1.17428657	-0.2333833
1437099_x_at	heterogeneous nuclear ribonucleoprotein F /// hypothetical LOC1	Hnrnpf /// LOC100503154	0.60855	-1.17766429	-0.2590767

1435487_at	glutamate receptor, ionotropic, delta 2	Grid2	0.60856	-1.17139397	-0.2295101
1422404_x_at	predicted gene 12597 /// predicted gene 13280 /// interferon alp	Gm12597 /// Gm13280 //	0.60869	-1.21132702	-0.2942416
1450967_at	protein tyrosine phosphatase-like A domain containing 2	Ptplad2	0.60877	-1.17894826	-0.2400493
1420970_at	adenylate cyclase 7	Adcy7	0.60877	-1.17495679	-0.2327161
1428945_at	ubiquitin-like modifier activating enzyme 6	Uba6	0.60885	-1.12124087	-0.1657812
1425779_a_at	T-box 1	Tbx1	0.60899	1.17859042	0.23538532
1460538_at	cadherin 10	Cdh10	0.60909	-1.18612531	-0.250864
1416002_x_at	coactosin-like 1 (Dictyostelium)	Cotl1	0.60913	1.1597379	0.20932999
1426662_at	cytidine monophospho-N-acetylneuraminic acid synthetase	Cmas	0.60914	1.11918837	0.16154986
AFFX-r2-Ec-bioB-I	---	---	0.60957	1.14564042	0.19239734
1436326_at	RAR-related orphan receptor alpha	Rora	0.60975	1.20415156	0.26254577
1442284_at	squamous cell carcinoma antigen recognized by T-cells 1	Sart1	0.6098	-1.17964412	-0.2438599
1454753_at	arginyl aminopeptidase (aminopeptidase B)-like 1	Rnpepl1	0.6098	1.13536256	0.18178254
1434049_at	ectonucleoside triphosphate diphosphohydrolase 3	Entpd3	0.60993	-1.17765113	-0.2395791
1442721_at	---	---	0.61	-1.19597186	-0.267665
1450738_at	kinesin family member 21A	Kif21a	0.61003	1.21468038	0.26908331
1459788_at	G protein-coupled receptor 107	Gpr107	0.61006	-1.16424171	-0.2215531
1416713_at	tubulin polymerization-promoting protein family member 3	Tppp3	0.61008	1.17903858	0.22869613
1418965_at	nitric oxide synthase interacting protein	Nosip	0.61012	1.13720237	0.18084661
1433506_at	leucine rich repeat containing 8D	Lrrc8d	0.61013	-1.13624542	-0.1873561
1447468_at	---	---	0.61017	1.19449242	0.2503602
1450070_s_at	p21 protein (Cdc42/Rac)-activated kinase 1	Pak1	0.61017	1.13445731	0.18145728
1416810_at	male enhanced antigen 1	Mea1	0.61019	1.1457223	0.19052913
1453136_at	F-box protein 30	Fbxo30	0.6102	-1.15428911	-0.2141188
1415968_a_at	kidney androgen regulated protein	Kap	0.61021	-1.20182698	-0.2807255
1424514_at	ring finger protein 126	Rnf126	0.61022	1.13738741	0.18209418
1446377_at	fibulin 5	Fbln5	0.61067	-1.17555618	-0.2367811
1429040_at	cadherin 11 pseudogene	6820431F20Rik	0.61108	-1.15560151	-0.2179106
1419404_s_at	seven in absentia 1A /// seven in absentia 1B	Siah1a /// Siah1b	0.61119	-1.16096711	-0.2274267
1418604_at	arginine vasopressin receptor 1A	Avpr1a	0.61121	-1.17867416	-0.2412021
1436142_at	A kinase (PRKA) anchor protein 5	Akap5	0.61127	1.2312982	0.2825096
1453065_at	aldhehyde dehydrogenase family 5, subfamily A1	Aldh5a1	0.61136	1.16102108	0.21432266
1424255_at	suppressor of Ty 5 homolog (S. cerevisiae)	Supt5h	0.61139	1.11379778	0.15532303
1418816_at	chromatin modifying protein 1B	Chmp1b	0.61143	-1.13242463	-0.1801197
1441550_at	RIKEN cDNA 9330184L24 gene	9330184L24Rik	0.61153	1.11356646	0.15371792
1447163_x_at	---	---	0.61174	1.13722751	0.17953226
1419236_at	helicase (DNA) B	Helb	0.61183	1.13687252	0.18386062
1427937_at	RIKEN cDNA 2610030H06 gene	2610030H06Rik	0.6119	-1.15588341	-0.2100451
1449184_at	peptidoglycan recognition protein 1	Pglyrp1	0.61195	1.19234078	0.24805954
1443376_at	---	---	0.61214	-1.20040287	-0.2759723
1418868_at	engrailed 2	En2	0.61225	1.1697132	0.22512005
1449775_x_at	solute carrier family 35, member A4	Slc35a4	0.61233	-1.17123681	-0.2332147
1426853_at	SET nuclear oncogene	Set	0.61236	-1.11056611	-0.154815
1441107_at	doublesex and mab-3 related transcription factor like family A2	Dmrta2	0.61242	-1.1880399	-0.2566787
1444605_at	RIKEN cDNA 1700061F12 gene	1700061F12Rik	0.61248	1.16705567	0.2169565
1421101_a_at	LIM domain binding 2	Ldb2	0.61263	-1.16844565	-0.2277177
1438398_at	---	---	0.61271	-1.26472832	-0.4003796
1452289_a_at	ring finger protein 135	Rnf135	0.61282	1.15096238	0.2026862
1445078_at	DNA segment, Chr 7, ERATO Doi 526, expressed	D7Ertd526e	0.61289	-1.18359099	-0.2451366
1417343_at	FXYD domain-containing ion transport regulator 6	Fxyd6	0.61304	-1.13917567	-0.1928494
1419484_a_at	glioblastoma amplified sequence	Gbas	0.61328	-1.18974435	-0.2750274
1433963_a_at	fermitin family homolog 3 (Drosophila)	Fermt3	0.61336	1.22524922	0.2791526
1416943_at	ubiquitin-conjugating enzyme E2E 1, UBC4/5 homolog (yeast)	Ube2e1	0.61347	-1.12429674	-0.1720676
1416918_at	discs, large homolog 3 (Drosophila)	Dlg3	0.61355	1.14706346	0.19689937
1435496_at	RIKEN cDNA 5730469M10 gene	5730469M10Rik	0.61358	1.15468757	0.2067468
1446532_at	---	---	0.61364	-1.17528092	-0.2387971
1428477_at	elaC homolog 2 (E. coli)	Elac2	0.61367	1.12407282	0.16813391
1434720_at	zinc finger protein 652	Zfp652	0.61369	-1.1646587	-0.2242802
1435551_at	formin homology 2 domain containing 3	Fhod3	0.61371	1.14637649	0.19563248
1417130_s_at	angiopoietin-like 4	Angptl4	0.61376	1.13512241	0.17994793
1424405_at	MAP3K12 binding inhibitory protein 1	Mbip	0.61383	1.1327611	0.17851629
1456077_x_at	cell division cycle 25 homolog C (S. pombe)	Cdc25c	0.61384	1.17790307	0.22639163
1438923_at	Fibroblast growth factor (acidic) intracellular binding protein	Fibp	0.61394	-1.18549441	-0.2488671
1430708_a_at	ubiquitin specific petidase 45	Usp45	0.61415	-1.15577249	-0.2093555
1430115_at	RIKEN cDNA 4833413E03 gene	4833413E03Rik	0.61416	-1.21061763	-0.2973816
1422646_at	MAX gene associated	Mga	0.61417	-1.22001698	-0.313238
1440752_at	---	---	0.61421	-1.14181702	-0.1919748
1444330_at	DNA segment, Chr 2, ERATO Doi 173, expressed	D2Ertd173e	0.61421	-1.19887879	-0.2750846
1436186_at	E2F transcription factor 8	E2f8	0.61425	-1.13196848	-0.1791706
1449170_at	piwi-like homolog 2 (Drosophila)	Piwil2	0.61433	-1.13461516	-0.1834455
1451460_a_at	solute carrier family 22 (organic anion transporter), member 7	Slc22a7	0.61433	1.16603572	0.21764483
1417059_at	keratinocyte associated protein 2	Krtcap2	0.61435	1.13806255	0.18220645
1457382_at	---	---	0.61439	-1.18427521	-0.2448341
1433623_at	zinc finger protein 367	Zfp367	0.61445	-1.15827308	-0.2171498
1434777_at	v-myc myelocytomatosis viral oncogene homolog 1, lung carcino	Mycl1	0.61448	1.18175943	0.23216539
1449070_x_at	adenomatosis polyposis coli down-regulated 1	Apcdd1	0.61455	1.16786099	0.22270904
1457435_x_at	myomesin 2	Myom2	0.61455	1.23751113	0.29152482
1450664_at	GA repeat binding protein, alpha	Gabpa	0.61456	-1.11343874	-0.1558389
1426332_a_at	claudin 3	Cldn3	0.61456	1.1662416	0.22138654
1419056_at	reticulon 2 (Z-band associated protein)	Rtn2	0.61457	1.18356485	0.23465488
1423642_at	tubulin, beta 2C	Tubb2c	0.61457	1.07713556	0.10704633
1420186_at	---	---	0.61458	1.13025433	0.17569821
1417724_at	THO complex 4	Thoc4	0.6146	1.14905011	0.19249153
1427304_at	vacuolar protein sorting 18 (yeast)	Vps18	0.61464	1.14231013	0.19102091
1445645_at	---	---	0.61469	-1.15515413	-0.2117688
1444246_at	---	---	0.61471	-1.23860688	-0.3489441
1437406_x_at	insulin-like growth factor binding protein 4	Igfbp4	0.61472	1.16439884	0.21375521
1439835_x_at	ATP-binding cassette, sub-family D (ALD), member 2	Abcd2	0.61474	-1.16033732	-0.2146089
1443815_x_at	STE20-like kinase (yeast)	Slk	0.61479	-1.1771668	-0.2394495
1436430_at	RIKEN cDNA B430105G09 gene	B430105G09Rik	0.61486	1.198826	0.25401
1425568_a_at	transmembrane protein 33	Tmem33	0.61486	-1.11431128	-0.1564915
1447269_at	family with sequence similarity 184, member B	Fam184b	0.61497	1.17611207	0.23096136
1451972_at	glucocorticoid induced transcript 1	Glcci1	0.61503	1.19905485	0.252972
1423021_s_at	insulin-like 3 /// Janus kinase 3	Insl3 /// Jak3	0.61507	1.13737399	0.18233052

1439544_at	TBC1 domain family, member 5	Tbc1d5	0.61514	-1.14310818	-0.2018115
1450337_a_at	NIMA (never in mitosis gene a)-related expressed kinase 8	Nek8	0.61517	1.16059388	0.2129513
1448114_a_at	TRM2 tRNA methyltransferase 2 homolog A (S. cerevisiae)	Trmt2a	0.61519	1.13135552	0.17581947
1444173_at	hypothetical LOC100503725	LOC100503725	0.61521	-1.18121017	-0.2483037
1452270_s_at	cubilin (intrinsic factor-cobalamin receptor)	Cubn	0.61526	-1.15573681	-0.2096836
1435710_at	expressed sequence AI661384	AI661384	0.61531	1.16636794	0.2184788
1448233_at	prion protein	Prnp	0.61531	1.14967469	0.19810986
1418685_at	toll-interleukin 1 receptor (TIR) domain-containing adaptor protein	Tirap	0.61538	-1.17987598	-0.2440504
1452025_a_at	zinc finger protein 2	Zfp2	0.6154	1.17606967	0.22956609
1431688_at	hypothetical LOC73899	LOC73899	0.61541	1.15223923	0.2042088
1438432_at	leucyl/cystinyl aminopeptidase	Lnpep	0.61543	-1.18601801	-0.2657464
1449341_a_at	stomatin	Stom	0.61545	-1.17453234	-0.2352607
1451416_a_at	transglutaminase 1, K polypeptide	Tgm1	0.61545	-1.12130891	-0.1669419
1424438_a_at	leptin receptor overlapping transcript	Leprot	0.61546	1.12705369	0.17069803
1441641_at	RIKEN cDNA D630032N06 gene	D630032N06Rik	0.61551	1.16017469	0.21402796
1459808_at	FK506 binding protein 4	Fkbp4	0.61555	-1.14716309	-0.1982473
1449535_at	zinc and ring finger 4	Znrf4	0.61561	1.15790195	0.21123331
1449815_a_at	single-stranded DNA binding protein 2	Ssbp2	0.61564	1.16128864	0.21569514
1438213_at	RIKEN cDNA A830018L16 gene	A830018L16Rik	0.61569	-1.16514359	-0.2215138
1454093_at	RIKEN cDNA 4933436I20 gene	4933436I20Rik	0.61571	1.16426641	0.21688149
1432068_a_at	ectonucleoside triphosphate diphosphohydrolase 7	Entpd7	0.61575	-1.16710994	-0.2236815
1438204_at	Histone cluster 1, H1c	Hist1h1c	0.61584	-1.14458607	-0.1975633
1426412_at	neurogenic differentiation 1	Neurod1	0.616	1.16710585	0.2143297
1419359_at	hexamethylene bis-acetamide inducible 1	Hexim1	0.616	1.14935585	0.20037055
1441311_at	ribosomal protein S6 kinase, polypeptide 2	Rps6ka2	0.61607	1.15266023	0.20463126
1424459_at	lysophosphatidylcholine acyltransferase 1	Lpcat1	0.61621	1.12469162	0.16878162
1435308_at	fucosyltransferase 9	Fut9	0.61621	1.17411475	0.22586059
1417557_at	UBX domain protein 6	Ubxn6	0.61629	1.127139	0.17221364
1415764_at	zinc finger CCCH type containing 11A	Zc3h11a	0.61629	-1.10891106	-0.1499282
1416802_a_at	cell division cycle associated 5	Cdca5	0.61635	1.11768052	0.1584172
1426217_at	transmembrane protein 216	Tmem216	0.6164	1.16210432	0.20930304
1433109_at	macrophage migration inhibitory factor	Mif	0.61644	1.18850056	0.2432471
1424607_a_at	predicted gene 4354	Gm4354	0.61645	-1.16555696	-0.2376518
1455303_at	regulatory factor X-associated protein	Rfxap	0.61646	1.20193248	0.25649708
1426360_at	zinc finger CCCH type containing 11A	Zc3h11a	0.6165	-1.23090552	-0.3465828
1448824_at	ubiquitin-conjugating enzyme E2, J1	Ube2j1	0.61653	1.1499463	0.1972782
1422350_at	formyl peptide receptor, related sequence 3	Fpr-rs3	0.61659	-1.21477492	-0.3043264
1439874_at	RIKEN cDNA 9330102E08 gene	9330102E08Rik	0.61662	1.17161767	0.22731188
1444247_at	---	---	0.61663	-1.1427582	-0.1929437
1417174_at	transmembrane protein 218	Tmem218	0.61663	1.16671508	0.22178805
1456531_x_at	PRP19/PSO4 pre-mRNA processing factor 19 homolog (S. cerevisi	Prpf19	0.61673	-1.11280432	-0.1590821
1448799_s_at	mitochondrial ribosomal protein S12	Mrps12	0.61682	1.1136633	0.15472893
1448179_at	upregulated during skeletal muscle growth 5	Usmg5	0.61684	1.1214541	0.16320045
1454640_at	coiled-coil-helix-coiled-coil-helix domain containing 7	Chchd7	0.61696	1.15283488	0.19584751
1427684_at	zinc finger protein 264	Zfp264	0.61704	-1.20431237	-0.2799297
1425636_at	hedgehog acyltransferase	Hhat	0.61716	1.21684683	0.27364147
1457970_at	ARP1 actin-related protein 1 homolog A, centractin alpha (yeast)	Actr1a	0.61717	1.15937826	0.21095765
1451981_at	predicted gene, Gm16517	Gm16517	0.61727	1.18399083	0.22818304
1439601_at	HEAT repeat containing 6	Heatr6	0.61739	1.19287973	0.24790439
1435750_at	GTP cyclohydrolase I feedback regulator	Gchfr	0.61743	1.18132144	0.2370161
1459381_at	---	---	0.61775	-1.19131567	-0.2637952
1444147_at	proprotein convertase subtilisin/kexin type 2	Pcsk2	0.61836	-1.14173566	-0.1981897
1454342_at	RIKEN cDNA C030007D22 gene	C030007D22Rik	0.61839	-1.13400476	-0.1818968
1454602_s_at	CCR4-NOT transcription complex, subunit 2	Cnot2	0.61844	1.11550244	0.15767963
1437519_x_at	hydroxyacyl glutathione hydrolase	Hagh	0.61846	1.14421656	0.18855752
1453620_at	cortactin binding protein 2	Cttnbp2	0.61851	-1.15489323	-0.207762
1424594_at	sterile alpha motif domain containing 4	Samd4	0.61852	-1.17359244	-0.2325406
1440891_at	glutamate receptor, ionotropic, AMPA4 (alpha 4)	Gria4	0.61853	1.12699702	0.17177752
1454656_at	spermatogenesis associated 13	Spata13	0.6186	1.14145653	0.19015559
1456036_x_at	glutathione S-transferase omega 1	Gsto1	0.61864	-1.18054087	-0.2635261
1436791_at	wingless-related MMTV integration site 5A	Wnt5a	0.61866	1.20598311	0.25844256
1448499_a_at	epoxide hydrolase 2, cytoplasmic	Ephx2	0.61869	-1.12553803	-0.1727467
1456032_x_at	predicted pseudogene 8203 /// H2A histone family, member Z //	Gm8203 /// H2afz /// LOC	0.61874	-1.18551749	-0.2763825
1417080_a_at	ECSIT homolog (Drosophila)	Ecsit	0.61889	1.12347599	0.16583325
1432034_at	neurogenin 3	Neurog3	0.61894	1.16802146	0.21868494
1449394_at	solute carrier organic anion transporter family, member 1b2	Slco1b2	0.61909	1.16522338	0.21882687
1441559_at	RIKEN cDNA 3110082D06 gene	3110082D06Rik	0.61912	1.15738107	0.20940168
1460468_s_at	DnaJ (Hsp40) homolog, subfamily C, member 22	Dnajc22	0.61935	1.16427082	0.2171558
1423149_at	S-phase kinase-associated protein 1A	Skp1a	0.61937	-1.11339128	-0.1574784
1423295_at	transmembrane 9 superfamily member 2	Tm9sf2	0.6194	-1.29010205	-0.4481311
1448382_at	enoyl-Coenzyme A, hydratase/3-hydroxyacyl Coenzyme A dehyd	Ehhadh	0.61941	1.20883931	0.26198892
1421159_at	cingulin-like 1	Cgnl1	0.61951	-1.16224628	-0.2190745
1420356_at	ninjurin 2	Ninj2	0.61954	-1.16454539	-0.2199869
1443561_at	---	---	0.61955	1.14665884	0.19625701
1422671_s_at	N-acetylated alpha-linked acidic dipeptidase 2	Naalad2	0.61959	-1.20886413	-0.29578
1427962_at	coiled-coil domain containing 102A	Ccdc102a	0.61971	1.13486937	0.18138515
1455388_at	protein-L-isoaspartate (D-aspartate) O-methyltransferase domain	Pcmtd1	0.61974	-1.13062636	-0.1796877
1440803_x_at	tachykinin receptor 3	Tacr3	0.61976	-1.18359077	-0.2478088
1416725_at	transcription factor 4	Tcf4	0.61993	-1.20572779	-0.2889672
1437047_at	zinc finger protein 664	Zfp664	0.62012	1.18852316	0.24309714
1420892_at	wingless-related MMTV integration site 7B	Wnt7b	0.62026	-1.14622189	-0.2010754
1453060_at	regulator of G-protein signaling 8	Rgs8	0.62028	-1.18265332	-0.2493944
1421720_a_at	deltex 2 homolog (Drosophila)	Dtx2	0.62048	1.11877985	0.16182212
1440327_at	expressed sequence AI195470	AI195470	0.62054	1.16568899	0.21834606
1437678_at	predicted gene 1564	Gm1564	0.62059	-1.22967405	-0.3398842
1455269_a_at	coronin, actin binding protein 1A	Coro1a	0.6207	1.21992268	0.26923646
1437342_x_at	pituitary tumor-transforming 1 interacting protein	Pttg1ip	0.62077	1.12789026	0.17323966
1424254_at	interferon induced transmembrane protein 1	Ifitm1	0.62083	-1.12485373	-0.1785587
1418344_at	transmembrane protein 8 (five membrane-spanning domains)	Tmem8	0.62085	1.12927764	0.17466382
1448562_at	uridine phosphorylase 1	Upp1	0.6209	1.11344236	0.15129957
1445730_at	---	---	0.62092	-1.17319254	-0.23044
1428704_at	zinc finger protein 661	Zfp661	0.62093	1.17040601	0.2226505
1452793_at	DAZ interacting protein 1	Dzip1	0.62107	-1.172403	-0.2300646
1444448_at	predicted gene 10392	Gm10392	0.6211	-1.16064165	-0.2171852

1452299_at	WW domain containing E3 ubiquitin protein ligase 1	Wwp1	0.6212	-1.13531185	-0.1873093
1423475_at	cyclin M2	Cnnm2	0.62126	1.13348396	0.17987887
1437156_at	N-terminal EF-hand calcium binding protein 1	Necab1	0.62131	-1.14878308	-0.2073185
1438143_s_at	ataxin 2	Atxn2	0.62146	-1.18028483	-0.2606783
1460616_at	solute carrier organic anion transporter family, member 4C1	Slco4c1	0.6217	-1.2268136	-0.320396
1446119_at	hypothetical LOC100503783	LOC100503783	0.62185	-1.15865644	-0.2137097
1428076_s_at	predicted pseudogene 3244 /// NADH dehydrogenase (ubiquinor	Gm3244 /// Ndufb4	0.62199	1.11885774	0.15930129
1437492_at	mohawk homeobox	Mkx	0.622	1.19405097	0.24803522
1459315_at	---	---	0.62207	-1.16848028	-0.2256094
1425344_at	nuclear prelamin A recognition factor	Narf	0.62221	1.19276007	0.24036715
1435403_at	RIKEN cDNA 1700007L15 gene /// hypothetical LOC100502742	1700007L15Rik /// LOC10	0.62223	1.15995936	0.20959386
1443092_at	myotubularin related protein 3	Mtmr3	0.62239	-1.22682896	-0.3246296
1435521_at	Musashi homolog 2 (Drosophila)	Msi2	0.62239	-1.24441916	-0.3617855
1449208_at	poly (A) polymerase beta (testis specific)	Papolb	0.62241	-1.18924265	-0.2599132
1430313_at	ADAMTS-like 1	Adamtsl1	0.62243	1.18955267	0.24552071
1426714_at	solute carrier family 46, member 1	Slc46a1	0.62246	1.18483381	0.23649255
1447664_x_at	expressed sequence AA986860	AA986860	0.6225	1.17184546	0.22661569
1452237_at	ArfGAP with FG repeats 1	Agfg1	0.62251	-1.12695372	-0.1775034
1428666_at	asparaginyl-tRNA synthetase	Nars	0.62254	-1.10168584	-0.1415265
1438300_at	CDKN2A interacting protein	Cdkn2aip	0.62256	-1.16560376	-0.2221304
1455428_at	family with sequence similarity 53, member B	Fam53b	0.62258	1.1709962	0.22692518
1418406_at	phosphodiesterase 8A	Pde8a	0.6226	1.19368051	0.24378562
1459082_at	---	---	0.62269	-1.16060884	-0.2154716
1432721_at	RIKEN cDNA 5830424K16 gene	5830424K16Rik	0.62274	1.14810459	0.19711846
1450879_at	ATPase, class II, type 9B	Atp9b	0.62279	1.15057537	0.19685163
1451306_at	cell division cycle associated 7 like	Cdca7l	0.6228	1.14286823	0.18737918
1457552_at	zinc finger protein 295	Zfp295	0.62284	-1.17239957	-0.2306176
1434168_at	progressive external ophthalmoplegia 1 (human)	Peo1	0.62287	1.12138059	0.16448095
1417637_a_at	high mobility group 20 B	Hmg20b	0.62292	1.17001144	0.21764526
1430078_a_at	8-oxoguanine DNA-glycosylase 1	Ogg1	0.62316	1.14732956	0.19699486
1443109_at	---	---	0.62322	1.16695949	0.22032297
1459783_s_at	cappuccino	Cno	0.62328	-1.14780414	-0.2090173
1444579_at	---	---	0.62334	-1.19441757	-0.2687233
1417013_at	heat shock protein 8	Hspb8	0.62342	1.14494903	0.19455242
1448701_a_at	KRIT1, ankyrin repeat containing	Krit1	0.62353	-1.12674995	-0.1728511
1423465_at	ferric-chelate reductase 1	Frrs1	0.62358	-1.12937392	-0.1790316
1449259_at	RAB3D, member RAS oncogene family	Rab3d	0.62369	1.16110476	0.21322147
1444521_at	Phosphatidylinositol glycan anchor biosynthesis, class L	Pigl	0.62405	-1.11864941	-0.1629291
1429197_s_at	RAB GTPase activating protein 1-like	Rabgap1l	0.62407	1.18045385	0.23601619
1427023_at	phytanoyl-CoA hydroxylase interacting protein-like	Phyhipl	0.62447	1.15294696	0.20270201
1449699_s_at	RIKEN cDNA C330027C09 gene	C330027C09Rik	0.62525	-1.1206849	-0.1657526
1444270_at	radial spoke head 4 homolog A (Chlamydomonas)	Rsph4a	0.62578	-1.28908781	-0.4483585
1440848_at	RIKEN cDNA 1110028C15 gene	1110028C15Rik	0.62589	1.18614465	0.23983631
1442626_at	---	---	0.62609	-1.20814224	-0.2909753
1442593_at	---	---	0.62632	1.20743114	0.2612927
1427659_at	---	---	0.62637	-1.16168625	-0.2163414
1423858_a_at	3-hydroxy-3-methylglutaryl-Coenzyme A synthase 2	Hmgcs2	0.62729	-1.17397912	-0.2371378
1439736_at	RIKEN cDNA 5830453J16 gene	5830453J16Rik	0.62734	1.15336216	0.19550196
1448071_at	---	---	0.62736	1.15368678	0.19572447
1460452_at	motile sperm domain containing 3	Mospd3	0.62746	1.1584391	0.20523567
1437648_at	phosphate cytidylyltransferase 1, choline, beta isoform	Pcyt1b	0.62747	1.14589238	0.19434941
1423418_at	farnesyl diphosphate synthetase	Fdps	0.62748	1.12159688	0.1622875
1424638_at	cyclin-dependent kinase inhibitor 1A (P21)	Cdkn1a	0.62751	1.11553928	0.156256
1422213_s_at	forkhead box H1	Foxh1	0.62752	1.17186804	0.21699109
1415760_s_at	ATX1 (antioxidant protein 1) homolog 1 (yeast)	Atox1	0.62754	1.13348023	0.17539818
1446223_at	---	---	0.62755	1.18407077	0.24110716
1425050_at	isochorismatase domain containing 1	Isoc1	0.62756	-1.18033277	-0.2614143
1435776_at	RIKEN cDNA 2310009B15 gene	2310009B15Rik	0.62756	1.21632592	0.26502175
1456016_x_at	cDNA sequence BC005624	BC005624	0.62757	-1.2457952	-0.3573126
1436009_at	ubiquitin specific peptidase 30	Usp30	0.62761	1.176671	0.23090134
1440691_at	cytochrome P450, family 2, subfamily j, polypeptide 6	Cyp2j6	0.62761	-1.17031485	-0.2320995
1426230_at	sphingosine kinase 2	Sphk2	0.62763	-1.16876576	-0.2252566
1443535_at	RAB GTPase activating protein 1	Rabgap1	0.62764	1.17867431	0.23042131
1428409_at	N(alpha)-acetyltransferase 50, NatE catalytic subunit	Naa50	0.62765	-1.11219902	-0.1558426
1435989_x_at	keratin 8	Krt8	0.62765	-1.15216601	-0.2100334
1449905_at	C-type lectin domain family 4, member f	Clec4f	0.62766	-1.1585633	-0.2137676
1434461_at	zinc finger protein 715	Zfp715	0.62766	1.14356327	0.19003457
1437084_at	peptidyl arginine deiminase, type VI	Padi6	0.62769	-1.1789909	-0.2449145
1442383_at	---	---	0.6277	1.22308367	0.27473926
1421313_s_at	cortactin	Cttn	0.62771	1.14241912	0.18883745
1441651_at	RIKEN cDNA 4930414N06 gene	4930414N06Rik	0.62773	-1.17763268	-0.2430579
1441837_at	cholinergic receptor, nicotinic, beta polypeptide 2 (neuronal)	Chrnb2	0.62775	-1.1715203	-0.2339571
1424845_a_at	centrosomal protein 68	Cep68	0.62776	1.17254819	0.22104767
1458200_at	---	---	0.62776	-1.20460512	-0.2882751
1417232_at	claudin 2	Cldn2	0.62777	-1.2038348	-0.2833698
1444615_x_at	runt-related transcription factor 1; translocated to, 1 (cyclin D-rela	Runx1t1	0.62777	-1.20886252	-0.3031535
1459800_s_at	mitogen-activated protein kinase kinase kinase 4	Map3k4	0.62778	1.13756363	0.1829116
1436532_at	doublecortin-like kinase 3	Dclk3	0.62778	1.17521093	0.23083951
1435589_at	coiled-coil domain containing 85B	Ccdc85b	0.62778	1.16227295	0.21432187
1437668_at	Chemokine (C-C motif) receptor-like 1	Ccrl1	0.6278	-1.18367489	-0.2511803
1425562_s_at	tRNA nucleotidyl transferase, CCA-adding, 1	Trnt1	0.6278	-1.12445399	-0.1738857
1453284_at	family with sequence similarity 151, member B	Fam151b	0.62784	-1.18167118	-0.2497354
1427277_at	sine oculis-related homeobox 1 homolog (Drosophila)	Six1	0.62784	1.2321301	0.28216873
1436346_at	CD109 antigen	Cd109	0.62786	1.18680256	0.24019037
1457383_at	---	---	0.62787	-1.16438086	-0.2212193
1458756_at	---	---	0.6279	-1.1379514	-0.1903818
1460276_a_at	transmembrane protein, adipocyte asscociated 1	Tpra1	0.6279	1.15303389	0.20430539
1441988_at	protein phosphatase 1K (PP2C domain containing)	Ppm1k	0.62791	-1.16166488	-0.2170558
1438701_at	bicaudal D homolog 1 (Drosophila)	Bicd1	0.62791	-1.17259384	-0.2305172
1455600_at	ribosomal protein S3	Rps3	0.62793	1.13850584	0.18119999
1434214_at	RIKEN cDNA 0910001L09 gene	0910001L09Rik	0.62794	1.14069781	0.18316592
1450106_a_at	Ena-vasodilator stimulated phosphoprotein	Evl	0.62795	1.16579151	0.21169479
1429296_at	RAB10, member RAS oncogene family	Rab10	0.62797	-1.21587906	-0.3345625
1439164_at	Cyclin-dependent kinase inhibitor 2C (p18, inhibits CDK4)	Cdkn2c	0.62798	-1.16992004	-0.2275678

1417420_at	cyclin D1	Ccnd1	0.62798	1.12206516	0.16342674
1422198_a_at	serine hydroxymethyltransferase 1 (soluble)	Shmt1	0.628	1.12105521	0.1646017
1428680_at	CDP-diacylglycerol synthase 1	Cds1	0.628	1.2318686	0.28221423
1452445_at	solute carrier family 41, member 2	Slc41a2	0.628	1.21602473	0.26588836
1459386_at	zinc finger protein 709	Zfp709	0.628	1.166249	0.22144588
1439429_x_at	deltex 2 homolog (Drosophila)	Dtx2	0.62801	1.13906936	0.18379884
1423710_at	dihydrolipoamide S-succinyltransferase (E2 component of 2-oxo-g	Dlst	0.62801	1.11664069	0.15832056
1449013_at	eukaryotic elongation factor-2 kinase	Eef2k	0.62802	1.15813752	0.20711367
1422039_at	tumor necrosis factor receptor superfamily, member 22	Tnfrsf22	0.62803	-1.16527226	-0.221513
1436952_at	Kruppel-like factor 9	Klf9	0.62803	-1.14310797	-0.1940766
1423049_a_at	tropomyosin 1, alpha	Tpm1	0.62804	1.11327281	0.15382765
1441350_at	fibroblast growth factor 3	Fgf3	0.62805	1.21240997	0.265039
1425350_a_at	myelin basic protein expression factor 2, repressor	Myef2	0.62805	-1.14168547	-0.2002485
1426900_at	jumonji domain containing 1C	Jmjd1c	0.62806	-1.104874	-0.1442748
1457141_at	---	---	0.62807	1.18376061	0.23971101
1438848_at	---	---	0.62808	1.31436904	0.3539946
1418395_at	solute carrier family 47, member 1	Slc47a1	0.62809	1.15303007	0.2013661
1452059_at	solute carrier family 35, member F5	Slc35f5	0.6281	1.1459917	0.19304078
1416030_a_at	minichromosome maintenance deficient 7 (S. cerevisiae)	Mcm7	0.6281	1.09289318	0.12777383
1449027_at	ras homolog gene family, member U	Rhou	0.6281	1.14182124	0.18970255
1442069_at	ethanolaminephosphotransferase 1 (CDP-ethanolamine-specific)	Ept1	0.62811	-1.12000182	-0.163644
1449227_at	cholesterol 25-hydroxylase	Ch25h	0.62811	-1.16923173	-0.2258865
1444778_at	septin 3	Sep-03	0.62812	-1.17385485	-0.2338244
1438412_at	---	---	0.62812	1.18409762	0.23624985
1444071_at	RIKEN cDNA 9630013A20 gene	9630013A20Rik	0.62813	1.17094886	0.2252571
1454850_at	TBC1 domain family, member 10c	Tbc1d10c	0.62813	1.15843758	0.20972884
1456162_x_at	adducin 3 (gamma)	Add3	0.62814	-1.13433174	-0.1848968
1438749_at	fuzzy homolog (Drosophila)	Fuz	0.62814	1.18867839	0.24777013
1450171_x_at	granzyme E	Gzme	0.62814	1.14147896	0.18908647
1437472_at	unc-13 homolog A (C. elegans)	Unc13a	0.62814	1.20695977	0.25999334
1442029_at	KCNQ1 overlapping transcript 1	Kcnq1ot1	0.62816	-1.20738121	-0.2956442
1434543_a_at	bolA-like 2 (E. coli)	Bola2	0.62816	1.15268844	0.19450854
1438756_at	ankyrin repeat domain 29	Ankrd29	0.62817	-1.16882953	-0.2347061
1437241_at	Kruppel-like factor 11	Klf11	0.62817	1.17745464	0.22549918
1433970_at	bolA-like 3 (E. coli)	Bola3	0.62817	1.14987561	0.19656498
1458623_at	---	---	0.62817	1.16853546	0.22360198
1454611_a_at	calmodulin 1	Calm1	0.62817	-1.11991266	-0.170472
1455705_at	transmembrane protein 161A	Tmem161a	0.62818	1.27159772	0.31599061
1424882_a_at	5'-nucleotidase domain containing 2	Nt5dc2	0.62819	1.12667492	0.16969783
1435478_at	WD repeat domain 26	Wdr26	0.62819	-1.12297417	-0.1721157
1437278_a_at	ubiquitin-like modifier activating enzyme 2	Uba2	0.62822	-1.10268455	-0.1453057
1458772_at	---	---	0.62822	1.19485201	0.24074226
1434309_at	farnesyltransferase, CAAX box, beta	Fntb	0.62822	-1.12550782	-0.1759094
1417133_at	peripheral myelin protein 22	Pmp22	0.62823	1.3073084	0.34491502
1422806_x_at	inhibitor of growth family, member 3	Ing3	0.62823	-1.13114215	-0.1801204
1456070_at	protein tyrosine phosphatase, receptor type, G	Ptprg	0.62824	-1.17237623	-0.2352257
1450776_at	1-acylglycerol-3-phosphate O-acyltransferase 6 (lysophosphatidi	Agpat6	0.62824	1.13748593	0.18170806
1454993_a_at	serine/arginine-rich splicing factor 3	Srsf3	0.62825	-1.11782716	-0.1638438
1433742_at	KN motif and ankyrin repeat domains 1	Kank1	0.62825	1.15238683	0.20220365
1419982_s_at	---	---	0.62826	-1.18962742	-0.2690789
1424573_at	transmembrane emp24 protein transport domain containing 5	Tmed5	0.62827	-1.168734	-0.2345281
1449324_at	ERO1-like (S. cerevisiae)	Ero1l	0.62827	1.12626126	0.17045861
1423445_at	Rho-associated coiled-coil containing protein kinase 1	Rock1	0.62828	-1.14289111	-0.2007542
1417747_at	complexin 1	Cplx1	0.62828	1.17438754	0.22317123
1455990_at	kinesin family member 23	Kif23	0.6283	1.12935735	0.17097959
1451110_at	EGL nine homolog 1 (C. elegans)	Egln1	0.6283	1.20022122	0.24519458
1454580_at	RIKEN cDNA 5430427N15 gene	5430427N15Rik	0.62831	-1.17436691	-0.2324742
1434111_at	latrophilin 2	Lphn2	0.62832	-1.14794514	-0.2033424
1434702_at	DDRGK domain containing 1	Ddrgk1	0.62835	1.13599503	0.18083133
1417948_s_at	interleukin enhancer binding factor 2	Ilf2	0.62837	-1.12460971	-0.1743268
1418347_at	coiled-coil domain containing 22	Ccdc22	0.62839	1.12606876	0.16994632
1438915_at	RIKEN cDNA 6720401G13 gene	6720401G13Rik	0.6284	1.19920501	0.25431078
1417593_at	tumor suppressor candidate 2	Tusc2	0.62841	1.12684381	0.17077629
1423749_s_at	RAN GTPase activating protein 1	Rangap1	0.62843	1.11177548	0.15127085
1428741_at	ELAV (embryonic lethal, abnormal vision, Drosophila)-like 4 (Hu a	Elavl4	0.62847	-1.17241801	-0.233684
1448054_at	---	---	0.62849	-1.17015794	-0.2306513
1440261_at	adaptor-related protein complex AP-4, epsilon 1	Ap4e1	0.62849	1.12429906	0.16838155
1434205_at	protein phosphatase 2, regulatory subunit B (B56), gamma isoforr	Ppp2r5c	0.6285	-1.14236935	-0.1951343
1436728_s_at	regulator of telomere elongation helicase 1	Rtel1	0.6285	1.13039248	0.17539804
1423242_at	mitochondrial ribosomal protein S36	Mrps36	0.62851	1.12684052	0.16883008
1420981_a_at	LIM domain only 4	Lmo4	0.62851	-1.14403815	-0.2041166
1427166_a_at	spastic paraplegia 7 homolog (human)	Spg7	0.62852	1.12337322	0.16709806
1450109_s_at	ATP-binding cassette, sub-family C (CFTR/MRP), member 2	Abcc2	0.62853	-1.17881225	-0.2477751
1451814_a_at	HIV-1 tat interactive protein 2, homolog (human)	Htatip2	0.62854	1.13801083	0.18640037
1444531_at	---	---	0.62854	-1.16672425	-0.2267366
1417746_at	complexin 1	Cplx1	0.62856	1.15494941	0.20294529
1435534_a_at	translocase of outer mitochondrial membrane 20 homolog (yeast	Tomm20	0.62857	-1.12817509	-0.1811798
1417480_at	f-box protein 9	Fbxo9	0.62858	1.13713387	0.18183442
1456398_at	Taurine upregulated gene 1	Tug1	0.62859	-1.20007343	-0.3057269
1416812_at	cytotoxic granule-associated RNA binding protein 1	Tia1	0.62864	1.15617112	0.19865172
1437632_at	mediator complex subunit 14	Med14	0.62865	-1.15083508	-0.2117451
1424854_at	histone cluster 1, H4a /// histone cluster 1, H4b /// histone cluster	Hist1h4a /// Hist1h4b ///	0.62866	1.18880269	0.23950369
1449187_at	platelet derived growth factor, alpha	Pdgfa	0.62866	-1.12431774	-0.1736974
1426877_a_at	polybromo 1	Pbrm1	0.62866	-1.1180085	-0.1629085
1440197_at	cDNA sequence BC032203	BC032203	0.62867	-1.14585174	-0.1964652
1433852_at	kinase D-interacting substrate 220	Kidins220	0.62867	1.15396362	0.20493722
1460706_s_at	RER1 retention in endoplasmic reticulum 1 homolog (S. cerevisiae	Rer1	0.62868	1.11280957	0.15394147
1429129_at	leucine rich repeat and coiled-coil domain containing 1	Lrrcc1	0.62868	-1.18197199	-0.2489336
1424316_at	solute carrier family 25 (mitochondrial thiamine pyrophosphate ca	Slc25a19	0.62869	1.13590045	0.18174811
1459969_x_at	---	---	0.62869	-1.15614578	-0.2106274
1455730_at	discs, large (Drosophila) homolog-associated protein 5	Dlgap5	0.62869	1.11458882	0.1558858
1429377_at	RIKEN cDNA 2410004A20 gene	2410004A20Rik	0.62871	1.12104356	0.16334757
1444588_at	---	---	0.62872	-1.16502621	-0.2215361
1433640_at	far upstream element (FUSE) binding protein 1	Fubp1	0.62873	-1.12848945	-0.1789393

1459923_at	brain expressed gene 6	Bex6	0.62875	-1.19114714	-0.2768537
1457928_at	RIKEN cDNA 4930435E12 gene	4930435E12Rik	0.62877	-1.17127971	-0.2314299
1458255_at	---	---	0.62877	-1.17132185	-0.2331126
1449649_at	ATPase, H+ transporting, lysosomal V1 subunit B2	Atp6v1b2	0.62879	-1.17141574	-0.2295745
1456648_at	---	---	0.6288	-1.16704966	-0.2264254
1452200_at	CDKN2A interacting protein N-terminal like	Cdkn2aipnl	0.62881	1.12112736	0.16350059
1436227_at	left-right determination factor 2	Lefty2	0.62881	-1.1428933	-0.1978151
1430283_s_at	leucine rich repeat containing 67	Lrrc67	0.62882	-1.14482212	-0.1959131
1424716_at	retinol saturase (all trans retinol 13,14 reductase)	Retsat	0.62884	1.16274406	0.2173796
1445714_at	RIKEN cDNA B930042K01 gene	B930042K01Rik	0.62884	-1.15141847	-0.2035715
1452031_at	solute carrier family 1 (glial high affinity glutamate transporter), m	Slc1a3	0.62885	-1.16282785	-0.2189114
1460330_at	annexin A3	Anxa3	0.62886	1.15235635	0.20344772
1451383_a_at	conserved helix-loop-helix ubiquitous kinase	Chuk	0.62887	-1.1313423	-0.1820859
1437856_at	inositol polyphosphate multikinase	Ipmk	0.62887	-1.13254022	-0.1812161
1426447_at	nucleoporin 35	Nup35	0.62888	1.11088641	0.1515256
1446736_at	---	---	0.62888	1.16109368	0.20710314
1420175_at	Tax1 (human T-cell leukemia virus type I) binding protein 1	Tax1bp1	0.62888	-1.17049662	-0.23104
1423754_at	interferon induced transmembrane protein 3	Ifitm3	0.62888	-1.10789914	-0.1533364
1417766_at	cytochrome b5 type B	Cyb5b	0.62889	1.12632453	0.16724611
1415972_at	myristoylated alanine rich protein kinase C substrate	Marcks	0.62889	1.17433013	0.21602433
1433384_at	RIKEN cDNA 9330199C07 gene	9330199C07Rik	0.6289	1.16330222	0.21728232
1439305_at	---	---	0.6289	1.17763058	0.23100233
1454655_at	diacylglycerol kinase, delta	Dgkd	0.62891	-1.1724744	-0.248629
1441010_at	hypothetical LOC100502834	LOC100502834	0.62892	1.24010671	0.28657653
1435792_at	component of Sp100-rs /// predicted gene 7592	Csprs /// Gm7592	0.62892	1.19550969	0.24924838
1448609_at	thiosulfate sulfurtransferase, mitochondrial	Tst	0.62893	1.14454613	0.1894018
1447440_at	nuclear transport factor 2 /// nuclear transport factor 2, pseudoge	Nutf2 /// Nutf2-ps1	0.62895	1.19819657	0.25085021
1426223_at	tetratricopeptide repeat domain 39C	Ttc39c	0.62896	1.17635558	0.23124197
1451021_a_at	Kruppel-like factor 5	Klf5	0.62897	-1.10361495	-0.147081
1449109_at	suppressor of cytokine signaling 2	Socs2	0.62898	-1.16275298	-0.2383025
1437874_s_at	hexosaminidase B	Hexb	0.62898	-1.11316074	-0.1562106
1445637_at	---	---	0.62899	-1.17226955	-0.2361773
1437396_at	cAMP responsive element binding protein 3-like 2	Creb3l2	0.62899	1.20331114	0.25053753
1437845_x_at	protein O-fucosyltransferase 2	Pofut2	0.629	-1.17005329	-0.2444522
1433672_at	TRM2 tRNA methyltransferase 2 homolog B (S. cerevisiae)	Trmt2b	0.629	1.16459362	0.21813109
1449538_a_at	glucosaminyl (N-acetyl) transferase 1, core 2	Gcnt1	0.62901	1.19509519	0.24480934
1421907_at	mediator complex subunit 1	Med1	0.62902	-1.14093117	-0.1971863
1426255_at	neurofilament, light polypeptide	Nefl	0.62904	1.13363782	0.17962385
1416067_at	interferon-related developmental regulator 1	Ifrd1	0.62905	-1.11945652	-0.1666601
1437330_at	leucine-rich repeat kinase 1	Lrrk1	0.62906	-1.19273421	-0.266257
1452949_at	polymerase (RNA) III (DNA directed) polypeptide B	Polr3b	0.62907	1.13564411	0.18166161
1443695_at	hyaluronic acid binding protein 2	Habp2	0.62908	-1.16216971	-0.2214876
1418434_at	makorin, ring finger protein, 1	Mkrn1	0.62909	-1.09896476	-0.1363663
1456714_at	WW domain containing E3 ubiquitin protein ligase 2	Wwp2	0.62909	1.15677559	0.20205933
1442182_at	DnaJ (Hsp40) homolog, subfamily C, member 19	Dnajc19	0.6291	-1.15520267	-0.2085073
1423696_a_at	proteasome (prosome, macropain) 26S subunit, non-ATPase, 6	Psmd6	0.6291	-1.24727305	-0.3902754
1428096_at	importin 11	Ipo11	0.6291	1.14328303	0.18640225
1450359_at	fucosyltransferase 1	Fut1	0.62911	-1.17174535	-0.2329417
1458790_at	---	---	0.62911	1.15422951	0.20503805
1432189_a_at	SRY-box containing gene 5	Sox5	0.62913	1.17657017	0.22918063
1440789_at	neogenin	Neo1	0.62914	1.18627088	0.23815511
1455471_at	canopy 1 homolog (zebrafish)	Cnpy1	0.62914	1.15657859	0.20764704
1434578_x_at	RAN, member RAS oncogene family	Ran	0.62915	-1.11535706	-0.1680351
1433906_at	clavesin 1	Clvs1	0.62916	-1.2057986	-0.293604
1456287_at	expressed sequence BB236558	BB236558	0.62916	1.17122077	0.22423113
1448191_at	polo-like kinase 1 (Drosophila)	Plk1	0.62918	1.10799489	0.14669568
1444039_at	dispatched homolog 2 (Drosophila)	Disp2	0.62918	1.16963607	0.22354737
1435172_at	eomesodermin homolog (Xenopus laevis)	Eomes	0.62919	1.18067108	0.23005932
1458964_at	---	---	0.62921	1.25837431	0.30050202
1437943_s_at	male enhanced antigen 1	Mea1	0.62923	1.18472872	0.22815853
1425857_at	F-box and WD-40 domain protein 9	Fbxw9	0.62923	1.1450064	0.19158197
1435410_at	testicular cell adhesion molecule 1	Tcam1	0.62924	-1.16605509	-0.2248376
1424704_at	runt related transcription factor 2	Runx2	0.62926	1.14276487	0.18658646
1426780_at	VATG14 autophagy related 14 homolog (S. cerevisiae)	Atg14	0.62926	1.11987133	0.16311022
1450413_at	platelet derived growth factor, B polypeptide	Pdgfb	0.62926	1.20735402	0.25721669
1444744_at	---	---	0.62927	1.19100756	0.24278315
1429993_s_at	predicted gene 10471 /// spermatogenesis associated glutamate	Gm10471 /// Speer4b	0.62927	1.15678976	0.20731562
1421757_at	5-hydroxytryptamine (serotonin) receptor 6	Htr6	0.62927	1.18849196	0.24074578
1435743_at	kelch-like 23 (Drosophila)	Klhl23	0.62927	1.22639009	0.27785791
1434931_at	neogenin	Neo1	0.62928	1.16339318	0.21280368
1420840_at	pleckstrin homology domain-containing, family A (phosphoinositi	Plekha3	0.62929	-1.13069011	-0.1792574
1421881_a_at	ELAV (embryonic lethal, abnormal vision, Drosophila)-like 2 (Hu a	Elavl2	0.62929	1.15397325	0.20209807
1423255_at	ATPase, H+ transporting, lysosomal V1 subunit G1	Atp6v1g1	0.62929	1.11382481	0.15405286
1423946_at	PDZ and LIM domain 2	Pdlim2	0.62931	1.15938804	0.20639782
1421875_a_at	mitochondrial ribosomal protein S23	Mrps23	0.62931	1.10943134	0.14951468
1427052_at	acetyl-Coenzyme A carboxylase beta	Acacb	0.62931	1.15775499	0.20564274
1428406_s_at	host cell factor C1 regulator 1 (XPO1-dependent)	Hcfc1r1	0.62934	1.11408365	0.14867988
1424986_s_at	F-box and WD-40 domain protein 7	Fbxw7	0.62934	-1.14365898	-0.2028779
1444699_at	SURP and G patch domain containing 1	Sugp1	0.62935	1.16944167	0.22296135
1427821_at	coatomer protein complex, subunit gamma 2, antisense 2	Copg2as2	0.62936	-1.17699727	-0.2401609
1425023_at	ubiquitin specific peptidase 3	Usp3	0.62936	1.17945005	0.22648152
1449042_at	CCCTC-binding factor	Ctcf	0.62937	-1.18659162	-0.2781527
1455233_at	mitochondrial ribosomal protein S11	Mrps11	0.62938	-1.14921145	-0.2158768
1458161_at	KCNQ1 overlapping transcript 1	Kcnq1ot1	0.62939	1.20384747	0.2494802
1417788_at	synuclein, gamma	Sncg	0.62939	1.22770683	0.27532705
1419089_at	tissue inhibitor of metalloproteinase 3	Timp3	0.6294	-1.17255008	-0.2345212
1426926_at	phospholipase C, gamma 2	Plcg2	0.62942	1.11621297	0.15804281
1437313_x_at	high mobility group box 2	Hmgb2	0.62942	-1.20724328	-0.3191651
1437674_at	RAS-related C3 botulinum substrate 1	Rac1	0.62942	-1.15688834	-0.2152595
1459181_at	---	---	0.62943	-1.17314023	-0.2328948
1451261_s_at	signal transducing adaptor family member 2	Stap2	0.62943	1.14152175	0.18824009
1458609_at	hypothetical LOC100504304	LOC100504304	0.62943	1.13725751	0.18281531
1427266_at	polybromo 1	Pbrm1	0.62945	-1.12553041	-0.1754884
1423672_at	tetratricopeptide repeat domain 30B	Ttc30b	0.62945	-1.15162612	-0.207141

1428065_at	solute carrier family 44, member 2	Slc44a2	0.62946	1.13676729	0.18214082
1428854_at	transmembrane emp24 domain containing 8	Tmed8	0.62946	-1.11974724	-0.1661504
1456106_x_at	serologically defined colon cancer antigen 3	Sdccag3	0.62946	-1.16509172	-0.2222774
1459302_at	RIKEN cDNA A030007N12 gene	A030007N12Rik	0.62946	-1.20716486	-0.2980241
1425724_at	protein tyrosine phosphatase, receptor type, N polypeptide 2	Ptprn2	0.62946	-1.18182908	-0.2492854
1449161_at	endothelin 2	Edn2	0.62947	1.24263213	0.29228092
1457866_at	expressed sequence C77717	C77717	0.62947	-1.16391699	-0.2195523
1423449_a_at	actinin alpha 4	Actn4	0.62947	-1.11868478	-0.1671638
1453964_at	RIKEN cDNA 1700021A07 gene	1700021A07Rik	0.62947	-1.16420595	-0.2248621
1456794_at	RIKEN cDNA E230016M11 gene	E230016M11Rik	0.62948	-1.17792108	-0.2409187
1425939_at	RAD50 homolog (S. cerevisiae)	Rad50	0.62948	1.18227056	0.23787746
1450077_at	chromodomain helicase DNA binding protein 1	Chd1	0.62951	-1.14738968	-0.2032014
1448531_at	lamin B2	Lmnb2	0.62951	1.11764679	0.15884342
1423543_at	SWA-70 protein	Swap70	0.62951	1.13443577	0.17911195
1435757_a_at	ubiquinol cytochrome c reductase core protein 2	Uqcrc2	0.62951	-1.13483507	-0.1928414
1437290_at	inositol monophosphatase domain containing 1	Impad1	0.62952	-1.14907194	-0.2118876
1417782_at	LAG1 homolog, ceramide synthase 4	Lass4	0.62953	-1.16032062	-0.2151514
1420537_at	potassium channel tetramerisation domain containing 4	Kctd4	0.62954	1.16485026	0.21899547
1423055_at	neuron specific gene family member 1	Nsg1	0.62955	-1.15368893	-0.2100929
1436381_at	discs, large (Drosophila) homolog-associated protein 3	Dlgap3	0.62955	1.13334648	0.17727394
1423365_at	calcium channel, voltage-dependent, T type, alpha 1G subunit	Cacna1g	0.62956	-1.17509378	-0.2435193
1434010_at	family with sequence similarity 117, member B	Fam117b	0.62957	1.13702888	0.18161692
1457300_at	aftiphilin	Aftph	0.62958	1.15336621	0.20228581
1438200_at	sulfatase 1	Sulf1	0.62958	1.13796745	0.18581575
1446259_at	---	---	0.62958	-1.17507708	-0.2369389
1460610_at	ATP/GTP binding protein-like 5	Agbl5	0.62958	1.14584185	0.19368073
1435838_at	alanine-glyoxylate aminotransferase 2	Agxt2	0.62959	-1.15290483	-0.2062115
1456575_at	Necdin	Ndn	0.62959	-1.16504889	-0.2249608
1429693_at	disabled homolog 2 (Drosophila)	Dab2	0.62959	-1.16791686	-0.2268161
1435608_at	zinc and ring finger 3	Znrf3	0.62959	-1.18065517	-0.2558406
1418902_at	chitinase domain containing 1	Chid1	0.6296	1.17963056	0.23343909
1417312_at	dickkopf homolog 3 (Xenopus laevis)	Dkk3	0.6296	1.1633513	0.21671009
1425858_at	ubiquitin-conjugating enzyme E2M (UBC12 homolog, yeast)	Ube2m	0.6296	-1.15780177	-0.2177719
1431686_a_at	glia maturation factor, beta	Gmfb	0.62961	-1.22908525	-0.3476823
1420967_at	solute carrier family 25 (mitochondrial carrier ornithine transporte	Slc25a15	0.62962	-1.12354985	-0.1703047
1428682_at	zinc finger CCCH type containing 6	Zc3h6	0.62962	-1.17146853	-0.2379333
1459500_at	expressed sequence C85351	C85351	0.62962	-1.14704734	-0.2027922
1422563_at	complement component (3b/4b) receptor 1-like	Cr1l	0.62963	-1.13175883	-0.1831505
1448310_at	intestinal cell kinase	Ick	0.62963	1.14943954	0.19950338
1433509_s_at	receptor accessory protein 1	Reep1	0.62964	1.15168523	0.20103906
1455346_at	mannan-binding lectin serine peptidase 1	Masp1	0.62964	1.18373091	0.2341996
1420935_a_at	serine/arginine repetitive matrix 1	Srrm1	0.62965	-1.18652929	-0.2773577
1442528_at	exportin 4	Xpo4	0.62965	-1.16451018	-0.2244993
1417198_at	WW, C2 and coiled-coil domain containing 2	Wwc2	0.62966	-1.16663176	-0.225955
1439487_at	ligase IV, DNA, ATP-dependent	Lig4	0.62967	-1.14585428	-0.197013
1451703_s_at	adenine phosphoribosyl transferase	Aprt	0.62967	1.11027372	0.14908963
1440106_at	---	---	0.62967	-1.16260059	-0.220344
1439441_x_at	large tumor suppressor 2	Lats2	0.62968	-1.16633224	-0.2275316
1456574_at	zinc finger protein 800	Zfp800	0.62969	-1.15594378	-0.2093042
1429488_at	zinc finger, DHHC domain containing 21	Zdhhc21	0.62969	-1.15464882	-0.217285
1438082_at	transmembrane protein 206	Tmem206	0.62969	-1.1558656	-0.2147091
1450021_at	ubiquilin 2	Ubqln2	0.62973	-1.11263406	-0.1554185
1446001_at	---	---	0.62973	1.18144941	0.23510343
1452433_at	---	---	0.62975	1.17109893	0.22003413
1439184_s_at	thioredoxin domain containing 17	Txndc17	0.62976	-1.15604936	-0.23028
1423075_at	lectin, mannose-binding 2	Lman2	0.62977	1.14367134	0.18708566
1424913_at	RIKEN cDNA 2310044G17 gene	2310044G17Rik	0.62978	-1.14350551	-0.1955239
1435234_at	nuclear receptor coactivator 2	Ncoa2	0.62978	1.18910632	0.23837163
1436321_at	UDP-GlcNAc:betaGal beta-1,3-N-acetylglucosaminyltransferase 7	B3gnt7	0.62978	1.12120516	0.164312
1439722_at	---	---	0.62979	1.15945648	0.2133752
1438509_at	Proteaseome (prosome, macropain) 28 subunit, 3	Psme3	0.62979	-1.15666533	-0.2116016
1435117_a_at	DnaJ (Hsp40) homolog, subfamily C, member 27	Dnajc27	0.62979	1.13618687	0.18350147
1437032_x_at	RNA binding motif protein 14	Rbm14	0.62981	1.10743949	0.14708729
1451415_at	RIKEN cDNA 1810011O10 gene	1810011O10Rik	0.62983	1.21147169	0.26429743
1432123_at	RIKEN cDNA 4930455H04 gene	4930455H04Rik	0.62983	-1.14797639	-0.2032433
1443203_at	---	---	0.62983	1.26428514	0.30695635
1446309_at	---	---	0.62983	-1.19030936	-0.2635906
1438452_at	nebulette	Nebl	0.62983	-1.23169585	-0.3342245
1450208_a_at	engulfment and cell motility 1, ced-12 homolog (C. elegans)	Elmo1	0.62983	1.26538803	0.30786104
1419455_at	interleukin 10 receptor, beta	Il10rb	0.62984	1.17532753	0.22772336
1416480_a_at	predicted gene 9790 /// HIG1 domain family, member 1A	Gm9790 /// Higd1a	0.62984	1.11659178	0.156384
1418743_a_at	tescalcin	Tesc	0.62986	1.17037262	0.22462309
1425054_a_at	RIKEN cDNA 2510006D16 gene	2510006D16Rik	0.62987	-1.15029066	-0.2182936
1459888_at	hypothetical protein LOC545261	LOC545261	0.62988	1.12898686	0.17465921
1425750_a_at	Janus kinase 3	Jak3	0.62988	1.13942215	0.18379407
1452875_at	HD domain containing 3	Hddc3	0.62988	1.18148036	0.23543637
1448425_at	eukaryotic translation initiation factor 3, subunit A	Eif3a	0.62989	-1.10336978	-0.1450836
1449958_a_at	fibroblast growth factor 14	Fgf14	0.62989	-1.1961929	-0.2732582
1418960_at	PHD finger protein 20-like 1	Phf20l1	0.62989	-1.20188585	-0.28713
1424264_at	mediator of RNA polymerase II transcription, subunit 6 homolog (	Med6	0.62989	-1.15506948	-0.2246881
1441052_at	---	---	0.6299	1.16727645	0.21998908
1426786_s_at	DEAH (Asp-Glu-Ala-His) box polypeptide 38	Dhx38	0.62992	1.11894967	0.16147953
1456484_at	dehydrogenase/reductase (SDR family) X chromosome	Dhrsx	0.62993	1.14894033	0.19833531
1435637_at	integrin alpha FG-GAP repeat containing 1	Itfg1	0.62993	1.17008799	0.22116126
1448839_at	KN motif and ankyrin repeat domains 3	Kank3	0.62995	1.14415526	0.18859098
1417084_at	eukaryotic translation initiation factor 4E binding protein 2	Eif4ebp2	0.62995	-1.25365198	-0.4025284
1443650_at	---	---	0.62996	-1.12964504	-0.1795172
1454868_at	transmembrane protein 201	Tmem201	0.62996	1.12936647	0.17405748
1428028_at	MKL/myocardin-like 2	Mkl2	0.62999	-1.15394468	-0.2074055
1432713_at	RIKEN cDNA 6430709C05 gene	6430709C05Rik	0.62999	1.15571944	0.20803298
1448169_at	keratin 18	Krt18	0.62999	1.16393037	0.2114036
1445310_at	synaptotagmin-like 3	Sytl3	0.63002	1.1850064	0.23463428
1455633_at	zinc finger protein 647	Zfp647	0.63002	-1.12249334	-0.1673695
1440954_at	---	---	0.63002	-1.16318455	-0.220458

1452827_at	RIKEN cDNA 1500009C09 gene	1500009C09Rik	0.63004	-1.18177005	-0.2518204
1438173_x_at	polyamine-modulated factor 1	Pmf1	0.63006	1.12080374	0.16164162
1444752_at	RIKEN cDNA C230073G13 gene	C230073G13Rik	0.63006	1.16730995	0.2227973
1442510_at	alpha-fetoprotein-like	LOC665792	0.63007	1.1669851	0.22119816
1418890_a_at	RAB3D, member RAS oncogene family	Rab3d	0.63009	1.1513081	0.20129256
1460419_a_at	protein kinase C, beta	Prkcb	0.6301	-1.13704149	-0.1853508
1428896_at	platelet-derived growth factor receptor-like	Pdgfrl	0.63011	-1.17378524	-0.2362039
1438299_at	RIKEN cDNA 9230108I15 gene	9230108I15Rik	0.63011	-1.1458219	-0.2003596
1443679_at	RIKEN cDNA 2510003B16 gene	2510003B16Rik	0.63012	1.19242531	0.24638503
1417629_at	proline dehydrogenase	Prodh	0.63014	1.14512273	0.19451986
1435809_at	ankyrin repeat domain 34A	Ankrd34a	0.63019	1.18868941	0.2379654
1417664_a_at	N-myc downstream regulated gene 3	Ndrg3	0.6302	1.18554927	0.23111529
1416419_s_at	gamma-aminobutyric acid (GABA) A receptor-associated protein-	Gabarapl1	0.6302	1.12632596	0.1709036
1423903_at	poliovirus receptor	Pvr	0.6302	1.13260054	0.17749402
1456983_at	---	---	0.63021	-1.25341405	-0.393067
1437477_at	leucine rich repeat (in FLII) interacting protein 1	Lrrfip1	0.63021	-1.19084258	-0.2658586
1435726_at	LIM domain and actin binding 1	Lima1	0.63023	1.20240444	0.25459215
1430597_at	janus kinase and microtubule interacting protein 1	Jakmip1	0.63028	1.16555608	0.21886634
1426679_at	zinc finger protein 706	Zfp706	0.63031	1.10999912	0.14911458
1422691_at	serine palmitoyltransferase, long chain base subunit 1	Sptlc1	0.63038	-1.17410676	-0.2365518
1419380_at	zinc finger protein 423	Zfp423	0.63042	-1.11442162	-0.1566386
1433284_at	RIKEN cDNA 4921522E08 gene	4921522E08Rik	0.63048	-1.16788251	-0.2263378
1451546_s_at	transmembrane protein 40	Tmem40	0.63049	1.20944984	0.25713184
1443068_at	RIKEN cDNA D130084N16 gene	D130084N16Rik	0.6305	-1.17157414	-0.2300734
1432775_at	RIKEN cDNA 9430087J23 gene	9430087J23Rik	0.63058	1.16505025	0.21579818
1445257_at	expressed sequence AU022531	AU022531	0.63059	1.14402923	0.19281139
1450744_at	elongation factor RNA polymerase II 2	Ell2	0.6306	-1.10706368	-0.1471872
1434287_at	1-acylglycerol-3-phosphate O-acyltransferase 5 (lysophosphatidi	Agpat5	0.63063	1.12791151	0.17268356
1429133_at	nucleoredoxin-like 2	Nxnl2	0.63064	1.18796023	0.24213289
1442755_at	---	---	0.63064	-1.1601024	-0.2178466
1455496_at	phosphoribosylformylglycinamidine synthase (FGAR amidotransf	Pfas	0.63064	-1.13818466	-0.1959049
1435397_at	zinc finger and BTB domain containing 44	Zbtb44	0.63066	-1.12366053	-0.1707293
1454235_a_at	inhibitor of growth family, member 5	Ing5	0.63067	1.13216804	0.17844479
1420877_at	septin 6	Sep-06	0.63069	1.18558421	0.24064053
1442679_at	---	---	0.6307	1.29202065	0.32899893
1422966_a_at	transferrin receptor	Tfrc	0.63071	1.18452331	0.22671334
1430729_at	melanocyte proliferating gene 1	Myg1	0.63073	1.2446929	0.29238265
1456528_x_at	nucleolin	Ncl	0.63077	-1.11947428	-0.1657407
1429025_a_at	hexamthylene bis-acetamide inducible 2	Hexim2	0.63078	1.167212	0.22023067
1457699_at	RIKEN cDNA E330009J07 gene	E330009J07Rik	0.6308	1.20549681	0.25799246
1426472_at	zinc finger protein 52	Zfp52	0.63081	1.23462175	0.28328862
1437588_at	POU domain, class 4, transcription factor 2	Pou4f2	0.63081	-1.15544542	-0.2125997
1426525_at	AT rich interactive domain 2 (ARID, RFX-like)	Arid2	0.63085	-1.12705038	-0.1796566
1418117_at	NADH dehydrogenase (ubiquinone) Fe-S protein 4	Ndufs4	0.63085	1.15402452	0.20562038
1451985_at	leucine-rich repeat kinase 1	Lrrk1	0.63086	1.17746005	0.2327703
1416672_s_at	mucolipin 1	Mcoln1	0.63089	1.13486319	0.18111591
1444430_at	---	---	0.6309	-1.17549525	-0.2354944
1439263_at	membrane protein, palmitoylated 6 (MAGUK p55 subfamily mem	Mpp6	0.6309	-1.19281915	-0.268903
1421067_a_at	PHD finger protein 2	Phf2	0.63091	1.13807301	0.17997765
1427351_s_at	immunoglobulin heavy chain 6 (heavy chain of IgM)	Igh-6	0.63093	1.16846764	0.22017559
1423668_at	zinc finger, DHHC domain containing 14	Zdhhc14	0.63095	-1.16961319	-0.2292417
1455855_x_at	heterogeneous nuclear ribonucleoprotein A/B	Hnrnpab	0.63095	-1.07062231	-0.0995929
1456823_at	predicted gene 70	Gm70	0.63095	1.17987924	0.23370506
1439495_at	RIKEN cDNA 4933407H18 gene	4933407H18Rik	0.63095	-1.16933278	-0.2266241
1447521_x_at	Rho GTPase activating protein 39	Arhgap39	0.63096	1.14875432	0.19370216
1416207_at	tafazzin	Taz	0.63097	1.13229392	0.17837866
1420012_at	X-box binding protein 1	Xbp1	0.63098	-1.16384486	-0.2275861
1440303_at	Solute carrier family 7, member 6 opposite strand	Slc7a6os	0.63098	1.20504757	0.25538757
1422551_at	zinc finger with KRAB and SCAN domains 3	Zkscan3	0.631	-1.16365722	-0.2266622
1453028_at	PWWP domain containing 2A	Pwwp2a	0.63101	-1.1485359	-0.2086586
1425672_a_at	transient receptor potential cation channel, subfamily C, member	Trpc2	0.63104	-1.19410408	-0.2746362
1440699_at	microtubule-associated protein 2	Mtap2	0.63104	1.17630665	0.23229066
1446115_at	---	---	0.63104	-1.17559033	-0.2446193
1423694_at	potassium channel tetramerisation domain containing 10	Kctd10	0.63106	1.12740918	0.17278655
1437465_a_at	prolyl 4-hydroxylase, beta polypeptide	P4hb	0.63106	1.1003151	0.13740305
1416361_a_at	dynein cytoplasmic 1 intermediate chain 1	Dync1i1	0.63107	-1.16435101	-0.2202479
1441763_at	zinc finger, DHHC domain containing 6	Zdhhc6	0.63107	1.19140613	0.24449683
1429590_at	transforming, acidic coiled-coil containing protein 1	Tacc1	0.63107	-1.19792501	-0.2948008
1447052_at	---	---	0.63108	-1.19047479	-0.2733451
1433014_at	RIKEN cDNA 6330436F06 gene	6330436F06Rik	0.63108	-1.15159557	-0.2055614
1442059_at	fragile X mental retardation gene 1, autosomal homolog	Fxr1	0.63109	-1.15058701	-0.2057008
1436415_at	mediator complex subunit 29	Med29	0.63109	1.1833874	0.23788956
1423348_at	frizzled homolog 8 (Drosophila)	Fzd8	0.63109	1.1568802	0.20850831
1459390_at	---	---	0.63109	-1.18768275	-0.2627132
1446956_at	NEDD4 binding protein 1	N4bp1	0.63109	-1.20732101	-0.2896459
1417435_at	like-glycosyltransferase	Large	0.6311	1.13348558	0.18069599
1434807_s_at	metaxin 3	Mtx3	0.6311	-1.15568587	-0.2139457
1456265_at	leucine, glutamate and lysine rich 1	Lekr1	0.6311	-1.16227215	-0.2170828
1438092_x_at	H2A histone family, member Z	H2afz	0.63112	-1.15623306	-0.2312617
1438937_x_at	angiogenin, ribonuclease, RNase A family, 5	Ang	0.63112	1.19460412	0.24654326
1438769_a_at	thymocyte nuclear protein 1	Thyn1	0.63113	1.14426412	0.18971169
1440057_at	hydroxysteroid (17-beta) dehydrogenase 7	Hsd17b7	0.63114	1.15338381	0.20500857
1427989_at	anti-Mullerian hormone type 2 receptor	Amhr2	0.63116	1.2112931	0.2575567
1429433_at	HLA-B associated transcript 2-like 2	Bat2l2	0.63116	-1.1716667	-0.2595537
1422804_at	serine (or cysteine) peptidase inhibitor, clade B, member 6b	Serpinb6b	0.63118	-1.17447577	-0.2360873
1449790_at	---	---	0.63119	1.17036701	0.22039726
1439620_at	carbonic anhydrase 13	Car13	0.63119	-1.12929425	-0.1759998
1454979_at	diaphanous homolog 1 (Drosophila)	Diap1	0.6312	1.11007224	0.14995827
1417379_at	IQ motif containing GTPase activating protein 1	Iqgap1	0.6312	-1.11623277	-0.1587236
1447946_at	a disintegrin and metallopeptidase domain 23	Adam23	0.63121	-1.13118358	-0.1790282
1451854_a_at	shroom family member 3	Shroom3	0.63121	1.13302923	0.18001713
1428323_at	glycerol phosphate dehydrogenase 2, mitochondrial	Gpd2	0.63123	-1.11688062	-0.1612832
1437196_x_at	ribosomal protein S16 /// ribosomal protein S16, pseudogene 2	Rps16 /// Rps16-ps2	0.63124	-1.05260195	-0.0743962
1416218_x_at	ribosomal protein L37a	Rpl37a	0.63124	1.10703855	0.14616956

1417777_at	prostaglandin reductase 1	Ptgr1	0.63124	1.12063148	0.16421606
1459178_at	DNA segment, Chr 8, ERATO Doi 503, expressed	D8Ertd503e	0.63125	-1.17336875	-0.2375505
1423804_a_at	isopentenyl-diphosphate delta isomerase	Idi1	0.63125	-1.15653511	-0.2302768
1439148_a_at	phosphofructokinase, liver, B-type	Pfkl	0.63126	1.10972711	0.14828146
1458899_at	ubiquitin specific peptidase 53	Usp53	0.63127	1.16118373	0.21551209
1452056_s_at	protein phosphatase 3, catalytic subunit, alpha isoform	Ppp3ca	0.63128	-1.14830212	-0.2035251
1423124_x_at	RAD54 like (S. cerevisiae)	Rad54l	0.63129	1.13518888	0.18229397
1427944_at	caprin family member 2	Caprin2	0.6313	1.12191725	0.16412671
1447977_x_at	predicted gene 14430 /// predicted gene 14434	Gm14430 /// Gm14434	0.63131	-1.1412986	-0.199333
1431996_at	predicted gene 4022	Gm4022	0.63131	1.1629421	0.21186832
1417863_at	spermatogenesis associated 19	Spata19	0.63132	1.1541144	0.20653149
1420088_at	nuclear factor of kappa light polypeptide gene enhancer in B-cells	Nfkbia	0.63134	-1.17404258	-0.263174
1451777_at	DEAD (Asp-Glu-Ala-Asp) box polypeptide 60	Ddx60	0.63134	-1.15781811	-0.2215217
1448210_at	RAB1, member RAS oncogene family	Rab1	0.63135	-1.11169255	-0.1539275
1417580_s_at	selenium binding protein 1	Selenbp1	0.63137	1.17470301	0.22783686
1427427_at	ryanodine receptor 3	Ryr3	0.63137	1.22793917	0.2770605
1435035_at	RNA (guanine-9-) methyltransferase domain containing 2	Rg9mtd2	0.63141	1.11572953	0.15656851
1458206_at	---	---	0.63143	-1.16918069	-0.2277439
1426854_a_at	SET nuclear oncogene	Set	0.63143	-1.0782655	-0.1109134
1442434_at	DNA segment, Chr 8, ERATO Doi 82, expressed	D8Ertd82e	0.63147	1.18519499	0.23512585
1418818_at	aquaporin 5	Aqp5	0.63147	-1.16885149	-0.2346037
1443534_at	---	---	0.6315	-1.1678559	-0.2287826
1422765_at	microtubule-associated protein, RP/EB family, member 1	Mapre1	0.63151	-1.17312095	-0.2390094
1434302_at	hypothetical LOC100504603 /// Ras association (RalGDS/AF-6) a	LOC100504603 /// Raph1	0.63153	-1.13148125	-0.1819701
1456135_s_at	paxillin	Pxn	0.63155	-1.11216802	-0.153947
1454551_at	RIKEN cDNA 9530034D02 gene	9530034D02Rik	0.63155	1.1723554	0.22509969
1420078_at	---	---	0.63156	-1.17808385	-0.2475718
1443758_at	WD repeat domain 18	Wdr18	0.63163	1.21411687	0.26282006
1426254_at	TM2 domain containing 1	Tm2d1	0.63165	-1.12569556	-0.1728272
1441391_at	---	---	0.63166	-1.15603127	-0.2214823
1428292_at	NADPH dependent diflavin oxidoreductase 1	Ndor1	0.63168	1.16704681	0.21737485
1456931_at	---	---	0.63169	-1.16947774	-0.2276645
1418721_at	COP9 (constitutive photomorphogenic) homolog, subunit 7b (Aral	Cops7b	0.63171	-1.14775844	-0.2067388
1442609_at	---	---	0.63174	-1.16445923	-0.2232167
1435188_at	predicted gene 129	Gm129	0.63175	1.16598652	0.21185944
1454748_at	nicotinate phosphoribosyltransferase domain containing 1	Naprt1	0.63177	1.13260867	0.17360119
1423195_at	hippocampus abundant gene transcript 1	Hiat1	0.63185	-1.11953776	-0.1647555
1447252_s_at	meprin 1 alpha	Mep1a	0.63194	1.13383201	0.18119827
1434902_at	ring finger protein 157	Rnf157	0.63198	1.13829221	0.18662077
1445056_at	RIKEN cDNA 9230102K24 gene	9230102K24Rik	0.63252	1.16297699	0.21669034
1460096_at	---	---	0.63258	1.20983168	0.2581921
1432218_a_at	nucleolar protein 9	Nol9	0.63299	-1.1348794	-0.1860298
1439616_at	---	---	0.63303	-1.15081595	-0.2098214
1421260_a_at	spermidine synthase	Srm	0.63304	1.09451073	0.12933327
1446807_at	ubiquitin specific peptidase 8	Usp8	0.63315	-1.17723662	-0.2479321
1446208_at	---	---	0.6332	1.14002267	0.18725377
1438144_x_at	ataxin 2	Atxn2	0.63321	1.15242573	0.20129032
1419540_at	ferritin, heavy polypeptide-like 17 /// predicted gene 14499 /// p	Fthl17 /// Gm14499 /// G	0.63329	1.15218642	0.19887315
1426686_s_at	mitogen-activated protein kinase kinase kinase 3	Map3k3	0.6333	-1.16364165	-0.2338631
1420928_at	beta galactoside alpha 2,6 sialyltransferase 1	St6gal1	0.63331	1.15572335	0.20683239
1430147_a_at	TATA box binding protein (Tbp)-associated factor, RNA polymeras	Taf1d	0.63334	-1.1567079	-0.2197894
1427841_at	keratin 14 /// keratin 17	Krt14 /// Krt17	0.63341	1.2343973	0.28156255
1442009_at	eukaryotic translation initiation factor 2C, 3	Eif2c3	0.63343	-1.13005733	-0.182323
1460457_at	RIKEN cDNA 2810405F17 gene	2810405F17Rik	0.63347	-1.15950306	-0.2163328
1420139_s_at	KRR1, small subunit (SSU) processome component, homolog (yea	Krr1	0.6337	-1.11292135	-0.1577027
1422465_a_at	nucleoredoxin	Nxn	0.63397	1.12905561	0.17273169
1447494_at	DNA segment, Chr 7, Brigham & Women's Genetics 0826 express	D7Bwg0826e	0.63427	1.15090184	0.20110338
1424756_at	huntingtin interacting protein 1	Hip1	0.63439	1.15503124	0.20517817
1426882_at	ubiquitin protein ligase E3C	Ube3c	0.63466	1.12886634	0.17119082
1441224_at	dystrophin related protein 2	Drp2	0.63469	-1.15625368	-0.2106627
1442315_at	forkhead box D2	Foxd2	0.63517	-1.17825722	-0.2465211
1435737_a_at	nuclear distribution gene E homolog 1 (A nidulans)	Nde1	0.6352	1.12331407	0.1672719
1455942_at	lysine (K)-specific demethylase 2A	Kdm2a	0.6352	1.12064349	0.16409217
1450662_at	testis specific protein kinase 1	Tesk1	0.63522	1.1635539	0.21263788
1425999_at	predicted gene, EG214403	EG214403	0.63527	-1.12971584	-0.1768146
1448660_at	Rho GDP dissociation inhibitor (GDI) gamma	Arhgdig	0.63528	1.13078809	0.17722815
1426893_at	family with sequence similarity 102, member A	Fam102a	0.63531	1.15939277	0.20848807
1444905_at	DNA segment, Chr 1, ERATO Doi 705, expressed	D1Ertd705e	0.63532	-1.12714688	-0.1748404
1452266_at	LAS1-like (S. cerevisiae)	Las1l	0.63534	1.11241306	0.15246252
1448396_at	transmembrane protein 131	Tmem131	0.63538	1.12381302	0.16459666
1422977_at	glycoprotein Ib, beta polypeptide	Gp1bb	0.63545	-1.15041745	-0.2049909
1424148_a_at	signal transducing adaptor family member 2	Stap2	0.63547	1.14734677	0.19455664
1434507_at	aminopeptidase-like 1	Npepl1	0.6355	1.1294545	0.17297243
1459274_at	G protein-coupled receptor 135	Gpr135	0.63551	1.16188527	0.21452163
1437878_s_at	tetratricopeptide repeat domain 14	Ttc14	0.63553	-1.14355324	-0.1989888
1460600_at	ubiquitin-conjugating enzyme E2 Q2-like	LOC634012	0.63553	1.17480452	0.223926
1418588_at	neurensin 1	Nrsn1	0.63555	1.15761151	0.21065703
1443131_at	low density lipoprotein-related protein 1B (deleted in tumors)	Lrp1b	0.63558	1.10679784	0.14176443
1417145_at	nuclear transcription factor, X-box binding-like 1	Nfxl1	0.63559	1.14064401	0.18486349
1450859_s_at	ubiquitin-conjugating enzyme E2D 3 (UBC4/5 homolog, yeast) ps	Gm4596 /// Ube2d3	0.63561	-1.11033492	-0.1514098
1456597_at	HEAT repeat containing 3	Heatr3	0.63562	-1.12804076	-0.1775103
1451818_at	mindbomb homolog 1 (Drosophila)	Mib1	0.63562	1.15570383	0.20553192
1415781_a_at	predicted gene 13430 /// SMT3 suppressor of mif two 3 homolog	Gm13430 /// Sumo2	0.63562	-1.05832831	-0.0821834
1428038_at	predicted gene 568	Gm568	0.63562	1.21141788	0.26442296
1431200_a_at	DPH3 homolog (KTI11, S. cerevisiae)	Dph3	0.63565	1.15325517	0.20564403
1427067_at	RIKEN cDNA 4933439F18 gene	4933439F18Rik	0.63567	-1.13367604	-0.1839035
1420283_at	PHD finger protein 14	Phf14	0.63569	1.12331111	0.1638666
1446694_at	---	---	0.63569	-1.169127	-0.2255681
1441652_at	bone morphogenic protein receptor, type II (serine/threonine kina	Bmpr2	0.63574	1.19103088	0.24253613
1430424_at	RIKEN cDNA 4933433C11 gene	4933433C11Rik	0.63575	-1.15049954	-0.2028686
1438420_at	RNA binding motif protein 39	Rbm39	0.63576	-1.18297317	-0.2769373
1449987_at	anaplastic lymphoma kinase	Alk	0.63578	-1.16594974	-0.2261235
1440602_at	---	---	0.63578	1.13696242	0.18504656
1456045_at	RIKEN cDNA 1700106N22 gene	1700106N22Rik	0.63581	-1.14723592	-0.1989429

1443164_at	---	---	0.63582	1.16722277	0.21816562
1422975_at	membrane metallo endopeptidase	Mme	0.63582	-1.14869764	-0.2025628
1452865_at	leucine rich repeat containing 27	Lrrc27	0.63584	1.16524453	0.2142374
1422068_at	POU domain, class 3, transcription factor 1	Pou3f1	0.6359	1.14564014	0.19564272
1417739_at	mediator of RNA polymerase II transcription, subunit 11 homolog	Med11	0.63594	1.14017291	0.18332472
1448505_at	C1D nuclear receptor co-repressor	C1d	0.63596	-1.11179079	-0.1550015
1439511_at	cyclin-dependent kinase 7	Cdk7	0.63597	-1.23324942	-0.3712577
1419234_at	helicase (DNA) B	Helb	0.63597	1.14871315	0.1925629
1460049_s_at	RIKEN cDNA 1500015O10 gene	1500015O10Rik	0.636	1.16163392	0.21295206
1429748_at	---	---	0.63611	1.14432558	0.19359266
1424945_at	chordin-like 1	Chrdl1	0.63612	1.16437849	0.21230519
1454791_a_at	retinoblastoma binding protein 4	Rbbp4	0.63615	-1.12359253	-0.1755346
1458146_at	DENN/MADD domain containing 5B	Dennd5b	0.63616	-1.18815941	-0.264624
1420425_at	PR domain containing 1, with ZNF domain	Prdm1	0.63616	1.17944396	0.22838751
1446137_at	glioblastoma amplified sequence	Gbas	0.63617	1.18230112	0.23333216
1422451_at	mitochondrial ribosomal protein S21	Mrps21	0.63617	1.13851779	0.17930545
1438773_at	six transmembrane epithelial antigen of prostate 2	Steap2	0.63619	-1.15287173	-0.2138392
1451233_at	TNF receptor-associated factor 2	Traf2	0.63624	1.13356928	0.17944688
1453054_at	secretory carrier membrane protein 1	Scamp1	0.63631	-1.16287757	-0.2243713
1420200_at	---	---	0.63633	1.18143841	0.22448961
1453737_at	WAS/WASL interacting protein family, member 2	Wipf2	0.63637	-1.18870382	-0.2713866
1416093_a_at	mitochondrial ribosomal protein L20	Mrpl20	0.63638	1.10565326	0.14398681
1449217_at	caspase 8 associated protein 2	Casp8ap2	0.63647	-1.10745075	-0.1473862
1421416_at	mitogen-activated protein kinase kinase 7	Map2k7	0.6365	1.15792382	0.21140978
1445499_at	zinc finger CCCH type containing 13	Zc3h13	0.63651	-1.16337739	-0.2288546
1458564_at	---	---	0.63658	-1.16115455	-0.2168153
1416813_at	cytotoxic granule-associated RNA binding protein 1	Tia1	0.63663	1.1290529	0.17241678
1450818_a_at	NADH dehydrogenase (ubiquinone) 1 alpha subcomplex, 7 (B14.5	Ndufa7	0.63664	1.09627609	0.13019255
1429005_at	malignant fibrous histiocytoma amplified sequence 1	Mfhas1	0.63668	1.21669631	0.26504041
1417275_at	myelin and lymphocyte protein, T-cell differentiation protein	Mal	0.63669	1.17634104	0.22755007
1448702_at	immediate early response 3 interacting protein 1	Ier3ip1	0.63669	1.12834838	0.16923206
1431753_x_at	ubiquitin related modifier 1 homolog (S. cerevisiae)	Urm1	0.63673	1.12622529	0.16908915
1446850_at	---	---	0.63678	1.17540655	0.23082658
1439022_at	phosphatase and actin regulator 1	Phactr1	0.63679	1.19763645	0.2494571
1452425_at	tumor necrosis factor receptor superfamily, member 14 (herpesvi	Tnfrsf14	0.6368	-1.17398948	-0.2391601
1416179_a_at	radixin	Rdx	0.63681	-1.17621049	-0.2638216
1431394_a_at	leucine-rich repeat kinase 2	Lrrk2	0.63691	1.19569954	0.24656511
1434070_at	jagged 1	Jag1	0.63699	1.16258866	0.20629046
1458352_at	Ligand dependent nuclear receptor corepressor-like	Lcorl	0.63703	1.2005577	0.25091426
1453684_s_at	zinc finger CCCH-type containing 15	Zc3h15	0.63711	-1.10853277	-0.1497613
1455360_at	solute carrier family 38, member 9	Slc38a9	0.63714	1.1734144	0.22466332
1434078_at	ubiquitin family domain containing 1	Ubfd1	0.63784	1.1046756	0.14325785
1436223_at	integrin beta 8	Itgb8	0.63787	1.11853558	0.16091457
1457899_at	kalirin, RhoGEF kinase	Kalrn	0.63798	1.16597196	0.21965902
1460500_at	RIKEN cDNA 5033421C21 gene	5033421C21Rik	0.63801	1.20121419	0.25152772
1436648_at	nanos homolog 1 (Drosophila)	Nanos1	0.63804	1.16592963	0.21972246
1458496_at	---	---	0.63805	1.18875153	0.23868283
1415975_at	calcium regulated heat stable protein 1	Carhsp1	0.63809	1.12196654	0.16549844
1437433_at	UDP-Gal:betaGlcNAc beta 1,3-galactosyltransferase, polypeptide	B3galt2	0.6381	1.10407518	0.1425136
1451540_at	mannose phosphate isomerase	Mpi	0.63812	1.1166505	0.1581359
1427838_at	tubulin, beta 2A	Tubb2a	0.63813	-1.16161986	-0.2185878
1443522_s_at	pleckstrin homology domain interacting protein	Phip	0.63814	-1.15288269	-0.2104379
1424680_at	family with sequence similarity 26, member E	Fam26e	0.63818	1.13461737	0.17632979
1418135_at	AF4/FMR2 family, member 1	Aff1	0.6383	-1.12699962	-0.1758941
1439785_at	RIKEN cDNA 9630013A20 gene	9630013A20Rik	0.63832	1.08869436	0.12012765
1435327_at	lysophosphatidylglycerol acyltransferase 1	Lpgat1	0.63838	-1.15878145	-0.2221845
1438058_s_at	prostate tumor over expressed gene 1	Ptov1	0.63846	1.1854378	0.22645971
1420824_at	sema domain, immunoglobulin domain (Ig), transmembrane dom	Sema4d	0.63852	1.14586996	0.18968358
1416523_at	ribonuclease, RNase A family, 1 (pancreatic)	Rnase1	0.63868	-1.16371773	-0.2190984
1432970_at	RIKEN cDNA 4933423K11 gene	4933423K11Rik	0.63871	-1.15977722	-0.2140913
1434664_at	mitotic spindle organizing protein 1	Mzt1	0.63874	-1.12681233	-0.1788823
1442365_at	---	---	0.63875	1.20567877	0.25362267
1417890_at	pyridoxal (pyridoxine, vitamin B6) phosphatase	Pdxp	0.63878	1.11395788	0.15464451
1416530_a_at	purine-nucleoside phosphorylase	Pnp	0.6388	1.08956981	0.12350059
1435273_at	tryptophanyl tRNA synthetase 2 (mitochondrial)	Wars2	0.63882	-1.15118038	-0.2033244
1456089_at	tripartite motif-containing 23	Trim23	0.63884	-1.17267601	-0.2460236
1417612_at	immediate early response 5	Ier5	0.63886	1.14630414	0.19518294
1434256_s_at	CDP-diacylglycerol synthase (phosphatidate cytidylyltransferase)	Cds2	0.63888	1.12311562	0.1632659
1423115_at	ST6 (alpha-N-acetyl-neuraminyl-2,3-beta-galactosyl-1,3)-N-acety	St6galnac6	0.63902	1.13639303	0.18181118
1417069_a_at	glia maturation factor, beta	Gmfb	0.63903	-1.22197968	-0.343604
1428141_at	golgi associated, gamma adaptin ear containing, ARF binding prot	Gga2	0.63921	1.10054098	0.13794314
1433521_at	ankyrin repeat domain 13c	Ankrd13c	0.64045	-1.10797104	-0.1499291
1460650_at	ATPase, H+ transporting, lysosomal V0 subunit A1	Atp6v0a1	0.64046	1.16363857	0.21300967
1419853_a_at	purinergic receptor P2X, ligand-gated ion channel, 7	P2rx7	0.64056	1.17089324	0.21929679
1437552_at	RIKEN cDNA 2410127L17 gene	2410127L17Rik	0.64062	-1.12153097	-0.1688274
1418188_a_at	metastasis associated lung adenocarcinoma transcript 1 (non-cod	Malat1	0.64071	-1.26574002	-0.426812
1449565_at	cytochrome P450, family 2, subfamily g, polypeptide 1	Cyp2g1	0.64082	-1.15367791	-0.2108465
1417460_at	interferon induced transmembrane protein 2	Ifitm2	0.64082	-1.09282096	-0.1306333
1421255_a_at	calcium binding protein 1	Cabp1	0.6409	1.17721688	0.22262164
1437172_x_at	hydroxyacyl-Coenzyme A dehydrogenase/3-ketoacyl-Coenzyme	Hadhb	0.64091	-1.14136217	-0.2034874
1442786_s_at	RUN and FYVE domain containing 3	Rufy3	0.64097	-1.19495155	-0.2802031
1435740_at	predicted gene 10397 /// zinc finger, MIZ-type containing 1	Gm10397 /// Zmiz1	0.64097	-1.12089564	-0.1695777
1428585_at	actinin, alpha 1	Actn1	0.64098	-1.11944635	-0.1687794
1450541_at	plasmacytoma variant translocation 1	Pvt1	0.64099	-1.19844854	-0.2860667
1445148_at	---	---	0.641	-1.15218535	-0.2061673
1426408_at	CUGBP, Elav-like family member 1	Celf1	0.64103	-1.12240241	-0.1737225
1419166_at	solute carrier family 5 (sodium/glucose cotransporter), member 2	Slc5a2	0.64104	-1.15750596	-0.2220348
1437040_at	ethanolamine kinase 2	Etnk2	0.64105	1.13663636	0.18332217
1418506_a_at	peroxiredoxin 2	Prdx2	0.64105	1.09877478	0.13542743
1454754_a_at	angio-associated migratory protein	Aamp	0.64105	1.10321832	0.14086036
1422670_at	Rho family GTPase 2	Rnd2	0.64108	1.12168505	0.16416086
1439028_at	Ubiquitin-fold modifier 1	Ufm1	0.64121	-1.1777515	-0.2532069
1428696_at	raftlin lipid raft linker 1	Rftn1	0.64123	1.14697112	0.19747748
1448241_at	GM2 ganglioside activator protein	Gm2a	0.64126	-1.12659375	-0.176724

1440253_at	proteasome (prosome, macropain) 26S subunit, non-ATPase, 11	Psmd11	0.6418	-1.13292644	-0.1925189
1425631_at	protein phosphatase 1, regulatory (inhibitor) subunit 3C	Ppp1r3c	0.64191	1.16422573	0.21833028
1453006_at	fibroblast growth factor binding protein 3	Fgfbp3	0.64203	1.20772687	0.25959712
1447632_at	Fanconi anemia, complementation group C	Fancc	0.64204	-1.14493275	-0.195691
1446568_at	---	---	0.64208	-1.16436316	-0.2208069
1426667_a_at	Sad1 and UNC84 domain containing 1	Sun1	0.64218	1.12088041	0.16421631
1420418_at	synaptotagmin II	Syt2	0.64237	1.15383075	0.20596772
1458498_at	---	---	0.64238	1.14446189	0.18990016
1454555_at	zinc finger protein 572	Zfp572	0.64272	-1.16468873	-0.222267
1443923_at	A kinase (PRKA) anchor protein 13	Akap13	0.64273	-1.20482554	-0.2959064
1436676_at	mitogen-activated protein kinase 8 interacting protein 3	Mapk8ip3	0.64282	1.16356372	0.21715843
1455277_at	Hedgehog-interacting protein	Hhip	0.64326	1.19470013	0.24616058
1419680_a_at	elaC homolog 2 (E. coli)	Elac2	0.64338	1.12325037	0.16604934
1424045_at	RIKEN cDNA 5730437N04 gene	5730437N04Rik	0.64352	1.15553173	0.19845695
1448013_at	ubiquitin specific peptidase 24	Usp24	0.64354	-1.13906096	-0.1965206
1431891_at	zinc finger, SWIM domain containing 5	Zswim5	0.64354	1.16742414	0.22135074
1457194_at	---	---	0.64366	1.15656681	0.20551634
1430726_at	Ras association (RalGDS/AF-6) domain family (N-terminal) memb	Rassf8	0.64383	-1.18696609	-0.2689875
1446426_at	---	---	0.64388	-1.16309402	-0.2276888
1432066_at	DnaJ (Hsp40) homolog, subfamily A, member 3	Dnaja3	0.6439	-1.1547257	-0.2093177
1441653_at	Snf2-related CREBBP activator protein	Srcap	0.64393	-1.20694871	-0.3041269
1453997_a_at	nestin	Nes	0.64396	1.15412654	0.20420674
1433813_at	transmembrane protein 48	Tmem48	0.64397	1.11665984	0.15797988
1452263_at	solute carrier family 35, member F4	Slc35f4	0.64401	1.14784292	0.19639244
1442259_at	---	---	0.64401	1.18866923	0.23906218
1435605_at	ARP3 actin-related protein 3 homolog B (yeast)	Actr3b	0.64402	1.13699372	0.18108269
1417760_at	nuclear receptor subfamily 0, group B, member 1	Nr0b1	0.64402	-1.10254607	-0.1422381
1447477_at	---	---	0.64402	1.20330502	0.25275966
1455602_x_at	RIKEN cDNA 1110028C15 gene	1110028C15Rik	0.64403	-1.21913853	-0.3275719
1423031_at	macrophage erythroblast attacher	Maea	0.64404	1.12967885	0.17124029
1456549_at	predicted gene 550	Gm550	0.64406	-1.16005672	-0.2151877
1429722_at	zinc finger and BTB domain containing 4	Zbtb4	0.64407	-1.17526927	-0.2390194
1455466_at	G protein-coupled receptor 133	Gpr133	0.64408	1.14120501	0.18815903
1417643_at	radial spoke head 1 homolog (Chlamydomonas)	Rsph1	0.6441	1.15917973	0.21120074
1427235_at	4lysine (K)-specific demethylase 6A	Kdm6a	0.64412	-1.12862314	-0.1777997
1447503_at	cDNA sequence BC016495	BC016495	0.64413	1.16287285	0.21558573
1437565_a_at	guanine nucleotide binding protein-like 2 (nucleolar) /// hypothet	Gnl2 /// LOC100505087	0.64415	-1.15904507	-0.2314948
1427434_at	NLR family, apoptosis inhibitory protein 6	Naip6	0.64416	-1.15230778	-0.2054155
AFFX-BioC-5_at	---	---	0.64417	1.18018206	0.22274189
1424142_at	inhibitor of kappa light polypeptide enhancer in B-cells, kinase cor	Ikbkap	0.64418	-1.14613193	-0.205062
1448346_at	cofilin 1, non-muscle	Cfl1	0.64418	1.10626764	0.14162824
1428554_a_at	RIKEN cDNA 1810035L17 gene	1810035L17Rik	0.64421	1.13091153	0.17043036
1452386_at	sal-like 3 (Drosophila)	Sall3	0.64423	1.14049563	0.18872465
1455693_x_at	predicted gene 13654 /// ribosomal protein S6 pseudogene /// ri	Gm13654 /// Gm16409 //	0.64424	-1.05366927	-0.0762977
1443659_at	---	---	0.64426	1.10217522	0.13673136
1426878_at	polybromo 1	Pbrm1	0.64427	-1.1387567	-0.195916
1436199_at	tripartite motif-containing 14	Trim14	0.64427	1.1775213	0.22952907
1423453_at	nucleolar protein 12	Nol12	0.6443	1.13134378	0.17195914
1451158_at	thyroid hormone receptor interactor 12	Trip12	0.64431	-1.16196272	-0.2389668
1438984_x_at	proteasome (prosome, macropain) subunit, beta type 4	Psmb4	0.64431	-1.13826681	-0.2046819
1454432_at	RIKEN cDNA 4933423N03 gene	4933423N03Rik	0.64433	-1.15745387	-0.2131516
1421656_at	sprouty homolog 2 (Drosophila)	Spry2	0.64433	-1.19449447	-0.2875874
1443900_at	chromodomain helicase DNA binding protein 8	Chd8	0.64434	1.16075652	0.21282952
1421750_a_at	von Hippel-Lindau binding protein 1	Vbp1	0.64435	-1.0963558	-0.1331364
1436502_at	mitochondrial tumor suppressor 1	Mtus1	0.64436	1.16255887	0.21098681
1458954_at	Predicted gene 13308	Gm13308	0.64436	-1.16323951	-0.2242745
1420825_at	leucine zipper-EF-hand containing transmembrane protein 1	Letm1	0.64437	1.1405784	0.18957793
1431792_a_at	serine/threonine kinase 11 interacting protein	Stk11ip	0.64437	-1.15035831	-0.2108187
1437574_at	a disintegrin-like and metallopeptidase (reprolysin type) with thro	Adamts18	0.64439	-1.14570427	-0.199451
1422433_s_at	isocitrate dehydrogenase 1 (NADP+), soluble	Idh1	0.6444	1.10475878	0.14321295
1440705_at	expressed sequence AU021720	AU021720	0.64443	1.16994058	0.21249068
1458667_at	ninein-like	Ninl	0.64445	-1.2411247	-0.3920817
1449450_at	prostaglandin E synthase	Ptges	0.64445	1.13499963	0.17933684
1455261_at	Luc7 homolog (S. cerevisiae)-like	Luc7l	0.64446	1.1158967	0.15813672
1455457_at	cytochrome P450, family 2, subfamily c, polypeptide 54	Cyp2c54	0.64446	-1.16158131	-0.2176818
1453286_at	plexin A2	Plxna2	0.64446	1.15357046	0.20339206
1431573_at	spermidine/spermine N1-acetyl transferase-like 1	Satl1	0.64447	-1.17153671	-0.2378693
1443064_at	RIKEN cDNA 9130004C02 gene	9130004C02Rik	0.64448	-1.14204397	-0.1950296
1418394_a_at	CD97 antigen	Cd97	0.6445	1.11881243	0.16073234
1418905_at	nucleotide binding protein 1	Nubp1	0.6445	1.10371871	0.14152744
1439765_x_at	keratin 42	Krt42	0.64451	1.10149531	0.13911437
1453949_s_at	lysophospholipase 1	Lypla1	0.64453	-1.18667217	-0.284927
1415715_at	transmembrane protein 129	Tmem129	0.64454	1.14041041	0.18950094
1422466_at	nucleoredoxin	Nxn	0.64454	1.15146851	0.20216104
1432653_at	---	---	0.64454	1.12763686	0.17010769
1447615_at	---	---	0.64455	1.1306588	0.17647548
1444439_at	Bri3 binding protein	Bri3bp	0.64459	-1.17446638	-0.2452772
1441083_at	cleft lip and palate associated transmembrane protein 1	Clptm1	0.6446	1.16504429	0.21571434
1450939_at	ectonucleoside triphosphate diphosphohydrolase 1	Entpd1	0.64461	1.1450545	0.19373797
1428948_at	potassium large conductance calcium-activated channel, subfami	Kcnma1	0.64462	1.17740754	0.22714117
1417697_at	sterol O-acyltransferase 1	Soat1	0.64463	1.16276867	0.21119333
1434520_at	sterol-C5-desaturase (fungal ERG3, delta-5-desaturase) homolog	Sc5d	0.64463	1.17190703	0.21989391
1433793_s_at	nuclear receptor interacting protein 2	Nrip2	0.64464	-1.14540685	-0.1965756
1445596_at	---	---	0.64464	1.17295435	0.21065267
1458655_at	---	---	0.64465	1.18710271	0.23455343
1453205_at	DPY30 domain containing 1	Dydc1	0.64467	1.12770743	0.17281813
1445884_at	---	---	0.64467	1.18402157	0.23118937
1417489_at	neuropeptide Y receptor Y2	Npy2r	0.64468	1.13812341	0.18402666
1449500_at	serine (or cysteine) peptidase inhibitor, clade B, member 7	Serpinb7	0.64468	-1.12649694	-0.1729208
1418791_at	SH3-domain GRB2-like 2	Sh3gl2	0.64469	-1.13869621	-0.199376
1454549_at	RIKEN cDNA 2900005I04 gene	2900005I04Rik	0.64469	-1.15876172	-0.2244728
1424071_s_at	cDNA sequence BC018507	BC018507	0.6447	-1.10696232	-0.1466709
1450268_at	fidgetin	Fign	0.64471	1.16675413	0.22205695
1443949_at	---	---	0.64472	1.19237503	0.24244658

1419140_at	activin receptor IIB	Acvr2b	0.64472	1.15672047	0.20025182
1450987_a_at	RIKEN cDNA 2310004I24 gene	2310004I24Rik	0.64473	1.11995738	0.16170096
1442179_at	RIKEN cDNA 9430053O09 gene	9430053O09Rik	0.64473	-1.11595285	-0.1591172
1424227_at	polymerase (RNA) III (DNA directed) polypeptide H	Polr3h	0.64476	1.10319324	0.14106043
1441738_at	---	---	0.64476	-1.16268135	-0.2220535
1443112_at	apoptosis inhibitor 5	Api5	0.64478	1.21355601	0.26410284
1451399_at	paroxysmal nonkinesiogenic dyskinesia	Pnkd	0.6448	1.14270828	0.1833219
1422054_a_at	SKI-like	Skil	0.64481	1.14831724	0.19659582
1447728_x_at	heat shock protein 9	Hspa9	0.64482	-1.10339329	-0.1424068
1452533_at	ryanodine receptor 3	Ryr3	0.64483	1.15624512	0.20819435
1460328_at	bromodomain containing 3	Brd3	0.64483	-1.12063905	-0.1673339
1437039_at	COP9 (constitutive photomorphogenic) homolog, subunit 2 (Arabi	Cops2	0.64484	-1.10666629	-0.1474188
1455824_x_at	STT3, subunit of the oligosaccharyltransferase complex, homolog	Stt3a	0.64484	-1.1188599	-0.1690563
1420650_at	zinc finger homeobox 3	Zfhx3	0.64484	-1.20690286	-0.3074899
1427549_s_at	keratin associated protein 16-10 /// keratin associated protein 16	Krtap16-10 /// Krtap16-6	0.64485	1.13708177	0.17967528
1416096_at	VPS33B interacting protein, apical-basolateral polarity regulator	Vipar	0.64485	1.11470207	0.15586511
1459311_at	phosphodiesterase 4D, cAMP specific	Pde4d	0.64486	1.17617155	0.22070156
1420443_at	protocadherin beta 19	Pcdhb19	0.64488	-1.1689346	-0.2285707
1451178_at	coiled-coil domain containing 142 /// mitochondrial ribosomal pro	Ccdc142 /// Mrpl53	0.64488	1.11876051	0.15992736
1442707_at	calcium/calmodulin-dependent protein kinase II alpha	Camk2a	0.64489	-1.16619742	-0.2242814
1416465_a_at	vesicle-associated membrane protein, associated protein A	Vapa	0.6449	-1.09674143	-0.1334983
1428547_at	5' nucleotidase, ecto	Nt5e	0.6449	-1.17016453	-0.2430277
1429314_at	synaptotagmin XI	Syt11	0.6449	1.14854953	0.19665962
1425217_a_at	synaptojanin 2	Synj2	0.6449	1.14427395	0.1922335
1416688_at	synaptosomal-associated protein 91	Snap91	0.64491	1.18866842	0.23722055
1447527_at	---	---	0.64491	1.15686917	0.20779618
1423473_at	septin 2	Sep-02	0.64492	1.3097088	0.34059592
1457635_s_at	nuclear receptor subfamily 3, group C, member 1	Nr3c1	0.64492	-1.20417287	-0.2989385
1441567_at	---	---	0.64493	-1.2048198	-0.2915908
1427049_s_at	smoothened homolog (Drosophila)	Smo	0.64493	1.13067049	0.17602537
1415696_at	SAR1 gene homolog A (S. cerevisiae)	Sar1a	0.64494	1.11795737	0.15881865
1439395_at	zinc finger protein 874b	Zfp874b	0.64494	1.15986445	0.21249964
1447940_a_at	Braf transforming gene	Braf	0.64495	-1.14014429	-0.1900425
1440785_at	relaxin/insulin-like family peptide receptor 1	Rxfp1	0.64497	1.15032357	0.19979841
1430740_at	predicted gene 11651	Gm11651	0.64498	-1.15430312	-0.2071544
1458659_at	predicted gene 10393 /// placenta specific 9	Gm10393 /// Plac9	0.64498	-1.19516521	-0.2744601
1434402_at	sterile alpha motif domain containing 8	Samd8	0.64499	-1.13754091	-0.1906931
1420372_at	syntrophin, basic 2	Sntb2	0.64499	-1.16765852	-0.2507917
1442804_at	Gardner-Rasheed feline sarcoma viral (Fgr) oncogene homolog	Fgr	0.64499	1.14523428	0.19449691
1449587_a_at	proline rich, lacrimal 1	Prol1	0.64499	1.13333077	0.17857247
1458785_at	---	---	0.645	-1.16011593	-0.2167277
1427942_at	Ras association (RalGDS/AF-6) domain family (N-terminal) memb	Rassf9	0.64501	1.17542205	0.22592385
1417161_at	CDK2-associated protein 2	Cdk2ap2	0.64501	1.14548923	0.18651024
1441108_at	---	---	0.64501	-1.16231986	-0.221208
1425807_at	cDNA sequence BC021891	BC021891	0.64502	-1.16562628	-0.228902
1454557_at	RIKEN cDNA 6720454L07 gene	6720454L07Rik	0.64502	-1.16856389	-0.2279881
1434643_at	transducin (beta)-like 1 X-linked	Tbl1x	0.64503	-1.142063	-0.2036412
1417049_at	Rh blood group, D antigen	Rhd	0.64506	1.13244799	0.1781384
1460196_at	carbonyl reductase 1	Cbr1	0.64506	1.11394965	0.15435313
1441705_at	expressed sequence AU015680	AU015680	0.64507	-1.16541407	-0.2243697
1447520_at	lipopolysaccharide binding protein	Lbp	0.64508	-1.16037854	-0.2172806
1456319_at	---	---	0.64508	1.18046697	0.22986243
1441912_x_at	complement component 2 (within H-2S)	C2	0.64509	-1.13526667	-0.1848363
1456088_at	X-linked inhibitor of apoptosis	Xiap	0.64509	-1.16607185	-0.2334409
1426975_at	amplified in osteosarcoma	Os9	0.6451	-1.14612114	-0.1990977
1434362_at	---	---	0.64511	1.15831243	0.20675259
1454862_at	pleckstrin homology-like domain, family B, member 2	Phldb2	0.64511	1.1546413	0.20730851
1446097_at	Retinoblastoma binding protein 8	Rbbp8	0.64512	-1.14072475	-0.1933209
1431710_at	CKLF-like MARVEL transmembrane domain containing 1	Cmtm1	0.64512	-1.15369935	-0.2067248
1435344_at	transcription factor Dp 2	Tfdp2	0.64514	-1.12840746	-0.1787324
1437543_at	far upstream element (FUSE) binding protein 1	Fubp1	0.64514	-1.19334575	-0.2815698
1420558_at	selectin, platelet	Selp	0.64515	-1.16578268	-0.2240297
1456536_at	THAP domain containing 6	Thap6	0.64517	-1.18598365	-0.2608634
1435544_at	alanyl-tRNA synthetase /// exosome component 6	Aars /// Exosc6	0.64517	1.1319967	0.17189774
1450491_at	prostaglandin E receptor 1 (subtype EP1)	Ptger1	0.64518	-1.14551315	-0.2002713
1444842_at	lysine (K)-specific demethylase 5A	Kdm5a	0.64518	-1.19986447	-0.2853724
1429943_at	chitobiase, di-N-acetyl-	Ctbs	0.64519	-1.15966152	-0.2177984
1434772_at	adenosine A2b receptor	Adora2b	0.64521	-1.17229013	-0.2378433
1424699_at	coiled-coil domain containing 136	Ccdc136	0.64521	1.12842562	0.17154763
1423154_at	cDNA sequence BC005537	BC005537	0.64522	-1.14786289	-0.2015807
1426205_at	protein phosphatase 1, catalytic subunit, beta isoform	Ppp1cb	0.64524	-1.102335	-0.1428087
1452276_at	SWI/SNF-related, matrix-associated actin-dependent regulator of	Smarcad1	0.64524	-1.11652635	-0.164
1431107_at	serine/threonine kinase 35	Stk35	0.64524	-1.13340058	-0.1825011
1426852_x_at	nephroblastoma overexpressed gene	Nov	0.64526	1.21710537	0.26785299
1449691_at	Zinc finger protein 644	Zfp644	0.64527	-1.21056761	-0.3055969
1452758_s_at	eukaryotic translation initiation factor 4, gamma 2	Eif4g2	0.64528	-1.07368025	-0.104863
1456059_at	proteasome (prosome, macropain) 26S subunit, non-ATPase, 11	Psmd11	0.64529	1.12635994	0.16705491
1445897_s_at	interferon-induced protein 35	Ifi35	0.64529	1.19846388	0.24542085
1419950_s_at	transportin 3	Tnpo3	0.64531	-1.15160305	-0.2230874
1455260_at	ligand dependent nuclear receptor corepressor-like	Lcorl	0.64531	-1.16117871	-0.2212524
1460213_at	golgi autoantigen, golgin subfamily a, 4	Golga4	0.64536	1.119899	0.16313423
1421037_at	neuronal PAS domain protein 2	Npas2	0.64536	-1.15450324	-0.2189326
1431287_at	pericentriolar material 1	Pcm1	0.64543	-1.15311524	-0.2143417
1425118_at	spire homolog 2 (Drosophila)	Spire2	0.64557	1.22915704	0.26407792
1429450_at	RIKEN cDNA 1700026J04 gene	1700026J04Rik	0.64558	-1.13278503	-0.1809128
1433138_at	---	---	0.64567	-1.1747723	-0.2412493
1437289_at	inositol monophosphatase domain containing 1	Impad1	0.64618	-1.13890737	-0.196779
1454213_at	armadillo repeat containing 9	Armc9	0.64644	1.15748746	0.21081176
1451142_at	exocyst complex component 1	Exoc1	0.64653	1.12054095	0.16353678
1420988_at	polymerase (DNA directed), eta (RAD 30 related)	Polh	0.64661	1.15211676	0.201856
1423611_at	alkaline phosphatase, liver/bone/kidney	Alpl	0.64661	-1.11296278	-0.1576397
1437830_x_at	zinc finger, BED domain containing 3	Zbed3	0.64675	-1.16142881	-0.2240021
1448813_at	arylacetamide deacetylase (esterase)	Aadac	0.64679	1.17941974	0.2300807
1427292_at	immunoglobulin lambda chain, variable 1	Igl-V1	0.64687	1.1581136	0.20574432

1447100_s_at	RIKEN cDNA 5730508B09 gene	5730508B09Rik	0.64689	-1.15549583	-0.2119702
1445958_at	---	---	0.64695	-1.15050088	-0.2023057
1435817_x_at	predicted gene 13654 /// ribosomal protein S6 pseudogene /// 4	Gm13654 /// Gm16409 //	0.64704	-1.11836449	-0.166851
1442044_at	---	---	0.64705	1.19327353	0.24225358
1453765_at	leucine-rich repeat-containing G protein-coupled receptor 4	Lgr4	0.64715	-1.16088764	-0.215231
1417862_at	family with sequence similarity 181, member B	Fam181b	0.64716	1.16469611	0.21545668
1452866_at	asparaginyl-tRNA synthetase	Nars	0.64719	-1.09596473	-0.1324389
1419760_a_at	RWD domain containing 2B	Rwdd2b	0.64728	1.12260887	0.16682018
1421326_at	colony stimulating factor 2 receptor, beta, low-affinity (granulocyt	Csf2rb	0.64728	-1.16895329	-0.2359256
1424589_s_at	RNA-binding region (RNP1, RRM) containing 3	Rnpc3	0.64731	-1.15319085	-0.221226
1423287_at	cerebellin 1 precursor protein	Cbln1	0.64747	1.17604354	0.23001293
1434112_at	latrophilin 2	Lphn2	0.64753	-1.13110946	-0.1792812
1429977_at	RIKEN cDNA 9030425L15 gene	9030425L15Rik	0.64755	-1.15111045	-0.2037792
1417027_at	tripartite motif-containing 2	Trim2	0.64758	1.133399	0.1782429
1422205_at	SRY-box containing gene 1	Sox1	0.64763	1.15868924	0.21114194
1453299_a_at	purine-nucleoside phosphorylase /// purine-nucleoside phosphor	Pnp /// Pnp2	0.64768	1.10158239	0.13954521
1423730_at	CLPTM1-like	Clptm1l	0.64772	1.1039242	0.14208918
1424711_at	transmembrane protein 2	Tmem2	0.64775	1.15033196	0.19065176
1417192_at	translocase of outer mitochondrial membrane 70 homolog A (yea	Tomm70a	0.64777	-1.10107484	-0.1399596
1457304_at	DNA segment, Chr 13, ERATO Doi 787, expressed	D13Ertd787e	0.64782	-1.24349742	-0.4003498
1452840_at	RIKEN cDNA 1500009L16 gene	1500009L16Rik	0.6479	1.11594599	0.15688699
1416253_at	cyclin-dependent kinase inhibitor 2D (p19, inhibits CDK4) /// pred	Cdkn2d /// Gm4694	0.64801	1.1232162	0.16659587
1434096_at	solute carrier family 4 (anion exchanger), member 4	Slc4a4	0.64803	-1.14428804	-0.1946091
1423985_at	guanine nucleotide binding protein (G protein), gamma 5	Gng5	0.64808	1.09674234	0.12990586
1427192_a_at	bromodomain containing 8	Brd8	0.64809	1.11316269	0.1525905
1449651_x_at	Predicted gene 9222	Gm9222	0.64809	-1.15463269	-0.208395
1451986_s_at	leucine-rich repeat kinase 1	Lrrk1	0.64813	1.16651996	0.22195687
1426888_at	euchromatic histone lysine N-methyltransferase 2	Ehmt2	0.64813	-1.13721899	-0.1929384
1427229_at	3-hydroxy-3-methylglutaryl-Coenzyme A reductase	Hmgcr	0.64814	-1.1314694	-0.1843521
1435143_at	ELK3, member of ETS oncogene family	Elk3	0.64819	1.17124394	0.22127107
1436405_at	dedicator of cytokinesis 4	Dock4	0.64819	-1.16718871	-0.2275298
1438942_x_at	transglutaminase 2, C polypeptide	Tgm2	0.64823	1.14810356	0.1989664
1418488_s_at	receptor-interacting serine-threonine kinase 4	Ripk4	0.64825	1.13487603	0.18050268
1438949_at	resistance to inhibitors of cholinesterase 8 homolog (C. elegans)	Ric8	0.64825	-1.1462791	-0.1974819
1430258_at	RIKEN cDNA 2810422J05 gene	2810422J05Rik	0.64827	-1.15606946	-0.2112583
1434751_at	iduronate 2-sulfatase	Ids	0.64828	-1.19393931	-0.2800388
1440331_at	3-ketodihydrosphingosine reductase	Kdsr	0.6483	1.1531649	0.20040961
1442197_at	---	---	0.64835	-1.18377843	-0.2617515
1430388_a_at	sulfatase 2	Sulf2	0.64835	1.10557358	0.14365895
1430074_x_at	RIKEN cDNA 8430426H19 gene	8430426H19Rik	0.64836	-1.22819521	-0.3480882
1451275_at	UHRF1 (ICBP90) binding protein 1-like	Uhrf1bp1l	0.64837	1.11695983	0.15713461
1424954_a_at	phosphatidylinositol-4-phosphate 5-kinase, type 1 gamma	Pip5k1c	0.64837	1.13242091	0.17499262
1430288_x_at	ribosomal protein S21	Rps21	0.64842	1.05729038	0.0795336
1446723_at	---	---	0.64852	1.11996018	0.16143539
1442698_at	---	---	0.64855	1.18679594	0.23297324
1434775_at	par-3 (partitioning defective 3) homolog (C. elegans)	Pard3	0.6486	-1.1495067	-0.209759
1439434_x_at	SH2 domain containing 5	Sh2d5	0.64862	-1.18549071	-0.2581843
1434814_x_at	glucose phosphate isomerase 1	Gpi1	0.64873	1.08818167	0.12155934
1425194_a_at	RIKEN cDNA 6330577E15 gene	6330577E15Rik	0.64873	-1.09981198	-0.1372806
1457436_at	TBC1 domain family, member 7	Tbc1d7	0.64876	-1.17418576	-0.2430964
1428982_at	ATPase family, AAA domain containing 2B	Atad2b	0.64877	-1.11418844	-0.1595888
1449163_at	single immunoglobulin and toll-interleukin 1 receptor (TIR) domai	Sigirr	0.64879	1.12275825	0.16253518
1459864_at	G protein-coupled receptor 146	Gpr146	0.64883	-1.14912094	-0.2043543
1445977_at	expressed sequence AU022793	AU022793	0.64938	1.13850658	0.18326388
1433731_at	insulin-like growth factor 2 mRNA binding protein 3	Igf2bp3	0.64951	-1.13865121	-0.194347
1434773_a_at	solute carrier family 2 (facilitated glucose transporter), member 1	Slc2a1	0.64968	1.11490758	0.15043547
1445824_at	zinc finger protein 458	Zfp458	0.65012	-1.21370591	-0.3221907
1436543_at	GTP-binding protein 10 (putative)	Gtpbp10	0.65014	-1.11831909	-0.1643357
1457929_at	transformed mouse 3T3 cell double minute 2	Mdm2	0.65021	-1.16947237	-0.24523
1428471_at	sorbin and SH3 domain containing 1	Sorbs1	0.65021	1.15865811	0.21234661
1459604_at	---	---	0.65023	1.14014025	0.18908889
1438226_at	expressed sequence AU022252	AU022252	0.65027	1.14822242	0.19071662
1418573_a_at	hnRNP-associated with lethal yellow	Raly	0.65028	1.1415504	0.18317882
1441005_at	regulation of nuclear pre-mRNA domain containing 1B	Rprd1b	0.65029	1.24155408	0.28648845
1449977_at	early growth response 4	Egr4	0.65043	-1.13804099	-0.1867234
1418640_at	sirtuin 1 (silent mating type information regulation 2, homolog) 1	Sirt1	0.65047	-1.16133241	-0.2400892
1444853_at	---	---	0.65052	1.12754427	0.17068466
1441395_at	expressed sequence AU021933	AU021933	0.65054	-1.16272768	-0.2180154
1460184_at	hydroxyacyl-Coenzyme A dehydrogenase	Hadh	0.65064	1.1171685	0.15700973
1454890_at	angiomotin	Amot	0.6507	1.15113463	0.2025629
1457166_at	---	---	0.6508	-1.15278633	-0.2074587
1426541_a_at	endonuclease domain containing 1	Endod1	0.65088	-1.15177438	-0.2138818
1431723_at	RIKEN cDNA 1600022D10 gene	1600022D10Rik	0.65089	1.14903752	0.19979178
1446861_at	glucosamine (N-acetyl)-6-sulfatase	Gns	0.65149	1.19109468	0.24143259
1434670_at	kinesin family member 5A	Kif5a	0.65215	1.17429775	0.22369823
1435607_at	glutamate receptor, metabotropic 2	Grm2	0.65225	1.12447655	0.16781265
1458662_at	dishevelled associated activator of morphogenesis 1	Daam1	0.65237	-1.14751362	-0.1992856
1434114_at	REX4, RNA exonuclease 4 homolog (S. cerevisiae)	Rexo4	0.65244	1.11753599	0.15987043
1440456_at	dynein, axonemal, heavy chain 7A	Dnahc7a	0.65248	1.23442511	0.27877686
1425977_a_at	STE20-like kinase (yeast)	Slk	0.65254	-1.13296638	-0.1836325
1415993_at	squalene epoxidase	Sqle	0.65258	1.12202357	0.16369975
1420387_at	MpV17 mitochondrial inner membrane protein	Mpv17	0.65262	-1.13432365	-0.1899009
1420495_a_at	vacuolar protein sorting 26 homolog A (yeast)	Vps26a	0.65263	1.10955425	0.14984141
1423045_at	nuclear cap binding protein subunit 2	Ncbp2	0.65269	1.10829648	0.14791143
1441844_at	---	---	0.65271	1.1874269	0.23512068
1419254_at	methylenetetrahydrofolate dehydrogenase (NAD+ dependent), m	Mthfd2	0.65272	-1.13031015	-0.1852767
1419612_at	RRS1 ribosome biogenesis regulator homolog pseudogene	4632415L05Rik	0.65273	1.17964068	0.22449862
1444930_at	---	---	0.65275	-1.15935654	-0.2167074
1434549_at	RAB11a, member RAS oncogene family	Rab11a	0.65276	1.12133557	0.16461586
1427231_at	roundabout homolog 1 (Drosophila)	Robo1	0.65277	-1.17498858	-0.2549477
1419055_a_at	protein tyrosine phosphatase, non-receptor type 21	Ptpn21	0.65277	-1.20895229	-0.3194329
1446256_at	---	---	0.65278	1.17689695	0.22770095
1459165_at	DNA segment, Chr 4, ERATO Doi 179, expressed	D4Ertd179e	0.65278	-1.12369508	-0.1690149
1427662_at	cDNA sequence BC025933	BC025933	0.65278	-1.15014178	-0.2047985

1420620_a_at	ring finger protein 13	Rnf13	0.65282	1.14053927	0.18615687
1417015_at	Ras association (RalGDS/AF-6) domain family member 3	Rassf3	0.65283	1.16575489	0.21233751
1427435_at	---	---	0.65286	-1.17443092	-0.2438837
1435543_at	adenomatosis polyposis coli	Apc	0.65287	-1.11970928	-0.1638486
1423935_x_at	keratin 14	Krt14	0.6529	-1.15702386	-0.2110299
1433950_at	immunoglobin superfamily, member 21	Igsf21	0.6529	-1.12865965	-0.1776548
1425388_a_at	thiamine pyrophosphokinase	Tpk1	0.6529	-1.1324114	-0.1816603
1455063_at	family with sequence similarity 82, member A1	Fam82a1	0.65292	-1.14090064	-0.1927437
1434495_at	POZ (BTB) and AT hook containing zinc finger 1	Patz1	0.65293	1.14103526	0.18746576
1421052_a_at	spermine synthase	Sms	0.65295	1.10502537	0.14233689
1451153_a_at	cysteine and histidine rich 1	Cyhr1	0.65295	1.13195488	0.17754625
1429891_at	calcyphosine-like	Capsl	0.65297	1.14478182	0.19240581
1425906_a_at	sema domain, immunoglobulin domain (Ig), short basic domain, s	Sema3e	0.65299	-1.17315123	-0.242647
1445068_at	mucosa associated lymphoid tissue lymphoma translocation gene	Malt1	0.65299	1.18092079	0.2319815
1451772_at	two pore channel 1	Tpcn1	0.65299	-1.1570372	-0.2221527
1426548_a_at	ATP binding domain 4	Atpbd4	0.65301	-1.12257487	-0.1678064
1448043_x_at	ring finger protein 2	Rnf2	0.65303	1.15055525	0.19544299
1426214_at	IMP4, U3 small nucleolar ribonucleoprotein, homolog (yeast)	Imp4	0.65303	-1.15400184	-0.2081333
1457799_at	potassium voltage-gated channel, delayed-rectifier, subfamily S,	Kcns3	0.65305	-1.16538721	-0.2282555
1445638_at	---	---	0.65305	1.14296476	0.18828379
1423920_at	non-SMC condensin I complex, subunit H	Ncaph	0.65305	1.11326941	0.15449919
1449580_s_at	histocompatibility 2, class II, locus Mb1 /// histocompatibility 2, cl	H2-DMb1 /// H2-DMb2	0.65307	-1.15657932	-0.2145968
1460550_at	myotubularin related protein 11	Mtmr11	0.65308	1.23230804	0.27714748
1428396_at	SMAD specific E3 ubiquitin protein ligase 1	Smurf1	0.65308	1.13064638	0.17714265
1418945_at	matrix metallopeptidase 3	Mmp3	0.65309	-1.17129444	-0.2360307
1436828_a_at	tumor protein D52-like 2	Tpd52l2	0.65309	1.10712041	0.14416984
1449065_at	acyl-CoA thioesterase 1	Acot1	0.65309	1.15581761	0.20394706
1459729_at	solute carrier family 13 (sodium-dependent citrate transporter), m	Slc13a5	0.65309	-1.17378638	-0.2408789
1439512_at	RIKEN cDNA 5830444B04 gene	5830444B04Rik	0.65309	1.19050746	0.24062043
1444550_at	RIKEN cDNA 1110021J02 gene	1110021J02Rik	0.6531	-1.14389802	-0.1970992
1427694_at	gonadotropin releasing hormone receptor	Gnrhr	0.65311	1.13137453	0.17279904
1455288_at	RIKEN cDNA 1110036O03 gene	1110036O03Rik	0.65311	-1.13594397	-0.1883792
1446479_at	TNNI3 interacting kinase	Tnni3k	0.65312	-1.17565043	-0.2476785
1453906_at	mediator complex subunit 13-like	Med13l	0.65312	-1.15411293	-0.2105446
1439970_at	---	---	0.65313	-1.16753148	-0.2328532
1421658_x_at	pregnancy specific glycoprotein 18	Psg18	0.65314	1.17180429	0.21939848
1443914_at	lipase, family member K	Lipk	0.65315	1.16661653	0.2129737
1455966_s_at	nudix (nucleoside diphosphate linked moiety X)-type motif 21	Nudt21	0.65315	-1.11265858	-0.1592546
1436703_x_at	small nuclear RNA activating complex, polypeptide 2	Snapc2	0.65315	1.10584759	0.14430535
1458158_at	WD repeat domain 67	Wdr67	0.65316	-1.1515469	-0.2052006
1455791_at	chromatin licensing and DNA replication factor 1	Cdt1	0.65319	1.16582567	0.21099447
1455510_at	---	---	0.65319	-1.17925622	-0.2510912
1432885_at	RIKEN cDNA 4632432E15 gene	4632432E15Rik	0.65319	1.18282261	0.23380838
1450709_at	defensin, alpha, 5 /// predicted gene 10104 /// predicted gene 15	Defa5 /// Gm10104 /// G	0.6532	-1.12937281	-0.1768446
1416568_a_at	apoptotic chromatin condensation inducer 1	Acin1	0.65321	-1.12470857	-0.1758327
1423890_x_at	ATPase, Na+/K+ transporting, beta 1 polypeptide	Atp1b1	0.65322	1.12308325	0.16141066
1426499_at	SH3-domain GRB2-like endophilin B2	Sh3glb2	0.65323	1.13755559	0.18080872
1440977_at	---	---	0.65323	-1.14143049	-0.1910078
1428860_at	RIKEN cDNA 4930572J05 gene	4930572J05Rik	0.65323	1.29333463	0.32016708
1421274_at	suppressor of cytokine signaling 4	Socs4	0.65324	-1.17087179	-0.2436172
1430778_a_at	nucleotide binding protein 1	Nubp1	0.65325	1.12846381	0.16787326
1435836_at	pyruvate dehydrogenase kinase, isoenzyme 1	Pdk1	0.65326	1.10478398	0.14351699
1439920_at	---	---	0.65327	1.22621282	0.27256052
1424683_at	family with sequence similarity 134, member B	Fam134b	0.65327	1.1590896	0.20569915
1441979_at	cDNA sequence BC060267	BC060267	0.65327	1.27726486	0.31169528
1435948_at	transmembrane protein 181A /// transmembrane protein 181B, p	Tmem181a /// Tmem181	0.65329	1.13492031	0.17971008
1445601_at	---	---	0.6533	-1.14053454	-0.1904248
1438386_x_at	methionine adenosyltransferase II, alpha	Mat2a	0.6533	-1.10606075	-0.1490334
1456322_at	Growth arrest specific 1	Gas1	0.65331	-1.1565924	-0.214157
1430805_s_at	RMI1, RecQ mediated genome instability 1, homolog (S. cerevisia	Rmi1	0.65331	-1.11079826	-0.1535317
1455435_s_at	choline dehydrogenase	Chdh	0.65331	-1.15354941	-0.2077528
1457265_at	A kinase (PRKA) anchor protein 17B	Akap17b	0.65332	1.12280449	0.1665352
1429428_at	transcription factor 7-like 2, T-cell specific, HMG-box	Tcf7l2	0.65334	-1.17960718	-0.2507116
1419700_a_at	prominin 1	Prom1	0.65334	1.21197455	0.25985611
1455057_at	guanine monophosphate synthetase	Gmps	0.65335	-1.2259773	-0.3542522
1460052_at	---	---	0.65335	1.16895614	0.22012423
1434426_at	non-SMC condensin II complex, subunit D3	Ncapd3	0.65336	1.12890458	0.17080826
1417267_s_at	FK506 binding protein 11	Fkbp11	0.65337	1.13739684	0.17898448
1421985_a_at	eukaryotic translation initiation factor 4E member 2	Eif4e2	0.65338	1.10144776	0.13826325
1455777_x_at	hydroxysteroid (17-beta) dehydrogenase 4	Hsd17b4	0.65338	-1.16643299	-0.2520943
1444723_at	RIKEN cDNA 6530418L21 gene	6530418L21Rik	0.65338	1.18003561	0.23086711
1418236_s_at	autophagy-related 5 (yeast)	Atg5	0.65341	1.12118749	0.16383338
1439579_at	---	---	0.65341	-1.15118616	-0.2036097
1457645_at	RIKEN cDNA C130079G13 gene	C130079G13Rik	0.65343	1.12012549	0.16298405
1437264_at	cDNA sequence BC051142	BC051142	0.65345	-1.16627437	-0.2364355
1426605_at	BRCA1/BRCA2-containing complex, subunit 3	Brcc3	0.65345	-1.20085574	-0.3016092
1438221_at	family with sequence similarity 126, member B	Fam126b	0.65345	-1.13595979	-0.1882511
1427257_at	versican	Vcan	0.65348	1.207405	0.25424763
1431558_at	RIKEN cDNA 2310016D03 gene	2310016D03Rik	0.65348	-1.16153385	-0.2228246
1419737_a_at	lactate dehydrogenase A	Ldha	0.6535	1.06543097	0.091062
1458113_at	RIKEN cDNA 9530019H20 gene	9530019H20Rik	0.6535	1.12816458	0.17256811
1426239_s_at	arrestin, beta 2	Arrb2	0.65351	1.13625788	0.17920549
1427274_at	ring finger protein 214	Rnf214	0.65351	1.14887338	0.19774953
1450464_at	E4F transcription factor 1	E4f1	0.65353	1.129482	0.17523865
1425291_at	forkhead box J1	Foxj1	0.65353	1.14930572	0.19964764
1445870_at	---	---	0.65354	1.163143	0.20899739
1417218_at	calcium homeostasis modulator 2	Calhm2	0.65355	1.21555521	0.26066782
1421928_at	Eph receptor A4	Epha4	0.65355	1.14204869	0.18513863
1420231_at	---	---	0.65358	1.18325591	0.22854126
1426978_at	kelch-like 2, Mayven (Drosophila) /// kelch-like protein 2-like	Klhl2 /// LOC100502725	0.65358	-1.11727984	-0.1629226
1447623_s_at	protein kinase D1	Prkd1	0.65358	1.21845085	0.26360451
1440942_at	dapper homolog 2, antagonist of beta-catenin (xenopus)	Dact2	0.65358	1.18607661	0.23091954
1420826_at	leucine zipper-EF-hand containing transmembrane protein 1	Letm1	0.65361	1.1064279	0.145653
1445497_at	---	---	0.65362	1.16015078	0.2117339

1431571_at	RIKEN cDNA 4933437I04 gene	4933437I04Rik	0.65362	-1.15846346	-0.2129137
1455745_at	ceroid-lipofuscinosis, neuronal 8	Cln8	0.65362	-1.15518517	-0.219322
1444671_at	RAS protein activator like 2	Rasal2	0.65363	-1.13004571	-0.177211
1430996_at	ethanolamine kinase 1	Etnk1	0.65363	-1.15919348	-0.2279336
1416484_at	tetratricopeptide repeat domain 3	Ttc3	0.65368	-1.12701552	-0.1781313
1460641_a_at	RNA binding motif protein 42	Rbm42	0.65369	1.11229139	0.15228568
1440916_at	RIKEN cDNA 2510049J12 gene	2510049J12Rik	0.65369	-1.17633942	-0.2544331
1429579_at	RIKEN cDNA 6330407I18 gene	6330407I18Rik	0.65369	-1.18399429	-0.2606407
1437177_at	La ribonucleoprotein domain family, member 4	Larp4	0.65369	-1.12367875	-0.1776982
1416065_a_at	ankyrin repeat domain 10	Ankrd10	0.65377	-1.11890829	-0.167934
1426516_a_at	lipin 1	Lpin1	0.65378	1.11617487	0.15813045
1417147_at	family with sequence similarity 69, member B	Fam69b	0.6538	1.1232044	0.16680214
1437179_at	Rap1 interacting factor 1 homolog (yeast)	Rif1	0.65381	-1.13185115	-0.1937253
1449804_at	phenylethanolamine-N-methyltransferase	Pnmt	0.65381	-1.12031431	-0.1647877
1442408_at	sulfatase 2	Sulf2	0.65381	-1.11432737	-0.1620103
1416654_at	solute carrier family 31, member 2	Slc31a2	0.65382	1.12559119	0.16832151
1449643_s_at	basic transcription factor 3	Btf3	0.65383	1.07312318	0.10151439
1416926_at	transformation related protein 53 inducible nuclear protein 1	Trp53inp1	0.65385	-1.11259825	-0.1591547
1427386_at	Rho guanine nucleotide exchange factor (GEF) 16	Arhgef16	0.65385	1.16963588	0.21703692
1440903_at	---	---	0.65387	1.20462876	0.25540079
1430351_at	spermatogenesis associated 18	Spata18	0.65388	-1.16104886	-0.216362
1455102_at	La ribonucleoprotein domain family, member 4	Larp4	0.6539	-1.11821507	-0.1669302
1428846_at	tetratricopeptide repeat domain 14	Ttc14	0.6539	-1.14619116	-0.2029246
1423873_at	LSM1 homolog, U6 small nuclear RNA associated (S. cerevisiae)	Lsm1	0.65391	1.10938485	0.14970521
1416199_at	kinesin family member C3	Kifc3	0.65392	-1.1184904	-0.1637734
1457812_at	transformation related protein 53 binding protein 1	Trp53bp1	0.65393	1.15554966	0.20528746
1431786_s_at	RIKEN cDNA 1190003J15 gene	1190003J15Rik	0.65394	1.12148196	0.15987037
1443398_at	---	---	0.65395	-1.1522424	-0.2052939
1442867_at	---	---	0.65399	-1.15628494	-0.209848
1456227_x_at	retinoblastoma binding protein 7	Rbbp7	0.65399	-1.10904073	-0.1587581
1425772_at	collagen, type IV, alpha 4	Col4a4	0.654	-1.14347595	-0.1937937
1448593_at	WNT1 inducible signaling pathway protein 1	Wisp1	0.65403	1.19159774	0.24032321
1416747_at	major facilitator superfamily domain containing 5	Mfsd5	0.65404	1.10397852	0.14219925
1417814_at	phospholipase A2, group V	Pla2g5	0.65405	1.16883752	0.220826
1436328_at	RNA (guanine-7-) methyltransferase	Rnmt	0.65407	1.11994832	0.16327813
1439432_x_at	hypothetical LOC100502863 /// mortality factor 4 like 2	LOC100502863 /// Morf4	0.65408	-1.07377537	-0.1029869
1452234_s_at	transmembrane protein 191C	Tmem191c	0.65411	-1.13937146	-0.1927339
1446629_at	---	---	0.65414	-1.09604622	-0.1328633
1426827_at	YTH domain containing 1	Ythdc1	0.65415	-1.11974031	-0.1687858
1442214_at	---	---	0.65421	1.17332568	0.22314233
1453406_a_at	RAB28, member RAS oncogene family	Rab28	0.65421	1.12503984	0.16971245
1460436_at	N-deacetylase/N-sulfotransferase (heparan glucosaminyl) 1	Ndst1	0.65422	1.12919999	0.17219838
1438817_at	DNA replication helicase 2 homolog (yeast)	Dna2	0.65424	1.14458954	0.19090375
1455781_at	GTP-binding protein 8 (putative)	Gtpbp8	0.65428	1.1520399	0.20325042
1431125_a_at	threonyl-tRNA synthetase 2, mitochondrial (putative)	Tars2	0.65432	1.12546989	0.16538411
1421100_a_at	disabled homolog 1 (Drosophila)	Dab1	0.65433	1.12479522	0.1684061
1423904_a_at	poliovirus receptor	Pvr	0.65435	-1.13966983	-0.1983928
1428629_at	zinc finger protein 518A	Zfp518a	0.65437	-1.12555826	-0.1755947
1431420_s_at	PRELI domain containing 1	Prelid1	0.65438	1.09283978	0.12652006
1422164_at	POU domain, class 3, transcription factor 4	Pou3f4	0.65441	-1.16339571	-0.2258876
1423541_at	RIKEN cDNA 4930511I11 gene	4930511I11Rik	0.65441	-1.16150992	-0.2191843
1440607_at	hypothetical protein LOC100503855	LOC100503855	0.65442	1.16867037	0.22222982
1454825_at	RIKEN cDNA 1110014N23 gene	1110014N23Rik	0.65448	1.13553451	0.18027144
1456786_at	LIM domain binding 2	Ldb2	0.65449	1.09432796	0.12984036
1452950_at	methyltransferase 5 domain containing 1	Mett5d1	0.65449	-1.16642493	-0.2352663
1435987_x_at	RIKEN cDNA 1110059G02 gene	1110059G02Rik	0.6545	-1.16106085	-0.2158443
1418505_at	nudix (nucleoside diphosphate linked moiety X)-type motif 4	Nudt4	0.65454	-1.10334291	-0.1445685
1422116_at	AF4/FMR2 family, member 2	Aff2	0.65459	1.15284009	0.20482232
1453676_at	RIKEN cDNA A930041D05 gene	A930041D05Rik	0.6546	-1.169059	-0.2337493
1455472_at	RIKEN cDNA A630071D13 gene /// vezatin, adherens junctions tr	A630071D13Rik /// Vezt	0.65462	1.17068826	0.22055993
1434492_at	RIKEN cDNA A130022J15 gene	A130022J15Rik	0.65465	1.15759168	0.20783809
1453025_at	MACRO domain containing 2	Macrod2	0.65472	-1.12662058	-0.1727539
1434149_at	transcription factor 4	Tcf4	0.65493	-1.17253279	-0.247631
1456933_at	---	---	0.65507	-1.16546114	-0.233234
1434061_at	retinitis pigmentosa 2 homolog (human)	Rp2h	0.6552	-1.13813919	-0.1879743
1416503_at	latexin	Lxn	0.65526	1.14283907	0.18832522
1430151_at	nischarin	Nisch	0.65531	-1.17278445	-0.2445143
1431507_a_at	COX16 cytochrome c oxidase assembly homolog (S. cerevisiae) /	Cox16 /// Synj2bp	0.65532	1.11609338	0.1574431
1441132_at	---	---	0.6554	1.17250094	0.22219142
1444250_at	---	---	0.6554	-1.14874582	-0.2028944
1455876_at	solute carrier family 4, sodium bicarbonate cotransporter, membe	Slc4a7	0.65618	-1.17418963	-0.2594678
1416457_at	dimethylarginine dimethylaminohydrolase 2	Ddah2	0.65626	1.0987703	0.13579064
1434335_at	DIP2 disco-interacting protein 2 homolog B (Drosophila)	Dip2b	0.65628	1.10131465	0.13862827
1450292_a_at	HORMA domain containing 1	Hormad1	0.65638	-1.14450832	-0.1973491
1417985_at	Notch-regulated ankyrin repeat protein	Nrarp	0.65639	1.16676321	0.21510999
1435603_at	sushi, nidogen and EGF-like domains 1	Sned1	0.6564	1.12583524	0.17088136
1420618_at	cytoplasmic polyadenylation element binding protein 4	Cpeb4	0.65642	-1.12877812	-0.1751796
1428224_at	heterogeneous nuclear ribonucleoprotein D-like	Hnrpdl	0.65643	-1.13415491	-0.1935221
1432968_at	RIKEN cDNA 4930456A14 gene	4930456A14Rik	0.65644	1.16381211	0.21171341
1441223_at	membrane-associated ring finger (C3HC4) 4	Mar-04	0.65646	-1.1529423	-0.205773
1443882_at	solute carrier family 26 (sulfate transporter), member 2	Slc26a2	0.65646	1.13932098	0.18540393
1438931_s_at	sestrin 1	Sesn1	0.65653	-1.19929246	-0.3065742
1428036_at	predicted gene 15498	Gm15498	0.65655	-1.15990276	-0.2177524
1415959_at	solute carrier family 2 (facilitated glucose transporter), member 4	Slc2a4	0.65656	1.13593718	0.18372683
1449338_at	anaphase promoting complex subunit 16	Anapc16	0.65656	1.10604071	0.14484829
1452845_at	hypoxia-inducible factor 1, alpha subunit inhibitor	Hif1an	0.65658	1.18337787	0.22764011
1424989_at	ORAI calcium release-activated calcium modulator 1	Orai1	0.6566	-1.15001131	-0.2040222
1423258_at	synaptotagmin IX	Syt9	0.65661	-1.12437411	-0.1702621
1436051_at	myosin VA	Myo5a	0.65661	-1.15743339	-0.2233836
1425477_x_at	histocompatibility 2, class II antigen A, beta 1	H2-Ab1	0.65668	1.23789219	0.28320091
1422231_a_at	tumor necrosis factor receptor superfamily, member 25	Tnfrsf25	0.65668	1.15712085	0.20523737
1421910_at	transcription factor 20	Tcf20	0.65678	-1.1446257	-0.2087445
1426357_at	TAO kinase 1	Taok1	0.65683	-1.11311315	-0.15538
1458405_at	predicted gene 5113	Gm5113	0.65686	1.20967063	0.25648143

1435405_at	SET domain containing 4	Setd4	0.65686	1.17871002	0.22698117
1445719_at	Splicing factor 3a, subunit 1	Sf3a1	0.65686	-1.14053805	-0.1921466
1444902_at	---	---	0.65691	-1.15792422	-0.2129457
1429356_s_at	geranylgeranyl diphosphate synthase 1	Ggps1	0.65773	-1.22398519	-0.3511763
1448229_s_at	cyclin D2	Ccnd2	0.65782	-1.21783433	-0.3373245
1416490_at	transmembrane emp24 protein transport domain containing 6	Tmed6	0.65784	1.19159947	0.23587785
1425051_at	isochorismatase domain containing 1	Isoc1	0.65787	-1.13363917	-0.1859358
1426005_at	dentin matrix protein 1	Dmp1	0.65789	1.15695618	0.20744953
1441784_at	hypothetical LOC100503546	LOC100503546	0.65794	-1.13619684	-0.1902962
1424376_at	CDC42 effector protein (Rho GTPase binding) 1	Cdc42ep1	0.65795	1.17754915	0.21981901
1428753_a_at	DiGeorge syndrome critical region gene 6	Dgcr6	0.65796	1.11057322	0.15020964
1419916_at	Ring finger protein 20	Rnf20	0.65797	-1.16466409	-0.2292818
1453488_at	RIKEN cDNA 4930458D05 gene	4930458D05Rik	0.65797	1.19408597	0.2397225
1446041_at	---	---	0.65799	-1.14066461	-0.1902685
1449157_at	nuclear receptor subfamily 2, group C, member 1	Nr2c1	0.65799	1.16533981	0.21583592
1429733_at	coiled-coil domain containing 94	Ccdc94	0.65801	1.12637985	0.1686982
1455847_at	p21 protein (Cdc42/Rac)-activated kinase 7	Pak7	0.65805	1.15111862	0.20164936
1434106_at	EPM2A (laforin) interacting protein 1	Epm2aip1	0.65807	-1.18420341	-0.2712233
1426743_at	adaptor protein, phosphotyrosine interaction, PH domain and leu	Appl2	0.65813	1.11437044	0.15539004
1446759_at	Lin-52 homolog (C. elegans)	Lin52	0.65814	-1.13310418	-0.1807395
1428958_at	progestin and adipoQ receptor family member VIII	Paqr8	0.65817	1.18840016	0.23962987
1450937_at	lin-7 homolog C (C. elegans)	Lin7c	0.6582	-1.14175671	-0.2047319
1425058_at	zinc finger protein 472	Zfp472	0.65827	1.11355217	0.15404071
1421960_at	adenylate cyclase 3	Adcy3	0.65828	1.12561029	0.16996088
1460466_at	RIKEN cDNA 1700047I17 gene 2 /// family with sequence similar	1700047I17Rik2 /// Fam1	0.65833	1.13308431	0.17978926
1447627_at	transmembrane protein 132E	Tmem132e	0.65833	-1.15422049	-0.2089427
1447978_at	expressed sequence C80719	C80719	0.65837	1.10342019	0.13747746
1416852_a_at	neurochondrin	Ncdn	0.65849	1.14159826	0.1844289
1425053_at	isochorismatase domain containing 1	Isoc1	0.65849	-1.12540542	-0.1767419
1417373_a_at	tubulin, alpha 4A	Tuba4a	0.65851	1.08471842	0.11612369
1416428_at	THAP domain containing 11	Thap11	0.65855	1.11040129	0.14920745
1427293_a_at	autism susceptibility candidate 2	Auts2	0.65857	1.19595278	0.244191
1442017_at	nitrogen fixation gene 1 (S. cerevisiae)	Nfs1	0.65859	-1.16405243	-0.224181
1449853_at	sideroflexin 2	Sfxn2	0.65861	-1.16660497	-0.2323933
1438107_x_at	expressed sequence BB236558	BB236558	0.65864	1.1479019	0.19373762
1416057_at	NADH dehydrogenase (ubiquinone) 1 beta subcomplex, 11	Ndufb11	0.65866	1.10389935	0.14013748
1418024_at	N(alpha)-acetyltransferase 15, NatA auxiliary subunit	Naa15	0.65875	-1.13276884	-0.1922608
1436457_at	ubiquitin-conjugating enzyme E2I	Ube2i	0.65877	1.16830708	0.21942112
1445668_at	---	---	0.65878	-1.14824042	-0.2013891
1453249_a_at	testis expressed gene 21	Tex21	0.65878	1.15432621	0.20631691
1450860_at	leucine aminopeptidase 3	Lap3	0.65886	-1.11064084	-0.1584969
1420740_at	interleukin 25	Il25	0.65889	-1.1663091	-0.2320386
1423342_at	BarH-like homeobox 1	Barx1	0.65903	1.15363346	0.20468897
1429946_at	coiled-coil domain containing 141	Ccdc141	0.65908	1.1619125	0.21487278
1452885_at	serine/arginine-rich splicing factor 2, interacting protein	Srsf2ip	0.65912	-1.19412241	-0.3037414
1439528_at	RIKEN cDNA 4833423E24 gene	4833423E24Rik	0.65916	-1.15644607	-0.2099221
1417079_s_at	lectin, galactose-binding, soluble 2	Lgals2	0.65918	1.15528363	0.20744446
1416348_at	multiple endocrine neoplasia 1	Men1	0.65921	1.11409612	0.15404188
1418108_at	rhotekin 2	Rtkn2	0.65926	1.14618369	0.19330379
1452471_at	interleukin 17 receptor D	Il17rd	0.65974	1.14874399	0.19989903
1437060_at	olfactomedin 4	Olfm4	0.66004	-1.13177362	-0.1791376
1428936_at	ATPase, Ca++ transporting, plasma membrane 1	Atp2b1	0.66033	-1.19259414	-0.3000696
1460698_a_at	SEC11 homolog C (S. cerevisiae)	Sec11c	0.66044	-1.12271537	-0.172323
1437600_at	---	---	0.66052	-1.13080456	-0.1791972
1444254_at	tensin 4	Tns4	0.66061	1.16089128	0.20842053
1451330_a_at	inositol polyphosphate-5-phosphatase B	Inpp5b	0.66068	1.11429546	0.15534532
1440148_at	G protein-coupled receptor 6	Gpr6	0.66075	-1.11695768	-0.1623708
1422141_s_at	component of Sp100-rs	Csprs	0.66084	1.14778604	0.19473448
1432454_at	RIKEN cDNA 2410137M14 gene	2410137M14Rik	0.66085	-1.14064253	-0.198959
1417684_at	THUMP domain containing 3	Thumpd3	0.66087	-1.10154699	-0.1405347
1421634_at	calpain 8	Capn8	0.66094	1.13663053	0.1781761
1426454_at	Rho, GDP dissociation inhibitor (GDI) beta	Arhgdib	0.66095	-1.12392159	-0.1699163
1431488_at	RIKEN cDNA 4833428L15 gene	4833428L15Rik	0.66097	1.16391831	0.21645777
1418716_at	mitochondrial ribosomal protein S25	Mrps25	0.66104	1.13502287	0.18004874
1437248_at	RIKEN cDNA 2700049A03 gene	2700049A03Rik	0.66105	-1.17801299	-0.2537653
1457142_at	N-terminal EF-hand calcium binding protein 1	Necab1	0.66105	-1.14578268	-0.2012744
1419721_at	niacin receptor 1	Niacr1	0.66105	-1.15878469	-0.2158988
1448579_at	golgi apparatus protein 1	Glg1	0.66106	1.11210934	0.15167796
1446364_at	---	---	0.66111	-1.14699388	-0.2106286
1436345_at	RIKEN cDNA 5730559C18 gene	5730559C18Rik	0.66114	1.1274369	0.16949415
1435443_at	eyes absent 3 homolog (Drosophila)	Eya3	0.66116	1.11443564	0.15573761
1450031_at	AF4/FMR2 family, member 4	Aff4	0.66121	-1.10220379	-0.1423018
1416035_at	hypoxia inducible factor 1, alpha subunit	Hif1a	0.66129	-1.13868587	-0.1912638
1430772_at	---	---	0.6614	1.14007124	0.18859817
1423667_at	methionine adenosyltransferase II, alpha	Mat2a	0.66141	-1.10809707	-0.1534022
1457052_at	potassium voltage-gated channel, subfamily G, member 1	Kcng1	0.66143	1.14634355	0.19626044
1434056_a_at	NADH dehydrogenase (ubiquinone) 1 beta subcomplex, 6	Ndufb6	0.66144	-1.14503381	-0.2177683
1425752_at	cDNA sequence BC014805	BC014805	0.66149	-1.15352901	-0.2140569
1440001_at	RNA imprinted and accumulated in nucleus	Rian	0.6615	-1.14690622	-0.1986103
1456094_at	ubiquitin specific peptidase 36	Usp36	0.66152	-1.15749939	-0.2214586
1425348_a_at	signal recognition particle receptor, B subunit	Srprb	0.66154	1.1190308	0.16065941
1460487_at	RIKEN cDNA 1110014L15 gene	1110014L15Rik	0.66159	1.16548241	0.20823871
1415816_at	chaperonin containing Tcp1, subunit 7 (eta)	Cct7	0.6616	1.08297224	0.11479442
1458702_at	RIKEN cDNA 2610316D01 gene	2610316D01Rik	0.66163	-1.15903114	-0.218968
1428889_at	alkB, alkylation repair homolog 3 (E. coli)	Alkbh3	0.66165	1.10653743	0.14561989
1444308_at	---	---	0.66165	-1.15914347	-0.217411
1451305_at	chibby homolog 1 (Drosophila)	Cby1	0.66166	1.10361291	0.14195694
1417744_a_at	v-ral simian leukemia viral oncogene homolog B (ras related)	Ralb	0.66168	-1.10700024	-0.1467291
1429455_at	GTPase activating protein and VPS9 domains 1	Gapvd1	0.66169	1.13593769	0.1787205
1431871_at	thioredoxin domain containing 3 (spermatozoa)	Txndc3	0.6617	1.30927926	0.33457383
1428164_at	nudix (nucleoside diphosphate linked moiety X)-type motif 9	Nudt9	0.66172	1.11322138	0.15256891
1434987_at	aldehyde dehydrogenase 2, mitochondrial	Aldh2	0.66172	-1.14420467	-0.1946985
1430521_s_at	copine VIII	Cpne8	0.66173	1.16457353	0.21424224
1460010_a_at	phosphatidylserine synthase 2	Ptdss2	0.66175	1.10967735	0.14955305

1428614_at	lactate dehydrogenase D	Ldhd	0.66175	1.17947206	0.22739812
1445356_at	hypothetical LOC100504206	LOC100504206	0.66177	1.14446963	0.19444297
1434352_at	RIKEN cDNA B630005N14 gene	B630005N14Rik	0.66178	-1.10846715	-0.1487214
1418897_at	coagulation factor II	F2	0.66178	1.13147026	0.17784545
1442293_at	helicase with zinc finger domain	Helz	0.66179	-1.17908097	-0.2620363
1446618_at	RIKEN cDNA A130088B03 gene	A130088B03Rik	0.6618	1.16348065	0.21322654
1421791_at	angiogenin, ribonuclease A family, member 3	Ang3	0.66186	-1.14270879	-0.1974992
1424895_at	G-protein signalling modulator 2 (AGS3-like, C. elegans)	Gpsm2	0.66188	1.12565907	0.17012344
1434866_x_at	carnitine palmitoyltransferase 1a, liver	Cpt1a	0.66188	1.11900688	0.16199906
1445711_at	expressed sequence BB163080	BB163080	0.66192	1.12813785	0.17354318
1417983_a_at	ubiquitin-conjugating enzyme E2 variant 2	Ube2v2	0.66192	-1.1021957	-0.1422857
1446419_at	---	---	0.66193	-1.15713856	-0.2145411
1454681_at	epithelial splicing regulatory protein 1	Esrp1	0.66193	1.17178264	0.21697955
1432726_at	proteasome (prosome, macropain) 26S subunit, non-ATPase, 11	Psmd11	0.66195	1.14047669	0.18815854
1437188_at	gamma-aminobutyric acid (GABA) B receptor, 1	Gabbr1	0.66195	-1.14313996	-0.1974421
1448522_at	seryl-aminoacyl-tRNA synthetase 2	Sars2	0.66195	1.11462289	0.15541483
1452631_at	RUN and FYVE domain-containing 2	Rufy2	0.66196	-1.14045629	-0.1898788
1454751_at	methyltransferase like 14	Mettl14	0.66196	-1.1558733	-0.2302342
1434739_at	fragile X mental retardation 1 neighbor	Fmr1nb	0.66196	1.13934648	0.18353406
1437057_at	multiple EGF-like-domains 6	Megf6	0.66198	-1.15390167	-0.211922
1434246_at	l(3)mbt-like 3 (Drosophila)	L3mbtl3	0.66198	-1.16551802	-0.2291766
1446071_at	six transmembrane epithelial antigen of prostate 2	Steap2	0.66199	-1.17523101	-0.2548062
1437714_x_at	Ubiquitin specific peptidase 14	Usp14	0.66203	-1.11115972	-0.1529283
1460065_at	expressed sequence AA763515	AA763515	0.66203	1.14949032	0.19349032
1424211_at	solute carrier family 25, member 33	Slc25a33	0.66206	1.10540043	0.14353953
1445735_at	---	---	0.66207	1.124079	0.16757217
1454897_at	RIKEN cDNA 6330509M05 gene	6330509M05Rik	0.66208	1.14529299	0.19135144
1434855_at	amidohydrolase domain containing 2	Amdhd2	0.66209	1.13079396	0.17286791
1434660_at	alkB, alkylation repair homolog 1 (E. coli)	Alkbh1	0.66209	-1.11286776	-0.1545696
1427504_s_at	serine/arginine-rich splicing factor 2	Srsf2	0.66214	-1.08204502	-0.1152721
1436882_at	ubiquitin-like 5	Ubl5	0.66216	1.22025626	0.26559905
1416472_at	synapse associated protein 1	Syap1	0.66217	1.12546181	0.16661035
1419975_at	Sterol carrier protein 2, liver	Scp2	0.66221	-1.27691969	-0.4775669
1434671_at	RIKEN cDNA B230337E12 gene	B230337E12Rik	0.66225	-1.10798739	-0.1501528
1436066_at	kalirin, RhoGEF kinase	Kalrn	0.66232	1.17453976	0.22164738
1446870_at	DNA segment, Chr 4, ERATO Doi 510, expressed	D4Ertd510e	0.66242	-1.15239618	-0.2121316
1439514_at	RIKEN cDNA A930004D18 gene	A930004D18Rik	0.66251	1.17503228	0.22426874
1426889_at	zinc finger protein 821	Zfp821	0.6629	1.1484757	0.19797491
1440556_at	predicted gene 11985	Gm11985	0.66306	1.15337281	0.20324233
1423079_a_at	translocase of outer mitochondrial membrane 20 homolog (yeast	Tomm20	0.66312	-1.06380241	-0.0896637
1429100_at	RIKEN cDNA 2010109K11 gene	2010109K11Rik	0.66316	1.1321485	0.17706965
1454015_a_at	cadherin 13	Cdh13	0.66319	-1.14167626	-0.1912902
1448796_s_at	protein TBRG4-like /// transforming growth factor beta regulated	LOC100046166 /// Tbrg4	0.66321	1.09936503	0.13661009
1451736_a_at	mitogen-activated protein kinase kinase 7	Map2k7	0.66321	-1.12379624	-0.1750007
1434196_at	DnaJ (Hsp40) homolog, subfamily A, member 4	Dnaja4	0.66324	-1.14552884	-0.1988561
1445558_at	RIKEN cDNA 5930430L01 gene	5930430L01Rik	0.66326	1.2666616	0.29859216
1455171_at	suppressor of variegation 4-20 homolog 1 (Drosophila)	Suv420h1	0.66326	1.18269685	0.2288375
1417853_at	chloride channel calcium activated 1	Clca1	0.66328	1.13421766	0.18040079
1443905_at	Human papillomavirus 18 E5 central sequence motif, pseudogene	Hpvc-ps	0.66332	-1.15191954	-0.2070003
1445610_at	---	---	0.66332	-1.15824418	-0.2218356
1430386_at	peptidylprolyl isomerase A pseudogene 8	E030024N20Rik	0.66334	1.19677151	0.24575662
1437988_x_at	RIKEN cDNA 1700003E24 gene	1700003E24Rik	0.66345	1.15749676	0.19987902
1419869_s_at	high density lipoprotein (HDL) binding protein	Hdlbp	0.66345	1.10436361	0.14155343
1443460_at	Predicted gene 4250	Gm4250	0.66346	-1.14166703	-0.1915174
1442862_at	---	---	0.66349	1.18275812	0.23408698
1459910_at	tankyrase, TRF1-interacting ankyrin-related ADP-ribose polymera	Tnks	0.66353	1.17038867	0.22076734
1446050_at	---	---	0.66364	-1.15677046	-0.219183
1446961_at	---	---	0.66371	1.14070376	0.18932581
1423769_at	pentatricopeptide repeat domain 2	Ptcd2	0.66376	1.11075011	0.14870396
1436562_at	DEAD (Asp-Glu-Ala-Asp) box polypeptide 58	Ddx58	0.66403	-1.09875102	-0.1366695
1448838_at	topoisomerase I binding, arginine/serine-rich	Topors	0.66432	-1.10038082	-0.139474
1457181_at	PPPDE peptidase domain containing 2	Pppde2	0.66437	1.16093573	0.20794449
1441882_at	---	---	0.66446	-1.14554911	-0.1964411
1430646_at	RIKEN cDNA 5830407P18 gene	5830407P18Rik	0.66446	-1.14783232	-0.2019516
1430327_at	adenosine deaminase, tRNA-specific 3, TAD2 homolog (S. cerevis	Adat3	0.66452	1.11843406	0.16099778
1436037_at	integrin alpha 4	Itga4	0.66452	-1.14803011	-0.2007068
1426787_at	Sfi1 homolog, spindle assembly associated (yeast)	Sfi1	0.66454	1.184054	0.23332267
1449304_at	RIKEN cDNA 2310061J03 gene	2310061J03Rik	0.66454	1.14264689	0.1864022
1421573_at	RIKEN cDNA 4930533N22 gene	4930533N22Rik	0.66458	-1.14823248	-0.1998662
1453782_at	ankyrin repeat domain 33B	Ankrd33b	0.66458	-1.16667829	-0.2369094
1437959_at	zinc finger protein 324	Zfp324	0.6646	1.16317141	0.21424178
1430075_at	Splicing factor 3b, subunit 3	Sf3b3	0.66461	1.18378875	0.22732538
1416299_at	Shc SH2-domain binding protein 1	Shcbp1	0.66463	-1.11923123	-0.1704474
1453109_at	arylsulfatase K	Arsk	0.66464	-1.15330861	-0.2120895
1436737_a_at	sorbin and SH3 domain containing 1	Sorbs1	0.66464	1.19613155	0.24230083
1431368_at	RIKEN cDNA 4930404N11 gene	4930404N11Rik	0.66464	-1.16023006	-0.2151074
1440254_at	small nucleolar RNA, C/D box 104	Snord104	0.66465	1.3027755	0.31956084
1429108_at	male-specific lethal 2 homolog (Drosophila)	Msl2	0.66466	-1.13514166	-0.1835132
1426298_at	Iroquois related homeobox 2 (Drosophila)	Irx2	0.66467	1.16228385	0.20838248
1418513_at	serine/threonine kinase 3 (Ste20, yeast homolog)	Stk3	0.66469	-1.14662173	-0.2028017
1441970_at	RIKEN cDNA E430010N07 gene	E430010N07Rik	0.66469	-1.13534195	-0.1862442
1454649_at	steroid 5 alpha-reductase 1	Srd5a1	0.66471	1.14791346	0.19632676
1436616_at	inositol polyphosphate-4-phosphatase, type I	Inpp4a	0.66471	1.15312377	0.20009788
1456758_at	RIKEN cDNA 9930017N22 gene	9930017N22Rik	0.66476	-1.14502497	-0.1961413
1423995_at	kinesin family member 1B	Kif1b	0.66478	-1.14215345	-0.1976826
1446651_at	---	---	0.66479	-1.14017003	-0.1918082
1454243_at	intestinal cell kinase	Ick	0.66482	1.150198	0.20031175
1450642_at	SECIS binding protein 2-like	Secisbp2l	0.66484	-1.11182684	-0.1542852
1451541_at	BCS1-like (yeast)	Bcs1l	0.66486	1.10122674	0.13905501
1424759_at	arrestin domain containing 4	Arrdc4	0.66486	1.12418618	0.16512052
1439160_at	PRAME family member 8	Pramef8	0.66489	-1.12977339	-0.1830828
1447509_at	---	---	0.66583	1.14623441	0.19684046
1444313_at	---	---	0.66595	1.16331162	0.20792455
1437615_s_at	vacuolar protein sorting 37C (yeast)	Vps37c	0.66598	1.12432488	0.16846844

1415995_at	caspase 6	Casp6	0.66615	1.13171927	0.17629415
1433892_at	sperm associated antigen 5	Spag5	0.6662	1.11663424	0.15846976
1417120_at	migration and invasion inhibitory protein	Miip	0.66623	1.13858231	0.18506513
1420980_at	p21 protein (Cdc42/Rac)-activated kinase 1	Pak1	0.66625	1.1309719	0.17315313
1424396_a_at	asparaginase like 1	Asrgl1	0.66629	-1.11579453	-0.1583819
1434421_at	immunoglobulin superfamily containing leucine-rich repeat 2	Islr2	0.6663	1.13998447	0.18701571
1448972_at	glutamate receptor, ionotropic, AMPA1 (alpha 1)	Gria1	0.6663	-1.12499574	-0.1702777
1457751_at	remodeling and spacing factor 1	Rsf1	0.66631	-1.15071898	-0.2132965
1439802_at	serine/threonine kinase 35	Stk35	0.66632	-1.11961996	-0.1678379
1456617_a_at	eukaryotic translation initiation factor 2, subunit 2 (beta)	Eif2s2	0.66633	-1.06860107	-0.0957613
1447028_at	RIKEN cDNA 4930563E22 gene	4930563E22Rik	0.66634	-1.16404951	-0.2301848
1439103_at	cell division cycle 73, Paf1/RNA polymerase II complex componen	Cdc73	0.66636	-1.13278039	-0.1819367
1453768_a_at	family with sequence similarity 110, member A	Fam110a	0.66637	1.19351923	0.23565161
1456687_at	predicted gene 3556	Gm3556	0.6664	-1.15977556	-0.2226215
1455614_at	translocase of outer mitochondrial membrane 40 homolog-like (y	Tomm40l	0.66642	1.16070085	0.20962989
1458483_at	RIKEN cDNA A330102I10 gene	A330102I10Rik	0.66643	-1.15460689	-0.217096
1445583_x_at	---	---	0.66643	-1.15861802	-0.2216342
1455849_at	neuron navigator 1	Nav1	0.66644	-1.1504396	-0.2107773
1442810_x_at	sodium channel, voltage-gated, type IX, alpha	Scn9a	0.66644	1.18336552	0.22767891
1419927_s_at	RAB interacting factor	Rabif	0.66646	-1.10384692	-0.1438898
1444185_at	---	---	0.66648	1.16490423	0.21512197
1429039_s_at	DET1 and DDB1 associated 1	Dda1	0.6665	1.10901979	0.14642987
1450067_a_at	magnesium-dependent phosphatase 1	Mdp1	0.66654	1.11895898	0.16010919
1419754_at	myosin VA	Myo5a	0.66654	-1.14034792	-0.1998326
1432028_at	RAB11 family interacting protein 2 (class I)	Rab11fip2	0.66655	1.14689129	0.19619238
1452508_x_at	parathymosin	Ptms	0.66658	1.17669809	0.22229445
1456683_at	SAFB-like, transcription modulator	Sltm	0.66661	1.13902222	0.18621171
1436954_at	WAS/WASL interacting protein family, member 1	Wipf1	0.66661	1.16726309	0.21626965
1419584_at	tetratricopeptide repeat domain 28	Ttc28	0.66662	-1.13860463	-0.1898875
1420780_at	achaete-scute complex homolog 3 (Drosophila)	Ascl3	0.66665	1.14948534	0.20021295
1438253_at	slingshot homolog 1 (Drosophila)	Ssh1	0.66668	1.12338549	0.16659143
1425800_at	RAD9 homolog B (S. cerevisiae)	Rad9b	0.66669	1.14237872	0.1893852
1452364_at	suppressor of zeste 12 homolog (Drosophila)	Suz12	0.6667	-1.14269768	-0.2110854
1456245_x_at	vesicle-associated membrane protein 3	Vamp3	0.66672	1.12120617	0.16094535
1419188_s_at	chemokine (C-C motif) ligand 27A /// chemokine (C-C motif) ligan	Ccl27a /// Ccl27b /// Gm1	0.66673	1.12415194	0.1683181
1456883_at	storkhead box 1	Stox1	0.66673	1.18228057	0.23214219
1440873_at	---	---	0.66673	-1.13432151	-0.1824716
1456790_at	zinc finger protein 800	Zfp800	0.66674	-1.17193474	-0.238494
1441344_at	ER lipid raft associated 1	Erlin1	0.66675	-1.15357243	-0.2079494
1426902_at	coenzyme Q6 homolog (yeast)	Coq6	0.66675	1.13960613	0.17932486
1443816_s_at	phosphoinositide-3-kinase, regulatory subunit 6	Pik3r6	0.66676	-1.12154201	-0.1683727
1459365_at	DIS3 mitotic control homolog (S. cerevisiae)-like 2	Dis3l2	0.66676	1.1722819	0.21785923
1457755_at	guanine nucleotide binding protein (G protein), gamma 8	Gng8	0.66677	1.1634502	0.21036687
1441317_x_at	janus kinase and microtubule interacting protein 1	Jakmip1	0.6668	1.10884518	0.14891959
1436319_at	sulfatase 1	Sulf1	0.6668	1.16013768	0.2017074
1446730_at	---	---	0.66682	1.1883228	0.2343499
1458887_at	transcription factor 21	Tcf21	0.66684	1.17759825	0.22729191
1452328_s_at	praja 2, RING-H2 motif containing	Pja2	0.66684	1.14405024	0.18868254
1427182_s_at	DNA segment, Chr 18, ERATO Doi 653, expressed	D18Ertd653e	0.66685	1.18785294	0.23434086
1425424_at	hypothetical protein LOC620031	MGC7817	0.66686	1.20365376	0.25056878
1425396_a_at	lymphocyte protein tyrosine kinase	Lck	0.66688	1.11372837	0.15253609
1422542_at	G protein-coupled receptor 34	Gpr34	0.66688	1.11871961	0.15925382
1460470_at	acyl-Coenzyme A oxidase-like	Acoxl	0.66691	1.17927902	0.22920673
1445292_at	CD300A antigen	Cd300a	0.66691	1.14142348	0.19059434
1425254_at	forkhead box N4	Foxn4	0.66691	1.13650049	0.18147585
1434958_at	sacsin	Sacs	0.66693	-1.13359037	-0.1853641
1420410_at	nuclear receptor subfamily 5, group A, member 2	Nr5a2	0.66695	-1.10873246	-0.1533047
1455594_at	exocyst complex component 3	Exoc3	0.66696	1.15879659	0.20711814
1442667_at	cell division cycle 40 homolog (yeast)	Cdc40	0.66699	-1.16079878	-0.2273309
1417774_at	N-acetylneuraminic acid synthase (sialic acid synthase)	Nans	0.66699	1.1217907	0.16258109
1427651_x_at	histocompatibility 2, D region locus 1 /// histocompatibility 2, D re	H2-D1 /// H2-L	0.667	1.15769362	0.20947047
1433981_s_at	K(lysine) acetyltransferase 5	Kat5	0.667	1.12390678	0.16568663
1428154_s_at	phosphatidic acid phosphatase type 2 domain containing 1B	Ppapdc1b	0.66701	1.13912203	0.18785866
1437985_a_at	RIKEN cDNA 2310061I04 gene	2310061I04Rik	0.66701	-1.11832831	-0.1701081
1420820_at	RIKEN cDNA 2900073G15 gene	2900073G15Rik	0.66702	-1.09001491	-0.1268956
1453953_at	RIKEN cDNA 9130015A21 gene	9130015A21Rik	0.66703	-1.14873737	-0.2005727
1436191_at	AT rich interactive domain 4A (RBP1-like)	Arid4a	0.66704	-1.13455314	-0.1824507
1420750_at	RIKEN cDNA 1110006O24 gene	1110006O24Rik	0.66704	1.15224692	0.20265424
1434745_at	cyclin D2	Ccnd2	0.66705	-1.14252738	-0.1972635
1429327_at	serologically defined colon cancer antigen 1	Sdccag1	0.66706	-1.15348699	-0.2178018
1429274_at	LY6/PLAUR domain containing 6B	Lypd6b	0.66707	-1.13768301	-0.1874136
1430090_at	coiled coil domain containing 28B	Ccdc28b	0.66711	-1.16232933	-0.2213477
1429630_at	enhancer of zeste homolog 1 (Drosophila)	Ezh1	0.66711	1.15923853	0.20892741
1424584_a_at	RAN binding protein 10	Ranbp10	0.66711	1.1148896	0.1562714
1452511_at	SRY-box containing gene 5	Sox5	0.66712	1.16906763	0.22129988
1418251_at	tubby-like protein 3	Tulp3	0.66714	-1.25616326	-0.573846
1421153_at	lysyl oxidase-like 4	Loxl4	0.66715	-1.16003131	-0.2192495
1455815_a_at	tyrosine 3-monooxygenase/tryptophan 5-monooxygenase activa	Ywhab	0.66715	-1.13254897	-0.1954054
1427877_at	zinc finger CCCH-type containing 15	Zc3h15	0.66717	-1.10258689	-0.1433752
1433466_at	expressed sequence AI467606	AI467606	0.66717	1.11671073	0.15905066
1426887_at	nudix (nucleoside diphosphate linked moiety X)-type motif 11	Nudt11	0.66719	1.1539987	0.20078481
1458028_at	RIKEN cDNA 1810012P15 gene	1810012P15Rik	0.66719	-1.15674922	-0.2164846
1456992_at	ubiquitin protein ligase E3C	Ube3c	0.66719	1.1503592	0.20158028
1424109_a_at	glyoxalase 1	Glo1	0.66721	1.0777959	0.10762175
1422520_at	neurofilament, medium polypeptide	Nefm	0.66723	1.127914	0.16741117
1423704_at	phospholipase A2, group XV	Pla2g15	0.66726	1.13161092	0.17449242
1441246_s_at	dihydropyrimidinase-like 5	Dpysl5	0.66727	1.15146754	0.19947327
1453950_a_at	X-ray repair complementing defective repair in Chinese hamster c	Xrcc2	0.66727	1.1598831	0.2057063
1451065_a_at	DEAD (Asp-Glu-Ala-Asp) box polypeptide 39	Ddx39	0.66727	-1.15210794	-0.2331926
1430981_s_at	GC-rich promoter binding protein 1	Gpbp1	0.66728	-1.12375626	-0.1793544
1453791_at	RIKEN cDNA C130071C03 gene	C130071C03Rik	0.6673	1.11216223	0.15172616
1455052_a_at	mitotic spindle organizing protein 1	Mzt1	0.6673	-1.09214177	-0.127573
1430260_at	---	---	0.66732	-1.14147211	-0.1957238
1418647_at	guanine nucleotide binding protein-like 1	Gnl1	0.66732	1.17227198	0.22235068

1419683_at	transformation related protein 53 regulating kinase	Trp53rk	0.66732	1.14536134	0.1922304
1456704_at	RIKEN cDNA 9530010C24 gene	9530010C24Rik	0.66732	1.16351389	0.21210962
1450133_at	UDP glucuronosyltransferase 2 family, polypeptide A3	Ugt2a3	0.66733	1.12796426	0.17263802
1416671_a_at	mucolipin 1	Mcoln1	0.66734	1.12834916	0.1713517
1447063_at	RIKEN cDNA 1700017B05 gene	1700017B05Rik	0.66734	-1.15359825	-0.2107722
1459694_at	expressed sequence AI428898	AI428898	0.66735	1.16783046	0.22054359
1424737_at	thyroid hormone responsive SPOT14 homolog (Rattus)	Thrsp	0.66737	1.16973419	0.22087817
1442216_at	---	---	0.66738	-1.17847324	-0.2590365
1436001_at	RIKEN cDNA 2310028O11 gene	2310028O11Rik	0.66739	-1.13490153	-0.1840773
1450682_at	fatty acid binding protein 6, ileal (gastrotropin)	Fabp6	0.6674	1.1606433	0.21223444
1451137_a_at	bromodomain containing 8	Brd8	0.6674	1.10112137	0.13881505
1442354_at	RIKEN cDNA B230325K18 gene	B230325K18Rik	0.66742	1.21971768	0.26393579
1419154_at	transmembrane protease, serine 2	Tmprss2	0.66743	1.15922788	0.21025052
1427071_at	F-box protein 42	Fbxo42	0.66744	1.13074007	0.17427399
1430619_a_at	mevalonate kinase	Mvk	0.66744	1.12521472	0.16876409
1448815_at	8-oxoguanine DNA-glycosylase 1	Ogg1	0.66744	1.13501901	0.18145456
1423602_at	TNF receptor-associated factor 1	Traf1	0.66744	1.19545409	0.24346187
1420171_s_at	myosin, heavy polypeptide 9, non-muscle	Myh9	0.66745	-1.11213839	-0.1570029
1424476_at	calcium/calmodulin-dependent protein kinase kinase 2, beta	Camkk2	0.66746	-1.23439094	-0.3960541
1422521_at	dynactin 1	Dctn1	0.66746	1.10821621	0.14782092
1452768_at	testis expressed gene 261	Tex261	0.66748	-1.13218632	-0.1821327
1428493_at	signal-induced proliferation-associated 1 like 3	Sipa1l3	0.6675	-1.12682342	-0.175766
1426510_at	saccharopine dehydrogenase (putative)	Sccpdh	0.66751	1.13319814	0.17950048
1416561_at	glutamic acid decarboxylase 1	Gad1	0.66751	1.2084555	0.25763894
1426904_s_at	DnaJ (Hsp40) homolog, subfamily C, member 10	Dnajc10	0.66752	1.11294394	0.15135945
1450124_a_at	ATPase, Ca++ transporting, ubiquitous	Atp2a3	0.66753	-1.12822479	-0.1744459
1432901_at	enhancer trap locus 4	Etl4	0.66753	-1.14194858	-0.1929963
1430261_at	RIKEN cDNA 1700024J04 gene	1700024J04Rik	0.66753	-1.15189947	-0.2103153
1448627_s_at	PDZ binding kinase	Pbk	0.66753	1.10913859	0.14654674
1451163_at	Terf1 (TRF1)-interacting nuclear factor 2	Tinf2	0.66756	1.09925531	0.13650082
1426054_at	neuropeptide Y receptor Y1	Npy1r	0.66759	-1.15836998	-0.2163214
1424936_a_at	dynein, axonemal, heavy chain 8	Dnahc8	0.66759	1.12072556	0.16116844
1440516_at	SLIT and NTRK-like family, member 4	Slitrk4	0.6676	1.13178063	0.17771357
1421423_at	prolactin family 7, subfamily c, member 1	Prl7c1	0.66762	1.19701246	0.24546465
1427587_at	zinc finger protein 28	Zfp28	0.66763	1.13996701	0.18828721
1432000_a_at	death effector domain-containing	Dedd	0.66764	1.12197818	0.16517071
1454952_s_at	non-SMC condensin II complex, subunit D3	Ncapd3	0.66764	-1.11174669	-0.15778
1444024_at	excision repair cross-complementing rodent repair deficiency com	Ercc6l	0.66765	-1.14820362	-0.2102463
1434682_at	zinc finger protein 770	Zfp770	0.66765	-1.1111256	-0.152563
1432164_a_at	glycine cleavage system protein H (aminomethyl carrier)	Gcsh	0.66765	-1.09081403	-0.1255366
1454234_at	Williams-Beuren syndrome chromosome region 28 (human)	Wbscr28	0.66766	-1.14231317	-0.1970284
1454641_at	CGG triplet repeat binding protein 1	Cggbp1	0.66767	-1.09571423	-0.1326526
1416951_a_at	ATPase, H+ transporting, lysosomal V1 subunit D	Atp6v1d	0.66767	-1.10167926	-0.141259
1459320_at	---	---	0.66768	1.14928936	0.19932605
1430130_at	von Willebrand factor C and EGF domains	Vwce	0.66768	-1.15387629	-0.2122657
1437345_a_at	Bernardinelli-Seip congenital lipodystrophy 2 homolog (human)	Bscl2	0.66768	1.12605348	0.16536988
1421797_a_at	sorting nexin 12	Snx12	0.66768	1.12178593	0.16344682
1419405_at	neuromedin B	Nmb	0.6677	1.14253123	0.18685715
1422676_at	SWI/SNF related, matrix associated, actin dependent regulator of	Smarce1	0.66771	1.11162736	0.1522246
1435299_at	cyclin-dependent kinase 7	Cdk7	0.66773	1.12270494	0.16655991
1426521_at	RIKEN cDNA D230025D16 gene	D230025D16Rik	0.66773	-1.11554911	-0.16015
1446662_at	RIKEN cDNA 4930562C15 gene	4930562C15Rik	0.66774	1.151525	0.19972206
1423497_at	kelch-like 10 (Drosophila)	Klhl10	0.66774	1.16699611	0.21604076
1442446_at	Zinc finger protein 523	Zfp523	0.66775	-1.15928155	-0.219788
1447497_at	---	---	0.66776	1.21705435	0.25329956
1444991_at	---	---	0.66776	-1.1444912	-0.1981163
1441453_at	RIKEN cDNA 8030453O22 gene	8030453O22Rik	0.66777	-1.15546123	-0.211232
1443853_x_at	Scm-like with four mbt domains 1	Sfmbt1	0.66778	-1.19156428	-0.2841549
1422751_at	transducin-like enhancer of split 1, homolog of Drosophila E(spl)	Tle1	0.6678	1.12751129	0.17198345
1430932_at	solute carrier family 9 (sodium/hydrogen exchanger), member 8	Slc9a8	0.66783	-1.16786788	-0.2369174
1438411_at	G protein-coupled receptor 81	Gpr81	0.66788	-1.15416913	-0.2127305
1438243_at	hypothetical LOC100502687	LOC100502687	0.66791	-1.15586243	-0.2093448
1423964_at	cleavage and polyadenylation specific factor 3-like	Cpsf3l	0.66797	1.09692883	0.13342429
1434026_at	ATPase, class I, type 8B, member 2	Atp8b2	0.668	1.11462563	0.1551155
1459036_at	---	---	0.66802	1.12922589	0.16787456
1457478_at	---	---	0.66805	-1.15797662	-0.2176377
1453572_a_at	proteolipid protein 2	Plp2	0.66813	1.09471221	0.130376
1424285_s_at	ADP-ribosylation factor-like 6 interacting protein 4	Arl6ip4	0.66815	1.10562275	0.14347005
1423015_at	kin of IRRE like (Drosophila)	Kirrel	0.66816	1.15772586	0.21055275
1431048_at	serine/threonine/tyrosine interaction protein	Styx	0.66818	1.1634761	0.21161532
1452607_at	RIKEN cDNA 2610030H06 gene	2610030H06Rik	0.66819	-1.12663684	-0.1800094
1416766_at	MOCO sulphurase C-terminal domain containing 2	Mosc2	0.66819	1.12772656	0.16814779
1454347_at	RIKEN cDNA 4930540E01 gene	4930540E01Rik	0.66819	-1.14063874	-0.1936152
1438292_x_at	adenosine kinase	Adk	0.66824	-1.13207723	-0.1939745
1423194_at	Rho GTPase activating protein 5	Arhgap5	0.66824	-1.15642657	-0.2303699
1449141_at	filamin binding LIM protein 1	Fblim1	0.66825	1.1157534	0.1528696
1455152_at	expressed sequence AI462493	AI462493	0.66826	1.11815777	0.15567024
1456069_at	dystrobrevin alpha	Dtna	0.66829	1.11767387	0.1600468
1448419_at	processing of precursor 4, ribonuclease P/MRP family, (S. cerevisi	Pop4	0.6683	1.10135768	0.13846057
1453885_at	solute carrier family 24 (sodium/potassium/calcium exchanger), r	Slc24a2	0.6683	-1.14905246	-0.2044814
1457922_at	Uracil DNA glycosylase	Ung	0.66831	1.15525692	0.20139411
1441703_at	zinc finger protein 787	Zfp787	0.66832	1.16143975	0.21277772
1437272_at	dpy-19-like 2 (C. elegans)	Dpy19l2	0.66832	1.1230447	0.16467232
1449836_x_at	BCL2-interacting killer	Bik	0.66832	1.12176168	0.16552372
1427742_a_at	Kruppel-like factor 6	Klf6	0.66832	-1.13865313	-0.1899923
1453361_at	helicase, lymphoid specific	Hells	0.66833	-1.13661854	-0.1854568
1455593_at	apolipoprotein B	Apob	0.66833	1.16971184	0.21752587
1435979_a_at	myosin XVB	Myo15b	0.66834	-1.15102637	-0.2082249
1453979_at	RNA binding motif protein 12B	Rbm12b	0.66836	1.14662647	0.1958136
1454269_s_at	RIKEN cDNA 4930519L02 gene	4930519L02Rik	0.66838	1.12904691	0.17345994
1455006_at	RIKEN cDNA 2310016M24 gene	2310016M24Rik	0.66842	1.11011422	0.14699166
1428004_at	small nuclear ribonucleoprotein 25 (U11/U12)	Snrnp25	0.66842	1.10414639	0.14182577
1433836_a_at	RIKEN cDNA 8430408G22 gene	8430408G22Rik	0.66842	-1.10668051	-0.1473008
1430977_at	family with sequence similarity 159, member B	Fam159b	0.66843	-1.13168255	-0.1800679

1429695_at	izumo sperm-egg fusion 1	Izumo1	0.66843	-1.1533573	-0.2109091
1420688_a_at	sarcoglycan, epsilon	Sgce	0.66845	1.1401729	0.18901328
1428986_at	solute carrier family 17 (sodium-dependent inorganic phosphate c	Slc17a7	0.66846	1.13980744	0.18704949
1420944_at	zinc finger protein 185	Zfp185	0.66846	1.12478116	0.16846694
1435762_at	phosphofurin acidic cluster sorting protein 1	Pacs1	0.66846	1.12206303	0.16386073
1457027_at	dehydrogenase E1 and transketolase domain containing 1	Dhtkd1	0.66847	-1.12816779	-0.1769259
1444084_at	proline rich Gla (G-carboxyglutamic acid) 4 (transmembrane)	Prrg4	0.66847	-1.14517107	-0.1975137
1415961_at	integral membrane protein 2C	Itm2c	0.66847	1.11163608	0.14984518
1433485_x_at	G protein-coupled receptor 56	Gpr56	0.6685	1.14206268	0.19022067
1442158_at	microtubule associated serine/threonine kinase family member 4	Mast4	0.66852	-1.11489588	-0.1603522
1451185_at	splicing factor 3b, subunit 5	Sf3b5	0.66853	1.10732967	0.1432145
1431137_at	RUN and SH3 domain containing 1	Rusc1	0.66853	1.15154137	0.20315428
1420085_at	fibroblast growth factor 4	Fgf4	0.66854	1.1207742	0.15846057
1452643_at	grainyhead-like 2 (Drosophila)	Grhl2	0.66855	-1.14601466	-0.2023776
1439645_at	adrenergic receptor, alpha 2b	Adra2b	0.66855	1.18171981	0.23218019
1420953_at	adducin 1 (alpha)	Add1	0.66855	1.11828989	0.15850244
1458801_at	expressed sequence AU022320	AU022320	0.66855	-1.15759586	-0.2175943
1420603_s_at	retinoic acid early transcript 1, alpha /// retinoic acid early transcr	Raet1a /// Raet1b /// Rae	0.66857	-1.11869408	-0.1677129
1421485_at	tau tubulin kinase 2	Ttbk2	0.66857	-1.15462719	-0.2084173
1438115_a_at	solute carrier family 9 (sodium/hydrogen exchanger), member 3 r	Slc9a3r1	0.66857	1.11006432	0.14702726
1420452_at	keratin associated protein 5-2	Krtap5-2	0.66857	-1.15239724	-0.2090542
1440375_at	---	---	0.66857	1.15896541	0.20752272
1432802_at	RIKEN cDNA 9230106L01 gene	9230106L01Rik	0.66858	1.16089442	0.21045119
1430991_at	RIKEN cDNA 1810014B01 gene	1810014B01Rik	0.66858	-1.14555882	-0.1966825
1417184_s_at	hemoglobin, beta adult major chain /// hemoglobin, beta adult m	Hbb-b1 /// Hbb-b2 /// LO	0.66858	-1.12695215	-0.1743422
1422297_at	prefoldin 5	Pfdn5	0.66859	1.10705956	0.14654629
1436090_at	ectonucleotide pyrophosphatase/phosphodiesterase 6	Enpp6	0.66859	-1.16548808	-0.2312349
1442019_at	---	---	0.6686	1.15882868	0.21017055
1448696_at	hephaestin	Heph	0.66864	1.13069228	0.17318902
1423823_at	MRT4, mRNA turnover 4, homolog (S. cerevisiae)	Mrto4	0.66865	1.11093037	0.147299
1444511_at	solute carrier family 41, member 1	Slc41a1	0.66866	-1.16396499	-0.2309049
1428871_at	male-specific lethal 1 homolog (Drosophila)	Msl1	0.66866	1.16012775	0.20203881
1420297_at	---	---	0.66866	1.13191148	0.17460127
1457419_s_at	---	---	0.66868	1.14451646	0.19413237
1440708_at	myosin, heavy polypeptide 9, non-muscle	Myh9	0.66868	-1.16028981	-0.2227204
1421998_at	torsin family 3, member A	Tor3a	0.66868	1.17216412	0.22247936
1459793_s_at	LYR motif containing 5	Lyrm5	0.6687	-1.17015775	-0.2454231
1458817_at	---	---	0.66872	-1.15613562	-0.2167019
1458781_at	---	---	0.66872	-1.15748847	-0.2175577
1444784_at	RIKEN cDNA 4930564K09 gene	4930564K09Rik	0.66874	1.17771518	0.2249759
1427853_a_at	predicted gene 9817	Gm9817	0.66874	-1.14282479	-0.2012246
1419864_x_at	transportin 1	Tnpo1	0.66876	1.20887735	0.2512624
1421343_at	---	---	0.66876	-1.15032246	-0.2036651
1452161_at	TCDD-inducible poly(ADP-ribose) polymerase	Tiparp	0.66877	-1.1261143	-0.1801572
1444422_at	protocadherin 19	Pcdh19	0.66877	-1.1330439	-0.1810666
1423042_at	DEAD/H (Asp-Glu-Ala-Asp/His) box polypeptide 3, X-linked	Ddx3x	0.66878	-1.09091898	-0.1280439
1451169_at	nodal modulator 1	Nomo1	0.66879	1.13874601	0.18086984
1441752_at	ADP-ribosyltransferase 3	Art3	0.66879	1.14107559	0.18626523
1423628_s_at	protocadherin gamma subfamily A, 1 /// protocadherin gamma s	Pcdhga1 /// Pcdhga10 ///	0.66881	1.10291698	0.14119311
1437657_at	S phase cyclin A-associated protein in the ER	Scaper	0.66882	-1.16303061	-0.2296615
1417363_at	zinc finger protein 61	Zfp61	0.66883	1.13816063	0.18535919
1455958_s_at	PTC7 protein phosphatase homolog (S. cerevisiae)	Pptc7	0.66883	1.12350233	0.16450399
1444740_at	LAG1 homolog, ceramide synthase 3	Lass3	0.66884	1.12075277	0.16340134
1458987_at	cyclin-dependent kinase 7	Cdk7	0.66885	1.15772682	0.2011614
1419179_at	thioredoxin-like 4A	Txnl4a	0.66885	1.14134017	0.18891458
1430431_at	RIKEN cDNA 4933407H18 gene	4933407H18Rik	0.66886	-1.15159713	-0.2092041
1432311_at	transmembrane protein 168	Tmem168	0.66886	-1.15777374	-0.2202743
1423933_a_at	RIKEN cDNA 1600029D21 gene	1600029D21Rik	0.66887	1.12842662	0.17314893
1433060_at	RIKEN cDNA 6230426I18 gene	6230426I18Rik	0.66887	-1.1624164	-0.2300455
1436837_at	maelstrom homolog (Drosophila)	Mael	0.66888	-1.13186454	-0.1833016
1456625_at	aminoadipate-semialdehyde dehydrogenase-phosphopantetheir	Aasdhppt	0.66888	-1.13381539	-0.1864991
1448539_a_at	aspartoacylase (aminoacylase) 3	Acy3	0.66889	1.14354628	0.19144495
1419526_at	Gardner-Rasheed feline sarcoma viral (Fgr) oncogene homolog	Fgr	0.6689	1.14533211	0.19154832
1447211_at	nuclear receptor interacting protein 1	Nrip1	0.66891	-1.17078178	-0.2411635
1447439_at	RIKEN cDNA 1700023E05 gene	1700023E05Rik	0.66892	-1.16558745	-0.2333174
1443191_at	---	---	0.66893	1.15217036	0.20071157
1447217_at	ubiquitin-like, containing PHD and RING finger domains 2	Uhrf2	0.66894	-1.12452662	-0.1738183
1448319_at	aldo-keto reductase family 1, member B3 (aldose reductase)	Akr1b3	0.66895	1.08419735	0.11614368
1450936_a_at	deoxyribonuclease 1-like 2	Dnase1l2	0.66896	-1.14795132	-0.1990867
1429952_at	motile sperm domain containing 4	Mospd4	0.66898	-1.16466583	-0.2292599
1424103_at	autophagy-related 4B (yeast)	Atg4b	0.66899	1.13026896	0.17331452
1448002_x_at	RIKEN cDNA 2610001J05 gene	2610001J05Rik	0.66899	1.13907613	0.18063413
1423277_at	protein tyrosine phosphatase, receptor type, K	Ptprk	0.66901	1.10951341	0.14914213
1429369_at	transportin 3	Tnpo3	0.66904	-1.14958019	-0.2237693
1439597_at	---	---	0.66905	1.14392936	0.1894854
1452842_at	heat shock protein, alpha-crystallin-related, B9	Hspb9	0.66909	1.15239898	0.20450343
1416858_a_at	FK506 binding protein 3	Fkbp3	0.66909	-1.13046463	-0.1945434
1451003_at	TGF-beta activated kinase 1/MAP3K7 binding protein 2	Tab2	0.66911	-1.13999269	-0.2030318
1438121_at	Sec24 related gene family, member A (S. cerevisiae)	Sec24a	0.66912	-1.19092235	-0.2844415
1423441_at	transcription factor B2, mitochondrial	Tfb2m	0.66913	-1.10637604	-0.1460418
1446860_at	---	---	0.66921	1.11679536	0.15783395
1434443_at	anaphase promoting complex subunit 1	Anapc1	0.66925	1.13788359	0.17898041
1457670_s_at	lamin A	Lmna	0.66925	1.16779492	0.21093515
1457353_at	Translocase of outer mitochondrial membrane 70 homolog A (yea	Tomm70a	0.66947	-1.11744174	-0.1613233
1455120_at	4-hydroxyphenylpyruvate dioxygenase-like	Hpdl	0.66955	1.14514626	0.18788261
1441266_at	Striatin, calmodulin binding protein 3	Strn3	0.6699	1.14711082	0.19222962
1440793_at	RIKEN cDNA 1700029M03 gene	1700029M03Rik	0.67004	1.1778418	0.21606876
1427145_at	IQ motif and Sec7 domain 1	Iqsec1	0.67018	-1.14448296	-0.1969331
1422826_at	insulin-like growth factor binding protein, acid labile subunit	Igfals	0.67029	1.13251272	0.17802562
1432379_at	RIKEN cDNA 4930590A17 gene	4930590A17Rik	0.67031	-1.10600759	-0.1457465
1435550_at	myeloid/lymphoid or mixed-lineage leukemia 2	Mll2	0.67051	-1.10980066	-0.1530805
1436036_at	Wolf-Hirschhorn syndrome candidate 1 (human)	Whsc1	0.67054	1.13606498	0.17889072
1429824_at	RIKEN cDNA 4930550C14 gene	4930550C14Rik	0.67086	-1.13871259	-0.1889273
1442020_at	predicted gene 12863	Gm12863	0.67088	-1.14440173	-0.1952049

1440299_at	RIKEN cDNA E330016A19 gene	E330016A19Rik	0.67089	1.16545591	0.21492253
1419129_at	solute carrier family 22 (organic cation transporter), member 13	Slc22a13	0.67095	1.13686764	0.1837274
1421963_a_at	cell division cycle 25 homolog B (S. pombe)	Cdc25b	0.67098	1.10750926	0.14730062
1416287_at	regulator of G-protein signaling 4	Rgs4	0.671	-1.16003604	-0.2216609
1427943_at	acylphosphatase 2, muscle type	Acyp2	0.67102	1.13922775	0.18389333
1449175_at	G-protein coupled receptor 65	Gpr65	0.6714	1.12211603	0.16452066
1432023_a_at	solute carrier family 17, member 9	Slc17a9	0.67142	1.1294074	0.17147705
1446575_at	expressed sequence C77583	C77583	0.67143	-1.1494613	-0.2069839
1428433_at	homeodomain interacting protein kinase 2	Hipk2	0.6715	-1.14252763	-0.2012108
1427874_at	ring finger protein 114	Rnf114	0.67155	1.10873395	0.14852686
1457451_at	---	---	0.67161	1.18106048	0.22923664
1456141_x_at	BRCA1/BRCA2-containing complex, subunit 3	Brcc3	0.67165	-1.15032839	-0.204915
1420726_x_at	trimethyllysine hydroxylase, epsilon	Tmlhe	0.67166	-1.12216259	-0.1688441
1444229_at	nuclear receptor subfamily 2, group F, member 2	Nr2f2	0.67167	1.21817121	0.2591924
1443175_at	RIKEN cDNA A830010M09 gene	A830010M09Rik	0.6717	1.16183792	0.20488815
1417205_at	KDEL (Lys-Asp-Glu-Leu) endoplasmic reticulum protein retention r	Kdelr2	0.6717	1.12588644	0.16653839
1422172_x_at	RIKEN cDNA 2810408B13 gene	2810408B13Rik	0.6717	-1.14991502	-0.2137523
1445926_at	---	---	0.67171	1.16807539	0.21645536
1439821_at	Lrp2 binding protein	Lrp2bp	0.67172	1.21519375	0.25705689
1433871_at	R3H domain 1 (binds single-stranded nucleic acids)	R3hdm1	0.67173	-1.11351149	-0.1597065
1447731_at	paternally expressed 3	Peg3	0.67174	1.12052226	0.1600545
1452221_a_at	CXXC finger 1 (PHD domain)	Cxxc1	0.67177	1.11319297	0.15316824
1431811_a_at	F-box protein 34	Fbxo34	0.6718	1.11435955	0.15501563
1446586_at	RIKEN cDNA 8030451A03 gene	8030451A03Rik	0.67182	-1.1564104	-0.212496
1430293_a_at	ferredoxin 1-like /// zinc finger, GATA-like protein 1	Fdx1l /// Zglp1	0.67182	1.12648799	0.16483967
1439389_s_at	myeloid-associated differentiation marker	Myadm	0.67183	-1.10375994	-0.146725
1441911_x_at	phosphoribosylglycinamide formyltransferase	Gart	0.67183	-1.15899597	-0.2286717
1434036_at	metastasis suppressor 1	Mtss1	0.67184	-1.10742886	-0.1505546
1424768_at	caldesmon 1	Cald1	0.67184	-1.14862927	-0.2146374
1422064_a_at	zinc finger and BTB domain containing 20	Zbtb20	0.67185	1.1593354	0.20967612
1429577_at	IQ motif containing F4	Iqcf4	0.6719	1.16445303	0.21138846
1419141_at	crystallin, gamma D	Crygd	0.6719	1.15542264	0.20404495
1416454_s_at	actin, alpha 2, smooth muscle, aorta	Acta2	0.67191	-1.12960934	-0.1758989
1449336_a_at	STE20-like kinase (yeast)	Slk	0.67192	-1.13232171	-0.1850786
1460017_at	predicted gene 5595	Gm5595	0.67192	-1.11736549	-0.1607153
1426859_at	inhibin beta-B	Inhbb	0.67196	-1.11816443	-0.1650249
1431932_s_at	tripartite motif-containing 44	Trim44	0.67197	-1.12564733	-0.1734087
1427147_at	RIKEN cDNA F730047E07 gene	F730047E07Rik	0.67198	1.11175034	0.15168251
1460561_x_at	selenoprotein W, muscle 1	Sepw1	0.67199	-1.10978822	-0.1561017
1438770_at	Predicted gene 266	Gm266	0.67207	1.14829659	0.19803813
1424581_at	SH3 and cysteine rich domain 2	Stac2	0.67208	1.1366865	0.18335613
1452991_at	chromodomain helicase DNA binding protein 2	Chd2	0.6721	-1.15039845	-0.2053952
1434482_at	DNA segment, Chr 4, ERATO Doi 22, expressed	D4Ertd22e	0.67211	1.10923908	0.14678666
1418207_at	FXYD domain-containing ion transport regulator 4	Fxyd4	0.67212	-1.15863623	-0.2297784
1449106_at	glutathione peroxidase 3	Gpx3	0.67218	1.1148686	0.15563752
1441447_at	nucleolar protein 4	Nol4	0.67223	-1.12981634	-0.1816726
1459804_at	CREB binding protein	Crebbp	0.67225	-1.15622307	-0.2193933
1458703_at	---	---	0.6723	1.14678439	0.19507215
1428081_at	kelch-like 21 (Drosophila)	Klhl21	0.67233	1.13074322	0.17355708
1432724_at	RIKEN cDNA 4930445G23 gene	4930445G23Rik	0.67238	-1.15679306	-0.2191308
1424238_at	sirtuin 7 (silent mating type information regulation 2, homolog) 7	Sirt7	0.67252	1.12919281	0.17007491
1450075_at	polymerase (DNA directed), eta (RAD 30 related)	Polh	0.67254	1.14530269	0.19518626
1434400_at	TGFB-induced factor homeobox 2	Tgif2	0.67255	1.10135486	0.13781487
1451156_s_at	very low density lipoprotein receptor	Vldlr	0.67265	-1.14861347	-0.2089046
1436497_at	hypothetical LOC100503785	LOC100503785	0.67293	1.12916066	0.17332873
1422604_at	urate oxidase	Uox	0.67295	1.16849575	0.21570693
1452534_a_at	high mobility group box 2	Hmgb2	0.67308	-1.09804057	-0.141234
1437480_at	family with sequence similarity 33, member A	Fam33a	0.67309	-1.11405876	-0.1595376
1434497_at	RIKEN cDNA 4933431E20 gene	4933431E20Rik	0.67323	1.16773368	0.21195206
1456516_x_at	UDP-N-acetylhexosamine pyrophosphorylase-like /// UDP-N-acet	LOC640502 /// Uap1	0.67324	-1.12944682	-0.1812263
1451017_at	ERGIC and golgi 3	Ergic3	0.67329	1.1292416	0.17055932
1426503_a_at	ring finger protein 121	Rnf121	0.67334	1.11281801	0.15416797
1457760_at	RIKEN cDNA A930004J17 gene	A930004J17Rik	0.67335	-1.15388262	-0.2116852
1451062_a_at	peroxisomal biogenesis factor 5-like	Pex5l	0.67337	-1.15471952	-0.2083312
1423496_a_at	immunoglobulin superfamily, DCC subclass, member 3	Igdcc3	0.67337	-1.14169648	-0.1925529
1428627_at	zinc finger and BTB domain containing 49	Zbtb49	0.67341	1.13131885	0.17482575
1430733_at	RIKEN cDNA 4932441P12 gene	4932441P12Rik	0.67343	1.12738558	0.16855792
1431190_x_at	fumarylacetoacetate hydrolase domain containing 2A	Fahd2a	0.67345	1.14036826	0.18522139
1441888_x_at	ZW10 homolog (Drosophila), centromere/kinetochore protein	Zw10	0.67349	1.19750376	0.24575797
1452042_a_at	transmembrane protein 144	Tmem144	0.67365	1.15371117	0.20240721
1452606_at	meiotic nuclear divisions 1 homolog (S. cerevisiae)	Mnd1	0.67368	1.12878857	0.16795063
1447059_at	---	---	0.6737	1.12394132	0.16852731
1447345_at	---	---	0.6737	1.1325919	0.17548753
1432265_at	RIKEN cDNA 4930445B16 gene	4930445B16Rik	0.6738	-1.1519945	-0.2121973
1417964_at	adaptor-related protein complex 3, delta 1 subunit	Ap3d1	0.6738	1.10555492	0.14357863
1438442_at	suppressor of IKBKE 1	Sike1	0.67386	-1.17619737	-0.2573986
1453624_at	tripartite motif-containing 6	Trim6	0.67391	-1.17791563	-0.2612301
1446425_at	RIKEN cDNA 4732418C07 gene	4732418C07Rik	0.67393	-1.15042063	-0.2099584
1430039_at	CDK5 regulatory subunit associated protein 1-like 1	Cdkal1	0.67396	-1.18223992	-0.2697083
1456140_at	zinc finger protein of the cerebellum 5	Zic5	0.67397	1.1590806	0.20371716
1417222_a_at	transmembrane protein 123	Tmem123	0.674	-1.15434339	-0.2240975
1456435_at	MORN repeat containing 1	Morn1	0.67401	1.17129738	0.22029178
1451514_at	ubiquitin specific peptidase 43	Usp43	0.67401	1.15291111	0.20172831
1452151_at	GRAM domain containing 4	Gramd4	0.67404	1.11804565	0.16053606
1416795_at	crystallin, lambda 1	Cryl1	0.67406	1.13288038	0.17907704
1438069_a_at	RNA binding motif protein 5	Rbm5	0.67412	1.13490065	0.18239067
1440348_at	zinc finger, FYVE domain containing 9	Zfyve9	0.67418	-1.14976098	-0.212063
1435105_at	ring finger protein 208	Rnf208	0.67434	1.16231092	0.20504596
1433028_at	RIKEN cDNA 4833411I10 gene	4833411I10Rik	0.67442	1.19434112	0.2392552
1441973_at	zinc finger protein 295	Zfp295	0.67448	-1.15795085	-0.2230345
1425989_a_at	eyes absent 3 homolog (Drosophila)	Eya3	0.67449	-1.22463488	-0.4149982
1419445_s_at	predicted gene 10094 /// Sin3-associated polypeptide 18	Gm10094 /// Sap18	0.67452	1.10932191	0.14564704
1424321_at	replication factor C (activator 1) 4	Rfc4	0.6751	-1.09759322	-0.1369002
1424312_at	adiponectin receptor 1	Adipor1	0.67513	1.12718558	0.16900985

1416775_at	ATP5S-like	Atp5sl	0.67514	1.10465231	0.14301698
1456426_at	C-type lectin domain family 2, member i	Clec2i	0.67518	1.10499438	0.14361556
1420362_a_at	BCL2-interacting killer	Bik	0.67522	1.17634475	0.22201052
1449222_at	Epstein-Barr virus induced gene 3	Ebi3	0.67522	1.17114593	0.21858271
1424377_at	ribosomal L24 domain containing 1	Rsl24d1	0.67525	1.09754171	0.13377916
1423011_at	MDS1 and EVI1 complex locus	Mecom	0.67575	1.12977564	0.17393119
1430584_s_at	carbonic anhydrase 3	Car3	0.67582	-1.18877486	-0.2856664
1459489_at	---	---	0.67583	-1.16648684	-0.2401158
1456927_at	microtubule associated serine/threonine kinase 2	Mast2	0.67583	1.25616032	0.29136953
1459530_at	---	---	0.67586	1.17525108	0.22200184
1434150_a_at	HIG1 domain family, member 1C /// methyltransferase like 7A1 /	Higd1c /// Mettl7a1 /// M	0.67587	1.17201086	0.21352101
1458750_at	hypothetical LOC228025	LOC228025	0.6759	-1.14335078	-0.1941758
1455972_x_at	hydroxyacyl-Coenzyme A dehydrogenase	Hadh	0.67596	1.0929966	0.12742664
1424645_at	trinucleotide repeat containing 6C	Tnrc6c	0.67597	-1.12062994	-0.1690962
1419235_s_at	helicase (DNA) B	Helb	0.67597	1.11873835	0.15970398
1455981_at	predicted gene 13654 /// ribosomal protein S6 pseudogene /// 4(	Gm13654 /// Gm16409 //	0.67608	-1.11557359	-0.164749
1454914_at	RIKEN cDNA 2610101N10 gene	2610101N10Rik	0.67609	-1.09748238	-0.1378315
1424823_s_at	SLAIN motif family, member 1	Slain1	0.67623	1.22320839	0.26075972
1439017_x_at	adiponectin receptor 1	Adipor1	0.67632	1.09828813	0.13405452
1458754_at	---	---	0.67642	1.11886356	0.15582263
1424051_at	collagen, type IV, alpha 2	Col4a2	0.67646	1.13414034	0.17772892
1421083_x_at	barrier to autointegration factor 1	Banf1	0.67657	1.10470577	0.13936832
1454976_at	superoxide dismutase 2, mitochondrial	Sod2	0.67668	-1.10665505	-0.1464078
1417499_at	translocase of inner mitochondrial membrane 10 homolog (yeast	Timm10	0.67671	1.0996732	0.13497799
1457924_at	muscleblind-like 1 (Drosophila)	Mbnl1	0.67673	-1.14231579	-0.1927585
1454680_at	DNA segment, Chr 5, ERATO Doi 579, expressed	D5Ertd579e	0.6768	-1.1145465	-0.1565096
1443233_at	---	---	0.67682	-1.12421965	-0.1703213
1452597_at	RIKEN cDNA 2310061C15 gene	2310061C15Rik	0.67683	1.11853221	0.15718056
1452239_at	gene trap ROSA 26, Philippe Soriano	Gt(ROSA)26Sor	0.67683	1.15008415	0.19544071
AFFX-TrpnX-3_at	---	---	0.67689	1.11857884	0.15990698
1450681_at	zinc finger protein 143	Zfp143	0.67691	-1.13506871	-0.184438
1440150_at	transglutaminase 3, E polypeptide	Tgm3	0.67691	-1.13260084	-0.1868744
1452334_at	centromere protein F	Cenpf	0.67692	1.14382854	0.19102407
1422336_at	homeobox A13	Hoxa13	0.67693	-1.14377245	-0.1957695
1454941_at	N-myristoyltransferase 1	Nmt1	0.67693	1.09277057	0.12795561
1416156_at	vinculin	Vcl	0.67694	1.10235201	0.13914487
1428484_at	oxysterol binding protein-like 3	Osbpl3	0.67695	1.14270338	0.19056772
1456751_x_at	Riken cDNA A530021J07 gene	A530021J07Rik	0.67701	1.11796988	0.15504282
1448359_a_at	HIG1 domain family, member 1A	Higd1a	0.67702	1.15435163	0.20379496
1420923_at	ubiquitin specific peptidase 9, X chromosome	Usp9x	0.67702	-1.17287242	-0.257269
1420313_x_at	---	---	0.67703	-1.12466315	-0.1705774
1453095_at	RAB10, member RAS oncogene family	Rab10	0.67704	-1.15149699	-0.2330128
1444815_at	expressed sequence C85328	C85328	0.67704	1.1234042	0.16287492
1419447_s_at	TBC1 domain family, member 1	Tbc1d1	0.67705	1.12084287	0.16190083
1450677_at	checkpoint kinase 1 homolog (S. pombe)	Chek1	0.67707	-1.09464574	-0.130487
1437693_at	DNA segment, Chr 1, Pasteur Institute 1	D1Pas1	0.67707	-1.12413684	-0.1745157
1432534_at	RIKEN cDNA 3300002I08 gene	3300002I08Rik	0.67708	-1.14353805	-0.1953496
1416514_a_at	fascin homolog 1, actin bundling protein (Strongylocentrotus purp	Fscn1	0.67708	-1.09415809	-0.1351373
1440849_at	RAS protein-specific guanine nucleotide-releasing factor 2	Rasgrf2	0.67708	1.15164412	0.20165175
1425227_a_at	ATPase, H+ transporting, lysosomal V0 subunit A1	Atp6v0a1	0.67709	1.14060327	0.18601136
1447691_x_at	---	---	0.6771	-1.17953479	-0.2635883
1426105_a_at	UBX domain protein 11	Ubxn11	0.67711	1.11968797	0.16228726
1428933_at	histone deacetylase 8	Hdac8	0.67711	-1.14977145	-0.203343
1453267_at	zinc finger homeobox 3	Zfhx3	0.67716	-1.17279589	-0.2508023
1444006_at	SET domain containing 2	Setd2	0.67716	1.20574678	0.24567507
1440242_at	family with sequence similarity 78, member B	Fam78b	0.67718	-1.13845098	-0.1894394
1431308_at	ankyrin repeat domain 33B	Ankrd33b	0.67719	-1.15992213	-0.2251525
1427493_at	sodium channel and clathrin linker 1	Sclt1	0.67719	-1.16072276	-0.2257244
1417156_at	keratin 19	Krt19	0.6772	1.1524149	0.19824404
1428767_at	gasdermin D	Gsdmd	0.6772	1.12034204	0.16345147
1448255_a_at	surfeit gene 4	Surf4	0.67721	1.1076773	0.14501507
1432352_at	cyclin Y	Ccny	0.67723	1.19374766	0.23915934
1418875_at	synaptogyrin 4	Syngr4	0.67723	1.19409782	0.2405488
1441411_at	LIM and senescent cell antigen-like domains 1	Lims1	0.67725	1.14546605	0.19321583
1425285_a_at	RAB27A, member RAS oncogene family	Rab27a	0.67726	-1.15020512	-0.2143235
1417248_at	ralA binding protein 1	Ralbp1	0.67727	-1.13867691	-0.2052072
1458901_at	---	---	0.6773	-1.13488021	-0.1827026
1442992_at	hypothetical LOC403343	130004C03	0.6773	1.15344628	0.20022673
1446821_at	Protocadherin 17	Pcdh17	0.67733	1.10611214	0.1451006
1435761_at	cDNA sequence BC100530 /// stefin A1	BC100530 /// Stfa1	0.67733	1.13525157	0.18271331
1458823_at	---	---	0.67734	-1.14795593	-0.2025787
1421937_at	dual adaptor for phosphotyrosine and 3-phosphoinositides 1	Dapp1	0.67735	-1.12213555	-0.1670472
1449004_at	mitochondrial ribosomal protein L46	Mrpl46	0.67738	1.10542218	0.1416155
1443978_at	ankyrin repeat and LEM domain containing 1	Ankle1	0.67739	1.13259284	0.17787908
1429587_at	RNA binding motif protein 34	Rbm34	0.67742	-1.09568295	-0.1330153
1442477_at	autophagy/beclin 1 regulator 1	Ambra1	0.67745	1.16031434	0.20843324
1444682_at	cDNA Sequence BC037032	BC037032	0.67747	1.14336132	0.19136524
1446839_at	---	---	0.67747	-1.1536082	-0.2142139
1438289_a_at	SMT3 suppressor of mif two 3 homolog 1 (yeast)	Sumo1	0.67747	-1.0827716	-0.1153191
1420638_at	phosphoribosyl pyrophosphate synthetase 2	Prps2	0.67751	-1.11908484	-0.165784
1427854_x_at	predicted gene 9817	Gm9817	0.67754	-1.15756884	-0.2255597
1455395_at	3-oxoacyl-ACP synthase, mitochondrial	Oxsm	0.67757	-1.1279366	-0.1775054
1448816_at	prostaglandin I2 (prostacyclin) synthase	Ptgis	0.67757	1.11130384	0.15061201
1457308_at	---	---	0.67759	-1.14026678	-0.1950677
1453773_at	ring finger protein 220	Rnf220	0.6776	1.13608841	0.18309693
1440444_at	fatty acid desaturase 1	Fads1	0.6776	1.15563961	0.20199121
1429083_at	amylo-1,6-glucosidase, 4-alpha-glucanotransferase	Agl	0.6776	1.13620443	0.18329089
1437711_x_at	ornithine decarboxylase, structural 1	Odc1	0.6776	-1.12898287	-0.1960111
1436980_x_at	CCR4-NOT transcription complex, subunit 2	Cnot2	0.67761	1.09802421	0.13405766
1418568_x_at	signal recognition particle 14	Srp14	0.67761	1.10122362	0.13714251
1431961_at	dedicator of cytokinesis 8	Dock8	0.67762	1.15283479	0.20194446
1456835_at	---	---	0.67766	1.16097589	0.20911873
1446894_at	---	---	0.67768	-1.16297269	-0.2301722
1441420_at	immunoglobulin superfamily, member 9	Igsf9	0.67768	1.15873455	0.20553681

1440138_at	---	---	0.67775	1.12024289	0.16323517
1422435_at	RIKEN cDNA 2210010C04 gene	2210010C04Rik	0.67775	-1.16108531	-0.230135
1459358_at	---	---	0.67775	1.16488176	0.21243444
1434856_at	ankyrin repeat domain 44	Ankrd44	0.67776	-1.13290504	-0.1856807
1441349_at	hypothetical LOC552904	LOC552904	0.67778	-1.17672361	-0.2529549
1436796_at	hypothetical LOC100503380 /// matrin 3	LOC100503380 /// Matr3	0.67787	1.09825217	0.13378971
1437602_at	family with sequence similarity 71, member B	Fam71b	0.67789	1.15534824	0.2022504
1430964_at	RIKEN cDNA 2310034O05 gene	2310034O05Rik	0.6779	-1.14321211	-0.1960662
1442440_at	---	---	0.67799	1.14713322	0.19257051
1447994_at	small nucleolar RNA host gene (non-protein coding) 10	Snhg10	0.67809	1.12531202	0.1674508
1437411_at	INO80 complex subunit D	Ino80d	0.6782	-1.14541333	-0.1995753
1451167_at	coiled-coil domain containing 101	Ccdc101	0.67825	1.13669652	0.17509841
1434791_at	ATPase, H+ transporting, lysosomal V0 subunit A2	Atp6v0a2	0.67825	1.12277676	0.16597115
1441656_at	RIKEN cDNA B930068K11 gene	B930068K11Rik	0.67827	-1.14810921	-0.2027427
1457958_at	---	---	0.67827	1.18446105	0.23146037
1455384_x_at	RIKEN cDNA D030056L22 gene	D030056L22Rik	0.67834	-1.10243842	-0.1412368
1417961_a_at	tripartite motif-containing 30A	Trim30a	0.67852	-1.15330411	-0.209723
1430352_at	a disintegrin-like and metallopeptidase (reprolysin type) with thro	Adamts9	0.67857	1.13684966	0.18403784
1450957_a_at	sequestosome 1	Sqstm1	0.67858	1.09026746	0.12426663
1419922_s_at	attractin like 1	Atrnl1	0.67859	1.1656586	0.21221433
1420997_a_at	glucose phosphate isomerase 1	Gpi1	0.67859	1.09064672	0.12446168
1426504_a_at	ring finger protein 121	Rnf121	0.6786	1.13215395	0.1738124
1442363_at	RIKEN cDNA 1110012J17 gene	1110012J17Rik	0.67861	-1.10961959	-0.152503
1443527_at	telomeric repeat binding factor 1	Terf1	0.67862	-1.14589814	-0.1989372
1415866_at	unc-45 homolog A (C. elegans)	Unc45a	0.67867	1.13051462	0.1729017
1453625_at	dystrobrevin alpha	Dtna	0.67868	1.09729011	0.13360084
1417507_at	cytochrome b-561	Cyb561	0.6787	1.11619394	0.15704615
1437613_s_at	protein tyrosine phosphatase domain containing 1	Ptpdc1	0.67874	1.20213745	0.24531351
1430495_at	inositol (myo)-1(or 4)-monophosphatase 1	Impa1	0.67876	-1.13081869	-0.1780632
1436722_a_at	actin, beta	Actb	0.67878	-1.05190506	-0.0744026
1422479_at	acyl-CoA synthetase short-chain family member 2	Acss2	0.67889	1.11762345	0.15889997
1419282_at	chemokine (C-C motif) ligand 12	Ccl12	0.67891	1.14155056	0.18452711
1434978_at	RIKEN cDNA 4933403F05 gene	4933403F05Rik	0.67891	1.12090955	0.16300157
1427157_at	coiled-coil domain containing 85A	Ccdc85a	0.67902	-1.15661947	-0.2194409
1430297_a_at	aprataxin and PNKP like factor	Aplf	0.67905	1.12598354	0.16917615
1450340_a_at	chloride channel Kb	Clcnkb	0.67908	1.13537558	0.18102695
1445164_at	---	---	0.67923	-1.14119494	-0.1913574
1454102_at	RIKEN cDNA 5031425E22 gene	5031425E22Rik	0.67937	-1.15852235	-0.2226413
1428842_a_at	nerve growth factor receptor (TNFRSF16) associated protein 1	Ngfrap1	0.67944	1.0845065	0.11577375
1450727_a_at	polymerase (DNA-directed), delta interacting protein 2	Poldip2	0.67949	1.09627701	0.13230438
1423200_at	nuclear receptor co-repressor 1	Ncor1	0.67965	-1.11077451	-0.1586665
1442464_at	F-box and leucine-rich repeat protein 20	Fbxl20	0.67971	-1.14252979	-0.1950052
1426739_at	downstream neighbor of SON	Donson	0.67977	1.10153426	0.13944969
1447996_at	---	---	0.67979	1.14618074	0.19284391
1452373_at	RIKEN cDNA 1700081L11 gene	1700081L11Rik	0.67986	1.13991408	0.18139089
1447726_at	ripply2 homolog (zebrafish)	Ripply2	0.67987	-1.08774256	-0.1215023
1457257_x_at	poliovirus receptor-related 3	Pvrl3	0.67987	1.10555457	0.14380639
1418107_at	transcription elongation factor A (SII), 2	Tcea2	0.67988	1.14491357	0.19024156
1452317_at	homeobox B9	Hoxb9	0.67988	-1.18250356	-0.2733127
1426953_at	HMG box domain containing 4	Hmgxb4	0.6799	1.10018758	0.13600312
1445229_at	diacylglycerol O-acyltransferase 1	Dgat1	0.67991	1.16571718	0.21096309
1433771_at	family with sequence similarity 108, member B	Fam108b	0.67992	-1.13562197	-0.1985393
1456005_a_at	BCL2-like 11 (apoptosis facilitator)	Bcl2l11	0.67993	1.1606849	0.20096412
1427577_x_at	predicted gene 7202 /// immunoglobulin kappa chain, constant re	Gm7202 /// Igk-C /// Igk-	0.67994	-1.15897412	-0.2220422
1451209_at	LAG1 homolog, ceramide synthase 5	Lass5	0.67994	1.09431441	0.12975013
1430756_at	RIKEN cDNA 5430427G11 gene	5430427G11Rik	0.68	1.16454144	0.21278809
1448557_at	family with sequence similarity 13, member C	Fam13c	0.68004	-1.12939238	-0.1830276
1431087_at	SPC24, NDC80 kinetochore complex component, homolog (S. cere	Spc24	0.68005	1.10481366	0.14174664
1442709_at	---	---	0.68006	-1.14299824	-0.1966675
1441378_at	---	---	0.68008	1.15655809	0.20560802
1457566_at	zinc finger protein 677	Zfp677	0.68009	-1.14162087	-0.1938448
1436320_at	---	---	0.68011	-1.21435063	-0.3541061
1447462_at	---	---	0.68011	1.15534376	0.20131747
1443043_at	otopetrin 2	Otop2	0.68012	-1.15683152	-0.2152839
1456038_at	F-box and leucine-rich repeat protein 4	Fbxl4	0.68015	1.13464395	0.18019191
1434631_at	DNA segment, Chr 3, ERATO Doi 751, expressed	D3Ertd751e	0.68019	-1.12784131	-0.1770828
1417309_at	transducer of ERBB2, 2	Tob2	0.68022	1.12020944	0.16348333
1416606_s_at	NHP2 ribonucleoprotein homolog (yeast)	Nhp2	0.68026	1.06951735	0.09605001
1454120_a_at	polycomb group ring finger 6	Pcgf6	0.68026	-1.10570446	-0.1453529
1442940_at	---	---	0.68027	-1.13885261	-0.1885911
1456212_x_at	suppressor of cytokine signaling 3	Socs3	0.68027	-1.10983949	-0.1586384
1437046_x_at	family with sequence similarity 63, member A	Fam63a	0.68029	-1.11163785	-0.1558365
1429559_at	guanine nucleotide binding protein, alpha q polypeptide	Gnaq	0.6803	-1.12414147	-0.1734761
1429251_at	hypothetical LOC100503505 /// PR domain containing 2, with ZNF	LOC100503505 /// Prdm2	0.68032	-1.11474215	-0.1608544
1448234_at	DnaJ (Hsp40) homolog, subfamily B, member 6	Dnajb6	0.68039	-1.09149246	-0.1298199
1442309_at	---	---	0.68039	1.13907821	0.18671597
1419278_at	ubiquitin specific peptidase 48	Usp48	0.68052	-1.10107599	-0.1398744
1450048_a_at	isocitrate dehydrogenase 2 (NADP+), mitochondrial	Idh2	0.68057	1.10856727	0.14726137
1430034_at	chaperonin containing Tcp1, subunit 4 (delta)	Cct4	0.68076	1.12370158	0.16599344
1444325_at	---	---	0.68084	-1.14626543	-0.2060051
1452954_at	ubiquitin-conjugating enzyme E2C	Ube2c	0.6809	1.08371133	0.11520943
1456431_at	Zinc finger protein 64	Zfp64	0.68099	-1.13445177	-0.1860925
1421333_a_at	myoneurin	Mynn	0.68118	1.1274928	0.17073439
1441873_at	prolactin family 7, subfamily a, member 1	Prl7a1	0.68131	1.12417773	0.16386054
1435517_x_at	v-ral simian leukemia viral oncogene homolog B (ras related)	Ralb	0.68137	-1.10426158	-0.1446728
1427807_at	predicted gene 10083 /// hypothetical protein LOC100503739	Gm10083 /// LOC100503	0.68137	-1.15730875	-0.220256
1417325_at	beta-transducin repeat containing protein	Btrc	0.68138	1.15712498	0.20500816
1428313_at	zinc finger, SWIM domain containing 3	Zswim3	0.68139	1.10888857	0.14894157
1442708_at	---	---	0.68146	1.15324224	0.20086871
1423282_at	phosphatidylinositol transfer protein, alpha	Pitpna	0.6815	1.11928216	0.16148785
1436764_at	par-3 (partitioning defective 3) homolog (C. elegans)	Pard3	0.6815	1.12410375	0.16645156
1440054_at	phosphatidylinositol 3-kinase, catalytic, alpha polypeptide	Pik3ca	0.6815	-1.14034992	-0.1899447
1452330_a_at	matrix-remodelling associated 8	Mxra8	0.6815	1.11641877	0.15862213
1427133_s_at	low density lipoprotein receptor-related protein 2	Lrp2	0.68151	1.13087301	0.17741022

1447612_x_at	KDM1 lysine (K)-specific demethylase 6B	Kdm6b	0.68154	-1.11202757	-0.1553973
1455479_a_at	ubiquitin-conjugating enzyme E2D 3 (UBC4/5 homolog, yeast)	Ube2d3	0.68161	-1.06760004	-0.0954626
1448003_at	RIKEN cDNA 1810043H04 gene	1810043H04Rik	0.68162	1.13449222	0.17470115
1452019_at	cysteine and tyrosine-rich protein 1	Cyyr1	0.68168	1.16713674	0.21401828
1417992_a_at	seminal vesicle secretory protein 3A /// seminal vesicle secretory	Svs3a /// Svs3b	0.6817	1.12045096	0.16341597
1423861_at	pleckstrin homology domain containing, family F (with FYVE dom	Plekhf2	0.68178	-1.11069226	-0.1524044
1457150_at	expressed sequence AI428301	AI428301	0.68208	-1.1733505	-0.2647244
1422992_s_at	phosphatidylinositol 3-kinase catalytic delta polypeptide	Pik3cd	0.68209	1.14113712	0.1861487
1458203_at	Spire homolog 1 (Drosophila)	Spire1	0.68209	1.20535409	0.24680627
1433429_at	phosphatidylinositol glycan anchor biosynthesis, class S	Pigs	0.68211	1.10748798	0.1458017
1443833_at	---	---	0.68224	1.14806071	0.19671881
1451473_a_at	crystallin, zeta (quinone reductase)-like 1	Cryzl1	0.68224	-1.0943198	-0.1314407
1437888_at	WNK lysine deficient protein kinase 1	Wnk1	0.68249	-1.12875425	-0.1765565
1428905_at	Ras-related GTP binding A	Rraga	0.6825	1.13740162	0.17371333
1423156_at	glucosamine-phosphate N-acetyltransferase 1	Gnpnat1	0.68252	-1.09667563	-0.1343338
1460653_at	ataxin 2	Atxn2	0.68267	1.09648708	0.13267181
1434250_at	p21 protein (Cdc42/Rac)-activated kinase 2	Pak2	0.68274	-1.1447372	-0.2055584
1426728_x_at	phosphatidylserine synthase 2	Ptdss2	0.68279	1.10556434	0.14392137
1437192_x_at	voltage-dependent anion channel 1	Vdac1	0.68283	-1.07276566	-0.101362
1428550_at	YdjC homolog (bacterial)	Ydjc	0.68283	1.11057678	0.15043829
1439270_x_at	RAN, member RAS oncogene family	Ran	0.68283	-1.06964431	-0.0989415
1458150_at	autophagy/beclin 1 regulator 1	Ambra1	0.6829	-1.15794624	-0.2222881
1450966_at	carnitine O-octanoyltransferase	Crot	0.6829	1.12631406	0.16579019
1452620_at	phosphoenolpyruvate carboxykinase 2 (mitochondrial)	Pck2	0.6829	-1.12806358	-0.1764984
1418007_at	GC-rich sequence DNA-binding factor 1	Gcfc1	0.68296	-1.10169991	-0.1440424
1428834_at	dual specificity phosphatase 4	Dusp4	0.68297	1.15969926	0.20852159
1433089_at	---	---	0.68299	-1.14234194	-0.1954572
1439390_at	RIKEN cDNA 1300018I17 gene	1300018I17Rik	0.68306	1.14620734	0.18693815
1418731_at	ring finger protein, LIM domain interacting	Rlim	0.68317	-1.21216301	-0.3492092
1423560_at	NEL-like 2 (chicken)	Nell2	0.6832	-1.13496539	-0.1847275
1417534_at	integrin beta 5	Itgb5	0.68326	1.1119584	0.15300112
1427217_at	zinc finger protein 455	Zfp455	0.68347	1.14517856	0.18806497
1449435_at	UDP-Gal:betaGlcNAc beta 1,4-galactosyltransferase, polypeptide	B4galt3	0.68348	1.10069552	0.13769714
1417103_at	D-dopachrome tautomerase	Ddt	0.68354	1.10670968	0.14170181
1433256_at	RIKEN cDNA A930011E06 gene	A930011E06Rik	0.68355	1.14106696	0.18173896
1451789_a_at	receptor-like tyrosine kinase	Ryk	0.68356	-1.11251447	-0.1577675
1431136_at	RAB36, member RAS oncogene family	Rab36	0.68356	1.14987797	0.19520638
1448415_a_at	sema domain, immunoglobulin domain (Ig), short basic domain, s	Sema3b	0.68359	1.13116918	0.17709049
1419114_at	asparagine-linked glycosylation 14 homolog (yeast)	Alg14	0.68359	1.11663456	0.15613426
1428025_s_at	phosphatidylinositol transfer protein, cytoplasmic 1	Pitpnc1	0.68367	-1.1055395	-0.1450182
1451809_s_at	RWD domain containing 3	Rwdd3	0.68372	-1.19879773	-0.3069335
1434442_at	starch binding domain 1	Stbd1	0.68378	1.15995839	0.20553785
1415855_at	kit ligand	Kitl	0.68381	1.16413021	0.21110161
1437014_x_at	peroxiredoxin 1	Prdx1	0.68393	-1.13388912	-0.1995272
1458663_at	---	---	0.68395	1.14464263	0.19057997
1419817_s_at	ankyrin repeat and zinc finger domain containing 1	Ankzf1	0.68395	1.13905964	0.17695558
1447213_at	---	---	0.68396	-1.14973977	-0.2081205
1448742_at	snail homolog 1 (Drosophila)	Snai1	0.68405	1.13126324	0.17751581
1416064_a_at	heat shock protein 5	Hspa5	0.6841	1.0810324	0.11212425
1437756_at	GTPase, IMAP family member 9	Gimap9	0.68411	1.17382844	0.21966247
1424128_x_at	aurora kinase B	Aurkb	0.68411	1.09870795	0.13461952
1433537_at	alpha thalassemia/mental retardation syndrome X-linked homolo	Atrx	0.68412	-1.10023603	-0.1393649
1449972_s_at	cDNA sequence BC018101 /// zinc finger protein 97	BC018101 /// Zfp97	0.68419	-1.10243597	-0.146167
1423718_at	adenylate kinase 3	Ak3	0.6842	1.12130893	0.16423013
1449118_at	dihydrolipoamide branched chain transacylase E2	Dbt	0.68423	-1.11467062	-0.1615772
1427903_at	phosphohistidine phosphatase 1	Phpt1	0.68433	1.10912536	0.1458046
1448597_at	cleavage stimulation factor, 3' pre-RNA, subunit 1	Cstf1	0.68439	1.09888921	0.13511156
1420444_at	solute carrier family 22 (organic cation transporter), member 3	Slc22a3	0.6844	-1.13455687	-0.1832786
1431057_a_at	protease, serine, 23	Prss23	0.68442	1.11264162	0.15391795
1458774_at	DNA segment, Chr 10, ERATO Doi 584, expressed	D10Ertd584e	0.68445	1.13388872	0.17924196
1454141_at	RIKEN cDNA 5730416O20 gene	5730416O20Rik	0.68446	1.20270642	0.24650211
1421169_at	zinc finger protein 46	Zfp46	0.68449	-1.14143945	-0.1911228
1445920_at	FAST kinase domains 3	Fastkd3	0.68449	-1.14975946	-0.2114189
1424772_at	H2A histone family, member J	H2afj	0.6845	1.12609218	0.17114201
1440905_at	Heparan sulfate 2-O-sulfotransferase 1	Hs2st1	0.68454	1.15047552	0.19684223
1419800_at	RIKEN cDNA 1810019N24 gene	1810019N24Rik	0.68454	1.15113679	0.20118156
1418643_at	tetraspanin 13	Tspan13	0.68461	-1.11051553	-0.1556002
1436231_at	RIKEN cDNA 2900052N01 gene	2900052N01Rik	0.68483	-1.13404515	-0.1827631
1459378_at	---	---	0.68506	-1.1229945	-0.1695124
1443050_at	fructosamine 3 kinase related protein	Fn3krp	0.68506	-1.1358335	-0.1839925
1453530_at	---	---	0.68513	-1.14333049	-0.197332
1459047_x_at	---	---	0.68514	1.13243392	0.1779127
1427091_at	zinc finger, NFX1-type containing 1	Znfx1	0.68517	1.11743898	0.16002263
1455668_at	Wolf-Hirschhorn syndrome candidate 1-like 1 (human)	Whsc1l1	0.68518	1.1443398	0.18642862
1437848_x_at	ADP-dependent glucokinase	Adpgk	0.68519	-1.14076744	-0.1989741
1428730_at	KRIT1, ankyrin repeat containing	Krit1	0.6852	-1.11775445	-0.1607686
1432705_at	RIKEN cDNA 5330428N10 gene	5330428N10Rik	0.68521	1.16806535	0.21335559
1456537_at	RIKEN cDNA A930033H14 gene	A930033H14Rik	0.68521	-1.17422518	-0.2568198
1434731_x_at	peroxiredoxin 1	Prdx1	0.68522	-1.05242217	-0.0737925
1425524_at	RNA binding motif protein 25	Rbm25	0.68525	-1.14439945	-0.1962223
1449429_at	FK506 binding protein 1b	Fkbp1b	0.68525	1.16371481	0.20625623
1445127_at	collagen and calcium binding EGF domains 1	Ccbe1	0.68526	-1.14591359	-0.2000577
1419170_at	family with sequence similarity 174, member A	Fam174a	0.68528	-1.1121888	-0.1566177
1423259_at	inhibitor of DNA binding 4	Id4	0.68532	1.19300577	0.23358698
1430907_at	RIKEN cDNA 4930455J16 gene	4930455J16Rik	0.68532	1.14141348	0.18882321
1438668_x_at	ataxin 2-like	Atxn2l	0.68532	-1.11972881	-0.1737005
1436232_a_at	GA repeat binding protein, beta 1	Gabpb1	0.68533	1.11103993	0.14848264
1431724_a_at	purinergic receptor P2Y, G-protein coupled 12	P2ry12	0.68534	-1.1386301	-0.1926581
1439660_at	human immunodeficiency virus type I enhancer binding protein 3	Hivep3	0.68534	1.1514243	0.19992329
1418698_a_at	ferrochelatase	Fech	0.68534	1.11865142	0.16118029
1460264_at	expressed sequence C81600	C81600	0.68536	-1.15094815	-0.2043912
1457422_at	Unc-79 homolog (C. elegans)	Unc79	0.6854	-1.1556547	-0.2229176
1444008_at	Predicted gene 2137	Gm2137	0.68543	1.14311363	0.18882595
1434148_at	transcription factor 4	Tcf4	0.68543	-1.11416372	-0.1586525

AFFX-BioB-3_at	---	---	0.68547	1.14123849	0.18193427
1451312_at	NADH dehydrogenase (ubiquinone) Fe-S protein 7	Ndufs7	0.6855	1.10643922	0.14328233
1436213_a_at	RIKEN cDNA 1110028C15 gene	1110028C15Rik	0.68554	-1.1500559	-0.2075072
1452061_s_at	spermatid perinuclear RNA binding protein	Strbp	0.68554	-1.09698654	-0.1354185
1426577_a_at	lin-37 homolog (C. elegans)	Lin37	0.68558	1.11902479	0.15959384
1433195_at	RIKEN cDNA 9530006O14 gene	9530006O14Rik	0.68568	-1.18382004	-0.2786852
AFFX-r2-Ec-bioC-3	---	---	0.6857	1.10657665	0.14336595
1444801_at	RIKEN cDNA 2900041M22 gene	2900041M22Rik	0.68572	-1.1412861	-0.2003321
AFFX-ThrX-3_at	---	---	0.68572	1.20880141	0.24599684
1459326_at	DNA segment, Chr 9, ERATO Doi 256, expressed	D9Ertd256e	0.68572	-1.15718455	-0.2206908
1430135_at	deoxyribonuclease II alpha	Dnase2a	0.68578	1.18684404	0.23130413
1429784_at	Mitogen-activated protein kinase 1 interacting protein 1-like	Mapk1ip1l	0.68581	1.20896924	0.24364705
1433945_at	family with sequence similarity 189, member A1	Fam189a1	0.68581	1.15821781	0.20413526
1425142_a_at	heterogeneous nuclear ribonucleoprotein D	Hnrnpd	0.68583	-1.08795361	-0.1246169
1452644_at	methyl-CpG binding domain protein 3-like 2	Mbd3l2	0.68585	-1.13851876	-0.1888758
1441989_at	BCL2/adenovirus E1B interacting protein 2	Bnip2	0.68587	1.14412855	0.19259254
1452003_at	RNA binding motif protein 14	Rbm14	0.68589	1.13409831	0.18137999
1439664_at	DDB1 and CUL4 associated factor 7	Dcaf7	0.68589	-1.13732659	-0.1878608
1447733_x_at	TatD DNase domain containing 3	Tatdn3	0.6859	1.14038568	0.17899357
1428955_x_at	solute carrier family 9 (sodium/hydrogen exchanger), member 3 r	Slc9a3r2	0.6859	1.11492103	0.15643983
1434760_at	leucine rich repeat transmembrane neuronal 3	Lrrtm3	0.68591	1.19822302	0.23799112
1422780_at	peroxisomal membrane protein 4	Pxmp4	0.68593	1.1259387	0.17026251
1460425_at	RIKEN cDNA 1700001C19 gene	1700001C19Rik	0.68593	-1.14873987	-0.2071941
1436874_x_at	solute carrier family 25 (mitochondrial carrier, adenine nucleotide	Slc25a5	0.68595	1.05826247	0.08135579
1452279_at	complement factor properdin	Cfp	0.68596	1.12692945	0.17208953
1457652_x_at	---	---	0.68606	1.12859844	0.16622178
1429265_a_at	ring finger protein 130	Rnf130	0.68612	1.10938514	0.14934009
1431743_a_at	solute carrier family 4 (anion exchanger), member 1	Slc4a1	0.68612	-1.13871525	-0.1883599
1417956_at	cell death-inducing DNA fragmentation factor, alpha subunit-like e	Cidea	0.68613	1.14089268	0.18855493
1426818_at	arrestin domain containing 4	Arrdc4	0.68615	1.12657278	0.16866014
1418620_at	paired-like homeobox 2a	Phox2a	0.68616	1.14537709	0.19274581
1430698_a_at	progesterone immunomodulatory binding factor 1	Pibf1	0.68617	-1.12014035	-0.1641978
1451234_at	family with sequence similarity 193, member B	Fam193b	0.6862	1.10923951	0.14949642
1439444_x_at	transmembrane emp24-like trafficking protein 10 (yeast)	Tmed10	0.68623	-1.16625737	-0.2623514
1422721_at	tyrosine kinase, non-receptor, 1	Tnk1	0.68624	1.11628376	0.15652268
1446846_at	---	---	0.68631	1.20907334	0.25202081
1455787_x_at	multiple inositol polyphosphate histidine phosphatase 1	Minpp1	0.68632	-1.21341926	-0.3570459
1426651_at	mitochondrial ribosomal protein L44	Mrpl44	0.68656	1.09607341	0.13152244
1430452_at	cytochrome P450, family 20, subfamily A, polypeptide 1	Cyp20a1	0.68723	1.12928458	0.17314474
1425198_at	protein tyrosine phosphatase, non-receptor type 2	Ptpn2	0.68738	1.1482461	0.19555515
1418695_a_at	potassium channel modulatory factor 1	Kcmf1	0.68746	-1.09716101	-0.1355597
1439600_at	---	---	0.68751	-1.14729171	-0.2039637
1438665_at	sphingomyelin phosphodiesterase 3, neutral	Smpd3	0.68752	-1.14768426	-0.2048645
1425112_at	ankyrin repeat domain 27 (VPS9 domain)	Ankrd27	0.68755	1.14840959	0.1921584
1453169_a_at	general transcription factor II H, polypeptide 1	Gtf2h1	0.68757	-1.10439051	-0.1484444
1427965_at	single-stranded DNA binding protein 1	Ssbp1	0.68758	-1.0821905	-0.1164133
1416475_at	ubiquitin-conjugating enzyme E2D 2	Ube2d2	0.6876	-1.11073597	-0.156518
1439926_at	HEG homolog 1 (zebrafish)	Heg1	0.68762	1.15382301	0.20024662
1431647_a_at	carcinoembryonic antigen-related cell adhesion molecule 13	Ceacam13	0.68763	1.11551006	0.15550375
1453043_at	RIKEN cDNA 0610012H03 gene	0610012H03Rik	0.68765	-1.13626684	-0.1846517
1454645_at	mahogunin, ring finger 1	Mgrn1	0.68766	1.10334719	0.14154553
1418697_at	indolethylamine N-methyltransferase	Inmt	0.68767	-1.17967776	-0.2694371
1458359_at	---	---	0.68768	1.14303232	0.18695092
1419898_s_at	zinc finger CCCH type containing 7 A	Zc3h7a	0.68771	1.13045068	0.17471929
1422043_at	tuberous sclerosis 1	Tsc1	0.68772	1.12656414	0.17093265
1451751_at	DNA-damage-inducible transcript 4-like	Ddit4l	0.68772	1.1388812	0.18340297
1435204_at	protein arginine N-methyltransferase 8	Prmt8	0.68775	1.10506646	0.14187877
1452835_a_at	polymerase (RNA) mitochondrial (DNA directed)	Polrmt	0.68775	1.09626326	0.132485
1445766_at	---	---	0.68775	-1.13975583	-0.1927717
1420954_a_at	adducin 1 (alpha)	Add1	0.68776	1.11942304	0.16088813
1421964_at	Notch gene homolog 3 (Drosophila)	Notch3	0.68776	1.33442385	0.34044601
1457562_at	ribosomal protein S6 kinase, polypeptide 1	Rps6kb1	0.68778	1.16840371	0.21622574
1453545_at	RIKEN cDNA 5730492I20 gene	5730492I20Rik	0.6878	1.15483307	0.20037468
1449126_at	zinc finger protein 90	Zfp90	0.68783	1.12578674	0.17050733
1422861_s_at	PDZ and LIM domain 5	Pdlim5	0.68788	-1.11983982	-0.1638372
1443745_s_at	dentin matrix protein 1	Dmp1	0.68794	1.11088699	0.15078724
1451050_at	5'-nucleotidase, cytosolic III	Nt5c3	0.68795	1.13175266	0.17545926
1452908_at	DIP2 disco-interacting protein 2 homolog A (Drosophila)	Dip2a	0.68795	1.12299295	0.16578604
1456006_at	BCL2-like 11 (apoptosis facilitator)	Bcl2l11	0.68795	1.17300261	0.21583506
1429752_x_at	CAP-GLY domain containing linker protein family, member 4	Clip4	0.68796	1.12882073	0.17402521
1421093_at	solute carrier family 7 (cationic amino acid transporter, y+ system	Slc7a10	0.68799	-1.14707211	-0.1989686
1422388_at	vomeronasal 1 receptor 25	Vmn1r25	0.688	1.12774829	0.1711197
1423030_at	valosin containing protein	Vcp	0.68803	1.08047253	0.11133605
1436816_at	nucleoporin 133	Nup133	0.68805	1.11263999	0.1491165
1419133_at	envoplakin	Evpl	0.68808	1.14931185	0.19366739
1451641_at	debranching enzyme homolog 1 (S. cerevisiae)	Dbr1	0.6881	-1.11499142	-0.1649162
1434717_at	cullin 3	Cul3	0.68811	-1.09733341	-0.1351743
1452752_at	leprecan 1	Lepre1	0.68811	1.12506964	0.16867027
1422892_s_at	histocompatibility 2, class II antigen E alpha, pseudogene /// h-2 c	H2-Ea-ps /// LOC1005044	0.68811	1.14869576	0.19859759
1431220_at	RIKEN cDNA 2810416G20 gene	2810416G20Rik	0.68811	-1.13750543	-0.1863661
1425530_a_at	syntaxin 3	Stx3	0.68816	-1.16726222	-0.2463884
1431055_a_at	sorting nexin 10	Snx10	0.68816	1.1276653	0.16862065
1435343_at	dedicator of cytokinesis 10	Dock10	0.68817	1.29762632	0.32187031
1449270_at	plexin domain containing 2	Plxdc2	0.68817	-1.13965402	-0.1891077
1438323_at	lens intrinsic membrane protein 2	Lim2	0.68818	-1.12543635	-0.1710136
1425108_a_at	small cell adhesion glycoprotein	Smagp	0.68822	1.11379747	0.15030718
1432714_at	RIKEN cDNA 6430709C05 gene	6430709C05Rik	0.68822	1.1355297	0.18117074
1425404_a_at	transmembrane protein 110	Tmem110	0.68824	1.1435092	0.18853804
1427602_at	diabetic embryopathy 1	Dep1	0.68824	-1.14907312	-0.2129115
1419116_at	asparagine-linked glycosylation 14 homolog (yeast)	Alg14	0.68825	-1.15717325	-0.2223346
1435930_at	S phase cyclin A-associated protein in the ER	Scaper	0.68828	1.12710072	0.16909777
1457741_at	---	---	0.68829	-1.12287619	-0.1681962
1457174_at	expressed sequence AU015680	AU015680	0.68829	1.20742572	0.2474171
1415733_a_at	translocase of outer mitochondrial membrane 5 homolog (yeast)	Tomm5	0.68829	1.09214126	0.12593628

1437041_at	serine/arginine-rich splicing factor 18	Sfrs18	0.68831	-1.10266271	-0.141188
1447053_x_at	signal sequence receptor, gamma	Ssr3	0.68831	-1.09228132	-0.1292382
1449183_at	catechol-O-methyltransferase 1	Comt1	0.68833	1.11863879	0.1581798
1445707_at	zinc finger, DHHC domain containing 6	Zdhhc6	0.68838	1.1466979	0.19477672
1428420_a_at	RIKEN cDNA 1200009I06 gene	1200009I06Rik	0.68842	-1.14411775	-0.1961241
1418116_at	torsin A interacting protein 2	Tor1aip2	0.68844	1.0992263	0.1354626
1422167_at	sema domain, seven thrombospondin repeats (type 1 and type 1	Sema5a	0.68845	1.16103473	0.20342621
1423460_at	GRB10 interacting GYF protein 1	Gigyf1	0.68846	1.12023219	0.1571961
1456624_at	WD repeat domain, phosphoinositide interacting 1	Wipi1	0.68846	-1.14219786	-0.1968974
1459983_at	inositol (myo)-1(or 4)-monophosphatase 2	Impa2	0.68849	1.11513044	0.15663318
1422045_a_at	protein tyrosine phosphatase, non-receptor type 12	Ptpn12	0.68853	-1.10715116	-0.1521191
1417515_at	U7 snRNP-specific Sm-like protein LSM10	Lsm10	0.68853	1.09986549	0.13648608
1437214_at	leucine rich repeat transmembrane neuronal 4	Lrrtm4	0.68853	1.09798714	0.1328198
1417591_at	prostaglandin E synthase 2	Ptges2	0.68857	1.10428125	0.14255138
1419172_at	dihydrofolate reductase	Dhfr	0.68858	-1.11727775	-0.1709894
1454612_at	mex3 homolog C (C. elegans)	Mex3c	0.68892	-1.1046127	-0.1483189
1457735_at	---	---	0.68894	1.14795415	0.1954261
1444735_at	---	---	0.68896	1.17545032	0.21872698
1438171_x_at	methyltransferase like 9	Mettl9	0.68898	-1.12745969	-0.1902221
1452319_at	zinc finger protein 82-like /// zinc finger protein 82	LOC100505067 /// Zfp82	0.68919	1.11410256	0.15569684
1444629_at	family with sequence similarity 117, member B	Fam117b	0.68921	1.14107532	0.18201036
1437074_at	sorting nexin 5	Snx5	0.68923	-1.16062362	-0.2402228
1458406_at	---	---	0.68926	-1.14326043	-0.2037817
1444417_at	RIKEN cDNA 9330101J02 gene	9330101J02Rik	0.68928	-1.15357224	-0.2115643
1415750_at	transducin (beta)-like 3	Tbl3	0.68932	1.13986792	0.17708165
1452656_at	zinc finger, DHHC domain containing 2	Zdhhc2	0.68933	1.13980393	0.18676958
1426545_at	trinucleotide repeat containing 6b	Tnrc6b	0.68934	1.11271407	0.15083959
1434937_at	MYC binding protein 2	Mycbp2	0.68935	-1.10499139	-0.1490664
1427108_at	RIKEN cDNA 9530068E07 gene	9530068E07Rik	0.68946	1.12080571	0.16110607
1424605_at	proprotein convertase subtilisin/kexin type 5	Pcsk5	0.68957	-1.12724782	-0.17729
1456505_at	Braf transforming gene	Braf	0.68974	-1.169851	-0.2546328
1453758_at	coiled-coil domain containing 34	Ccdc34	0.68974	1.14922406	0.19399248
1426089_a_at	cDNA sequence BC003331	BC003331	0.68983	1.12944865	0.1700864
1420038_at	ATPase, H+ transporting, lysosomal V1 subunit E1	Atp6v1e1	0.68984	-1.14110262	-0.1922258
1448675_at	ankyrin repeat and zinc finger domain containing 1	Ankzf1	0.68988	1.17351215	0.2156733
1448134_at	cDNA sequence X99384	X99384	0.6903	1.09021503	0.12439734
1458771_at	---	---	0.69036	-1.11078422	-0.1518114
1447354_at	---	---	0.69039	1.13738724	0.17613131
1416320_at	SEC22 vesicle trafficking protein homologe A (S. cerevisiae)	Sec22a	0.6904	1.12019675	0.16153425
1433583_at	zinc finger protein 365	Zfp365	0.6905	-1.1253881	-0.1764065
1423292_a_at	periaxin	Prx	0.6905	1.09510919	0.1302301
1447488_at	---	---	0.69065	1.13616191	0.17566057
1452619_a_at	ATP/GTP binding protein-like 3	Agbl3	0.69076	1.15867003	0.20281835
1443822_s_at	CDGSH iron sulfur domain 1	Cisd1	0.69086	-1.10995308	-0.151823
1424373_at	armadillo repeat containing, X-linked 3	Armcx3	0.69093	-1.17014163	-0.2571309
1418221_at	casein alpha s2-like B	Csn1s2b	0.69094	1.12170969	0.16479197
1421118_a_at	G protein-coupled receptor 56	Gpr56	0.69094	1.15838034	0.20851456
1437013_x_at	ATPase, H+ transporting, lysosomal V0 subunit B	Atp6v0b	0.69099	1.09835827	0.13398934
1434194_at	microtubule-associated protein 2	Mtap2	0.69108	1.14543864	0.19286169
1442212_at	carboxypeptidase D	Cpd	0.69108	1.12645859	0.17014259
1450995_at	folate receptor 1 (adult)	Folr1	0.69113	-1.10452565	-0.1474759
1451717_s_at	SUMO/sentrin specific peptidase 2	Senp2	0.69119	1.09672731	0.13164227
1446341_at	RIKEN cDNA 6030403N03 gene	6030403N03Rik	0.69119	1.15212359	0.20151926
1433507_a_at	high mobility group nucleosomal binding domain 2 /// high mobil	Hmgn2 /// Hmgn4	0.6912	-1.06879551	-0.0977497
1441926_x_at	transmembrane inner ear	Tmie	0.69122	-1.1445658	-0.2010715
1451187_at	RIKEN cDNA 0610037P05 gene	0610037P05Rik	0.69127	1.10366428	0.13939448
1450691_at	CASK-interacting protein 2	Caskin2	0.69129	1.14216189	0.18356548
1449910_at	RIKEN cDNA 2210418O10 gene /// novel KRAB box and zinc finge	2210418O10Rik /// LOC6	0.69133	-1.13531112	-0.1908736
1441076_at	MpV17 mitochondrial inner membrane protein	Mpv17	0.69138	-1.13833283	-0.1884168
1422644_at	SH3-binding domain glutamic acid-rich protein	Sh3bgr	0.69146	-1.15719871	-0.227074
1456168_at	cyclin J-like	Ccnjl	0.69151	1.15327324	0.20192111
1439500_at	secernin 1	Scrn1	0.69154	1.15218199	0.19532091
1444920_at	---	---	0.69158	-1.11062366	-0.1517292
1425301_at	neural cell adhesion molecule 2	Ncam2	0.69159	-1.13412471	-0.1840208
1439424_x_at	HERPUD family member 2	Herpud2	0.69159	-1.12464314	-0.1696587
1455776_x_at	bolA-like 2 (E. coli)	Bola2	0.69159	1.1320389	0.17046842
1422839_at	neurogenin 2	Neurog2	0.69164	1.13674144	0.18266673
1458102_at	---	---	0.69164	1.13561581	0.17993653
1439964_at	---	---	0.69165	-1.14792785	-0.2188802
1418904_at	glutamine fructose-6-phosphate transaminase 1	Gfpt1	0.69165	-1.14390572	-0.2031176
1419611_at	RRS1 ribosome biogenesis regulator homolog pseudogene	4632415L05Rik	0.69165	-1.13169849	-0.1809641
1445767_at	protein tyrosine phosphatase, receptor type, D	Ptprd	0.69165	1.11460095	0.15497155
1430244_at	RIKEN cDNA 4921509J17 gene	4921509J17Rik	0.69166	1.11737138	0.15829556
1440818_s_at	splicing factor 3b, subunit 1	Sf3b1	0.69167	-1.11792975	-0.1724975
1425948_a_at	solute carrier family 25, member 30	Slc25a30	0.69167	1.13907679	0.18565862
1433567_at	guanine monophosphate synthetase	Gmps	0.69168	1.0905138	0.12409328
1436028_at	transmembrane protein 33	Tmem33	0.69168	1.13338665	0.17746159
1420731_a_at	cysteine and glycine-rich protein 2	Csrp2	0.69171	1.08787219	0.12093576
1450433_at	recombination signal binding protein for immunoglobulin kappa J	Rbpjl	0.69171	-1.14077285	-0.1920596
1422964_at	RAD23a homolog (S. cerevisiae)	Rad23a	0.69174	1.10185967	0.13890737
1453567_s_at	transmembrane protein 216	Tmem216	0.69174	1.10682024	0.14514741
1421706_at	matrix metallopeptidase 20 (enamelysin)	Mmp20	0.69175	1.15167307	0.19993102
1460578_at	FYVE, RhoGEF and PH domain containing 5	Fgd5	0.69176	1.13690673	0.18129623
1418049_at	latent transforming growth factor beta binding protein 3	Ltbp3	0.69185	1.16131559	0.20998337
1447873_x_at	BH3 interacting domain death agonist	Bid	0.69185	-1.1483411	-0.2082241
1444720_at	expressed sequence AA408456	AA408456	0.69188	1.15438726	0.20077949
1452917_at	replication factor C (activator 1) 5	Rfc5	0.69191	1.09560355	0.12906401
1439959_at	fibroblast growth factor 11	Fgf11	0.69192	1.14700456	0.1959316
1447113_at	---	---	0.69192	-1.14045028	-0.1903386
1443300_at	---	---	0.69194	-1.15116411	-0.2131088
1428034_a_at	tumor necrosis factor receptor superfamily, member 9	Tnfrsf9	0.69195	1.1253433	0.1699072
1435650_at	hyaluronan and proteoglycan link protein 4	Hapln4	0.69201	1.1423273	0.18806021
1445786_at	Braf transforming gene	Braf	0.69203	-1.12977866	-0.1787294
1448417_at	ninjurin 1	Ninj1	0.69208	1.15496369	0.19637768

1453351_at	T-box 20	Tbx20	0.69209	1.28318715	0.30766736
1417210_at	eukaryotic translation initiation factor 2, subunit 3, structural gene	Eif2s3y	0.6921	-1.09808572	-0.1353418
1446203_at	muskelin 1, intracellular mediator containing kelch motifs	Mkln1	0.69214	1.16083974	0.20669429
1437331_a_at	ADP-ribosylation factor 3	Arf3	0.69214	-1.09487187	-0.1341179
1418745_at	osteomodulin	Omd	0.69215	-1.13414846	-0.1816909
1451126_at	MAF1 homolog (S. cerevisiae)	Maf1	0.69216	1.09268042	0.12674928
1425945_at	RIKEN cDNA 4933426I21 gene	4933426I21Rik	0.69218	-1.16451518	-0.2354797
1438189_s_at	erythrocyte protein band 4.9	Epb4.9	0.69221	-1.14539832	-0.2020047
1439788_at	coiled-coil domain containing 111	Ccdc111	0.69221	1.16541875	0.20784562
1428480_at	cell division cycle associated 8	Cdca8	0.69223	1.09429195	0.12908203
1416186_at	proline-rich nuclear receptor coactivator 2	Pnrc2	0.69223	-1.10788954	-0.153108
1426358_at	TAO kinase 1	Taok1	0.69224	-1.0980796	-0.1360037
1431609_a_at	acid phosphatase 5, tartrate resistant	Acp5	0.69224	1.13407088	0.17767384
1438684_at	NUAK family, SNF1-like kinase, 1	Nuak1	0.69228	1.13438778	0.17892063
1454956_at	ribosomal protein S6 kinase, polypeptide 1	Rps6kb1	0.69228	-1.11502781	-0.1632946
1416535_at	microspherule protein 1	Mcrs1	0.69229	1.08964583	0.12286915
1456401_at	calcium channel, voltage-dependent, beta 2 subunit	Cacnb2	0.69229	-1.14396061	-0.1973829
1424971_at	coiled-coil domain containing 99	Ccdc99	0.69229	1.10356532	0.14181887
1437971_at	superkiller viralicidic activity 2-like 2 (S. cerevisiae)	Skiv2l2	0.69229	1.13521107	0.18284787
1447142_at	cathepsin 7	Cts7	0.69231	1.17518091	0.21311053
1424826_s_at	metastasis suppressor 1	Mtss1	0.69231	-1.09213809	-0.1272016
1453504_at	TAF15 RNA polymerase II, TATA box binding protein (TBP)-associa	Taf15	0.69231	-1.16019807	-0.2303471
1431503_at	RIKEN cDNA 4930513O06 gene	4930513O06Rik	0.69231	-1.12696269	-0.1822948
1438363_at	PNMA-like 2	Pnmal2	0.69234	1.138007	0.18595685
1436492_x_at	LEM domain containing 1	Lemd1	0.69234	1.12682855	0.16573249
1449281_at	neurturin	Nrtn	0.69234	1.11633508	0.15543066
1417074_at	carcinoembryonic antigen-related cell adhesion molecule 10	Ceacam10	0.69235	1.17016489	0.21652966
1416147_at	heat shock protein 4	Hspa4	0.69237	1.13237107	0.17012451
1446816_at	---	---	0.69238	1.13920617	0.18695389
1454816_at	retinitis pigmentosa 2 homolog (human)	Rp2h	0.69238	-1.1374871	-0.1879637
1447232_at	---	---	0.69238	1.15850021	0.20671709
1456698_s_at	heterogeneous nuclear ribonucleoprotein D-like	Hnrpdl	0.69238	-1.10017042	-0.1416937
1439173_at	hook homolog 1 (Drosophila)	Hook1	0.69239	1.19853153	0.23852881
1433708_at	signal recognition particle 68	Srp68	0.6924	1.08978598	0.12343359
1439545_at	---	---	0.69243	-1.14090898	-0.1910466
1436751_at	WEE1 homolog 2 (S. pombe)	Wee2	0.69243	-1.1332376	-0.1862319
1436567_a_at	NADH dehydrogenase (ubiquinone) 1 alpha subcomplex, 7 (B14.5	Ndufa7	0.69244	1.10045293	0.13237354
1427469_at	helicase with zinc finger domain	Helz	0.69244	-1.13296286	-0.1963055
1416367_at	RIKEN cDNA 1110001J03 gene	1110001J03Rik	0.69244	1.17136379	0.20642644
1431875_a_at	E2F transcription factor 1	E2f1	0.69245	1.11514264	0.15219822
1431247_at	RIKEN cDNA 1300014J16 gene	1300014J16Rik	0.69246	-1.14322334	-0.193714
1425582_a_at	endomucin	Emcn	0.69248	1.15059029	0.19635649
1459241_at	---	---	0.69249	-1.14712863	-0.2040497
1442530_at	---	---	0.6925	-1.14935219	-0.2043038
1420021_s_at	suppressor of zeste 12 homolog (Drosophila)	Suz12	0.6925	-1.10692003	-0.1527865
1426108_s_at	calcium channel, voltage-dependent, beta 1 subunit	Cacnb1	0.69251	1.12620575	0.16883777
1456063_at	family with sequence similarity 120, member C	Fam120c	0.69252	-1.16689273	-0.2472869
1430813_at	phosphopantothenoylcysteine decarboxylase	Ppcdc	0.69253	-1.14207773	-0.1945748
1416811_s_at	cytotoxic T lymphocyte-associated protein 2 alpha /// cytotoxic T	Ctla2a /// Ctla2b	0.69255	1.13354996	0.17945806
1436663_at	expressed sequence AI846148	AI846148	0.69255	-1.13739584	-0.1857383
1435554_at	transmembrane and coiled coil domains 3	Tmcc3	0.69256	-1.16573324	-0.2373018
1434210_s_at	leucine-rich repeats and immunoglobulin-like domains 1	Lrig1	0.69256	-1.12727795	-0.1796764
1458979_at	---	---	0.69257	-1.13259796	-0.1805875
1427985_at	spindlin family, member 4	Spin4	0.69259	-1.14942937	-0.2089136
1453851_a_at	growth arrest and DNA-damage-inducible 45 gamma	Gadd45g	0.69259	1.18412983	0.2231519
1437365_at	NOL1/NOP2/Sun domain family member 3	Nsun3	0.69261	-1.14175202	-0.1964253
1455749_x_at	NADH dehydrogenase (ubiquinone) 1 alpha subcomplex, 7 (B14.5	Ndufa7	0.69262	1.12092713	0.15855421
1442028_at	beta-1,4-N-acetyl-galactosaminyl transferase 2	B4galnt2	0.69267	1.13788937	0.18374315
1453550_a_at	fatty acyl CoA reductase 1	Far1	0.69267	-1.13097413	-0.1874296
1455565_at	B-cell CLL/lymphoma 9	Bcl9	0.69268	1.15257404	0.19958368
1420632_a_at	Bernardinelli-Seip congenital lipodystrophy 2 homolog (human)	Bscl2	0.6927	1.11403678	0.15356836
1417061_at	solute carrier family 40 (iron-regulated transporter), member 1	Slc40a1	0.69274	1.13732642	0.17649677
1437766_at	---	---	0.69274	-1.12750592	-0.1737565
1457475_at	expressed sequence C80993	C80993	0.69276	1.14913413	0.19902368
1455300_at	tet oncogene family member 2	Tet2	0.69276	-1.11299441	-0.1603702
1446152_at	---	---	0.69277	-1.15339082	-0.2165394
AFFX-r2-Bs-phe-3	---	---	0.69281	1.19844856	0.2368333
1426959_at	3-hydroxybutyrate dehydrogenase, type 1	Bdh1	0.69281	1.17848259	0.22456448
1430906_at	RIKEN cDNA 2510016G02 gene	2510016G02Rik	0.69282	-1.12701114	-0.1734375
1456606_a_at	carbohydrate sulfotransferase 11 /// phosphatase and actin regu	Chst11 /// Phactr1	0.69284	1.14015218	0.18493801
1424508_at	tetratricopeptide repeat domain 5	Ttc5	0.69284	1.10347553	0.13867493
1455826_a_at	beta-site APP cleaving enzyme 1	Bace1	0.69289	1.12867986	0.1719147
1453738_at	RIKEN cDNA C330018D20 gene	C330018D20Rik	0.69291	-1.13216746	-0.1860035
1429374_at	cysteine-rich perinuclear theca 12	Cypt12	0.69292	1.14508874	0.19097583
1434515_at	nuclear receptor coactivator 1	Ncoa1	0.69294	-1.11338711	-0.1573004
1449826_a_at	fibroblast growth factor 2	Fgf2	0.69298	1.10847389	0.14783683
1456833_at	G protein-coupled receptor 17	Gpr17	0.69302	1.15028317	0.1985096
1451467_s_at	GTP binding protein 5	Gtpbp5	0.69305	1.09743599	0.13392803
1443633_at	RIKEN cDNA 1700086O06 gene	1700086O06Rik	0.69306	1.18490181	0.22854356
1424084_at	ROD1 regulator of differentiation 1 (S. pombe)	Rod1	0.69308	-1.13042851	-0.1773022
1426559_at	sno, strawberry notch homolog 1 (Drosophila)	Sbno1	0.69312	-1.12195361	-0.1809791
1443597_at	mediator complex subunit 14	Med14	0.69312	1.15862572	0.20450112
1438855_x_at	tumor necrosis factor, alpha-induced protein 2	Tnfaip2	0.69314	-1.15185979	-0.2183176
1422664_at	RAB10, member RAS oncogene family	Rab10	0.69318	-1.1071896	-0.1500206
1456848_at	coiled-coil domain containing 123	Ccdc123	0.69325	1.19022433	0.23410669
1451833_a_at	SET domain, bifurcated 1	Setdb1	0.69328	1.09612826	0.1310834
1436183_at	zinc finger CCCH type, antiviral 1	Zc3hav1	0.69332	-1.1183227	-0.1620951
1450141_at	ATP-binding cassette, sub-family G (WHITE), member 3	Abcg3	0.69337	1.15747219	0.20386401
1448015_at	DAZ interacting protein 3, zinc finger	Dzip3	0.69338	1.21008233	0.25111951
1428300_at	sperm antigen with calponin homology and coiled-coil domains 1-	Specc1l	0.69339	1.1097501	0.1485029
AFFX-BioB-5_at	---	---	0.69345	1.12152847	0.16049742
1432621_at	RIKEN cDNA 5730410E19 gene	5730410E19Rik	0.69345	1.1395286	0.18715701
1455874_at	transmembrane protein 179B	Tmem179b	0.69346	1.12763938	0.17189266
1450648_s_at	histocompatibility 2, class II antigen A, beta 1	H2-Ab1	0.69349	1.15798061	0.20488761

1430453_a_at	BCL2-like 2	Bcl2l2	0.69349	-1.14174737	-0.195315
1453933_at	RIKEN cDNA 4933435E02 gene	4933435E02Rik	0.69355	-1.14309905	-0.1989705
1441628_at	---	---	0.69358	-1.15535029	-0.2190301
1444416_at	Centromere protein A	Cenpa	0.69368	-1.14100977	-0.1941666
1449176_a_at	deoxycytidine kinase	Dck	0.69381	-1.14225773	-0.2183591
1455994_x_at	elongation of very long chain fatty acids (FEN1/Elo2, SUR4/Elo3, y	Elovl1	0.69384	1.11475351	0.15385075
1431820_at	RIKEN cDNA 4632404H12 gene	4632404H12Rik	0.69385	1.16513689	0.21222595
1417716_at	glutamate oxaloacetate transaminase 2, mitochondrial	Got2	0.69394	1.10020031	0.13480203
1416549_at	solute carrier family 35, member B4	Slc35b4	0.69397	1.11489623	0.15684788
1435374_at	Chromodomain protein, Y chromosome-like 2	Cdyl2	0.69397	-1.09622669	-0.1349834
1438706_at	family with sequence similarity 5, member B	Fam5b	0.69399	1.14412895	0.19399861
1451421_a_at	rogdi homolog (Drosophila)	Rogdi	0.69402	1.10373072	0.14156436
1441646_at	Vps20-associated 1 homolog (S. cerevisiae)	Vta1	0.69405	-1.13850622	-0.1876103
1459780_at	transformation related protein 53	Trp53	0.69407	-1.10145481	-0.1440809
1435250_at	integrator complex subunit 8	Ints8	0.69412	-1.09667411	-0.1349139
1445039_at	---	---	0.69415	1.14577679	0.19170791
1431392_at	RIKEN cDNA 5730596B20 gene /// homeobox A3	5730596B20Rik /// Hoxa3	0.69415	1.17439135	0.21928794
1450088_a_at	myelin-associated oligodendrocytic basic protein	Mobp	0.69416	1.15420389	0.20099128
1453628_s_at	leucine rich repeat containing 2	Lrrc2	0.69416	-1.09977756	-0.1402002
1440616_at	expressed sequence AU042410	AU042410	0.69418	1.14071198	0.18634729
1424725_at	DNA segment, Chr 16, ERATO Doi 472, expressed	D16Ertd472e	0.69419	-1.13960278	-0.1923798
1432625_at	RIKEN cDNA 5830487K18 gene	5830487K18Rik	0.6942	-1.15936886	-0.2263391
1450905_at	plexin C1	Plxnc1	0.6942	-1.1436841	-0.1975386
1443618_at	hypothetical LOC100502711	LOC100502711	0.69422	-1.16417294	-0.2444635
1437173_at	sphingosine-1-phosphate receptor 3	S1pr3	0.69423	-1.1537964	-0.2121095
1420096_at	Zinc finger and SCAN domain containing 21	Zscan21	0.69427	-1.20187348	-0.3266677
1417002_at	RIKEN cDNA 0610012G03 gene	0610012G03Rik	0.69427	1.11952437	0.15528254
1441803_at	interaction protein for cytohesin exchange factors 1	Ipcef1	0.69428	-1.1294071	-0.1813273
1444876_at	---	---	0.69429	-1.13767721	-0.1883624
1440778_x_at	Zinc finger protein 712	Zfp712	0.69436	-1.12304577	-0.1686464
1436938_at	RNA binding motif, single stranded interacting protein	Rbms3	0.69436	1.12889187	0.17480824
1458224_at	---	---	0.69439	-1.14347065	-0.1951162
1444616_x_at	---	---	0.6944	-1.15979692	-0.2329709
1430273_at	RIKEN cDNA 2410087M07 gene	2410087M07Rik	0.69441	-1.13298316	-0.1853193
1438569_at	coiled-coil domain containing 38	Ccdc38	0.69441	1.12677262	0.16908533
1448584_at	arginine/serine-rich coiled-coil 1	Rsrc1	0.69442	-1.11342514	-0.1550457
1455586_at	ring finger protein 168	Rnf168	0.69444	-1.13025798	-0.1801316
1450084_s_at	influenza virus NS1A binding protein	Ivns1abp	0.69445	-1.10620538	-0.1537283
1434489_at	engulfment and cell motility 3, ced-12 homolog (C. elegans)	Elmo3	0.69445	1.13176677	0.17523783
1432174_a_at	Na+/H+ exchanger domain containing 1	Nhedc1	0.69451	1.11988443	0.16306505
1442030_at	---	---	0.69455	1.12693433	0.17105559
1438570_at	---	---	0.69456	1.147244	0.19598186
1418822_a_at	ADP-ribosylation factor 6	Arf6	0.69457	-1.09420962	-0.1344051
1446668_at	---	---	0.69461	-1.13547034	-0.186587
1433599_at	bromodomain adjacent to zinc finger domain 1A	Baz1a	0.6947	-1.10111913	-0.1391121
1418924_at	Ras association (RalGDS/AF-6) domain family (N-terminal) memb	Rassf7	0.69481	1.10353438	0.14068216
1422155_at	histone cluster 2, H3c2, pseudogene	Hist2h3c2-ps	0.69483	-1.1899608	-0.2858994
1453167_at	RIKEN cDNA 1700013G24 gene	1700013G24Rik	0.69483	1.14038796	0.18683041
1417346_at	PYD and CARD domain containing	Pycard	0.69489	1.11139524	0.14839787
1459994_x_at	transferrin receptor 2	Trfr2	0.6949	1.15300791	0.20050347
1428360_x_at	NADH dehydrogenase (ubiquinone) 1 alpha subcomplex, 7 (B14.5	Ndufa7	0.69496	1.08316451	0.11346299
1417656_at	myeloblastosis oncogene-like 2	Mybl2	0.69496	-1.09098465	-0.1284322
1434180_at	fermitin family homolog 2 (Drosophila)	Fermt2	0.69498	-1.08651848	-0.1202174
1448478_at	mediator complex subunit 20	Med20	0.695	1.10132433	0.13834308
1428223_at	major facilitator superfamily domain containing 2A	Mfsd2a	0.69511	1.14186921	0.18749479
1446920_at	---	---	0.69544	1.15928062	0.2072153
1430371_x_at	eukaryotic translation initiation factor 2 alpha kinase 3	Eif2ak3	0.69551	1.18999606	0.23192439
1442688_at	---	---	0.69557	1.19408134	0.23322867
1455032_at	cyclin Y-like 1	Ccnyl1	0.6956	1.09886356	0.13535171
1436783_x_at	tyrosine 3-monooxygenase/tryptophan 5-monooxygenase activa	Ywhab	0.69563	-1.13245528	-0.2027855
1433346_at	RIKEN cDNA 5830432F11 gene	5830432F11Rik	0.69565	-1.12801812	-0.1760529
1431174_at	---	---	0.69567	-1.13303992	-0.1825775
1456492_at	RIKEN cDNA 9130404D08 gene	9130404D08Rik	0.69567	-1.12465885	-0.1728953
1460740_at	clathrin, light polypeptide (Lcb)	Cltb	0.69578	1.11571125	0.15621464
1439546_at	golgi autoantigen, golgin subfamily a, 7B	Golga7b	0.69583	-1.125334	-0.1714691
1417874_at	TMEM9 domain family, member B	Tmem9b	0.69583	1.11422449	0.15501182
1458710_at	---	---	0.69585	-1.13839881	-0.1974157
1451295_a_at	chromodomain helicase DNA binding protein 4	Chd4	0.69588	-1.11310346	-0.1642047
1434140_at	mcf.2 transforming sequence-like	Mcf2l	0.69593	1.1322904	0.17367316
AFFX-MURINE_b	---	---	0.69595	1.17279456	0.20274069
1422114_at	Ets2 repressor factor	Erf	0.69595	-1.19777894	-0.3298971
1445383_at	transmembrane protein 44	Tmem44	0.69599	1.14165127	0.18633306
1453553_at	---	---	0.69606	1.15726357	0.20294635
1425261_at	CCAAT/enhancer binding protein (C/EBP), gamma	Cebpg	0.69609	-1.14058001	-0.1939864
1424108_at	glyoxalase 1	Glo1	0.6961	1.09441153	0.12919755
1450990_at	glypican 3	Gpc3	0.69616	1.13061278	0.17208168
1429643_a_at	phosphodiesterase 1C	Pde1c	0.69617	1.16121324	0.20710983
1453603_at	RIKEN cDNA 2700022O18 gene	2700022O18Rik	0.6962	-1.13558737	-0.1850307
1459499_at	---	---	0.6962	-1.09181815	-0.1282717
1415693_at	Der1-like domain family, member 1	Derl1	0.6963	1.10500095	0.14341908
1417990_at	protein phosphatase 1, regulatory (inhibitor) subunit 14D	Ppp1r14d	0.69631	1.14215548	0.18150449
1430368_s_at	RIKEN cDNA 1700019D03 gene	1700019D03Rik	0.69631	1.10797588	0.14384314
1451417_at	breast cancer 1	Brca1	0.69632	1.1230155	0.16159501
1459208_at	expressed sequence AU021889	AU021889	0.69632	1.13500515	0.17942035
1460208_at	fibrillin 1	Fbn1	0.69633	-1.16272725	-0.2369879
1433787_at	NEL-like 1 (chicken)	Nell1	0.69634	1.10615914	0.14518714
1420719_at	testis expressed gene 15	Tex15	0.69635	1.13954017	0.18612536
1459215_at	---	---	0.69636	-1.16203437	-0.2359063
1419073_at	transmembrane protein with EGF-like and two follistatin-like dom	Tmeff2	0.69637	-1.15204561	-0.2086277
1424044_at	lysine (K)-specific demethylase 4B	Kdm4b	0.69647	1.10945022	0.14974257
1460551_at	RAN, member RAS oncogene family	Ran	0.6965	1.09348238	0.12834015
1419194_s_at	glia maturation factor, gamma	Gmfg	0.69652	1.10963362	0.1491352
1447984_at	DNA segment, Chr 1, ERATO Doi 75, expressed	D1Ertd75e	0.69658	-1.14619037	-0.2060264
1445882_at	CD300 antigen like family member B	Cd300lb	0.69658	-1.13581522	-0.1887793

1416583_at	BCL2-associated agonist of cell death	Bad	0.6966	1.12016432	0.1634548
1421691_at	keratin associated protein 16-7	Krtap16-7	0.6966	-1.17181727	-0.2535846
1425637_at	---	---	0.69664	1.13641546	0.18407011
1440020_at	---	---	0.69671	1.14491438	0.18498525
1422873_at	proteoglycan 2, bone marrow	Prg2	0.69692	1.13102008	0.1771237
1437009_a_at	ring finger protein 115	Rnf115	0.69693	-1.10906941	-0.1528756
1451103_at	HAUS augmin-like complex, subunit 4	Haus4	0.69697	1.09856485	0.13332079
1450585_at	taste receptor, type 2, member 119	Tas2r119	0.69703	-1.16126617	-0.2321847
1428175_at	transmembrane protein 161B	Tmem161b	0.69705	-1.10323813	-0.1422488
1446113_at	---	---	0.69705	1.14847445	0.19776542
1416522_a_at	gene rich cluster, C10 gene	Grcc10	0.69709	1.11011602	0.14590208
1435925_at	G protein-coupled receptor kinase-interactor 2	Git2	0.6974	1.13973107	0.18398227
1415911_at	imprinted and ancient	Impact	0.69758	1.09887881	0.13459175
1426216_at	component of oligomeric golgi complex 6	Cog6	0.69758	1.11226638	0.15187699
1424613_at	G protein-coupled receptor, family C, group 5, member B	Gprc5b	0.69762	1.13970098	0.18469732
1452202_at	phosphodiesterase 2A, cGMP-stimulated	Pde2a	0.69763	1.16149524	0.20576006
1440965_at	phosphatidylinositol glycan anchor biosynthesis, class L	Pigl	0.69769	-1.11387853	-0.1566757
1427833_at	serine protease inhibitor 16	Spi16	0.69769	-1.1306429	-0.1785973
1419446_at	TBC1 domain family, member 1	Tbc1d1	0.6977	1.13682438	0.18077284
1450083_at	CCR4-NOT transcription complex, subunit 4	Cnot4	0.69776	-1.13824664	-0.2015454
1435499_at	leucine zipper-EF-hand containing transmembrane protein 2	Letm2	0.69778	1.18399046	0.22968993
1421061_at	guanylate cyclase activator 1a (retina)	Guca1a	0.69784	-1.13415297	-0.1841927
1418527_a_at	serine/arginine-rich splicing factor 10	Srsf10	0.698	-1.10664267	-0.1516255
1449215_at	solute carrier family 22 (organic cation transporter), member 21	Slc22a21	0.69808	1.12817586	0.1739431
1441248_at	---	---	0.6981	1.14086674	0.18707545
1433193_at	RIKEN cDNA 4930433M22 gene	4930433M22Rik	0.69812	-1.14671608	-0.2034257
1434532_at	cysteine-serine-rich nuclear protein 2	Csrnp2	0.69814	1.10673225	0.14477487
1423227_at	keratin 17	Krt17	0.69819	1.16046949	0.19996998
1433451_at	cyclin-dependent kinase 5, regulatory subunit 1 (p35)	Cdk5r1	0.69824	1.14213038	0.18899943
1444080_at	neuron navigator 2	Nav2	0.69828	-1.1131322	-0.1596103
1429164_at	protease, serine, 36	Prss36	0.69832	1.10592776	0.14440472
1455555_at	vasoactive intestinal peptide receptor 1	Vipr1	0.69833	1.16425314	0.20684341
1443358_s_at	5'-nucleotidase domain containing 3	Nt5dc3	0.69833	1.11217893	0.15057712
1453225_at	RIKEN cDNA A930038C07 gene	A930038C07Rik	0.69834	1.08775438	0.11873555
1447568_at	Glutamyl-tRNA(Gln) amidotransferase, subunit C homolog (bacter	Gatc	0.6984	1.15442858	0.20038591
1419093_at	tryptophan 2,3-dioxygenase	Tdo2	0.69846	1.12640211	0.16654512
1419061_at	ras homolog gene family, member D	Rhod	0.69861	1.13434911	0.17842587
1434726_at	transmembrane protein 146	Tmem146	0.69871	1.18913204	0.23233234
1446101_at	---	---	0.69873	-1.1516392	-0.2162741
1443962_at	transcription factor Dp 2	Tfdp2	0.69874	-1.11280015	-0.157346
1424204_at	mitochondrial ribosomal protein L13	Mrpl13	0.69881	1.10004086	0.13483747
1427154_at	keratin 2	Krt2	0.69882	1.11526467	0.15458188
1427784_at	defensin beta 13	Defb13	0.69882	-1.12839076	-0.1782011
1444166_at	thyroid hormone responsive SPOT14 homolog (Rattus)	Thrsp	0.69882	1.13035249	0.17070629
1433172_at	RIKEN cDNA 3110049I03 gene	3110049I03Rik	0.69884	-1.11518172	-0.1664747
1422759_a_at	exportin 6	Xpo6	0.69886	1.10017435	0.13620235
1430506_at	RIKEN cDNA 8430406P12 gene	8430406P12Rik	0.69888	-1.13012905	-0.1765084
1426855_at	DNA segment, Chr 10, ERATO Doi 610, expressed	D10Ertd610e	0.69889	1.10884487	0.14824248
1451719_at	mediator complex subunit 17	Med17	0.6989	-1.13965584	-0.1909615
1446770_at	phosphatidylinositol 3-kinase catalytic delta polypeptide	Pik3cd	0.69894	-1.13446095	-0.1882323
1444737_at	DNA segment, Chr 2, Wayne State University 107, expressed	D2Wsu107e	0.69895	-1.12837318	-0.17569
1425879_at	zinc finger protein 352	Zfp352	0.69895	-1.14339473	-0.1982235
1427643_at	predicted gene 16499	Gm16499	0.69896	-1.14984646	-0.2076703
1444454_at	---	---	0.69897	-1.13837164	-0.1893242
1429246_a_at	annexin A6	Anxa6	0.69901	1.15077437	0.18774508
1418927_a_at	hyaluronic acid binding protein 4	Habp4	0.69902	1.13945868	0.18455656
1444135_at	Transmembrane protein 229A	Tmem229a	0.69906	1.11818089	0.1602139
1460632_at	retinol dehydrogenase 10 (all-trans)	Rdh10	0.69911	1.14481469	0.19475478
1425469_a_at	---	---	0.69914	-1.10821238	-0.151485
1427969_s_at	zinc finger protein 654	Zfp654	0.69946	-1.11051991	-0.1551617
1438972_x_at	RIKEN cDNA 2810410L24 gene	2810410L24Rik	0.69973	-1.13904205	-0.2042754
1459619_at	erythrocyte protein band 4.1-like 2	Epb4.1l2	0.69974	-1.16691663	-0.2458578
1417532_at	cytochrome P450, family 2, subfamily j, polypeptide 5	Cyp2j5	0.69977	-1.10720627	-0.1560994
1423138_at	WD repeat domain 4	Wdr4	0.69983	1.09779381	0.13452076
1458013_at	RNA binding motif protein 27	Rbm27	0.69984	-1.12095285	-0.1668043
1446063_at	RIKEN cDNA C730034F03 gene	C730034F03Rik	0.69985	-1.12462168	-0.171233
1455960_at	multiple EGF-like-domains 9	Megf9	0.69994	-1.121252	-0.1685528
1429832_at	peptidyl prolyl isomerase H	Ppih	0.69996	1.11517204	0.15419598
1442203_at	hypothetical LOC552880	LOC552880	0.69997	1.16148429	0.20634919
1443067_at	---	---	0.69999	-1.14070068	-0.1924084
1447432_s_at	zinc finger protein 263	Zfp263	0.70001	-1.14153268	-0.2185478
1460291_at	cyclin-dependent kinase 6	Cdk6	0.70006	-1.17546438	-0.2693405
1437688_x_at	ATPase, H+ transporting, lysosomal accessory protein 2	Atp6ap2	0.70008	-1.08998665	-0.1252162
1449742_at	expressed sequence AA522020	AA522020	0.70013	1.14453223	0.18164725
1445861_at	Ubiquitin specific peptidase 25	Usp25	0.70023	-1.14129946	-0.1928217
1426604_at	ribonuclease L (2', 5'-oligoisoadenylate synthetase-dependent)	Rnasel	0.70024	1.14305196	0.18771695
1421778_at	vomeronasal 1, receptor 49	Vmn1r49	0.70027	1.12774297	0.16801755
1457909_at	DNA replication helicase 2 homolog (yeast)	Dna2	0.70027	1.18730293	0.22913219
1431317_at	RIKEN cDNA 2410018L13 gene	2410018L13Rik	0.70028	-1.13079058	-0.1781073
1454354_at	RIKEN cDNA 8030476L19 gene	8030476L19Rik	0.7003	1.13774795	0.18470593
1437655_at	RIKEN cDNA D430020J02 gene	D430020J02Rik	0.70033	1.12892541	0.17136682
1455559_at	pecanex-like 2 (Drosophila)	Pcnxl2	0.70035	-1.16758564	-0.251789
1424378_at	low density lipoprotein receptor adaptor protein 1	Ldlrap1	0.70038	1.1064059	0.14424902
1420086_x_at	fibroblast growth factor 4	Fgf4	0.70038	1.10331403	0.13801512
1438037_at	hect domain and RLD 6	Herc6	0.70038	1.13623536	0.18227247
1434789_at	DEP domain containing 1B	Depdc1b	0.70038	1.10275197	0.13894976
1441519_at	nidogen 1	Nid1	0.70043	1.12976379	0.17571818
1445901_at	low density lipoprotein receptor-related protein 2	Lrp2	0.70044	-1.16495955	-0.2366716
1453635_at	C-type lectin domain family 16, member A	Clec16a	0.70048	1.13065512	0.17442158
1456804_at	predicted gene 6792	Gm6792	0.70052	1.11227124	0.14941126
1449947_s_at	zinc finger homeobox 3	Zfhx3	0.70057	-1.13232377	-0.1872891
1459453_at	---	---	0.70059	1.13963859	0.18743139
1457608_at	---	---	0.70061	-1.13991008	-0.1924155
1458512_at	transducin-like enhancer of split 3, homolog of Drosophila E(spl)	Tle3	0.70061	-1.16922324	-0.2555179

1458708_at	---	---	0.70062	-1.16922429	-0.2617277
1448876_at	Ellis van Creveld gene homolog (human)	Evc	0.70064	1.1153762	0.15625738
1460365_a_at	dynamin 1	Dnm1	0.70065	1.12932838	0.17266179
1460705_at	ribosomal protein S6 kinase, polypeptide 1	Rps6kb1	0.70065	-1.10605973	-0.1487372
1418032_at	integrin alpha FG-GAP repeat containing 2	Itfg2	0.70067	1.11591081	0.15612382
1455345_at	PHD finger protein 15	Phf15	0.70071	1.11665933	0.15380109
1428809_at	RIKEN cDNA 1810010H24 gene	1810010H24Rik	0.70071	1.13585367	0.18161947
1445914_at	Nuclear respiratory factor 1	Nrf1	0.70071	1.15093386	0.19460232
1459668_at	---	---	0.70073	1.13127519	0.17352322
1422617_at	predicted gene 10058 /// predicted gene 10230 /// predicted ger	Gm10058 /// Gm10230 //	0.70079	1.10830356	0.14688865
1457301_at	protein arginine methyltransferase 10 (putative)	Prmt10	0.7008	-1.11238254	-0.1611814
1446627_at	---	---	0.70081	1.16375044	0.20746112
1450509_at	carbohydrate sulfotransferase 11	Chst11	0.70084	-1.14240955	-0.1969306
1452303_at	Rho guanine nucleotide exchange factor (GEF) 10	Arhgef10	0.70086	1.15490573	0.20036714
1459137_at	promyelocytic leukemia	Pml	0.70088	1.16193008	0.20915718
1418767_at	cytochrome P450, family 4, subfamily f, polypeptide 13	Cyp4f13	0.70089	1.12513381	0.1680643
1455031_at	cyclin-dependent kinase 19	Cdk19	0.70096	1.12524662	0.16868319
1457615_at	---	---	0.701	-1.11545519	-0.1601252
1453490_at	spindle assembly 6 homolog (C. elegans)	Sass6	0.70101	1.12418427	0.16723153
1445444_at	hypothetical LOC100503873	LOC100503873	0.70103	1.14504266	0.19203626
1453594_at	RIKEN cDNA 4930451G09 gene	4930451G09Rik	0.70105	1.14782213	0.19328569
1448662_at	frizzled homolog 6 (Drosophila)	Fzd6	0.70111	1.13582792	0.18303817
1418925_at	cadherin, EGF LAG seven-pass G-type receptor 1 (flamingo homo	Celsr1	0.70118	1.09796897	0.13461582
1416242_at	kelch-like 13 (Drosophila)	Klhl13	0.70121	-1.11440029	-0.163973
1439519_at	solute carrier family 34 (sodium phosphate), member 3	Slc34a3	0.70121	1.13966205	0.18332468
1452145_at	hexose-6-phosphate dehydrogenase (glucose 1-dehydrogenase)	H6pd	0.70123	1.13258782	0.17286092
1417726_at	Sjogren's syndrome/scleroderma autoantigen 1 homolog (humar	Sssca1	0.7013	1.10181995	0.13530901
1456839_at	---	---	0.7015	-1.13716028	-0.1873066
1434484_at	RIKEN cDNA 1100001G20 gene	1100001G20Rik	0.70175	1.14630377	0.19583905
1446027_at	---	---	0.7018	1.13407105	0.17930394
1434725_at	GRAM domain containing 1C	Gramd1c	0.70181	1.15723685	0.20019879
1455381_at	RIKEN cDNA 4921513D23 gene	4921513D23Rik	0.70182	-1.14306561	-0.2066949
1430367_at	STAM binding protein like 1	Stambpl1	0.70183	-1.14096167	-0.1908756
1418457_at	chemokine (C-X-C motif) ligand 14	Cxcl14	0.70184	1.1597239	0.20601526
1439931_at	glycogen synthase kinase 3 beta	Gsk3b	0.70187	1.14200775	0.18730516
1419969_at	Expressed sequence C77370	C77370	0.70188	-1.14070365	-0.1999227
1417042_at	solute carrier family 37 (glucose-6-phosphate transporter), memb	Slc37a4	0.70188	1.11222142	0.1523479
1423537_at	growth associated protein 43	Gap43	0.70189	-1.12588265	-0.1761337
1443301_at	---	---	0.70189	1.14745375	0.18953388
1437003_at	---	---	0.70191	-1.11807926	-0.1642499
1434721_at	ankyrin repeat and LEM domain containing 2	Ankle2	0.70192	1.10890523	0.14724623
1431386_s_at	membrane-bound transcription factor peptidase, site 1	Mbtps1	0.70198	1.14223429	0.1868536
1440222_at	---	---	0.70199	-1.14562548	-0.2056266
1426911_at	desmocollin 2	Dsc2	0.70199	1.12549219	0.17016919
1458970_at	---	---	0.70199	-1.15202377	-0.2208287
1436015_s_at	serine/threonine kinase 4	Stk4	0.70201	-1.09327998	-0.130117
1424365_at	RIKEN cDNA 1810037I17 gene	1810037I17Rik	0.70202	1.10382829	0.13741313
1452854_at	SEC63-like (S. cerevisiae)	Sec63	0.70205	1.12198127	0.16247617
1441599_at	EF-hand domain family, member A2	Efha2	0.70207	-1.13205201	-0.1872297
1435901_at	ubiquitin specific peptidase 40	Usp40	0.70211	1.16083794	0.20184885
1444282_at	Sterile alpha motif domain containing 10	Samd10	0.70212	-1.14577925	-0.2078931
1428677_at	WD repeat domain 73	Wdr73	0.70214	1.1050697	0.14285299
1456323_at	protein O-fucosyltransferase 1	Pofut1	0.70216	1.13380982	0.17912009
1453230_at	zinc finger protein 74	Zfp74	0.70218	1.12279878	0.16592947
1445424_at	---	---	0.70218	-1.14087247	-0.1960368
1429168_at	zinc finger and BTB domain containing 46	Zbtb46	0.7022	1.12306259	0.16477206
1451667_at	family with sequence similarity 20, member B	Fam20b	0.7022	-1.12766656	-0.181544
1452727_at	R3H domain containing 2	R3hdm2	0.70221	-1.10352897	-0.1439219
1456522_at	---	---	0.70226	1.15534319	0.197125
1446989_at	---	---	0.70227	-1.10061695	-0.1399353
1453125_at	SRY-box containing gene 11	Sox11	0.70232	1.17957154	0.21971222
1426690_a_at	sterol regulatory element binding transcription factor 1	Srebf1	0.70233	1.09944263	0.13671503
1415692_s_at	calnexin	Canx	0.70234	-1.08588383	-0.1204216
1421955_a_at	neural precursor cell expressed, developmentally down-regulated	Nedd4	0.7024	-1.11808513	-0.1759432
1427253_s_at	suppressor of zeste 12 homolog (Drosophila)	Suz12	0.7024	-1.13951094	-0.2153136
1441941_x_at	serine (or cysteine) peptidase inhibitor, clade B, member 5	Serpinb5	0.70253	1.11384146	0.15172623
1416825_at	syntrophin, acidic 1	Snta1	0.70254	1.10852517	0.14710947
1441372_at	RIKEN cDNA 5930405F01 gene	5930405F01Rik	0.70298	1.18552791	0.22807955
1436803_a_at	NADH dehydrogenase (ubiquinone) 1 beta subcomplex, 9	Ndufb9	0.70305	1.10472089	0.13929348
1446762_at	---	---	0.70306	1.15133209	0.19756716
1420693_at	myomesin 1	Myom1	0.70307	1.12853656	0.17325633
1447415_at	B double prime 1, subunit of RNA polymerase III transcription initi	Bdp1	0.70308	1.1422033	0.18529858
1423128_at	aryl-hydrocarbon receptor-interacting protein	Aip	0.70309	1.09795304	0.13305981
1416682_at	ubiquitin protein ligase E3A	Ube3a	0.70312	-1.1186314	-0.1667023
1436485_s_at	whirlin	Whrn	0.70313	1.1495499	0.1926916
1450030_at	dynactin 4	Dctn4	0.70314	1.12233312	0.16241082
1451201_s_at	ribonuclease/angiogenin inhibitor 1	Rnh1	0.70317	1.10687244	0.1436732
1416008_at	special AT-rich sequence binding protein 1	Satb1	0.70317	1.18499702	0.21934746
1449410_a_at	growth arrest specific 5	Gas5	0.70318	1.12182494	0.15680906
1417818_at	WW domain containing transcription regulator 1	Wwtr1	0.7032	1.11849471	0.15555712
1460091_at	protein phosphatase 1, regulatory (inhibitor) subunit 12B	Ppp1r12b	0.7032	-1.16314526	-0.2400329
1417475_at	ATPase type 13A1	Atp13a1	0.70321	1.10383351	0.1393405
1457417_at	---	---	0.70322	-1.13691389	-0.1857522
1443250_at	regulator of G-protein signaling 2	Rgs2	0.70323	1.13114819	0.17672657
1454196_at	RIKEN cDNA 4930568A13 gene	4930568A13Rik	0.70325	1.10904768	0.14624823
1426402_at	synaptotagmin binding, cytoplasmic RNA interacting protein	Syncrip	0.70326	-1.08725124	-0.1220035
1454527_at	RIKEN cDNA 8430437B07 gene	8430437B07Rik	0.70326	1.11265364	0.15043786
1439750_at	---	---	0.70326	-1.1590626	-0.2322279
1415970_at	cytochrome c oxidase, subunit VIc	Cox6c	0.70327	1.0849272	0.11505723
1442634_at	---	---	0.7033	1.13231339	0.17910928
1454468_at	RIKEN cDNA 5430434N17 gene	5430434N17Rik	0.70332	1.13215411	0.17471376
1424342_at	forty-two-three domain containing 1	Fyttd1	0.70332	-1.10244106	-0.1451472
1421011_at	hydroxysteroid (17-beta) dehydrogenase 11	Hsd17b11	0.70334	1.16896894	0.20719899
1450883_a_at	CD36 antigen	Cd36	0.70334	-1.10170765	-0.1413831

1418128_at	adenylate cyclase 6	Adcy6	0.70334	1.14289384	0.18636183
1460003_at	expressed sequence AI956758	AI956758	0.70334	1.23617081	0.26821884
1427048_at	smoothened homolog (Drosophila)	Smo	0.70337	1.1269653	0.16637138
1460599_at	endoplasmic reticulum metallopeptidase 1	Ermp1	0.70338	1.11791313	0.15651079
1445323_at	gene regulated by estrogen in breast cancer protein	Greb1	0.70339	1.1637008	0.21207106
1448195_at	TAF5-like RNA polymerase II, p300/CBP-associated factor (PCAF)	Taf5l	0.70341	1.10421245	0.14047016
1459677_at	---	---	0.70343	1.22666188	0.26148966
1457939_at	RIKEN cDNA 4930578N16 gene	4930578N16Rik	0.70346	1.13382373	0.180593
1452054_at	ubiquitin-conjugating enzyme E2W (putative)	Ube2w	0.70347	-1.11251808	-0.1559733
1452493_s_at	homeobox B7 /// homeobox B8	Hoxb7 /// Hoxb8	0.70348	-1.13929796	-0.1950627
1448186_at	pancreatic lipase-related protein 2	Pnliprp2	0.70348	1.17325394	0.21817149
1460546_at	leucine-rich repeat LGI family, member 3	Lgi3	0.70348	-1.13978588	-0.1940973
1431342_at	predicted gene 15622	Gm15622	0.70349	-1.13979297	-0.1946436
1453999_at	URB1 ribosome biogenesis 1 homolog (S. cerevisiae)	Urb1	0.70351	-1.13750552	-0.1881186
1427181_at	DNA segment, Chr 18, ERATO Doi 653, expressed	D18Ertd653e	0.70353	1.11048483	0.14744482
1421287_a_at	platelet/endothelial cell adhesion molecule 1	Pecam1	0.70354	1.11551066	0.15379123
1438875_at	zinc finger protein 616	Zfp616	0.70362	1.09753197	0.13150936
1436411_at	ATPase type 13A5	Atp13a5	0.7037	1.11494152	0.1564959
1432196_a_at	Down syndrome cell adhesion molecule-like 1	Dscaml1	0.70374	1.13866483	0.18635493
1421752_a_at	serine (or cysteine) peptidase inhibitor, clade B, member 5	Serpinb5	0.70376	-1.14224932	-0.2019815
1421267_a_at	Cbp/p300-interacting transactivator, with Glu/Asp-rich carboxy-te	Cited2	0.70382	-1.13652718	-0.2049298
1438954_x_at	c-fos induced growth factor	Figf	0.70385	1.17233465	0.21622449
1452730_at	ribosomal protein S4, Y-linked 2	Rps4y2	0.70394	1.05909895	0.08205348
1447493_at	phosphoinositide-interacting regulator of transient receptor poten	Pirt	0.70395	1.13488074	0.18170343
1428111_at	solute carrier family 38, member 4	Slc38a4	0.704	-1.10414556	-0.150342
1456315_a_at	protein tyrosine phosphatase-like (proline instead of catalytic arg	Ptpla	0.70401	1.1132481	0.15190653
1431061_s_at	pellino 1	Peli1	0.70414	1.15575918	0.19812157
1427121_at	F-box protein 4	Fbxo4	0.70415	1.18650814	0.23114855
1452350_at	bromodomain containing 8	Brd8	0.70459	-1.13635163	-0.2007241
1416946_a_at	acetyl-Coenzyme A acyltransferase 1A /// acetyl-Coenzyme A ac	Acaa1a /// Acaa1b	0.70463	1.10312365	0.13968737
1421749_at	Lin-28 homolog (C. elegans)	Lin28	0.70483	-1.15135015	-0.2431839
1434581_at	RIKEN cDNA 2410066E13 gene	2410066E13Rik	0.70489	1.14043695	0.18527989
1453838_at	RIKEN cDNA 4930471G03 gene	4930471G03Rik	0.70493	1.14089463	0.18584281
1441227_at	---	---	0.70502	1.15300162	0.19771333
1446072_at	RRS1 ribosome biogenesis regulator homolog pseudogene	4632415L05Rik	0.70505	-1.12202436	-0.1695048
1431554_a_at	annexin A9	Anxa9	0.70508	-1.11436427	-0.1609568
1424460_s_at	lysophosphatidylcholine acyltransferase 1	Lpcat1	0.70513	1.0930199	0.12711652
1425603_at	transmembrane protein 176A	Tmem176a	0.70514	-1.129188	-0.1766741
1434651_a_at	claudin 3	Cldn3	0.70515	1.1276396	0.16880892
1432119_at	mucin 4	Muc4	0.70516	1.13699597	0.18204833
1419152_at	RIKEN cDNA 2810417H13 gene	2810417H13Rik	0.70524	1.16948225	0.21325862
1434222_at	signal-induced proliferation-associated 1 like 1	Sipa1l1	0.70556	1.10025779	0.13783684
1418619_at	intercellular adhesion molecule 5, telencephalin	Icam5	0.70563	1.12977752	0.17409931
1459444_at	zinc finger protein 91	Zfp91	0.70567	-1.12682147	-0.175193
1446138_at	---	---	0.70568	-1.14269206	-0.2028153
1427087_at	LUC7-like 2 (S. cerevisiae)	Luc7l2	0.7057	-1.10399467	-0.1434256
1418160_at	makorin, ring finger protein, 3	Mkrn3	0.70575	-1.12469869	-0.1736175
1432181_s_at	SCO cytochrome oxidase deficient homolog 2 (yeast)	Sco2	0.70578	-1.11844913	-0.1636852
1432082_at	RIKEN cDNA 4933436I20 gene	4933436I20Rik	0.70579	1.15472787	0.1929308
1438559_x_at	solute carrier family 44, member 2	Slc44a2	0.70585	1.09420577	0.12856965
1440124_at	RIKEN cDNA B230334C09 gene	B230334C09Rik	0.70592	1.11003221	0.15052897
1429588_at	RIKEN cDNA 2810474O19 gene	2810474O19Rik	0.70593	-1.08749424	-0.1215308
1423732_at	translocating chain-associating membrane protein 1	Tram1	0.70597	-1.09946944	-0.1377287
1436125_at	DNA segment, Chr 16, ERATO Doi 472, expressed	D16Ertd472e	0.70603	-1.10878906	-0.1543481
AFFX-b-ActinMur	actin, beta	Actb	0.70606	-1.05295204	-0.0748369
1426512_at	olfactomedin 3	Olfm3	0.70606	-1.1078403	-0.1490245
1422417_at	---	---	0.70608	-1.12558096	-0.1711372
1455051_at	ring finger protein 31	Rnf31	0.70613	1.1186546	0.1575435
1418628_at	KH domain containing, RNA binding, signal transduction associate	Khdrbs1	0.70617	-1.10081009	-0.1399843
1457643_x_at	RIKEN cDNA 2610316D01 gene	2610316D01Rik	0.70621	1.11998978	0.15663959
1450470_at	---	---	0.70622	1.16841603	0.21303397
1439003_s_at	RIKEN cDNA 1700008F21 gene /// zinc finger protein 821	1700008F21Rik /// Zfp82	0.70623	1.16121928	0.20445363
1445669_at	sprouty homolog 4 (Drosophila)	Spry4	0.70624	-1.10326864	-0.1452586
1446381_at	predicted gene 3948	Gm3948	0.70626	1.12212391	0.16371263
1433845_x_at	dual specificity phosphatase 9	Dusp9	0.70627	1.11517867	0.15242331
1448867_at	TMEM9 domain family, member B	Tmem9b	0.70629	1.12168985	0.16200192
1441445_at	period homolog 3 (Drosophila)	Per3	0.70631	-1.14611657	-0.2053275
1437069_at	oxysterol binding protein-like 8	Osbpl8	0.70631	-1.11823427	-0.168504
1448496_a_at	inhibitor of growth family, member 1	Ing1	0.70632	1.08563412	0.11841469
1457123_at	neuregulin 4	Nrg4	0.70633	1.13657865	0.18382332
1453317_a_at	KH domain containing, RNA binding, signal transduction associate	Khdrbs3	0.70634	1.10860623	0.14512133
1431145_a_at	CUE domain containing 2	Cuedc2	0.70634	1.09732379	0.13207557
1448906_at	cadherin 16	Cdh16	0.70634	-1.13236056	-0.179663
1432565_at	predicted gene 15247	Gm15247	0.70634	1.14689942	0.19527238
1424884_at	F-box and WD-40 domain protein 2	Fbxw2	0.70638	-1.11212079	-0.1540779
1453489_at	RIKEN cDNA 1700020O03 gene	1700020O03Rik	0.7064	-1.13677307	-0.1948694
1447947_at	zinc finger, FYVE domain containing 16	Zfyve16	0.7064	-1.12732997	-0.1734041
1445991_at	---	---	0.7064	-1.11885942	-0.1658821
1459985_at	WD repeat domain 61	Wdr61	0.70641	1.08532765	0.11761472
1436103_at	RAB3A interacting protein	Rab3ip	0.70643	1.12018814	0.16333803
1456075_at	protein kinase D2	Prkd2	0.70643	1.12330565	0.16509894
1431884_at	RIKEN cDNA 1110019B22 gene	1110019B22Rik	0.70643	-1.16134148	-0.2318596
1448322_a_at	cytochrome c oxidase subunit IV isoform 1	Cox4i1	0.70644	1.07335475	0.09956681
1441944_s_at	G protein-coupled receptor 135	Gpr135	0.70645	1.11983428	0.16323671
1445103_at	---	---	0.70645	-1.12575292	-0.1733564
1453352_at	ATPase, class V, type 10B	Atp10b	0.70645	-1.14011352	-0.1916143
1449693_at	Mitogen-activated protein kinase kinase kinase 7	Map3k7	0.70645	-1.14783416	-0.2088089
1449120_a_at	pericentriolar material 1	Pcm1	0.70647	-1.15513062	-0.2335064
1459667_at	Rho GTPase activating protein 17	Arhgap17	0.70647	1.12776534	0.17095919
1422236_at	tetratricopeptide repeat domain 39D	Ttc39d	0.70648	1.1328216	0.17862165
1458338_x_at	nuclear receptor subfamily 2, group C, member 1	Nr2c1	0.70648	1.12695683	0.17176991
1445269_at	---	---	0.70648	1.15287252	0.19918284
1439618_at	phosphodiesterase 10A	Pde10a	0.7065	1.10450926	0.14152519
1419477_at	C-type lectin domain family 2, member d	Clec2d	0.70651	1.13570566	0.1808541

1421126_at	ryanodine receptor 2, cardiac	Ryr2	0.70651	1.13215246	0.17854692
1423167_at	MOB1, Mps One Binder kinase activator-like 3 (yeast)	Mobkl3	0.70652	-1.08617075	-0.1204119
1423712_a_at	glutaminyl-tRNA synthetase	Qars	0.70653	1.09100063	0.12499506
1419080_at	glial cell line derived neurotrophic factor	Gdnf	0.70653	-1.14003671	-0.1944084
1429454_at	GTPase activating protein and VPS9 domains 1	Gapvd1	0.70655	1.09945298	0.13525921
1455984_at	zinc finger, MIZ-type containing 1	Zmiz1	0.70655	-1.13687124	-0.1889403
1455127_at	X Kell blood group precursor-related family, member 5	Xkr5	0.70658	1.12939204	0.16838503
1432515_at	RIKEN cDNA 2410124H12 gene	2410124H12Rik	0.70658	-1.13512426	-0.1894249
1447510_at	RIKEN cDNA C530014P21 gene	C530014P21Rik	0.7066	1.20489011	0.24305153
1425473_at	mediator complex subunit 17	Med17	0.70661	1.10365712	0.13800434
1445434_at	snurportin 1	Snupn	0.70662	-1.12977165	-0.1773791
1441701_at	---	---	0.70662	-1.12719276	-0.1747515
1447867_x_at	RIKEN cDNA 4930444A02 gene	4930444A02Rik	0.70664	-1.15126816	-0.2259404
1438690_at	thymidylate synthase	Tyms	0.70664	1.15994866	0.20505243
1452588_at	zinc finger protein 688	Zfp688	0.70666	1.12578428	0.16351446
1423112_at	ubiquitin-conjugating enzyme E2D 3 (UBC4/5 homolog, yeast)	Ube2d3	0.70666	1.11535317	0.1543153
1434322_at	MICAL-like 2	Micall2	0.70667	1.14192158	0.18955563
1440961_at	RIKEN cDNA 9130604C24 gene	9130604C24Rik	0.70668	1.1577098	0.20152576
1460334_at	drebrin-like	Dbnl	0.70671	1.10839109	0.1456302
1428983_at	scleraxis	Scx	0.70672	1.13762687	0.18386833
1453677_a_at	Der1-like domain family, member 3	Derl3	0.70673	1.11425973	0.15356895
1427033_at	dynamin binding protein	Dnmbp	0.70675	1.12560134	0.16294286
1441913_at	leucine zipper protein 2	Luzp2	0.70679	1.10736662	0.14578779
1422103_a_at	signal transducer and activator of transcription 5B	Stat5b	0.70682	1.17959559	0.21582338
1445873_at	Transcription factor Dp 2	Tfdp2	0.70683	-1.13696277	-0.1928055
1420665_at	integrin beta 3 binding protein (beta3-endonexin)	Itgb3bp	0.70686	1.14105229	0.18734982
1449712_s_at	ATPase, H+ transporting, lysosomal V1 subunit E1	Atp6v1e1	0.70686	1.08744739	0.11992284
1458340_at	---	---	0.70686	1.14481767	0.18131393
1450668_s_at	heat shock protein 1 (chaperonin 10)	Hspe1	0.70687	-1.05736393	-0.0813473
1425329_a_at	cytochrome b5 reductase 3	Cyb5r3	0.7069	1.12668405	0.16218548
1418699_s_at	ferrochelatase	Fech	0.70696	1.09640136	0.13181041
1422081_at	kyphoscoliosis peptidase	Ky	0.70697	1.13079414	0.17596553
1416237_at	myelin protein zero-like 2	Mpzl2	0.70699	-1.13312752	-0.1928772
1444790_at	heat shock factor binding protein 1-like 1	Hsbp1l1	0.7071	1.23380108	0.26538861
1454043_a_at	potassium voltage-gated channel, shaker-related subfamily, beta	Kcnab1	0.70761	-1.1465727	-0.2041675
1447545_at	DNA segment, Chr 7, ERATO Doi 193, expressed	D7Ertd193e	0.70762	1.13052066	0.1748843
1419475_a_at	ets homologous factor	Ehf	0.70827	1.1320887	0.17088924
1417651_at	cytochrome P450, family 2, subfamily c, polypeptide 29	Cyp2c29	0.70831	-1.15868268	-0.2343827
1417616_at	ST6 (alpha-N-acetyl-neuraminyl-2,3-beta-galactosyl-1,3)-N-acety	St6galnac2	0.70844	1.1411493	0.18691188
1439893_at	---	---	0.70851	-1.15877767	-0.2336216
1435924_at	transcription factor B1, mitochondrial	Tfb1m	0.70864	1.10792726	0.14735756
1447923_at	RIKEN cDNA 1810026B05 gene	1810026B05Rik	0.70866	-1.11165193	-0.1598295
1448918_at	solute carrier organic anion transporter family, member 3a1	Slco3a1	0.70875	-1.14896464	-0.2162102
1420460_a_at	peroxisomal biogenesis factor 11 beta	Pex11b	0.70879	1.10305687	0.14121501
1424609_a_at	predicted gene 4354	Gm4354	0.70884	-1.14097013	-0.2161184
1429360_at	Kruppel-like factor 3 (basic)	Klf3	0.70885	1.15187861	0.19122505
1427619_a_at	SH3 domain and tetratricopeptide repeats 1	Sh3tc1	0.70887	1.11492324	0.15593277
1438285_at	RIKEN cDNA 2210015D19 gene	2210015D19Rik	0.70888	1.11450553	0.15538444
1423153_x_at	complement component factor h	Cfh	0.7089	1.09295941	0.12614364
1451454_at	protocadherin 20	Pcdh20	0.70892	1.16957292	0.20539533
1447892_at	glyceraldehyde-3-phosphate dehydrogenase pseudogene	Gm7788	0.70897	-1.14852622	-0.2050899
1448510_at	ephrin A1	Efna1	0.70897	1.18869365	0.23022269
1450808_at	formyl peptide receptor 1	Fpr1	0.70898	1.16740399	0.21071997
1441264_x_at	RIKEN cDNA A930005H10 gene	A930005H10Rik	0.70901	1.11370833	0.15391057
1437948_x_at	eukaryotic translation initiation factor 3, subunit L	Eif3l	0.70904	-1.14216215	-0.1996596
1449630_s_at	MAP/microtubule affinity-regulating kinase 1	Mark1	0.70904	1.14687516	0.19299597
1442948_at	expressed sequence C79452	C79452	0.70905	-1.13431434	-0.1871657
1448001_x_at	cell division cycle associated 3	Cdca3	0.70907	-1.13676099	-0.1858863
1440264_at	---	---	0.70907	-1.136098	-0.1910249
1431313_at	Rho GTPase activating protein 27 /// SH3 domain containing 20	Arhgap27 /// Sh3d20	0.70909	1.14227897	0.18890897
1453014_a_at	Sec31 homolog A (S. cerevisiae)	Sec31a	0.7091	1.10062516	0.13612852
1446766_at	expressed sequence AU022746	AU022746	0.70911	1.12942882	0.17470231
1448530_at	guanosine monophosphate reductase	Gmpr	0.70916	1.13720445	0.17787853
1451890_at	kinesin family member 13A	Kif13a	0.70919	1.14486958	0.18732456
1446519_at	RIKEN cDNA A730091E23 gene	A730091E23Rik	0.70922	1.16061035	0.20573754
1448351_at	coronin, actin binding protein 1B	Coro1b	0.70923	1.09617729	0.13232141
1416616_s_at	caseinolytic peptidase, ATP-dependent, proteolytic subunit homol	Clpp	0.70924	1.10861589	0.14336949
1445397_at	---	---	0.70928	-1.13022451	-0.1793924
1427661_a_at	tumor-suppressing subchromosomal transferable fragment 4	Tssc4	0.70928	1.12970842	0.16756019
1425831_at	zinc finger protein 101	Zfp101	0.70929	1.15888943	0.20171565
1436347_a_at	RIKEN cDNA 5530601H04 gene	5530601H04Rik	0.7093	1.12717591	0.17217977
1430825_at	RIKEN cDNA E130102C15 gene	E130102C15Rik	0.70931	1.13714834	0.18494606
1442784_at	Zinc finger protein 608	Zfp608	0.70932	-1.13106977	-0.1781406
1457534_at	---	---	0.70933	-1.15157544	-0.2167656
1436441_at	---	---	0.70933	-1.14019278	-0.1948673
1438846_x_at	inositol hexaphosphate kinase 1	Ip6k1	0.70934	-1.11956437	-0.1703174
1441050_at	---	---	0.70934	-1.14577066	-0.2076213
1417690_at	protein kinase, AMP-activated, gamma 1 non-catalytic subunit	Prkag1	0.70935	1.09214146	0.12642444
1441969_at	tripartite motif-containing 36	Trim36	0.70936	1.19668332	0.2363314
1428816_a_at	GATA binding protein 2	Gata2	0.70938	1.14549339	0.19205407
1418070_at	chromodomain protein, Y chromosome-like	Cdyl	0.7094	-1.09765752	-0.1375083
1454617_at	arrestin domain containing 3	Arrdc3	0.7094	-1.11839914	-0.1682094
1456676_a_at	6-phosphofructo-2-kinase/fructose-2,6-biphosphatase 3	Pfkfb3	0.70943	1.13952407	0.18530924
1427327_at	paired immunoglobin-like type 2 receptor alpha	Pilra	0.70946	-1.15008004	-0.2081433
1432214_at	RIKEN cDNA 1700017D01 gene	1700017D01Rik	0.70947	1.11322666	0.15186685
1451544_at	TAP binding protein-like	Tapbpl	0.70949	1.18064666	0.22240362
1432167_at	---	---	0.70949	1.15247243	0.19858351
1435174_at	rosbin, round spermatid basic protein 1	Rsbn1	0.7095	-1.09760112	-0.1347109
1415854_at	kit ligand	Kitl	0.70953	1.12691396	0.17194343
1449707_at	Nuclear receptor subfamily 5, group A, member 2	Nr5a2	0.70964	-1.14812476	-0.2052677
1428270_at	glycosyltransferase 8 domain containing 1	Glt8d1	0.70965	1.12865521	0.16778647
1447475_at	family with sequence similarity 189, member A2	Fam189a2	0.70973	1.15721292	0.20148758
1460324_at	DNA methyltransferase 3A	Dnmt3a	0.70976	-1.1214434	-0.1671435
1433367_at	RIKEN cDNA 8430401P03 gene	8430401P03Rik	0.70976	-1.16415469	-0.2474505

1443165_at	mitochondrial ribosomal protein S31	Mrps31	0.71001	-1.13983616	-0.1979942
1422300_at	noggin	Nog	0.71011	-1.13840512	-0.192979
1427528_a_at	Eph receptor A7	Epha7	0.71012	1.14709666	0.19322854
1451678_at	nuclear prelamin A recognition factor	Narf	0.71012	1.11207213	0.15174123
1433565_at	PRP38 pre-mRNA processing factor 38 (yeast) domain containing	Prpf38a	0.71015	1.08527496	0.11794185
1453671_at	RIKEN cDNA 9430041J12 gene	9430041J12Rik	0.71023	-1.12659604	-0.1728441
1430016_at	RIKEN cDNA 4930584F24 gene	4930584F24Rik	0.71024	1.15137126	0.19354488
1456607_at	valosin containing protein (p97)/p47 complex interacting protein 1	Vcpip1	0.71024	-1.13290966	-0.1895543
1423535_at	striatin, calmodulin binding protein 3	Strn3	0.71025	-1.0941906	-0.1322996
1436758_at	histone deacetylase 4	Hdac4	0.71026	1.15977981	0.19673566
1440974_at	RIKEN cDNA 9030203C11 gene	9030203C11Rik	0.71027	1.13438627	0.18028233
1447701_x_at	isocitrate dehydrogenase 3 (NAD+) alpha	Idh3a	0.71029	-1.12777203	-0.1781747
1420836_at	solute carrier family 25, member 30	Slc25a30	0.7103	1.13654515	0.17453053
1453311_at	RIKEN cDNA 2310008B10 gene	2310008B10Rik	0.71033	-1.13266965	-0.1838835
1423380_s_at	nuclear factor of activated T-cells, cytoplasmic, calcineurin-depend	Nfatc4	0.71034	1.10699233	0.14540256
1416578_at	ring-box 1	Rbx1	0.71034	1.08531904	0.1181108
1420985_at	ash1 (absent, small, or homeotic)-like (Drosophila)	Ash1l	0.71039	-1.12022355	-0.1667304
1436534_at	TROVE domain family, member 2	Trove2	0.71039	-1.10442629	-0.145392
1429659_at	structural maintenance of chromosomes 2	Smc2	0.71042	-1.13714549	-0.2038107
1417769_at	proteasome (prosome, macropain) 26S subunit, ATPase, 6	Psmc6	0.71043	-1.1292014	-0.1881103
1437571_at	hypermethylated in cancer 2	Hic2	0.71045	-1.09968878	-0.1372952
1460569_x_at	claudin 3	Cldn3	0.71049	1.15130682	0.19300151
1437701_at	Smith-Magenis syndrome chromosome region, candidate 8 homo	Smcr8	0.71051	-1.13525082	-0.1855975
1424504_at	RAB22A, member RAS oncogene family	Rab22a	0.71052	-1.15084782	-0.212294
1460677_at	spermatogenesis associated, serine-rich 2	Spats2	0.71054	1.10348846	0.13773244
1424879_at	leucine-rich repeats and calponin homology (CH) domain containi	Lrch4 /// Lrch4-sap25 ///	0.71082	1.09593491	0.13168764
1416023_at	fatty acid binding protein 3, muscle and heart	Fabp3	0.71092	1.11081798	0.14510617
1438679_at	Tripartite motif-containing 8	Trim8	0.71093	-1.12371108	-0.1711266
1452093_at	transmembrane protein 185B	Tmem185b	0.71097	-1.10455921	-0.1464363
1436229_at	family with sequence similarity 126, member B	Fam126b	0.71102	-1.12222526	-0.1715168
1442836_at	exportin 7	Xpo7	0.71104	-1.11053965	-0.153199
1424224_at	ankyrin repeat and SOCS box-containing 8	Asb8	0.71106	1.10773368	0.14476682
1425400_a_at	Cbp/p300-interacting transactivator, with Glu/Asp-rich carboxy-te	Cited4	0.71111	-1.17517345	-0.278071
1416756_at	DnaJ (Hsp40) homolog, subfamily B, member 1	Dnajb1	0.71114	1.09545094	0.1311602
1442892_at	---	---	0.71117	1.15519922	0.19842945
1438325_at	MDS1 and EVI1 complex locus	Mecom	0.71118	1.16675738	0.21189771
1423169_at	TAF7 RNA polymerase II, TATA box binding protein (TBP)-associat	Taf7	0.71119	-1.09336325	-0.1300009
1452380_at	Eph receptor A7	Epha7	0.71125	-1.09737738	-0.1423936
1447944_at	zinc finger with KRAB and SCAN domains 1	Zkscan1	0.71125	-1.13212455	-0.1806598
1425322_at	predicted gene 13271 /// predicted gene 13275 /// predicted gen	Gm13271 /// Gm13275 //	0.71128	1.12828158	0.17106054
1454984_at	leukemia inhibitory factor receptor	Lifr	0.71128	-1.09886708	-0.1370845
1429019_s_at	paraoxonase 2	Pon2	0.71129	1.1185339	0.15973354
1454355_at	RIKEN cDNA 1810021M19 gene	1810021M19Rik	0.71129	-1.13537074	-0.1879234
1419547_at	fumarylacetoacetate hydrolase domain containing 1	Fahd1	0.71131	1.13796133	0.18013589
1444795_at	expressed sequence AU015723	AU015723	0.71134	-1.13412106	-0.1855643
1423688_at	mannosidase, alpha, class 2C, member 1	Man2c1	0.71137	1.08945629	0.12355454
1426225_at	retinol binding protein 4, plasma	Rbp4	0.71138	-1.128844	-0.1755433
1424268_at	spermine oxidase	Smox	0.71138	1.12477947	0.16496311
1417296_at	activating transcription factor 1	Atf1	0.71141	-1.08470976	-0.1192338
1434602_at	mediator complex subunit 13-like	Med13l	0.71146	-1.12204436	-0.178025
1455507_s_at	ataxin 1-like	Atxn1l	0.71147	-1.11779185	-0.1670398
1436767_at	LUC7-like 2 (S. cerevisiae)	Luc7l2	0.71167	-1.14792626	-0.224584
1435905_at	family with sequence similarity 48, member A	Fam48a	0.71175	1.12327592	0.16243835
1423658_at	signal peptide peptidase 3	Sppl3	0.71181	1.10272341	0.13923389
1446416_at	sulfatase 1	Sulf1	0.71184	1.13518767	0.17970898
1422444_at	integrin alpha 6	Itga6	0.71193	1.1013678	0.13564365
1435351_at	BEN domain containing 5	Bend5	0.71201	1.14492457	0.18723915
1433690_at	RIKEN cDNA 2210016L21 gene	2210016L21Rik	0.71206	1.10459014	0.14263365
1450081_x_at	glucose phosphate isomerase 1	Gpi1	0.71208	1.08702507	0.11912626
1446685_at	predicted gene 7455	Gm7455	0.71208	-1.12277066	-0.1789629
1450569_a_at	RNA binding motif protein 14	Rbm14	0.71211	1.08732083	0.11941184
1438929_at	---	---	0.71216	-1.15778883	-0.2266752
1443089_at	exportin 7	Xpo7	0.71224	1.13477984	0.18198161
1419238_at	ATP-binding cassette, sub-family A (ABC1), member 7	Abca7	0.71234	1.12797526	0.16843694
1420473_at	myotrophin	Mtpn	0.71242	-1.08265663	-0.1148377
1428491_at	COMM domain containing 10	Commd10	0.71244	-1.0906921	-0.1271162
1437149_at	solute carrier family 6 (neurotransmitter transporter, taurine), me	Slc6a6	0.71245	-1.1072349	-0.1564471
1456720_at	---	---	0.71246	-1.14253804	-0.2035796
1422483_a_at	cytochrome c, somatic	Cycs	0.71246	1.07612806	0.10549276
1447305_at	---	---	0.71247	1.08230947	0.11395699
1451124_at	superoxide dismutase 1, soluble	Sod1	0.71249	-1.13529697	-0.2122513
1458558_at	---	---	0.71274	1.11309638	0.15384497
1421315_s_at	cortactin	Cttn	0.71281	1.07567623	0.10332873
1424418_at	solute carrier family 25, member 38	Slc25a38	0.71283	1.10830709	0.14697591
1452346_at	UDP-GlcNAc:betaGal beta-1,3-N-acetylglucosaminyltransferase 1	B3gnt1	0.71286	1.12187359	0.16414448
1419568_at	mitogen-activated protein kinase 1	Mapk1	0.71291	1.09814783	0.13415789
1433443_a_at	3-hydroxy-3-methylglutaryl-Coenzyme A synthase 1	Hmgcs1	0.71301	1.08403908	0.11567962
1429505_at	neurobeachin like 1	Nbeal1	0.71308	-1.12313247	-0.1770234
1442563_at	Transmembrane protein 8 (five membrane-spanning domains)	Tmem8	0.71315	-1.15042261	-0.2202064
1440170_at	ankyrin repeat domain 10	Ankrd10	0.71318	1.11133906	0.1500146
1424859_at	homer homolog 3 (Drosophila)	Homer3	0.71319	1.18243873	0.21902431
1451552_at	lipoyltransferase 1	Lipt1	0.71321	1.11914687	0.15981506
1443560_at	predicted gene 10941	Gm10941	0.71321	-1.13205262	-0.1831804
1450695_at	aryl-hydrocarbon receptor	Ahr	0.71322	-1.15041534	-0.21765
1459612_at	---	---	0.71326	1.16462158	0.20816592
1457370_at	hypothetical protein LOC100504612	LOC100504612	0.7133	-1.1235513	-0.1688124
1416341_at	polymerase (RNA) II (DNA directed) polypeptide C	Polr2c	0.71333	1.08586581	0.11838532
1450404_at	solute carrier family 23 (nucleobase transporters), member 1	Slc23a1	0.71335	1.12982139	0.17271726
1450641_at	vimentin	Vim	0.71336	1.08111648	0.112283
1457497_at	---	---	0.71337	-1.13339222	-0.1894932
1426474_at	ATP synthase mitochondrial F1 complex assembly factor 2	Atpaf2	0.71339	1.10457682	0.14232936
1447400_at	receptor-like tyrosine kinase	Ryk	0.71339	1.14152777	0.18691122
1456481_at	extended synaptotagmin-like protein 3	Esyt3	0.7134	1.14796448	0.18771404
1447653_x_at	ribosomal protein L24	Rpl24	0.71341	-1.11896192	-0.1766042

1429458_at	RIKEN cDNA 2410127L17 gene	2410127L17Rik	0.71341	-1.10350714	-0.151713
1423390_at	seven in absentia 1A	Siah1a	0.71341	1.10736567	0.14408526
1442929_at	---	---	0.71342	-1.13579316	-0.1893431
1452203_at	oligonucleotide/oligosaccharide-binding fold containing 2A	Obfc2a	0.71344	-1.10752309	-0.1541325
1455694_at	neurobeachin-like 2	Nbeal2	0.71345	1.11891961	0.15991755
1434294_at	family with sequence similarity 199, X-linked	Fam199x	0.71346	-1.11765901	-0.1673282
1443322_at	---	---	0.71347	1.11877727	0.1597971
1420789_at	killer cell lectin-like receptor, subfamily A, member 5	Klra5	0.71349	1.20960696	0.24758739
1415749_a_at	Ras-related GTP binding C	Rragc	0.71351	1.09014584	0.12404454
1425602_a_at	rabaptin, RAB GTPase binding effector protein 2	Rabep2	0.71363	1.12153434	0.16391284
1419806_at	high density lipoprotein (HDL) binding protein	Hdlbp	0.7137	-1.12246553	-0.1678935
1444239_at	Gem (nuclear organelle) associated protein 8	Gemin8	0.71398	1.13862483	0.18480432
1437122_at	B-cell leukemia/lymphoma 2	Bcl2	0.71407	1.34347857	0.34317298
1449407_at	intraflagellar transport 81 homolog (Chlamydomonas)	Ift81	0.71409	1.11357593	0.15466335
1442599_at	solute carrier family 12 (potassium/chloride transporters), membe	Slc12a9	0.71416	1.19043206	0.23220129
1443828_x_at	HERPUD family member 2	Herpud2	0.71422	-1.13413855	-0.192351
1430546_at	crystallin, zeta (quinone reductase)-like 1	Cryzl1	0.71426	-1.10794519	-0.153602
1442925_at	SMG1 homolog, phosphatidylinositol 3-kinase-related kinase (C.	Smg1	0.71429	1.15406835	0.1955061
1440217_at	family with sequence similarity 169, member B	Fam169b	0.71434	-1.13021849	-0.178347
1452990_at	metal response element binding transcription factor 1	Mtf1	0.71437	-1.13470248	-0.187111
1432559_at	RIKEN cDNA 2210409D07 gene	2210409D07Rik	0.7144	-1.14925012	-0.2146686
1447225_at	Solute carrier family 16 (monocarboxylic acid transporters), meml	Slc16a5	0.71492	-1.14428884	-0.2104588
1444783_at	---	---	0.71499	-1.13556	-0.1886147
1422482_at	RuvB-like protein 2	Ruvbl2	0.71511	1.08499096	0.11511377
1456255_at	expressed sequence AI314180	AI314180	0.71524	-1.15844248	-0.2316551
1437533_at	X-linked inhibitor of apoptosis	Xiap	0.71558	-1.09246263	-0.1281326
1451883_at	cDNA sequence BC002189	BC002189	0.71561	1.1324859	0.17933829
1453119_at	OTU domain containing 1	Otud1	0.71595	-1.12491921	-0.1797818
1420270_at	---	---	0.71617	-1.11624993	-0.1598868
1453994_at	echinoderm microtubule associated protein like 6	Eml6	0.7162	1.16438718	0.20772174
1451315_at	transmembrane protein 101	Tmem101	0.71624	1.12227543	0.1627539
1427673_a_at	sema domain, immunoglobulin domain (Ig), short basic domain, s	Sema3e	0.7163	-1.12982112	-0.1791036
1420614_at	dynein light chain Tctex-type 3	Dynlt3	0.7163	-1.11294969	-0.1567117
1450702_at	hemochromatosis	Hfe	0.71631	-1.12602507	-0.1742203
1441520_at	asp (abnormal spindle)-like, microcephaly associated (Drosophila	Aspm	0.71632	-1.22264898	-0.418444
1424920_at	solute carrier family 37 (glycerol-3-phosphate transporter), memb	Slc37a3	0.71636	-1.13553792	-0.1909618
1433674_a_at	small nucleolar RNA host gene (non-protein coding) 1	Snhg1	0.71638	1.08525627	0.1175441
1422998_a_at	glutaredoxin 2 (thioltransferase)	Glrx2	0.71642	-1.09748708	-0.1369374
1447256_at	---	---	0.71643	1.16433496	0.20431145
1416731_at	topoisomerase (DNA) II beta	Top2b	0.71644	-1.10196522	-0.1407265
1437967_at	coiled-coil domain containing 141	Ccdc141	0.71644	1.19083797	0.23171764
1429710_at	serine/threonine/tyrosine interaction protein	Styx	0.71648	-1.11200891	-0.158259
1426722_at	solute carrier family 38, member 2	Slc38a2	0.71651	-1.08102239	-0.1129122
1443020_at	homeobox containing 1	Hmbox1	0.71651	-1.15503391	-0.2301138
1430738_at	myozenin 3	Myoz3	0.71652	1.18202514	0.22197766
1420118_s_at	---	---	0.71653	-1.14808405	-0.2157493
1455069_x_at	solute carrier family 25 (mitochondrial carrier, adenine nucleotide	Slc25a4	0.71654	1.0895381	0.12246229
1417770_s_at	proteasome (prosome, macropain) 26S subunit, ATPase, 6	Psmc6	0.7166	-1.09015781	-0.1245715
1417358_s_at	sorbin and SH3 domain containing 1	Sorbs1	0.71661	-1.13651	-0.1904158
1453864_at	retinol dehydrogenase 14 (all-trans and 9-cis)	Rdh14	0.71665	-1.16101418	-0.2369613
1417094_at	acyl-CoA thioesterase 7	Acot7	0.71667	1.09184657	0.12673694
1445022_at	---	---	0.71671	-1.11483811	-0.1597444
1418262_at	spleen tyrosine kinase	Sykb	0.71671	1.13534947	0.17527996
1433909_at	synaptotagmin XVII	Syt17	0.71675	1.14160911	0.18762552
1426003_at	neurotrophic tyrosine kinase, receptor, type 3	Ntrk3	0.71689	1.13607247	0.18232992
1433100_at	lipase maturation factor 2	Lmf2	0.71691	-1.1320774	-0.1843089
1456670_at	RIKEN cDNA A930007A09 gene	A930007A09Rik	0.71692	-1.12838998	-0.1770777
1433940_at	sperm associated antigen 7	Spag7	0.71695	1.09417549	0.12796457
1455906_at	RIKEN cDNA 6030446N20 gene	6030446N20Rik	0.717	1.15690698	0.19946071
1416615_at	caseinolytic peptidase, ATP-dependent, proteolytic subunit homol	Clpp	0.71703	1.10382412	0.13809029
1449617_at	RIKEN cDNA 2900092E17 gene	2900092E17Rik	0.7171	1.11460597	0.15634599
1421817_at	glutathione reductase	Gsr	0.71713	1.09976703	0.13450197
1436571_at	---	---	0.71716	1.12491976	0.16513077
1446633_at	autophagy-related 7 (yeast)	Atg7	0.71717	1.19212196	0.22875538
1436779_at	cytochrome b-245, beta polypeptide	Cybb	0.71717	-1.12877598	-0.1747688
1444407_at	CAMP responsive element binding protein-like 2	Crebl2	0.71722	-1.13758215	-0.1901815
1434991_at	F-box and WD-40 domain protein 17	Fbxw17	0.71722	1.11568071	0.15547142
1439526_at	---	---	0.71725	1.12383625	0.16574825
1439014_at	RIKEN cDNA 1600021P15 gene	1600021P15Rik	0.71727	-1.09506248	-0.1321838
1415721_a_at	N-acetyltransferase 15 (GCN5-related, putative)	Nat15	0.7173	1.09243927	0.12755041
1430922_at	RIKEN cDNA 5530401A14 gene	5530401A14Rik	0.71733	1.1338258	0.1800352
1433572_a_at	family with sequence similarity 120, member A	Fam120a	0.71735	-1.09604698	-0.1371095
1423641_s_at	CCR4-NOT transcription complex, subunit 7	Cnot7	0.71736	-1.09361774	-0.129726
1423294_at	mesoderm specific transcript	Mest	0.71741	1.09523433	0.12852451
1454777_at	solute carrier organic anion transporter family, member 2b1	Slco2b1	0.71745	1.09269774	0.12752534
1443953_at	testis expressed gene 2	Tex2	0.71746	-1.12916519	-0.1772975
1432528_at	RIKEN cDNA 1700021P04 gene	1700021P04Rik	0.71749	-1.13841106	-0.1953888
1451816_at	zinc finger protein 451	Zfp451	0.71751	1.15426815	0.19657187
1457596_at	---	---	0.71752	-1.12637333	-0.1828443
1423092_at	inner centromere protein	Incenp	0.71752	1.09580009	0.12948611
1448663_s_at	mevalonate (diphospho) decarboxylase	Mvd	0.71752	1.12578128	0.16266879
1424765_at	epidermal growth factor receptor pathway substrate 15-like 1	Eps15l1	0.71753	1.11026773	0.14800242
1445456_at	---	---	0.71754	-1.12655767	-0.1756346
1432302_s_at	RIKEN cDNA 2410024N13 gene	2410024N13Rik	0.71754	1.1490046	0.18508508
1421997_s_at	integrin alpha 3	Itga3	0.71754	1.15637257	0.19033076
1434801_x_at	solute carrier family 25 (mitochondrial carrier, adenine nucleotide	Slc25a5	0.71755	1.05752798	0.08019793
1438427_at	family with sequence similarity 120, member B	Fam120b	0.71757	1.12403905	0.1671764
1446670_at	---	---	0.71758	1.13289012	0.17720162
1450383_at	low density lipoprotein receptor	Ldlr	0.7176	1.10992475	0.1485425
1417844_at	mediator of RNA polymerase II transcription, subunit 4 homolog (	Med4	0.7176	1.09757398	0.13052042
1447220_at	---	---	0.71761	-1.12410468	-0.1688476
1437687_x_at	FK506 binding protein 9	Fkbp9	0.71763	-1.08797073	-0.1229261
1439728_at	proline-rich transmembrane protein 4	Prrt4	0.71768	1.13414358	0.18129134
1459122_at	sperm flagellar 2	Spef2	0.71771	1.08137761	0.11250102

1424234_s_at	mesenchyme homeobox 2	Meox2	0.71775	1.13674878	0.17857542
1451381_at	RIKEN cDNA 1810020D17 gene	1810020D17Rik	0.71776	1.12649302	0.16590362
1423398_at	TAF12 RNA polymerase II, TATA box binding protein (TBP)-associ	Taf12	0.71778	1.11298894	0.1513595
1429983_at	RIKEN cDNA 2010002M09 gene	2010002M09Rik	0.71781	-1.14160123	-0.201193
1450741_at	staufen (RNA binding protein) homolog 1 (Drosophila)	Stau1	0.71783	-1.11949646	-0.1732625
1419449_a_at	guanine nucleotide binding protein (G protein), alpha inhibiting 2	Gnai2	0.71783	-1.15807985	-0.2637904
1454377_at	RIKEN cDNA 2700080J24 gene	2700080J24Rik	0.71784	-1.14005709	-0.1952202
1430178_at	RIKEN cDNA 2810408A11 gene	2810408A11Rik	0.71789	1.15348103	0.19663657
1417010_at	zinc finger protein 238	Zfp238	0.71789	-1.11445944	-0.1628711
1426581_at	protein tyrosine phosphatase, mitochondrial 1	Ptpmt1	0.71792	1.11461058	0.14842771
1447706_at	---	---	0.71794	1.16002521	0.20427137
1441733_s_at	nucleoporin 153	Nup153	0.71795	-1.10217178	-0.1454431
1427802_a_at	T-cell receptor gamma, variable 4	Tcrg-V4	0.71797	1.10028021	0.13615354
1429329_at	COX10 homolog, cytochrome c oxidase assembly protein, heme A	Cox10	0.71797	1.11386491	0.15244599
1447382_at	phosphatidylinositol glycan anchor biosynthesis, class T	Pigt	0.71797	1.17293756	0.21572389
1445548_at	---	---	0.71799	-1.1256424	-0.1715184
1434215_at	RIKEN cDNA B230308N11 gene	B230308N11Rik	0.71799	-1.10153838	-0.142115
1421340_at	mitogen-activated protein kinase kinase kinase 5	Map3k5	0.71801	1.13823495	0.18170016
1433695_at	cannabinoid receptor interacting protein 1	Cnrip1	0.71807	-1.13720133	-0.1914619
1436647_at	tau tubulin kinase 2	Ttbk2	0.71815	1.15454288	0.19470266
1420276_x_at	multimerin 2	Mmrn2	0.71817	-1.10409585	-0.1456619
1425317_x_at	serine threonine kinase 31	Stk31	0.7182	-1.15038105	-0.2200158
1449569_at	thrombopoietin	Thpo	0.71822	1.12420138	0.16702356
1460667_at	cDNA sequence U90926	U90926	0.71824	-1.13303769	-0.1835313
1433914_at	lipase, member O1	Lipo1	0.71826	-1.11008044	-0.1510651
1450019_at	chemokine (C-X3-C) receptor 1	Cx3cr1	0.71827	-1.16877428	-0.2514497
1441477_at	calumenin	Calu	0.71832	1.20433518	0.24262691
1454627_a_at	predicted gene 10709 /// ribosomal protein L29 pseudogene ///	Gm10709 /// Gm6344 ///	0.71834	1.0559412	0.07717179
1424233_at	mesenchyme homeobox 2	Meox2	0.71837	1.10686584	0.14512482
1423586_at	AXL receptor tyrosine kinase	Axl	0.71909	1.13287723	0.17939032
1428284_at	RIKEN cDNA 8430427H17 gene	8430427H17Rik	0.71934	-1.12409154	-0.1732985
1417313_at	LSM7 homolog, U6 small nuclear RNA associated (S. cerevisiae)	Lsm7	0.71939	1.09978633	0.13307564
1447683_x_at	methyltransferase like 1	Mettl1	0.71944	1.17268036	0.20646751
1451922_at	lectin, mannose-binding 1 like	Lman1l	0.71949	1.16262773	0.20402687
1442125_at	KRIT1, ankyrin repeat containing	Krit1	0.71951	-1.10432003	-0.1439754
1416166_a_at	peroxiredoxin 4	Prdx4	0.71953	-1.19380164	-0.353442
1439175_at	---	---	0.7196	-1.15064375	-0.2130547
1423832_at	protein kinase, AMP-activated, gamma 2 non-catalytic subunit	Prkag2	0.71961	-1.15868323	-0.2411344
1416779_at	serum deprivation response	Sdpr	0.71964	1.11900154	0.15696901
1449016_at	zona pellucida glycoprotein 2	Zp2	0.71967	-1.12408041	-0.1692773
1416673_at	beta-site APP-cleaving enzyme 2	Bace2	0.7197	-1.1322772	-0.1866646
1423917_a_at	cortactin	Cttn	0.71971	1.08892977	0.12287735
1436128_at	pleckstrin homology domain containing, family A (phosphoinositi	Plekha8	0.71974	1.13410286	0.17315995
1417107_at	tumor protein D52-like 2	Tpd52l2	0.71978	1.11223537	0.14944363
1429708_at	NADH dehydrogenase (ubiquinone) 1 alpha subcomplex 11	Ndufa11	0.71978	1.11511224	0.1474258
1430165_at	serine/threonine kinase 17b (apoptosis-inducing)	Stk17b	0.7198	1.13998677	0.18520694
1427970_at	zinc finger protein 689	Zfp689	0.7198	1.17338165	0.21552434
1434970_a_at	mitochondrial ribosomal protein L15	Mrpl15	0.71983	-1.08827553	-0.1256286
1444316_at	---	---	0.71984	1.15366807	0.19726994
1423850_at	NOL1/NOP2/Sun domain family member 2	Nsun2	0.71985	1.07858609	0.1077863
1455468_at	dpy-19-like 3 (C. elegans)	Dpy19l3	0.71988	-1.13097379	-0.1826609
1433513_x_at	NADH dehydrogenase (ubiquinone) 1 alpha subcomplex, 12	Ndufa12	0.71988	1.09570796	0.12868392
1423849_a_at	CDC-like kinase 3	Clk3	0.71991	1.13291002	0.16853553
1440180_x_at	zinc finger and BTB domain containing 3	Zbtb3	0.71992	1.15580682	0.19628086
1424563_at	solute carrier family 25 (mitochondrial carrier, adenine nucleotide	Slc25a4	0.71992	1.13384528	0.17869135
1458003_at	zinc finger protein 398	Zfp398	0.71993	-1.11879134	-0.1656968
1458209_at	Kinase non-catalytic C-lobe domain (KIND) containing 1	Kndc1	0.71993	-1.12851523	-0.1805447
1428780_at	threonine aldolase 1	Tha1	0.71994	1.1589662	0.20355817
1418543_s_at	coiled-coil domain containing 43	Ccdc43	0.71995	1.08626163	0.11900035
1416784_at	phosphoglycerate kinase 2	Pgk2	0.71995	-1.14746928	-0.2095333
1417757_at	unc-13 homolog B (C. elegans)	Unc13b	0.71996	1.11967738	0.16271049
1434530_at	odd Oz/ten-m homolog 4 (Drosophila)	Odz4	0.71997	-1.11073755	-0.151553
1431110_at	plexin domain containing 2	Plxdc2	0.71998	-1.12204675	-0.1663881
1432361_a_at	centromere protein P	Cenpp	0.71999	-1.08853767	-0.1236047
1440036_x_at	---	---	0.71999	-1.09666359	-0.1335888
1418805_at	secretin	Sct	0.72	1.1282585	0.17252431
1450918_s_at	Rous sarcoma oncogene	Src	0.72001	1.13672955	0.17330378
1428429_at	RGM domain family, member B	Rgmb	0.72002	1.12995092	0.16999079
1434092_at	ATG9 autophagy related 9 homolog B (S. cerevisiae)	Atg9b	0.72002	1.18752778	0.22359334
1450125_at	GATA binding protein 5	Gata5	0.72002	-1.1431545	-0.2031996
1429370_a_at	proteasome (prosome, macropain) 26S subunit, non-ATPase, 11	Psmd11	0.72004	1.10664673	0.14011817
1439279_at	RIKEN cDNA 3110007F17 gene	3110007F17Rik	0.72005	-1.12378511	-0.1791056
1429126_at	RIKEN cDNA 2600001M11 gene /// nudix (nucleoside diphosphat	2600001M11Rik /// Nudt	0.72005	-1.11011853	-0.1510222
1438644_x_at	COMM domain containing 9	Commd9	0.72012	1.15904194	0.19449513
1424615_at	post-GPI attachment to proteins 2	Pgap2	0.72015	1.09549625	0.13107179
1429887_at	nitric oxide synthase 1, neuronal	Nos1	0.72016	-1.1156355	-0.1648038
1431160_x_at	RIKEN cDNA 6030426L16 gene /// similar to development and di	6030426L16Rik /// LOC10	0.72019	1.17137511	0.2104579
1416661_at	eukaryotic translation initiation factor 3, subunit A	Eif3a	0.72021	-1.11193736	-0.1599234
1438182_x_at	Ankyrin repeat and SOCS box-containing 6	Asb6	0.72027	1.10821665	0.14508214
1444038_at	expressed sequence AU015836	AU015836	0.72031	-1.13343702	-0.1919048
1423286_at	cerebellin 1 precursor protein	Cbln1	0.72031	-1.12481938	-0.1759167
1442378_x_at	hypothetical LOC100504701	LOC100504701	0.72035	-1.12938846	-0.1776321
1451828_a_at	acyl-CoA synthetase long-chain family member 4	Acsl4	0.72038	1.19766955	0.23334529
1439778_at	CDK5 and Abl enzyme substrate 1	Cables1	0.72038	-1.13893922	-0.1918614
1433319_at	SH3-binding domain glutamic acid-rich protein	Sh3bgr	0.72061	1.16141464	0.20581073
1454371_at	synaptophysin-like 2	Sypl2	0.72067	-1.13228449	-0.1798495
1444678_at	RIKEN cDNA 2810403D21 gene	2810403D21Rik	0.72067	-1.1039368	-0.1441821
1439211_at	uromodulin-like 1	Umodl1	0.72076	-1.12988641	-0.182726
1419809_s_at	component of oligomeric golgi complex 4	Cog4	0.7208	1.13179371	0.16666458
1442980_at	---	---	0.72081	1.1170324	0.158131
1420720_at	neuronal pentraxin 2	Nptx2	0.72088	1.08773852	0.12108859
1420403_at	ATPase, Ca++ transporting, plasma membrane 2	Atp2b2	0.72088	-1.13529312	-0.191662
1444539_at	RIKEN cDNA D530039A21 gene	D530039A21Rik	0.7209	-1.11834416	-0.161671
1423842_a_at	ring finger protein 41	Rnf41	0.72091	1.11084507	0.1486184

1439826_at	heat shock protein 14	Hspa14	0.72093	1.1433357	0.18279287
1446953_at	---	---	0.72098	1.11891268	0.160474
1422803_at	follistatin-like 3	Fstl3	0.72104	1.11487094	0.15609552
1436106_x_at	coiled-coil domain containing 74A	Ccdc74a	0.72104	-1.11021841	-0.1524433
1420654_a_at	glucan (1,4-alpha-), branching enzyme 1	Gbe1	0.72109	1.16966748	0.21105548
1456120_at	SECIS binding protein 2-like	Secisbp2l	0.72112	-1.17142033	-0.2744362
1448145_at	WW domain containing E3 ubiquitin protein ligase 2	Wwp2	0.72114	1.11020339	0.14741034
1440088_at	---	---	0.72116	1.13632691	0.18010714
1420540_a_at	Ras-like without CAAX 1	Rit1	0.72116	1.11383418	0.15227497
1429175_at	transmembrane protein 178	Tmem178	0.72137	-1.12951723	-0.1798649
1419111_at	inhibitor of growth family, member 2	Ing2	0.72139	1.1053557	0.14321728
1446840_at	---	---	0.72144	-1.12125069	-0.1688866
1434865_a_at	exocyst complex component 7	Exoc7	0.72146	1.12369227	0.16254302
1454279_at	ornithine decarboxylase antizyme 3	Oaz3	0.7215	-1.13565427	-0.1865122
1437575_at	minichromosome maintenance complex component 9	Mcm9	0.72152	1.0751992	0.10251879
1443396_at	survival motor neuron domain containing 1	Smndc1	0.72154	1.11441273	0.15367434
1433528_at	general transcription factor II A, 2	Gtf2a2	0.72157	-1.09587798	-0.1373312
1437561_at	T cell receptor associated transmembrane adaptor 1	Trat1	0.72164	-1.1266358	-0.1735279
1459835_s_at	DnaJ (Hsp40) homolog, subfamily A, member 1	Dnaja1	0.72171	-1.16467523	-0.27378
1452683_at	DnaJ (Hsp40) homolog, subfamily C, member 8	Dnajc8	0.72171	1.08379935	0.11317181
1446437_at	RIKEN cDNA 9630028H03 gene	9630028H03Rik	0.72181	-1.12661398	-0.1738182
1460319_at	fucosyltransferase 8	Fut8	0.72181	1.11911491	0.1560128
1448361_at	tetratricopeptide repeat domain 3	Ttc3	0.72182	1.09399246	0.12915151
1416120_at	ribonucleotide reductase M2	Rrm2	0.72186	1.09149735	0.12322581
1457575_at	expressed sequence AU021128	AU021128	0.72191	1.12901098	0.17209539
1428263_a_at	transcription elongation factor B (SIII), polypeptide 2	Tceb2	0.72197	1.08329788	0.11258357
1454547_at	RIKEN cDNA 2310045N14 gene	2310045N14Rik	0.72205	1.12606741	0.17090082
1419913_at	Serine/threonine kinase receptor associated protein	Strap	0.72206	-1.13730592	-0.1961102
1424937_at	perilipin 5	Plin5	0.72207	1.13304731	0.17757713
1448890_at	Kruppel-like factor 2 (lung)	Klf2	0.72208	1.09968502	0.13215028
1444453_at	transformation related protein 53 regulating kinase	Trp53rk	0.72212	-1.13857812	-0.1991213
1456926_at	regulating synaptic membrane exocytosis 4	Rims4	0.72212	-1.13993639	-0.1989858
1436867_at	sarcalumenin	Srl	0.72212	-1.11430632	-0.1571118
1437837_x_at	polymerase (DNA-directed), delta interacting protein 3	Poldip3	0.72213	-1.08855115	-0.1261276
1434301_at	family with sequence similarity 84, member B	Fam84b	0.72218	-1.13234679	-0.1900247
1433641_at	MAD homolog 5 (Drosophila)	Smad5	0.72218	1.11142308	0.15063379
1454607_s_at	phosphoserine aminotransferase 1	Psat1	0.7222	-1.10894064	-0.1603661
1432151_at	Obg-like ATPase 1	Ola1	0.72225	-1.13855534	-0.1960919
1425548_a_at	leukocyte specific transcript 1	Lst1	0.72227	-1.11836872	-0.1633709
1433274_at	Na+/K+ transporting ATPase interacting 1	Nkain1	0.72229	-1.13090102	-0.1797371
1433895_at	transmembrane protein 127	Tmem127	0.7223	1.11317286	0.15399922
1425436_x_at	killer cell lectin-like receptor, subfamily A, member 3 /// killer cell	Klra3 /// Klra9	0.72231	-1.16518741	-0.2485358
1445021_at	spastic paraplegia 11	Spg11	0.72232	-1.14096761	-0.2016235
1456699_s_at	YTH domain containing 1	Ythdc1	0.72234	-1.0899878	-0.1250142
1427369_at	NLR family, pyrin domain containing 6	Nlrp6	0.72235	1.13332404	0.17747448
1446970_at	---	---	0.72237	1.1785	0.21703616
1433468_at	RIKEN cDNA 6430527G18 gene	6430527G18Rik	0.72238	-1.13518619	-0.2117562
1456773_at	nucleoporin like 2	Nupl2	0.72239	1.12408738	0.16621201
1420939_at	heparan sulfate 6-O-sulfotransferase 2	Hs6st2	0.72241	-1.11888914	-0.1723431
1428661_at	nuclear factor of kappa light polypeptide gene enhancer in B-cells	Nfkbil2	0.72243	1.08842528	0.1220442
1443337_at	---	---	0.72244	-1.13534213	-0.1956298
1444348_at	apolipoprotein B mRNA editing enzyme, catalytic polypeptide 2	Apobec2	0.72247	1.16270876	0.20338366
1428951_at	nucleolar protein 8	Nol8	0.72248	-1.12117579	-0.1829658
1425954_a_at	apurinic/apyrimidinic endonuclease 2	Apex2	0.72248	1.11845017	0.16103383
1435148_at	ATPase, Na+/K+ transporting, beta 2 polypeptide	Atp1b2	0.72249	1.09923936	0.13594996
1419620_at	pituitary tumor-transforming gene 1	Pttg1	0.72251	-1.12490364	-0.1748455
1423375_at	corepressor interacting with RBPJ, 1	Cir1	0.72252	1.144291	0.18375403
1416890_at	WD repeat domain 74	Wdr74	0.72254	1.08578536	0.11780413
1416637_at	solute carrier family 4 (anion exchanger), member 2	Slc4a2	0.72255	1.14713794	0.18757795
1439847_s_at	Kruppel-like factor 12	Klf12	0.72255	-1.14868931	-0.2245474
1418162_at	toll-like receptor 4	Tlr4	0.72256	-1.09926163	-0.1396627
1421905_at	trimethylguanosine synthase homolog (S. cerevisiae)	Tgs1	0.72257	-1.12172719	-0.1755996
1458600_at	---	---	0.72257	-1.1248713	-0.1703485
1448040_at	TCF3 (E2A) fusion partner	Tfpt	0.72262	-1.12457639	-0.1700011
1423971_at	THO complex 3	Thoc3	0.72263	-1.0838095	-0.116127
1419593_at	gene regulated by estrogen in breast cancer protein	Greb1	0.72263	-1.1280368	-0.177538
1453231_at	cyclin-dependent kinase-like 1 (CDC2-related kinase)	Cdkl1	0.72264	1.11623771	0.15320175
1419231_s_at	keratin 12	Krt12	0.72264	-1.12680202	-0.1778285
1424673_at	C-type lectin domain family 2, member h	Clec2h	0.72265	1.11150949	0.14673973
1442277_at	choline kinase alpha	Chka	0.72265	-1.14483163	-0.2055446
1444769_at	Testis expressed gene 9	Tex9	0.72267	-1.15565205	-0.2295492
1440565_at	---	---	0.72268	1.12077587	0.16362766
1417078_at	lectin, galactose-binding, soluble 2	Lgals2	0.72273	1.1193704	0.16143529
1453977_at	exocyst complex component 4	Exoc4	0.72274	1.11430518	0.15608145
1436928_s_at	adenylate cyclase 3	Adcy3	0.72274	1.10904099	0.14890225
1425429_s_at	hypoxia inducible factor 3, alpha subunit	Hif3a	0.72277	1.11732782	0.15885495
1448735_at	ceruloplasmin	Cp	0.72283	1.09761079	0.13069589
1454242_at	RIKEN cDNA 2310079G19 gene	2310079G19Rik	0.72283	-1.15249769	-0.223299
1452684_at	AKT1 substrate 1 (proline-rich)	Akt1s1	0.72284	1.11763549	0.15730901
1417328_at	excision repair cross-complementing rodent repair deficiency, com	Ercc1	0.72284	1.11107756	0.14632285
1429660_s_at	structural maintenance of chromosomes 2	Smc2	0.72284	-1.14440576	-0.2236003
1451815_at	UDP-GlcNAc:betaGal beta-1,3-N-acetylglucosaminyltransferase 4	B3gnt4	0.72285	1.14810566	0.19340413
1448719_at	transcriptional adaptor 3	Tada3	0.72286	1.16453402	0.20555049
1460665_a_at	CCR4-NOT transcription complex, subunit 7	Cnot7	0.72288	-1.11861661	-0.1752306
1452769_at	ring finger protein 145	Rnf145	0.72288	1.09174925	0.12460401
1416573_at	protein O-fucosyltransferase 2	Pofut2	0.72289	1.11986039	0.16177034
1433497_at	aquarius	Aqr	0.72291	-1.0850708	-0.1180361
1432955_at	RIKEN cDNA 4930444K16 gene	4930444K16Rik	0.72291	-1.1353428	-0.1895915
1430814_at	cytochrome P450, family 2, subfamily d, polypeptide 40	Cyp2d40	0.72291	1.14677413	0.1933099
1416517_at	patatin-like phospholipase domain containing 6	Pnpla6	0.72291	1.10541797	0.14093848
1415804_at	chemokine (C-X3-C motif) ligand 1	Cx3cl1	0.72291	-1.11394726	-0.1562785
1448497_at	excision repair cross-complementing rodent repair deficiency, com	Ercc3	0.72292	1.08661714	0.11900792
1429453_a_at	mitochondrial ribosomal protein L55	Mrpl55	0.72292	1.11494356	0.15166813
1448135_at	activating transcription factor 4	Atf4	0.72293	-1.07417449	-0.104389

1439010_at	La ribonucleoprotein domain family, member 4	Larp4	0.72293	-1.08359127	-0.1166991
1419287_at	transmembrane protein 208	Tmem208	0.72293	1.11088379	0.14554784
1434404_at	family with sequence similarity 73, member A	Fam73a	0.72294	-1.10781018	-0.149945
1416424_at	perilipin 3	Plin3	0.72295	1.11140283	0.14691669
1459615_at	RIKEN cDNA B130052P14 gene	B130052P14Rik	0.72295	-1.1297424	-0.1819214
1424647_at	gamma-aminobutyric acid (GABA) A receptor, pi	Gabrp	0.72296	-1.13006025	-0.1775283
1442966_at	---	---	0.72296	-1.13092017	-0.1821897
1417016_at	MAP kinase-activated protein kinase 5	Mapkapk5	0.72296	1.09636322	0.12987362
1439294_at	predicted gene 5112	Gm5112	0.72296	-1.13762871	-0.1909577
1418180_at	trans-acting transcription factor 1	Sp1	0.72296	-1.10018328	-0.1463992
1439915_at	---	---	0.72298	-1.12078857	-0.1732731
1455679_at	oligonucleotide/oligosaccharide-binding fold containing 2A	Obfc2a	0.72298	-1.12441053	-0.1728055
1448418_s_at	DDB1 and CUL4 associated factor 11	Dcaf11	0.72299	1.09263287	0.12533847
1429395_at	glutathione S-transferase, C-terminal domain containing	Gstcd	0.723	1.10541321	0.14308166
1416394_at	BCL2-associated athanogene 1	Bag1	0.72302	1.0852624	0.11663672
1445240_at	expressed sequence AU019796	AU019796	0.72302	-1.13361946	-0.1818872
1433511_at	general transcription factor II A, 1	Gtf2a1	0.72303	-1.12693425	-0.1760263
1427102_at	schlafen 4	Slfn4	0.72303	-1.11417118	-0.1567993
1429405_at	RIKEN cDNA 2010317E24 gene	2010317E24Rik	0.72305	1.14329554	0.18682655
1425625_at	interleukin 13 receptor, alpha 1	Il13ra1	0.72306	1.17511159	0.21676221
1429635_at	coiled-coil domain containing 89	Ccdc89	0.72306	-1.1473468	-0.2121377
1446237_at	A kinase (PRKA) anchor protein (yotiao) 9	Akap9	0.72306	-1.13002841	-0.1776814
1450631_x_at	defensin, alpha, 24	Defa24	0.72306	1.12545445	0.16922757
1452920_a_at	peptidylprolyl isomerase (cyclophilin)-like 2	Ppil2	0.72308	1.08708603	0.11960211
1432712_at	RIKEN cDNA 4933425M03 gene	4933425M03Rik	0.72308	-1.1292931	-0.1774271
1452186_at	RNA binding motif protein 5	Rbm5	0.7231	1.09030837	0.12409933
1446619_at	RIKEN cDNA A130038J17 gene	A130038J17Rik	0.72311	1.10758169	0.14520795
1432050_at	RIKEN cDNA 4930571B16 gene	4930571B16Rik	0.72312	-1.13461703	-0.1911837
1420474_at	myotrophin	Mtpn	0.72314	-1.09827032	-0.1425292
1445869_at	---	---	0.72314	-1.1263163	-0.1726068
1443883_at	SYS1 Golgi-localized integral membrane protein homolog (S. cere	Sys1	0.72314	-1.13326747	-0.1837882
1449937_at	endonuclease, polyU-specific	Endou	0.72316	-1.12577196	-0.1712355
1442489_at	DNA segment, Chr 1, ERATO Doi 564, expressed	D1Ertd564e	0.72318	-1.12537861	-0.1732864
1419182_at	sushi, von Willebrand factor type A, EGF and pentraxin domain co	Svep1	0.72321	-1.14482429	-0.208579
1429563_x_at	Sp110 nuclear body protein	Sp110	0.72321	-1.13521638	-0.184513
1458208_s_at	methylcrotonoyl-Coenzyme A carboxylase 1 (alpha)	Mccc1	0.72323	1.13150646	0.17593189
1441527_at	RIKEN cDNA 4930558J18 gene	4930558J18Rik	0.72326	1.13665341	0.18106682
1427826_a_at	solute carrier organic anion transporter family, member 1b2	Slco1b2	0.72327	1.11308351	0.14884695
1432064_at	U box domain containing 5	Ubox5	0.72328	-1.13106984	-0.1797622
1443865_at	gamma-aminobutyric acid (GABA) A receptor, subunit alpha 2	Gabra2	0.72329	-1.13002035	-0.1822172
1447640_s_at	pre B-cell leukemia transcription factor 3	Pbx3	0.7233	1.13626164	0.17393874
1459066_at	---	---	0.72335	1.12096801	0.16440828
1427433_s_at	homeobox A3	Hoxa3	0.72335	1.16731034	0.21130815
1431375_s_at	parvin, alpha	Parva	0.72335	1.15184687	0.19878198
1420409_at	keratin 35	Krt35	0.72336	1.14669427	0.19009053
1454225_s_at	DNA segment, Chr 3, ERATO Doi 751, expressed	D3Ertd751e	0.72339	-1.16394156	-0.2519495
1420430_a_at	coiled-coil domain containing 159	Ccdc159	0.72339	1.14317032	0.18754404
1454689_at	serine/arginine repetitive matrix 1	Srrm1	0.72339	-1.09761009	-0.13887
1445432_at	---	---	0.72341	1.22730064	0.2616468
1445948_at	---	---	0.72345	-1.12144168	-0.1722262
1451944_a_at	tumor necrosis factor (ligand) superfamily, member 11	Tnfsf11	0.72355	1.17828041	0.21863093
1443864_at	denticleless homolog (Drosophila)	Dtl	0.7239	1.11541513	0.1542612
1458057_at	hypothetical protein LOC100503921	LOC100503921	0.72393	1.14464484	0.18653367
1436905_x_at	lysosomal-associated protein transmembrane 5	Laptm5	0.72415	-1.11503465	-0.1731137
1451477_at	predicted gene 13139	Gm13139	0.72426	-1.08964254	-0.1253285
1422994_at	phosphoinositide kinase, FYVE finger containing	Pikfyve	0.72428	-1.14274177	-0.2116157
1448871_at	mitogen-activated protein kinase 13	Mapk13	0.72428	1.11864868	0.15639491
1422422_at	defensin, alpha, 4	Defa4	0.72429	-1.12664518	-0.1755531
1450342_at	bone morphogenetic protein 8b	Bmp8b	0.72435	-1.12444713	-0.1703558
1458911_at	sperm flagellar 2	Spef2	0.72444	-1.12221273	-0.166435
1456493_at	Sp110 nuclear body protein	Sp110	0.72444	-1.13088081	-0.1798808
1460736_at	biorientation of chromosomes in cell division 1-like	Bod1l	0.72445	-1.10447259	-0.1477323
1423634_at	gasdermin A	Gsdma	0.72446	1.17393709	0.21362333
1441522_at	DCN1, defective in cullin neddylation 1, domain containing 2 (S. ce	Dcun1d2	0.72448	-1.14426768	-0.2075473
1425107_a_at	leukemia inhibitory factor receptor	Lifr	0.72453	1.14934237	0.18882148
1450647_at	Hermansky-Pudlak syndrome 3 homolog (human)	Hps3	0.72454	1.12815135	0.16773805
1424440_at	mitochondrial ribosomal protein S6	Mrps6	0.72455	1.09671933	0.13249753
1425179_at	serine hydroxymethyltransferase 1 (soluble)	Shmt1	0.72458	1.09673305	0.13003478
1457585_at	C2 calcium-dependent domain containing 2-like	C2cd2l	0.72458	-1.14229198	-0.1996811
1441006_at	dihydrouridine synthase 4-like (S. cerevisiae)	Dus4l	0.7246	1.15434654	0.19773472
1438147_at	scavenger receptor cysteine rich domain containing, group B (4 do	Srcrb4d	0.72461	1.13683661	0.17303796
1417701_at	protein phosphatase 1, regulatory (inhibitor) subunit 14c	Ppp1r14c	0.72462	-1.13004405	-0.1810458
1458029_at	---	---	0.72465	1.12967157	0.17438166
1438097_at	RAB20, member RAS oncogene family	Rab20	0.72466	1.12734623	0.16990415
1452379_at	autism susceptibility candidate 2	Auts2	0.72466	1.16256812	0.20297761
1431328_at	protein phosphatase 1, catalytic subunit, beta isoform	Ppp1cb	0.72467	-1.10711785	-0.1509156
1419989_at	COMM domain containing 5	Commd5	0.72467	-1.1304194	-0.1807825
1433282_at	---	---	0.7247	-1.10468461	-0.1457177
1443677_at	RIKEN cDNA E030011O05 gene	E030011O05Rik	0.72473	-1.13064658	-0.1772735
1450161_at	inhibitor of kappaB kinase gamma	Ikbkg	0.72473	1.13646337	0.18070895
1446786_at	---	---	0.72475	-1.12986995	-0.1779243
1457614_at	MICAL-like 2	Micall2	0.72477	-1.14420994	-0.215524
1416540_at	HGF-regulated tyrosine kinase substrate	Hgs	0.72477	1.09321052	0.1262954
1435760_at	cystatin A	Csta	0.72482	1.12881832	0.16696599
1447534_at	---	---	0.72483	-1.1326298	-0.1874851
1459090_at	---	---	0.72483	-1.12990078	-0.1772743
1455367_at	dead end homolog 1 (zebrafish)	Dnd1	0.72484	1.11609774	0.15235726
1460155_at	family with sequence similarity 82, member B	Fam82b	0.72484	-1.12689938	-0.1801573
1439608_at	---	---	0.72485	-1.12761864	-0.1748664
1450079_at	Nik related kinase	Nrk	0.72489	-1.18671853	-0.3327971
1434860_at	dpy-19-like 4 (C. elegans)	Dpy19l4	0.72489	-1.10560316	-0.1458788
1419038_a_at	casein kinase 2, alpha 1 polypeptide	Csnk2a1	0.7249	-1.15273879	-0.2555045
1430219_at	thymoma viral proto-oncogene 1 interacting protein	Aktip	0.7249	1.09256333	0.12722055
1427321_s_at	coxsackie virus and adenovirus receptor	Cxadr	0.72491	-1.14585531	-0.2208869

1437558_at	RIKEN cDNA B130021B11 gene	B130021B11Rik	0.72492	-1.1338696	-0.1814761
1418811_at	BarH-like 1 (Drosophila)	Barhl1	0.72492	1.14248927	0.18471308
1421330_at	protein tyrosine phosphatase, non-receptor type 4	Ptpn4	0.72492	-1.11966155	-0.1655819
1451264_at	FERM domain containing 6	Frmd6	0.72494	1.13141015	0.16945799
1428560_at	exportin 5	Xpo5	0.72494	1.08231291	0.11406141
1430781_at	adenylate kinase 7	Ak7	0.72495	1.10955537	0.1468766
1431340_a_at	non-protein coding RNA 81	Ncrna00081	0.72495	1.157252	0.19855399
1453606_at	RIKEN cDNA 4931403G20 gene	4931403G20Rik	0.72495	1.13424891	0.17891664
1453432_at	RIKEN cDNA 1700007G11 gene	1700007G11Rik	0.72497	1.13871534	0.18460011
1433907_at	Pbx/knotted 1 homeobox 2	Pknox2	0.72497	-1.14890254	-0.2183469
1443001_at	expressed sequence C78344	C78344	0.72497	-1.11204445	-0.163809
1436830_at	MARVEL (membrane-associating) domain containing 1	Marveld1	0.72503	-1.12561072	-0.1735516
1445195_at	expressed sequence C77631	C77631	0.72506	1.13187084	0.16792431
1452391_at	coxsackie virus and adenovirus receptor	Cxadr	0.72506	-1.13139549	-0.1797269
1440672_at	zinc finger protein 541	Zfp541	0.72507	-1.14293947	-0.2041284
1458685_at	Ral GTPase activating protein, alpha subunit 1	Ralgapa1	0.72507	-1.12777658	-0.1831439
1432529_at	bol, boule-like (Drosophila)	Boll	0.72507	1.16728089	0.20702817
1423956_at	stromal membrane-associated protein 1	Smap1	0.72507	-1.10418921	-0.1479065
1440522_at	cysteine-rich hydrophobic domain 1	Chic1	0.72509	-1.11832295	-0.1630483
1460042_at	solute carrier family 23 (nucleobase transporters), member 3	Slc23a3	0.72511	-1.13926128	-0.2023733
1458088_at	---	---	0.72511	-1.12750565	-0.1736256
1416466_at	vesicle-associated membrane protein, associated protein A	Vapa	0.72511	-1.08013509	-0.1141669
1427148_at	praja 2, RING-H2 motif containing	Pja2	0.72512	1.13719849	0.18107149
1458586_at	---	---	0.72514	-1.12997576	-0.1816432
1445763_at	RIKEN cDNA 1700013F07 gene	1700013F07Rik	0.72514	1.16413238	0.20366092
1450510_a_at	calcium channel, voltage-dependent, P/Q type, alpha 1A subunit	Cacna1a	0.72516	1.10819295	0.14700574
1437118_at	ubiquitin specific peptidase 7	Usp7	0.72517	-1.13238245	-0.2007718
1456837_at	aspartate beta-hydroxylase domain containing 1	Asphd1	0.72521	1.11752431	0.15911569
1453004_at	solute carrier family 22, member 23	Slc22a23	0.72524	1.17165794	0.21336737
1445585_at	ring finger protein 114	Rnf114	0.72537	1.15402344	0.19456027
AFFX-b-ActinMur	actin, beta	Actb	0.72584	-1.04835474	-0.069651
1435794_at	RIKEN cDNA 5031425E22 gene	5031425E22Rik	0.72626	1.12627174	0.17062755
1417761_at	apolipoprotein A-IV	Apoa4	0.72663	-1.14867216	-0.2182727
1460243_at	serine palmitoyltransferase, long chain base subunit 2	Sptlc2	0.72668	1.10162579	0.13770372
1437079_at	solute carrier family 18 (vesicular monoamine), member 2	Slc18a2	0.7267	-1.12262022	-0.177447
1435425_at	phosphatidylserine decarboxylase /// phosphatidylserine decarbo	Pisd /// Pisd-ps3	0.72673	-1.12170782	-0.1706267
1418349_at	heparin-binding EGF-like growth factor	Hbegf	0.72674	1.15059267	0.18815384
1447650_at	NMD3 homolog (S. cerevisiae)	Nmd3	0.72675	1.1429903	0.18646578
1442215_at	smoothened homolog (Drosophila)	Smo	0.72683	1.12319893	0.16423901
1449148_a_at	putative homeodomain transcription factor 1	Phtf1	0.72685	1.10407898	0.14098654
1443278_at	---	---	0.72685	1.12952932	0.16802947
1437295_at	protein kinase N2	Pkn2	0.72688	-1.11460664	-0.1687866
1459705_at	---	---	0.72691	1.1384604	0.17782553
1442662_at	transmembrane protein 167	Tmem167	0.72691	-1.16311753	-0.2488157
1447881_x_at	ubiquitin specific peptidase 15	Usp15	0.72693	-1.11451504	-0.1568582
1441860_x_at	insulin degrading enzyme	Ide	0.72694	-1.15383631	-0.2426931
1453853_a_at	Rho guanine nucleotide exchange factor (GEF) 12	Arhgef12	0.72695	1.24461561	0.27228445
1444993_at	predicted gene 7420	Gm7420	0.72695	-1.0986885	-0.1397811
1420480_at	cyclin M3	Cnnm3	0.72697	1.109751	0.14908651
1426258_at	sortilin-related receptor, LDLR class A repeats-containing	Sorl1	0.72704	1.1147979	0.14919586
1416074_a_at	ribosomal protein L28	Rpl28	0.72705	1.04667907	0.06475976
1422099_a_at	opioid receptor-like 1	Oprl1	0.72706	-1.12494059	-0.1739582
1433720_s_at	coiled-coil-helix-coiled-coil-helix domain containing 10	Chchd10	0.7271	1.05958721	0.08276716
1451852_at	2-cell-stage, variable group, member 3	Tcstv3	0.72713	-1.11119827	-0.1555767
1457329_at	---	---	0.72715	1.13550656	0.17970271
1445563_at	eukaryotic translation initiation factor 2a	Eif2a	0.72715	-1.10542697	-0.1482658
1418702_a_at	RIKEN cDNA 2810428I15 gene	2810428I15Rik	0.72721	1.12610439	0.15853267
1456175_a_at	coatomer protein complex, subunit beta 2 (beta prime)	Copb2	0.72723	-1.10058595	-0.1436379
1423931_s_at	anaphase promoting complex subunit 4	Anapc4	0.72723	1.08684188	0.1194961
1428894_at	RIKEN cDNA 1300018J18 gene	1300018J18Rik	0.72724	-1.1219957	-0.1699228
1441161_at	RIKEN cDNA B230216G23 gene	B230216G23Rik	0.72725	1.13635383	0.17936261
1425488_at	SLU7 splicing factor homolog (S. cerevisiae)	Slu7	0.7273	-1.11352804	-0.1613556
1434005_at	RNA binding motif, single stranded interacting protein 1	Rbms1	0.72738	-1.09433667	-0.1342055
1427329_a_at	immunoglobulin heavy chain 6 (heavy chain of IgM)	Igh-6	0.72738	-1.1342345	-0.1850914
1431725_at	formin 2	Fmn2	0.72742	1.14135899	0.18259789
1445534_at	Filamin, beta	Flnb	0.72744	-1.14699688	-0.2185933
1424499_s_at	atlastin GTPase 3	Atl3	0.7275	1.119696	0.16236329
1453189_at	ubiquitin-conjugating enzyme E2I	Ube2i	0.7275	-1.14177095	-0.2192535
1428561_at	RIKEN cDNA 2610002J23 gene	2610002J23Rik	0.72753	-1.09725097	-0.1347118
1419110_at	RIO kinase 1 (yeast)	Riok1	0.72754	-1.09603097	-0.1349343
1427337_at	aldehyde dehydrogenase 8 family, member A1	Aldh8a1	0.72765	1.13264857	0.17536382
1437096_at	tetratricopeptide repeat domain 29	Ttc29	0.72767	-1.14740276	-0.2158704
1455822_x_at	surfeit gene 4	Surf4	0.72782	-1.14277119	-0.2153844
1428652_at	RIKEN cDNA 0610010F05 gene	0610010F05Rik	0.72806	-1.09915312	-0.1401994
1436392_s_at	transcription factor AP-2, gamma	Tcfap2c	0.72806	1.10439201	0.14025676
1429031_at	RIKEN cDNA 4833408D11 gene /// hypothetical LOC100503722	4833408D11Rik /// LOC10	0.7281	-1.10427216	-0.1462003
1457341_at	---	---	0.72812	-1.13081655	-0.1816445
1442907_at	---	---	0.72813	1.13471888	0.17873176
1451176_at	mitochondrial antiviral signaling protein	Mavs	0.72815	1.09973179	0.13523295
1444472_at	---	---	0.72817	-1.13280063	-0.1868707
1435653_at	abhydrolase domain containing 2	Abhd2	0.72819	-1.1161905	-0.1733465
1429123_at	RAB27A, member RAS oncogene family	Rab27a	0.72823	-1.10924371	-0.1509983
1430236_s_at	gasdermin A2	Gsdma2	0.72823	-1.13877858	-0.1991744
1423532_at	ring finger protein 44	Rnf44	0.72824	-1.11229294	-0.1702499
1417250_at	ring finger protein, LIM domain interacting	Rlim	0.72833	1.08269967	0.11402789
1456754_at	zinc finger, FYVE domain containing 20	Zfyve20	0.72839	-1.10337081	-0.144903
1448959_at	NADH dehydrogenase (ubiquinone) Fe-S protein 4	Ndufs4	0.7284	1.08564605	0.11780371
1418182_at	protein tyrosine phosphatase 4a3	Ptp4a3	0.72842	1.18111662	0.22045401
1438925_x_at	ATPase, H+ transporting, lysosomal V0 subunit C	Atp6v0c	0.72842	1.07920416	0.10965397
1450098_at	dihydrodipicolinate synthase-like, mitochondrial	Dhdpsl	0.72843	-1.13086497	-0.1842971
1449150_at	KRAB-A domain containing 1	Krba1	0.72845	1.11008182	0.14899325
1450362_at	---	---	0.72846	-1.08863713	-0.12451
1448115_at	TRM2 tRNA methyltransferase 2 homolog A (S. cerevisiae)	Trmt2a	0.72849	1.08554421	0.1176809
1425534_at	staufen (RNA binding protein) homolog 2 (Drosophila)	Stau2	0.72849	-1.13846338	-0.1933513

1426160_a_at	serine/threonine kinase 16	Stk16	0.7285	1.11304143	0.15022081
1422432_at	diazepam binding inhibitor	Dbi	0.72852	1.13276916	0.16546536
1421532_at	relaxin/insulin-like family peptide receptor 2	Rxfp2	0.72853	-1.12445031	-0.1703314
1445441_at	predicted gene 5142	Gm5142	0.72855	-1.12426663	-0.1700617
1442542_at	eyes absent 4 homolog (Drosophila)	Eya4	0.72855	1.13124327	0.17071885
1433571_at	serine incorporator 5	Serinc5	0.72856	1.14187062	0.18338339
1459080_at	---	---	0.72856	1.12184483	0.16395407
1448508_at	TRAF3 interacting protein 2	Traf3ip2	0.72859	1.10698022	0.14343376
1447499_s_at	oocyte maturation, alpha /// oocyte maturation, beta	Omt2a /// Omt2b	0.72859	-1.12896459	-0.1817644
1449547_at	ankyrin repeat and SOCS box-containing 14	Asb14	0.72863	1.11622262	0.15755061
1422786_at	solute carrier family 30 (zinc transporter), member 1	Slc30a1	0.72864	1.11480841	0.15659819
1436417_at	solute carrier family 19 (sodium/hydrogen exchanger), member 3	Slc19a3	0.72864	-1.13352643	-0.1860297
1427871_at	platelet-activating factor receptor	Ptafr	0.72866	1.11669447	0.15788511
1456054_a_at	pumilio 1 (Drosophila)	Pum1	0.72868	-1.14214945	-0.2271672
1441890_x_at	transmembrane protein with EGF-like and two follistatin-like dom	Tmeff1	0.72868	-1.16570017	-0.263901
1444156_at	RIKEN cDNA 9230112E08 gene	9230112E08Rik	0.72869	1.12461898	0.16617728
1456691_s_at	steroid 5 alpha-reductase 3	Srd5a3	0.7287	1.11930684	0.16090052
1441571_at	RIKEN cDNA 2410089E03 gene	2410089E03Rik	0.72871	-1.14059528	-0.1978548
1456214_at	protocadherin 7	Pcdh7	0.72872	1.11102244	0.14777618
1433341_at	RIKEN cDNA 5730416F02 gene	5730416F02Rik	0.72873	-1.12701603	-0.1780661
1422361_at	---	---	0.72876	-1.13711767	-0.1951489
1447309_at	---	---	0.72876	-1.12106912	-0.166881
1460699_at	ribosomal protein S27	Rps27	0.72878	1.11133561	0.15219571
1419271_at	paired box gene 6	Pax6	0.72878	1.13836724	0.18266407
1452515_a_at	xylosyltransferase II	Xylt2	0.7288	1.13017051	0.16861556
1429471_at	RIKEN cDNA 1110017D15 gene	1110017D15Rik	0.72881	-1.14874959	-0.2182636
1423298_at	adducin 3 (gamma)	Add3	0.72884	1.10148492	0.13930505
1458111_at	family with sequence similarity 20, member B	Fam20b	0.72885	1.14096075	0.1852863
1450214_at	adenosine A2b receptor	Adora2b	0.72887	1.14074758	0.18387612
1441917_s_at	transmembrane protein 40	Tmem40	0.72887	1.13587839	0.17369963
1418191_at	ubiquitin specific peptidase 18	Usp18	0.72887	1.1658435	0.20593448
AFFX-LysX-3_at	---	---	0.72889	1.15708433	0.19545358
1431929_a_at	syntaxin 17	Stx17	0.7289	-1.1248688	-0.1717539
1454416_at	RIKEN cDNA 5730575I04 gene	5730575I04Rik	0.72892	1.13103053	0.17478503
1436447_at	RIKEN cDNA A630026N12 gene	A630026N12Rik	0.72893	-1.14203312	-0.2040015
1460403_at	PC4 and SFRS1 interacting protein 1	Psip1	0.72895	-1.08728716	-0.1219098
1416282_at	proteasome (prosome, macropain) 26S subunit, ATPase 3	Psmc3	0.72896	1.07731924	0.10731251
1446008_at	DNA segment, Chr 3, ERATO Doi 246, expressed	D3Ertd246e	0.72897	-1.09375222	-0.1308186
1437877_at	tetratricopeptide repeat domain 14	Ttc14	0.72898	-1.1705189	-0.271987
1439180_at	INO80 complex subunit D	Ino80d	0.72901	-1.16870151	-0.2716016
1452557_a_at	---	---	0.72901	-1.13220648	-0.1801136
1419903_at	dysbindin (dystrobrevin binding protein 1) domain containing 2	Dbndd2	0.72901	1.1469438	0.19119376
1448242_at	Sec61 alpha 1 subunit (S. cerevisiae)	Sec61a1	0.72902	1.09184866	0.1240135
1425729_at	bestrophin 2	Best2	0.72903	1.12209473	0.16413495
1424334_at	tetraspanin 17	Tspan17	0.72906	1.10609508	0.14428032
1416118_at	tripartite motif-containing 59	Trim59	0.72908	-1.09015001	-0.1271977
1427412_s_at	Rap guanine nucleotide exchange factor (GEF) 6	Rapgef6	0.7291	1.14999366	0.19006086
1440364_a_at	aminoadipate-semialdehyde dehydrogenase	Aasdh	0.72912	1.11546503	0.15640314
1438222_at	DNA segment, Chr 2, ERATO Doi 612, expressed	D2Ertd612e	0.72914	-1.13227304	-0.1984934
1439461_x_at	non-SMC element 4 homolog A (S. cerevisiae)	Nsmce4a	0.72915	-1.10489779	-0.1455949
1424223_at	RIKEN cDNA 1700020C11 gene	1700020C11Rik	0.72915	1.11106905	0.15051053
1420034_at	Protein phosphatase 2, regulatory subunit B, delta isoform	Ppp2r2d	0.72917	-1.13908081	-0.2070667
1455064_at	RAB36, member RAS oncogene family	Rab36	0.72919	-1.13500454	-0.1866008
1423197_a_at	SMEK homolog 2, suppressor of mek1 (Dictyostelium)	Smek2	0.72919	1.09377939	0.12915626
1418429_at	kinesin family member 5B	Kif5b	0.72925	-1.09718784	-0.1372603
1420804_s_at	C-type lectin domain family 4, member d	Clec4d	0.72926	1.17081764	0.2131191
1452987_at	TATA box binding protein (Tbp)-associated factor, RNA polymeras	Taf1d	0.72929	-1.13136697	-0.1817803
1431843_a_at	nuclear factor of kappa light polypeptide gene enhancer in B-cells	Nfkbie	0.72933	1.13242303	0.17717183
1424259_at	lipase maturation factor 1	Lmf1	0.72941	1.16624093	0.20145487
1426627_at	mitogen-activated protein kinase kinase kinase 7	Map3k7	0.72962	-1.13077506	-0.190171
1436177_at	pleckstrin homology domain-containing, family A (phosphoinositi	Plekha2	0.72964	1.13632883	0.18031625
1436484_at	RIKEN cDNA C030019I05 gene	C030019I05Rik	0.72966	1.16497175	0.20579854
1449645_s_at	chaperonin containing Tcp1, subunit 3 (gamma)	Cct3	0.72968	1.0838128	0.1146072
1443667_at	expressed sequence C79407	C79407	0.72968	-1.11929139	-0.1663466
1438029_at	regulation of nuclear pre-mRNA domain containing 2	Rprd2	0.72975	-1.10061673	-0.1394835
1426845_at	programmed cell death 2-like	Pdcd2l	0.72979	1.08393439	0.11564946
1417203_at	ethylmalonic encephalopathy 1	Ethe1	0.7298	1.11495992	0.15198673
1446905_at	---	---	0.72986	-1.13235852	-0.1905264
1451892_at	klotho	Kl	0.72993	-1.12389315	-0.1715416
1435129_at	---	---	0.73031	-1.08935278	-0.1287663
1425167_a_at	guanine nucleotide binding protein (G protein), gamma transducin	Gngt1	0.73039	1.14326354	0.18534617
1439672_at	---	---	0.73043	-1.13566009	-0.188619
1423141_at	lysosomal acid lipase A	Lipa	0.73044	-1.1434494	-0.2052147
1438858_x_at	histocompatibility 2, class II antigen A, alpha	H2-Aa	0.73046	-1.1416947	-0.2010638
1446734_at	expressed sequence C77438	C77438	0.73047	-1.11658121	-0.1592404
1440539_at	---	---	0.73048	1.11159822	0.15159391
1436270_at	cytochrome b5 domain containing 1	Cyb5d1	0.73052	1.11437653	0.15528892
1446486_at	DTW domain containing 2	Dtwd2	0.73053	1.12477749	0.16608779
1452142_at	solute carrier family 6 (neurotransmitter transporter, GABA), mem	Slc6a1	0.73054	-1.10386405	-0.142679
1448853_at	synaptojanin 2 binding protein	Synj2bp	0.73054	1.1142784	0.1479457
1416545_at	zinc finger, DHHC domain containing 7	Zdhhc7	0.73055	1.100592	0.1365595
1415784_at	vacuolar protein sorting 35	Vps35	0.73055	-1.13417693	-0.215354
1426675_at	translocase of outer mitochondrial membrane 70 homolog A (yea	Tomm70a	0.73058	-1.09538746	-0.1377773
1439442_x_at	tyrosyl-tRNA synthetase 2 (mitochondrial)	Yars2	0.73058	-1.1360605	-0.2153642
1416003_at	claudin 11	Cldn11	0.73058	1.10840117	0.14840433
1427809_at	---	---	0.73062	-1.12963686	-0.1774028
1451776_s_at	HOP homeobox	Hopx	0.73068	-1.11972357	-0.1677235
1423391_at	G protein-coupled receptor kinase-interactor 2	Git2	0.73068	1.09690167	0.1317906
1428836_at	RIKEN cDNA 2300009A05 gene	2300009A05Rik	0.73069	1.13449963	0.17471011
1432796_at	RIKEN cDNA 3110067C02 gene	3110067C02Rik	0.7307	-1.11236271	-0.1616752
1424169_at	Tax1 (human T-cell leukemia virus type I) binding protein 3	Tax1bp3	0.7307	1.1208655	0.15758429
1436523_s_at	RIKEN cDNA 1810022K09 gene	1810022K09Rik	0.7307	-1.08553254	-0.1220371
1455091_at	protein phosphatase 2, regulatory subunit B'', alpha	Ppp2r3a	0.7307	-1.1319736	-0.182186
1433915_s_at	epsin 2	Epn2	0.73072	1.09825506	0.13466257

1448698_at	cyclin D1	Ccnd1	0.73073	1.09056725	0.12475152
1419293_at	Down syndrome cell adhesion molecule	Dscam	0.73074	-1.12243117	-0.1679533
1450532_at	RIKEN cDNA 2310002J15 gene	2310002J15Rik	0.73077	1.12873278	0.17179751
1456367_at	fucosyltransferase 8	Fut8	0.73079	1.14563221	0.18502639
1418230_a_at	LIM and senescent cell antigen-like domains 1	Lims1	0.73083	-1.16727349	-0.2745873
1455433_at	zinc finger pseudogene	3110048L19Rik	0.73084	-1.13003857	-0.1838804
1431697_at	synaptojanin 2	Synj2	0.73084	1.13173258	0.17628541
1457805_at	---	---	0.73087	1.12906108	0.16643843
1443502_at	baculoviral IAP repeat-containing 7 (livin)	Birc7	0.7309	1.13237194	0.17598624
1452153_at	F-box protein 18	Fbxo18	0.73091	1.09841441	0.13374115
1451266_at	mitochondrial ribosomal protein L50	Mrpl50	0.73092	-1.09443116	-0.1334014
1452158_at	glutamyl-prolyl-tRNA synthetase	Eprs	0.73099	-1.14025053	-0.2315556
1444958_at	expressed sequence C79017	C79017	0.73104	-1.13353809	-0.1899258
1436677_at	RIKEN cDNA 1810032O08 gene	1810032O08Rik	0.73106	-1.10930668	-0.1518096
1429859_a_at	ADP-ribosylation factor-like 2 binding protein	Arl2bp	0.73107	1.10287994	0.13981621
1460361_at	RIKEN cDNA 5033414D02 gene	5033414D02Rik	0.73111	1.09773005	0.13198409
1428868_a_at	ornithine decarboxylase antizyme 1 pseudogene /// ornithine dec	Gm9786 /// Oaz1	0.73118	1.06817136	0.09505385
1432130_a_at	tetratricopeptide repeat domain 14	Ttc14	0.73126	-1.11028041	-0.156762
1423254_x_at	ribosomal protein S27-like	Rps27l	0.73126	1.07176793	0.09678167
1452953_at	family with sequence similarity 18, member B	Fam18b	0.73126	-1.09266116	-0.130178
1417073_a_at	quaking	Qk	0.73127	-1.09399723	-0.1310013
1418213_at	keratin 23	Krt23	0.73127	1.13529337	0.18013699
1443777_at	---	---	0.73128	1.15021391	0.19141547
1420397_a_at	SPEN homolog, transcriptional regulator (Drosophila)	Spen	0.73135	-1.11088439	-0.1605384
1451313_a_at	RIKEN cDNA 1110067D22 gene	1110067D22Rik	0.73135	-1.12550831	-0.185833
1432350_at	syncoilin	Sync	0.73137	-1.12739157	-0.1767403
1418676_at	insulin related protein 2 (islet 2)	Isl2	0.7314	-1.13081238	-0.1806971
1421038_a_at	potassium intermediate/small conductance calcium-activated cha	Kcnn4	0.7314	1.15846273	0.19857325
1422607_at	ets variant gene 1	Etv1	0.73145	-1.09229999	-0.1300489
1440890_a_at	zinc finger protein 809	Zfp809	0.73153	-1.10437275	-0.1484409
1433481_at	FK506 binding protein 14	Fkbp14	0.73155	1.11147687	0.15209389
1438670_at	protein tyrosine phosphatase, non-receptor type 1	Ptpn1	0.73156	1.13587808	0.17277911
1424912_at	solute carrier family 25 (mitochondrial carrier, peroxisomal memb	Slc25a17	0.73156	1.1051263	0.14252817
1422901_at	meningioma expressed antigen 5 (hyaluronidase)	Mgea5	0.73158	1.1088714	0.14215266
1455110_at	GA repeat binding protein, beta 2	Gabpb2	0.73159	-1.09191924	-0.1294494
1448272_at	B-cell translocation gene 2, anti-proliferative	Btg2	0.73163	1.11014062	0.1455259
1454572_at	RIKEN cDNA 2810414N06 gene	2810414N06Rik	0.73164	-1.12692849	-0.1751239
1457968_at	---	---	0.73164	-1.13463623	-0.1859625
1448367_at	stromal cell derived factor 4	Sdf4	0.73165	1.14902703	0.19210527
1415684_at	autophagy-related 5 (yeast)	Atg5	0.73168	1.09105616	0.12446332
1420431_at	repetin	Rptn	0.73168	1.11172112	0.15162003
1420909_at	vascular endothelial growth factor A	Vegfa	0.73169	-1.10324946	-0.1446594
1442199_at	butyrophilin-like 2	Btnl2	0.73193	-1.12849194	-0.1776826
1434981_at	RIKEN cDNA E130303B06 gene	E130303B06Rik	0.73204	1.11280296	0.15199027
1438625_s_at	cyclin-dependent kinase 16	Cdk16	0.73212	-1.09037833	-0.1304647
1433476_at	expressed sequence C78339	C78339	0.73218	1.10217045	0.13726462
1447387_at	---	---	0.73221	-1.11335111	-0.1573961
1450373_at	---	---	0.73222	1.14465335	0.18586762
1435045_s_at	mitogen-activated protein kinase 8 interacting protein 2	Mapk8ip2	0.73224	-1.12573737	-0.171905
1449219_at	fatty acid desaturase 3	Fads3	0.73226	1.16290895	0.20414231
1420559_a_at	short stature homeobox 2	Shox2	0.73229	1.12065864	0.1617151
1430999_a_at	short coiled-coil protein	Scoc	0.73229	-1.10625333	-0.1478121
1418764_a_at	bisphosphate 3'-nucleotidase 1	Bpnt1	0.73233	1.10704823	0.14259458
1451610_at	chemokine (C-X-C motif) ligand 17	Cxcl17	0.73233	-1.11597157	-0.1583634
1432145_at	RIKEN cDNA 2310026I22 gene	2310026I22Rik	0.73234	1.13569168	0.18097035
1420948_s_at	alpha thalassemia/mental retardation syndrome X-linked homolo	Atrx	0.73236	-1.12663599	-0.1852732
1423713_at	ATP-binding cassette, sub-family B (MDR/TAP), member 8	Abcb8	0.7324	1.11933983	0.1597073
1427454_at	homeobox C6	Hoxc6	0.7324	-1.14973901	-0.2190315
1451522_s_at	leucine-rich repeats and calponin homology (CH) domain containi	Lrch4 /// Lrch4-sap25 ///	0.73242	1.09242551	0.12687369
1435623_at	ring finger protein, transmembrane 2	Rnft2	0.73244	1.14232808	0.18684305
1440816_x_at	DEAD (Asp-Glu-Ala-Asp) box polypeptide 1	Ddx1	0.73245	-1.1290995	-0.2030994
1417262_at	prostaglandin-endoperoxide synthase 2	Ptgs2	0.73245	1.10354662	0.14049372
1457726_at	ribosomal protein S15A	Rps15a	0.73249	1.12400125	0.16691107
1428404_at	fibrosin-like 1	Fbrsl1	0.73249	1.11084258	0.15141076
1452247_at	fragile X mental retardation gene 1, autosomal homolog	Fxr1	0.7325	-1.08309489	-0.1160629
1434650_at	pogo transposable element with ZNF domain	Pogz	0.73252	-1.09845758	-0.1401033
1431646_a_at	syntaxin 6	Stx6	0.73253	1.1175215	0.15857696
1456840_at	---	---	0.73254	-1.12838697	-0.1798101
1441150_x_at	SAM domain, SH3 domain and nuclear localization signals, 1	Samsn1	0.73254	1.10738087	0.14540512
1425339_at	phospholipase C, beta 4	Plcb4	0.73259	-1.16552527	-0.2690684
1430886_at	RIKEN cDNA 1700112E06 gene	1700112E06Rik	0.7326	1.09596347	0.13035933
1416631_at	adaptor-related protein complex AP-4, beta 1	Ap4b1	0.7326	1.11047609	0.14944576
1437158_at	Nipped-B homolog (Drosophila)	Nipbl	0.73261	-1.12456789	-0.1892723
1422896_at	vesicle-associated membrane protein 4	Vamp4	0.73264	1.18438903	0.22486008
1448753_at	signal recognition particle 9	Srp9	0.73264	1.08909679	0.12175163
1439034_at	sialophorin	Spn	0.73265	-1.1050646	-0.1450832
1424702_a_at	ATG2 autophagy related 2 homolog B (S. cerevisiae)	Atg2b	0.73268	1.11060356	0.14982011
1434839_s_at	transducin (beta)-like 1X-linked receptor 1	Tbl1xr1	0.7327	-1.09552848	-0.1352121
1453465_x_at	Ppp1r14c pseudogene	Gm14057	0.73273	1.12489238	0.16820191
1452416_at	interleukin 6 receptor, alpha	Il6ra	0.73274	-1.10625055	-0.1509115
1451093_at	polymerase (RNA) II (DNA directed) polypeptide E	Polr2e	0.73275	-1.11592075	-0.1602033
1435392_at	WD repeat domain 17	Wdr17	0.73277	1.12116208	0.16287868
1439412_at	---	---	0.73285	1.15148797	0.1920849
1418223_at	SEC11 homolog A (S. cerevisiae)	Sec11a	0.73308	1.07857837	0.10892367
1444289_at	Yip1 domain family, member 5	Yipf5	0.73321	1.13684812	0.18074167
1440815_x_at	predicted gene 8267	Gm8267	0.73322	1.1446729	0.18947731
1444794_at	---	---	0.73326	-1.13965702	-0.2004814
1440598_at	syntrophin, gamma 1	Sntg1	0.73327	1.11636452	0.1493801
1444418_at	Inositol 1,4,5-triphosphate receptor 2	Itpr2	0.73332	-1.12784198	-0.1809935
1417662_at	ELK3, member of ETS oncogene family	Elk3	0.73336	1.1607365	0.20075406
1428008_at	dynamin 1-like	Dnm1l	0.73337	-1.08701871	-0.1241911
1441069_at	zinc finger, DHHC domain containing 23	Zdhhc23	0.73366	1.12530814	0.16389004
1431088_at	forkhead box P4	Foxp4	0.73369	-1.10087852	-0.1425404
1442495_at	---	---	0.73375	-1.12358208	-0.1693923

1434110_x_at	novel member of the major urinary protein (Mup) gene family ///	LOC100048884 /// Mup1	0.73379	1.10946907	0.14626113
1422266_at	myc-like oncogene, s-myc protein	Mycs	0.73383	-1.13079659	-0.177669
1438394_x_at	keratin 4	Krt4	0.73385	-1.126332	-0.1734431
1456941_at	telomerase reverse transcriptase	Tert	0.73387	1.12584304	0.16961988
1435969_at	BTB (POZ) domain containing 12	Btbd12	0.73387	1.11146037	0.14735128
1426515_a_at	torsin family 1, member A (torsin A)	Tor1a	0.7339	1.09619351	0.13052384
1450520_at	calcium channel, voltage-dependent, gamma subunit 3	Cacng3	0.73398	-1.13193658	-0.1846304
1425966_x_at	ubiquitin C	Ubc	0.73411	1.10897875	0.14229001
1454797_at	transmembrane protein 55b	Tmem55b	0.73423	1.09146163	0.12474685
1418412_at	tumor protein D52-like 1	Tpd52l1	0.73427	1.09781569	0.13250762
1444253_at	a disintegrin-like and metallopeptidase (reprolysin type) with thro	Adamts18	0.73429	1.1118173	0.15043093
1418667_at	RIKEN cDNA 2410002O22 gene	2410002O22Rik	0.73456	1.10194167	0.13703525
1424246_a_at	testis derived transcript	Tes	0.7346	1.10212277	0.14009064
1426364_at	mitochondrial ribosome recycling factor	Mrrf	0.7347	1.09863412	0.13443049
1457260_at	RIKEN cDNA 5730409E04Rik gene	5730409E04Rik	0.73473	1.14430761	0.18582069
1431607_at	lysophospholipase 1	Lypla1	0.73473	1.13069606	0.1734361
1424456_at	poliovirus receptor-related 2	Pvrl2	0.73477	1.10708035	0.14172991
1434616_at	solute carrier family 38, member 10	Slc38a10	0.73478	1.16198518	0.20074431
1441986_at	zinc finger, CCHC domain containing 6	Zcchc6	0.73483	-1.12284775	-0.1679284
1440236_at	RIKEN cDNA A830039H05 gene	A830039H05Rik	0.73487	1.11413935	0.1547864
1417012_at	syndecan 2	Sdc2	0.73492	1.13225316	0.17518156
1429093_at	DNA-damage inducible protein 2	Ddi2	0.73494	-1.13009804	-0.1962054
1423288_s_at	cerebellin 1 precursor protein	Cbln1	0.73495	-1.114244	-0.160734
1451599_at	sestrin 2	Sesn2	0.73496	-1.10258638	-0.1479481
1450901_a_at	SMEK homolog 2, suppressor of mek1 (Dictyostelium)	Smek2	0.73498	-1.11223909	-0.1612627
1443849_x_at	uroporphyrinogen decarboxylase	Urod	0.73501	1.08487512	0.11667425
1446143_at	---	---	0.73501	-1.14776819	-0.2166448
1458479_at	Structural maintenance of chromosomes 2	Smc2	0.73502	-1.13521756	-0.185741
1460044_at	one cut domain, family member 2	Onecut2	0.73504	1.10726513	0.14639867
1448740_at	RAN guanine nucleotide release factor	Rangrf	0.73504	1.09406444	0.12599495
1431428_a_at	nitric oxide synthase interacting protein	Nosip	0.73505	1.09856826	0.13265863
1421733_a_at	protein-tyrosine sulfotransferase 1	Tpst1	0.73505	1.08669711	0.11975871
1424018_at	histidine triad nucleotide binding protein 1	Hint1	0.73505	1.09158311	0.11974182
1450454_at	torsin family 3, member A	Tor3a	0.73506	1.15262483	0.19406642
1444760_at	nuclear receptor co-repressor 1	Ncor1	0.73507	1.1475659	0.18981009
1439350_s_at	phosphatidylinositol glycan anchor biosynthesis, class U	Pigu	0.73509	-1.09815833	-0.1405521
1459858_x_at	cartilage acidic protein 1	Crtac1	0.73509	1.12629297	0.16639567
1455339_at	FK506 binding protein 15	Fkbp15	0.73509	1.10422325	0.14302131
1442238_a_at	kinesin family member 6	Kif6	0.7351	1.15676684	0.19964283
1418423_s_at	predicted gene 11397 /// serine (or cysteine) peptidase inhibitor,	Gm11397 /// Serpinb9e /	0.73511	-1.12777075	-0.1854193
1447103_at	CDK5 regulatory subunit associated protein 2	Cdk5rap2	0.73511	1.1563407	0.19671395
1433222_at	RIKEN cDNA 2610311E24 gene	2610311E24Rik	0.73513	1.13661719	0.17972361
1418465_at	neutrophil cytosolic factor 4	Ncf4	0.73514	-1.11558413	-0.1754158
1418377_a_at	SIVA1, apoptosis-inducing factor	Siva1	0.73516	1.12045007	0.15289717
1427432_a_at	transformer 2 beta homolog (Drosophila)	Tra2b	0.73516	-1.06957947	-0.0973525
1422476_at	interferon gamma inducible protein 30	Ifi30	0.73517	1.11043773	0.14640799
1451338_at	nischarin	Nisch	0.73518	1.10196922	0.13967721
1448572_at	prolactin family 4, subfamily a, member 1	Prl4a1	0.73519	-1.13576869	-0.187552
1427060_at	mitogen-activated protein kinase 3	Mapk3	0.73519	1.08652348	0.11857836
1431069_at	SPHK1 interactor, AKAP domain containing	Sphkap	0.73521	1.12835131	0.16993057
1436215_at	inositol polyphosphate multikinase	Ipmk	0.73523	-1.10741029	-0.1520256
1418870_at	RIKEN cDNA 4930579J09 gene	4930579J09Rik	0.73524	1.17606226	0.21496409
1453587_at	gamma-glutamyltransferase 6	Ggt6	0.73524	1.12063412	0.16215838
1432586_at	RIKEN cDNA 5730596P11 gene	5730596P11Rik	0.73525	-1.10131667	-0.1399234
1446509_at	spermine oxidase	Smox	0.73526	-1.10259707	-0.1410871
1426362_at	transmembrane protein 144	Tmem144	0.73526	-1.12353709	-0.1704808
1426354_at	Brca1 associated protein 1	Bap1	0.73527	1.09756671	0.13084147
1435734_x_at	dihydrouridine synthase 1-like (S. cerevisiae)	Dus1l	0.73527	1.09345977	0.12645984
1452781_a_at	general transcription factor IIIC, polypeptide 2, beta	Gtf3c2	0.73528	-1.10568813	-0.1562589
1441430_at	---	---	0.73528	1.08771598	0.11870354
1443063_at	clavesin 2	Clvs2	0.73529	1.11813413	0.16086447
1436327_a_at	vacuolar protein sorting 53 (yeast)	Vps53	0.7353	1.12455906	0.16401395
1425460_at	myotubularin related protein 2	Mtmr2	0.73531	-1.11938941	-0.1749668
1440095_at	---	---	0.73531	-1.13656749	-0.1918811
1433879_a_at	mitogen-activated protein kinase 1 interacting protein 1-like	Mapk1ip1l	0.73532	-1.09063179	-0.1302234
1451108_at	ring finger protein 185	Rnf185	0.73533	1.09230027	0.12666648
1453062_at	RIKEN cDNA A930026I22 gene	A930026I22Rik	0.73533	1.10068027	0.13744008
1453393_a_at	carbohydrate (chondroitin 6/keratan) sulfotransferase 4	Chst4	0.73534	-1.12734024	-0.1749647
1449611_at	CD82 antigen	Cd82	0.73534	-1.10668931	-0.1488636
1451107_at	TBC1 domain family, member 22a	Tbc1d22a	0.73535	1.10585202	0.14266217
1453912_at	zinc finger protein 558	Zfp558	0.73535	1.15686144	0.19240257
1443835_x_at	family with sequence similarity 100, member B /// 1110014K08R	Fam100b /// Gm7367	0.73535	-1.12095416	-0.1711981
1457540_at	---	---	0.73537	-1.14761503	-0.2163385
1438351_at	homeobox, msh-like 2	Msx2	0.73539	1.10221094	0.13549412
1450885_at	DNA fragmentation factor, alpha subunit	Dffa	0.73539	1.10950509	0.14621682
1449906_at	selectin, platelet	Selp	0.73539	1.15155636	0.19233716
1445942_at	expressed sequence AU015858	AU015858	0.7354	1.11035494	0.14851597
1445500_at	---	---	0.73543	-1.13114714	-0.1874901
1432462_a_at	mediator complex subunit 27	Med27	0.73543	1.09936939	0.13441129
1450169_at	solute carrier family 4 (anion exchanger), member 4	Slc4a4	0.73543	1.12539031	0.16694154
1443500_at	---	---	0.73544	-1.16680163	-0.2668096
1455502_at	MAP-kinase activating death domain	Madd	0.73545	1.10039527	0.13753445
1444457_at	---	---	0.73546	-1.12409066	-0.1740494
1423707_at	transmembrane protein 50B	Tmem50b	0.73548	1.10593301	0.14166695
1427017_at	special AT-rich sequence binding protein 2	Satb2	0.73552	-1.12645772	-0.1752736
1443246_at	FAST kinase domains 2	Fastkd2	0.73558	1.15342226	0.19659244
1424658_at	TAO kinase 1	Taok1	0.73562	-1.12793546	-0.1767915
1438333_at	protogenin homolog (Gallus gallus)	Prtg	0.73565	-1.13997997	-0.2017873
1437883_s_at	---	---	0.73569	-1.17310667	-0.2913727
1443698_at	XIAP associated factor 1	Xaf1	0.73571	1.15718922	0.20130346
1428689_at	trypsin domain containing 1	Tysnd1	0.73576	1.09686344	0.13325303
1455899_x_at	suppressor of cytokine signaling 3	Socs3	0.73579	-1.11027404	-0.1672239
1427031_s_at	coiled-coil domain containing 52	Ccdc52	0.7358	-1.10032955	-0.1436436
1439207_at	paraneoplastic antigen family 5	Pnma5	0.73597	1.12021035	0.16103104

1454694_a_at	topoisomerase (DNA) II alpha	Top2a	0.73604	1.07955747	0.10784487
1448647_at	mannosidase 2, alpha 1	Man2a1	0.73609	1.09805393	0.13235302
1453599_at	tripartite motif-containing 71	Trim71	0.73612	-1.15384577	-0.2575167
1420798_s_at	protocadherin alpha 1 /// protocadherin alpha 10 /// protocadher	Pcdha1 /// Pcdha10 /// Pc	0.73616	1.11241822	0.15064405
1458152_at	electron transferring flavoprotein, dehydrogenase	Etfdh	0.73623	-1.12415526	-0.1815084
1419614_at	phospholipase A2, group XIIB	Pla2g12b	0.73624	1.14050667	0.18264158
1457991_at	---	---	0.73631	1.13111348	0.17612399
1451422_at	myosin XVIIIA	Myo18a	0.73633	1.09742232	0.13165404
1459940_at	ACN9 homolog (S. cerevisiae)	Acn9	0.73635	1.11147126	0.14884868
1432827_x_at	ubiquitin C	Ubc	0.73642	1.09967252	0.13062188
1459996_at	Calcium channel, voltage-dependent, P/Q type, alpha 1A subunit	Cacna1a	0.73643	-1.12971613	-0.1866627
1424320_a_at	TNF receptor-associated factor 7	Traf7	0.73644	1.08198901	0.11318688
1460259_s_at	chloride channel calcium activated 1 /// chloride channel calcium	Clca1 /// Clca2	0.73645	1.17951947	0.21481569
1458518_at	cytoplasmic polyadenylation element binding protein 2	Cpeb2	0.73647	1.1620407	0.20307777
1455177_at	Abelson helper integration site 1	Ahi1	0.73652	1.11360272	0.15413999
1441142_at	RIKEN cDNA 2700081L22 gene	2700081L22Rik	0.73652	1.13788197	0.17927275
1426709_a_at	ubiquitin specific peptidase 33	Usp33	0.73653	-1.09340251	-0.1292901
1425306_at	cDNA sequence BC027072	BC027072	0.73653	-1.12300456	-0.1678187
1427243_at	RELT-like 1	Rell1	0.73655	1.10055696	0.13606757
1420724_at	RIKEN cDNA 1700067P10 gene	1700067P10Rik	0.73656	-1.12753893	-0.1754197
1416101_a_at	histone cluster 1, H1c	Hist1h1c	0.73656	1.11034697	0.14596997
1449892_at	lysozyme-like 1	Lyzl1	0.7366	1.12298795	0.16719111
1454154_at	RIKEN cDNA 4933416I08 gene	4933416I08Rik	0.73662	-1.15490114	-0.2366173
1448283_a_at	ubiquitin-like modifier activating enzyme 2	Uba2	0.73662	1.06919462	0.09583375
1416165_at	RAB31, member RAS oncogene family	Rab31	0.73663	1.09670297	0.13258239
1420227_at	---	---	0.73666	1.11547684	0.15670468
1458330_x_at	Cyclin M3	Cnnm3	0.73673	1.0997219	0.13554916
1436913_at	CDC14 cell division cycle 14 homolog A (S. cerevisiae)	Cdc14a	0.73673	1.13186536	0.17472467
1419981_at	RIO kinase 3 (yeast)	Riok3	0.73674	-1.14489192	-0.2133831
1420682_at	cholinergic receptor, nicotinic, beta polypeptide 1 (muscle)	Chrnb1	0.7368	1.13401013	0.17474172
1430594_at	RAB11 family interacting protein 1 (class I)	Rab11fip1	0.7368	1.11060984	0.15127726
1430432_at	tripartite motif-containing 42	Trim42	0.73683	1.17909129	0.21472289
1453725_a_at	mitchondrial ribosomal protein S7	Mrps7	0.73684	1.08597323	0.11722535
1446453_at	---	---	0.73684	1.17584064	0.21658275
1456688_at	---	---	0.73684	1.11698361	0.15192614
1435618_at	paraneoplastic antigen MA2	Pnma2	0.73684	1.11297582	0.14837791
1423733_a_at	Flt3 interacting zinc finger protein 1	Fiz1	0.73686	1.08590432	0.11729666
1450561_a_at	surfeit gene 1	Surf1	0.73687	1.09126515	0.1259479
1430630_at	---	---	0.73687	1.11909177	0.16111377
1458470_at	---	---	0.7369	-1.11446612	-0.1601003
1449598_at	---	---	0.73691	-1.13412292	-0.1926546
1447986_at	expressed sequence D17892	D17892	0.73692	-1.13337561	-0.1923681
1445420_at	---	---	0.73693	-1.09600679	-0.1324259
1447559_at	G protein-coupled receptor 25	Gpr25	0.73693	-1.12387656	-0.1693562
1450552_at	ATPase, H+ transporting, lysosomal V1 subunit E2	Atp6v1e2	0.73695	1.13057784	0.17321038
1439571_at	RIKEN cDNA E230008J23 gene	E230008J23Rik	0.73695	-1.14161738	-0.2043372
1415990_at	voltage-dependent anion channel 2	Vdac2	0.73696	1.06179209	0.08624995
1434198_at	ATPase inhibitory factor 1	Atpif1	0.73697	-1.11578829	-0.1601927
1452850_s_at	breast cancer metastasis-suppressor 1-like	Brms1l	0.73697	1.10572919	0.14131358
1449000_at	DNA segment, Chr 10, Johns Hopkins University 81 expressed	D10Jhu81e	0.73697	1.09197596	0.12338606
1449531_at	leprecan-like 2	Leprel2	0.737	-1.12043935	-0.1664221
1418266_at	arachidonate 12-lipoxygenase, 12R type	Alox12b	0.73701	1.13736326	0.18115578
1438474_at	ankyrin repeat domain 35	Ankrd35	0.73701	1.11574845	0.15499697
1449830_at	prolactin family 3, subfamily c, member 1	Prl3c1	0.73701	1.13964267	0.17851929
1427282_a_at	frataxin	Fxn	0.73701	1.10205802	0.13643653
1450092_at	immunoglobulin mu binding protein 2	Ighmbp2	0.73702	1.10634735	0.1420241
1427795_s_at	---	---	0.73702	1.17175681	0.20769387
1432513_a_at	RIKEN cDNA 1700001C02 gene	1700001C02Rik	0.73703	1.12953353	0.16903534
1418757_at	tripartite motif-containing 69	Trim69	0.73704	-1.129419	-0.1816751
1443620_at	Glypican 4	Gpc4	0.73704	-1.13209547	-0.1807004
1429843_at	glycine/arginine rich protein 1	Grrp1	0.73705	1.10724061	0.14618835
1430274_a_at	STARD3 N-terminal like	Stard3nl	0.73705	1.0875462	0.12025959
1443877_a_at	Rap guanine nucleotide exchange factor (GEF) 6	Rapgef6	0.73705	-1.1299364	-0.1849853
1425010_at	zinc finger protein 119a	Zfp119a	0.73706	-1.12845888	-0.1771987
1420111_at	Zinc finger protein 334	Zfp334	0.73706	1.11750518	0.16001382
1444631_at	Zinc finger protein 410	Zfp410	0.73706	-1.1164875	-0.1599469
1417104_at	epithelial membrane protein 3	Emp3	0.73706	1.12337824	0.16383292
1428307_at	zinc finger, DHHC domain containing 13	Zdhhc13	0.73706	1.09014261	0.1229716
1419288_at	junction adhesion molecule 2	Jam2	0.73707	-1.11202327	-0.1651681
1431400_a_at	growth arrest specific 7	Gas7	0.73707	1.1132678	0.15303975
1418874_a_at	proteasome (prosome, macropain) 26S subunit, non-ATPase, 4	Psmd4	0.73708	1.07861795	0.10858835
1416266_at	prodynorphin	Pdyn	0.73709	1.13150783	0.17605685
1428847_a_at	microtubule-actin crosslinking factor 1	Macf1	0.73709	-1.08989468	-0.1271619
1455649_at	tetratricopeptide repeat domain 9	Ttc9	0.73709	1.12246754	0.1658732
1416322_at	proline arginine-rich end leucine-rich repeat	Prelp	0.73711	1.12256813	0.16368688
1423820_at	elongation factor 1 homolog (ELF1, S. cerevisiae)	Elof1	0.73711	1.09571115	0.12849112
1442746_at	hypothetical LOC100504965	LOC100504965	0.73712	-1.12456928	-0.173348
1442186_at	ataxin 7	Atxn7	0.73712	-1.12635533	-0.1728795
1443726_at	SET and MYND domain containing 1	Smyd1	0.73712	-1.12705023	-0.17909
1417214_at	RAB27b, member RAS oncogene family	Rab27b	0.73713	-1.10622487	-0.1508457
1426215_at	dopa decarboxylase	Ddc	0.73713	1.11670851	0.15197936
1434527_at	NLR family, pyrin domain containing 4B	Nlrp4b	0.73714	1.13765683	0.17487514
1437566_at	hypothetical LOC100505087	LOC100505087	0.73714	1.1280473	0.17033395
1445737_at	RIKEN cDNA 1700057H21 gene	1700057H21Rik	0.73714	1.11763236	0.16007723
1421480_a_at	adenosine deaminase, RNA-specific, B1	Adarb1	0.73715	1.11624002	0.15797961
1434799_x_at	aldolase A, fructose-bisphosphate	Aldoa	0.73715	-1.05558316	-0.0787473
AFFX-BioB-M_at	---	---	0.73716	1.15546256	0.18835154
1438667_at	syntabulin (syntaxin-interacting)	Sybu	0.73716	1.12673422	0.16917474
1459514_at	---	---	0.73717	-1.13654852	-0.1893734
1446202_at	---	---	0.73717	-1.12220515	-0.1686312
1416933_at	P450 (cytochrome) oxidoreductase	Por	0.73717	1.10218238	0.13869773
1442321_at	COMM domain containing 2	Commd2	0.73717	-1.14126401	-0.2043598
1438409_at	centrosomal protein 63	Cep63	0.73719	1.11449817	0.15154767
1433244_at	RIKEN cDNA 4930573G07 gene	4930573G07Rik	0.73719	-1.13392786	-0.1849938

1428906_at	general transcription factor IIH, polypeptide 5	Gtf2h5	0.73721	1.08216816	0.11362146
1436971_x_at	tyrosine 3-monooxygenase/tryptophan 5-monooxygenase activa	Ywhaz	0.73722	-1.07844937	-0.1125361
1432138_at	RIKEN cDNA 1700080N15 gene	1700080N15Rik	0.73723	-1.13416861	-0.1959889
1452068_at	N-acylethanolamine acid amidase	Naaa	0.73723	1.14078258	0.18595941
1447119_at	---	---	0.73723	-1.12216787	-0.1718412
1437134_at	---	---	0.73724	1.11383235	0.1553226
1438951_x_at	nucleoporin 54	Nup54	0.73725	-1.08299696	-0.116082
1444406_at	---	---	0.73725	-1.13705302	-0.1983044
1455500_at	ring finger protein 213	Rnf213	0.73726	1.12112699	0.15538618
1430703_at	predicted gene 11559	Gm11559	0.73726	1.13726691	0.17363163
1436963_x_at	transmembrane protein 179B	Tmem179b	0.73728	1.12263425	0.16370848
1415675_at	dolichol-phosphate (beta-D) mannosyltransferase 2	Dpm2	0.73729	1.10321965	0.13847048
1430300_at	SCO cytochrome oxidase deficient homolog 1 (yeast)	Sco1	0.73729	-1.13133176	-0.193904
1443321_at	---	---	0.7373	-1.13163357	-0.1800399
1451532_s_at	six transmembrane epithelial antigen of the prostate 1	Steap1	0.7373	-1.08091801	-0.1127891
1445823_at	---	---	0.73731	1.11581416	0.15457364
1450572_at	alkaline phosphatase 3, intestine, not Mn requiring	Akp3	0.73732	-1.12885251	-0.1821535
1448840_at	transmembrane and ubiquitin-like domain containing 1	Tmub1	0.73732	1.11310269	0.15432799
1448202_x_at	PRELI domain containing 1	Prelid1	0.73733	1.075706	0.10435279
1431014_at	cat eye syndrome chromosome region, candidate 2 homolog (hu	Cecr2	0.73733	-1.10158273	-0.1417124
1449770_x_at	transmembrane protein 191C	Tmem191c	0.73733	-1.12478429	-0.1780115
1418298_s_at	dihydropyrimidinase-like 4	Dpysl4	0.73734	-1.13142162	-0.1860249
1449210_at	insulin-like growth factor 2 mRNA binding protein 1	Igf2bp1	0.73734	-1.12917045	-0.2053869
1448589_at	NADH dehydrogenase (ubiquinone) 1 beta subcomplex, 5	Ndufb5	0.73735	1.08340267	0.1120379
1443866_at	leucine-rich repeats and transmembrane domains 1	Lrtm1	0.73735	1.12777976	0.17198476
1445297_at	syntaxin binding protein 5-like	Stxbp5l	0.73735	-1.11510243	-0.158026
1453573_at	histone cluster 1, H3d	Hist1h3d	0.73736	1.12874363	0.17165228
1434486_x_at	UDP-glucose pyrophosphorylase 2	Ugp2	0.73736	-1.08847626	-0.1266313
1428361_x_at	hemoglobin alpha, adult chain 1 /// hemoglobin alpha, adult chai	Hba-a1 /// Hba-a2	0.73736	1.10877459	0.14845436
1438039_at	HECT domain containing 1	Hectd1	0.73737	-1.1381653	-0.2187152
1436859_at	RIKEN cDNA 2700007P21 gene	2700007P21Rik	0.73737	-1.08922295	-0.1278421
1457814_at	---	---	0.73737	-1.15892295	-0.2460064
1454854_at	organic solute transporter beta	Ostb	0.73738	1.11640367	0.15659939
1425801_x_at	coactosin-like 1 (Dictyostelium)	Cotl1	0.7374	1.10327262	0.13879674
1460254_at	RIKEN cDNA 1810049H13 gene	1810049H13Rik	0.7374	1.15224209	0.19004364
1428147_at	coronin 7	Coro7	0.7374	1.10608001	0.14433631
1423202_a_at	nuclear receptor co-repressor 1	Ncor1	0.73743	-1.08875224	-0.125914
1431276_at	RIKEN cDNA 9130019P16 gene	9130019P16Rik	0.73744	1.12759704	0.17194993
1448148_at	granulin	Grn	0.73744	1.0899373	0.12424021
1452355_at	retinal degeneration 3	Rd3	0.73745	-1.12236593	-0.1687145
1425783_at	tandem C2 domains, nuclear	Tc2n	0.73745	1.13084237	0.17181879
1426213_at	IMP4, U3 small nucleolar ribonucleoprotein, homolog (yeast)	Imp4	0.73746	1.0911907	0.12463359
1443329_at	membrane-associated ring finger (C3HC4) 7	Mar-07	0.73746	-1.11490663	-0.1574103
1453078_at	RIKEN cDNA 2610002M06 gene	2610002M06Rik	0.73748	1.16787029	0.20803282
1420013_s_at	lanosterol synthase	Lss	0.73748	1.08976598	0.12346487
1434863_at	DnaJ (Hsp40) homolog, subfamily C, member 18	Dnajc18	0.73749	1.09864986	0.13083013
1417032_at	ubiquitin-conjugating enzyme E2G 2	Ube2g2	0.73749	1.10525873	0.13977556
1441220_at	---	---	0.73749	-1.12745802	-0.1838103
1422489_at	mannosyl-oligosaccharide glucosidase	Mogs	0.73749	1.10996982	0.14504798
1424634_at	transcription elongation factor A (SII)-like 1	Tceal1	0.7375	-1.14165487	-0.2057237
1424069_at	N-ethylmaleimide sensitive fusion protein attachment protein ga	Napg	0.73751	1.10429049	0.1388684
1428706_at	centromere protein V	Cenpv	0.73752	-1.08550129	-0.1204854
1453734_at	alpha thalassemia/mental retardation syndrome X-linked homolo	Atrx	0.73752	-1.11572752	-0.1696636
1442071_at	ATP-binding cassette, sub-family E (OABP), member 1	Abce1	0.73754	1.18728271	0.21977126
1422488_at	NTF2-related export protein 1	Nxt1	0.73757	1.09006558	0.12226597
1423813_at	kinesin family member 22	Kif22	0.73757	1.09893825	0.13155575
1443860_at	protein tyrosine phosphatase, receptor type, D	Ptprd	0.73758	-1.12075469	-0.1687689
1432371_a_at	RIKEN cDNA 1700109K24 gene	1700109K24Rik	0.7376	1.1051313	0.13934196
1453398_at	RIKEN cDNA A930009A15 gene	A930009A15Rik	0.7376	1.16199623	0.20180889
1452047_at	calcyclin binding protein	Cacybp	0.7376	-1.07186464	-0.1016708
1435060_at	tropomodulin 2	Tmod2	0.73763	-1.15143674	-0.2310576
1452628_at	BCL2-associated athanogene 5	Bag5	0.73764	1.09355654	0.12699782
1428496_at	SECIS binding protein 2	Secisbp2	0.73766	1.09495427	0.1300839
1445273_at	DNA segment, Chr 10, ERATO Doi 638, expressed	D10Ertd638e	0.73768	-1.12702656	-0.1832388
1438100_at	RIKEN cDNA B230208H17 gene	B230208H17Rik	0.7377	-1.10239535	-0.1443951
1453380_a_at	XRCC6 binding protein 1	Xrcc6bp1	0.7377	1.09741661	0.13268572
1438661_a_at	ADP-ribosylation factor 2	Arf2	0.73771	1.12036183	0.16329777
1437284_at	frizzled homolog 1 (Drosophila)	Fzd1	0.73772	1.1228657	0.16553386
1418294_at	erythrocyte protein band 4.1-like 4b	Epb4.1l4b	0.73773	1.13903014	0.17588627
1458936_at	---	---	0.73774	-1.12977056	-0.1773853
1423570_at	ATP-binding cassette, sub-family G (WHITE), member 1	Abcg1	0.73774	1.1060093	0.14005585
1441573_at	Sex comb on midleg homolog 1	Scmh1	0.73775	1.22823063	0.25395556
1429427_s_at	transcription factor 7-like 2, T-cell specific, HMG-box	Tcf7l2	0.73775	-1.16105239	-0.2585156
1448779_at	CDKN1A interacting zinc finger protein 1	Ciz1	0.73775	1.10393578	0.14138289
1450283_at	ATPase, Cu++ transporting, beta polypeptide	Atp7b	0.73775	-1.13131766	-0.1808844
1434223_at	organic solute carrier partner 1	Oscp1	0.73775	1.13896103	0.18152923
1444256_at	RAE1 RNA export 1 homolog (S. pombe)	Rae1	0.73775	-1.13037217	-0.1817208
1436790_a_at	SRY-box containing gene 11	Sox11	0.73775	1.18323806	0.21603318
1445142_at	---	---	0.7378	-1.12284104	-0.1695951
1433379_at	RIKEN cDNA 9430019H13 gene	9430019H13Rik	0.73781	1.13747756	0.17891047
1421075_s_at	cytochrome P450, family 7, subfamily b, polypeptide 1	Cyp7b1	0.73782	1.1272529	0.16934714
1423681_at	FtsJ methyltransferase domain containing 2	Ftsjd2	0.73783	1.1036795	0.13974965
1453416_at	growth arrest-specific 2 like 3	Gas2l3	0.73784	-1.10817538	-0.1539889
1429065_at	IKBKB interacting protein	Ikbip	0.73786	1.1236612	0.16709243
1419766_at	salt inducible kinase 1	Sik1	0.73786	-1.11130261	-0.1535473
1438562_a_at	protein tyrosine phosphatase, non-receptor type 2	Ptpn2	0.73786	-1.08119607	-0.1144819
AFFX-r2-Bs-lys-3_	---	---	0.73787	1.14176905	0.18389993
1437151_at	ubiquitin specific peptidase 22	Usp22	0.73787	1.10503266	0.13749715
1439134_s_at	RIKEN cDNA 4930524O07 gene	4930524O07Rik	0.73791	1.16281829	0.2021332
1441417_at	STT3, subunit of the oligosaccharyltransferase complex, homolog	Stt3a	0.73793	-1.11539473	-0.1648515
1426701_at	family with sequence similarity 160, member A2	Fam160a2	0.73793	1.09455667	0.12816288
1438958_x_at	FK506 binding protein 1a	Fkbp1a	0.73794	-1.07977989	-0.1125036
1440499_at	DNA segment, Chr 9, ERATO Doi 26, expressed	D9Ertd26e	0.73794	-1.1388722	-0.2007292
1446957_s_at	NEDD4 binding protein 1	N4bp1	0.73797	-1.09286978	-0.1343453

1424184_at	acyl-Coenzyme A dehydrogenase, very long chain	Acadvl	0.73798	1.10478736	0.14148884
1438794_x_at	predicted gene 12270 /// ribosomal protein S13	Gm12270 /// Rps13	0.73798	-1.06078226	-0.0859438
1452099_at	expressed sequence AA408296	AA408296	0.73798	1.0795296	0.11011827
1420599_at	predicted gene 11554 /// predicted gene 11569 /// keratin assoc	Gm11554 /// Gm11569 //	0.73801	-1.11858043	-0.1627416
1436391_s_at	chloride channel CLIC-like 1	Clcc1	0.73802	1.11971424	0.15912747
1458720_at	---	---	0.73802	1.13011353	0.16996312
1453990_at	RIKEN cDNA 4930554G24 gene	4930554G24Rik	0.73802	1.12957534	0.16594316
1443188_at	ubiquitin-conjugating enzyme E2W (putative)	Ube2w	0.73804	-1.13010942	-0.1842005
1423618_at	bridging integrator 1	Bin1	0.73804	1.16153538	0.20025782
1424527_at	protein phosphatase 2, regulatory subunit B, delta isoform	Ppp2r2d	0.73804	-1.08169224	-0.1141182
1447224_at	---	---	0.73806	1.12589468	0.16959836
1430998_at	sulfide quinone reductase-like (yeast)	Sqrdl	0.73806	-1.13450193	-0.1952975
1418389_at	RIKEN cDNA 2810453I06 gene	2810453I06Rik	0.73807	1.09874083	0.13531844
1426517_at	guanine nucleotide binding protein, alpha z subunit	Gnaz	0.73807	1.12070784	0.1609324
1438752_at	RIKEN cDNA A230058F20 gene	A230058F20Rik	0.73808	1.10267732	0.1403417
1440857_at	carbamoyl-phosphate synthetase 2, aspartate transcarbamylase	Cad	0.73808	-1.11017312	-0.1536622
1455545_at	RIKEN cDNA 1110065P20 gene	1110065P20Rik	0.73809	1.10256967	0.13565376
1426875_s_at	sulfiredoxin 1 homolog (S. cerevisiae)	Srxn1	0.7381	1.09286446	0.12743982
1422163_at	SH3 and PX domains 2A	Sh3pxd2a	0.73811	-1.10346664	-0.1424452
1416112_at	cytochrome c oxidase, subunit VIIIa	Cox8a	0.73811	1.0833778	0.11169095
1416528_at	SH3 domain binding glutamic acid-rich protein-like 3	Sh3bgrl3	0.73811	1.08489884	0.11645017
1456296_at	RIKEN cDNA 5830418K08 gene	5830418K08Rik	0.73812	1.17257877	0.20506257
1426785_s_at	monoglyceride lipase	Mgll	0.73813	-1.13731481	-0.1971657
1435267_at	RIKEN cDNA A430108E01 gene /// hypothetical LOC100504289	A430108E01Rik /// LOC1(	0.73813	-1.10636768	-0.1511249
1425734_a_at	coiled-coil domain containing 77	Ccdc77	0.73814	1.12599051	0.1641522
1432146_at	cation channel, sperm associated 3	Catsper3	0.73814	1.15931936	0.19981494
1458347_s_at	transmembrane protease, serine 2	Tmprss2	0.73814	-1.13169931	-0.1876488
1423209_at	transmembrane protein 167	Tmem167	0.73816	1.13995022	0.1822495
1436637_at	---	---	0.73817	1.16413815	0.20635441
1437772_s_at	fucosidase, alpha-L- 1, tissue	Fuca1	0.73817	1.09952294	0.13547653
1423476_at	solute carrier family 46, member 2	Slc46a2	0.73817	-1.1200498	-0.1654646
1449066_a_at	Rho guanine nucleotide exchange factor (GEF7)	Arhgef7	0.73817	1.09542999	0.13051712
1442377_at	hypothetical LOC100504141	LOC100504141	0.73818	1.13488543	0.17525427
1417506_at	geminin	Gmnn	0.73819	1.08338364	0.1143046
1432560_at	kallikrein 1-related peptidase b7, pseudogene	Klk1b7-ps	0.7382	-1.12087348	-0.1669129
1457069_at	activating signal cointegrator 1 complex subunit 3	Ascc3	0.7382	-1.11480967	-0.161181
1423847_at	non-SMC condensin I complex, subunit D2	Ncapd2	0.7382	1.08297724	0.11318277
1424858_at	L-2-hydroxyglutarate dehydrogenase	L2hgdh	0.73821	1.11829824	0.15305886
1450866_a_at	mitochondrial ribosomal protein L17	Mrpl17	0.73821	1.09930524	0.13019615
1424384_a_at	zinc and ring finger 1	Znrf1	0.73821	1.10698176	0.14570766
1422443_at	X-prolyl aminopeptidase (aminopeptidase P) 1, soluble	Xpnpep1	0.73822	1.08574501	0.11721291
1416292_at	peroxiredoxin 3	Prdx3	0.73822	1.08340861	0.11511189
1436798_at	Ribosomal protein L9	Rpl9	0.73824	-1.12855425	-0.179049
1448818_at	wingless-related MMTV integration site 5A	Wnt5a	0.73825	1.12662757	0.1708481
1446201_at	---	---	0.73825	1.14030266	0.18288518
1420661_a_at	RIKEN cDNA 4933439F18 gene	4933439F18Rik	0.73827	1.1024327	0.14015986
1434439_at	glycogen synthase kinase 3 beta	Gsk3b	0.73828	1.13055015	0.1749618
1449618_s_at	RIKEN cDNA 2900092E17 gene	2900092E17Rik	0.73828	1.10213276	0.13418511
1451875_at	RIKEN cDNA 4930444G20 gene /// cDNA sequence AF366264	4930444G20Rik /// AF36(	0.73829	1.12570836	0.16636609
1429989_at	RIKEN cDNA 1700008A04 gene	1700008A04Rik	0.73831	1.12721832	0.17078016
1445924_at	predicted gene 13151 /// predicted gene 13235 /// zinc finger pr	Gm13151 /// Gm13235 //	0.73832	-1.11600024	-0.1641571
1448292_at	ubiquinol-cytochrome c reductase, complex III subunit XI	Uqcr11	0.73832	1.11146925	0.14292162
1429749_at	Scm-like with four mbt domains 1	Sfmbt1	0.73833	-1.16551963	-0.2634558
1452434_s_at	DiGeorge syndrome critical region gene 6	Dgcr6	0.73838	1.10599871	0.14092036
1434535_at	keratin 222	Krt222	0.73839	1.15341715	0.19424482
1430670_at	coiled-coil domain containing 91	Ccdc91	0.7384	-1.12594388	-0.1763928
1453283_at	phosphoglucomutase 1	Pgm1	0.73841	1.09788412	0.13154062
1449359_at	paired box gene 1	Pax1	0.73842	-1.1269535	-0.1773155
1431877_a_at	grainyhead-like 2 (Drosophila)	Grhl2	0.73843	-1.11651614	-0.1626958
1460188_at	protein tyrosine phosphatase, non-receptor type 6	Ptpn6	0.73844	1.09448744	0.12954872
1437415_at	RIKEN cDNA 4933427D06 gene	4933427D06Rik	0.73848	1.12822496	0.16979287
1436356_at	sterile alpha motif domain containing 4	Samd4	0.7385	-1.13112264	-0.1880053
1432216_s_at	membrane protein, palmitoylated 7 (MAGUK p55 subfamily mem	Mpp7	0.73851	-1.13595025	-0.1944783
1455146_at	spermatid perinuclear RNA binding protein	Strbp	0.73853	-1.11211746	-0.1558298
1415791_at	ring finger protein 34	Rnf34	0.73855	1.08590524	0.11771085
1430248_at	RIKEN cDNA 4930415O11 gene	4930415O11Rik	0.73855	-1.11870061	-0.1623552
1453517_at	methionyl aminopeptidase type 1D (mitochondrial)	Metap1d	0.73856	1.10414997	0.14196027
1427783_at	v-erb-a erythroblastic leukemia viral oncogene homolog 4 (avian	Erbb4	0.73857	1.12692376	0.16860979
1440621_at	---	---	0.73861	-1.11983843	-0.1648993
1423131_at	NSA2 ribosome biogenesis homolog (S. cerevisiae)	Nsa2	0.73863	-1.1246369	-0.1774023
1456650_at	---	---	0.73863	1.12326549	0.16486494
1448381_at	G elongation factor, mitochondrial 1	Gfm1	0.73863	1.1108407	0.15099865
1432262_at	family with sequence similarity 63, member A	Fam63a	0.73864	1.12633735	0.16374891
1422833_at	forkhead box A2	Foxa2	0.73864	1.13591195	0.17960193
1439421_x_at	chromobox homolog 3 (Drosophila HP1 gamma)	Cbx3	0.73864	1.17397732	0.20085074
1442138_at	G protein-coupled receptor 62	Gpr62	0.73868	1.12112316	0.16454679
1425581_s_at	UDP-N-acetyl-alpha-D-galactosamine: polypeptide N-acetylgalac	Galnt7	0.73868	1.14465571	0.18555794
1430545_at	DBF4 homolog (S. cerevisiae)	Dbf4	0.7387	1.18154883	0.21761589
1427437_at	---	---	0.73871	-1.10950465	-0.1522325
1439627_at	zinc finger protein of the cerebellum 1	Zic1	0.73871	1.14980143	0.18421014
1423967_at	paralemmin	Palm	0.73871	1.11475897	0.15051038
1445876_at	---	---	0.73872	1.14286289	0.181627
1458194_at	---	---	0.73873	1.12906307	0.17210396
1442920_at	Kruppel-like factor 3 (basic)	Klf3	0.73874	-1.11829695	-0.1634106
1441273_at	---	---	0.73874	-1.11766138	-0.161006
1419897_at	Tet oncogene 1	Tet1	0.73875	-1.12817607	-0.1809794
1441450_s_at	lysine (K)-specific demethylase 5C	Kdm5c	0.73878	-1.13340362	-0.2083521
1429616_at	zinc finger protein 91	Zfp91	0.73878	-1.14493883	-0.2128479
1449052_a_at	DNA methyltransferase 3B	Dnmt3b	0.73878	-1.08111637	-0.1130826
1416620_at	SWI/SNF related matrix associated, actin dependent regulator of	Smarcal1	0.73879	1.10436632	0.13786152
1425467_a_at	proteolipid protein (myelin) 1	Plp1	0.73879	-1.10589665	-0.1481498
1448571_a_at	glia maturation factor, beta	Gmfb	0.7388	-1.10400599	-0.1430591
1441655_at	predicted gene 884	Gm884	0.7388	-1.10706563	-0.1469193
1434408_at	ataxin 3	Atxn3	0.7388	-1.11930654	-0.1693047

1423500_a_at	SRY-box containing gene 5	Sox5	0.7388	-1.12079882	-0.1678875
1428973_s_at	Tctex1 domain containing 2	Tctex1d2	0.7388	1.10177671	0.13614338
1443171_at	---	---	0.73882	-1.14174251	-0.2056884
1422063_a_at	peroxisomal biogenesis factor 5	Pex5	0.73885	1.09415733	0.12944608
1418814_s_at	NADH dehydrogenase (ubiquinone) 1 alpha subcomplex, 12	Ndufa12	0.73886	1.09752887	0.12945082
1436885_a_at	calcium homeostasis endoplasmic reticulum protein	Cherp	0.73886	-1.09292776	-0.1301191
1457302_at	Solute carrier family 20, member 2	Slc20a2	0.73886	-1.1051395	-0.1462257
1422567_at	family with sequence similarity 129, member A	Fam129a	0.73886	-1.09965703	-0.1407545
1435609_at	transformation related protein 53 binding protein 1	Trp53bp1	0.73887	1.113431	0.15406163
1435004_at	pantothenate kinase 4	Pank4	0.73888	1.089202	0.12270005
1459464_at	---	---	0.73889	-1.13223755	-0.1844067
1426257_a_at	seryl-aminoacyl-tRNA synthetase	Sars	0.73889	-1.07508576	-0.1047231
1457524_at	---	---	0.7389	-1.11872046	-0.1631624
1431853_at	RIKEN cDNA 4933413C19 gene	4933413C19Rik	0.7389	-1.12797597	-0.1830702
1441503_at	doublecortin domain containing 2a	Dcdc2a	0.73892	-1.12825118	-0.1786364
1446958_at	DNA segment, Chr 13, ERATO Doi 150, expressed	D13Ertd150e	0.73892	-1.12293434	-0.1679067
1450793_at	RIKEN cDNA 4930550L24 gene	4930550L24Rik	0.73893	1.12591414	0.16821993
1436729_at	actin filament associated protein 1	Afap1	0.73894	1.12230091	0.16415943
1428722_at	creatine kinase, mitochondrial 2	Ckmt2	0.73895	-1.14246846	-0.2162967
1458339_at	cytidine and dCMP deaminase domain containing 1	Cdadc1	0.73895	-1.12279949	-0.1700889
1440592_at	---	---	0.73897	1.11439642	0.15516869
1435358_at	CUE domain containing 1	Cuedc1	0.73899	1.10705562	0.14473301
1430806_at	RIKEN cDNA 2810047F03 gene	2810047F03Rik	0.73899	1.10662305	0.14233341
1444110_at	---	---	0.73899	-1.13265357	-0.1802816
1457905_at	DNA segment, Chr 7, ERATO Doi 59, expressed	D7Ertd59e	0.73899	-1.12948409	-0.1932586
1425699_a_at	abhydrolase domain containing 14A	Abhd14a	0.73899	1.10310585	0.13820075
1458263_at	---	---	0.73901	1.12490786	0.16978488
1428156_at	guanine nucleotide binding protein (G protein), gamma 2	Gng2	0.73902	-1.10561189	-0.1471531
1437106_at	lysine (K)-specific demethylase 5A	Kdm5a	0.73902	-1.1278845	-0.1785819
1436406_at	---	---	0.73906	-1.13157781	-0.1802968
1439221_s_at	CD40 antigen	Cd40	0.73906	1.12701872	0.17058955
1440123_at	---	---	0.73908	-1.1119477	-0.1540894
1447935_at	Fanconi anemia, complementation group M	Fancm	0.73912	-1.10485733	-0.1491624
1423014_at	protocadherin beta 6	Pcdhb6	0.7392	-1.15758484	-0.2454209
1449026_at	interferon (alpha and beta) receptor 1	Ifnar1	0.7392	1.09559689	0.13081514
1456373_x_at	ribosomal protein S20	Rps20	0.73929	-1.07101547	-0.1080899
1425266_a_at	RAP1, GTP-GDP dissociation stimulator 1	Rap1gds1	0.7393	1.09581893	0.13176029
1421978_at	glutamic acid decarboxylase 2	Gad2	0.7393	1.13238467	0.17509205
1446653_at	---	---	0.73931	1.1284553	0.1717075
1458076_at	---	---	0.73932	-1.11707948	-0.1625162
1433504_at	brain glycogen phosphorylase	Pygb	0.73935	1.10639322	0.14353142
1442441_at	ribonuclease, RNase K	Rnasek	0.73935	1.149194	0.18965224
1431794_at	RIKEN cDNA 4932434E15 gene	4932434E15Rik	0.73938	-1.12422327	-0.1747615
1425566_at	RE1-silencing transcription factor	Rest	0.7394	-1.1248753	-0.1901419
1422187_at	gamma-aminobutyric acid (GABA) A receptor, subunit gamma 3	Gabrg3	0.7394	-1.11665602	-0.1607745
1428155_at	COMM domain containing 9	Commd9	0.73941	1.11766878	0.15251303
1439679_at	chaperonin containing Tcp1, subunit 3 (gamma)	Cct3	0.73942	-1.11579389	-0.1582799
1422731_at	LIM domains containing 1	Limd1	0.73942	-1.15621117	-0.2651073
1415906_at	thymosin, beta 4, X chromosome	Tmsb4x	0.73943	1.0575605	0.07944286
1420344_x_at	granzyme D	Gzmd	0.73944	-1.07415312	-0.1037659
1446148_x_at	RNA binding motif protein 39	Rbm39	0.73945	-1.13858047	-0.2176735
1446693_at	FSHD region gene 1	Frg1	0.73946	1.13520407	0.17853417
1426337_a_at	TEA domain family member 4	Tead4	0.73947	1.10826843	0.14654085
1425325_at	membrane-spanning 4-domains, subfamily A, member 4B	Ms4a4b	0.73949	-1.09006664	-0.1295167
1441042_at	fibroblast growth factor 1	Fgf1	0.7395	-1.12759478	-0.1766358
1435326_at	lysophosphatidylglycerol acyltransferase 1	Lpgat1	0.7395	1.09960504	0.13640318
1434261_at	signal-induced proliferation-associated 1 like 2	Sipa1l2	0.73953	1.10192761	0.13893771
1450269_a_at	phosphofructokinase, liver, B-type	Pfkl	0.73958	1.08675755	0.11826008
1454724_x_at	family with sequence similarity 108, member B	Fam108b	0.73963	-1.08620464	-0.1207318
1435357_at	DNA segment, Chr 4, Wayne State University 53, expressed	D4Wsu53e	0.73963	1.12292635	0.16234498
1445992_at	---	---	0.73963	1.11858009	0.16047887
1440493_at	UDP-N-acetyl-alpha-D-galactosamine:polypeptide N-acetylgalact	Galnt10	0.73967	1.1351988	0.17762624
1460563_at	nuclear undecaprenyl pyrophosphate synthase 1 homolog (S. cer	Nus1	0.73969	-1.08800442	-0.1246853
1452219_at	transmembrane protein 63b	Tmem63b	0.73969	1.10368436	0.13893607
1455211_a_at	translocase of inner mitochondrial membrane 13 homolog (yeast	Timm13	0.73972	1.08835616	0.11873647
1421699_at	enamelin	Enam	0.74005	1.13168902	0.17534344
1451428_x_at	EGF-like domain 7	Egfl7	0.74009	1.102624	0.13692159
1450311_at	solute carrier family 8 (sodium/calcium exchanger), member 3	Slc8a3	0.74038	-1.12572112	-0.1755241
1421919_a_at	chemokine (C-C motif) receptor 9	Ccr9	0.74048	-1.11767464	-0.1639906
1446069_at	---	---	0.74056	-1.11607884	-0.1653181
1435656_at	guanine monophosphate synthetase	Gmps	0.74057	-1.11879942	-0.1643908
1416643_at	tumor protein, translationally-controlled 1	Tpt1	0.74066	1.11657568	0.15611588
1450460_at	aquaporin 3	Aqp3	0.74067	-1.10755724	-0.1535512
1444295_at	neogenin	Neo1	0.74078	1.2127273	0.24595798
1449248_at	chloride channel 2	Clcn2	0.74081	1.09482072	0.12907342
1422053_at	inhibin beta-A	Inhba	0.74081	1.11543595	0.15756424
1427233_at	teashirt zinc finger family member 1	Tshz1	0.74083	1.13006224	0.16987729
1435862_at	Son DNA binding protein	Son	0.74101	-1.08511501	-0.1204162
1428135_a_at	eukaryotic translation elongation factor 1 delta (guanine nucleoti	Eef1d	0.74117	1.10193245	0.13937798
1456639_at	zinc finger protein 398	Zfp398	0.74127	-1.12826983	-0.1749888
1430664_at	zinc finger protein 169	Zfp169	0.74128	1.13795462	0.17925834
1437223_s_at	X-box binding protein 1	Xbp1	0.74129	1.08642913	0.1169702
1448166_a_at	proteasome (prosome, macropain) subunit, beta type 1	Psmb1	0.74135	1.07352348	0.10105843
1455099_at	monoacylglycerol O-acyltransferase 2	Mogat2	0.74143	-1.14039738	-0.208231
1428516_a_at	alkB, alkylation repair homolog 7 (E. coli)	Alkbh7	0.74153	1.09364772	0.12628106
1443141_at	---	---	0.74155	1.13762413	0.17965347
1454775_at	histone deacetylase 10	Hdac10	0.74159	-1.10512752	-0.1451537
1423432_at	pleckstrin homology domain interacting protein	Phip	0.74159	-1.11707178	-0.179426
1425975_a_at	mitogen-activated protein kinase 8 interacting protein 3	Mapk8ip3	0.74167	1.1139512	0.15435814
1439012_a_at	deoxycytidine kinase	Dck	0.74198	-1.11529368	-0.1764239
1417298_at	emopamil binding protein-like	Ebpl	0.74236	1.1041122	0.13882104
1446536_at	sema domain, transmembrane domain (TM), and cytoplasmic do	Sema6d	0.74242	-1.12117576	-0.1677449
1454075_s_at	nudix (nucleoside diphosphate linked moiety X)-type motif 13	Nudt13	0.74246	1.11502056	0.15469849
1448669_at	dickkopf homolog 3 (Xenopus laevis)	Dkk3	0.74248	1.16337367	0.19913429

1436568_at	junction adhesion molecule 2	Jam2	0.7425	-1.10036956	-0.1437816
1443508_at	discs, large (Drosophila) homolog-associated protein 1	Dlgap1	0.74251	-1.12378698	-0.1761863
1433013_at	RIKEN cDNA 4930485G23 gene	4930485G23Rik	0.74253	1.13454285	0.17766381
1446242_at	---	---	0.74254	-1.12184801	-0.165962
1440904_at	SUMO/sentrin specific peptidase 5	Senp5	0.7426	1.12859327	0.16823639
1457678_at	RIKEN cDNA 2310035C23 gene	2310035C23Rik	0.74261	-1.10563471	-0.149297
1442424_at	---	---	0.74268	1.07455643	0.10095876
1418501_a_at	oxidation resistance 1	Oxr1	0.74287	-1.09537144	-0.1314928
1448339_at	transmembrane protein 30A	Tmem30a	0.74288	-1.09331317	-0.1342371
1440617_at	carboxypeptidase A6	Cpa6	0.74291	-1.11224884	-0.1555881
1457338_at	---	---	0.74293	-1.11817978	-0.1667156
1424648_at	intraflagellar transport 27 homolog (Chlamydomonas)	Ift27	0.74298	1.11278175	0.15061092
1436978_at	wingless-type MMTV integration site 9A	Wnt9a	0.74301	1.1351302	0.18043515
1422688_a_at	neuroblastoma ras oncogene	Nras	0.74304	-1.08964469	-0.1239783
1432768_at	RIKEN cDNA 6430710M23 gene	6430710M23Rik	0.74308	1.13869667	0.17969798
1426789_s_at	structure specific recognition protein 1	Ssrp1	0.7431	1.0876159	0.11843675
1457120_at	IL2-inducible T-cell kinase	Itk	0.74317	1.12621006	0.15963868
1417320_at	GrpE-like 1, mitochondrial	Grpel1	0.74327	1.08223319	0.11218254
1438750_at	Alpha thalassemia/mental retardation syndrome X-linked homolo	Atrx	0.74327	-1.10198595	-0.1456049
1455013_at	ariadne homolog 2 (Drosophila)	Arih2	0.74337	1.09111787	0.12505239
1453705_at	RIKEN cDNA B230110C06 gene	B230110C06Rik	0.74339	1.12733327	0.17095615
1450667_a_at	citrate synthase	Cs	0.74341	1.10615218	0.13834446
1425062_at	Fc receptor-like 1	Fcrl1	0.74342	-1.10443537	-0.1477827
1456377_x_at	LIM domain containing 2	Limd2	0.74342	-1.1143811	-0.1713444
1456198_at	RIKEN cDNA 1810007D17 gene	1810007D17Rik	0.74343	1.10978683	0.1465455
1419797_at	expressed sequence AA672641	AA672641	0.74345	1.10136024	0.13734974
1434388_at	MOB1, Mps One Binder kinase activator-like 2A (yeast)	Mobkl2a	0.74347	1.09864612	0.13422078
1446603_at	---	---	0.74347	-1.10485376	-0.1453703
1426343_at	STT3, subunit of the oligosaccharyltransferase complex, homolog	Stt3b	0.74351	1.08614336	0.11733659
1448172_at	malate dehydrogenase 1, NAD (soluble)	Mdh1	0.74352	1.08141424	0.11219171
1456547_at	hypothetical protein LOC100502668	LOC100502668	0.74353	1.1793062	0.2114784
1426384_a_at	tyrosine 3-monooxygenase/tryptophan 5-monooxygenase activa	Ywhae	0.74358	-1.06510539	-0.0920548
1428844_a_at	BCL2-associated transcription factor 1	Bclaf1	0.74359	-1.08191702	-0.1139949
1448982_at	kallikrein related-peptidase 6	Klk6	0.74363	-1.11562277	-0.1590249
1442935_at	---	---	0.74372	-1.12029833	-0.1647167
1434451_at	predicted gene 10786	Gm10786	0.74372	-1.11676876	-0.1610784
1452489_at	vacuolar protein sorting 11 (yeast)	Vps11	0.74373	1.1312102	0.17338107
1436976_a_at	YOD1 OTU deubiquitinating enzyme 1 homologue (S. cerevisiae)	Yod1	0.74374	1.12238138	0.16444839
1420058_s_at	family with sequence similarity 54, member B	Fam54b	0.74377	1.08887357	0.12273336
1459293_at	---	---	0.74378	-1.14092569	-0.2101175
1447065_at	predicted gene 5107	Gm5107	0.7438	-1.13019088	-0.1815366
1425810_a_at	cysteine and glycine-rich protein 1	Csrp1	0.74381	1.10824391	0.1427422
1455172_at	expressed sequence AU020094	AU020094	0.74384	1.10469114	0.13902308
1442497_at	SMC hinge domain containing 1	Smchd1	0.74386	-1.13345348	-0.1932842
1439754_at	SRY-box containing gene 12	Sox12	0.74386	1.10627022	0.14544559
1450430_at	mannose receptor, C type 1	Mrc1	0.74387	1.11506614	0.15279656
1436248_at	RAS protein activator like 2	Rasal2	0.74388	-1.10357462	-0.1438703
1446824_at	---	---	0.74389	-1.13484438	-0.1916253
1422206_at	UDP-Gal:betaGlcNAc beta 1,3-galactosyltransferase, polypeptide	B3galt1	0.74391	1.12217361	0.16423621
1439993_at	---	---	0.74391	-1.10546801	-0.1453703
1427451_a_at	cDNA sequence BC018473	BC018473	0.74391	1.12740939	0.16978317
1416252_at	serine/threonine kinase 38	Stk38	0.74393	1.07946045	0.11014166
1431776_at	transducer of ERBB2, 2	Tob2	0.74394	1.12052592	0.16293371
1447843_at	proteasome (prosome, macropain) 26S subunit, non-ATPase, 13	Psmd13	0.74394	-1.11025859	-0.1521456
1423795_at	splicing factor proline/glutamine rich (polypyrimidine tract binding	Sfpq	0.74396	-1.08300465	-0.1191398
1448968_at	ubiquitin family domain containing 1	Ubfd1	0.74396	1.09292317	0.1243874
1422486_a_at	MAD homolog 4 (Drosophila)	Smad4	0.74399	1.10728796	0.14010178
1426984_at	RIKEN cDNA 2310067B10 gene	2310067B10Rik	0.74403	-1.11493725	-0.1638818
1441171_at	predicted gene 1614	Gm1614	0.74403	-1.12611314	-0.1769764
1442578_at	regulatory factor X, 2 (influences HLA class II expression)	Rfx2	0.74406	-1.13526173	-0.1979061
1458724_at	RIKEN cDNA E230008O15 gene	E230008O15Rik	0.74406	-1.13142455	-0.1886004
1444314_at	---	---	0.7441	1.12785423	0.17119449
1439462_x_at	transmembrane emp24-like trafficking protein 10 (yeast)	Tmed10	0.74412	-1.13678062	-0.2228861
1431223_at	RIKEN cDNA 2700054A10 gene	2700054A10Rik	0.74413	1.13840322	0.18357223
1422807_at	ADP-ribosylation factor 5	Arf5	0.74416	1.18446323	0.21115531
1423725_at	plastin 3 (T-isoform)	Pls3	0.74418	-1.11814968	-0.1733665
1450764_at	acyloxyacyl hydrolase	Aoah	0.74421	-1.11973755	-0.1777196
1443105_at	---	---	0.74423	1.14299596	0.18185031
1432281_a_at	integrin beta 6	Itgb6	0.74423	-1.11173274	-0.1532602
1431219_at	beta-1,3-glucuronyltransferase 3 (glucuronosyltransferase I)	B3gat3	0.74424	-1.12610862	-0.1783298
1424930_s_at	family with sequence similarity 83, member F	Fam83f	0.74428	-1.13108743	-0.1903882
1456014_s_at	fermitin family homolog 3 (Drosophila)	Fermt3	0.7443	1.11649659	0.1579096
1437089_at	pyridine nucleotide-disulphide oxidoreductase domain 2	Pyroxd2	0.74433	-1.13065901	-0.181427
1425859_a_at	proteasome (prosome, macropain) 26S subunit, non-ATPase, 4	Psmd4	0.74436	1.08257276	0.11342522
1450625_at	collagen, type V, alpha 2	Col5a2	0.74437	-1.11882866	-0.163779
1421790_a_at	potassium voltage-gated channel, shaker-related subfamily, beta	Kcnab3	0.7444	-1.11993099	-0.1661613
1427326_at	RIKEN cDNA 4732471D19 gene	4732471D19Rik	0.7444	1.10174463	0.13842862
1416398_at	mesoderm development candidate 1	Mesdc1	0.74441	1.12667448	0.16859685
1434545_x_at	bolA-like 2 (E. coli)	Bola2	0.74441	1.13375895	0.16482202
1432211_a_at	f-box protein 9	Fbxo9	0.74441	1.10694192	0.14579874
1437326_x_at	chymotrypsin-like elastase family, member 3B	Cela3b	0.74447	-1.11765014	-0.1616876
1452341_at	enoyl Coenzyme A hydratase, short chain, 1, mitochondrial	Echs1	0.74447	1.08336033	0.11531016
1430029_a_at	tetraspanin 31	Tspan31	0.7445	1.08511593	0.11716207
1421922_at	SH3-domain binding protein 5 (BTK-associated)	Sh3bp5	0.74453	-1.12891473	-0.183383
1438781_at	Tet oncogene family member 2	Tet2	0.74454	1.17748091	0.20543286
1431670_at	NADH dehydrogenase (ubiquinone) 1, subcomplex unknown, 2	Ndufc2	0.74454	1.13849793	0.18011871
1458969_at	expressed sequence AU019559	AU019559	0.74457	-1.11927011	-0.1643211
1430955_at	RIKEN cDNA 2810403A07 gene	2810403A07Rik	0.74457	-1.12019163	-0.1666466
1437984_x_at	HLA-B-associated transcript 1A	Bat1a	0.7446	1.07268858	0.09841732
1452036_a_at	thymopoietin	Tmpo	0.74462	-1.08611664	-0.1242818
1451864_at	calcium channel, voltage-dependent, gamma subunit 8	Cacng8	0.74463	-1.1257102	-0.1839973
1426643_at	elongation protein 3 homolog (S. cerevisiae)	Elp3	0.74465	1.08252548	0.11433957
1434689_at	zinc finger protein 637	Zfp637	0.74466	1.10053331	0.13748325
1420031_at	checkpoint kinase 1 homolog (S. pombe)	Chek1	0.74467	-1.12417686	-0.1721109

1434231_x_at	ribosomal protein L35	Rpl35	0.74467	1.04049753	0.05703552
1452672_at	THO complex 5	Thoc5	0.74468	1.07951157	0.1098355
1417939_at	RAD51 associated protein 1	Rad51ap1	0.74469	1.12374751	0.16091308
1426937_at	RIKEN cDNA 6330406I15 gene	6330406I15Rik	0.74469	1.15501052	0.19276393
1427729_at	EH-domain containing 2	Ehd2	0.7447	1.11941303	0.15629913
1429953_at	RIKEN cDNA 2210011C24 gene	2210011C24Rik	0.74471	1.13554879	0.17185083
1446749_at	kinectin 1	Ktn1	0.74471	1.10489705	0.14064979
1436196_at	hypothetical protein C030046G05	C030046G05	0.74473	1.16308705	0.19629519
1434656_at	Ral GTPase activating protein, beta subunit (non-catalytic)	Ralgapb	0.74474	1.09808483	0.13414221
1420193_at	keratin 17	Krt17	0.74475	-1.13182704	-0.1878996
1417093_a_at	general transcription factor II H, polypeptide 4	Gtf2h4	0.74476	1.09124989	0.12248368
1435986_x_at	succinate dehydrogenase complex, subunit C, integral membrane	Sdhc	0.74477	-1.08473612	-0.1184977
1446529_at	---	---	0.7448	-1.12305208	-0.1739074
1429334_at	lysozyme G-like 1	Lyg1	0.7448	-1.12543032	-0.1731105
1424465_at	coiled-coil domain containing 58	Ccdc58	0.74481	1.08339369	0.11393959
1430476_at	signal recognition particle 54B	Srp54b	0.74482	1.18555144	0.22055568
1459959_at	---	---	0.74485	-1.12686211	-0.1795748
1454041_at	progesterone immunomodulatory binding factor 1	Pibf1	0.74486	1.15407908	0.19186702
1421652_at	5-hydroxytryptamine (serotonin) receptor 3B	Htr3b	0.74486	1.1248065	0.16767881
1435697_a_at	cytohesin 1 interacting protein	Cytip	0.74488	1.15040621	0.19096381
1426788_a_at	structure specific recognition protein 1	Ssrp1	0.74492	1.07695128	0.10517058
1440004_at	---	---	0.74492	1.12314795	0.16570215
1457494_at	---	---	0.745	1.10343355	0.13735822
1459299_at	myosin IIIB	Myo3b	0.74509	-1.1298635	-0.1932738
1438026_at	zinc finger protein 560	Zfp560	0.74513	-1.10968976	-0.1522084
1430845_at	RIKEN cDNA 2410018L13 gene	2410018L13Rik	0.74516	1.12289868	0.16563828
1446931_at	---	---	0.74522	1.14200689	0.18381952
1429501_s_at	protein phosphatase 1A, magnesium dependent, alpha isoform	Ppm1a	0.74524	-1.12018637	-0.1772326
1422976_x_at	NADH dehydrogenase (ubiquinone) 1 alpha subcomplex, 7 (B14.5	Ndufa7	0.7456	1.07409783	0.10162214
1419646_a_at	myelin basic protein	Mbp	0.74564	1.1410329	0.18145598
1447818_x_at	Ras homolog enriched in brain like 1	Rhebl1	0.74565	-1.09939516	-0.1465457
1442819_at	rhomboid, veinlet-like 2 (Drosophila)	Rhbdl2	0.74566	-1.12747684	-0.1789893
1441755_at	mitogen-activated protein kinase 15	Mapk15	0.74568	-1.11421475	-0.1582617
1419022_a_at	enolase 1, alpha non-neuron /// predicted gene 5506 /// alpha-e	Eno1 /// Gm5506 /// LOC	0.74569	1.04055067	0.05697474
1424928_at	RIKEN cDNA 2210018M11 gene	2210018M11Rik	0.74573	1.09728168	0.13135483
1421078_at	transcription factor 23	Tcf23	0.74573	-1.12223292	-0.1717485
1449128_at	coiled-coil domain containing 43	Ccdc43	0.74576	-1.11204556	-0.1649908
1455964_at	cyclin-dependent kinase 12	Cdk12	0.74576	-1.08882159	-0.1244644
1428583_at	nuclear fragile X mental retardation protein interacting protein 2	Nufip2	0.74578	-1.09511479	-0.1316643
1431369_at	ArfGAP with SH3 domain, ankyrin repeat and PH domain 2	Asap2	0.74589	1.12023022	0.16375778
1455108_at	eukaryotic translation initiation factor 4E member 2	Eif4e2	0.74593	1.10027812	0.13561109
1424187_at	coiled-coil domain containing 80	Ccdc80	0.74597	-1.12660545	-0.1779419
1452742_at	trafficking protein, kinesin binding 1	Trak1	0.74649	1.11853482	0.15778538
1459019_at	shadow of prion protein	Sprn	0.74657	-1.1282052	-0.1798074
1437780_at	Fanconi anemia, complementation group B	Fancb	0.74661	1.12792525	0.16588726
1424005_at	RIKEN cDNA B230219D22 gene	B230219D22Rik	0.74666	-1.08194418	-0.114512
1430701_a_at	RIKEN cDNA 5730528L13 gene	5730528L13Rik	0.7467	1.12547867	0.15952121
1457152_at	---	---	0.74676	-1.10643829	-0.1504155
1416986_a_at	signal-regulatory protein alpha	Sirpa	0.74677	1.13703829	0.17910294
1439127_at	expressed sequence AI314180	AI314180	0.7468	-1.12331408	-0.1696209
1428476_a_at	elaC homolog 2 (E. coli)	Elac2	0.74684	1.08976744	0.12322684
1445485_at	DNA segment, Chr 7, ERATO Doi 187, expressed	D7Ertd187e	0.74687	1.12103771	0.16087939
1422645_at	hemochromatosis	Hfe	0.74687	1.12992435	0.17199884
1422710_a_at	calcium channel, voltage-dependent, T type, alpha 1H subunit	Cacna1h	0.74691	1.11670491	0.15684233
1426200_at	predicted gene 1499 /// predicted gene 1524	Gm1499 /// Gm1524	0.74692	1.13729485	0.17774428
1437389_x_at	KH domain containing, RNA binding, signal transduction associate	Khdrbs1	0.74694	-1.09113363	-0.1310099
1420612_s_at	protein tyrosine phosphatase 4a2	Ptp4a2	0.74698	-1.13076499	-0.2148019
1460047_at	---	---	0.74703	1.13119487	0.17588754
1427104_at	zinc finger protein 612	Zfp612	0.74704	-1.13044565	-0.1880963
1452189_at	WD repeat domain containing 82	Wdr82	0.74705	-1.08069126	-0.1124809
1417977_at	eukaryotic translation initiation factor 4E member 3	Eif4e3	0.74726	1.16092772	0.19659843
1434009_at	glucocorticoid receptor DNA binding factor 1	Grlf1	0.74732	1.09205156	0.12631133
1458798_at	---	---	0.74734	1.12954627	0.17366902
1425418_at	WAP four-disulfide core domain 5	Wfdc5	0.74734	1.11753757	0.15969071
1434570_at	BEN domain containing 3	Bend3	0.74736	1.08718392	0.11911249
1440976_at	RIKEN cDNA 1110035M17 gene	1110035M17Rik	0.74738	-1.1209834	-0.1681525
1422370_at	olfactory receptor 49	Olfr49	0.7474	-1.12987304	-0.1850877
1417006_at	COMM domain containing 4	Commd4	0.74741	1.09571081	0.12689361
1423742_at	RNA binding motif protein 10	Rbm10	0.74742	1.09951192	0.13412651
1422454_at	keratin 13	Krt13	0.74744	1.17591852	0.21272891
1416425_at	peroxisomal biogenesis factor 19	Pex19	0.74747	1.08894168	0.1224496
1420622_a_at	heat shock protein 8	Hspa8	0.74752	-1.13784488	-0.2373787
1448085_at	DNA segment, Chr 5, ERATO Doi 102, expressed	D5Ertd102e	0.74754	1.11568201	0.1576027
1448868_at	SCAN domain-containing 1	Scand1	0.74755	1.1242727	0.15746232
1438484_at	2-aminoethanethiol (cysteamine) dioxygenase	Ado	0.74757	-1.11510399	-0.1636101
1445481_at	expressed sequence AI317158	AI317158	0.74762	1.11788691	0.15840359
1423683_at	cell division cycle associated 4	Cdca4	0.74762	1.08034548	0.11055813
1421129_a_at	ATPase, Ca++ transporting, ubiquitous	Atp2a3	0.74763	-1.09580215	-0.1348594
1421461_at	myeloproliferative leukemia virus oncogene	Mpl	0.74765	-1.12555271	-0.1774646
1446594_at	eukaryotic translation initiation factor 3, subunit I pseudogene	Gm4371	0.74765	-1.11962593	-0.1673595
1430737_at	zinc finger protein 773	Zfp773	0.74766	-1.12212983	-0.16775
1441044_at	RIKEN cDNA 2610015P09 gene	2610015P09Rik	0.74768	-1.10379008	-0.143003
1425024_at	RIKEN cDNA E430018J23 gene	E430018J23Rik	0.74768	1.11880955	0.16171767
1428181_at	electron transferring flavoprotein, beta polypeptide	Etfb	0.74769	1.10248786	0.13366512
1427750_at	predicted gene 5571	Gm5571	0.74769	-1.11399835	-0.1577974
1448820_a_at	eukaryotic translation initiation factor 2, subunit 2 (beta)	Eif2s2	0.74769	-1.14655483	-0.2586692
1427294_a_at	solute carrier family 38, member 10	Slc38a10	0.74769	1.08010716	0.11054625
1416031_s_at	minichromosome maintenance deficient 7 (S. cerevisiae)	Mcm7	0.74769	1.06921907	0.09632665
1435785_at	EH-domain containing 2	Ehd2	0.7477	-1.13488341	-0.1931994
1418030_at	solute carrier organic anion transporter family, member 3a1	Slco3a1	0.74771	-1.13615698	-0.1931547
1438653_x_at	ataxin 10	Atxn10	0.74772	-1.05860687	-0.0826811
1433604_x_at	aldolase A, fructose-bisphosphate	Aldoa	0.74772	-1.06701969	-0.0951935
1453096_x_at	ribosomal protein L27	Rpl27	0.74772	-1.08511774	-0.1288673
1430908_at	RIKEN cDNA 4933429O19 gene	4933429O19Rik	0.74773	1.13698127	0.17348908

1434130_at	lipoma HMGIC fusion partner-like 2	Lhfpl2	0.74774	-1.1291681	-0.187048
1438808_at	transformation related protein 53	Trp53	0.74776	-1.13964951	-0.2104784
1445317_at	---	---	0.74778	1.16635205	0.18926868
1440960_at	---	---	0.74778	1.10455948	0.13965674
1459960_at	RIKEN cDNA 8430419K02 gene	8430419K02Rik	0.74779	1.0787908	0.10885184
1427612_at	defensin beta 9	Defb9	0.74779	1.13904346	0.17689968
1431374_at	RIKEN cDNA 6330407A03 gene	6330407A03Rik	0.74779	-1.1208425	-0.1658008
1430926_at	tetratricopeptide repeat domain 18	Ttc18	0.74779	1.1451153	0.185924
1451530_at	epidermal growth factor receptor	Egfr	0.7478	-1.12965184	-0.1851758
1438637_x_at	splicing factor 3b, subunit 2	Sf3b2	0.74781	-1.08941889	-0.1294027
1453786_at	RIKEN cDNA 4933430H15 gene	4933430H15Rik	0.74781	-1.12858993	-0.1783514
1416665_at	demethyl-Q 7	Coq7	0.74782	1.09295092	0.12414258
1442739_at	zinc finger protein 119b	Zfp119b	0.74783	-1.12267524	-0.1779836
1449704_at	expressed sequence C80171	C80171	0.74784	-1.13263715	-0.1983051
1458665_at	Chloride intracellular channel 1	Clic1	0.74786	-1.12118429	-0.1676154
1448045_at	predicted gene 10658	Gm10658	0.74787	1.22358792	0.2505978
1418458_at	anaphase promoting complex subunit 7	Anapc7	0.74788	1.08098813	0.11130104
1458533_at	RIKEN cDNA 9330169L03 gene	9330169L03Rik	0.74788	-1.0908431	-0.1270751
1423990_at	RAB28, member RAS oncogene family	Rab28	0.74789	1.09128793	0.12506171
1448360_s_at	angel homolog 2 (Drosophila)	Angel2	0.7479	1.08542931	0.1163678
1431974_at	RIKEN cDNA 4930443G12 gene	4930443G12Rik	0.74791	-1.15637798	-0.2421923
1435931_at	---	---	0.74793	-1.17074004	-0.2985069
1428764_at	maternally expressed 3	Meg3	0.74799	-1.12350527	-0.1743138
1437329_at	protein tyrosine phosphatase-like (proline instead of catalytic arg	Ptplb	0.74802	1.12765463	0.16725082
1448407_at	RIKEN cDNA 4632428N05 gene	4632428N05Rik	0.74804	-1.11414589	-0.1608445
1425588_at	protein tyrosine phosphatase, receptor type, J	Ptprj	0.74804	-1.13446743	-0.1988591
1447160_at	non-POU-domain-containing, octamer binding protein	Nono	0.74806	-1.10326443	-0.1478844
1416698_a_at	CDC28 protein kinase 1b	Cks1b	0.74811	-1.0543356	-0.0763823
1452070_at	death effector domain-containing DNA binding protein 2	Dedd2	0.74814	1.10077359	0.13845324
1435009_at	solute carrier family 9 (sodium/hydrogen exchanger), member 6	Slc9a6	0.74816	1.11217154	0.15061509
1429207_at	RIKEN cDNA 5730408K05 gene	5730408K05Rik	0.74826	1.1440379	0.17583055
1442740_at	PR domain containing 5	Prdm5	0.74827	-1.1337737	-0.1902053
1458127_at	---	---	0.74831	-1.12202794	-0.1667707
1444022_at	---	---	0.74833	-1.11867922	-0.1631824
1455068_at	pleckstrin and Sec7 domain containing 4	Psd4	0.74837	-1.1148692	-0.1665722
1431362_a_at	SPARC related modular calcium binding 2	Smoc2	0.74841	1.11764379	0.15644887
1460067_at	chemokine (C-C motif) receptor 2	Ccr2	0.74842	-1.10183516	-0.1401376
1427083_a_at	mitogen-activated protein kinase kinase kinase kinase 5	Map4k5	0.74845	-1.10827699	-0.1492207
1435819_at	---	---	0.74846	-1.13143302	-0.1889744
1428881_at	kinesin light chain 1	Klc1	0.74873	1.1362154	0.17356749
1432230_at	huntingtin interacting protein 1	Hip1	0.74877	-1.12482461	-0.1760231
1436904_at	mediator complex subunit 13	Med13	0.74881	-1.12911732	-0.1793959
1429423_at	RIKEN cDNA 4930518I15 gene	4930518I15Rik	0.74882	1.1535287	0.19407657
1445690_at	---	---	0.74889	1.14922968	0.1826838
1448723_at	retinol dehydrogenase 7	Rdh7	0.74889	1.11038045	0.14387822
1452345_at	leiomodin 2 (cardiac)	Lmod2	0.74917	-1.13552366	-0.2032533
1454465_at	RIKEN cDNA 5830435N06 gene	5830435N06Rik	0.74922	-1.12310592	-0.1735837
1435681_s_at	DEAD (Asp-Glu-Ala-Asp) box polypeptide 49	Ddx49	0.74926	1.10964566	0.14998127
1445727_at	ubiquitin protein ligase E3A	Ube3a	0.74929	-1.11216854	-0.1537415
1455071_at	zinc finger and BTB domain containing 7B	Zbtb7b	0.74932	1.10410634	0.13984356
1439377_x_at	cell division cycle 20 homolog (S. cerevisiae)	Cdc20	0.74933	-1.0658775	-0.0930468
1432649_at	RIKEN cDNA 3300002A11 gene /// hypothetical protein LOC1005	3300002A11Rik /// LOC10	0.74934	-1.12203549	-0.1684767
1446580_at	apolipoprotein O-like	Apool	0.74935	1.12997575	0.17159068
1452856_at	CREB/ATF bZIP transcription factor	Crebzf	0.74936	-1.08574899	-0.1228335
1416846_a_at	PDZ domain containing RING finger 3	Pdzrn3	0.74938	1.13511458	0.17494991
1451976_s_at	CKLF-like MARVEL transmembrane domain containing 1 /// CKLF	Cmtm1 /// Cmtm2a	0.7494	1.11330315	0.14738882
1417446_at	solute carrier family 12, member 4	Slc12a4	0.74941	1.10252835	0.13984165
1453748_a_at	kinesin family member 23	Kif23	0.74943	1.09443073	0.12726738
1436535_at	TROVE domain family, member 2	Trove2	0.74946	-1.10242098	-0.1445294
1440943_at	RIKEN cDNA B230208H17 gene	B230208H17Rik	0.74948	1.11717783	0.15910421
1459702_at	---	---	0.74949	-1.11904754	-0.1667066
1437257_at	WD repeat domain 47	Wdr47	0.74952	-1.13166467	-0.1945063
1416786_at	activin A receptor, type 1	Acvr1	0.74957	1.13594342	0.17188673
1455488_at	HAUS augmin-like complex, subunit 6	Haus6	0.74961	-1.08351185	-0.1167563
1420423_at	T-cell leukemia/lymphoma 1B, 4	Tcl1b4	0.74964	-1.1095167	-0.1501969
1459832_s_at	adaptor-related protein complex AP-1, mu subunit 1	Ap1m1	0.74973	1.08659034	0.11880714
1441723_at	Solute carrier family 22 (organic cation transporter), member 13	Slc22a13	0.7498	1.10407996	0.13818516
1446577_at	phosphodiesterase 4B, cAMP specific	Pde4b	0.74982	1.09894292	0.13373528
1459070_at	---	---	0.74982	-1.11895932	-0.1700572
1458163_at	cDNA sequence BC066028	BC066028	0.74984	1.18659453	0.21455749
1441790_at	---	---	0.74985	1.15453732	0.19429355
1430177_at	ubiquitin-conjugating enzyme E2B, RAD6 homology (S. cerevisiae	Ube2b	0.74987	-1.1313375	-0.1945146
1440120_at	---	---	0.74987	1.16217618	0.1928275
1458648_at	expressed sequence AU042950	AU042950	0.74988	-1.11082569	-0.1549634
1421526_at	tolloid-like 2	Tll2	0.7499	1.12101534	0.16407181
1424966_at	transmembrane protein 40	Tmem40	0.74991	1.12931106	0.16312735
1446687_at	splicing factor 3a, subunit 1	Sf3a1	0.74994	1.17170376	0.20736151
1454103_at	dynein, axonemal, heavy chain 2	Dnahc2	0.74994	-1.13929855	-0.2062602
1437285_at	RIKEN cDNA 1110020G09 gene	1110020G09Rik	0.74995	-1.13852854	-0.2100601
1434998_at	IQ motif containing GTPase activating protein 1	Iqgap1	0.74995	-1.0854228	-0.1190847
1459199_at	---	---	0.74996	-1.12994353	-0.1834512
AFFX-b-ActinMur	actin, beta	Actb	0.74997	-1.0494618	-0.0708185
1440281_at	required for meiotic nuclear division 1 homolog (S. cerevisiae)	Rmnd1	0.74999	1.13180013	0.17411236
1425279_at	PDLIM1 interacting kinase 1 like	Pdik1l	0.75	-1.13413898	-0.1914043
1435964_a_at	TAO kinase 3	Taok3	0.75	1.11879903	0.15931416
1428399_a_at	armadillo repeat containing 9	Armc9	0.75002	1.14819742	0.184054
1447240_at	---	---	0.75005	1.12225672	0.1649784
1458192_at	predicted gene 15834 /// hypothetical LOC100504455	Gm15834 /// LOC100504	0.75005	1.12876028	0.17235757
1431381_at	RIKEN cDNA 3110005L24 gene	3110005L24Rik	0.75006	-1.12265679	-0.1770806
1434842_s_at	UPF3 regulator of nonsense transcripts homolog B (yeast)	Upf3b	0.75007	-1.08416235	-0.1202702
1423764_s_at	mitochondrial ribosomal protein L37	Mrpl37	0.75008	1.09642339	0.12941079
1444505_at	zinc finger, MIZ-type containing 1	Zmiz1	0.75008	-1.12175491	-0.1717393
1420242_at	---	---	0.75009	-1.12203758	-0.1695561
1432426_a_at	ubiquitin-conjugating enzyme E2F (putative)	Ube2f	0.7501	1.08576178	0.11780673

1450610_at	urocortin	Ucn	0.7501	-1.11483271	-0.1574012
1430079_at	RIKEN cDNA 5033406O09 gene	5033406O09Rik	0.75011	1.1601228	0.19820293
1450956_at	stearoyl-coenzyme A desaturase 3	Scd3	0.75011	1.11417189	0.15337523
1431499_at	RIKEN cDNA 4933436F18 gene	4933436F18Rik	0.75011	-1.10697749	-0.1535559
1420051_at	---	---	0.75012	-1.11589333	-0.1593739
1422858_at	thyroid hormone receptor interactor 4	Trip4	0.75012	1.09962139	0.1337006
1456213_x_at	glutaminyl-tRNA synthetase	Qars	0.75013	-1.08917229	-0.1243155
1456995_at	shisa homolog 9 (Xenopus laevis)	Shisa9	0.75014	1.1144857	0.15239541
1434887_at	neuronal calcium sensor 1	Ncs1	0.75014	1.150261	0.18544271
1416768_at	RIKEN cDNA 1110003E01 gene	1110003E01Rik	0.75015	1.08002682	0.11093447
1454277_at	RIKEN cDNA 1700049J03 gene	1700049J03Rik	0.75015	-1.12072975	-0.1664099
1444339_at	---	---	0.75015	1.1108801	0.14245697
1454812_at	RIKEN cDNA 5730601F06 gene	5730601F06Rik	0.75016	1.12676485	0.16687644
1418520_at	trans-golgi network protein	Tgoln1	0.75017	-1.11943087	-0.1680979
1459276_at	RIKEN cDNA A530088E08 gene	A530088E08Rik	0.75017	1.14059851	0.18004487
1456592_at	---	---	0.75018	-1.14953945	-0.2350836
1457636_x_at	---	---	0.75019	1.11893306	0.15529895
1416438_at	poly-U binding splicing factor 60	Puf60	0.7502	1.08452303	0.11386196
1458329_x_at	double homeobox B-like /// predicted gene 10394	Duxbl /// Gm10394	0.7502	-1.10499318	-0.1501967
1434197_at	attractin	Atrn	0.75021	1.12225852	0.16360818
1444103_at	Epithelial stromal interaction 1 (breast)	Epsti1	0.75021	1.11649118	0.15743418
1450414_at	platelet derived growth factor, B polypeptide	Pdgfb	0.75021	1.12326768	0.16281612
1453200_at	retinoic acid induced 1	Rai1	0.75022	1.12345704	0.15952618
1416781_at	PRA1 domain family 2	Praf2	0.75022	1.0966559	0.12735692
1429551_at	RIKEN cDNA 4930579G22 gene	4930579G22Rik	0.75025	1.1291351	0.16963349
1436697_at	---	---	0.75027	1.13374462	0.17534371
1452958_at	aspartate beta-hydroxylase domain containing 2	Asphd2	0.75027	1.10194123	0.13930789
1433280_at	RIKEN cDNA 4933417G07 gene	4933417G07Rik	0.75028	-1.11314674	-0.1560676
1434376_at	CD44 antigen	Cd44	0.75028	1.13364734	0.17424104
1444858_at	---	---	0.7503	-1.12041874	-0.1656257
1433741_at	CD38 antigen	Cd38	0.75033	1.10154235	0.13802031
1422596_at	Na+/K+ transporting ATPase interacting 4	Nkain4	0.75034	1.09712555	0.13339282
1459722_at	Zinc finger, SWIM domain containing 6	Zswim6	0.75037	1.22706997	0.2519842
1422197_at	potassium voltage-gated channel, shaker-related subfamily, mem	Kcna2	0.75038	-1.12059331	-0.1644576
1443263_at	---	---	0.75038	1.14686974	0.18664836
1442136_at	ankyrin repeat and IBR domain containing 1	Ankib1	0.75039	-1.11836523	-0.1690061
1449012_s_at	fibronectin type III domain containing 4	Fndc4	0.7504	-1.12412246	-0.1742065
1434808_at	partner and localizer of BRCA2	Palb2	0.7504	-1.09374311	-0.1295236
1456705_at	---	---	0.7504	1.1177448	0.15338892
1460618_x_at	ring finger protein 19A pseudogene	Gm7444	0.7504	-1.12382113	-0.1703691
1424317_at	solute carrier family 25 (mitochondrial thiamine pyrophosphate ca	Slc25a19	0.75041	1.09667976	0.13267015
1419548_at	karyopherin (importin) alpha 1	Kpna1	0.75041	-1.09787314	-0.1466468
1418796_at	C-type lectin domain family 11, member a	Clec11a	0.75042	1.10739933	0.14591466
1418147_at	transcription factor AP-2, gamma	Tcfap2c	0.75043	1.11975437	0.15655521
1422171_at	prostaglandin D receptor	Ptgdr	0.75044	-1.12122655	-0.1696829
1434131_at	RUN and FYVE domain containing 1	Rufy1	0.75044	1.1334752	0.17186883
1417118_a_at	N(alpha)-acetyltransferase 10, NatA catalytic subunitNalpha acet	Naa10	0.75044	1.0998033	0.13145142
1450665_at	GA repeat binding protein, alpha	Gabpa	0.75044	-1.11342723	-0.1704437
1430421_a_at	transmembrane protein 205	Tmem205	0.75045	1.1293514	0.162609
1421869_at	tripartite motif-containing 44	Trim44	0.75045	-1.11586705	-0.1606265
1424970_at	purine-rich element binding protein G	Purg	0.75046	1.11232396	0.15120557
1458749_at	---	---	0.75046	-1.14872888	-0.2281446
1450767_at	neural precursor cell expressed, developmentally down-regulated	Nedd9	0.75047	1.11801246	0.15889743
1436487_x_at	F-box and WD-40 domain protein 2	Fbxw2	0.75047	1.17241265	0.20958847
1417281_a_at	matrix metallopeptidase 23	Mmp23	0.75048	1.1246678	0.16648603
1418211_at	oculocutaneous albinism II	Oca2	0.75049	-1.12429128	-0.1724111
1420150_at	splA/ryanodine receptor domain and SOCS box containing 1	Spsb1	0.75049	1.16403156	0.19851927
1426598_at	ubiquitously transcribed tetratricopeptide repeat gene, Y chromos	Uty	0.75049	-1.112496	-0.1641598
1441548_at	FERM domain containing 4B	Frmd4b	0.75051	1.11814716	0.15832139
1433846_s_at	family with sequence similarity 175, member B	Fam175b	0.75051	1.08504043	0.11676037
1452086_at	TRM5 tRNA methyltransferase 5 homolog (S. cerevisiae)	Trmt5	0.75051	1.08218357	0.11391888
1437564_at	polymerase (DNA directed), gamma	Polg	0.75052	1.10406653	0.14091916
1423978_at	SH3-binding kinase 1	Sbk1	0.75052	1.08931129	0.12197128
1436180_at	DnaJ (Hsp40) homolog, subfamily C, member 5	Dnajc5	0.75053	1.08541799	0.11752243
1430971_a_at	aquarius	Aqr	0.75056	-1.13536523	-0.2193672
1417670_at	translocase of inner mitochondrial membrane 44	Timm44	0.75056	1.09597459	0.13030199
1419426_s_at	chemokine (C-C motif) ligand 21A (serine) /// chemokine (C-C mo	Ccl21a /// Ccl21b /// Ccl2	0.75058	-1.11675515	-0.1605846
1435465_at	kelch repeat and BTB (POZ) domain containing 11	Kbtbd11	0.75059	1.07993017	0.11020887
1446405_at	MYST histone acetyltransferase (monocytic leukemia) 3	Myst3	0.75059	-1.12501755	-0.1707628
1447730_at	---	---	0.75059	-1.10957026	-0.1510157
1453147_at	polymerase (RNA) III (DNA directed) polypeptide E	Polr3e	0.75059	-1.10325324	-0.1446283
1432224_at	RIKEN cDNA 4831407H17 gene	4831407H17Rik	0.75059	1.12696705	0.16882775
1431594_at	Dipeptidylpeptidase 3	Dpp3	0.75059	-1.11911166	-0.1669899
1416805_at	family with sequence similarity 198, member B	Fam198b	0.75059	1.13549392	0.17330504
1450697_at	solute carrier family 30 (zinc transporter), member 7	Slc30a7	0.7506	1.09872963	0.13573987
1422601_at	serine (or cysteine) peptidase inhibitor, clade B, member 9	Serpinb9	0.75061	1.16059904	0.19968555
1415713_a_at	DEAD (Asp-Glu-Ala-Asp) box polypeptide 24	Ddx24	0.75061	1.08164026	0.11220734
1438477_a_at	methylmalonyl CoA epimerase	Mcee	0.75061	1.1002138	0.1337331
1456104_at	proteasome (prosome, macropain) 26S subunit, non-ATPase, 11	Psmd11	0.75061	-1.12814336	-0.1834965
1419881_x_at	---	---	0.75062	-1.09126226	-0.1267611
1438118_x_at	vimentin	Vim	0.75064	-1.11247828	-0.1752658
1447930_at	bromodomain adjacent to zinc finger domain 1A /// bromodomai	Baz1a /// LOC100505185	0.75065	-1.12437211	-0.1793923
1429489_at	REX1, RNA exonuclease 1 homolog (S. cerevisiae)	Rexo1	0.75065	-1.11167154	-0.1572546
1456871_a_at	PHD finger protein 20-like 1	Phf20l1	0.75066	-1.09920372	-0.1366194
1417708_at	synaptotagmin III	Syt3	0.75067	1.11970537	0.16033116
1424560_at	proline-serine-threonine phosphatase-interacting protein 1	Pstpip1	0.75068	1.11823259	0.15809416
1456123_at	solute carrier family 5 (sodium/glucose cotransporter), member 1	Slc5a12	0.75068	1.10753464	0.14062546
1419527_at	cartilage oligomeric matrix protein	Comp	0.75069	-1.12296498	-0.1692156
1458545_at	Transmembrane protein 57	Tmem57	0.75069	-1.11582243	-0.1583222
1451204_at	scavenger receptor class A, member 5 (putative)	Scara5	0.7507	1.12894667	0.17099266
1459904_at	cDNA sequence BC030870	BC030870	0.7507	1.10661805	0.14589539
1438459_x_at	splicing factor proline/glutamine rich (polypyrimidine tract binding	Sfpq	0.75071	-1.090451	-0.1317166
1450122_at	protein tyrosine phosphatase, receptor type, G	Ptprg	0.75073	1.09735718	0.13333078
1434348_at	fasciculation and elongation protein zeta 2 (zygin II)	Fez2	0.75073	1.11500852	0.15127616

1427520_a_at	myosin, heavy polypeptide 1, skeletal muscle, adult	Myh1	0.75074	-1.09451407	-0.1303438
1439764_s_at	insulin-like growth factor 2 mRNA binding protein 2	Igf2bp2	0.75075	-1.10295152	-0.1498942
1417162_at	transmembrane BAX inhibitor motif containing 1	Tmbim1	0.75075	1.10849807	0.14566497
1426226_at	dual-specificity tyrosine-(Y)-phosphorylation regulated kinase 1a	Dyrk1a	0.75075	1.1001537	0.13516141
1441681_at	exportin, tRNA (nuclear export receptor for tRNAs)	Xpot	0.75075	-1.09247946	-0.128349
1458295_at	cDNA sequence BC038331	BC038331	0.75076	-1.10897413	-0.1509886
1452824_at	DNA segment, Chr 1, Brigham & Women's Genetics 0212 express	D1Bwg0212e	0.75077	1.12487629	0.16081704
1457131_at	furry homolog (Drosophila)	Fry	0.75077	1.11601841	0.15815552
1452726_a_at	PIH1 domain containing 1	Pih1d1	0.75077	1.09593465	0.12976809
1415845_at	synaptotagmin IV	Syt4	0.75078	-1.1157499	-0.1598841
1451280_at	cyclic AMP-regulated phosphoprotein, 21	Arpp21	0.75082	-1.12123959	-0.1679414
1432296_a_at	integrin alpha V	Itgav	0.75082	1.11721937	0.15856111
1417584_at	solute carrier family 11 (proton-coupled divalent metal ion transp	Slc11a2	0.75084	-1.10409323	-0.142959
1427236_a_at	myeloid/lymphoid or mixed-lineage leukemia 5	Mll5	0.75084	1.16191102	0.19381954
1424060_at	nei like 3 (E. coli)	Neil3	0.75087	1.1937464	0.22394352
1437086_at	achaete-scute complex homolog 1 (Drosophila)	Ascl1	0.75087	-1.11137461	-0.1615539
1422669_at	estrogen receptor-binding fragment-associated gene 9	Ebag9	0.75087	1.10356063	0.13897337
1427221_at	solute carrier family 6 (neurotransmitter transporter), member 20	Slc6a20a	0.75088	1.12261881	0.15877571
1449191_at	WAP four-disulfide core domain 12	Wfdc12	0.75088	1.12205778	0.16140501
1456991_at	---	---	0.75089	-1.13035368	-0.1906641
1433242_at	RIKEN cDNA 5830415B17 gene	5830415B17Rik	0.75092	-1.150558	-0.2392091
1432251_at	RIKEN cDNA 4930403O15 gene	4930403O15Rik	0.75092	-1.13884998	-0.2086295
1444923_at	---	---	0.75095	1.14316976	0.18425628
1455736_at	myosin binding protein C, fast-type	Mybpc2	0.75098	1.11371455	0.15344773
1438481_at	PRP4 pre-mRNA processing factor 4 homolog (yeast)	Prpf4	0.75101	1.14590112	0.18391422
1434094_at	transmembrane protein 125	Tmem125	0.75102	-1.11128409	-0.1536208
1451291_at	oligonucleotide/oligosaccharide-binding fold containing 2B	Obfc2b	0.75103	1.0917881	0.12560426
1446660_at	---	---	0.75104	1.08226856	0.11191575
1439437_x_at	carboxypeptidase E	Cpe	0.75106	-1.12686555	-0.1816957
1451866_a_at	hepatocyte growth factor	Hgf	0.75106	1.14438701	0.1822167
1448731_at	interleukin 10 receptor, alpha	Il10ra	0.7511	-1.11958445	-0.1681498
1447202_at	IKBKB interacting protein	Ikbip	0.75112	-1.12750236	-0.1779877
1427867_at	Myosin, heavy polypeptide 1, skeletal muscle, adult	Myh1	0.75112	-1.11683007	-0.1665522
1439401_x_at	protein phosphatase 2, regulatory subunit B (B56), epsilon isoform	Ppp2r5e	0.75114	-1.10057545	-0.1472998
1437745_at	chromodomain helicase DNA binding protein 7	Chd7	0.75115	-1.15073042	-0.2525661
1424887_at	kelch domain containing 4	Klhdc4	0.75115	1.09370067	0.1286514
1455400_at	dimethylarginine dimethylaminohydrolase 1	Ddah1	0.75119	1.12140107	0.15703958
1424659_at	slit homolog 2 (Drosophila)	Slit2	0.75121	1.15318043	0.19154293
1448564_at	calcium and integrin binding 1 (calmyrin)	Cib1	0.75124	1.11460058	0.14550193
1423389_at	MAD homolog 7 (Drosophila)	Smad7	0.75127	-1.09397978	-0.138236
1437907_a_at	tubulin cofactor A	Tbca	0.75129	-1.09445645	-0.1410203
1445249_at	---	---	0.7513	1.09656387	0.12986735
1450473_at	protocadherin 12	Pcdh12	0.75137	1.2646932	0.27841409
1452354_at	RIKEN cDNA 2810459M11 gene	2810459M11Rik	0.75141	-1.13095648	-0.190829
1436091_at	spermatogenesis associated, serine-rich 2-like	Spats2l	0.75142	-1.11303868	-0.1546976
1424355_a_at	transcriptional regulator, SIN3B (yeast)	Sin3b	0.75145	1.08238267	0.11325295
1458524_at	fibronectin type III domain containing 3A	Fndc3a	0.75145	-1.12709767	-0.1811804
1459851_x_at	RIO kinase 1 (yeast)	Riok1	0.75147	-1.09600306	-0.133197
1416241_at	SEC13 homolog (S. cerevisiae)	Sec13	0.75152	1.08170236	0.11096832
1419640_at	purine rich element binding protein B	Purb	0.75153	-1.12365792	-0.2013643
1423160_at	sprouty protein with EVH-1 domain 1, related sequence	Spred1	0.75153	-1.08662677	-0.1215529
1443236_at	RIKEN cDNA B230118H07 gene	B230118H07Rik	0.75156	-1.12939149	-0.181155
1445826_at	ankyrin repeat domain 17	Ankrd17	0.75159	1.09468469	0.12947925
1443390_at	---	---	0.75161	1.12050226	0.15866491
1452779_at	ubiquitin-conjugating enzyme E2Q family-like 1	Ube2ql1	0.75161	-1.10765086	-0.1499489
1438592_at	NIMA (never in mitosis gene a)-related expressed kinase 1	Nek1	0.75162	1.14644675	0.1840701
1456326_at	fibronectin type III domain containing 3C1	Fndc3c1	0.75162	1.12642008	0.16780996
1425027_s_at	SFT2 domain containing 2	Sft2d2	0.75164	1.09615173	0.13188274
1415826_at	ATPase, H+ transporting, lysosomal V1 subunit H	Atp6v1h	0.75165	-1.08828088	-0.1267758
1460104_at	vacuolar protein sorting 4b (yeast)	Vps4b	0.75165	-1.12528342	-0.1837018
1422978_at	cytochrome b-245, beta polypeptide	Cybb	0.75167	-1.12253617	-0.1799558
1423395_at	similar to translin associated protein X /// translin-associated fact	LOC100039215 /// Tsnax	0.75168	1.07915968	0.10965767
1429187_at	transmembrane emp24 protein transport domain containing 7	Tmed7	0.7517	-1.10055914	-0.1484813
1456733_x_at	serine (or cysteine) peptidase inhibitor, clade H, member 1	Serpinh1	0.7517	1.09649977	0.13057909
1434514_at	RNA binding motif protein 15	Rbm15	0.75176	1.09110419	0.12574604
1419717_at	sema domain, immunoglobulin domain (Ig), short basic domain, s	Sema3e	0.75178	-1.11511875	-0.1591936
1451848_a_at	crystallin, zeta	Cryz	0.75183	1.08166081	0.11277788
1426097_a_at	coiled-coil domain containing 106	Ccdc106	0.75187	1.11632547	0.15478954
1429411_a_at	enhancer of yellow 2 homolog (Drosophila)	Eny2	0.75189	-1.10372221	-0.1579229
1434264_at	ankyrin 2, brain	Ank2	0.75189	1.19743819	0.23192358
1420737_at	polyamine modulated factor 1 binding protein 1	Pmfbp1	0.75194	-1.11413296	-0.1579721
1437023_at	zinc finger protein 12	Zfp12	0.75194	1.09584505	0.13190385
1448739_x_at	ribosomal protein S18	Rps18	0.75195	1.04067634	0.05686466
1454943_a_at	PAX interacting (with transcription-activation domain) protein 1	Paxip1	0.75195	-1.09383681	-0.1318792
1446986_at	scratch homolog 2, zinc finger protein (Drosophila)	Scrt2	0.75195	-1.09694782	-0.139583
1457785_at	---	---	0.75195	1.15383041	0.19284423
1458386_at	RIKEN cDNA C030009J22 gene	C030009J22Rik	0.75195	1.13007064	0.17349138
1423104_at	insulin receptor substrate 1	Irs1	0.75197	1.13718273	0.1804612
1441190_at	actin related protein 2/3 complex, subunit 5-like	Arpc5l	0.75198	-1.1291799	-0.1799279
1451213_at	peroxisomal biogenesis factor 11 beta	Pex11b	0.75198	1.11833175	0.15722132
1458446_at	RIKEN cDNA 3222401L13 gene	3222401L13Rik	0.75198	1.12860242	0.17095304
1453852_at	DEAD (Asp-Glu-Ala-Asp) box polypeptide 50	Ddx50	0.75199	1.13637754	0.17669376
1435542_s_at	CTTNBP2 N-terminal like	Cttnbp2nl	0.752	-1.09363982	-0.1293538
1426078_a_at	G protein-coupled receptor 108	Gpr108	0.752	1.09859588	0.13151849
1441328_at	---	---	0.75204	1.14300008	0.18247428
1427456_at	WD repeat and FYVE domain containing 3	Wdfy3	0.75204	-1.11925675	-0.1792408
1460273_a_at	NLR family, apoptosis inhibitory protein 2	Naip2	0.75205	-1.11982784	-0.1656075
1431761_at	ectonucleoside triphosphate diphosphohydrolase 4	Entpd4	0.75206	-1.14067415	-0.2126825
1450277_at	arginine vasopressin receptor 2	Avpr2	0.75207	-1.11425544	-0.1584061
1418876_at	forkhead box D1	Foxd1	0.75207	1.12150043	0.16316835
1432334_at	cylicin, basic protein of sperm head cytoskeleton 2	Cylc2	0.75208	1.09259676	0.1275304
1450018_s_at	solute carrier family 25, member 30	Slc25a30	0.75208	1.1359492	0.17111665
1432660_at	RIKEN cDNA 5830420C07 gene	5830420C07Rik	0.75209	1.11694561	0.15702641
1456236_s_at	COMM domain containing 10	Commd10	0.7521	-1.09307951	-0.1360791

1455298_at	---	---	0.7521	1.12527452	0.16462604
1415965_at	stearoyl-Coenzyme A desaturase 1	Scd1	0.75211	1.09450595	0.12841018
1425515_at	phosphatidylinositol 3-kinase, regulatory subunit, polypeptide 1 (p	Pik3r1	0.75212	1.11997408	0.16206306
1437536_at	fukutin related protein	Fkrp	0.75212	1.15294924	0.18409848
1419582_at	cytochrome P450, family 2, subfamily c, polypeptide 55	Cyp2c55	0.75213	-1.123934	-0.1706345
1452783_at	fibronectin type III domain containing 3B	Fndc3b	0.75214	1.10875568	0.14694105
1433434_at	expressed sequence AW551984	AW551984	0.75215	1.15839593	0.19815775
1444310_at	polymerase (RNA) III (DNA directed) polypeptide A	Polr3a	0.75215	-1.12153423	-0.1722806
1442504_at	---	---	0.75215	-1.11349636	-0.1584067
1430161_at	dihydrolipoamide S-succinyltransferase (E2 component of 2-oxo-g	Dlst	0.75216	1.14792751	0.18688549
1417195_at	WW, C2 and coiled-coil domain containing 2	Wwc2	0.75216	-1.09552337	-0.1379377
1422441_x_at	cyclin-dependent kinase 4	Cdk4	0.75217	1.0690668	0.09545661
1430470_at	RIKEN cDNA 5730411F24 gene	5730411F24Rik	0.75217	1.1380211	0.1771667
1436224_at	kinesin family member 1C	Kif1c	0.75217	-1.12216263	-0.17327
1440496_at	Sorting and assembly machinery component 50 homolog (S. cere	Samm50	0.75218	1.12335469	0.16727385
1418248_at	galactosidase, alpha	Gla	0.75218	1.1032839	0.13709327
1418967_a_at	suppression of tumorigenicity 7	St7	0.75218	1.15159095	0.19133608
1429688_at	aryl hydrocarbon receptor nuclear translocator-like 2	Arntl2	0.75219	1.11436844	0.15322309
1451298_at	pleckstrin homology domain containing, family H (with MyTH4 do	Plekhh3	0.7522	1.14943567	0.18677499
1435636_at	RIKEN cDNA 2310051F07 gene	2310051F07Rik	0.7522	-1.09020086	-0.1273822
1425205_at	DEAD (Asp-Glu-Ala-Asp) box polypeptide 19b	Ddx19b	0.7522	-1.12758088	-0.1905671
1453475_at	RIKEN cDNA 4930445K14 gene	4930445K14Rik	0.75222	1.11485304	0.15428123
1452166_a_at	keratin 10	Krt10	0.75222	1.10377633	0.14230505
1453425_at	coiled-coil domain containing 83	Ccdc83	0.75223	-1.11371729	-0.1603919
1455231_s_at	adenomatosis polyposis coli 2	Apc2	0.75223	1.14399332	0.18480306
1425169_at	protease, serine, 42	Prss42	0.75223	-1.10729402	-0.1498124
1437779_at	forkhead box H1	Foxh1	0.75224	1.11443314	0.14686815
1443245_at	SET domain, bifurcated 1 pseudogene	Gm4349	0.75224	1.11076157	0.15061921
1429397_a_at	family with sequence similarity 71, member D	Fam71d	0.75224	1.1280916	0.16652879
1447032_at	---	---	0.75225	1.13237303	0.17190938
1450881_s_at	G protein-coupled receptor 137B	Gpr137b	0.75225	1.12697882	0.16410935
1447702_x_at	immunoglobulin superfamily, member 1	Igsf1	0.75225	1.08051987	0.11055665
1432819_at	proline-rich coiled-coil 1	Prrc1	0.75226	-1.11547508	-0.1598774
1420774_a_at	RIKEN cDNA 4930583H14 gene	4930583H14Rik	0.75227	1.12398943	0.15763267
1423524_at	microtubule associated serine/threonine kinase-like	Mastl	0.75227	-1.10860521	-0.1584164
1452591_a_at	mitotic spindle organizing protein 2	Mzt2	0.75228	1.08342093	0.11437375
1417481_at	receptor (calcitonin) activity modifying protein 1	Ramp1	0.75229	-1.11987692	-0.1639486
1439577_at	---	---	0.7523	-1.11998591	-0.1669245
1431098_at	CAP-GLY domain containing linker protein 1	Clip1	0.75231	1.13984937	0.17983294
1436436_at	cornichon homolog 4 (Drosophila)	Cnih4	0.75231	1.10568165	0.14368978
1455552_at	small nuclear RNA activating complex, polypeptide 4	Snapc4	0.75231	1.08461294	0.11673888
1418826_at	membrane-spanning 4-domains, subfamily A, member 6B	Ms4a6b	0.75233	-1.1230297	-0.171096
1439493_at	zinc finger protein 827	Zfp827	0.75233	1.10913693	0.14705281
1430636_at	protein phosphatase 4, regulatory subunit 1-like, pseudogene	Ppp4r1l-ps	0.75233	1.11735168	0.15624488
1433893_s_at	sperm associated antigen 5	Spag5	0.75235	1.08663833	0.11980513
1444303_at	hypothetical LOC100503986	LOC100503986	0.75235	-1.11942179	-0.1659372
1444489_at	Solute carrier family 25 (mitochondrial carrier, Aralar), member 12	Slc25a12	0.75237	-1.11111498	-0.1540129
1429836_at	hypothetical LOC100503775 /// UDP-glucose glycoprotein glucos	LOC100503775 /// Uggt2	0.75237	-1.12339152	-0.1745135
1416564_at	SRY-box containing gene 7	Sox7	0.75237	-1.10559543	-0.146326
1441147_at	DNA segment, Chr 3, ERATO Doi 229, expressed	D3Ertd229e	0.75238	-1.11410811	-0.1586507
1431164_at	Ras-related GTP binding D	Rragd	0.75238	1.14806704	0.19145337
1441616_at	---	---	0.75239	-1.09077487	-0.1279934
1448715_x_at	cDNA sequence BC094435 /// CCR4 carbon catabolite repression	BC094435 /// Ccrn4l /// C	0.7524	1.06262209	0.08691536
1455456_a_at	translocase of inner mitochondrial membrane 50 homolog (yeast	Timm50	0.75242	1.08412592	0.11482189
1423852_at	shisa homolog 2 (Xenopus laevis)	Shisa2	0.75242	1.11439627	0.1518161
1428913_at	DNL-type zinc finger	Dnlz	0.75243	1.10348952	0.13673195
1419984_s_at	zinc finger protein 644	Zfp644	0.75243	-1.08249199	-0.1158207
1439268_x_at	eukaryotic translation initiation factor 3, subunit E	Eif3e	0.75244	-1.07299411	-0.1049357
1428063_at	ankyrin repeat domain 46	Ankrd46	0.75244	1.08786079	0.12143206
1441896_x_at	---	---	0.75244	-1.12122984	-0.1671076
1428161_a_at	coiled-coil-helix-coiled-coil-helix domain containing 2	Chchd2	0.75246	-1.0446159	-0.0638025
1438615_x_at	RIKEN cDNA 2010317E24 gene	2010317E24Rik	0.75247	1.11257256	0.15314458
1434153_at	src homology 2 domain-containing transforming protein B	Shb	0.75248	1.10540628	0.13985341
1442630_at	---	---	0.75249	1.13540644	0.17183899
1433667_at	leucine-rich repeat LGI family, member 3	Lgi3	0.75249	-1.11594782	-0.1644783
1418399_at	potassium channel tetramerisation domain containing 9	Kctd9	0.75249	1.10166509	0.13750006
1456659_at	hypothetical LOC552902	LOC552902	0.75254	1.14357587	0.18479249
1449774_at	---	---	0.75256	-1.09003788	-0.1244084
1437749_s_at	mitochondrial ribosomal protein L9	Mrpl9	0.75257	-1.09535807	-0.1419549
1435203_at	mannosidase 2, alpha 2	Man2a2	0.75263	1.12009052	0.15923805
1451086_s_at	RAS-related C3 botulinum substrate 1	Rac1	0.75263	1.07244062	0.09922364
1437586_at	CCR4-NOT transcription complex, subunit 4	Cnot4	0.75264	1.08876315	0.12205584
1422006_at	eukaryotic translation initiation factor 2-alpha kinase 2	Eif2ak2	0.75268	1.12167394	0.15948331
1421933_at	chromobox homolog 5 (Drosophila HP1a)	Cbx5	0.7527	-1.10888684	-0.1577814
1446442_at	RNA (guanine-7-) methyltransferase	Rnmt	0.7527	-1.14717992	-0.2267338
1427586_at	sema domain, immunoglobulin domain (Ig), transmembrane dom	Sema4b	0.75273	1.10545218	0.14461225
1457026_at	lipase, member H	Liph	0.75276	1.09574419	0.12824967
1452257_at	3-hydroxybutyrate dehydrogenase, type 1	Bdh1	0.75276	1.09300132	0.12705296
1431036_a_at	transmembrane emp24 domain trafficking protein 2	Tmed2	0.75279	1.07990266	0.11077926
1450997_at	serine/threonine kinase 17b (apoptosis-inducing)	Stk17b	0.7528	1.10048015	0.13604741
1440114_x_at	KN motif and ankyrin repeat domains 3	Kank3	0.75281	1.10907656	0.14851315
1442536_at	RIKEN cDNA 6430590A07 gene	6430590A07Rik	0.75283	1.12173288	0.1607001
1423783_at	torsin family 2, member A	Tor2a	0.75285	1.1014339	0.1353546
1415676_a_at	proteasome (prosome, macropain) subunit, beta type 5	Psmb5	0.75287	1.06871692	0.09563267
1426668_at	solute carrier family 30 (zinc transporter), member 9	Slc30a9	0.75287	1.12873561	0.16208149
1453308_at	RIKEN cDNA 8430429K09 gene /// hypothetical LOC100503029	8430429K09Rik /// LOC10	0.75288	1.13769551	0.17773383
1416135_at	apurinic/apyrimidinic endonuclease 1	Apex1	0.7529	1.06900007	0.09553499
1425340_a_at	protein tyrosine phosphatase, receptor type, A	Ptpra	0.75292	1.09256205	0.12714803
1441247_at	---	---	0.75293	-1.12742106	-0.1825522
1438458_a_at	splicing factor proline/glutamine rich (polypyrimidine tract binding	Sfpq	0.75294	-1.11545182	-0.1836901
1431661_at	RIKEN cDNA 4930562A09 gene	4930562A09Rik	0.75298	-1.10794542	-0.1679625
1449773_s_at	growth arrest and DNA-damage-inducible 45 beta	Gadd45b	0.75299	1.11335822	0.14886372
1423851_a_at	shisa homolog 2 (Xenopus laevis)	Shisa2	0.753	-1.13168116	-0.1962298
1419014_at	Rhesus blood group-associated A glycoprotein	Rhag	0.753	1.12348606	0.15754541

1449673_s_at	Tu translation elongation factor, mitochondrial	Tufm	0.75301	-1.11910323	-0.1644606
1417803_at	RIKEN cDNA 1110032A04 gene	1110032A04Rik	0.75309	-1.09051735	-0.1254325
1454208_at	RIKEN cDNA 4930483O08 gene	4930483O08Rik	0.75309	-1.14287166	-0.2178459
1416806_at	ferredoxin reductase	Fdxr	0.75309	1.09639117	0.13258188
1456950_at	Alstrom syndrome 1 homolog (human)	Alms1	0.7531	1.13457098	0.17381345
1435341_at	peptidyl-prolyl isomerase G (cyclophilin G)	Ppig	0.75312	-1.08406066	-0.1174233
1453958_at	RIKEN cDNA 2610203C22 gene	2610203C22Rik	0.75317	-1.11915255	-0.1646513
1454670_at	arginine glutamic acid dipeptide (RE) repeats	Rere	0.75319	1.10850354	0.14211055
1450260_at	gastrin releasing peptide receptor	Grpr	0.75322	-1.13540127	-0.1909456
1449387_at	keratin 33A	Krt33a	0.75322	-1.12736131	-0.1862951
1456009_x_at	topoisomerase (DNA) III beta	Top3b	0.75334	-1.0871235	-0.122987
1417415_at	solute carrier family 6 (neurotransmitter transporter, dopamine), r	Slc6a3	0.75341	1.12081193	0.1636769
1437739_a_at	MEF2 activating motif and SAP domain containing transcriptional	Mamstr	0.75361	-1.14479983	-0.2240043
1438053_at	Trk-fused gene	Tfg	0.75362	-1.11253726	-0.1551321
1447069_at	---	---	0.75367	-1.10967849	-0.1511126
1454971_x_at	TSC22 domain family, member 1	Tsc22d1	0.75369	1.05130266	0.0719651
1417209_at	SERTA domain containing 2	Sertad2	0.75407	1.08553981	0.11774303
1448189_a_at	flightless I homolog (Drosophila)	Flii	0.75415	1.0891893	0.12155665
1421562_at	CD209c antigen	Cd209c	0.75457	-1.11581925	-0.1597727
1448986_x_at	deoxyribonuclease II alpha	Dnase2a	0.75459	-1.13310148	-0.2123117
1439079_a_at	Erbb2 interacting protein	Erbb2ip	0.75462	1.22907038	0.25097678
1444260_at	---	---	0.75482	1.14974095	0.18615238
1448256_at	golgi SNAP receptor complex member 1	Gosr1	0.75493	1.11280483	0.14839185
1458838_at	RIKEN cDNA 4732468M13 gene	4732468M13Rik	0.75495	-1.08192721	-0.1171873
1452154_at	isoleucine-tRNA synthetase	Iars	0.75499	-1.08822778	-0.1288021
1451675_a_at	aminolevulinic acid synthase 2, erythroid	Alas2	0.755	-1.1540829	-0.2454403
1443965_at	---	---	0.755	-1.12035678	-0.1648405
1454882_at	l(3)mbt-like 3 (Drosophila)	L3mbtl3	0.75501	-1.1437031	-0.221794
1433425_at	RIKEN cDNA 1700101I19 gene	1700101I19Rik	0.75502	-1.10158029	-0.1399963
1453976_at	RIKEN cDNA 4432414F05 gene	4432414F05Rik	0.75503	-1.12225714	-0.1759884
1427084_a_at	mitogen-activated protein kinase kinase kinase kinase 5	Map4k5	0.75503	1.10477928	0.14049876
1436689_a_at	aldehyde dehydrogenase 9, subfamily A1	Aldh9a1	0.75506	-1.0849001	-0.1213603
1440229_at	RIKEN cDNA 2310034G01 gene	2310034G01Rik	0.75507	1.13461703	0.1741839
1416328_a_at	ATPase, H+ transporting, lysosomal V0 subunit E	Atp6v0e	0.75509	1.08346268	0.11293362
1449111_a_at	growth factor receptor bound protein 2	Grb2	0.7551	-1.08577384	-0.123293
1435158_at	RNA binding motif protein 12B	Rbm12b	0.75512	-1.11371117	-0.162999
1450334_at	interleukin 21	Il21	0.75512	1.11347257	0.15474253
1451174_at	leucine rich repeat containing 33	Lrrc33	0.75512	1.11468973	0.15585097
1438271_at	LIM domain containing preferred translocation partner in lipoma	Lpp	0.75513	1.11709062	0.15829143
1421221_at	beta-carotene oxygenase 2	Bco2	0.75513	-1.11666219	-0.1604572
1441038_at	---	---	0.75516	1.12718299	0.16863095
1422051_a_at	gamma-aminobutyric acid (GABA) B receptor, 1	Gabbr1	0.75531	-1.11928749	-0.1749111
1434544_at	bolA-like 2 (E. coli)	Bola2	0.75533	1.11342962	0.14308242
1432964_at	RIKEN cDNA 2410049M19 gene	2410049M19Rik	0.75533	-1.12452185	-0.1733949
1439896_at	LIM motif-containing protein kinase 2	Limk2	0.75533	-1.10812772	-0.1497044
1432465_at	RIKEN cDNA 4933414I06 gene	4933414I06Rik	0.75533	-1.1268069	-0.1784001
1425716_s_at	BCL2-antagonist/killer 1	Bak1	0.75535	-1.13871576	-0.2312572
1434020_at	PDGFA associated protein 1	Pdap1	0.7554	-1.1223132	-0.2017568
1432398_at	RIKEN cDNA 1700084J12 gene	1700084J12Rik	0.7554	-1.12480981	-0.175928
1432136_s_at	zinc finger, DHHC domain containing 4	Zdhhc4	0.75542	1.09147066	0.12574299
1458099_at	---	---	0.75545	1.15089287	0.18844208
1442554_s_at	kalirin, RhoGEF kinase	Kalrn	0.75546	-1.10508303	-0.1495881
1424201_a_at	SEH1-like (S. cerevisiae	Seh1l	0.75547	-1.0818883	-0.1166837
1449952_s_at	protease, serine, 30	Prss30	0.75554	-1.11828609	-0.1675048
1419366_at	zinc finger, matrin type 5	Zmat5	0.75554	1.11479422	0.14798239
1450134_at	lysyl oxidase-like 4	Loxl4	0.75555	1.12903153	0.16941808
1438346_at	RIKEN cDNA 4930525G20 gene	4930525G20Rik	0.75559	1.10375492	0.13996487
1417731_at	polyglutamine binding protein 1	Pqbp1	0.7556	1.09298015	0.12429063
1452927_x_at	triosephosphate isomerase 1	Tpi1	0.75564	1.06042905	0.0844794
1423828_at	fatty acid synthase	Fasn	0.75567	1.07221795	0.10057971
1443784_at	RIKEN cDNA A230046K03 gene	A230046K03Rik	0.75575	1.09260126	0.12618779
1417540_at	E74-like factor 1	Elf1	0.75577	-1.11517421	-0.1717319
1459497_at	---	---	0.75579	1.15561826	0.19135591
1460241_a_at	ST3 beta-galactoside alpha-2,3-sialyltransferase 5	St3gal5	0.75579	-1.13759205	-0.206346
1455449_at	RIKEN cDNA 2010107G12 gene	2010107G12Rik	0.75583	1.12442798	0.16523112
1428934_at	CKLF-like MARVEL transmembrane domain containing 2B	Cmtm2b	0.75583	-1.12771233	-0.1846717
1423921_at	integrator complex subunit 3	Ints3	0.75583	1.11444178	0.14707668
1417217_at	melanoma antigen, family L, 2	Magel2	0.75584	-1.13603662	-0.2070956
1433624_at	HLA-B associated transcript 2-like	Bat2l	0.75587	1.07857524	0.10824681
1451957_at	interleukin 1 family, member 10	Il1f10	0.75591	1.12574283	0.16805337
1428104_at	TPX2, microtubule-associated protein homolog (Xenopus laevis)	Tpx2	0.75592	1.07974668	0.10996102
1450261_a_at	solute carrier family 10 (sodium/bile acid cotransporter family), m	Slc10a1	0.75592	-1.13397221	-0.2031175
1433517_at	myeloma overexpressed 2	Myeov2	0.75593	1.17912995	0.21171285
1455106_a_at	creatine kinase, brain	Ckb	0.75594	-1.08340302	-0.1174236
1458966_at	expressed sequence C80278	C80278	0.75598	-1.1085674	-0.1519735
1429255_at	RIKEN cDNA 2010007H12 gene	2010007H12Rik	0.75598	1.09560627	0.12825461
1437865_at	spermatogenesis associated 13	Spata13	0.75598	1.13344765	0.16872408
1452363_a_at	ATPase, Ca++ transporting, cardiac muscle, slow twitch 2	Atp2a2	0.75604	1.0912896	0.12505287
1424144_at	chromatin licensing and DNA replication factor 1	Cdt1	0.75608	1.07702921	0.10688484
1444966_at	---	---	0.75609	-1.11492293	-0.157986
1435400_at	EF-hand domain family, member A2	Efha2	0.75609	-1.17303617	-0.3043991
1421396_at	proprotein convertase subtilisin/kexin type 1	Pcsk1	0.75616	1.11863868	0.15945712
1427810_at	thymidylate synthase	Tyms	0.75618	1.206048	0.23311035
1428771_at	kelch domain containing 10	Klhdc10	0.75618	1.08783881	0.12107053
1457092_at	family with sequence similarity 19, member A1	Fam19a1	0.75621	-1.10588615	-0.1452089
1417661_at	RAD52 motif 1	Rdm1	0.75625	1.08579342	0.11606002
1450867_at	mitochondrial ribosomal protein L17	Mrpl17	0.7563	1.12801261	0.16920741
1419842_at	predicted gene 2682	Gm2682	0.75631	-1.13775878	-0.2086605
1449189_at	cDNA sequence BC003266	BC003266	0.75632	1.09739154	0.12990606
1459579_at	calcium channel, voltage-dependent, gamma subunit 8	Cacng8	0.75633	-1.13272663	-0.1955384
1456510_x_at	HIG1 domain family, member 1C /// methyltransferase like 7A2	Higd1c /// Mettl7a2	0.75639	1.13458324	0.1714115
1424735_at	solute carrier family 25 (mitochondrial carrier, phosphate carrier),	Slc25a25	0.75697	1.0977651	0.13266993
1422717_at	acid phosphatase 1, soluble	Acp1	0.75703	1.10118907	0.13649875
1417995_at	protein tyrosine phosphatase, non-receptor type 22 (lymphoid)	Ptpn22	0.75736	-1.13731972	-0.2021515

1434619_at	regulatory associated protein of MTOR, complex 1	Rptor	0.75778	1.09209357	0.12439142
1444158_at	lysine (K)-specific demethylase 5C	Kdm5c	0.75814	-1.08539937	-0.1193423
1451019_at	cathepsin F	Ctsf	0.75823	1.10240544	0.13882542
1434251_at	CCR4-NOT transcription complex, subunit 1	Cnot1	0.75831	1.07323655	0.10172135
1460012_at	WAP four-disulfide core domain 3	Wfdc3	0.75832	-1.11773866	-0.1637966
1419968_at	expressed sequence C77370	C77370	0.75843	-1.12326476	-0.1785561
1431162_a_at	enabled homolog (Drosophila)	Enah	0.75844	-1.16473673	-0.3509802
1446544_at	---	---	0.75847	-1.11221592	-0.1547634
1438287_x_at	DEAD (Asp-Glu-Ala-Asp) box polypeptide 39	Ddx39	0.75859	-1.10831897	-0.1498482
1453188_at	RIKEN cDNA 6230424C14 gene	6230424C14Rik	0.75861	1.12033269	0.16243611
1453719_at	RIKEN cDNA 4930506C21 gene	4930506C21Rik	0.75867	1.1130693	0.15399446
1434262_at	---	---	0.75869	1.11903437	0.1587255
1432331_a_at	paired related homeobox 2	Prrx2	0.75871	1.11485976	0.15316683
1424865_at	peptide YY	Pyy	0.75875	1.13876221	0.17613206
1419653_a_at	DEAD (Asp-Glu-Ala-Asp) box polypeptide 5	Ddx5	0.75877	-1.07901553	-0.1162753
1417940_s_at	RAD51 associated protein 1	Rad51ap1	0.75879	1.09797525	0.133741
1457923_at	---	---	0.7588	-1.11913861	-0.1644972
1451096_at	NADH dehydrogenase (ubiquinone) Fe-S protein 2	Ndufs2	0.7588	1.07509184	0.10439067
1434136_at	transmembrane protein 229A	Tmem229a	0.75883	1.12832571	0.16380921
1451165_at	limb region 1 like	Lmbr1l	0.75885	1.09909969	0.13513526
1457790_at	ankyrin repeat and SOCS box-containing 3	Asb3	0.75885	-1.11386724	-0.1592747
1416833_at	kidney expressed gene 1	Keg1	0.75887	-1.11167361	-0.1549788
1457770_at	solute carrier family 39 (zinc transporter), member 14	Slc39a14	0.7589	-1.13996706	-0.2142246
1416502_a_at	prolactin regulatory element binding	Preb	0.7589	1.07887774	0.10880341
1417881_at	solute carrier family 39 (zinc transporter), member 3	Slc39a3	0.75892	-1.11818025	-0.1641352
1436795_at	consortin, connexin sorting protein	Cnst	0.75895	1.1081414	0.14463417
1444001_at	---	---	0.75896	-1.12996193	-0.1948328
1444491_at	Neuregulin 2	Nrg2	0.75896	-1.12076459	-0.1669145
1444573_at	Lemur tyrosine kinase 3	Lmtk3	0.75902	1.13816264	0.177901
1445663_at	---	---	0.75903	1.1129063	0.14884134
1435274_at	speckle-type POZ protein-like	Spopl	0.75905	-1.10843182	-0.153145
1460571_at	Dicer1, Dcr-1 homolog (Drosophila)	Dicer1	0.75907	1.09191653	0.12516324
1453105_at	zinc finger protein 263	Zfp263	0.75907	-1.09792616	-0.1375277
1435243_at	zinc finger protein 746	Zfp746	0.75908	1.08991076	0.12351273
1438640_x_at	phosphoglycerate kinase 1	Pgk1	0.75909	1.05912005	0.08245064
1434387_at	integrin alpha FG-GAP repeat containing 3	Itfg3	0.75909	1.09690342	0.13307154
1458500_at	expressed sequence AU021034	AU021034	0.7591	-1.12519165	-0.1791114
1453646_at	RIKEN cDNA 3110005L21 gene	3110005L21Rik	0.7591	1.10468586	0.13789199
1443924_at	---	---	0.75911	-1.08848527	-0.123466
1459763_at	---	---	0.75912	-1.09606005	-0.1396244
1428258_at	RIKEN cDNA 2010107E04 gene	2010107E04Rik	0.75912	1.09838904	0.12801906
1451262_a_at	aminoacyl tRNA synthetase complex-interacting multifunctional p	Aimp2	0.75913	1.07411615	0.10246261
1443503_at	inositol hexaphosphate kinase 3	Ip6k3	0.75915	-1.11910591	-0.1707045
1448063_at	IQ motif and Sec7 domain 2	Iqsec2	0.75916	-1.1169491	-0.1653285
1431003_a_at	RIKEN cDNA 2610002J02 gene	2610002J02Rik	0.75916	1.10082511	0.13468134
1423099_a_at	methyltransferase like 3	Mettl3	0.75917	1.07908632	0.10867429
1438530_at	tissue factor pathway inhibitor	Tfpi	0.75917	1.14543015	0.18076213
1420486_at	nucleolar protein 7	Nol7	0.75918	1.08367016	0.1143823
1445913_at	RIKEN cDNA A730090H04 gene	A730090H04Rik	0.75918	1.12058897	0.16137636
1418859_at	regulatory factor X-associated protein	Rfxap	0.75919	1.11208243	0.14745124
1453932_at	ATP/GTP binding protein-like 4	Agbl4	0.7592	-1.12687623	-0.1837512
1426757_at	adenosine monophosphate deaminase 2	Ampd2	0.7592	1.10092898	0.13386828
1445370_at	---	---	0.7592	1.12261561	0.16397167
1428589_at	mitochondrial ribosomal protein L41	Mrpl41	0.75922	1.09241825	0.12377341
1453995_a_at	TRM2 tRNA methyltransferase 2 homolog A (S. cerevisiae)	Trmt2a	0.75924	1.08649862	0.11935422
1433159_at	kinesin family member 13B	Kif13b	0.75924	-1.11830314	-0.1662219
1440090_at	Solute carrier family 25, member 27	Slc25a27	0.75925	1.10608201	0.14454155
1454675_at	thyroid hormone receptor alpha	Thra	0.75926	1.09011652	0.12446898
1442655_at	DNA methyltransferase 3B	Dnmt3b	0.75929	-1.12902059	-0.1973307
1437319_at	unc-13 homolog C (C. elegans)	Unc13c	0.75929	1.09365468	0.1287827
1432931_at	neurexin III	Nrxn3	0.7593	-1.11558509	-0.1651186
1433477_at	active BCR-related gene	Abr	0.7593	1.11041029	0.1443733
1439780_at	ribosomal protein L7-like 1	Rpl7l1	0.75933	1.08992136	0.12355851
1430720_at	Y box protein 1	Ybx1	0.75933	-1.129486	-0.1875589
1450690_at	RAN binding protein 2	Ranbp2	0.75934	-1.08572037	-0.1233055
1431774_a_at	LYR motif containing 1	Lyrm1	0.75936	1.11551922	0.15499802
1448174_at	cullin 1	Cul1	0.75936	1.0846964	0.11718104
1459917_at	gametogenetin binding protein 2	Ggnbp2	0.75937	1.22457576	0.2474758
1434244_x_at	TRM2 tRNA methyltransferase 2 homolog A (S. cerevisiae)	Trmt2a	0.75937	1.08695335	0.12017932
1448776_at	growth arrest and DNA-damage-inducible, gamma interacting pro	Gadd45gip1	0.75938	1.10115627	0.13672544
1454332_at	RIKEN cDNA 6030442H21 gene	6030442H21Rik	0.75939	1.095529	0.13098732
1446863_at	---	---	0.7594	1.12878884	0.16834552
1420302_at	expressed sequence AA414903	AA414903	0.75941	-1.12205542	-0.1803915
1420225_at	---	---	0.75944	1.07551439	0.10472333
1434391_at	expressed sequence AI503316	AI503316	0.75944	-1.09940206	-0.1415087
1452361_at	ring finger protein 20	Rnf20	0.75946	1.11620713	0.15393713
1442105_at	SH3 domain containing ring finger 3	Sh3rf3	0.75946	-1.13220835	-0.1969102
1442380_at	lipocalin 4	Lcn4	0.75947	-1.12462563	-0.1834264
1460169_a_at	cyclin-dependent kinase 16	Cdk16	0.75947	1.08992983	0.12148304
1424525_at	gastrin releasing peptide	Grp	0.75949	-1.11851819	-0.1641742
1450173_at	receptor (TNFRSF)-interacting serine-threonine kinase 2	Ripk2	0.7595	1.1034788	0.13878144
1433853_at	mindbomb homolog 1 (Drosophila)	Mib1	0.75952	-1.16688149	-0.2852179
1430173_x_at	cytochrome P450, family 4, subfamily f, polypeptide 16	Cyp4f16	0.75953	1.13934518	0.17746087
1439706_at	RIKEN cDNA A330106F07 gene	A330106F07Rik	0.75954	-1.11331405	-0.1650968
1428296_at	predicted pseudogene 10774 /// polymerase (RNA) II (DNA direc	Gm10774 /// Polr2l /// Us	0.75957	1.10761591	0.13667419
1425922_a_at	v-myc myelocytomatosis viral related oncogene, neuroblastoma	Mycn	0.75957	-1.09751106	-0.1382871
1428024_at	microtubule-associated protein 1 light chain 3 beta	Map1lc3b	0.75958	1.13947142	0.17893751
1451671_at	golgi reassembly stacking protein 1	Gorasp1	0.75958	1.1088318	0.14244146
1445275_at	family with sequence similarity 190, member A	Fam190a	0.75958	1.13135942	0.17026221
1444960_at	cytochrome P450, family 2, subfamily u, polypeptide 1	Cyp2u1	0.75959	-1.11492857	-0.1586167
1452694_at	inositol hexaphosphate kinase 1	Ip6k1	0.75961	1.10822276	0.1413011
1424822_at	SLAIN motif family, member 1	Slain1	0.75961	1.11775799	0.15540657
1426421_s_at	hypothetical LOC100502987 /// RNA binding motif protein 26	LOC100502987 /// Rbm2	0.75962	-1.08853507	-0.1234871
1442267_at	syntaxin binding protein 4	Stxbp4	0.75962	-1.14411408	-0.228971

1435669_at	zinc finger protein 532	Zfp532	0.75964	1.08703516	0.11720026
1424322_at	apurinic/apyrimidinic endonuclease 2	Apex2	0.75964	1.10052502	0.13547395
1449828_at	prostaglandin F receptor	Ptgfr	0.75967	1.09741028	0.13290037
1445097_at	---	---	0.75968	-1.1155766	-0.1598533
1416835_s_at	S-adenosylmethionine decarboxylase 1	Amd1	0.75968	-1.07815843	-0.1114623
1458897_at	uronyl-2-sulfotransferase	Ust	0.75968	-1.1094466	-0.1559795
1440534_at	RIKEN cDNA 6330403A02 gene /// predicted gene 10001	6330403A02Rik /// Gm10	0.75969	1.15218309	0.19221047
1439729_at	---	---	0.75969	1.15591726	0.19403013
1422633_at	RIKEN cDNA 1700020D05 gene	1700020D05Rik	0.7597	1.17487426	0.20955864
1436521_at	solute carrier family 36 (proton/amino acid symporter), member 2	Slc36a2	0.7597	1.14753485	0.1843671
1458807_at	erythrocyte protein band 4.1	Epb4.1	0.75971	-1.1217354	-0.1684716
1459555_at	---	---	0.75971	1.11957549	0.15790411
1459632_at	---	---	0.75972	1.11730925	0.15602949
1452602_a_at	RIKEN cDNA 1700001C19 gene	1700001C19Rik	0.75972	1.1245844	0.16474496
1417402_at	family with sequence similarity 165, member B	Fam165b	0.75972	1.08828856	0.12200769
1452941_at	succinate dehydrogenase complex assembly factor 2	Sdhaf2	0.75974	1.09310574	0.12759488
1435735_x_at	histocompatibility 47	H47	0.75974	-1.09239566	-0.132861
1446150_at	---	---	0.75975	1.1603184	0.19744799
1417780_at	LAG1 homolog, ceramide synthase 4	Lass4	0.75976	1.10811444	0.14373933
1431146_a_at	copine VIII	Cpne8	0.75977	-1.12498704	-0.1791671
1453703_at	dynein, axonemal, heavy chain 17	Dnahc17	0.75977	1.1231894	0.16526954
1442039_at	thymocyte selection-associated high mobility group box	Tox	0.75977	-1.11548059	-0.1580415
1417177_at	galactokinase 1	Galk1	0.75977	1.10152172	0.13144866
1457148_at	CUB and Sushi multiple domains 2	Csmd2	0.75978	-1.12190385	-0.1723162
1431812_a_at	solute carrier family 6 (neurotransmitter transporter, glycine), me	Slc6a9	0.75978	-1.09747289	-0.1367283
1420266_at	predicted gene 10627	Gm10627	0.75979	1.11439145	0.15259525
1431640_at	RIKEN cDNA 4933431J24 gene	4933431J24Rik	0.75979	1.1529515	0.1883611
1428349_s_at	early B-cell factor 3	Ebf3	0.75979	1.12519641	0.16735044
1436296_x_at	dynein, axonemal, intermediate chain 2	Dnaic2	0.75985	-1.16351334	-0.3108327
1443381_at	ets variant gene 4 (E1A enhancer binding protein, E1AF)	Etv4	0.75985	-1.13538463	-0.2006182
1458815_at	expressed sequence AU021884	AU021884	0.75986	-1.08094538	-0.1126022
1430281_at	GLI pathogenesis-related 1 like 1	Glipr1l1	0.75987	1.09797758	0.13481565
1416152_a_at	serine/arginine-rich splicing factor 3	Srsf3	0.75988	-1.07138712	-0.0996311
1416169_at	trophoblast specific protein beta	Tpbpb	0.75989	1.09129175	0.12563587
1425459_at	myotubularin related protein 2	Mtmr2	0.75989	1.08873764	0.12097801
1424315_at	RIKEN cDNA 1110004E09 gene	1110004E09Rik	0.75992	1.12224271	0.15333884
1422190_at	complement component 5a receptor 1	C5ar1	0.75993	1.17115393	0.20658902
1425430_at	keratin associated protein 16-5	Krtap16-5	0.75994	1.13625111	0.17448438
1447424_at	---	---	0.75995	-1.11255791	-0.1581689
1446464_at	Proteasome (prosome, macropain) activator subunit 4	Psme4	0.75995	1.1365271	0.17339739
1451007_at	cyclin M2	Cnnm2	0.75996	1.10436842	0.14184354
1428495_at	RIKEN cDNA 2410003K15 gene	2410003K15Rik	0.75996	1.08782545	0.11719941
1424496_at	RIKEN cDNA 5133401N09 gene	5133401N09Rik	0.75996	1.09434738	0.1293034
1421972_s_at	host cell factor C1	Hcfc1	0.75996	-1.09403201	-0.1371958
1449064_at	L-threonine dehydrogenase	Tdh	0.75997	1.0672592	0.09332866
1430481_at	RIKEN cDNA 4930545L23 gene	4930545L23Rik	0.75998	-1.12604709	-0.1769586
1417180_at	proprotein convertase subtilisin/kexin type 7	Pcsk7	0.75998	1.09801228	0.13363179
1441105_at	predicted gene 11110	Gm11110	0.75998	1.14163343	0.1799528
1449196_a_at	ribosomal protein S27A	Rps27a	0.75999	-1.03528748	-0.0501718
1434480_at	pyruvate dehydrogenase phosphatase regulatory subunit	Pdpr	0.76002	-1.11321835	-0.1666659
1434012_at	integrator complex subunit 5	Ints5	0.76002	1.0848612	0.11577443
1439641_at	hypothetical LOC100503774 /// hypothetical LOC553090	LOC100503774 /// LOC55	0.76004	1.12439989	0.16665334
1459394_at	RIKEN cDNA D130052B06 gene	D130052B06Rik	0.76004	1.16540484	0.20165359
1441472_at	---	---	0.76004	-1.11563574	-0.1603152
1448132_at	solute carrier family 19 (sodium/hydrogen exchanger), member 1	Slc19a1	0.76006	1.09150195	0.12497174
1452124_at	ankyrin 3, epithelial	Ank3	0.76006	-1.12230656	-0.1737784
1419260_a_at	small nuclear ribonucleoprotein B	Snrpb	0.76006	1.07012923	0.09772774
1455956_x_at	cyclin D2	Ccnd2	0.76007	-1.11426034	-0.169894
1428613_at	lactate dehydrogenase D	Ldhd	0.76008	-1.12040369	-0.1719319
1454997_at	methionine sulfoxide reductase B3	Msrb3	0.76011	1.11159137	0.15077965
1434354_at	monoamine oxidase B	Maob	0.76011	-1.11755275	-0.165121
1449317_at	CASP8 and FADD-like apoptosis regulator	Cflar	0.76013	1.10421865	0.14026466
1438767_at	oncostatin M	Osm	0.76013	-1.10944069	-0.1555755
1453362_x_at	ribosomal protein S24	Rps24	0.76014	-1.04471353	-0.0636948
1420417_at	sema domain, immunoglobulin domain (Ig), short basic domain, s	Sema3a	0.76015	1.17318384	0.20396367
1444290_at	six transmembrane epithelial antigen of prostate 2	Steap2	0.76017	1.11804223	0.15895988
1432582_at	RIKEN cDNA 3110054G05 gene	3110054G05Rik	0.76018	-1.11162154	-0.1572127
1455812_x_at	vasorin	Vasn	0.76018	1.11098482	0.14886572
1418854_at	baculoviral IAP repeat-containing 2	Birc2	0.76019	1.08729638	0.11724232
1452141_a_at	selenoprotein P, plasma, 1	Sepp1	0.76019	1.0998137	0.13405579
1445113_at	---	---	0.7602	-1.11273468	-0.155057
1453318_at	transmembrane protein 102	Tmem102	0.7602	1.10396457	0.14214924
1438845_at	inositol hexaphosphate kinase 1	Ip6k1	0.7602	-1.11335298	-0.1671255
1439163_at	zinc finger and BTB domain containing 16	Zbtb16	0.76021	1.11024412	0.15056347
1440995_at	RIKEN cDNA 6430531B16 gene /// hypothetical LOC100503791	6430531B16Rik /// LOC10	0.76021	-1.12680003	-0.1827306
1433397_at	RIKEN cDNA 9430012M22 gene	9430012M22Rik	0.76022	-1.13400112	-0.2081383
1450917_at	myomesin 2	Myom2	0.76022	1.15809984	0.19426369
1432848_a_at	FERM domain containing 8	Frmd8	0.76026	1.08285705	0.11326613
1432149_at	DPH1 homolog (S. cerevisiae)	Dph1	0.76028	-1.11338147	-0.1571855
1447555_at	---	---	0.76029	1.11757462	0.15943985
1437410_at	aldehyde dehydrogenase 2, mitochondrial	Aldh2	0.76029	-1.10717704	-0.1512582
1422496_at	high mobility group nucleosomal binding domain 1	Hmgn1	0.7603	-1.14991539	-0.2466667
1422199_at	olfactory marker protein	Omp	0.7603	1.12363054	0.16245752
1426070_a_at	potassium voltage-gated channel, Shal-related family, member 3	Kcnd3	0.76031	1.11967559	0.16222931
1421044_at	mannose receptor, C type 2	Mrc2	0.76032	-1.1262727	-0.1763996
1425475_at	collagen, type IV, alpha 5	Col4a5	0.76032	-1.13091726	-0.188892
1436720_s_at	oogenesin 3	Oog3	0.76034	1.08557651	0.11712255
1420775_at	haloacid dehalogenase-like hydrolase domain containing 1A	Hdhd1a	0.76034	1.15148356	0.19006546
1452573_a_at	RIKEN cDNA 0610007P08 gene	0610007P08Rik	0.76036	1.12448938	0.16199352
1426633_s_at	potassium channel tetramerisation domain containing 14	Kctd14	0.76036	-1.1231044	-0.1751578
1448858_at	Unc-51 like kinase 2 (C. elegans)	Ulk2	0.76036	1.12179673	0.16039498
1436221_at	immunoglobulin-like domain containing receptor 2	Ildr2	0.76037	1.12530098	0.16853829
1426556_at	zinc finger protein 280D	Zfp280d	0.76037	-1.09009304	-0.124502
1438852_x_at	minichromosome maintenance deficient 6 (MIS5 homolog, S. pon	Mcm6	0.76037	-1.11174931	-0.1769883

1439232_at	RIKEN cDNA 1500016L03 gene	1500016L03Rik	0.76038	-1.09585589	-0.1338323
1442613_at	spondin 1, (f-spondin) extracellular matrix protein	Spon1	0.76038	-1.11405037	-0.1651973
1418807_at	RIKEN cDNA 3110070M22 gene	3110070M22Rik	0.76039	1.13093366	0.16714945
1449623_at	thioredoxin reductase 3	Txnrd3	0.7604	1.0971988	0.13358589
1435892_at	additional sex combs like 3 (Drosophila)	Asxl3	0.7604	1.1407434	0.17915032
1446154_at	hypothetical protein LOC100047123	LOC100047123	0.7604	-1.12514915	-0.1804584
1444890_at	---	---	0.7604	1.11976755	0.16269855
1419489_at	family with sequence similarity 19, member A5	Fam19a5	0.7604	1.1319747	0.17176531
1438783_at	---	---	0.76041	1.12037123	0.16068782
1426479_a_at	canopy 3 homolog (zebrafish)	Cnpy3	0.76041	1.1077548	0.14054125
1436251_at	phosphodiesterase 1C	Pde1c	0.76042	-1.11187189	-0.1566163
1454729_at	transmembrane protein 108	Tmem108	0.76042	-1.12769079	-0.190321
1429614_at	PRP18 pre-mRNA processing factor 18 homolog (yeast)	Prpf18	0.76042	1.08193183	0.113091
1426567_a_at	PQ loop repeat containing 1	Pqlc1	0.76043	1.10109919	0.13379434
1440830_at	G protein-coupled receptor 116	Gpr116	0.76044	1.14968924	0.18668864
1456529_at	Forkhead box O1	Foxo1	0.76044	-1.10493958	-0.1448431
1450341_at	protocadherin beta 8	Pcdhb8	0.76045	-1.1420067	-0.2160403
1441689_at	nucleoporin 153	Nup153	0.76045	-1.13058189	-0.192449
1431416_a_at	junction adhesion molecule 2	Jam2	0.76046	-1.08752059	-0.1221191
1438973_x_at	gap junction protein, alpha 1	Gja1	0.76047	-1.10425295	-0.1439913
1457103_at	expressed sequence AU046084	AU046084	0.76047	-1.12355264	-0.1797373
1435704_at	RIKEN cDNA C920006O11 gene	C920006O11Rik	0.76047	1.10006453	0.13708726
1440389_at	---	---	0.76048	1.14830669	0.18208532
1423882_at	ring finger and WD repeat domain 3	Rfwd3	0.76048	1.08245867	0.11330753
1415818_at	annexin A6	Anxa6	0.76048	1.11793682	0.14948022
1423927_at	solute carrier family 35, member B2	Slc35b2	0.76048	1.09929061	0.13375827
1418292_at	arsA arsenite transporter, ATP-binding, homolog 1 (bacterial)	Asna1	0.76049	-1.07127048	-0.0998857
1432856_at	---	---	0.76049	1.12921413	0.16941153
1428085_at	RIKEN cDNA 1110057K04 gene	1110057K04Rik	0.76049	-1.0869441	-0.1211141
1450059_at	Fanconi anemia, complementation group G	Fancg	0.76049	-1.104526	-0.1447923
1434490_at	scavenger receptor class F, member 1	Scarf1	0.76049	1.11923463	0.15745214
1428503_a_at	NFKB inhibitor interacting Ras-like protein 1	Nkiras1	0.7605	1.1054637	0.1410694
1419369_at	ring finger protein 138	Rnf138	0.76051	-1.08827215	-0.1227127
1432790_at	RIKEN cDNA 9030218A15 gene	9030218A15Rik	0.76051	1.13606097	0.17549104
1422997_s_at	acyl-CoA thioesterase 1 /// acyl-CoA thioesterase 2	Acot1 /// Acot2	0.76051	1.11868353	0.15432204
1450179_at	sclerostin	Sost	0.76052	-1.11253734	-0.158812
1442449_at	solute carrier family 6 (neurotransmitter transporter, noradrenalin	Slc6a2	0.76053	-1.12460805	-0.1767897
1456465_at	solute carrier family 25, member 44	Slc25a44	0.76055	-1.1040446	-0.1432985
1443060_at	hypothetical LOC100503797	LOC100503797	0.76055	1.1231433	0.16679193
1418845_at	protein C	Proc	0.76056	1.11525147	0.15638642
1444307_at	---	---	0.76056	-1.13337516	-0.2052017
1434406_at	SLIT-ROBO Rho GTPase activating protein 2	Srgap2	0.76056	1.08835984	0.12128737
1445492_at	---	---	0.76058	1.13912098	0.17941093
1438868_at	DNA segment, Chr 14, ERATO Doi 668, expressed	D14Ertd668e	0.76058	-1.08832145	-0.1228206
1448384_at	protein O-fucosyltransferase 2	Pofut2	0.76059	1.09790099	0.13197212
1453561_x_at	RIKEN cDNA 8430431K14 gene	8430431K14Rik	0.76059	1.09207005	0.12451076
1424241_at	solute carrier family 30 (zinc transporter), member 6	Slc30a6	0.7606	1.08527983	0.1169292
1448924_at	transmembrane protein 186	Tmem186	0.76061	-1.1090041	-0.1553957
1438975_x_at	zinc finger, DHHC domain containing 14	Zdhhc14	0.76062	1.15814823	0.19437414
1434879_at	cell division cycle 34 homolog (S. cerevisiae)	Cdc34	0.76062	1.08424679	0.11120767
1426653_at	minichromosome maintenance deficient 3 (S. cerevisiae)	Mcm3	0.76062	1.07990296	0.109378
1417444_at	E2F transcription factor 5	E2f5	0.76064	-1.10092719	-0.1513772
1425044_at	potassium inwardly-rectifying channel, subfamily J, member 6	Kcnj6	0.76065	-1.11082168	-0.1521352
1430692_a_at	sel-1 suppressor of lin-12-like (C. elegans)	Sel1l	0.76066	1.09646257	0.13215132
1417344_at	RIKEN cDNA 2900064A13 gene	2900064A13Rik	0.76067	1.07965915	0.11019784
1435661_at	amyotrophic lateral sclerosis 2 (juvenile) chromosome region, can	Als2cr4	0.76067	-1.09052363	-0.1295609
1437851_x_at	ribonucleic acid binding protein S1	Rnps1	0.76068	-1.07457982	-0.1088911
1431321_at	purinergic receptor P2X, ligand-gated ion channel, 3	P2rx3	0.7607	-1.11917115	-0.1708366
1453770_at	carboxypeptidase A4	Cpa4	0.7607	-1.119731	-0.1687703
1423652_at	iron-sulfur cluster assembly 1 homolog (S. cerevisiae)	Isca1	0.76072	1.08472597	0.11558987
1455254_at	RIKEN cDNA 4833420G11 gene	4833420G11Rik	0.76073	-1.08456775	-0.1200117
1445752_at	---	---	0.76076	1.13675119	0.18016297
1445321_at	---	---	0.76077	-1.11633717	-0.1627977
1452278_a_at	HECT domain and ankyrin repeat containing, E3 ubiquitin protein	Hace1	0.76077	-1.10903058	-0.158306
1458077_at	---	---	0.76078	1.1491627	0.18671894
1417117_at	cleavage stimulation factor, 3' pre-RNA, subunit 1	Cstf1	0.76078	1.12135164	0.15859815
1439124_at	WD repeat domain 91	Wdr91	0.7608	1.11859455	0.1582957
1446473_at	RIKEN cDNA 4732418C07 gene	4732418C07Rik	0.76084	-1.12451395	-0.1719799
1432704_at	RIKEN cDNA 5730405A17 gene	5730405A17Rik	0.76085	-1.11480426	-0.1604434
1426850_a_at	mitogen-activated protein kinase kinase 6	Map2k6	0.76087	1.12854493	0.1627083
1452412_at	homeobox C8	Hoxc8	0.76089	1.09900146	0.13359422
1436753_at	aarF domain containing kinase 5	Adck5	0.76104	1.09167071	0.1231295
1457672_at	chromodomain helicase DNA binding protein 9	Chd9	0.76104	1.08573791	0.11720797
1424408_at	LIM and senescent cell antigen like domains 2	Lims2	0.76105	1.10000966	0.13673383
1430930_at	RIKEN cDNA 0610038B21 gene	0610038B21Rik	0.76112	-1.12765979	-0.1838551
1443004_at	---	---	0.76116	1.08937211	0.12068574
1456611_at	family with sequence similarity 13, member A	Fam13a	0.76117	1.15635655	0.19215915
1448567_at	transmembrane protein 115	Tmem115	0.76117	1.08815665	0.12135974
1421202_at	cholinergic receptor, nicotinic, alpha polypeptide 4	Chrna4	0.76118	-1.12413439	-0.1766883
1456657_at	expressed sequence AA415437	AA415437	0.76118	-1.11832978	-0.16592
1443902_at	RIKEN cDNA 6430573F11 gene	6430573F11Rik	0.7612	-1.10894693	-0.1503347
1417561_at	apolipoprotein C-I	Apoc1	0.7612	1.1218632	0.15327809
1419289_a_at	synaptogyrin 1	Syngr1	0.7612	1.1030929	0.13347372
1455892_x_at	---	---	0.76125	-1.08643342	-0.1215764
1418572_x_at	tumor necrosis factor receptor superfamily, member 12a	Tnfrsf12a	0.76127	1.10818549	0.14563758
1423948_at	BCL2-associated athanogene 2	Bag2	0.76129	1.11036015	0.14846192
1439797_at	peroxisome proliferator activator receptor delta	Ppard	0.76137	1.11004133	0.14214269
1432418_a_at	creatine kinase, mitochondrial 1, ubiquitous	Ckmt1	0.76137	1.09876283	0.13463573
1425946_at	glutathione S-transferase, mu 7	Gstm7	0.76149	-1.10877405	-0.1493327
1447206_at	Rho GTPase activating protein 21	Arhgap21	0.76154	1.11964258	0.16058998
1418098_at	adenylate cyclase 4	Adcy4	0.76156	-1.11335862	-0.1579824
1423117_at	pumilio 1 (Drosophila)	Pum1	0.76162	-1.0904161	-0.1306718
1460084_at	---	---	0.76164	1.21841564	0.24294887
1448390_a_at	dehydrogenase/reductase (SDR family) member 3	Dhrs3	0.76169	-1.11199485	-0.153654

1444203_at	---	---	0.76172	-1.09857623	-0.1423346
1417351_a_at	small nuclear ribonucleoprotein polypeptide A'	Snrpa1	0.76177	-1.09091511	-0.1361648
1435927_at	DENN/MADD domain containing 3	Dennd3	0.76177	1.1358313	0.17055668
1444027_at	solute carrier family 30 (zinc transporter), member 8	Slc30a8	0.76179	-1.12454209	-0.1808764
1440781_at	RIKEN cDNA B830007D08 gene	B830007D08Rik	0.76182	1.15405833	0.19139988
1458549_at	---	---	0.76183	1.10957342	0.14911122
1460472_at	cyclin-dependent kinase 3, pseudogene	Cdk3-ps	0.76185	-1.11401042	-0.156711
1448927_at	potassium intermediate/small conductance calcium-activated cha	Kcnn2	0.76187	1.14851913	0.18478626
1419667_at	sarcoglycan, beta (dystrophin-associated glycoprotein)	Sgcb	0.76188	1.13248208	0.17147686
1458888_at	predicted gene 11696	Gm11696	0.7619	1.11371075	0.15191073
1445881_at	RIKEN cDNA 2310035P21 gene	2310035P21Rik	0.76191	-1.10167668	-0.1421372
1448757_at	promyelocytic leukemia	Pml	0.76193	1.08903728	0.11917282
1430766_at	RIKEN cDNA 5033403F01 gene	5033403F01Rik	0.76194	1.11086991	0.14993162
1422256_at	somatostatin receptor 2	Sstr2	0.76195	1.16038285	0.19521826
1440443_at	RIKEN cDNA E030016H06 gene	E030016H06Rik	0.76195	-1.11807377	-0.1612865
1438395_at	adenylate kinase 4	Ak4	0.76195	1.13016498	0.16996106
1421505_at	Mix1 homeobox-like 1 (Xenopus laevis)	Mixl1	0.76197	-1.11197576	-0.1544406
1429516_at	ubiquitin protein ligase E3 component n-recognin 2	Ubr2	0.76198	1.11565984	0.1561693
1423518_at	c-src tyrosine kinase	Csk	0.762	1.08849541	0.12136838
1441827_x_at	heat shock protein 90, alpha (cytosolic), class A member 1	Hsp90aa1	0.76201	-1.12380846	-0.1791889
1428514_at	copine III	Cpne3	0.76203	-1.15254294	-0.2601869
1455619_at	cDNA sequence BC062258	BC062258	0.76204	1.17192569	0.20340667
1436071_at	ankyrin repeat domain 26	Ankrd26	0.76205	-1.11302024	-0.1609567
1422107_at	RIKEN cDNA 2410066E13 gene	2410066E13Rik	0.76206	1.1185087	0.15979193
1430422_at	RIKEN cDNA 1700020A23 gene	1700020A23Rik	0.7621	1.13676913	0.17725906
1418799_a_at	collagen, type XVII, alpha 1	Col17a1	0.7621	1.17465322	0.20918448
1425723_at	nuclear receptor subfamily 1, group I, member 2	Nr1i2	0.76213	1.11790277	0.15893594
1447600_at	---	---	0.76214	1.10555767	0.14311749
1451070_at	guanosine diphosphate (GDP) dissociation inhibitor 1	Gdi1	0.76214	1.0797354	0.10963907
1448337_at	ubiquinol-cytochrome c reductase complex chaperone, CBP3 hom	Uqcc	0.76216	1.10854366	0.14625591
1453983_a_at	methyltransferase 10 domain containing	Mett10d	0.76217	-1.07411239	-0.1034272
1416102_at	tyrosine 3-monooxygenase/tryptophan 5-monooxygenase activa	Ywhaz	0.76217	1.08256955	0.11115824
1428638_at	EF-hand domain (C-terminal) containing 2	Efhc2	0.76218	1.13125531	0.17064718
1459482_at	transient receptor potential cation channel, subfamily M, membe	Trpm2	0.76219	-1.10961468	-0.1520564
1422974_at	5' nucleotidase, ecto	Nt5e	0.7622	-1.11138074	-0.1600852
1428555_at	SET domain containing 2	Setd2	0.76222	1.07839812	0.10874911
1417170_at	leucine zipper transcription factor-like 1	Lztfl1	0.76222	-1.08774803	-0.126327
1447448_s_at	Kruppel-like factor 6	Klf6	0.76222	1.1175671	0.1548133
1446569_at	---	---	0.76222	1.12639354	0.16517111
1457543_at	NOL1/NOP2/Sun domain family, member 7	Nsun7	0.76223	-1.11436589	-0.1583386
1450699_at	selenium binding protein 1	Selenbp1	0.76223	1.19233795	0.2208835
1444287_at	expressed sequence AI853106	AI853106	0.76225	1.11967898	0.15908914
1427270_a_at	BSD domain containing 1	Bsdc1	0.76225	-1.08233289	-0.1141994
1445805_x_at	potassium voltage-gated channel, subfamily H (eag-related), mer	Kcnh3	0.76226	1.11506865	0.15093876
1453319_at	cell division cycle and apoptosis regulator 1	Ccar1	0.76227	-1.12479276	-0.1757313
1438738_at	---	---	0.76228	-1.10930371	-0.1523393
1422429_at	ring finger protein 14	Rnf14	0.76228	1.08470726	0.11679049
1452942_at	transmembrane protein 65	Tmem65	0.7623	1.09858723	0.13261517
1423456_at	basic leucine zipper and W2 domains 2	Bzw2	0.7623	-1.11742877	-0.1905485
1422499_at	LIM domain and actin binding 1	Lima1	0.76231	1.09881811	0.13531673
1427770_a_at	solute carrier family 2 (facilitated glucose transporter), member 3	Slc2a3	0.76237	-1.10851207	-0.1671554
1431513_at	POTE ankyrin domain family, member G	Poteg	0.7624	-1.10690507	-0.1523666
1448133_at	NMD3 homolog (S. cerevisiae)	Nmd3	0.76241	-1.07876281	-0.112549
1435225_s_at	bromodomain and PHD finger containing, 3	Brpf3	0.76244	1.10439935	0.13798403
1442200_at	RIKEN cDNA 1810063B07 gene	1810063B07Rik	0.76245	-1.1180405	-0.1701368
1432995_at	RIKEN cDNA 2900057E15 gene	2900057E15Rik	0.76245	-1.1288019	-0.1948642
1423240_at	Rous sarcoma oncogene	Src	0.76249	1.11562771	0.14713195
1417380_at	IQ motif containing GTPase activating protein 1	Iqgap1	0.7625	-1.08342991	-0.1198902
1434429_at	synaptotagmin XVI	Syt16	0.76251	-1.09479018	-0.1375668
1457600_x_at	naked cuticle 2 homolog (Drosophila)	Nkd2	0.76252	1.11055845	0.15062879
1437712_x_at	exosome component 4	Exosc4	0.76254	1.09605287	0.12681601
1423080_at	translocase of outer mitochondrial membrane 20 homolog (yeast	Tomm20	0.76254	1.06351824	0.08773264
1416808_at	nidogen 1	Nid1	0.76257	-1.08393941	-0.1208183
1429097_at	ring finger protein 150	Rnf150	0.76258	1.12995398	0.16888482
1422124_a_at	protein tyrosine phosphatase, receptor type, C	Ptprc	0.76259	-1.09495971	-0.1310703
1444300_at	G protein-coupled receptor 125	Gpr125	0.76259	-1.11365505	-0.1556379
1460186_at	transmembrane 9 superfamily member 3	Tm9sf3	0.76262	1.08244021	0.11220671
1457625_s_at	cyclin-dependent kinase-like 2 (CDC2-related kinase)	Cdkl2	0.76263	-1.11830619	-0.1679197
1449358_at	DNA segment, Chr 6, Miriam Meisler 5, expressed	D6Mm5e	0.76263	1.17396794	0.20500914
1448606_at	lysophosphatidic acid receptor 1	Lpar1	0.76264	-1.10344787	-0.1476674
1438228_at	RIKEN cDNA 4930452B06 gene	4930452B06Rik	0.76265	1.10779121	0.14392184
1421999_at	thyroid stimulating hormone receptor	Tshr	0.76266	1.17891649	0.21055368
1422593_at	adaptor-related protein complex 3, sigma 1 subunit	Ap3s1	0.76268	-1.07578822	-0.1057061
1416245_at	aurora kinase A interacting protein 1	Aurkaip1	0.76269	1.08378399	0.11242159
1420182_x_at	doublesex and mab-3 related transcription factor 3	Dmrt3	0.7627	1.12763752	0.16785703
1437344_x_at	keratin 13	Krt13	0.7627	1.11757059	0.15936568
1439705_at	---	---	0.76274	1.15040839	0.18819495
1446271_at	---	---	0.76274	1.11352247	0.14827789
1445469_at	---	---	0.76277	1.09920433	0.13616611
1434195_at	protease, serine, 35	Prss35	0.76277	-1.11124647	-0.1546312
1440334_at	---	---	0.76278	1.12879563	0.16867687
1417066_at	aarF domain containing kinase 3	Adck3	0.7628	1.10474038	0.13986055
1434809_at	Rho GTPase activating protein 28	Arhgap28	0.76282	-1.09612674	-0.1357332
1425861_x_at	calcium channel, voltage-dependent, alpha2/delta subunit 1	Cacna2d1	0.76285	1.09424098	0.12626017
1429751_at	zinc finger, SWIM domain containing 2	Zswim2	0.76285	-1.12576869	-0.1762052
1424188_at	RAB GTPase activating protein 1	Rabgap1	0.76287	1.07625891	0.10528326
1427958_at	abhydrolase domain containing 10	Abhd10	0.76289	1.09992093	0.13718754
1438577_at	---	---	0.7629	-1.07897283	-0.1141238
1444335_at	ER degradation enhancer, mannosidase alpha-like 2	Edem2	0.76296	1.16239438	0.19693883
1418317_at	LIM homeobox protein 2	Lhx2	0.76299	-1.11703207	-0.1618983
1442283_at	---	---	0.76301	-1.10965226	-0.1562208
1448533_at	tubulin folding cofactor B	Tbcb	0.76301	1.08387187	0.11310582
1430771_a_at	mutS homolog 5 (E. coli)	Msh5	0.76301	-1.11637141	-0.1629788
1436060_at	predicted gene 15706 /// RALY RNA binding protein-like	Gm15706 /// Ralyl	0.76303	1.11605306	0.15460066

1459084_at	predicted gene 9898	Gm9898	0.76305	1.13461711	0.17035854
1415735_at	damage specific DNA binding protein 1	Ddb1	0.76306	1.07648114	0.10550994
1456310_a_at	RIKEN cDNA 2610002J02 gene	2610002J02Rik	0.7631	1.10266857	0.13666087
1442433_at	---	---	0.7631	-1.10671947	-0.1479676
1419632_at	tectorin alpha	Tecta	0.76314	1.12038853	0.1602987
1416580_a_at	STIP1 homology and U-Box containing protein 1	Stub1	0.76315	1.07191284	0.09940545
1417353_x_at	small nuclear ribonucleoprotein polypeptide A'	Snrpa1	0.76316	-1.10115146	-0.1574408
1443568_x_at	---	---	0.7632	1.07813834	0.10701782
1447320_x_at	polymerase (RNA) I polypeptide D	Polr1d	0.76321	1.06785884	0.09463996
1444518_at	Acyl-Coenzyme A oxidase 1, palmitoyl	Acox1	0.76322	-1.11324529	-0.1552197
1431983_at	myosin IIIA	Myo3a	0.76322	1.13014445	0.1680904
1425710_a_at	homer homolog 1 (Drosophila)	Homer1	0.76323	1.1124009	0.15046393
1447831_s_at	myotubularin related protein 7	Mtmr7	0.76326	-1.13955067	-0.2160188
1454986_at	zinc finger protein 668	Zfp668	0.76328	1.10259813	0.13840181
1417294_at	aldo-keto reductase family 7, member A5 (aflatoxin aldehyde red	Akr7a5	0.7633	1.09702736	0.13012504
1452757_s_at	hemoglobin alpha, adult chain 1 /// hemoglobin alpha, adult chain	Hba-a1 /// Hba-a2	0.76331	-1.12512664	-0.174887
1458395_at	hypothetical protein B930054O08	B930054O08	0.76331	1.12110941	0.16305843
1420951_a_at	Son DNA binding protein	Son	0.76332	-1.12056373	-0.1903817
1458130_at	---	---	0.76333	-1.11878084	-0.1652512
1437969_s_at	RIKEN cDNA 0610007P22 gene	0610007P22Rik	0.76334	1.10067678	0.13400749
1422842_at	5'-3' exoribonuclease 2	Xrn2	0.76338	-1.08751524	-0.1255639
1452978_at	transmembrane protein 138	Tmem138	0.76339	1.10353866	0.13807894
1427679_at	large tumor suppressor	Lats1	0.76339	-1.13966917	-0.2194363
1424200_s_at	SEH1-like (S. cerevisiae	Seh1l	0.76341	-1.07629146	-0.1075419
1438734_at	RIKEN cDNA A530016L24 gene	A530016L24Rik	0.76342	1.11782422	0.15837657
1436070_at	glyoxalase 1	Glo1	0.76345	1.0908385	0.1248812
1456307_s_at	adenylate cyclase 7	Adcy7	0.76345	1.11349363	0.15268119
1429101_at	zinc finger protein 697	Zfp697	0.76347	-1.10082772	-0.1390562
1428140_at	3-oxoacid CoA transferase 1	Oxct1	0.76347	1.08190226	0.11236259
1456279_a_at	B-cell receptor-associated protein 31	Bcap31	0.76348	-1.07778745	-0.1115424
1453582_at	choline kinase alpha	Chka	0.76348	-1.12523647	-0.1797586
1435852_at	sprouty-related, EVH1 domain containing 3	Spred3	0.76348	1.12408304	0.1647991
1426793_a_at	ribosomal protein L14 /// ribosomal protein L14, pseudogene 1	Rpl14 /// Rpl14-ps1	0.76349	-1.07213647	-0.1088399
1448861_at	TNF receptor-associated factor 5	Traf5	0.76349	-1.11853005	-0.1653479
1444438_at	calcium and integrin binding family member 3	Cib3	0.7635	-1.12555848	-0.1783455
1459997_s_at	transmembrane protein 17	Tmem17	0.7635	1.13674417	0.1727709
1456900_at	---	---	0.7635	-1.11882574	-0.1624633
1419599_s_at	membrane-spanning 4-domains, subfamily A, member 6D	Ms4a6d	0.76355	1.1273204	0.16709567
1432358_at	mucin 16	Muc16	0.76362	1.13439336	0.17429434
1438593_at	---	---	0.7637	1.11114882	0.1477098
1427954_at	cDNA sequence BC048403	BC048403	0.7639	-1.11140989	-0.1686289
1438553_x_at	RIKEN cDNA 4930453N24 gene	4930453N24Rik	0.7639	-1.11229661	-0.1635134
1454144_a_at	cyclin C	Ccnc	0.7639	-1.10928929	-0.1533528
1449946_a_at	zinc finger protein 593	Zfp593	0.76393	1.09396912	0.1289172
1428364_at	sodium channel modifier 1	Scnm1	0.76395	1.08819602	0.12044041
1424869_at	dehydrogenase/reductase (SDR family) member 7B	Dhrs7b	0.76395	1.08428599	0.11522333
1424534_at	monocyte to macrophage differentiation-associated 2	Mmd2	0.76396	-1.11018498	-0.1577951
1454495_at	RIKEN cDNA 4930432H08 gene	4930432H08Rik	0.76396	-1.11254404	-0.1545477
1422315_x_at	phosphorylase kinase gamma 1	Phkg1	0.76397	1.12007965	0.16168119
1425880_x_at	zinc finger protein 352	Zfp352	0.76398	-1.11933841	-0.1730813
1436595_at	RNA binding motif protein 34	Rbm34	0.76398	1.08585805	0.11867751
1426645_at	heat shock protein 90, alpha (cytosolic), class A member 1	Hsp90aa1	0.764	-1.03820256	-0.0545975
1439132_at	PHD finger protein 8	Phf8	0.76406	1.12592873	0.16473595
1427996_at	cDNA sequence BC028528	BC028528	0.76422	1.09855565	0.13165226
1423127_at	inositol (myo)-1(or 4)-monophosphatase 1	Impa1	0.76424	-1.08739973	-0.1261166
1459151_x_at	interferon-induced protein 35	Ifi35	0.76425	1.09909643	0.13368292
1438959_x_at	---	---	0.76426	-1.08318407	-0.116403
1423208_at	transmembrane protein 167	Tmem167	0.76438	-1.09171557	-0.1286415
1441825_x_at	Immunoglobulin mu binding protein 2	Ighmbp2	0.76439	1.15292489	0.18869298
1443417_at	expressed sequence C87122	C87122	0.76442	1.12543115	0.16339225
1460440_at	latrophilin 3	Lphn3	0.76446	-1.12181615	-0.1730923
1440179_x_at	ring finger protein 217	Rnf217	0.76447	1.10569862	0.14423129
1434774_at	rhomboid 5 homolog 2 (Drosophila)	Rhbdf2	0.76447	-1.08643156	-0.1197098
1432833_at	RIKEN cDNA 4933435G04 gene	4933435G04Rik	0.76448	-1.11387951	-0.1582267
1417996_at	neuroglobin	Ngb	0.76451	1.14301337	0.17955346
1454055_at	ribosomal protein L17 pseudogene	1600029O15Rik	0.76458	-1.10888853	-0.1498395
1459658_at	minichromosome maintenance deficient 5, cell division cycle 46 (S	Mcm5	0.76464	1.10486344	0.14071862
1455854_a_at	slingshot homolog 1 (Drosophila)	Ssh1	0.76467	1.12031203	0.1570445
1423898_a_at	thyroid hormone receptor interactor 12	Trip12	0.76473	-1.06362415	-0.0891886
1427138_at	coiled-coil domain containing 88C	Ccdc88c	0.76473	1.1010817	0.1346776
1438873_at	zinc finger protein 389	Zfp389	0.76481	1.09942274	0.13520064
1451364_at	polymerase (RNA) III (DNA directed) polypeptide G like	Polr3gl	0.76482	1.12175164	0.15353943
1456327_at	RIKEN cDNA A530020G20 gene	A530020G20Rik	0.76488	-1.11698476	-0.1661016
1425016_at	Eph receptor B2	Ephb2	0.76488	1.13196076	0.16752621
1457289_at	nuclear receptor subfamily 2, group E, member 1	Nr2e1	0.76488	-1.12561682	-0.1803794
1456090_at	pyruvate dehydrogenase complex, component X	Pdhx	0.76493	1.0792182	0.10924783
1419529_at	interleukin 23, alpha subunit p19	Il23a	0.76493	1.12639303	0.1675945
1423362_at	sortilin 1	Sort1	0.76497	1.09900488	0.13438475
1430456_at	RIKEN cDNA 5830406C21 gene	5830406C21Rik	0.76499	-1.11181613	-0.1574773
1434247_at	lactate dehydrogenase A-like 6B	Ldhal6b	0.765	-1.11079815	-0.1543623
1438336_at	F-box and WD-40 domain protein 11	Fbxw11	0.765	1.13348215	0.17089425
1438294_at	ataxin 1	Atxn1	0.76501	-1.1244414	-0.1811694
1434432_at	ring finger and FYVE like domain containing protein	Rffl	0.76501	-1.09514063	-0.1316328
1451752_at	forkhead box K1	Foxk1	0.76501	1.12158491	0.15902762
1443646_at	Immunoglobulin superfamily, member 10	Igsf10	0.76502	1.09172468	0.12645273
1455340_at	DENN/MADD domain containing 5B	Dennd5b	0.76502	1.12910713	0.16823929
1434189_at	stromal antigen 1	Stag1	0.76503	1.08507464	0.11724341
1445427_at	---	---	0.76505	-1.11845644	-0.1632102
1424959_at	annexin A13	Anxa13	0.76506	1.14237545	0.16949969
1442499_at	carnitine deficiency-associated gene expressed in ventricle 3	Cdv3	0.76507	-1.10217008	-0.1418443
1456478_at	phosphoglucomutase 2-like 1	Pgm2l1	0.76507	1.11252352	0.14984906
1447830_s_at	regulator of G-protein signaling 2	Rgs2	0.7651	1.14776995	0.18238603
1427093_at	zinc finger protein 707	Zfp707	0.76512	1.10194092	0.13761241
1435692_at	potassium channel tetramerisation domain containing 21	Kctd21	0.7652	1.1367846	0.17423783

1421071_at	von Hippel-Lindau tumor suppressor	Vhl	0.76521	1.1031334	0.14084661
1422768_at	synaptotagmin binding, cytoplasmic RNA interacting protein	Syncrip	0.76522	-1.08017392	-0.1132891
1452642_at	hypothetical LOC100502979	LOC100502979	0.76523	1.16109454	0.19591464
1432926_at	---	---	0.76523	1.1397868	0.1791916
1460566_at	microtubule-associated protein 1 A	Mtap1a	0.7653	1.12548432	0.15746808
1448504_a_at	chromobox homolog 3 (Drosophila HP1 gamma)	Cbx3	0.76531	1.17809072	0.19720604
1419158_a_at	histidyl-tRNA synthetase 2, mitochondrial (putative)	Hars2	0.76532	1.09011423	0.12378993
1433837_at	RIKEN cDNA 8430408G22 gene	8430408G22Rik	0.76535	-1.11471608	-0.1615713
1418775_at	expressed sequence AI837181	AI837181	0.76538	1.09253716	0.12582888
1452695_at	endoplasmic reticulum protein 27	Erp27	0.76539	-1.11300975	-0.1609422
1434868_at	RIKEN cDNA 4933431E20 gene	4933431E20Rik	0.76543	1.14593305	0.18216147
1435920_x_at	ATPase, Na+/K+ transporting, alpha 1 polypeptide	Atp1a1	0.76544	1.1396753	0.17707538
1459290_at	RIKEN cDNA C030015D19 gene	C030015D19Rik	0.76544	-1.10367048	-0.144209
1430091_at	RIKEN cDNA 1700001P01 gene	1700001P01Rik	0.76545	-1.11253952	-0.1584004
1417855_at	F-box and leucine-rich repeat protein 15	Fbxl15	0.76545	1.12046149	0.154587
1441014_at	---	---	0.76546	1.11435773	0.1555906
1459759_s_at	RIKEN cDNA 1700065I17 gene	1700065I17Rik	0.76549	1.16463953	0.20154796
1416177_at	RNA binding motif protein, X chromosome retrogene	Rbmxrt	0.76549	1.06421227	0.08969024
1429717_at	importin 11 /// leucine-rich repeat-containing protein 70-like	Ipo11 /// LOC100505126	0.76554	1.13542584	0.17424985
1425124_at	ring finger protein 183	Rnf183	0.76555	-1.09348873	-0.1320094
1457498_at	mannosidase 2, alpha 2	Man2a2	0.76558	1.11834089	0.15774797
1426492_at	tyrosyl-DNA phosphodiesterase 1	Tdp1	0.7656	1.09224533	0.12471204
1435423_x_at	RIKEN cDNA 4933433P14 gene	4933433P14Rik	0.76564	-1.10186297	-0.1420755
1456517_at	transmembrane protein 44	Tmem44	0.76567	1.10407976	0.13593771
1419223_a_at	dystrobrevin alpha	Dtna	0.76569	1.12365292	0.16428186
1419444_at	predicted gene 10094 /// Sin3-associated polypeptide 18	Gm10094 /// Sap18	0.76569	1.17182223	0.19437375
1455681_at	zinc finger protein 369	Zfp369	0.76569	1.08686734	0.11873611
1438560_x_at	chaperonin containing Tcp1, subunit 4 (delta)	Cct4	0.76596	-1.09120298	-0.1412333
1420592_a_at	acidic (leucine-rich) nuclear phosphoprotein 32 family, member E	Anp32e	0.766	-1.07054547	-0.0986067
1458718_at	---	---	0.766	-1.11286963	-0.1612053
1452104_at	ADP-ribosylation factor-like 16	Arl16	0.76602	-1.10999402	-0.154017
1443680_at	Otx2 opposite strand transcript 1	Otx2os1	0.76633	-1.12107581	-0.1770727
1426986_at	family with sequence similarity 76, member B	Fam76b	0.76635	-1.09419799	-0.1351461
1455566_s_at	spermatogenesis associated, serine-rich 2-like	Spats2l	0.76643	-1.12486202	-0.1849594
1425628_a_at	general transcription factor II I	Gtf2i	0.76644	1.07090701	0.09855202
1458464_at	HECT, C2 and WW domain containing E3 ubiquitin protein ligase 2	Hecw2	0.76645	-1.10403291	-0.1449206
1441858_at	---	---	0.76648	1.14445009	0.18089539
1416911_a_at	akirin 1	Akirin1	0.7665	-1.08588352	-0.1233901
1457736_at	vacuolar protein sorting 37D (yeast)	Vps37d	0.76651	1.12813702	0.16317578
1431789_s_at	transmembrane emp24 protein transport domain containing 5	Tmed5	0.76651	-1.10179304	-0.1461341
1451206_s_at	cytohesin 1 interacting protein	Cytip	0.76653	1.10286873	0.13660165
1449959_x_at	late cornified envelope 1H	Lce1h	0.76654	-1.10066672	-0.1450847
1440882_at	low density lipoprotein receptor-related protein 8, apolipoprotein e	Lrp8	0.76657	1.10080235	0.13820009
1440371_at	---	---	0.76657	-1.13465543	-0.2039297
1455746_at	kinesin family member 13A	Kif13a	0.76659	1.09282426	0.12520948
1418034_at	mitochondrial ribosomal protein S9	Mrps9	0.7667	1.0874404	0.11839922
1424940_s_at	cDNA sequence BC022687	BC022687	0.76675	1.11672329	0.15212715
1452576_at	---	---	0.76678	-1.10192366	-0.1424762
1425856_at	Calcium and integrin binding 1 (calmyrin)	Cib1	0.76683	-1.12510322	-0.1807006
1432743_at	RIKEN cDNA 4930443G03 gene	4930443G03Rik	0.76685	1.10030248	0.13781816
1417427_at	ribonuclease H2, subunit C	Rnaseh2c	0.76686	1.08545632	0.11586437
1439563_at	zinc finger protein 3	Zfp3	0.76687	1.14356298	0.18027427
1427468_at	protein phosphatase 3, catalytic subunit, beta isoform	Ppp3cb	0.76689	-1.10829971	-0.1670892
1427505_a_at	CASP2 and RIPK1 domain containing adaptor with death domain	Cradd	0.7669	1.09176024	0.12466946
1444780_at	neuron navigator 2	Nav2	0.76692	1.13736098	0.17643762
1436887_x_at	glutamate-rich WD repeat containing 1	Grwd1	0.76696	-1.09245391	-0.1368942
1427101_at	meteorin, glial cell differentiation regulator	Metrn	0.76704	1.10551172	0.14213331
1422025_at	microphthalmia-associated transcription factor	Mitf	0.76718	1.14175935	0.17811995
1440971_x_at	zinc finger protein 771	Zfp771	0.7672	1.09510344	0.12973846
1445071_at	---	---	0.76721	1.21118965	0.23119449
1419030_at	ERO1-like (S. cerevisiae)	Ero1l	0.76724	1.12357138	0.15390413
1426546_at	testis-specific kinase 2	Tesk2	0.76725	1.11929922	0.15195868
1458299_s_at	nuclear factor of kappa light polypeptide gene enhancer in B-cells	Nfkbie	0.76727	1.12790997	0.16542929
1417576_a_at	OTU domain, ubiquitin aldehyde binding 2	Otub2	0.76729	-1.14648951	-0.2415279
1430119_at	fibronectin type III domain containing 1 /// fibronectin type III do	Fndc1 /// LOC100039091	0.76729	1.13229517	0.17164043
1422980_a_at	blocked early in transport 1 homolog (S. cerevisiae)-like	Bet1l	0.76729	1.10711353	0.14150387
1459486_at	expressed sequence AU022240	AU022240	0.7673	1.17270422	0.20331875
1445454_at	DNA segment, Chr 2, ERATO Doi 282, expressed	D2Ertd282e	0.76731	-1.10533058	-0.1451744
1438774_s_at	phosphoglucomutase 2-like 1	Pgm2l1	0.76731	-1.12419479	-0.1854542
1433617_s_at	UDP-Gal:betaGlcNAc beta 1,4-galactosyltransferase, polypeptide	B4galt5	0.76731	1.09361159	0.12629974
1418987_at	phospholipase A2, group IID	Pla2g2d	0.76732	1.12416111	0.16315989
1442106_at	Fanconi anemia, complementation group M	Fancm	0.76732	-1.15535097	-0.2686685
1423893_x_at	amyloid beta (A4) precursor protein-binding, family B, member 1	Apbb1	0.76733	1.11967047	0.15353
1451154_a_at	CUGBP, Elav-like family member 2	Celf2	0.76733	-1.12865456	-0.1909245
1427538_at	zinc finger protein 369	Zfp369	0.76734	1.12573206	0.16296352
1436277_at	ring finger protein 207	Rnf207	0.76736	1.1114629	0.15197215
1447484_x_at	Small nucleolar RNA host gene (non-protein coding) 7	Snhg7	0.76736	-1.10672478	-0.1478761
1424140_at	galactose-4-epimerase, UDP	Gale	0.76736	1.10526151	0.13612151
1460467_at	IQ motif containing G	Iqcg	0.76736	1.10723993	0.14178401
1445808_at	---	---	0.76736	-1.08616924	-0.1258475
1441629_at	---	---	0.76736	-1.11504326	-0.1616461
1425222_x_at	RIKEN cDNA D630002G06 gene	D630002G06Rik	0.76737	1.11429407	0.1478608
1425682_a_at	Tp53rk binding protein	Tprkb	0.76737	-1.08090739	-0.1129222
1421915_a_at	ST3 beta-galactoside alpha-2,3-sialyltransferase 3	St3gal3	0.76737	1.13245108	0.1703581
1455169_at	RAB11 family interacting protein 2 (class I)	Rab11fip2	0.76737	-1.09426287	-0.1311087
1443072_at	---	---	0.76738	-1.1121007	-0.1539023
1419671_a_at	interleukin 17 receptor C	Il17rc	0.76738	1.10612943	0.14144586
1433961_at	cDNA sequence BC023814	BC023814	0.76738	1.09985715	0.13520432
1457510_at	---	---	0.76739	-1.11654447	-0.1638748
1433830_at	heterogeneous nuclear ribonucleoprotein A2/B1	Hnrnpa2b1	0.76739	-1.07319155	-0.1023311
1450606_at	phenylethanolamine-N-methyltransferase	Pnmt	0.76739	1.11455351	0.15237835
1416636_at	Ras homolog enriched in brain	Rheb	0.76739	-1.06874726	-0.0962781
1428457_at	tocopherol (alpha) transfer protein-like	Ttpal	0.7674	1.11338547	0.1530193
1423071_x_at	hypothetical LOC100505088	LOC100505088	0.7674	1.10054537	0.1345455

1452605_at	threonine synthase-like 1 (bacterial)	Thnsl1	0.76741	1.10602503	0.13784007
1451259_at	REX2, RNA exonuclease 2 homolog (S. cerevisiae)	Rexo2	0.76741	1.08463588	0.11550774
1425746_at	RIKEN cDNA D730039F16 gene	D730039F16Rik	0.76741	-1.11158239	-0.1535427
1419079_at	sodium channel, nonvoltage-gated 1 gamma	Scnn1g	0.76742	-1.11452136	-0.1601009
1429534_a_at	inner membrane protein, mitochondrial	Immt	0.76742	1.0763433	0.10537457
1460341_at	pleckstrin homology domain containing, family B (evectins) mem	Plekhb2	0.76743	1.11990804	0.15550233
1421604_a_at	Kruppel-like factor 3 (basic)	Klf3	0.76743	1.08096221	0.11227395
1427359_at	jumonji C domain-containing histone demethylase 1 homolog D (S	Jhdm1d	0.76743	-1.11132191	-0.1612466
1418489_a_at	calcitonin receptor-like	Calcrl	0.76743	-1.11452272	-0.1641214
1438971_x_at	ubiquitin-conjugating enzyme E2H	Ube2h	0.76743	1.10268925	0.13923048
1415943_at	syndecan 1	Sdc1	0.76744	1.08805822	0.11969168
1451014_at	receptor tyrosine kinase-like orphan receptor 1	Ror1	0.76745	1.13434562	0.17333365
1460229_at	stromal antigen 3	Stag3	0.76746	-1.10537807	-0.1568759
1448665_at	dystrophin, muscular dystrophy	Dmd	0.76747	-1.08420814	-0.1198096
1459750_s_at	G protein-coupled receptor 123	Gpr123	0.76747	-1.11070136	-0.1592382
1423231_at	neurogranin	Nrgn	0.76748	-1.12140575	-0.1778671
1460668_at	galanin	Gal	0.76748	-1.08892165	-0.1257482
1434569_at	transcriptional adaptor 2B	Tada2b	0.76751	1.10809601	0.145469
1434624_x_at	ribosomal protein S9	Rps9	0.76751	-1.09110854	-0.1326887
1446879_at	---	---	0.76751	1.12762952	0.16726029
1441902_x_at	solute carrier family 29 (nucleoside transporters), member 4	Slc29a4	0.76751	-1.08514623	-0.1180436
1416069_at	phosphofructokinase, platelet	Pfkp	0.76751	1.0804998	0.10975648
1456939_at	family with sequence similarity 154, member B	Fam154b	0.76752	-1.1158432	-0.1657274
1452512_a_at	ankyrin 1, erythroid	Ank1	0.76752	-1.10400657	-0.1427568
1460639_a_at	ATX1 (antioxidant protein 1) homolog 1 (yeast)	Atox1	0.76752	1.09417101	0.1231062
1448618_at	major vault protein	Mvp	0.76753	1.10496821	0.1402398
1417888_at	tripartite motif-containing 13	Trim13	0.76754	1.08684748	0.11832002
1418567_a_at	signal recognition particle 14	Srp14	0.76754	1.09829663	0.12779551
1447045_at	---	---	0.76754	1.10185469	0.13699737
1452339_at	a disintegrin-like and metallopeptidase (reprolysin type) with thro	Adamts7	0.76755	1.12448795	0.16307623
1449459_s_at	ankyrin repeat and SOCS box-containing 13	Asb13	0.76755	1.12517413	0.16267682
1444375_at	---	---	0.76756	-1.1194114	-0.1673248
1451386_at	biliverdin reductase B (flavin reductase (NADPH))	Blvrb	0.76757	1.10703136	0.13752322
1443148_at	ubiquitin-conjugating enzyme E2K (UBC1 homolog, yeast)	Ube2k	0.76758	-1.13328984	-0.2045207
1416095_x_at	SAP domain containing ribonucleoprotein	Sarnp	0.76758	-1.07066034	-0.0990075
1433416_at	regulatory factor X, 2 (influences HLA class II expression)	Rfx2	0.76758	1.12512479	0.16477681
1455528_at	---	---	0.76758	1.15617887	0.19117515
1447516_at	---	---	0.7676	-1.11390676	-0.1561718
1456425_at	RIKEN cDNA 9230115E21 gene	9230115E21Rik	0.7676	-1.13172745	-0.1955435
1457889_at	expressed sequence AU014972	AU014972	0.7676	1.11772498	0.16041754
1419257_at	transcription elongation factor A (SII) 1	Tcea1	0.76761	-1.09244378	-0.1392436
1439276_at	Adenosine deaminase, RNA-specific	Adar	0.76761	1.12372073	0.15963242
1442282_at	Vomeronasal 2, receptor 84	Vmn2r84	0.76762	1.09169727	0.12589536
1456884_at	RIKEN cDNA 6820445E23 gene /// hypothetical protein LOC1005	6820445E23Rik /// LOC10	0.76763	1.11176785	0.15232558
1452193_a_at	Wiskott-Aldrich syndrome-like (human)	Wasl	0.76764	-1.09590001	-0.1431947
1450830_a_at	phosphodiesterase 6C, cGMP specific, cone, alpha prime	Pde6c	0.76765	1.15388506	0.19191796
1452855_at	lymphocyte antigen 6 complex, locus K	Ly6k	0.76766	-1.11680819	-0.1653022
1437028_at	surfactant associated protein B	Sftpb	0.76766	-1.09973522	-0.1384206
1423001_at	sprouty-related, EVH1 domain containing 2	Spred2	0.76767	-1.11636611	-0.172942
1439701_at	RIKEN cDNA 9330121K16 gene	9330121K16Rik	0.76768	1.0964139	0.13066744
1426468_at	RIKEN cDNA 0610037L13 gene	0610037L13Rik	0.76768	1.09359458	0.12758018
1432697_at	RIKEN cDNA 5830431M20 gene	5830431M20Rik	0.76769	1.10277884	0.13965099
1451556_a_at	RIKEN cDNA 2700078E11 gene	2700078E11Rik	0.7677	1.08900918	0.11975991
1451649_a_at	WD repeat domain 75	Wdr75	0.76771	-1.07968092	-0.1138441
1440521_x_at	hypothetical LOC100503953	LOC100503953	0.76773	1.10965796	0.14933304
1432810_at	tetratricopeptide repeat domain 32	Ttc32	0.76773	-1.11428227	-0.1606418
1418178_at	internexin neuronal intermediate filament protein, alpha	Ina	0.76775	1.13503352	0.16237886
1429907_at	RIKEN cDNA 1700094D03 gene	1700094D03Rik	0.76776	1.11398028	0.15146859
1452738_at	stomatin-like 1	Stoml1	0.76777	1.10171744	0.1334977
1434491_a_at	cytochrome c oxidase, subunit VIc	Cox6c	0.76777	-1.09386515	-0.1443124
1430191_at	RIKEN cDNA 9130004J05 gene	9130004J05Rik	0.76777	1.13429112	0.17244545
1429404_at	RIKEN cDNA 2010317E24 gene	2010317E24Rik	0.76778	1.12169274	0.15579224
1422729_at	protocadherin beta 10	Pcdhb10	0.76783	1.18697805	0.21102517
1429193_at	ankyrin repeat and IBR domain containing 1	Ankib1	0.76783	-1.09815912	-0.1406359
1434928_at	growth arrest-specific 2 like 1	Gas2l1	0.76784	1.09527919	0.12863989
1419698_at	chemokine (C-X-C motif) ligand 11	Cxcl11	0.76785	-1.09153994	-0.1309879
1420643_at	LFNG O-fucosylpeptide 3-beta-N-acetylglucosaminyltransferase	Lfng	0.76786	1.09596925	0.13113094
1451578_at	coiled-coil domain containing 65	Ccdc65	0.76788	1.15004208	0.18782288
1459097_at	---	---	0.76789	-1.133652	-0.1999438
1448760_at	zinc finger protein 68	Zfp68	0.76792	1.10322537	0.14070847
1447347_at	---	---	0.76792	1.1279483	0.16961482
1447893_x_at	solute carrier family 39 (metal ion transporter), member 6	Slc39a6	0.76793	1.10540835	0.14359435
1457378_at	---	---	0.76793	-1.10966495	-0.155106
1417123_at	vav 3 oncogene	Vav3	0.76796	1.1113143	0.15066947
1440795_x_at	rabaptin, RAB GTPase binding effector protein 2	Rabep2	0.76796	1.10396418	0.13571239
1440909_at	immunity-related GTPase family, cinema 1	Irgc1	0.76798	1.11697725	0.15350395
1420045_at	---	---	0.76799	-1.13293282	-0.2060175
1450933_at	phosphodiesterase 7A	Pde7a	0.76802	1.10595817	0.14109933
1442547_at	---	---	0.76802	-1.12087195	-0.1703371
1456193_x_at	glutathione peroxidase 4	Gpx4	0.76802	-1.07405286	-0.1088744
1439373_x_at	wingless-related MMTV integration site 5B	Wnt5b	0.76804	1.12889446	0.17013446
1439946_at	---	---	0.76806	-1.11006937	-0.1528656
1444208_at	---	---	0.7681	-1.12914079	-0.1979693
1449556_at	RIKEN cDNA C920025E04 gene /// histocompatibility 2, T region l	C920025E04Rik /// H2-T2	0.76814	-1.11361765	-0.1580708
1440405_at	---	---	0.76815	1.12482545	0.16366242
1459755_x_at	RIKEN cDNA 1700082C02 gene	1700082C02Rik	0.76819	1.08897586	0.1221563
1419629_at	mesoderm posterior 2	Mesp2	0.76819	-1.10985834	-0.1612761
1434817_s_at	regulation of nuclear pre-mRNA domain containing 2	Rprd2	0.76821	-1.07959898	-0.1111847
1439831_at	---	---	0.76821	1.14833038	0.18438381
1431949_at	RIKEN cDNA 4930544F09 gene	4930544F09Rik	0.76822	-1.119754	-0.1795415
1442518_at	RIKEN cDNA C030044O21 gene	C030044O21Rik	0.76822	-1.10968917	-0.1547653
1425239_at	SET domain containing 4	Setd4	0.76823	1.09231764	0.12464558
1435779_at	centrosomal protein 110	Cep110	0.76824	1.10112465	0.13795604
1444098_at	---	---	0.76825	-1.11413884	-0.1563271

1442762_at	tubulin tyrosine ligase-like family, member 10	Ttll10	0.76825	-1.11816869	-0.1723829
1454018_at	tousled-like kinase 2 (Arabidopsis)	Tlk2	0.76826	-1.08509378	-0.1198428
1426566_s_at	interleukin 17 receptor E	Il17re	0.76827	1.12290564	0.15834809
1421627_at	even skipped homeotic gene 1 homolog	Evx1	0.76829	-1.11974675	-0.1763358
1439862_at	RAR-related orphan receptor beta	Rorb	0.7683	1.12373276	0.15934427
1455305_x_at	heterogeneous nuclear ribonucleoprotein A1	Hnrnpa1	0.7683	-1.0761397	-0.1083638
1440684_at	lysophosphatidylcholine acyltransferase 2	Lpcat2	0.76831	-1.10117763	-0.1403149
1419453_at	ubiquitin carboxyl-terminal esterase L5	Uchl5	0.76831	-1.1087784	-0.1530734
1437933_at	Hedgehog-interacting protein	Hhip	0.76832	-1.13481499	-0.2087475
1419392_at	piccolo (presynaptic cytomatrix protein)	Pclo	0.76832	1.19443889	0.22144194
1444055_at	---	---	0.76832	-1.11197441	-0.153791
1454947_a_at	ubiquitin-like domain containing CTD phosphatase 1	Ublcp1	0.76835	-1.08024166	-0.1142828
1446628_at	---	---	0.76835	-1.11475796	-0.1567749
1438288_x_at	RIKEN cDNA 1110059G02 gene	1110059G02Rik	0.76835	-1.11229675	-0.1549094
1417505_s_at	predicted gene 13305 /// predicted gene 2002 /// interleukin 11	Gm13305 /// Gm2002 ///	0.76836	1.13821837	0.17477871
1450822_at	lysozyme 1	Lyz1	0.76837	1.13309672	0.16913209
1452729_at	dolichyl-phosphate mannosyltransferase polypeptide 3	Dpm3	0.76839	1.15203677	0.18681782
1443986_at	cell division cycle 73, Paf1/RNA polymerase II complex componer	Cdc73	0.76839	-1.10087534	-0.1455956
1418371_at	dynein light chain LC8-type 2	Dynll2	0.76839	1.08871033	0.11887021
1445842_at	---	---	0.7684	1.12160769	0.1612774
1441254_at	par-3 partitioning defective 3 homolog B (C. elegans)	Pard3b	0.76843	1.11344915	0.15399886
1427676_a_at	glutamate receptor, ionotropic, kainate 1	Grik1	0.76844	1.10354067	0.14059753
1439900_at	---	---	0.76848	-1.11546242	-0.1617895
1454123_at	RIKEN cDNA 4933406K04 gene	4933406K04Rik	0.7685	-1.12028482	-0.1734279
1416140_a_at	DEAH (Asp-Glu-Ala-His) box polypeptide 30	Dhx30	0.76852	1.08378322	0.11266273
1437166_at	Terf1 (TRF1)-interacting nuclear factor 2	Tinf2	0.76853	1.16822741	0.19919893
1433214_x_at	RIKEN cDNA 2700008E08 gene	2700008E08Rik	0.76854	1.11614491	0.1564804
1425598_a_at	Yamaguchi sarcoma viral (v-yes-1) oncogene homolog	Lyn	0.76855	1.12412343	0.16032413
1441897_at	RIKEN cDNA B230120H23 gene	B230120H23Rik	0.76855	1.11391265	0.15261202
1446030_at	---	---	0.76857	-1.09005554	-0.1275819
1452589_at	PTK7 protein tyrosine kinase 7	Ptk7	0.76857	1.12567442	0.15709233
1427583_at	RPTOR independent companion of MTOR, complex 2	Rictor	0.76859	-1.12274056	-0.1754655
1419135_at	lymphotoxin B	Ltb	0.76859	-1.10569636	-0.1509227
1447137_at	---	---	0.7686	-1.10279455	-0.1487559
1459535_at	---	---	0.76861	1.11880602	0.15533491
1451160_s_at	poliovirus receptor	Pvr	0.76862	1.10241906	0.13405296
1432499_a_at	ubiquitination factor E4B, UFD2 homolog (S. cerevisiae)	Ube4b	0.76863	1.0939533	0.1230381
1431673_at	RIKEN cDNA 4930557A04 gene	4930557A04Rik	0.76863	-1.102703	-0.1416388
1423552_at	leptin receptor overlapping transcript-like 1	Leprotl1	0.76864	1.08486181	0.11462626
1448575_at	interleukin 7 receptor	Il7r	0.76864	-1.11223348	-0.1562729
1427977_x_at	oogenesin 1	Oog1	0.76864	1.08703382	0.11990118
1426800_at	RAB8B, member RAS oncogene family	Rab8b	0.76865	-1.10912959	-0.1499999
1443997_at	G protein regulated inducer of neurite outgrowth 2	Gprin2	0.76867	1.12014693	0.16076933
1424585_at	RAN binding protein 10	Ranbp10	0.7687	1.10336982	0.13867187
1431419_at	PRELI domain containing 1	Prelid1	0.7687	1.09817317	0.13408342
1446667_at	---	---	0.7687	1.10501587	0.14381829
1459882_at	ASF1 anti-silencing function 1 homolog A (S. cerevisiae)	Asf1a	0.7687	-1.11686417	-0.159845
1424552_at	caspase 8	Casp8	0.76871	-1.11753508	-0.1635898
1428949_at	exportin, tRNA (nuclear export receptor for tRNAs)	Xpot	0.76871	-1.07419303	-0.1061366
1435995_at	mitochondrial ribosomal protein L22	Mrpl22	0.76873	1.07552597	0.10418531
1443159_at	RIKEN cDNA 9130221J17 gene	9130221J17Rik	0.76873	1.10331006	0.14114459
1420116_s_at	golgi phosphoprotein 3	Golph3	0.76873	-1.08054379	-0.1128681
1423902_s_at	Rho guanine nucleotide exchange factor (GEF) 12	Arhgef12	0.76874	-1.08983536	-0.1267104
1420754_at	transcription termination factor, RNA polymerase I	Ttf1	0.76874	-1.09171474	-0.1290213
1440453_at	zinc finger protein 735	Zfp735	0.76874	1.08624564	0.11747883
1430095_at	RIKEN cDNA D930020B18 gene	D930020B18Rik	0.76876	-1.11367183	-0.1566405
1428494_a_at	polymerase (RNA) II (DNA directed) polypeptide I	Polr2i	0.76877	1.08537361	0.11480546
1453683_a_at	centrosomal protein 55	Cep55	0.76878	-1.09058447	-0.1293729
1452538_at	expressed sequence AI324046 /// immunoglobulin heavy chain 6	AI324046 /// Igh-6 /// Igh	0.76879	-1.10192881	-0.1448543
1416778_at	serum deprivation response	Sdpr	0.7688	-1.09636445	-0.1384797
1434147_at	RELT-like 2	Rell2	0.7688	1.10424459	0.14300356
1459337_at	---	---	0.76881	-1.11442958	-0.1615365
1452081_a_at	RIKEN cDNA 9130017N09 gene	9130017N09Rik	0.76882	1.10174355	0.13810652
1433751_at	solute carrier family 39 (zinc transporter), member 10	Slc39a10	0.76884	1.08245951	0.11157405
1458186_at	---	---	0.76884	1.15556866	0.18984765
1444125_at	---	---	0.76886	-1.12157567	-0.176143
1442957_at	RIKEN cDNA G730013B05 gene	G730013B05Rik	0.76889	1.19137227	0.22118333
1456788_at	mitochondrial ribosomal protein L54	Mrpl54	0.76889	-1.11009877	-0.1511661
1423367_at	wingless-related MMTV integration site 7A	Wnt7a	0.7689	-1.12348282	-0.1833093
1449701_at	Eukaryotic translation termination factor 1	Etf1	0.7689	-1.11216028	-0.1609765
1421772_a_at	cytochrome c oxidase subunit VIIa polypeptide 2-like	Cox7a2l	0.76891	1.07392593	0.10256207
1448305_at	RAB6, member RAS oncogene family	Rab6	0.76892	-1.07285949	-0.1015913
1418320_at	protease, serine, 8 (prostasin)	Prss8	0.76892	1.1196473	0.15647097
1416304_at	LPS-induced TN factor	Litaf	0.76893	1.08631506	0.11845021
1436741_at	oocyte specific homeobox 5	Obox5	0.76895	-1.11726595	-0.1732558
1440817_x_at	zinc finger protein 771	Zfp771	0.76896	1.08793232	0.11808603
1435584_at	RIKEN cDNA A630033H20 gene	A630033H20Rik	0.76896	1.08772882	0.12035176
1432359_at	RIKEN cDNA 4930435F05 gene	4930435F05Rik	0.76896	-1.10486264	-0.1445144
1422738_at	discoidin domain receptor family, member 2	Ddr2	0.76896	-1.1073682	-0.1512375
1451118_a_at	family with sequence similarity 53, member A	Fam53a	0.76898	1.08012079	0.11045494
1436989_s_at	solute carrier family 12, member 6	Slc12a6	0.76899	1.12322108	0.16304353
1422455_s_at	N-ethylmaleimide sensitive fusion protein	Nsf	0.76899	-1.08536728	-0.1240983
1439405_x_at	Cyclin N-terminal domain containing 1	Cntd1	0.76902	-1.07361571	-0.1043519
1435433_at	ArfGAP with GTPase domain, ankyrin repeat and PH domain 1	Agap1	0.76903	-1.10314756	-0.1430145
1439343_at	---	---	0.76903	-1.11103906	-0.1576323
1427047_at	nucleoporin 188	Nup188	0.76904	1.07671463	0.10625256
1421419_at	potassium channel, subfamily K, member 4	Kcnk4	0.76905	-1.10603437	-0.1458487
1433067_at	RIKEN cDNA 6330582A15 gene	6330582A15Rik	0.76906	1.14439557	0.18058777
1438375_at	fibulin 2	Fbln2	0.76907	-1.08983305	-0.1291007
1447043_at	---	---	0.76907	-1.12159183	-0.1818864
1458767_at	---	---	0.76909	-1.10893039	-0.1507341
1455430_at	RNA binding motif protein 33	Rbm33	0.7691	1.08766549	0.12049749
1434646_s_at	predicted gene 10094 /// Sin3-associated polypeptide 18	Gm10094 /// Sap18	0.7691	1.07665583	0.10406876
1449402_at	carbohydrate (N-acetylglucosamino) sulfotransferase 7	Chst7	0.76913	-1.11732965	-0.1720986

1454458_at	RIKEN cDNA 6230426I18 gene	6230426I18Rik	0.76914	-1.1193862	-0.1665227
1449095_at	vacuolar protein sorting 54 (yeast)	Vps54	0.7692	-1.08515745	-0.1197098
1451783_a_at	kinesin-associated protein 3	Kifap3	0.76921	1.07418898	0.10280589
1441647_at	hypothetical LOC100504626	LOC100504626	0.76922	-1.10898396	-0.1529403
1417356_at	paternally expressed 3	Peg3	0.76922	-1.10702989	-0.1514598
1418346_at	insulin-like 6	Insl6	0.76923	1.12268981	0.16137348
1445665_at	---	---	0.76924	-1.10390775	-0.142984
1425835_a_at	bobby sox homolog (Drosophila)	Bbx	0.76925	-1.0999836	-0.1433816
1424626_at	RIKEN cDNA 2010003K11 gene	2010003K11Rik	0.76925	-1.10667593	-0.1464136
1421700_at	testis expressed gene 16	Tex16	0.76926	-1.09951019	-0.1387378
1459002_at	ribosomal protein L21 pseudogene /// predicted gene 5445 /// pr	Gm16416 /// Gm5445 ///	0.76927	1.13040627	0.16724966
1446194_at	nuclear receptor subfamily 2, group C, member 1	Nr2c1	0.76928	-1.11622682	-0.1705153
1452646_at	transformation related protein 53 inducible nuclear protein 2	Trp53inp2	0.76928	1.092646	0.1272279
1448964_at	S100 calcium binding protein G	S100g	0.76929	1.11994705	0.15958062
1428395_at	SMAD specific E3 ubiquitin protein ligase 1	Smurf1	0.76932	1.10256343	0.13719374
1448886_at	GATA binding protein 3	Gata3	0.76934	1.10384153	0.14246354
1459414_at	---	---	0.76935	-1.09727645	-0.1339711
1425677_a_at	ankyrin 1, erythroid	Ank1	0.76935	1.10248469	0.13684005
1420484_a_at	vitronectin	Vtn	0.76936	1.15689161	0.19364342
1434146_at	glutamate receptor, ionotropic, AMPA2 (alpha 2)	Gria2	0.76941	1.16931118	0.19254071
1420269_at	RIKEN cDNA 2610201A13 gene	2610201A13Rik	0.76942	1.13443716	0.16934621
1440046_at	family with sequence similarity 199, X-linked	Fam199x	0.76943	1.13098668	0.17074377
1421867_at	nuclear receptor subfamily 3, group C, member 1	Nr3c1	0.76945	1.10314313	0.13693791
1421530_a_at	glutamate receptor, metabotropic 8	Grm8	0.76946	1.11190865	0.15204244
1439398_x_at	nasal embryonic LHRH factor	Nelf	0.76952	-1.10232617	-0.1426064
1438245_at	---	---	0.76954	1.14222243	0.17644052
1429483_at	calcium binding and coiled-coil domain 2	Calcoco2	0.76958	-1.07882924	-0.110935
1421539_at	zinc finger protein of the cerebellum 4	Zic4	0.76962	-1.12496605	-0.1847731
1441153_at	---	---	0.76963	-1.11297347	-0.1641373
1444244_at	---	---	0.76963	1.11870674	0.15580474
1437043_a_at	family with sequence similarity 125, member A	Fam125a	0.76966	1.07943277	0.109576
1416764_at	fission 1 (mitochondrial outer membrane) homolog (yeast)	Fis1	0.76966	1.08409645	0.11363726
1449996_a_at	tropomyosin 3, gamma	Tpm3	0.76968	1.10918886	0.14775033
1420966_at	solute carrier family 25 (mitochondrial carrier ornithine transporte	Slc25a15	0.76969	-1.08221024	-0.1140679
1448969_at	FtsJ homolog 2 (E. coli)	Ftsj2	0.76969	1.08475252	0.11664285
1456891_at	DENN/MADD domain containing 2C	Dennd2c	0.7697	1.07456668	0.10284534
1447921_at	---	---	0.76975	1.1134661	0.15158885
1426093_at	tripartite motif-containing 34	Trim34	0.76975	1.09757548	0.13140175
1454329_at	RIKEN cDNA 4933415J04 gene	4933415J04Rik	0.76976	1.11455812	0.15347795
1451044_at	survival of motor neuron protein interacting protein 1	Sip1	0.76977	-1.09157795	-0.1320626
1440626_at	homeobox D13	Hoxd13	0.76979	-1.11618483	-0.1621485
1423278_at	protein tyrosine phosphatase, receptor type, K	Ptprk	0.76979	1.09363859	0.12744288
1445178_at	SH3 domain containing ring finger 1	Sh3rf1	0.76981	-1.11491652	-0.1627858
1457879_at	RIKEN cDNA A230107O07 gene	A230107O07Rik	0.76982	1.14206368	0.18089296
1427393_at	coagulation factor IX	F9	0.76985	-1.13702838	-0.2233067
1442375_at	---	---	0.76985	-1.11942993	-0.1685857
1460396_at	DEAD (Asp-Glu-Ala-Asp) box polypeptide 54	Ddx54	0.76987	1.08201521	0.11150232
1454942_at	family with sequence similarity 129, member A	Fam129a	0.76991	-1.0931743	-0.1314958
1440769_at	RIKEN cDNA 2010305A19 gene	2010305A19Rik	0.76992	-1.10068292	-0.1386461
1416446_at	transmembrane protein 30A	Tmem30a	0.77029	-1.10315879	-0.1515441
1421809_at	DiGeorge syndrome critical region gene 2	Dgcr2	0.7703	1.11329167	0.14738158
1420352_at	protease, serine, 22	Prss22	0.77031	1.12814312	0.16654768
1447087_at	---	---	0.77034	-1.11299504	-0.157754
1445246_at	G elongation factor, mitochondrial 2	Gfm2	0.77034	1.10878245	0.14719372
1416683_at	plexin B2	Plxnb2	0.77036	1.08145824	0.1126494
1428330_at	dopey family member 2	Dopey2	0.77038	1.1068408	0.14434798
1452201_at	RIKEN cDNA 2310047B19 gene	2310047B19Rik	0.77046	1.09364122	0.12478611
1425360_at	myeloid/lymphoid or mixed-lineage leukemia (trithorax homolog,	Mllt6	0.77048	-1.10330917	-0.1425012
1452000_s_at	seryl-aminoacyl-tRNA synthetase	Sars	0.77048	-1.07756628	-0.1114428
1429876_at	suppressor of Ty 7 (S. cerevisiae)-like	Supt7l	0.7705	1.09208252	0.12526078
1435846_x_at	---	---	0.77052	1.13246641	0.17389587
1438639_x_at	exocyst complex component 3-like 2	Exoc3l2	0.77054	-1.10092398	-0.1412016
1454688_x_at	transmembrane emp24-like trafficking protein 10 (yeast)	Tmed10	0.77058	-1.10840211	-0.1701619
1420582_at	CD209e antigen	Cd209e	0.77058	-1.10245629	-0.1410057
1455490_at	polymeric immunoglobulin receptor	Pigr	0.77068	1.1122765	0.15085078
1460560_at	BAH domain and coiled-coil containing 1	Bahcc1	0.77068	1.09021248	0.12310888
1443840_x_at	RIKEN cDNA 1700010H22 gene	1700010H22Rik	0.77071	-1.09876608	-0.1375241
1458221_at	mastermind-like domain containing 1	Mamld1	0.77074	-1.11298924	-0.1566767
1434167_at	solute carrier family 35, member E4	Slc35e4	0.77077	1.09852081	0.13436181
1442759_at	Thymoma viral proto-oncogene 1	Akt1	0.7708	-1.10666767	-0.1462758
1421833_at	phosphatidylinositol-4-phosphate 5-kinase, type 1 beta	Pip5k1b	0.77087	1.10154623	0.13823608
1429254_at	aquaporin 11	Aqp11	0.77089	1.14429315	0.18090479
1442710_at	---	---	0.77098	-1.14638588	-0.2372257
1447646_at	HEAT repeat containing 2	Heatr2	0.77101	1.12944812	0.16936486
1436632_at	ubinuclein 2	Ubn2	0.77104	-1.09630049	-0.1391632
1417861_at	cyclin C	Ccnc	0.77123	-1.10887745	-0.1646019
1437683_x_at	small EDRK-rich factor 2	Serf2	0.77125	1.08459405	0.11195958
1419072_at	glutathione S-transferase, mu 7	Gstm7	0.77133	-1.1147512	-0.1573565
1419977_s_at	epithelial splicing regulatory protein 2	Esrp2	0.77133	1.12078787	0.16108714
1451387_s_at	cutA divalent cation tolerance homolog (E. coli)	Cuta	0.77137	1.08030429	0.10978022
1440329_s_at	G patch domain containing 2	Gpatch2	0.77138	1.09799607	0.13427139
1435754_at	zyg-ll homolog B (C. elegans)	Zyg11b	0.7714	-1.08342811	-0.1156507
1449674_s_at	programmed cell death 6 interacting protein	Pdcd6ip	0.7714	1.08857102	0.1193442
1441512_at	RIKEN cDNA 6030445D17 gene	6030445D17Rik	0.77141	-1.1193867	-0.1722522
1444018_at	RIKEN cDNA B930098A02 gene /// cysteine-rich hydrophobic dom	B930098A02Rik /// Chic2	0.77143	-1.10889642	-0.1504335
1426742_at	ATP synthase, H+ transporting, mitochondrial F0 complex, subunit	Atp5f1	0.77145	1.06265123	0.08710963
1449198_a_at	ST3 beta-galactoside alpha-2,3-sialyltransferase 5	St3gal5	0.77146	-1.10053122	-0.1393248
1431737_at	predicted gene 7008	Gm7008	0.77147	1.15330707	0.18778864
1445747_at	---	---	0.77147	-1.11457265	-0.157037
1434759_at	leucine rich repeat transmembrane neuronal 3	Lrrtm3	0.77147	1.07999587	0.11065486
1428068_at	sorting and assembly machinery component 50 homolog (S. cere	Samm50	0.77147	1.07015055	0.0962202
1422917_at	Eph receptor A1	Epha1	0.77148	1.11343447	0.14939243
1419639_at	ephrin B2	Efnb2	0.77149	-1.11845624	-0.1735768
1424282_at	PET112-like (yeast)	Pet112l	0.77149	1.08388986	0.11515873

1445990_at	expressed sequence AU020096	AU020096	0.7715	1.09585307	0.12787703
1427490_at	ATP-binding cassette, sub-family B (MDR/TAP), member 7	Abcb7	0.77152	-1.12429751	-0.1957152
1452699_at	methyltransferase 11 domain containing 1	Mett11d1	0.77153	1.08374375	0.11399614
1453152_at	MAM domain containing 2	Mamdc2	0.77153	1.11860073	0.15723381
1446406_at	---	---	0.77155	1.19429652	0.21805906
1444947_at	---	---	0.77155	-1.10842658	-0.150104
1432007_s_at	adaptor protein complex AP-2, alpha 2 subunit	Ap2a2	0.77156	1.10662332	0.14279009
1420518_a_at	immunoglobulin superfamily, member 9	Igsf9	0.77156	1.10003171	0.1339886
1426271_at	structural maintenance of chromosomes 5	Smc5	0.77157	-1.07606734	-0.1064651
1452230_at	DnaJ (Hsp40) homolog, subfamily C, member 10	Dnajc10	0.77157	1.09383192	0.12548257
1456806_at	RIKEN cDNA A130010C12 gene	A130010C12Rik	0.77158	-1.11869333	-0.1729812
1420157_s_at	ATP-binding cassette, sub-family F (GCN20), member 1	Abcf1	0.77159	1.06914555	0.09620663
1431268_at	predicted gene 9802	Gm9802	0.77159	-1.09917438	-0.1367807
1442285_at	synaptic nuclear envelope 2	Syne2	0.7716	-1.11079492	-0.1556864
1432436_a_at	adenylate kinase 3	Ak3	0.77161	1.08968116	0.12364232
1455538_at	RIKEN cDNA 6330403M23 gene	6330403M23Rik	0.77161	-1.09743878	-0.1353865
1439833_at	septin 3	Sep-03	0.77161	-1.10427734	-0.1529881
1440322_at	---	---	0.77161	1.10756455	0.14669702
1422662_at	lectin, galactose binding, soluble 8	Lgals8	0.77163	1.12332276	0.15679613
1443267_at	---	---	0.77163	1.10722934	0.14623982
1418190_at	paraoxonase 1	Pon1	0.77163	-1.11876855	-0.1672978
1435782_at	predicted gene 4802	Gm4802	0.77165	-1.09738078	-0.1383085
1456485_at	nuclear protein in the AT region	Npat	0.77166	-1.0877702	-0.1238707
1424794_at	ring finger protein 186	Rnf186	0.77167	-1.11329287	-0.1581818
1453171_s_at	protein phosphatase 1A, magnesium dependent, alpha isoform	Ppm1a	0.77167	1.08928991	0.12248437
1459458_at	DNA segment, Chr 9, ERATO Doi 338, expressed	D9Ertd338e	0.77167	1.09842562	0.13411958
1424011_at	aquaporin 9	Aqp9	0.77168	-1.11679655	-0.1654293
1434512_x_at	serine/arginine-rich splicing factor 3	Srsf3	0.77168	-1.08097454	-0.1170101
1421328_at	hypothetical protein A730034C02 /// microtubule-associated pro	A730034C02 /// Mtap2	0.77168	-1.10834351	-0.1555134
1425498_at	PRP4 pre-mRNA processing factor 4 homolog B (yeast)	Prpf4b	0.77169	-1.07555283	-0.1055001
1442298_at	---	---	0.77169	-1.10796509	-0.1491857
1444970_at	expressed sequence C79870	C79870	0.77169	-1.10553814	-0.1457865
1458065_at	---	---	0.77169	1.14342191	0.17853806
1449205_at	ovo-like 2 (Drosophila)	Ovol2	0.77169	-1.11268485	-0.1578055
1459966_at	---	---	0.77169	1.10528383	0.14357184
1431449_at	solute carrier organic anion transporter family, member 6d1	Slco6d1	0.7717	-1.08568322	-0.118963
1449515_at	zinc finger protein 292	Zfp292	0.7717	-1.08923591	-0.1276429
1448520_at	DNA cross-link repair 1B, PSO2 homolog (S. cerevisiae)	Dclre1b	0.7717	1.09627535	0.13024687
1456206_at	AlkB, alkylation repair homolog 3 (E. coli)	Alkbh3	0.77171	-1.1238397	-0.1792072
1424539_at	ubiquitin-like 4	Ubl4	0.77171	1.09721845	0.13096335
1449668_s_at	folliculin interacting protein 1	Fnip1	0.77171	-1.08661469	-0.1237808
1443999_at	---	---	0.77171	1.11466556	0.1552531
1459837_at	---	---	0.77172	1.1307756	0.16796427
1416222_at	NAD(P) dependent steroid dehydrogenase-like	Nsdhl	0.77172	1.07885856	0.10804894
1446262_at	---	---	0.77173	-1.1078042	-0.1500042
1440378_at	SEC11 homolog C (S. cerevisiae)	Sec11c	0.77173	1.14345168	0.18402057
1438088_at	---	---	0.77173	1.11998019	0.15691303
1432414_at	predicted gene 13999	Gm13999	0.77174	-1.11515606	-0.1658603
1422217_a_at	cytochrome P450, family 1, subfamily a, polypeptide 1	Cyp1a1	0.77174	-1.11075979	-0.1548465
1456469_x_at	LIM and SH3 protein 1	Lasp1	0.77174	1.10902823	0.1425717
1432947_at	RIKEN cDNA 4921519G19 gene	4921519G19Rik	0.77175	1.18804133	0.21361971
1426232_at	cDNA sequence BC024479	BC024479	0.77176	-1.08410882	-0.1210261
1456765_at	NHS-like 2	Nhsl2	0.77176	1.13016101	0.16787372
1449520_at	tetratricopeptide repeat domain 28	Ttc28	0.77176	-1.11231754	-0.1545007
1442702_at	---	---	0.77177	-1.11137367	-0.1579499
1451510_s_at	oleoyl-ACP hydrolase	Olah	0.77177	1.10348198	0.14058236
1425003_at	urocanase domain containing 1	Uroc1	0.77177	-1.10026051	-0.140287
1448849_at	mitochondrial ribosomal protein L40	Mrpl40	0.77177	1.08005567	0.10775287
1438079_at	zinc finger protein 867	Zfp867	0.77178	1.12265997	0.16078598
1423949_at	ubiquitin-conjugating enzyme E2Z (putative)	Ube2z	0.77178	1.07161492	0.0995278
1459077_at	---	---	0.7718	1.14686871	0.18471624
1448236_at	radixin	Rdx	0.7718	1.0852187	0.11319513
1419839_x_at	PRP19/PSO4 pre-mRNA processing factor 19 homolog (S. cerevisi	Prpf19	0.7718	1.07552371	0.10378931
1449703_at	Zinc finger, AN1-type domain 2A	Zfand2a	0.77181	1.1414881	0.17784722
1440541_at	RIKEN cDNA 2810442I21 gene	2810442I21Rik	0.77182	-1.10231496	-0.1424437
1424506_at	zinc finger protein 768	Zfp768	0.77183	1.16813409	0.20046307
1432639_at	---	---	0.77185	-1.09267853	-0.1305423
1446652_at	RALBP1 associated Eps domain containing protein 2	Reps2	0.77185	-1.10762201	-0.1538354
1429888_a_at	heat shock protein 2	Hspb2	0.77186	1.10814707	0.14413518
1429517_at	zinc finger, FYVE domain containing 20	Zfyve20	0.77186	1.11212177	0.14953129
1416576_at	suppressor of cytokine signaling 3	Socs3	0.77186	1.09054043	0.11959207
1451839_a_at	phosphodiesterase 7A	Pde7a	0.77188	1.09117564	0.12548342
1427262_at	inactive X specific transcripts	Xist	0.77188	-1.10561084	-0.1571877
1442517_a_at	RIKEN cDNA 9630013A20 gene	9630013A20Rik	0.7719	1.11265371	0.15048641
1429443_at	copine IV	Cpne4	0.7719	-1.12134723	-0.1759604
1450458_at	nuclear receptor coactivator 2	Ncoa2	0.77191	1.12883417	0.16871696
1422414_a_at	calmodulin 2	Calm2	0.77191	-1.06984586	-0.0982269
1422280_at	granzyme K	Gzmk	0.77191	1.11053573	0.14753693
1446945_at	---	---	0.77192	1.11333677	0.15226015
1436165_at	LUC7-like 2 (S. cerevisiae)	Luc7l2	0.77193	-1.09090703	-0.1311081
1432849_at	---	---	0.77193	-1.11649375	-0.1681365
1433059_at	RIKEN cDNA 4930439G18 gene	4930439G18Rik	0.77194	-1.12851778	-0.2015643
1460446_at	tryptophan rich basic protein	Wrb	0.77194	-1.0858907	-0.1193344
1447090_s_at	ADP-ribosylation factor-like 1	Arl1	0.77195	1.07486382	0.10369284
1425126_at	neural cell adhesion molecule 1	Ncam1	0.77196	1.12468203	0.16180078
1429931_at	RIKEN cDNA 4930553P18 gene	4930553P18Rik	0.77196	-1.11164534	-0.153769
1451115_at	protein inhibitor of activated STAT 3	Pias3	0.77196	1.09830992	0.12967251
1449889_a_at	OCIA domain containing 1	Ociad1	0.77196	1.10676196	0.135842
1429259_a_at	RIKEN cDNA 1190007I07 gene /// RIKEN cDNA 1810014B01 gene	1190007I07Rik /// 18100	0.77197	1.09595077	0.12717179
1433937_at	transformation related protein 53 binding protein 2	Trp53bp2	0.77199	1.09243662	0.12387543
1460407_at	Spi-B transcription factor (Spi-1/PU.1 related)	Spib	0.77199	-1.12036886	-0.175723
1454939_at	---	---	0.772	-1.12866369	-0.1947105
1453888_at	copine IV	Cpne4	0.77201	-1.10716286	-0.1515686
1451812_at	a disintegrin and metallopeptidase domain 22	Adam22	0.77204	1.1085344	0.14814117

1445486_at	Leucine rich repeat containing 49	Lrrc49	0.77205	-1.10593835	-0.1546885
1440828_x_at	PHD finger protein 7	Phf7	0.77205	1.10140511	0.13544788
1459880_at	heterogeneous nuclear ribonucleoprotein A/B	Hnrnpab	0.77206	-1.11483273	-0.1610934
1450206_at	deleted in liver cancer 1	Dlc1	0.77206	1.1075523	0.14516288
1445212_at	---	---	0.77206	1.15033207	0.18458033
1444398_at	---	---	0.77208	-1.12849691	-0.1964946
1455239_at	RIKEN cDNA 6330512M04 gene	6330512M04Rik	0.77208	1.11438664	0.14680378
1438328_at	host cell factor C2	Hcfc2	0.7721	-1.12009897	-0.173741
1441106_at	zinc finger, AN1-type domain 5	Zfand5	0.7721	1.11554204	0.15441845
1454619_at	lipase maturation factor 2	Lmf2	0.7721	1.07743627	0.10758025
1446521_at	---	---	0.77211	-1.11442017	-0.1582649
1442470_at	---	---	0.77211	1.13076806	0.17075099
1438581_at	sperm antigen with calponin homology and coiled-coil domains 1-	Specc1l	0.77213	-1.11527267	-0.1636266
1436710_at	zinc finger, SWIM domain containing 4	Zswim4	0.77213	1.0931413	0.12824458
1425043_s_at	coiled-coil domain containing 163	Ccdc163	0.77213	1.11495984	0.15219846
1455059_at	helicase with zinc finger domain	Helz	0.77214	-1.09884324	-0.1380102
1443517_at	---	---	0.77214	1.19067394	0.21953381
1427095_at	CUB domain containing protein 1	Cdcp1	0.77215	-1.11163709	-0.1541667
1447164_at	---	---	0.77216	-1.13671542	-0.2161111
1440069_at	---	---	0.77218	-1.10785822	-0.1484551
1415727_at	apolipoprotein A-I binding protein	Apoa1bp	0.77218	1.08729897	0.11626089
1449628_s_at	START domain containing 7	Stard7	0.77219	1.07899569	0.10801971
1446926_at	PYD and CARD domain containing	Pycard	0.77219	1.11027643	0.14725435
1444305_at	myosin, heavy chain 7B, cardiac muscle, beta	Myh7b	0.7722	1.11693076	0.15372319
1436918_at	dual-specificity tyrosine-(Y)-phosphorylation regulated kinase 2	Dyrk2	0.7722	1.09625565	0.12964994
1425311_at	RIKEN cDNA 4930432F04 gene	4930432F04Rik	0.77221	1.14156429	0.17957756
1451082_at	FtsJ methyltransferase domain containing 2	Ftsjd2	0.77222	1.0795667	0.10965919
1429796_at	kalirin, RhoGEF kinase	Kalrn	0.77222	-1.09897906	-0.137701
1450635_at	alkaline phosphatase, placental-like 2	Alppl2	0.77223	1.10162848	0.13725354
1419113_at	adaptor protein complex AP-1, gamma 2 subunit	Ap1g2	0.77226	1.10010832	0.13356726
1440787_s_at	Bardet-Biedl syndrome 10 (human)	Bbs10	0.77227	1.10776065	0.14349678
1452910_at	BCL6 interacting corepressor	Bcor	0.77227	1.11129927	0.1469553
1457446_at	opioid binding protein/cell adhesion molecule-like	Opcml	0.77228	1.11625789	0.15429012
1421561_at	basic helix-loop-helix family, member e23	Bhlhe23	0.7723	-1.1114817	-0.1573532
1428863_at	ankyrin repeat domain 39	Ankrd39	0.77234	1.10538837	0.13862887
1419591_at	gasdermin C	Gsdmc	0.77234	-1.13177468	-0.1982829
1428588_a_at	mitochondrial ribosomal protein L41	Mrpl41	0.77235	1.09600454	0.12520599
1426611_at	proteasome (prosome, macropain) 26S subunit, ATPase 2	Psmc2	0.77237	1.07196091	0.09836803
1443728_at	---	---	0.7724	1.08108779	0.11204692
1434714_at	ERO1-like beta (S. cerevisiae)	Ero1lb	0.77241	1.09615698	0.13207241
1445271_at	RIKEN cDNA 9230105E10 gene	9230105E10Rik	0.77241	1.10530022	0.14393295
1450336_at	SET domain containing 1A	Setd1a	0.77242	-1.11433745	-0.1656445
1444986_at	---	---	0.77244	1.09594802	0.13001536
1417649_at	cyclin-dependent kinase inhibitor 1C (P57)	Cdkn1c	0.77244	1.08421588	0.11428473
1450857_a_at	collagen, type I, alpha 2	Col1a2	0.77244	-1.10959125	-0.1516321
1431256_at	RIKEN cDNA 5033423O07 gene	5033423O07Rik	0.77247	1.10921545	0.14917467
1442077_at	RIKEN cDNA 2310076G05 gene	2310076G05Rik	0.77247	-1.13299305	-0.2146349
1437185_s_at	thymosin, beta 10	Tmsb10	0.7725	1.03906913	0.05447372
1454683_at	splicing factor, suppressor of white-apricot homolog (Drosophila)	Sfswap	0.7725	-1.10514492	-0.1464112
1460363_at	trinucleotide repeat containing 6C	Tnrc6c	0.7725	-1.08083639	-0.1127499
1442373_at	---	---	0.77251	1.10306898	0.13725292
1421509_at	VPS10 domain receptor protein SORCS 1	Sorcs1	0.77251	1.11913124	0.16021967
1459539_at	Polymerase (DNA directed), eta (RAD 30 related)	Polh	0.77253	1.12035538	0.1588474
1457514_at	YLP motif containing 1	Ylpm1	0.77253	-1.11466209	-0.1744652
1450948_a_at	mitochondrial ribosomal protein L1	Mrpl1	0.77253	-1.09356752	-0.1357933
1446394_at	opioid receptor, kappa 1	Oprk1	0.77255	-1.10421724	-0.1432319
1439732_at	---	---	0.77255	1.08513303	0.11606459
1455543_at	kelch-like 18 (Drosophila)	Klhl18	0.77256	1.14178445	0.17529962
1444766_at	ataxin 7-like 1	Atxn7l1	0.77256	1.13189904	0.16766996
1436902_x_at	thymosin, beta 10	Tmsb10	0.77256	1.13549347	0.15949238
1438980_x_at	peptidase M20 domain containing 1	Pm20d1	0.77257	1.12512333	0.16280169
1442659_at	protocadherin 9	Pcdh9	0.77258	-1.11449215	-0.159003
1439772_at	DNA segment, Chr 1, Brigham & Women's Genetics 0212 express	D1Bwg0212e	0.77258	1.11012021	0.14163737
1425171_at	rhodopsin	Rho	0.77258	1.11228333	0.14993963
1447631_at	MYST histone acetyltransferase 2	Myst2	0.77259	-1.13706111	-0.2187249
1425376_at	arachidonate 8-lipoxygenase	Alox8	0.77259	-1.11781514	-0.1756304
1443768_at	Ras homolog gene family, member A	Rhoa	0.7726	-1.13529077	-0.2274638
1445491_at	expressed sequence C79685	C79685	0.77261	-1.09970432	-0.1616648
1434834_at	suppressor of cytokine signaling 7	Socs7	0.77261	1.10426675	0.13835733
1423028_at	interferon alpha 2	Ifna2	0.77263	1.13569964	0.17307114
1441529_at	---	---	0.77264	1.11735637	0.15505131
1440027_at	MON2 homolog (yeast)	Mon2	0.77264	-1.10726244	-0.1516917
1451367_at	COP9 (constitutive photomorphogenic) homolog, subunit 6 (Arabi	Cops6	0.77264	1.11556981	0.1525717
1439649_at	arginine decarboxylase	Adc	0.77264	-1.09767743	-0.1388283
1454125_a_at	WD repeat domain 69	Wdr69	0.77264	1.1116484	0.14792912
1423158_at	glucosamine-phosphate N-acetyltransferase 1	Gnpnat1	0.77265	-1.0747218	-0.105019
1453257_at	1-acylglycerol-3-phosphate O-acyltransferase 5 (lysophosphatidi	Agpat5	0.77266	1.07677522	0.10610663
1427358_a_at	death associated protein kinase 1	Dapk1	0.77268	1.08282826	0.11369395
1437045_at	mitogen-activated protein kinase 8	Mapk8	0.77268	-1.11686115	-0.1674305
1444177_at	Predicted gene 6648	E330020D12Rik	0.77268	1.17824531	0.20842414
1442952_at	---	---	0.77269	1.17872718	0.20876847
1424556_at	pyrroline-5-carboxylate reductase 1	Pycr1	0.77269	-1.083773	-0.1227767
1426145_at	---	---	0.77269	-1.10173006	-0.1436326
AFFX-ThrX-5_at	---	---	0.7727	-1.12486678	-0.1888037
1454643_at	ubiquitin associated protein 2-like	Ubap2l	0.7727	1.07701438	0.10694679
1455737_at	protein phosphatase 1H (PP2C domain containing)	Ppm1h	0.77271	-1.1225309	-0.1773756
1438647_x_at	centrin 2	Cetn2	0.77271	-1.08277989	-0.1174358
1457638_x_at	replication factor C (activator 1) 2	Rfc2	0.77272	1.11918214	0.15481303
1424750_at	zinc finger and BTB domain containing 1	Zbtb1	0.77272	-1.09242863	-0.1300988
1420490_at	kallikrein 1-related peptidase b16	Klk1b16	0.77273	1.10531658	0.14003944
1416585_at	RuvB-like protein 1	Ruvbl1	0.77274	1.0804982	0.10885097
1438974_x_at	phosphatidylinositol transfer protein, membrane-associated 1	Pitpnm1	0.77274	1.23346406	0.24966772
1447325_at	---	---	0.77274	1.13280281	0.16949319
1417916_a_at	fractured callus expressed transcript 1	Fxc1	0.77275	1.10535327	0.13367963

1418307_a_at	spermine binding protein /// spermine binding protein-like	Sbp /// Sbpl	0.77275	-1.11135221	-0.1591409
1457299_at	glutamate receptor, metabotropic 4	Grm4	0.77275	1.11400514	0.15389657
1416284_at	mitochondrial ribosomal protein L28	Mrpl28	0.77275	1.08086746	0.10970575
1452017_at	SRY-box containing gene 15	Sox15	0.77275	1.14011883	0.16973756
1455268_at	DPH3 homolog (KTI11, S. cerevisiae)	Dph3	0.77276	1.10607701	0.14264717
1441959_s_at	JNK1/MAPK8-associated membrane protein	Jkamp	0.77276	-1.10816702	-0.1590976
1416834_x_at	NADH dehydrogenase (ubiquinone) 1 beta subcomplex, 2	Ndufb2	0.77277	1.11740252	0.1449842
1449451_at	serine (or cysteine) peptidase inhibitor, clade B (ovalbumin), mem	Serpinb11	0.77277	-1.12129159	-0.1827763
1448718_at	RIKEN cDNA 2400001E08 gene	2400001E08Rik	0.77277	1.12249045	0.15003396
1441713_at	anaphase promoting complex subunit 7	Anapc7	0.77278	-1.11476702	-0.1674048
1444596_at	paired box gene 7	Pax7	0.77278	-1.10040927	-0.1381218
1418504_at	heat shock protein 9	Hspa9	0.77278	-1.05957209	-0.084675
1444646_at	basonuclin 2	Bnc2	0.77279	1.10228604	0.13596627
1424625_a_at	DENN/MADD domain containing 1A	Dennd1a	0.77279	1.11440848	0.15268048
1451073_at	signal peptide peptidase 3	Sppl3	0.7728	1.08073271	0.11066856
1423827_s_at	nucleolar complex associated 4 homolog (S. cerevisiae)	Noc4l	0.7728	1.07661242	0.10485894
1422920_at	claudin 13	Cldn13	0.7728	-1.11031984	-0.1546187
1453785_at	tafazzin	Taz	0.7728	-1.09884181	-0.1384538
1432431_s_at	MACRO domain containing 2	Macrod2	0.77281	1.08899533	0.12136323
1420776_a_at	AU RNA binding protein/enoyl-coenzyme A hydratase	Auh	0.77281	1.10797103	0.14420988
1427112_at	tubulin tyrosine ligase	Ttl	0.77282	1.09315362	0.12838616
1428852_at	dedicator of cyto-kinesis 3	Dock3	0.77284	1.16221628	0.19406762
1425727_at	claudin 19	Cldn19	0.77284	-1.1190489	-0.1733983
1457000_at	cDNA sequence BC060267	BC060267	0.77284	1.11627552	0.15262805
1452774_at	heterogeneous nuclear ribonucleoprotein A3	Hnrnpa3	0.77285	-1.07578022	-0.1083215
1454887_at	p21 protein (Cdc42/Rac)-activated kinase 2	Pak2	0.77285	1.08598412	0.11764998
1429075_a_at	RIKEN cDNA 1700018B08 gene	1700018B08Rik	0.77286	-1.12036523	-0.1752196
1442600_at	---	---	0.77286	1.12659544	0.16527722
1456264_at	COMM domain containing 7	Commd7	0.77286	1.13483462	0.1730505
1450949_at	katanin p60 (ATPase-containing) subunit A1	Katna1	0.77286	1.08161621	0.111067
1446823_at	DNA segment, Chr 4, ERATO Doi 103, expressed	D4Ertd103e	0.77288	-1.08935102	-0.1301461
1424136_a_at	peptidyl-prolyl cis-trans isomerase H-like /// peptidyl prolyl isome	LOC433064 /// Ppih	0.77289	1.08212841	0.11109401
1456249_x_at	Synaptophysin	Syp	0.77289	-1.11991384	-0.1782998
1429604_at	RIKEN cDNA 1810011H11 gene	1810011H11Rik	0.7729	1.11949725	0.15805394
1453771_at	GULP, engulfment adaptor PTB domain containing 1	Gulp1	0.77292	-1.1088364	-0.1504178
1432173_at	serine (or cysteine) peptidase inhibitor, clade B (ovalbumin), mem	Serpinb12	0.77292	-1.10604971	-0.1477433
1449708_s_at	checkpoint kinase 1 homolog (S. pombe)	Chek1	0.77292	-1.0769931	-0.1071801
1440753_at	---	---	0.77294	1.11549595	0.15483504
1439066_at	angiopoietin 1	Angpt1	0.77294	1.10646191	0.1429418
1443767_at	---	---	0.77295	1.08152612	0.11219432
1420436_x_at	testis expressed gene 21	Tex21	0.77295	1.16560255	0.1969834
1446504_at	---	---	0.77295	1.128897	0.16446741
1422022_at	zinc finger protein 319	Zfp319	0.77295	-1.11359438	-0.1577154
1429339_a_at	acyl-Coenzyme A dehydrogenase family, member 10	Acad10	0.77298	1.11178584	0.14603053
1431007_at	predicted gene 3435	Gm3435	0.77298	1.09414118	0.12573608
1418837_at	quinolinate phosphoribosyltransferase	Qprt	0.77299	-1.09761867	-0.1360381
1445896_at	RIKEN cDNA 2810433D01 gene	2810433D01Rik	0.77299	1.09444208	0.12905137
1428539_at	SMG1 homolog, phosphatidylinositol 3-kinase-related kinase (C.	Smg1	0.77301	-1.09523804	-0.1398917
1438505_s_at	ribonuclease 3-like /// ribonuclease III, nuclear	LOC100045148 /// Rnase	0.77301	-1.07336818	-0.1036954
1423129_at	soc-2 (suppressor of clear) homolog (C. elegans)	Shoc2	0.77303	-1.0784286	-0.1105734
1435205_at	transcription factor AP-2, epsilon	Tcfap2e	0.77304	1.15407204	0.19097821
1451902_at	zinc finger protein 758	Zfp758	0.77306	-1.11110628	-0.1527047
1451468_s_at	exportin 5	Xpo5	0.77307	1.07379421	0.10245836
1450979_at	carcinoembryonic antigen-related cell adhesion molecule 14	Ceacam14	0.77308	1.09035835	0.11844446
1456163_at	family with sequence similarity 72, member A	Fam72a	0.77315	1.097843	0.12887684
1421170_a_at	phospholipase C, beta 1	Plcb1	0.77316	-1.14242856	-0.2262598
1451679_at	RIKEN cDNA 6530401D17 gene	6530401D17Rik	0.77316	1.09181544	0.12258535
1435416_x_at	phosphatidylinositol glycan anchor biosynthesis, class Q	Pigq	0.77318	1.08037267	0.11021293
1440306_at	hypothetical LOC100503998	LOC100503998	0.77319	1.13079402	0.16959124
1459937_at	Argininosuccinate synthetase 1	Ass1	0.7732	-1.1087008	-0.1489841
1418528_a_at	defender against cell death 1	Dad1	0.77321	1.07976794	0.10798719
1417933_at	insulin-like growth factor binding protein 6	Igfbp6	0.77322	-1.11224611	-0.1647906
1452832_s_at	CDP-diacylglycerol synthase (phosphatidate cytidylyltransferase)	Cds2	0.77322	1.11153961	0.14355602
1421424_a_at	alanyl (membrane) aminopeptidase	Anpep	0.77322	1.09568294	0.13056828
1449797_x_at	---	---	0.77323	-1.11125024	-0.1549296
1421300_at	adenosine deaminase, RNA-specific, B2	Adarb2	0.77323	1.12434587	0.16172362
1427418_a_at	hypoxia inducible factor 1, alpha subunit	Hif1a	0.77324	-1.0823989	-0.1200668
1436301_at	dual serine/threonine and tyrosine protein kinase	Dstyk	0.77325	1.09072963	0.12443822
1433732_x_at	insulin-like growth factor 2 mRNA binding protein 3	Igf2bp3	0.77326	-1.10555259	-0.1585448
1454829_at	RUN domain containing 1	Rundc1	0.77328	1.10810773	0.14434133
1423133_at	CWC15 homolog (S. cerevisiae)	Cwc15	0.77329	1.07443573	0.10244518
1438861_at	basonuclin 2	Bnc2	0.7733	1.07892172	0.10953704
1451779_at	pyridine nucleotide-disulphide oxidoreductase domain 1	Pyroxd1	0.77332	1.13602606	0.16865198
1451427_a_at	EGF-like domain 7	Egfl7	0.77337	1.12280577	0.15238235
1419604_at	Z-DNA binding protein 1	Zbp1	0.77337	1.10508756	0.13887947
1420107_at	Bardet-Biedl syndrome 4 (human)	Bbs4	0.77343	-1.10894029	-0.150879
1448913_at	SWI/SNF related, matrix associated, actin dependent regulator of	Smarcd1	0.77346	1.09302506	0.12143231
1455807_at	testis-specific protein, Y-encoded-like 5	Tspyl5	0.77349	-1.11365081	-0.1563609
1418426_at	general transcription factor IIA, 1-like	Gtf2a1l	0.77357	-1.10990954	-0.1510469
1423431_a_at	MYB binding protein (P160) 1a	Mybbp1a	0.77358	1.06236277	0.08665506
1417653_at	parvalbumin	Pvalb	0.77367	1.16048986	0.19219456
1438080_at	mitochondrial ribosomal protein L11	Mrpl11	0.77367	-1.11229973	-0.1551417
1456303_at	hypothetical LOC100504223	LOC100504223	0.77368	1.1170175	0.15525813
1456306_a_at	uromodulin	Umod	0.77368	-1.07120431	-0.1013302
1443355_at	---	---	0.77368	-1.12576968	-0.194562
1423932_at	CUGBP, Elav-like family member 1	Celf1	0.77368	-1.09820108	-0.1375108
1458321_at	phosphatidylinositol 3-kinase catalytic delta polypeptide	Pik3cd	0.77368	-1.0966504	-0.1371342
1426826_at	RNA binding motif protein 16	Rbm16	0.77369	1.08994865	0.11818963
1455464_x_at	uroplakin 1B	Upk1b	0.77369	-1.10901647	-0.1513562
1419876_at	RIKEN cDNA 2810449G22 gene	2810449G22Rik	0.77371	1.0934152	0.12777742
1454904_at	X-linked myotubular myopathy gene 1	Mtm1	0.77374	-1.04427007	-0.0639445
1451232_at	CD151 antigen	Cd151	0.77375	1.08427923	0.11448129
1453401_at	RIKEN cDNA 4930428O21 gene	4930428O21Rik	0.77376	1.12453727	0.16189852
1454335_at	RIKEN cDNA 9130214F15 gene	9130214F15Rik	0.7738	1.09062959	0.12503951

1445968_at	---	---	0.77383	1.10078189	0.13846698
1456195_x_at	integrin beta 5	Itgb5	0.77387	1.10270153	0.13597404
1425621_at	tripartite motif-containing 35	Trim35	0.77388	1.08926096	0.12097652
1437164_x_at	ATP synthase, H+ transporting, mitochondrial F1 complex, O subu	Atp5o	0.7739	-1.12397155	-0.2227112
1426706_s_at	xylulokinase homolog (H. influenzae)	Xylb	0.7739	-1.10607419	-0.1564697
1417767_at	cytochrome b5 type B	Cyb5b	0.77391	1.07695086	0.10585274
1428782_a_at	ubiquinol-cytochrome c reductase core protein 1	Uqcrc1	0.77391	1.06444574	0.08873941
1444241_at	WW domain containing adaptor with coiled-coil	Wac	0.77392	1.10511526	0.14292831
1421262_at	lipase, endothelial	Lipg	0.77392	-1.11976162	-0.1774921
1436886_x_at	XPA binding protein 2	Xab2	0.77394	1.0829956	0.11092195
1447755_at	---	---	0.77394	1.10060568	0.13088758
1454906_at	retinoic acid receptor, beta	Rarb	0.77396	-1.11467049	-0.1628177
1434782_at	ubiquitin specific peptidase 42	Usp42	0.77397	-1.0827337	-0.1155521
1453563_at	NmrA-like family domain containing 1	Nmral1	0.77397	1.1068733	0.14582504
1422119_at	RAB5B, member RAS oncogene family	Rab5b	0.77398	-1.11099694	-0.1661436
1428050_a_at	transmembrane BAX inhibitor motif containing 4	Tmbim4	0.774	1.07741121	0.10726703
1440011_at	---	---	0.77401	-1.10961948	-0.1656686
1443084_at	cathepsin O	Ctso	0.77402	-1.08796289	-0.1279509
1420117_at	---	---	0.77402	-1.12756685	-0.1977527
1440863_at	---	---	0.77403	1.12008377	0.15391865
1435631_x_at	exocyst complex component 6	Exoc6	0.77404	1.10623768	0.14526441
1434659_at	AVL9 homolog (S. cerevisiase)	Avl9	0.77404	-1.09039837	-0.1256552
1424935_at	ganglioside-induced differentiation-associated protein 1-like 1	Gdap1l1	0.77407	1.10932955	0.14841346
1451414_at	NOP16 nucleolar protein homolog (yeast)	Nop16	0.77407	1.12223273	0.16466897
1451304_at	transmembrane protein 143	Tmem143	0.77411	1.12416527	0.15703093
1450432_s_at	MUS81 endonuclease homolog (yeast)	Mus81	0.77413	1.09298594	0.12733285
1458319_at	wntless homolog (Drosophila)	Wls	0.77414	-1.09718533	-0.1393358
1433815_at	janus kinase and microtubule interacting protein 1	Jakmip1	0.77415	1.19359225	0.22182754
1455372_at	cytoplasmic polyadenylation element binding protein 3	Cpeb3	0.77416	-1.10244468	-0.1407719
1432804_at	RIKEN cDNA A230101C19 gene	A230101C19Rik	0.77416	1.13166612	0.1690933
1426990_at	cubilin (intrinsic factor-cobalamin receptor)	Cubn	0.77417	1.17631805	0.20630434
1458792_at	DNA segment, Chr 12, ERATO Doi 208, expressed	D12Ertd208e	0.77419	-1.11056803	-0.1545516
1439875_at	zinc finger protein 128	Zfp128	0.7742	1.10246227	0.13804172
1416863_at	abhydrolase domain containing 8	Abhd8	0.77421	1.0954368	0.13096392
1440912_at	ring finger protein 39	Rnf39	0.77421	1.10043932	0.13770755
1427341_at	Bardet-Biedl syndrome 9 (human)	Bbs9	0.77424	1.14816945	0.18120299
1422024_at	Friend leukemia integration 1	Fli1	0.77425	1.1332643	0.16877763
1456108_x_at	ring finger protein 112	Rnf112	0.77425	1.11081397	0.14291685
1444806_at	---	---	0.77426	1.14254782	0.17651982
1437386_at	leucine rich repeat and Ig domain containing 1	Lingo1	0.77431	-1.10223062	-0.1404465
1454345_at	RIKEN cDNA 1700081H04 gene	1700081H04Rik	0.77434	1.10232066	0.1387083
1427444_at	ATP-binding cassette, sub-family F (GCN20), member 1	Abcf1	0.77434	1.10269578	0.1394825
1459991_at	myosin IXa	Myo9a	0.77438	1.10680084	0.14514429
1436823_x_at	hemoglobin Y, beta-like embryonic chain /// hemoglobin subunit	Hbb-y /// LOC100503273	0.7744	1.136207	0.1654426
1420142_s_at	proliferation-associated 2G4	Pa2g4	0.77442	1.08245712	0.11029316
1453216_at	glutamic pyruvic transaminase, soluble	Gpt	0.77443	1.11002383	0.14332193
1439659_at	mediator complex subunit 20 /// ubiquitin specific peptidase 49	Med20 /// Usp49	0.77447	1.10308245	0.13683972
1426666_a_at	Sad1 and UNC84 domain containing 1	Sun1	0.77448	1.07766592	0.10667433
1447310_at	aminoglycoside phosphotransferase domain containing 1	Agphd1	0.77449	-1.10787178	-0.1481665
1448902_at	tetratricopeptide repeat domain 23	Ttc23	0.7745	-1.11800259	-0.1724983
1453988_a_at	insulin degrading enzyme	Ide	0.7745	-1.0757898	-0.1086698
1456486_at	zinc finger protein 574	Zfp574	0.77452	1.10032068	0.13414746
1450135_at	frizzled homolog 3 (Drosophila)	Fzd3	0.77453	1.1477558	0.18066331
1424718_at	microtubule-associated protein tau	Mapt	0.77453	-1.10283656	-0.145833
1433502_s_at	TSR1, 20S rRNA accumulation, homolog (yeast)	Tsr1	0.77454	1.07361672	0.10217408
1428151_x_at	cysteine conjugate-beta lyase 1	Ccbl1	0.77454	1.13848618	0.17415297
1440850_at	ring finger protein, LIM domain interacting	Rlim	0.77454	1.12363215	0.16095035
1426296_at	RAD52 homolog (S. cerevisiae)	Rad52	0.77455	1.12186153	0.1562548
1453763_at	thioredoxin domain containing 11	Txndc11	0.77456	1.11249874	0.15120667
1438910_a_at	stomatin	Stom	0.77457	1.11312363	0.14894926
1450058_at	aspartate-beta-hydroxylase	Asph	0.77459	1.11544323	0.14851911
1447740_at	RIKEN cDNA 1700034J05 gene	1700034J05Rik	0.77459	-1.08967693	-0.1308949
1423868_at	thioredoxin reductase 3	Txnrd3	0.77459	1.09538057	0.13009189
1430829_s_at	fat mass and obesity associated	Fto	0.7746	-1.1028809	-0.147976
1448548_at	tubby like protein 4	Tulp4	0.7746	1.10782869	0.1422022
1430046_at	OTU domain containing 3	Otud3	0.77461	1.09747701	0.13096107
1453042_at	ankyrin repeat and sterile alpha motif domain containing 4B	Anks4b	0.77461	-1.13815264	-0.2298433
1429412_at	enhancer of yellow 2 homolog (Drosophila)	Eny2	0.77462	-1.12320464	-0.1860369
1417883_at	glutathione S-transferase, theta 2	Gstt2	0.77462	1.11835341	0.15007948
1456780_at	---	---	0.77462	-1.11274136	-0.1573867
1448251_at	RIKEN cDNA 9030425E11 gene	9030425E11Rik	0.77463	-1.10169773	-0.160558
1420984_at	phosphatidylcholine transfer protein	Pctp	0.77463	1.1064836	0.14294178
1459847_x_at	glial cell line derived neurotrophic factor family receptor alpha 2	Gfra2	0.77463	1.10402099	0.14207397
1445866_at	microtubule associated serine/threonine kinase family member 4	Mast4	0.77464	1.1752806	0.20757701
1419654_at	transducin-like enhancer of split 3, homolog of Drosophila E(spl)	Tle3	0.77465	-1.123182	-0.1962208
1458670_at	adenosine deaminase, RNA-specific, B2	Adarb2	0.77465	1.14290767	0.17066892
1449422_at	cadherin 4	Cdh4	0.77465	1.15901236	0.1911672
1455073_at	cytidine and dCMP deaminase domain containing 1	Cdadc1	0.77465	1.12998132	0.16399121
1443673_x_at	---	---	0.77466	1.12270483	0.16029388
1447714_x_at	ATP synthase, H+ transporting, mitochondrial F0 complex, subunit	Atp5g2	0.77467	1.08837669	0.12027248
1432803_at	RIKEN cDNA A230101C19 gene	A230101C19Rik	0.77469	-1.09255673	-0.1327919
1451150_at	zinc finger protein 410	Zfp410	0.77469	1.09654169	0.13011286
1456766_at	coiled-coil domain containing 151	Ccdc151	0.77469	1.115301	0.15160062
1416381_a_at	peroxiredoxin 5	Prdx5	0.77469	1.08788579	0.11754222
1425651_at	nonhomologous end-joining factor 1	Nhej1	0.7747	-1.0949653	-0.1385986
1427931_s_at	pyridoxal (pyridoxine, vitamin B6) kinase	Pdxk	0.7747	1.10498354	0.13687479
1451834_at	calcium channel, voltage-dependent, beta 1 subunit	Cacnb1	0.77472	-1.11244406	-0.158533
1451004_at	activin receptor IIA	Acvr2a	0.77472	1.11413862	0.14866926
1426155_a_at	odd-skipped related 2 (Drosophila)	Osr2	0.77472	1.11144394	0.14451083
1444826_at	---	---	0.77472	1.1218743	0.15870103
1433456_at	expressed sequence AU022751	AU022751	0.77473	-1.09822836	-0.1373895
1444117_at	Adhesion molecule with Ig like domain 1	Amigo1	0.77473	-1.11186575	-0.1565471
1456584_x_at	3-phosphoglycerate dehydrogenase	Phgdh	0.77475	-1.07508879	-0.1066627
1434779_at	cerebellin 2 precursor protein	Cbln2	0.77475	-1.11280718	-0.1592786

1441806_at	---	---	0.77475	-1.10960359	-0.1530952
1434126_at	RIKEN cDNA 4930402H24 gene	4930402H24Rik	0.77476	1.11613338	0.15514117
1454487_at	RIKEN cDNA 5830490A04 gene	5830490A04Rik	0.77476	1.14396033	0.18085506
1440366_at	---	---	0.77476	1.15402739	0.18553619
1451051_a_at	SCY1-like 1 (S. cerevisiae)	Scyl1	0.77477	1.08969262	0.12167082
1448459_at	Kv channel-interacting protein 1	Kcnip1	0.77477	1.10554465	0.14346893
1417008_at	carnitine acetyltransferase	Crat	0.77477	-1.10883835	-0.150772
1447837_x_at	polymerase (DNA directed), eta (RAD 30 related)	Polh	0.77478	-1.11144568	-0.1724983
1445626_at	---	---	0.77481	1.16392228	0.19458865
1454288_at	RIKEN cDNA 5830468K08 gene	5830468K08Rik	0.77481	1.10182481	0.1397861
1457777_at	gasdermin A	Gsdma	0.77481	1.14119412	0.17476299
1452664_a_at	transmembrane 7 superfamily member 3	Tm7sf3	0.77481	1.08379668	0.11451834
1447096_at	---	---	0.77481	1.11954804	0.15419284
1427098_at	WW domain containing E3 ubiquitin protein ligase 1	Wwp1	0.77481	-1.10512647	-0.1572455
1443624_at	---	---	0.77482	1.10910531	0.14902532
1434510_at	3'-phosphoadenosine 5'-phosphosulfate synthase 2	Papss2	0.77483	1.12584447	0.16651627
1417040_a_at	BCL2-related ovarian killer protein	Bok	0.77484	1.08332341	0.11536454
1418726_a_at	troponin T2, cardiac	Tnnt2	0.77484	1.1384244	0.16780327
1441921_x_at	estrogen related receptor, beta	Esrrb	0.77486	-1.07777635	-0.1101211
1421528_a_at	mediator complex subunit 22	Med22	0.7749	1.10698267	0.13738177
1454604_s_at	tetraspanin 12	Tspan12	0.77491	1.09791292	0.13238167
1452613_at	ubiquinol-cytochrome c reductase, complex III subunit VII	Uqcrq	0.77491	-1.0989143	-0.1371536
1427981_a_at	cysteine sulfinic acid decarboxylase	Csad	0.77492	-1.08500021	-0.1221089
1440200_at	family with sequence similarity 184, member B	Fam184b	0.77498	1.15958832	0.19192284
1455192_at	transmembrane protein 198	Tmem198	0.77512	-1.10356006	-0.1444189
1442513_at	cDNA sequence BC016423	BC016423	0.77521	1.12888838	0.16330302
1457097_at	src family associated phosphoprotein 2	Skap2	0.77522	1.10644458	0.14238532
1434758_at	cysteine-rich secretory protein LCCL domain containing 2	Crispld2	0.77525	-1.10973043	-0.1520783
1423692_at	NADH dehydrogenase (ubiquinone) 1 alpha subcomplex, 8	Ndufa8	0.77527	1.08316022	0.11156283
1460384_a_at	AT rich interactive domain 4B (RBP1-like)	Arid4b	0.77527	1.16544168	0.19710493
1444609_at	---	---	0.77528	-1.10881588	-0.15176
1460735_at	supervillin	Svil	0.77549	1.09521398	0.12965464
1427762_x_at	histone cluster 1, H2bp	Hist1h2bp	0.77565	1.13955446	0.17596874
1428657_at	ras responsive element binding protein 1	Rreb1	0.7757	-1.08301879	-0.1186336
1449635_at	PRP19/PSO4 pre-mRNA processing factor 19 homolog (S. cerevisi	Prpf19	0.77573	1.07288829	0.10034832
1452637_a_at	bolA-like 1 (E. coli)	Bola1	0.77577	1.10792994	0.1359028
1445146_at	RIKEN cDNA B230219N05 gene	B230219N05Rik	0.77579	-1.11464954	-0.1578748
1456964_at	RNA binding motif protein 12	Rbm12	0.77592	1.12111251	0.15308918
1445758_at	---	---	0.77592	-1.11953923	-0.1712493
1418169_at	zinc finger, CCHC domain containing 14	Zcchc14	0.77594	1.1019294	0.13773698
1447961_s_at	mitochondrial ribosomal protein L38	Mrpl38	0.77603	1.07539146	0.10334688
1432259_s_at	RIKEN cDNA 2810487C13 gene /// RIKEN cDNA 4931415C17 gen	2810487C13Rik /// 49314	0.77606	-1.11832186	-0.1779994
1445130_at	expressed sequence AA407111	AA407111	0.77612	1.1056579	0.14313612
1416115_at	origin recognition complex, subunit 3	Orc3	0.77614	1.0820331	0.11235022
1444669_at	leucine rich repeat and fibronectin type III domain containing 1	Lrfn1	0.77621	1.09773892	0.13094434
1452236_at	ATP-binding cassette, sub-family F (GCN20), member 1	Abcf1	0.77621	-1.10723018	-0.1666621
1435690_at	RIKEN cDNA 2310008H09 gene	2310008H09Rik	0.77622	1.07800426	0.10685604
1445741_at	---	---	0.77628	1.12096134	0.15714505
1458439_a_at	DAZ interacting protein 3, zinc finger	Dzip3	0.77632	-1.11145093	-0.1680195
1424540_at	homeodomain interacting protein kinase 1	Hipk1	0.7764	1.09086852	0.12030897
1428318_at	submandibular gland protein C	Smgc	0.77644	1.0880272	0.1209481
1459410_at	---	---	0.77658	1.09523131	0.12925648
1453149_at	solute carrier family 25, member 32	Slc25a32	0.77671	1.09417355	0.12860259
1441046_at	---	---	0.77683	1.15122167	0.18523953
1447899_x_at	epithelial cell adhesion molecule	Epcam	0.77686	-1.09239697	-0.130979
1455733_at	TAO kinase 3	Taok3	0.7769	1.09319169	0.12749617
1419071_at	cystin 1	Cys1	0.77716	1.11114653	0.14880556
1445691_at	chimerin (chimaerin) 1	Chn1	0.77717	-1.109659	-0.15483
1416198_at	TH1-like homolog (Drosophila)	Th1l	0.77717	1.08673393	0.11650971
1428899_at	transmembrane protein 182	Tmem182	0.77722	1.12232909	0.16080788
1448829_at	structural maintenance of chromosomes 6	Smc6	0.77723	-1.09621519	-0.1366712
1429035_at	dipeptidase 3	Dpep3	0.77723	-1.12305869	-0.1868019
1424310_at	molybdenum cofactor synthesis 2	Mocs2	0.77723	-1.11138552	-0.1568427
1439818_at	protein arginine methyltransferase 10 (putative)	Prmt10	0.77726	-1.09954379	-0.137082
1428106_at	RIKEN cDNA 1300001I01 gene	1300001I01Rik	0.77728	1.07620668	0.10520629
1454830_at	fibrillin 2	Fbn2	0.77728	-1.10622188	-0.1467378
1439654_at	predicted gene 7111	Gm7111	0.77729	1.08688083	0.11931815
1440584_at	RIKEN cDNA 9130221L21 gene	9130221L21Rik	0.7773	-1.10132333	-0.1434535
1428644_at	mannoside acetylglucosaminyltransferase 5	Mgat5	0.77731	1.11222299	0.15238143
1450728_at	four jointed box 1 (Drosophila)	Fjx1	0.77731	-1.09664659	-0.1343665
1451360_at	ERGIC and golgi 2	Ergic2	0.77731	-1.1034508	-0.1481316
1460659_at	---	---	0.77731	-1.12330569	-0.1832812
1423613_at	sperm specific antigen 2	Ssfa2	0.77732	1.09396444	0.12613395
1441615_at	core-binding factor, runt domain, alpha subunit 2, translocated to,	Cbfa2t2	0.77732	1.117901	0.1530272
1425209_at	zinc finger protein 84	Zfp84	0.77733	1.09934531	0.13531849
1436973_at	chaperonin containing Tcp1, subunit 8 (theta)	Cct8	0.77733	1.09844769	0.13240633
1432609_at	---	---	0.77733	-1.10386607	-0.1430611
1428515_at	zinc finger, SWIM-type containing 7	Zswim7	0.77733	1.08764814	0.11844135
1416575_at	cell division cycle 45 homolog (S. cerevisiae)	Cdc45	0.77733	1.08184363	0.11309034
1459906_at	---	---	0.77734	-1.09493607	-0.1367228
1433729_x_at	peptidase (mitochondrial processing) beta	Pmpcb	0.77735	-1.09115691	-0.1400708
1430748_at	RIKEN cDNA 1810063B07 gene	1810063B07Rik	0.77735	-1.10581367	-0.1475931
1421216_a_at	iduronate 2-sulfatase	Ids	0.77736	-1.10708239	-0.1479755
1440831_at	BTB and CNC homology 1	Bach1	0.77736	-1.08066996	-0.1140186
1433715_at	copine VII	Cpne7	0.77737	1.1107131	0.1486972
1442535_at	---	---	0.77741	-1.11711463	-0.1698892
1422420_at	Myoglobin	Mb	0.77742	1.10712169	0.14500956
1451147_x_at	cold shock domain containing C2, RNA binding	Csdc2	0.77743	-1.10529376	-0.1469941
1436069_at	inhibitor of growth family, member 5	Ing5	0.77744	1.07829668	0.10587969
1418118_at	solute carrier family 22 (organic cation transporter), member 1	Slc22a1	0.77746	-1.10685349	-0.1469445
1437841_x_at	cold shock domain containing C2, RNA binding	Csdc2	0.77746	-1.11326239	-0.1667153
1447040_at	---	---	0.77746	1.10413856	0.13825322
1456686_at	---	---	0.77746	-1.12905563	-0.1994029
1418261_at	spleen tyrosine kinase	Sykb	0.77747	1.08643599	0.1189232

1418877_at	Forkhead box D1	Foxd1	0.77749	-1.08324338	-0.1159007
1433904_at	gametogenetin binding protein 1	Ggnbp1	0.77749	1.10737508	0.14279277
1441576_at	---	---	0.7775	1.11342108	0.15241248
1418001_at	integral membrane protein 2B	Itm2b	0.77755	-1.1173776	-0.1724253
1423106_at	ubiquitin-conjugating enzyme E2B, RAD6 homology (S. cerevisiae	Ube2b	0.77756	1.09221562	0.12680331
1421848_at	solute carrier family 22 (organic cation transporter), member 5	Slc22a5	0.77761	-1.11677807	-0.1705756
1458136_at	Musashi homolog 2 (Drosophila)	Msi2	0.77763	1.09137013	0.1257675
1432680_at	RIKEN cDNA 9130009M17 gene	9130009M17Rik	0.77766	1.11241412	0.15355415
1452196_a_at	NCK-associated protein 1	Nckap1	0.7777	1.07090704	0.09740829
1456768_a_at	multimerin 2	Mmrn2	0.77776	-1.08722129	-0.1282906
1423877_at	chromatin assembly factor 1, subunit B (p60)	Chaf1b	0.77777	1.0899178	0.12244009
1438620_x_at	secreted frizzled-related protein 1	Sfrp1	0.77779	-1.08898206	-0.1243745
1426691_at	tight junction associated protein 1	Tjap1	0.77779	1.07657682	0.10632349
1455581_x_at	hypothetical protein 9530028C05	9530028C05	0.77783	1.11229854	0.14848911
1454608_x_at	transthyretin	Ttr	0.77783	1.08868637	0.12012111
1434555_at	acidic (leucine-rich) nuclear phosphoprotein 32 family, member A	Anp32a	0.77783	1.08519941	0.11443434
1420786_a_at	RNA binding motif protein, Y chromosome, family 1, member A1	Rbmy1a1	0.77784	1.12573083	0.16152412
1424566_s_at	polymerase (RNA) III (DNA directed) polypeptide D	Polr3d	0.77784	1.08806678	0.11932919
1436314_at	SCY1-like 2 (S. cerevisiae)	Scyl2	0.77785	1.16739965	0.19513555
1436002_at	signal peptide, CUB domain, EGF-like 3	Scube3	0.77785	1.1193912	0.15728929
1426422_at	coiled-coil and C2 domain containing 1A	Cc2d1a	0.77788	1.12163899	0.15347207
1430104_at	R-spondin 2 homolog (Xenopus laevis)	Rspo2	0.77789	1.11789046	0.15510157
1453395_at	RIKEN cDNA 5330403D14 gene	5330403D14Rik	0.77789	-1.11394698	-0.1667406
1430623_s_at	oligonucleotide/oligosaccharide-binding fold containing 2A	Obfc2a	0.7779	-1.09809009	-0.1491194
1419590_at	cytochrome P450, family 2, subfamily b, polypeptide 9	Cyp2b9	0.7779	1.13083213	0.16897326
1445227_at	RIKEN cDNA 2010012P19 gene	2010012P19Rik	0.77791	1.13719197	0.17313974
1455207_at	RIKEN cDNA 2410017P09 gene	2410017P09Rik	0.77791	1.11605918	0.14670139
1442340_x_at	cysteine rich protein 61	Cyr61	0.77792	1.10988105	0.1451374
1430245_at	---	---	0.77792	-1.11471725	-0.1612183
1436333_a_at	synaptojanin 1	Synj1	0.77793	-1.11827628	-0.1914982
1417067_s_at	aarF domain containing kinase 3	Adck3	0.77793	1.09190147	0.12400103
1437050_s_at	angel homolog 2 (Drosophila)	Angel2	0.77793	1.08380189	0.11542871
1426151_a_at	syntaxin 3	Stx3	0.77795	-1.12638399	-0.2107382
1422990_at	met proto-oncogene	Met	0.77795	-1.10990429	-0.1542634
1444116_at	N(alpha)-acetyltransferase 35, NatC auxiliary subunit	Naa35	0.77796	-1.10699245	-0.1467815
1422087_at	v-myc myelocytomatosis viral oncogene homolog 1, lung carcino	Mycl1	0.77796	1.11996125	0.1567393
1449364_at	aurora kinase C	Aurkc	0.77797	-1.0929378	-0.1304994
1419217_at	secretion regulating guanine nucleotide exchange factor	Sergef	0.77797	-1.09701976	-0.1375904
1429268_at	RIKEN cDNA 2610318N02 gene	2610318N02Rik	0.77797	1.08965742	0.11852864
1448105_at	protamine 2	Prm2	0.77797	1.11400134	0.15160942
1449934_at	purine rich element binding protein A	Pura	0.778	-1.08386142	-0.1245929
1449689_at	expressed sequence C81600	C81600	0.778	1.11459254	0.15251828
1428788_at	phosphoglycolate phosphatase	Pgp	0.77801	1.08282168	0.11265271
1449917_at	paired-like homeodomain transcription factor 3	Pitx3	0.77801	1.1079688	0.14436623
1418356_at	mercaptopyruvate sulfurtransferase	Mpst	0.77802	1.09352667	0.12737788
1427515_at	pigeon homolog (Drosophila)	Pion	0.77803	-1.09603331	-0.1325942
1448602_at	muscle glycogen phosphorylase	Pygm	0.77803	1.11480567	0.15235578
1416384_a_at	coatomer protein complex, subunit epsilon	Cope	0.77803	1.08907548	0.11738352
1457718_at	Snf2-related CREBBP activator protein	Srcap	0.77804	-1.10242772	-0.1471442
1418503_at	heat shock protein 9	Hspa9	0.77806	-1.06170558	-0.0871104
1423323_at	tumor-associated calcium signal transducer 2	Tacstd2	0.77806	-1.08371949	-0.116837
1425557_x_at	TSC22 domain family, member 3	Tsc22d3	0.77806	1.12263743	0.1585834
1441783_at	RIKEN cDNA 2410022M11 gene	2410022M11Rik	0.77807	-1.10253483	-0.1445729
1450587_at	histocompatibility 2, M region locus 10.1	H2-M10.1	0.77807	-1.11037201	-0.164166
1420148_at	solute carrier family 6 (neurotransmitter transporter, taurine), me	Slc6a6	0.77809	-1.09581791	-0.1398214
1425320_at	zinc finger protein 605	Zfp605	0.77812	1.10626829	0.14441444
1457565_at	ATPase family, AAA domain containing 5	Atad5	0.77812	-1.10205145	-0.1421273
1431046_at	protein tyrosine phosphatase, receptor type, f polypeptide (PTPRF	Ppfia3	0.77813	1.10944404	0.14431602
1442936_at	---	---	0.77814	1.09681735	0.13094003
1419347_x_at	seminal vesicle secretory protein 5	Svs5	0.77815	-1.10499906	-0.1581156
1456452_at	protease, serine, 45	Prss45	0.77815	1.15588697	0.18908278
1456053_at	---	---	0.77816	-1.1160936	-0.1812538
1445390_at	PRKC, apoptosis, WT1, regulator	Pawr	0.77816	1.11158388	0.15036936
1434829_at	Casitas B-lineage lymphoma	Cbl	0.77816	-1.09705282	-0.1463124
1446424_at	dynein, axonemal, heavy chain 12	Dnahc12	0.77817	1.10601543	0.1443188
1418636_at	ets variant gene 3	Etv3	0.77817	1.10372884	0.14133404
1441536_at	3-hydroxy-3-methylglutaryl-Coenzyme A synthase 1	Hmgcs1	0.77819	1.1529951	0.18614016
1452880_at	zinc finger, HIT type 3	Znhit3	0.7782	1.07768505	0.10655886
1444049_at	adenosine deaminase, tRNA-specific 1	Adat1	0.7782	1.11384346	0.14649213
1416097_at	leucine rich repeat containing 4	Lrrc4	0.7782	1.12791621	0.16742387
1420264_at	---	---	0.7782	-1.13357872	-0.2156159
1436951_x_at	thioredoxin domain containing 9	Txndc9	0.77821	-1.06981847	-0.0987795
1437569_at	transmembrane protein 132E	Tmem132e	0.77821	-1.10630487	-0.1460942
1423854_a_at	RAS-like, family 11, member B	Rasl11b	0.77822	1.09269878	0.1271525
1434308_at	solute carrier family 43, member 2	Slc43a2	0.77823	1.09054601	0.12504303
1435951_at	glutamate receptor interacting protein 1	Grip1	0.77823	1.10635425	0.14522457
1433510_x_at	ribosomal protein L36	Rpl36	0.77823	1.06897036	0.09245238
1426903_at	fibronectin type III domain containing 3A	Fndc3a	0.77824	-1.07357924	-0.1045634
1418390_at	PHD finger protein 21A	Phf21a	0.77824	1.11281258	0.14073186
1428142_at	ets variant gene 5	Etv5	0.77824	-1.06721484	-0.0961205
1423318_at	RAD18 homolog (S. cerevisiae)	Rad18	0.77825	1.10793886	0.14268262
1427314_at	transmembrane emp24 protein transport domain containing 7	Tmed7	0.77826	-1.08662847	-0.1305709
1417984_at	ubiquitin-conjugating enzyme E2 variant 2	Ube2v2	0.77826	-1.09234129	-0.1326604
1427869_at	Immunoglobulin heavy chain 6 (heavy chain of IgM)	Igh-6	0.77828	1.11378032	0.15378405
1441597_at	---	---	0.77828	-1.09944083	-0.1383908
1440452_at	dystrophin related protein 2	Drp2	0.77828	-1.09129842	-0.1261753
1440927_x_at	apolipoprotein L 11b	Apol11b	0.7783	1.09566583	0.12723655
1454316_at	RIKEN cDNA 5830426C09 gene	5830426C09Rik	0.77831	-1.11071321	-0.1599124
1428110_x_at	vacuolar protein sorting 11 (yeast)	Vps11	0.77831	1.0951949	0.12508005
1440552_at	---	---	0.77832	-1.12730607	-0.205981
1422374_s_at	olfactory receptor 66	Olfr66	0.77832	1.11142277	0.14927898
1425135_a_at	dynamin 2	Dnm2	0.77832	-1.0872716	-0.1210636
1430438_at	Biorientation of chromosomes in cell division 1-like	Bod1l	0.77832	-1.10529424	-0.1492893
1453444_at	RIKEN cDNA 5730437C12 gene	5730437C12Rik	0.77834	-1.09117005	-0.1281505

1420481_at	cyclin M3	Cnnm3	0.77834	1.09037692	0.12276994
1423379_at	nuclear factor of activated T-cells, cytoplasmic, calcineurin-depen	Nfatc4	0.77835	1.15720886	0.18894004
1455128_x_at	trinucleotide repeat containing 6a	Tnrc6a	0.77835	-1.08448666	-0.1185949
1430174_at	RIKEN cDNA A930010G16 gene	A930010G16Rik	0.77835	1.0928372	0.12793418
1418684_at	zinc finger protein 33B	Zfp33b	0.77835	1.08318215	0.11375576
1425406_at	C-type lectin domain family 4, member a2	Clec4a2	0.77836	-1.11942202	-0.1786195
1453159_at	EF-hand domain (C-terminal) containing 1	Efhc1	0.77836	1.11613637	0.15498083
1441320_a_at	expressed sequence AI413194 /// tripartite motif-containing 56	AI413194 /// Trim56	0.77837	1.13560046	0.16735315
1439840_at	polymerase (DNA directed), beta	Polb	0.77837	1.17508609	0.20282558
1454228_a_at	coiled-coil domain containing 77	Ccdc77	0.77838	1.1301431	0.16299345
1417024_at	histidyl-tRNA synthetase	Hars	0.77838	-1.09213009	-0.1375232
1458529_at	predicted gene 4793	Gm4793	0.77838	-1.10481789	-0.1455063
1440189_at	protein phosphatase 2, regulatory subunit B (B56), alpha isoform	Ppp2r5a	0.77838	1.11963524	0.15368552
1460287_at	tissue inhibitor of metalloproteinase 2	Timp2	0.77839	1.09250428	0.12481724
1416018_at	down-regulator of transcription 1	Dr1	0.77839	-1.07637509	-0.1065356
1418923_at	solute carrier family 17 (sodium phosphate), member 3	Slc17a3	0.77839	-1.10959208	-0.1501578
1433380_at	RIKEN cDNA 4921509A06 gene	4921509A06Rik	0.7784	-1.104065	-0.1442092
1419572_a_at	ATP-binding cassette, sub-family D (ALD), member 4	Abcd4	0.77841	1.087777	0.12123661
1428650_at	tensin 1	Tns1	0.77841	1.09984546	0.13375633
1431481_at	RIKEN cDNA 4930443O20 gene	4930443O20Rik	0.77841	-1.12484286	-0.1868336
1421669_at	sulfotransferase family 3A, member 1	Sult3a1	0.77841	-1.08741473	-0.1231022
1441346_at	---	---	0.77842	-1.11233891	-0.164905
1438157_s_at	nuclear factor of kappa light polypeptide gene enhancer in B-cells	Nfkbia	0.77842	-1.0999351	-0.1584532
1453371_at	regulation of nuclear pre-mRNA domain containing 2	Rprd2	0.77842	1.13205707	0.169965
1458196_at	---	---	0.77843	-1.07791209	-0.1114285
1445640_at	---	---	0.77843	-1.12697966	-0.1983595
1429962_at	CCAAT/enhancer binding protein zeta	Cebpz	0.77844	1.10731274	0.14278015
1419310_s_at	regulatory factor X-associated ankyrin-containing protein	Rfxank	0.77844	1.11922952	0.15299157
1433098_at	non-SMC element 1 homolog (S. cerevisiae)	Nsmce1	0.77844	1.14221526	0.17488366
1427617_at	fucosyltransferase 10	Fut10	0.77845	-1.10756421	-0.1499965
1455986_at	zinc finger, DHHC domain containing 17	Zdhhc17	0.77845	-1.11487769	-0.162823
1446872_at	expressed sequence AU021877	AU021877	0.77846	1.12842832	0.15857859
1456724_x_at	WD repeat containing, antisense to TP53	Wrap53	0.77847	-1.1054569	-0.1466666
1457317_at	---	---	0.77847	-1.09072924	-0.1262337
1423947_at	RIKEN cDNA 1110008P14 gene	1110008P14Rik	0.77847	1.0927657	0.12253083
1431851_at	preferentially expressed antigen in melanoma	Prame	0.77847	1.12864804	0.16503834
1423727_at	cornichon homolog (Drosophila)	Cnih	0.77847	1.07011339	0.09750766
1456348_x_at	progestin and adipoQ receptor family member V	Paqr5	0.77848	-1.10878188	-0.1535266
1431303_at	serine/threonine kinase 38	Stk38	0.77848	-1.10670685	-0.1525139
1432676_at	golgi-specific brefeldin A-resistance factor 1	Gbf1	0.77849	-1.11706613	-0.1708769
1439352_at	tripartite motif-containing 7	Trim7	0.7785	1.13663509	0.17184105
1420098_s_at	DNA segment, Chr 13, ERATO Doi 787, expressed	D13Ertd787e	0.7785	-1.11352572	-0.179752
1444610_at	---	---	0.77852	1.10105807	0.13548914
1456892_at	RIKEN cDNA D030036P13 gene	D030036P13Rik	0.77852	-1.12482047	-0.1887417
1427971_at	cell division cycle 73, Paf1/RNA polymerase II complex componen	Cdc73	0.77853	-1.07103653	-0.0995905
1418597_at	topoisomerase (DNA) III alpha	Top3a	0.77854	1.10678719	0.13934988
1419785_at	expressed sequence AA516738	AA516738	0.77855	1.16602793	0.19479355
1450827_at	kinesin family member 23	Kif23	0.77855	1.11269398	0.1490117
1452100_at	Dullard homolog (Xenopus laevis)	Dullard	0.77855	1.07903999	0.10705008
1416421_a_at	Sjogren syndrome antigen B	Ssb	0.77855	-1.07355798	-0.1026565
1444799_at	expressed sequence AU022054	AU022054	0.77855	1.08216486	0.11252304
1453433_at	WD repeat domain 89	Wdr89	0.77855	1.08178205	0.11319975
1433334_at	RIKEN cDNA 1700024P12 gene	1700024P12Rik	0.77856	-1.08825807	-0.1220595
1456446_at	RIKEN cDNA 4930523C07 gene	4930523C07Rik	0.77856	-1.10206262	-0.1408702
1454192_at	RIKEN cDNA 4930579C15 gene	4930579C15Rik	0.77857	-1.08329775	-0.1165443
1416534_at	D4, zinc and double PHD fingers family 2	Dpf2	0.77858	1.07495052	0.1028869
1451192_a_at	tetratricopeptide repeat domain 4	Ttc4	0.77858	1.08661128	0.11923971
1417391_a_at	interleukin 16	Il16	0.77859	1.11201121	0.15267581
1457118_at	SHC (Src homology 2 domain containing) family, member 4	Shc4	0.77859	1.14778777	0.17919298
1455306_at	family with sequence similarity 193, member A	Fam193a	0.7786	1.09029033	0.12137152
1445106_at	hypothetical protein LOC100503654	LOC100503654	0.77861	1.0923371	0.12654333
1422170_at	solute carrier family 5 (inositol transporters), member 3	Slc5a3	0.77861	-1.10256287	-0.1422507
1435333_at	NADH dehydrogenase (ubiquinone) 1 alpha subcomplex, assemb	Ndufaf4	0.77861	-1.07853142	-0.1116984
1435043_at	phospholipase C, beta 1	Plcb1	0.77863	1.11106561	0.14909498
1422469_at	TANK-binding kinase 1	Tbk1	0.77864	-1.08228684	-0.1153583
1428509_at	myosin IE	Myo1e	0.77864	1.10844034	0.14049997
1460461_at	lysophosphatidylcholine acyltransferase 2B	Lpcat2b	0.77865	-1.10279692	-0.1438992
1418895_at	src family associated phosphoprotein 2	Skap2	0.77866	1.08713147	0.11536867
1451523_a_at	MIF4G domain containing	Mif4gd	0.77867	1.08237791	0.11357615
1426197_at	ig heavy chain V region 108A-like	LOC636979	0.77867	1.1053829	0.14026116
1416459_at	ADP-ribosylation factor 2	Arf2	0.77868	1.09359929	0.12869572
1423186_at	T-cell lymphoma invasion and metastasis 2	Tiam2	0.77869	1.11869665	0.15997878
1433851_at	protein phosphatase 4, regulatory subunit 2	Ppp4r2	0.7787	1.09675963	0.13226741
1444914_at	---	---	0.77871	-1.10711015	-0.149254
1443439_at	---	---	0.77871	-1.10423119	-0.1467368
1457076_at	family with sequence similarity 131, member C	Fam131c	0.77872	1.09498643	0.128367
1445564_at	---	---	0.77873	1.1003407	0.13410423
1423065_at	DNA methyltransferase 3A	Dnmt3a	0.77874	-1.09208634	-0.1299479
1457223_at	---	---	0.77874	1.11364224	0.15074954
1453715_at	synaptic vesicle glycoprotein 2c	Sv2c	0.77874	-1.10885954	-0.1516621
1451424_at	gamma-aminobutyric acid (GABA) A receptor, pi	Gabrp	0.77876	-1.10973505	-0.1520652
1416848_at	ubiquitin-like 5	Ubl5	0.77877	1.10336689	0.13375745
1434372_at	expressed sequence AW112010	AW112010	0.77879	-1.09059053	-0.1252482
1419127_at	neuropeptide Y	Npy	0.77879	1.10247674	0.13933486
1431022_at	synuclein, alpha	Snca	0.7788	-1.09274285	-0.1290596
1448800_at	reticulon 4 interacting protein 1	Rtn4ip1	0.7788	1.1381113	0.16790627
1454742_at	RasGEF domain family, member 1B	Rasgef1b	0.77881	1.11172555	0.14919101
1419200_at	FXYD domain-containing ion transport regulator 7	Fxyd7	0.77881	-1.11792918	-0.1691913
1455317_at	enhancer of polycomb homolog 2 (Drosophila)	Epc2	0.77883	-1.07497415	-0.1043049
1460079_at	tetratricopeptide repeat domain 5	Ttc5	0.77885	1.10426001	0.14269238
1430056_at	ubinuclein 2	Ubn2	0.77886	-1.1015592	-0.1451892
1422271_at	pancreatic polypeptide receptor 1	Ppyr1	0.77888	-1.10678395	-0.1480147
1454771_at	predicted gene 2695	Gm2695	0.77892	1.09561968	0.12919927
1418031_at	myosin IXb	Myo9b	0.77895	1.10077892	0.13256628

1435990_at	a disintegrin-like and metallopeptidase (reprolysin type) with thro	Adamts2	0.77897	1.11340359	0.15106376
1440581_at	phosphoinositide-interacting regulator of transient receptor poten	Pirt	0.77899	1.11941632	0.1583692
1429230_at	kallikrein related-peptidase 5	Klk5	0.77914	-1.10882085	-0.150977
1453223_s_at	developmental pluripotency associated 2	Dppa2	0.77927	-1.06419655	-0.0923637
1445516_at	---	---	0.77933	1.12270426	0.15984635
1453129_a_at	regulator of G-protein signaling 12	Rgs12	0.77934	1.08816501	0.12127223
1436804_s_at	SCY1-like 1 (S. cerevisiae)	Scyl1	0.77934	-1.08571776	-0.1250087
1437105_at	lysine (K)-specific demethylase 5A	Kdm5a	0.77937	-1.08339961	-0.1174943
1417820_at	torsin family 1, member B	Tor1b	0.77939	1.13246563	0.16099753
1420172_at	---	---	0.77941	-1.09107212	-0.1391332
1422924_at	tumor necrosis factor (ligand) superfamily, member 9	Tnfsf9	0.77944	1.10622211	0.13822294
1449579_at	Sh3 domain YSC-like 1	Sh3yl1	0.77946	1.10142437	0.13756369
1437867_at	---	---	0.77947	-1.11225375	-0.1728639
1457902_at	---	---	0.77952	1.12343774	0.16177081
1443756_at	Serine (or cysteine) peptidase inhibitor, clade I, member 1	Serpini1	0.77957	-1.08064319	-0.1136217
1442842_at	Hspb associated protein 1	Hspbap1	0.77959	-1.1132927	-0.1713494
1426417_at	Yip1 domain family, member 4	Yipf4	0.77965	-1.11152733	-0.155392
1420596_at	calcium channel, voltage-dependent, gamma subunit 2	Cacng2	0.7797	1.13931706	0.17052955
1418411_at	F-box and leucine-rich repeat protein 8	Fbxl8	0.7797	1.13562707	0.16605408
1431070_a_at	RIKEN cDNA 9130404D08 gene	9130404D08Rik	0.7797	1.09990159	0.1328394
1444312_at	solute carrier family 22 (organic anion/cation transporter), memb	Slc22a15	0.77971	-1.1051334	-0.1459983
1433927_at	ubiquitin specific peptidase like 1	Uspl1	0.77971	1.07852398	0.10683999
1445414_at	---	---	0.77972	-1.10905563	-0.1543596
1457984_at	corticotropin releasing hormone	Crh	0.7798	1.12886974	0.15537097
1434243_s_at	translocase of outer mitochondrial membrane 70 homolog A (yea	Tomm70a	0.77986	-1.07638091	-0.1063588
1441348_at	zinc finger protein 955A /// zinc finger protein 955B	Zfp955a /// Zfp955b	0.7799	-1.10383709	-0.1436957
1424343_a_at	eukaryotic translation initiation factor 1A	Eif1a	0.77993	-1.06898281	-0.0962397
1441575_at	predicted gene 9930	Gm9930	0.77994	-1.07783866	-0.1106227
1439325_at	---	---	0.77996	1.11242837	0.15087329
1460272_at	ubiquitin-like 4B	Ubl4b	0.77997	-1.10456302	-0.1444872
1447177_at	---	---	0.77998	-1.1038879	-0.1473682
AFFX-LysX-5_at	---	---	0.77999	1.11823543	0.15643918
1431346_at	RIKEN cDNA 6330405D24 gene	6330405D24Rik	0.78	-1.10684034	-0.1490318
1429176_at	leucine rich repeat and sterile alpha motif containing 1	Lrsam1	0.78001	1.11088914	0.14495048
1435238_x_at	RIKEN cDNA 2310009A05 gene	2310009A05Rik	0.78001	1.09597158	0.12644616
1448515_at	translin	Tsn	0.78001	-1.08091694	-0.1182829
1427085_at	RIKEN cDNA 2810432D09 gene	2810432D09Rik	0.78002	1.08139643	0.10908951
1452988_at	RIKEN cDNA 2610306M01 gene	2610306M01Rik	0.78004	1.0960612	0.13147398
1427896_at	suppressor of defective silencing 3 homolog (S. cerevisiae)	Suds3	0.78004	1.08109749	0.11094528
1425733_a_at	epidermal growth factor receptor pathway substrate 8	Eps8	0.78006	1.10698961	0.14193119
1420415_at	cDNA sequence AY026312	AY026312	0.78006	1.12518677	0.16230144
1426734_at	family with sequence similarity 43, member A	Fam43a	0.78007	1.0907234	0.11979782
1420369_a_at	casein beta	Csn2	0.78007	1.11092019	0.14610788
1441172_at	---	---	0.78007	-1.0966302	-0.1340548
1432446_at	septin 14	Sep-14	0.78011	-1.10182998	-0.1409194
1444614_x_at	enolase 4	Eno4	0.78018	1.11092119	0.14827361
1428273_at	abhydrolase domain containing 13	Abhd13	0.78021	-1.08683268	-0.1203864
1428393_at	neuritin 1	Nrn1	0.78028	-1.11520202	-0.162675
1440944_at	ankyrin repeat and sterile alpha motif domain containing 1B	Anks1b	0.78029	1.09802831	0.12755521
1441376_at	gamma-aminobutyric acid (GABA) A receptor-associated protein-	Gabarapl2	0.78029	-1.09496503	-0.1364561
1456041_at	sorting nexin 16	Snx16	0.78033	-1.0951366	-0.1372512
1430764_at	RIKEN cDNA 1700023F06 gene	1700023F06Rik	0.78033	1.12550086	0.16107531
1455673_at	alkB, alkylation repair homolog 2 (E. coli)	Alkbh2	0.78037	1.09933157	0.12992462
1422648_at	solute carrier family 7 (cationic amino acid transporter, y+ system	Slc7a2	0.78039	-1.10720061	-0.1659468
1454727_at	actin filament associated protein 1-like 1	Afap1l1	0.78042	1.10102138	0.13650927
1419311_at	tripartite motif-containing 10	Trim10	0.78047	1.16474543	0.19599636
1459614_at	laminin, alpha 5	Lama5	0.7805	1.1232543	0.15776323
1425193_at	RIKEN cDNA 2010106G01 gene	2010106G01Rik	0.7805	-1.09922875	-0.1442574
1459525_at	---	---	0.78051	-1.10615336	-0.1461935
1419476_at	ADAM-like, decysin 1	Adamdec1	0.78053	-1.10667177	-0.1475899
1436986_at	syntrophin, basic 2	Sntb2	0.78058	-1.11356	-0.1669138
1421642_a_at	cysteinyl leukotriene receptor 2	Cysltr2	0.78059	-1.10752951	-0.151937
1448111_at	cytidine 5'-triphosphate synthase 2	Ctps2	0.78059	1.08574687	0.11685819
1444451_at	pappalysin 2	Pappa2	0.78063	1.10236988	0.13846473
1429271_at	RIKEN cDNA 9130011E15 gene	9130011E15Rik	0.78064	1.11288928	0.14545043
1426833_at	eukaryotic translation initiation factor 4 gamma, 3	Eif4g3	0.78064	-1.08676878	-0.1258867
1444589_at	predicted gene 4944	Gm4944	0.78065	-1.11115838	-0.1566351
1445322_x_at	---	---	0.78066	-1.09118412	-0.128483
1419944_at	hypothetical LOC100504316 /// hypothetical LOC100505168	LOC100504316 /// LOC10	0.78067	-1.11569318	-0.1748805
1448733_at	Bmi1 polycomb ring finger oncogene	Bmi1	0.78067	-1.12075189	-0.1803514
1447461_at	---	---	0.78068	1.1316221	0.16386132
1427244_at	tetratricopeptide repeat domain 15	Ttc15	0.78069	-1.08027166	-0.1120602
1440628_at	---	---	0.78069	-1.12155972	-0.1827943
1455053_a_at	DCN1, defective in cullin neddylation 1, domain containing 1 (S. ce	Dcun1d1	0.78071	1.11461656	0.15397684
1460057_at	growth differentiation factor 3	Gdf3	0.78071	1.07744641	0.1074851
1430531_at	biphenyl hydrolase-like (serine hydrolase, breast epithelial mucin-	Bphl	0.78074	1.10852428	0.14524384
1452040_a_at	cell division cycle associated 3	Cdca3	0.78078	1.06979069	0.09720194
1425136_x_at	dynamin 2	Dnm2	0.78078	-1.07782882	-0.1084914
1451845_a_at	peptidyl-tRNA hydrolase 2	Ptrh2	0.78085	1.07582653	0.10437061
1430869_a_at	hyaluronic acid binding protein 4	Habp4	0.78086	1.10606175	0.14435773
1457372_at	expressed sequence AW121686	AW121686	0.78087	-1.11693297	-0.1737844
1458075_at	---	---	0.78088	1.15698669	0.18716762
1421844_at	interleukin 1 receptor accessory protein	Il1rap	0.78088	-1.11331568	-0.1655206
1457880_at	---	---	0.78091	1.12184553	0.15897404
1455090_at	angiopoietin-like 2	Angptl2	0.78091	-1.10066048	-0.1402951
1419947_at	DNA segment, Chr 4, ERATO Doi 117, expressed	D4Ertd117e	0.78091	-1.08723001	-0.1226376
1429617_at	cylindromatosis (turban tumor syndrome)	Cyld	0.78093	1.08923788	0.12210217
1431760_a_at	serologically defined colon cancer antigen 3	Sdccag3	0.78094	1.0912138	0.12498286
1417903_at	deafness, autosomal dominant 5 (human)	Dfna5	0.78094	1.1639451	0.19430432
1428000_at	transmembrane protein 60	Tmem60	0.78095	1.09171801	0.12247353
1458462_at	Rho GTPase activating protein 30	Arhgap30	0.78099	1.13618964	0.17036888
1439688_at	fibulin 1	Fbln1	0.78101	-1.10599885	-0.1487734
1441819_x_at	translocase of inner mitochondrial membrane 50 homolog (yeast	Timm50	0.78103	-1.11578502	-0.1724159
1455402_at	suppressor of cytokine signaling 7	Socs7	0.78104	-1.08441502	-0.1185072

1425717_at	LPS-responsive beige-like anchor	Lrba	0.78105	-1.1020869	-0.1416593
1423347_at	SEC23A (S. cerevisiae)	Sec23a	0.78106	-1.07592695	-0.1095722
1449975_a_at	Parkinson disease (autosomal recessive, juvenile) 2, parkin	Park2	0.78111	-1.09955986	-0.1369949
1441078_at	RIKEN cDNA 1700023F02 gene	1700023F02Rik	0.78113	-1.11307546	-0.159966
1457251_x_at	---	---	0.78141	-1.10392053	-0.1520196
1435909_at	RIKEN cDNA C030034I22 gene	C030034I22Rik	0.78148	1.12353465	0.16045971
1423619_at	RAS, dexamethasone-induced 1	Rasd1	0.7815	1.10150492	0.13855785
1428556_at	phosphatidylinositol glycan anchor biosynthesis, class Y	Pigy	0.78191	1.10450078	0.13654347
1428598_at	TBC1 domain family, member 7	Tbc1d7	0.78194	1.08671708	0.11711911
1427404_x_at	predicted gene 5506	Gm5506	0.78195	1.03446769	0.04882155
1428755_at	cAMP responsive element binding protein 1	Creb1	0.78213	-1.08099406	-0.1124567
1430497_at	retinoid X receptor alpha	Rxra	0.78226	-1.10933536	-0.1541665
1431651_at	coiled-coil domain containing 151	Ccdc151	0.7823	1.11161404	0.14931539
1434924_at	PHD finger protein 2	Phf2	0.78233	1.11169668	0.14511104
1444532_at	nicastrin	Ncstn	0.78234	1.1204204	0.1585049
1419810_x_at	Rho GTPase activating protein 9	Arhgap9	0.78234	-1.11667162	-0.1798238
1436284_s_at	zinc finger protein 319	Zfp319	0.78234	1.13797267	0.17195607
1421961_a_at	DnaJ (Hsp40) homolog, subfamily B, member 5	Dnajb5	0.78235	-1.08535871	-0.1187604
1435768_at	AT rich interactive domain 4B (RBP1-like)	Arid4b	0.78236	-1.08464996	-0.1210177
1449813_at	zinc finger protein 30	Zfp30	0.78238	1.11490953	0.1491586
1420629_a_at	DnaJ (Hsp40) homolog, subfamily A, member 3	Dnaja3	0.78238	1.08008353	0.10719681
1436123_at	bassoon	Bsn	0.7824	-1.11001366	-0.155693
1422777_at	complement component 1, q subcomponent-like 1	C1ql1	0.78241	-1.13406492	-0.2184305
1458455_at	actin-binding Rho activating protein	Abra	0.78241	1.09822259	0.13516655
1441580_at	---	---	0.78241	-1.10093857	-0.1394304
1446039_at	---	---	0.78242	-1.08770545	-0.124544
1416269_at	ATP synthase, H+ transporting, mitochondrial F0 complex, subunit	Atp5j2	0.78243	1.0803202	0.1055186
1445878_at	RIKEN cDNA C920006O11 gene	C920006O11Rik	0.78244	-1.10734903	-0.1495677
1426083_a_at	B-cell translocation gene 1, anti-proliferative	Btg1	0.78246	1.07553523	0.10438434
1442677_at	---	---	0.78246	-1.10028742	-0.1395773
1450675_at	stromal membrane-associated GTPase-activating protein 2	Smap2	0.78247	1.11775991	0.14807845
1424916_x_at	zinc finger protein 764	Zfp764	0.78247	1.09084609	0.12328996
1427683_at	early growth response 2	Egr2	0.78247	1.1046701	0.13947419
1416537_at	cysteine-rich with EGF-like domains 1	Creld1	0.78248	1.07917834	0.1094996
1442262_at	---	---	0.78248	1.12618991	0.16192453
1456235_at	expressed sequence BB165335	BB165335	0.78249	1.1242765	0.15817306
1456764_at	solute carrier family 35, member F3	Slc35f3	0.78249	1.1123095	0.14882508
1435401_at	tetratricopeptide repeat domain 26	Ttc26	0.7825	1.11372571	0.14686098
1453541_at	phosphofructokinase, liver, B-type	Pfkl	0.78251	-1.10091027	-0.1410357
1459175_at	---	---	0.78251	1.10492757	0.13598036
1451977_at	dual-specificity tyrosine-(Y)-phosphorylation regulated kinase 1a	Dyrk1a	0.78253	1.08587627	0.11714006
1447836_x_at	RIKEN cDNA 4921525O09 gene	4921525O09Rik	0.78253	-1.1056819	-0.1505094
1444839_at	---	---	0.78254	1.08241526	0.11234714
1418153_at	laminin, alpha 1	Lama1	0.78254	1.080812	0.1118436
1420827_a_at	cyclin G1	Ccng1	0.78255	-1.08066761	-0.1179773
1430820_a_at	bobby sox homolog (Drosophila)	Bbx	0.78256	-1.12795385	-0.2182269
1439214_a_at	apoptosis inhibitor 5	Api5	0.78256	-1.0789436	-0.115203
1424013_at	eukaryotic translation termination factor 1	Etf1	0.78258	1.05880194	0.08239637
1434381_at	ATM interactor	Atmin	0.78258	1.08364025	0.1151285
1435450_at	copine III	Cpne3	0.78259	-1.08580362	-0.1278072
1421006_at	collagen, type IV, alpha 6	Col4a6	0.7826	-1.10519537	-0.1473553
1416407_at	phosphoprotein enriched in astrocytes 15A	Pea15a	0.7826	1.10594853	0.13628606
1454654_at	disrupted in renal carcinoma 2 (human)	Dirc2	0.78261	1.09557332	0.1274675
1418145_at	tuftelin interacting protein 11	Tfip11	0.78261	1.07854819	0.10667088
1443552_at	RIKEN cDNA E230008N13 gene	E230008N13Rik	0.78262	-1.10365385	-0.1443708
1446451_at	---	---	0.78262	1.09815238	0.13139815
1432351_at	RIKEN cDNA 9030625G05 gene	9030625G05Rik	0.78263	-1.11266428	-0.1585472
1427916_at	suppression of tumorigenicity 7-like	St7l	0.78265	1.08842149	0.12208598
1437042_at	aryl hydrocarbon receptor nuclear translocator	Arnt	0.78266	-1.09119413	-0.1309207
1421958_at	L1 cell adhesion molecule	L1cam	0.78267	1.10956933	0.14675604
1428977_at	carbohydrate (N-acetylgalactosamine 4-0) sulfotransferase 8	Chst8	0.78267	-1.11342119	-0.1635244
1435896_at	sideroflexin 2	Sfxn2	0.78267	1.09169896	0.12611043
1442854_at	---	---	0.78268	1.12295366	0.15920739
1426502_s_at	glutamic pyruvic transaminase, soluble	Gpt	0.78269	1.08517009	0.11733631
1456556_at	RIKEN cDNA A230009B12 gene	A230009B12Rik	0.78271	1.13784392	0.17219674
1424054_at	BTB (POZ) domain containing 2	Btbd2	0.78271	1.07414163	0.1028098
1439297_at	PIF1 5'-to-3' DNA helicase homolog (S. cerevisiae)	Pif1	0.78273	-1.10313168	-0.151789
1442850_at	expressed sequence AU045717	AU045717	0.78274	-1.12348801	-0.1894123
1450512_at	netrin 4	Ntn4	0.78275	1.10394867	0.14179974
1443011_at	protein arginine N-methyltransferase 7	Prmt7	0.78275	1.09773781	0.13396527
1456943_a_at	dysbindin (dystrobrevin binding protein 1) domain containing 2	Dbndd2	0.78276	1.09924537	0.13498707
1426635_at	acyl-Coenzyme A binding domain containing 3	Acbd3	0.78276	1.10001144	0.13450469
1421178_at	mannosyl (alpha-1,3-)-glycoprotein beta-1,4-N-acetylglucosamin	Mgat4c	0.78279	1.13212524	0.16610545
1419401_at	ankyrin repeat and SOCS box-containing 13	Asb13	0.78279	1.13689037	0.16870736
1420683_at	BCL2/adenovirus E1B 19kD interacting protein like	Bnipl	0.78279	1.11605958	0.15350176
1443857_at	hook homolog 3 (Drosophila)	Hook3	0.78279	-1.11584332	-0.1687852
1428664_at	vasoactive intestinal polypeptide	Vip	0.7828	1.11674836	0.15322553
1419269_at	deoxyuridine triphosphatase	Dut	0.7828	1.09369543	0.12400919
1440166_x_at	5-hydroxytryptamine (serotonin) receptor 1D	Htr1d	0.78281	1.1141332	0.1497862
1442889_at	---	---	0.78281	-1.10963869	-0.1571061
1422787_at	FK506 binding protein-like	Fkbpl	0.78282	1.11490194	0.14563909
1450186_s_at	GNAS (guanine nucleotide binding protein, alpha stimulating) com	Gnas	0.78283	1.06727429	0.09345516
1450262_at	cardiotrophin-like cytokine factor 1	Clcf1	0.78283	-1.09519586	-0.1331699
1424037_at	inositol 1,4,5-trisphosphate 3-kinase A	Itpka	0.78283	1.09834688	0.13197251
1439009_at	guanine nucleotide binding protein-like 3 (nucleolar)-like	Gnl3l	0.78285	-1.07944657	-0.1141483
1430132_at	keratin 28	Krt28	0.78286	-1.09815815	-0.135313
1440842_at	RIKEN cDNA C230085N15 gene	C230085N15Rik	0.78287	1.11306207	0.15075564
1440512_at	---	---	0.78287	-1.10503215	-0.1459245
1451339_at	sulfite oxidase	Suox	0.78288	1.09689103	0.13110141
1447824_x_at	heat shock protein 5	Hspa5	0.78289	-1.07283424	-0.1019746
1422744_at	phosphorylase kinase alpha 1	Phka1	0.78289	-1.11439301	-0.1645806
1427908_at	BCL2/adenovirus E1B interacting protein 1	Bnip1	0.78289	1.10698234	0.13725155
1451858_at	MAS-related GPR, member A2A /// MAS-related GPR, member A	Mrgpra2a /// Mrgpra2b	0.7829	-1.10682603	-0.1501893
1444591_at	---	---	0.7829	1.09856542	0.13390943

1425052_at	---	---	0.78292	-1.07426683	-0.1049459
1439300_at	cysteine-rich hydrophobic domain 1	Chic1	0.78293	-1.10983516	-0.1539957
1459646_at	heparan sulfate (glucosamine) 3-O-sulfotransferase 6	Hs3st6	0.78293	1.09309193	0.12610945
1420106_at	Seven in absentia 1A	Siah1a	0.78294	-1.10113579	-0.146138
1430447_a_at	leukocyte-associated Ig-like receptor 1	Lair1	0.78295	1.10524205	0.14380247
1455174_at	ribosomal protein S19 binding protein 1	Rps19bp1	0.78297	1.08303456	0.10997819
1460582_x_at	predicted gene 14226	Gm14226	0.78297	-1.1114138	-0.1613518
1438172_x_at	exoribonuclease 2	Eri2	0.78297	-1.10280102	-0.1576213
1448327_at	actinin alpha 2	Actn2	0.78297	1.10188484	0.13995605
1434458_at	follistatin	Fst	0.78297	1.11276986	0.14881388
1458083_at	---	---	0.78298	-1.11195147	-0.1763351
1441185_at	Musashi homolog 2 (Drosophila)	Msi2	0.78298	1.13390608	0.16248812
1457662_x_at	thiamine pyrophosphokinase	Tpk1	0.78298	-1.10165067	-0.1398163
1455333_at	tensin 3	Tns3	0.78298	-1.07429744	-0.1078515
1429460_at	G protein-coupled receptor 115	Gpr115	0.78298	-1.1065642	-0.1526897
1428528_at	golgi to ER traffic protein 4 homolog (S. cerevisiae)	Get4	0.783	1.07523817	0.10360739
1460191_at	YKT6 homolog (S. Cerevisiae)	Ykt6	0.783	1.08547376	0.11559587
1443542_at	---	---	0.78301	1.09724147	0.12636017
1454389_at	Leucine-rich repeats and calponin homology (CH) domain contain	Lrch4	0.78301	1.13288999	0.16804456
1439698_at	zinc finger protein (C2H2 type) 276	Zfp276	0.78302	1.10773183	0.14655821
1431963_at	RIKEN cDNA 4930556A20 gene	4930556A20Rik	0.78302	-1.11026659	-0.1545803
1459330_at	---	---	0.78302	1.11249163	0.1461734
1460004_x_at	syntaxin 6	Stx6	0.78303	1.08064768	0.10905763
1422474_at	phosphodiesterase 4B, cAMP specific	Pde4b	0.78303	-1.12180331	-0.1816032
1417748_x_at	forkhead box M1	Foxm1	0.78303	1.08991077	0.12185332
1418354_at	component of oligomeric golgi complex 1	Cog1	0.78303	1.09690482	0.12832327
1450422_a_at	KDEL (Lys-Asp-Glu-Leu) containing 1	Kdelc1	0.78303	1.0856897	0.11561307
1449888_at	endothelial PAS domain protein 1	Epas1	0.78303	-1.08887648	-0.1293446
1434674_at	lysosomal trafficking regulator	Lyst	0.78304	1.10132072	0.13634366
1429564_at	polycomb group ring finger 5	Pcgf5	0.78304	1.08584426	0.11686054
1458237_at	UDP glycosyltransferases 3 family, polypeptide A1 pseudogene	Gm5219	0.78305	1.10217689	0.13225366
1441305_at	neural precursor cell expressed, developmentally down-regulated	Nedd4l	0.78305	-1.10738764	-0.1573329
1434755_at	coronin, actin binding protein, 2B	Coro2b	0.78305	1.09813345	0.13082363
1442191_at	RIKEN cDNA 5033411D12 gene	5033411D12Rik	0.78305	1.11976572	0.15508987
1446286_at	---	---	0.78306	-1.10902748	-0.1567287
1433295_at	RIKEN cDNA 4930449I04 gene	4930449I04Rik	0.78306	-1.10783109	-0.1583669
1447887_x_at	versican	Vcan	0.78307	1.08799111	0.12009791
1427831_s_at	zinc finger protein 260	Zfp260	0.78307	-1.11457495	-0.1821616
1429103_at	translocase of outer mitochondrial membrane 22 homolog (yeast	Tomm22	0.78307	1.07531636	0.10177325
1446163_at	---	---	0.78308	-1.11579406	-0.1732494
1447057_at	---	---	0.78308	-1.10723359	-0.1526538
1433831_at	DDB1 and CUL4 associated factor 17	Dcaf17	0.78308	-1.08730502	-0.1251136
1419239_at	zinc finger protein 54	Zfp54	0.78308	-1.12383701	-0.1941786
1459204_at	expressed sequence AU015449	AU015449	0.78308	1.12256173	0.15726704
1437947_x_at	voltage-dependent anion channel 1	Vdac1	0.7831	-1.06835741	-0.0978924
1423043_s_at	DEAD/H (Asp-Glu-Ala-Asp/His) box polypeptide 3, X-linked	Ddx3x	0.7831	-1.07287467	-0.1092135
1415976_a_at	calcium regulated heat stable protein 1	Carhsp1	0.78311	1.08829552	0.1173132
1456300_at	ilvB (bacterial acetolactate synthase)-like	Ilvbl	0.78311	1.15770169	0.18883167
1437927_at	discs, large homolog 2 (Drosophila)	Dlg2	0.78312	-1.09457272	-0.1308855
1418536_at	histocompatibility 2, Q region locus 7	H2-Q7	0.78312	1.13928458	0.17415026
1454718_at	N-acetylglucosamine-1-phosphodiester alpha-N-acetylglucosami	Nagpa	0.78313	1.078138	0.10833301
1445588_at	ankyrin repeat domain 44	Ankrd44	0.78313	1.12311669	0.15947846
1422569_at	YY1 transcription factor	Yy1	0.78314	-1.07348988	-0.1044236
1447620_at	cappuccino	Cno	0.78314	-1.09883806	-0.1403264
1453103_at	actin-binding LIM protein 1	Ablim1	0.78314	1.13129068	0.16777102
1457831_at	tripartite motif-containing 61	Trim61	0.78315	-1.10370444	-0.145251
1440844_at	transducer of ErbB-2.1	Tob1	0.78315	1.1156384	0.15143142
1453126_at	leucine rich repeat and fibronectin type III domain containing 2	Lrfn2	0.78316	1.10805666	0.14578605
1431172_at	origin recognition complex, subunit 4	Orc4	0.78317	-1.11420273	-0.1649295
1445995_at	cell division cycle 25 homolog A (S. pombe)	Cdc25a	0.78317	-1.08656744	-0.1259778
1421734_at	chemokine (C-X-C motif) receptor 2	Cxcr2	0.78318	-1.10810997	-0.1528309
1433385_at	RIKEN cDNA 4933428C20 gene	4933428C20Rik	0.78319	-1.10370828	-0.1435755
1444928_at	---	---	0.78319	1.12473198	0.15811595
1440922_at	RIKEN cDNA 9130208D14 gene	9130208D14Rik	0.78319	-1.09321026	-0.1355788
1439798_at	homeobox C10	Hoxc10	0.7832	-1.09279169	-0.1314624
1431294_at	spermatogenesis associated glutamate (E)-rich protein 7, pseudo	Speer7-ps1 /// Speer8-ps	0.7832	-1.10943397	-0.1583184
1438315_x_at	aldo-keto reductase family 7, member A5 (aflatoxin aldehyde red	Akr7a5	0.7832	1.0852481	0.1132717
1417404_at	ELOVL family member 6, elongation of long chain fatty acids (yea	Elovl6	0.78321	-1.07175796	-0.1009109
1429313_at	receptor tyrosine kinase-like orphan receptor 1	Ror1	0.78321	-1.10415237	-0.1478676
1458448_at	expressed sequence AA408296	AA408296	0.78321	1.10671794	0.14329076
1416962_at	regulator of chromosome condensation 1	Rcc1	0.78321	1.07421203	0.10170979
1448518_at	translocase of inner mitochondrial membrane 22 homolog (yeast	Timm22	0.78322	-1.10220746	-0.1428879
1444840_at	expressed sequence C87487	C87487	0.78322	-1.10741606	-0.1663743
1434949_at	armadillo repeat containing 8	Armc8	0.78322	-1.0792981	-0.1132194
1449494_at	RAB3C, member RAS oncogene family	Rab3c	0.78322	1.10846803	0.14638886
1456785_at	mediator complex subunit 14	Med14	0.78322	-1.09789089	-0.1390638
1422843_at	5'-3' exoribonuclease 2	Xrn2	0.78322	-1.11296592	-0.1657955
1432460_at	polymorphic derived intron containing	Pldi	0.78322	-1.1007294	-0.1388855
1438193_at	neurexin III	Nrxn3	0.78323	1.11278869	0.1509799
1436034_at	centrosomal protein 68	Cep68	0.78323	-1.08738927	-0.1294656
1415724_a_at	cell division cycle 42 homolog (S. cerevisiae)	Cdc42	0.78324	-1.07829528	-0.1141928
1415873_a_at	ARP1 actin-related protein 1 homolog A, centractin alpha (yeast)	Actr1a	0.78324	1.07351402	0.10210597
1449584_at	diacylglycerol kinase, gamma	Dgkg	0.78324	-1.10696007	-0.1473092
1437327_x_at	enolase-phosphatase 1	Enoph1	0.78324	-1.07808895	-0.1136762
1417934_at	DnaJ (Hsp40) homolog, subfamily C, member 4	Dnajc4	0.78324	1.08388075	0.11576195
1415903_at	solute carrier family 38, member 1	Slc38a1	0.78324	-1.08822949	-0.1324184
1428799_at	Leber congenital amaurosis 5 (human)	Lca5	0.78325	-1.10458374	-0.1538043
1455121_at	ligand dependent nuclear receptor corepressor	Lcor	0.78325	1.15078011	0.18204451
1420844_at	ubiquilin 2	Ubqln2	0.78325	-1.134723	-0.3617965
1426715_s_at	solute carrier family 46, member 1	Slc46a1	0.78326	1.08642005	0.11913726
1449709_s_at	arginyltransferase 1	Ate1	0.78327	-1.08252687	-0.1189528
1453691_at	RIKEN cDNA 4921528I07 gene	4921528I07Rik	0.78327	-1.11751432	-0.1788793
1427785_x_at	small optic lobes homolog (Drosophila)	Solh	0.78327	-1.08065425	-0.1132456
1423793_at	ATG13 autophagy related 13 homolog (S. cerevisiae)	Atg13	0.78327	1.09025498	0.12159547

1446187_at	---	---	0.78328	1.12845272	0.16435232
1446867_at	WD repeat and FYVE domain containing 4	Wdfy4	0.78328	-1.10405582	-0.1464109
1450034_at	signal transducer and activator of transcription 1	Stat1	0.78328	1.10869073	0.14280472
1430068_at	RIKEN cDNA C030011L09 gene	C030011L09Rik	0.78328	-1.10144454	-0.1440217
1416349_at	mitochondrial ribosomal protein L34	Mrpl34	0.78328	1.09898465	0.12632819
1416130_at	prion protein	Prnp	0.78329	1.12514304	0.15590745
1430418_at	transmembrane protein 57	Tmem57	0.78329	1.22380707	0.2324301
1417669_at	abhydrolase domain containing 12	Abhd12	0.78329	1.07912407	0.10784789
1447582_x_at	---	---	0.78329	1.09754363	0.13301887
1454695_at	WD repeat domain 18	Wdr18	0.78329	1.08816996	0.12167734
1453702_at	coiled-coil domain containing 105	Ccdc105	0.7833	-1.10484615	-0.151181
1421605_a_at	aquaporin 9	Aqp9	0.7833	-1.11455853	-0.1608736
1455204_at	phosphatidylinositol transfer protein, cytoplasmic 1	Pitpnc1	0.78331	-1.08774949	-0.1317411
1437807_x_at	catenin (cadherin associated protein), alpha 1	Ctnna1	0.78331	-1.07231626	-0.1078772
1435089_at	RIKEN cDNA 2010111I01 gene	2010111I01Rik	0.78331	1.09853422	0.1326044
1432263_a_at	cytochrome c oxidase subunit VIIa polypeptide 2-like	Cox7a2l	0.78332	1.06874932	0.09586553
1457065_at	uroplakin 1B	Upk1b	0.78332	-1.12049392	-0.1794741
1441900_x_at	Hspb associated protein 1	Hspbap1	0.78332	-1.08293478	-0.1181484
1445419_at	GTP-binding protein 8 (putative)	Gtpbp8	0.78333	1.10449173	0.14292701
1429252_at	RIKEN cDNA 0610010K14 gene	0610010K14Rik	0.78333	1.09711848	0.12458915
1433740_at	transmembrane protein 87B	Tmem87b	0.78333	1.09536305	0.12769989
1457668_x_at	DNA segment, Chr 4, ERATO Doi 617, expressed	D4Ertd617e	0.78333	1.12710796	0.16173081
1426024_a_at	drebrin 1	Dbn1	0.78334	1.0805893	0.11133216
1447543_at	WD repeat and FYVE domain containing 1	Wdfy1	0.78334	-1.10141535	-0.1750009
1416246_a_at	coronin, actin binding protein 1A	Coro1a	0.78334	1.12765799	0.16048472
1423506_a_at	neuronatin	Nnat	0.78335	1.15469263	0.18604001
1443223_at	---	---	0.78335	1.17252216	0.20095052
1445938_at	RIKEN cDNA 5930427L02 gene	5930427L02Rik	0.78335	-1.10907135	-0.155908
1439651_at	---	---	0.78336	-1.10848923	-0.1519791
1449271_a_at	heme binding protein 2	Hebp2	0.78336	1.09566117	0.12978464
1455163_at	GUF1 GTPase homolog (S. cerevisiae)	Guf1	0.78336	1.18181234	0.20560184
1426462_at	gephyrin	Gphn	0.78336	-1.0994209	-0.1503753
1426988_at	kelch domain containing 5	Klhdc5	0.78336	-1.10028496	-0.1411313
1438208_at	TAO kinase 2	Taok2	0.78337	1.10377064	0.13631483
1430376_at	leucine rich repeat containing 9	Lrrc9	0.78337	1.11648229	0.15403225
1430106_at	transformation related protein 53 target 5	Trp53tg5	0.78337	1.08972354	0.12160349
1438524_x_at	proline-rich nuclear receptor coactivator 1	Pnrc1	0.78337	-1.07641043	-0.1076842
1427045_at	synaptopodin	Synpo	0.78337	-1.10566166	-0.1483611
1431065_at	ELL associated factor 1	Eaf1	0.78338	1.11642616	0.14958058
1437890_at	BTB (POZ) domain containing 17	Btbd17	0.78338	1.10242346	0.14025051
1452370_s_at	RIKEN cDNA B230208H17 gene	B230208H17Rik	0.78338	-1.11884675	-0.1887632
1418908_at	peptidylglycine alpha-amidating monooxygenase	Pam	0.78338	1.10097141	0.1362346
1431285_at	mahogunin, ring finger 1	Mgrn1	0.7834	1.08670695	0.11697597
1437534_at	---	---	0.7834	-1.1008424	-0.1392957
1417725_a_at	Sjogren's syndrome/scleroderma autoantigen 1 homolog (human	Sssca1	0.7834	1.08572755	0.11293189
1429758_at	RIKEN cDNA 1700017B05 gene	1700017B05Rik	0.7834	1.08384693	0.11342314
1428279_a_at	ataxin 7-like 1	Atxn7l1	0.7834	1.09456236	0.12990597
1431705_a_at	mucolipin 2	Mcoln2	0.78341	1.11360327	0.15382961
1419954_s_at	zinc finger, AN1-type domain 3	Zfand3	0.78341	1.08780877	0.12040836
1458268_s_at	insulin-like growth factor binding protein 3	Igfbp3	0.78341	1.11513954	0.14888918
1423843_at	leucine rich repeat containing 61	Lrrc61	0.78341	1.0917891	0.12630499
1434326_x_at	coronin, actin binding protein, 2B	Coro2b	0.78341	1.12302604	0.15718032
1429520_a_at	alkaline ceramidase 3	Acer3	0.78341	1.0860845	0.11904446
1451100_a_at	carnitine deficiency-associated gene expressed in ventricle 3	Cdv3	0.78342	-1.08583563	-0.1230919
1419576_at	homeobox B13	Hoxb13	0.78342	1.1182338	0.15397989
1432518_at	RIKEN cDNA 4930430J20 gene	4930430J20Rik	0.78343	1.10949838	0.14626198
1419542_at	deleted in azoospermia-like	Dazl	0.78343	-1.08385131	-0.1182175
1435114_at	WD repeat and HMG-box DNA binding protein 1	Wdhd1	0.78343	1.08008864	0.10746702
1449803_x_at	transporter 1, ATP-binding cassette, sub-family B (MDR/TAP)	Tap1	0.78343	1.14202105	0.17454718
1425271_at	proteasome (prosome, macropain) 26S subunit, ATPase 3, interac	Psmc3ip	0.78343	1.08935289	0.11927595
1436255_at	RIKEN cDNA D830044I16 gene	D830044I16Rik	0.78343	-1.11051222	-0.1599659
1434349_at	valyl-tRNA synthetase 2, mitochondrial (putative)	Vars2	0.78343	1.0977661	0.13336641
1426040_a_at	outer dense fiber of sperm tails 2	Odf2	0.78344	1.08314841	0.11448751
1430589_at	phosphatidylinositol glycan anchor biosynthesis, class H	Pigh	0.78344	1.10490304	0.14172064
1416494_at	NADH dehydrogenase (ubiquinone) Fe-S protein 5	Ndufs5	0.78344	1.1198679	0.14660606
1447296_at	F-box protein 27	Fbxo27	0.78344	1.12014978	0.15581891
1453482_at	CASP8 and FADD-like apoptosis regulator	Cflar	0.78344	1.15331142	0.18362702
1418517_at	Iroquois related homeobox 3 (Drosophila)	Irx3	0.78345	1.11528615	0.1533016
1420128_s_at	phosphatidylinositol glycan anchor biosynthesis, class B	Pigb	0.78345	-1.12177062	-0.1927377
1455928_x_at	leucine-zipper-like transcriptional regulator, 1	Lztr1	0.78345	-1.10923605	-0.175933
1456488_at	WD repeat domain 33	Wdr33	0.78345	-1.10035959	-0.1449689
1441176_at	---	---	0.78346	1.10000231	0.13714119
1438720_at	RIKEN cDNA 9330159F19 gene	9330159F19Rik	0.78346	-1.1101222	-0.1568122
1419665_a_at	nuclear protein 1	Nupr1	0.78346	-1.11724479	-0.1985073
1428182_at	phosphoribosyl pyrophosphate synthetase-associated protein 1	Prpsap1	0.78346	1.07650624	0.10302825
1438145_at	SPT2, Suppressor of Ty, domain containing 1 (S. cerevisiae)	Spty2d1	0.78346	-1.10948819	-0.1734501
1419417_at	vascular endothelial growth factor C	Vegfc	0.78346	1.08446136	0.11295027
1456723_at	---	---	0.78346	1.12248082	0.15823524
1415945_at	minichromosome maintenance deficient 5, cell division cycle 46 (S	Mcm5	0.78347	1.06347397	0.08745911
1428047_s_at	zinc finger protein, autosomal /// zinc finger protein X-linked	Zfa /// Zfx	0.78347	-1.10191459	-0.1414021
1453800_at	ring finger protein 181	Rnf181	0.78347	-1.11518441	-0.1707779
1458478_at	RIKEN cDNA 9230110F11 gene	9230110F11Rik	0.78348	-1.11176483	-0.1564974
1459336_at	Guanylate cyclase 1, soluble, alpha 2	Gucy1a2	0.78348	-1.10390032	-0.1481222
1432264_x_at	cytochrome c oxidase subunit VIIa polypeptide 2-like	Cox7a2l	0.78348	1.06704187	0.09328538
1423393_at	chloride intracellular channel 4 (mitochondrial)	Clic4	0.78348	-1.07897408	-0.1165946
1446602_at	---	---	0.78349	1.10672881	0.13827707
1433894_at	JAZF zinc finger 1	Jazf1	0.78349	-1.08783096	-0.1240193
1427968_at	TGFB-induced factor homeobox 2-like, X-linked 1 /// TGFB-induce	Tgif2lx1 /// Tgif2lx2	0.78349	1.10505292	0.14008254
1450849_at	heterogeneous nuclear ribonucleoprotein U	Hnrnpu	0.78349	-1.07496136	-0.1106855
1447275_at	Bardet-Biedl syndrome 12 (human)	Bbs12	0.78349	1.11834575	0.1569116
1438898_at	hypothetical LOC100504583	LOC100504583	0.78349	1.10642748	0.13853798
1434258_s_at	phosphatase and actin regulator 4	Phactr4	0.7835	1.09052504	0.12019259
1430040_at	heat shock protein 12A	Hspa12a	0.7835	1.10454691	0.14064078
1433030_at	RIKEN cDNA 5730406E14 gene	5730406E14Rik	0.7835	1.12037789	0.15555402

1447538_at	---	---	0.7835	1.10205407	0.13682455
1454827_at	pogo transposable element with ZNF domain	Pogz	0.7835	1.09767443	0.13186964
1453166_at	coiled-coil domain containing 109A	Ccdc109a	0.7835	1.0804119	0.11141203
1446688_at	---	---	0.78351	1.10066008	0.13820025
1421565_at	roundabout homolog 3 (Drosophila)	Robo3	0.78351	1.24126564	0.24837376
1421824_at	beta-site APP cleaving enzyme 1	Bace1	0.78351	1.11279471	0.14468915
1436330_x_at	predicted gene 7072	Gm7072	0.78351	-1.10808271	-0.1639346
1416907_at	translin	Tsn	0.78351	-1.07558319	-0.1081156
1457387_at	Replication initiator 1	Repin1	0.78351	-1.10831997	-0.157602
1443750_s_at	ribonuclease P 40 subunit (human)	Rpp40	0.78351	1.08535123	0.11539668
1428635_at	catechol-O-methyltransferase domain containing 1	Comtd1	0.78352	1.09910337	0.13042034
1434306_at	RAB3A interacting protein	Rab3ip	0.78352	1.08630419	0.11783842
1416584_at	mannosidase 2, alpha B2	Man2b2	0.78352	1.09185206	0.12397455
1439957_at	guanine nucleotide binding protein, alpha stimulating, olfactory ty	Gnal	0.78352	1.11464322	0.15300474
1443350_at	metastasis associated 3	Mta3	0.78352	1.09314484	0.12730645
1446427_at	zinc finger, GATA-like protein 1	Zglp1	0.78353	1.09956311	0.13258692
1427556_at	myosin, light polypeptide kinase 2, skeletal muscle	Mylk2	0.78353	-1.11246856	-0.1633168
1458535_at	---	---	0.78353	-1.0880674	-0.1218249
1435777_at	inositol 1,4,5-triphosphate receptor interacting protein-like 2	Itpripl2	0.78353	-1.12822449	-0.2026716
1420077_at	---	---	0.78353	1.10415617	0.14166785
1426161_at	---	---	0.78354	-1.09963661	-0.1449802
1417493_at	Bmi1 polycomb ring finger oncogene	Bmi1	0.78354	-1.08840035	-0.1232731
1434500_at	tweety homolog 2 (Drosophila)	Ttyh2	0.78355	1.11866826	0.15171641
1448681_at	interleukin 15 receptor, alpha chain	Il15ra	0.78355	1.10855148	0.14838509
1432199_at	RIKEN cDNA 4930568D16 gene	4930568D16Rik	0.78355	1.09905	0.1345607
1460493_at	ataxin 7-like 3B	Atxn7l3b	0.78355	-1.11030102	-0.1641543
1446631_at	predicted gene 14047	Gm14047	0.78356	1.09377952	0.12796515
1417897_at	breast cancer metastasis-suppressor 1	Brms1	0.78356	1.10402722	0.13440328
1426557_at	mesoderm posterior 1	Mesp1	0.78357	1.10629616	0.14070868
1428183_at	ankyrin repeat domain 23	Ankrd23	0.78357	-1.10382312	-0.1444109
1428316_a_at	FUN14 domain containing 2	Fundc2	0.78358	-1.07555514	-0.1091207
1419939_at	Rho guanine nucleotide exchange factor (GEF) 17	Arhgef17	0.78358	-1.10057699	-0.1384093
1448260_at	ubiquitin carboxy-terminal hydrolase L1	Uchl1	0.78358	1.1154852	0.14461054
1453412_a_at	SEC14-like 1 (S. cerevisiae)	Sec14l1	0.78358	1.10829083	0.13602825
1452997_at	cadherin 11 pseudogene	2610005L07Rik	0.78358	-1.07258633	-0.1038902
1424068_at	T-cell leukemia translocation altered gene	Tcta	0.78358	-1.10149858	-0.1401844
1448347_a_at	cell cycle associated protein 1 /// hypothetical LOC100504494	Caprin1 /// LOC10050449	0.78358	-1.05498869	-0.0800729
1451878_a_at	junction-mediating and regulatory protein	Jmy	0.78358	-1.1231366	-0.2071774
1428015_at	synaptojanin 2 binding protein	Synj2bp	0.78358	-1.09666094	-0.1342423
1419581_at	discs, large homolog 4 (Drosophila)	Dlg4	0.78358	-1.1166366	-0.169939
1438012_at	protein phosphatase 1 (formerly 2C)-like	Ppm1l	0.78359	1.09969957	0.13647357
1459964_at	---	---	0.7836	-1.10405043	-0.146616
1455263_at	RIKEN cDNA 9030625A04 gene	9030625A04Rik	0.78361	1.09399165	0.12879279
1454511_at	RIKEN cDNA 9430018C23 gene	9430018C23Rik	0.78361	-1.10008098	-0.1450443
1428702_at	DEAD (Asp-Glu-Ala-Asp) box polypeptide 28	Ddx28	0.78361	1.08334701	0.11299053
1451361_a_at	patatin-like phospholipase domain containing 7	Pnpla7	0.78361	1.10331953	0.13765325
1440420_at	---	---	0.78361	1.11510182	0.15588373
1425055_at	protein phosphatase 6, regulatory subunit 2	Ppp6r2	0.78362	1.10188532	0.13415766
1431343_at	predicted gene 10044	Gm10044	0.78362	-1.12206777	-0.1931065
1428216_s_at	translocase of outer mitochondrial membrane 7 homolog (yeast)	Tomm7	0.78363	1.10603005	0.14175683
1416693_at	forkhead box C2	Foxc2	0.78363	-1.10437696	-0.152313
1449852_a_at	EH-domain containing 4	Ehd4	0.78363	1.12981324	0.15836931
1441991_at	protease, serine, 53	Prss53	0.78363	1.10241999	0.14028424
1455525_at	endo/exonuclease (5'-3'), endonuclease G-like	Exog	0.78363	1.07662876	0.10318669
1432617_at	RIKEN cDNA 4933424C09 gene	4933424C09Rik	0.78364	-1.10568219	-0.1505383
1458452_at	Ankyrin repeat domain 11	Ankrd11	0.78364	-1.10600584	-0.1559821
1430270_at	RIKEN cDNA 4933411K05 gene	4933411K05Rik	0.78365	1.10634105	0.14415815
1460611_at	expressed sequence C87499	C87499	0.78365	-1.11531075	-0.1626938
1453260_a_at	protein phosphatase 2 (formerly 2A), regulatory subunit B (PR 52)	Ppp2r2a	0.78365	-1.08155108	-0.12228
1420261_at	presenilin 1	Psen1	0.78366	1.11061314	0.14608393
1423635_at	bone morphogenetic protein 2	Bmp2	0.78367	1.097664	0.13154958
1432153_at	RIKEN cDNA 4933426B08 gene	4933426B08Rik	0.78367	-1.10667642	-0.156702
1415814_at	ATPase, H+ transporting, lysosomal V1 subunit B2	Atp6v1b2	0.78367	1.07130178	0.09867907
1417229_at	calpain 1	Capn1	0.78367	-1.09381295	-0.1407199
1430454_x_at	BCL2-like 2	Bcl2l2	0.78368	-1.11386654	-0.1735459
1446540_at	---	---	0.78369	-1.10656594	-0.1481372
1448749_at	pleckstrin	Plek	0.78369	-1.08906352	-0.1239804
1421128_at	zinc finger (CCCH type), RNA binding motif and serine/arginine ricl	Zrsr2	0.7837	-1.08684756	-0.1207478
1416251_at	minichromosome maintenance deficient 6 (MIS5 homolog, S. por	Mcm6	0.7837	1.05911433	0.08234549
1420339_at	mediator of cell motility 1	Memo1	0.7837	-1.09497558	-0.1337535
1426951_at	cysteine rich transmembrane BMP regulator 1 (chordin like)	Crim1	0.7837	1.1807836	0.20435707
1417924_at	p21 protein (Cdc42/Rac)-activated kinase 3	Pak3	0.78371	-1.09910653	-0.1392515
1434587_x_at	phosphatidylserine synthase 2	Ptdss2	0.78371	1.06853994	0.09551156
1456836_at	IL2-inducible T-cell kinase	Itk	0.78371	-1.10836257	-0.1514468
1435895_at	limbic system-associated membrane protein	Lsamp	0.78371	-1.11104671	-0.1527464
1453584_at	serine/threonine kinase 36 (fused homolog, Drosophila)	Stk36	0.78371	-1.09959796	-0.1378638
1431940_at	hypothetical protein LOC74457	LOC74457	0.78372	1.15431465	0.18766498
1432553_at	RIKEN cDNA 1700069L16 gene	1700069L16Rik	0.78372	1.10750105	0.14487332
1443059_at	hydroxysteroid (17-beta) dehydrogenase 11	Hsd17b11	0.78372	1.12098232	0.15754114
1433021_at	---	---	0.78373	1.08760237	0.11530842
1443851_at	protein phosphatase 4, regulatory subunit 4	Ppp4r4	0.78373	-1.10811878	-0.1489809
1424331_at	Rab40c, member RAS oncogene family	Rab40c	0.78373	1.09728375	0.12456109
1419414_at	guanine nucleotide binding protein (G protein), gamma 13	Gng13	0.78374	1.1745998	0.19948737
1428286_at	RIKEN cDNA 2900097C17 gene /// hypothetical LOC100503107	2900097C17Rik /// LOC10	0.78375	-1.06861048	-0.0957558
1450679_at	suppressor of IKBKE 1	Sike1	0.78375	1.09188194	0.12371048
1417126_a_at	ribosomal protein L22 like 1	Rpl22l1	0.78376	-1.0759589	-0.1169814
1444796_at	expressed sequence C76876	C76876	0.78376	-1.10044834	-0.1384952
1418413_at	caveolin 3	Cav3	0.78377	-1.11941804	-0.1824713
1428059_at	---	---	0.78378	1.13475742	0.17004124
1451790_a_at	tissue factor pathway inhibitor	Tfpi	0.78378	1.07359734	0.10033956
1426874_at	enhancer of mRNA decapping 4	Edc4	0.7838	1.0814054	0.10962674
1450897_at	Rho GTPase activating protein 5	Arhgap5	0.78381	-1.07635183	-0.1073313
1433155_at	RIKEN cDNA B230204H03 gene	B230204H03Rik	0.78381	-1.12811599	-0.2006971
1441404_at	---	---	0.78382	-1.11225476	-0.1641753

1455792_x_at	necdin	Ndn	0.78382	1.15049362	0.17821792
1433362_at	RIKEN cDNA 4930527F18 gene	4930527F18Rik	0.78383	1.11100437	0.14942244
1429834_a_at	RIKEN cDNA 1110014N23 gene	1110014N23Rik	0.78384	1.10590381	0.13697332
1451554_a_at	anterior pharynx defective 1a homolog (C. elegans)	Aph1a	0.78384	1.10895962	0.14171313
1444788_x_at	---	---	0.78385	-1.11630119	-0.1767385
1420862_at	dynactin 4	Dctn4	0.78385	1.08570198	0.11640481
1457427_at	adherens junction associated protein 1	Ajap1	0.78386	1.11746875	0.15337701
1418922_at	cell adhesion molecule 3	Cadm3	0.78386	-1.10059178	-0.1386609
1445108_at	---	---	0.78388	-1.09694278	-0.1374988
1437063_at	feminization 1 homolog a (C. elegans)	Fem1a	0.78388	-1.10963434	-0.1641684
1415984_at	acyl-Coenzyme A dehydrogenase, medium chain	Acadm	0.78388	-1.07021034	-0.0997744
1431799_at	myeloid/lymphoid or mixed-lineage leukemia (trithorax homolog,	Mllt4	0.78388	1.14916562	0.18031672
1458879_at	expressed sequence C76798	C76798	0.78389	-1.12695662	-0.2060549
1442116_at	G protein-coupled receptor 176	Gpr176	0.78391	1.12472568	0.16066844
1450101_a_at	membrane associated guanylate kinase, WW and PDZ domain co	Magi3	0.78391	1.13593536	0.17087751
1434757_at	core-binding factor, runt domain, alpha subunit 2, translocated to,	Cbfa2t2	0.78391	1.07009135	0.09714757
1447849_s_at	avian musculoaponeurotic fibrosarcoma (v-maf) AS42 oncogene	Maf	0.78392	-1.08760519	-0.1225412
1417050_at	C1q and tumor necrosis factor related protein 4	C1qtnf4	0.78393	1.14251145	0.17741079
1443392_at	transient receptor potential cation channel, subfamily V, member	Trpv1	0.78393	1.10625332	0.14035796
1431004_at	lysyl oxidase-like 2	Loxl2	0.78393	1.12568171	0.15796504
1459408_at	---	---	0.78394	-1.09757505	-0.1362065
1455882_x_at	von Willebrand factor C domain containing 2	Vwc2	0.78395	-1.11360836	-0.1756032
1451657_a_at	ecto-NOX disulfide-thiol exchanger 2	Enox2	0.78396	1.10629482	0.14490084
1451787_at	cytochrome P450, family 2, subfamily b, polypeptide 10	Cyp2b10	0.78396	1.12134167	0.15333732
1435802_at	zinc finger and BTB domain containing 45	Zbtb45	0.78397	1.08170784	0.11042354
1447196_at	predicted gene 9789	Gm9789	0.78398	-1.08407407	-0.1224208
1422040_at	sema domain, immunoglobulin domain (Ig), and GPI membrane a	Sema7a	0.78398	1.10082489	0.13854581
1438017_at	RUN and SH3 domain containing 1	Rusc1	0.78398	1.09413895	0.1287205
1457845_at	---	---	0.78399	-1.11588573	-0.1726929
1453059_at	RIKEN cDNA 2310046A06 gene	2310046A06Rik	0.78399	1.1307103	0.1666946
1421387_at	kringle containing transmembrane protein 1	Kremen1	0.784	-1.10928732	-0.1514998
1455915_at	UDP-N-acetyl-alpha-D-galactosamine:polypeptide N-acetylgalact	Galnt4	0.78401	1.1684566	0.19665073
1454586_at	hypothetical LOC100503673	LOC100503673	0.78401	1.09160371	0.12597898
1453143_at	galactosidase, beta 1 like 3	Glb1l3	0.78402	-1.07998784	-0.1128281
1425231_a_at	zinc finger protein 46	Zfp46	0.78402	1.10199258	0.1352528
1416598_at	GLIS family zinc finger 2	Glis2	0.78402	1.0910402	0.12055295
1422535_at	cyclin E2	Ccne2	0.78403	-1.09711116	-0.140797
1448432_at	phospholipase C, delta 1	Plcd1	0.78404	1.10951366	0.14238633
1453669_at	RIKEN cDNA 4930578C19 gene	4930578C19Rik	0.78404	-1.08862681	-0.1249516
1458497_at	---	---	0.78404	-1.09308638	-0.1289664
1423952_a_at	keratin 7	Krt7	0.78405	1.13182744	0.16706084
1454750_a_at	DDB1 and CUL4 associated factor 15	Dcaf15	0.78406	1.09412987	0.12217034
1452473_at	proline rich 15	Prr15	0.78406	-1.10705628	-0.1567465
1427406_at	thyroid hormone receptor interactor 11	Trip11	0.78406	-1.11624022	-0.1788283
1454669_at	transmembrane protein 11	Tmem11	0.78407	1.07906419	0.10707764
1450597_at	olfactory receptor 870	Olfr870	0.78407	-1.12323884	-0.1876443
1421636_at	TBC1 domain family, member 8B	Tbc1d8b	0.78407	-1.0931932	-0.1292767
1459594_at	listerin E3 ubiquitin protein ligase 1	Ltn1	0.78408	1.10807266	0.14339716
1440352_at	RIKEN cDNA 1700028E10 gene	1700028E10Rik	0.78408	1.11650343	0.15271162
1422084_at	BMX non-receptor tyrosine kinase	Bmx	0.78408	1.12564438	0.16130977
1458314_at	---	---	0.78408	1.10903928	0.14527942
1459641_at	---	---	0.78409	1.09327785	0.12683884
1450393_a_at	adenosine deaminase, tRNA-specific 3, TAD2 homolog (S. cerevis	Adat3 /// Scamp4	0.78409	1.07465371	0.10380387
1454774_at	zinc finger protein 445	Zfp445	0.78409	-1.07362591	-0.1038232
1441352_at	RIKEN cDNA 9430034N14 gene	9430034N14Rik	0.78409	-1.10087368	-0.1418661
1442979_at	---	---	0.78409	-1.09685164	-0.1373329
1439347_at	tensin 4	Tns4	0.7841	-1.10924173	-0.1536295
1426830_a_at	S-adenosylhomocysteine hydrolase-like 1	Ahcyl1	0.7841	1.075424	0.10327789
1425191_at	occludin/ELL domain containing 1	Ocel1	0.7841	1.10699797	0.14232748
1436479_a_at	dipeptidylpeptidase 7	Dpp7	0.7841	1.13517812	0.16335483
1422571_at	thrombospondin 2	Thbs2	0.78411	1.10254343	0.13965981
1451283_at	family with sequence similarity 114, member A2	Fam114a2	0.78411	-1.08321372	-0.1181895
1415739_at	RNA binding motif protein 42	Rbm42	0.78412	1.08149335	0.10902521
1427976_at	oogenesin 1	Oog1	0.78412	-1.08089541	-0.1167734
1416594_at	secreted frizzled-related protein 1	Sfrp1	0.78412	1.08158889	0.11155523
1455922_at	RAB3 GTPase activating protein subunit 1	Rab3gap1	0.78413	-1.08040208	-0.1163098
1415901_at	procollagen-lysine, 2-oxoglutarate 5-dioxygenase 3	Plod3	0.78413	1.06792431	0.09422649
1446776_at	expressed sequence C77581	C77581	0.78414	1.10903255	0.14708278
1419294_at	RIKEN cDNA 1700011H14 gene	1700011H14Rik	0.78415	1.09632575	0.12921646
1451067_at	small glutamine-rich tetratricopeptide repeat (TPR)-containing, al	Sgta	0.78416	1.07770576	0.1049188
1452636_x_at	GTP binding protein 5	Gtpbp5	0.78416	1.08875665	0.11940529
1434080_at	AE binding protein 2	Aebp2	0.78416	-1.07269978	-0.1027499
1432258_at	RIKEN cDNA 9430014N10 gene	9430014N10Rik	0.78416	-1.12637736	-0.2002628
1423992_at	GATA zinc finger domain containing 2A	Gatad2a	0.78416	1.08140351	0.10720069
1418192_at	max binding protein	Mnt	0.78417	1.0903848	0.12157862
1419052_at	OVO homolog-like 1 (Drosophila)	Ovol1	0.78417	-1.09982567	-0.1381068
1460506_s_at	NADH dehydrogenase (ubiquinone) 1, subcomplex unknown, 2	Ndufc2	0.78417	1.07228738	0.09918616
1456218_at	sorting nexin 22	Snx22	0.78417	-1.11136047	-0.1564332
1444061_at	proline rich 9	Prr9	0.78418	-1.08925461	-0.1261377
1460024_at	trinucleotide repeat containing 6b	Tnrc6b	0.78418	-1.09218876	-0.1311413
1443052_at	RIKEN cDNA C330019L16 gene /// predicted gene 2381	C330019L16Rik /// Gm23	0.78418	-1.08111832	-0.1172071
1419357_at	ISY1 splicing factor homolog (S. cerevisiae)	Isy1	0.78418	1.08094104	0.11152402
1432941_at	RIKEN cDNA 5730408A14 gene	5730408A14Rik	0.78418	1.11809672	0.14438567
1453414_at	yippee-like 2 (Drosophila)	Ypel2	0.78418	-1.09509831	-0.1312239
1432952_at	RIKEN cDNA 4930448E22 gene	4930448E22Rik	0.78418	1.10405555	0.1418389
1424448_at	tripartite motif-containing 6	Trim6	0.78418	1.0707227	0.09830634
1440087_at	---	---	0.78419	1.11483065	0.14377482
1422505_at	chromatin accessibility complex 1	Chrac1	0.78419	1.08511972	0.11515699
1431000_at	coiled-coil domain containing 75	Ccdc75	0.78421	-1.10472508	-0.1473325
1429090_at	gamma-glutamyl cyclotransferase	Ggct	0.78421	-1.10798215	-0.157025
1421292_a_at	cofactor of BRCA1	Cobra1	0.78421	1.07657513	0.10478861
1454215_at	RIKEN cDNA 2410007B07 gene	2410007B07Rik	0.78421	1.0767213	0.10662643
1418099_at	tumor necrosis factor receptor superfamily, member 1b	Tnfrsf1b	0.78421	1.11039915	0.14541596
1457125_at	---	---	0.78422	1.10729848	0.14406168

1441557_at	SET domain containing (lysine methyltransferase) 8	Setd8	0.78422	-1.10202512	-0.1429658
1447107_at	DEAD (Asp-Glu-Ala-Asp) box polypeptide 55	Ddx55	0.78422	1.09982757	0.13650269
1442397_at	nuclear transcription factor, X-box binding 1	Nfx1	0.78422	1.09549578	0.12882611
1450650_at	myosin X	Myo10	0.78423	1.07836367	0.10554491
1433886_at	eukaryotic translation initiation factor 2B, subunit 5 epsilon	Eif2b5	0.78423	1.07228941	0.09943099
1453108_at	arylsulfatase K	Arsk	0.78423	-1.10123765	-0.1409507
1452871_at	nei endonuclease VIII-like 1 (E. coli)	Neil1	0.78424	1.08622488	0.11861952
1443501_at	---	---	0.78424	1.09888558	0.13325759
1426825_at	formin-like 3	Fmnl3	0.78424	1.08439898	0.11551164
1455647_at	androgen receptor	Ar	0.78424	1.1316204	0.16717706
1453202_at	RIKEN cDNA E330016A19 gene	E330016A19Rik	0.78424	1.0926129	0.12619509
1459049_at	---	---	0.78425	-1.08491756	-0.1191202
1453367_a_at	abhydrolase domain containing 12	Abhd12	0.78425	1.07172267	0.0996238
1423799_at	eukaryotic translation initiation factor 1	Eif1	0.78425	1.06246726	0.08691144
1417972_s_at	processing of precursor 5, ribonuclease P/MRP family (S. cerevisia	Pop5	0.78426	1.09278625	0.11939842
1436839_at	RIKEN cDNA 2010109K11 gene	2010109K11Rik	0.78426	1.10259247	0.13854807
1425643_at	glycophorin A	Gypa	0.78426	-1.11276102	-0.1669109
1438712_at	DENN/MADD domain containing 2D	Dennd2d	0.78427	1.12433903	0.16251797
1428752_at	solute carrier family 5 (sodium/glucose cotransporter), member 1	Slc5a11	0.78427	-1.09378266	-0.1306479
1424942_a_at	myelocytomatosis oncogene	Myc	0.78429	1.08350989	0.11348388
1433212_at	RIKEN cDNA 2700008E08 gene	2700008E08Rik	0.78429	1.10188287	0.13574824
1436955_at	tumor suppressing subtransferable candidate 1	Tssc1	0.78429	1.08577825	0.1134076
1458283_at	predicted gene 12758	Gm12758	0.78429	-1.10519278	-0.1487995
1424637_s_at	coiled-coil domain containing 47	Ccdc47	0.7843	-1.07413295	-0.1037374
1416657_at	thymoma viral proto-oncogene 1	Akt1	0.78431	1.10411338	0.1336771
1424124_at	motile sperm domain containing 2	Mospd2	0.78431	-1.08380849	-0.1168701
1431797_at	RIKEN cDNA 4921518K17 gene	4921518K17Rik	0.78431	-1.10987447	-0.1599814
1428883_at	transmembrane protein 57	Tmem57	0.78431	1.07156573	0.09861938
1455671_at	COMM domain containing 8	Commd8	0.78432	-1.09325762	-0.1315833
1447178_at	---	---	0.78432	-1.09511226	-0.1376723
1433551_at	vesicle amine transport protein 1 homolog-like (T. californica)	Vat1l	0.78434	-1.1061071	-0.1523193
1415742_at	ancient ubiquitous protein 1	Aup1	0.78434	1.10490469	0.13933823
1417755_at	topoisomerase I binding, arginine/serine-rich	Topors	0.78435	-1.08193954	-0.1150263
1441448_at	Pumilio 2 (Drosophila)	Pum2	0.78435	-1.09758653	-0.1398088
1419365_at	peroxisomal biogenesis factor 11 alpha	Pex11a	0.7844	1.11007853	0.14445235
1431567_at	RIKEN cDNA 1700018A04 gene	1700018A04Rik	0.78441	1.14145788	0.17593277
1433645_at	solute carrier family 44, member 1	Slc44a1	0.78442	1.07066935	0.09718761
1438068_at	---	---	0.78466	1.0902806	0.12341958
1456745_x_at	ERGIC and golgi 3	Ergic3	0.78467	1.07918731	0.10903137
1448588_at	CD320 antigen	Cd320	0.7847	-1.0960956	-0.135057
1419424_at	pancreas specific transcription factor, 1a	Ptf1a	0.78471	1.11579317	0.15422912
1424662_at	peptidase (mitochondrial processing) alpha	Pmpca	0.78471	1.06807991	0.09482969
1448693_at	glycolipid transfer protein domain containing 1	Gltpd1	0.78471	1.08600097	0.11789522
1433686_at	calcineurin binding protein 1	Cabin1	0.78472	1.07983661	0.11080727
1423176_at	transducer of ErbB-2.1	Tob1	0.78474	-1.07411753	-0.1072003
1421660_at	sodium channel, voltage-gated, type IX, alpha	Scn9a	0.78479	-1.07874093	-0.1095113
1459369_at	---	---	0.78479	1.08159248	0.11064818
1437113_s_at	phospholipase D1	Pld1	0.7848	-1.1050446	-0.1503011
1430615_at	tubulin tyrosine ligase-like family, member 7	Ttll7	0.78482	-1.10217314	-0.1403675
1451593_at	Histocompatibility 2, K1, K region	H2-K1	0.78483	-1.0908804	-0.1280191
1434708_at	von Hippel-Lindau tumor suppressor	Vhl	0.78484	1.08359072	0.11570411
1460358_s_at	nudix (nucleoside diphosphate linked moiety X)-type motif 22	Nudt22	0.78488	1.08665813	0.11911161
1428751_at	PARK2 co-regulated	Pacrg	0.78489	-1.11234366	-0.1628786
1420708_at	TRAF-interacting protein	Traip	0.7849	1.09267015	0.12608313
1447551_x_at	latrophilin 3	Lphn3	0.78491	-1.1243559	-0.2269866
1441137_at	bicaudal C homolog 1 (Drosophila)	Bicc1	0.78494	-1.10835557	-0.1575289
1425531_at	zinc finger, HIT domain containing 1	Znhit1	0.78496	1.10441709	0.13248675
1440211_at	cytochrome P450, family 2, subfamily j, polypeptide 11, pseudoge	Cyp2j11-ps	0.78497	-1.10413208	-0.1448297
1424684_at	RAB5C, member RAS oncogene family	Rab5c	0.785	1.09877627	0.12728794
1420359_at	seminal vesicle antigen	Sva	0.78501	-1.10966941	-0.1671549
1433268_at	RIKEN cDNA 4930553M12 gene	4930553M12Rik	0.78502	-1.1036232	-0.1474985
1420411_a_at	phosphatidylinositol 4-kinase type 2 beta	Pi4k2b	0.78503	-1.09914845	-0.1431127
1431960_at	WW domain-containing oxidoreductase	Wwox	0.78503	-1.10084756	-0.1445218
1431830_at	zinc finger protein 329	Zfp329	0.78504	-1.0984591	-0.139319
1416213_x_at	surfeit gene 4	Surf4	0.78505	1.09428468	0.12357285
1438241_at	RGM domain family, member A	Rgma	0.78507	-1.11383377	-0.1648294
1424401_at	aldehyde dehydrogenase 1 family, member L1	Aldh1l1	0.78507	1.12150363	0.15797815
1417914_at	RAP2B, member of RAS oncogene family	Rap2b	0.78508	-1.08576355	-0.1248911
1421102_a_at	vesicle-associated membrane protein 3	Vamp3	0.78509	1.08891825	0.11792038
1450632_at	ras homolog gene family, member A	Rhoa	0.78509	-1.07256609	-0.1046166
1419065_at	nephrocan	Nepn	0.78511	-1.10477596	-0.1451313
1445875_at	RIKEN cDNA A230057D06 gene	A230057D06Rik	0.78511	1.13888125	0.16814087
1419534_at	oxidized low density lipoprotein (lectin-like) receptor 1	Olr1	0.78513	1.08320889	0.11262417
1427582_at	fibroblast growth factor 6	Fgf6	0.78515	-1.10867183	-0.1564124
1457261_at	RIKEN cDNA A930025H08 gene	A930025H08Rik	0.78516	1.10744312	0.13825051
1430527_a_at	ring finger protein 167	Rnf167	0.78517	1.07140125	0.09870376
1420998_at	ets variant gene 5	Etv5	0.78519	-1.06630553	-0.0926263
1455735_at	adaptor-related protein complex AP-1, sigma 3	Ap1s3	0.7852	-1.08744713	-0.1216973
1447929_at	slingshot homolog 3 (Drosophila)	Ssh3	0.78522	1.10411492	0.1420919
1418582_at	core-binding factor, runt domain, alpha subunit 2, translocated to,	Cbfa2t3	0.78522	-1.11072004	-0.1571007
1417355_at	paternally expressed 3	Peg3	0.78522	-1.09896092	-0.1494532
1442341_at	kalirin, RhoGEF kinase	Kalrn	0.78522	1.09327573	0.12658053
1429155_at	RIKEN cDNA 4933411K20 gene	4933411K20Rik	0.78524	1.08140593	0.1121992
1452194_at	tubulin-specific chaperone d	Tbcd	0.78524	1.07103549	0.09743778
1452159_at	RIKEN cDNA 2310001A20 gene	2310001A20Rik	0.78525	1.08535485	0.1161147
1449772_at	TBC1 domain family, member 14	Tbc1d14	0.78526	-1.10218477	-0.1459195
1425711_a_at	thymoma viral proto-oncogene 1	Akt1	0.78527	1.11292862	0.14317805
1445871_at	expressed sequence AI315376	AI315376	0.78527	-1.10119914	-0.139778
1453621_at	THO complex 7 homolog (Drosophila)	Thoc7	0.78527	-1.08555442	-0.1265331
1424092_at	erythrocyte protein band 4.1	Epb4.1	0.78528	1.0869751	0.11654993
1416239_at	argininosuccinate synthetase 1	Ass1	0.78529	-1.07790398	-0.1155754
1432594_at	RIKEN cDNA 4933413I22 gene	4933413I22Rik	0.78529	1.11329161	0.14941
1441124_at	vezatin, adherens junctions transmembrane protein	Vezt	0.7853	1.10241688	0.14054507
1417982_at	insulin induced gene 2	Insig2	0.7853	1.08411331	0.11357027

1428729_at	KRIT1, ankyrin repeat containing	Krit1	0.7853	-1.11611496	-0.1775502
1437511_x_at	chloride channel CLIC-like 1	Clcc1	0.78531	1.09078917	0.1224703
1448227_at	growth factor receptor bound protein 7	Grb7	0.78531	1.07372925	0.10140142
1454454_at	ELAV (embryonic lethal, abnormal vision, Drosophila)-like 2 (Hu a	Elavl2	0.78531	-1.11118836	-0.1602344
1426053_a_at	xenotropic and polytropic retrovirus receptor 1	Xpr1	0.78532	1.18412297	0.20802272
1420455_at	glial cells missing homolog 2 (Drosophila)	Gcm2	0.78533	1.11956048	0.15698699
1460309_at	T-cell acute lymphocytic leukemia 2	Tal2	0.78534	1.12375419	0.15952701
1436054_at	KH and NYN domain containing	Khnyn	0.78534	1.11466818	0.14767428
1457657_at	---	---	0.78535	1.12375345	0.1595337
1443244_at	prostaglandin reductase 2	Ptgr2	0.78535	-1.10000726	-0.1399681
1417261_at	mbt domain containing 1	Mbtd1	0.78535	-1.09019754	-0.1382429
1417723_at	ubiquitin-conjugating enzyme E2, J1	Ube2j1	0.78535	1.10411271	0.13910013
1431187_s_at	discs, large homolog 5 (Drosophila)	Dlg5	0.78536	1.1381183	0.1676995
1455975_x_at	ring finger protein 114	Rnf114	0.78536	1.08887483	0.11698778
1450024_at	suppressor of fused homolog (Drosophila)	Sufu	0.78537	1.08522396	0.11648776
1429600_at	methylenetetrahydrofolate dehydrogenase (NADP+ dependent)	Mthfd2l	0.78538	1.10787353	0.14325172
1436402_at	deoxyhypusine hydroxylase/monooxygenase /// hypothetical pr	Dohh /// LOC100503793	0.78538	1.1087164	0.14572931
1441690_at	cadherin 8	Cdh8	0.7854	1.09074419	0.12250542
1422459_a_at	proteasome (prosome, macropain) 26S subunit, non-ATPase, 13	Psmd13	0.7854	1.07618376	0.10307078
1449843_at	ST8 alpha-N-acetyl-neuraminide alpha-2,8-sialyltransferase 2	St8sia2	0.7854	-1.09055117	-0.128112
1434732_x_at	translocase of outer mitochondrial membrane 7 homolog (yeast)	Tomm7	0.7854	-1.1047593	-0.1762331
1418076_at	suppression of tumorigenicity 14 (colon carcinoma)	St14	0.78541	1.08175148	0.11140066
1420100_s_at	DNA segment, Chr 13, ERATO Doi 787, expressed	D13Ertd787e	0.78542	-1.10886773	-0.1578951
1424829_at	RIKEN cDNA A830007P12 gene	A830007P12Rik	0.78543	1.08484886	0.11675711
1428260_at	atlastin GTPase 1	Atl1	0.78543	-1.11605225	-0.1736948
1416470_a_at	ribophorin I	Rpn1	0.78546	1.07525126	0.10120589
1431458_at	RIKEN cDNA 4921507L20 gene	4921507L20Rik	0.78546	-1.10318718	-0.1425938
1450624_at	betaine-homocysteine methyltransferase	Bhmt	0.78546	1.10378061	0.14187554
1433790_at	trophinin associated protein	Troap	0.7855	1.0815847	0.11235606
1425976_x_at	zinc finger protein 353	Zfp353	0.7855	-1.07427764	-0.1081853
1427173_a_at	mitochondrial ribosomal protein S33	Mrps33	0.7855	1.0756056	0.10275953
1427856_a_at	---	---	0.78552	-1.09148134	-0.1313984
1426586_at	solute carrier family 25 (mitochondrial carrier oxoglutarate carrier	Slc25a11	0.78555	1.07225572	0.1001127
1445547_at	---	---	0.7856	-1.09340698	-0.1298381
1456936_at	calcium binding protein 4	Cabp4	0.78561	1.13275876	0.16597715
1417365_a_at	calmodulin 1	Calm1	0.78561	-1.06909255	-0.0977687
1456746_a_at	CD99 antigen-like 2	Cd99l2	0.78562	1.11651111	0.15168522
1454663_at	eukaryotic translation initiation factor 5	Eif5	0.78562	-1.08258278	-0.1270135
1443254_at	---	---	0.78563	-1.10884177	-0.1570616
1431515_at	RIKEN cDNA 4932414N04 gene	4932414N04Rik	0.78565	1.10828974	0.14107062
1447363_s_at	budding uninhibited by benzimidazoles 1 homolog, beta (S. cerev	Bub1b	0.78567	1.0689371	0.09584767
1440580_at	EFR3 homolog B (S. cerevisiae)	Efr3b	0.78568	-1.1150897	-0.1689213
1416759_at	microtubule associated monoxygenase, calponin and LIM domair	Mical1	0.78568	1.09302281	0.12570287
1446095_at	antisense Igf2r RNA	Airn	0.78569	1.13894446	0.16806545
1453291_at	HMG box domain containing 4	Hmgxb4	0.78571	1.14430644	0.17729235
1455584_at	stromal cell derived factor 4	Sdf4	0.78571	1.12752835	0.15941228
1452377_at	myeloid/lymphoid or mixed-lineage leukemia 1	Mll1	0.78571	-1.11584292	-0.1913022
1459828_at	---	---	0.78572	1.13624486	0.16914013
1447828_x_at	endonuclease/exonuclease/phosphatase family domain containi	Eepd1	0.78572	-1.10975412	-0.1601864
1420275_at	---	---	0.78572	-1.11240956	-0.1681331
1457402_at	---	---	0.78576	-1.11111651	-0.1654964
1429154_at	solute carrier family 35, member F2	Slc35f2	0.78576	1.07321979	0.10175328
1445172_at	Ligatin	Lgtn	0.78578	1.10375762	0.14139037
1422906_at	ATP-binding cassette, sub-family G (WHITE), member 2	Abcg2	0.78582	-1.07349203	-0.1023216
1458729_at	---	---	0.78584	1.12979896	0.16140652
1442032_at	cDNA sequence BC030500	BC030500	0.78592	-1.10259319	-0.1436251
1451505_at	coiled-coil-helix-coiled-coil-helix domain containing 5	Chchd5	0.78603	1.10440234	0.13447137
1458716_at	dual specificity phosphatase 27 (putative)	Dusp27	0.78604	1.1163474	0.14339768
1456352_a_at	splicing factor 3b, subunit 2	Sf3b2	0.78634	1.07291457	0.1003182
1417308_at	pyruvate kinase, muscle	Pkm2	0.78638	1.03996049	0.05617708
1456615_a_at	bromodomain PHD finger transcription factor	Bptf	0.78639	-1.07537715	-0.1107485
1421181_at	neuronal pentraxin chromo domain /// neuronal pentraxin recept	Npcd /// Nptxr	0.78644	1.11321353	0.14859188
1419431_at	epiregulin	Ereg	0.78645	1.09060293	0.12417779
1415771_at	nucleolin	Ncl	0.78649	-1.03995475	-0.0570812
1452280_at	FERM, RhoGEF (Arhgef) and pleckstrin domain protein 1 (chondro	Farp1	0.7865	1.13655102	0.1666996
1443956_at	zinc finger protein 397	Zfp397	0.78652	1.09595907	0.12624962
1456093_at	zinc finger protein 536	Zfp536	0.78653	1.10940386	0.1457545
1446344_at	nyctalopin	Nyx	0.78655	-1.12016313	-0.1848609
1459643_at	---	---	0.78663	-1.10257129	-0.1450606
1457794_at	Wolf-Hirschhorn syndrome candidate 1-like 1 (human)	Whsc1l1	0.78671	1.10153236	0.13332063
1417810_a_at	potassium voltage gated channel, Shab-related subfamily, memb	Kcnb1 /// Pacsin2	0.78678	1.06432633	0.08990291
1441780_at	---	---	0.78681	-1.084135	-0.1181837
1439879_at	Zinc finger protein 568	Zfp568	0.78686	-1.11118054	-0.1641987
1454176_at	excision repaiross-complementing rodent repair deficiency, comp	Ercc8	0.78688	1.12108338	0.15346791
1428342_at	REST corepressor 3	Rcor3	0.78689	-1.10222527	-0.1415944
1450921_at	aprataxin	Aptx	0.78693	1.10223872	0.13344246
AFFX-BioDn-3_at	---	---	0.78695	1.09052861	0.1161592
1435003_at	phosphatidylinositol 4-kinase, catalytic, alpha polypeptide	Pi4ka	0.78721	1.08052723	0.11036881
1457077_at	---	---	0.78727	-1.11400613	-0.1714807
1455853_x_at	tetraspanin 31	Tspan31	0.7874	-1.12160336	-0.186721
1419024_at	protein tyrosine phosphatase 4a1	Ptp4a1	0.78744	-1.09344731	-0.1369173
1450419_at	Serine hydrolase-like	Serhl	0.78744	-1.11181412	-0.1652459
1423038_at	syntaxin 6	Stx6	0.78745	1.08944625	0.11881052
1428824_at	RIKEN cDNA 2310003C23 gene	2310003C23Rik	0.78747	1.07328636	0.10023592
1443178_at	ring finger protein 13	Rnf13	0.78749	1.12088761	0.15581644
1437666_x_at	ubiquitin C	Ubc	0.78752	1.06913571	0.09579049
1454247_a_at	glycoprotein A33 (transmembrane)	Gpa33	0.78753	-1.11118359	-0.1885846
1459255_at	---	---	0.78758	1.14612125	0.17866215
1416325_at	cysteine-rich secretory protein 1	Crisp1	0.78759	1.10415077	0.13574429
1416161_at	RAD21 homolog (S. pombe)	Rad21	0.78759	-1.08258808	-0.127202
1442843_at	RIKEN cDNA 4933411D12 gene	4933411D12Rik	0.78759	1.12678569	0.15879095
1460421_at	zinc finger protein 133	Zfp133	0.78759	-1.10577635	-0.1567231
1458004_at	paired related homeobox 1	Prrx1	0.7876	-1.09628978	-0.1395613
1459008_at	---	---	0.7876	-1.10203106	-0.1522176

1436797_a_at	surfeit gene 4	Surf4	0.7876	1.08052888	0.11067202
1432430_a_at	RIKEN cDNA 1700081L11 gene	1700081L11Rik	0.7876	1.08403905	0.11618824
1457335_at	hypothetical LOC100504734	LOC100504734	0.78761	-1.08571676	-0.1188245
1422999_at	mitogen-activated protein kinase kinase kinase 14	Map3k14	0.78764	1.11199357	0.1443311
1452413_at	RIKEN cDNA C230081A13 gene	C230081A13Rik	0.78764	1.09397914	0.12836018
1459020_at	adhesion molecule with Ig like domain 1	Amigo1	0.78765	-1.10902982	-0.1559397
1424263_at	allograft inflammatory factor 1-like	Aif1l	0.78766	1.09632764	0.12754425
1419959_s_at	cytoplasmic polyadenylated homeobox	Cphx	0.78766	-1.12781355	-0.2231983
1459443_at	---	---	0.78766	-1.10702096	-0.1507006
1455921_at	tetratricopeptide repeat domain 30A2	Ttc30a2	0.78766	1.10717791	0.14259027
1421613_at	H2A histone family, member Y3	H2afy3	0.78767	-1.108003	-0.160537
1440807_at	---	---	0.78769	1.07491877	0.10360072
1459397_at	ariadne ubiquitin-conjugating enzyme E2 binding protein homolog	Arih1	0.7877	-1.08884834	-0.1241407
1434420_x_at	translocase of outer mitochondrial membrane 22 homolog (yeast	Tomm22	0.78771	-1.08982031	-0.1410517
1441214_at	exophilin 5	Exph5	0.78772	-1.09601068	-0.135545
1459526_at	---	---	0.78772	-1.10135674	-0.1421987
1456585_x_at	RIKEN cDNA E130309D02 gene	E130309D02Rik	0.78772	1.08459517	0.11601249
1419565_a_at	zinc finger protein X-linked	Zfx	0.78772	-1.08827367	-0.1222292
1446285_at	---	---	0.78772	-1.10858634	-0.1517926
1418949_at	growth differentiation factor 15	Gdf15	0.78772	-1.09362888	-0.1368933
1453783_at	RIKEN cDNA 6330411E07 gene	6330411E07Rik	0.78773	-1.10579825	-0.161939
1433664_at	ubiquitin-conjugating enzyme E2Q (putative) 2	Ube2q2	0.78775	-1.07730576	-0.1077099
1437428_x_at	eukaryotic translation initiation factor 2B, subunit 2 beta	Eif2b2	0.78775	1.07678455	0.10551747
1421903_at	mediator complex subunit 29	Med29	0.78776	1.11724967	0.14601295
1447051_at	Ring finger protein 43	Rnf43	0.78777	1.13778728	0.16838918
1426057_a_at	Eph receptor A3	Epha3	0.78778	1.10128639	0.13184508
1416587_a_at	X-ray repair complementing defective repair in Chinese hamster c	Xrcc1	0.78779	1.0744171	0.10288956
1427374_at	mucin 3, intestinal	Muc3	0.7878	1.11975488	0.1543165
1445893_at	procollagen-lysine, 2-oxoglutarate 5-dioxygenase 1	Plod1	0.78781	1.11743135	0.15324705
1450912_at	membrane-spanning 4-domains, subfamily A, member 1	Ms4a1	0.78781	1.08601179	0.11597548
1440542_at	RIKEN cDNA 7420416P09 gene	7420416P09Rik	0.78783	1.10069977	0.13644444
1439386_x_at	methionine adenosyltransferase II, alpha	Mat2a	0.78784	-1.1144576	-0.18303
1416151_at	serine/arginine-rich splicing factor 3	Srsf3	0.78785	-1.06631336	-0.0929823
1425120_x_at	interferon, alpha-inducible protein 27 like 2B	Ifi27l2b	0.78785	-1.1094698	-0.1708012
1430077_at	serine/arginine-rich splicing factor 11	Srsf11	0.78789	-1.09208558	-0.1274308
1430976_a_at	mitochondrial ribosomal protein L9	Mrpl9	0.7879	1.07355113	0.10151726
1456695_x_at	anaphase-promoting complex subunit 5	Anapc5	0.78792	1.06161261	0.08560712
1460257_a_at	predicted gene 2382 /// 5, 10-methenyltetrahydrofolate syntheta	Gm2382 /// Mthfs	0.78793	1.12471103	0.15144001
1416619_at	RIKEN cDNA 4632428N05 gene	4632428N05Rik	0.78793	1.15320113	0.1842884
1457154_at	---	---	0.78794	1.15182701	0.18169423
1436409_at	cytochrome c oxidase, subunit VIIIa	Cox8a	0.78795	-1.09899523	-0.1386826
1425143_a_at	NADH dehydrogenase (ubiquinone) Fe-S protein 1	Ndufs1	0.78795	1.06882553	0.09473685
1455229_x_at	phosphatidylglycerophosphate synthase 1	Pgs1	0.78796	1.07239858	0.1006756
1460478_at	RIKEN cDNA 2200002J24 gene	2200002J24Rik	0.78796	-1.11242128	-0.1670261
1430485_at	transient receptor potential cation channel, subfamily C, member	Trpc2	0.78797	-1.11896316	-0.2040952
1451830_a_at	spectrin beta 2	Spnb2	0.78798	1.0979044	0.13227653
1443773_at	YLP motif containing 1	Ylpm1	0.78799	-1.10491377	-0.1482003
1447327_at	predicted gene 7969	Gm7969	0.788	-1.11122148	-0.1627942
1424881_at	tribbles homolog 1 (Drosophila)	Trib1	0.78801	-1.10738037	-0.1597651
1431199_at	gametogenetin binding protein 1 /// hypothetical LOC100504137	Ggnbp1 /// LOC10050413	0.78803	1.11153277	0.14733358
1431630_a_at	Kruppel-like factor 3 (basic)	Klf3	0.78804	1.07387754	0.10050731
1424038_a_at	RIKEN cDNA 2310044H10 gene	2310044H10Rik	0.78804	1.0766103	0.10549585
1432709_at	RIKEN cDNA 4930505O19 gene	4930505O19Rik	0.78805	1.09852117	0.13486921
1421965_s_at	Notch gene homolog 3 (Drosophila)	Notch3	0.78806	1.09496817	0.12307086
1436420_a_at	importin 4	Ipo4	0.78806	-1.0985838	-0.1588987
1448257_at	solute carrier family 29 (nucleoside transporters), member 2	Slc29a2	0.78806	1.11082443	0.14313164
1416157_at	vinculin	Vcl	0.78807	1.15886999	0.18208821
1422881_s_at	synaptophysin-like protein	Sypl	0.78807	-1.08041818	-0.1129044
1434917_at	cordon-bleu	Cobl	0.78808	-1.06816319	-0.097703
1454642_a_at	COMM domain containing 3	Commd3	0.78808	-1.09631145	-0.1485724
1420492_s_at	submaxillary gland androgen regulated protein 3A	Smr3a	0.78809	-1.11695394	-0.1753911
1457856_at	nuclear receptor subfamily 2, group E, member 3	Nr2e3	0.7881	1.09896752	0.13281673
1422549_at	ADP-ribosylation factor-like 2	Arl2	0.7881	1.08508995	0.11277837
1436585_at	expressed sequence BB182297	BB182297	0.7881	-1.07526599	-0.1066823
1421090_at	erythrocyte protein band 4.1-like 1	Epb4.1l1	0.7881	1.06553472	0.09007734
1450202_at	glutamate receptor, ionotropic, NMDA1 (zeta 1)	Grin1	0.78812	1.15378424	0.18322287
1448558_a_at	phospholipase A2, group IVA (cytosolic, calcium-dependent)	Pla2g4a	0.78813	1.09722768	0.13001317
1417231_at	claudin 2	Cldn2	0.78814	1.10695658	0.14178403
1441091_at	ELOVL family member 7, elongation of long chain fatty acids (yea	Elovl7	0.78815	1.12210389	0.15688627
1415890_at	3'-phosphoadenosine 5'-phosphosulfate synthase 1	Papss1	0.78817	1.09354923	0.12363813
1432830_at	coiled-coil domain containing 138	Ccdc138	0.78817	1.0896609	0.1233298
1452209_at	plakophilin 4	Pkp4	0.78817	-1.06877906	-0.096371
1432585_at	RIKEN cDNA 4931428A05 gene	4931428A05Rik	0.78818	1.16299093	0.18862956
1427997_at	NADH dehydrogenase (ubiquinone) 1 alpha subcomplex, assembl	Ndufaf4	0.78818	-1.07381217	-0.1033212
1420663_at	zinc finger and BTB domain containing 7B	Zbtb7b	0.78818	-1.08984333	-0.1252393
1448331_at	NADH dehydrogenase (ubiquinone) 1 beta subcomplex, 7	Ndufb7	0.78819	1.09797041	0.12360987
1452562_at	lysine (K)-specific demethylase 5D	Kdm5d	0.7882	-1.09087355	-0.1265395
1445532_at	myosin XVI	Myo16	0.7882	-1.09596847	-0.1344325
1454056_at	hypothetical LOC100504389	LOC100504389	0.78821	1.11239384	0.14627472
1445274_at	zinc finger protein 781	Zfp781	0.78821	1.09741359	0.13236048
1460278_a_at	PPPDE peptidase domain containing 2	Pppde2	0.78822	1.07820451	0.10715763
1429264_at	RIKEN cDNA C030044B11 gene	C030044B11Rik	0.78822	-1.09558	-0.1377433
1429258_at	RIKEN cDNA A030001D20 gene	A030001D20Rik	0.78823	-1.10558639	-0.150129
1434798_at	ATPase, H+ transporting, lysosomal V0 subunit D2	Atp6v0d2	0.78825	-1.09991839	-0.1424865
1439139_at	DNA segment, Chr 2, ERATO Doi 640, expressed	D2Ertd640e	0.78825	-1.10215007	-0.1456792
1428910_at	RIKEN cDNA 2310022B05 gene	2310022B05Rik	0.78827	1.08078234	0.11036071
1429239_a_at	StAR-related lipid transfer (START) domain containing 4	Stard4	0.78833	1.07767333	0.10722945
1448880_at	ubiquitin-conjugating enzyme E2L 3	Ube2l3	0.78845	1.08617877	0.11408587
1455056_at	LIM domain only 7	Lmo7	0.78855	-1.09076984	-0.1254043
1432072_at	kinesin family member 2A	Kif2a	0.78858	1.12657081	0.16024321
AFFX-r2-Bs-phe-M	---	---	0.78863	1.20287847	0.2203217
1423889_at	predicted gene 5617	Gm5617	0.78887	1.10919147	0.13830017
1450111_a_at	nudix (nucleoside diphosphate linked moiety X)-type motif 8	Nudt8	0.78907	1.09054744	0.11978227
1432239_at	RIKEN cDNA 1700102J08 gene	1700102J08Rik	0.78907	-1.10267641	-0.1445605

1424769_s_at	caldesmon 1	Cald1	0.78915	-1.08099955	-0.1160222
1434316_at	chondroitin sulfate synthase 1	Chsy1	0.78917	-1.07656716	-0.1132189
1454594_at	RIKEN cDNA 2900078I11 gene	2900078I11Rik	0.78918	-1.10940925	-0.1619224
1434644_at	transducin (beta)-like 1 X-linked	Tbl1x	0.78929	1.11034413	0.14051236
1423035_s_at	thioredoxin domain containing 17	Txndc17	0.78931	1.06943838	0.09504054
1428033_at	predicted gene 4979	Gm4979	0.78936	-1.08926442	-0.1236726
1430990_s_at	mitochondrial ribosomal protein L44	Mrpl44	0.78936	1.08065793	0.10722662
1438413_at	SUMO1/sentrin specific peptidase 7	Senp7	0.78939	1.10009929	0.13610734
1448333_at	ADP-ribosylarginine hydrolase	Adprh	0.78939	1.0660072	0.09099557
1460465_at	RIKEN cDNA A930038C07 gene	A930038C07Rik	0.78939	1.12022146	0.15740856
1427200_at	zinc finger, RAN-binding domain containing 1	Zranb1	0.7894	-1.09254373	-0.129167
1428411_at	---	---	0.78942	-1.08003045	-0.1120045
1437842_at	phosphatidylinositol-specific phospholipase C, X domain containin	Plcxd1	0.78942	1.15497149	0.18421144
1423264_at	block of proliferation 1	Bop1	0.78942	1.06671447	0.09258735
1441310_at	---	---	0.78944	-1.11815674	-0.1789107
1445098_at	expressed sequence AU022706	AU022706	0.78944	1.08944759	0.12245882
1437174_at	transcription factor Dp 2	Tfdp2	0.78944	-1.07375588	-0.1045937
1454704_at	scavenger receptor class B, member 2	Scarb2	0.78946	1.07522076	0.10287251
1428524_at	cysteinyl-tRNA synthetase 2 (mitochondrial)(putative)	Cars2	0.78948	1.08140181	0.11135677
1451326_at	abhydrolase domain containing 14b	Abhd14b	0.78952	1.09363466	0.12791371
1457669_x_at	replication factor C (activator 1) 2	Rfc2	0.78954	1.0872353	0.11785763
1417503_at	replication factor C (activator 1) 2	Rfc2	0.78955	1.07414821	0.1008271
1415752_at	cDNA sequence BC031181	BC031181	0.78955	1.06958436	0.09648731
1427533_at	ALS2 C-terminal like	Als2cl	0.78959	1.09387692	0.12680455
1419122_at	methyltransferase like 1	Mettl1	0.78962	1.17223929	0.1902839
1430609_at	MORN repeat containing 1	Morn1	0.78969	-1.1053507	-0.1498021
1418288_at	lipin 1	Lpin1	0.78974	-1.10048445	-0.1498631
1422660_at	RNA binding motif protein 3	Rbm3	0.78982	-1.0558536	-0.0800827
1416060_at	TBC1 domain family, member 15	Tbc1d15	0.7899	-1.07990456	-0.11649
1436324_at	START domain containing 9	Stard9	0.7899	-1.10547243	-0.1488226
1418360_at	ring finger protein 112	Rnf112	0.78991	-1.10950662	-0.1563498
1425267_a_at	platelet endothelial aggregation receptor 1	Pear1	0.78995	-1.09426979	-0.1302874
1430780_a_at	phosphomannomutase 1	Pmm1	0.78998	1.1141343	0.13888276
1445029_at	---	---	0.79004	-1.09382549	-0.1332842
1440550_at	---	---	0.79023	1.08430949	0.11325134
1449017_at	nuclear transport factor 2	Nutf2	0.7903	1.07255678	0.1003989
1423174_a_at	par-6 (partitioning defective 6) homolog beta (C. elegans)	Pard6b	0.79031	1.08642339	0.11758371
1439162_at	RIKEN cDNA 1700011J10 gene /// hypothetical LOC100503849	1700011J10Rik /// LOC10	0.79037	1.10135866	0.13851401
1438418_at	MU-2/AP1M2 domain containing, death-inducing	Mudeng	0.79037	-1.08474768	-0.1207547
1422115_a_at	FMS-like tyrosine kinase 3 ligand /// ribosomal protein L13A	Flt3l /// Rpl13a	0.79037	1.11565129	0.15289177
1420801_at	neuronal PAS domain protein 1	Npas1	0.79038	-1.10319803	-0.1478774
1416627_at	serine protease inhibitor, Kunitz type 1	Spint1	0.79044	1.07427117	0.09981634
1455823_at	Bardet-Biedl syndrome 4 (human)	Bbs4	0.79044	1.13391914	0.16387713
1421932_at	chromobox homolog 5 (Drosophila HP1a)	Cbx5	0.79044	-1.10567935	-0.1513617
1442946_at	---	---	0.7905	1.10717417	0.14126398
1459061_at	---	---	0.79052	1.10037146	0.1365087
1436836_x_at	calponin 3, acidic	Cnn3	0.79054	-1.06095442	-0.0879332
1452294_at	protocadherin 1	Pcdh1	0.79054	1.14694034	0.17800093
1423430_at	MYB binding protein (P160) 1a	Mybbp1a	0.79056	1.06361594	0.088825
1417559_at	sideroflexin 1	Sfxn1	0.79056	1.07889259	0.10520915
1420882_a_at	adrenocortical dysplasia	Acd	0.79057	1.07975297	0.10924049
1456380_x_at	calponin 3, acidic	Cnn3	0.79058	-1.06740991	-0.0952268
1418788_at	endothelial-specific receptor tyrosine kinase	Tek	0.79059	-1.09405403	-0.1356121
1459697_at	---	---	0.79059	-1.09221238	-0.1286959
1439814_at	ATPase, class I, type 8B, member 4	Atp8b4	0.7906	-1.09957872	-0.137391
1436792_at	WAS protein homolog associated with actin, golgi membranes an	Whamm	0.7906	1.11257358	0.14533681
1420692_at	interleukin 2 receptor, alpha chain	Il2ra	0.7906	1.10811575	0.14699992
1427364_a_at	ornithine decarboxylase, structural 1	Odc1	0.79061	1.06508115	0.09063145
1417277_at	cytochrome P450, family 4, subfamily f, polypeptide 16	Cyp4f16	0.79062	1.11278945	0.14543607
1447949_at	zinc finger protein 787	Zfp787	0.79062	1.09598741	0.12813831
1454286_at	RIKEN cDNA 1110004M10 gene	1110004M10Rik	0.79064	1.10131999	0.13709355
1431023_at	RIKEN cDNA C030046E11 gene	C030046E11Rik	0.79065	-1.11358611	-0.1733897
1444910_at	---	---	0.79066	-1.09737016	-0.1382847
1454860_x_at	defender against cell death 1	Dad1	0.79066	1.08161664	0.10966726
1427442_a_at	amyloid beta (A4) precursor protein	App	0.79067	-1.09842804	-0.1372989
1428727_at	centrosomal protein 192	Cep192	0.79068	-1.07126928	-0.1007221
1432954_at	RIKEN cDNA 4921520E09 gene	4921520E09Rik	0.79068	-1.09709603	-0.1346398
1455790_at	E2F transcription factor 2	E2f2	0.79069	1.10082721	0.13462668
1436074_at	nuclear factor of kappa light polypeptide gene enhancer in B-cells	Nfkbid	0.79073	1.08204028	0.11319149
1423572_at	BCL2-like 2	Bcl2l2	0.79074	1.12446909	0.15266487
1455927_x_at	Non-SMC element 1 homolog (S. cerevisiae)	Nsmce1	0.79077	1.07741786	0.10484609
1449667_at	---	---	0.79077	-1.10574803	-0.1536056
1431528_at	RIKEN cDNA 5830427D02 gene	5830427D02Rik	0.79078	1.12118327	0.15580069
1449660_s_at	coronin, actin binding protein 1C	Coro1c	0.79079	1.08164009	0.10868445
1455213_at	thymosin beta 15b like	Tmsb15l	0.79079	1.09477116	0.12411126
1433415_at	regulatory factor X, 2 (influences HLA class II expression)	Rfx2	0.79079	-1.10760614	-0.1562326
1451647_at	solute carrier family 24 (sodium/potassium/calcium exchanger), r	Slc24a1	0.7908	1.10662611	0.1412784
1430196_at	RIKEN cDNA 8430408J09 gene	8430408J09Rik	0.79081	-1.09887687	-0.1373946
1457408_at	---	---	0.79081	1.13183569	0.16594591
1457053_at	---	---	0.79083	-1.11335905	-0.1718061
1453124_at	transportin 3	Tnpo3	0.79083	-1.09746834	-0.159627
1460251_at	Fas (TNF receptor superfamily member 6)	Fas	0.79085	1.09794339	0.13394546
1423943_at	dihydrouridine synthase 1-like (S. cerevisiae)	Dus1l	0.79086	1.08376731	0.11037247
1424644_at	tubulin-specific chaperone C	Tbcc	0.79087	1.0782175	0.10784259
1420667_at	double C2, beta	Doc2b	0.79088	-1.10537669	-0.1446181
1419753_at	nuclear transcription factor, X-box binding 1	Nfx1	0.79089	1.09536941	0.12664597
1424023_at	cytosolic thiouridylase subunit 1 homolog (S. pombe)	Ctu1	0.79089	1.08747932	0.11829904
1428500_at	RIKEN cDNA 2210419D22 gene /// low density lipoprotein recepto	2210419D22Rik /// Lrp6	0.79089	-1.07697574	-0.109446
1428662_a_at	HOP homeobox	Hopx	0.7909	-1.08679716	-0.1290812
1458948_at	RIKEN cDNA D630013N20 gene	D630013N20Rik	0.79092	-1.10313725	-0.1483744
1420570_x_at	T-cell leukemia/lymphoma 1B, 3	Tcl1b3	0.79092	1.06744843	0.09379426
1429319_at	ras homolog gene family, member H	Rhoh	0.79093	-1.10481819	-0.1544129
1437343_x_at	ATPase family, AAA domain containing 3A	Atad3a	0.79093	1.0725749	0.09829556
1436659_at	doublecortin-like kinase 1	Dclk1	0.79093	1.0879169	0.12046906

1423150_at	secretogranin V	Scg5	0.79095	1.11236963	0.14527587
1458401_at	RIKEN cDNA 4932438A13 gene	4932438A13Rik	0.79097	-1.11256102	-0.1683443
1451121_a_at	glioma tumor suppressor candidate region gene 2	Gltscr2	0.79098	1.07818536	0.10512799
1457813_at	---	---	0.79099	1.11466482	0.15102111
1423300_at	zinc finger, DHHC domain containing 6	Zdhhc6	0.79099	-1.07674294	-0.1085845
1459802_at	---	---	0.79099	-1.11023906	-0.1657698
1433338_at	RIKEN cDNA 6720460K10 gene	6720460K10Rik	0.791	-1.09881607	-0.1361636
1443594_at	expressed sequence AI043046	AI043046	0.79101	-1.09741607	-0.1342146
1427268_at	filaggrin	Flg	0.79102	1.08136913	0.11025405
1439744_at	---	---	0.79105	-1.11071725	-0.1622564
1429595_at	RIKEN cDNA 2700049A03 gene	2700049A03Rik	0.79106	1.10000062	0.12849924
1450672_a_at	ATR interacting protein /// three prime repair exonuclease 1	Atrip /// Trex1	0.79107	1.10252732	0.13551071
1445689_at	---	---	0.79108	-1.10276347	-0.1423898
1424480_s_at	thymoma viral proto-oncogene 2	Akt2	0.79111	1.08534205	0.11617349
1427768_s_at	myosin, light polypeptide 3	Myl3	0.79112	1.10361141	0.14144537
1432695_at	RIKEN cDNA 4921527H02 gene	4921527H02Rik	0.79112	1.11984954	0.14818388
1432527_at	RIKEN cDNA 1700108M19 gene	1700108M19Rik	0.79113	-1.11047869	-0.1697451
1434977_at	RIKEN cDNA 4933403F05 gene	4933403F05Rik	0.79113	1.08954043	0.12194373
1429304_at	ankyrin repeat domain 10	Ankrd10	0.79113	-1.09021648	-0.1258499
1443939_at	predicted gene 12824	Gm12824	0.79116	1.11421583	0.15008033
1416733_at	muskelin 1, intracellular mediator containing kelch motifs	Mkln1	0.79116	1.08020002	0.10995445
1437548_at	bicaudal D homolog 1 (Drosophila)	Bicd1	0.79118	-1.11996912	-0.1795734
1431659_at	transmembrane protein 86B	Tmem86b	0.79119	-1.08819248	-0.1255659
1448350_at	argininosuccinate lyase	Asl	0.79138	1.08007253	0.109388
1423444_at	Rho-associated coiled-coil containing protein kinase 1	Rock1	0.79144	-1.09466288	-0.1459308
1432522_s_at	coiled-coil domain containing 46	Ccdc46	0.79146	1.1071595	0.14358433
1424133_at	transmembrane protein 98	Tmem98	0.79147	1.11314936	0.14344128
1436108_at	thioredoxin domain containing 9	Txndc9	0.79147	-1.10478666	-0.1486881
1454566_at	phosphatidylserine decarboxylase, pseudogene 1 /// phosphatidy	Pisd-ps1 /// Pisd-ps3	0.79147	1.10972486	0.13800772
1458175_at	spondin 2, extracellular matrix protein	Spon2	0.79149	1.10979042	0.14368528
1450852_s_at	coagulation factor II (thrombin) receptor	F2r	0.79149	-1.07625232	-0.1101647
1438290_x_at	Surfactant associated protein C	Sftpc	0.7915	1.102359	0.13780547
1451644_a_at	MHC class I like protein GS10 /// histocompatibility 2, Q region lo	H2-gs10 /// H2-Q6 /// LO	0.79152	1.09837733	0.12800952
1438497_at	major facilitator superfamily domain containing 8	Mfsd8	0.79155	-1.10630728	-0.1497302
1437570_at	expressed sequence AI503301	AI503301	0.79156	1.09216671	0.12604705
1449604_at	acid phosphatase 1, soluble	Acp1	0.79157	-1.1023726	-0.1457476
1428681_at	predicted gene 608	Gm608	0.79158	-1.08933431	-0.1278956
1456760_at	gastrulation brain homeobox 1	Gbx1	0.79158	1.16736011	0.19172268
1455698_at	SEC62 homolog (S. cerevisiae)	Sec62	0.79159	1.12027507	0.14848466
1459675_at	---	---	0.79161	1.08170173	0.11238138
1435103_x_at	phenylalanyl-tRNA synthetase, beta subunit	Farsb	0.79161	-1.07619704	-0.114706
1457859_at	---	---	0.79161	-1.09946981	-0.1407806
1426330_at	calcium channel, voltage-dependent, gamma subunit 5	Cacng5	0.79162	-1.09702707	-0.134327
1446172_at	---	---	0.79162	1.12399625	0.15966062
1428726_at	THUMP domain containing 2	Thumpd2	0.79162	-1.09633657	-0.1364146
1429968_at	JNK1/MAPK8-associated membrane protein	Jkamp	0.79162	-1.12048877	-0.1926535
1459038_at	---	---	0.79162	1.11653808	0.1500932
1423084_at	UDP-Gal:betaGlcNAc beta 1,3-galactosyltransferase, polypeptide	B3galt2	0.79162	-1.07571259	-0.107382
1417496_at	ceruloplasmin	Cp	0.79163	-1.11039292	-0.1664038
1447117_at	---	---	0.79163	-1.10189643	-0.1461549
1435983_at	hypothetical LOC100502684	LOC100502684	0.79163	-1.10322437	-0.1472076
1438449_at	CDC42 binding protein kinase alpha	Cdc42bpa	0.79165	-1.10134002	-0.1416718
1456187_at	solute carrier family 7 (cationic amino acid transporter, y+ system	Slc7a14	0.79165	1.10000186	0.13669516
1455904_at	growth arrest specific 5 /// small nucleolar RNA, C/D box 47	Gas5 /// Snord47	0.79166	-1.07787777	-0.1126672
1418141_at	doublecortin	Dcx	0.79167	1.10469522	0.14023813
1422767_at	bystin-like	Bysl	0.79167	1.06693829	0.09333162
1452475_at	proprotein convertase subtilisin/kexin type 5	Pcsk5	0.79167	-1.11268891	-0.1691267
1438697_at	transmembrane protein 132C	Tmem132c	0.79167	-1.10543193	-0.1500436
1441403_at	RIKEN cDNA 6430501K19 gene	6430501K19Rik	0.79168	1.10612312	0.14278334
1428226_at	neuroguidin, EIF4E binding protein	Ngdn	0.79168	1.06705635	0.09346565
1440634_at	---	---	0.79168	-1.1018574	-0.1473858
1437483_at	zinc finger protein 513	Zfp513	0.79169	1.09263592	0.12363469
1428171_at	PRP39 pre-mRNA processing factor 39 homolog (yeast)	Prpf39	0.79169	-1.0884034	-0.1275098
1417101_at	heat shock protein 2	Hspa2	0.79169	1.09988944	0.13432652
1416300_a_at	solute carrier family 25 (mitochondrial carrier, phosphate carrier),	Slc25a3	0.7917	1.04700496	0.06574829
1453637_at	RIKEN cDNA 5430410E06 gene	5430410E06Rik	0.7917	1.11800426	0.15357029
1452116_s_at	activating transcription factor 2	Atf2	0.79171	-1.06476709	-0.0906639
1432519_at	RIKEN cDNA 1810059H22 gene	1810059H22Rik	0.79172	1.12444889	0.15874034
1435134_at	arylacetamide deacetylase-like 1	Nceh1	0.79172	1.09358979	0.12857016
1431100_at	Ras and Rab interactor-like	Rinl	0.79172	-1.10309402	-0.1448586
1424522_at	HEAT repeat containing 1	Heatr1	0.79172	1.06622481	0.09212056
1447166_at	---	---	0.79173	1.1693605	0.19373989
1448825_at	pyruvate dehydrogenase kinase, isoenzyme 2	Pdk2	0.79173	1.14126338	0.17250373
1428391_at	RAB3A interacting protein (rabin3)-like 1	Rab3il1	0.79174	1.12697352	0.15793489
1421291_at	interleukin 18 receptor accessory protein	Il18rap	0.79175	-1.10675759	-0.1567249
1416323_at	potassium channel tetramerisation domain containing 20	Kctd20	0.79175	1.07621506	0.1058272
1459421_at	hexokinase domain containing 1	Hkdc1	0.79176	-1.10820886	-0.1547878
1442790_at	---	---	0.79176	-1.11508601	-0.1772019
1444419_at	prolylcarboxypeptidase (angiotensinase C)	Prcp	0.79176	1.11435951	0.14962953
1436187_at	RIKEN cDNA 1110054M08 gene	1110054M08Rik	0.79177	1.11712379	0.15205449
1430477_s_at	RIKEN cDNA 1700024N20Rik gene	1700024N20Rik	0.79177	-1.10865469	-0.1586235
1430105_at	RIKEN cDNA 1700042O10 gene	1700042O10Rik	0.79177	1.10521524	0.14098138
1446326_at	collagen, type I, alpha 2	Col1a2	0.79178	1.07953747	0.10786165
1448976_at	tuftelin interacting protein 11	Tfip11	0.79178	1.07594269	0.1051125
1418853_at	apolipoprotein N	Apon	0.79178	-1.10312731	-0.1485853
1457036_at	---	---	0.79179	1.15057755	0.18195224
1455813_at	predicted gene 13023	Gm13023	0.79179	1.07791864	0.10762294
1433494_at	downstream of Stk11	Dos	0.79179	1.09898607	0.13245641
1424419_at	leucine rich repeat containing 14	Lrrc14	0.79179	1.08952826	0.12006687
1459963_at	---	---	0.7918	-1.10280809	-0.146863
1459805_x_at	dihydrouridine synthase 3-like (S. cerevisiae)	Dus3l	0.79181	1.07390889	0.10092037
1419734_at	actin, beta	Actb	0.79181	-1.11987511	-0.2217682
1450155_at	integrin alpha 4	Itga4	0.79181	1.11798029	0.15284996
1421144_at	retinitis pigmentosa GTPase regulator interacting protein 1	Rpgrip1	0.79181	-1.10321009	-0.1546057

1447483_s_at	Small nucleolar RNA host gene (non-protein coding) 7	Snhg7	0.79181	-1.09693997	-0.1404404
1427206_at	AFG3(ATPase family gene 3)-like 2 (yeast)	Afg3l2	0.79181	1.09447818	0.12407904
1425873_a_at	leptin receptor	Lepr	0.79182	-1.1025813	-0.1416535
1423385_at	ARP8 actin-related protein 8 homolog (S. cerevisiae)	Actr8	0.79182	1.06577852	0.09134442
1435587_at	PCI domain containing 2	Pcid2	0.79182	-1.07604865	-0.1077535
1434139_at	poly (ADP-ribose) polymerase family, member 11	Parp11	0.79182	1.09420641	0.1257149
1457206_at	---	---	0.79182	-1.09351589	-0.1293527
1454216_at	predicted gene 14757	Gm14757	0.79183	-1.10779115	-0.1546987
1459440_at	---	---	0.79183	1.07195529	0.09721861
1433500_at	DENN/MADD domain containing 2A	Dennd2a	0.79184	1.11102131	0.14376355
1429256_at	maternally expressed 3	Meg3	0.79185	-1.09949902	-0.1384776
1457918_at	Tumor necrosis factor, alpha-induced protein 2	Tnfaip2	0.79185	-1.10492943	-0.1488197
1424086_at	OAF homolog (Drosophila)	Oaf	0.79185	1.08806231	0.12090192
1422959_s_at	ring finger protein 114	Rnf114	0.79186	1.06615765	0.0921591
1423656_x_at	RIKEN cDNA 1500010J02 gene	1500010J02Rik	0.79186	1.06490383	0.09053076
1424591_at	RIKEN cDNA 5830433M19 gene	5830433M19Rik	0.79186	-1.08067765	-0.1125136
1452248_at	pleckstrin homology domain containing, family G (with RhoGef do	Plekhg5	0.79187	1.09385928	0.12104802
1416264_at	ATP-binding cassette, sub-family B (MDR/TAP), member 9	Abcb9	0.79187	1.08985527	0.12073121
1457663_at	RIKEN cDNA 1700006F04 gene	1700006F04Rik	0.79187	1.09886524	0.12906423
1440757_at	---	---	0.79187	1.0856228	0.11840252
1454299_at	RIKEN cDNA 4833422B07 gene	4833422B07Rik	0.79187	-1.11114026	-0.1649669
1428873_a_at	male-specific lethal 1 homolog (Drosophila)	Msl1	0.79188	1.14141882	0.16731982
1452106_at	nephronectin	Npnt	0.79188	1.15193427	0.18152431
1419258_at	transcription elongation factor A (SII) 1	Tcea1	0.79188	-1.05804185	-0.0815183
1453132_a_at	gastrokine 2	Gkn2	0.79188	-1.09796286	-0.1380665
1425398_at	predicted gene 11277 /// predicted gene 13646	Gm11277 /// Gm13646	0.79188	1.11119294	0.14908129
1451813_at	opioid receptor, kappa 1	Oprk1	0.79188	-1.10571214	-0.15041
1424919_at	v-erb-b2 erythroblastic leukemia viral oncogene homolog 2, neur	Erbb2	0.79189	1.0864924	0.11949858
1420337_at	gastrulation brain homeobox 2	Gbx2	0.79189	-1.06853201	-0.0975066
1431391_at	Ral GEF with PH domain and SH3 binding motif 1	Ralgps1	0.7919	-1.10284339	-0.1456124
1446705_at	---	---	0.7919	1.13717349	0.16588516
1455646_at	RIKEN cDNA 2010004M13 gene	2010004M13Rik	0.7919	1.1267709	0.16074693
1430757_at	prune homolog 2 (Drosophila)	Prune2	0.79191	-1.09433555	-0.1328449
1419339_at	neuraminidase 3	Neu3	0.79193	1.1095864	0.14544059
1431935_at	lipoic acid synthetase	Lias	0.79193	-1.10183604	-0.1476928
1432925_at	RIKEN cDNA 4933416A02 gene	4933416A02Rik	0.79193	1.11571949	0.15201544
1453067_at	apoptosis-inducing, TAF9-like domain 1	Apitd1	0.79193	1.08715878	0.11558718
1459406_at	---	---	0.79194	1.10572072	0.14158227
1417284_at	mitogen-activated protein kinase associated protein 1	Mapkap1	0.79194	1.08258724	0.11088974
1415878_at	ribonucleotide reductase M1	Rrm1	0.79194	1.0764938	0.10293089
1449237_at	arachidonate lipoxygenase 3	Aloxe3	0.79194	1.09118371	0.12475208
1427074_at	protein-L-isoaspartate (D-aspartate) O-methyltransferase domair	Pcmtd2	0.79194	1.09050092	0.12149995
1433570_s_at	N(alpha)-acetyltransferase 35, NatC auxiliary subunit	Naa35	0.79195	-1.06900463	-0.0986012
1437870_at	solute carrier organic anion transporter family, member 4C1	Slco4c1	0.79195	-1.10907897	-0.1628912
1448219_a_at	tyrosine 3-monooxygenase/tryptophan 5-monooxygenase activa	Ywhaz	0.79196	1.06016873	0.08243258
1423067_at	CDK5 regulatory subunit associated protein 3	Cdk5rap3	0.79197	1.0734085	0.10013779
1442604_at	excision repair cross-complementing rodent repair deficiency, com	Ercc6	0.79197	-1.08460537	-0.117917
1457684_at	RIKEN cDNA 1700037H04 gene	1700037H04Rik	0.79198	-1.09973003	-0.1374176
1434074_x_at	ADP-ribosylation factor 4	Arf4	0.79198	-1.09468583	-0.1379571
1460108_at	---	---	0.79198	-1.09504654	-0.1328541
AFFX-PheX-3_at	---	---	0.792	1.11316675	0.14368963
1452105_a_at	tuberous sclerosis 2	Tsc2	0.79201	1.07135524	0.09910954
1422953_at	formyl peptide receptor 2	Fpr2	0.79201	1.11062998	0.14602189
1437197_at	sorbin and SH3 domain containing 2	Sorbs2	0.79202	-1.0907623	-0.1264262
1427365_at	keratin 86	Krt86	0.79202	-1.1037948	-0.1491436
1445349_at	---	---	0.79202	-1.09024618	-0.1268784
1453130_at	E1A binding protein p400	Ep400	0.79202	-1.09833986	-0.1384718
1460548_a_at	Era (G-protein)-like 1 (E. coli)	Eral1	0.79203	1.06511755	0.09058646
1418646_at	guanine nucleotide binding protein-like 1	Gnl1	0.79204	1.0755438	0.1031772
1418372_at	adenylosuccinate lyase	Adsl	0.79204	1.07063664	0.09591008
1426363_x_at	H2A histone family, member Y2	H2afy2	0.79204	-1.07891862	-0.1132993
1458045_at	---	---	0.79204	-1.10574222	-0.1529883
1450316_at	protocadherin beta 13	Pcdhb13	0.79204	-1.09219247	-0.1283973
1439693_a_at	F-box and WD-40 domain protein 16	Fbxw16	0.79204	-1.08428299	-0.1174623
1418552_at	opsin 1 (cone pigments), short-wave-sensitive (color blindness, tri	Opn1sw	0.79205	1.09995428	0.13161439
1436482_a_at	syndecan 3	Sdc3	0.79205	-1.10297391	-0.1456052
1439118_at	solute carrier family 35, member E4	Slc35e4	0.79206	1.12914352	0.16345368
1457134_at	cytoplasmic FMR1 interacting protein 1	Cyfip1	0.79206	1.12586318	0.16029765
1451994_s_at	glyoxylate reductase 1 homolog (Arabidopsis)	Glyr1	0.79206	1.06458693	0.0898977
1442450_at	DNA segment, Chr 2, ERATO Doi 501, expressed	D2Ertd501e	0.79206	1.08747357	0.11778556
1430966_at	camello-like 3	Cml3	0.79207	1.10119815	0.13810012
1448445_at	acid phosphatase 6, lysophosphatidic	Acp6	0.79207	1.06887602	0.0946003
1419478_at	secreted and transmembrane 1B	Sectm1b	0.79208	1.09605394	0.13057657
1455107_at	serine/arginine-rich protein specific kinase 1	Srpk1	0.79208	-1.08705909	-0.1342627
1419270_a_at	deoxyuridine triphosphatase	Dut	0.79209	1.07064875	0.09720686
1427377_x_at	hydroxy-delta-5-steroid dehydrogenase, 3 beta- and steroid delta	Hsd3b3	0.7921	-1.10049775	-0.1495888
1441371_at	plexin A4	Plxna4	0.7921	-1.08865783	-0.124166
1454276_at	RIKEN cDNA 1700021O21 gene	1700021O21Rik	0.7921	1.14491625	0.1747741
1441421_at	RIKEN cDNA C530030P08 gene	C530030P08Rik	0.7921	-1.09570724	-0.1339762
1431906_at	RIKEN cDNA 2700068H02 gene	2700068H02Rik	0.79212	1.10327944	0.13762796
1437360_at	protocadherin 19	Pcdh19	0.79212	1.10660229	0.14161709
1446802_at	ATX1 (antioxidant protein 1) homolog 1 (yeast)	Atox1	0.79212	1.15664151	0.18188551
1438004_at	PAP associated domain containing 7	Papd7	0.79213	1.07442783	0.10330871
1438821_at	---	---	0.79213	-1.1088688	-0.1597309
1419105_at	nuclear receptor subfamily 1, group H, member 4	Nr1h4	0.79213	1.13087736	0.16307114
1455688_at	discoidin domain receptor family, member 2	Ddr2	0.79213	-1.09823456	-0.1410223
1452593_a_at	transcription elongation factor B (SIII), polypeptide 1	Tceb1	0.79213	-1.10797233	-0.1728066
1456809_at	hypothetical LOC100503200	LOC100503200	0.79214	-1.11782297	-0.1806734
1457865_at	---	---	0.79215	1.14158251	0.17026484
1435481_at	zinc finger protein 653	Zfp653	0.79215	1.1037209	0.13701333
1429713_at	sulfatase modifying factor 2	Sumf2	0.79216	1.09502001	0.12407899
1418730_at	ring finger protein, LIM domain interacting	Rlim	0.79216	1.0835465	0.11553122
1444274_at	---	---	0.79216	1.10768021	0.14570372
1422914_at	trans-acting transcription factor 5	Sp5	0.79216	1.08881264	0.11973928

1422685_at	exocyst complex component 4	Exoc4	0.79217	-1.07708809	-0.1092262
1416091_at	microtubule-associated protein 4	Mtap4	0.79217	1.09418159	0.12308235
1425421_at	hypothetical LOC100134980 /// hypothetical protein LOC100504	LOC100134980 /// LOC10	0.79218	1.11748452	0.15330546
1437889_x_at	biglycan	Bgn	0.79218	1.10381822	0.14054653
1423892_at	amyloid beta (A4) precursor protein-binding, family B, member 1	Apbb1	0.79218	1.10893194	0.14137091
1440919_at	MOB1, Mps One Binder kinase activator-like 2C (yeast)	Mobkl2c	0.79218	1.09547271	0.13017089
1444929_at	---	---	0.7922	1.10402104	0.14132237
1447482_at	---	---	0.7922	1.1166418	0.15276503
1418595_at	perilipin 4	Plin4	0.79221	-1.1024167	-0.1462814
1451130_at	unconventional SNARE in the ER 1 homolog (S. cerevisiae)	Use1	0.79221	1.08353709	0.10992729
1416055_at	amylase 2a4 /// amylase 2a5	Amy2a4 /// Amy2a5	0.79222	1.09810281	0.12839261
1416709_a_at	neugrin, neurite outgrowth associated	Ngrn	0.79222	1.06990143	0.09608107
1445236_at	---	---	0.79222	1.0885891	0.12216616
1419805_s_at	geranylgeranyl diphosphate synthase 1	Ggps1	0.79223	-1.07977018	-0.110841
1436276_at	---	---	0.79223	1.10172683	0.13862517
1421463_at	sialic acid binding Ig-like lectin E	Siglece	0.79224	-1.09798071	-0.1404039
1433258_at	RIKEN cDNA A330102K18 gene	A330102K18Rik	0.79224	1.10791294	0.13572422
1421189_at	gamma-aminobutyric acid (GABA) A receptor, subunit beta 3	Gabrb3	0.79224	1.14472329	0.17776024
1433271_at	RIKEN cDNA 9530004M16 gene	9530004M16Rik	0.79224	-1.09351067	-0.1309337
1430792_at	---	---	0.79225	1.11455205	0.14839639
1419499_at	glycerol-3-phosphate acyltransferase, mitochondrial	Gpam	0.79225	1.08952995	0.11820722
1430277_at	RIKEN cDNA 9430034F23 gene	9430034F23Rik	0.79227	1.17911113	0.20202249
1448116_at	ubiquitin-like modifier activating enzyme 1	Uba1	0.79227	1.06507032	0.0904266
1455936_a_at	RNA binding protein gene with multiple splicing	Rbpms	0.79227	-1.07751852	-0.1123899
1447991_at	proprotein convertase subtilisin/kexin type 2	Pcsk2	0.79228	-1.08274981	-0.1198457
1451359_at	lysophosphatidylcholine acyltransferase 1	Lpcat1	0.79228	1.08829182	0.11871493
1425748_at	DIRAS family, GTP-binding RAS-like 1	Diras1	0.79228	-1.11175799	-0.1694856
1452574_x_at	Immunoglobulin heavy chain (J558 family)	Igh-VJ558	0.79228	1.07441927	0.10312699
1416651_at	zinc finger, HIT domain containing 2, pseudogene	Znhit2-ps	0.79229	1.07492222	0.10283348
1447431_at	2-deoxyribose-5-phosphate aldolase homolog (C. elegans)	Dera	0.79229	-1.11138563	-0.1701872
1439387_x_at	cytosolic thiouridylase subunit 2 homolog (S. pombe)	Ctu2	0.7923	-1.07211004	-0.1035896
1451250_at	G protein-regulated inducer of neurite outgrowth 1	Gprin1	0.7923	-1.09864832	-0.144094
1443885_at	---	---	0.7923	-1.10252817	-0.1412916
1447465_at	---	---	0.7923	1.10283144	0.13839752
1428269_a_at	glycosyltransferase 8 domain containing 1	Glt8d1	0.7923	-1.08440875	-0.1170589
1449702_at	zinc finger, AN1-type domain 2A	Zfand2a	0.79231	-1.10594636	-0.1599239
1457089_at	---	---	0.79232	-1.0978317	-0.1399425
1455658_at	CGG triplet repeat binding protein 1	Cggbp1	0.79233	-1.07134449	-0.1032777
1434045_at	cyclin-dependent kinase inhibitor 1B	Cdkn1b	0.79233	-1.07310249	-0.1026347
1436735_at	NOL1/NOP2/Sun domain family member 3	Nsun3	0.79234	-1.09229442	-0.129393
1432924_at	RIKEN cDNA 2810404I24 gene	2810404I24Rik	0.79234	1.11804924	0.15449813
1419023_x_at	enolase 1, alpha non-neuron /// predicted gene 5506 /// alpha-e	Eno1 /// Gm5506 /// LOC	0.79234	1.03301175	0.04653352
1434695_at	denticleless homolog (Drosophila)	Dtl	0.79234	1.07093537	0.0986025
1431212_a_at	tRNA methyltransferase 6 homolog (S. cerevisiae)	Trmt6	0.79236	1.0794391	0.10933467
1440016_at	---	---	0.79237	1.10208985	0.13732537
1451459_at	AT hook containing transcription factor 1	Ahctf1	0.79237	1.08203251	0.10958798
1442649_at	RIKEN cDNA 1500009L16 gene	1500009L16Rik	0.79238	-1.10499831	-0.1499398
1427634_at	potassium voltage-gated channel, subfamily H (eag-related), mer	Kcnh7	0.79238	-1.10438162	-0.1516186
1423859_a_at	prostaglandin D2 synthase (brain)	Ptgds	0.79239	-1.08503546	-0.1208078
1443395_at	DNA segment, Chr 6, ERATO Doi 490, expressed	D6Ertd490e	0.7924	-1.09867003	-0.1459102
1439329_a_at	BR serine/threonine kinase 2	Brsk2	0.79242	1.12933742	0.16545437
1424399_at	uridine-cytidine kinase 1	Uck1	0.79242	1.09206051	0.12002391
1446398_at	hypothetical LOC433197	LOC433197	0.79243	1.1178491	0.15292016
1454493_at	RIKEN cDNA 5033425B01 gene	5033425B01Rik	0.79244	1.10874797	0.14532452
1418732_s_at	WD repeat domain containing 83	Wdr83	0.79246	1.09338532	0.12505726
1415714_a_at	small nuclear ribonucleoprotein 27 (U4/U6.U5)	Snrnp27	0.79246	1.0654895	0.09136826
1420382_at	apolipoprotein B48 receptor	Apob48r	0.79247	1.10353229	0.1348164
1418983_at	InaD-like (Drosophila)	Inadl	0.79249	1.09348794	0.1262943
1443538_at	---	---	0.79249	1.10656405	0.13987131
1432409_at	protein kinase, cAMP dependent regulatory, type II alpha	Prkar2a	0.79253	-1.10123436	-0.1430784
1431473_at	RIKEN cDNA 5330423I11 gene	5330423I11Rik	0.79253	-1.08497731	-0.1178473
1444009_at	Ras association (RalGDS/AF-6) domain family member 4	Rassf4	0.79253	1.10216749	0.13845585
1424216_a_at	poly (A) polymerase alpha	Papola	0.79254	-1.066425	-0.0937365
1446447_at	---	---	0.79254	-1.10390293	-0.150216
1455289_at	ankyrin repeat domain 13b	Ankrd13b	0.79254	1.08707663	0.11890253
1438397_a_at	RNA binding motif protein 39	Rbm39	0.79263	-1.08802594	-0.1331455
1456903_at	pentraxin related gene	Ptx3	0.79275	-1.10325066	-0.1471046
1418677_at	actinin alpha 3	Actn3	0.79288	1.07008579	0.09709179
1433420_at	RIKEN cDNA 4930527J03 gene	4930527J03Rik	0.7929	-1.10266943	-0.1430115
1434853_x_at	makorin, ring finger protein, 1	Mkrn1	0.79291	-1.05349973	-0.0757973
1436312_at	IKAROS family zinc finger 1	Ikzf1	0.79291	1.12522675	0.1584788
1455654_at	Holliday junction recognition protein	Hjurp	0.79292	-1.10524449	-0.1546545
1435791_x_at	predicted pseudogene 10166 /// predicted pseudogene 10294 /	Gm10166 /// Gm10294 //	0.79292	-1.03949348	-0.0571797
1439157_at	butyrophilin-like 9	Btnl9	0.79295	1.08820969	0.11770229
1427371_at	ATP-binding cassette, sub-family A (ABC1), member 8a	Abca8a	0.79296	-1.09552266	-0.1322433
1427216_at	interferon zeta	Ifnz	0.79298	1.09919932	0.13509532
1445581_at	---	---	0.79299	-1.10137027	-0.1493
1458249_at	---	---	0.79305	-1.09751083	-0.1374342
1441914_x_at	Fibroblast growth factor 3	Fgf3	0.79313	1.13613973	0.16780138
1447001_at	expressed sequence C87114	C87114	0.7932	1.08627985	0.11817353
1451803_a_at	vascular endothelial growth factor B	Vegfb	0.79325	1.09882936	0.12714205
1417220_at	fumarylacetoacetate hydrolase	Fah	0.79328	1.08353793	0.11484576
1453012_at	TSC22 domain family, member 2	Tsc22d2	0.79329	-1.10787519	-0.1695613
1438940_x_at	high mobility group nucleosomal binding domain 1	Hmgn1	0.79329	-1.04891273	-0.0723872
1419253_at	methylenetetrahydrofolate dehydrogenase (NAD+ dependent), m	Mthfd2	0.79334	-1.0838157	-0.122086
1445700_at	proteasome (prosome, macropain) assembly chaperone 4	Psmg4	0.79334	1.09624563	0.12969581
1424432_at	ubiquitin domain containing 1	Ubtd1	0.79335	1.077809	0.10750322
1434770_at	IQ calmodulin-binding motif containing 1	Iqcb1	0.79335	1.08376231	0.11466614
1441499_at	glutamate receptor, ionotropic, delta 1	Grid1	0.79347	-1.09494966	-0.1323758
1447604_at	cDNA sequence BC053393	BC053393	0.79348	1.12487589	0.15472781
1433925_at	dynein, cytoplasmic 1 light intermediate chain 2	Dync1li2	0.79348	-1.08713489	-0.1254129
1454291_at	RIKEN cDNA 4933428P19 gene	4933428P19Rik	0.79351	1.14956	0.18122937
1448664_a_at	SPEG complex locus	Speg	0.79358	-1.1174436	-0.1871063
1446598_at	---	---	0.79362	1.12751355	0.16134777

1460369_at	cDNA sequence BC003267	BC003267	0.79369	-1.087838	-0.1284394
1433146_at	---	---	0.7937	1.11433415	0.14962425
1455696_a_at	PRP4 pre-mRNA processing factor 4 homolog B (yeast)	Prpf4b	0.79375	-1.08313382	-0.1243389
1423143_at	GTP binding protein 4	Gtpbp4	0.79377	-1.07305226	-0.1074941
1446448_at	protein inhibitor of activated STAT 1	Pias1	0.79385	1.10274419	0.14080989
1424597_at	open reading frame 19	ORF19	0.7939	1.09212412	0.12194301
1426629_at	DEAH (Asp-Glu-Ala-His) box polypeptide 8	Dhx8	0.79391	1.07054935	0.09728187
1449213_at	L antigen family, member 3	Lage3	0.79394	1.07419479	0.10005339
1447681_x_at	---	---	0.79397	-1.10014538	-0.1424976
1427190_at	adaptor protein, phosphotyrosine interaction, PH domain and leuc	Appl1	0.79403	-1.08156613	-0.1160693
1447187_at	---	---	0.79405	-1.1054342	-0.1511056
1419803_s_at	coiled-coil domain containing 12	Ccdc12	0.79405	1.07283748	0.0988152
1428992_at	unc-13 homolog D (C. elegans)	Unc13d	0.79408	1.10264287	0.13941804
1433901_at	cell cycle associated protein 1	Caprin1	0.7941	-1.06846982	-0.0968587
1435775_at	circadian locomotor output cycles kaput	Clock	0.79411	-1.11498817	-0.1809309
1428918_at	SCY1-like 3 (S. cerevisiae)	Scyl3	0.79413	-1.08484193	-0.1197376
1424064_at	RAB1B, member RAS oncogene family	Rab1b	0.79413	-1.06481004	-0.0909503
1444988_at	---	---	0.79413	1.08864813	0.12020069
1433254_at	RIKEN cDNA 5033423K11 gene	5033423K11Rik	0.79413	1.08042598	0.11103549
1434685_at	DNA segment, Chr 3, Brigham & Women's Genetics 0562 express	D3Bwg0562e	0.79415	1.06566229	0.09123824
1448650_a_at	polymerase (DNA directed), epsilon	Pole	0.79417	1.06613854	0.09155976
1452012_a_at	exosome component 1	Exosc1	0.79418	1.06780459	0.09412016
1445574_at	---	---	0.79418	-1.10069368	-0.1413637
1460320_at	beclin 1, autophagy related	Becn1	0.79419	1.07403018	0.10269077
1439895_at	expressed sequence AU021025	AU021025	0.7942	1.0953536	0.12783338
1417595_at	mesenchyme homeobox 1	Meox1	0.7942	1.09891684	0.13191693
1444380_at	expressed sequence AI844869	AI844869	0.7942	1.10010159	0.13627466
1440820_x_at	transmembrane and coiled-coil domains 2	Tmco2	0.79421	1.08854637	0.12026507
1438623_x_at	predicted gene 9840 /// ring-box 1	Gm9840 /// Rbx1	0.79421	-1.09575971	-0.1348511
1434915_s_at	leucine rich repeat containing 19	Lrrc19	0.79422	1.07867554	0.10661405
1429446_at	serologically defined colon cancer antigen 1	Sdccag1	0.79422	-1.07530062	-0.1074462
1444700_at	---	---	0.79423	-1.10801078	-0.1661764
1435509_x_at	CDK2 (cyclin-dependent kinase 2)-associated protein 1	Cdk2ap1	0.79423	-1.0609026	-0.0878675
1458280_at	---	---	0.79423	-1.10577424	-0.1521013
1434990_at	protein phosphatase 1E (PP2C domain containing)	Ppm1e	0.79423	1.13973983	0.16945551
1424839_a_at	NOL1/NOP2/Sun domain family, member 4	Nsun4	0.79423	1.08111299	0.1101715
1430032_at	RNA binding motif protein 4B	Rbm4b	0.79424	-1.10761314	-0.1573642
1428049_a_at	nudix (nucleoside diphosphate linked moiety X)-type motif 16-like	Nudt16l1	0.79424	1.08626294	0.11469015
1447157_at	RIKEN cDNA 4930480K23 gene	4930480K23Rik	0.79425	1.10336442	0.13999511
1460271_at	triggering receptor expressed on myeloid cells 3	Trem3	0.79425	-1.10115073	-0.1421972
1440328_at	nuclear autoantigenic sperm protein (histone-binding)	Nasp	0.79428	1.11219885	0.14762314
1425141_at	lactamase, beta 2	Lactb2	0.79429	-1.08031158	-0.1170277
1458802_at	human immunodeficiency virus type I enhancer binding protein 3	Hivep3	0.79429	1.10572678	0.13867655
1417051_at	protocadherin 8	Pcdh8	0.7943	1.12018831	0.15531723
1450489_at	sal-like 1 (Drosophila)	Sall1	0.79431	-1.09182874	-0.145753
1446298_at	RIKEN cDNA C630016I17 gene	C630016I17Rik	0.79433	-1.10139537	-0.148186
1442000_at	major facilitator superfamily domain containing 2B	Mfsd2b	0.79434	1.09991597	0.13443455
1416015_s_at	ATP-binding cassette, sub-family E (OABP), member 1	Abce1	0.79434	-1.06569512	-0.0928797
1436810_x_at	RIKEN cDNA 2900010M23 gene	2900010M23Rik	0.79434	1.08282637	0.11163537
1422286_a_at	TGFB-induced factor homeobox 1	Tgif1	0.79435	1.07403303	0.09935907
1430409_at	N-ethylmaleimide sensitive fusion protein	Nsf	0.79438	-1.09518678	-0.140653
1421979_at	phosphate regulating gene with homologies to endopeptidases o	Phex	0.79438	-1.08063932	-0.1155028
1417039_a_at	cullin 7	Cul7	0.79438	1.07070948	0.09721489
1428463_a_at	protein phosphatase 2, regulatory subunit B (B56), epsilon isoform	Ppp2r5e	0.79439	-1.07620746	-0.1080687
1441721_at	---	---	0.7944	-1.1038509	-0.1476005
1460227_at	tissue inhibitor of metalloproteinase 1	Timp1	0.79441	1.06910723	0.09491588
1422988_at	N-sulfoglucosamine sulfohydrolase (sulfamidase)	Sgsh	0.79441	-1.09727554	-0.1355067
1417230_at	Ral GEF with PH domain and SH3 binding motif 2	Ralgps2	0.79441	1.08696066	0.11993687
1431881_at	RIKEN cDNA 2410024F20 gene	2410024F20Rik	0.79441	1.1347006	0.15983019
1443243_at	---	---	0.79441	-1.09514193	-0.1370311
1422684_a_at	exocyst complex component 4	Exoc4	0.79442	-1.07731999	-0.1169852
1445180_at	Steroid 5 alpha-reductase 3	Srd5a3	0.79444	1.1131786	0.14808531
1421420_at	chemokine (C-C motif) receptor 10	Ccr10	0.79445	-1.09302848	-0.1328516
1420867_at	transmembrane emp24 domain trafficking protein 2	Tmed2	0.79446	1.07460988	0.10027608
1416451_s_at	TAF8 RNA polymerase II, TATA box binding protein (TBP)-associat	Taf8	0.79446	1.07552881	0.10484239
1419601_at	potassium inwardly-rectifying channel, subfamily J, member 10	Kcnj10	0.79446	-1.10368021	-0.1511304
1434234_at	zinc finger protein 341	Zfp341	0.79449	1.10545131	0.13811075
1454106_a_at	CXXC finger 1 (PHD domain)	Cxxc1	0.7945	1.10666228	0.13742386
1451808_at	potassium inwardly-rectifying channel, subfamily J, member 4	Kcnj4	0.7945	1.09571489	0.13077935
1441134_at	zinc finger protein 518B	Zfp518b	0.79453	-1.09231626	-0.131783
1439037_at	DEAD (Asp-Glu-Ala-Asp) box polypeptide 17	Ddx17	0.79457	-1.08927365	-0.1236135
1442274_at	zinc finger, DHHC domain containing 15	Zdhhc15	0.79458	-1.1039734	-0.1552046
1459751_s_at	protein phosphatase 1, regulatory (inhibitor) subunit 16A	Ppp1r16a	0.79458	1.07237784	0.09976628
1434669_at	Ral GEF with PH domain and SH3 binding motif 1	Ralgps1	0.7946	1.10518968	0.13788724
1418077_at	tripartite motif-containing 21	Trim21	0.79461	1.08808035	0.12080331
1427678_at	zinc finger, imprinted 3	Zim3	0.79462	-1.10169714	-0.1439698
1447183_at	---	---	0.79462	1.13546815	0.1693214
1452881_at	GINS complex subunit 2 (Psf2 homolog)	Gins2	0.79463	1.07070269	0.09687773
1424861_at	RIKEN cDNA D930016D06 gene	D930016D06Rik	0.79464	1.08456949	0.11564131
1429337_at	transmembrane protein 87B	Tmem87b	0.79464	1.10357632	0.1375897
1434171_at	zinc finger protein 874a	Zfp874a	0.79465	-1.10301008	-0.1468454
1441693_at	a disintegrin-like and metallopeptidase (reprolysin type) with thro	Adamts3	0.79465	-1.09545091	-0.1349534
1428859_at	polyamine oxidase (exo-N4-amino)	Paox	0.79465	1.082457	0.1099517
1443370_at	---	---	0.79466	1.07288981	0.09773364
1428742_at	F-box protein 45	Fbxo45	0.79469	1.08186735	0.1133819
1444109_at	RIKEN cDNA C130009A20 gene	C130009A20Rik	0.7947	-1.10927469	-0.165945
1452101_at	bleomycin hydrolase	Blmh	0.79471	1.07128731	0.09791999
1450640_x_at	ATP synthase, H+ transporting, mitochondrial F1F0 complex, subu	Atp5k	0.79473	1.0797284	0.10336149
1440360_at	---	---	0.79476	-1.08926837	-0.1357313
1431827_a_at	tousled-like kinase 2 (Arabidopsis)	Tlk2	0.79478	-1.0810922	-0.1192027
1451311_a_at	adiponectin receptor 1	Adipor1	0.79484	1.0699922	0.09726312
1423482_at	uroporphyrinogen III synthase	Uros	0.79487	1.08245094	0.11182902
1419443_at	Sin3-associated polypeptide 18	Sap18	0.79488	1.07045707	0.09714334
1448436_a_at	interferon regulatory factor 1	Irf1	0.7949	1.07056641	0.09818772

1438787_at	zinc finger and SCAN domain containing 10	Zscan10	0.79493	1.0674591	0.0921783
1453554_a_at	WD repeat domain 33	Wdr33	0.79493	-1.06620704	-0.0930888
1456203_at	RIKEN cDNA 1110020A10 gene	1110020A10Rik	0.79493	1.11435113	0.14611048
1444977_at	---	---	0.79494	-1.11342882	-0.1751328
1424276_at	sorting nexin 16	Snx16	0.79495	1.07545863	0.10481024
1422457_s_at	SMT3 suppressor of mif two 3 homolog 3 (yeast)	Sumo3	0.79497	1.05662442	0.07813528
1416076_at	cyclin B1	Ccnb1	0.79499	1.06443635	0.0894392
1435124_at	von Willebrand factor A domain containing 5B2	Vwa5b2	0.79502	-1.10984235	-0.1598
1422820_at	lipase, hormone sensitive	Lipe	0.79502	1.09553845	0.12604
1453207_at	RIKEN cDNA 2900053A13 gene /// hypothetical protein LOC1005	2900053A13Rik /// LOC1(	0.79504	-1.08159242	-0.1180829
1440257_at	oocyte specific homeobox 6	Obox6	0.79504	1.11298724	0.14863861
1446368_at	RIKEN cDNA 9130221J18 gene	9130221J18Rik	0.79505	-1.09259874	-0.1381507
1423737_at	NADH dehydrogenase (ubiquinone) Fe-S protein 3	Ndufs3	0.79508	1.0695355	0.094425
1432132_at	RIKEN cDNA 4933401D09 gene	4933401D09Rik	0.79509	1.13170739	0.16726013
1442860_at	diacylglycerol kinase, beta	Dgkb	0.7951	-1.06433232	-0.0917469
1446149_at	protein phosphatase 3, catalytic subunit, beta isoform	Ppp3cb	0.79512	1.10800161	0.13796258
1435693_at	mal, T-cell differentiation protein-like	Mall	0.79518	1.10612774	0.14173142
1451500_at	Usher syndrome 1C binding protein 1	Ushbp1	0.79519	1.12039588	0.15466986
1458919_at	muskelin 1, intracellular mediator containing kelch motifs	Mkln1	0.79522	1.11951899	0.15548899
1454098_at	olfactory receptor 112	Olfr112	0.79522	-1.09323233	-0.1290708
1423818_a_at	ADP-ribosylation factor-like 6 interacting protein 1	Arl6ip1	0.79524	1.07248747	0.09566011
1457456_at	mitogen-activated protein kinase kinase kinase 10	Map3k10	0.79527	-1.09786062	-0.1442747
1436550_at	F-box protein 30	Fbxo30	0.79527	1.09600912	0.12608903
1421873_s_at	RAB24, member RAS oncogene family	Rab24	0.79527	1.09345903	0.12265749
1426138_a_at	ubiquitin-conjugating enzyme E2, J2 homolog (yeast)	Ube2j2	0.79527	1.06239431	0.08727781
1418866_at	cytochrome P450, family 24, subfamily a, polypeptide 1	Cyp24a1	0.79527	1.17092239	0.19239911
1421688_a_at	chemokine (C-C motif) ligand 1	Ccl1	0.79531	1.10707285	0.14406895
1426461_at	UDP-glucose pyrophosphorylase 2	Ugp2	0.79532	1.09610223	0.13029905
1447507_at	expressed sequence C80406	C80406	0.79532	-1.10893833	-0.1654472
1420633_a_at	casein alpha s2-like A	Csn1s2a	0.79534	-1.09329688	-0.1296011
1454004_at	phosphotyrosine interaction domain containing 1	Pid1	0.79535	-1.10037149	-0.1394784
1459238_at	---	---	0.79536	-1.09835519	-0.1396173
1436966_at	pellino 2	Peli2	0.79537	-1.10672577	-0.1528142
1452530_a_at	runt related transcription factor 1	Runx1	0.79537	1.11576206	0.15046433
1415846_a_at	lactate dehydrogenase C	Ldhc	0.79537	1.10295837	0.13747601
1452171_at	glutamate-rich WD repeat containing 1	Grwd1	0.79539	1.07018816	0.09549078
1420519_a_at	Era (G-protein)-like 1 (E. coli)	Eral1	0.79541	1.08367451	0.11431301
1458837_at	RIKEN cDNA 2810006K23 gene	2810006K23Rik	0.79543	-1.09676227	-0.1351839
1441746_at	---	---	0.79543	-1.06581609	-0.0957433
1433308_at	RIKEN cDNA 9330159N22 gene	9330159N22Rik	0.79544	1.14114549	0.17024619
1429431_at	IKAROS family zinc finger 5	Ikzf5	0.79544	-1.09967953	-0.1627198
1431309_at	tubulin tyrosine ligase-like family, member 10	Ttll10	0.79548	1.07688523	0.10515638
1451237_s_at	RNA binding motif protein 7	Rbm7	0.79548	-1.07232008	-0.1061892
1416201_at	v-crk sarcoma virus CT10 oncogene homolog (avian)	Crk	0.7955	1.10329465	0.12807559
1435934_at	---	---	0.7955	-1.10314935	-0.1476805
1458850_at	---	---	0.7955	1.10296957	0.13890719
1417292_at	interferon gamma inducible protein 47	Ifi47	0.79551	1.10651017	0.14521375
1432376_at	RIKEN cDNA 3830403N18 gene	3830403N18Rik	0.79554	1.11025324	0.13763742
1416294_at	secretory carrier membrane protein 3	Scamp3	0.79554	1.07450525	0.10324304
1438275_at	RIKEN cDNA C130046K22 gene	C130046K22Rik	0.79555	1.12476507	0.15884869
1449264_at	synaptotagmin XI	Syt11	0.79557	1.12646781	0.15959331
1449253_at	structural maintenance of chromosomes 1B	Smc1b	0.79558	1.1074855	0.13948596
1446972_at	DNA segment, Chr 15, Wayne State University 126, expressed	D15Wsu126e	0.79558	-1.09113107	-0.130955
1430580_at	RIKEN cDNA 2810030E01 gene	2810030E01Rik	0.7956	1.10712755	0.1356179
1418509_at	carbonyl reductase 2	Cbr2	0.7956	1.10209886	0.13850674
1422831_at	fibrillin 2	Fbn2	0.7956	1.10228722	0.13572094
1425343_at	haloacid dehalogenase-like hydrolase domain containing 3	Hdhd3	0.79561	1.11639411	0.15059998
1458489_at	hypothetical LOC100503500	LOC100503500	0.79563	1.14478777	0.17462433
1455818_at	RIKEN cDNA 4930427A07 gene	4930427A07Rik	0.79563	1.08850148	0.11677749
1425134_a_at	phosphatidylinositol glycan anchor biosynthesis, class X	Pigx	0.79564	1.06493912	0.09036365
1432782_at	RIKEN cDNA 4933435C09 gene	4933435C09Rik	0.79565	-1.09537303	-0.1347072
1428097_at	RIKEN cDNA 2510009E07 gene	2510009E07Rik	0.79566	-1.10776334	-0.1645706
1426060_at	---	---	0.79566	1.1255438	0.15315144
1435420_at	solute carrier family 4, sodium bicarbonate cotransporter, membe	Slc4a5	0.79567	1.09642677	0.13203297
1439322_at	---	---	0.79568	1.07220689	0.09724008
1443918_at	RIKEN cDNA 2700050L05 gene	2700050L05Rik	0.79568	1.08161718	0.11318637
1449760_at	---	---	0.79569	-1.09744521	-0.1362985
1429813_at	pantothenate kinase 1	Pank1	0.79569	-1.09706887	-0.1345986
1460720_at	transient receptor potential cation channel, subfamily C, member	Trpc4ap	0.7957	1.06828479	0.09402688
1420195_at	---	---	0.79571	1.08730292	0.11739906
1442500_at	expressed sequence C77406	C77406	0.79571	-1.08382181	-0.1197686
1445018_at	---	---	0.79572	-1.1032415	-0.1504046
1439215_at	ATPase, Ca++-sequestering	Atp2c1	0.79572	-1.08368417	-0.1211209
1454857_at	ring finger protein 122	Rnf122	0.79573	1.12027906	0.15309557
1435211_at	tetratricopeptide repeat domain 12	Ttc12	0.79574	1.08959734	0.12301261
1449983_a_at	NAD(P)H dehydrogenase, quinone 2	Nqo2	0.79574	1.09140355	0.12171662
1420194_at	---	---	0.79574	1.1027263	0.13686276
1450938_at	pinin	Pnn	0.79575	-1.07041879	-0.0990328
1426427_at	tubulin tyrosine ligase-like 1	Ttll1	0.79576	1.08068663	0.10898865
1456514_at	---	---	0.79578	1.12621896	0.15936238
1451470_s_at	eukaryotic translation initiation factor 5A	Eif5a	0.79578	-1.0336354	-0.0481509
1452329_at	pleckstrin homology domain containing, family N member 1	Plekhn1	0.79578	-1.09023623	-0.127397
1427199_at	furry homolog-like (Drosophila)	Fryl	0.7958	-1.10258125	-0.1566379
1415957_a_at	ribosomal RNA processing 1 homolog (S. cerevisiae)	Rrp1	0.79581	1.09604259	0.12369605
1451574_at	B-cell CLL/lymphoma 9	Bcl9	0.79582	-1.08320208	-0.1184554
1443119_at	glutamate receptor, metabotropic 7	Grm7	0.79584	-1.08383312	-0.1168462
1424116_x_at	protein phosphatase 5, catalytic subunit	Ppp5c	0.79585	1.06882581	0.09469902
1439591_at	Unc-51 like kinase 2 (C. elegans)	Ulk2	0.7959	1.09161156	0.12570227
1445396_at	---	---	0.7959	-1.08459845	-0.1198031
1433387_at	RIKEN cDNA 2900022M07 gene	2900022M07Rik	0.79597	1.09269094	0.12486208
1457231_at	---	---	0.79597	1.08503355	0.11624914
1460568_at	tripartite motif-containing 46	Trim46	0.79624	1.12239704	0.15386569
1418784_at	glutamate receptor, ionotropic, kainate 5 (gamma 2)	Grik5	0.79671	1.12483568	0.15427717
1441397_at	---	---	0.79673	1.16770433	0.19131211

1420964_at	ectodermal-neural cortex 1	Enc1	0.79674	-1.09407787	-0.136698
1446471_at	---	---	0.79675	-1.09757885	-0.168246
1427735_a_at	actin, alpha 1, skeletal muscle	Acta1	0.79677	-1.09544532	-0.1326234
1416721_s_at	serine/arginine-rich splicing factor 6	Srsf6	0.79679	1.07643585	0.10252736
1442275_at	RIKEN cDNA 4933402J07 gene	4933402J07Rik	0.7968	1.108411	0.14440565
1444079_at	---	---	0.7968	-1.09429966	-0.1300502
1449961_at	rabphilin 3A	Rph3a	0.79681	-1.10789863	-0.1499103
1420222_at	---	---	0.79682	1.13930686	0.16911243
1433980_at	K(lysine) acetyltransferase 5	Kat5	0.79683	1.08266015	0.10832003
1418713_at	pterin 4 alpha carbinolamine dehydratase/dimerization cofactor d	Pcbd1	0.79683	1.1107629	0.14147995
1442248_at	---	---	0.79683	1.14804677	0.17667043
1453440_at	RIKEN cDNA 4921539E11 gene	4921539E11Rik	0.79684	1.09830582	0.13220227
1447150_at	MYC binding protein 2	Mycbp2	0.79684	1.12198375	0.15405924
1449130_at	CD1d1 antigen	Cd1d1	0.79684	-1.08673853	-0.1203971
1453073_at	RIKEN cDNA 5830403F22 gene	5830403F22Rik	0.79684	1.09181457	0.12544577
1432481_a_at	lysozyme-like 6	Lyzl6	0.79684	1.08420633	0.11595627
1424189_at	phosphatidylinositol glycan anchor biosynthesis, class C	Pigc	0.79686	1.09574884	0.12879852
1457278_at	RIKEN cDNA 6720489N17 gene	6720489N17Rik	0.79688	-1.09395979	-0.1298248
1416628_at	transmembrane protein 223	Tmem223	0.79688	1.08349603	0.10909703
1427913_at	RWD domain containing 1	Rwdd1	0.79689	-1.07647883	-0.1153797
1446096_at	RIKEN cDNA 2310001H17 gene	2310001H17Rik	0.79689	-1.10216054	-0.1447768
1456345_at	RIKEN cDNA A630057N01 gene	A630057N01Rik	0.79689	1.08985458	0.12322086
1451001_at	RIKEN cDNA 1700019N19 gene	1700019N19Rik	0.79694	-1.10807559	-0.1602026
1423706_a_at	phosphogluconate dehydrogenase	Pgd	0.79695	1.07469257	0.10002531
1459450_at	---	---	0.79696	-1.10002815	-0.146339
1424894_at	RAB13, member RAS oncogene family	Rab13	0.79696	1.08503317	0.11738747
1419358_at	sortilin-related VPS10 domain containing receptor 2	Sorcs2	0.79697	1.09276779	0.12639334
1415900_a_at	kit oncogene	Kit	0.79698	1.13777944	0.16321527
1418238_at	isovaleryl coenzyme A dehydrogenase	Ivd	0.79699	1.08649461	0.11558738
1441719_at	predicted gene 12689	Gm12689	0.79699	-1.09173161	-0.1278095
1434366_x_at	complement component 1, q subcomponent, beta polypeptide	C1qb	0.797	1.08277395	0.11449298
1446029_at	---	---	0.79707	1.10264275	0.13958348
1417083_at	Sec61 beta subunit	Sec61b	0.79711	1.07308747	0.09606116
1434957_at	cell adhesion molecule-related/down-regulated by oncogenes	Cdon	0.79731	1.08201191	0.11268152
1434592_at	solute carrier family 16 (monocarboxylic acid transporters), memb	Slc16a10	0.79747	1.10574552	0.14105053
1425330_a_at	protein phosphatase 1B, magnesium dependent, beta isoform	Ppm1b	0.79748	-1.07842002	-0.1166463
1441149_at	---	---	0.79754	1.10327707	0.13648125
1430319_at	RIKEN cDNA 4833411C07 gene	4833411C07Rik	0.79756	-1.09872109	-0.1374292
1441141_at	Antagonist of mitotic exit network 1 homolog (S. cerevisiae)	Amn1	0.79756	-1.1013948	-0.1398645
1420393_at	nitric oxide synthase 2, inducible	Nos2	0.79764	-1.10143744	-0.1459043
1437356_at	G protein-coupled receptor 183	Gpr183	0.79771	1.09872772	0.13512584
1421841_at	fibroblast growth factor receptor 3	Fgfr3	0.79782	1.09677479	0.12923853
1447522_s_at	tankyrase, TRF1-interacting ankyrin-related ADP-ribose polymera	Tnks2	0.79784	-1.07299787	-0.1051503
1450530_at	UDP-Gal:betaGlcNAc beta 1,3-galactosyltransferase, polypeptide	B3galt1	0.79784	-1.08656726	-0.1200091
1447301_at	A kinase (PRKA) anchor protein 5	Akap5	0.79788	1.09751429	0.12928832
1420312_s_at	---	---	0.7979	-1.09742387	-0.1411666
1453272_at	RIKEN cDNA 4930579F01 gene	4930579F01Rik	0.7979	1.10383113	0.13852456
1423459_at	COP9 (constitutive photomorphogenic) homolog, subunit 2 (Arabi	Cops2	0.79791	1.06990554	0.09524908
1417709_at	cytochrome P450, family 46, subfamily a, polypeptide 1	Cyp46a1	0.79791	-1.10110656	-0.1409366
1427110_at	ribonucleoprotein, PTB-binding 1	Raver1	0.79791	1.0771361	0.1037303
1437138_at	forkhead box M1	Foxm1	0.79794	1.10744417	0.13612082
1451094_at	gamma-glutamyltransferase 7	Ggt7	0.79795	-1.09885007	-0.1408855
1423965_at	CD99 antigen-like 2	Cd99l2	0.79795	1.09059645	0.12177957
1435118_at	tRNA splicing endonuclease 2 homolog (S. cerevisiae)	Tsen2	0.79795	1.08970811	0.11851663
1446352_at	---	---	0.79795	-1.08548644	-0.119906
1436847_s_at	cell division cycle associated 8	Cdca8	0.79796	-1.06472832	-0.0927869
1442476_at	intraflagellar transport 81 homolog (Chlamydomonas)	Ift81	0.79797	1.09779433	0.13445464
1448897_at	makorin, ring finger protein, 2	Mkrn2	0.79798	1.07285179	0.10077556
1435579_at	---	---	0.79798	-1.09953831	-0.1436832
1427129_a_at	heterogeneous nuclear ribonucleoprotein R	Hnrnpr	0.79798	-1.06376929	-0.089912
1450388_s_at	twisted gastrulation homolog 1 (Drosophila)	Twsg1	0.79798	1.09336629	0.12160938
1458277_at	chemokine (C-C motif) ligand 25	Ccl25	0.79798	1.09252645	0.12408922
1448582_at	catenin, beta like 1	Ctnnbl1	0.79798	1.0732901	0.09925306
1420938_at	heparan sulfate 6-O-sulfotransferase 2	Hs6st2	0.79799	1.08496713	0.11372573
1437748_at	fucosyltransferase 11	Fut11	0.79801	1.11628008	0.14685232
1429537_at	serine/arginine-rich splicing factor 18	Sfrs18	0.79801	-1.08363239	-0.1181611
1456424_s_at	phospholipid transfer protein	Pltp	0.79802	-1.06531585	-0.0914958
1421853_at	presenilin 1	Psen1	0.79802	1.07790874	0.10561217
1446403_at	expressed sequence AU022899	AU022899	0.79803	-1.09691976	-0.1398462
1427276_at	structural maintenance of chromosomes 4	Smc4	0.79803	-1.09131347	-0.148232
1434186_at	lysophosphatidic acid receptor 4	Lpar4	0.79803	1.12940525	0.16240455
1442961_at	---	---	0.79803	-1.1061446	-0.1526472
1454677_at	tissue inhibitor of metalloproteinase 2	Timp2	0.79804	1.0964431	0.13265062
1428876_at	signal recognition particle 72	Srp72	0.79805	-1.06788968	-0.0970381
1446890_at	Predicted gene 5891	Gm5891	0.79805	-1.09571203	-0.1359828
1444221_at	TBC1D12: TBC1 domain family, member 12	Tbc1d12	0.79805	1.09759516	0.12872593
1416129_at	ERBB receptor feedback inhibitor 1	Errfi1	0.79805	1.07459939	0.10359248
1442269_at	hypothetical LOC100503627	LOC100503627	0.79806	1.13253325	0.16361888
1422295_at	MDS1 and EVI1 complex locus	Mecom	0.79806	1.15375868	0.18195756
1422583_at	RAB3B, member RAS oncogene family	Rab3b	0.79807	1.10762363	0.14203441
1424407_s_at	chromobox homolog 6	Cbx6	0.79807	-1.07881161	-0.1185291
1417293_at	heparan sulfate 6-O-sulfotransferase 1	Hs6st1	0.79807	1.10133387	0.12919748
1423910_at	ArfGAP with GTPase domain, ankyrin repeat and PH domain 3	Agap3	0.79807	1.09806735	0.12983283
1431809_at	RIKEN cDNA 4932442L08 gene	4932442L08Rik	0.79808	-1.10251952	-0.1464118
1415681_at	mitochondrial ribosomal protein L43	Mrpl43	0.7981	1.06114341	0.08542042
1421894_a_at	tripeptidyl peptidase II	Tpp2	0.79811	-1.06650688	-0.0933033
1426240_at	chromatin modifying protein 4B	Chmp4b	0.79811	-1.0734978	-0.1069593
1416896_at	ribosomal protein S6 kinase polypeptide 1	Rps6ka1	0.79811	1.07348273	0.09926553
1445868_at	cytoplasmic polyadenylation element binding protein 3	Cpeb3	0.79813	-1.09428315	-0.133401
1432452_at	RIKEN cDNA 1700054O19 gene	1700054O19Rik	0.79813	1.13197202	0.16264338
1448810_at	glucosamine	Gne	0.79813	1.10822026	0.13797805
1435149_at	phospholipase C, gamma 1	Plcg1	0.79814	1.07269993	0.10019551
1423087_a_at	prickle homolog 4 (Drosophila) /// translocase of outer mitochond	Prickle4 /// Tomm6	0.79814	1.07240679	0.09565703
1443431_at	---	---	0.79814	-1.09278987	-0.1291321

1431331_at	monoglyceride lipase	Mgll	0.79814	1.10740346	0.1394681
1420055_at	solute carrier family 35, member C2	Slc35c2	0.79815	-1.09882618	-0.137461
1422423_at	melanoma antigen, family A, 7, pseudogene	Magea7-ps	0.79815	-1.09009616	-0.1265976
1440726_at	---	---	0.79815	1.06529435	0.09042595
1438312_s_at	latent transforming growth factor beta binding protein 3	Ltbp3	0.79816	-1.08552104	-0.1200852
1424892_at	zinc finger with KRAB and SCAN domains 5	Zkscan5	0.79816	1.08523422	0.11433283
1456268_at	hypothetical LOC100034739	LOC100034739	0.79816	-1.10027174	-0.146102
1431492_at	lipase, family member N	Lipn	0.79816	-1.07865551	-0.109382
1442888_at	---	---	0.79817	-1.08998963	-0.1253218
1446924_at	---	---	0.79817	-1.09788328	-0.1409704
1460692_at	euchromatic histone lysine N-methyltransferase 2	Ehmt2	0.79818	-1.0738092	-0.109671
1456237_x_at	HEAT repeat containing 2	Heatr2	0.79818	1.09939188	0.13288056
1417445_at	NDC80 homolog, kinetochore complex component (S. cerevisiae)	Ndc80	0.79819	1.07782527	0.10485296
1437006_x_at	beclin 1, autophagy related	Becn1	0.79819	-1.09062374	-0.1325566
1436580_at	Heparan-alpha-glucosaminide N-acetyltransferase	Hgsnat	0.79819	1.09762816	0.13321645
1426977_at	ubiquitin specific peptidase 47	Usp47	0.79819	1.0747745	0.10302937
1418906_at	nucleotide binding protein 1	Nubp1	0.7982	1.06964601	0.09697699
1426961_at	PHD finger protein 20	Phf20	0.7982	-1.07343632	-0.1063917
1442541_at	Ornithine decarboxylase antizyme 2	Oaz2	0.79821	-1.09029709	-0.1281753
1423546_at	zinc finger protein 207	Zfp207	0.79821	1.12086589	0.15663662
1452705_at	pyridoxal-dependent decarboxylase domain containing 1	Pdxdc1	0.79821	1.07534299	0.1044649
1420700_s_at	folate receptor 4 (delta)	Folr4	0.79821	-1.09125393	-0.128982
1433447_x_at	chaperonin containing Tcp1, subunit 4 (delta)	Cct4	0.79821	-1.06415413	-0.09721
1431085_a_at	protein-L-isoaspartate (D-aspartate) O-methyltransferase 1	Pcmt1	0.79821	-1.08842049	-0.136638
1423235_at	adaptor-related protein complex 3, beta 1 subunit	Ap3b1	0.79822	1.08830301	0.11597022
1424031_at	sorting nexin 11	Snx11	0.79822	-1.09236867	-0.1414001
1416301_a_at	early B-cell factor 1	Ebf1	0.79822	-1.10057154	-0.1459762
1425962_at	killer cell lectin-like receptor subfamily B member 1F	Klrb1f	0.79823	1.10631856	0.14220687
1457117_at	nuclear factor, erythroid derived 2, like 2	Nfe2l2	0.79823	1.11535105	0.14980715
1453022_at	GPI-anchored HDL-binding protein 1	Gpihbp1	0.79824	-1.09530804	-0.1354386
1440764_at	v-raf murine sarcoma 3611 viral oncogene homolog	Araf	0.79824	-1.10285148	-0.1518121
1448156_at	trefoil factor 1	Tff1	0.79824	-1.07165991	-0.1012372
1418075_at	ST6 (alpha-N-acetyl-neuraminyl-2,3-beta-galactosyl-1,3)-N-acety	St6galnac4	0.79824	-1.08518193	-0.122149
1436414_at	obscurin-like 1	Obsl1	0.79825	1.11004776	0.13754832
1459363_at	---	---	0.79826	1.13017166	0.15860171
1433844_a_at	dual specificity phosphatase 9	Dusp9	0.79826	1.08735062	0.11337769
1430774_at	RIKEN cDNA A430106A12 gene	A430106A12Rik	0.79826	1.10876171	0.14285465
1444412_at	---	---	0.79827	-1.09565473	-0.1328591
1433023_at	RIKEN cDNA 2310068G24 gene	2310068G24Rik	0.79827	1.11360907	0.14391624
1455537_at	RIKEN cDNA 6430547I21 gene	6430547I21Rik	0.79828	-1.08256071	-0.1164469
1440595_at	erythrocyte protein band 4.1-like 3	Epb4.1l3	0.79828	1.10549092	0.14109862
1437675_at	solute carrier family 8 (sodium/calcium exchanger), member 1	Slc8a1	0.79828	1.11208278	0.14477203
1456501_at	RIKEN cDNA 2610528E23 gene	2610528E23Rik	0.79828	1.13418857	0.1661528
AFFX-TransRecM	transferrin receptor	Tfrc	0.79828	1.14551059	0.16707091
1447019_at	cytidine monophospho-N-acetylneuraminic acid hydroxylase	Cmah	0.79829	-1.0891366	-0.1239239
1441640_at	---	---	0.79829	1.10115707	0.13636396
1448332_at	peroxisomal biogenesis factor 19	Pex19	0.79829	1.0752426	0.10239029
1425248_a_at	TYRO3 protein tyrosine kinase 3	Tyro3	0.7983	1.09126992	0.12061367
1426976_at	ubiquitin specific peptidase 47	Usp47	0.7983	1.07846273	0.10749598
1424272_at	signal transducer and activator of transcription 3	Stat3	0.7983	-1.09116452	-0.1273171
1433603_at	NADH dehydrogenase (ubiquinone) Fe-S protein 6	Ndufs6	0.79831	1.07785323	0.1029708
1430496_at	acylglycerol kinase	Agk	0.79831	-1.09712403	-0.134842
1431101_a_at	steroid 5 alpha-reductase 1	Srd5a1	0.79831	1.09651613	0.1321697
1422225_s_at	predicted gene 9880 /// t-complex protein 10a /// t-complex prot	Gm9880 /// Tcp10a /// Tc	0.79831	-1.09882841	-0.1365251
1455878_at	RIKEN cDNA 2700023E23 gene	2700023E23Rik	0.79831	1.08983306	0.11791255
1425680_a_at	beta-transducin repeat containing protein	Btrc	0.79831	1.0785403	0.10650146
1421201_a_at	trophinin	Tro	0.79832	1.09041143	0.12274957
1454138_a_at	serine threonine kinase 31	Stk31	0.79832	-1.0990292	-0.1423401
1422886_a_at	CDC like kinase 4	Clk4	0.79832	1.07318997	0.10105926
1428776_at	solute carrier family 10 (sodium/bile acid cotransporter family), m	Slc10a6	0.79832	-1.10077172	-0.1504359
1424382_at	reticulocalbin 3, EF-hand calcium binding domain	Rcn3	0.79832	-1.09386112	-0.1425595
1435170_at	TSR2, 20S rRNA accumulation, homolog (S. cerevisiae)	Tsr2	0.79832	1.10172635	0.13710362
1417452_a_at	Finkel-Biskis-Reilly murine sarcoma virus (FBR-MuSV) ubiquitousl	Fau	0.79833	1.04813799	0.06544821
1446592_at	---	---	0.79833	-1.10223517	-0.1489978
1458869_at	RIKEN cDNA 2900076A13 gene	2900076A13Rik	0.79833	1.12076728	0.1556847
1427028_at	leucine-rich repeat-containing G protein-coupled receptor 6	Lgr6	0.79834	-1.10375375	-0.1476507
1436241_s_at	histone cell cycle regulation defective homolog A (S. cerevisiae)	Hira	0.79835	1.08147895	0.11096081
1457949_at	5'-nucleotidase, cytosolic II	Nt5c2	0.79835	-1.09174584	-0.1307287
1441183_at	---	---	0.79836	-1.09782318	-0.135178
1450716_at	a disintegrin-like and metallopeptidase (reprolysin type) with thro	Adamts1	0.79837	-1.10350304	-0.1493857
1447012_at	predicted gene 10791	Gm10791	0.79837	-1.0829279	-0.1152777
1446686_at	---	---	0.79837	-1.09905499	-0.1445584
1425881_at	pregnancy-specific glycoprotein 28	Psg28	0.79837	1.11334893	0.14745085
1454995_at	dimethylarginine dimethylaminohydrolase 1	Ddah1	0.79837	1.08727819	0.11391162
1445781_at	---	---	0.79837	1.12224414	0.15292834
1447732_x_at	paternally expressed 3	Peg3	0.79838	1.08550579	0.11726369
1441932_at	---	---	0.79838	1.08687156	0.11721824
1420384_at	collagen, type IV, alpha 3 (Goodpasture antigen) binding protein	Col4a3bp	0.79838	-1.09802956	-0.1430658
1417189_at	proteasome (prosome, macropain) 28 subunit, beta	Psme2	0.79838	1.10004438	0.12604396
1447616_at	---	---	0.79839	1.08687857	0.1156474
1447035_at	RIKEN cDNA A230091C14 gene	A230091C14Rik	0.79839	-1.09579861	-0.1322277
1420675_at	zinc finger protein 113	Zfp113	0.7984	1.10913999	0.1434667
1443144_at	protein kinase C, beta	Prkcb	0.7984	-1.09757017	-0.1391466
1419462_s_at	gene trap locus 3	Gtl3	0.79841	1.08185926	0.10836284
1443540_at	---	---	0.79841	1.11313055	0.14800253
1452894_at	ELAV (embryonic lethal, abnormal vision, Drosophila)-like 4 (Hu a	Elavl4	0.79841	-1.10151875	-0.1438185
1428424_at	polycomb group ring finger 3	Pcgf3	0.79842	1.12587259	0.1598001
1443876_at	---	---	0.79842	-1.09205008	-0.1285775
1446715_at	ATP-binding cassette, sub-family A (ABC1), member 13	Abca13	0.79842	-1.09920047	-0.1508629
1431916_at	hydroxy-delta-5-steroid dehydrogenase, 3 beta- and steroid delta	Hsd3b3	0.79843	-1.10321429	-0.1529271
1427212_at	mitogen-activated protein kinase associated protein 1	Mapkap1	0.79843	1.07755203	0.10499205
1418336_at	AFG3(ATPase family gene 3)-like 1 (yeast)	Afg3l1	0.79843	1.09153911	0.12178794
1431050_at	ribosomal protein S6 kinase, polypeptide 5	Rps6ka5	0.79843	-1.08408476	-0.1169478
1421157_at	frizzled homolog 3 (Drosophila)	Fzd3	0.79844	1.11081705	0.14294664

1443841_x_at	UDP-N-acteylglucosamine pyrophosphorylase 1-like 1	Uap1l1	0.79844	1.07757324	0.10630983
1446183_at	Amyotrophic lateral sclerosis 2 (juvenile) homolog (human)	Als2	0.79845	1.10212704	0.13753443
1452048_at	mitochondrial ribosomal protein L12	Mrpl12	0.79846	1.06998332	0.09515672
1438074_at	RIKEN cDNA 2210010C17 gene	2210010C17Rik	0.79848	1.08377969	0.11168536
1441062_at	DEAD/H (Asp-Glu-Ala-Asp/His) box polypeptide 11 (CHL1-like hel	Ddx11	0.79849	1.10006139	0.13354106
1443499_at	hypothetical protein A430075N02	A430075N02	0.7985	1.09844964	0.13539708
1445693_at	v-raf murine sarcoma 3611 viral oncogene homolog	Araf	0.7985	-1.10035374	-0.1452562
1415672_at	golgi autoantigen, golgin subfamily a, 7	Golga7	0.79852	-1.06941538	-0.10193
1448587_at	TBC1 domain family, member 10a	Tbc1d10a	0.79853	1.07139923	0.09823512
1431747_at	ZW10 homolog (Drosophila), centromere/kinetochore protein	Zw10	0.79855	-1.0940169	-0.1464818
1437308_s_at	coagulation factor II (thrombin) receptor	F2r	0.79858	-1.07493338	-0.1091424
1434824_at	bromodomain adjacent to zinc finger domain, 1B	Baz1b	0.79858	1.0709492	0.09680191
1429727_at	solute carrier family 16 (monocarboxylic acid transporters), memb	Slc16a9	0.79858	1.11804149	0.15185609
1422758_at	carbohydrate sulfotransferase 2	Chst2	0.7986	-1.0990814	-0.1370465
1418253_a_at	heat shock protein 4 like	Hspa4l	0.7986	1.09404475	0.12704406
1440421_at	---	---	0.79862	1.13978202	0.16892357
1459268_at	G protein-coupled receptor 179	Gpr179	0.79866	-1.09329106	-0.1301596
1421621_at	RAS protein-specific guanine nucleotide-releasing factor 2	Rasgrf2	0.79866	1.11641923	0.15044716
1418168_at	zinc finger, CCHC domain containing 14	Zcchc14	0.79867	1.08356672	0.11489167
1449576_at	eukaryotic translation initiation factor 1A, X-linked	Eif1ax	0.79867	1.07193464	0.0984515
1451444_s_at	HEAT repeat containing 6	Heatr6	0.79867	1.11515271	0.14239969
1436027_at	oxysterol binding protein-like 11	Osbpl11	0.7987	1.08257834	0.11067928
1430081_at	PHD finger protein 15	Phf15	0.7987	1.10761023	0.13924509
1422925_s_at	acyl-CoA thioesterase 3	Acot3	0.79871	1.11186745	0.14382162
1436244_a_at	transducin-like enhancer of split 2, homolog of Drosophila E(spl)	Tle2	0.79871	1.1108144	0.1440211
1437189_x_at	eukaryotic translation initiation factor 3, subunit M	Eif3m	0.79871	-1.09656969	-0.1405543
1440199_at	solute carrier family 25, member 48	Slc25a48	0.79873	-1.10096328	-0.1475969
1419694_at	ST8 alpha-N-acetyl-neuraminide alpha-2,8-sialyltransferase 1	St8sia1	0.79877	1.10566166	0.14088175
1436831_at	IQ motif and ubiquitin domain containing	Iqub	0.79877	-1.07633684	-0.1072974
1424902_at	plexin domain containing 1	Plxdc1	0.79878	1.09619477	0.1302194
1440689_at	forkhead box K2	Foxk2	0.79878	-1.09460469	-0.1328644
1451951_at	predicted gene, 16939	Gm16939	0.79878	1.10368616	0.13841479
1419575_s_at	zinc finger protein 292	Zfp292	0.79878	-1.0699368	-0.0980026
1441456_at	matrix metallopeptidase 24	Mmp24	0.79879	1.1181716	0.15135991
1447769_x_at	adhesion molecule with Ig like domain 2	Amigo2	0.7988	-1.09652816	-0.1513269
1458940_at	RIKEN cDNA 5730507A11 gene	5730507A11Rik	0.79883	1.09085158	0.12226504
1426935_at	RIKEN cDNA 6330590E21 gene	6330590E21Rik	0.79884	-1.10164579	-0.1424871
1445757_at	T-box 3	Tbx3	0.79885	-1.09518622	-0.1325791
1452132_at	TLC domain containing 1	Tlcd1	0.79891	1.07655969	0.10550856
1430789_at	RIKEN cDNA 4930452B06 gene	4930452B06Rik	0.79893	1.10839078	0.14237451
1458870_x_at	MYC binding protein 2	Mycbp2	0.79894	-1.10794402	-0.1622957
1454459_at	RIKEN cDNA 4933423L19 gene	4933423L19Rik	0.79895	-1.10493441	-0.1481913
1421741_at	cytochrome P450, family 3, subfamily a, polypeptide 16	Cyp3a16	0.79897	-1.08487208	-0.1244456
1427400_at	ladybird homeobox homolog 1 (Drosophila)	Lbx1	0.79897	1.08333642	0.11305008
1446595_at	intersectin 2	Itsn2	0.79898	1.1294919	0.16058156
1423644_at	aconitase 1	Aco1	0.79899	1.08324576	0.1118797
1451491_at	BCDIN3 domain containing	Bcdin3d	0.79899	1.07583222	0.10340462
1448471_a_at	cytotoxic T lymphocyte-associated protein 2 alpha	Ctla2a	0.799	1.09901868	0.12967836
1454664_a_at	eukaryotic translation initiation factor 5	Eif5	0.799	-1.07824468	-0.118088
1456220_at	F-box and leucine-rich repeat protein 7	Fbxl7	0.79903	1.11571842	0.14981524
1445007_at	---	---	0.79903	-1.09400001	-0.1309034
1419900_at	---	---	0.79903	-1.12994154	-0.2418651
1421712_at	selectin, endothelial cell	Sele	0.79904	-1.09285705	-0.1314457
1452775_at	cysteine and glycine-rich protein 2 binding protein	Csrp2bp	0.79905	1.0747334	0.10267308
1426540_at	endonuclease domain containing 1	Endod1	0.79905	-1.09305006	-0.1366151
1426165_a_at	caspase 3	Casp3	0.79905	-1.08021153	-0.1132171
1437161_x_at	RNA binding protein gene with multiple splicing	Rbpms	0.79905	-1.06407976	-0.0899161
1457733_at	---	---	0.79907	-1.07752987	-0.1085171
1433478_at	presenilin associated, rhomboid-like	Parl	0.7991	1.06365809	0.08845818
1432250_at	UTP14, U3 small nucleolar ribonucleoprotein, homolog A (yeast)	Utp14a	0.79911	1.09157013	0.1231792
1459764_x_at	Sorting nexin 10	Snx10	0.79912	-1.10795713	-0.1643105
1435575_at	kinetochore associated 1	Kntc1	0.79913	-1.0800322	-0.1135831
1455712_at	histone cluster 3, H2a	Hist3h2a	0.79913	1.09940424	0.13157079
1451602_at	sorting nexin 6	Snx6	0.79916	-1.08271063	-0.1193662
1435606_at	galactose-3-O-sulfotransferase 4 /// glypican 2 (cerebroglycan)	Gal3st4 /// Gpc2	0.79918	1.14405107	0.17510678
1433448_at	solute carrier family 25, member 44	Slc25a44	0.79923	-1.08369806	-0.118412
1434663_at	mitotic spindle organizing protein 1	Mzt1	0.79924	-1.06959169	-0.0976417
1459848_x_at	dopamine beta hydroxylase	Dbh	0.79928	-1.09307499	-0.1439915
1459177_at	expressed sequence AU015741	AU015741	0.79946	1.14944335	0.17760947
1448167_at	interferon gamma receptor 1	Ifngr1	0.79949	-1.08759297	-0.1241575
1451443_at	nuclear factor I/X	Nfix	0.79957	-1.10084601	-0.1406117
1443273_at	---	---	0.79957	1.15533135	0.18131677
1452580_a_at	mitochondrial ribosomal protein L21	Mrpl21	0.7997	1.07422073	0.09993498
1440240_at	neuropeptide B	Npb	0.7999	1.09803351	0.13247292
1426191_a_at	BCL2-like 1	Bcl2l1	0.79994	-1.08911637	-0.1254581
1417463_a_at	RIKEN cDNA 2400001E08 gene	2400001E08Rik	0.79995	-1.06819338	-0.0976556
1426733_at	inositol 1,3,4-triphosphate 5/6 kinase	Itpk1	0.80003	1.07123876	0.096851
1416818_at	parvin, alpha	Parva	0.80003	1.11912341	0.15267971
1456708_at	RIKEN cDNA 2610028E06 gene	2610028E06Rik	0.80015	-1.08993508	-0.1271966
1427866_x_at	hemoglobin, beta adult minor chain	Hbb-b2	0.80016	1.09526003	0.12211401
1434511_at	phosphorylase kinase beta	Phkb	0.80017	-1.07265131	-0.102827
1443251_at	---	---	0.80025	1.10194177	0.13938407
1440729_at	epidermal growth factor receptor pathway substrate 15	Eps15	0.8007	1.09361394	0.12771764
1435626_a_at	homocysteine-inducible, endoplasmic reticulum stress-inducible, u	Herpud1	0.80076	-1.0801681	-0.1185666
1460685_at	translocase of inner mitochondrial membrane 17b	Timm17b	0.8008	1.07256058	0.09987603
1456157_at	---	---	0.80083	-1.1004683	-0.1389288
1434162_at	RIKEN cDNA 2700078E11 gene	2700078E11Rik	0.80083	1.06853549	0.09536769
1430400_at	RIKEN cDNA 4930521A18 gene	4930521A18Rik	0.80083	1.09346502	0.12312785
1433923_at	keratin 77	Krt77	0.80084	-1.10902856	-0.1580027
1453343_s_at	vaccinia related kinase 2	Vrk2	0.80084	-1.09456756	-0.1379961
1442986_at	---	---	0.80084	1.12562967	0.15947965
1437777_at	activating transcription factor 2	Atf2	0.80084	-1.10643108	-0.1548454
1438842_at	mitochondrial carrier homolog 2 (C. elegans)	Mtch2	0.80085	-1.1008744	-0.1407513
1428040_at	one cut domain, family member 3	Onecut3	0.80085	1.10636739	0.14343142

1460490_at	mitochondrial ribosomal protein L15	Mrpl15	0.80086	-1.09631229	-0.1328838
1435284_at	reticulon 4	Rtn4	0.80086	1.12742669	0.15302891
1451564_at	poly (ADP-ribose) polymerase family, member 14	Parp14	0.80087	1.11683561	0.14755423
1434399_at	UDP-N-acetyl-alpha-D-galactosamine:polypeptide N-acetylgalact	Galnt6	0.80089	1.08190634	0.11243981
1421916_at	platelet derived growth factor receptor, alpha polypeptide	Pdgfra	0.8009	1.10316815	0.13865253
1417815_a_at	serine incorporator 3	Serinc3	0.80091	-1.07599075	-0.1124148
1425367_at	integrin alpha L	Itgal	0.80091	1.09748199	0.13193234
1434342_at	S100 protein, beta polypeptide, neural	S100b	0.80091	1.08615876	0.11855592
1448053_at	---	---	0.80092	1.08216389	0.11153282
1422957_at	chemokine (C-C motif) receptor 3	Ccr3	0.80093	-1.09791039	-0.146273
1450265_at	MAD homolog 9 (Drosophila)	Smad9	0.80095	-1.09917614	-0.136754
1426519_at	procollagen-proline, 2-oxoglutarate 4-dioxygenase (proline 4-hyd	P4ha1	0.80095	1.08757285	0.11928753
1447188_at	---	---	0.80096	-1.10272197	-0.1427909
1429663_at	katanin p60 subunit A-like 2	Katnal2	0.80097	1.10111807	0.13599641
1422372_at	olfactory receptor 15	Olfr15	0.80098	-1.09720802	-0.1377352
1424113_at	laminin B1	Lamb1	0.80098	-1.09769484	-0.1452432
1425623_a_at	cystathionine beta-synthase	Cbs	0.80099	1.08350354	0.11405573
1426881_at	ubiquitin protein ligase E3C	Ube3c	0.80101	1.08408912	0.1121146
1422693_a_at	SUB1 homolog (S. cerevisiae)	Sub1	0.80101	-1.05217057	-0.0742496
1418770_at	CD2 antigen	Cd2	0.80102	1.10492095	0.13787749
1458023_at	G patch domain and KOW motifs	Gpkow	0.80103	1.09802951	0.13404987
1440700_a_at	rho/rac guanine nucleotide exchange factor (GEF) 18	Arhgef18	0.80105	1.09262904	0.12573331
1433838_at	aspartyl-tRNA synthetase 2 (mitochondrial)	Dars2	0.80105	1.07662016	0.1043762
1420167_at	---	---	0.80106	-1.10045111	-0.1404367
1448253_at	glutamate dehydrogenase 1	Glud1	0.80106	1.0606212	0.08476154
1433683_at	epithelial splicing regulatory protein 2	Esrp2	0.80106	1.08189696	0.11337311
1431598_a_at	LIM homeobox protein 9	Lhx9	0.80107	1.09244241	0.12673062
1459513_at	---	---	0.80108	-1.0976394	-0.1381253
1416635_at	sphingomyelin phosphodiesterase, acid-like 3A	Smpdl3a	0.80111	1.13329955	0.16171243
1422439_a_at	cyclin-dependent kinase 4	Cdk4	0.80113	1.07303695	0.09878795
1421225_a_at	solute carrier family 4 (anion exchanger), member 4	Slc4a4	0.80114	-1.11027204	-0.1726153
1421995_at	transcription factor AP-2, alpha	Tcfap2a	0.80114	-1.10793136	-0.1620929
1437868_at	family with sequence similarity 46, member A	Fam46a	0.80114	1.10467478	0.13464433
1416185_a_at	alcohol dehydrogenase 5 (class III), chi polypeptide	Adh5	0.80115	-1.05195857	-0.0738786
1449792_at	---	---	0.80115	-1.09877589	-0.1427871
1438550_x_at	serine racemase	Srr	0.80116	1.08678415	0.11860129
1453298_at	protein tyrosine phosphatase, non-receptor type 21	Ptpn21	0.80119	1.11326866	0.14364155
1424050_s_at	fibroblast growth factor receptor 1	Fgfr1	0.80119	1.14733083	0.16818064
1427860_at	---	---	0.8012	1.08707215	0.11989827
1456154_at	---	---	0.8012	-1.09548207	-0.1377239
1430402_at	succinate-Coenzyme A ligase, ADP-forming, beta subunit	Sucla2	0.8012	1.0791403	0.10749995
1417646_a_at	sorting nexin 5	Snx5	0.80131	-1.07697829	-0.1192047
1419291_x_at	growth arrest specific 5	Gas5	0.80132	1.0667249	0.09189557
1418760_at	retinol dehydrogenase 11	Rdh11	0.80134	-1.08893427	-0.1330198
1440571_at	Eukaryotic translation initiation factor 4 gamma, 3	Eif4g3	0.80135	-1.10011327	-0.1447393
1438488_at	esterase D/formylglutathione hydrolase	Esd	0.80135	1.09383447	0.12508729
1447179_at	---	---	0.80136	-1.09763441	-0.1372195
1424417_at	vacuolar protein sorting 36 (yeast)	Vps36	0.80137	-1.09140537	-0.1313068
1452083_a_at	praja1, RING-H2 motif containing	Pja1	0.80138	-1.09974465	-0.1597928
1416969_at	G two S phase expressed protein 1	Gtse1	0.80138	1.07315924	0.09888399
1436081_a_at	zinc finger protein 414	Zfp414	0.80139	1.0720273	0.09896223
1439090_at	TBC1 domain family, member 23	Tbc1d23	0.80139	1.13241119	0.16274438
1460139_at	expressed sequence C79256	C79256	0.80141	-1.07568544	-0.1113104
1440632_at	protocadherin beta 4	Pcdhb4	0.80142	1.11236003	0.13781931
1451775_s_at	interleukin 13 receptor, alpha 1	Il13ra1	0.80143	-1.11176728	-0.1792653
1459126_at	---	---	0.80143	1.09256467	0.12647191
1416486_at	aminoacyl tRNA synthetase complex-interacting multifunctional p	Aimp1	0.80146	1.06080727	0.08510972
1436468_at	zinc finger, DHHC domain containing 8	Zdhhc8	0.80146	-1.09310855	-0.1301216
1458946_at	---	---	0.8015	-1.09676204	-0.1356753
1455299_at	vestigial like 3 (Drosophila)	Vgll3	0.80151	-1.09466575	-0.1418485
1452460_at	ankyrin repeat domain 26	Ankrd26	0.80152	-1.08627184	-0.1193896
1427026_at	myosin, heavy polypeptide 4, skeletal muscle	Myh4	0.80155	-1.09727181	-0.1419748
1457506_at	---	---	0.80156	1.10158968	0.13669945
1450165_at	schlafen 2	Slfn2	0.80157	-1.08608551	-0.120908
1438033_at	thyrotroph embryonic factor	Tef	0.80158	1.10788434	0.14477798
1457535_at	RIKEN cDNA 4932438A13 gene	4932438A13Rik	0.80159	1.12508605	0.15753243
1435571_at	myelin protein zero-like 3	Mpzl3	0.80162	-1.09763446	-0.135501
1427092_at	---	---	0.80164	-1.09856901	-0.1542164
1418227_at	origin recognition complex, subunit 2	Orc2	0.80164	-1.07088389	-0.1011666
1451543_at	F-box protein 21	Fbxo21	0.80176	1.07433122	0.10249054
1445423_at	---	---	0.80177	1.10832734	0.14346089
1423485_at	RAD54 like 2 (S. cerevisiae)	Rad54l2	0.80179	-1.08202199	-0.1145211
1420952_at	Son DNA binding protein	Son	0.80179	1.10395694	0.12770235
1460171_at	COP9 (constitutive photomorphogenic) homolog, subunit 5 (Arabi	Cops5	0.8018	1.0654582	0.08984435
1428646_at	pre B-cell leukemia transcription factor 1	Pbx1	0.80184	-1.09257712	-0.1321752
1452305_s_at	centromere protein N	Cenpn	0.80185	1.07809185	0.10604321
1445208_at	exonuclease 3'-5' domain containing 2	Exd2	0.80185	1.11086424	0.14622138
1437904_at	RNA binding motif protein 45	Rbm45	0.8019	-1.06618005	-0.0939004
1455327_at	SUMO/sentrin specific peptidase 2	Senp2	0.8019	1.09816991	0.13048617
1448017_at	ubiquitin associated protein 2-like	Ubap2l	0.80193	-1.09255051	-0.1313523
1458337_at	---	---	0.80199	-1.10711625	-0.15356
1446873_at	---	---	0.80201	-1.08200926	-0.1183064
1423517_at	chaperonin containing Tcp1, subunit 6a (zeta)	Cct6a	0.80202	1.04372097	0.06038152
1442711_at	---	---	0.80203	-1.09541009	-0.1378268
1425502_x_at	---	---	0.80203	-1.1008625	-0.1452237
1425129_a_at	transaldolase 1	Taldo1	0.80203	1.05914273	0.08273868
1431887_at	RNA binding motif 31, Y-linked	Rbm31y	0.80226	1.09569032	0.12748885
1444888_at	expressed sequence AU022852	AU022852	0.80228	1.1146687	0.14773835
1426864_a_at	neural cell adhesion molecule 1	Ncam1	0.80229	-1.07682947	-0.1071917
1434715_at	RIKEN cDNA 1600014C10 gene	1600014C10Rik	0.80235	1.11408592	0.14621912
1457130_at	RIKEN cDNA 2900035I09 gene	2900035I09Rik	0.80235	1.10078315	0.13648754
1459895_at	protein phosphatase 4, regulatory subunit 1-like, pseudogene	Ppp4r1l-ps	0.80236	-1.09526052	-0.1321004
1419062_at	erythrocyte protein band 4.1-like 3	Epb4.1l3	0.80249	1.09991972	0.13074042
1433745_at	triple functional domain (PTPRF interacting)	Trio	0.80249	1.10872428	0.13679454

1453653_at	RIKEN cDNA 6330576A10 gene	6330576A10Rik	0.80249	1.13021264	0.16287161
1423081_a_at	translocase of outer mitochondrial membrane 20 homolog (yeast	Tomm20	0.8025	1.0715406	0.09961126
1428086_at	dynamin 1-like	Dnm1l	0.80252	-1.06364442	-0.0900268
1424362_at	phosphatidic acid phosphatase type 2 domain containing 3	Ppapdc3	0.80252	1.09897068	0.13531438
1428708_x_at	parathymosin	Ptms	0.80253	1.08488354	0.10946397
1431407_at	RIKEN cDNA 2310024H09 gene	2310024H09Rik	0.80253	-1.0792335	-0.1150935
1430344_at	zinc finger and BTB domain containing 25	Zbtb25	0.80254	1.10490424	0.14024259
1431402_at	kin of IRRE like 3 (Drosophila)	Kirrel3	0.80254	1.16389411	0.1869911
1456031_at	tubulin, beta 2C	Tubb2c	0.80255	-1.07859603	-0.1137212
1435891_x_at	RIKEN cDNA 2610021A01 gene	2610021A01Rik	0.80255	-1.07364187	-0.10377
1429897_a_at	DNA segment, Chr 16, ERATO Doi 472, expressed	D16Ertd472e	0.80256	-1.07740882	-0.1133496
1457679_at	DNA cross-link repair 1C, PSO2 homolog (S. cerevisiae)	Dclre1c	0.80256	-1.09479152	-0.1348376
1455739_at	predicted gene 4980	Gm4980	0.80256	-1.08521543	-0.1193351
1443472_at	expressed sequence C77691	C77691	0.80257	-1.10135334	-0.145455
1450706_a_at	ADP-ribosylation factor-like 3	Arl3	0.80257	-1.07842471	-0.1108054
1428125_at	ataxin 7-like 3B	Atxn7l3b	0.80258	-1.07334423	-0.1074701
1443798_at	phosphatidylinositol 3-kinase catalytic delta polypeptide	Pik3cd	0.80258	-1.07161219	-0.1020302
1456190_a_at	acyl-CoA synthetase medium-chain family member 2	Acsm2	0.80259	1.08183898	0.11276506
1432841_at	RIKEN cDNA E130116L18 gene	E130116L18Rik	0.80259	-1.09906878	-0.1451187
1454757_s_at	interferon, alpha-inducible protein 27 like 1	Ifi27l1	0.8026	-1.091148	-0.1275935
1439296_at	Prickle homolog 3 (Drosophila)	Prickle3	0.8026	-1.11077692	-0.177292
1450588_at	olfactory receptor 67	Olfr67	0.80261	1.08514631	0.11785864
1431096_at	integrator complex subunit 8	Ints8	0.80261	-1.1025946	-0.1645933
1438067_at	neurofibromatosis 1	Nf1	0.80261	1.10616549	0.13704099
1434270_at	neuronal pentraxin chromo domain /// neuronal pentraxin recept	Npcd /// Nptxr	0.80261	1.08748711	0.12090024
1460118_at	---	---	0.80262	1.10299541	0.1361136
1424114_s_at	laminin B1	Lamb1	0.80262	-1.06220069	-0.0876269
1429217_at	zinc finger protein 655	Zfp655	0.80263	1.08832308	0.11882481
1439873_at	diacylglycerol kinase, delta	Dgkd	0.80263	1.09592999	0.13016226
1428358_at	zymogen granule protein 16	Zg16	0.80263	1.11512459	0.14855433
1452924_at	family with sequence similarity 83, member D	Fam83d	0.80264	1.07934398	0.10811131
1448487_at	leucine rich repeat (in FLII) interacting protein 1	Lrrfip1	0.80266	1.09987632	0.13238757
1419131_at	coagulation factor XIII, beta subunit	F13b	0.80266	1.08427832	0.11666795
1421583_at	cAMP responsive element binding protein 1	Creb1	0.80266	1.08862758	0.11683458
1432600_at	RIKEN cDNA 2310061A09 gene	2310061A09Rik	0.80266	1.13643922	0.16343863
1448809_at	chromosome segregation 1-like (S. cerevisiae)	Cse1l	0.80266	1.06985388	0.09417339
1459913_at	tumor necrosis factor (ligand) superfamily, member 10	Tnfsf10	0.80267	-1.11002558	-0.1678983
1424319_at	oral cancer overexpressed 1	Oraov1	0.80267	1.07651352	0.10572897
1420554_a_at	RAS-related C3 botulinum substrate 3	Rac3	0.80267	1.08743995	0.1156183
1429432_at	HLA-B associated transcript 2-like 2	Bat2l2	0.80268	-1.10410912	-0.1633878
1422242_at	defensin, alpha, related sequence 10	Defa-rs10	0.80269	-1.07707631	-0.1077713
1439503_at	zinc finger protein 28	Zfp28	0.80269	1.09394505	0.12908892
1421174_at	interferon regulatory factor 4	Irf4	0.80269	1.11137798	0.14564004
1429468_at	RIKEN cDNA 1110018F16 gene /// DnaJ (Hsp40) homolog, subfar	1110018F16Rik /// Dnajb	0.80269	-1.10909906	-0.1629952
1442645_at	ATPase, Ca++ transporting, plasma membrane 3	Atp2b3	0.80269	-1.09853727	-0.1404579
1417171_at	IL2-inducible T-cell kinase	Itk	0.8027	-1.10003986	-0.1483892
1459376_at	---	---	0.8027	-1.09156454	-0.1308076
1422078_at	thymoma viral proto-oncogene 3	Akt3	0.8027	1.12706263	0.15752182
1424082_at	TBC1 domain family, member 13	Tbc1d13	0.8027	1.07603501	0.10392611
1422979_at	suppressor of variegation 3-9 homolog 2 (Drosophila)	Suv39h2	0.8027	-1.10232315	-0.175895
1439348_at	S100 calcium binding protein A10 (calpactin)	S100a10	0.8027	-1.09661286	-0.1359055
1440696_at	TMF1-regulated nuclear protein 1	Trnp1	0.80271	-1.09515814	-0.1323735
1432143_a_at	high mobility group box transcription factor 1	Hbp1	0.80271	1.09514804	0.12211622
1435580_at	RIKEN cDNA C230081A13 gene	C230081A13Rik	0.80272	-1.0725003	-0.1018571
1453882_at	RIKEN cDNA 1700034F02 gene	1700034F02Rik	0.80272	-1.10176682	-0.1576007
1426617_a_at	tweety homolog 1 (Drosophila)	Ttyh1	0.80272	-1.08774656	-0.1303035
1427346_at	predicted gene 10439 /// predicted gene 15080 /// predicted ger	Gm10439 /// Gm15080 ///	0.80273	-1.10012478	-0.144247
1437453_s_at	proprotein convertase subtilisin/kexin type 9	Pcsk9	0.80273	-1.09872137	-0.1389901
1453543_at	RIKEN cDNA 4930556H04 gene	4930556H04Rik	0.80273	-1.09579245	-0.1407878
1459164_at	expressed sequence AU014678	AU014678	0.80273	1.09506425	0.12869408
1456690_at	---	---	0.80273	1.1309419	0.1596166
1417792_at	zinc finger, matrin-like	Zfml	0.80273	-1.0759843	-0.1082178
1426656_at	family with sequence similarity 63, member A	Fam63a	0.80275	-1.07430482	-0.1092285
1424121_at	COMM domain containing 1	Commd1	0.80275	-1.08103577	-0.1205798
1446167_at	---	---	0.80275	1.09311878	0.12789269
1433801_at	RIKEN cDNA 9930012K11 gene	9930012K11Rik	0.80275	1.07683878	0.10353942
1440346_at	KDM1 lysine (K)-specific demethylase 6B	Kdm6b	0.80275	-1.07415534	-0.1053994
1459850_x_at	glycine receptor, beta subunit	Glrb	0.80275	-1.09539847	-0.133293
1419212_at	icos ligand	Icosl	0.80275	-1.08504541	-0.1211481
1442584_at	RIKEN cDNA 9330121J05 gene	9330121J05Rik	0.80275	1.09543552	0.13091181
1439605_at	---	---	0.80276	-1.10151961	-0.1403375
1434226_at	cytochrome b-561 domain containing 1	Cyb561d1	0.80276	1.13204084	0.16224455
1447452_at	Receptor accessory protein 3	Reep3	0.80276	1.09681533	0.1307052
1460567_at	regulatory factor X, 7	Rfx7	0.80276	-1.08509334	-0.129902
1455250_at	SH3-domain binding protein 4	Sh3bp4	0.80276	-1.10176804	-0.1465992
1457358_at	---	---	0.80276	1.10901529	0.14143212
1448321_at	SPARC related modular calcium binding 1	Smoc1	0.80276	1.07132186	0.09788369
1437387_at	sushi domain containing 5	Susd5	0.80277	1.13156204	0.16343466
1431529_at	RIKEN cDNA A930002H02 gene	A930002H02Rik	0.80277	-1.09518693	-0.1321977
1445026_at	---	---	0.80277	-1.07895739	-0.1110167
1447018_at	---	---	0.80277	-1.10143901	-0.1492223
1435520_at	Musashi homolog 2 (Drosophila)	Msi2	0.80278	-1.07905169	-0.1112677
1416358_at	major facilitator superfamily domain containing 10	Mfsd10	0.80278	1.07209214	0.09989581
1426891_at	RNA polymerase II associated protein 1	Rpap1	0.80278	1.07216051	0.0988881
1417240_at	zyxin	Zyx	0.80278	1.07362527	0.1011995
1422385_at	olfactory receptor 1264	Olfr1264	0.80278	1.11302898	0.15026417
1420742_at	a disintegrin and metallopeptidase domain 7	Adam7	0.80278	-1.09764378	-0.142918
1424057_at	ganglioside-induced differentiation-associated-protein 2	Gdap2	0.80278	1.07542068	0.10481419
1433184_at	RIKEN cDNA 6720477C19 gene	6720477C19Rik	0.80279	-1.092711	-0.1279647
1448832_a_at	complexin 1	Cplx1	0.80279	1.08168099	0.10976569
1427180_at	solute carrier family 27 (fatty acid transporter), member 3	Slc27a3	0.8028	1.09627808	0.12855526
1441531_at	phospholipase C, beta 4	Plcb4	0.8028	-1.12341721	-0.2218988
1453336_at	family with sequence similarity 175, member A	Fam175a	0.8028	-1.08837871	-0.1227939
1439711_at	---	---	0.8028	1.07684864	0.10427889

1451573_a_at	syntaxin 4A (placental)	Stx4a	0.80281	-1.08139289	-0.1180067
1444549_at	gamma-aminobutyric acid (GABA) A receptor, subunit gamma 3	Gabrg3	0.80281	-1.08638609	-0.1206161
1448131_at	mitofusin 2	Mfn2	0.80281	1.11006495	0.13655305
1459573_at	ribosome production factor 1 homolog (S. cerevisiae)	Rpf1	0.80281	-1.08842293	-0.1233953
1427225_at	epsin 2	Epn2	0.80281	1.11169044	0.13580072
1441079_at	methyl-CpG binding domain protein 4	Mbd4	0.80281	-1.09368622	-0.1423836
1426139_a_at	chemokine (C-C motif) receptor-like 1	Ccrl1	0.80281	-1.07860073	-0.1141867
1456262_at	RNA binding motif protein 5	Rbm5	0.80281	-1.10046373	-0.1481658
1431418_at	RIKEN cDNA C030026M15 gene	C030026M15Rik	0.80281	-1.10132798	-0.1489817
1417720_at	polymerase (RNA) II (DNA directed) polypeptide J	Polr2j	0.80282	1.06872192	0.094124
1448912_at	C1q and tumor necrosis factor related protein 1	C1qtnf1	0.80282	1.10634982	0.13872776
1450094_at	RAN binding protein 17	Ranbp17	0.80282	1.09047727	0.12226741
1427035_at	solute carrier family 39 (zinc transporter), member 14	Slc39a14	0.80282	1.07733568	0.1050068
1450308_a_at	5'-3' exoribonuclease 1	Xrn1	0.80282	1.1353428	0.1611082
1459339_at	---	---	0.80282	1.09864587	0.13209128
1430221_at	RIKEN cDNA 9130008F23 gene	9130008F23Rik	0.80282	-1.10494115	-0.1513785
1418485_at	solute carrier family 4 (anion exchanger), member 3	Slc4a3	0.80282	1.08432223	0.11462089
1419252_at	epidermal growth factor receptor pathway substrate 15	Eps15	0.80282	1.07432602	0.10281752
1460607_at	immunoglobulin superfamily, member 11	Igsf11	0.80282	-1.10175575	-0.1537848
1427264_at	crystallin, gamma B	Crygb	0.80282	1.12283997	0.15402929
1417902_at	solute carrier family 19 (thiamine transporter), member 2	Slc19a2	0.80283	-1.06692744	-0.0945711
1429074_at	RIKEN cDNA 1700026D08 gene	1700026D08Rik	0.80283	1.0876144	0.12055974
1423598_at	ATPase, aminophospholipid transporter (APLT), class I, type 8A, m	Atp8a1	0.80283	-1.10048637	-0.1511894
1443102_at	zinc finger protein 597	Zfp597	0.80283	1.09412697	0.12861912
1416495_s_at	NADH dehydrogenase (ubiquinone) Fe-S protein 5	Ndufs5	0.80283	1.09120639	0.11588302
1425614_x_at	histocompatibility 2, D region locus 1	H2-D1	0.80283	1.10010671	0.1339827
1454987_a_at	H2-K region expressed gene 6	H2-Ke6	0.80283	1.08477974	0.11605423
1417919_at	protein phosphatase 1, regulatory (inhibitor) subunit 7	Ppp1r7	0.80283	1.06830674	0.09482871
1453655_at	RIKEN cDNA 2900005J15 gene	2900005J15Rik	0.80283	1.15858388	0.18473804
1432781_at	inturned planar cell polarity effector homolog (Drosophila)	Intu	0.80284	-1.09094647	-0.1269353
1445508_at	---	---	0.80284	-1.10058535	-0.1552106
1451117_a_at	target of myb1-like 1 (chicken)	Tom1l1	0.80284	-1.08588328	-0.1205985
1438116_x_at	solute carrier family 9 (sodium/hydrogen exchanger), member 3 r	Slc9a3r1	0.80285	1.06772407	0.09307746
1423715_a_at	neural precursor cell expressed, developmentally down-regulated	Nedd8	0.80285	1.06399425	0.08811144
1456472_at	armadillo repeat containing 4	Armc4	0.80285	1.10930666	0.14319247
1434081_at	adaptor protein complex AP-1, gamma 1 subunit	Ap1g1	0.80285	1.07992659	0.10706449
1428109_at	vacuolar protein sorting 11 (yeast)	Vps11	0.80286	1.0741502	0.10183727
1459980_x_at	RAB3A, member RAS oncogene family	Rab3a	0.80286	1.08000634	0.10656267
1444867_at	expressed sequence C76751	C76751	0.80286	-1.08159303	-0.1141834
1435619_at	PHD finger protein 21A	Phf21a	0.80286	-1.08515853	-0.124127
1454283_at	solute carrier family 38, member 9	Slc38a9	0.80286	-1.09967402	-0.1416699
1446035_at	Predicted gene 7173	Gm7173	0.80286	1.08807132	0.11662398
1437645_at	activating transcription factor 7	Atf7	0.80286	-1.09643971	-0.1427652
1460132_at	Ubiquitin-conjugating enzyme E2B, RAD6 homology (S. cerevisiae	Ube2b	0.80286	-1.09318094	-0.1294929
1427175_at	expressed sequence AI428936	AI428936	0.80286	1.0791863	0.10897248
1457162_at	low density lipoprotein receptor adaptor protein 1	Ldlrap1	0.80287	-1.09519866	-0.1332795
1457454_at	ubiquitin specific peptidase 47	Usp47	0.80287	-1.09643725	-0.1350719
1434021_at	RIKEN cDNA C230096C10 gene	C230096C10Rik	0.80287	1.07306155	0.1011154
1422169_a_at	brain derived neurotrophic factor	Bdnf	0.80287	-1.1002772	-0.1455691
1420986_s_at	kinesin family member 3B	Kif3b	0.80287	1.08953135	0.12118301
1434785_at	calcium channel, voltage-dependent, gamma subunit 5	Cacng5	0.80287	1.11559937	0.14819486
1439406_x_at	phenylalanine-tRNA synthetase 2 (mitochondrial)	Fars2	0.80287	-1.09492124	-0.1458413
1454101_at	RIKEN cDNA 4933401P06 gene	4933401P06Rik	0.80287	1.10353495	0.13784865
1415986_at	chloride channel 4-2	Clcn4-2	0.80287	1.11261059	0.14632026
1440560_at	---	---	0.80287	1.09773222	0.13421054
1432531_at	RIKEN cDNA 9230104L09 gene	9230104L09Rik	0.80288	1.08757812	0.12033402
1451705_a_at	opioid receptor, mu 1	Oprm1	0.80288	1.09970203	0.13273542
1429079_a_at	RIKEN cDNA 2310045N01 gene	2310045N01Rik	0.80288	1.08131566	0.10892198
1457067_at	vacuolar protein sorting 4b (yeast)	Vps4b	0.80288	1.10384477	0.13833844
1457691_at	G protein-coupled receptor 183	Gpr183	0.80288	-1.09845151	-0.1387198
1418583_at	histidine triad nucleotide binding protein 3	Hint3	0.80288	1.09401476	0.12073147
1416262_at	transmembrane protein 19	Tmem19	0.80288	-1.07593141	-0.1083611
1425511_at	MAP/microtubule affinity-regulating kinase 1	Mark1	0.80288	1.11386643	0.14359571
1423181_s_at	chloride channel, nucleotide-sensitive, 1A	Clns1a	0.80288	1.06297035	0.0874925
1450180_a_at	retinoic acid receptor, alpha	Rara	0.80288	1.10193953	0.12802501
1429869_at	WD repeat domain 65	Wdr65	0.80288	-1.08542359	-0.1190771
1436675_at	WD repeat domain 63	Wdr63	0.80288	1.09539183	0.12547862
1424441_at	solute carrier family 27 (fatty acid transporter), member 4	Slc27a4	0.80288	1.07729733	0.10546049
1428328_at	nucleoporin 50	Nup50	0.80288	1.06831167	0.09393315
1433812_at	Lix1-like	Lix1l	0.80289	1.07652367	0.10628176
1454949_at	ubiquitin specific peptidase 7	Usp7	0.80289	1.10299131	0.1316929
1445681_at	Cell division cycle associated 7	Cdca7	0.8029	1.1062209	0.13700953
1455949_at	ATPase, Na+/K+ transporting, beta 1 polypeptide	Atp1b1	0.8029	-1.09594083	-0.1451552
1431171_at	RIKEN cDNA D730001G18 gene	D730001G18Rik	0.8029	1.11151202	0.14405162
1424816_at	cat eye syndrome chromosome region, candidate 5 homolog (hur	Cecr5	0.8029	-1.09085543	-0.1308932
1435500_at	RAB26, member RAS oncogene family	Rab26	0.8029	1.09236435	0.12701173
1442118_at	---	---	0.8029	1.09243493	0.12632464
1427331_at	adenosine A1 receptor	Adora1	0.80291	-1.1073321	-0.1812542
1430607_at	TBC1 domain family, member 30	Tbc1d30	0.80291	1.10627042	0.14138325
1424381_at	SURP and G patch domain containing 1	Sugp1	0.80291	1.08359601	0.10818255
1420884_at	sarcolipin	Sln	0.80291	-1.09033248	-0.1278374
1458834_at	---	---	0.80291	-1.07544093	-0.1058296
1431727_at	submandibular gland protein C	Smgc	0.80292	-1.0938224	-0.1303118
1433288_at	RIKEN cDNA 1520401O13 gene	1520401O13Rik	0.80292	-1.10225202	-0.1536391
1453651_a_at	brain protein 44-like /// brain protein 44-like protein-like	Brp44l /// LOC100503245	0.80292	1.07209034	0.09745923
1426179_a_at	twisted gastrulation homolog 1 (Drosophila)	Twsg1	0.80292	1.13486573	0.16020673
1442443_at	---	---	0.80292	-1.08588309	-0.1193429
1447875_x_at	zinc finger protein 800	Zfp800	0.80292	-1.09865213	-0.1367047
1452709_at	polymerase (DNA-directed), delta interacting protein 3	Poldip3	0.80292	-1.06137323	-0.0869015
1417100_at	CD320 antigen	Cd320	0.80292	1.06618515	0.09219131
1424164_at	mitochondrial ribosomal protein L50	Mrpl50	0.80292	1.07190631	0.09749175
1441196_at	RIKEN cDNA C230076A16 gene	C230076A16Rik	0.80292	1.0979828	0.1327846
1435270_x_at	N-6 adenine-specific DNA methyltransferase 2 (putative)	N6amt2	0.80293	1.07143194	0.09863305
1433518_at	leucine carboxyl methyltransferase 2	Lcmt2	0.80293	1.07820926	0.10669697

1440509_at	SRY-box containing gene 30	Sox30	0.80293	-1.08233137	-0.1157513
1416762_at	S100 calcium binding protein A10 (calpactin)	S100a10	0.80293	1.08083071	0.10509641
1424785_at	angiopoietin-like 6	Angptl6	0.80293	1.07830519	0.10826507
1442141_at	cDNA sequence BC028454	BC028454	0.80293	-1.08206771	-0.1168003
1449417_at	ameloblastin	Ambn	0.80293	1.09586908	0.12911043
1433962_at	tRNA methyltransferase 61 homolog A (S. cerevisiae)	Trmt61a	0.80293	1.07657082	0.10352434
1441603_at	somatostatin receptor 3	Sstr3	0.80293	-1.07052149	-0.1001315
1425615_a_at	phosphoenolpyruvate carboxykinase 2 (mitochondrial)	Pck2	0.80293	-1.06515147	-0.0927169
1443520_at	---	---	0.80293	-1.09182083	-0.1287749
1439110_at	RIKEN cDNA A930012O16 gene /// hypothetical LOC100502840	A930012O16Rik /// LOC1	0.80294	-1.10026362	-0.1473279
1447216_at	neurexin I	Nrxn1	0.80294	1.09143013	0.1256807
1440864_at	expressed sequence AI836737	AI836737	0.80294	1.11122435	0.14145413
1436331_at	vacuolar protein sorting 13 D (yeast)	Vps13d	0.80294	1.10347907	0.13638501
1425284_a_at	RAB27A, member RAS oncogene family	Rab27a	0.80294	-1.09633098	-0.1442045
1444564_at	apolipoprotein D	Apod	0.80295	1.14470265	0.17233501
1451226_at	peroxisomal biogenesis factor 6	Pex6	0.80295	1.07438002	0.10171142
1428113_at	transmembrane and tetratricopeptide repeat containing 4	Tmtc4	0.80295	1.09325202	0.1230658
1437834_s_at	protein kinase C and casein kinase substrate in neurons 3	Pacsin3	0.80295	1.09176411	0.12458881
1435395_s_at	ATP synthase, H+ transporting, mitochondrial F0 complex, subunit	Atp5j2	0.80295	1.05767967	0.07757963
1455704_at	X Kell blood group precursor related family member 8 homolog	Xkr8	0.80295	1.08157069	0.11274494
1454866_s_at	chloride intracellular channel 6	Clic6	0.80296	1.10671297	0.142362
1457153_at	hypothetical LOC552882	LOC552882	0.80296	-1.09187347	-0.1284404
1440790_x_at	---	---	0.80296	-1.10484016	-0.1610762
1424047_at	2-deoxyribose-5-phosphate aldolase homolog (C. elegans)	Dera	0.80296	1.06246406	0.08716871
1455137_at	Rap guanine nucleotide exchange factor (GEF) 5	Rapgef5	0.80296	1.09204028	0.12119186
1456955_at	---	---	0.80297	-1.10441752	-0.1668133
1438401_at	ubinuclein 1	Ubn1	0.80297	-1.10215982	-0.1765266
1429980_x_at	solute carrier family 38, member 10	Slc38a10	0.80297	1.10045367	0.1347025
1422438_at	epoxide hydrolase 1, microsomal	Ephx1	0.80297	1.09508952	0.12380433
1427125_s_at	leucine rich repeat containing 41	Lrrc41	0.80297	1.06894373	0.09559627
1435883_at	transmembrane and coiled-coil domains 7	Tmco7	0.80297	1.08957736	0.11739922
1441222_x_at	---	---	0.80297	1.14687924	0.17394219
1418008_at	GC-rich sequence DNA-binding factor 1	Gcfc1	0.80297	1.07801455	0.10815293
1448312_at	proprotein convertase subtilisin/kexin type 2	Pcsk2	0.80298	1.11567139	0.14944185
1416392_a_at	ATPase, H+ transporting, lysosomal V0 subunit C	Atp6v0c	0.80298	1.05490124	0.07657437
1447112_s_at	crystallin, lambda 1	Cryl1	0.80298	1.09417618	0.12448948
1446669_at	---	---	0.80298	-1.08754429	-0.1240231
1422073_a_at	cadherin, EGF LAG seven-pass G-type receptor 2 (flamingo homol	Celsr2	0.80298	1.10812815	0.14251129
1427724_at	topoisomerase (DNA) II alpha	Top2a	0.80299	-1.09168137	-0.1280176
1426500_at	isoprenylcysteine carboxyl methyltransferase	Icmt	0.80299	1.08126936	0.10832721
1428268_at	pleckstrin and Sec7 domain containing 2	Psd2	0.80299	-1.10562966	-0.159233
1447308_at	LAG1 homolog, ceramide synthase 5	Lass5	0.80299	1.10895114	0.14206737
1448492_a_at	proteasome (prosome, macropain) 26S subunit, non-ATPase, 12	Psmd12	0.80299	1.05846495	0.08190088
1453002_at	SRY-box containing gene 11	Sox11	0.80299	1.10700412	0.14042997
1446912_at	---	---	0.80299	1.13407496	0.15631031
1445277_at	---	---	0.80299	1.10550846	0.13889768
1415835_at	prolactin family 3, subfamily b, member 1	Prl3b1	0.80299	1.0930663	0.12727711
1420557_at	Eph receptor A5	Epha5	0.803	-1.08684453	-0.1236128
1433680_x_at	SIVA1, apoptosis-inducing factor	Siva1	0.803	-1.09742122	-0.1563029
1454069_at	RIKEN cDNA 9030409C19 gene	9030409C19Rik	0.803	-1.08815532	-0.1236718
1443187_at	R-spondin 3 homolog (Xenopus laevis)	Rspo3	0.803	-1.08979756	-0.1267766
1431440_at	polymerase (DNA directed), epsilon 2 (p59 subunit)	Pole2	0.803	1.15605535	0.17869567
1459322_at	---	---	0.80301	1.12403339	0.1577406
1445336_at	RIKEN cDNA 1110032A03 gene	1110032A03Rik	0.80301	-1.11412461	-0.1789145
1438871_at	hypothetical LOC100503146	LOC100503146	0.80302	1.16230425	0.18568062
1448032_at	5-azacytidine induced gene 2	Azi2	0.80302	-1.09145034	-0.1294807
1459597_at	myotrophin	Mtpn	0.80302	-1.08735199	-0.1217735
1450772_at	wingless-related MMTV integration site 11	Wnt11	0.80302	-1.09599083	-0.1329715
1442339_at	stefin A2 like 1	Stfa2l1	0.80302	1.10528818	0.13795012
1435560_at	integrin alpha L	Itgal	0.80302	-1.0962069	-0.141264
1417090_at	reticulocalbin 1	Rcn1	0.80302	-1.06254862	-0.0882931
1442764_at	suppressor of variegation 4-20 homolog 1 (Drosophila)	Suv420h1	0.80302	1.09159538	0.12028437
1423509_a_at	islet amyloid polypeptide	Iapp	0.80302	-1.09419245	-0.1399561
1448879_at	ubiquitin-conjugating enzyme E2L 3	Ube2l3	0.80303	1.05765455	0.08085143
1451184_at	predicted gene 11847 /// heterogeneous nuclear ribonucleoprote	Gm11847 /// Gm6793 ///	0.80303	-1.05417331	-0.0796475
1442185_at	---	---	0.80303	1.09698322	0.13156029
1427951_s_at	coiled-coil domain containing 28A	Ccdc28a	0.80303	-1.08336737	-0.1157305
1447776_x_at	RAB6, member RAS oncogene family	Rab6	0.80303	-1.08778803	-0.1396767
1431754_at	RIKEN cDNA 4930578N16 gene	4930578N16Rik	0.80303	1.09096724	0.12299232
1457376_at	---	---	0.80303	-1.09006434	-0.1307025
1457107_at	---	---	0.80303	-1.0943502	-0.1322522
1439425_x_at	CDNA sequence BC024814	BC024814	0.80304	-1.09852249	-0.1422108
1420505_a_at	syntaxin binding protein 1	Stxbp1	0.80304	1.07880517	0.10904672
1457386_at	---	---	0.80304	-1.0919249	-0.1406627
1416321_s_at	proline arginine-rich end leucine-rich repeat	Prelp	0.80304	1.09015374	0.12307945
1418086_at	protein phosphatase 1, regulatory (inhibitor) subunit 14A	Ppp1r14a	0.80304	1.09698533	0.12617966
1430264_at	RIKEN cDNA 2610030P05 gene	2610030P05Rik	0.80304	-1.09759077	-0.1346149
1436860_at	SUMO1/sentrin specific peptidase 7	Senp7	0.80305	1.09354862	0.12842918
1458240_at	Membrane associated guanylate kinase, WW and PDZ domain co	Magi1	0.80305	1.09423257	0.12722765
1438025_at	mitochondrial translational release factor 1-like	Mtrf1l	0.80305	1.07201693	0.0993564
1423695_at	ER degradation enhancer, mannosidase alpha-like 2	Edem2	0.80305	1.08466079	0.11034643
1421192_a_at	intersectin 1 (SH3 domain protein 1A)	Itsn1	0.80306	1.13604608	0.16491469
1431193_at	TAF4B RNA polymerase II, TATA box binding protein (TBP)-associ	Taf4b	0.80306	-1.08790707	-0.1331538
1440275_at	runt related transcription factor 3	Runx3	0.80306	-1.0984356	-0.1416312
1434820_s_at	protein kinase inhibitor, gamma	Pkig	0.80306	-1.08425314	-0.1255757
1439738_at	methyltransferase like 13	Mettl13	0.80306	1.0866234	0.11938313
1420305_at	---	---	0.80306	1.08487375	0.11108414
1450357_a_at	chemokine (C-C motif) receptor 6	Ccr6	0.80307	1.1163019	0.14748588
1457037_at	zinc finger and BTB domain containing 2	Zbtb2	0.80307	1.12148705	0.15285888
1426150_at	GIPC PDZ domain containing family, member 3	Gipc3	0.80307	-1.09429289	-0.1320188
1416798_a_at	non-metastatic cells 4, protein expressed in	Nme4	0.80307	1.12453858	0.14724183
1425984_at	collagen, type V, alpha 3	Col5a3	0.80307	-1.09936591	-0.1496808
1440073_at	ribonucleotide reductase M1	Rrm1	0.80308	-1.09834289	-0.1468259
1456658_at	actin, alpha 2, smooth muscle, aorta	Acta2	0.80308	-1.10049782	-0.1399297

1439376_x_at	cyclin D binding myb-like transcription factor 1	Dmtf1	0.80308	-1.09368467	-0.1398289
1444169_at	RIKEN cDNA 3110052M02 gene	3110052M02Rik	0.80308	1.08818571	0.12158417
1457410_at	Rho GTPase activating protein 5	Arhgap5	0.80308	1.10807798	0.1434009
1428736_at	GRAM domain containing 3	Gramd3	0.80308	1.08870116	0.11910194
1455610_at	synemin, intermediate filament protein	Synm	0.80308	-1.10497586	-0.1606277
1420140_at	methylphosphate capping enzyme	Mepce	0.80309	1.08858354	0.11796209
1450674_at	cyclin-dependent kinase 5	Cdk5	0.80309	1.08243113	0.1116911
1448245_at	ribosomal protein SA	Rpsa	0.80309	1.03119275	0.04425753
1421684_at	cathepsin L-like 3	Ctsll3	0.80309	-1.09982051	-0.1445051
1456642_x_at	S100 calcium binding protein A10 (calpactin)	S100a10	0.80309	-1.07284056	-0.1098686
1416533_at	EGL nine homolog 2 (C. elegans)	Egln2	0.80309	1.06661838	0.09206291
1416053_at	leucine rich repeat protein 1, neuronal	Lrrn1	0.8031	1.11288997	0.14112265
1455040_s_at	NHS-like 2	Nhsl2	0.8031	1.09654133	0.12967939
1446637_at	predicted gene 8154	Gm8154	0.8031	1.11162105	0.14277379
1441156_at	COP9 (constitutive photomorphogenic) homolog, subunit 3 (Arabi	Cops3	0.8031	-1.09890186	-0.1420632
1418654_at	hydroxyacid oxidase 2	Hao2	0.8031	-1.07125304	-0.1014933
1430129_a_at	COMM domain containing 8	Commd8	0.8031	-1.06840693	-0.0960231
1417340_at	glutaredoxin 3	Glrx3	0.8031	-1.08021161	-0.127739
1424720_at	mannoside acetylglucosaminyltransferase 4, isoenzyme B	Mgat4b	0.8031	1.06155658	0.08610023
1452272_a_at	growth factor, erv1 (S. cerevisiae)-like (augmenter of liver regene	Gfer	0.80311	1.09128306	0.11603408
1422614_s_at	biogenesis of lysosome-related organelles complex-1, subunit 1 /	Bloc1s1 /// Rdh5	0.80312	1.09734944	0.12438848
1452287_at	Musashi homolog 1(Drosophila)	Msi1	0.80312	-1.10384549	-0.172903
1426614_at	zinc finger, MYND-type containing 8	Zmynd8	0.80313	1.08950223	0.11537928
1449509_at	small EDRK-rich factor 1	Serf1	0.80313	1.10412854	0.13213085
1433130_at	RIKEN cDNA 8430418B16 gene	8430418B16Rik	0.80313	-1.11392782	-0.1758492
1439932_at	UBX domain protein 2B	Ubxn2b	0.80313	-1.11710391	-0.2118876
1435755_at	RIKEN cDNA 1110001A16 gene	1110001A16Rik	0.80313	1.08238821	0.10888661
1434999_at	suppressor of variegation 4-20 homolog 1 (Drosophila)	Suv420h1	0.80313	1.09294872	0.12485045
1418693_at	heterogeneous nuclear ribonucleoprotein C	Hnrnpc	0.80313	-1.06348146	-0.0900364
1416132_at	EFR3 homolog A (S. cerevisiae)	Efr3a	0.80314	-1.10152732	-0.1679224
1445259_at	sodium channel, voltage-gated, type II, alpha 1	Scn2a1	0.80314	-1.09268784	-0.1311666
1421441_at	angiopoietin 1	Angpt1	0.80315	-1.09379855	-0.1305973
1459187_at	RIKEN cDNA 9330161A08 gene	9330161A08Rik	0.80315	1.11774253	0.1508412
1441856_x_at	trypsin domain containing 1	Tysnd1	0.80315	1.09048039	0.12180027
1458641_at	Braf transforming gene	Braf	0.80315	-1.08578065	-0.1217719
1441871_at	RIKEN cDNA 1810044D09 gene	1810044D09Rik	0.80315	-1.07763529	-0.1122424
1454700_at	leucine rich repeat and fibronectin type III domain containing 4	Lrfn4	0.80315	1.09416081	0.12515479
1448018_at	---	---	0.80315	-1.09581849	-0.1386741
1447047_at	---	---	0.80315	-1.09987212	-0.1499644
1449518_at	glutaminyl-peptide cyclotransferase-like	Qpctl	0.80315	1.1209072	0.15051854
1450247_a_at	secretory carrier membrane protein 5	Scamp5	0.80315	-1.07407499	-0.1037166
1420645_at	polycomb group ring finger 2	Pcgf2	0.80315	-1.09112875	-0.1494653
1439273_at	receptor (TNFRSF)-interacting serine-threonine kinase 1	Ripk1	0.80316	1.08803024	0.11891102
1448657_a_at	DnaJ (Hsp40) homolog, subfamily B, member 2	Dnajb2	0.80316	1.0862629	0.11836814
1436182_at	special AT-rich sequence binding protein 1	Satb1	0.80316	1.1167169	0.14830709
1435251_at	sorting nexin 13	Snx13	0.80316	-1.08464126	-0.117569
1453426_a_at	WD repeat and FYVE domain containing 1	Wdfy1	0.80316	1.09638728	0.12855291
AFFX-MUR_b2_a	---	---	0.80316	-1.07150742	-0.1035612
1456668_at	zinc finger, CCHC domain containing 11	Zcchc11	0.80316	1.09397072	0.12946052
1451913_a_at	hypoxia up-regulated 1	Hyou1	0.80316	1.08473016	0.11707537
1418687_at	activity regulated cytoskeletal-associated protein	Arc	0.80317	1.09788634	0.12290054
1423377_at	insulin-like growth factor binding protein-like 1	Igfbpl1	0.80317	1.07725447	0.10634342
1437813_at	absent in melanoma 1-like	Aim1l	0.80317	1.08482874	0.11513906
1446514_at	---	---	0.80317	-1.0890041	-0.1275274
1449468_at	ST6 (alpha-N-acetyl-neuraminyl-2,3-beta-galactosyl-1,3)-N-acety	St6galnac5	0.80318	1.11811153	0.14961547
1459135_at	---	---	0.80318	-1.0970027	-0.1379921
1452235_at	mannosidase, alpha, class 1B, member 1	Man1b1	0.80318	1.09141574	0.11731017
1418675_at	oncostatin M receptor	Osmr	0.80318	-1.10547689	-0.1604511
1433216_at	RIKEN cDNA 9430024C03 gene	9430024C03Rik	0.80319	-1.10066363	-0.1562316
1439027_at	N(alpha)-acetyltransferase 25, NatB auxiliary subunit	Naa25	0.80319	-1.06511412	-0.092838
1428308_at	p53 and DNA damage regulated 1	Pdrg1	0.80319	1.07670955	0.10473967
1423477_at	zinc finger protein of the cerebellum 1	Zic1	0.80319	-1.0688314	-0.1003436
1453622_s_at	myeloid/lymphoid or mixed-lineage leukemia (trithorax homolog,	Mllt3	0.80319	1.11150008	0.1458933
1427353_at	CLIP associating protein 1	Clasp1	0.80319	-1.10490585	-0.1530996
1420496_at	coagulation factor XII (Hageman factor)	F12	0.80319	-1.09112154	-0.128681
1426847_at	sirtuin 4 (silent mating type information regulation 2 homolog) 4 (	Sirt4	0.80319	1.08617646	0.11548204
1419695_at	ST8 alpha-N-acetyl-neuraminide alpha-2,8-sialyltransferase 1	St8sia1	0.8032	1.10401855	0.13942915
1453844_at	chitinase 1 (chitotriosidase)	Chit1	0.8032	1.10169914	0.13481633
1423404_at	gastrokine 1	Gkn1	0.8032	1.08628986	0.11804051
1427339_at	solute carrier family 30 (zinc transporter), member 2	Slc30a2	0.8032	1.09916412	0.12830431
1418326_at	solute carrier family 7 (cationic amino acid transporter, y+ system	Slc7a5	0.8032	-1.06234908	-0.0881008
1448641_at	mbt domain containing 1	Mbtd1	0.8032	1.06067487	0.08458132
1458891_at	---	---	0.8032	-1.09460747	-0.1320081
1450537_at	midline 2	Mid2	0.8032	-1.07879286	-0.1094486
1427698_at	breast cancer 1	Brca1	0.80321	-1.09732417	-0.1379918
1440811_x_at	CD8 antigen, alpha chain	Cd8a	0.80321	-1.09395714	-0.140375
1449614_s_at	expressed sequence AI314976	AI314976	0.80321	1.06781214	0.09317711
1438897_at	zinc finger, DHHC domain containing 3	Zdhhc3	0.80321	1.10932102	0.14242891
1446911_at	---	---	0.80321	1.10907078	0.13954167
1431982_at	Rho GTPase activating protein 19	Arhgap19	0.80322	1.12507661	0.15315065
1449486_at	carboxylesterase 1G	Ces1g	0.80322	1.0870085	0.11949964
1442264_at	RAS, guanyl releasing protein 2	Rasgrp2	0.80322	1.12119584	0.15101794
1425992_at	solute carrier family 6 (neurotransmitter transporter, glycine), me	Slc6a5	0.80322	-1.10198678	-0.1461795
1459786_at	---	---	0.80322	1.09299282	0.12445564
1420353_at	lymphotoxin A	Lta	0.80322	1.09785607	0.13242785
1418380_at	telomeric repeat binding factor 1	Terf1	0.80322	-1.07908647	-0.1182624
1443015_at	Ras homolog gene family, member H	Rhoh	0.80322	1.08451598	0.11554918
1436981_a_at	hypothetical LOC100505062 /// tyrosine 3-monooxygenase/tryp	LOC100505062 /// Ywhaz	0.80322	-1.11578498	-0.2703244
1431501_at	RIKEN cDNA 1700037C18 gene	1700037C18Rik	0.80323	1.08954389	0.12200646
1444768_at	---	---	0.80323	-1.09299875	-0.1283071
1442894_at	dynein, axonemal, heavy chain 6	Dnahc6	0.80323	-1.09617547	-0.1374221
1456867_x_at	ERGIC and golgi 3	Ergic3	0.80323	1.06805309	0.09490203
1424176_a_at	annexin A4	Anxa4	0.80324	-1.07785506	-0.1084576
1438001_x_at	receptor accessory protein 5	Reep5	0.80324	1.09219572	0.11686244

1431973_at	septin 6	Sep-06	0.80324	1.11485579	0.14522756
1435414_s_at	dynactin 1 /// hypothetical LOC100503586	Dctn1 /// LOC100503586	0.80324	1.07545694	0.10043846
1425894_at	MAS-related GPR, member F	Mrgprf	0.80324	-1.09509646	-0.1321733
1418056_at	IQ motif containing F3	Iqcf3	0.80324	1.09614846	0.13049766
1446247_at	a disintegrin-like and metallopeptidase (reprolysin type) with thro	Adamts18	0.80324	1.15415087	0.17645052
1424150_at	glycerophosphodiester phosphodiesterase domain containing 5	Gdpd5	0.80324	1.08738781	0.11658335
1453242_x_at	RIKEN cDNA 2810047C21 gene 1	2810047C21Rik1	0.80324	-1.09340442	-0.1292259
1447089_at	ADP-ribosylation factor-like 1	Arl1	0.80324	1.09407947	0.12957294
1427557_at	asparagine-linked glycosylation 12 homolog (yeast, alpha-1,6-ma	Alg12	0.80325	1.08451609	0.11686957
1450291_s_at	membrane-spanning 4-domains, subfamily A, member 4C	Ms4a4c	0.80325	1.11392255	0.14042252
1432641_at	RIKEN cDNA 5830490A12 gene	5830490A12Rik	0.80325	1.09367346	0.12631954
1424279_at	fibrinogen alpha chain	Fga	0.80325	1.09605973	0.12593166
1434523_x_at	eukaryotic translation initiation factor 3, subunit E	Eif3e	0.80325	-1.05174095	-0.0728729
1441385_at	ras homolog gene family, member H	Rhoh	0.80325	1.09814688	0.13303413
1417045_at	BH3 interacting domain death agonist	Bid	0.80325	1.07255225	0.09991695
1429456_a_at	polymerase (RNA) III (DNA directed) polypeptide E	Polr3e	0.80325	1.07968872	0.10616642
1422737_at	nuclear receptor coactivator 3	Ncoa3	0.80325	-1.07460986	-0.1100181
1445286_at	vomeronasal 2, receptor 29	Vmn2r29	0.80325	-1.07417174	-0.1060166
1427014_at	DENN/MADD domain containing 4B	Dennd4b	0.80326	1.08177953	0.1106856
1433976_at	Receptor accessory protein 3	Reep3	0.80326	-1.09313603	-0.1360872
1455296_at	adenylate cyclase 5	Adcy5	0.80326	1.13722078	0.16567045
1431320_a_at	myosin VA	Myo5a	0.80326	-1.08212159	-0.1139372
1426923_at	ArfGAP with FG repeats 1	Agfg1	0.80326	-1.06266232	-0.0900005
1455116_at	RELT tumor necrosis factor receptor	Relt	0.80327	1.07641162	0.10530955
1437584_at	---	---	0.80327	1.09078787	0.12534364
1439541_at	RIKEN cDNA 4930414L22 gene	4930414L22Rik	0.80327	1.10793287	0.14048178
1445620_at	---	---	0.80327	-1.10899726	-0.1669353
1434513_at	ATPase type 13A3	Atp13a3	0.80327	-1.07793329	-0.1174138
1445537_at	RIKEN cDNA 2310039F13 gene	2310039F13Rik	0.80327	-1.0969027	-0.1369113
1444611_at	---	---	0.80328	-1.09458845	-0.1438681
1417567_at	catenin beta interacting protein 1	Ctnnbip1	0.80328	1.11447242	0.14317014
1422501_s_at	isocitrate dehydrogenase 3 (NAD+) alpha	Idh3a	0.80328	1.07510797	0.10245702
1424703_at	HemK methyltransferase family member 1	Hemk1	0.80328	1.07945278	0.10908758
1417848_at	zinc finger protein 704	Zfp704	0.80328	-1.08880406	-0.1282918
1438169_a_at	FERM domain containing 4B	Frmd4b	0.80328	1.12107669	0.14583625
1448626_at	CDK5 regulatory subunit associated protein 1	Cdk5rap1	0.80328	1.06888281	0.09398986
1446622_at	RIKEN cDNA A330068G13 gene	A330068G13Rik	0.80329	-1.08549706	-0.1207236
1420915_at	signal transducer and activator of transcription 1	Stat1	0.80329	1.13730588	0.16753677
1451066_at	membrane bound O-acyltransferase domain containing 7	Mboat7	0.80329	1.06871478	0.09468504
1423991_at	NOP14 nucleolar protein homolog (yeast)	Nop14	0.80329	1.07430554	0.10147263
1428723_at	RIKEN cDNA 2310047M10 gene	2310047M10Rik	0.80329	1.08731222	0.11855487
1442447_at	---	---	0.80329	-1.09306023	-0.1284739
1433999_at	STE20-like kinase (yeast)	Slk	0.80329	-1.07208686	-0.1009777
1436202_at	metastasis associated lung adenocarcinoma transcript 1 (non-cod	Malat1	0.80329	1.08053274	0.11060147
1459715_at	---	---	0.8033	-1.0997871	-0.1414179
1426723_at	WD repeat domain 48	Wdr48	0.8033	1.06078031	0.08505866
1452337_at	RIKEN cDNA 4930427A07 gene	4930427A07Rik	0.8033	-1.09224495	-0.1366752
1434042_s_at	myotubularin related protein 3	Mtmr3	0.8033	1.06819558	0.09293542
1442896_at	Heat shock protein 12A	Hspa12a	0.8033	-1.10037125	-0.1397981
1439661_at	solute carrier family 16 (monocarboxylic acid transporters), memb	Slc16a14	0.8033	-1.09796904	-0.1410803
1459707_at	---	---	0.80331	1.12133587	0.15342254
1451278_a_at	methyltransferase like 11A	Mettl11a	0.80331	1.07109097	0.09659153
1436745_at	golgin, RAB6-interacting	Gorab	0.80331	-1.07964771	-0.1127412
1455517_at	RNA binding motif protein 4	Rbm4	0.80331	1.08638561	0.11914261
1425298_a_at	NLR family, apoptosis inhibitory protein 1	Naip1	0.80331	-1.09221325	-0.1313003
AFFX-BioDn-5_at	---	---	0.80331	1.08437758	0.10929703
1442926_at	RIKEN cDNA 1700011B04 gene /// hypothetical LOC100503727	1700011B04Rik /// LOC10	0.80331	1.0875009	0.11830486
1420745_a_at	cyclin D-type binding-protein 1	Ccndbp1	0.80331	1.07234723	0.10040451
1448476_at	nucleosome assembly protein 1-like 4	Nap1l4	0.80331	1.066447	0.09175101
1446561_at	paired box gene 8	Pax8	0.80332	-1.09675337	-0.1410065
1417297_at	inositol 1,4,5-triphosphate receptor 3	Itpr3	0.80332	1.06710145	0.09283675
1445320_at	UBX domain protein 7	Ubxn7	0.80332	1.10447064	0.13827561
1433868_at	BTB (POZ) domain containing 3	Btbd3	0.80332	1.07244611	0.10077497
1429263_at	RIKEN cDNA 4933425D22 gene	4933425D22Rik	0.80332	1.15585314	0.17867016
1417857_at	methylmalonic aciduria (cobalamin deficiency) type A	Mmaa	0.80332	1.10508298	0.13501234
1424627_at	cystatin 12	Cst12	0.80333	-1.09727728	-0.1528567
1442723_at	---	---	0.80333	1.10689584	0.13995864
1437825_at	leucine rich repeat containing 4C	Lrrc4c	0.80333	1.0762883	0.10561345
1435711_at	hypothetical LOC100503186	LOC100503186	0.80333	1.10056587	0.13204055
1457867_at	sphingosine-1-phosphate phosphotase 2	Sgpp2	0.80333	-1.09639689	-0.141383
1454940_at	predicted gene 8394 /// proteasome (prosome, macropain) subu	Gm8394 /// Psma5	0.80334	1.09603286	0.1306189
1454723_at	family with sequence similarity 172, member A	Fam172a	0.80334	-1.09641311	-0.1486026
1450959_at	RIKEN cDNA D930014E17 gene	D930014E17Rik	0.80335	1.08809	0.11595171
1422011_s_at	RIKEN cDNA 3830403N18 gene /// X-linked lymphocyte-regulate	3830403N18Rik /// Xlr	0.80335	1.06043404	0.08345183
1439588_at	solute carrier organic anion transporter family, member 5A1	Slco5a1	0.80335	1.101977	0.13638805
1435555_at	hypothetical LOC100503933 /// POU domain, class 2, transcriptio	LOC100503933 /// Pou2f1	0.80335	-1.07028172	-0.09842
1416032_at	transmembrane protein 109	Tmem109	0.80335	1.1521029	0.17139662
1423620_at	centromere protein Q	Cenpq	0.80335	-1.06425748	-0.0920274
1437609_at	ubiquitin-conjugating enzyme E2U (putative)	Ube2u	0.80335	1.09149886	0.12622485
1427415_at	F-box protein 43	Fbxo43	0.80336	1.0966692	0.13010809
1450745_at	core 1 synthase, glycoprotein-N-acetylgalactosamine 3-beta-gala	C1galt1	0.80336	1.0901762	0.11946301
1446763_at	---	---	0.80336	1.08872794	0.12078545
1427636_at	testis specific protein-Y encoded, pseudogene	Tspy-ps	0.80336	-1.09862215	-0.1413119
1445337_at	DnaJ (Hsp40) homolog, subfamily C, member 13	Dnajc13	0.80336	-1.09223158	-0.1302022
1458090_at	family with sequence similarity 63, member A	Fam63a	0.80336	-1.09306196	-0.1392098
1445658_at	RIKEN cDNA 2410003L11 gene	2410003L11Rik	0.80336	-1.0851715	-0.1203631
1451088_a_at	oxidase assembly 1-like	Oxa1l	0.80336	1.06565132	0.09061794
1440673_at	---	---	0.80336	1.09094978	0.12192492
1424053_a_at	transcription factor 25 (basic helix-loop-helix)	Tcf25	0.80336	1.06287932	0.08766177
1445291_at	---	---	0.80337	-1.10341666	-0.1493436
1431495_at	RIKEN cDNA 6030440G07 gene	6030440G07Rik	0.80337	1.11636629	0.1469852
1429165_at	RIKEN cDNA 3110001I22 gene	3110001I22Rik	0.80337	1.0822728	0.11019562
1424875_at	spastic paraplegia 20, spartin (Troyer syndrome) homolog (huma	Spg20	0.80337	1.1140525	0.14773074
1431412_at	RIKEN cDNA 2810455B08 gene	2810455B08Rik	0.80337	-1.09482789	-0.1336767

1421520_at	junctophilin 1	Jph1	0.80338	-1.08980052	-0.1250052
1425521_at	polyadenylate binding protein-interacting protein 1	Paip1	0.80338	-1.07986758	-0.1176265
1427526_at	FGFR1 oncogene partner 2	Fgfr1op2	0.80338	-1.10768833	-0.1705673
1416499_a_at	dynactin 6	Dctn6	0.80338	-1.08223964	-0.1225512
1452579_at	IscU iron-sulfur cluster scaffold homolog (E. coli)	Iscu	0.80338	1.07165405	0.09653543
1419164_at	zinc finger protein 260	Zfp260	0.80338	-1.08789079	-0.1334084
1421478_a_at	zinc finger protein 318	Zfp318	0.80339	-1.09464979	-0.1446734
1456662_at	expressed sequence AA386476	AA386476	0.80339	1.17611409	0.19381359
1423353_at	cysteine-rich secretory protein LCCL domain containing 1	Crispld1	0.80339	-1.09316313	-0.1344026
1443038_at	---	---	0.80339	-1.10769206	-0.1596993
1435742_at	SMEK homolog 1, suppressor of mek1 (Dictyostelium)	Smek1	0.80339	1.06935901	0.09474449
1443610_at	---	---	0.80339	-1.08413845	-0.1177008
1443032_at	---	---	0.80339	-1.09184005	-0.1279038
1444201_at	centrosomal protein 135	Cep135	0.8034	1.08961687	0.1224807
1460029_at	---	---	0.8034	-1.09683818	-0.1382865
1439292_at	---	---	0.8034	1.09343423	0.12760614
1456408_x_at	RIKEN cDNA 4933439C10 gene	4933439C10Rik	0.8034	1.12187359	0.15254544
1450477_at	5-hydroxytryptamine (serotonin) receptor 2C	Htr2c	0.8034	-1.10877221	-0.1699256
1425788_a_at	enoyl Coenzyme A hydratase domain containing 2	Echdc2	0.8034	1.07417887	0.10266462
1453157_at	glycerol-3-phosphate dehydrogenase 1-like pseudogene	1700022A21Rik	0.80341	1.11830238	0.15056439
1453303_at	RIKEN cDNA 4833417J20 gene	4833417J20Rik	0.80341	1.09301789	0.12775765
1422031_a_at	zinc finger, AN1-type domain 6	Zfand6	0.80341	1.07251766	0.09793033
1429959_at	---	---	0.80342	-1.0911786	-0.1301486
1417510_at	vacuolar protein sorting 4a (yeast)	Vps4a	0.80342	1.06765047	0.09418343
1456292_a_at	vimentin	Vim	0.80342	-1.07206443	-0.1098911
1416547_at	NADH dehydrogenase (ubiquinone) 1 beta subcomplex 3	Ndufb3	0.80342	1.07284517	0.09747143
1423281_at	stathmin-like 2	Stmn2	0.80342	1.06564927	0.08807232
1425186_at	LMBR1 domain containing 1	Lmbrd1	0.80342	-1.09421836	-0.1313529
1442817_at	RIKEN cDNA A730091E23 gene	A730091E23Rik	0.80342	-1.08122836	-0.1139275
1459411_at	---	---	0.80343	-1.10530819	-0.1641228
1453334_at	RIKEN cDNA B230216N24 gene	B230216N24Rik	0.80343	1.09744948	0.13247136
1442244_at	InaD-like (Drosophila)	Inadl	0.80343	-1.07839163	-0.1121498
1417249_at	polymerase (DNA directed), mu	Polm	0.80343	1.08346904	0.11370085
1460664_at	IKAROS family zinc finger 4	Ikzf4	0.80343	1.09793784	0.13335952
1456161_at	RIKEN cDNA 0610040B10 gene	0610040B10Rik	0.80344	1.11477039	0.14905537
1415951_at	FK506 binding protein 10	Fkbp10	0.80344	-1.08705166	-0.1274023
1453092_at	cysteine-rich C-terminal 1	Crct1	0.80345	1.13619683	0.16302225
1445636_at	---	---	0.80345	-1.0957132	-0.1407402
1457330_at	---	---	0.80345	1.15385522	0.17802843
1422844_a_at	WD repeat domain 77	Wdr77	0.80345	-1.06645087	-0.1000047
1435549_at	transient receptor potential cation channel, subfamily M, membe	Trpm4	0.80345	1.07833739	0.10819136
1423271_at	gap junction protein, beta 2	Gjb2	0.80345	-1.09679277	-0.1334535
1449756_at	DnaJ (Hsp40) homolog, subfamily C, member 17	Dnajc17	0.80346	-1.10236767	-0.1551307
1444622_at	---	---	0.80347	-1.08999653	-0.1258102
1432243_a_at	RIKEN cDNA 4933433G15 gene	4933433G15Rik	0.80347	-1.09408477	-0.1316287
1446288_at	expressed sequence C78692	C78692	0.80347	1.08762426	0.12032305
1422419_s_at	transition protein 2	Tnp2	0.80347	1.09923127	0.13138989
1455077_a_at	RIKEN cDNA A730098P11 gene /// mortality factor 4 like 1	A730098P11Rik /// Morf4	0.80347	-1.05025542	-0.0738862
1419533_at	nescient helix loop helix 1	Nhlh1	0.80348	1.11616968	0.1501183
1424550_at	zinc finger, FYVE domain containing 27	Zfyve27	0.80349	1.08021795	0.11080494
1458994_at	---	---	0.80349	1.13459573	0.16459715
1454315_at	RIKEN cDNA 3300002A11 gene	3300002A11Rik	0.80349	-1.09163297	-0.1281189
1459625_at	---	---	0.80349	-1.09445861	-0.1312776
1446303_at	insulin-like growth factor I receptor	Igf1r	0.8035	-1.09788146	-0.1377019
1458467_at	keratin associated protein 22-2	Krtap22-2	0.8035	-1.09166945	-0.1304887
1445667_at	TBC1 domain family, member 10a	Tbc1d10a	0.8035	-1.10843259	-0.1649587
1441037_at	---	---	0.80351	1.09872676	0.13492455
1452482_at	v-erb-b2 erythroblastic leukemia viral oncogene homolog 3 (avia	Erbb3	0.80351	-1.10826992	-0.1626391
1442572_at	---	---	0.80351	1.08125638	0.11195988
1443954_at	RAD18 homolog (S. cerevisiae)	Rad18	0.80351	1.13611562	0.16223302
1423415_at	G protein-coupled receptor 83	Gpr83	0.80352	-1.10126918	-0.1430699
1427732_s_at	ATP-binding cassette, sub-family G (WHITE), member 4	Abcg4	0.80352	-1.09008584	-0.1279199
1416979_at	proteasome maturation protein	Pomp	0.80352	1.06130154	0.08454906
1426809_at	taperin	Tprn	0.80353	1.08099218	0.11028644
1425146_at	RIKEN cDNA 2410075B13 gene	2410075B13Rik	0.80353	1.10601365	0.13453856
1417056_at	proteasome (prosome, macropain) 28 subunit, alpha	Psme1	0.80354	1.06977733	0.09449326
1448620_at	Fc receptor, IgG, low affinity III	Fcgr3	0.80354	1.13038419	0.16343462
1457409_at	fucosyltransferase 9	Fut9	0.80354	1.14450842	0.17161756
1442403_at	RIKEN cDNA B130034C11 gene	B130034C11Rik	0.80355	1.12694761	0.15387376
1433180_at	MORN repeat containing 1	Morn1	0.80355	1.12358005	0.15486307
1437768_at	ankyrin repeat and IBR domain containing 1	Ankib1	0.80356	-1.10190119	-0.153567
1442754_at	RIKEN cDNA C030013G03 gene	C030013G03Rik	0.80356	1.11553205	0.14890277
1417418_s_at	cytochrome c oxidase, subunit VI a, polypeptide 1	Cox6a1	0.80357	1.07884942	0.10101969
1444400_at	retinoblastoma 1	Rb1	0.80357	-1.10522384	-0.1608785
1431414_at	RIKEN cDNA 1700003G18 gene	1700003G18Rik	0.80357	-1.09434777	-0.1363268
1435806_at	UTP23, small subunit (SSU) processome component, homolog (ye	Utp23	0.80357	-1.07268697	-0.1012763
AFFX-PheX-5_at	---	---	0.80357	1.15941262	0.18245259
1417102_a_at	NADH dehydrogenase (ubiquinone) 1 beta subcomplex, 5	Ndufb5	0.80358	-1.06466391	-0.0941029
1453872_at	doublesex and mab-3 related transcription factor like family C2	Dmrtc2	0.80358	1.09969717	0.13520527
1420586_at	zinc finger protein 493	Zfp493	0.80359	1.10240136	0.12954183
1432443_at	family with sequence similarity 71, member E1	Fam71e1	0.80359	1.09034576	0.124754
1438630_x_at	methionine adenosyltransferase II, alpha	Mat2a	0.80359	-1.06213739	-0.0874193
1425425_a_at	Wnt inhibitory factor 1	Wif1	0.8036	1.07908792	0.10561072
1430587_at	neuroblastoma amplified sequence	Nbas	0.8036	-1.08859459	-0.1229183
1459253_at	RIKEN cDNA 1700023H06 gene	1700023H06Rik	0.8036	1.25372616	0.24441122
1418069_at	apolipoprotein C-II	Apoc2	0.80361	-1.09155695	-0.1264364
1419329_at	sorbin and SH3 domain containing 3	Sorbs3	0.80361	-1.08352138	-0.1194703
1433633_at	interferon regulatory factor 2 binding protein 2	Irf2bp2	0.80362	1.12005838	0.15192891
1443489_at	---	---	0.80362	1.14312009	0.16988045
1440013_at	---	---	0.80362	-1.10119851	-0.1515186
1420681_at	RIKEN cDNA 1700120K04 gene	1700120K04Rik	0.80363	-1.10359025	-0.1603451
1452796_at	differentially expressed in FDCP 6	Def6	0.80363	1.07476685	0.10240696
1455081_at	thioredoxin-like 4B	Txnl4b	0.80364	-1.08351814	-0.1157417
1444765_at	RNA binding protein gene with multiple splicing	Rbpms	0.80364	1.13768039	0.16453616

1431010_a_at	retinol dehydrogenase 12	Rdh12	0.80365	1.09935232	0.1335261
1451958_at	cDNA sequence AB069917 /// predicted gene, 16710 /// immuno	AB069917 /// Gm16710 /	0.80365	-1.08905887	-0.1269523
1422447_at	insulin I	Ins1	0.80365	-1.08330655	-0.1160659
1438167_x_at	folliculin	Flcn	0.80366	-1.08494201	-0.1299327
1442983_at	---	---	0.80366	-1.09157491	-0.1277619
1438851_x_at	claudin 1	Cldn1	0.80366	1.08969694	0.11732582
1455359_at	protein tyrosine phosphatase, non-receptor type 14	Ptpn14	0.80366	-1.0819726	-0.1179056
1456305_x_at	oocyte specific homeobox 2	Obox2	0.80367	1.09890324	0.13477653
1433964_s_at	fermitin family homolog 3 (Drosophila)	Fermt3	0.80367	1.12880349	0.15794636
1422991_at	phosphatidylinositol 3-kinase catalytic delta polypeptide	Pik3cd	0.80368	-1.09138016	-0.1298024
1447673_x_at	RIKEN cDNA 1700015C15 gene	1700015C15Rik	0.80369	-1.095395	-0.1434684
1421877_at	mitogen-activated protein kinase 9	Mapk9	0.80369	1.06581194	0.09154089
1421301_at	zinc finger protein of the cerebellum 2	Zic2	0.80369	1.1502494	0.17685425
1456697_x_at	cyclin D binding myb-like transcription factor 1	Dmtf1	0.80369	-1.10000585	-0.1428265
1460528_at	RIKEN cDNA 4930413G21 gene	4930413G21Rik	0.8037	1.15193943	0.17494652
1448994_at	trans-acting transcription factor 1	Sp1	0.80371	-1.08438132	-0.1273505
1426001_at	eomesodermin homolog (Xenopus laevis)	Eomes	0.80371	1.11867702	0.14847749
1451429_at	glucose-fructose oxidoreductase domain containing 2	Gfod2	0.80372	1.08372979	0.11415527
1446490_at	---	---	0.80373	-1.06739901	-0.097297
1427460_at	TAF4A RNA polymerase II, TATA box binding protein (TBP)-associ	Taf4a	0.80373	1.07755356	0.10583328
1458281_at	---	---	0.80374	-1.10144013	-0.1446316
1420401_a_at	receptor (calcitonin) activity modifying protein 3	Ramp3	0.80374	1.07923165	0.1076627
1433606_at	DCP1 decapping enzyme homolog A (S. cerevisiae)	Dcp1a	0.80374	1.07772122	0.10636281
1457809_at	K(lysine) acetyltransferase 2B	Kat2b	0.80375	1.12944739	0.16034103
1430501_at	RIKEN cDNA C030047K22 gene	C030047K22Rik	0.80375	1.11907856	0.1483693
1432116_at	RIKEN cDNA 1700031F05 gene	1700031F05Rik	0.80375	-1.09656251	-0.1339277
1424411_at	transmembrane protein 189	Tmem189	0.80377	1.07870753	0.10773597
1444618_at	---	---	0.80377	-1.10549708	-0.155377
1458107_at	---	---	0.80379	-1.10178887	-0.1440041
1423062_at	insulin-like growth factor binding protein 3	Igfbp3	0.80379	1.11530845	0.14517892
1418986_a_at	ubiquitously expressed transcript	Uxt	0.80379	1.10440847	0.12889549
1419668_at	sarcoglycan, beta (dystrophin-associated glycoprotein)	Sgcb	0.8038	1.1254844	0.1570424
1429140_at	spinster homolog 3 (Drosophila)	Spns3	0.80381	-1.07968222	-0.1130213
1455155_at	LSM14 homolog B (SCD6, S. cerevisiae)	Lsm14b	0.80381	1.09356246	0.11912898
1454878_at	DAZ interacting protein 3, zinc finger	Dzip3	0.80381	-1.08484277	-0.1324978
1438429_at	RIKEN cDNA 2610319H10 gene	2610319H10Rik	0.80382	1.10387813	0.12839479
1448301_s_at	serine (or cysteine) peptidase inhibitor, clade B, member 1a	Serpinb1a	0.80382	1.14612022	0.17160779
1433255_at	RIKEN cDNA 8430437B07 gene	8430437B07Rik	0.80382	-1.08407752	-0.1169947
1431215_at	DnaJ (Hsp40) homolog, subfamily C, member 6	Dnajc6	0.80383	1.09618785	0.12919385
1448324_at	ribonucleic acid binding protein S1	Rnps1	0.80383	1.06023251	0.08337155
1418611_at	G protein-coupled receptor 162	Gpr162	0.80384	1.08256968	0.1126292
1419352_at	lethal, Chr 7, Rinchik 6	l7Rn6	0.80384	1.05960355	0.08343619
1453187_at	OCIA domain containing 2	Ociad2	0.80385	-1.08864249	-0.1262051
1456392_at	neuronal growth regulator 1	Negr1	0.80385	1.08312089	0.1116957
1430366_at	RIKEN cDNA 5430405H02 gene	5430405H02Rik	0.80385	1.1008069	0.13441463
1459790_x_at	aristaless-like homeobox 3	Alx3	0.80385	-1.09207436	-0.1294599
1427899_at	ring finger protein (C3H2C3 type) 6	Rnf6	0.80385	-1.09732712	-0.1525111
1425278_at	ubiquitination factor E4A, UFD2 homolog (S. cerevisiae)	Ube4a	0.80388	1.09073505	0.12430748
1430230_at	RCSD domain containing 1	Rcsd1	0.80388	-1.09998724	-0.1410947
1443441_x_at	predicted gene 5141	Gm5141	0.80388	-1.08438844	-0.1180041
1424656_s_at	ubiquitin specific peptidase 19	Usp19	0.80389	1.08402751	0.11006203
1436956_at	---	---	0.80389	1.08675129	0.11964901
1458325_x_at	BCL2 modifying factor	Bmf	0.80389	1.09492514	0.12709762
1417806_at	popeye domain containing 2	Popdc2	0.80392	1.09971307	0.13326776
1445935_at	---	---	0.80392	-1.09414651	-0.1311292
1435098_at	---	---	0.80392	1.14652516	0.17395399
1451456_at	Holliday junction recognition protein	Hjurp	0.80393	-1.09460745	-0.135581
1444939_at	---	---	0.80393	-1.09738701	-0.1357858
1437221_at	ribonucleotide reductase M2 B (TP53 inducible)	Rrm2b	0.80394	1.07251018	0.10059247
1443066_at	---	---	0.80394	-1.09322885	-0.1364297
1446679_at	---	---	0.80397	-1.07499615	-0.1065375
1433321_at	RIKEN cDNA 9430087B13 gene	9430087B13Rik	0.80398	-1.09555981	-0.1373479
1419961_s_at	Cytoplasmic polyadenylated homeobox	Cphx	0.80398	-1.10078992	-0.1468745
1431623_at	RIKEN cDNA 1700023H06 gene	1700023H06Rik	0.80398	-1.10108953	-0.1506067
1424721_at	microfibrillar-associated protein 3	Mfap3	0.80399	1.08314327	0.10781964
1415910_s_at	cytokine induced apoptosis inhibitor 1	Ciapin1	0.80399	1.06688505	0.09181357
1425706_a_at	damage specific DNA binding protein 2	Ddb2	0.80399	1.08494119	0.11740865
1425164_a_at	phosphorylase kinase gamma 1	Phkg1	0.80401	-1.09072541	-0.1267536
1453975_a_at	RIKEN cDNA 1700029M20 gene	1700029M20Rik	0.80401	1.09854776	0.13278889
1437349_at	cytoskeleton associated protein 5	Ckap5	0.80403	1.06851029	0.09308126
1428703_at	RIKEN cDNA 1700012B07 gene	1700012B07Rik	0.80405	-1.10124406	-0.1433697
1436046_x_at	predicted gene 10709 /// predicted gene 3550 /// ribosomal prot	Gm10709 /// Gm3550 ///	0.80405	1.03941916	0.05528309
1444399_at	---	---	0.80406	1.09228547	0.12330774
1416873_a_at	cyclin-dependent kinase 2	Cdk2	0.80407	1.06436411	0.08971084
1436498_at	ariadne ubiquitin-conjugating enzyme E2 binding protein homolog	Arih1	0.80409	1.07337655	0.09922181
1445554_at	---	---	0.80411	1.08266428	0.11113215
1428655_at	KLRAQ motif containing 1	Klraq1	0.80413	1.06710132	0.09301591
1420394_s_at	glycoprotein 49 A /// leukocyte immunoglobulin-like receptor, su	Gp49a /// Lilrb4	0.80427	1.10715242	0.13776522
1449724_s_at	DNA segment, Chr 8, ERATO Doi 738, expressed	D8Ertd738e	0.80434	1.07127271	0.0948051
1421946_at	C-reactive protein, pentraxin-related	Crp	0.80434	-1.08932271	-0.1270833
1459042_at	regulating synaptic membrane exocytosis 3	Rims3	0.80451	1.09712856	0.13372223
1444261_at	---	---	0.80452	1.11173199	0.14276145
1449063_at	SEC22 vesicle trafficking protein homolog B (S. cerevisiae)	Sec22b	0.80457	1.06330472	0.08769885
1435966_x_at	mitochondrial ribosomal protein L13	Mrpl13	0.80458	-1.08431332	-0.1309735
1437249_at	src family associated phosphoprotein 1	Skap1	0.80459	-1.09032207	-0.1283437
1456677_at	---	---	0.8046	-1.09809588	-0.145231
1429755_a_at	RIKEN cDNA 4933406F09 gene	4933406F09Rik	0.80461	1.12435505	0.15649393
1435733_x_at	ribonuclease H2, subunit C	Rnaseh2c	0.80462	1.08315783	0.11029764
1426966_at	axin 1	Axin1	0.80462	1.07821894	0.10586169
1443065_at	---	---	0.80465	-1.09714721	-0.1400356
1438934_x_at	sema domain, immunoglobulin domain (Ig), transmembrane dom	Sema4a	0.80466	-1.08100271	-0.1165494
1448368_at	dynactin 6	Dctn6	0.80467	1.07759141	0.10626142
1425911_a_at	fibroblast growth factor receptor 1	Fgfr1	0.80468	1.16781694	0.18003468
1425585_at	mediator of RNA polymerase II transcription, subunit 12 homolog	Med12	0.8047	1.08857979	0.11593116

1430980_a_at	eukaryotic translation initiation factor 4A1	Eif4a1	0.80471	-1.03983467	-0.0575942
1427483_at	solute carrier family 25 (mitochondrial carrier, phosphate carrier),	Slc25a24	0.80473	1.13589811	0.16155749
1428108_x_at	transmembrane and coiled-coil domains 2	Tmcc2	0.80473	1.09217188	0.1216577
1432707_at	3-phosphoglycerate dehydrogenase pseudogene	Gm8096	0.80476	-1.0936478	-0.1369832
1448164_at	kelch domain containing 3	Klhdc3	0.80477	1.06106853	0.08537396
1431770_at	calcium homeostasis endoplasmic reticulum protein	Cherp	0.80477	1.0971964	0.12696576
1443497_at	---	---	0.80477	1.1010879	0.13587235
1439568_at	gene regulated by estrogen in breast cancer protein	Greb1	0.80477	1.11552976	0.14896739
1449076_x_at	acireductone dioxygenase 1	Adi1	0.80482	-1.06089579	-0.0856478
1459446_at	leucine rich repeat containing 41	Lrrc41	0.80483	1.1100499	0.13916615
1420170_at	myosin, heavy polypeptide 9, non-muscle	Myh9	0.80484	-1.08561204	-0.1285114
1460657_at	wingless related MMTV integration site 10a	Wnt10a	0.80485	-1.09121867	-0.1270963
1427631_x_at	major urinary protein 3	Mup3	0.80485	-1.08178026	-0.1274351
1455828_at	G protein-regulated inducer of neurite outgrowth 1	Gprin1	0.80486	-1.08244124	-0.1143008
1426539_at	ubiquitin specific peptidase 11	Usp11	0.80486	1.06198345	0.08622908
1415709_s_at	golgi-specific brefeldin A-resistance factor 1	Gbf1	0.80487	1.07760818	0.10417628
1433307_at	RIKEN cDNA 4930509E22 gene	4930509E22Rik	0.80488	-1.08592779	-0.1206888
1450263_at	protocadherin beta 5	Pcdhb5	0.80489	-1.08983821	-0.1261554
1458477_at	---	---	0.80489	-1.09270438	-0.1286587
1446967_at	---	---	0.8049	-1.10261865	-0.1503807
1446849_at	---	---	0.80492	-1.10858818	-0.1758609
1433417_at	RIKEN cDNA 8030497O21 gene	8030497O21Rik	0.80493	1.10273952	0.13691098
1439108_at	myeloid/lymphoid or mixed-lineage leukemia 5	Mll5	0.80493	1.16521857	0.18233588
1438856_x_at	serine (or cysteine) peptidase inhibitor, clade B, member 5	Serpinb5	0.80494	1.08194265	0.10939619
1441412_s_at	tripartite motif-containing 45	Trim45	0.80496	1.10345571	0.13419323
1432996_at	---	---	0.80496	-1.10027861	-0.1420808
1437265_at	RIKEN cDNA 5330438D12 gene /// hypothetical protein LOC1005	5330438D12Rik /// LOC10	0.80496	1.11637832	0.14617953
1432506_at	transmembrane protein 86B	Tmem86b	0.80496	-1.09371704	-0.1301058
1448140_at	cytokine induced apoptosis inhibitor 1	Ciapin1	0.80498	1.07807302	0.10458363
1427573_at	cysteine-rich hydrophobic domain 1	Chic1	0.80498	-1.085503	-0.1224768
1451251_at	amyloid beta precursor protein (cytoplasmic tail) binding protein 2	Appbp2	0.80499	-1.08352142	-0.1333762
1428784_at	Gem-interacting protein	Gmip	0.805	1.09332601	0.12639553
1455170_at	RIKEN cDNA 2810001G20 gene	2810001G20Rik	0.80502	1.08784097	0.11959625
1422185_a_at	cytochrome b5 reductase 3	Cyb5r3	0.80503	1.06362224	0.08753762
1438602_s_at	mannan-binding lectin serine peptidase 1	Masp1	0.80503	-1.08943005	-0.13042
1447661_at	---	---	0.80504	1.07380749	0.10204298
1421521_at	RIKEN cDNA 4930430A15 gene	4930430A15Rik	0.80505	1.08622281	0.1182649
1424266_s_at	carboxylesterase 1F	Ces1f	0.80505	1.09335167	0.12661099
1424345_s_at	ubiquitin-conjugating enzyme E2M (UBC12 homolog, yeast)	Ube2m	0.80509	1.06384316	0.088391
1449677_s_at	transmembrane protein 38B	Tmem38b	0.80512	-1.07137021	-0.1030874
1418471_at	placental growth factor	Pgf	0.80513	-1.09833645	-0.1514186
1453759_at	---	---	0.80514	-1.08625053	-0.1217165
1419006_s_at	pellino 2	Peli2	0.80517	1.09383173	0.12747954
1436969_at	RIKEN cDNA 4922501L14 gene	4922501L14Rik	0.80517	1.09362791	0.12468683
1430909_at	nucleoporin 210-like	Nup210l	0.80518	1.09387108	0.12710659
1447109_at	---	---	0.80518	-1.11610307	-0.2065638
1425606_at	solute carrier family 5 (iodide transporter), member 8	Slc5a8	0.80518	1.08713695	0.12016508
1427086_at	Slit homolog 3 (Drosophila)	Slit3	0.80521	1.1026269	0.13442604
1428157_at	guanine nucleotide binding protein (G protein), gamma 2	Gng2	0.80522	-1.08614757	-0.1236752
1422735_at	forkhead box Q1	Foxq1	0.80522	-1.09793485	-0.151492
1437716_x_at	kinesin family member 22	Kif22	0.80523	-1.05221796	-0.0736481
1419398_a_at	receptor accessory protein 5	Reep5	0.80526	1.10174569	0.12730409
1457572_at	---	---	0.80528	1.16536093	0.18546604
1416307_at	adaptor-related protein complex AP-1, mu subunit 1	Ap1m1	0.80528	1.07834785	0.10696533
1434628_a_at	rhophilin, Rho GTPase binding protein 2	Rhpn2	0.80528	1.06779933	0.09424411
1445128_at	---	---	0.80529	1.09351003	0.12445403
1456247_x_at	proteolipid protein 2	Plp2	0.8053	-1.09252409	-0.1292739
1432273_a_at	Duffy blood group, chemokine receptor	Darc	0.80531	-1.08905949	-0.1261638
1452408_at	---	---	0.80531	1.10384291	0.13612724
1455821_x_at	complement component 1, q subcomponent binding protein	C1qbp	0.80532	1.03620396	0.05115089
1434557_at	huntingtin interacting protein 1	Hip1	0.80537	1.09023485	0.11995009
1457740_at	Aryl hydrocarbon receptor nuclear translocator-like	Arntl	0.80537	-1.09540005	-0.137428
1431829_a_at	ral guanine nucleotide dissociation stimulator-like 3	Rgl3	0.8054	1.11247692	0.14635895
1459676_at	tetratricopeptide repeat domain 15	Ttc15	0.80541	-1.09132061	-0.1266796
1455859_at	RIKEN cDNA A330021E22 gene	A330021E22Rik	0.80541	1.1063318	0.13857712
1426450_at	phospholipase C-like 2	Plcl2	0.80542	1.09688719	0.130977
1456736_x_at	mitochondrial fission factor	Mff	0.80544	-1.06052342	-0.0848499
1420628_at	---	---	0.80547	-1.0645459	-0.0913618
1428099_a_at	serine/arginine-rich splicing factor 1	Srsf1	0.8055	-1.05534548	-0.0812959
1442480_at	---	---	0.80553	-1.09296767	-0.1341855
1426676_s_at	translocase of outer mitochondrial membrane 70 homolog A (yea	Tomm70a	0.80555	-1.07356498	-0.1133227
1453429_at	RIKEN cDNA 9530057J20 gene	9530057J20Rik	0.80563	-1.09403179	-0.1335337
1438961_s_at	bleomycin hydrolase	Blmh	0.80563	1.0722908	0.09684895
1448512_at	histone H1-like protein in spermatids 1	Hils1	0.80586	1.1458025	0.17173712
1427068_x_at	RIKEN cDNA 4933439F18 gene	4933439F18Rik	0.80603	-1.08020659	-0.1142572
1457391_at	Vesicle-associated membrane protein 3	Vamp3	0.80606	1.10184786	0.13502433
1453740_a_at	cyclin L2	Ccnl2	0.80606	1.07712338	0.10475887
1426513_at	RNA binding motif protein 28	Rbm28	0.80607	-1.07992438	-0.1111384
1420462_at	interleukin 1 receptor accessory protein-like 2	Il1rapl2	0.80609	1.08942884	0.12250636
1456142_x_at	mortality factor 4 like 1	Morf4l1	0.80609	-1.04427402	-0.0637546
1449490_at	methyl-CpG binding domain protein 4	Mbd4	0.80611	1.09984097	0.12536819
1456634_at	RIKEN cDNA 9830001H06 gene	9830001H06Rik	0.80611	1.08273016	0.11255336
1434995_s_at	death effector domain-containing	Dedd	0.80613	1.08101018	0.10801678
1456563_at	RIKEN cDNA 4933429F08 gene	4933429F08Rik	0.80614	1.09599024	0.12852648
1445260_at	DCN1, defective in cullin neddylation 1, domain containing 1 (S. ce	Dcun1d1	0.80615	1.10836629	0.13923304
1444868_at	Leucine rich repeat containing 9	Lrrc9	0.80615	1.10545079	0.13915913
1435012_x_at	chymotrypsin-like elastase family, member 3B /// predicted gene	Cela3b /// Gm13011	0.80617	1.09294431	0.12277357
1452594_at	dual specificity phosphatase 11 (RNA/RNP complex 1-interacting)	Dusp11	0.80618	1.07906819	0.10652882
1440105_at	lysine (K)-specific demethylase 4D	Kdm4d	0.80618	-1.10525261	-0.1557106
1457709_a_at	RIKEN cDNA A930005H10 gene	A930005H10Rik	0.8062	1.09611667	0.12511469
1426804_at	SWI/SNF related, matrix associated, actin dependent regulator of	Smarca4	0.80621	1.06588185	0.09076379
1431359_a_at	RIKEN cDNA 1110007C09 gene	1110007C09Rik	0.80622	1.10804021	0.13766922
1458808_at	---	---	0.80623	1.07256919	0.09908863
1437462_x_at	matrix metallopeptidase 15	Mmp15	0.80624	1.08556413	0.11501849

1436206_at	F-box protein 10	Fbxo10	0.80626	1.08930091	0.12010666
1433328_at	RIKEN cDNA 1700095A21 gene	1700095A21Rik	0.80627	1.09696715	0.13172537
1455078_at	hypothetical protein LOC100504120 /// slingshot homolog 2 (Dro	LOC100504120 /// Ssh2	0.80629	1.0949512	0.12336428
1433733_a_at	cryptochrome 1 (photolyase-like)	Cry1	0.80629	-1.0742665	-0.1043513
1445215_at	---	---	0.80629	1.08582518	0.11400119
1453961_a_at	motile sperm domain containing 3	Mospd3	0.8063	1.08909876	0.11976681
1417223_at	CD2 antigen (cytoplasmic tail) binding protein 2	Cd2bp2	0.80632	1.06565233	0.09096474
1456043_at	ubiquitin specific peptidase 22	Usp22	0.80634	1.06914529	0.0959106
1417621_at	nuclear factor of activated T-cells, cytoplasmic, calcineurin-depen	Nfatc1	0.80635	-1.09105493	-0.1280507
1420404_at	CD86 antigen	Cd86	0.80636	-1.09648563	-0.1368747
1452283_at	Ras association (RalGDS/AF-6) domain family (N-terminal) memb	Rassf8	0.80638	1.08461128	0.11417082
1449100_at	par-6 (partitioning defective 6,) homolog alpha (C. elegans)	Pard6a	0.80639	1.07881076	0.10767788
1420125_at	T-cell leukemia translocation altered gene	Tcta	0.80641	-1.08511842	-0.1185591
1436869_at	sonic hedgehog	Shh	0.80641	1.1037023	0.1328778
1423346_at	degenerative spermatocyte homolog 1 (Drosophila)	Degs1	0.80642	-1.06926478	-0.1004372
1429196_at	RAB GTPase activating protein 1-like	Rabgap1l	0.80643	1.14065349	0.16488392
1420781_at	ectopic ossification 1	Etos1	0.80644	-1.10559129	-0.1559096
1444636_at	RIKEN cDNA 4930592I03 gene	4930592I03Rik	0.80644	-1.10131753	-0.152704
1426153_a_at	desmoglein 2	Dsg2	0.80644	1.09454101	0.12245791
1444709_at	inversin	Invs	0.80647	-1.08318521	-0.1211983
1422083_at	toll-like receptor 9	Tlr9	0.80648	-1.08984982	-0.1345701
1425116_a_at	spectrin beta 4	Spnb4	0.80649	1.07034317	0.09675717
1422348_at	histocompatibility 2, D region locus 1 /// histocompatibility 2, D re	H2-D1 /// H2-L /// H2-Q2	0.8065	-1.09535437	-0.1314968
1433601_at	adrenergic receptor, alpha 2a	Adra2a	0.80651	-1.09511514	-0.1330219
1430507_at	RIKEN cDNA 1700027J07 gene	1700027J07Rik	0.80653	1.11428576	0.14678307
1439671_at	RIKEN cDNA 4930466K18 gene	4930466K18Rik	0.80655	1.09557537	0.12894744
1431105_a_at	transmembrane protein 33	Tmem33	0.80655	1.06944499	0.09584457
1426010_a_at	erythrocyte protein band 4.1-like 3	Epb4.1l3	0.80656	1.09115674	0.12329093
1445950_at	cDNA sequence, C87926	C87926	0.80657	-1.09243367	-0.1340183
1417998_at	prostaglandin E synthase 3 (cytosolic)	Ptges3	0.80658	-1.05380497	-0.0779375
1436733_at	RIKEN cDNA E130309F12 gene	E130309F12Rik	0.80659	-1.11653208	-0.1911255
1417087_at	golgi apparatus protein 1	Glg1	0.8066	-1.07058792	-0.1040803
1441386_at	---	---	0.80663	1.09932439	0.13291167
1455316_x_at	cDNA sequence BC094435	BC094435	0.80663	1.05141625	0.07051211
1452419_at	HEAT repeat containing 1	Heatr1	0.80663	-1.10672167	-0.202709
1421088_at	glypican 4	Gpc4	0.80666	-1.07055412	-0.1005345
1448228_at	lysyl oxidase	Lox	0.80667	-1.08400775	-0.1165373
1434867_at	solute carrier family 4, sodium bicarbonate transporter-like, mem	Slc4a11	0.80668	1.07301142	0.10129898
1451716_at	v-maf musculoaponeurotic fibrosarcoma oncogene family, protei	Mafb	0.8067	-1.09923994	-0.1483549
1422397_a_at	interleukin 15 receptor, alpha chain	Il15ra	0.8067	-1.10382115	-0.158553
1448636_at	myozenin 1	Myoz1	0.80674	-1.11050443	-0.1800763
1450319_at	gamma-aminobutyric acid (GABA) A receptor, subunit beta 2	Gabrb2	0.80679	1.10808171	0.14076396
1444762_at	---	---	0.80685	1.11403392	0.14129011
1447901_x_at	Sfi1 homolog, spindle assembly associated (yeast)	Sfi1	0.80687	1.13874403	0.16598189
1424915_s_at	RIKEN cDNA 2310044G17 gene	2310044G17Rik	0.8069	-1.07269119	-0.1021234
1442524_at	---	---	0.80691	-1.09961083	-0.1596619
1447760_x_at	ets homologous factor	Ehf	0.80692	-1.0943364	-0.1323841
1422293_a_at	potassium channel tetramerisation domain containing 1	Kctd1	0.80696	1.0890935	0.12236905
1433859_at	INO80 complex subunit C	Ino80c	0.80699	1.07969542	0.10813686
1416529_at	epithelial membrane protein 1	Emp1	0.80703	-1.0784522	-0.1174953
1442413_at	potassium voltage-gated channel, shaker-related subfamily, mem	Kcna1	0.8071	-1.10002459	-0.1453299
1443044_at	hypothetical A830091E24	A830091E24	0.8075	-1.09753912	-0.1469027
1428254_at	purine rich element binding protein B	Purb	0.80759	-1.07115905	-0.1047417
1452874_at	RIKEN cDNA 2510003E04 gene	2510003E04Rik	0.80761	1.07166491	0.09630369
1451838_a_at	tandem C2 domains, nuclear	Tc2n	0.80761	1.09388279	0.12072012
1439902_at	complement component 5a receptor 1	C5ar1	0.80761	1.09891648	0.12829436
1420230_at	expressed sequence AA414993	AA414993	0.80762	-1.08911082	-0.1318759
1452060_a_at	LIM motif-containing protein kinase 2	Limk2	0.80762	1.07371304	0.09943314
1445929_at	Predicted gene 3837	Gm3837	0.80763	-1.09040373	-0.1282138
1447080_at	aconitase 1	Aco1	0.80765	1.09714918	0.13159212
1452165_at	prolactin family 2, subfamily b, member 1	Prl2b1	0.80766	1.07786185	0.104656
1442439_at	expressed sequence W91776	W91776	0.80766	1.10669296	0.13860289
1449296_a_at	2',3'-cyclic nucleotide 3' phosphodiesterase	Cnp	0.80766	1.07866852	0.10755635
1460676_at	Josephin domain containing 1	Josd1	0.80767	1.06470483	0.08978803
1450439_at	host cell factor C1	Hcfc1	0.80767	-1.07892935	-0.1163076
1450044_at	frizzled homolog 7 (Drosophila)	Fzd7	0.80767	1.07186643	0.09662063
1448622_at	LSM4 homolog, U6 small nuclear RNA associated (S. cerevisiae)	Lsm4	0.80774	1.06514987	0.08854717
1444220_at	RIKEN cDNA 2410089E03 gene	2410089E03Rik	0.80774	-1.08937195	-0.1303766
1452523_a_at	coiled-coil domain containing 130	Ccdc130	0.80775	1.09572965	0.12738351
1451329_at	nudix (nucleoside diphosphate linked moiety X)-type motif 22	Nudt22	0.80779	1.09498706	0.12285101
1423048_a_at	toll interacting protein	Tollip	0.80779	1.09074249	0.12054716
1421725_at	solute carrier family 26, member 5	Slc26a5	0.8078	1.09931757	0.13511646
1444475_at	---	---	0.8078	-1.08686381	-0.1203921
1415720_s_at	MAD2L1 binding protein	Mad2l1bp	0.8078	1.08233951	0.11010736
1433712_at	expressed sequence AW555464	AW555464	0.80783	1.06920543	0.09477677
1419500_at	poly(A) binding protein, cytoplasmic 2	Pabpc2	0.80784	-1.09247834	-0.1302138
1416172_at	pescadillo homolog 1, containing BRCT domain (zebrafish)	Pes1	0.80784	1.05979968	0.08358089
1442047_at	ras responsive element binding protein 1	Rreb1	0.80785	1.08749315	0.11684995
1452262_at	GrpE-like 2, mitochondrial	Grpel2	0.80786	1.07163815	0.09858945
1417239_at	centrin 3	Cetn3	0.80786	-1.06383969	-0.0916279
1434169_at	RIKEN cDNA 9030409G11 gene	9030409G11Rik	0.80786	1.07659259	0.10409165
1423433_at	TROVE domain family, member 2	Trove2	0.80788	-1.08886253	-0.1355511
1436473_at	zinc finger protein 248	Zfp248	0.80788	-1.10047029	-0.1565183
1435527_at	nuclear factor I/C	Nfic	0.80791	-1.07349507	-0.1083133
1455407_at	zinc finger protein 236	Zfp236	0.80791	-1.09023802	-0.1299273
1423116_at	DOM-3 homolog Z (C. elegans)	Dom3z	0.80791	1.07080871	0.09830675
1419715_at	RIKEN cDNA 1700029F12 gene	1700029F12Rik	0.80792	-1.09045069	-0.1252868
1449578_at	suppressor of Ty 16 homolog (S. cerevisiae)	Supt16h	0.80792	-1.06682427	-0.0981431
1424853_s_at	cytochrome P450, family 4, subfamily a, polypeptide 10 /// cytoc	Cyp4a10 /// Cyp4a31	0.80793	-1.09517221	-0.1346884
1432312_a_at	protease, serine, 41	Prss41	0.80793	-1.09358309	-0.1333315
1449997_at	tropomyosin 3, gamma	Tpm3	0.80794	-1.10177997	-0.1463233
1427554_at	helicase, POLQ-like	Helq	0.80794	-1.09062774	-0.1295536
1436435_at	hypothetical LOC100503057	LOC100503057	0.80794	1.10084363	0.1299353
1435539_at	huntingtin /// hypothetical LOC100503657	Htt /// LOC100503657	0.80795	-1.08484705	-0.1229582

1440642_at	RIKEN cDNA D630042P16 gene	D630042P16Rik	0.80796	-1.08402636	-0.1177422
1426687_at	mitogen-activated protein kinase kinase kinase 3	Map3k3	0.80799	1.08076569	0.11058422
1436693_x_at	solute carrier family 35, member E4	Slc35e4	0.80799	1.08410801	0.11643189
1457827_at	arylsulfatase J	Arsj	0.808	1.07876695	0.10632724
1419979_s_at	cAMP responsive element binding protein 3	Creb3	0.80802	-1.07252719	-0.1029921
1453798_at	coiled-coil domain containing 93	Ccdc93	0.80806	-1.10492471	-0.1585529
1421467_at	runt related transcription factor 3	Runx3	0.8083	1.08377505	0.11514431
1456371_a_at	shroom family member 1	Shroom1	0.80845	-1.09173182	-0.1276471
1445738_at	---	---	0.80846	1.10528156	0.13898575
1431952_at	RIKEN cDNA 4930532I03 gene	4930532I03Rik	0.8085	1.13452911	0.15454112
1425619_s_at	desmoglein 2	Dsg2	0.8085	1.19695822	0.20180916
1457328_at	adrenergic receptor, alpha 2c	Adra2c	0.80851	1.10762849	0.13991017
1423145_a_at	titin-cap	Tcap	0.80853	-1.07896154	-0.1098611
1423374_at	nuclear receptor coactivator 6	Ncoa6	0.80854	-1.06330602	-0.089329
1453350_at	upregulator of cell proliferation	Urgcp	0.80855	1.09769998	0.1317267
1456945_at	nudix (nucleoside diphosphate linked moiety X)-type motif 6	Nudt6	0.80858	-1.09492643	-0.1356272
1454855_at	membrane associated guanylate kinase, WW and PDZ domain co	Magi2	0.80858	-1.0934301	-0.1398222
1458216_at	---	---	0.80859	-1.09003459	-0.1323095
1455726_at	predicted gene 71	Gm71	0.80859	1.07237852	0.0996024
1447413_at	---	---	0.80861	-1.10468509	-0.1603786
1423761_at	methyltransferase like 13	Mettl13	0.80862	-1.07597357	-0.1089475
1450226_at	prolactin receptor	Prlr	0.80863	1.12224683	0.14999074
1451175_at	signal peptidase complex subunit 3 homolog (S. cerevisiae)	Spcs3	0.80863	1.06066056	0.08491718
1448962_at	myosin, heavy polypeptide 11, smooth muscle	Myh11	0.80864	-1.08352849	-0.1169768
1450014_at	claudin 1	Cldn1	0.80864	-1.0993296	-0.1393675
1428734_at	RIKEN cDNA 3200002M19 gene	3200002M19Rik	0.80865	1.08481514	0.11343611
1429740_at	tetratricopeptide repeat domain 7	Ttc7	0.80865	1.09051926	0.1240394
1457688_at	zinc finger protein 398	Zfp398	0.80868	-1.10106228	-0.1544049
1417795_at	cell adhesion molecule with homology to L1CAM	Chl1	0.80868	1.07842518	0.10648822
1417558_at	Fyn proto-oncogene	Fyn	0.80868	-1.06653483	-0.0941481
1438154_x_at	RIKEN cDNA 2610002J02 gene	2610002J02Rik	0.80868	1.07404986	0.10301743
1438482_at	WW domain containing E3 ubiquitin protein ligase 2	Wwp2	0.8087	-1.09722109	-0.1463555
1432981_at	RIKEN cDNA 4930423D22 gene	4930423D22Rik	0.8087	1.09966193	0.12900539
1456274_at	---	---	0.8087	1.14214805	0.16824593
1457282_x_at	Tubulin, gamma complex associated protein 5	Tubgcp5	0.8087	1.10630769	0.13843278
1441251_a_at	RIKEN cDNA 2010001A14 gene	2010001A14Rik	0.8087	-1.10407242	-0.1576541
1443869_at	phosphodiesterase 12	Pde12	0.80873	-1.0819845	-0.1146384
1424614_at	post-GPI attachment to proteins 2	Pgap2	0.80873	1.09805514	0.12289431
1439172_at	ATPase type 13A5	Atp13a5	0.80873	-1.09777154	-0.1465498
1435244_at	vav 2 oncogene	Vav2	0.80875	1.09428934	0.1251058
1427605_at	homeobox B3	Hoxb3	0.80877	-1.09688633	-0.1445491
1445114_at	---	---	0.80878	1.09536113	0.12963021
1460377_a_at	transmembrane protein 8 (five membrane-spanning domains)	Tmem8	0.80879	1.06817815	0.09475633
1441250_at	ENTH domain containing 1	Enthd1	0.8088	1.12406186	0.15204561
1454898_s_at	isoamyl acetate-hydrolyzing esterase 1 homolog (S. cerevisiae)	Iah1	0.80883	-1.07244304	-0.1038886
1436416_x_at	fractured callus expressed transcript 1	Fxc1	0.80899	1.08489491	0.10856836
1419757_at	phosphatidylinositol transfer protein, membrane-associated 2	Pitpnm2	0.80911	-1.08783514	-0.1249045
1448577_x_at	synaptogyrin 2	Syngr2	0.80912	1.07326142	0.10025424
1454211_a_at	shroom family member 3	Shroom3	0.80918	1.07767864	0.10721412
1447337_at	---	---	0.80918	-1.09336612	-0.1342189
1417973_at	inter-alpha trypsin inhibitor, heavy chain 1	Itih1	0.80919	-1.10817225	-0.1773661
1445158_at	synaptophysin-like protein	Sypl	0.8092	-1.08541645	-0.1188474
1436111_at	transmembrane protein 212	Tmem212	0.8092	1.09342542	0.1282721
1442776_at	---	---	0.80921	-1.08915524	-0.1251234
1442653_at	---	---	0.80921	-1.07561494	-0.1087721
1418889_a_at	casein kinase 1, delta	Csnk1d	0.80922	1.06202542	0.08604971
1450110_at	alcohol dehydrogenase 7 (class IV), mu or sigma polypeptide	Adh7	0.80924	-1.07974348	-0.1115028
1431628_at	RIKEN cDNA 4930435H24 gene	4930435H24Rik	0.80926	1.0873924	0.11903411
1456530_x_at	elongation of very long chain fatty acids (FEN1/Elo2, SUR4/Elo3, y	Elovl1	0.80926	1.06769337	0.09392447
1455742_x_at	RIKEN cDNA A730098P11 gene /// mortality factor 4 like 1	A730098P11Rik /// Morf4	0.80926	-1.04527932	-0.0654597
1453767_a_at	5',3'-nucleotidase, mitochondrial	Nt5m	0.80927	1.08063116	0.1116923
1424078_s_at	peroxisomal biogenesis factor 6	Pex6	0.80928	1.07345158	0.10025526
1449904_at	dopey family member 2	Dopey2	0.80928	1.09567057	0.12996342
1433223_at	RIKEN cDNA 5830442K09 gene	5830442K09Rik	0.80948	1.10826731	0.14004729
1425244_a_at	testicular haploid expressed gene	Theg	0.80954	1.09804241	0.13143391
1437275_at	Catenin (cadherin associated protein), alpha 1	Ctnna1	0.80959	1.0817383	0.10959318
1440640_at	RIKEN cDNA 2010111I01 gene	2010111I01Rik	0.80959	1.11225161	0.14605624
1425654_a_at	LIM domain and actin binding 1	Lima1	0.80972	1.08740409	0.11655689
1439451_x_at	G protein-coupled receptor 172B	Gpr172b	0.80972	1.08325404	0.1132295
1453470_a_at	guanine nucleotide binding protein, alpha 13	Gna13	0.80975	1.07802757	0.10526532
1434018_at	family with sequence similarity 168, member B	Fam168b	0.80975	1.07618765	0.10422277
1445366_at	---	---	0.80977	1.14245796	0.16553678
1428337_at	magnesium-dependent phosphatase 1	Mdp1	0.80981	1.07038754	0.09787825
1424554_at	protein phosphatase 1, regulatory (inhibitor) subunit 8	Ppp1r8	0.80981	1.07291663	0.09730096
1443104_at	---	---	0.80985	1.11061679	0.14152829
1438085_at	HEAT repeat containing 5B	Heatr5b	0.80989	-1.11212228	-0.1918044
1422471_at	peroxisomal biogenesis factor 13	Pex13	0.80991	1.06405564	0.08844956
1431041_at	GTP-binding protein 8 (putative)	Gtpbp8	0.80991	-1.09011223	-0.1258833
1454358_at	RIKEN cDNA 2900060N12 gene	2900060N12Rik	0.80991	-1.0946387	-0.1332457
1442753_at	tumor necrosis factor, alpha-induced protein 8	Tnfaip8	0.80992	1.10086142	0.1321999
1455444_at	gamma-aminobutyric acid (GABA) A receptor, subunit alpha 2	Gabra2	0.80992	-1.06886698	-0.0973546
1438863_at	RIKEN cDNA A530079E22 gene	A530079E22Rik	0.80993	-1.09425376	-0.1328967
1452064_at	mediator complex subunit 23	Med23	0.80993	1.06395505	0.08932695
1420451_at	amiloride-sensitive cation channel 5, intestinal	Accn5	0.80994	-1.09665791	-0.1357992
1455631_at	histocompatibility 13	H13	0.80995	1.13042432	0.16128523
1423648_at	protein disulfide isomerase associated 6	Pdia6	0.80995	1.05908406	0.08261507
1427169_at	par-3 partitioning defective 3 homolog B (C. elegans)	Pard3b	0.80997	-1.09709377	-0.1484341
1422986_at	estrogen related receptor, beta	Esrrb	0.80997	-1.0656201	-0.0918137
1438879_at	---	---	0.80997	-1.09811354	-0.1408477
1454490_at	RIKEN cDNA 4930423C22 gene	4930423C22Rik	0.80998	1.08509333	0.11739673
1416285_at	NADH dehydrogenase (ubiquinone) 1, subcomplex unknown, 1	Ndufc1	0.80999	1.07872518	0.10234817
1433390_at	cDNA sequence BC049349	BC049349	0.81	-1.09468464	-0.1398467
1455738_at	coiled-coil domain containing 55	Ccdc55	0.81	-1.06813827	-0.0956554
1426952_at	Rho GTPase activating protein 18	Arhgap18	0.81001	-1.10164566	-0.1559118

1436602_x_at	calcium channel, voltage-dependent, N type, alpha 1B subunit	Cacna1b	0.81001	1.07087998	0.09790958
1422692_at	SUB1 homolog (S. cerevisiae)	Sub1	0.81002	1.07831311	0.10451833
1445463_at	aminopeptidase puromycin sensitive	Npepps	0.81002	-1.10344322	-0.158362
1420039_s_at	chromobox homolog 7	Cbx7	0.81003	-1.10027187	-0.187037
1417447_at	transcription factor 21	Tcf21	0.81003	1.16362608	0.18203084
1430783_at	RIKEN cDNA 4932416J16 gene	4932416J16Rik	0.81005	-1.09077093	-0.1306728
1432135_s_at	methyltransferase like 4, pseudogene 1 /// speedy homolog A (X	Mettl4-ps1 /// Spdya	0.81005	1.06452813	0.08865699
1438158_at	ATP/GTP binding protein-like 4	Agbl4	0.81005	-1.09429633	-0.1414686
1451024_at	sphingosine-1-phosphate receptor 4	S1pr4	0.81009	1.07758682	0.10735301
1444145_at	---	---	0.8101	-1.08526163	-0.1184757
1420978_at	nuclear respiratory factor 1	Nrf1	0.8101	1.08600917	0.11632506
1440262_at	expressed sequence AI414108	AI414108	0.81011	-1.0752349	-0.104841
1456543_at	prokineticin receptor 1	Prokr1	0.81012	-1.06751365	-0.0954617
1415865_s_at	2,3-bisphosphoglycerate mutase	Bpgm	0.81013	1.08019572	0.10995985
1443683_at	---	---	0.81013	-1.08782222	-0.1272114
1421013_at	phosphatidylinositol transfer protein, beta	Pitpnb	0.81013	-1.061917	-0.0879044
1454150_at	RIKEN cDNA 4930453O03 gene	4930453O03Rik	0.81014	1.10310603	0.13737087
1450005_x_at	delta-like 2 homolog (Drosophila)	Dlk2	0.81014	1.08388633	0.11490994
1457605_at	---	---	0.81015	1.09450308	0.12220091
1454191_at	keratin associated protein 5-3	Krtap5-3	0.81016	1.1502607	0.17370095
1425263_a_at	myelin basic protein	Mbp	0.81016	1.11728574	0.14770081
1427956_at	polycomb group ring finger 1	Pcgf1	0.81016	1.09341682	0.1235779
1430760_a_at	ubiquitin specific peptidase 50	Usp50	0.81016	1.10030669	0.1341512
1431257_at	RIKEN cDNA 6230409E13 gene	6230409E13Rik	0.81017	1.09420236	0.12901736
1434668_at	nuclear casein kinase and cyclin-dependent kinase substrate 1	Nucks1	0.81017	-1.07564714	-0.1129201
1444901_at	---	---	0.81017	-1.08130186	-0.1205318
1457653_at	---	---	0.81018	1.09684499	0.12930868
1431895_at	RIKEN cDNA 4933434M16 gene	4933434M16Rik	0.81019	-1.09229812	-0.1349096
1433351_at	RIKEN cDNA 4933412L11 gene	4933412L11Rik	0.81019	1.12017036	0.15242367
1434089_at	synaptopodin	Synpo	0.81019	-1.11205376	-0.1804919
1460695_a_at	RIKEN cDNA 2010111I01 gene	2010111I01Rik	0.81019	1.06435692	0.08926968
1427720_a_at	ribosomal RNA processing 1 homolog (S. cerevisiae)	Rrp1	0.81019	1.07371926	0.09762432
1447988_at	---	---	0.8102	1.13322532	0.16062966
1456243_x_at	myeloid cell leukemia sequence 1	Mcl1	0.8102	-1.06781444	-0.1012188
1432921_at	---	---	0.8102	1.09305158	0.12564337
1425753_a_at	uracil DNA glycosylase	Ung	0.81021	-1.05940675	-0.0845078
1453823_a_at	coiled-coil domain containing 76	Ccdc76	0.81022	1.07817173	0.10812899
1423372_at	polymerase (DNA-directed), epsilon 4 (p12 subunit)	Pole4	0.81022	1.07807542	0.10602589
1460427_a_at	a disintegrin and metallopeptidase domain 28	Adam28	0.81023	-1.08922662	-0.123551
1444044_at	---	---	0.81026	1.1142857	0.14506609
1446648_at	---	---	0.81027	-1.09397078	-0.1309801
1418502_a_at	oxidation resistance 1	Oxr1	0.81028	-1.07264372	-0.1038578
1440589_at	---	---	0.81029	-1.09162076	-0.1270719
1416772_at	carnitine palmitoyltransferase 2	Cpt2	0.81029	1.07881676	0.10590778
1437918_at	RIKEN cDNA 4930539E08 gene	4930539E08Rik	0.81029	-1.08716076	-0.1267525
1420123_at	T-cell leukemia translocation altered gene	Tcta	0.8103	1.09447545	0.12586842
1449601_x_at	---	---	0.81031	-1.10676312	-0.1777511
1422358_at	formyl peptide receptor, related sequence 4	Fpr-rs4	0.81033	-1.08774766	-0.1217505
1419262_at	acyl-Coenzyme A dehydrogenase family, member 8	Acad8	0.81034	1.07994989	0.10666408
1429390_at	acid phosphatase-like 2	Acpl2	0.81036	1.10184307	0.13495345
1453502_at	RIKEN cDNA 2210408I21 gene	2210408I21Rik	0.81036	-1.09757619	-0.1432995
1449192_at	activating transcription factor 7 interacting protein	Atf7ip	0.81038	1.07226634	0.09916941
1432763_at	proline-rich Gla (G-carboxyglutamic acid) polypeptide 2	Prrg2	0.81039	1.0963081	0.1301043
1428829_at	DENN/MADD domain containing 1B	Dennd1b	0.81041	-1.07093641	-0.0999955
1429809_at	transmembrane and tetratricopeptide repeat containing 2	Tmtc2	0.81041	-1.10491827	-0.1749607
1454516_at	RIKEN cDNA 4930424E08 gene	4930424E08Rik	0.81043	1.09334272	0.12760918
1421284_at	phosphatidylinositol glycan anchor biosynthesis, class N	Pign	0.81044	1.11909947	0.14908134
1423963_at	WD repeat domain 26	Wdr26	0.81044	-1.08770901	-0.1313307
1434599_a_at	tight junction protein 2	Tjp2	0.81046	-1.06111701	-0.0865664
1421986_at	eukaryotic translation initiation factor 4E member 2	Eif4e2	0.81046	1.0994682	0.12711898
1425101_a_at	FK506 binding protein 6	Fkbp6	0.8105	1.11799018	0.14359515
1451721_a_at	histocompatibility 2, class II antigen A, beta 1	H2-Ab1	0.8105	1.11164141	0.14375429
1426291_at	DIM1 dimethyladenosine transferase 1-like (S. cerevisiae)	Dimt1	0.81053	1.09085158	0.12350068
1440520_a_at	RIKEN cDNA 1700051A21 gene /// hypothetical LOC100503953	1700051A21Rik /// LOC1(	0.81054	1.10440932	0.13482421
1446019_at	---	---	0.81056	-1.08977281	-0.1286292
1454257_at	serine palmitoyltransferase, long chain base subunit 2	Sptlc2	0.81059	1.13261903	0.16019399
1416669_s_at	nascent polypeptide-associated complex alpha polypeptide	Naca	0.8106	-1.04311125	-0.0614236
1447326_s_at	zinc finger, MYM-type 3	Zmym3	0.81061	1.0839521	0.11070155
1432027_a_at	TBC1 domain family, member 14	Tbc1d14	0.81061	1.09433658	0.12221165
1442063_at	ADAMTS-like 1	Adamtsl1	0.81062	1.09634733	0.12858156
1425255_s_at	heterogeneous nuclear ribonucleoprotein L-like	Hnrpll	0.81064	1.07660257	0.10065099
1459373_at	---	---	0.81065	1.15358612	0.17819327
1420145_at	---	---	0.81066	-1.08550937	-0.1224392
1418959_at	transmembrane protease, serine 5 (spinesin)	Tmprss5	0.81069	1.12840134	0.15992082
1446386_at	---	---	0.8107	1.10953443	0.14177104
1454915_at	RAB3 GTPase activating protein subunit 2	Rab3gap2	0.81074	1.06888001	0.09435226
AFFX-r2-Ec-bioD-	---	---	0.81076	1.08485619	0.10566133
1416012_at	EH-domain containing 1	Ehd1	0.81076	-1.07217884	-0.1044688
1452879_at	synaptopodin 2	Synpo2	0.81078	-1.09226155	-0.136111
1445038_at	---	---	0.8108	-1.08874874	-0.1234979
1418522_at	metaxin 1	Mtx1	0.8108	1.06703378	0.09185676
1439504_s_at	zinc finger protein 28	Zfp28	0.81083	1.1214763	0.1495077
1421945_a_at	ribosome production factor 2 homolog (S. cerevisiae)	Rpf2	0.81084	1.06822941	0.09256549
1450924_at	hepatoma-derived growth factor, related protein 3	Hdgfrp3	0.81088	-1.0963109	-0.144905
1420398_at	regulator of G-protein signaling 18	Rgs18	0.81102	1.07696516	0.10320337
1449902_at	late cornified envelope 1C /// late cornified envelope 1F /// late c	Lce1c /// Lce1f /// Lce1g	0.81105	-1.08348401	-0.1183068
1430984_at	antizyme inhibitor 1	Azin1	0.81116	-1.10040352	-0.1712336
1444859_at	---	---	0.81121	1.09853225	0.12310445
1418037_at	complement component 4 binding protein	C4bp	0.81121	-1.09722165	-0.1450095
1454692_x_at	heterogeneous nuclear ribonucleoprotein K	Hnrnpk	0.81126	-1.06462173	-0.0938071
1450778_a_at	phosphorylated adaptor for RNA export	Phax	0.81128	1.05932101	0.08312338
1431355_s_at	transient receptor potential cation channel, subfamily M, membe	Trpm7	0.81129	-1.10398055	-0.162526
1451621_at	PPPDE peptidase domain containing 1	Pppde1	0.8113	-1.08060402	-0.1253766
1436715_s_at	CDP-diacylglycerol--inositol 3-phosphatidyltransferase (phosphat	Cdipt	0.81131	1.06613124	0.090854

1430959_at	non-protein coding RNA 85	Ncrna00085	0.81132	1.08394706	0.11495589
1434413_at	insulin-like growth factor 1	Igf1	0.81134	1.08508483	0.11720147
1428352_at	arrestin domain containing 2	Arrdc2	0.81136	-1.08731132	-0.1246244
1417895_a_at	transmembrane protein 54	Tmem54	0.81137	-1.10436996	-0.1571672
1435833_at	---	---	0.81139	1.08898032	0.12232616
1421095_a_at	transient receptor potential cation channel, subfamily C, member	Trpc1	0.8114	1.10232245	0.13298491
1439927_at	---	---	0.81142	-1.09379329	-0.1325714
1418861_at	protein inhibitor of activated STAT 4	Pias4	0.81142	1.0891521	0.11408998
1434099_at	peroxisome proliferative activated receptor, gamma, coactivator	Ppargc1a	0.81144	1.06988038	0.09631948
1429216_at	progestin and adipoQ receptor family member III	Paqr3	0.81144	-1.08222064	-0.1149844
1455935_at	RIKEN cDNA 2410131K14 gene	2410131K14Rik	0.81146	1.08549315	0.1170878
1436985_at	zinc finger protein 644	Zfp644	0.81148	-1.09870326	-0.1659828
1435953_at	BTAF1 RNA polymerase II, B-TFIID transcription factor-associated	Btaf1	0.81159	1.08553056	0.11512682
1436033_at	cDNA sequence BC031353	BC031353	0.81159	-1.0986524	-0.1518928
1449988_at	GTPase, IMAP family member 1	Gimap1	0.81168	-1.09508252	-0.1368834
1445703_at	patched domain containing 1	Ptchd1	0.81185	1.06249192	0.08529708
1417327_at	caveolin 2	Cav2	0.8119	1.08404892	0.11396502
1436162_at	RIKEN cDNA C730048C13 gene	C730048C13Rik	0.81192	1.16357902	0.1799546
1445346_at	---	---	0.81194	-1.09602797	-0.1441492
1456843_at	Yamaguchi sarcoma viral (v-yes) oncogene homolog 1	Yes1	0.81196	-1.10346587	-0.1615795
1429205_at	myeloid/lymphoid or mixed-lineage leukemia (trithorax homolog	Mllt3	0.81196	1.11535895	0.14627215
1429004_at	pleckstrin homology domain interacting protein	Phip	0.81199	-1.06942461	-0.0997081
1422599_s_at	zinc finger protein 143	Zfp143	0.81199	-1.07670219	-0.1143569
1452776_a_at	negative regulator of ubiquitin-like proteins 1	Nub1	0.81199	1.07371466	0.1009935
1455992_at	vestigial like 4 (Drosophila)	Vgll4	0.812	1.08855172	0.12092408
1450636_s_at	alkaline phosphatase, placental-like 2	Alppl2	0.812	-1.09360336	-0.1303147
1425449_at	phosphatidic acid phosphatase type 2A	Ppap2a	0.81201	1.09714545	0.13107338
1452900_at	DiGeorge syndrome critical region gene 6	Dgcr6	0.81201	1.10385274	0.13585079
1459979_x_at	zinc finger protein 68	Zfp68	0.81202	-1.07574873	-0.1094869
1418793_at	iduronidase, alpha-L-	Idua	0.81202	-1.08154518	-0.1194477
1418209_a_at	profilin 2	Pfn2	0.81208	1.06573159	0.09128039
1457658_x_at	annexin A4	Anxa4	0.81208	1.0878803	0.11666418
1447182_at	expressed sequence C77815	C77815	0.81209	1.09837142	0.1269627
1450227_at	ankyrin repeat domain 6	Ankrd6	0.8121	1.09743514	0.12738834
1458791_at	expressed sequence AU015849	AU015849	0.8121	-1.08445311	-0.1254611
1440836_at	RIKEN cDNA 9430025N12 gene	9430025N12Rik	0.81215	-1.07340222	-0.109676
1427744_at	cyclin B3	Ccnb3	0.81216	1.08119937	0.10904207
1454174_a_at	RIKEN cDNA C330007P06 gene	C330007P06Rik	0.8122	-1.0782025	-0.1124597
1424143_a_at	chromatin licensing and DNA replication factor 1	Cdt1	0.81227	1.05229116	0.07326515
1424217_at	poly (A) polymerase alpha	Papola	0.81227	1.12970684	0.1547826
1458974_at	---	---	0.81229	1.12543304	0.15556769
1416548_at	solute carrier family 35, member B4	Slc35b4	0.81232	1.07671025	0.10561809
1420600_at	thyroid peroxidase	Tpo	0.81235	-1.09339326	-0.129017
1449859_at	golgi transport 1 homolog B (S. cerevisiae)	Golt1b	0.81235	-1.08237013	-0.1222562
1426812_a_at	family with sequence similarity 129, member B	Fam129b	0.81242	1.10436882	0.12624751
1444704_at	---	---	0.81245	-1.09317147	-0.1300176
1419637_s_at	RIKEN cDNA 4833420G17 gene	4833420G17Rik	0.81246	1.0793903	0.10552021
1450012_x_at	tyrosine 3-monooxygenase/tryptophan 5-monooxygenase activa	Ywhag	0.81249	-1.06095277	-0.0919117
1423436_at	glutathione S-transferase, alpha 3	Gsta3	0.81252	1.09968619	0.12947155
1436410_at	Rho guanine nucleotide exchange factor (GEF) 33	Arhgef33	0.81252	-1.10542526	-0.1646274
1424061_at	mannosidase, beta A, lysosomal-like	Manbal	0.81253	1.06902378	0.09628732
1443143_at	fibronectin leucine rich transmembrane protein 1	Flrt1	0.81253	1.12138232	0.14542065
1427921_s_at	RIKEN cDNA 2310061C15 gene	2310061C15Rik	0.81254	1.12303269	0.14438808
1446615_at	---	---	0.81255	-1.09140763	-0.12661
1419436_at	complement factor H-related 1	Cfhr1	0.81255	1.07045184	0.09698348
1423350_at	suppressor of cytokine signaling 5	Socs5	0.81255	1.09954511	0.13401708
1441748_at	---	---	0.81255	1.094848	0.12756229
1416767_a_at	RIKEN cDNA 1110003E01 gene	1110003E01Rik	0.81256	-1.07528935	-0.1109939
1425639_at	ArfGAP with dual PH domains 2	Adap2	0.81256	1.10759138	0.13919326
1458349_s_at	---	---	0.81258	-1.09642076	-0.1510478
1438152_at	---	---	0.81259	-1.07516846	-0.1072041
1440395_at	terminal uridylyl transferase 1, U6 snRNA-specific	Tut1	0.81259	-1.09033158	-0.1273831
1453902_at	eukaryotic translation initiation factor 2 alpha kinase 1	Eif2ak1	0.81259	-1.0963608	-0.1366663
1450016_at	cyclin G1	Ccng1	0.81259	-1.0632649	-0.0909046
1443468_at	---	---	0.8126	1.09084982	0.12539233
1417181_a_at	kinesin-associated protein 3	Kifap3	0.8126	1.10071365	0.12619273
1416155_at	high mobility group box 3	Hmgb3	0.8126	1.08185801	0.10929497
1418561_at	splicing factor 3b, subunit 1	Sf3b1	0.8126	-1.06563335	-0.0927414
1435056_x_at	protein O-fucosyltransferase 2	Pofut2	0.8126	-1.07894071	-0.1201471
1447209_at	---	---	0.81261	1.09350468	0.12108552
1437957_at	RIKEN cDNA 7030407O06 gene	7030407O06Rik	0.81261	1.10385439	0.13661396
1440578_at	---	---	0.81262	1.10688096	0.13785897
1435843_x_at	mitochondrial ribosomal protein S9	Mrps9	0.81263	-1.07862938	-0.1198713
1431972_a_at	granule cell antiserum positive 14	Gcap14	0.81263	1.08473881	0.11408414
1436317_at	hypothetical protein LOC100504348	LOC100504348	0.81263	1.07820784	0.10645948
1423401_at	ets variant gene 6 (TEL oncogene)	Etv6	0.81263	-1.08096263	-0.1192493
1425943_at	neuromedin U receptor 2	Nmur2	0.81264	-1.09334782	-0.1299859
1415699_a_at	G protein pathway suppressor 1	Gps1	0.81264	1.06309158	0.086375
1443580_at	Syntaxin binding protein 6 (amisyn)	Stxbp6	0.81265	1.10749956	0.1303793
1430727_at	RIKEN cDNA 4930432O21 gene	4930432O21Rik	0.81265	-1.07833936	-0.1156012
1453617_at	zinc finger, RAN-binding domain containing 3	Zranb3	0.81265	1.09354756	0.12355122
1427995_at	carboxypeptidase A5	Cpa5	0.81266	-1.08863039	-0.1265112
1426177_a_at	NK1 transcription factor related, locus 2 (Drosophila)	Nkx1-2	0.81267	1.09494354	0.1277379
1459975_at	MYST histone acetyltransferase 2	Myst2	0.81267	1.11176446	0.14051779
1442823_at	SH3/ankyrin domain gene 2	Shank2	0.81268	-1.08804161	-0.1246555
1424414_at	opioid growth factor receptor-like 1	Ogfrl1	0.81268	1.09430075	0.12326512
1432575_at	RIKEN cDNA 4930548F15 gene	4930548F15Rik	0.81269	-1.09345323	-0.1314593
1425951_a_at	C-type lectin domain family 4, member n	Clec4n	0.81269	1.07741416	0.106781
1435153_at	BTB (POZ) domain containing 6	Btbd6	0.81269	1.09270102	0.12344464
1459209_at	ring finger protein 10	Rnf10	0.8127	-1.10225096	-0.1596505
1441834_x_at	---	---	0.8127	1.10425091	0.13101286
1456034_at	tetratricopeptide repeat domain 18	Ttc18	0.8127	1.08644683	0.11954305
1442908_at	---	---	0.81271	-1.0948302	-0.1355258
1441935_at	ankyrin repeat, family A (RFXANK-like), 2	Ankra2	0.81271	1.1132882	0.14461536

1441779_at	RIKEN cDNA 9530006C21 gene	9530006C21Rik	0.81272	-1.07978268	-0.112705
1416994_at	tetratricopeptide repeat domain 1	Ttc1	0.81273	1.07493401	0.09965247
1442642_at	---	---	0.81275	1.0978804	0.13223378
1426063_a_at	GTP binding protein (gene overexpressed in skeletal muscle)	Gem	0.81275	1.09899624	0.130599
1430694_at	---	---	0.81277	1.08477178	0.11470363
1418865_at	zinc finger protein 385A	Zfp385a	0.81278	-1.08113819	-0.1268653
1424393_s_at	alcohol dehydrogenase, iron containing, 1	Adhfe1	0.81278	-1.09035173	-0.1273382
1430535_at	TSC22 domain family, member 2	Tsc22d2	0.81281	-1.08395851	-0.1265001
1450049_a_at	histone cell cycle regulation defective homolog A (S. cerevisiae)	Hira	0.81283	1.11219829	0.13730117
1449646_s_at	tigger transposable element derived 5	Tigd5	0.81295	-1.08876172	-0.139746
1457887_at	SCO-spondin	Sspo	0.81302	1.08796521	0.12062886
1437178_at	predicted gene 7056 /// hypothetical LOC100504812	Gm7056 /// LOC1005048	0.81303	-1.06457771	-0.0917578
1433333_at	RIKEN cDNA C030004M13 gene	C030004M13Rik	0.81305	-1.09539449	-0.1358707
1451722_s_at	SET and MYND domain containing 5	Smyd5	0.81307	1.06939137	0.09584631
1431149_at	RIKEN cDNA B430105A11 gene	B430105A11Rik	0.81309	1.09062025	0.12081327
1441792_at	RIKEN cDNA A630033E08 gene	A630033E08Rik	0.8131	1.09309204	0.1269797
1420374_at	forkhead box J2	Foxj2	0.81312	-1.08924984	-0.1322656
1432382_at	RIKEN cDNA 4930434B07 gene	4930434B07Rik	0.81313	-1.08490246	-0.1178877
1440333_at	MAM domain containing 4	Mamdc4	0.81313	-1.0925359	-0.1437629
1420895_at	transforming growth factor, beta receptor I	Tgfbr1	0.81318	-1.06526281	-0.0925042
1422954_at	zinc finger protein 60	Zfp60	0.81324	-1.09202253	-0.1407855
1450525_at	GLI-Kruppel family member GLI3	Gli3	0.81324	1.1079594	0.13848401
1446132_at	---	---	0.81326	1.1019686	0.13553151
1423452_at	serine/threonine kinase 17b (apoptosis-inducing)	Stk17b	0.81328	1.07439658	0.09906815
1420173_at	DNA segment, Chr 11, ERATO Doi 326, expressed	D11Ertd326e	0.81333	1.0908708	0.12476083
1433366_at	RIKEN cDNA 6030458A19 gene	6030458A19Rik	0.81334	1.1110398	0.14180577
1455605_at	RUN and FYVE domain containing 3	Rufy3	0.81335	-1.08049881	-0.1136654
1422776_at	serine (or cysteine) peptidase inhibitor, clade B, member 8	Serpinb8	0.81337	-1.09069922	-0.1320914
1444935_at	---	---	0.81338	-1.11014452	-0.2017682
1459224_at	---	---	0.81339	-1.08761303	-0.1216104
1417944_at	guanine nucleotide binding protein (G protein), gamma 4	Gng4	0.81341	-1.08657976	-0.1214969
1417665_a_at	cleavage and polyadenylation specific factor 1	Cpsf1	0.81341	1.0619691	0.08521194
1435208_at	deltex 3-like (Drosophila)	Dtx3l	0.81342	1.08549466	0.11414266
1416278_a_at	ATP synthase, H+ transporting, mitochondrial F1 complex, O subu	Atp5o	0.81344	1.07647169	0.09764088
1458561_at	neuralized homolog 1A (Drosophila)	Neurl1a	0.81344	1.08712253	0.12026278
1441136_at	---	---	0.81345	-1.09038595	-0.125659
1417500_a_at	transglutaminase 2, C polypeptide	Tgm2	0.81345	1.07391827	0.10112082
1423604_at	neurocan	Ncan	0.81348	1.11246402	0.14396331
1450462_at	corticotropin releasing hormone receptor 2	Crhr2	0.81349	-1.08695443	-0.1209042
1458380_at	additional sex combs like 1 (Drosophila)	Asxl1	0.81349	-1.08431423	-0.1198569
1438437_a_at	RIKEN cDNA 4933439C10 gene	4933439C10Rik	0.8135	1.08242871	0.11386253
1421626_at	transmembrane channel-like gene family 1	Tmc1	0.8135	1.09398995	0.12649256
1417834_at	synaptojanin 2 binding protein	Synj2bp	0.81352	-1.06629356	-0.0933096
1419587_s_at	retinitis pigmentosa 2 homolog (human)	Rp2h	0.81352	1.10069462	0.13402588
1460424_at	transmembrane protein 160	Tmem160	0.81353	1.07320729	0.09804014
1442115_at	family with sequence similarity 38, member B	Fam38b	0.81353	-1.09841658	-0.1574793
1424175_at	thyrotroph embryonic factor	Tef	0.81354	1.07504901	0.10178479
1438546_x_at	solute carrier family 25 (mitochondrial carrier, adenine nucleotide	Slc25a5	0.81354	1.06509791	0.08890873
1420793_at	major urinary protein 4	Mup4	0.81354	1.08919296	0.11744608
1452432_at	tissue factor pathway inhibitor	Tfpi	0.81355	1.10694536	0.13575241
1419522_at	zinc finger, MYND domain containing 19	Zmynd19	0.81355	1.06853784	0.09354198
1440879_at	ATP-binding cassette, sub-family A (ABC1), member 9	Abca9	0.81355	-1.09063171	-0.125191
1437304_at	Casitas B-lineage lymphoma b	Cblb	0.81356	1.07951492	0.10827835
1450603_s_at	vomeronasal 1 receptor 47 /// vomeronasal 1 receptor 48	Vmn1r47 /// Vmn1r48	0.81356	1.09658923	0.1251664
1441587_at	phospholipase C, eta 2	Plch2	0.81358	1.09448271	0.12841218
1448274_at	complement component 1, q subcomponent binding protein	C1qbp	0.81358	1.0480456	0.06737727
1454558_at	RIKEN cDNA 5430416B10 gene	5430416B10Rik	0.81359	1.09746228	0.13096491
1452063_at	zinc finger and BTB domain containing 8a	Zbtb8a	0.8136	-1.06959844	-0.1020697
1416010_a_at	EH-domain containing 1	Ehd1	0.8136	-1.07088837	-0.1069057
1447170_at	---	---	0.8136	1.09322429	0.12476032
1448802_at	nuclear fragile X mental retardation protein interacting protein 1	Nufip1	0.81361	-1.0602638	-0.0857986
1434765_at	E1A binding protein p300	Ep300	0.81361	-1.06206432	-0.089024
1419524_at	tryptophan hydroxylase 1	Tph1	0.81364	1.10316311	0.13415117
1415740_at	protease (prosome, macropain) 26S subunit, ATPase 5	Psmc5	0.81365	1.05906802	0.08224541
1431012_a_at	peroxisomal delta3, delta2-enoyl-Coenzyme A isomerase	Peci	0.81368	1.06898639	0.09411634
1427063_at	RIKEN cDNA 5330417C22 gene	5330417C22Rik	0.81369	-1.092415	-0.1283895
1432684_at	RIKEN cDNA 4921521C08 gene	4921521C08Rik	0.81371	-1.09267193	-0.1372629
1424886_at	protein tyrosine phosphatase, receptor type, D	Ptprd	0.81371	-1.09613891	-0.139638
1446444_at	ring finger and CCCH-type zinc finger domains 2	Rc3h2	0.81371	-1.09105781	-0.1325779
1431275_at	chromatin assembly factor 1, subunit B (p60)	Chaf1b	0.81372	-1.08884942	-0.1245821
1427793_at	---	---	0.81387	-1.08309344	-0.1151902
1440205_at	zinc finger, MYND domain containing 19	Zmynd19	0.81394	1.10962174	0.13810086
1422159_at	protein phosphatase, EF hand calcium-binding domain 2	Ppef2	0.81394	-1.09168877	-0.1297806
1455664_at	reticulon 4 receptor-like 1	Rtn4rl1	0.81395	-1.09464834	-0.13131
1452583_s_at	galactose mutarotase	Galm	0.81396	1.07547177	0.10414686
1442676_at	monoamine oxidase A	Maoa	0.81397	-1.09676275	-0.1455426
1460102_at	CLIP associating protein 1	Clasp1	0.81401	1.09551108	0.1266435
1420546_at	tyrosine hydroxylase	Th	0.81402	1.09802854	0.13036424
1454045_a_at	phosphatidylglycerophosphate synthase 1	Pgs1	0.81402	1.06271458	0.08653904
1440323_at	synaptotagmin II	Syt2	0.81403	-1.09905401	-0.1413929
1432993_at	RIKEN cDNA 4930403L11 gene	4930403L11Rik	0.81406	1.13924641	0.16470016
1421799_at	urotensin 2	Uts2	0.81407	-1.10123313	-0.1540046
1457175_at	---	---	0.81408	-1.08756262	-0.1212973
1425864_a_at	VPS10 domain receptor protein SORCS 1	Sorcs1	0.81408	1.08852315	0.12210801
1419933_at	p53 and DNA damage regulated 1	Pdrg1	0.81409	1.09858791	0.1304803
1438520_at	solute carrier family 25, member 36	Slc25a36	0.81411	1.07722706	0.10689878
1424016_at	alpha- and gamma-adaptin binding protein	Aagab	0.81412	1.07094627	0.09666862
1424141_at	HECT domain containing 1	Hectd1	0.81413	1.07429059	0.09827358
1449436_s_at	ubiquitin pseudogene /// ubiquitin B	Gm1821 /// Ubb	0.81415	1.02876627	0.0406258
1446265_at	dynamin 3	Dnm3	0.81428	-1.08220189	-0.1158212
1429509_at	LSM12 homolog (S. cerevisiae)	Lsm12	0.81434	-1.06813667	-0.1005273
1432806_at	RIKEN cDNA 9430099H24 gene	9430099H24Rik	0.81436	-1.09880676	-0.1463233
1425385_a_at	Immunoglobulin heavy chain 6 (heavy chain of IgM)	Igh-6	0.81441	-1.08928741	-0.1244116
1441057_at	Myosin, heavy polypeptide 10, non-muscle	Myh10	0.81457	1.10535338	0.13788563

1416960_at	beta-1,3-glucuronyltransferase 3 (glucuronosyltransferase I)	B3gat3	0.81481	1.08916234	0.11478638
1456511_x_at	ES cell-expressed Ras	Eras	0.81483	1.07515407	0.09853683
1431706_at	importin 5	Ipo5	0.81485	1.09330509	0.1278561
1451080_at	ubiquitin specific peptidase 1	Usp1	0.81487	-1.06065258	-0.0882873
1425958_at	interleukin 1 family, member 9	Il1f9	0.81487	-1.09062689	-0.1266471
1447239_at	---	---	0.81488	1.11100074	0.14327941
1432825_at	RIKEN cDNA 2900018N21 gene	2900018N21Rik	0.81488	-1.09235896	-0.1294765
1446502_at	---	---	0.8149	1.07495071	0.10067416
1458794_at	---	---	0.8149	-1.09584164	-0.142271
1459159_a_at	coiled-coil domain containing 6	Ccdc6	0.81491	1.09557015	0.12382037
1430637_at	RIKEN cDNA 2210016H18 gene	2210016H18Rik	0.81491	1.10188544	0.13067534
1448844_at	cytochrome b5 type B	Cyb5b	0.81492	1.06338577	0.08745163
1460190_at	adaptor protein complex AP-1, mu 2 subunit	Ap1m2	0.81493	1.08228313	0.10978114
1422743_at	phosphorylase kinase alpha 1	Phka1	0.81493	1.09348889	0.12717298
1418714_at	dual specificity phosphatase 8	Dusp8	0.81494	-1.08839965	-0.1274512
1459635_at	---	---	0.81496	-1.11015083	-0.2232175
1451135_at	general transcription factor IIB	Gtf2b	0.81497	1.06519091	0.09054184
1458554_at	RIKEN cDNA 9330169B04 gene	9330169B04Rik	0.81497	-1.07700009	-0.1113258
1416154_at	signal recognition particle 54A /// signal recognition particle 54B	Srp54a /// Srp54b	0.81499	-1.0990616	-0.1434366
1436155_at	nicotinamide nucleotide adenylyltransferase 2	Nmnat2	0.815	1.08292967	0.11224485
1450459_at	RIKEN cDNA 2010106G01 gene	2010106G01Rik	0.81501	-1.09615449	-0.1528008
1441127_at	SLIT and NTRK-like family, member 2	Slitrk2	0.81506	-1.07987935	-0.1111254
1440810_x_at	---	---	0.81507	-1.08244641	-0.1149542
1436325_at	RAR-related orphan receptor alpha	Rora	0.81513	1.10965909	0.1386088
1446112_at	---	---	0.81515	1.09969494	0.12867498
1442789_at	---	---	0.81515	-1.08355044	-0.1171873
1444525_at	---	---	0.81519	-1.08832631	-0.1297048
1442909_at	---	---	0.8152	-1.09121479	-0.1275145
1423731_at	aldehyde dehydrogenase 16 family, member A1	Aldh16a1	0.81522	1.06729123	0.09325807
1427523_at	sine oculis-related homeobox 3 homolog (Drosophila)	Six3	0.81524	1.09200302	0.12456895
1416020_a_at	ATP synthase, H+ transporting, mitochondrial F0 complex, subunit	Atp5g1	0.81525	1.07221386	0.0947795
1417149_at	procollagen-proline, 2-oxoglutarate 4-dioxygenase (proline 4-hyd	P4ha2	0.81526	1.09097505	0.11981321
1455755_at	predicted gene 88	Gm88	0.8153	-1.09033888	-0.1306356
1455523_at	cleavage stimulation factor, 3' pre-RNA subunit 2	Cstf2	0.8154	-1.08021764	-0.1163209
1422736_at	RAN binding protein 9	Ranbp9	0.81544	1.07329443	0.09735248
1446025_at	---	---	0.81548	1.13590822	0.162371
1454170_at	RIKEN cDNA E130101E03 gene	E130101E03Rik	0.81549	1.09674562	0.1298435
1439967_at	RIKEN cDNA 1700071A11 gene	1700071A11Rik	0.81556	-1.08795654	-0.1234465
1444296_a_at	serine (or cysteine) peptidase inhibitor, clade A, member 4, pseud	Serpina4-ps1	0.81556	1.09206408	0.12430694
1424972_at	---	---	0.81559	1.09145389	0.1251502
1457238_at	---	---	0.81559	1.09433389	0.12694627
1447029_at	---	---	0.81561	1.11328378	0.14483794
1423741_at	RNA binding motif protein 10	Rbm10	0.81562	1.06921433	0.09504162
1428481_s_at	cell division cycle associated 8	Cdca8	0.81562	1.06005163	0.08344223
1438216_at	ras responsive element binding protein 1	Rreb1	0.81562	-1.1038994	-0.1645408
1424820_a_at	Nedd4 family interacting protein 1	Ndfip1	0.81563	-1.07760644	-0.1228004
1423701_at	Coenzyme A synthase	Coasy	0.81563	1.07751321	0.10501191
1430362_at	RIKEN cDNA 5730409N24 gene	5730409N24Rik	0.81563	-1.09426852	-0.1426668
1431129_at	ubiquitin specific peptidase 31	Usp31	0.81564	-1.09218707	-0.1344257
1441089_at	eukaryotic translation initiation factor 2C, 3	Eif2c3	0.81564	1.09992412	0.13291503
1432733_at	RIKEN cDNA 4933402E15 gene	4933402E15Rik	0.81565	-1.08738095	-0.1223896
1455202_at	mannosidase, endo-alpha-like	Maneal	0.81565	1.09295028	0.12115917
1443492_at	---	---	0.81565	-1.10155954	-0.157188
1435731_x_at	---	---	0.81566	-1.08771697	-0.1301593
1427072_at	START domain containing 8	Stard8	0.81566	1.1049012	0.13651096
1448537_at	tetratricopeptide repeat domain 1	Ttc1	0.81566	1.06906737	0.09370096
1432731_at	RIKEN cDNA 5830437K03 gene	5830437K03Rik	0.81567	-1.09354005	-0.1360004
1445632_at	oxoglutarate dehydrogenase (lipoamide)	Ogdh	0.81568	1.10312167	0.13464087
1435306_a_at	kinesin family member 11	Kif11	0.81568	-1.06459699	-0.0931607
1460432_a_at	eukaryotic translation initiation factor 3, subunit E	Eif3e	0.8157	-1.05027824	-0.0732361
1456990_at	hypothetical LOC100503134	LOC100503134	0.81574	-1.09216046	-0.1306249
1452689_at	zinc finger protein 512	Zfp512	0.81574	1.06402852	0.08827429
1431633_x_at	RIKEN cDNA 4930526L06 gene	4930526L06Rik	0.81577	1.10128456	0.13241782
1430408_at	calcium channel, voltage-dependent, P/Q type, alpha 1A subunit	Cacna1a	0.8158	1.10739516	0.13600632
1418065_at	recombination activating gene 2	Rag2	0.81583	1.09824904	0.13040799
1428353_at	forkhead box K2	Foxk2	0.81586	1.08941812	0.11505661
1455183_at	serine/threonine kinase 38 like	Stk38l	0.81586	1.0685937	0.09568392
1441538_at	---	---	0.81589	1.18618688	0.19774639
1434918_at	SRY-box containing gene 6	Sox6	0.81591	-1.08987211	-0.1314753
1446380_at	RIKEN cDNA 9430076C15 gene	9430076C15Rik	0.81593	-1.09004054	-0.1247285
1458743_at	---	---	0.81598	1.08043318	0.11089842
1425099_a_at	aryl hydrocarbon receptor nuclear translocator-like	Arntl	0.816	1.07988252	0.10625872
1447598_x_at	polymerase (RNA) I polypeptide A	Polr1a	0.81602	-1.08845781	-0.1470077
1454565_at	RIKEN cDNA C030046M01 gene	C030046M01Rik	0.81604	1.09887808	0.13096855
1424562_a_at	solute carrier family 25 (mitochondrial carrier, adenine nucleotide	Slc25a4	0.81605	1.06797828	0.09050764
1418851_at	tripartite motif-containing 39	Trim39	0.81607	1.09066857	0.11921251
1437132_x_at	neural precursor cell expressed, developmentally down-regulated	Nedd9	0.81607	-1.0968617	-0.1509887
1444587_at	expressed sequence AI481207	AI481207	0.81607	1.13192113	0.16067462
1453347_at	RIKEN cDNA 4930589P08 gene	4930589P08Rik	0.81607	-1.09341641	-0.1366259
1440725_at	G protein-coupled receptor 63	Gpr63	0.81609	-1.08127844	-0.1179659
1428553_at	glutaredoxin 5 homolog (S. cerevisiae)	Glrx5	0.8161	1.05751584	0.08053226
1452965_at	ankyrin repeat domain 13 family, member D	Ankrd13d	0.81611	1.09279343	0.12473741
1435582_at	WD repeat and FYVE domain containing 4	Wdfy4	0.81612	1.10107758	0.13361572
1434166_at	RIKEN cDNA 9330151L19 gene	9330151L19Rik	0.81613	1.08700024	0.11955147
1437323_a_at	islet amyloid polypeptide	Iapp	0.81613	-1.07892352	-0.1230006
1415712_at	zinc finger, RAN-binding domain containing 1	Zranb1	0.81614	-1.07620064	-0.1090009
1436291_a_at	dihydropyrimidinase	Dpys	0.81614	-1.07631393	-0.1149183
1460222_at	SH3-domain binding protein 1	Sh3bp1	0.81615	1.07165792	0.09843223
1426009_a_at	phosphatidylinositol-4-phosphate 5-kinase, type 1 alpha	Pip5k1a	0.81615	1.07559462	0.10127876
1420067_at	CD160 antigen	Cd160	0.81616	1.09779133	0.13010131
1418472_at	aspartoacylase	Aspa	0.81617	-1.10182428	-0.163991
1424863_a_at	homeodomain interacting protein kinase 2	Hipk2	0.81618	1.09021353	0.12088997
1416107_at	neuron specific gene family member 2	Nsg2	0.8162	1.08931983	0.12238231
1434712_at	Smith-Magenis syndrome chromosome region, candidate 7-like (	Smcr7l	0.81621	1.06708542	0.092325

1421017_at	neuregulin 3	Nrg3	0.81622	1.07505917	0.10182303
1452677_at	polyribonucleotide nucleotidyltransferase 1	Pnpt1	0.81622	-1.07951589	-0.114372
1449353_at	zinc finger matrin type 3	Zmat3	0.81623	-1.06737992	-0.0981558
1428465_at	transmembrane protein 147	Tmem147	0.81625	1.06493395	0.08841085
1435504_at	CAP-GLY domain containing linker protein family, member 4	Clip4	0.81625	-1.10301722	-0.1603022
1441291_at	c-abl oncogene 1, non-receptor tyrosine kinase	Abl1	0.81629	-1.09787752	-0.1463619
1421566_at	plasmacytoma expressed transcript 2	Pet2	0.81629	-1.08450442	-0.130995
1421745_at	pleiomorphic adenoma gene 1	Plag1	0.81629	1.13044877	0.157795
1417535_at	F-box protein 25	Fbxo25	0.81653	1.08394245	0.1128733
1453516_at	RIKEN cDNA 4931431F19 gene	4931431F19Rik	0.8166	1.13088132	0.15649735
1439005_x_at	hypothetical LOC100505062	LOC100505062	0.81666	-1.09273257	-0.1528754
1438149_at	PIF1 5'-to-3' DNA helicase homolog (S. cerevisiae)	Pif1	0.8168	-1.08754265	-0.1223196
1437213_at	nudix (nucleoside diphosphate linked moiety X)-type motif 21	Nudt21	0.81693	-1.07304135	-0.1080453
1451642_at	kinesin family member 1B	Kif1b	0.81693	-1.09626523	-0.1476791
1449112_at	solute carrier family 27 (fatty acid transporter), member 5	Slc27a5	0.81694	-1.089545	-0.1284466
1433432_x_at	ribosomal protein S12	Rps12	0.81694	-1.04196928	-0.0638684
1454828_at	G protein-coupled receptor 107	Gpr107	0.81695	1.08199477	0.10978862
1418540_a_at	protein tyrosine phosphatase, receptor type, E	Ptpre	0.81696	-1.09507119	-0.143611
1458363_at	zinc finger, DHHC domain containing 17	Zdhhc17	0.81696	-1.08723018	-0.1238845
1451798_at	interleukin 1 receptor antagonist	Il1rn	0.81704	1.13165499	0.15724775
1434586_a_at	phosphatidylserine synthase 2	Ptdss2	0.81706	1.07035187	0.09412559
1439633_at	synaptotagmin VII	Syt7	0.81712	-1.08091993	-0.1142391
1460244_at	ureidopropionase, beta	Upb1	0.81713	1.12065936	0.14786629
1432842_s_at	14-3-3 protein theta-like /// tyrosine 3-monooxygenase/tryptopl	LOC100503129 /// Ywhaq	0.81714	-1.05522654	-0.0852945
1429243_at	RIKEN cDNA 1110054O05 gene	1110054O05Rik	0.81714	1.07027672	0.09667493
1438133_a_at	cysteine rich protein 61	Cyr61	0.81715	1.19874353	0.20798879
1418909_at	erythroblast membrane-associated protein	Ermap	0.81717	1.13035305	0.1582929
1451914_a_at	adducin 2 (beta)	Add2	0.81719	-1.0932091	-0.1307601
1441517_at	---	---	0.81723	1.08346829	0.10837431
1420165_s_at	DnaJ (Hsp40) homolog, subfamily C, member 17	Dnajc17	0.81727	1.09370138	0.12093541
1445387_at	SUMO/sentrin specific peptidase 6	Senp6	0.81729	-1.08968329	-0.127181
1437489_x_at	succinate dehydrogenase complex, subunit D, integral membrane	Sdhd	0.8173	-1.07890075	-0.1241409
1420881_at	nuclear receptor-binding SET-domain protein 1	Nsd1	0.81733	-1.09085968	-0.1365566
1459856_at	---	---	0.81733	-1.09958341	-0.1579223
1458684_at	---	---	0.81734	-1.08673018	-0.1293038
1436077_a_at	FCH domain only 1	Fcho1	0.81734	1.09403341	0.12118088
1454116_a_at	MTERF domain containing 1	Mterfd1	0.81734	-1.0628538	-0.088015
1429894_a_at	microtubule-associated protein 7	Mtap7	0.81735	1.11298701	0.14425325
1432918_at	RIKEN cDNA 4921511E18 gene	4921511E18Rik	0.81736	-1.09467745	-0.1386968
1446557_at	RIKEN cDNA E130309F12 gene	E130309F12Rik	0.81736	1.0893929	0.12120001
1436189_at	NAD(P)H dehydrogenase, quinone 2	Nqo2	0.81736	-1.09194986	-0.1347252
1456742_x_at	transmembrane 9 superfamily member 2	Tm9sf2	0.81737	-1.10052435	-0.1548465
1430311_at	Myristoylated alanine rich protein kinase C substrate	Marcks	0.81738	1.12001388	0.14517038
1445192_at	DNA segment, Chr 8, ERATO Doi 575, expressed	D8Ertd575e	0.81738	-1.07997441	-0.123165
1456149_at	DNA segment, Chr 11, Brigham & Women's Genetics 0517 expres	D11Bwg0517e	0.81738	1.11506354	0.14182392
1431738_at	RIKEN cDNA 4921531P14 gene	4921531P14Rik	0.81739	-1.09791261	-0.1500506
1455150_at	HECT, C2 and WW domain containing E3 ubiquitin protein ligase 2	Hecw2	0.81739	1.09192166	0.12019244
1448981_x_at	T-cell leukemia/lymphoma 1B, 1	Tcl1b1	0.81741	-1.08531228	-0.1181782
1440714_at	---	---	0.81744	1.11672059	0.14136652
1454561_at	RIKEN cDNA 9430087B13 gene	9430087B13Rik	0.81745	-1.08651872	-0.1206437
1416849_at	CDGSH iron sulfur domain 1	Cisd1	0.81748	-1.07208639	-0.1120034
1454158_at	membrane protein, palmitoylated 7 (MAGUK p55 subfamily mem	Mpp7	0.81752	-1.09584024	-0.154478
1440192_at	tetratricopeptide repeat domain 39B	Ttc39b	0.81765	-1.08473606	-0.1236077
1436850_at	cellular repressor of E1A-stimulated genes 2	Creg2	0.81774	1.09244751	0.12300736
1445910_at	expressed sequence C79999	C79999	0.81777	-1.09696268	-0.1389214
1433230_at	RIKEN cDNA 5730415C11 gene	5730415C11Rik	0.81786	-1.09618591	-0.1421193
1433325_at	RIKEN cDNA 9330154F10 gene	9330154F10Rik	0.81811	-1.07489757	-0.1046481
1444370_at	expressed sequence C77058	C77058	0.81816	-1.09432681	-0.1311486
1432526_a_at	SNF8, ESCRT-II complex subunit, homolog (S. cerevisiae)	Snf8	0.81816	1.06181231	0.08561665
1449108_at	ferredoxin 1	Fdx1	0.81817	1.07908569	0.10377678
1421697_at	Usher syndrome 2A (autosomal recessive, mild) homolog (humar	Ush2a	0.81818	-1.09071202	-0.1286345
1424388_at	clusterin associated protein 1	Cluap1	0.8182	1.08044678	0.10847237
1416906_at	anaphase-promoting complex subunit 5	Anapc5	0.81821	1.06369977	0.08634011
1458997_at	IgLON family member 5	Iglon5	0.81825	-1.0845411	-0.1187217
1417588_at	UDP-N-acetyl-alpha-D-galactosamine:polypeptide N-acetylgalact	Galnt3	0.81826	1.08510726	0.11714233
1426122_a_at	coronin 6	Coro6	0.8183	1.09524449	0.13049968
1439041_at	solute carrier family 39 (zinc transporter), member 10	Slc39a10	0.81831	1.10891628	0.13276812
1433173_at	RIKEN cDNA 5430440L12 gene	5430440L12Rik	0.81831	1.10097238	0.12752464
1448934_at	NADH dehydrogenase (ubiquinone) 1 alpha subcomplex 10	Ndufa10	0.81831	-1.05730067	-0.0804251
1426008_a_at	solute carrier family 7 (cationic amino acid transporter, y+ system	Slc7a2	0.81832	-1.09106438	-0.1394903
1457035_at	expressed sequence AI607873	AI607873	0.81832	1.06005239	0.0828813
1446864_at	S100P binding protein	S100pbp	0.81833	-1.07547005	-0.1148533
1420499_at	GTP cyclohydrolase 1	Gch1	0.81836	1.14196169	0.16759831
1453484_at	phosphatidylethanolamine binding protein 4	Pebp4	0.81836	1.11415181	0.1437123
1442088_at	RIKEN cDNA 9230111E07 gene	9230111E07Rik	0.81836	1.09970606	0.13404002
1416959_at	nuclear receptor subfamily 1, group D, member 2	Nr1d2	0.81837	-1.1026383	-0.1653202
1445764_at	hypothetical LOC100502602	LOC100502602	0.81838	-1.08656505	-0.1218791
1436707_x_at	non-SMC condensin I complex, subunit H	Ncaph	0.81838	1.07266426	0.09745083
1435184_at	natriuretic peptide receptor 3	Npr3	0.8184	-1.080831	-0.1131888
1426227_s_at	vacuolar protein sorting 37C (yeast)	Vps37c	0.8184	1.10352267	0.12949372
1432961_at	RIKEN cDNA 2610024D14 gene	2610024D14Rik	0.81842	1.07668866	0.1027381
1417055_at	RIKEN cDNA 0610009D07 gene	0610009D07Rik	0.81843	-1.06108581	-0.0876461
1421501_a_at	ELKS/RAB6-interacting/CAST family member 1	Erc1	0.81846	-1.0815517	-0.1167091
1424380_at	vacuolar protein sorting 37B (yeast)	Vps37b	0.81848	1.08037914	0.11140795
1437658_a_at	small nucleolar RNA host gene (non-protein coding) 1	Snhg1	0.8185	-1.06986548	-0.0992445
1432448_at	RIKEN cDNA 2600006K01 gene	2600006K01Rik	0.81851	-1.09677353	-0.1466639
1457606_x_at	expressed sequence AU015228	AU015228	0.81851	1.11351364	0.14368274
1428724_at	cleavage and polyadenylation factor subunit homolog (S. cerevisi	Pcf11	0.81851	-1.09256353	-0.1311426
1442522_at	---	---	0.81856	-1.06615478	-0.0929716
1441197_at	RIKEN cDNA 9530059O14 gene	9530059O14Rik	0.81856	-1.08565769	-0.1190271
1431632_at	RIKEN cDNA 4930526L06 gene	4930526L06Rik	0.81857	1.10070467	0.13417941
1426449_a_at	praja1, RING-H2 motif containing	Pja1	0.81857	1.07353423	0.10008114
1439046_at	coiled-coil domain containing 55	Ccdc55	0.81857	-1.06806253	-0.0964474
1440906_at	---	---	0.81858	1.08161238	0.11093903

1443796_at	interleukin 16	Il16	0.81858	-1.08905446	-0.1252093
1429515_at	ubiquitin protein ligase E3 component n-recognin 2	Ubr2	0.81859	1.07078915	0.09828201
1435337_at	teashirt zinc finger family member 3	Tshz3	0.81859	-1.10124609	-0.1512571
1423119_at	radial spoke 3A homolog (Chlamydomonas) /// radial spoke 3B h	Rsph3a /// Rsph3b	0.81861	1.07482228	0.10349754
1428067_at	RAS-like, family 12	Rasl12	0.81862	1.09255532	0.12660481
1431519_at	RIKEN cDNA 4933406L23 gene	4933406L23Rik	0.81862	-1.08506983	-0.1194366
1446784_at	---	---	0.81863	-1.08109353	-0.1193933
1451116_at	acetoacetyl-CoA synthetase	Aacs	0.81864	-1.08335479	-0.1160666
1453331_at	RIKEN cDNA 1700013H16 gene	1700013H16Rik	0.81864	1.07288379	0.09947546
1451962_at	immunoglobulin kappa chain variable 19 (V19)-20	Igk-V19-20	0.81865	1.12192258	0.14686291
1437423_a_at	steroid receptor RNA activator 1	Sra1	0.81866	1.0638904	0.08794886
1449560_at	keratin associated protein 1-5	Krtap1-5	0.81867	-1.09813722	-0.153243
1435475_at	lectin, mannose-binding 2-like	Lman2l	0.81868	1.06763246	0.09366956
1452641_at	YjeF N-terminal domain containing 3	Yjefn3	0.81869	1.12592752	0.15041387
1443895_at	predicted gene 6225	Gm6225	0.8187	-1.08658189	-0.121617
1434415_at	dapper homolog 3, antagonist of beta-catenin (xenopus)	Dact3	0.81871	1.07207801	0.09829087
1456248_at	late cornified envelope 3F	Lce3f	0.81873	1.15207608	0.17353444
1438632_x_at	transition protein 1	Tnp1	0.81873	1.10291961	0.13108356
1415798_at	discoidin domain receptor family, member 1	Ddr1	0.81873	1.10014898	0.12838349
1427574_s_at	SH3 domain protein D19	Sh3d19	0.81874	-1.08391391	-0.1169332
1441180_at	RIKEN cDNA C330024D21 gene	C330024D21Rik	0.81875	1.08705242	0.1147253
1422123_s_at	carcinoembryonic antigen-related cell adhesion molecule 1 /// ca	Ceacam1 /// Ceacam2	0.81878	1.15521681	0.16923461
1444951_at	DENN/MADD domain containing 1B	Dennd1b	0.81879	-1.0952795	-0.1429399
1420498_a_at	disabled homolog 2 (Drosophila)	Dab2	0.8188	1.07510409	0.1035185
1444604_at	---	---	0.81881	-1.0920584	-0.1299035
1430197_a_at	phosphatidylinositol transfer protein, membrane-associated 2	Pitpnm2	0.81886	1.09041359	0.12343875
1424024_at	multiple coagulation factor deficiency 2	Mcfd2	0.81899	1.05905895	0.08270534
1442065_at	Rho GTPase activating protein 17	Arhgap17	0.81905	-1.08666144	-0.1202764
1431568_at	RIKEN cDNA 4933427E13 gene	4933427E13Rik	0.81921	-1.0775828	-0.1080908
1436573_at	secernin 3	Scrn3	0.81922	1.07041397	0.09711664
1459536_at	Calcitonin receptor-like	Calcrl	0.81925	-1.08040264	-0.1210821
1444442_at	oral cancer overexpressed 1	Oraov1	0.81927	1.11333238	0.14439997
1440419_at	predicted gene 1679	Gm1679	0.81929	1.08931171	0.12299684
1436651_at	PR domain containing 11	Prdm11	0.81929	1.10986555	0.13952916
1438421_at	poliovirus receptor-related 1	Pvrl1	0.8193	-1.1093177	-0.2202593
1449634_a_at	ankyrin repeat and sterile alpha motif domain containing 1B	Anks1b	0.8193	1.10989018	0.13882106
1421023_at	phosphatidylinositol 3-kinase, C2 domain containing, alpha polype	Pik3c2a	0.81931	-1.06914277	-0.0998962
1417226_at	F-box and WD-40 domain protein 4	Fbxw4	0.81932	1.0790583	0.10951699
1458476_at	eukaryotic translation initiation factor 2C, 3	Eif2c3	0.81936	-1.08958209	-0.1286172
1420102_at	---	---	0.81936	-1.10142099	-0.1667986
1434655_at	forkhead box K1	Foxk1	0.81942	1.12613791	0.14979127
1428134_at	coenzyme Q9 homolog (yeast)	Coq9	0.81942	1.07361785	0.10087167
1422318_at	forkhead box D4	Foxd4	0.81943	1.09274568	0.12500117
1456184_at	transmembrane protein 63c	Tmem63c	0.81949	-1.0933984	-0.1337675
1460565_at	solute carrier family 41, member 1	Slc41a1	0.81953	1.06850914	0.09499307
1437808_x_at	interleukin 10 receptor, alpha	Il10ra	0.81955	-1.09293272	-0.133848
1426886_at	ceroid-lipofuscinosis, neuronal 5	Cln5	0.81956	1.06274731	0.08657106
1458030_at	---	---	0.81957	-1.07410013	-0.1032463
1430267_at	RIKEN cDNA 4833422M21 gene	4833422M21Rik	0.81958	-1.096101	-0.1394317
1437838_x_at	G-rich RNA sequence binding factor 1	Grsf1	0.81958	-1.07300859	-0.1161207
1456011_x_at	acetyl-Coenzyme A acyltransferase 1A	Acaa1a	0.81958	1.15141918	0.17159175
1442117_at	---	---	0.81959	1.11422912	0.14504087
1418658_at	family with sequence similarity 82, member B	Fam82b	0.81959	-1.08616471	-0.1206043
1448569_at	malectin	Mlec	0.81961	1.07470861	0.10230156
1444362_at	neurogenic differentiation 2	Neurod2	0.81962	-1.08379644	-0.1191861
1437378_x_at	scavenger receptor class B, member 1	Scarb1	0.81962	1.10778551	0.13298677
1436915_x_at	lysosomal-associated protein transmembrane 4B	Laptm4b	0.81963	-1.07233327	-0.1124286
1456192_x_at	cysteine-rich perinuclear theca 3	Cypt3	0.81964	1.09801596	0.13095527
1456743_x_at	mortality factor 4 like 2	Morf4l2	0.81964	-1.0462722	-0.0682106
1435842_at	N-acetyltransferase 8-like	Nat8l	0.81964	-1.08649125	-0.1212605
1443907_at	leucyl/cystinyl aminopeptidase	Lnpep	0.81965	-1.07135732	-0.099762
1445358_at	tankyrase, TRF1-interacting ankyrin-related ADP-ribose polymera	Tnks	0.81966	1.11370394	0.14335548
1460402_at	bromodomain and PHD finger containing, 1	Brpf1	0.81968	-1.06904814	-0.0968058
1426530_a_at	kelch-like 5 (Drosophila)	Klhl5	0.81968	-1.08012113	-0.1126969
1418144_a_at	phosphatidylinositol-4-phosphate 5-kinase, type 1 alpha	Pip5k1a	0.81968	-1.06634068	-0.0932392
1453057_at	family with sequence similarity 187, member B	Fam187b	0.81971	1.10795502	0.13881666
1454006_a_at	UBX domain protein 8	Ubxn8	0.81971	1.08314613	0.11048985
1418193_at	max binding protein	Mnt	0.81972	-1.08096777	-0.1139878
1448326_a_at	cellular retinoic acid binding protein I	Crabp1	0.81972	1.15855534	0.17696181
1459756_at	CCR4-NOT transcription complex, subunit 10	Cnot10	0.81974	-1.0744705	-0.1062743
1425392_a_at	nuclear receptor subfamily 1, group I, member 3	Nr1i3	0.81974	1.09446408	0.12779781
1445728_at	retinol dehydrogenase 16	Rdh16	0.81975	1.13582475	0.15899779
1446260_at	RIKEN cDNA 4930447C04 gene	4930447C04Rik	0.81976	-1.09393939	-0.1413864
1429190_at	arylsulfatase B	Arsb	0.81976	1.08554547	0.11773276
1451948_at	predicted gene 1409	Gm1409	0.81976	1.10008451	0.12569355
1454376_at	RIKEN cDNA 4833410I11 gene	4833410I11Rik	0.8198	1.11068574	0.13975957
1433897_at	expressed sequence AI597468	AI597468	0.81983	-1.06262021	-0.088026
1415920_at	cleavage stimulation factor, 3' pre-RNA subunit 2, tau	Cstf2t	0.81991	-1.05818994	-0.0835439
1452667_at	RAB2B, member RAS oncogene family	Rab2b	0.81992	1.0827224	0.11240402
1457356_at	hypothetical LOC100502594	LOC100502594	0.81992	-1.08557153	-0.1197451
1420413_at	solute carrier family 7 (cationic amino acid transporter, y+ system	Slc7a11	0.81993	1.08331398	0.11510806
1426640_s_at	tribbles homolog 2 (Drosophila)	Trib2	0.81994	1.084785	0.11521655
1425428_at	hypoxia inducible factor 3, alpha subunit	Hif3a	0.81994	1.07194061	0.09949808
1440319_at	RNA binding motif protein 44	Rbm44	0.81995	1.12450441	0.15170804
1455788_x_at	polymerase (DNA-directed), delta interacting protein 3	Poldip3	0.81995	-1.0613117	-0.0890884
1451801_at	triadin	Trdn	0.81996	1.07569887	0.10200626
1445865_at	---	---	0.81996	1.08422924	0.11578481
1454033_at	protein disulfide isomerase-like, testis expressed	Pdilt	0.81997	-1.09554647	-0.1484729
1421464_at	NK3 homeobox 2	Nkx3-2	0.81997	-1.08404948	-0.1189966
1436390_a_at	chloride channel CLIC-like 1	Clcc1	0.81997	-1.09555812	-0.1545781
1434703_at	exostoses (multiple)-like 3	Extl3	0.81997	1.07601036	0.10189989
1428582_at	methyltransferase like 10	Mettl10	0.81997	1.0771487	0.10511232
1420493_a_at	phosphate cytidylyltransferase 2, ethanolamine	Pcyt2	0.81997	1.06855472	0.09461119
1450209_at	homeobox D4	Hoxd4	0.81998	-1.08526984	-0.1232774

1441090_at	Nuclear mitotic apparatus protein 1	Numa1	0.81998	-1.11180853	-0.1890439
1431610_at	RIKEN cDNA 5330439A09 gene	5330439A09Rik	0.81999	-1.08353515	-0.1188777
1418589_a_at	myeloid leukemia factor 1	Mlf1	0.81999	-1.08725354	-0.121545
1432219_at	nucleolar protein 9	Nol9	0.81999	-1.08966835	-0.1306342
1430404_at	hypothetical LOC75771	LOC75771	0.82	1.11843026	0.14721619
1454282_at	RIKEN cDNA 1700084C06 gene	1700084C06Rik	0.82	-1.08526047	-0.1199355
1450272_at	tumor necrosis factor (ligand) superfamily, member 8	Tnfsf8	0.82	-1.08551496	-0.1185026
1447139_at	B-cell CLL/lymphoma 7C	Bcl7c	0.82	-1.09077168	-0.1305437
1443988_at	RNA binding motif protein 39	Rbm39	0.82001	-1.08679036	-0.121246
1421195_at	cholecystokinin A receptor	Cckar	0.82001	-1.08396956	-0.1177347
1435286_at	Eph receptor A5	Epha5	0.82002	1.07951447	0.10633163
1416614_at	EP300 interacting inhibitor of differentiation 1	Eid1	0.82002	-1.07408122	-0.1078738
1459657_s_at	polymerase (RNA) I polypeptide D	Polr1d	0.82002	1.06265607	0.0868631
1448464_at	YKT6 homolog (S. Cerevisiae)	Ykt6	0.82002	1.06853963	0.09213114
1444789_at	---	---	0.82002	1.09976902	0.12974029
1433344_at	glyceraldehyde-3-phosphate dehydrogenase pseudogene	4930448K20Rik	0.82002	1.08604679	0.11787543
1448594_at	WNT1 inducible signaling pathway protein 1	Wisp1	0.82002	1.10340128	0.13577628
1450591_at	olfactory receptor 154	Olfr154	0.82002	-1.08635652	-0.1210689
1416196_at	ribosomal protein SA	Rpsa	0.82003	1.10122123	0.13108241
1447579_at	Reticulon 4 interacting protein 1	Rtn4ip1	0.82003	-1.06854678	-0.0968837
1426879_at	RIKEN cDNA 1190005F20 gene	1190005F20Rik	0.82003	1.09926894	0.12419306
1427959_at	abhydrolase domain containing 10	Abhd10	0.82005	1.06316686	0.08793276
1421141_a_at	forkhead box P1	Foxp1	0.82005	-1.09239113	-0.1656537
1452160_at	TCDD-inducible poly(ADP-ribose) polymerase	Tiparp	0.82005	-1.07392751	-0.110429
1440746_at	---	---	0.82006	-1.09360877	-0.140214
1421368_at	scratch homolog 1, zinc finger protein (Drosophila)	Scrt1	0.82007	1.12625079	0.15619361
1446883_at	RIKEN cDNA 4933427D06 gene	4933427D06Rik	0.82007	1.07846507	0.1072894
1450710_at	jumonji, AT rich interactive domain 2	Jarid2	0.82007	-1.06208443	-0.0905719
1441686_at	---	---	0.82007	-1.08832317	-0.1385102
1459748_at	---	---	0.82007	-1.08778853	-0.1389416
1424032_at	hydrogen voltage-gated channel 1	Hvcn1	0.82007	1.073272	0.10023878
1456557_at	coiled-coil domain containing 30	Ccdc30	0.82007	1.08353516	0.11446246
1450765_a_at	phosphodiesterase 6H, cGMP-specific, cone, gamma	Pde6h	0.82007	-1.08956069	-0.1294253
1438002_at	---	---	0.82007	-1.08091356	-0.1143489
1423029_at	hairy and enhancer of split 2 (Drosophila)	Hes2	0.82008	1.10155827	0.1331135
1440425_at	---	---	0.82008	1.12911034	0.15459568
1443357_at	Zinc finger RNA binding protein	Zfr	0.82009	-1.08590979	-0.126468
1447000_at	---	---	0.8201	-1.09350864	-0.1369099
1430164_a_at	growth factor receptor bound protein 10	Grb10	0.8201	-1.09560657	-0.1477893
1430877_at	RIKEN cDNA 8030425K09 gene	8030425K09Rik	0.8201	-1.09791481	-0.1507978
1417932_at	interleukin 18	Il18	0.8201	-1.08778959	-0.1225516
1431634_at	RIKEN cDNA 4930455C13 gene	4930455C13Rik	0.82011	-1.08728126	-0.121537
1417034_at	trafficking protein particle complex 6A	Trappc6a	0.82011	1.09832165	0.12006981
1455001_x_at	ribosomal protein L13A	Rpl13a	0.82011	1.0345882	0.04905193
1457006_at	RIKEN cDNA 1700106J12 gene	1700106J12Rik	0.82012	-1.07671795	-0.1120377
1433453_a_at	ankyrin repeat and BTB (POZ) domain containing 2	Abtb2	0.82013	1.09959238	0.13082521
1460211_a_at	KDEL (Lys-Asp-Glu-Leu) endoplasmic reticulum protein retention r	Kdelr1	0.82013	1.08057107	0.10468463
1436629_at	transmembrane protein 91	Tmem91	0.82013	1.1263891	0.15346165
1419893_at	RIKEN cDNA D930048N14 gene	D930048N14Rik	0.82013	1.08519513	0.11420963
1423103_at	regulatory factor X, 5 (influences HLA class II expression)	Rfx5	0.82013	1.08130648	0.10987318
1459294_at	mitochondrial intermediate peptidase	Mipep	0.82013	-1.08853547	-0.1224344
1421994_a_at	HCLS1 binding protein 3	Hs1bp3	0.82013	1.07548261	0.1041188
1454239_at	RIKEN cDNA 4930578G10 gene	4930578G10Rik	0.82014	-1.09085898	-0.1256492
1449596_at	expressed sequence C78142	C78142	0.82015	1.09866798	0.12554642
1426487_a_at	retinoblastoma binding protein 6	Rbbp6	0.82016	-1.06855149	-0.0985957
1459580_at	RIKEN cDNA G630055G22 gene	G630055G22Rik	0.82016	1.07833966	0.10573876
1446277_at	---	---	0.82016	-1.08612963	-0.1197511
1430625_at	keratin associated protein 3-2	Krtap3-2	0.82017	-1.07685313	-0.1168634
1435741_at	phosphodiesterase 8B	Pde8b	0.82017	-1.08246682	-0.1154534
1439409_x_at	tyrosinase-related protein 1	Tyrp1	0.82017	1.09053698	0.12356479
1451928_a_at	RAD18 homolog (S. cerevisiae)	Rad18	0.82018	1.09811717	0.12335148
1443474_at	DNA segment, Chr 7, ERATO Doi 495, expressed	D7Ertd495e	0.82018	-1.0913213	-0.1336216
1455809_x_at	resistance to inhibitors of cholinesterase 8 homolog (C. elegans)	Ric8	0.82018	-1.08881233	-0.1489912
1424115_at	protein phosphatase 5, catalytic subunit	Ppp5c	0.82018	1.06706021	0.0915038
1456400_at	telomeric repeat binding factor 2, interacting protein	Terf2ip	0.82019	1.08128561	0.11181187
1430634_a_at	phosphofructokinase, platelet	Pfkp	0.82019	1.07960251	0.10249446
1418486_at	vanin 1	Vnn1	0.8202	-1.08463756	-0.1237108
1436396_at	WD repeat domain 60	Wdr60	0.8202	1.09463897	0.12347444
1460564_at	zinc finger protein 280B	Zfp280b	0.8202	1.09349708	0.11782772
1431485_at	RIKEN cDNA 4833447I15 gene	4833447I15Rik	0.8202	1.09204314	0.11937334
1458376_at	RIKEN cDNA B930025B16 gene	B930025B16Rik	0.82021	1.09211925	0.12426573
1429908_at	small nuclear ribonucleoprotein 48 (U11/U12)	Snrnp48	0.82021	-1.11429109	-0.2391685
1416565_at	cytochrome c oxidase, subunit VIb polypeptide 1	Cox6b1	0.82021	1.05574153	0.0766536
1435120_at	---	---	0.82021	-1.05908811	-0.0856019
1448365_at	exosome component 7	Exosc7	0.82021	1.05746941	0.07993644
1437789_at	baculoviral IAP repeat-containing 6	Birc6	0.82022	1.15500621	0.17516991
1452893_s_at	energy homeostasis associated	Enho	0.82022	1.09744851	0.12827274
1418361_at	growth arrest specific 8	Gas8	0.82023	1.08870027	0.11733162
1441956_s_at	Cut-like homeobox 1	Cux1	0.82023	1.19556668	0.20165273
1427499_at	zinc finger protein 81	Zfp81	0.82023	1.22596572	0.22258189
1460730_at	eukaryotic translation initiation factor 2B, subunit 1 (alpha)	Eif2b1	0.82023	1.05886237	0.0819606
1440307_at	general transcription factor IIIC, polypeptide 2, beta	Gtf3c2	0.82024	-1.08144787	-0.1140142
1426532_at	zinc finger, MYND domain containing 11	Zmynd11	0.82024	1.08601448	0.11266174
1452627_at	SUMO/sentrin specific peptidase 6	Senp6	0.82024	1.06709659	0.09199064
1444764_at	---	---	0.82024	-1.08715011	-0.1223338
1435613_x_at	cytochrome c oxidase, subunit Vb	Cox5b	0.82025	1.04487556	0.06205896
1434161_at	lin-52 homolog (C. elegans)	Lin52	0.82025	-1.08145537	-0.1196996
1429662_at	spermatogenesis associated 1	Spata1	0.82025	1.10542686	0.13657921
1438964_x_at	TCF3 (E2A) fusion partner	Tfpt	0.82025	1.08700045	0.11344759
1452731_x_at	predicted gene, ENSMUSG00000068790 /// alpha-takusan pseud	ENSMUSG00000068790 /	0.82025	1.0671962	0.09366749
1450023_at	GTP binding protein 1	Gtpbp1	0.82026	1.07118193	0.09553606
1420307_a_at	phosphatidylinositol transfer protein, beta	Pitpnb	0.82026	1.06219441	0.08659076
1455988_a_at	chaperonin containing Tcp1, subunit 6a (zeta)	Cct6a	0.82026	-1.04371899	-0.0644115
1432354_at	RIKEN cDNA 1700003H04 gene	1700003H04Rik	0.82026	-1.07965088	-0.1139132

1455501_at	solute carrier family 2 (facilitated glucose transporter), member 1	Slc2a12	0.82026	-1.09406659	-0.1364426
1424515_at	RIKEN cDNA B230354K17 gene	B230354K17Rik	0.82026	1.06504065	0.08838013
1416988_at	mutS homolog 2 (E. coli)	Msh2	0.82027	1.06200641	0.08549749
1450476_at	cannabinoid receptor 2 (macrophage)	Cnr2	0.82027	1.09633303	0.12903344
1443765_at	RIKEN cDNA 1810058N05 gene	1810058N05Rik	0.82028	-1.08886282	-0.1282651
1459436_at	---	---	0.82028	1.09428339	0.12702021
1421152_a_at	guanine nucleotide binding protein, alpha O	Gnao1	0.82028	1.08555986	0.11610243
1439463_x_at	predicted gene 2710 /// high-mobility group (nonhistone chromo	Gm2710 /// Hmg1l1 /// H	0.82028	-1.0617779	-0.0943461
1453094_at	forkhead box N3	Foxn3	0.82029	1.07409954	0.10248021
1453471_at	isoamyl acetate-hydrolyzing esterase 1 homolog (S. cerevisiae)	Iah1	0.82029	-1.08865178	-0.1235843
1440228_at	RAN binding protein 6	Ranbp6	0.8203	-1.09606484	-0.1504883
1448635_at	structural maintenance of chromosomes 2	Smc2	0.8203	-1.05977186	-0.0843972
1444291_at	LysM, putative peptidoglycan-binding, domain containing 4	Lysmd4	0.8203	1.11360642	0.14410789
1457969_at	RAB interacting factor	Rabif	0.8203	-1.0967971	-0.1445263
1446346_at	---	---	0.8203	-1.08128813	-0.1134084
1425212_a_at	tumor necrosis factor receptor superfamily, member 19	Tnfrsf19	0.82031	1.08418999	0.1155222
1453339_at	RIKEN cDNA 1700008I05 gene	1700008I05Rik	0.82031	-1.09152929	-0.1356585
1453378_at	IKAROS family zinc finger 3	Ikzf3	0.82032	-1.09576448	-0.1539717
1455448_at	diacylglycerol lipase, alpha	Dagla	0.82032	-1.09264545	-0.135303
1459233_at	CLIP associating protein 1	Clasp1	0.82032	-1.08879904	-0.1230588
1451538_at	SRY-box containing gene 9	Sox9	0.82032	1.07489026	0.10221665
1422046_at	integrin alpha M	Itgam	0.82033	1.14443057	0.168151
1448240_at	membrane-bound transcription factor peptidase, site 1	Mbtps1	0.82033	1.06027667	0.08409289
1446581_at	---	---	0.82034	1.05501187	0.07707216
1459824_at	SWI/SNF related, matrix associated, actin dependent regulator of	Smarcc1	0.82034	-1.08961301	-0.1264991
1433702_at	endoplasmic reticulum metallopeptidase 1	Ermp1	0.82034	1.06388294	0.08892622
1431281_at	dysferlin interacting protein 1	Dysfip1	0.82035	1.09211354	0.12321949
1458673_at	RIKEN cDNA 1700093J21 gene	1700093J21Rik	0.82035	1.10043088	0.13306696
1459261_at	SRY-box containing gene 5	Sox5	0.82035	1.08084872	0.1081751
1444124_a_at	RIKEN cDNA E330017L17 gene	E330017L17Rik	0.82035	-1.09180778	-0.1332696
1449162_at	processing of precursor 7, ribonuclease P family, (S. cerevisiae)	Pop7	0.82035	1.07605497	0.10346721
1441813_at	predicted gene 9484	Gm9484	0.82035	1.08235918	0.10998044
1426031_a_at	nuclear factor of activated T-cells, cytoplasmic, calcineurin-depen	Nfatc2	0.82036	-1.09534776	-0.1443205
1452349_x_at	interferon activated gene 205 /// myeloid cell nuclear differentiat	Ifi205 /// Mnda	0.82036	-1.07593499	-0.1057224
1442583_a_at	expressed sequence AI854703	AI854703	0.82036	1.09134785	0.12434733
1452250_a_at	collagen, type VI, alpha 2	Col6a2	0.82036	1.08486721	0.11572916
1442871_at	---	---	0.82036	-1.08011001	-0.1137973
1416716_at	embryonal Fyn-associated substrate	Efs	0.82036	1.07041523	0.09762223
1435488_at	methylenetetrahydrofolate dehydrogenase (NADP+ dependent)	Mthfd2l	0.82037	1.15383593	0.17183798
1433688_x_at	ribosomal protein L14	Rpl14	0.82037	1.03969052	0.05522582
1422354_at	olfactory receptor 544	Olfr544	0.82037	-1.08803456	-0.12346
1434733_at	serine/threonine kinase 36 (fused homolog, Drosophila)	Stk36	0.82038	1.12421158	0.15264645
1436088_at	family with sequence similarity 49, member B	Fam49b	0.82038	1.07379696	0.10023948
1452055_at	CTD (carboxy-terminal domain, RNA polymerase II, polypeptide A	Ctdsp1	0.82038	1.07923466	0.10405406
1448581_at	zinc finger protein 346	Zfp346	0.82038	1.0772038	0.10437999
1449767_x_at	synaptotagmin VI	Syt6	0.82038	1.08224915	0.11075413
1450916_at	staufen (RNA binding protein) homolog 2 (Drosophila)	Stau2	0.82038	-1.09224961	-0.13583
1448423_at	transmembrane emp24 protein transport domain containing 4	Tmed4	0.82038	1.07645328	0.10507961
1423915_at	olfactomedin-like 2B	Olfml2b	0.82039	1.08917891	0.11802733
1426913_at	---	---	0.82039	1.08115075	0.11127455
1425774_at	serine/arginine repetitive matrix 4	Srrm4	0.82039	-1.092951	-0.1344038
1417648_s_at	sorting nexin 5	Snx5	0.82039	1.06401911	0.08786149
1429750_at	multiple inositol polyphosphate histidine phosphatase 1	Minpp1	0.82039	-1.08728304	-0.1216859
1429985_at	RIKEN cDNA 5430439G13 gene	5430439G13Rik	0.82039	-1.08724868	-0.1354939
1427984_at	SUMO/sentrin specific peptidase 6	Senp6	0.8204	-1.10163523	-0.1590413
1424226_at	RIKEN cDNA 9030617O03 gene	9030617O03Rik	0.8204	1.09320815	0.12270214
1448291_at	matrix metallopeptidase 9	Mmp9	0.8204	-1.08699164	-0.1223016
1445037_at	G protein-coupled receptor kinase-interactor 2	Git2	0.8204	1.12261713	0.15242796
1421081_a_at	barrier to autointegration factor 1	Banf1	0.8204	1.06477315	0.08660577
1446415_at	heme binding protein 2	Hebp2	0.82041	-1.09821105	-0.1566008
1437625_at	---	---	0.82042	1.07887846	0.10895978
1455719_at	tubulin, beta 5	Tubb5	0.82042	-1.059144	-0.0860012
1444351_at	---	---	0.82042	1.07665157	0.10580985
1446934_at	---	---	0.82042	1.08925476	0.118567
1425305_at	zinc finger protein 295	Zfp295	0.82043	1.07193648	0.09915981
1454963_at	phosphodiesterase 12	Pde12	0.82043	-1.06244167	-0.0893143
1419283_s_at	tensin 1	Tns1	0.82043	1.08746496	0.11432594
1438843_x_at	mitochondrial carrier homolog 2 (C. elegans)	Mtch2	0.82043	-1.0576571	-0.0812648
1444802_at	SEC22 vesicle trafficking protein homologe A (S. cerevisiae)	Sec22a	0.82044	1.07790617	0.10747416
1431103_at	RIKEN cDNA 1700003P14 gene	1700003P14Rik	0.82044	-1.08835167	-0.1367726
1421042_at	rho/rac guanine nucleotide exchange factor (GEF) 2	Arhgef2	0.82044	1.06821683	0.09519771
1425778_at	indoleamine 2,3-dioxygenase 2	Ido2	0.82044	1.11742628	0.14530327
1439064_at	NYN domain and retroviral integrase containing	Nynrin	0.82044	1.09074396	0.12101078
1455330_at	nucleolar protein 9	Nol9	0.82044	1.06970112	0.09313367
1451745_a_at	zinc finger, HIT domain containing 1	Znhit1	0.82044	1.08850384	0.11057335
1448075_at	expressed sequence AA408251	AA408251	0.82045	-1.09022016	-0.1325482
1435421_at	fibronectin type 3 and SPRY domain-containing protein	Fsd1	0.82045	1.06844553	0.09325614
1421968_a_at	non imprinted in Prader-Willi/Angelman syndrome 2 homolog (hu	Nipa2	0.82045	-1.07134239	-0.1078558
1416439_at	dCTP pyrophosphatase 1	Dctpp1	0.82045	1.06521602	0.08728935
1432935_at	RIKEN cDNA 5330433J24 gene	5330433J24Rik	0.82046	-1.09582276	-0.1411146
1428288_at	Kruppel-like factor 9	Klf9	0.82046	-1.05554795	-0.0791686
1421430_at	RAD51-like 1 (S. cerevisiae)	Rad51l1	0.82046	1.09381873	0.12691633
1453756_at	RIKEN cDNA 2900075N08 gene /// neuroligin 3	2900075N08Rik /// Nlgn3	0.82046	1.09833145	0.12970629
1440682_at	---	---	0.82046	-1.08131723	-0.1192223
1434473_at	solute carrier family 16 (monocarboxylic acid transporters), memb	Slc16a5	0.82046	1.11765467	0.14690141
1427077_a_at	adaptor-related protein complex 2, beta 1 subunit	Ap2b1	0.82047	-1.06453863	-0.0930697
1445682_at	NDC80 homolog, kinetochore complex component pseudogene	2700099C18Rik	0.82047	-1.08422812	-0.1238279
1459085_at	DNA segment, Chr 1, ERATO Doi 259, expressed	D1Ertd259e	0.82048	-1.09792301	-0.1482854
1444484_at	RIKEN cDNA A130009E19 gene	A130009E19Rik	0.82048	1.09157269	0.12295451
1417003_at	RIKEN cDNA 0610012G03 gene	0610012G03Rik	0.82048	1.11482142	0.14347105
1453727_at	ESF1, nucleolar pre-rRNA processing protein, homolog (S. cerevisi	Esf1	0.82049	-1.08874091	-0.1530201
1422007_at	aquaporin 3	Aqp3	0.82049	-1.08350438	-0.1197846
1417819_at	torsin family 1, member B	Tor1b	0.82049	1.12925311	0.15084002
1424329_a_at	proline-rich Gla (G-carboxyglutamic acid) polypeptide 2	Prrg2	0.82049	1.09380548	0.12370947

1435383_x_at	necdin	Ndn	0.82049	-1.07316465	-0.1026607
1416280_at	ubiquitin-like modifier activating enzyme 2	Uba2	0.8205	1.05073832	0.06952615
1446222_at	---	---	0.8205	1.08162067	0.11052712
1452765_at	solute carrier family 39 (zinc transporter), member 9	Slc39a9	0.82051	1.0773631	0.10529199
1446452_at	---	---	0.82051	1.08936864	0.12077894
1439843_at	calcium/calmodulin-dependent protein kinase IV	Camk4	0.82052	-1.093946	-0.1513985
1416677_at	apolipoprotein H	Apoh	0.82052	-1.1000376	-0.1519425
1420885_a_at	seizure related gene 6	Sez6	0.82053	1.10734092	0.13395288
1428474_at	protein phosphatase 3, catalytic subunit, beta isoform	Ppp3cb	0.82054	-1.08973631	-0.1307297
1432624_at	RIKEN cDNA 5830487K18 gene	5830487K18Rik	0.82054	-1.09188537	-0.1337003
1438194_at	solute carrier family 1 (glial high affinity glutamate transporter), m	Slc1a2	0.82054	-1.08741175	-0.1213806
1426172_a_at	CD209a antigen	Cd209a	0.82055	1.09462678	0.13036972
1416143_at	ATP synthase, H+ transporting, mitochondrial F0 complex, subunit	Atp5j	0.82056	1.05168197	0.07170695
1457911_at	RIKEN cDNA A930002I21 gene	A930002I21Rik	0.82057	1.0946367	0.12503953
1452913_at	Purkinje cell protein 4-like 1	Pcp4l1	0.82057	1.08764345	0.12081514
1451872_a_at	neuralized homolog 1A (Drosophila)	Neurl1a	0.82057	-1.08537169	-0.1239128
1430113_at	SH2 domain containing 6	Sh2d6	0.82058	1.10039686	0.13272671
1425914_a_at	armadillo repeat containing, X-linked 1	Armcx1	0.82058	-1.07288621	-0.10916
1458128_at	RIKEN cDNA 1110028C15 gene	1110028C15Rik	0.82059	-1.0809003	-0.11513
1417154_at	solute carrier family 25 (mitochondrial carrier, brain), member 14	Slc25a14	0.82061	1.10123254	0.12698598
1447980_s_at	small G protein signaling modulator 1	Sgsm1	0.82061	1.08583013	0.11722939
1431411_a_at	retinoic acid induced 12	Rai12	0.82061	1.06096085	0.08433353
1456176_x_at	solute carrier family 25, member 39	Slc25a39	0.82062	-1.06321543	-0.0939935
1423050_s_at	heterogeneous nuclear ribonucleoprotein U	Hnrnpu	0.82063	-1.0547201	-0.0785281
1451734_a_at	drebrin 1	Dbn1	0.82063	1.08744924	0.11513806
1442731_at	PDS5, regulator of cohesion maintenance, homolog A (S. cerevisia	Pds5a	0.82063	-1.09826504	-0.1512647
1441410_at	DNA segment, Chr 2, ERATO Doi 112, expressed	D2Ertd112e	0.82064	-1.0896571	-0.1253498
1426770_at	peroxisomal biogenesis factor 5	Pex5	0.82064	1.07268602	0.09977468
1442918_at	neuron navigator 3	Nav3	0.82064	1.13243738	0.1597663
1441384_at	glutamate decarboxylase-like 1	Gadl1	0.82065	-1.08550221	-0.1268366
1435431_at	proteasome (prosome, macropain) assembly chaperone 4	Psmg4	0.82065	1.10961778	0.12956836
1458078_at	chromodomain helicase DNA binding protein 9	Chd9	0.82066	1.08884013	0.12093154
1437809_x_at	E26 avian leukemia oncogene 2, 3' domain	Ets2	0.82066	-1.07456024	-0.1079269
1430144_at	AVL9 homolog (S. cerevisiase)	Avl9	0.82066	1.11100689	0.14126454
1448409_at	lymphoid-restricted membrane protein	Lrmp	0.82066	1.09319013	0.12602224
1420130_s_at	DNA segment, Chr 10, Wayne State University 52, expressed	D10Wsu52e	0.82067	-1.11062792	-0.2063099
1451749_at	interleukin-1 receptor-associated kinase 4	Irak4	0.82067	1.0890325	0.12092661
1446214_at	RIKEN cDNA D430018E03 gene	D430018E03Rik	0.82068	-1.08923877	-0.1247288
1455135_at	proline rich 24	Prr24	0.82068	1.09115056	0.12426868
1451288_s_at	RIKEN cDNA 1810043G02 gene	1810043G02Rik	0.82068	1.11180063	0.14085962
1421177_at	THAP domain containing, apoptosis associated protein 2	Thap2	0.82069	1.1004574	0.12981292
1417266_at	chemokine (C-C motif) ligand 6	Ccl6	0.82069	-1.09739276	-0.1425687
1417898_a_at	granzyme A	Gzma	0.82069	-1.09613312	-0.1521176
1454324_at	RIKEN cDNA 4930487N04 gene	4930487N04Rik	0.82069	-1.09322739	-0.1352584
1457516_at	---	---	0.82069	1.08710222	0.11307466
1453938_at	RIKEN cDNA 9330198N18 gene	9330198N18Rik	0.82069	-1.08182944	-0.1157246
1436712_at	phospholipase A2, group IVC (cytosolic, calcium-independent)	Pla2g4c	0.8207	-1.08747718	-0.1214567
1446329_at	---	---	0.8207	1.0947317	0.1261606
1442286_at	---	---	0.82071	1.12251688	0.14992344
1426414_a_at	ring finger protein 7	Rnf7	0.82071	1.05677729	0.0793131
1438596_at	RIKEN cDNA 1500017E21 gene	1500017E21Rik	0.82071	1.0578933	0.08032721
1416501_at	3-phosphoinositide dependent protein kinase 1	Pdpk1	0.82071	-1.09117518	-0.1548828
1425453_x_at	killer cell lectin-like receptor subfamily A, member 12	Klra12	0.82071	1.06931513	0.09447503
AFFX-r2-Bs-thr-3_	---	---	0.82072	1.14039436	0.15908685
1429884_at	SLIT-ROBO Rho GTPase activating protein 2	Srgap2	0.82072	1.06887845	0.09550572
1442767_s_at	ubiquitin-like modifier activating enzyme 1	Uba1	0.82072	1.09893396	0.1299366
1435877_at	serine/threonine kinase 38 like	Stk38l	0.82072	1.07852582	0.10835152
1444975_at	---	---	0.82072	1.09912875	0.13065042
1444552_at	---	---	0.82073	-1.09152741	-0.1287187
1424404_at	RIKEN cDNA 0610040J01 gene	0610040J01Rik	0.82073	1.11696188	0.14386819
1437861_s_at	protein kinase C, epsilon	Prkce	0.82073	1.13484924	0.16044985
1424290_at	oxidative stress induced growth inhibitor family member 2	Osgin2	0.82073	1.07780867	0.10520262
1426420_at	RNA binding motif protein 26	Rbm26	0.82074	-1.08265989	-0.1253602
1457541_at	A kinase (PRKA) anchor protein 14	Akap14	0.82074	-1.0861791	-0.1201385
1421871_at	SH3-binding domain glutamic acid-rich protein like	Sh3bgrl	0.82074	-1.08127618	-0.1157551
1452586_at	anaphase promoting complex subunit 13	Anapc13	0.82074	1.06803179	0.09145888
1439142_at	SWI/SNF related matrix associated, actin dependent regulator of	Smarcal1	0.82074	-1.0890624	-0.1241782
1429013_at	MAP7 domain containing 2	Mtap7d2	0.82074	1.09721935	0.12283641
1417740_at	cell division cycle 37 homolog (S. cerevisiae)-like 1	Cdc37l1	0.82074	1.06070529	0.08474387
1427960_at	UDP glucuronosyltransferase 2 family, polypeptide B34	Ugt2b34	0.82075	1.09186983	0.12061842
1417127_at	homeobox, msh-like 1	Msx1	0.82075	1.08745626	0.11454607
1449180_at	potassium channel modulatory factor 1	Kcmf1	0.82075	-1.0743392	-0.1068925
1458002_at	---	---	0.82075	1.09145542	0.12517509
1432133_at	Ryanodine receptor 3	Ryr3	0.82075	-1.0896279	-0.1298171
1436750_a_at	3-oxoacid CoA transferase 1	Oxct1	0.82075	-1.10827515	-0.2421687
1445477_at	---	---	0.82076	-1.09609803	-0.1454994
1458361_at	DNA cross-link repair 1C, PSO2 homolog (S. cerevisiae)	Dclre1c	0.82076	-1.0886978	-0.1259079
1421166_at	attractin	Atrn	0.82077	-1.09067549	-0.1324068
1439963_x_at	family with sequence similarity 154, member B	Fam154b	0.82077	1.08814896	0.12066677
1418387_at	M-phase phosphoprotein 8	Mphosph8	0.82078	1.0671821	0.09012736
1429332_at	RIKEN cDNA 4632427E13 gene	4632427E13Rik	0.82078	-1.09204682	-0.1426763
1418161_at	junctophilin 3	Jph3	0.82078	-1.08549116	-0.118443
1433786_x_at	small EDRK-rich factor 2-like /// small EDRK-rich factor 2	LOC100503839 /// Serf2	0.82078	1.05599744	0.07637081
1439476_at	desmoglein 2	Dsg2	0.82079	1.08373127	0.10815083
1446462_at	---	---	0.82079	1.09677194	0.12826429
1444741_at	---	---	0.82079	-1.08953354	-0.1262136
1441605_at	---	---	0.82079	1.09238617	0.12401507
1451365_at	RNA binding motif protein 19	Rbm19	0.82079	1.07003544	0.09603019
1440495_at	rhabdomyosarcoma 2 associated transcript (non-coding RNA)	Rmst	0.8208	-1.0816497	-0.1200919
1459781_x_at	transformation related protein 53	Trp53	0.8208	-1.06833045	-0.0955977
1429163_at	dachsous 1 (Drosophila)	Dchs1	0.8208	1.12426911	0.15220251
1455540_at	carbamoyl-phosphate synthetase 1	Cps1	0.8208	-1.09175251	-0.1333209
1426486_at	UBX domain protein 4	Ubxn4	0.8208	-1.06127092	-0.0858577
1434216_a_at	nudix (nucleoside diphosphate linked moiety X)-type motif 19	Nudt19	0.8208	1.07324479	0.09814409

1431836_x_at	MEF2 activating motif and SAP domain containing transcriptional	Mamstr	0.8208	-1.08689544	-0.1232613
1433512_at	Friend leukemia integration 1	Fli1	0.82081	1.07716394	0.10235177
1419087_s_at	splicing factor 3a, subunit 1	Sf3a1	0.82081	1.07640504	0.10440644
1449432_a_at	membrane metallo-endopeptidase-like 1	Mmel1	0.82081	1.08975888	0.12224512
1454307_at	RIKEN cDNA 1810058N15 gene	1810058N15Rik	0.82081	-1.08858572	-0.122517
1451781_at	nuclear factor of activated T-cells, cytoplasmic, calcineurin-depend	Nfatc2ip	0.82081	1.09030129	0.11765042
1441243_at	---	---	0.82081	1.07952546	0.10876091
1449347_a_at	x-linked lymphocyte-regulated protein 3A-like /// X-linked lymph	LOC100505359 /// Xlr4a /	0.82082	1.07447951	0.10316875
1433833_at	fibronectin type III domain containing 3B	Fndc3b	0.82082	1.06785935	0.09438931
1416184_s_at	high mobility group AT-hook 1 /// high mobility group AT-hook I,	Hmga1 /// Hmga1-rs1	0.82082	1.05611901	0.07535386
1443346_at	---	---	0.82082	-1.08566263	-0.1188937
1460112_at	---	---	0.82082	1.08530163	0.116946
1450207_at	leukemia inhibitory factor receptor	Lifr	0.82082	1.09019354	0.12220156
1427002_s_at	arylsulfatase G	Arsg	0.82083	1.0869771	0.11926788
1434356_a_at	proteasome (prosome, macropain) subunit, alpha type 5	Psma5	0.82083	-1.04950598	-0.0702261
1453867_at	F-box and WD-40 domain protein 2	Fbxw2	0.82084	-1.08206731	-0.1179483
1426624_a_at	yippee-like 3 (Drosophila)	Ypel3	0.82084	1.07296189	0.09719123
1437662_at	acyl-CoA synthetase medium-chain family member 5	Acsm5	0.82085	1.10006142	0.12903893
1453045_at	coiled-coil domain containing 41	Ccdc41	0.82085	-1.06855787	-0.0959101
1422970_at	Max dimerization protein 3	Mxd3	0.82085	1.09933939	0.1273593
1420660_at	leucine rich repeat containing 6 (testis)	Lrrc6	0.82085	1.08262112	0.11251497
1451550_at	Eph receptor B3	Ephb3	0.82085	1.07142748	0.09750074
1430765_at	RIKEN cDNA 5730521K06 gene	5730521K06Rik	0.82085	-1.07279586	-0.1037264
1458046_at	---	---	0.82086	-1.09121113	-0.1388288
1418055_at	neurogenic differentiation 4	Neurod4	0.82086	-1.07943673	-0.1149066
1454880_s_at	BCL2 modifying factor	Bmf	0.82087	-1.06867151	-0.0968292
1416059_at	SEC23B (S. cerevisiae)	Sec23b	0.82087	1.05925315	0.08170825
1418017_at	pumilio 2 (Drosophila)	Pum2	0.82088	-1.0718981	-0.1101455
1454000_s_at	RIKEN cDNA 4930578N16 gene	4930578N16Rik	0.82088	1.0865097	0.11776181
1456289_at	hepcidin antimicrobial peptide 2	Hamp2	0.82089	-1.08326358	-0.1188135
1434972_x_at	serine/arginine-rich splicing factor 1	Srsf1	0.8209	-1.04712589	-0.0671897
1438874_at	non-metastatic cells 7, protein expressed in (nucleoside-diphospha	Nme7	0.8209	-1.09874249	-0.1577057
1416489_at	phosphatidylinositol 4-kinase type 2 beta	Pi4k2b	0.82091	1.06952577	0.09560054
1445696_x_at	bolA-like 3 (E. coli)	Bola3	0.82091	-1.0824016	-0.1150676
1454104_a_at	solute carrier family 16 (monocarboxylic acid transporters), memb	Slc16a9	0.82092	1.10985576	0.13810361
1443091_at	---	---	0.82092	-1.08618342	-0.1233569
1429481_at	hypothetical protein LOC100503894 /// non-catalytic region of ty	LOC100503894 /// Nck2	0.82093	1.09804726	0.12492758
1449114_at	serine/threonine kinase 3 (Ste20, yeast homolog)	Stk3	0.82093	-1.09201305	-0.13272
1417841_at	peroxisomal membrane protein 2	Pxmp2	0.82093	1.07122549	0.09768216
1451870_a_at	bromodomain containing 4	Brd4	0.82094	-1.08736626	-0.1253048
1452890_at	tubulin tyrosine ligase-like family, member 5	Ttll5	0.82094	-1.08355354	-0.1194311
1437753_at	RIKEN cDNA 6230409E13 gene	6230409E13Rik	0.82095	1.12508497	0.15364238
1417243_at	family with sequence similarity 192, member A	Fam192a	0.82095	1.05833981	0.08145979
1442848_at	---	---	0.82095	-1.08408483	-0.1184285
1450151_at	zinc finger protein 316	Zfp316	0.82096	1.09963105	0.13072596
1431229_at	imprinted gene in the Prader-Willi syndrome region	Ipw	0.82097	1.09136754	0.12600903
1427567_a_at	tropomyosin 3, gamma	Tpm3	0.82097	-1.06248437	-0.0933508
1449295_at	SAP30 binding protein	Sap30bp	0.82097	1.07589229	0.10134848
1421851_at	microtubule-associated protein 1B	Mtap1b	0.82098	1.09083516	0.12120628
1433889_at	SRY-box containing gene 9	Sox9	0.82098	1.09955643	0.12790832
1419128_at	integrin alpha X	Itgax	0.82098	-1.10391062	-0.1626496
1418937_at	deiodinase, iodothyronine, type II	Dio2	0.82098	-1.08966671	-0.1267359
1431694_a_at	catenin beta interacting protein 1	Ctnnbip1	0.82099	1.0766814	0.10176091
1437456_x_at	YTH domain family 1	Ythdf1	0.821	1.05873155	0.08124931
1448493_at	polyadenylate-binding protein-interacting protein 2	Paip2	0.821	1.05671945	0.07842742
1452812_at	latrophilin 1	Lphn1	0.82101	-1.05986479	-0.0856864
1450892_a_at	ubiquitin specific peptidase 4 (proto-oncogene)	Usp4	0.82102	1.06027139	0.08429796
1421226_at	triggering receptor expressed on myeloid cells 2	Trem2	0.82102	1.08560631	0.11696804
1424209_at	arginyl-tRNA synthetase 2, mitochondrial	Rars2	0.82103	1.06670889	0.09064098
1420229_at	---	---	0.82103	-1.0743479	-0.1050924
1453721_a_at	solute carrier family 31, member 2	Slc31a2	0.82103	1.08303837	0.10990417
1438850_at	claudin 1	Cldn1	0.82103	1.06406149	0.08932197
1435944_s_at	centromere protein B	Cenpb	0.82105	1.0905888	0.12322972
1428290_at	mitochondrial intermediate peptidase	Mipep	0.82106	1.06116748	0.08561549
1453304_s_at	lymphocyte antigen 6 complex, locus E	Ly6e	0.82107	1.08356969	0.1060881
1423359_at	phospholamban	Pln	0.82107	-1.09003113	-0.1311303
1424787_a_at	nuclear respiratory factor 1	Nrf1	0.82107	1.07838747	0.10261875
1435898_x_at	guanosine diphosphate (GDP) dissociation inhibitor 2	Gdi2	0.82107	-1.04775192	-0.0682116
1430137_at	RIKEN cDNA 1300002E11 gene	1300002E11Rik	0.82107	-1.09341595	-0.1342216
1432914_at	leucine rich repeat containing 52	Lrrc52	0.82107	-1.07783373	-0.1186366
1431840_at	DENN/MADD domain containing 1C	Dennd1c	0.82108	1.12139464	0.14977118
1453357_at	tudor domain containing 9	Tdrd9	0.82109	1.13178982	0.15718943
1448442_a_at	proteasome (prosome, macropain) subunit, alpha type 3	Psma3	0.82109	-1.045239	-0.0638877
1416898_a_at	interferon regulatory factor 3	Irf3	0.82109	1.07586541	0.10409917
1439858_at	hypothetical LOC100502927	LOC100502927	0.8211	1.08910341	0.11187739
1416170_at	TNF receptor-associated protein 1	Trap1	0.8211	1.06415331	0.08701697
1416939_at	pyrophosphatase (inorganic) 1	Ppa1	0.8211	1.05243169	0.07266435
1442968_at	DNA segment, Chr 9, ERATO Doi 241, expressed	D9Ertd241e	0.8211	1.08608839	0.11896057
1432305_at	RIKEN cDNA 4930542C16 gene	4930542C16Rik	0.82111	-1.0987902	-0.1531722
1451763_at	cyclic nucleotide gated channel alpha 1	Cnga1	0.82111	1.10749794	0.13745676
1435827_at	RIKEN cDNA 4933404O12 gene	4933404O12Rik	0.82112	1.0673351	0.09369414
1418260_at	hormonally upregulated Neu-associated kinase	Hunk	0.82112	1.06561287	0.09054217
1442975_at	---	---	0.82113	1.09973822	0.13112505
1453039_at	zinc finger protein 335	Zfp335	0.82113	1.07317418	0.10119713
1436754_at	transmembrane protein 179	Tmem179	0.82113	-1.08501971	-0.1203507
1429122_a_at	RIKEN cDNA 1700040I03 gene	1700040I03Rik	0.82113	1.08037214	0.10797398
1426909_at	uridine-cytidine kinase 2	Uck2	0.82114	1.09305383	0.1149671
1447307_at	---	---	0.82114	-1.08515048	-0.1202942
1424927_at	GLI pathogenesis-related 1 (glioma)	Glipr1	0.82114	-1.08972381	-0.125867
1456074_at	4short chain dehydrogenase/reductase family 9C, member 7	Sdr9c7	0.82114	1.10083372	0.13282628
1426181_a_at	interleukin 24	Il24	0.82115	-1.08942576	-0.1267369
1427039_at	epsin 1	Epn1	0.82116	1.07997037	0.10671233
1425732_a_at	Max interacting protein 1	Mxi1	0.82116	1.08591068	0.11154553
1421380_at	insulin receptor	Insr	0.82116	-1.09170026	-0.1284467

1444450_at	RAB42, member RAS oncogene family, pseudogene	Rab42-ps	0.82116	1.09848086	0.13116619
1437446_at	RAB5B, member RAS oncogene family	Rab5b	0.82116	1.11558483	0.14312806
1451726_at	myotubularin related protein 6	Mtmr6	0.82117	1.0838794	0.11090685
1418803_a_at	Fas ligand (TNF superfamily, member 6)	Fasl	0.82117	-1.08546569	-0.1200356
1456667_at	huntingtin	Htt	0.82119	1.08257832	0.11173204
1434948_at	RAB11 family interacting protein 2 (class I)	Rab11fip2	0.82119	-1.0825462	-0.1196411
1431452_at	N-deacetylase/N-sulfotransferase (heparin glucosaminyl) 4	Ndst4	0.82121	-1.09255329	-0.1309717
1450245_at	solute carrier family 10, member 2	Slc10a2	0.82121	1.08630713	0.1118923
1436491_at	X-linked lymphocyte-regulated 5 pseudogene	Gm11346	0.82122	1.09203141	0.12607241
1455880_s_at	beclin 1, autophagy related	Becn1	0.82122	-1.06304515	-0.0906417
1439025_at	zinc finger protein 446	Zfp446	0.82124	1.07868174	0.10719631
1423311_s_at	trophoblast glycoprotein	Tpbg	0.82124	1.0837802	0.11533606
1447621_s_at	transmembrane protein 173	Tmem173	0.82127	-1.09497576	-0.1406137
1426572_at	NAD-dependent malic enzyme, mitochondrial-like /// malic enzyı	LOC100505264 /// Me2	0.82127	1.06560676	0.08988904
1450797_a_at	chromobox homolog 1 (Drosophila HP1 beta)	Cbx1	0.82128	-1.09349391	-0.1406278
1434812_s_at	docking protein 7	Dok7	0.8213	-1.0749649	-0.105684
1434806_at	metaxin 3	Mtx3	0.8213	1.10049607	0.13261565
1434176_x_at	polymerase (DNA-directed), delta interacting protein 3	Poldip3	0.82131	-1.05852612	-0.0828838
1424397_at	DEAH (Asp-Glu-Ala-His) box polypeptide 36	Dhx36	0.82132	-1.06134638	-0.0862156
1446080_at	---	---	0.82132	-1.08539148	-0.1259001
1417554_at	hydroxy-delta-5-steroid dehydrogenase, 3 beta- and steroid delta	Hsd3b4	0.82132	-1.09289722	-0.1378545
1424129_at	major facilitator superfamily domain containing 1	Mfsd1	0.82134	1.07129224	0.09721591
1449638_at	RIKEN cDNA C920021L13 gene	C920021L13Rik	0.82135	-1.09224897	-0.1418128
1456507_at	zinc finger protein 454	Zfp454	0.82136	1.10710134	0.13422511
1452916_at	WW domain binding protein 7	Wbp7	0.82136	1.06363449	0.08886115
1440357_at	microRNA let7b	Mirlet7b	0.82137	1.10419247	0.13593654
1438708_x_at	tyrosine 3-monooxygenase/tryptophan 5-monooxygenase activa	Ywhab	0.82139	-1.084405	-0.1189393
1417970_at	ATP synthase, H+ transporting, mitochondrial F0 complex, subunit	Atp5s	0.82139	1.08330759	0.1101606
1448801_a_at	translocase of inner mitochondrial membrane 44	Timm44	0.82139	1.07471645	0.09906697
1458251_at	expressed sequence AU022166	AU022166	0.8214	1.07829795	0.10835291
1438184_a_at	ankyrin repeat domain 5	Ankrd5	0.82141	-1.0979084	-0.1626273
1429503_at	family with sequence similarity 69, member A	Fam69a	0.82143	-1.06991233	-0.0991898
1460342_s_at	myosin phosphatase Rho interacting protein	Mprip	0.82144	1.06653222	0.09188512
1427185_at	myocyte enhancer factor 2A	Mef2a	0.82144	1.07399174	0.10203002
1424849_at	WD repeat domain 62	Wdr62	0.82145	1.0785722	0.10519954
1437054_x_at	protamine 1	Prm1	0.82147	1.07206446	0.09721925
1448300_at	microsomal glutathione S-transferase 3	Mgst3	0.82147	1.07379738	0.09955017
1443450_at	hypothetical LOC100503622	LOC100503622	0.82148	1.09251903	0.12585887
1427941_at	Dicer1, Dcr-1 homolog (Drosophila)	Dicer1	0.82149	1.06433814	0.089487
1426210_x_at	poly (ADP-ribose) polymerase family, member 3	Parp3	0.82149	1.07633085	0.10586375
1445350_at	---	---	0.82149	-1.09137465	-0.1380723
1425011_x_at	syntaxin 18	Stx18	0.8215	1.0734874	0.09888992
1441232_at	---	---	0.82151	1.09293395	0.12362126
1454911_at	F-box and WD-40 domain protein 24	Fbxw24	0.82152	-1.08707385	-0.1274741
1441945_s_at	abhydrolase domain containing 14A	Abhd14a	0.82152	1.06868311	0.09398223
1417528_at	sperm associated antigen 6	Spag6	0.82153	-1.08231636	-0.1163561
1444873_at	---	---	0.82156	-1.08411735	-0.1173646
1431857_at	PHD finger protein 19	Phf19	0.82157	-1.07350329	-0.1030369
1446847_at	coiled-coil domain containing 88C	Ccdc88c	0.82157	-1.08961349	-0.1266495
1425679_a_at	mitogen-activated protein kinase 8 interacting protein 1	Mapk8ip1	0.82157	-1.07545639	-0.1098622
1426995_a_at	growth factor, erv1 (S. cerevisiae)-like (augmenter of liver regene	Gfer	0.82157	1.07704908	0.1001416
1418427_at	kinesin family member 5B	Kif5b	0.82158	-1.09590535	-0.1881526
1449132_at	opsin 1 (cone pigments), short-wave-sensitive (color blindness, tri	Opn1sw	0.82158	-1.08503809	-0.1181036
1418538_at	KDEL (Lys-Asp-Glu-Leu) endoplasmic reticulum protein retention r	Kdelr3	0.82159	-1.0649936	-0.0927487
1442120_at	RIKEN cDNA G730007D18 gene	G730007D18Rik	0.8216	1.09373961	0.12235046
1428160_at	NADH dehydrogenase (ubiquinone) 1, alpha/beta subcomplex, 1	Ndufab1	0.82161	-1.09142294	-0.1471695
1422458_at	T-cell lymphoma breakpoint 1	Tcl1	0.82161	1.05686515	0.07949955
1442988_at	---	---	0.82162	1.08788665	0.12003672
1460708_s_at	cell division cycle 42 homolog (S. cerevisiae)	Cdc42	0.82162	-1.05646311	-0.0809028
1442302_at	N-methylpurine-DNA glycosylase	Mpg	0.82163	1.11538827	0.14332579
1436219_at	RIKEN cDNA 4933403F05 gene	4933403F05Rik	0.82163	1.08626109	0.11626874
1444444_at	---	---	0.82165	-1.08285394	-0.1156415
1452149_at	ubiquitin protein ligase E3B	Ube3b	0.82165	1.07151636	0.09727484
1453695_at	vomeronasal 1 receptor 90	Vmn1r90	0.82165	-1.08015198	-0.1138435
1456081_a_at	acetoacetyl-CoA synthetase	Aacs	0.82165	1.07317982	0.10007858
1427263_at	inactive X specific transcripts	Xist	0.82166	1.13971123	0.16076753
1458305_at	transmembrane and tetratricopeptide repeat containing 3	Tmtc3	0.82167	1.12251505	0.14911334
1418033_s_at	zinc finger with KRAB and SCAN domains 6	Zkscan6	0.82167	1.07421126	0.10293889
1417718_at	eukaryotic translation initiation factor 3, subunit G	Eif3g	0.82167	1.04911791	0.06885401
1419372_at	golgi SNAP receptor complex member 2	Gosr2	0.82168	1.07625482	0.1024601
1426925_at	ring finger and CCCH-type zinc finger domains 2	Rc3h2	0.82168	1.11564265	0.14569478
1437366_at	membrane-associated ring finger (C3HC4) 9	Mar-09	0.8217	-1.0842498	-0.1224813
1445045_at	Mastermind like 1 (Drosophila)	Maml1	0.82171	-1.08535451	-0.1269042
1459104_at	budding uninhibited by benzimidazoles 3 homolog (S. cerevisiae)	Bub3	0.82171	-1.09603661	-0.1510748
1421378_s_at	ATP-binding cassette, sub-family C (CFTR/MRP), member 1	Abcc1	0.82174	1.09986563	0.12581361
1436173_at	deleted in liver cancer 1	Dlc1	0.82174	1.06478448	0.08821864
1441939_x_at	RIKEN cDNA 2410003I16 gene	2410003I16Rik	0.82176	-1.09251464	-0.1443734
1437253_at	family with sequence similarity 116, member A	Fam116a	0.82176	-1.06931612	-0.1002277
1418385_at	CUE domain containing 2	Cuedc2	0.82176	1.09942338	0.12803503
1451350_a_at	leptin receptor overlapping transcript	Leprot	0.82177	-1.06929603	-0.0970744
1443176_at	Scm-like with four mbt domains 2	Sfmbt2	0.82177	1.09693384	0.1281495
1432097_a_at	DNA cross-link repair 1A, PSO2 homolog (S. cerevisiae)	Dclre1a	0.82177	1.10457437	0.13195821
1459785_at	zinc finger protein 383	Zfp383	0.82178	1.11065961	0.13402748
1455969_at	nudix (nucleoside diphosphate linked moiety X)-type motif 15	Nudt15	0.82179	-1.07705931	-0.115002
1445676_at	---	---	0.82179	1.08939936	0.12033977
1417182_at	DnaJ (Hsp40) homolog, subfamily A, member 2	Dnaja2	0.8218	1.06455667	0.08902269
1439231_at	---	---	0.8218	1.08547677	0.11516094
1442033_at	C1q and tumor necrosis factor related protein 9	C1qtnf9	0.82181	-1.09728717	-0.1607536
1425275_at	aspartate-beta-hydroxylase	Asph	0.82181	1.09117683	0.12349407
1449545_at	fibroblast growth factor 18	Fgf18	0.82181	1.09291338	0.12422342
1418329_at	pyroglutamyl-peptidase I	Pgpep1	0.82181	1.08336854	0.10766903
1423815_at	DEAD (Asp-Glu-Ala-Asp) box polypeptide 56	Ddx56	0.82182	1.06307271	0.08777473
1459095_at	---	---	0.82183	-1.07549589	-0.1131834
1442343_at	---	---	0.82183	1.16844815	0.18338404

1421466_at	ankyrin repeat and SOCS box-containing 10	Asb10	0.82183	1.08653744	0.11891655
1439469_at	---	---	0.82183	1.11870065	0.14917517
1457321_at	RIKEN cDNA D130037M23 gene	D130037M23Rik	0.82183	-1.07613318	-0.1063981
1445788_at	---	---	0.82183	-1.09152674	-0.1354305
1437685_x_at	fibromodulin	Fmod	0.82185	-1.08788778	-0.122687
1437710_x_at	family with sequence similarity 71, member E1	Fam71e1	0.82185	1.09428567	0.12764348
1459252_at	predicted gene 4981	Gm4981	0.82187	-1.09541921	-0.1432244
1433486_at	chloride channel 3	Clcn3	0.82187	-1.06250945	-0.0876727
1451317_at	YTH domain family 2	Ythdf2	0.82188	1.07127248	0.09391901
1452612_at	listerin E3 ubiquitin protein ligase 1	Ltn1	0.82188	1.06248385	0.08666488
1453197_at	mediator of RNA polymerase II transcription, subunit 25 homolog	Med25	0.82189	1.06856401	0.09454091
1448463_at	RIKEN cDNA 4933434E20 gene	4933434E20Rik	0.82189	1.06033204	0.08371571
1436939_at	unc-45 homolog B (C. elegans)	Unc45b	0.8219	-1.09906778	-0.1613403
1452859_at	RIKEN cDNA 1200016B10 gene	1200016B10Rik	0.82191	1.06995315	0.09661048
1439742_at	---	---	0.82191	1.09998259	0.13007926
1460354_a_at	mitochondrial ribosomal protein L13	Mrpl13	0.82191	-1.07687411	-0.1284872
1429240_at	StAR-related lipid transfer (START) domain containing 4	Stard4	0.82193	1.07031082	0.09721121
1426820_at	RIKEN cDNA 2610507B11 gene	2610507B11Rik	0.82193	1.06366343	0.0867328
1430488_at	RIKEN cDNA 1700066B19 gene	1700066B19Rik	0.82195	-1.08177523	-0.116416
1415707_at	anaphase promoting complex subunit 2	Anapc2	0.82195	1.06397124	0.08830254
1454229_a_at	exoribonuclease 2	Eri2	0.82195	-1.09088537	-0.1348339
1449425_at	wingless-related MMTV integration site 2	Wnt2	0.82195	1.10160564	0.13312128
1445841_at	leucine rich repeat containing 39	Lrrc39	0.82195	-1.08961668	-0.1259707
1444936_at	DNA segment, Chr 5, ERATO Doi 566, expressed	D5Ertd566e	0.82196	1.08658183	0.11635057
1446645_at	RIKEN cDNA A730037C10 gene	A730037C10Rik	0.82196	1.08778331	0.12044028
1437670_x_at	CD151 antigen	Cd151	0.82197	1.06681279	0.09043085
1431566_at	RIKEN cDNA 9030622O22 gene	9030622O22Rik	0.82198	-1.08326881	-0.1159464
1424036_at	PRP6 pre-mRNA splicing factor 6 homolog (yeast)	Prpf6	0.82198	1.06491811	0.087793
1438662_at	adherens junction associated protein 1	Ajap1	0.82199	-1.09039851	-0.1302226
1424026_s_at	cDNA sequence BC013529	BC013529	0.82199	-1.07134292	-0.1010837
1459400_at	RIKEN cDNA A630038E17 gene	A630038E17Rik	0.82199	-1.08196606	-0.114475
1439567_at	T-box 3	Tbx3	0.822	-1.09725501	-0.1501526
1441098_at	poly(A)-specific ribonuclease (PARN)-like domain containing 1	Pnldc1	0.822	1.07235749	0.0981003
1437491_at	bicaudal D homolog 2 (Drosophila)	Bicd2	0.822	1.09073102	0.12240929
1415763_a_at	RIKEN cDNA 2510006D16 gene /// hypothetical LOC100505293	2510006D16Rik /// LOC1(	0.822	1.05729483	0.08012415
1443206_at	---	---	0.82201	-1.08445135	-0.1192063
1440881_at	bromodomain and WD repeat domain containing 3	Brwd3	0.82201	-1.08219639	-0.1219631
1458271_at	---	---	0.82201	1.08948128	0.11874294
1422690_at	serine palmitoyltransferase, long chain base subunit 1	Sptlc1	0.82202	1.06303592	0.08573079
1452970_at	zinc finger, MYM-type 2	Zmym2	0.82202	1.07448468	0.09802203
1445566_at	---	---	0.82202	1.09984408	0.12843864
1426712_at	solute carrier family 6 (neurotransmitter transporter), member 15	Slc6a15	0.82202	-1.08640531	-0.1368353
1460529_at	RIKEN cDNA 4930455M05 gene	4930455M05Rik	0.82203	-1.07817922	-0.1090161
1436947_a_at	thioredoxin-like 1	Txnl1	0.82204	-1.05922538	-0.0869952
1417980_a_at	insulin induced gene 2	Insig2	0.82204	-1.07019975	-0.1106911
1453764_at	RIKEN cDNA 3110021N24 gene	3110021N24Rik	0.82204	-1.09040384	-0.1250682
1454902_at	protein kinase C, zeta	Prkcz	0.82204	1.08864623	0.12075975
1441695_at	---	---	0.82205	1.08313715	0.11497391
1437266_at	thioredoxin domain containing 2 (spermatozoa)	Txndc2	0.82205	-1.09114263	-0.1279893
1455948_x_at	matrilin 3	Matn3	0.82205	-1.10365013	-0.1744715
1456205_x_at	tubulin cofactor A	Tbca	0.82205	-1.05556177	-0.0792862
1430873_at	---	---	0.82205	1.09353853	0.12264109
1446160_x_at	transmembrane protein 69	Tmem69	0.82206	-1.07992036	-0.1226046
1432909_at	RIKEN cDNA 4933424H11 gene	4933424H11Rik	0.82207	1.07354642	0.09681043
1449469_at	polycystic kidney disease 2-like 2	Pkd2l2	0.82207	-1.08434531	-0.1210546
1445531_at	CUB and Sushi multiple domains 1	Csmd1	0.82208	-1.07872209	-0.1102446
1427484_at	echinoderm microtubule associated protein like 5	Eml5	0.82208	-1.07641685	-0.1071993
1450177_at	nerve growth factor receptor (TNFR superfamily, member 16)	Ngfr	0.82208	1.12145251	0.14374933
1446988_at	---	---	0.82208	-1.08820489	-0.1262737
1456919_at	RIKEN cDNA A930013B10 gene	A930013B10Rik	0.82208	1.12129444	0.14354635
1423010_at	sacsin	Sacs	0.82208	-1.07994393	-0.1153657
1425532_a_at	bridging integrator 1	Bin1	0.82208	1.11573034	0.1434865
1427534_at	RIKEN cDNA 4930535I16 gene	4930535I16Rik	0.82208	-1.09762365	-0.1477813
1447316_at	---	---	0.82209	-1.08885881	-0.1260248
1438042_at	short stature homeobox 2	Shox2	0.82209	1.08449325	0.1162027
1415919_at	neural proliferation, differentiation and control gene 1	Npdc1	0.8221	1.06929621	0.09561877
1451605_at	dyslexia susceptibility 1 candidate 1 homolog (human)	Dyx1c1	0.82211	1.09802497	0.1268036
1433946_at	zinc finger protein interacting with K protein 1	Zik1	0.82211	-1.07834446	-0.110299
1448854_s_at	murinoglobulin, pseudogene 1 /// murinoglobulin 1 /// murinoglo	Mug-ps1 /// Mug1 /// M(	0.82211	1.1019154	0.12749559
1421996_at	transcription factor AP-2, alpha	Tcfap2a	0.82211	-1.07994174	-0.1130721
1445307_at	---	---	0.82212	-1.08431007	-0.1195822
1443955_at	RIKEN cDNA 4933434E20 gene	4933434E20Rik	0.82212	-1.07357282	-0.104913
1418593_at	TAF6 RNA polymerase II, TATA box binding protein (TBP)-associat	Taf6	0.82213	1.0574907	0.08022074
1428331_at	RIKEN cDNA 2210016F16 gene	2210016F16Rik	0.82213	1.06812895	0.09376558
1430861_at	RIKEN cDNA 2010111I01 gene	2010111I01Rik	0.82213	1.09516253	0.12504936
1415989_at	vascular cell adhesion molecule 1	Vcam1	0.82213	-1.0916607	-0.1290413
1434579_x_at	NADH dehydrogenase (ubiquinone) Fe-S protein 8	Ndufs8	0.82214	1.06459452	0.08720516
1443983_at	---	---	0.82214	1.08492017	0.11570608
1447660_at	transducin (beta)-like 3	Tbl3	0.82214	1.08787252	0.11561981
1437894_at	prospero-related homeobox 1	Prox1	0.82214	-1.07718546	-0.117202
1457102_at	RIKEN cDNA A030001D16 gene	A030001D16Rik	0.82214	-1.09459087	-0.1387761
1422813_at	calcium channel, voltage-dependent, gamma subunit 1	Cacng1	0.82216	-1.09095155	-0.1327933
1421056_at	deoxyribonuclease 1-like 3	Dnase1l3	0.82216	1.10784115	0.13493768
1438558_x_at	forkhead box Q1	Foxq1	0.82216	-1.09154686	-0.1413512
1426212_s_at	transmembrane protein 161A	Tmem161a	0.82216	-1.06658749	-0.0943589
1442359_at	six transmembrane epithelial antigen of prostate 2	Steap2	0.82216	-1.06594884	-0.0928631
1451695_a_at	glutathione peroxidase 4	Gpx4	0.82217	-1.04381663	-0.0642006
1439978_at	Snf2-related CREBBP activator protein	Srcap	0.82217	1.14140822	0.16252016
1451020_at	glycogen synthase kinase 3 beta	Gsk3b	0.82217	1.0805737	0.10746625
1444126_at	---	---	0.82217	1.15406627	0.17146635
1460352_s_at	phosphatidylinositol 3 kinase, regulatory subunit, polypeptide 4, p	Pik3r4	0.82217	-1.06932344	-0.1006423
1444361_at	---	---	0.82218	1.11453396	0.13787702
1420879_a_at	tyrosine 3-monooxygenase/tryptophan 5-monooxygenase activa	Ywhab	0.82218	-1.08253897	-0.1403849
1429285_at	serine (or cysteine) peptidase inhibitor, clade A (alpha-1 antiprote	Serpina9	0.82218	-1.0978536	-0.1613194

1449747_at	Methylphosphate capping enzyme	Mepce	0.82218	-1.07860108	-0.1129037
1427116_at	SET domain containing 1A	Setd1a	0.82218	1.0664305	0.0923729
1460453_at	T-cell activation Rho GTPase-activating protein /// T-cell activatio	Tagap /// Tagap1	0.82219	1.07228393	0.1005699
1418943_at	RIKEN cDNA B230120H23 gene	B230120H23Rik	0.8222	-1.10754496	-0.1929743
1426584_a_at	sorbitol dehydrogenase	Sord	0.8222	-1.0763003	-0.1111738
1440579_at	---	---	0.8222	1.11970566	0.14657257
1438891_at	---	---	0.82221	1.11169336	0.13920186
1448827_s_at	myosin, heavy polypeptide 6, cardiac muscle, alpha /// myosin, h	Myh6 /// Myh7	0.82221	1.10944345	0.13690911
1418281_at	RAD51 homolog (S. cerevisiae)	Rad51	0.82222	1.05896565	0.0820821
1446987_at	Defects in morphology 1 homolog (S. cerevisiae)	Dem1	0.82222	1.09145966	0.11973479
1423009_at	secretory blood group 1	Sec1	0.82222	-1.08679394	-0.1211326
1453537_a_at	WD repeat domain 17	Wdr17	0.82222	1.13356762	0.15987034
1435632_at	nuclear fragile X mental retardation protein interacting protein 2	Nufip2	0.82223	1.08179019	0.10914745
1441390_at	speedy homolog A (Xenopus laevis)	Spdya	0.82224	1.08702872	0.11588861
1446753_at	---	---	0.82225	1.06769908	0.08977371
1435287_at	adducin 2 (beta)	Add2	0.82225	-1.08160405	-0.1135348
1431879_at	RIKEN cDNA 9030417H13 gene	9030417H13Rik	0.82227	1.08555984	0.11673135
1457100_at	expressed sequence AW552889	AW552889	0.82227	-1.07418426	-0.1042231
1456857_at	RIKEN cDNA 1500011B03 gene	1500011B03Rik	0.82228	1.06752682	0.09121614
1442015_at	RIKEN cDNA 2500002B13 gene	2500002B13Rik	0.82228	1.06242257	0.08520361
1446691_at	Platelet-derived growth factor, D polypeptide	Pdgfd	0.82228	1.09115165	0.12095209
1443306_at	---	---	0.82228	-1.07095105	-0.1035478
1416265_at	calpain 10	Capn10	0.82229	1.07885495	0.10542849
1456911_at	CLIP associating protein 2	Clasp2	0.8223	1.10118499	0.12879474
1437705_at	zinc fingr protein 551	Zfp551	0.82231	1.11370133	0.14216148
1458168_at	predicted gene 10726	Gm10726	0.82232	-1.07318373	-0.1033018
1458426_at	---	---	0.82233	1.09670781	0.12763255
1438334_at	Dedicator of cyto-kinesis 2	Dock2	0.82234	-1.09147012	-0.1358135
1437781_at	insulinoma-associated 2	Insm2	0.82235	1.10488332	0.13428508
1434821_at	bromodomain containing 1	Brd1	0.82235	-1.0623971	-0.0886937
1454205_at	RIKEN cDNA 4930448A20 gene	4930448A20Rik	0.82236	1.15253226	0.17524204
1458121_at	---	---	0.82236	-1.09939136	-0.158801
1441740_at	---	---	0.82238	1.10365545	0.13064263
1427308_at	disabled homolog 1 (Drosophila)	Dab1	0.82238	1.10779526	0.13556937
1443809_at	---	---	0.82239	-1.07346171	-0.1057998
1425405_a_at	adenosine deaminase, RNA-specific	Adar	0.82239	1.06663155	0.0927962
1450192_at	luteinizing hormone/choriogonadotropin receptor	Lhcgr	0.82239	1.08065666	0.11135203
1456593_at	---	---	0.82242	1.10132809	0.12371785
1456394_at	RAD50 interactor 1	Rint1	0.82243	1.08257884	0.11295894
1422650_a_at	RIO kinase 3 (yeast)	Riok3	0.82243	1.05616096	0.07880427
1427978_at	RIKEN cDNA 4732418C07 gene	4732418C07Rik	0.82244	-1.07803929	-0.1147181
1447038_x_at	pleckstrin homology domain interacting protein	Phip	0.82245	-1.08299513	-0.115461
1425049_at	2'-5' oligoadenylate synthetase 1H	Oas1h	0.82246	1.10907292	0.13990822
1423134_at	Rab interacting lysosomal protein-like 2	Rilpl2	0.82248	1.08769069	0.11194139
1442353_at	inosine triphosphatase (nucleoside triphosphate pyrophosphatase	Itpa	0.82248	-1.0907499	-0.1332233
1452112_a_at	RNA binding motif protein 4B	Rbm4b	0.82248	1.12006458	0.14613366
1418592_at	DnaJ (Hsp40) homolog, subfamily A, member 4	Dnaja4	0.8225	-1.08925271	-0.1257204
1417553_at	placental specific protein 1	Plac1	0.82251	-1.0888537	-0.1271015
1460285_at	integrin alpha 9	Itga9	0.82252	-1.07553912	-0.1074867
1419588_at	sperm associated antigen 1	Spag1	0.82253	-1.08172057	-0.113458
1450777_at	5'-3' exoribonuclease 2	Xrn2	0.82254	1.05773578	0.08055494
1458988_at	---	---	0.82254	-1.08408031	-0.1187884
1458033_at	Ubiquitin protein ligase E3 component n-recognin 1	Ubr1	0.82255	-1.09878144	-0.1519246
1437700_at	Schwannomin interacting protein 1	Schip1	0.82255	-1.08301584	-0.1209753
1418958_at	acyl-malonyl condensing enzyme 1	Amac1	0.82259	1.11440789	0.1405503
1447165_at	---	---	0.82259	1.1061009	0.13004858
1443688_at	zinc finger protein 599	Zfp599	0.8226	-1.08089447	-0.1139409
1418753_at	glutamine fructose-6-phosphate transaminase 2	Gfpt2	0.82262	1.06587653	0.08959563
1446396_at	Angiopoietin-like 1	Angptl1	0.82264	-1.07038371	-0.0998861
1419471_a_at	nuclear distribution gene C homolog (Aspergillus)	Nudc	0.82265	-1.06804122	-0.1039718
1455291_s_at	zinc and ring finger 2	Znrf2	0.82266	-1.08046046	-0.1165276
1423471_at	polypyrimidine tract binding protein 2	Ptbp2	0.82268	1.08846978	0.12073706
1434450_s_at	adrenergic receptor kinase, beta 2	Adrbk2	0.82269	1.10920888	0.13707514
1457294_at	Transmembrane and tetratricopeptide repeat containing 3	Tmtc3	0.8227	1.12735942	0.14810975
1443451_at	---	---	0.82272	1.12859883	0.15382525
1416726_s_at	ubiquitin-conjugating enzyme E2S	Ube2s	0.82287	1.05593707	0.07505088
1416044_at	flightless I homolog (Drosophila)	Flii	0.82289	1.06478362	0.08906027
1436700_a_at	---	---	0.8229	1.08163731	0.11276801
1440363_at	---	---	0.82304	1.10184493	0.12883678
1430348_at	RIKEN cDNA 2900019E01 gene	2900019E01Rik	0.82318	-1.09070407	-0.1322871
1456934_at	calbindin 1	Calb1	0.82321	-1.07359333	-0.1029071
1434540_a_at	clathrin, light polypeptide (Lca)	Clta	0.82323	1.05339838	0.0741874
1421811_at	thrombospondin 1	Thbs1	0.82323	1.10131492	0.13042083
1422168_a_at	brain derived neurotrophic factor	Bdnf	0.82325	1.10578772	0.1377381
1425249_a_at	TYRO3 protein tyrosine kinase 3	Tyro3	0.82329	1.07208279	0.09950711
1418475_at	sodium channel, nonvoltage-gated 1 beta	Scnn1b	0.82329	1.08366741	0.11487623
1442689_at	---	---	0.82341	1.12871133	0.1566294
1452869_at	PRP38 pre-mRNA processing factor 38 (yeast) domain containing	Prpf38b	0.82347	-1.05853092	-0.0845229
1432539_a_at	nucleoporin 54	Nup54	0.82347	-1.08447549	-0.1291887
1433151_at	RIKEN cDNA 4933415B22 gene	4933415b22rik	0.82348	-1.0873882	-0.1284697
1455569_at	---	---	0.82348	1.08491962	0.11641176
1437320_s_at	xeroderma pigmentosum, complementation group A	Xpa	0.8235	1.0835388	0.11426425
1459550_at	synapsin III	Syn3	0.8235	-1.0895431	-0.1430091
1446704_at	---	---	0.8235	-1.075783	-0.1071154
1419655_at	transducin-like enhancer of split 3, homolog of Drosophila E(spl)	Tle3	0.8235	-1.06813861	-0.1037445
1425763_x_at	immunoglobulin heavy chain 2 (serum IgA) /// immunoglobulin h	Igh-2 /// Igh-VJ558 /// LO	0.82351	-1.08731395	-0.1330992
1432590_at	RIKEN cDNA 4930573O21 gene	4930573O21Rik	0.82351	-1.09423499	-0.1454079
1430466_at	RIKEN cDNA 2310005G13 gene	2310005G13Rik	0.82351	-1.08237034	-0.1241107
1423798_a_at	eukaryotic translation initiation factor 1	Eif1	0.82352	-1.05041541	-0.0750197
1438677_at	plakophilin 4	Pkp4	0.82355	-1.08294191	-0.1336388
1443787_x_at	caspase 14	Casp14	0.82355	-1.07253552	-0.1021085
1423808_at	WD repeat containing, antisense to TP53	Wrap53	0.82355	1.07031509	0.09626037
1430965_at	RIKEN cDNA 9430064K01 gene	9430064K01Rik	0.82355	1.12703866	0.15125678
1430263_at	RIKEN cDNA 1700109F18 gene	1700109F18Rik	0.82356	1.08537969	0.11706671

1423638_at	UDP-N-acetyl-alpha-D-galactosamine:polypeptide N-acetylgalact	Galnt4	0.82356	-1.08886415	-0.1309558
1457836_at	major facilitator superfamily domain containing 11	Mfsd11	0.82358	1.08814097	0.12088273
1421151_a_at	Eph receptor A2	Epha2	0.82359	1.06779877	0.0929502
1423959_at	ropporin 1-like	Ropn1l	0.82359	1.11251101	0.14293333
1459961_a_at	Signal transducer and activator of transcription 3	Stat3	0.82362	1.18580952	0.19470948
1429522_at	ankyrin repeat domain 42	Ankrd42	0.82363	1.07810587	0.10795996
1428616_at	zinc finger protein 131	Zfp131	0.82365	-1.05739893	-0.0809337
1420046_s_at	MAF1 homolog (S. cerevisiae)	Maf1	0.82371	1.06650182	0.08934037
1416621_at	lethal giant larvae homolog 1 (Drosophila)	Llgl1	0.82376	1.06073393	0.08426934
1438435_at	alkaline ceramidase 3	Acer3	0.82379	1.08845782	0.11657072
1442193_at	RIKEN cDNA A430028G04 gene	A430028G04Rik	0.8238	-1.08233403	-0.1162661
1430858_at	RIKEN cDNA 4632433K11 gene	4632433K11Rik	0.82381	-1.09352816	-0.1394641
1434304_s_at	nuclear undecaprenyl pyrophosphate synthase 1 homolog (S. cer	Nus1	0.82382	-1.07196501	-0.1096154
1446963_at	---	---	0.82385	1.0874267	0.1199281
1458581_at	hypothetical LOC553093	LOC553093	0.82385	-1.08983244	-0.124614
1453707_at	RIKEN cDNA 4930480G23 gene	4930480G23Rik	0.82386	1.06970603	0.09706733
1442187_at	Bradykinin receptor, beta 2	Bdkrb2	0.82388	1.12366515	0.15131186
1445922_at	---	---	0.82388	1.10853154	0.13576454
1435676_at	centrosomal protein 164	Cep164	0.8239	-1.08095444	-0.1199214
1457745_at	G protein-coupled receptor 4	Gpr4	0.8239	-1.07302156	-0.1069758
1455109_at	transducin (beta)-like 1X-linked receptor 1	Tbl1xr1	0.82391	-1.08393231	-0.1243502
1458148_at	NLR family, CARD domain containing 3	Nlrc3	0.82391	-1.0830388	-0.1182399
1450978_at	dishevelled, dsh homolog 1 (Drosophila)	Dvl1	0.82391	1.06187389	0.08546707
1458722_at	---	---	0.82394	1.08270049	0.11433701
1433337_at	RIKEN cDNA 6720460K10 gene	6720460K10Rik	0.82394	-1.09344628	-0.1510301
1427787_at	trans-acting transcription factor 6	Sp6	0.82394	1.09182212	0.12450355
1435382_at	necdin	Ndn	0.82395	-1.07477611	-0.1098053
1420010_at	serine incorporator 4	Serinc4	0.82396	-1.08570925	-0.1193383
1426283_at	neurotrimin	Ntm	0.82397	1.14061203	0.16315067
1417981_at	insulin induced gene 2	Insig2	0.82398	1.06710493	0.09310853
1434676_at	myotubularin related protein 9	Mtmr9	0.82399	1.07353086	0.09949741
1427480_at	liver-expressed antimicrobial peptide 2	Leap2	0.82399	1.09961326	0.12954352
1433719_at	solute carrier family 9 (sodium/hydrogen exchanger), member 9	Slc9a9	0.82401	1.09301749	0.12365151
1448197_at	carcinoembryonic antigen-related cell adhesion molecule 11	Ceacam11	0.82401	1.06793925	0.09477906
1422311_a_at	polymerase (RNA) II (DNA directed) polypeptide A	Polr2a	0.82403	-1.09583499	-0.2353574
1416639_at	solute carrier family 2 (facilitated glucose transporter), member 5	Slc2a5	0.82404	1.07967182	0.10787132
1450238_at	glucosaminyl (N-acetyl) transferase 2, I-branching enzyme	Gcnt2	0.82404	-1.08647583	-0.1214163
1433303_at	RIKEN cDNA 4930483C01 gene	4930483C01Rik	0.82406	1.07804613	0.10809558
1417348_at	RIKEN cDNA 2310039H08 gene	2310039H08Rik	0.82407	1.09438625	0.11759663
1452780_at	general transcription factor IIIC, polypeptide 2, beta	Gtf3c2	0.82407	1.06336478	0.08715977
1428222_at	doublecortin-like kinase 2	Dclk2	0.82408	1.0767467	0.10637757
1417322_at	deoxyhypusine hydroxylase/monooxygenase	Dohh	0.82409	1.07901183	0.101144
1450437_a_at	neural cell adhesion molecule 1	Ncam1	0.8241	-1.08966293	-0.1320834
1449706_s_at	nuclear receptor subfamily 5, group A, member 2	Nr5a2	0.8241	-1.07273852	-0.1039359
1433094_at	solute carrier family 1 (glial high affinity glutamate transporter), n	Slc1a2	0.82411	1.08852741	0.11994868
1442347_at	low density lipoprotein receptor-related protein 8, apolipoprotein	Lrp8	0.82413	1.09895167	0.12728275
1453878_at	Ubiquitin protein ligase E3 component n-recognin 2	Ubr2	0.82413	-1.08547126	-0.1245776
1420853_at	syndecan 3	Sdc3	0.82414	-1.07361628	-0.1025671
1430833_at	IL2-inducible T-cell kinase	Itk	0.82414	1.08172351	0.11320198
1457837_at	---	---	0.82414	1.07938036	0.10685313
1451521_x_at	eukaryotic translation initiation factor 4H	Eif4h	0.82414	1.05408079	0.07565287
1456957_at	---	---	0.82415	1.09488265	0.1277441
1447480_at	---	---	0.82415	1.07864437	0.10465018
1449366_at	matrix metallopeptidase 8	Mmp8	0.82416	-1.08595784	-0.1202581
1444603_at	---	---	0.82416	1.08184228	0.1122043
1432198_at	---	---	0.82417	-1.08929755	-0.1419078
1452739_at	F-box protein 7	Fbxo7	0.82417	1.06926742	0.09484417
1416942_at	endoplasmic reticulum aminopeptidase 1	Erap1	0.82418	1.0928106	0.12742225
1434069_at	phosphatidylinositol-3,4,5-trisphosphate-dependent Rac exchang	Prex1	0.82418	1.085421	0.11473027
1423663_at	folliculin	Flcn	0.82419	1.06907603	0.09469784
1416052_at	phosphoribosyl pyrophosphate synthetase 1	Prps1	0.82419	1.05429292	0.07563329
1437939_s_at	cathepsin C	Ctsc	0.82419	1.07107596	0.09570838
1424565_at	polymerase (RNA) III (DNA directed) polypeptide D	Polr3d	0.82419	1.07163479	0.09654981
1428744_s_at	Bri3 binding protein	Bri3bp	0.8242	1.08262666	0.10532465
1439446_at	cDNA sequence BC048507	BC048507	0.82421	-1.09095981	-0.1316223
1444994_at	RIKEN cDNA A230038B01 gene	A230038B01Rik	0.82421	1.07232903	0.10025896
1454573_at	RIKEN cDNA 4933412L11 gene	4933412L11Rik	0.82422	1.10844004	0.13862752
1428095_a_at	C2 calcium-dependent domain containing 2-like	C2cd2l	0.82423	1.0799324	0.10781301
1416433_at	replication protein A2	Rpa2	0.82423	1.05586125	0.07754129
1424091_at	zinc finger protein 868	Zfp868	0.82424	-1.0691331	-0.0980365
1454931_at	EP300 interacting inhibitor of differentiation 2	Eid2	0.82425	1.06231588	0.08537728
1443507_at	---	---	0.82425	1.089005	0.11937345
1420190_at	---	---	0.82425	1.07942869	0.10742806
1448201_at	secreted frizzled-related protein 2	Sfrp2	0.82426	1.07817612	0.10831396
1451539_at	BAI1-associated protein 2-like 1	Baiap2l1	0.82427	-1.07723102	-0.1080168
1457110_at	pantothenate kinase 1	Pank1	0.82427	-1.0975069	-0.1490092
1435256_at	CAP-GLY domain containing linker protein 3	Clip3	0.82429	-1.08007993	-0.114051
1426562_a_at	olfactomedin 1	Olfm1	0.82429	1.12450303	0.15034902
1430214_a_at	RIKEN cDNA 2610020H08 gene	2610020H08Rik	0.8243	1.0827205	0.11245075
1451713_a_at	Fc receptor, IgE, low affinity II, alpha polypeptide	Fcer2a	0.82433	1.10773901	0.13811016
1439302_at	ubiquitin-like modifier activating enzyme 6	Uba6	0.82435	-1.07830283	-0.1130222
1424094_at	NIMA (never in mitosis gene a)-related expressed kinase 9	Nek9	0.82438	1.06807798	0.09319482
1429606_at	ubiquitin-like domain containing CTD phosphatase 1	Ublcp1	0.82439	-1.08236649	-0.1201058
1434921_at	nuclear receptor subfamily 2, group E, member 1	Nr2e1	0.8244	1.10247017	0.12664391
1454606_at	RIKEN cDNA 4933426M11 gene	4933426M11Rik	0.82444	1.0666341	0.0915881
1428118_at	leucine rich repeat and Ig domain containing 1	Lingo1	0.82444	1.09086849	0.12131837
1440546_at	RIKEN cDNA 9630002D21 gene	9630002D21Rik	0.82445	-1.08624773	-0.1323434
1432417_a_at	tetraspanin 2	Tspan2	0.82447	-1.07778196	-0.1110951
1440349_at	RIKEN cDNA 4930420K17 gene	4930420K17Rik	0.82447	1.09784746	0.12621937
1455546_s_at	splicing factor 3a, subunit 2	Sf3a2	0.82447	-1.0645451	-0.0971667
1424364_a_at	ubiquinol-cytochrome c reductase, complex III subunit X	Uqcr10	0.82448	1.06741138	0.08962807
1453806_at	NADH dehydrogenase (ubiquinone) 1 beta subcomplex, 2	Ndufb2	0.82449	1.07827395	0.1081064
1436948_a_at	family with sequence similarity 70, member A	Fam70a	0.8245	-1.08746475	-0.1307643
1452902_at	dehydrogenase/reductase (SDR family) member 13	Dhrs13	0.8245	1.07537076	0.10309212

1441821_at	---	---	0.82451	1.08046967	0.11102051
1431614_at	RIKEN cDNA 2810049E08 gene	2810049E08Rik	0.82451	-1.07750045	-0.1173118
1419973_at	3-oxoacyl-ACP synthase, mitochondrial	Oxsm	0.82451	-1.08320757	-0.119817
1420149_at	---	---	0.82451	-1.08390232	-0.1175135
1460026_s_at	zinc finger, FYVE domain containing 27	Zfyve27	0.82451	-1.08009908	-0.1142875
1423621_a_at	solute carrier family 33 (acetyl-CoA transporter), member 1	Slc33a1	0.82452	-1.08365271	-0.1364986
1423495_at	2-4-dienoyl-Coenzyme A reductase 2, peroxisomal	Decr2	0.82452	-1.07678067	-0.1091819
1418947_at	NIMA (never in mitosis gene a)-related expressed kinase 3	Nek3	0.82452	1.07370657	0.10117355
1448203_at	ATP synthase, H+ transporting, mitochondrial F0 complex, subunit	Atp5l /// Gm10221	0.82452	1.0803913	0.09999721
1417655_a_at	serrate RNA effector molecule homolog (Arabidopsis)	Srrt	0.82453	1.05711522	0.07871457
1454916_s_at	ADP-ribosylation factor interacting protein 1	Arfip1	0.82453	-1.06766879	-0.0969712
1423114_at	ubiquitin-conjugating enzyme E2D 3 (UBC4/5 homolog, yeast)	Ube2d3	0.82453	-1.06100016	-0.0896766
1448263_a_at	CNDP dipeptidase 2 (metallopeptidase M20 family)	Cndp2	0.82453	-1.06152598	-0.0907738
1445598_at	---	---	0.82454	1.08532036	0.11393149
1456127_at	canopy 1 homolog (zebrafish)	Cnpy1	0.82455	1.07246137	0.09975202
1425968_s_at	SPEG complex locus	Speg	0.82455	-1.07653654	-0.1085139
1459663_at	---	---	0.82456	-1.07947641	-0.1113353
1443963_at	AF4/FMR2 family, member 2	Aff2	0.82456	-1.07708999	-0.1206036
1417843_s_at	EPS8-like 2	Eps8l2	0.82456	1.10923652	0.13304137
1456018_at	bromodomain containing 9	Brd9	0.82456	1.06733213	0.09303931
1422814_at	asp (abnormal spindle)-like, microcephaly associated (Drosophila	Aspm	0.82456	-1.0572713	-0.0815508
1424753_at	nudix (nucleoside diphosphate linked moiety X)-type motif 14	Nudt14	0.82457	1.10913935	0.12896806
1429500_at	protein phosphatase 1A, magnesium dependent, alpha isoform	Ppm1a	0.82457	-1.09550893	-0.1581717
1423388_at	adaptor protein complex AP-1, gamma 1 subunit	Ap1g1	0.82457	1.06352861	0.08777957
1432535_at	RIKEN cDNA 5530400C23 gene	5530400C23Rik	0.82457	-1.08592862	-0.1258982
1416607_at	RIKEN cDNA 4931406C07 gene	4931406C07Rik	0.82458	1.0696586	0.09482496
1419485_at	forkhead box C1	Foxc1	0.82459	1.10409057	0.12927311
1427213_at	6-phosphofructo-2-kinase/fructose-2,6-biphosphatase 1	Pfkfb1	0.82459	1.08862501	0.11414325
1442588_at	predicted gene 5101	Gm5101	0.82459	1.1116324	0.13981427
1448690_at	potassium channel, subfamily K, member 1	Kcnk1	0.8246	1.07980159	0.10884306
1421113_at	pepsinogen 5, group I	Pga5	0.8246	1.08175639	0.11295468
1419767_at	peptidyl arginine deiminase, type III	Padi3	0.8246	-1.08521658	-0.122358
1451782_a_at	solute carrier family 29 (nucleoside transporters), member 1	Slc29a1	0.82461	1.08120671	0.10263988
1417904_at	DNA cross-link repair 1A, PSO2 homolog (S. cerevisiae)	Dclre1a	0.82462	1.09194165	0.11665709
1422903_at	lymphocyte antigen 86	Ly86	0.82462	1.1105276	0.13814265
1429343_at	RNA binding motif protein, X-linked-like 2	Rbmxl2	0.82462	1.07055232	0.09399107
1420163_at	expressed sequence AA511261	AA511261	0.82462	-1.09121925	-0.1336713
1445671_at	---	---	0.82462	1.08994833	0.12153453
1446706_at	expressed sequence C79123	C79123	0.82462	-1.08214277	-0.1142383
1460061_at	---	---	0.82462	-1.0852287	-0.1192148
1455607_at	R-spondin 3 homolog (Xenopus laevis)	Rspo3	0.82463	1.12728501	0.15368084
1430323_at	RIKEN cDNA 1700049E17 gene, gene 1 /// tandem duplication of	1700049E17Rik1 /// 1700	0.82463	1.10822965	0.13585942
1426922_s_at	ArfGAP with FG repeats 1	Agfg1	0.82464	-1.06028003	-0.0852135
1434508_at	ubiquitin-conjugating enzyme E2Q (putative) 1	Ube2q1	0.82464	1.07607253	0.10202931
1433993_at	RIKEN cDNA 4931406P16 gene	4931406P16Rik	0.82465	1.07152179	0.09634857
1426338_a_at	netrin G1	Ntng1	0.82465	1.08872943	0.12149803
1418163_at	toll-like receptor 4	Tlr4	0.82465	1.10218285	0.13201249
1435497_at	RIKEN cDNA 5730590G19 gene	5730590G19Rik	0.82465	-1.0730536	-0.1123796
1422638_s_at	Ras association (RalGDS/AF-6) domain family member 5	Rassf5	0.82465	-1.09276184	-0.1286687
1441128_at	RIKEN cDNA 2900016J10 gene	2900016J10Rik	0.82466	-1.09445852	-0.150738
1434591_at	zinc finger protein 862	Zfp862	0.82466	1.09261282	0.12097241
1419894_at	proteasome (prosome, macropain) subunit, beta type 7 pseudoge	Gm6787	0.82466	-1.09488549	-0.1480456
1454303_at	epsin 2	Epn2	0.82466	1.08731525	0.11566085
1430359_a_at	NAD synthetase 1	Nadsyn1	0.82467	1.08308323	0.11379012
1420713_a_at	MyoD family inhibitor	Mdfi	0.82468	-1.10050748	-0.194898
1432877_at	RIKEN cDNA 4930544N03 gene	4930544N03Rik	0.82468	-1.08436045	-0.1238671
1433891_at	leucine-rich repeat-containing G protein-coupled receptor 4	Lgr4	0.82469	1.08304426	0.10461205
1447210_at	---	---	0.82469	1.08682837	0.1197719
1425076_at	DnaJ (Hsp40) homolog, subfamily C, member 18	Dnajc18	0.8247	1.08630417	0.11316065
1419309_at	podoplanin	Pdpn	0.82471	1.06008982	0.08414932
1440651_at	---	---	0.82471	1.14809495	0.16974888
1435302_at	TAF4B RNA polymerase II, TATA box binding protein (TBP)-associ	Taf4b	0.82471	-1.06231465	-0.089275
1427496_at	centrosomal protein 152	Cep152	0.82471	1.07812241	0.10755285
1433862_at	extra spindle poles-like 1 (S. cerevisiae)	Espl1	0.82472	1.06303837	0.08577415
1446809_at	RIKEN cDNA A230059L01 gene	A230059L01Rik	0.82472	-1.07452715	-0.108486
1419674_a_at	dipeptidase 1 (renal)	Dpep1	0.82472	-1.0960067	-0.1460081
1430904_at	ADP-ribosylation factor GTPase activating protein 3	Arfgap3	0.82472	1.09660037	0.12503634
1445279_at	---	---	0.82474	1.08269204	0.11031024
1455450_at	protein tyrosine phosphatase, non-receptor type 3	Ptpn3	0.82475	1.07615023	0.10486091
1456496_at	Death-associated protein kinase 3	Dapk3	0.82476	-1.09350289	-0.142626
1435822_at	RIKEN cDNA D830012I24 gene	D830012I24Rik	0.82476	-1.07473047	-0.1089725
1453316_at	protease, serine, 55	Prss55	0.82477	1.07918601	0.10973758
1460232_s_at	hydroxy-delta-5-steroid dehydrogenase, 3 beta- and steroid delta	Hsd3b2 /// Hsd3b3 /// Hs	0.82478	-1.07774379	-0.1089316
1456740_x_at	component of oligomeric golgi complex 1 /// conserved oligomer	Cog1 /// LOC100504959	0.82479	-1.07610842	-0.1070639
1449125_at	tumor necrosis factor, alpha-induced protein 8-like 1	Tnfaip8l1	0.82479	1.07573056	0.10434018
1418642_at	lymphocyte cytosolic protein 2	Lcp2	0.8248	1.10035695	0.1309374
1450996_at	follicle stimulating hormone beta	Fshb	0.8248	1.09711842	0.12684089
1423480_at	nucleolar protein 11	Nol11	0.8248	1.06388398	0.08803786
1441373_at	---	---	0.82481	-1.08357078	-0.1185822
1438317_a_at	endonuclease G	Endog	0.82481	1.0703402	0.09726205
1428045_a_at	E74-like factor 2	Elf2	0.82482	-1.0778835	-0.1114109
1435967_s_at	3-hydroxyisobutyrate dehydrogenase	Hibadh	0.82484	-1.05598971	-0.0786769
1435569_at	RIKEN cDNA D630029K05 gene	D630029K05Rik	0.82484	1.07449232	0.10124472
1418174_at	D site albumin promoter binding protein	Dbp	0.82484	1.09636466	0.12596151
1426642_at	fibronectin 1	Fn1	0.82485	1.06117885	0.08310965
1432468_at	lipocalin 9	Lcn9	0.82485	-1.08909504	-0.1241144
1450799_at	adenylate cyclase activating polypeptide 1 receptor 1	Adcyap1r1	0.82485	1.07972126	0.10932997
1425383_a_at	pre B-cell leukemia transcription factor 1	Pbx1	0.82486	-1.08654121	-0.1282656
1434319_at	malate dehydrogenase 1, NAD (soluble)	Mdh1	0.82486	-1.08971311	-0.1335887
1422036_at	striatin, calmodulin binding protein	Strn	0.82487	-1.0891294	-0.1269185
1428497_at	SECIS binding protein 2	Secisbp2	0.82487	-1.06992064	-0.0982831
1424708_at	transmembrane emp24-like trafficking protein 10 (yeast)	Tmed10	0.82487	1.06033766	0.083005
1438789_s_at	dihydropyrimidinase-like 3	Dpysl3	0.82488	1.10575379	0.13276001
1452130_at	thioredoxin-related transmembrane protein 2	Tmx2	0.82489	-1.06662292	-0.0967461

1422623_x_at	ribosomal protein L41	Rpl41	0.8249	1.02970971	0.04142229
1432242_at	RIKEN cDNA 4930447N08 gene	4930447N08Rik	0.82491	-1.07981875	-0.1180064
1435818_at	kelch-like 15 (Drosophila)	Klhl15	0.82491	-1.0919366	-0.1431179
1442227_at	---	---	0.82491	1.0659018	0.09154741
1439877_at	---	---	0.82492	1.13108081	0.15149913
1429719_at	forkhead box P4	Foxp4	0.82492	-1.06514047	-0.095522
1415887_at	Trk-fused gene	Tfg	0.82493	1.06088391	0.08440539
1438136_at	astacin-like metalloendopeptidase (M12 family)	Astl	0.82495	-1.08673662	-0.1202994
1443548_at	---	---	0.82495	-1.06750123	-0.0942546
1458243_at	Predicted gene 7844	Gm7844	0.82495	1.12301889	0.14504488
1433632_at	interferon regulatory factor 2 binding protein 2	Irf2bp2	0.82496	1.09857933	0.12510993
1434746_at	MAX gene associated	Mga	0.82496	-1.0608568	-0.0897696
1424523_at	engulfment and cell motility 1, ced-12 homolog (C. elegans)	Elmo1	0.82497	1.07061699	0.09810624
1444293_at	cDNA sequence BC028471	BC028471	0.82497	1.13250726	0.15720699
1451885_at	---	---	0.82497	1.07720683	0.10510565
1439861_at	zinc finger protein 583	Zfp583	0.82501	-1.08520086	-0.1354203
1453056_at	solute carrier family 16 (monocarboxylic acid transporters), memb	Slc16a13	0.82501	1.08288716	0.11242411
1442104_at	---	---	0.82501	1.10301378	0.1365307
1438585_at	synaptic nuclear envelope 2	Syne2	0.82502	1.09464938	0.12090045
1425512_at	mitogen-activated protein kinase kinase 7	Map2k7	0.82503	1.07461877	0.09801997
1430385_a_at	galactosidase, beta 1-like	Glb1l	0.82504	1.10347765	0.13339693
1460200_s_at	leucine zipper transcription factor-like 1	Lztfl1	0.82509	-1.08312776	-0.1233113
1452057_at	ARP1 actin-related protein 1 homolog B, centractin beta (yeast)	Actr1b	0.82509	1.06977635	0.09373007
1445657_at	expressed sequence AW111846	AW111846	0.82509	1.09311573	0.1238775
1438008_at	golgi associated, gamma adaptin ear containing, ARF binding prot	Gga3	0.8251	1.07067677	0.09672693
1452692_a_at	NADH dehydrogenase (ubiquinone) flavoprotein 2	Ndufv2	0.8251	-1.06032964	-0.0892051
1457093_at	GTPase activating protein and VPS9 domains 1	Gapvd1	0.8251	-1.07455144	-0.1056104
1451945_at	---	---	0.82511	1.098367	0.12203296
1430365_at	nuclear transcription factor, X-box binding-like 1	Nfxl1	0.82512	1.08606023	0.11599337
1425721_at	pleckstrin homology domain interacting protein	Phip	0.82513	-1.1018077	-0.1743846
1428707_at	parathymosin	Ptms	0.82514	1.08070464	0.10245113
1434478_at	headcase homolog (Drosophila)	Heca	0.82515	1.08351071	0.11061169
1442062_at	RIKEN cDNA 7120426M23 gene	7120426M23Rik	0.82516	-1.06581692	-0.0943591
1424015_at	DENN/MADD domain containing 5A	Dennd5a	0.82517	1.05826097	0.08141472
1423442_a_at	F-box and WD-40 domain protein 2	Fbxw2	0.82519	1.05963118	0.08280579
1445231_at	---	---	0.82519	1.12755054	0.15143462
1460689_at	PPPDE peptidase domain containing 2	Pppde2	0.8252	1.0625076	0.08671172
1422989_a_at	dystrophia myotonica-protein kinase	Dmpk	0.8252	-1.08614585	-0.1247552
1445496_at	AU RNA binding protein/enoyl-coenzyme A hydratase	Auh	0.8252	1.08332398	0.11252333
1428504_at	X-ray repair complementing defective repair in Chinese hamster c	Xrcc6	0.82521	-1.09971169	-0.1683201
1435452_at	transmembrane protein 20	Tmem20	0.82521	-1.06605934	-0.0942846
1454837_at	ceroid-lipofuscinosis, neuronal 6	Cln6	0.82522	1.08195094	0.10890326
1456960_at	---	---	0.82525	1.14219696	0.16400348
1430650_at	zinc finger protein 191	Zfp191	0.82526	1.07537348	0.10414648
1449526_a_at	glycerophosphodiester phosphodiesterase domain containing 3	Gdpd3	0.82526	1.08681677	0.11826741
1421310_at	RIKEN cDNA 4921517D21 gene	4921517D21Rik	0.82526	1.11691006	0.14442464
1438356_x_at	RIKEN cDNA 4933432K03 gene	4933432K03Rik	0.82527	1.12682588	0.14940267
1441811_x_at	transmembrane protein 176A	Tmem176a	0.82527	-1.08956756	-0.1367723
1432884_at	family with sequence similarity 23, member A	Fam23a	0.82528	-1.07347992	-0.1034428
1459111_at	RIKEN cDNA 4831440E17 gene	4831440E17Rik	0.82528	-1.07983594	-0.1121141
1433037_at	RIKEN cDNA 5830434F19 gene	5830434F19Rik	0.82529	1.10171623	0.13194742
1440740_at	CTD (carboxy-terminal domain, RNA polymerase II, polypeptide A	Ctdspl2	0.82529	-1.09232945	-0.1467285
1416133_at	EFR3 homolog A (S. cerevisiae)	Efr3a	0.82531	-1.06582647	-0.0978419
1433196_at	RIKEN cDNA 9530006O14 gene	9530006O14Rik	0.82532	-1.09151969	-0.137617
1458036_at	---	---	0.82532	1.1233761	0.14536152
1422077_at	acyl-CoA thioesterase 4	Acot4	0.82532	-1.08044904	-0.1140713
1454610_at	septin 7	Sep-07	0.82532	-1.05476885	-0.078312
1439095_at	serine/arginine-rich splicing factor 11	Srsf11	0.82533	-1.08616369	-0.1217919
1431828_a_at	synaptojanin 2	Synj2	0.82534	-1.09157097	-0.1311023
1434593_at	eukaryotic translation initiation factor 5A2	Eif5a2	0.82534	-1.09105863	-0.1459878
1423556_at	aldo-keto reductase family 1, member B7	Akr1b7	0.82534	1.0869331	0.11800675
1441509_at	RIKEN cDNA A130009I22 gene	A130009I22Rik	0.82535	-1.08344688	-0.119518
1422544_at	myosin X	Myo10	0.82537	1.06083553	0.08388847
1422907_at	guanine nucleotide binding protein, alpha transducing 2	Gnat2	0.82539	-1.08107881	-0.1126335
1428720_s_at	RIKEN cDNA 2010309G21 gene /// immunoglobulin lambda chain	2010309G21Rik /// Igl-C2	0.82542	-1.08402771	-0.119833
1432017_at	huntingtin interacting protein 1	Hip1	0.82543	-1.08559159	-0.1290472
1439448_x_at	transmembrane emp24 protein transport domain containing 9	Tmed9	0.82544	-1.05755954	-0.0815521
1460087_at	cDNA sequence BC017643	BC017643	0.82547	-1.08678597	-0.1236753
1456744_x_at	folliculin	Flcn	0.82547	-1.08041784	-0.1184917
1449337_at	tryptophan 2,3-dioxygenase	Tdo2	0.82547	-1.08016306	-0.1159785
1454339_at	RIKEN cDNA 4930551I15 gene	4930551I15Rik	0.82548	-1.07884522	-0.1099848
1425119_at	2'-5' oligoadenylate synthetase 1B	Oas1b	0.8255	-1.07870551	-0.1116382
1446124_at	---	---	0.8255	1.09154583	0.12534785
1441981_at	transmembrane protease, serine 11f	Tmprss11f	0.82552	1.07415036	0.10019605
1452734_at	ribonuclease T2A /// ribonuclease T2B	Rnaset2a /// Rnaset2b	0.82553	1.0646828	0.08692992
1455655_a_at	TAR DNA binding protein	Tardbp	0.82553	-1.05672077	-0.0804284
1456849_at	---	---	0.82553	-1.09555469	-0.1556022
1434936_at	HIRA interacting protein 3	Hirip3	0.82555	1.06312103	0.08559157
1454758_a_at	TSC22 domain family, member 1	Tsc22d1	0.82555	1.05953948	0.07883617
1442095_at	additional sex combs like 3 (Drosophila)	Asxl3	0.82555	1.1085611	0.13843925
1432220_at	RIKEN cDNA 4933430L12 gene	4933430L12Rik	0.82556	-1.0796761	-0.1115571
1422950_at	hairy and enhancer of split 7 (Drosophila)	Hes7	0.82556	1.08123556	0.11227105
1416845_at	transmembrane protein 132A	Tmem132a	0.82556	1.08015394	0.10664022
1415896_x_at	small nuclear ribonucleoprotein N /// SNRPN upstream reading fra	Snrpn /// Snurf	0.82557	1.06468625	0.0865528
1444893_at	---	---	0.82558	-1.08527281	-0.1207108
1429652_at	proline-rich coiled-coil 1	Prrc1	0.82558	1.06415137	0.08820527
1442486_at	---	---	0.82558	1.0969986	0.12205262
1429975_at	protease, serine, 54	Prss54	0.82559	-1.0968666	-0.1557975
1445524_at	---	---	0.82559	-1.08575376	-0.1242736
1418051_at	Eph receptor B6	Ephb6	0.8256	1.09856212	0.12869808
1419441_at	ribosomal protein, large, P0	Rplp0	0.82561	-1.03214161	-0.0471585
1457172_at	Rho guanine nucleotide exchange factor (GEF) 15	Arhgef15	0.82561	1.10431971	0.13167232
1420453_at	crystallin, gamma S	Crygs	0.82561	-1.09566616	-0.1428226
1420349_at	prostaglandin F receptor	Ptgfr	0.82563	-1.08356029	-0.1207847

1432256_at	RIKEN cDNA 1700111N16 gene	1700111N16Rik	0.82563	-1.08125481	-0.1201617
1428185_at	potassium channel tetramerisation domain containing 18	Kctd18	0.82564	1.079838	0.10896645
1431938_a_at	phosphomannomutase 2	Pmm2	0.82564	1.08086754	0.10594713
1426979_at	MLX interacting protein	Mlxip	0.82566	1.07645925	0.1036646
1452901_at	cAMP responsive element binding protein 1	Creb1	0.82566	-1.07122627	-0.1066677
1459952_at	ankyrin repeat domain 17	Ankrd17	0.82569	-1.08454001	-0.1264832
1430709_at	RIKEN cDNA 4833405L11 gene	4833405L11Rik	0.82574	-1.08470284	-0.1221918
1457840_at	plexin A4	Plxna4	0.82574	-1.08748965	-0.1276747
1423778_at	ubiquitin specific peptidase 20	Usp20	0.82575	-1.07972153	-0.1125779
1421570_at	interleukin 9 receptor	Il9r	0.82594	1.0897891	0.11885893
1453754_at	S100P binding protein	S100pbp	0.82595	-1.09398739	-0.1857356
1431463_at	coiled-coil domain containing 30	Ccdc30	0.82598	-1.07524778	-0.1105583
1455123_at	suppression of tumorigenicity 18	St18	0.826	1.12774185	0.14585425
1456278_x_at	G protein-coupled receptor 172B	Gpr172b	0.82615	1.0849256	0.11560073
1443948_at	tetratricopeptide repeat domain 9C	Ttc9c	0.82619	-1.07154579	-0.1017701
1458022_at	RIKEN cDNA D830039M14 gene	D830039M14Rik	0.82623	-1.07983689	-0.1111317
1453818_a_at	RIKEN cDNA 2210404J11 gene	2210404J11Rik	0.82623	1.09723533	0.1228294
1438876_at	RIKEN cDNA A130014A01 gene	A130014A01Rik	0.82626	1.14511506	0.16776593
AFFX-r2-Ec-bioD-	---	---	0.82626	1.07279293	0.09246447
1426498_at	lysine (K)-specific demethylase 5C	Kdm5c	0.82628	-1.0872222	-0.1242686
1431564_at	breast cancer anti-estrogen resistance 3	Bcar3	0.82629	-1.09106583	-0.1338522
1450242_at	toll-like receptor 5	Tlr5	0.82629	-1.08771046	-0.1236986
1427003_at	protein phosphatase 2, regulatory subunit B (B56), gamma isoform	Ppp2r5c	0.8263	1.06012217	0.08211594
1437816_at	Paired box gene 6	Pax6	0.8263	1.09093768	0.12105765
1455977_x_at	kallikrein 1	Klk1	0.82632	1.0872597	0.11755111
1433187_at	RIKEN cDNA B230112I24 gene	B230112I24Rik	0.82633	-1.08546881	-0.1199692
1428098_a_at	transmembrane 7 superfamily member 3	Tm7sf3	0.82633	1.0705723	0.09352431
1450704_at	Indian hedgehog	Ihh	0.82634	-1.07719504	-0.1282847
1457178_at	---	---	0.82635	-1.08822458	-0.1225605
1449097_at	thioredoxin reductase 2	Txnrd2	0.82636	1.07761207	0.10728888
1422328_at	gap junction protein, alpha 10	Gja10	0.82636	1.09194826	0.12402263
1442253_at	Excision repair cross-complementing rodent repair deficiency, com	Ercc2	0.82637	-1.08211414	-0.1174609
1420017_at	tetraspanin 8	Tspan8	0.82639	-1.08787348	-0.1293163
1437987_at	---	---	0.82639	-1.0820164	-0.1196205
1457071_x_at	RIKEN cDNA C130013H08 gene	C130013H08Rik	0.8264	-1.07497441	-0.1051169
1445580_at	---	---	0.82641	-1.09028676	-0.1349112
1458783_at	---	---	0.82642	1.08159964	0.11019271
1433205_at	Nedd4 family interacting protein 2	Ndfip2	0.82642	1.09684614	0.12699955
1421220_at	ankyrin repeat domain 17	Ankrd17	0.82643	1.10173538	0.13272259
1421820_a_at	neurofibromatosis 2	Nf2	0.82643	1.06071679	0.08389898
1450609_at	lipocalin 8	Lcn8	0.82644	-1.08648802	-0.1220003
1434597_at	La ribonucleoprotein domain family, member 4B	Larp4b	0.82644	-1.05665566	-0.0825279
1415889_a_at	heat shock protein 90, beta (Grp94), member 1	Hsp90b1	0.82645	-1.05966245	-0.0880352
1447338_at	---	---	0.82646	1.09225579	0.12330625
1454648_s_at	DNA segment, Chr 10, Wayne State University 102, expressed	D10Wsu102e	0.82648	1.06163863	0.08392857
1436394_at	tripartite motif-containing 37	Trim37	0.82651	1.08203304	0.10506593
1432871_at	RIKEN cDNA 4932429P19 gene	4932429P19Rik	0.82652	1.07821411	0.10644731
1420590_at	hyaluronan synthase1	Has1	0.82655	-1.07890835	-0.1175484
1440878_at	runt related transcription factor 1	Runx1	0.82657	1.12097679	0.14729105
1440765_at	Fraser syndrome 1 homolog (human)	Fras1	0.82658	1.10555871	0.12891643
1416581_at	WD repeat domain 5	Wdr5	0.82661	1.05392426	0.07529121
1419660_at	RIKEN cDNA 1600012F09 gene	1600012F09Rik	0.82662	-1.06503181	-0.0980573
1433776_at	lipoma HMGIC fusion partner	Lhfp	0.82663	1.10143593	0.13222202
1459689_at	---	---	0.82663	-1.08774934	-0.1298238
1419738_a_at	tropomyosin 2, beta	Tpm2	0.82664	-1.06299605	-0.0910223
1440390_at	family with sequence similarity 171, member A2	Fam171a2	0.82664	-1.09198444	-0.130195
1451905_a_at	myxovirus (influenza virus) resistance 1	Mx1	0.82665	1.09188564	0.12303202
1420479_a_at	nucleosome assembly protein 1-like 1	Nap1l1	0.82666	-1.05335503	-0.0752799
1446123_at	tetratricopeptide repeat domain 34	Ttc34	0.82668	-1.08073046	-0.1228136
1450628_at	solute carrier family 2, (facilitated glucose transporter), member 8	Slc2a8	0.8267	1.06606171	0.0893006
1417432_a_at	guanine nucleotide binding protein (G protein), beta 1	Gnb1	0.82691	-1.05461403	-0.0780761
1432101_a_at	testis-specific serine kinase 4	Tssk4	0.82696	-1.08675646	-0.124218
1445242_at	glycerol kinase	Gyk	0.82703	1.10018785	0.13172214
1423205_at	transmembrane 9 superfamily protein member 4	Tm9sf4	0.82704	1.06815465	0.09282161
1429053_at	RIKEN cDNA 1110012J17 gene	1110012J17Rik	0.82706	-1.07002137	-0.1024493
1446917_at	---	---	0.82706	-1.08142205	-0.114184
1458621_at	WD repeat domain 78	Wdr78	0.82707	-1.08880613	-0.1267243
1437664_at	homeobox D3	Hoxd3	0.82709	-1.09087159	-0.1378392
1431878_at	grainyhead-like 2 (Drosophila)	Grhl2	0.82709	1.09600744	0.12620694
1458019_at	---	---	0.82709	1.07845679	0.10595343
1418009_at	GC-rich sequence DNA-binding factor 1	Gcfc1	0.8271	-1.0814	-0.1206369
1439532_s_at	kinesin family member 13A	Kif13a	0.8271	-1.09521311	-0.1529248
1436979_x_at	RNA binding motif protein 14	Rbm14	0.82712	1.05413123	0.07602309
1426644_at	TBC1 domain family, member 20	Tbc1d20	0.82712	1.0580958	0.08128401
1423358_at	endothelin converting enzyme 2	Ece2	0.82712	1.06549702	0.0909889
1452725_a_at	ribonuclease H2, large subunit	Rnaseh2a	0.82713	1.06971958	0.09234444
1443904_at	fatty acid desaturase domain family, member 6	Fads6	0.82714	1.08929699	0.12047495
1427251_at	ATPase, Ca++ transporting, cardiac muscle, slow twitch 2	Atp2a2	0.82714	1.07603848	0.10428158
1455173_at	G1 to S phase transition 1	Gspt1	0.82714	-1.06671312	-0.1015496
1455554_at	RIKEN cDNA A830039N20 gene	A830039N20Rik	0.82714	-1.08106001	-0.1131259
1431233_at	cyclin M4	Cnnm4	0.82715	1.101298	0.12413348
1451822_a_at	secernin 2	Scrn2	0.82716	1.08616788	0.11563051
1445213_at	cylindromatosis (turban tumor syndrome)	Cyld	0.82716	1.10849818	0.13639511
1457713_at	excision repair cross-complementing rodent repair deficiency, com	Ercc5	0.82716	-1.07705232	-0.1104262
1429472_at	RIKEN cDNA 4921525O09 gene	4921525O09Rik	0.82717	-1.08367637	-0.1166905
1424547_at	carbonic anhydrase 10	Car10	0.82717	-1.06851487	-0.0967568
1425007_at	zinc finger protein 566	Zfp566	0.82718	1.07424174	0.09780892
1457038_at	Fras1 related extracellular matrix protein 2	Frem2	0.82718	1.14887633	0.16830299
1418255_s_at	serum response factor	Srf	0.82718	-1.07042889	-0.1068354
1445688_at	---	---	0.8272	1.07343102	0.10056729
1436717_x_at	hemoglobin Y, beta-like embryonic chain /// hemoglobin subunit	Hbb-y /// LOC100503273	0.8272	1.07010057	0.09531569
1415754_at	polymerase (RNA) II (DNA directed) polypeptide F	Polr2f	0.82721	-1.05271541	-0.0747237
1416687_at	procollagen lysine, 2-oxoglutarate 5-dioxygenase 2	Plod2	0.82721	1.09360599	0.1144606
1448021_at	family with sequence similarity 46, member C	Fam46c	0.82721	1.16717937	0.17840016

1422720_at	ISL1 transcription factor, LIM/homeodomain	Isl1	0.82722	1.09273723	0.12198044
1452486_a_at	crystallin, alpha A	Cryaa	0.82722	-1.0806082	-0.1138101
1436917_s_at	G-protein signalling modulator 1 (AGS3-like, C. elegans)	Gpsm1	0.82724	1.10693463	0.13448172
1421139_a_at	zinc finger protein 386 (Kruppel-like)	Zfp386	0.82726	1.10770976	0.13733382
1457757_at	TOX high mobility group box family member 2	Tox2	0.82727	1.08947859	0.12162398
1426121_at	MAS-related GPR, member A3	Mrgpra3	0.82728	-1.0863101	-0.1308543
1454804_at	nuclear protein localization 4 homolog (S. cerevisiae)	Nploc4	0.82728	1.06802114	0.09178919
1437087_at	RIKEN cDNA 2210408K08 gene	2210408K08Rik	0.82729	1.07897785	0.10902507
1455478_at	protein tyrosine phosphatase, receptor type, f polypeptide (PTPRF	Ppfia1	0.82732	1.07118777	0.09913809
1416964_at	eukaryotic elongation factor, selenocysteine-tRNA-specific	Eefsec	0.82734	1.06239331	0.08692166
1457283_at	neuregulin 2	Nrg2	0.82734	1.10437577	0.13219672
1456706_at	RIKEN cDNA 4833441D16 gene	4833441D16Rik	0.82737	1.11286449	0.13924283
1443779_s_at	ligand dependent nuclear receptor corepressor	Lcor	0.82739	-1.08668233	-0.137825
1430540_at	coiled-coil-helix-coiled-coil-helix domain containing 8	Chchd8	0.82741	-1.0825747	-0.1154042
1456082_x_at	chaperonin containing Tcp1, subunit 4 (delta)	Cct4	0.82741	-1.07033199	-0.1026497
1426899_at	TBC1 domain family, member 23	Tbc1d23	0.82742	1.06808894	0.0925117
1438606_a_at	chloride intracellular channel 4 (mitochondrial)	Clic4	0.82743	-1.06068206	-0.0880876
1429162_at	RIKEN cDNA 1500015A07 gene	1500015A07Rik	0.82743	1.15934523	0.17386255
1456513_at	---	---	0.82744	1.09496855	0.12216958
1442509_at	---	---	0.82745	1.12346082	0.1486714
1416526_a_at	Parkinson disease (autosomal recessive, early onset) 7	Park7	0.82746	1.05784583	0.07885762
1449373_at	DnaJ (Hsp40) homolog, subfamily C, member 3	Dnajc3	0.82746	-1.06671221	-0.0948665
1460742_at	asparagine-linked glycosylation 3 homolog (yeast, alpha-1,3-man	Alg3	0.82747	1.0619565	0.08564373
1445407_at	---	---	0.82747	-1.08057536	-0.1122485
1453596_at	inhibitor of DNA binding 2	Id2	0.82747	1.07587183	0.10530548
1442235_at	Pleiomorphic adenoma gene-like 2	Plagl2	0.82748	-1.09153736	-0.1367341
1445289_at	---	---	0.82749	-1.09158412	-0.1355622
1448893_at	nuclear receptor co-repressor 2	Ncor2	0.82751	1.07133562	0.09616359
1444179_at	RIKEN cDNA A530023O14 gene	A530023O14Rik	0.82752	1.12257061	0.14831286
1423412_at	RNA binding motif protein 47	Rbm47	0.82753	-1.07317647	-0.1050318
1434923_at	COX19 cytochrome c oxidase assembly homolog (S. cerevisiae)	Cox19	0.82753	1.06379845	0.08859759
1418441_at	collagen, type VIII, alpha 1	Col8a1	0.82753	-1.08936845	-0.1332603
1429224_at	paraneoplastic antigen MA1	Pnma1	0.82754	1.08932399	0.11960991
1436819_at	septin 6	Sep-06	0.82755	1.07945793	0.109128
1434975_x_at	phosphatidylserine decarboxylase, pseudogene 3	Pisd-ps3	0.82755	-1.0577331	-0.0822723
1451548_at	uridine phosphorylase 2	Upp2	0.82756	1.06620272	0.09035137
1457070_at	---	---	0.82756	-1.09674209	-0.1498211
1458143_at	a disintegrin-like and metallopeptidase (reprolysin type) with thro	Adamts3	0.82756	-1.07713769	-0.112161
1431001_at	RIKEN cDNA 9030624J02 gene	9030624J02Rik	0.82756	1.10873534	0.13610157
1420105_at	DNA segment, Chr 13, ERATO Doi 205, expressed	D13Ertd205e	0.82756	1.07738598	0.10609672
1419804_at	RIKEN cDNA 1700051A21 gene	1700051A21Rik	0.82757	-1.08648033	-0.1256162
1450718_at	SH2B adaptor protein 2	Sh2b2	0.82757	1.10212834	0.13089336
1446897_at	---	---	0.82758	1.08176018	0.11237181
1420470_at	sulfotransferase family, cytosolic, 1C, member 1	Sult1c1	0.8276	-1.08833706	-0.1259508
1454510_at	RIKEN cDNA 2900034C19 gene	2900034C19Rik	0.8276	-1.07960665	-0.113341
1426591_at	G elongation factor, mitochondrial 2	Gfm2	0.82761	1.09836616	0.12723896
1450913_at	UDP-Gal:betaGlcNAc beta 1,4-galactosyltransferase, polypeptide	B4galt6	0.82761	-1.08109748	-0.1312256
1454378_at	hypothetical LOC100503202 /// hypothetical LOC100504854	LOC100503202 /// LOC10	0.82761	-1.07770434	-0.1094037
1418257_at	solute carrier family 12, member 7	Slc12a7	0.82761	1.06572082	0.08984228
1426647_at	RIKEN cDNA 9130011J15 gene	9130011J15Rik	0.82762	-1.08209273	-0.1155491
1447274_at	---	---	0.82762	-1.08220428	-0.1234777
1445155_at	---	---	0.82762	1.10811662	0.13657833
1431455_at	transmembrane protein 30C	Tmem30c	0.82764	-1.08368329	-0.1161249
1445201_at	zinc finger protein 53	Zfp53	0.82764	-1.08574488	-0.122532
1435910_at	fatty acid desaturase 3	Fads3	0.82766	-1.08396909	-0.1174083
1454818_at	glucocorticoid modulatory element binding protein 2	Gmeb2	0.82766	1.06644685	0.09219336
1458212_at	RIKEN cDNA A730009E18 gene	A730009E18Rik	0.82768	-1.08744035	-0.1286973
1420899_at	RAB18, member RAS oncogene family	Rab18	0.82769	1.06857179	0.09259819
1424449_at	fibroblast growth factor receptor substrate 3	Frs3	0.82769	1.07609879	0.10339756
1457198_at	neuropilin 1	Nrp1	0.8277	-1.08594748	-0.1194555
1450862_at	RAD54 like (S. cerevisiae)	Rad54l	0.8277	1.06121225	0.08512169
1434752_at	hypothetical LOC100044751	LOC100044751	0.82772	-1.09707119	-0.1739702
1451373_at	UDP glycosyltransferases 3 family, polypeptide A1	Ugt3a1	0.82773	-1.08563141	-0.1245842
1427613_at	cDNA sequence AY074887	AY074887	0.82773	-1.07945036	-0.1113765
1447822_x_at	UFM1-specific peptidase 1	Ufsp1	0.82774	-1.08658863	-0.1240315
1439606_at	katanin p60 subunit A-like 1	Katnal1	0.82774	1.10046478	0.1308541
1430099_at	RIKEN cDNA 4930509E16 gene	4930509E16Rik	0.82776	1.14042913	0.1612199
1442213_at	hypothetical LOC552908	LOC552908	0.82778	-1.07103778	-0.1013696
1435523_s_at	RIKEN cDNA 2700089E24 gene	2700089E24Rik	0.82778	-1.080693	-0.128007
1426030_a_at	acylpeptide hydrolase	Apeh	0.82781	1.063571	0.08674727
1457428_at	hypothetical protein C230040D14	C230040D14	0.82783	1.08863419	0.12132937
1440085_at	ectodysplasin A2 receptor	Eda2r	0.82784	-1.06228145	-0.0885142
1425517_s_at	O-linked N-acetylglucosamine (GlcNAc) transferase (UDP-N-acety	Ogt	0.82787	1.08318414	0.10609511
1455756_at	PDX1 C-terminal inhibiting factor 1	Pcif1	0.8279	1.08209142	0.11061768
1438348_x_at	cysteine conjugate-beta lyase 1	Ccbl1	0.8279	1.11020229	0.13755884
1437634_at	THO complex 2	Thoc2	0.82791	-1.0656587	-0.096173
1437455_a_at	B-cell translocation gene 1, anti-proliferative	Btg1	0.82792	-1.08094089	-0.1397357
1416014_at	ATP-binding cassette, sub-family E (OABP), member 1	Abce1	0.82792	-1.06064342	-0.087332
1448472_at	valyl-tRNA synthetase	Vars	0.82792	1.06702325	0.08962493
1429846_at	RIKEN cDNA 9030411K21 gene	9030411K21Rik	0.82793	-1.06994908	-0.0992593
1460300_a_at	leukocyte tyrosine kinase	Ltk	0.82794	1.10736898	0.13728289
1420956_at	adenomatosis polyposis coli	Apc	0.82795	-1.06823924	-0.1024028
1457997_at	membrane bound O-acyltransferase domain containing 7	Mboat7	0.82796	-1.0848567	-0.1255344
1457086_at	RIKEN cDNA D930028M14 gene	D930028M14Rik	0.82796	1.0952175	0.1234709
1460031_at	G protein-coupled receptor 45	Gpr45	0.82797	-1.08343037	-0.1159544
1443780_at	Polo-like kinase 2 (Drosophila)	Plk2	0.82799	-1.08808478	-0.1309464
1454026_a_at	regulator of G-protein signaling 3	Rgs3	0.828	1.08462203	0.11598097
1450589_at	olfactory receptor 749	Olfr749	0.82802	-1.08398133	-0.1220097
1419458_at	Rho-guanine nucleotide exchange factor	Rgnef	0.82802	-1.09481567	-0.1529491
1459572_at	expressed sequence C86371	C86371	0.82802	1.06358918	0.08625431
1426609_at	DIS3 mitotic control homolog (S. cerevisiae)	Dis3	0.82803	-1.05976841	-0.0871381
1419664_at	serine racemase	Srr	0.82803	-1.0881855	-0.1271758
1422960_at	steroid 5 alpha-reductase 2	Srd5a2	0.82804	-1.08467686	-0.1174588
1433174_a_at	RIKEN cDNA 5430440L12 gene	5430440L12Rik	0.82806	-1.0704055	-0.1028208

1430414_at	RIKEN cDNA 5730406G12 gene	5730406G12Rik	0.82807	1.09850856	0.12930698
1430275_a_at	aquarius	Aqr	0.82808	-1.07920974	-0.1143227
1421425_a_at	regulator of calcineurin 2	Rcan2	0.8281	1.08742234	0.11897656
1453234_at	RIKEN cDNA 1300002K09 gene	1300002K09Rik	0.82814	1.08472092	0.11540106
1457558_at	RIKEN cDNA A330050F15 gene	A330050F15Rik	0.82814	-1.08299995	-0.116544
1423070_at	ribosomal protein L21	Rpl21	0.82815	1.07595465	0.10348361
1441296_at	---	---	0.82815	-1.0860123	-0.1240289
1433498_at	upregulator of cell proliferation	Urgcp	0.82815	1.08536359	0.11143997
1438703_at	ankyrin repeat domain 26	Ankrd26	0.82816	-1.07854286	-0.113314
1453083_at	vomeronasal 2, receptor 29	Vmn2r29	0.82819	1.1059235	0.13587777
1452959_a_at	calpain 10	Capn10	0.82819	1.07425864	0.1017589
1455438_at	peroxisomal membrane protein 4	Pxmp4	0.82822	1.10293272	0.13135751
1418809_at	paired-Ig-like receptor A2	Pira2	0.82823	1.10573311	0.13002926
1441245_a_at	zinc finger protein 512B	Znf512b	0.82823	1.08319843	0.11316469
1440447_at	abhydrolase domain containing 2	Abhd2	0.82823	-1.096004	-0.1535139
1447918_x_at	Immunoglobulin lambda chain, variable 1	Igl-V1	0.82826	1.08324339	0.11406465
1429608_at	alcohol dehydrogenase 6A (class V)	Adh6a	0.82826	1.09065287	0.12213859
1426323_x_at	SIVA1, apoptosis-inducing factor	Siva1	0.82827	1.07984269	0.1008737
1426665_at	katanin p80 (WD40-containing) subunit B 1	Katnb1	0.82827	1.06502899	0.08982673
1445150_at	predicted gene 10399	Gm10399	0.82829	1.1371148	0.16151542
1448859_at	chemokine (C-X-C motif) ligand 13	Cxcl13	0.82829	-1.08583664	-0.1272968
1444101_at	outer dense fiber of sperm tails 2	Odf2	0.8283	-1.0914893	-0.1384734
1456712_at	ligand dependent nuclear receptor corepressor-like	Lcorl	0.8283	-1.08629278	-0.125748
1436542_at	---	---	0.82831	-1.07523117	-0.1063617
1428466_at	chromodomain helicase DNA binding protein 3	Chd3	0.82831	-1.07275266	-0.1043838
1439529_at	RIKEN cDNA A430110N23 gene	A430110N23Rik	0.82831	-1.0617536	-0.087827
1429546_at	RIKEN cDNA 4930423K06 gene /// hypothetical LOC100502740	4930423K06Rik /// LOC10	0.82831	1.12392603	0.14942733
1429469_at	RIKEN cDNA A930013F10 gene	A930013F10Rik	0.82831	1.10502919	0.13283451
1439161_at	protein phosphatase 6, regulatory subunit 3	Ppp6r3	0.82832	1.0965141	0.12065968
AFFX-r2-P1-cre-5	---	---	0.82832	1.0521708	0.0669778
1420099_at	DNA segment, Chr 13, ERATO Doi 787, expressed	D13Ertd787e	0.82833	-1.09216335	-0.1471885
1439655_at	---	---	0.82834	-1.08813275	-0.130928
1437466_at	activated leukocyte cell adhesion molecule	Alcam	0.82834	1.07965804	0.11018562
1452763_at	non imprinted in Prader-Willi/Angelman syndrome 1 homolog (hu	Nipa1	0.82836	-1.10119511	-0.1790574
1417524_at	cornichon homolog 2 (Drosophila)	Cnih2	0.82836	-1.07936345	-0.1143428
1419875_at	---	---	0.82836	1.07171478	0.09558465
1442426_at	wings apart-like homolog (Drosophila)	Wapal	0.82837	1.10725521	0.1353819
1458776_at	---	---	0.82838	-1.09695296	-0.1655605
1443281_at	neuron-glia-CAM-related cell adhesion molecule	Nrcam	0.82838	-1.08569037	-0.1271977
1453161_at	mediator complex subunit 13	Med13	0.8284	1.08649861	0.11715233
1419992_x_at	RAB3A interacting protein (rabin3)-like 1	Rab3il1	0.8284	1.08314911	0.10938418
1448835_at	E2F transcription factor 6	E2f6	0.82841	-1.05973506	-0.0838216
1417131_at	cell division cycle 25 homolog A (S. pombe)	Cdc25a	0.82842	-1.06725827	-0.1002541
1437256_at	DCN1, defective in cullin neddylation 1, domain containing 5 (S. ce	Dcun1d5	0.82842	1.08272731	0.11362669
1457693_a_at	enolase 4	Eno4	0.82843	-1.08765378	-0.1310269
1446743_at	LAS1-like (S. cerevisiae)	Las1l	0.82843	-1.09231638	-0.1466289
1452009_at	tetratricopeptide repeat domain 39B	Ttc39b	0.82844	-1.08180286	-0.1313475
1423179_at	potassium voltage gated channel, Shab-related subfamily, memb	Kcnb1	0.82845	1.09864522	0.12509403
1438601_at	protein kinase, membrane associated tyrosine/threonine 1	Pkmyt1	0.82846	1.06540171	0.09027757
1444012_at	Yip1 domain family, member 3	Yipf3	0.82846	1.09643656	0.1269162
1436310_at	gem (nuclear organelle) associated protein 5	Gemin5	0.82846	1.05859898	0.08131732
1437794_at	calcineurin binding protein 1	Cabin1	0.82848	-1.08405837	-0.119624
1457203_at	---	---	0.82849	-1.08854969	-0.1406889
1442057_at	expressed sequence AA986695	AA986695	0.82851	1.09433577	0.12408685
1447676_x_at	S100 calcium binding protein A16	S100a16	0.82851	1.06926696	0.09591689
1440245_at	coiled-coil domain containing 88B	Ccdc88b	0.82854	1.11991076	0.14549219
1430469_at	tubulin tyrosine ligase-like family, member 5	Ttll5	0.82855	-1.09000951	-0.1354726
1458348_at	THUMP domain containing 2	Thumpd2	0.82857	1.14849762	0.16717743
1421643_at	IKAROS family zinc finger 2	Ikzf2	0.82857	-1.08716991	-0.126805
1434700_at	G2/M-phase specific E3 ubiquitin ligase	G2e3	0.82857	-1.07769385	-0.1172119
1420471_at	hypocretin	Hcrt	0.82859	1.08047249	0.10875839
1418323_at	feminization 1 homolog b (C. elegans)	Fem1b	0.8286	1.06960381	0.09488061
1434190_at	spermine synthase	Sms	0.8286	1.06067791	0.08267231
1437301_a_at	dishevelled, dsh homolog 1 (Drosophila)	Dvl1	0.82862	1.10590782	0.12629528
1430023_at	spermatogenesis associated 24	Spata24	0.82863	1.09304401	0.12147543
1441145_at	PHD finger protein 21A	Phf21a	0.82864	1.162578	0.17335946
1417878_at	E2F transcription factor 1	E2f1	0.82867	1.07826532	0.10379891
1433750_at	solute carrier family 31, member 1	Slc31a1	0.82867	1.07347361	0.09844911
1433181_at	RIKEN cDNA 4930431N21 gene	4930431N21Rik	0.82869	-1.08316539	-0.1176926
1433841_at	RIKEN cDNA C330006K01 gene	C330006K01Rik	0.82869	1.06777895	0.09437836
1441552_at	T-cell receptor alpha chain	Tcra	0.8287	1.08547951	0.11621716
1441238_at	PDS5, regulator of cohesion maintenance, homolog A (S. cerevisia	Pds5a	0.82871	-1.07663774	-0.1092162
1415969_s_at	kidney androgen regulated protein	Kap	0.82872	1.07908844	0.1079841
1440719_at	GLE1 RNA export mediator (yeast)	Gle1	0.82872	-1.09353184	-0.1607342
1427106_at	zinc finger and BTB domain containing 3	Zbtb3	0.82872	1.07802755	0.10788175
1429048_at	biogenesis of lysosome-related organelles complex-1, subunit 2	Bloc1s2	0.82873	-1.05659007	-0.0805037
1430916_at	Fanconi anemia, complementation group D2	Fancd2	0.82874	-1.08693931	-0.1236579
1422522_at	fragile X mental retardation, autosomal homolog 2	Fxr2	0.82889	-1.06832289	-0.1052346
1446417_at	---	---	0.82894	-1.08524286	-0.1214331
1434568_at	---	---	0.82895	1.09182329	0.11736641
1455778_at	zinc finger protein 192	Zfp192	0.82896	-1.07143714	-0.1030858
1431106_a_at	RIKEN cDNA 9530053H05 gene	9530053H05Rik	0.829	-1.08580469	-0.1230502
1417350_at	pallidin	Pldn	0.82902	1.08115102	0.10885704
1426297_at	transcription factor 3	Tcf3	0.82903	1.0579806	0.08106419
1441579_at	doublesex and mab-3 related transcription factor like family A1	Dmrta1	0.82903	1.0696953	0.09593786
1449825_at	actin-related protein T1	Actrt1	0.82911	-1.08029534	-0.1120883
1452858_at	ELAV (embryonic lethal, abnormal vision, Drosophila)-like 1 (Hu a	Elavl1	0.82912	1.10918638	0.13562994
1429280_at	collagen, type XXII, alpha 1	Col22a1	0.82918	1.08473032	0.1166484
1444089_at	spectrin beta 2	Spnb2	0.82925	1.1045765	0.12685491
1455850_at	RIKEN cDNA 2310003H01 gene	2310003H01Rik	0.82929	1.07989415	0.10702147
1437872_at	N-acyl phosphatidylethanolamine phospholipase D	Napepld	0.8293	1.08130331	0.10979853
1453201_at	Ras association (RalGDS/AF-6) domain family (N-terminal) memb	Rassf10	0.82934	-1.07497533	-0.1046009
1432524_at	RIKEN cDNA 4930550G17 gene	4930550G17Rik	0.82936	1.09381415	0.12331601
1442947_x_at	---	---	0.82939	-1.07798396	-0.1101253

1448467_a_at	EH domain binding protein 1-like 1	Ehbp1l1	0.82941	1.09256985	0.12072376
1433331_at	RIKEN cDNA C030046M01 gene	C030046M01Rik	0.82941	1.06818148	0.09364706
1424707_at	transmembrane emp24-like trafficking protein 10 (yeast)	Tmed10	0.82942	-1.0603699	-0.0869791
1418849_x_at	aquaporin 7	Aqp7	0.82944	1.06822659	0.0925067
1442048_at	ring finger protein 11	Rnf11	0.82948	-1.08610784	-0.127437
1420235_at	biphenyl hydrolase-like (serine hydrolase, breast epithelial mucin	Bphl	0.82949	1.10359146	0.12816188
1439887_at	ring finger protein 152	Rnf152	0.82951	1.06664785	0.09156867
1455292_x_at	regulator of sex limited protein 1	Rsl1	0.82953	-1.09650565	-0.1541573
1454838_s_at	protein kinase domain containing, cytoplasmic	Pkdcc	0.82955	-1.10154107	-0.1773004
1437798_at	RIKEN cDNA 6720422M22 gene	6720422M22Rik	0.82958	-1.08637789	-0.1369498
1441513_at	TRAF family member-associated Nf-kappa B activator	Tank	0.82973	-1.08489988	-0.1215554
1433398_at	FYVE, RhoGEF and PH domain containing 3	Fgd3	0.83011	-1.08105112	-0.113444
1425902_a_at	nuclear factor of kappa light polypeptide gene enhancer in B-cells	Nfkb2	0.83011	-1.0762325	-0.1111258
1418979_at	aldo-keto reductase family 1, member C14	Akr1c14	0.83011	1.10077476	0.12501377
1456980_at	RAB44, member RAS oncogene family	Rab44	0.83015	1.09630203	0.12355169
1458694_at	kinesin family member 16B	Kif16b	0.83019	-1.08438718	-0.11766
1439950_at	dynein cytoplasmic 1 heavy chain 1	Dync1h1	0.83022	1.08148595	0.11247037
1440471_x_at	---	---	0.83024	1.05833148	0.08084862
1435187_at	hypothetical LOC100502591	LOC100502591	0.83025	1.09330288	0.11639123
1421589_at	keratin 31	Krt31	0.83026	-1.09003578	-0.1346871
1422042_at	gap junction protein, gamma 3	Gjc3	0.83027	-1.08892981	-0.1277801
1459935_at	EST AA517562	AA517562	0.83027	1.10408415	0.13130753
1449263_at	ubiquitin-fold modifier 1	Ufm1	0.83028	-1.06427749	-0.0932573
1444114_at	---	---	0.83029	1.15127892	0.1670797
1446795_at	---	---	0.8303	-1.08215956	-0.1266415
1438709_at	WD repeat domain, phosphoinositide interacting 1	Wipi1	0.8303	1.12187377	0.14611605
1416510_at	mitochondrial ribosomal protein L4	Mrpl4	0.83031	1.06478016	0.08639982
1418534_at	frizzled homolog 2 (Drosophila)	Fzd2	0.83031	-1.08248397	-0.119164
1425997_a_at	phosphatidylinositol glycan anchor biosynthesis, class N	Pign	0.83031	1.12385707	0.14775382
1432109_at	RIKEN cDNA 4930578I07 gene	4930578I07Rik	0.83031	-1.08224854	-0.1162565
1423826_at	nucleolar complex associated 4 homolog (S. cerevisiae)	Noc4l	0.83032	1.07824907	0.10008188
1440194_at	microRNA mir-1199	Mir1199	0.83032	1.0794792	0.1051938
1439314_at	circadian locomotor output cycles kaput	Clock	0.83032	-1.08843142	-0.1251442
1449431_at	transient receptor potential cation channel, subfamily C, member	Trpc6	0.83032	1.05511634	0.07680642
1433487_at	chloride channel 3	Clcn3	0.83032	1.07262029	0.09721659
1457639_at	ATPase, H+ transporting, lysosomal V1 subunit H	Atp6v1h	0.83032	-1.08211772	-0.1186381
1419781_at	RIKEN cDNA D630004N19 gene	D630004N19Rik	0.83032	-1.08068473	-0.1134334
1423504_at	junction adhesion molecule 3	Jam3	0.83034	-1.10046092	-0.1877371
1457176_at	---	---	0.83036	-1.0821405	-0.1173817
1437754_at	karyopherin alpha 7 (importin alpha 8)	Kpna7	0.83036	1.11356531	0.14047938
1421883_at	ELAV (embryonic lethal, abnormal vision, Drosophila)-like 2 (Hu a	Elavl2	0.83036	1.06537423	0.09095532
1445836_at	---	---	0.83037	-1.09280582	-0.1392118
1424301_at	zinc finger protein 219	Zfp219	0.83037	-1.0559747	-0.0799323
1439257_x_at	ribophorin I	Rpn1	0.83038	1.05752869	0.07992618
1429435_x_at	phosphatidylinositol 3-kinase, catalytic, alpha polypeptide	Pik3ca	0.83038	-1.079904	-0.1118055
1455861_at	erythrocyte protein band 4.1-like 5	Epb4.1l5	0.83039	1.07635853	0.10442285
1421124_at	cyclin-dependent kinase 5, regulatory subunit 1 (p35)	Cdk5r1	0.8304	1.11275438	0.14114935
1424267_at	RIKEN cDNA 1810043G02 gene	1810043G02Rik	0.83042	1.08734483	0.11662419
1416092_a_at	microtubule-associated protein 4	Mtap4	0.83042	-1.07987098	-0.1420022
1449413_at	Mpv17 transgene, kidney disease mutant-like	Mpv17l	0.83042	1.0802182	0.1069372
1428522_at	transcription termination factor, RNA polymerase II	Ttf2	0.83042	1.06234824	0.08633392
1422050_at	NK1 transcription factor related, locus 2 (Drosophila)	Nkx1-2	0.83043	1.11991949	0.14772764
1437080_s_at	proteasome (prosome, macropain) 26S subunit, non-ATPase, 11	Psmd11	0.83043	1.04860413	0.06762484
1445783_at	---	---	0.83043	-1.08031461	-0.1143428
1437892_at	zinc finger with KRAB and SCAN domains 3	Zkscan3	0.83044	1.0893091	0.11893971
1442250_at	---	---	0.83044	-1.08386796	-0.1181962
1446877_at	RIKEN cDNA C230014O12 gene	C230014O12Rik	0.83045	1.07745729	0.10682254
1430730_at	RIKEN cDNA 9430024F10 gene	9430024F10Rik	0.83046	1.10728109	0.13642875
1448258_a_at	signal peptidase complex subunit 1 homolog (S. cerevisiae)	Spcs1	0.83047	1.0593246	0.08131282
1428178_s_at	trafficking protein particle complex 6B	Trappc6b	0.83048	-1.07417764	-0.1180416
1443054_at	potassium inwardly-rectifying channel, subfamily J, member 15	Kcnj15	0.83048	-1.06975658	-0.1005903
1417443_at	family with sequence simliarity 151, member A	Fam151a	0.83048	-1.0845432	-0.1214147
1431159_at	MACRO domain containing 2	Macrod2	0.83048	1.09738248	0.12228446
1416719_a_at	ribosomal protein S10	Rps10	0.83049	1.03163882	0.04413879
1418916_a_at	secreted phosphoprotein 2	Spp2	0.83049	-1.08043561	-0.1121229
1437790_at	echinoderm microtubule associated protein like 5	Eml5	0.83049	-1.09249166	-0.1401808
1460686_at	cyclin N-terminal domain containing 1	Cntd1	0.83049	1.10652421	0.13027145
1449203_at	solute carrier organic anion transporter family, member 1a5	Slco1a5	0.8305	1.08811062	0.12074491
1430567_at	serine peptidase inhibitor, Kazal type 5	Spink5	0.8305	1.07021052	0.09252137
1443195_at	---	---	0.83051	-1.08227859	-0.1151427
1436900_x_at	leptin receptor overlapping transcript	Leprot	0.83051	-1.06282007	-0.0951685
1424653_at	tetraspanin 15	Tspan15	0.83051	1.08091258	0.10728392
1420995_at	plexin A3	Plxna3	0.83051	1.08136689	0.11112408
1434117_at	transcription elongation factor B (SIII), polypeptide 3	Tceb3	0.83051	1.07209341	0.09923645
1453761_at	PHD finger protein 6	Phf6	0.83051	1.1175	0.13997139
1442770_at	zinc finger protein 652	Zfp652	0.83051	-1.08561151	-0.1335088
1432938_at	RIKEN cDNA 5430439C14 gene	5430439C14Rik	0.83052	1.09076433	0.11808385
1440100_at	Coiled-coil domain containing 130	Ccdc130	0.83054	1.08709055	0.11860274
1449776_at	---	---	0.83054	-1.09086634	-0.1367482
1430881_at	RIKEN cDNA 7630403G23 gene	7630403G23Rik	0.83054	1.07776243	0.10438474
1430071_at	brain protein 44	Brp44	0.83054	-1.08777335	-0.1355118
1419994_s_at	anaphase promoting complex subunit 16	Anapc16	0.83055	1.06338337	0.08711585
1432208_at	RIKEN cDNA 4930459C07 gene	4930459C07Rik	0.83056	-1.08788525	-0.1297664
1457953_at	---	---	0.83056	1.08477963	0.11373402
1420936_s_at	cleavage and polyadenylation specific factor 2	Cpsf2	0.83056	1.06817671	0.09271506
1417453_at	cullin 4B	Cul4b	0.83057	-1.05274415	-0.0743934
1435878_at	serine/threonine kinase 38 like	Stk38l	0.83058	1.08357623	0.1096571
1417422_at	glycine N-methyltransferase	Gnmt	0.83059	1.0882209	0.11785084
1438502_x_at	ribosomal protein S17	Rps17	0.83061	-1.077402	-0.1132749
1455740_at	heterogeneous nuclear ribonucleoprotein A1	Hnrnpa1	0.83062	-1.09372499	-0.2415992
1460693_a_at	collagen, type IX, alpha 3	Col9a3	0.83064	-1.08909967	-0.1387652
1447445_at	---	---	0.83065	-1.08431652	-0.123146
1436901_at	Notch gene homolog 4 (Drosophila)	Notch4	0.83066	1.08391186	0.11405287
1421268_at	UDP-glucose ceramide glucosyltransferase	Ugcg	0.83067	1.09232434	0.1157918

1454592_at	RIKEN cDNA 9430012M22 gene	9430012M22Rik	0.83067	1.07971561	0.11058896
1424379_at	carbonic anhydrase 11	Car11	0.83069	-1.09149808	-0.1487431
1455385_at	exocyst complex component 6	Exoc6	0.83071	1.08460986	0.11510771
1450780_s_at	high mobility group AT-hook 2	Hmga2	0.83073	-1.08070177	-0.1122062
1452999_at	survival motor neuron domain containing 1	Smndc1	0.83074	-1.05618796	-0.0792696
1420858_at	protein kinase inhibitor, alpha	Pkia	0.83077	-1.07812561	-0.1105526
1427543_s_at	ubiquitin-activating enzyme E1, Chr Y 1	Ube1y1	0.83078	1.07669291	0.10599586
1432916_at	RIKEN cDNA 5730407I07 gene	5730407I07Rik	0.83082	1.11787499	0.14188544
1451440_at	chondrolectin	Chodl	0.83082	-1.08577836	-0.1267373
1458267_at	zinc finger protein 248	Zfp248	0.83084	1.08525716	0.11424421
1426932_at	DNA segment, Chr 19, Brigham & Women's Genetics 1357 expres	D19Bwg1357e	0.83086	1.06762728	0.09017935
1427357_at	cytidine deaminase	Cda	0.83088	1.08160075	0.11151089
1418239_at	apolipoprotein F	Apof	0.8309	1.11072933	0.13831067
1423425_at	phospholipase B domain containing 2	Plbd2	0.8309	1.07361953	0.10057408
1459259_at	predicted gene 5084	Gm5084	0.8309	-1.08596542	-0.1210141
1451959_a_at	vascular endothelial growth factor A	Vegfa	0.83091	-1.06509	-0.0926475
1458942_at	RIKEN cDNA C230037E05 gene	C230037E05Rik	0.83091	-1.07935333	-0.1104337
1435717_at	solute carrier family 30 (zinc transporter), member 7	Slc30a7	0.83092	1.07826384	0.10493842
1458427_at	BRCA1 interacting protein C-terminal helicase 1	Brip1	0.83093	1.08095947	0.10906121
1456171_at	membrane-associated ring finger (C3HC4) 10	Mar-10	0.83093	1.0980833	0.12739537
1429220_at	WD repeat domain 85	Wdr85	0.83094	-1.06409311	-0.0936246
1425708_at	ring finger protein 17	Rnf17	0.83094	-1.07035293	-0.1043174
1428075_at	predicted pseudogene 3244 /// NADH dehydrogenase (ubiquinor	Gm3244 /// Ndufb4	0.83096	1.05913091	0.08044042
1455419_at	RIKEN cDNA D630045J12 gene	D630045J12Rik	0.83096	1.08804214	0.11767403
1443753_at	Galactosamine (N-acetyl)-6-sulfate sulfatase	Galns	0.83096	-1.08121237	-0.1184185
1420028_s_at	minichromosome maintenance deficient 3 (S. cerevisiae)	Mcm3	0.83096	1.05191735	0.07136354
1446227_at	---	---	0.83096	1.07674999	0.10342663
1419437_at	single-minded homolog 2 (Drosophila)	Sim2	0.83097	-1.08056321	-0.1173677
1420784_at	sodium channel, voltage-gated, type XI, alpha	Scn11a	0.83097	-1.08877988	-0.1325802
1456951_at	myeloblastosis oncogene-like 1	Mybl1	0.83098	-1.09060324	-0.1380398
1428282_at	tubulin-specific chaperone E	Tbce	0.83098	1.07525192	0.09870194
1460136_at	---	---	0.83099	1.08450314	0.11299013
1444443_at	---	---	0.83099	1.08211068	0.11290526
1428084_at	KRR1, small subunit (SSU) processome component, homolog (yea	Krr1	0.831	-1.0541761	-0.0776997
1421122_at	Casitas B-lineage lymphoma-like 1	Cbll1	0.83102	-1.06246455	-0.0907193
1455411_at	---	---	0.83102	1.09798491	0.12656668
1428558_at	O-sialoglycoprotein endopeptidase-like 1	Osgepl1	0.83102	1.07102826	0.09766614
1452570_at	DNA segment, KIST 5	D0Kist5	0.83102	1.0819824	0.11200923
1447426_at	---	---	0.83102	1.11323087	0.13969999
1418039_at	RAB, member of RAS oncogene family-like 3	Rabl3	0.83103	1.06364009	0.08717929
1428102_at	carboxypeptidase B1 (tissue)	Cpb1	0.83104	-1.09538253	-0.1537246
1419053_at	peroxisomal biogenesis factor 14	Pex14	0.83104	1.06189976	0.08499076
1430413_at	transmembrane protein 29	Tmem29	0.83107	-1.07903258	-0.1106169
1449049_at	toll-like receptor 1	Tlr1	0.83107	1.07921623	0.10977624
1426049_a_at	telomeric repeat binding factor 2, interacting protein	Terf2ip	0.83108	1.0751256	0.10324217
1429788_at	actin related protein M1	Arpm1	0.83109	1.08791581	0.11752722
1420530_at	D4, zinc and double PHD fingers family 1	Dpf1	0.83109	1.07011012	0.09461925
1423617_at	peptide deformylase (mitochondrial)	Pdf	0.8311	1.06947542	0.0943734
1440017_at	pleckstrin homology domain containing, family G (with RhoGef do	Plekhg3	0.8311	-1.06359873	-0.0903047
1426086_a_at	fragile X mental retardation syndrome 1 homolog	Fmr1	0.83111	1.07113194	0.09761878
1423253_at	myelin protein zero	Mpz	0.83111	1.08129548	0.11193436
1422575_at	Max dimerization protein 4	Mxd4	0.83112	1.08699817	0.11762838
1438320_s_at	minichromosome maintenance deficient 7 (S. cerevisiae)	Mcm7	0.83112	1.03929313	0.05506866
1452138_a_at	angiotensin I converting enzyme (peptidyl-dipeptidase A) 2	Ace2	0.83112	-1.06384579	-0.090731
1455883_a_at	leucine rich repeat transmembrane neuronal 1	Lrrtm1	0.83112	1.06622125	0.09034392
1427728_at	cholinergic receptor, nicotinic, gamma polypeptide	Chrng	0.83113	-1.0793957	-0.1108591
1424237_at	zinc finger protein 639	Zfp639	0.83113	1.05894743	0.08088717
1444640_at	phosphatidylinositol glycan anchor biosynthesis, class G	Pigg	0.83114	1.08127136	0.10863612
1418936_at	v-maf musculoaponeurotic fibrosarcoma oncogene family, protei	Maff	0.83115	1.07269249	0.09884032
1449300_at	CTTNBP2 N-terminal like	Cttnbp2nl	0.83116	1.08129034	0.10772651
1419888_at	---	---	0.83116	-1.08622353	-0.1224947
1418950_at	dopamine receptor D2	Drd2	0.83117	-1.09526139	-0.1421107
1426738_at	diacylglycerol kinase zeta	Dgkz	0.83118	1.0810752	0.10423059
1447585_s_at	mitochondrial ribosomal protein S6	Mrps6	0.83118	1.06651206	0.0886552
1456445_at	RIKEN cDNA 4930563D23 gene	4930563D23Rik	0.83119	-1.08302926	-0.1195515
1418410_at	jerky	Jrk	0.83119	1.07785767	0.10382182
1444911_at	ATPase, class V, type 10B	Atp10b	0.83119	-1.0797799	-0.1117227
1425944_a_at	RAD51-like 3 (S. cerevisiae)	Rad51l3	0.83119	1.0781719	0.10494203
1422594_at	suppressor of IKBKE 1	Sike1	0.83121	-1.07296448	-0.1035588
1424537_at	predicted gene 10922 /// predicted gene 14345 /// predicted ger	Gm10922 /// Gm14345 //	0.83122	1.07623838	0.1052993
1417072_at	solute carrier family 22 (organic anion transporter), member 6	Slc22a6	0.83123	1.08240684	0.11251716
1435682_at	leucyl-tRNA synthetase, mitochondrial	Lars2	0.83126	-1.0737518	-0.1031759
1438019_at	inositol 1,3,4,5,6-pentakisphosphate 2-kinase	Ippk	0.83126	1.06329333	0.08815928
1455562_at	SRY-box containing gene 12	Sox12	0.83129	1.05575163	0.07804801
1442639_at	---	---	0.83129	-1.09493934	-0.1558428
1423054_at	WD repeat domain 1	Wdr1	0.83129	1.06449932	0.08795679
1443848_at	---	---	0.83129	1.09386996	0.12292647
1420644_a_at	Sec61, alpha subunit 2 (S. cerevisiae)	Sec61a2	0.83131	1.07392203	0.0991001
1430668_a_at	ankyrin repeat, family A (RFXANK-like), 2	Ankra2	0.83131	1.07701956	0.10213679
1418420_at	myogenic differentiation 1	Myod1	0.83132	1.09234608	0.12211706
1430987_s_at	WW domain binding protein 11	Wbp11	0.83133	-1.06777057	-0.1009516
1453623_a_at	RAD23a homolog (S. cerevisiae)	Rad23a	0.83135	1.1371739	0.15126679
1421375_a_at	S100 calcium binding protein A6 (calcyclin)	S100a6	0.83141	-1.07125278	-0.1065668
1433292_at	succinate dehydrogenase complex, subunit A, flavoprotein (Fp)	Sdha	0.83143	-1.07905278	-0.114324
1423566_a_at	heat shock 105kDa/110kDa protein 1	Hsph1	0.83144	1.05395813	0.07559338
1441265_at	---	---	0.83144	-1.08561266	-0.1209344
1440474_at	carnitine O-octanoyltransferase	Crot	0.83145	-1.08043513	-0.1131054
1452655_at	zinc finger, DHHC domain containing 2	Zdhhc2	0.83146	-1.07755811	-0.1078722
1423270_at	neural precursor cell expressed, developmentally down-regulated	Nedd4l	0.83148	-1.08312105	-0.1185882
1455772_at	progesterone receptor	Pgr	0.83148	-1.08004991	-0.1111862
1458187_at	zinc finger protein 142	Zfp142	0.83149	1.08915594	0.12178963
1421352_at	toll-like receptor 6	Tlr6	0.83149	-1.08479843	-0.1217661
1455695_at	ST8 alpha-N-acetyl-neuraminide alpha-2,8-sialyltransferase 1	St8sia1	0.83151	-1.07830243	-0.1138513
1444786_at	nucleolar protein 3 (apoptosis repressor with CARD domain)	Nol3	0.83152	-1.07617381	-0.1070206

1459710_at	---	---	0.83152	1.11067047	0.13767279
1417441_at	DnaJ (Hsp40) homolog, subfamily C, member 12	Dnajc12	0.83152	1.08169321	0.10648004
1426158_at	T-cell receptor beta, joining region	Tcrb-J	0.83152	1.080637	0.11155072
1460690_at	family with sequence similarity 195, member B	Fam195b	0.83156	1.07621416	0.10028891
1448780_at	solute carrier family 12, member 2	Slc12a2	0.83165	-1.08876454	-0.1487726
1417473_a_at	phosphopantothenoylcysteine synthetase	Ppcs	0.83166	1.06895633	0.09307672
1437955_at	ubiquilin-like	Ubqlnl	0.83168	1.09675122	0.12532047
1455048_at	immunoglobulin superfamily, member 3	Igsf3	0.83168	1.10951442	0.13151676
1437676_at	sperm associated antigen 9	Spag9	0.83169	-1.07025232	-0.0981949
1459418_at	---	---	0.8317	-1.06703531	-0.0971279
1435538_at	RIKEN cDNA 0610030E20 gene	0610030E20Rik	0.83171	1.07687305	0.10620994
1438361_at	RIKEN cDNA 2310035C23 gene	2310035C23Rik	0.83171	1.08705911	0.11815534
1458725_at	---	---	0.83172	1.10651853	0.13448619
1423720_a_at	SAR1 gene homolog A (S. cerevisiae)	Sar1a	0.83172	1.06421818	0.0871536
1427849_a_at	similar to T-cell receptor beta-2 chain C region	LOC665506	0.83173	-1.09275137	-0.1400572
1439598_at	---	---	0.83174	1.08615026	0.11533946
1441318_at	lysine (K)-specific demethylase 5A	Kdm5a	0.83174	-1.08437668	-0.1185442
1428272_at	eukaryotic translation initiation factor 1B	Eif1b	0.83174	1.06001576	0.083437
1450427_at	cholinergic receptor, nicotinic, alpha polypeptide 6	Chrna6	0.83175	-1.08352392	-0.1188675
1429637_at	family with sequence similarity 198, member B	Fam198b	0.83175	-1.09244804	-0.1650439
1450212_at	formin-like 1	Fmnl1	0.83176	-1.08017958	-0.1197725
1449172_a_at	lin-7 homolog B (C. elegans)	Lin7b	0.83176	-1.09391889	-0.1525439
1418210_at	profilin 2	Pfn2	0.83176	1.06735756	0.09239323
1439988_at	mitochondrial ribosomal protein L15	Mrpl15	0.83177	-1.07514386	-0.1118879
1421568_at	potassium voltage-gated channel, shaker-related, subfamily, mer	Kcna6	0.83177	-1.0950024	-0.1491987
1453388_at	RIKEN cDNA 4933416M07 gene	4933416M07Rik	0.83178	1.09076285	0.11988085
1433660_at	tubulin, gamma complex associated protein 4	Tubgcp4	0.83179	1.06522721	0.08809387
1416291_at	proteasome (prosome, macropain) 26S subunit, ATPase, 4	Psmc4	0.83179	1.06134882	0.08349823
1453560_at	feminization 1 homolog a (C. elegans)	Fem1a	0.8318	1.09830112	0.12737761
1421614_at	zonadhesin	Zan	0.8318	1.08224837	0.11379739
1432154_at	RIKEN cDNA 4933407I08 gene	4933407I08Rik	0.8318	-1.08809483	-0.1310596
1429966_at	RIKEN cDNA 3110057O12 gene	3110057O12Rik	0.8318	1.09408085	0.12439793
1423417_at	SWI/SNF related, matrix associated, actin dependent regulator of	Smarcc1	0.8318	-1.07185858	-0.1133118
1448915_at	zinc finger protein 524	Zfp524	0.83181	-1.07858664	-0.1169864
1443394_at	---	---	0.83181	1.11671604	0.14143637
1441379_at	expressed sequence AU045094	AU045094	0.83181	-1.06110149	-0.0886595
1451260_at	aldehyde dehydrogenase 1 family, member B1	Aldh1b1	0.83182	-1.0871286	-0.1370581
1434649_at	cerebral cavernous malformation 2 homolog (human)	Ccm2	0.83182	1.06327271	0.08846318
1440451_at	predicted gene 4368	Gm4368	0.83183	1.10162163	0.12888587
1415703_at	HECT, UBA and WWE domain containing 1	Huwe1	0.83184	-1.0618343	-0.0923657
1429568_x_at	ubiquitin-conjugating enzyme E2F (putative)	Ube2f	0.83185	1.06028529	0.0821422
1422605_at	protein phosphatase 1, regulatory (inhibitor) subunit 1A	Ppp1r1a	0.83186	-1.06632572	-0.099388
1456872_a_at	harbinger transposase derived 1	Harbi1	0.83186	-1.06180424	-0.0879791
1458407_s_at	---	---	0.83187	-1.07974473	-0.1206604
1444279_at	HECT, UBA and WWE domain containing 1	Huwe1	0.83188	-1.08715153	-0.1318726
1416437_a_at	mitogen-activated protein kinase 8 interacting protein 3	Mapk8ip3	0.83188	-1.07415656	-0.1047684
1415778_at	mortality factor 4 like 2	Morf4l2	0.83189	1.04590444	0.06374068
1444127_at	Ankyrin repeat and SOCS box-containing 1	Asb1	0.8319	-1.07883827	-0.1128332
1434211_at	SH3 domain binding glutamic acid-rich protein like 2	Sh3bgrl2	0.8319	1.08447771	0.11240875
1435861_at	protein tyrosine phosphatase, receptor type, f polypeptide (PTPRF	Ppfia1	0.83192	1.06792704	0.09298976
1452357_at	glycoprotein Ib, beta polypeptide /// septin 5	Gp1bb /// Sept5	0.83192	-1.08262298	-0.1152693
1425805_a_at	ubiquitin specific peptidase 12	Usp12	0.83193	-1.07427967	-0.1082179
1450507_at	POU domain, class 2, transcription factor 3	Pou2f3	0.83193	1.0927574	0.11869675
1448124_at	glucuronidase, beta	Gusb	0.83193	1.09026904	0.11357046
1444481_at	---	---	0.83193	1.07567426	0.10367194
1439093_at	heat shock protein 4 like	Hspa4l	0.83194	-1.07968817	-0.1165899
1427687_at	protocadherin alpha 10	Pcdha10	0.83194	-1.09160326	-0.1402763
1451302_at	RIKEN cDNA 1110012L19 gene	1110012L19Rik	0.83195	1.09004552	0.11118592
1419144_at	CD163 antigen	Cd163	0.83196	1.07918559	0.10304064
1450953_at	cytosolic iron-sulfur protein assembly 1 homolog (S. cerevisiae)	Ciao1	0.83196	1.05680774	0.07911256
1431589_at	hydrocephalus inducing	Hydin	0.83196	-1.08522359	-0.1221928
1425927_a_at	activating transcription factor 5	Atf5	0.83196	-1.07229364	-0.1107222
1427291_at	synaptonemal complex protein 1	Sycp1	0.83196	-1.07768992	-0.1082846
1453238_s_at	RIKEN cDNA 3930401B19 gene /// RIKEN cDNA A130040M12 ge	3930401B19Rik /// A130	0.83197	-1.0748181	-0.1057904
1429718_at	SLIT and NTRK-like family, member 5	Slitrk5	0.83198	-1.08853975	-0.1285385
1427563_at	distal-less homeobox 6, antisense	Dlx6as	0.83198	-1.08282433	-0.1200267
1417011_at	syndecan 2	Sdc2	0.83198	1.09073089	0.12146607
1460489_at	RIKEN cDNA 1700097M23 gene	1700097M23Rik	0.83198	1.06850188	0.0944638
1460572_a_at	zinc finger protein 511	Zfp511	0.83198	-1.08086713	-0.1274194
1445170_at	RIKEN cDNA 2700012I20 gene	2700012I20Rik	0.83199	1.09943812	0.12895956
1456953_at	collagen, type XIX, alpha 1	Col19a1	0.83199	1.09609277	0.12379644
1437109_s_at	LSM6 homolog, U6 small nuclear RNA associated (S. cerevisiae)	Lsm6	0.83199	1.06012915	0.0822919
1430598_at	cAMP responsive element modulator	Crem	0.83199	1.100512	0.12796719
1428732_at	RIKEN cDNA 1700008J07 gene	1700008J07Rik	0.832	1.07738897	0.10433583
1432537_at	RIKEN cDNA 4930465A12 gene	4930465A12Rik	0.832	1.09210546	0.12173693
1434911_s_at	Rho GTPase activating protein 19	Arhgap19	0.832	1.06712683	0.091216
1424235_at	ORM1-like 2 (S. cerevisiae)	Ormdl2	0.832	1.05815462	0.08116058
1419098_at	stomatin	Stom	0.83201	1.15839319	0.17423554
1437876_at	interleukin 20 receptor beta	Il20rb	0.83201	-1.08704208	-0.1307401
1435664_at	zinc finger protein 397	Zfp397	0.83201	-1.07455607	-0.1120855
1454559_at	SH3-binding domain glutamic acid-rich protein	Sh3bgr	0.83202	-1.08760617	-0.1291024
1446057_at	rearranged L-myc fusion sequence	Rlf	0.83202	-1.07679018	-0.1100864
1436133_at	coiled-coil domain containing 127	Ccdc127	0.83202	-1.08386715	-0.1237941
1458902_at	---	---	0.83202	-1.07409285	-0.1046283
1446330_at	---	---	0.83203	-1.08585132	-0.1427271
1433824_x_at	G-rich RNA sequence binding factor 1	Grsf1	0.83203	-1.0583691	-0.087013
1430116_at	cytochrome P450, family 4, subfamily f, polypeptide 41 pseudoge	Cyp4f41-ps	0.83204	-1.08373144	-0.1168304
1431297_a_at	RIKEN cDNA 4933436C20 gene	4933436C20Rik	0.83204	-1.08913681	-0.1348188
1428996_at	pyruvate dehyrogenase phosphatase catalytic subunit 2	Pdp2	0.83204	-1.07586283	-0.1071849
1418082_at	N-myristoyltransferase 1	Nmt1	0.83204	1.07868028	0.10481262
1442694_at	---	---	0.83204	-1.0820867	-0.1172118
1458461_at	ubiquitin-conjugating enzyme E2Q (putative) 2 pseudogene /// u	E330021D16Rik /// LOC10	0.83204	-1.08092219	-0.1175658
1422255_at	potassium voltage-gated channel, shaker-related subfamily, mem	Kcna4	0.83205	1.08341644	0.11494086
1448252_a_at	eukaryotic translation elongation factor 1 beta 2	Eef1b2	0.83205	-1.02933482	-0.0418191

1436653_at	Tctex1 domain containing 4	Tctex1d4	0.83205	-1.08140196	-0.1131089
1450890_a_at	abl-interactor 1	Abi1	0.83205	-1.05826312	-0.0840502
1423094_at	cereblon	Crbn	0.83205	1.06278661	0.08550846
1454659_at	dCMP deaminase	Dctd	0.83206	1.06353813	0.08801129
1450813_a_at	troponin I, skeletal, slow 1	Tnni1	0.83206	1.08014595	0.1112012
1438091_a_at	H2A histone family, member Z /// hypothetical LOC100504949	H2afz /// LOC100504949	0.83206	-1.05312413	-0.0817476
1428900_s_at	methyltransferase 5 domain containing 1	Mett5d1	0.83206	-1.07292058	-0.1089882
1455329_at	---	---	0.83206	1.09943813	0.12596408
1428827_at	Wolf-Hirschhorn syndrome candidate 1 (human)	Whsc1	0.83207	1.0742116	0.10070635
1433145_at	RIKEN cDNA 4933431C10 gene	4933431C10Rik	0.83207	-1.08422795	-0.1180926
1447068_at	---	---	0.83207	-1.08580338	-0.1270847
1458214_at	zinc finger protein 786	Zfp786	0.83207	1.15000196	0.16691911
1416899_at	undifferentiated embryonic cell transcription factor 1	Utf1	0.83207	1.05078758	0.06972448
1419178_at	CD3 antigen, gamma polypeptide	Cd3g	0.83208	-1.0820919	-0.1207056
1458559_at	RIKEN cDNA A730028G07 gene	A730028G07Rik	0.83208	1.13302353	0.15168497
1439747_at	prostaglandin E synthase	Ptges	0.83209	1.14935339	0.16476187
1455386_at	---	---	0.8321	1.08826475	0.11903297
1442448_at	RIKEN cDNA 1810064F22 gene	1810064F22Rik	0.8321	1.0808071	0.10637277
1417979_at	tenomodulin	Tnmd	0.83211	1.10698715	0.13034786
1419657_a_at	solute carrier family 25, member 36	Slc25a36	0.83211	-1.05590492	-0.0797352
1430133_at	TBC1 domain family, member 8B	Tbc1d8b	0.83211	1.14260819	0.16480792
1444292_at	hypothetical LOC100303645	LOC100303645	0.83211	-1.06640169	-0.1000138
1459277_at	---	---	0.83211	1.07727421	0.10722791
1431524_at	RIKEN cDNA 4933427I22 gene	4933427I22Rik	0.83212	-1.0854039	-0.1262192
1456437_x_at	complement component 1, r subcomponent A /// complement co	C1ra /// C1rb	0.83212	-1.07092158	-0.1055283
1421673_s_at	syntaxin 1B	Stx1b	0.83212	-1.07726944	-0.1087451
1456467_s_at	nemo like kinase	Nlk	0.83212	-1.08526631	-0.141489
1426076_at	cell division cycle 27 homolog (S. cerevisiae)	Cdc27	0.83212	-1.07472534	-0.111232
1430627_at	p21 protein (Cdc42/Rac)-activated kinase 2	Pak2	0.83212	-1.08693394	-0.1237491
1439272_at	ligand dependent nuclear receptor corepressor-like	Lcorl	0.83213	-1.08475587	-0.1201755
1450372_a_at	ribosomal protein L18	Rpl18	0.83214	1.03577255	0.04944156
1417324_at	microtubule associated serine/threonine kinase 2	Mast2	0.83214	1.06739075	0.09165251
1425689_at	dihydropyrimidinase	Dpys	0.83215	-1.0814515	-0.1165256
1441570_at	---	---	0.83216	1.08232338	0.1116409
1428412_at	transmembrane 9 superfamily member 3	Tm9sf3	0.83216	1.06588146	0.09187087
1420025_s_at	AP2 associated kinase 1	Aak1	0.83216	-1.08292953	-0.118698
1428174_x_at	KH-type splicing regulatory protein	Khsrp	0.83217	-1.07853236	-0.1419428
1430443_at	annexin A10	Anxa10	0.83217	-1.08500085	-0.1353501
1452491_at	Predicted gene 11767	Gm11767	0.83217	-1.09219252	-0.1556249
1426322_a_at	potassium large conductance calcium-activated channel, subfami	Kcnmb2	0.83217	-1.08207451	-0.1158837
1416891_at	numb gene homolog (Drosophila)	Numb	0.83218	1.05960754	0.08350244
1436875_at	dynamin 3	Dnm3	0.83218	1.08579872	0.11025052
1442356_at	MAX gene associated	Mga	0.83219	-1.09339693	-0.1501827
1448031_at	guanine nucleotide binding protein, alpha O	Gnao1	0.83219	-1.07822663	-0.1088752
1424595_at	F11 receptor	F11r	0.8322	1.05826647	0.08059432
1437707_at	slowmo homolog 1 (Drosophila)	Slmo1	0.83221	-1.08912521	-0.14627
1439538_at	coiled-coil domain containing 127	Ccdc127	0.83221	1.09004478	0.12069273
1452558_at	---	---	0.83222	-1.06052161	-0.0853142
1452108_at	insulin-like growth factor I receptor	Igf1r	0.83222	-1.08590682	-0.1229678
1418439_at	mitochondrial ribosomal protein L42	Mrpl42	0.83222	1.05341063	0.07388065
1457654_at	O-sialoglycoprotein endopeptidase	Osgep	0.83222	-1.08210512	-0.1253431
1430747_at	PQ loop repeat containing 1	Pqlc1	0.83223	1.09915153	0.12891359
1432863_a_at	NK2 transcription factor related, locus 4 (Drosophila)	Nkx2-4	0.83223	-1.07953647	-0.1105632
1440647_at	signal-induced proliferation-associated 1 like 1	Sipa1l1	0.83223	-1.08107594	-0.1175346
1424192_at	RIKEN cDNA 1500011H22 gene	1500011H22Rik	0.83223	1.05720038	0.07998678
1436509_at	malectin	Mlec	0.83224	1.05862928	0.0809562
1454761_at	cDNA sequence BC005764	BC005764	0.83224	1.08848524	0.11752934
1435372_a_at	proliferation-associated 2G4	Pa2g4	0.83224	1.07124008	0.09102357
1422285_at	orthopedia homolog (Drosophila)	Otp	0.83224	-1.07553565	-0.1063912
1432823_at	synaptophysin-like 2	Sypl2	0.83225	1.10235047	0.1302839
1453680_at	RIKEN cDNA 8030456M14 gene	8030456M14Rik	0.83225	-1.06564558	-0.0953892
1447231_at	---	---	0.83225	1.11217538	0.1373334
1452411_at	leucine rich repeat containing 1	Lrrc1	0.83226	-1.07156025	-0.1002989
1419075_s_at	serum amyloid A 1	Saa1	0.83227	-1.08771116	-0.1336361
1444592_at	O-sialoglycoprotein endopeptidase	Osgep	0.83228	-1.08404834	-0.1266904
1437055_x_at	major facilitator superfamily domain containing 1	Mfsd1	0.83228	-1.07587831	-0.1130957
1455627_at	collagen, type VIII, alpha 1	Col8a1	0.83228	1.06155731	0.0855638
1435651_a_at	SWI/SNF-related, matrix-associated actin-dependent regulator of	Smarcad1	0.83229	-1.06726054	-0.1068729
1416390_at	regulator of chromosome condensation (RCC1) and BTB (POZ) do	Rcbtb2	0.83229	1.08338918	0.11429473
1431593_a_at	tRNA splicing endonuclease 34 homolog (S. cerevisiae)	Tsen34	0.8323	1.05496971	0.07676126
1416327_at	ubiquitin-fold modifier conjugating enzyme 1	Ufc1	0.83231	1.06465392	0.08693235
1443081_at	GATA binding protein 6	Gata6	0.83231	-1.09397913	-0.1506926
1416602_a_at	RAD52 homolog (S. cerevisiae)	Rad52	0.83231	1.08371083	0.11090102
1432875_at	RIKEN cDNA 4632409D06 gene	4632409D06Rik	0.83231	1.08809014	0.11406135
1436592_at	Zinc finger protein 799	Zfp799	0.83232	1.09651672	0.12467606
1447786_at	cytohesin 1	Cyth1	0.83232	1.10949694	0.13483688
1438823_at	---	---	0.83232	1.0797694	0.11045007
1419914_s_at	nuclear undecaprenyl pyrophosphate synthase 1 homolog (S. cer	Nus1	0.83232	-1.06212042	-0.0929861
1430057_s_at	leucine rich repeat containing 57	Lrrc57	0.83234	1.07686512	0.10353594
1418233_a_at	trafficking protein particle complex 5	Trappc5	0.83235	1.09827543	0.11892508
1440287_at	---	---	0.83235	-1.07896592	-0.1121714
1457819_at	sodium channel, voltage-gated, type IX, alpha	Scn9a	0.83236	-1.08618995	-0.1241289
1455971_at	stress-associated endoplasmic reticulum protein family member 2	Serp2	0.83236	1.11306165	0.13521328
1443805_at	dapper homolog 3, antagonist of beta-catenin (xenopus)	Dact3	0.83236	1.09328432	0.12334336
1443959_at	transmembrane protein 72	Tmem72	0.83236	1.08490558	0.11485331
1442108_at	---	---	0.83237	1.08113574	0.11135053
1456339_at	RIKEN cDNA 2810410D24 gene	2810410D24Rik	0.83238	1.09642929	0.1227711
1448534_at	signal-regulatory protein alpha	Sirpa	0.83238	-1.08219851	-0.1263125
1416257_at	calpain 2	Capn2	0.83238	1.07023553	0.09517493
1443824_s_at	carbonic anhydrase 7	Car7	0.83239	-1.09459839	-0.1522561
1444029_at	poly (ADP-ribose) polymerase family, member 11	Parp11	0.83239	1.08346233	0.11525052
1450050_at	histone cell cycle regulation defective homolog A (S. cerevisiae)	Hira	0.83239	1.12324707	0.14158515
1436640_x_at	1-acylglycerol-3-phosphate O-acyltransferase 4 (lysophosphatidi	Agpat4	0.83239	1.06097449	0.08344058
1420566_at	---	---	0.83241	1.11048731	0.13665466

1423630_at	cytoglobin	Cygb	0.83241	1.12821923	0.15092277
1423223_a_at	peroxiredoxin 6	Prdx6	0.83243	1.05498268	0.07592109
1423645_a_at	DEAD (Asp-Glu-Ala-Asp) box polypeptide 5	Ddx5	0.83243	-1.05269945	-0.0755339
1419490_at	family with sequence similarity 19, member A5	Fam19a5	0.83244	1.10026491	0.12832547
1436732_s_at	F-box and WD-40 domain protein 8	Fbxw8	0.83244	1.05867707	0.08086859
1432769_at	solute carrier family 44, member 1	Slc44a1	0.83244	-1.0808382	-0.1275087
1421135_a_at	CCR4-NOT transcription complex, subunit 8	Cnot8	0.83244	1.05772845	0.08087239
1435678_at	RIKEN cDNA 2610017I09 gene	2610017I09Rik	0.83245	-1.08435071	-0.1199764
1450085_at	angiopoietin-like 2	Angptl2	0.83245	1.12966436	0.15184331
1460509_at	UDP-Gal:betaGlcNAc beta 1,3-galactosyltransferase, polypeptide	B3galt1	0.83245	1.08588778	0.10724809
1444672_at	---	---	0.83246	-1.07064867	-0.100551
1440454_at	pigeon homolog (Drosophila)	Pion	0.83246	-1.09044536	-0.1502334
1452474_a_at	ADP-ribosyltransferase 3	Art3	0.83248	1.08362251	0.10898922
1440645_at	---	---	0.83248	1.07373435	0.10061215
1456350_at	zinc finger protein 451	Zfp451	0.83249	1.10519415	0.13291585
1432919_at	RIKEN cDNA 4921511E18 gene	4921511E18Rik	0.8325	-1.06620449	-0.093765
1428473_at	protein phosphatase 3, catalytic subunit, beta isoform	Ppp3cb	0.8325	-1.05943021	-0.0840722
1422133_at	sialophorin	Spn	0.8325	-1.07282006	-0.1056024
1445514_at	---	---	0.83253	1.11826307	0.14155203
1420577_at	activation-induced cytidine deaminase	Aicda	0.83253	-1.08255082	-0.1205604
1426965_at	RAS related protein 2a	Rap2a	0.83253	-1.07494778	-0.1058605
1452532_x_at	carcinoembryonic antigen-related cell adhesion molecule 1	Ceacam1	0.83254	1.16718526	0.17555371
1444258_at	---	---	0.83254	-1.07278576	-0.1045673
1416705_at	ribulose-5-phosphate-3-epimerase	Rpe	0.83254	-1.05956647	-0.0837691
1419986_at	Coiled-coil domain containing 69	Ccdc69	0.83255	1.11290765	0.14130379
1433931_at	RIKEN cDNA C030046I01 gene	C030046I01Rik	0.83255	1.06970371	0.09432171
1430086_at	cholinergic receptor, nicotinic, alpha polypeptide 9	Chrna9	0.83255	-1.0704077	-0.102139
1445827_at	Zinc finger, MYND-type containing 8	Zmynd8	0.83257	1.08958496	0.11942815
1450874_at	matrin 3	Matr3	0.83258	-1.05708335	-0.0841608
1430618_at	RIKEN cDNA 2610020C07 gene	2610020C07Rik	0.83258	1.11833923	0.14288099
1459013_at	hypothetical LOC100504636	LOC100504636	0.83259	1.08903494	0.11620754
1415827_a_at	stress-associated endoplasmic reticulum protein 1	Serp1	0.83259	-1.06438915	-0.0972513
1418633_at	Notch gene homolog 1 (Drosophila)	Notch1	0.83259	-1.07192516	-0.1042637
1424931_s_at	immunoglobulin lambda chain, constant region 1 /// immunoglob	Igl-C1 /// Igl-V1 /// LOC43	0.83259	1.0806989	0.11018068
1457895_at	kinesin family member 18A	Kif18a	0.8326	1.07188261	0.09660328
1436501_at	mitochondrial tumor suppressor 1	Mtus1	0.8326	1.08781707	0.11319553
1455199_at	expressed sequence AI429214	AI429214	0.83262	-1.08820691	-0.1336278
1452828_at	F-box protein 21	Fbxo21	0.83264	1.06506495	0.08820573
1415795_at	spindlin 1	Spin1	0.83264	1.06356252	0.08814081
1432957_at	RIKEN cDNA 5730407O05 gene	5730407O05Rik	0.83266	1.09305105	0.11564586
1460522_at	RIKEN cDNA 6030458E02 gene	6030458E02Rik	0.83267	1.12518195	0.14741751
1450855_at	armadillo repeat gene deleted in velo-cardio-facial syndrome	Arvcf	0.83269	1.07712493	0.1043115
1443213_at	glycosyltransferase-like domain containing 1	Gtdc1	0.83269	1.094894	0.12371619
1442177_at	solute carrier family 6 (neurotransmitter transporter, glycine), me	Slc6a5	0.83271	-1.09628809	-0.1588573
1417687_at	lectin, galactose binding, soluble 12	Lgals12	0.83272	-1.08438589	-0.1237883
1428370_at	RIKEN cDNA 1500011B03 gene	1500011B03Rik	0.83272	1.06286992	0.08780725
1452590_a_at	predicted gene 10393 /// placenta specific 9	Gm10393 /// Plac9	0.83273	1.08449002	0.10873374
1436618_at	sideroflexin 5	Sfxn5	0.83273	1.11643872	0.14052212
1451370_at	coiled-coil domain containing 107	Ccdc107	0.83273	1.09988951	0.11997176
1438796_at	nuclear receptor subfamily 4, group A, member 3	Nr4a3	0.83274	-1.08535516	-0.1232457
1433896_at	transmembrane protein 127	Tmem127	0.83275	-1.0798132	-0.1196246
1448072_at	RIKEN cDNA 2010010I01 gene	2010010I01Rik	0.83279	-1.07770538	-0.1082844
1439535_at	---	---	0.83281	1.07504809	0.10384638
1430633_s_at	RIKEN cDNA C430045I18 gene	C430045I18Rik	0.83284	1.1376473	0.15367923
1458705_at	RIKEN cDNA 1700031C06 gene	1700031C06Rik	0.83295	-1.06826658	-0.0987137
1448904_at	family with sequence similarity 3, member C	Fam3c	0.83304	1.06277451	0.08610667
1457169_at	---	---	0.83305	-1.07317674	-0.1021077
1460648_at	nuclear receptor subfamily 2, group F, member 6	Nr2f6	0.83306	1.06085778	0.0843048
1439918_at	outer dense fiber of sperm tails 2	Odf2	0.83306	1.0867288	0.11455021
1438950_x_at	resistance to inhibitors of cholinesterase 8 homolog (C. elegans)	Ric8	0.83307	-1.07140046	-0.1042827
1429596_at	solute carrier family 7, member 6 opposite strand	Slc7a6os	0.83308	1.09775217	0.11870285
1420399_at	growth factor independent 1B	Gfi1b	0.83308	-1.07458425	-0.1061807
1419880_x_at	---	---	0.83308	-1.06712182	-0.0955049
1438643_at	Calcium/calmodulin-dependent protein kinase ID	Camk1d	0.8331	-1.09122882	-0.1557208
1459370_at	---	---	0.8331	1.09945468	0.12596132
1446760_at	Thyroid hormone receptor interactor 13	Trip13	0.83312	-1.07831817	-0.1101194
1436160_at	keratin 26	Krt26	0.83312	1.08271775	0.11434722
1436533_at	TROVE domain family, member 2	Trove2	0.83313	1.11281528	0.13440547
1454873_at	zinc finger protein 775	Zfp775	0.83314	1.10202944	0.12467361
1415933_a_at	cytochrome c oxidase, subunit Va	Cox5a	0.83314	-1.0453293	-0.0694986
1433792_at	nuclear receptor interacting protein 2	Nrip2	0.83315	1.11376929	0.13621921
1447854_s_at	histone cluster 2, H2be	Hist2h2be	0.83315	1.14998713	0.16559615
1437913_at	B-cell leukemia/lymphoma 2 related protein A1a /// B-cell leuken	Bcl2a1a /// Bcl2a1b /// Bc	0.83315	1.09805435	0.12525768
1425546_a_at	transferrin	Trf	0.83317	1.07513758	0.10156368
1434396_a_at	predicted pseudogene 5526 /// myosin, light polypeptide 6, alkal	Gm5526 /// Myl6	0.83318	-1.04341315	-0.0616568
1421746_a_at	F-box protein 17	Fbxo17	0.83318	1.07200341	0.09781008
1458052_at	---	---	0.8332	1.11269469	0.13801732
1439407_x_at	transgelin 2	Tagln2	0.83321	1.06020143	0.08345907
1460338_a_at	cytokine receptor-like factor 3	Crlf3	0.83322	1.06767956	0.09023525
1426390_a_at	ADP-ribosylation factor 1	Arf1	0.83322	1.03860992	0.05449898
1447723_at	---	---	0.83329	1.06941022	0.09282431
1434324_x_at	eukaryotic translation initiation factor 3, subunit H	Eif3h	0.83337	1.08509784	0.10940362
1424507_at	Ras and Rab interactor 1	Rin1	0.8334	1.08359254	0.11392432
1416232_at	oligodendrocyte transcription factor 2	Olig2	0.83342	1.07094987	0.09819154
1416996_at	TBC1 domain family, member 8	Tbc1d8	0.83343	1.07240292	0.09857105
1430199_at	RIKEN cDNA 1700007E05 gene	1700007E05Rik	0.83344	1.07821569	0.10359518
1425336_x_at	histocompatibility 2, K1, K region	H2-K1	0.83344	-1.06455359	-0.0933729
1426685_a_at	CCR4-NOT transcription complex, subunit 6	Cnot6	0.83345	1.07258591	0.09457456
1430868_at	RIKEN cDNA 5830403M04 gene	5830403M04Rik	0.83347	1.0650292	0.08768155
1454703_x_at	small nucleolar RNA host gene (non-protein coding) 1	Snhg1	0.83348	-1.06877524	-0.0994097
1453698_at	RIKEN cDNA 6030451C04 gene	6030451C04Rik	0.83349	1.10618505	0.13153974
1438694_at	casein alpha s2-like A	Csn1s2a	0.83356	1.08235791	0.11125759
1422619_at	phosphatidic acid phosphatase type 2A	Ppap2a	0.83362	-1.06795704	-0.1022898
1436589_x_at	protein kinase D2	Prkd2	0.83366	1.08211743	0.11124692

1456988_at	expressed sequence AI848258	AI848258	0.83372	-1.08455279	-0.1181049
1441859_x_at	---	---	0.83373	1.08376826	0.10648783
1419579_at	solute carrier family 7 (cationic amino acid transporter, y+ system	Slc7a12	0.83374	1.08415265	0.10856179
1425145_at	interleukin 1 receptor-like 1	Il1rl1	0.83376	1.08683508	0.11692289
1434064_at	ORAI calcium release-activated calcium modulator 3	Orai3	0.83379	1.07965519	0.10424625
1442427_at	---	---	0.83379	1.12389021	0.14408333
1418052_at	mevalonate kinase	Mvk	0.83381	1.06704694	0.09301411
1434218_at	RIKEN cDNA C330019G07 gene	C330019G07Rik	0.83381	-1.07505422	-0.1117856
1433368_x_at	RIKEN cDNA 8430401P03 gene	8430401P03Rik	0.83382	-1.08098243	-0.1188281
1451114_at	CKLF-like MARVEL transmembrane domain containing 6	Cmtm6	0.83382	1.08093939	0.10345111
1426077_at	monooxygenase, DBH-like 2	Moxd2	0.83382	-1.08031462	-0.1115022
1431504_at	RIKEN cDNA 2310061N02 gene	2310061N02Rik	0.83382	-1.08617392	-0.1362966
1431274_a_at	heat shock protein 9	Hspa9	0.83383	-1.07554558	-0.1212437
1455946_x_at	thymosin, beta 10	Tmsb10	0.83383	1.07507493	0.09902979
1456363_at	Suppression of tumorigenicity 7-like	St7l	0.83383	1.07055668	0.09768497
1446307_at	---	---	0.83384	1.10365944	0.13033575
1437155_a_at	WW domain containing transcription regulator 1	Wwtr1	0.83384	1.10442641	0.12600137
1443169_at	---	---	0.83385	1.08331339	0.11404389
1421755_at	G protein-coupled receptor 132	Gpr132	0.83385	-1.08167926	-0.1197725
1424910_at	kinesin family member 12	Kif12	0.83385	1.10290054	0.13154785
1458896_at	glucocorticoid modulatory element binding protein 1	Gmeb1	0.83385	1.0872816	0.11672044
1456126_at	mucosa associated lymphoid tissue lymphoma translocation gene	Malt1	0.83386	1.07634865	0.10602008
1443669_at	---	---	0.83387	1.0785318	0.10693701
1430377_at	RIKEN cDNA 1300015D01 gene	1300015D01Rik	0.83387	1.09208982	0.12154553
1422701_at	zeta-chain (TCR) associated protein kinase	Zap70	0.83387	1.1007144	0.12615123
1427457_a_at	bone morphogenetic protein 1	Bmp1	0.83388	-1.07743876	-0.1115196
1426943_at	integrator complex subunit 1	Ints1	0.83388	1.06580462	0.09052604
1416395_at	guanylate kinase 1	Guk1	0.83389	1.06114972	0.08262204
1449750_at	expressed sequence AA407331	AA407331	0.83389	-1.08398312	-0.1298336
1421864_at	diazepam binding inhibitor-like 5	Dbil5	0.8339	-1.08083691	-0.1185878
1455869_at	---	---	0.8339	1.0899351	0.11853008
1423468_at	STEAP family member 3	Steap3	0.8339	-1.0918008	-0.1605873
1426428_at	THAP domain containing 7	Thap7	0.8339	1.08637869	0.11123963
1424674_at	solute carrier family 39 (metal ion transporter), member 6	Slc39a6	0.8339	1.06432577	0.08951198
1445066_at	---	---	0.83391	-1.07277777	-0.1071309
1416978_at	Fc receptor, IgG, alpha chain transporter	Fcgrt	0.83392	-1.07620574	-0.1120778
1439944_at	---	---	0.83395	-1.07699575	-0.1177298
1428626_at	LysM, putative peptidoglycan-binding, domain containing 2	Lysmd2	0.83395	1.07986853	0.10659192
1429927_at	RIKEN cDNA 5830409B07 gene	5830409B07Rik	0.83398	-1.08391129	-0.1294488
1460083_at	A disintegrin and metallopeptidase domain 10	Adam10	0.83398	-1.08607341	-0.1324406
1446434_at	---	---	0.83402	1.09744366	0.12622619
1430121_at	RIKEN cDNA 1700006E09 gene	1700006E09Rik	0.83404	1.07907403	0.10312856
1440220_at	occludin	Ocln	0.83407	1.08444676	0.11385276
1431950_at	RIKEN cDNA 4930528G23 gene	4930528G23Rik	0.83414	-1.07918916	-0.1107912
1419396_at	AT rich interactive domain 3A (BRIGHT-like)	Arid3a	0.83416	1.0808469	0.10848001
1449654_s_at	expressed sequence C77545	C77545	0.83419	-1.0783222	-0.1123495
1436338_at	---	---	0.83421	-1.07414042	-0.1052494
1444665_at	pyruvate dehydrogenase phosphatase regulatory subunit	Pdpr	0.83425	1.08231985	0.11238715
1418376_at	fibroblast growth factor 15	Fgf15	0.83425	-1.08727527	-0.1435745
1441460_at	---	---	0.83426	1.11639462	0.14299139
1444818_at	---	---	0.83435	1.0721173	0.10024827
1453217_at	predicted gene 14590 /// predicted gene 16405 /// predicted gen	Gm14590 /// Gm16405 //	0.83437	1.07122838	0.09470205
1416122_at	cyclin D2	Ccnd2	0.83446	-1.07161979	-0.1064963
1426143_at	triadin	Trdn	0.83449	1.07857562	0.10673293
1429387_at	GRB2-related adaptor protein	Grap	0.83452	1.11131341	0.13676687
1426279_at	family with sequence similarity 133, member B	Fam133b	0.83453	1.05864604	0.08191939
1428601_at	RIKEN cDNA 1700003E16 gene	1700003E16Rik	0.83456	1.07864157	0.10651267
1436884_x_at	Ewing sarcoma breakpoint region 1	Ewsr1	0.83456	-1.05089414	-0.0717744
1459852_x_at	tribbles homolog 2 (Drosophila)	Trib2	0.83457	1.06794244	0.09323192
1435152_at	leukocyte receptor cluster (LRC) member 8	Leng8	0.8346	-1.07331265	-0.1188172
1428246_at	vacuolar protein sorting 26 homolog B (yeast)	Vps26b	0.8346	1.07070762	0.09356756
1443436_at	---	---	0.83462	1.07525048	0.10295842
1419909_at	M-phase phosphoprotein 9	Mphosph9	0.83463	1.06398193	0.08914336
1432530_s_at	bol, boule-like (Drosophila)	Boll	0.83465	-1.08289488	-0.1158008
1460173_at	LIM and SH3 protein 1	Lasp1	0.83465	-1.06065805	-0.0907819
1436856_x_at	threonyl-tRNA synthetase 2, mitochondrial (putative)	Tars2	0.83466	1.08302064	0.10505598
1423670_a_at	signal recognition particle receptor ('docking protein')	Srpr	0.83468	1.05504791	0.07710052
1447729_s_at	protease, serine, 44	Prss44	0.83469	1.07126002	0.09716762
1444367_at	fibronectin type III domain containing 7	Fndc7	0.8347	-1.08480978	-0.1189919
1420942_s_at	regulator of G-protein signaling 5	Rgs5	0.83471	-1.07375742	-0.1158188
1454258_at	RIKEN cDNA 1110008E08 gene	1110008E08Rik	0.83473	1.09406774	0.12437426
1439490_at	nuclear receptor subfamily 2, group C, member 1	Nr2c1	0.83474	1.08919092	0.11523221
1427153_at	branched chain ketoacid dehydrogenase E1, beta polypeptide	Bckdhb	0.83476	1.05614004	0.07804291
1435221_at	forkhead box P1	Foxp1	0.83477	1.08144605	0.10850855
1450381_a_at	B-cell leukemia/lymphoma 6	Bcl6	0.8348	1.07995698	0.10954487
1448514_at	cytochrome c oxidase, subunit Vb	Cox5b	0.8348	-1.08997504	-0.133119
1456130_at	thrombospondin, type I, domain containing 7A	Thsd7a	0.83482	1.05936652	0.08270122
1450315_at	vomeronasal 1 receptor 148	Vmn1r148	0.83482	1.097554	0.12391959
1451656_at	calmin	Clmn	0.83482	1.12721895	0.14963579
1420876_a_at	septin 6	Sep-06	0.83484	-1.07450818	-0.1058995
1450243_a_at	regulator of calcineurin 2	Rcan2	0.83484	1.09096388	0.12250451
1418321_at	dodecenoyl-Coenzyme A delta isomerase (3,2 trans-enoyl-Coeny	Dci	0.83486	1.07433921	0.09830211
1452651_a_at	myosin, light polypeptide 1	Myl1	0.83489	-1.08195439	-0.1210414
1458460_at	---	---	0.83494	1.1102667	0.13364599
1418407_at	phosphodiesterase 8A	Pde8a	0.83514	1.16742976	0.1800277
1438247_at	kelch-like 15 (Drosophila)	Klhl15	0.83518	1.12807499	0.15006674
1428984_a_at	RIKEN cDNA 1700012B09 gene	1700012B09Rik	0.83521	-1.08864199	-0.1313885
1417523_at	pleckstrin	Plek	0.83522	-1.0878551	-0.1258329
1427260_a_at	tropomyosin 3, gamma	Tpm3	0.83523	-1.0467822	-0.0670663
1424710_a_at	golgi reassembly stacking protein 2	Gorasp2	0.83523	1.05732521	0.07916162
1460456_at	prolyl-tRNA synthetase domain containing 1	Prorsd1	0.83524	-1.06416344	-0.0923514
1421236_at	receptor (TNFRSF)-interacting serine-threonine kinase 2	Ripk2	0.83526	1.07871724	0.10563087
1448643_at	Sjogren's syndrome nuclear autoantigen 1	Ssna1	0.8353	1.06654597	0.08927539
1444479_at	---	---	0.83531	-1.08130062	-0.1146013

1442821_at	---	---	0.83531	1.0950239	0.1243148
1452516_at	sperm motility kinase 3A	Smok3a	0.83534	1.08937737	0.11923748
1450483_at	gap junction protein, gamma 2	Gjc2	0.83536	-1.09377407	-0.1634816
1432056_at	carboxypeptidase, vitellogenic-like	Cpvl	0.8354	1.12502923	0.14713243
1427053_at	ABI gene family, member 3 (NESH) binding protein	Abi3bp	0.83541	1.09344847	0.11113815
1450321_at	zinc finger protein 354C	Zfp354c	0.83546	1.10276382	0.12775578
1458147_at	---	---	0.83551	1.08976949	0.1177213
1448638_at	Mdm2, transformed 3T3 cell double minute p53 binding protein	Mtbp	0.83561	1.09256211	0.11571958
1460293_at	neuronal calcium sensor 1	Ncs1	0.8357	-1.08341257	-0.1270637
1422090_a_at	6-phosphofructo-2-kinase/fructose-2,6-biphosphatase 2	Pfkfb2	0.83573	1.08075027	0.10907119
1423875_at	family with sequence similarity 160, member B1	Fam160b1	0.83576	1.08756629	0.11329652
1451932_a_at	ADAMTS-like 4	Adamtsl4	0.83577	1.07594526	0.10344863
1450126_at	GATA binding protein 5	Gata5	0.83581	-1.08629998	-0.1305038
1459012_at	RIKEN cDNA A730041O05 gene	A730041O05Rik	0.83583	1.05521924	0.07555523
1428653_x_at	ELAV (embryonic lethal, abnormal vision, Drosophila)-like 1 (Hu a	Elavl1	0.83584	-1.07334631	-0.1040433
1434684_at	Ras and Rab interactor 3	Rin3	0.83588	1.08579368	0.11553331
1430592_at	transmembrane protein 106B	Tmem106b	0.83589	-1.08385986	-0.1256355
1444172_at	hypothetical LOC100502914	LOC100502914	0.83589	1.10475157	0.1319362
1435868_at	ankyrin repeat domain 13c	Ankrd13c	0.83589	-1.07101794	-0.1097645
1434122_at	F-box protein 27	Fbxo27	0.8359	-1.06928241	-0.100765
1418914_s_at	betaine-homocysteine methyltransferase 2	Bhmt2	0.8359	1.07564468	0.10368974
1440538_at	---	---	0.8359	1.11436814	0.13911244
1451268_at	translocation associated membrane protein 1-like 1	Tram1l1	0.8359	-1.0788834	-0.109954
1444625_at	hypothetical LOC100503307	LOC100503307	0.8359	1.07777779	0.10595108
1432473_a_at	ATP-binding cassette, sub-family A (ABC1), member 14	Abca14	0.83591	1.07140899	0.09771155
1423478_at	protein kinase C, beta	Prkcb	0.83591	-1.08643818	-0.140559
1428010_at	translocase of inner mitochondrial membrane 9 homolog (yeast)	Timm9	0.83592	-1.09147673	-0.1449967
1440561_at	---	---	0.83592	-1.09041194	-0.1437466
1441894_s_at	GRP1 (general receptor for phosphoinositides 1)-associated scaffo	Grasp	0.83592	1.06068384	0.08305284
1437516_at	---	---	0.83592	-1.07673934	-0.1101681
1436449_at	protocadherin 11 X-linked	Pcdh11x	0.83593	-1.07365213	-0.1038585
1432546_at	RIKEN cDNA 4930529I22 gene	4930529I22Rik	0.83594	-1.0812991	-0.1195476
1430894_at	RIKEN cDNA 5530401N12 gene /// hypothetical LOC100503359	5530401N12Rik /// LOC10	0.83594	1.08095316	0.11088974
1458890_at	RIKEN cDNA D030055H07 gene	D030055H07Rik	0.83594	-1.08398977	-0.1202003
1447291_at	nephrosis 1 homolog, nephrin antisense	Nphs1as	0.83595	1.10424491	0.1319429
1419988_at	mitogen-activated protein kinase kinase kinase 7	Map3k7	0.83595	-1.06662213	-0.0933893
1442922_at	checkpoint with forkhead and ring finger domains	Chfr	0.83596	-1.07978762	-0.1158627
1442914_at	TATA element modulatory factor 1	Tmf1	0.83597	-1.06464772	-0.0908903
1431183_at	RIKEN cDNA 1700066M21 gene	1700066M21Rik	0.836	-1.0840296	-0.130998
1442718_at	---	---	0.836	1.09588994	0.12328494
1425147_at	RIKEN cDNA 2410075B13 gene	2410075B13Rik	0.83601	-1.06800929	-0.0951555
1417459_at	demilune cell and parotid protein 1	Dcpp1	0.83601	-1.07891016	-0.1118967
1432976_at	carboxylesterase 2F	Ces2f	0.83602	1.09167713	0.11579208
1428797_at	SET domain containing 6	Setd6	0.83605	-1.06420819	-0.0918772
1424488_a_at	pyrophosphatase (inorganic) 2	Ppa2	0.83605	1.06115981	0.08396391
1420213_x_at	---	---	0.83609	1.06137736	0.08315763
1424251_a_at	heterogeneous nuclear ribonucleoprotein D-like	Hnrpdl	0.83612	1.03938301	0.05560894
1455744_at	---	---	0.83612	-1.08851199	-0.1646992
1425702_a_at	ectonucleotide pyrophosphatase/phosphodiesterase 5	Enpp5	0.83614	1.16449301	0.17448594
1444041_at	expressed sequence AU041133	AU041133	0.83615	-1.08748846	-0.1256503
1415723_at	eukaryotic translation initiation factor 5	Eif5	0.83616	1.05633237	0.07705975
1429057_at	N(alpha)-acetyltransferase 16, NatA auxiliary subunit	Naa16	0.83618	-1.07117626	-0.1129964
1444844_at	fatty acid binding protein 3, muscle and heart	Fabp3	0.83619	1.08777059	0.11891474
AFFX-ThrX-M_at	---	---	0.8362	1.1067905	0.13361297
1426209_at	striatin, calmodulin binding protein 4	Strn4	0.8362	1.05396125	0.07559418
1446720_at	---	---	0.83621	1.07670096	0.10497873
1449841_at	kinesin family member 3A	Kif3a	0.83622	-1.06878435	-0.0980429
1447751_x_at	dihydrouridine synthase 2-like (SMM1, S. cerevisiae)	Dus2l	0.83622	-1.06661845	-0.0948827
1442879_at	---	---	0.83623	1.06026407	0.08198688
1449935_a_at	DnaJ (Hsp40) homolog, subfamily A, member 3	Dnaja3	0.83626	1.05894563	0.08209091
1430852_at	RIKEN cDNA 1600029I14 gene	1600029I14Rik	0.83626	-1.07233346	-0.10236
1419259_at	Ras suppressor protein 1	Rsu1	0.83626	-1.05536481	-0.0778403
1444091_a_at	RIKEN cDNA 6430571L13 gene	6430571L13Rik	0.83629	1.07983935	0.11068333
1417035_at	SAC3 domain containing 1	Sac3d1	0.83631	1.05370779	0.07483287
1422100_at	cytochrome P450, family 7, subfamily a, polypeptide 1	Cyp7a1	0.83631	-1.08293354	-0.1278706
1433171_at	RIKEN cDNA 3110049I03 gene	3110049I03Rik	0.83631	-1.0695355	-0.0983974
1455578_x_at	ribosomal protein L41	Rpl41	0.83632	1.03136384	0.04334431
1423657_at	CDP-diacylglycerol--inositol 3-phosphatidyltransferase (phosphat	Cdipt	0.83632	1.05426559	0.07578561
1435092_at	ADP-ribosylation factor-like 4A	Arl4a	0.83633	1.08958777	0.11613318
1425468_at	proteolipid protein (myelin) 1	Plp1	0.83633	1.08878028	0.11620837
1434181_at	fermitin family homolog 2 (Drosophila)	Fermt2	0.83634	-1.06256359	-0.0943207
1459911_at	cerebellar degeneration-related protein 2-like	Cdr2l	0.83634	1.07226051	0.09673815
1418396_at	G-protein signalling modulator 3 (AGS3-like, C. elegans)	Gpsm3	0.83634	1.07922594	0.10332338
1440813_s_at	plexin B3	Plxnb3	0.83634	-1.06494957	-0.0908938
1444937_at	---	---	0.83634	-1.09103969	-0.1565641
1449287_at	src-related kinase lacking C-terminal regulatory tyrosine and N-te	Srms	0.83634	1.07785436	0.10664729
1433725_at	activin A receptor, type 1B	Acvr1b	0.83636	1.05724188	0.07892718
1428253_at	chromatin modifying protein 2B	Chmp2b	0.83636	1.06408903	0.08715594
1459260_at	predicted gene 13212	Gm13212	0.83636	-1.08451116	-0.1378696
1449858_at	CD86 antigen	Cd86	0.83637	1.12648629	0.14675733
1432127_at	RIKEN cDNA 4933401L05 gene	4933401L05Rik	0.83637	1.09147373	0.12071196
1442159_at	DNA segment, Chr 2, ERATO Doi 48, expressed	D2Ertd48e	0.83637	-1.07877851	-0.1115041
1441708_at	sperm associated antigen 16	Spag16	0.83637	1.09329402	0.11935417
1420854_at	elastin	Eln	0.83639	-1.07724216	-0.1082953
1424494_s_at	FLYWCH family member 2	Flywch2	0.8364	1.09546488	0.11748807
1443297_at	---	---	0.8364	-1.09079226	-0.1402248
1445083_at	---	---	0.8364	1.09078366	0.12178409
1452737_at	RIKEN cDNA 2810008M24 gene	2810008M24Rik	0.83641	-1.05077964	-0.071584
1425090_s_at	potassium voltage gated channel, Shaw-related subfamily, meml	Kcnc4	0.83641	-1.08026225	-0.1145949
1455863_at	spermatogenesis associated 5-like 1	Spata5l1	0.83642	-1.07495465	-0.1058009
1435008_at	solute carrier family 9 (sodium/hydrogen exchanger), member 6	Slc9a6	0.83642	1.10471218	0.12589328
1424416_at	NFKB inhibitor interacting Ras-like protein 2	Nkiras2	0.83642	1.05969275	0.08310526
1454034_a_at	ubiquitin specific peptidase 21	Usp21	0.83642	1.05819981	0.08071919
1424348_at	RIKEN cDNA 1110007A13 gene	1110007A13Rik	0.83642	1.05655518	0.07827899

1419791_at	Zinc fingers and homeoboxes 3	Zhx3	0.83643	-1.08316445	-0.116219
1415917_at	methylenetetrahydrofolate dehydrogenase (NADP+ dependent),	Mthfd1	0.83643	1.05467737	0.07550215
1455315_at	transformation related protein 53 inducible protein 13	Trp53i13	0.83643	1.0971501	0.11974324
1421739_a_at	megakaryocyte-associated tyrosine kinase	Matk	0.83643	1.12539185	0.14758336
1420113_s_at	RIKEN cDNA 2410022L05 gene	2410022L05Rik	0.83644	1.07639731	0.098089
1434395_at	mannosidase, alpha, class 1A, member 2	Man1a2	0.83646	1.06846243	0.09521429
1439699_at	---	---	0.83646	-1.07300491	-0.108909
1452639_at	ectonucleotide pyrophosphatase/phosphodiesterase 4	Enpp4	0.83646	-1.0861394	-0.1316158
1446127_at	Zinc finger E-box binding homeobox 1	Zeb1	0.83647	-1.09089577	-0.147042
1430222_at	RIKEN cDNA 9130007G19 gene	9130007G19Rik	0.83647	1.08936099	0.12032273
1420930_s_at	catenin (cadherin associated protein), alpha-like 1	Ctnnal1	0.83648	1.06181107	0.0857539
1426835_at	methionyl aminopeptidase 1	Metap1	0.83648	1.05574288	0.07793657
1442107_at	filamin, beta	Flnb	0.83648	1.1328745	0.15139
1416686_at	procollagen lysine, 2-oxoglutarate 5-dioxygenase 2	Plod2	0.83649	1.14001938	0.14961054
1424225_at	ankyrin repeat and SOCS box-containing 8	Asb8	0.8365	1.06438673	0.08988833
1421732_at	glutamine repeat protein 1	Glrp1	0.8365	1.08166823	0.1094786
1435459_at	flavin containing monooxygenase 2	Fmo2	0.83651	-1.07938538	-0.1129508
1440788_at	growth arrest specific 8	Gas8	0.83651	-1.08286218	-0.1229893
1417722_at	6-phosphogluconolactonase	Pgls	0.83653	1.07042182	0.0913619
1415761_at	mitochondrial ribosomal protein L52	Mrpl52	0.83653	1.08229812	0.10205476
1444738_at	RIKEN cDNA 5730419I09 gene	5730419I09Rik	0.83655	1.08999237	0.11589012
1432482_at	lysozyme-like 6	Lyzl6	0.83656	1.07807129	0.1078159
1420464_s_at	predicted pseudogene 10693 /// predicted gene 14548 /// leuko	Gm10693 /// Gm14548 //	0.83656	1.09392026	0.11528402
1419976_s_at	nuclear factor of activated T-cells, cytoplasmic, calcineurin-depen	Nfatc3	0.83656	1.0601231	0.08412569
1442129_at	---	---	0.8366	1.07144023	0.09904036
1423085_at	ephrin B3	Efnb3	0.8366	1.07807356	0.10581257
1424685_at	exosome component 4	Exosc4	0.83675	1.06967816	0.09033825
1434719_at	alpha-2-macroglobulin	A2m	0.83679	1.06802878	0.09401136
1431242_at	RIKEN cDNA 6330575P09 gene	6330575P09Rik	0.83684	-1.08170498	-0.122731
1451796_s_at	histidine decarboxylase	Hdc	0.83686	-1.08691567	-0.1361359
1420544_at	germinal center expressed transcript 2	Gcet2	0.8369	-1.07696283	-0.1137404
1452720_a_at	FIP1 like 1 (S. cerevisiae)	Fip1l1	0.83704	-1.06366683	-0.0902828
1425865_a_at	ligase III, DNA, ATP-dependent	Lig3	0.8371	1.07507794	0.10147089
1442072_at	RIKEN cDNA C230081A13 gene	C230081A13Rik	0.83712	1.10159734	0.1282048
1447002_at	---	---	0.83713	-1.07997185	-0.116315
1430787_at	RIKEN cDNA 2310050B05 gene	2310050B05Rik	0.83717	-1.0687464	-0.0968932
1438480_a_at	thymocyte nuclear protein 1	Thyn1	0.8372	1.07374889	0.09646682
1450326_at	src homology 2 domain-containing transforming protein C3	Shc3	0.83722	1.0827348	0.1119965
1438199_at	expressed sequence AI316807	AI316807	0.83724	-1.07174102	-0.1054505
1458106_at	transmembrane protein 60	Tmem60	0.83726	-1.10023305	-0.1785059
1427645_a_at	interleukin 1 receptor accessory protein-like 2	Il1rapl2	0.83729	1.07168401	0.09969116
1423784_at	glycyl-tRNA synthetase	Gars	0.83735	-1.04511173	-0.0640667
1457458_at	zinc finger CCCH-type containing 4	Zc3h4	0.83736	1.1047909	0.13415831
1456406_at	predicted gene 14964	Gm14964	0.83738	1.09241088	0.12037166
1436664_a_at	solute carrier family 35 (UDP-galactose transporter), member A2	Slc35a2	0.8374	1.10743706	0.13002239
1437379_x_at	TNF receptor-associated protein 1	Trap1	0.83741	-1.06709808	-0.1013463
1457088_at	pallidin	Pldn	0.83742	1.07712283	0.10297122
1455793_at	family with sequence similarity 149, member A	Fam149a	0.83743	1.14251566	0.15950195
1421987_at	3'-phosphoadenosine 5'-phosphosulfate synthase 2	Papss2	0.83743	-1.08488852	-0.1228088
1429734_at	RIKEN cDNA 4632434I11 gene	4632434I11Rik	0.83744	-1.0655768	-0.1004831
1442763_s_at	tubulin tyrosine ligase-like family, member 10	Ttll10	0.83745	-1.08368518	-0.1205683
1435528_at	DNL-type zinc finger	Dnlz	0.83746	1.0777606	0.10618983
1438711_at	pyruvate kinase liver and red blood cell	Pklr	0.83746	-1.08252898	-0.1175535
1416788_a_at	isocitrate dehydrogenase 3 (NAD+), gamma	Idh3g	0.83747	1.05261465	0.07320387
1450623_at	guanine nucleotide binding protein (G protein), beta 2	Gnb2	0.83749	1.05620079	0.07768996
1451643_a_at	RAB4B, member RAS oncogene family	Rab4b	0.83749	1.05981081	0.08156764
1425422_a_at	poly(A)-specific ribonuclease (deadenylation nuclease)	Parn	0.8375	1.05887083	0.08180153
1448061_at	macrophage scavenger receptor 1	Msr1	0.8375	-1.09072812	-0.1568601
1448793_a_at	syndecan 4	Sdc4	0.8375	1.07104265	0.09222449
1425492_at	bone morphogenetic protein receptor, type 1A	Bmpr1a	0.83751	1.06630715	0.08767291
1456139_at	antisense Igf2r RNA	Airn	0.83751	1.11028454	0.13771105
1439717_at	gamma-aminobutyric acid (GABA) A receptor, subunit gamma 3	Gabrg3	0.83752	-1.08081154	-0.119207
1457394_at	RIKEN cDNA 2900002K06 gene	2900002K06Rik	0.83754	-1.08826145	-0.1355533
1442864_at	estrogen related receptor, alpha	Esrra	0.83754	-1.08502608	-0.1250299
1451501_a_at	growth hormone receptor	Ghr	0.83755	-1.08459078	-0.1292078
1444490_at	---	---	0.83756	-1.08201215	-0.1179178
1452051_at	ARP3 actin-related protein 3 homolog (yeast)	Actr3	0.83756	1.06720906	0.08713618
1425741_at	SLIT-ROBO Rho GTPase activating protein 3	Srgap3	0.83756	-1.09220172	-0.1425817
1418533_s_at	frizzled homolog 2 (Drosophila)	Fzd2	0.83757	1.10950326	0.13399022
1446335_at	---	---	0.83757	-1.08263745	-0.1179674
1430126_at	RIKEN cDNA 2310035C23 gene	2310035C23Rik	0.83757	1.10489554	0.12932545
1423188_a_at	RIKEN cDNA 6720456B07 gene	6720456B07Rik	0.83758	-1.0533584	-0.0751868
1460160_at	---	---	0.83758	-1.08433859	-0.1177621
1422202_at	thyroid hormone receptor beta	Thrb	0.83758	-1.08381068	-0.1168435
1455817_x_at	zinc finger, X-linked, duplicated B	Zxdb	0.83759	-1.08268603	-0.1244581
1430842_at	RIKEN cDNA 1500002F19 gene	1500002F19Rik	0.83759	-1.07770571	-0.1087563
1455618_x_at	tetraspanin 33	Tspan33	0.8376	1.08294035	0.11334735
1417495_x_at	ceruloplasmin	Cp	0.8376	1.07002134	0.09727691
1424846_at	centrosomal protein 68	Cep68	0.8376	1.07248448	0.09749776
1454633_at	ethanolamine kinase 1	Etnk1	0.8376	-1.07866766	-0.1322875
1419725_at	solute carrier family 26, member 4	Slc26a4	0.83761	-1.08069436	-0.1165612
1454256_s_at	RIKEN cDNA 1700113I22 gene /// alanyl-tRNA synthetase domai	1700113I22Rik /// Aarsd1	0.83762	1.10739118	0.13148418
1423406_at	synaptic vesicle glycoprotein 2 a	Sv2a	0.83762	1.07856464	0.10655172
1456894_at	RIKEN cDNA C130039O16 gene	C130039O16Rik	0.83763	1.10056981	0.12701556
1428453_at	N(alpha)-acetyltransferase 30, NatC catalytic subunit	Naa30	0.83763	-1.05766178	-0.0811241
1435254_at	plexin B1	Plxnb1	0.83764	1.08060598	0.10914606
1438358_x_at	prefoldin 5	Pfdn5	0.83765	-1.07430143	-0.1087197
1423603_at	zinc finger protein, multitype 1	Zfpm1	0.83766	1.08372222	0.11301669
1422463_a_at	mitochondrial ribosomal protein L3	Mrpl3	0.83766	-1.0550952	-0.0791614
1453682_at	hypothetical LOC100504029	LOC100504029	0.83767	1.10355411	0.12766821
1421991_a_at	insulin-like growth factor binding protein 4	Igfbp4	0.83768	1.09253586	0.1184017
1433347_at	RIKEN cDNA A930036A04 gene	A930036A04Rik	0.83768	1.08179554	0.1127606
1427699_a_at	protein tyrosine phosphatase, non-receptor type 11	Ptpn11	0.83769	1.08543763	0.11445186
1438203_at	Scavenger receptor class F, member 2	Scarf2	0.83769	1.114239	0.14033853

1437136_at	RIKEN cDNA 5830436I19 gene	5830436I19Rik	0.83769	1.0778466	0.10618073
1422391_at	vomeronasal 1 receptor 11	Vmn1r11	0.83771	1.05445703	0.07515295
1455968_x_at	transmembrane emp24 domain trafficking protein 2	Tmed2	0.83772	-1.05277908	-0.0758285
1442574_at	predicted gene 10785	Gm10785	0.83774	1.09166482	0.12079116
1417675_a_at	midasin homolog (yeast)	Mdn1	0.83776	-1.05696843	-0.081748
1454698_at	protein tyrosine phosphatase-like A domain containing 1	Ptplad1	0.83776	1.05663022	0.0790226
1439715_at	O-sialoglycoprotein endopeptidase-like 1	Osgepl1	0.83776	1.0913973	0.12292116
1425967_a_at	mast cell protease 4	Mcpt4	0.83779	-1.0914924	-0.1452894
1425525_a_at	purinergic receptor P2X, ligand-gated ion channel 4	P2rx4	0.8378	-1.07767436	-0.1153524
1447851_x_at	ATPase, class V, type 10A	Atp10a	0.83781	1.119663	0.14340428
1454302_at	RIKEN cDNA 5730410E19 gene	5730410E19Rik	0.83781	-1.06362685	-0.089152
1435164_s_at	ubiquitin-like modifier activating enzyme 3	Uba3	0.83782	-1.06289089	-0.0973815
1460542_s_at	transformed mouse 3T3 cell double minute 4	Mdm4	0.83784	1.06090301	0.08508345
1430554_at	leucine-rich repeats and immunoglobulin-like domains 3	Lrig3	0.83784	-1.06732577	-0.0985128
1416366_at	NADH dehydrogenase (ubiquinone) 1, subcomplex unknown, 2	Ndufc2	0.83785	1.07026139	0.09129737
1428663_at	sphingomyelin synthase 2	Sgms2	0.83785	-1.08072512	-0.1158588
1416029_at	Kruppel-like factor 10	Klf10	0.83788	1.06112654	0.08440951
1436832_at	RIKEN cDNA 1810013D10 gene	1810013D10Rik	0.83804	1.06635909	0.09207978
1458232_at	dickkopf homolog 1 (Xenopus laevis)	Dkk1	0.83805	1.07602237	0.10170962
1431031_at	AT rich interactive domain 4B (RBP1-like)	Arid4b	0.83806	1.09093568	0.11912888
1419520_at	N-acetyltransferase 8 (GCN5-related, putative)	Nat8	0.83807	-1.07561105	-0.1095962
1420691_at	interleukin 2 receptor, alpha chain	Il2ra	0.83809	1.10023386	0.12805162
1453574_at	hemoglobin alpha, adult chain 1	Hba-a1	0.8381	1.08358273	0.11544915
1444195_at	---	---	0.8381	1.11427799	0.13822505
1425478_x_at	ubiquitin-conjugating enzyme E2I	Ube2i	0.83811	1.07656682	0.10440647
1443722_at	---	---	0.83811	-1.07925635	-0.1147701
1429528_at	RAE1 RNA export 1 homolog (S. pombe)	Rae1	0.83811	1.051483	0.07214165
1428208_at	B-cell CLL/lymphoma 7A	Bcl7a	0.83812	-1.08389067	-0.1220842
1440812_at	---	---	0.83812	1.07413795	0.09360866
1419459_a_at	magnesium transporter 1	Magt1	0.83812	-1.06433836	-0.0915502
1417953_at	family with sequence similarity 3, member C	Fam3c	0.83813	1.06050177	0.08230767
1421760_at	pre T-cell antigen receptor alpha	Ptcra	0.83814	1.08158277	0.11168777
1416073_a_at	nucleoporin 85	Nup85	0.83814	1.05558119	0.07727091
1421891_at	ST3 beta-galactoside alpha-2,3-sialyltransferase 2	St3gal2	0.83814	1.0875106	0.11180321
1452872_at	ankyrin 3, epithelial	Ank3	0.83814	1.09297427	0.12293345
1455651_at	telomeric repeat binding factor 2	Terf2	0.83814	-1.05777218	-0.0814695
1434658_at	RIKEN cDNA 3110056O03 gene	3110056O03Rik	0.83815	1.06872496	0.09207034
1459129_at	---	---	0.83815	-1.08270383	-0.1215059
1460614_at	mesoderm induction early response 1, family member 3	Mier3	0.83815	-1.07699611	-0.1274965
1459564_at	---	---	0.83816	1.08173076	0.11017249
1457584_at	expressed sequence AI848100	AI848100	0.83816	1.05555457	0.07626086
1430960_at	RIKEN cDNA 4933440J02 gene	4933440J02Rik	0.83816	1.08714843	0.11264872
1458661_at	---	---	0.83816	1.09590742	0.12161045
1431286_at	RIKEN cDNA 5830416I19 gene	5830416I19Rik	0.83817	1.11634305	0.13998788
1422096_at	RIKEN cDNA 4933434I20 gene	4933434I20Rik	0.83817	1.07895274	0.10941269
1420686_at	crystallin, beta A4	Cryba4	0.83817	-1.08746365	-0.134133
1455348_x_at	predicted gene 10709 /// ribosomal protein L29 pseudogene ///	Gm10709 /// Gm6344 ///	0.83818	1.03218275	0.04523942
1425870_a_at	Kv channel-interacting protein 2	Kcnip2	0.83818	1.10450794	0.13191811
1460343_at	neuralized homolog 1A (Drosophila)	Neurl1a	0.83818	1.09509829	0.11811201
1429817_at	spermatogenesis and oogenesis specific basic helix-loop-helix 2	Sohlh2	0.83819	1.06186068	0.08587728
1427100_at	meteorin, glial cell differentiation regulator	Metrn	0.83819	1.10027428	0.12116257
1458844_at	---	---	0.83819	-1.08099627	-0.1258213
1421049_at	RIKEN cDNA 0610009O20 gene	0610009O20Rik	0.83819	1.08190657	0.10808466
1441405_at	---	---	0.8382	1.11563252	0.14006471
1450286_at	natriuretic peptide receptor 3	Npr3	0.8382	-1.0809193	-0.1211569
1450416_at	chromobox homolog 5 (Drosophila HP1a)	Cbx5	0.8382	1.05629206	0.0784624
1455779_at	microtubule-associated protein 1 A	Mtap1a	0.8382	1.08492388	0.11573169
1460545_at	thyroid hormone receptor associated protein 3	Thrap3	0.8382	-1.05735195	-0.0841945
1453663_at	RIKEN cDNA 4932442E05 gene	4932442E05Rik	0.83821	-1.08160668	-0.1241897
1436049_at	TOX high mobility group box family member 4	Tox4	0.83821	1.07422529	0.10240662
1440524_at	4lysine (K)-specific demethylase 6A	Kdm6a	0.83821	1.09406148	0.12085642
1440437_at	hect (homologous to the E6-AP (UBE3A) carboxyl terminus) doma	Herc1	0.83821	-1.10124242	-0.2061741
1420664_s_at	protein C receptor, endothelial	Procr	0.83823	1.08123281	0.10995309
1427814_at	oncofetal antigen	Ofa	0.83823	1.08026198	0.10473676
1456728_x_at	aconitase 1	Aco1	0.83824	-1.0608497	-0.0877026
1420501_at	DnaJ (Hsp40) homolog, subfamily C, member 1	Dnajc1	0.83825	-1.06839133	-0.0966852
1431892_a_at	phospholipase C, delta 3	Plcd3	0.83825	-1.06536412	-0.0915114
1455987_at	Sec61 alpha 1 subunit (S. cerevisiae)	Sec61a1	0.83825	1.10366123	0.12766404
1457854_at	expressed sequence AU018877	AU018877	0.83826	1.08178257	0.11267412
1445014_at	expressed sequence AU019157	AU019157	0.83826	1.08624361	0.11486913
1444327_at	ubiquitin protein ligase E3 component n-recognin 5	Ubr5	0.83826	-1.0912336	-0.1621883
1454807_a_at	sorting nexin 12	Snx12	0.83826	1.06539972	0.08833751
1430255_at	Kruppel-like factor 5	Klf5	0.83826	-1.08123269	-0.1139976
1434392_at	ubiquitin specific peptidase 34	Usp34	0.83827	-1.05848732	-0.0863157
1431406_at	alanine-glyoxylate aminotransferase 2-like 1	Agxt2l1	0.83827	1.08258531	0.10926463
1438906_at	---	---	0.83827	1.10703799	0.13239135
1426963_at	phosphofurin acidic cluster sorting protein 2	Pacs2	0.83827	1.06183596	0.08639455
1445070_at	---	---	0.83828	1.09003273	0.11679619
1445264_at	cation channel, sperm associated 2	Catsper2	0.83829	1.07647614	0.10447579
1449806_at	---	---	0.83829	-1.07414659	-0.1033113
1417758_at	integrin alpha 2b	Itga2b	0.83829	1.06811628	0.09465383
1447125_at	---	---	0.83829	1.08003397	0.1098293
1454173_at	SH2 domain containing 4B	Sh2d4b	0.83829	1.08186227	0.11150646
1450201_at	protein Z, vitamin K-dependent plasma glycoprotein	Proz	0.8383	1.0759075	0.10299642
1458806_at	---	---	0.8383	-1.08121121	-0.1132884
1417306_at	tyrosine kinase 2	Tyk2	0.8383	1.05936642	0.08233859
1434967_at	zinc finger, SWIM domain containing 6	Zswim6	0.83831	1.05682472	0.07968515
1452258_at	PHD finger protein 20	Phf20	0.83831	1.07727836	0.10143138
1460661_at	sphingosine-1-phosphate receptor 3	S1pr3	0.83832	-1.08814869	-0.1360404
1429260_at	RIKEN cDNA 1810014B01 gene	1810014B01Rik	0.83834	-1.08941321	-0.1335918
1434672_at	G protein-coupled receptor 22	Gpr22	0.83834	1.07674238	0.10635052
1458270_at	---	---	0.83834	1.09317851	0.12152403
1426266_s_at	zinc finger and BTB domain containing 8 opposite strand	Zbtb8os	0.83835	1.06585588	0.08704645
1440195_at	serpine1 mRNA binding protein 1	Serbp1	0.83838	-1.0685182	-0.0993756

1455592_at	vang-like 2 (van gogh, Drosophila)	Vangl2	0.83839	1.10227681	0.12253546
1460738_at	LIM domain containing 2	Limd2	0.8384	1.10580554	0.13170557
1426958_at	ribosomal protein S9	Rps9	0.83841	1.11146103	0.13018184
1438135_at	---	---	0.83845	1.11651422	0.1393041
1441549_at	Phosphatidylinositol 4-kinase, catalytic, beta polypeptide	Pi4kb	0.83847	1.10984061	0.13693569
1419216_at	5-azacytidine induced gene 1	Azi1	0.83848	1.07572525	0.10060324
1446278_at	---	---	0.83849	-1.09142083	-0.1420895
1436555_at	solute carrier family 7 (cationic amino acid transporter, y+ system	Slc7a2	0.8385	-1.08043729	-0.1199716
1418453_a_at	ATPase, Na+/K+ transporting, beta 1 polypeptide	Atp1b1	0.83852	1.08217938	0.10155213
1426157_a_at	CD209b antigen	Cd209b	0.83854	1.10797967	0.13269255
1423640_at	synaptoporin	Synpr	0.83856	-1.06401098	-0.0924844
1433568_at	PAP associated domain containing 4	Papd4	0.83857	-1.08745284	-0.153304
1445591_at	---	---	0.83858	-1.08204098	-0.1197733
1439763_at	DnaJ (Hsp40) homolog, subfamily C, member 11	Dnajc11	0.83859	-1.07209837	-0.1004978
1459927_at	microRNA 425	Mir425	0.83861	-1.09150394	-0.1553829
1426043_a_at	calpain 3	Capn3	0.83861	1.08728331	0.11282033
1418891_a_at	RAB3D, member RAS oncogene family	Rab3d	0.83862	1.07964844	0.10720722
1439727_at	chloride channel calcium activated 6	Clca6	0.83863	-1.08113183	-0.1139105
1438827_at	Glutaminase	Gls	0.83864	1.08761278	0.11542867
1419199_at	chromobox homolog 8 (Drosophila Pc class)	Cbx8	0.83865	1.08248945	0.11271014
1437698_at	myosin VB	Myo5b	0.83865	1.09744344	0.12549418
1445084_at	---	---	0.83865	-1.08738627	-0.1390916
1427772_at	defensin beta 15	Defb15	0.83865	-1.07833438	-0.1154273
1428769_at	TatD DNase domain containing 3	Tatdn3	0.83866	1.08390543	0.10847073
1452792_at	DAZ interacting protein 1	Dzip1	0.83866	1.08662872	0.11835432
1436104_a_at	coiled-coil domain containing 74A	Ccdc74a	0.83867	1.08189311	0.11146639
1444855_at	---	---	0.83868	-1.08163636	-0.1170782
1418969_at	S-phase kinase-associated protein 2 (p45)	Skp2	0.83868	-1.05546525	-0.0785453
1449409_at	sulfotransferase family, cytosolic, 1C, member 2	Sult1c2	0.83869	-1.08947922	-0.1397528
1431135_at	---	---	0.8387	-1.07991046	-0.1188344
1418843_at	solute carrier family 30 (zinc transporter), member 4	Slc30a4	0.8387	-1.07575949	-0.1068429
1433102_at	RIKEN cDNA 3100002H20 gene	3100002H20Rik	0.83871	-1.06443521	-0.0922427
1418929_at	intraflagellar transport 57 homolog (Chlamydomonas)	Ift57	0.83872	1.06806398	0.09350188
1446308_at	RIKEN cDNA 1700106J16 gene	1700106J16Rik	0.83872	-1.07901016	-0.1133517
1418725_at	DnaJ (Hsp40) homolog, subfamily C, member 5 beta	Dnajc5b	0.83873	1.09371516	0.12284758
1440717_at	expressed sequence AA407881	AA407881	0.83873	1.11486424	0.13914341
1443537_at	RIKEN cDNA C130009A20 gene	C130009A20Rik	0.83874	-1.0632231	-0.0907949
1457437_at	family with sequence similarity 163, member B	Fam163b	0.83875	-1.07904645	-0.1138342
1452147_at	Sec24 related gene family, member C (S. cerevisiae)	Sec24c	0.83875	1.05291894	0.07393232
1439001_at	RIKEN cDNA 6030419C18 gene	6030419C18Rik	0.83879	1.08406251	0.11364483
1453962_at	profilin 3	Pfn3	0.83879	1.07054486	0.09659025
1427096_s_at	signal sequence receptor, delta	Ssr4	0.8388	-1.07259134	-0.1035352
1415762_x_at	mitochondrial ribosomal protein L52	Mrpl52	0.83883	1.08081216	0.10021995
1419573_a_at	lectin, galactose binding, soluble 1	Lgals1	0.83884	1.05747055	0.07809141
1447592_at	dopamine beta hydroxylase	Dbh	0.83884	1.09639014	0.12318605
1443057_at	---	---	0.83885	1.07494077	0.10397681
1441289_at	---	---	0.83888	1.07537554	0.10396076
1421639_at	RIKEN cDNA 1700082M22 gene	1700082M22Rik	0.83889	-1.08062061	-0.1180962
1458881_at	RIKEN cDNA 9530001P21 gene	9530001P21Rik	0.83889	-1.08231538	-0.1179718
1432209_at	RIKEN cDNA 1810010K12 gene	1810010K12Rik	0.8389	1.12497259	0.14489102
1436143_at	neuroblastoma amplified sequence	Nbas	0.83896	1.07648206	0.10147443
1422763_at	GIPC PDZ domain containing family, member 1	Gipc1	0.83897	1.09036012	0.10883648
1432742_at	---	---	0.83897	1.0865953	0.11654632
1419615_at	transient receptor potential cation channel, subfamily V, member	Trpv6	0.83898	1.07674326	0.1062962
1416871_at	a disintegrin and metallopeptidase domain 8	Adam8	0.839	-1.08963866	-0.1377395
1449231_at	zinc finger protein 296	Zfp296	0.83904	-1.05979897	-0.0887343
1436977_at	---	---	0.83905	-1.07990266	-0.1111341
1440872_at	---	---	0.83906	-1.08677321	-0.1314232
1446943_at	---	---	0.83906	-1.08092326	-0.1154325
1447879_x_at	DALR anticodon binding domain containing 3 /// WD repeat doma	Dalrd3 /// Wdr6	0.83907	-1.05349197	-0.0755023
1435132_at	dispatched homolog 1 (Drosophila)	Disp1	0.83909	1.0908288	0.11824019
1435110_at	unc-5 homolog B (C. elegans)	Unc5b	0.83909	1.07657014	0.10188177
1459779_s_at	SET domain containing (lysine methyltransferase) 8	Setd8	0.83909	1.07240412	0.09694919
1435816_at	predicted gene 13654 /// ribosomal protein S6 pseudogene /// 4	Gm13654 /// Gm16409 //	0.83911	-1.05652479	-0.0823872
1445692_x_at	---	---	0.83912	-1.0929519	-0.174074
1422592_at	catenin (cadherin associated protein), delta 2	Ctnnd2	0.83913	-1.08384416	-0.1208017
1457465_at	shroom family member 4	Shroom4	0.83913	1.07899445	0.10367484
1448732_at	cathepsin B	Ctsb	0.83914	1.06155732	0.0838056
1449744_at	Solute carrier family 19 (sodium/hydrogen exchanger), member 1	Slc19a1	0.83914	1.08493839	0.11424836
1428502_at	ARP6 actin-related protein 6 homolog (yeast)	Actr6	0.83914	-1.06244508	-0.0904918
1435061_at	nudix (nucleoside diphosphate linked moiety X)-type motif 10	Nudt10	0.83915	-1.08804065	-0.13045
1454846_at	UTP15, U3 small nucleolar ribonucleoprotein, homolog (yeast)	Utp15	0.83915	-1.05835463	-0.0826399
1420082_at	DNA segment, Chr 2, ERATO Doi 750, expressed	D2Ertd750e	0.83915	-1.08060659	-0.1209675
1460645_at	cysteine and histidine-rich domain (CHORD)-containing, zinc-bindi	Chordc1	0.83915	-1.05373542	-0.0755986
1436594_at	zinc finger protein 719	Zfp719	0.83916	-1.05774482	-0.0845051
1428919_at	Fgfr1 oncogene partner	Fgfr1op	0.83917	1.06027605	0.08275327
1445460_at	---	---	0.83918	-1.07813645	-0.1176787
1423962_at	WD repeat domain 26	Wdr26	0.83919	-1.07716092	-0.1171624
1436011_at	engulfment and cell motility 2, ced-12 homolog (C. elegans)	Elmo2	0.83919	1.07670043	0.10206901
1430917_at	RIKEN cDNA 9130401L11 gene	9130401L11Rik	0.8392	-1.08690988	-0.12654
1420538_at	G protein-coupled receptor, family C, group 5, member D	Gprc5d	0.83921	-1.07645709	-0.1065318
1439337_at	target of myb1-like 1 (chicken)	Tom1l1	0.83921	-1.07427095	-0.1064291
1456183_at	oogenesin 4	Oog4	0.83922	-1.07015583	-0.0979284
1453952_at	RIKEN cDNA 4930572G02 gene	4930572G02Rik	0.83922	-1.0831673	-0.1238525
1435005_at	centromere protein E	Cenpe	0.83927	1.07502194	0.09802692
1416471_at	choline/ethanolaminephosphotransferase 1	Cept1	0.8393	1.05811599	0.08107609
1422661_at	lectin, galactose binding, soluble 8	Lgals8	0.83931	-1.08196465	-0.1191217
1459774_at	---	---	0.83932	-1.07810239	-0.108686
1426740_s_at	armadillo repeat containing 5	Armc5	0.83932	1.09130624	0.11382607
1420878_a_at	tyrosine 3-monooxygenase/tryptophan 5-monooxygenase activa	Ywhab	0.83933	-1.06025951	-0.089474
1445290_at	---	---	0.83934	-1.08487623	-0.120171
1457787_at	---	---	0.83934	-1.08988762	-0.1443157
1445437_at	coiled-coil domain containing 74A	Ccdc74a	0.83935	1.08443028	0.11325318
1435652_a_at	guanine nucleotide binding protein (G protein), alpha inhibiting 2	Gnai2	0.83936	-1.05172944	-0.0733916

1428964_at	solute carrier family 25 (mitochondrial carrier), member 18	Slc25a18	0.83936	-1.08218442	-0.1216814
1439547_at	RIKEN cDNA A930007A09 gene	A930007A09Rik	0.83936	-1.08347737	-0.1216951
1420338_at	arachidonate 15-lipoxygenase	Alox15	0.83936	-1.08503451	-0.1302139
1424444_a_at	RIKEN cDNA 1600014C10 gene	1600014C10Rik	0.83937	1.08288516	0.10709775
1425333_at	RAB43, member RAS oncogene family	Rab43	0.83937	1.07536552	0.10093263
1450615_at	eosinophil-associated, ribonuclease A family, member 4	Ear4	0.83937	1.0816824	0.11004288
1429967_at	RIKEN cDNA 2600002B07 gene	2600002B07Rik	0.83937	-1.06809641	-0.0952219
1434522_at	WD repeat domain 4	Wdr4	0.83937	1.07705029	0.10282023
1439096_at	D-aspartate oxidase	Ddo	0.83939	1.06337122	0.0861836
1451164_a_at	mitochondrial ribosomal protein S18B	Mrps18b	0.83939	1.05796059	0.07846933
1431849_at	RIKEN cDNA 4933432B09 gene	4933432B09Rik	0.83939	-1.09108705	-0.1414529
1444468_at	progestin and adipoQ receptor family member VIII	Paqr8	0.8394	1.11321188	0.13676339
1422342_at	neuromedin B receptor	Nmbr	0.8394	-1.08177818	-0.1170998
1458244_at	transmembrane and coiled-coil domains 5	Tmco5	0.83941	1.09938569	0.12405054
1446835_at	---	---	0.83941	1.10659781	0.13260951
1432329_a_at	megakaryocyte-associated tyrosine kinase	Matk	0.83941	-1.08658002	-0.1417426
1456572_x_at	chaperonin containing Tcp1, subunit 4 (delta)	Cct4	0.83941	-1.07419297	-0.1063095
1453411_at	COX assembly mitochondrial protein homolog (S. cerevisiae)	Cmc1	0.83942	1.0846133	0.11309825
1444423_at	---	---	0.83942	-1.0783835	-0.109108
1447911_at	---	---	0.83942	-1.08057907	-0.1167064
1452788_at	protein phosphatase 2, regulatory subunit B (B56), epsilon isoform	Ppp2r5e	0.83943	-1.06905489	-0.1034316
1436442_at	protein tyrosine phosphatase, non-receptor type 14	Ptpn14	0.83943	-1.07611801	-0.11179
1457440_at	somatostatin receptor 4	Sstr4	0.83944	-1.0807954	-0.1154312
1427109_at	open reading frame 61	ORF61	0.83944	1.06360632	0.08544742
1459894_at	IQ motif containing GTPase activating protein 2	Iqgap2	0.83944	1.08311455	0.11256987
1459432_at	---	---	0.83945	1.12177312	0.14255105
1433698_a_at	thioredoxin-like 4A	Txnl4a	0.83945	1.05337085	0.07471756
1439411_a_at	exportin 7	Xpo7	0.83945	-1.05149885	-0.0734807
1459542_at	---	---	0.83946	-1.07045028	-0.1078945
1418894_s_at	pre B-cell leukemia transcription factor 2	Pbx2	0.83946	-1.06617905	-0.0970305
1443033_at	RNA binding motif protein 14	Rbm14	0.83947	-1.08186739	-0.1202337
1421238_a_at	protein tyrosine phosphatase, receptor type, T	Ptprt	0.83947	1.07672255	0.10643452
1422525_at	ATP synthase, H+ transporting, mitochondrial F1F0 complex, subu	Atp5k	0.83947	1.05210129	0.07070971
1455390_at	alkB, alkylation repair homolog 6 (E. coli)	Alkbh6	0.83948	1.07407148	0.09681807
AFFX-TrpnX-M_a	---	---	0.83948	1.10426179	0.12747368
1457589_at	FAT tumor suppressor homolog 3 (Drosophila)	Fat3	0.83949	-1.0777983	-0.1125202
1427611_at	growth hormone releasing hormone receptor	Ghrhr	0.83949	-1.07557176	-0.1063656
1454092_a_at	general transcription factor IIH, polypeptide 3	Gtf2h3	0.83949	1.05807408	0.08019144
1446003_at	---	---	0.8395	1.07647741	0.10612811
1460349_at	proline rich 14	Prr14	0.8395	1.06575167	0.09093675
1425363_at	beta-1,4-N-acetyl-galactosaminyl transferase 1	B4galnt1	0.8395	1.09215209	0.12130087
1457290_at	---	---	0.8395	-1.07921792	-0.1203655
1428498_at	ring finger protein 219	Rnf219	0.8395	-1.07207017	-0.1027047
1429299_at	dimethylarginine dimethylaminohydrolase 1	Ddah1	0.83951	1.09142596	0.11768342
1417465_at	farnesyltransferase, CAAX box, alpha	Fnta	0.83951	1.05617267	0.07824966
1432549_s_at	RIKEN cDNA 1700112M01 gene	1700112M01Rik	0.83952	1.08453184	0.11410779
1442144_at	EMI domain containing 1	Emid1	0.83952	1.08422275	0.11362401
1431502_a_at	exosome component 3	Exosc3	0.83952	1.05652056	0.07754035
1452759_s_at	PTPRF interacting protein, binding protein 1 (liprin beta 1)	Ppfibp1	0.83953	-1.05985435	-0.0847586
1432423_a_at	RIKEN cDNA C530008M17 gene	C530008M17Rik	0.83953	1.08233806	0.11242823
1420589_at	hyaluronan synthase 3	Has3	0.83953	1.08294613	0.11406244
1435412_at	RIKEN cDNA 1700007E06 gene	1700007E06Rik	0.83954	1.11628132	0.13924513
1455933_at	---	---	0.83954	-1.0778819	-0.1112364
1431825_at	serine/arginine-rich protein specific kinase 3	Srpk3	0.83954	-1.08194256	-0.1217355
1436008_at	tumor protein D52	Tpd52	0.83954	1.09525812	0.12051017
1448695_at	protein kinase C, iota	Prkci	0.83955	1.09751244	0.11742862
1455820_x_at	scavenger receptor class B, member 1	Scarb1	0.83955	1.08781465	0.11064321
1423498_at	aldolase 1 A retrogene 2	Aldoart2	0.83955	1.0667303	0.0915731
1432437_at	transmembrane protein 217	Tmem217	0.83956	-1.08012497	-0.1116107
1425742_a_at	TSC22 domain family, member 1	Tsc22d1	0.83956	1.05253655	0.07025137
1418688_at	calcitonin receptor	Calcr	0.83956	1.18732534	0.187854
1430942_at	RIKEN cDNA 8430437O03 gene	8430437O03Rik	0.83956	-1.08634561	-0.1292002
1417144_at	tubulin, gamma 1	Tubg1	0.83958	1.05355491	0.07523438
1448775_at	interferon activated gene 203	Ifi203	0.83958	-1.08052821	-0.1201378
1456497_x_at	ribosomal protein S10	Rps10	0.83958	1.03151237	0.04366275
1455258_at	potassium voltage gated channel, Shaw-related subfamily, meml	Kcnc2	0.83958	-1.07326928	-0.1112888
1424387_at	prolactin family 8, subfamily a, member 81	Prl8a8	0.83958	1.07262789	0.10069112
1422700_at	arachidonate 12-lipoxygenase	Alox12	0.83959	1.09461384	0.12058043
1445957_at	---	---	0.8396	-1.08062147	-0.11331
1432254_at	RIKEN cDNA C030013D06 gene	C030013D06Rik	0.8396	1.08220534	0.11221919
1458635_at	RIKEN cDNA 4832428D23 gene	4832428D23Rik	0.8396	1.08299726	0.1148667
1456553_at	---	---	0.83961	1.1288544	0.151444
1456097_a_at	integrin beta 3 binding protein (beta3-endonexin)	Itgb3bp	0.83961	1.07415211	0.09872516
1417381_at	complement component 1, q subcomponent, alpha polypeptide	C1qa	0.83961	1.12296817	0.14746611
1440715_s_at	CDKN2A interacting protein N-terminal like	Cdkn2aipnl	0.83961	1.05765114	0.07921896
1447749_at	SET and MYND domain containing 5	Smyd5	0.83961	1.14741895	0.16306583
1455148_at	transmembrane protein 130	Tmem130	0.83961	1.08062844	0.11109971
1456246_x_at	trophoblast specific protein beta	Tpbpb	0.83961	1.08236362	0.11178675
1434228_at	pyruvate dehyrogenase phosphatase catalytic subunit 1	Pdp1	0.83962	1.08078746	0.10780362
1445055_at	---	---	0.83963	1.07772148	0.1018907
1453510_s_at	family with sequence similarity 184, member A	Fam184a	0.83963	1.08350631	0.11256503
1424046_at	budding uninhibited by benzimidazoles 1 homolog (S. cerevisiae)	Bub1	0.83964	-1.05381548	-0.0761672
1433610_at	expressed sequence AA986860	AA986860	0.83964	1.11358689	0.13540995
1445262_at	---	---	0.83964	1.07944948	0.10914039
1453689_at	Fanconi anemia, complementation group E	Fance	0.83965	1.06618982	0.09233418
1443623_a_at	---	---	0.83965	-1.07576748	-0.1082064
1422347_at	neuropeptide Y receptor Y6	Npy6r	0.83965	1.0993078	0.12411707
1458356_at	---	---	0.83965	-1.07895825	-0.1121334
1455167_at	cytochrome c oxidase, subunit VIIIc	Cox8c	0.83965	-1.06153443	-0.0865823
1441118_at	predicted gene 10003	Gm10003	0.83965	-1.07848098	-0.1107615
1435796_at	WSC domain containing 2	Wscd2	0.83965	1.08477739	0.11374842
1456416_at	core-binding factor, runt domain, alpha subunit 2, translocated to,	Cbfa2t2	0.83965	1.06533374	0.09059355
1433404_at	RIKEN cDNA 4930423O20 gene	4930423O20Rik	0.83965	-1.06845302	-0.0982487
1442664_at	---	---	0.83966	-1.09065618	-0.145716

1448884_at	general transcription factor II E, polypeptide 2 (beta subunit)	Gtf2e2	0.83966	1.0559365	0.07668412
1430555_s_at	leucine-rich repeats and immunoglobulin-like domains 3	Lrig3	0.83966	-1.07837989	-0.1310031
1457374_at	neural precursor cell expressed, developmentally down-regulated	Nedd4l	0.83966	-1.0854117	-0.1360101
1424014_at	RIKEN cDNA 2900092E17 gene	2900092E17Rik	0.83966	1.07809103	0.10179854
1430648_at	sodium channel, voltage-gated, type II, beta	Scn2b	0.83967	1.08596967	0.11662091
1442567_at	zinc finger and BTB domain containing 24	Zbtb24	0.83967	-1.08223303	-0.1177948
1420254_at	---	---	0.83967	-1.05855363	-0.0841704
1448952_at	RIKEN cDNA A030009H04 gene	A030009H04Rik	0.83967	-1.07858883	-0.1116044
1423219_a_at	mitochondrial ribosomal protein L49	Mrpl49	0.83968	1.06129412	0.08214615
1419818_x_at	ankyrin repeat and zinc finger domain containing 1	Ankzf1	0.83968	1.06156986	0.08538395
1428414_at	cyclin Y	Ccny	0.83968	1.21262553	0.20254807
1454108_at	RIKEN cDNA 4930524O08 gene	4930524O08Rik	0.83968	-1.07817875	-0.1189294
1446735_at	---	---	0.83969	1.08466206	0.11408006
1416659_at	eukaryotic translation initiation factor 3, subunit A	Eif3a	0.83969	-1.05385246	-0.0777647
1422546_at	interleukin enhancer binding factor 3	Ilf3	0.83969	-1.08236447	-0.1420438
1445877_at	Rieske (Fe-S) domain containing	Rfesd	0.83969	1.10077875	0.12369982
1458992_at	---	---	0.83969	-1.07591687	-0.1060475
1457883_at	peptidase M20 domain containing 2	Pm20d2	0.83969	-1.07670895	-0.1080668
1429420_at	RIKEN cDNA 4931414P19 gene	4931414P19Rik	0.83969	1.08190868	0.10907368
1443845_x_at	RIKEN cDNA 1700012P22 gene	1700012P22Rik	0.83969	1.09503827	0.11807404
1435442_at	DDB1 and CUL4 associated factor 13	Dcaf13	0.8397	1.05352193	0.07477218
1431091_at	pygopus 1	Pygo1	0.8397	1.0807497	0.11045211
1441727_s_at	zinc finger protein 467	Zfp467	0.8397	1.09175384	0.11766929
1419759_at	ATP-binding cassette, sub-family B (MDR/TAP), member 1A	Abcb1a	0.8397	-1.05977827	-0.083792
1439569_at	G protein-coupled receptor 83	Gpr83	0.8397	-1.08568275	-0.125749
1455251_at	integrin alpha 1	Itga1	0.8397	1.12340994	0.14612057
1453466_at	ribosomal protein S6	Rps6	0.83971	1.08400618	0.11149356
1437496_at	RIKEN cDNA 9330129D05 gene	9330129D05Rik	0.83971	1.08795799	0.11316915
1439669_at	RIKEN cDNA 6430571L13 gene	6430571L13Rik	0.83971	-1.08339063	-0.1232545
1435639_at	RIKEN cDNA 2610528A11 gene	2610528A11Rik	0.83971	1.07750247	0.10766259
1416243_a_at	predicted gene 10269 /// ribosomal protein L35	Gm10269 /// Rpl35	0.83971	1.03103883	0.04335517
1458814_at	expressed sequence AU022855	AU022855	0.83971	-1.08225453	-0.1163456
1453407_at	RIKEN cDNA 1700018L24 gene	1700018L24Rik	0.83971	-1.07977456	-0.1130426
1445542_at	---	---	0.83971	1.07471276	0.1027805
1452127_a_at	protein tyrosine phosphatase, non-receptor type 13	Ptpn13	0.83971	1.06779817	0.09417005
1427544_a_at	poly (A) polymerase alpha	Papola	0.83971	-1.06635926	-0.0953666
1423734_at	RAS-related C3 botulinum substrate 1	Rac1	0.83971	1.05047998	0.07042191
1433692_at	N-acetyltransferase 10	Nat10	0.83971	1.0548372	0.0763673
1440098_at	nuclear receptor coactivator 2 pseudogene /// hypothetical LOC1	D030018L15Rik /// LOC1(	0.83971	-1.09111094	-0.1655436
1444648_at	family with sequence similarity 178, member B	Fam178b	0.83972	-1.06965407	-0.0976738
1459145_at	RIKEN cDNA A930033H14 gene	A930033H14Rik	0.83972	1.08594985	0.11386441
1452805_at	DNA segment, Chr 11, Wayne State University 47, expressed	D11Wsu47e	0.83972	1.06282238	0.08646927
1426459_s_at	expressed sequence AW549877	AW549877	0.83972	-1.06540027	-0.0998226
1433484_at	DNA segment, Chr 6, ERATO Doi 527, expressed	D6Ertd527e	0.83972	1.08037121	0.10969759
1438784_at	B-cell leukemia/lymphoma 11B	Bcl11b	0.83972	-1.08434535	-0.1235985
1441510_at	hypothetical protein LOC100503148	LOC100503148	0.83972	-1.08091741	-0.1154001
1415885_at	chromogranin B	Chgb	0.83972	1.09831264	0.1240673
1455161_at	expressed sequence AI504432	AI504432	0.83972	1.09946071	0.12821238
1455710_x_at	mature T-cell proliferation 1	Mtcp1	0.83973	1.09001328	0.11674144
1433250_at	RIKEN cDNA 4931409D07 gene	4931409D07Rik	0.83973	-1.08596582	-0.1412502
1416158_at	nuclear receptor subfamily 2, group F, member 2	Nr2f2	0.83973	1.09447287	0.11638042
1444090_at	PML-RAR alpha-regulated adaptor molecule 1	Pram1	0.83973	-1.07640062	-0.1146997
1458583_at	RIKEN cDNA 1700123O12 gene	1700123O12Rik	0.83973	-1.07953189	-0.1174017
1430522_a_at	vesicle-associated membrane protein 5	Vamp5	0.83973	1.09280288	0.12225654
1452715_at	HAUS augmin-like complex, subunit 5	Haus5	0.83973	1.07154035	0.09689424
1446220_at	netrin 5	Ntn5	0.83974	1.08349014	0.1106404
1444107_at	RIKEN cDNA C130039O16 gene	C130039O16Rik	0.83974	-1.06091892	-0.0916609
1428090_at	pentatricopeptide repeat domain 3	Ptcd3	0.83974	1.06506614	0.08795345
1434683_at	---	---	0.83974	1.09455801	0.12324258
1430026_at	heat shock protein 70 family, member 13	Hspa13	0.83974	1.08975817	0.11806609
1458436_at	AU RNA binding protein/enoyl-coenzyme A hydratase	Auh	0.83974	-1.0790025	-0.1169473
1438818_at	NMD3 homolog (S. cerevisiae)	Nmd3	0.83974	-1.08536836	-0.1322621
1453841_at	RIKEN cDNA 2310050P20 gene	2310050P20Rik	0.83974	1.10040095	0.12166094
1422860_at	neurotensin	Nts	0.83974	-1.07755707	-0.112017
1417736_at	structural maintenance of chromosomes 6	Smc6	0.83975	-1.06882719	-0.1030216
1427932_s_at	RIKEN cDNA 1200003I10 gene /// RIKEN cDNA 1200015M12 gen	1200003I10Rik /// 12000	0.83975	-1.07995739	-0.1209393
1441115_at	DNA segment, Chr 18, ERATO Doi 232, expressed	D18Ertd232e	0.83975	-1.08076164	-0.1194092
1429043_at	survival motor neuron domain containing 1	Smndc1	0.83975	-1.05226988	-0.0746631
1443160_at	SET binding factor 2	Sbf2	0.83975	-1.08418911	-0.1381562
1460737_at	immunoglobulin (CD79A) binding protein 1	Igbp1	0.83975	1.05441095	0.07526386
1454780_at	UDP-N-acetyl-alpha-D-galactosamine:polypeptide N-acetylgalact	Galntl4	0.83975	1.08005588	0.109541
1428697_at	dipeptidylpeptidase 8	Dpp8	0.83975	1.09464539	0.11916382
1460366_at	echinoderm microtubule associated protein like 3	Eml3	0.83975	1.06710532	0.09289533
1431570_at	RIKEN cDNA 4930588G17 gene	4930588G17Rik	0.83976	-1.07825886	-0.1132263
1416697_at	dipeptidylpeptidase 4	Dpp4	0.83976	1.09133233	0.11656991
1428041_at	cDNA sequence BC028777	BC028777	0.83976	1.07276389	0.09862804
1435207_at	DIX domain containing 1	Dixdc1	0.83976	1.11116326	0.13308746
1448226_at	ribonucleotide reductase M2	Rrm2	0.83976	1.04582858	0.06428765
1434983_at	coiled-coil domain containing 108	Ccdc108	0.83976	-1.07856041	-0.1169261
1418971_x_at	B-cell leukemia/lymphoma 10	Bcl10	0.83976	1.06489703	0.08946049
1429686_at	polymerase (RNA) III (DNA directed) polypeptide F	Polr3f	0.83976	1.06303575	0.08773196
1451411_at	G protein-coupled receptor, family C, group 5, member B	Gprc5b	0.83976	1.0946027	0.1229381
1432922_at	RIKEN cDNA 4933416E14 gene	4933416E14Rik	0.83976	1.0722647	0.09829393
1431173_at	family with sequence similarity 53, member B	Fam53b	0.83977	1.13817515	0.1557965
1428020_at	ribosome production factor 1 homolog (S. cerevisiae)	Rpf1	0.83977	-1.07797887	-0.1119873
1430839_at	RIKEN cDNA 9430076G02 gene	9430076G02Rik	0.83977	-1.07985194	-0.1160143
1451755_a_at	apolipoprotein B mRNA editing enzyme, catalytic polypeptide 1	Apobec1	0.83977	-1.06844192	-0.1016216
1416999_at	sphingomyelin phosphodiesterase 2, neutral	Smpd2	0.83977	1.05947235	0.08299995
1426511_at	sushi domain containing 2	Susd2	0.83978	1.06695893	0.09262189
1454554_at	RIKEN cDNA 9330159N22 gene	9330159N22Rik	0.83978	1.06592093	0.09000075
1433069_at	---	---	0.83978	1.10124542	0.12669847
1418848_at	aquaporin 7	Aqp7	0.83978	-1.09072157	-0.13764
1459254_at	cell division cycle associated 2	Cdca2	0.83978	-1.07665092	-0.1084844
1421162_a_at	nuclear factor I/A	Nfia	0.83978	1.09930399	0.12888869

1430621_at	multimerin 1	Mmrn1	0.83978	1.05375228	0.07446085
1426573_at	NAD-dependent malic enzyme, mitochondrial-like /// malic enzym	LOC100505264 /// Me2	0.83978	-1.07516891	-0.1214385
1448262_at	proteasome (prosome, macropain) subunit, beta type 2	Psmb2	0.83978	1.06702836	0.08797323
1436843_at	RIKEN cDNA C430048L16 gene	C430048L16Rik	0.83979	-1.07508787	-0.1107184
1441146_at	expressed sequence C76132	C76132	0.83979	-1.0805763	-0.1160116
1456000_at	DEAD (Asp-Glu-Ala-Asp) box polypeptide 18	Ddx18	0.83979	-1.07844664	-0.1122325
1430052_at	RIKEN cDNA C030048H21 gene	C030048H21Rik	0.83979	-1.08720528	-0.1511702
1446962_at	---	---	0.8398	-1.07341886	-0.1080178
1452957_at	keratin associated protein 3-3	Krtap3-3	0.8398	1.08593787	0.11288862
1424611_x_at	TruB pseudouridine (psi) synthase homolog 2 (E. coli)	Trub2	0.8398	-1.06484513	-0.0935007
1454031_at	RIKEN cDNA 2310029O18 gene	2310029O18Rik	0.8398	1.0779754	0.10826963
1459982_a_at	aquaporin 6	Aqp6	0.8398	-1.08492945	-0.1247484
1424968_at	carboxylesterase 2G	Ces2g	0.83981	1.08773293	0.11454719
1435514_at	leucine zipper transcription factor-like 1	Lztfl1	0.83981	1.07077907	0.09341507
1454747_a_at	kelch domain containing 3	Klhdc3	0.83981	1.0652215	0.08640959
1423564_a_at	phosphoribosylaminoimidazole carboxylase, phosphoribosylamin	Paics	0.83981	-1.03583435	-0.0510081
1450275_x_at	ubiquitin-conjugating enzyme E2F (putative)	Ube2f	0.83981	1.05498427	0.07689597
1422044_at	N-deacetylase/N-sulfotransferase (heparan glucosaminyl) 1	Ndst1	0.83982	1.08500943	0.11459751
1453164_a_at	phosphatidylserine synthase 2	Ptdss2	0.83982	1.06636014	0.08988284
1417729_at	myosin, heavy polypeptide 6, cardiac muscle, alpha	Myh6	0.83982	1.07032016	0.09282447
1422227_at	Kruppel-like factor 12	Klf12	0.83982	-1.07924769	-0.112894
1419716_a_at	POU domain, class 2, transcription factor 1	Pou2f1	0.83982	-1.06193913	-0.0904184
1435365_at	RIKEN cDNA 4732415M23 gene	4732415M23Rik	0.83982	1.07732608	0.10680959
1451435_at	cut-like homeobox 1	Cux1	0.83983	1.07097472	0.0982085
1430635_at	melan-A	Mlana	0.83983	1.10934641	0.13348474
1443627_at	---	---	0.83983	1.08448221	0.11511473
1451906_at	UBX domain protein 10	Ubxn10	0.83983	1.11556588	0.1394309
1458907_at	---	---	0.83983	1.12726877	0.14746558
1430811_a_at	NUF2, NDC80 kinetochore complex component, homolog (S. cerev	Nuf2	0.83983	-1.06365279	-0.0949424
1433046_at	RIKEN cDNA 4930535F04 gene	4930535F04Rik	0.83984	-1.07390594	-0.1051454
1428817_at	MON2 homolog (yeast)	Mon2	0.83984	1.06086137	0.08206811
1424166_at	mutS homolog 3 (E. coli)	Msh3	0.83985	1.06696714	0.08971628
1415912_a_at	ribosomal protein S13	Rps13	0.83985	1.03777875	0.05144323
1434145_s_at	serine hydrolase-like	Serhl	0.83985	-1.07574321	-0.1061976
1447433_at	WD repeat and FYVE domain containing 3	Wdfy3	0.83986	1.09342275	0.12048134
1424443_at	transmembrane 6 superfamily member 1	Tm6sf1	0.83986	-1.0774779	-0.1096667
1437887_at	sterile alpha motif domain containing 5	Samd5	0.83987	1.08343543	0.11290578
1415964_at	stearoyl-Coenzyme A desaturase 1	Scd1	0.83987	1.06414242	0.08564663
1450750_a_at	nuclear receptor subfamily 4, group A, member 2	Nr4a2	0.83987	-1.08616224	-0.1381548
1432194_at	RIKEN cDNA 9530020I12 gene	9530020I12Rik	0.83988	-1.08511768	-0.1394274
1442540_at	expressed sequence C77609	C77609	0.83988	-1.08201267	-0.1195951
1455285_at	solute carrier family 31, member 1	Slc31a1	0.83988	-1.08308773	-0.1445474
1439743_at	---	---	0.83988	-1.0833774	-0.1206444
1440450_at	predicted gene 10501	Gm10501	0.83988	1.12398158	0.14505143
1457025_at	short chain dehydrogenase/reductase family 16C, member 6	Sdr16c6	0.83988	1.06816015	0.09227277
1441354_at	expressed sequence AU018740	AU018740	0.83988	-1.0887831	-0.1352122
1457041_at	small Cajal body-specific RNA 1	Scarna13	0.83991	1.10534104	0.12769155
1420136_a_at	---	---	0.83991	-1.07567368	-0.1054712
1444785_at	---	---	0.83991	1.0739209	0.10154604
1435051_at	WD repeat domain 44	Wdr44	0.83991	-1.06675967	-0.09626
1444706_at	---	---	0.83992	1.12200163	0.14131936
1438381_x_at	DEAD (Asp-Glu-Ala-Asp) box polypeptide 47	Ddx47	0.83992	1.07556573	0.10455836
1419036_at	casein kinase 2, alpha 1 polypeptide	Csnk2a1	0.83992	-1.05362045	-0.0765268
1431814_at	RIKEN cDNA 4932443L11 gene	4932443L11Rik	0.83993	1.10412982	0.13011289
1444743_at	---	---	0.83993	-1.0665897	-0.0976127
1448222_x_at	cytochrome c oxidase, subunit VIIIa	Cox8a	0.83993	1.05896412	0.07781995
1421836_at	microtubule-associated protein 7	Mtap7	0.83993	-1.06705931	-0.0984673
1434225_at	SWA-70 protein	Swap70	0.83993	1.06729114	0.09135752
1459977_x_at	COX10 homolog, cytochrome c oxidase assembly protein, heme A	Cox10	0.83994	-1.08626064	-0.1378919
1450176_at	endoplasmic reticulum (ER) to nucleus signalling 1	Ern1	0.83994	-1.0819087	-0.1154516
1444803_at	---	---	0.83994	-1.06493233	-0.0928432
1431924_at	RIKEN cDNA 4930432N10 gene	4930432N10Rik	0.83994	-1.08190754	-0.1227316
1452331_s_at	glutamine and serine rich 1	Qser1	0.83994	-1.06769129	-0.098224
1446090_at	---	---	0.83994	-1.07805975	-0.1154525
1440367_at	---	---	0.83995	-1.08607029	-0.1493823
1446995_at	Cytochrome P450, family 7, subfamily b, polypeptide 1	Cyp7b1	0.83995	1.06326219	0.08840121
1418156_at	potassium voltage-gated channel, Isk-related subfamily, gene 4	Kcne4	0.83995	-1.08200083	-0.1229833
1429547_at	RIKEN cDNA 4930578I06 gene	4930578I06Rik	0.83995	-1.07601484	-0.1075801
1424198_at	discs, large homolog 5 (Drosophila)	Dlg5	0.83996	1.06092711	0.08437215
1442548_at	---	---	0.83997	1.0867191	0.11633653
1426260_a_at	UDP glucuronosyltransferase 1 family, polypeptide A1 /// UDP gl	Ugt1a1 /// Ugt1a10 /// U	0.83997	1.17926502	0.18212255
1421545_a_at	synaptic nuclear envelope 1	Syne1	0.83997	1.08904847	0.11672388
1426810_at	lysine (K)-specific demethylase 3A	Kdm3a	0.83997	1.05438666	0.07595949
1426430_at	jagged 2	Jag2	0.83997	1.07136352	0.09705007
1456735_x_at	acid phosphatase-like 2	Acpl2	0.83998	1.14109518	0.15654399
1439992_at	---	---	0.83998	-1.06878388	-0.1022416
1454644_at	family with sequence similarity 40, member A	Fam40a	0.83998	1.06607552	0.08717472
1452808_at	snurportin 1	Snupn	0.83999	1.05737916	0.08018181
1423329_at	ganglioside-induced differentiation-associated-protein 1	Gdap1	0.83999	1.0762744	0.1057789
1444372_at	---	---	0.83999	-1.07872416	-0.1149723
1442296_at	WD repeat domain 75	Wdr75	0.83999	1.17333344	0.18079734
1453020_at	RIKEN cDNA 1810048J11 gene	1810048J11Rik	0.83999	1.08386826	0.10816339
1438448_at	otopetrin 1	Otop1	0.83999	1.07305246	0.09936794
1438212_at	ubiquitin specific peptidase 37	Usp37	0.83999	-1.06467111	-0.0907916
1452974_at	nucleolar protein 8	Nol8	0.83999	-1.06056394	-0.0889106
1456656_at	lin-7 homolog A (C. elegans)	Lin7a	0.83999	-1.07321778	-0.1039637
1458094_at	---	---	0.84	-1.08946126	-0.1507777
1416039_x_at	cysteine rich protein 61	Cyr61	0.84	-1.0783387	-0.1154058
1426382_at	protein phosphatase 1B, magnesium dependent, beta isoform	Ppm1b	0.84	-1.06034002	-0.0921612
1419421_at	ankyrin 1, erythroid	Ank1	0.84	1.08046585	0.10660977
1453053_at	centromere protein W	Cenpw	0.84001	1.07519682	0.10009876
1454261_at	RIKEN cDNA E130304I02 gene	E130304I02Rik	0.84001	-1.07700488	-0.1097597
1442423_at	Neurexin III	Nrxn3	0.84002	1.07593466	0.10415309
1416224_at	zinc finger and BTB domain containing 17	Zbtb17	0.84002	1.06325296	0.08775923

1447778_x_at	BRCA1/BRCA2-containing complex, subunit 3	Brcc3	0.84002	-1.0707884	-0.1094613
1449684_at	3-oxoacyl-ACP synthase, mitochondrial	Oxsm	0.84003	-1.07200796	-0.1046694
1448297_a_at	tyrosine kinase, non-receptor, 2	Tnk2	0.84004	-1.05696315	-0.0805136
1449734_s_at	Bardet-Biedl syndrome 4 (human)	Bbs4	0.84004	1.07573597	0.0999432
1433236_at	RIKEN cDNA 3830422I06 gene	3830422I06Rik	0.84004	-1.07642175	-0.1081027
1425106_a_at	tryptophanyl-tRNA synthetase	Wars	0.84005	-1.06310903	-0.0927712
1457988_at	---	---	0.84005	-1.08141798	-0.1152634
1453706_at	RIKEN cDNA 2900042A17 gene	2900042A17Rik	0.84006	1.08178811	0.11259192
1425401_at	cleavage and polyadenylation specific factor 4-like	Cpsf4l	0.84007	-1.07950115	-0.1133116
1436587_at	WD repeat domain 38	Wdr38	0.84007	-1.0769134	-0.1090727
1424202_at	SEH1-like (S. cerevisiae	Seh1l	0.84012	-1.05494631	-0.0771807
1421184_a_at	cDNA sequence AB099516 /// predicted gene 10035 /// HIG1 do	AB099516 /// Gm10035 /	0.84015	1.07688694	0.1031053
1453687_at	solute carrier family 4 (anion exchanger), member 3	Slc4a3	0.84017	-1.07954276	-0.1140241
1459356_at	---	---	0.84018	-1.07973899	-0.1146556
1449711_at	ATPase, H+ transporting, lysosomal V1 subunit E1	Atp6v1e1	0.84018	1.08255482	0.11294603
1450902_at	bromodomain containing 3	Brd3	0.8402	1.096577	0.11548152
1445369_at	RIKEN cDNA B230359F08 gene	B230359F08Rik	0.84021	1.078867	0.10795201
1424430_at	MTERF domain containing 2	Mterfd2	0.84023	1.0604379	0.0825952
1437258_at	small proline-rich protein 2A1 /// small proline-rich protein 2A2	Sprr2a1 /// Sprr2a2	0.84023	1.06691236	0.09288067
1448726_at	small nuclear RNA activating complex, polypeptide 2	Snapc2	0.84028	1.06390771	0.08837768
1418571_at	tumor necrosis factor receptor superfamily, member 12a	Tnfrsf12a	0.84028	1.07371158	0.0984895
1423612_at	CLP1, cleavage and polyadenylation factor I subunit, homolog (S.	Clp1	0.84028	1.05927955	0.07992262
1421337_at	E74-like factor 4 (ets domain transcription factor)	Elf4	0.84028	1.09248053	0.12019508
1435375_at	family with sequence similarity 105, member A	Fam105a	0.8403	-1.07876477	-0.1106924
1445355_at	spermatogenesis associated 6	Spata6	0.84031	1.06038994	0.08278201
1428698_at	dipeptidylpeptidase 8	Dpp8	0.84033	-1.07763618	-0.1094508
1450505_a_at	family with sequence similarity 134, member B	Fam134b	0.84037	1.1030428	0.1281098
1432994_at	RIKEN cDNA 2900057E15 gene	2900057E15Rik	0.84037	1.06154493	0.08344626
1447799_x_at	RIKEN cDNA 1700001P01 gene	1700001P01Rik	0.84038	-1.07857563	-0.1097203
1446313_at	zinc finger protein 30	Zfp30	0.84039	-1.08350885	-0.1302181
1458431_at	eukaryotic translation initiation factor 4, gamma 2	Eif4g2	0.84039	-1.0867163	-0.1301417
1451036_at	spastic paraplegia 21 homolog (human)	Spg21	0.8404	1.0790691	0.09953159
1450369_at	folliculogenesis specific basic helix-loop-helix	Figla	0.8404	1.07965415	0.1089727
1436192_at	ADP-ribosylation factor guanine nucleotide-exchange factor 2 (bre	Arfgef2	0.84041	1.06625691	0.09029402
1449346_s_at	RIO kinase 1 (yeast)	Riok1	0.84042	-1.05715038	-0.0811407
1434430_s_at	adenosine A2b receptor	Adora2b	0.84043	1.08539061	0.11229554
1436127_at	corticotropin releasing hormone binding protein	Crhbp	0.84043	1.07761019	0.10387597
1456887_at	chemokine-like receptor 1	Cmklr1	0.84043	1.08250621	0.10814594
1448679_at	hyaluronoglucosaminidase 2	Hyal2	0.84044	1.06144391	0.08533924
1440292_at	DNA segment, Chr 13, ERATO Doi 666, expressed	D13Ertd666e	0.84044	-1.08278593	-0.124292
1416993_at	component of oligomeric golgi complex 4	Cog4	0.84044	1.05950466	0.08123675
1446158_at	---	---	0.84044	-1.07683619	-0.1101772
1428605_at	coiled-coil domain containing 124	Ccdc124	0.84045	1.0752103	0.09600858
1457224_at	hypothetical LOC100503647	LOC100503647	0.84045	-1.08668619	-0.1392821
1449025_at	interferon-induced protein with tetratricopeptide repeats 3	Ifit3	0.84045	1.10222598	0.12800773
1438255_at	forkhead box N3	Foxn3	0.84045	1.09297514	0.11514204
1456652_at	denticleless homolog (Drosophila)	Dtl	0.84046	-1.08364845	-0.1415274
1434872_x_at	60S ribosomal protein L37-like /// ribosomal protein L37	LOC100502825 /// Rpl37	0.84046	-1.04406148	-0.069643
1459201_at	---	---	0.84047	-1.07727323	-0.1114134
1420808_at	predicted gene 6768 /// nuclear receptor coactivator 4	Gm6768 /// Ncoa4	0.84048	-1.05012722	-0.0706171
1427765_a_at	T-cell receptor beta, joining region	Tcrb-J	0.84048	1.09384884	0.11968204
1428392_at	Ras association (RalGDS/AF-6) domain family member 2	Rassf2	0.84048	1.09843682	0.12580901
1417512_at	ecotropic viral integration site 5	Evi5	0.84048	-1.0591955	-0.0832362
1451439_at	cDNA sequence BC027231	BC027231	0.84048	1.0570922	0.07953952
1458866_at	transmembrane protein 215	Tmem215	0.84049	-1.06930221	-0.1005985
1445605_s_at	family with sequence similarity 135, member A	Fam135a	0.84049	-1.07453361	-0.104889
1430874_at	RIKEN cDNA 9430085M18 gene	9430085M18Rik	0.84049	-1.07683318	-0.1075939
1447404_at	RIKEN cDNA 8030462N17 gene	8030462N17Rik	0.8405	-1.08477087	-0.1321547
1441279_at	RIKEN cDNA C430002E04 gene	C430002E04Rik	0.8405	-1.06609137	-0.094086
1446678_at	DNA segment, Chr 14, ERATO Doi 16, expressed	D14Ertd16e	0.8405	1.0787047	0.10670796
1425091_at	retinoic acid receptor responder (tazarotene induced) 2	Rarres2	0.84051	-1.07864427	-0.1142785
1416921_x_at	aldolase A, fructose-bisphosphate	Aldoa	0.84051	-1.0321186	-0.0473187
1439247_at	dedicator of cytokinesis 10	Dock10	0.84051	1.07281934	0.0972482
1425673_at	LIM domain containing preferred translocation partner in lipoma	Lpp	0.84052	-1.07278762	-0.1032163
1445256_at	---	---	0.84052	-1.06711512	-0.0991749
1442882_at	---	---	0.84052	-1.07978901	-0.1144426
1425785_a_at	TXK tyrosine kinase	Txk	0.84052	1.0929935	0.12051866
1436136_at	nucleolar complex associated 2 homolog (S. cerevisiae)	Noc2l	0.84052	1.07867563	0.10270141
1458306_at	phosphatidylinositol glycan anchor biosynthesis, class Z	Pigz	0.84053	-1.07348431	-0.1034795
1416903_at	nucleobindin 1	Nucb1	0.84053	1.05914029	0.08283595
1434939_at	forkhead box F1a	Foxf1a	0.84053	-1.08031429	-0.1186412
1446305_at	---	---	0.84053	-1.08522826	-0.1271523
1440603_at	---	---	0.84054	1.09059403	0.12012236
1426908_at	UDP-N-acetyl-alpha-D-galactosamine: polypeptide N-acetylgalac	Galnt7	0.84054	1.06090372	0.08528909
1422105_at	CD3 antigen, epsilon polypeptide	Cd3e	0.84054	-1.07098278	-0.1022252
1452735_at	PEST proteolytic signal containing nuclear protein	Pcnp	0.84054	-1.06275307	-0.0978912
1438836_at	elongation factor Tu GTP binding domain containing 2	Eftud2	0.84054	-1.08033181	-0.1200703
1416342_at	tenascin C	Tnc	0.84054	1.08972197	0.11860738
1421347_at	ferritin mitochondrial	Ftmt	0.84054	-1.07778073	-0.1115138
1458978_at	---	---	0.84054	1.09837664	0.12607889
1418892_at	ras homolog gene family, member J	Rhoj	0.84054	-1.07570568	-0.10604
1444217_at	mitochondrial ribosomal protein L38	Mrpl38	0.84055	1.06987513	0.09636771
1422641_at	docking protein 5	Dok5	0.84055	1.10183634	0.1274362
1446182_at	---	---	0.84055	1.11518265	0.1399129
1457462_at	zinc finger protein 68	Zfp68	0.84055	1.10019531	0.12460351
1422902_s_at	meningioma expressed antigen 5 (hyaluronidase)	Mgea5	0.84056	1.07400481	0.09526221
1437317_at	ubiquitin-like modifier activating enzyme 7	Uba7	0.84056	-1.06541862	-0.0928993
1428531_at	integrator complex subunit 7	Ints7	0.84057	1.0586494	0.08019186
1457333_at	sidekick homolog 2 (chicken)	Sdk2	0.84057	-1.08488295	-0.1245539
1422708_at	phosphoinositide-3-kinase, catalytic, gamma polypeptide	Pik3cg	0.84058	-1.07851936	-0.114748
1435342_at	potassium inwardly-rectifying channel, subfamily K, member 6	Kcnk6	0.84058	1.0704338	0.09815122
1423589_at	actin related protein 2/3 complex, subunit 4	Arpc4	0.84058	-1.05385366	-0.076935
1441256_at	---	---	0.84059	-1.0697036	-0.0984805
1432728_at	RIKEN cDNA 5830433D23 gene	5830433D23Rik	0.84059	-1.0756139	-0.1061449

1422543_at	ropporin, rhophilin associated protein 1	Ropn1	0.84059	-1.07683568	-0.1092963
1419305_a_at	F-box protein 36	Fbxo36	0.84059	1.0668382	0.09105114
1451990_at	microtubule-associated protein, RP/EB family, member 2	Mapre2	0.84059	1.07755383	0.10702814
1420616_at	ash2 (absent, small, or homeotic)-like (Drosophila)	Ash2l	0.8406	1.04670691	0.06573034
1444570_at	---	---	0.8406	-1.07970839	-0.1136869
1441572_at	deleted in colorectal carcinoma	Dcc	0.8406	-1.08754958	-0.1520547
1453909_at	RIKEN cDNA 4930505N22 gene	4930505N22Rik	0.8406	-1.08090141	-0.1165443
1416411_at	glutathione S-transferase, mu 2	Gstm2	0.8406	1.07828765	0.103021
1426100_a_at	thymidine kinase 2, mitochondrial	Tk2	0.84061	1.08004809	0.10593574
1416171_at	RIKEN cDNA 2310037I24 gene	2310037I24Rik	0.84061	1.05143861	0.07183972
1429941_at	solute carrier family 10 (sodium/bile acid cotransporter family), m	Slc10a7	0.84061	1.08755389	0.11188196
1439905_at	RIKEN cDNA A330062J17 gene	A330062J17Rik	0.84062	1.07834254	0.10710616
1427905_at	RIKEN cDNA 1810063B07 gene	1810063B07Rik	0.84062	1.07207146	0.09576952
1415757_at	golgi-specific brefeldin A-resistance factor 1	Gbf1	0.84063	1.05973464	0.08217866
1420174_s_at	Tax1 (human T-cell leukemia virus type I) binding protein 1	Tax1bp1	0.84063	-1.07392172	-0.1247499
1421497_at	glycoprotein hormone alpha 2	Gpha2	0.84063	1.0705101	0.09612904
1428293_at	biorientation of chromosomes in cell division 1	Bod1	0.84063	1.05368704	0.07483397
1438455_at	poly(A) binding protein, cytoplasmic 4-like	Pabpc4l	0.84063	-1.08126649	-0.1250833
1459210_at	transmembrane protein 108	Tmem108	0.84064	-1.08282183	-0.1202908
1436540_at	microRNA let7d	Mirlet7d	0.84064	1.08332126	0.11018069
1443581_at	Beta galactoside alpha 2,6 sialyltransferase 1	St6gal1	0.84064	1.08227367	0.1133646
1449297_at	caspase 12	Casp12	0.84065	1.09343124	0.11642227
1416985_at	signal-regulatory protein alpha	Sirpa	0.84065	1.0950387	0.12167299
1431045_at	family with sequence similarity 49, member A	Fam49a	0.84066	-1.08663926	-0.1366769
1435104_at	zinc finger, DHHC domain containing 21	Zdhhc21	0.84066	-1.06863077	-0.1003052
1454773_at	retinoid X receptor alpha	Rxra	0.84066	1.07320617	0.09991102
1453046_at	RIKEN cDNA 1700110I01 gene	1700110I01Rik	0.84067	-1.06581631	-0.0936656
1432255_at	microtubule associated serine/threonine kinase 1	Mast1	0.8407	1.08249888	0.11406525
1431948_a_at	pantothenate kinase 2	Pank2	0.8407	1.05336114	0.07423452
1443858_at	RIKEN cDNA 9230105E10 gene	9230105E10Rik	0.84072	1.08854336	0.116479
1423807_a_at	calmodulin 2	Calm2	0.84072	-1.03603127	-0.051341
1454713_s_at	histidine decarboxylase	Hdc	0.84072	-1.0619986	-0.0888544
1448482_at	solute carrier family 39 (metal ion transporter), member 8	Slc39a8	0.84073	-1.07115249	-0.1019191
1449283_a_at	mitogen-activated protein kinase 12	Mapk12	0.84074	1.07238854	0.09724913
1427506_at	peptidylprolyl isomerase (cyclophilin) like 5	Ppil5	0.84074	1.06871614	0.09436398
1446666_at	a disintegrin-like and metallopeptidase (reprolysin type) with thro	Adamts12	0.84074	-1.06190369	-0.091975
1456636_at	RIKEN cDNA 4930414L22 gene	4930414L22Rik	0.84074	-1.08456188	-0.1339007
1446814_at	---	---	0.84076	-1.06672561	-0.0954934
1421246_at	paired box gene 9	Pax9	0.84076	1.08461951	0.11171618
1460263_at	Programmed cell death 6 interacting protein	Pdcd6ip	0.84077	-1.08298375	-0.1247499
1446366_at	transient receptor potential cation channel, subfamily C, member	Trpc4	0.84077	1.09648274	0.12280049
1435778_at	ankyrin repeat domain 11	Ankrd11	0.84077	-1.05356317	-0.0767171
1420253_at	DNA segment, Chr 2, ERATO Doi 63, expressed	D2Ertd63e	0.84082	-1.07849769	-0.1162307
1441048_at	RIKEN cDNA 5930430L01 gene	5930430L01Rik	0.84083	-1.07114634	-0.1044114
1432062_at	MAM domain containing glycosylphosphatidylinositol anchor 1	Mdga1	0.84087	1.06830341	0.09521589
1431206_at	RIKEN cDNA 5730601F06 gene	5730601F06Rik	0.84088	1.08687232	0.11170712
1433816_at	mitochondrial carrier triple repeat 1	Mcart1	0.84088	1.07355169	0.09574074
1426002_a_at	cell division cycle 7 (S. cerevisiae)	Cdc7	0.84088	1.0571909	0.08017999
1439533_at	RIKEN cDNA 1700125D06 gene	1700125D06Rik	0.84091	1.08044741	0.1066723
1442154_at	zinc finger (CCCH type), RNA binding motif and serine/arginine ricl	Zrsr2	0.84092	-1.07457276	-0.1078423
1422863_s_at	PDZ and LIM domain 5	Pdlim5	0.84093	-1.07130114	-0.1099671
1437659_at	amyotrophic lateral sclerosis 2 (juvenile) chromosome region, car	Als2cr11	0.84094	-1.07654251	-0.120766
1423605_a_at	transformed mouse 3T3 cell double minute 2	Mdm2	0.84096	1.06608645	0.08762573
1439755_at	signal-induced proliferation-associated 1 like 1	Sipa1l1	0.84097	1.092387	0.11738605
1457388_at	RIKEN cDNA A830080L01 gene	A830080L01Rik	0.841	1.1302684	0.14816448
1451547_at	iodotyrosine deiodinase	Iyd	0.84105	-1.07146642	-0.1008427
1430645_at	CD200 receptor 3	Cd200r3	0.84105	-1.06847947	-0.0988252
1439681_at	---	---	0.84106	1.05904568	0.08084979
1457392_at	---	---	0.84106	-1.07335238	-0.1087297
1440786_x_at	Sideroflexin 2	Sfxn2	0.84106	1.06486007	0.08741412
1428375_at	RIKEN cDNA 4932415G12 gene	4932415G12Rik	0.84108	1.09091336	0.11763762
1431075_a_at	eukaryotic translation initiation factor 4E nuclear import factor 1	Eif4enif1	0.84108	1.06713	0.08795212
1445864_at	---	---	0.84109	1.10105614	0.12731148
1437494_at	mitogen-activated protein kinase-activated protein kinase 3	Mapkapk3	0.8411	1.05847847	0.07919669
1431466_at	translocase of outer mitochondrial membrane 20 homolog (yeast	Tomm20l	0.8411	1.09986512	0.12516754
1425982_a_at	Werner syndrome homolog (human)	Wrn	0.8411	-1.07913108	-0.1113371
1457211_at	glutaminase	Gls	0.8411	1.08898725	0.11317837
1433529_at	peptidase domain containing associated with muscle regeneratio	Pamr1	0.8411	1.09618483	0.12353504
1428636_at	six transmembrane epithelial antigen of prostate 2	Steap2	0.84111	-1.06060001	-0.0861072
1438030_at	RAS, guanyl releasing protein 3	Rasgrp3	0.84111	1.08460043	0.11169183
1443559_at	Arginine/serine-rich coiled-coil 2	Rsrc2	0.84111	1.07068414	0.09810779
1427657_at	coiled-coil domain containing 134	Ccdc134	0.84113	-1.08239959	-0.1237248
1451276_at	UHRF1 (ICBP90) binding protein 1-like	Uhrf1bp1l	0.84113	-1.06005928	-0.0868976
1448798_at	EPS8-like 3	Eps8l3	0.84113	-1.08390786	-0.1197187
1427743_at	---	---	0.84113	1.09546318	0.12157374
1419906_at	Hydroxyprostaglandin dehydrogenase 15 (NAD)	Hpgd	0.84114	1.12043993	0.1405774
1431617_at	RIKEN cDNA 4933405E24 gene	4933405E24Rik	0.84114	1.08827733	0.11505714
1444820_at	---	---	0.84114	-1.07709076	-0.1115234
1421637_at	solute carrier family 5, member 4a	Slc5a4a	0.84114	-1.08370164	-0.1225059
1438631_x_at	tetratricopeptide repeat domain 13	Ttc13	0.84115	-1.06585121	-0.0983333
1451728_at	WD repeat domain 13	Wdr13	0.84115	1.07232315	0.09752977
1429346_a_at	centrosomal protein 72	Cep72	0.84115	1.06899034	0.09584676
1423305_at	exostoses (multiple)-like 1	Extl1	0.84115	1.07533272	0.10112372
1457640_x_at	phosphatidylinositol glycan anchor biosynthesis, class S	Pigs	0.84115	1.08279884	0.11216318
1438396_at	oculocerebrorenal syndrome of Lowe	Ocrl	0.84115	-1.06988527	-0.0988693
1428478_at	receptor accessory protein 4	Reep4	0.84115	-1.0509499	-0.0719969
1423368_at	lysosomal-associated protein transmembrane 4A	Laptm4a	0.84116	1.05103324	0.07144941
1437601_at	orthodenticle homolog 1 (Drosophila)	Otx1	0.84116	1.08052719	0.10773993
1437291_at	RIKEN cDNA 2700081O15 gene	2700081O15Rik	0.84116	1.07668452	0.10376451
1435774_at	hypothetical LOC106740	LOC106740	0.84116	-1.07004289	-0.098284
1432839_at	RIKEN cDNA 6430598H11 gene	6430598H11Rik	0.84117	-1.07174066	-0.1005867
1458565_at	ADP-ribosylation factor-like 2	Arl2	0.84117	-1.08123531	-0.1153364
1446334_at	---	---	0.84117	1.07945124	0.10601221
1455132_at	RAS protein activator like 3	Rasal3	0.84117	1.14181882	0.156169

1457675_at	RIKEN cDNA 2510002D24 gene	2510002D24Rik	0.84117	1.06831477	0.09529979
1425132_at	neuropilin (NRP) and tolloid (TLL)-like 1	Neto1	0.84117	-1.06728841	-0.0950091
1421917_at	platelet derived growth factor receptor, alpha polypeptide	Pdgfra	0.84118	1.09065225	0.11788819
1445147_at	proteasome (prosome, macropain) activator subunit 4	Psme4	0.84118	-1.08122766	-0.1230185
1436286_at	RIKEN cDNA B830017H08 gene	B830017H08Rik	0.84119	1.14559267	0.1619918
1448441_at	CDC28 protein kinase 1b	Cks1b	0.84119	1.04828588	0.0678721
1415974_at	mitogen-activated protein kinase kinase 2	Map2k2	0.84119	1.06653086	0.08730813
1456859_at	limb and neural patterns	Lnp	0.84119	-1.08041938	-0.1217774
1425586_a_at	melanophilin	Mlph	0.84119	-1.06976361	-0.0976157
1444845_at	Sec1 family domain containing 1	Scfd1	0.84119	1.10065263	0.12522351
1444989_at	---	---	0.84119	-1.08099873	-0.1210493
1437485_at	nitric oxide synthase 1 (neuronal) adaptor protein	Nos1ap	0.84119	1.08130545	0.11098505
1419985_s_at	coiled-coil domain containing 69	Ccdc69	0.8412	1.07684828	0.10409638
1439891_at	---	---	0.8412	-1.08136632	-0.1202604
1445092_at	---	---	0.8412	1.07841825	0.10817152
1456017_x_at	oocyte specific homeobox 2	Obox2	0.8412	1.08133259	0.10850169
1450285_at	ubiquitin-activating enzyme E1, Chr Y 1	Ube1y1	0.8412	1.08041961	0.1056457
1447304_at	---	---	0.8412	-1.07779564	-0.1083961
1417672_at	solute carrier family 4, sodium bicarbonate cotransporter-like, me	Slc4a10	0.8412	-1.06768173	-0.0947736
1450759_at	bone morphogenetic protein 6	Bmp6	0.84121	-1.08163338	-0.1191633
1439051_a_at	MAP/microtubule affinity-regulating kinase 4	Mark4	0.84121	1.1028519	0.12363319
1417713_at	eukaryotic translation initiation factor 2, subunit 2 (beta)	Eif2s2	0.84121	1.06449276	0.08212133
1441978_at	aquaporin 6	Aqp6	0.84121	-1.0810343	-0.1240714
1422558_at	guanidinoacetate methyltransferase	Gamt	0.84121	1.07577848	0.10492426
1450742_at	bystin-like	Bysl	0.84121	1.05762383	0.08051373
1452356_at	IQ motif containing C	Iqcc	0.84121	1.06849215	0.09372634
1451741_a_at	cyclin-dependent kinase 7	Cdk7	0.84121	1.06401466	0.08452247
1451924_a_at	endothelin 1	Edn1	0.84122	1.11587348	0.13797023
1419812_s_at	coiled-coil domain containing 56	Ccdc56	0.84122	1.05723924	0.07751982
1454891_at	CDP-diacylglycerol synthase (phosphatidate cytidylyltransferase)	Cds2	0.84123	1.0723378	0.09584019
1450669_at	mitogen-activated protein kinase kinase kinase 11	Map3k11	0.84123	1.06680845	0.09173144
1459834_x_at	sparc/osteonectin, cwcv and kazal-like domains proteoglycan 3	Spock3	0.84123	1.07829726	0.10842824
1442601_at	glucocorticoid modulatory element binding protein 1	Gmeb1	0.84123	1.07970461	0.11043185
1434576_at	testis specific gene A14	Tsga14	0.84124	1.07429636	0.10135538
1458222_at	---	---	0.84124	-1.07791809	-0.1197227
1455019_x_at	cytoskeleton-associated protein 4	Ckap4	0.84124	-1.05806149	-0.0894813
1427930_at	pyridoxal (pyridoxine, vitamin B6) kinase	Pdxk	0.84124	1.06602199	0.09197135
1420014_at	Lanosterol synthase	Lss	0.84124	-1.07786338	-0.1105516
1446409_at	glycosyltransferase-like domain containing 1	Gtdc1	0.84124	1.10860882	0.13084081
1443972_at	expressed sequence AI465300	AI465300	0.84125	1.07847638	0.1035798
1418237_s_at	collagen, type XVIII, alpha 1	Col18a1	0.84125	1.06392321	0.08548221
1448427_at	NADH dehydrogenase (ubiquinone) 1 alpha subcomplex, 6 (B14)	Ndufa6	0.84125	1.06219971	0.08165519
1433190_at	RIKEN cDNA 4933433N18 gene	4933433N18Rik	0.84125	1.07836316	0.10666908
1438604_at	ATPase, class I, type 8B, member 3	Atp8b3	0.84125	1.07512513	0.10260897
1442181_at	---	---	0.84125	1.09404751	0.11795053
1435202_at	zinc finger protein 574	Zfp574	0.84125	1.07476256	0.09643716
1457395_at	neuron navigator 2	Nav2	0.84126	1.0863786	0.11156959
1433865_at	RIKEN cDNA E330010L02 gene /// RIKEN cDNA E330016L19 gene	E330010L02Rik /// E3300	0.84126	-1.05896885	-0.0858347
1441263_a_at	RIKEN cDNA A930005H10 gene	A930005H10Rik	0.84126	1.07339335	0.09753428
1437734_at	protein phosphatase 1, regulatory (inhibitor) subunit 12A	Ppp1r12a	0.84126	-1.06098557	-0.0917373
1443076_at	leucine zipper-EF-hand containing transmembrane protein 2	Letm2	0.84127	-1.07730816	-0.1078403
1417119_at	zinc finger like protein 1	Zfpl1	0.84127	1.0827267	0.1049987
1447434_at	---	---	0.84127	1.08379856	0.11035315
1460359_at	armadillo repeat containing, X-linked 3	Armcx3	0.84127	-1.08797284	-0.1498563
1457533_at	---	---	0.84127	1.08075822	0.11014328
1447649_x_at	DnaJ (Hsp40) homolog, subfamily C, member 1	Dnajc1	0.84127	1.09576184	0.12037096
1434786_at	protein phosphatase 1, regulatory (inhibitor) subunit 12B	Ppp1r12b	0.84127	1.08995646	0.11863054
1451401_a_at	mitochondrial ubiquitin ligase activator of NFKB 1	Mul1	0.84128	1.05795083	0.08048747
1425069_at	cytochrome P450, family 2, subfamily d, polypeptide 34	Cyp2d34	0.84128	-1.07884324	-0.1164135
1430334_at	zinc finger protein 192	Zfp192	0.84128	1.07847649	0.1080872
1448285_at	regulator of G-protein signaling 4	Rgs4	0.84128	1.0835048	0.10632276
1454404_at	RIKEN cDNA 2610011E03 gene	2610011E03Rik	0.84128	-1.08196361	-0.1148153
1442967_at	expressed sequence AU020147	AU020147	0.84128	1.09346432	0.12123374
1418023_at	N(alpha)-acetyltransferase 15, NatA auxiliary subunit	Naa15	0.84128	-1.05627154	-0.082377
1459602_at	cyclin I	Ccni	0.84129	-1.0760553	-0.1058655
1448020_at	RAS-related protein-1a	Rap1a	0.84129	1.06900406	0.09613261
1453208_at	RIKEN cDNA 2700089E24 gene	2700089E24Rik	0.84129	-1.070052	-0.1029194
1452681_at	deoxythymidylate kinase	Dtymk	0.8413	1.05438425	0.07480766
1418690_at	protein tyrosine phosphatase, receptor type Z, polypeptide 1	Ptprz1	0.8413	1.08544212	0.10647314
1453877_at	RIKEN cDNA 4930467D21 gene	4930467D21Rik	0.8413	1.07503161	0.09824852
1458730_at	hypothetical LOC100504106	LOC100504106	0.8413	1.08546932	0.11254335
1453928_a_at	Sjogren syndrome antigen B	Ssb	0.8413	-1.05526798	-0.0805813
1445961_at	---	---	0.8413	-1.08156849	-0.1197416
1436701_at	tripartite motif-containing 61	Trim61	0.8413	1.12622779	0.14640077
1419353_at	dolichol-phosphate (beta-D) mannosyltransferase 1	Dpm1	0.84131	1.10082914	0.12643271
1447060_at	---	---	0.84131	-1.08805378	-0.1624687
1438909_at	saccharopine dehydrogenase (putative)	Sccpdh	0.84131	-1.07692174	-0.1112369
1433188_at	RIKEN cDNA B230112I24 gene	B230112I24Rik	0.84131	1.093062	0.11672781
1433987_at	hippocalcin-like 4	Hpcal4	0.84131	1.08164995	0.10929971
1450129_a_at	suppressor of cytokine signaling 6	Socs6	0.84131	1.08673302	0.107338
1432083_a_at	leucine rich repeat containing 23	Lrrc23	0.84131	-1.06820916	-0.0991416
1456084_x_at	fibromodulin	Fmod	0.84132	1.0675686	0.09401341
1417838_at	hypothetical protein LOC100039614 /// spermiogenesis specific t	LOC100039614 /// LOC10	0.84132	1.08255403	0.10998252
1420141_at	expressed sequence AA517023	AA517023	0.84132	-1.0672655	-0.1004835
1444651_at	hypothetical LOC553089	LOC553089	0.84132	1.07442282	0.1017053
1434811_at	cancer antigen 1	Cage1	0.84133	1.09631858	0.12449535
1425653_at	activating transcription factor 7	Atf7	0.84134	-1.07805661	-0.1115495
1436964_at	DNA segment, Chr 7, ERATO Doi 715, expressed	D7Ertd715e	0.84134	-1.08363183	-0.1519224
1442931_at	---	---	0.84134	-1.07285585	-0.1067669
1426603_at	ribonuclease L (2', 5'-oligoisoadenylate synthetase-dependent)	Rnasel	0.84134	1.10664404	0.13167482
1439822_at	---	---	0.84134	1.10862073	0.13190396
1460156_at	acyloxyacyl hydrolase	Aoah	0.84134	1.05035103	0.07037337
1416977_at	signal transducing adaptor molecule (SH3 domain and ITAM mot	Stam2	0.84134	1.15447068	0.16741592
1448314_at	cyclin-dependent kinase 1	Cdk1	0.84135	1.05246184	0.07201254

1448822_at	proteasome (prosome, macropain) subunit, beta type 6	Psmb6	0.84136	1.04803878	0.06628958
1430171_at	fibroblast growth factor (acidic) intracellular binding protein	Fibp	0.84136	1.08290764	0.10899587
1432386_a_at	PHD finger protein 7	Phf7	0.84136	1.08490446	0.11312692
1460124_at	non-specific cytotoxic cell receptor protein 1 homolog (zebrafish)	Nccrp1	0.84136	-1.0758342	-0.1091123
1447362_at	budding uninhibited by benzimidazoles 1 homolog, beta (S. cerev	Bub1b	0.84136	-1.07952347	-0.1133108
1439800_at	CAP-GLY domain containing linker protein 1 /// hypothetical prote	Clip1 /// LOC100503214	0.84136	1.0773655	0.10671219
1458550_at	myosin ID	Myo1d	0.84136	-1.07578007	-0.1079983
1448117_at	kit ligand	Kitl	0.84136	1.13936372	0.15493729
1455716_at	expressed sequence BB187676	BB187676	0.84137	-1.06683204	-0.0933994
1451221_at	cDNA sequence BC018507	BC018507	0.84137	-1.05884394	-0.0839159
1454799_at	1-acylglycerol-3-phosphate O-acyltransferase 9	Agpat9	0.84137	-1.07602498	-0.1096636
1434738_at	threonyl-tRNA synthetase-like 2	Tarsl2	0.84137	1.08598678	0.11137991
1438162_x_at	predicted gene 4871	Gm4871	0.84138	-1.0643165	-0.100449
1424205_at	SWI/SNF related, matrix associated, actin dependent regulator of	Smarca5	0.84138	-1.05154753	-0.0728162
1434358_x_at	ribosomal protein S21	Rps21	0.84138	-1.03494416	-0.0526734
1454620_x_at	predicted gene 13654 /// ribosomal protein S6 pseudogene /// 4(	Gm13654 /// Gm16409 //	0.84138	-1.02590952	-0.0376715
1453145_at	phosphatidylserine decarboxylase, pseudogene 3	Pisd-ps3	0.84138	-1.06865142	-0.1005927
1451585_x_at	splA/ryanodine receptor domain and SOCS box containing 2	Spsb2	0.84138	1.08397411	0.11004026
1449824_at	proteoglycan 4 (megakaryocyte stimulating factor, articular supe	Prg4	0.84139	1.08605864	0.11301921
1431305_at	mucolipin 1	Mcoln1	0.84139	1.12646938	0.14759719
1443172_at	origin recognition complex, subunit 1	Orc1	0.84139	1.10231808	0.12423543
1447694_x_at	neogenin	Neo1	0.84139	-1.07599503	-0.1147296
1421245_at	sclerostin	Sost	0.8414	-1.08514202	-0.1257411
1421036_at	neuronal PAS domain protein 2	Npas2	0.8414	-1.07964718	-0.116787
1420426_at	myosin VIIB	Myo7b	0.8414	-1.06886789	-0.1018093
1453192_at	family with sequence similarity 101, member A	Fam101a	0.8414	1.10653356	0.13067428
1433696_at	hematological and neurological expressed 1-like	Hn1l	0.8414	-1.04922504	-0.0694145
1437048_x_at	syntaxin binding protein 2	Stxbp2	0.8414	1.06118577	0.08485521
1427696_at	ferrochelatase	Fech	0.8414	-1.07893129	-0.1130091
1446376_at	ribosomal protein S6 kinase, polypeptide 1	Rps6kb1	0.8414	1.07433438	0.10279843
1435724_at	regulatory factor X 8	Rfx8	0.84141	1.08259097	0.11280246
1454584_at	zinc finger protein 54	Zfp54	0.84141	-1.08040106	-0.121222
1421510_at	transmembrane protease, serine 15	Tmprss15	0.84141	-1.07178966	-0.1013727
1432086_a_at	RIB43A domain with coiled-coils 2	Ribc2	0.84142	-1.08033155	-0.1177148
1456559_at	empty spiracles homolog 2 (Drosophila) opposite strand	Emx2os	0.84142	-1.08331492	-0.1204484
1428376_at	RIKEN cDNA 4932415G12 gene	4932415G12Rik	0.84142	-1.07876591	-0.1170965
1446374_at	Rho guanine nucleotide exchange factor (GEF) 10	Arhgef10	0.84142	1.0771916	0.10518058
1455529_at	mex3 homolog A (C. elegans)	Mex3a	0.84143	-1.05600192	-0.0812702
1441530_at	---	---	0.84143	1.10654434	0.13180829
1454119_at	MAP kinase-interacting serine/threonine kinase 1	Mknk1	0.84143	-1.0722221	-0.1029932
1427748_at	Immunoglobulin kappa chain variable 1-117	Igkv1-117	0.84143	-1.07516101	-0.1055136
1457108_at	---	---	0.84144	1.07574265	0.10412325
1415776_at	aldehyde dehydrogenase family 3, subfamily A2	Aldh3a2	0.84144	1.0671716	0.09194255
1418409_at	jerky	Jrk	0.84145	1.07785365	0.09946061
1446051_at	predicted gene 1140	Gm1140	0.84145	-1.07254127	-0.1138138
1457717_at	DNA segment, Chr 14, ERATO Doi 449, expressed	D14Ertd449e	0.84145	-1.07784049	-0.1156538
1442026_at	---	---	0.84145	1.08414853	0.1130152
1434558_at	WD repeat domain 47	Wdr47	0.84145	-1.06616012	-0.0957602
1420813_at	histone deacetylase 7	Hdac7	0.84145	1.08108269	0.10531512
1427947_at	B9 protein domain 2	B9d2	0.84145	1.07162853	0.09580791
1428775_at	RIKEN cDNA 1110008L16 gene	1110008L16Rik	0.84145	1.06222655	0.08234303
1447932_at	zinc finger protein 830	Zfp830	0.84145	-1.07158399	-0.1002942
1453220_at	family with sequence similarity 55, member B	Fam55b	0.84146	1.07914055	0.10675374
1435663_at	estrogen receptor 1 (alpha)	Esr1	0.84146	1.05655859	0.07931103
1447016_at	TBC1 domain family, member 1	Tbc1d1	0.84147	1.09283635	0.12175202
1425060_s_at	CAP-GLY domain containing linker protein 1	Clip1	0.84147	1.05464447	0.07665561
1457426_at	RIKEN cDNA 1700048O20 gene	1700048O20Rik	0.84147	1.0757818	0.10319581
1429698_at	mitochondrial transcription termination factor	Mterf	0.84147	-1.0611323	-0.0867356
1427516_a_at	biregional cell adhesion molecule-related/down-regulated by onc	Boc	0.84147	-1.07512677	-0.1136989
1450338_x_at	vomeronasal 2, receptor, pseudogene 159 /// vomeronasal 2, rec	Vmn2r-ps159 /// Vmn2r-|	0.84148	1.07036362	0.09301747
1458125_at	---	---	0.84148	1.06819653	0.0928841
1425790_a_at	glutamate receptor, ionotropic, kainate 2 (beta 2)	Grik2	0.84148	-1.07957574	-0.121343
1431170_at	ephrin A3	Efna3	0.8415	-1.07186512	-0.112759
1443375_at	GNAS (guanine nucleotide binding protein, alpha stimulating) com	Gnas	0.8415	-1.06535635	-0.0921685
1430227_at	RIKEN cDNA 1700007I08 gene	1700007I08Rik	0.8415	-1.07477485	-0.1063314
1436581_at	tetratricopeptide repeat domain 14	Ttc14	0.8415	-1.08047868	-0.1220424
1442874_at	---	---	0.8415	1.0905599	0.11875528
1444267_at	---	---	0.84151	1.06603429	0.09061505
1443634_at	---	---	0.84152	1.07700587	0.10511756
1436017_at	alanyl-tRNA synthetase 2, mitochondrial (putative)	Aars2	0.84152	1.06054914	0.08383926
1435024_at	RIKEN cDNA 4931428F04 gene	4931428F04Rik	0.84152	1.10132673	0.12161518
1424261_at	zinc finger protein 672	Zfp672	0.84153	1.12226503	0.14237011
1444236_at	expressed sequence AW552393	AW552393	0.84153	-1.08247418	-0.1253406
1440847_at	metastasis suppressor 1	Mtss1	0.84153	-1.08155426	-0.1628402
1420236_at	---	---	0.84154	-1.07809788	-0.1102077
1426286_at	nucleolar complex associated 3 homolog (S. cerevisiae)	Noc3l	0.84154	-1.06178043	-0.0895959
1428458_at	processing of precursor 1, ribonuclease P/MRP family, (S. cerevisi	Pop1	0.84155	1.06562469	0.08702542
1427350_a_at	GTP binding protein 6 (putative)	Gtpbp6	0.84156	1.05844424	0.08188459
1424695_at	RIKEN cDNA 2010011I20 gene	2010011I20Rik	0.84156	1.07759308	0.10564169
1441278_at	RIKEN cDNA 4930513N10 gene	4930513N10Rik	0.84157	-1.0900568	-0.1527877
1423811_at	splicing factor 3a, subunit 3	Sf3a3	0.84157	1.05656214	0.07730604
1418435_at	makorin, ring finger protein, 1	Mkrn1	0.84158	-1.05137562	-0.0743806
1422506_a_at	cystatin B	Cstb	0.84158	1.05897655	0.0801022
1430347_at	RIKEN cDNA 4930524J08 gene	4930524J08Rik	0.84158	-1.07432282	-0.1125327
1453876_at	RIKEN cDNA 1700120B22 gene	1700120B22Rik	0.84159	-1.08327518	-0.1309504
1435328_at	cysteine and histidine rich 1	Cyhr1	0.84159	1.06237043	0.08548715
1444908_at	hyaluronic acid binding protein 4	Habp4	0.8416	1.08618528	0.11252321
1449067_at	solute carrier family 2 (facilitated glucose transporter), member 2	Slc2a2	0.8416	1.06956598	0.09671261
1457249_at	ubiquitin associated protein 2-like	Ubap2l	0.84161	-1.08194621	-0.129224
1460372_at	dual oxidase maturation factor 1	Duoxa1	0.84162	1.11128538	0.12970517
1455139_at	SWI/SNF related matrix associated, actin dependent regulator of	Smarcal1	0.84162	-1.0825927	-0.1193129
1433888_at	ATPase, Ca++ transporting, plasma membrane 2	Atp2b2	0.84164	-1.08260641	-0.1195281
1446549_at	Kelch repeat and BTB (POZ) domain containing 11	Kbtbd11	0.84164	1.08028538	0.11117321
1451228_a_at	syncollin	Sycn	0.84165	-1.08245063	-0.1204131

1447780_x_at	Tu translation elongation factor, mitochondrial	Tufm	0.84166	1.05345752	0.07478638
1449696_at	Aryl hydrocarbon receptor nuclear translocator	Arnt	0.84166	-1.08181656	-0.124664
1450516_a_at	RAB17, member RAS oncogene family	Rab17	0.84166	-1.07605784	-0.1087679
1420883_at	sarcolipin	Sln	0.84167	-1.07678297	-0.107361
1456267_at	RIKEN cDNA 2310045N01 gene	2310045N01Rik	0.84167	1.08699327	0.11462799
1460318_at	cysteine and glycine-rich protein 3	Csrp3	0.84167	1.08092277	0.11153846
1417419_at	cyclin D1	Ccnd1	0.84167	1.07060868	0.09283776
1443182_at	---	---	0.84168	-1.07992165	-0.1136382
1421554_at	LIM homeobox transcription factor 1 alpha	Lmx1a	0.84168	-1.08552878	-0.1320568
1417384_at	ectonucleoside triphosphate diphosphohydrolase 5	Entpd5	0.84169	1.11482643	0.13643975
1459225_at	Guanine nucleotide binding protein-like 3 (nucleolar)-like	Gnl3l	0.84169	1.09571538	0.12245207
1457648_x_at	family with sequence similarity 20, member C	Fam20c	0.84172	-1.08611852	-0.1480023
1436683_at	RIKEN cDNA 1700011M02 gene	1700011M02Rik	0.84172	-1.07410183	-0.1032598
1456105_at	ring finger and CCCH-type zinc finger domains 2	Rc3h2	0.84173	-1.0710665	-0.1026482
1420040_at	Family with sequence similarity 73, member B	Fam73b	0.84173	1.0781212	0.10611936
1445813_at	WD repeat domain 27	Wdr27	0.84174	-1.0836791	-0.1246001
1452740_at	myosin, heavy polypeptide 10, non-muscle	Myh10	0.84175	-1.05735906	-0.0858156
1447102_at	---	---	0.84176	1.09415561	0.11979265
1448935_at	chromodomain protein, Y chromosome-like	Cdyl	0.84177	1.06668986	0.08797624
1443570_at	COP9 (constitutive photomorphogenic) homolog, subunit 3 (Arabi	Cops3	0.84177	1.05413566	0.07372114
1426708_at	anthrax toxin receptor 2	Antxr2	0.84178	-1.07145623	-0.1033393
1431144_at	SRR1 domain containing	Srrd	0.84179	-1.07536418	-0.1049369
1437210_a_at	bromodomain containing 2	Brd2	0.84181	-1.06250674	-0.1000159
1422533_at	cytochrome P450, family 51	Cyp51	0.84182	-1.06368645	-0.0906535
1425848_a_at	dual specificity phosphatase 26 (putative)	Dusp26	0.84183	1.09268478	0.12071012
1446792_at	---	---	0.84183	1.05727742	0.0791498
1425444_a_at	transforming growth factor, beta receptor II	Tgfbr2	0.84186	-1.07464009	-0.1089195
1435894_at	hypothetical protein C030014L02	C030014L02	0.84187	-1.07937118	-0.112761
1446384_at	---	---	0.84188	-1.06911095	-0.0971591
1417244_a_at	interferon regulatory factor 7	Irf7	0.84201	-1.07988303	-0.1186236
1431718_at	kinesin family member 15	Kif15	0.84206	-1.07619093	-0.1100255
1452014_a_at	insulin-like growth factor 1	Igf1	0.8421	-1.08071832	-0.1282482
1444865_at	---	---	0.84212	1.08916639	0.11363485
1457987_at	RIKEN cDNA 6030458C11 gene	6030458C11Rik	0.84241	-1.08022045	-0.1302929
1418689_at	cathepsin 6	Cts6	0.84242	1.06005296	0.0839868
1445846_at	gamma-aminobutyric acid (GABA) A receptor, subunit gamma 3	Gabrg3	0.84245	1.09038166	0.11876014
1418115_s_at	torsin A interacting protein 2	Tor1aip2	0.84245	1.05805603	0.07910868
1422226_at	---	---	0.84247	-1.07568149	-0.1100394
1431989_at	acyloxyacyl hydrolase	Aoah	0.84248	-1.07846342	-0.1166961
1444237_at	---	---	0.84248	-1.07738217	-0.1111085
1445776_at	---	---	0.84249	1.07537701	0.10451423
1459389_at	---	---	0.8425	-1.07465175	-0.1068028
1436747_at	family with sequence similarity 100, member B	Fam100b	0.84251	1.05009568	0.06928056
1457459_at	expressed sequence AU014973	AU014973	0.84252	-1.08766346	-0.1352098
1426110_a_at	lysophosphatidic acid receptor 1	Lpar1	0.84252	-1.08230162	-0.1302367
1417868_a_at	cathepsin Z	Ctsz	0.84253	-1.05334326	-0.0788242
1420842_at	protein tyrosine phosphatase, receptor type, F	Ptprf	0.84253	-1.05355207	-0.0760136
1432450_at	RIKEN cDNA 8030423J24 gene	8030423J24Rik	0.84254	-1.07776636	-0.1131692
1446410_at	---	---	0.84255	1.11609171	0.13746723
1416127_a_at	aspartyl aminopeptidase	Dnpep	0.84255	1.05417806	0.07416697
1451211_a_at	ligatin	Lgtn	0.84255	1.05431051	0.07522737
1442872_at	KRI1 homolog (S. cerevisiae)	Kri1	0.84256	1.12340529	0.14083291
1433876_at	leucine rich repeat containing 24	Lrrc24	0.84256	-1.07752551	-0.108309
1453483_at	RIKEN cDNA 1700023E05 gene	1700023E05Rik	0.84256	-1.07661409	-0.1073578
1424657_at	TAO kinase 1	Taok1	0.84257	-1.06901569	-0.1128337
1445802_at	---	---	0.84257	-1.08150471	-0.1217173
1430897_at	RIKEN cDNA 4931428L18 gene	4931428L18Rik	0.84257	1.0844879	0.10938382
1451651_at	V-set and immunoglobulin domain containing 4	Vsig4	0.84258	1.09674584	0.12264509
1449619_s_at	Rho GTPase activating protein 9	Arhgap9	0.84258	1.08046284	0.10643433
1441155_at	---	---	0.84258	-1.06749693	-0.0984344
1423394_at	prenylcysteine oxidase 1	Pcyox1	0.84258	1.06583905	0.08909275
1415782_at	SMT3 suppressor of mif two 3 homolog 2 (yeast)	Sumo2	0.84259	-1.03833805	-0.0561591
1419845_at	distal-less homeobox 1, antisense	Dlx1as	0.84259	1.08738084	0.11350088
1436380_at	CDC42 binding protein kinase alpha	Cdc42bpa	0.84259	1.07414959	0.10151553
1427381_at	immunoresponsive gene 1	Irg1	0.84259	1.09715882	0.12418226
1459045_at	golgi integral membrane protein 4	Golim4	0.84259	1.10398801	0.12335806
1428825_at	nuclear receptor subfamily 6, group A, member 1	Nr6a1	0.84259	1.06384216	0.08675607
1448096_at	2-oxoglutarate and iron-dependent oxygenase domain containing	Ogfod1	0.84259	1.15432069	0.16415325
1421481_at	---	---	0.84259	1.07643919	0.1047996
1427751_a_at	keratin 36	Krt36	0.84259	1.1051025	0.13134553
1435242_at	PDS5, regulator of cohesion maintenance, homolog B (S. cerevisia	Pds5b	0.8426	1.07355701	0.09506866
1440658_at	AMME chromosomal region gene 1-like	Ammecr1l	0.8426	-1.07487577	-0.1049258
1455181_at	RAS p21 protein activator 2	Rasa2	0.8426	-1.06304503	-0.0897433
1448506_at	serine (or cysteine) peptidase inhibitor, clade A, member 6	Serpina6	0.8426	1.08813461	0.11425763
1438385_s_at	glutamic pyruvate transaminase (alanine aminotransferase) 2	Gpt2	0.8426	-1.06403718	-0.1039148
1457122_at	---	---	0.8426	-1.06460238	-0.0909232
1421384_at	lysosomal trafficking regulator	Lyst	0.84261	1.07348135	0.10208528
1437195_x_at	mitogen-activated protein kinase 10	Mapk10	0.84261	-1.05756724	-0.0831043
1432142_at	solute carrier family 35, member F4	Slc35f4	0.84261	1.08897949	0.11645312
1444414_at	---	---	0.84261	1.14849072	0.16232126
1446503_at	---	---	0.84261	-1.06929257	-0.0998853
1442018_at	B-cell translocation gene 1, anti-proliferative	Btg1	0.84261	1.09494886	0.1239873
1442156_at	RIKEN cDNA E030030I06 gene	E030030I06Rik	0.84261	1.08445129	0.11243308
1437187_at	E2F transcription factor 7	E2f7	0.84262	1.06726341	0.0913485
1453455_at	calmodulin binding transcription activator 1	Camta1	0.84262	1.07892534	0.10939018
1435992_at	IQ motif containing with AAA domain	Iqca	0.84262	1.07511347	0.10364196
1415674_a_at	trafficking protein particle complex 4	Trappc4	0.84262	1.05701549	0.07844169
1458519_at	hypothetical LOC100503742	LOC100503742	0.84262	-1.07263271	-0.1120628
1419787_a_at	zinc finger protein 628	Zfp628	0.84262	1.07642115	0.10211776
1417483_at	nuclear factor of kappa light polypeptide gene enhancer in B-cells	Nfkbiz	0.84263	1.08621027	0.11419797
1418771_a_at	carboxypeptidase B2 (plasma)	Cpb2	0.84263	-1.07069695	-0.1021123
1456482_at	phosphatidylinositol 3 kinase, regulatory subunit, polypeptide 3 (p	Pik3r3	0.84263	1.09766759	0.12059229
1449098_a_at	polymerase (DNA directed), iota	Poli	0.84263	1.0648016	0.08609039
1455220_at	frequently rearranged in advanced T-cell lymphomas 2	Frat2	0.84263	1.06572007	0.08995232

1455943_at	zinc finger protein 451	Zfp451	0.84263	-1.06997498	-0.0984726
1457870_at	---	---	0.84264	1.08823479	0.11663495
1416541_at	ClpB caseinolytic peptidase B homolog (E. coli)	Clpb	0.84264	1.05238223	0.07333789
1451195_a_at	thioredoxin-related transmembrane protein 1	Tmx1	0.84264	1.05850518	0.08093144
1459683_at	predicted gene 10044	Gm10044	0.84264	-1.07309567	-0.1020642
1456470_x_at	tubulin, beta 2C	Tubb2c	0.84264	-1.07438463	-0.1173241
1455871_s_at	ribosomal protein L13 /// Tax1 (human T-cell leukemia virus type	Rpl13 /// Tax1bp3	0.84265	1.07155958	0.09244415
1431818_at	RIKEN cDNA 1700012B15 gene	1700012B15Rik	0.84265	-1.06996496	-0.0986032
1455953_x_at	phosphoseryl-tRNA kinase	Pstk	0.84265	-1.05925893	-0.0864544
1421645_at	RIKEN cDNA 9930013L23 gene	9930013L23Rik	0.84265	1.08167635	0.11104905
1431965_at	RIKEN cDNA 4933411E08 gene	4933411E08Rik	0.84266	1.09522942	0.12113634
1446311_at	extended synaptotagmin-like protein 1	Esyt1	0.84266	-1.07881467	-0.1108261
1427176_s_at	expressed sequence AI428936	AI428936	0.84266	1.07376401	0.09581496
1457701_at	transmembrane protein 136	Tmem136	0.84267	1.07449168	0.1002212
1447552_s_at	---	---	0.84267	1.08871463	0.11133428
1429399_at	ring finger protein 125	Rnf125	0.84267	-1.05773687	-0.0820276
1450692_at	kinesin family member 4	Kif4	0.84267	1.06855037	0.08928129
1435159_at	RIKEN cDNA 9430038I01 gene	9430038I01Rik	0.84267	1.06230613	0.08658729
1459617_at	Mitogen-activated protein kinase 14	Mapk14	0.84268	1.07777151	0.10288723
1435766_at	periphilin 1	Pphln1	0.84268	1.0687572	0.09334321
1435265_at	hypothetical LOC100504698	LOC100504698	0.84268	1.08353463	0.1113673
1438947_x_at	sema domain, immunoglobulin domain (Ig), short basic domain, s	Sema3f	0.84268	1.07561596	0.09600431
1424249_a_at	Rho GTPase activating protein 9	Arhgap9	0.84268	-1.07058337	-0.0994696
1421682_a_at	predicted gene 3417 /// predicted gene 3448 /// t-complex-asso	Gm3417 /// Gm3448 ///	0.84268	1.13645523	0.14662668
1451136_a_at	eukaryotic translation initiation factor 2B, subunit 2 beta	Eif2b2	0.84269	1.0508122	0.07055549
1440841_at	---	---	0.84269	-1.07211877	-0.1123289
1431207_at	metallo-beta-lactamase domain containing 2	Mblac2	0.84269	1.0762111	0.10368866
1454871_at	RNA binding motif protein 15B	Rbm15b	0.84269	1.0658688	0.08795584
1429925_at	C-type lectin domain family 12, member B	Clec12b	0.84269	-1.07795429	-0.1161146
1444687_at	complement component 1, q subcomponent-like 2	C1ql2	0.84269	-1.08160073	-0.1189475
1460214_at	Purkinje cell protein 4	Pcp4	0.84269	1.08723269	0.11386718
1428851_at	RIKEN cDNA 1300014I06 gene	1300014I06Rik	0.8427	1.07484563	0.09969639
1431056_a_at	lipoprotein lipase	Lpl	0.8427	-1.07491426	-0.1058067
1451518_at	zinc finger protein 709	Zfp709	0.8427	-1.07549861	-0.1072168
1447331_at	---	---	0.8427	1.0877999	0.11869209
1448814_at	growth factor receptor bound protein 2-associated protein 1	Gab1	0.84271	-1.06539749	-0.1051544
1436631_at	catenin (cadherin associated protein), alpha 1	Ctnna1	0.84271	1.10128862	0.12454878
1444680_at	Mediator complex subunit 15	Med15	0.84271	-1.07901737	-0.1122832
1449185_at	cone-rod homeobox containing gene	Crx	0.84271	-1.07967554	-0.1258675
1452444_at	N-ethylmaleimide sensitive fusion protein attachment protein be	Napb	0.84271	1.07928948	0.10921123
1455725_a_at	H3 histone, family 3B	H3f3b	0.84271	1.03664709	0.05124901
1418312_at	zinc finger protein (C2H2 type) 276	Zfp276	0.84271	1.08388177	0.11054052
1425013_at	solute carrier family 25 (mitochondrial carrier, ornithine transporte	Slc25a2	0.84271	1.10328705	0.12744893
1442870_at	---	---	0.84272	1.07961173	0.10720638
1439617_s_at	phosphoenolpyruvate carboxykinase 1, cytosolic	Pck1	0.84272	1.09170726	0.11844712
1422019_at	transforming growth factor, beta receptor II	Tgfbr2	0.84272	-1.06173575	-0.0871591
1457659_x_at	outer dense fiber of sperm tails 1	Odf1	0.84273	-1.06831578	-0.1004739
1456666_at	RIKEN cDNA 2610020C07 gene	2610020C07Rik	0.84273	-1.07658912	-0.1084822
1446994_at	Exosome component 2	Exosc2	0.84273	1.14358522	0.15652893
1459176_at	---	---	0.84273	1.07251296	0.09971347
1454054_at	RIKEN cDNA 4930459I23 gene	4930459I23Rik	0.84273	-1.07148872	-0.0997462
1435329_at	lysine (K)-specific demethylase 2A	Kdm2a	0.84273	1.08627585	0.10906119
1440897_at	---	---	0.84273	1.09255143	0.11875167
1434311_at	CCR4-NOT transcription complex, subunit 6-like	Cnot6l	0.84274	1.07585813	0.10349639
1439359_x_at	neurexin I	Nrxn1	0.84274	1.08347008	0.11112675
1432523_at	homeodomain interacting protein kinase 2	Hipk2	0.84274	-1.07652979	-0.1093042
1453865_a_at	OTU domain containing 5	Otud5	0.84274	1.05153203	0.07228368
1450293_at	melanoma antigen, family B, 3	Mageb3	0.84275	1.07355233	0.10118017
1422154_at	G protein-coupled receptor 27	Gpr27	0.84275	1.10653794	0.12810092
1433935_at	hypothetical LOC100504230	LOC100504230	0.84275	-1.05564812	-0.080757
1453110_at	POC1 centriolar protein homolog B (Chlamydomonas)	Poc1b	0.84275	1.09212186	0.12060193
1440638_at	RIKEN cDNA A230107N01 gene	A230107N01Rik	0.84275	-1.07416885	-0.1058366
1447840_x_at	E2F transcription factor 1	E2f1	0.84275	-1.0839705	-0.140796
1446927_at	---	---	0.84275	1.07197347	0.10019213
1419518_at	tubulin, alpha 8	Tuba8	0.84276	1.07506971	0.10284108
1441974_at	calcium/calmodulin-dependent protein kinase IV	Camk4	0.84276	-1.07015742	-0.0982067
1444077_at	syntaxin binding protein 5-like	Stxbp5l	0.84277	1.08111208	0.11211985
1425971_at	---	---	0.84277	-1.07948526	-0.11613
1458921_at	hypothetical LOC434348	LOC434348	0.84277	-1.08266343	-0.1394355
1457569_at	DNA segment, Chr 8, ERATO Doi 51, expressed	D8Ertd51e	0.84277	-1.0884221	-0.1769806
1422922_at	RecQ protein-like 4	Recql4	0.84277	1.05770556	0.08027002
1446255_at	KCNQ1 overlapping transcript 1	Kcnq1ot1	0.84278	1.07241459	0.09544454
1416312_at	arginyl-tRNA synthetase	Rars	0.84278	1.05220912	0.07141744
1424323_at	nucleolar complex associated 2 homolog (S. cerevisiae)	Noc2l	0.84278	1.07807325	0.09808598
1452673_at	RAN binding protein 3	Ranbp3	0.84278	1.05384264	0.07530744
1423499_at	synuclein, alpha interacting protein (synphilin)	Sncaip	0.84278	1.08089792	0.11120449
1445917_at	---	---	0.84278	-1.08164471	-0.1233499
1432024_at	tetratricopeptide repeat domain 28	Ttc28	0.84278	-1.07725304	-0.1116166
1441967_at	Parkinson disease 7 domain containing 1	Pddc1	0.84278	1.06325963	0.08701673
1432226_at	RIKEN cDNA 4930548G14 gene	4930548G14Rik	0.84279	-1.0855873	-0.1477397
1444861_at	DNA segment, Chr 13, ERATO Doi 297, expressed	D13Ertd297e	0.84279	1.08569388	0.11196484
1430638_at	coiled-coil domain containing 30	Ccdc30	0.84279	-1.07703848	-0.1100356
1434501_at	yippee-like 4 (Drosophila)	Ypel4	0.8428	1.10968956	0.13124135
1417719_at	sin3 associated polypeptide	Sap30	0.8428	1.05149436	0.07172842
1451542_at	single-stranded DNA binding protein 2	Ssbp2	0.8428	1.15268654	0.16487861
1428121_at	family with sequence similarity 125, member B	Fam125b	0.8428	-1.05458209	-0.0784964
1440605_at	fascin homolog 2, actin-bundling protein, retinal (Strongylocentrot	Fscn2	0.8428	1.07815804	0.10565418
1446639_at	---	---	0.8428	1.06920701	0.09593461
1425843_at	mitochondrial ribosomal protein L33	Mrpl33	0.84281	-1.08183715	-0.114701
1428117_x_at	dynein light chain Tctex-type 1A /// dynein light chain Tctex-type	Dynlt1a /// Dynlt1b /// Dy	0.84281	1.08825826	0.11634337
1418931_at	regenerating islet-derived family, member 4	Reg4	0.84281	1.08763346	0.1156456
1424689_at	protease, serine, 32	Prss32	0.84282	1.08755465	0.11821529
1429487_at	protein phosphatase 1, regulatory (inhibitor) subunit 12A	Ppp1r12a	0.84282	1.0842279	0.10240014
1417825_at	esterase D/formylglutathione hydrolase	Esd	0.84282	-1.04715639	-0.0666046

1423386_at	proteasome (prosome, macropain) 26S subunit, non-ATPase, 9	Psmd9	0.84283	1.06022282	0.08296058
1453181_x_at	phospholipid scramblase 1	Plscr1	0.84283	-1.06501422	-0.1006604
1423144_at	phosphatidylinositol 3-kinase, catalytic, alpha polypeptide	Pik3ca	0.84283	1.06764146	0.09301854
1430292_a_at	RIKEN cDNA 1810030N24 gene	1810030N24Rik	0.84283	1.06652963	0.08659493
1439518_at	multimerin 2	Mmrn2	0.84284	-1.06367125	-0.0969307
1458589_at	---	---	0.84284	1.07165563	0.09841003
1451218_at	ER degradation enhancer, mannosidase alpha-like 1	Edem1	0.84284	1.07586228	0.0985834
1438369_x_at	transmembrane protein 85	Tmem85	0.84285	1.0736823	0.09579754
1452396_at	serine/arginine-rich splicing factor 15	Srsf15	0.84285	1.06901311	0.09269575
1438311_at	RIKEN cDNA 2010002M12 gene	2010002M12Rik	0.84285	-1.06128349	-0.0868354
1454913_at	RIKEN cDNA 9930104L06 gene	9930104L06Rik	0.84285	-1.07396943	-0.1030923
1451835_at	SRY-box containing gene 21	Sox21	0.84285	1.15648782	0.16746719
1418418_a_at	prolactin family 8, subfamily a, member 6	Prl8a6	0.84285	1.069849	0.09549526
1415673_at	phosphoserine phosphatase	Psph	0.84286	-1.0546949	-0.0823168
1453943_a_at	acid phosphatase, prostate	Acpp	0.84286	-1.08024653	-0.1148257
1446091_at	---	---	0.84286	1.08576744	0.11423378
1457623_x_at	transformation related protein 53	Trp53	0.84287	-1.08438258	-0.1309495
1418327_at	RIKEN cDNA 1110058L19 gene	1110058L19Rik	0.84287	1.05801949	0.07802741
1441203_at	predicted gene 14204	Gm14204	0.84287	-1.08112796	-0.127266
1417082_at	acidic (leucine-rich) nuclear phosphoprotein 32 family, member B	Anp32b	0.84287	-1.03993069	-0.0565292
1442752_at	---	---	0.84287	-1.06811229	-0.0980712
1442279_at	---	---	0.84287	-1.09169547	-0.1601829
1426095_a_at	tumor necrosis factor receptor superfamily, member 22	Tnfrsf22	0.84289	-1.0849697	-0.1384115
1454049_at	gasdermin C-like, pseudogene	Gsdmcl-ps	0.84289	-1.07630871	-0.1088178
1427677_a_at	SRY-box containing gene 6	Sox6	0.84289	1.08431652	0.10876259
1432645_at	---	---	0.8429	-1.073345	-0.1035215
1439961_x_at	---	---	0.8429	-1.07597111	-0.1136327
1441491_at	RIKEN cDNA A330068G13 gene	A330068G13Rik	0.8429	-1.07088325	-0.1007973
1437743_at	AE binding protein 2	Aebp2	0.8429	-1.06549212	-0.0928021
1449257_at	DNA segment, Chr 11, Wayne State University 99, expressed	D11Wsu99e	0.8429	1.07258476	0.09775375
1420787_at	RIKEN cDNA 4933436I01 gene	4933436I01Rik	0.8429	-1.07266871	-0.1053705
1448963_at	nuclear transcription factor-Y gamma	Nfyc	0.8429	1.05461599	0.07587762
1437855_at	microtubule-associated protein 4	Mtap4	0.8429	1.08227579	0.10806084
1456360_at	testis derived transcript 3, pseudogene	Tes3-ps	0.8429	1.09506844	0.11989665
1423989_at	tectonin beta-propeller repeat containing 1	Tecpr1	0.84291	1.06790736	0.09276359
1421728_at	oligodendrocyte transcription factor 3	Olig3	0.84291	1.08408639	0.11056549
1424410_at	tetratricopeptide repeat domain 8	Ttc8	0.84291	1.10059899	0.12214224
1426806_at	oligonucleotide/oligosaccharide-binding fold containing 2A	Obfc2a	0.84291	-1.06672592	-0.1037629
1423373_at	ribonuclease P/MRP 30 subunit (human)	Rpp30	0.84291	1.05620238	0.07664865
1440596_at	calcineurin binding protein 1	Cabin1	0.84291	1.08309751	0.11237817
1449235_at	Fas ligand (TNF superfamily, member 6)	Fasl	0.84291	-1.07637421	-0.1187411
1421977_at	matrix metallopeptidase 19	Mmp19	0.84291	1.11113532	0.13231511
1421794_at	killer cell lectin-like receptor subfamily C, member 3	Klrc3	0.84292	1.08189553	0.10194702
1422304_at	lipocalin 4	Lcn4	0.84292	-1.08545257	-0.1560063
1446929_at	RIKEN cDNA D130062J21 gene	D130062J21Rik	0.84292	1.08848722	0.1155682
1428242_at	histocompatibility (minor) HA-1	Hmha1	0.84293	1.08495716	0.10879187
1430739_at	RIKEN cDNA 4930471M09 gene	4930471M09Rik	0.84293	-1.0734559	-0.1057974
1443547_at	---	---	0.84293	-1.07808799	-0.1136842
1428363_at	eukaryotic translation initiation factor 4, gamma 2	Eif4g2	0.84293	1.0800121	0.10964565
1418976_s_at	cell death-inducing DNA fragmentation factor, alpha subunit-like e	Cideb	0.84293	-1.07967838	-0.1210941
1428525_at	RIKEN cDNA 4930488B01 gene	4930488B01Rik	0.84293	1.10290943	0.12720656
1449801_at	---	---	0.84293	1.08465689	0.11157686
1421653_a_at	immunoglobulin heavy chain 2 (serum IgA) /// immunoglobulin h	Igh-2 /// Igh-VJ558 /// LO	0.84293	-1.08249282	-0.1325261
1435364_at	cysteine and histidine rich 1	Cyhr1	0.84293	1.06070017	0.08260886
1458563_at	zinc finger protein 668	Zfp668	0.84294	-1.08485532	-0.1226277
1417988_at	regulated endocrine-specific protein 18	Resp18	0.84294	1.09429161	0.11851308
1429340_at	HRAS-like suppressor family, member 5	Hrasls5	0.84294	-1.07829587	-0.1108999
1420296_at	Chloride channel 5	Clcn5	0.84294	1.11478403	0.13706489
1424872_at	RIKEN cDNA 2310001H12 gene	2310001H12Rik	0.84294	-1.05524998	-0.079477
1423203_a_at	centrin 1	Cetn1	0.84295	-1.06324675	-0.0896484
1453749_at	RIKEN cDNA 2610507I01 gene	2610507I01Rik	0.84295	1.08107314	0.11077442
1452682_at	family with sequence similarity 122, member B	Fam122b	0.84295	-1.06256562	-0.0951967
1439882_at	Sec23 interacting protein	Sec23ip	0.84295	1.07536643	0.10132725
1438264_a_at	tripeptidyl peptidase II	Tpp2	0.84296	-1.06572404	-0.1072431
1429204_at	calcium/calmodulin-dependent protein kinase II inhibitor 2	Camk2n2	0.84296	1.06934484	0.0965335
1450972_at	RIKEN cDNA 3110040N11 gene	3110040N11Rik	0.84296	1.05407535	0.07522261
1420602_a_at	extraembryonic, spermatogenesis, homeobox 1	Esx1	0.84296	1.07601183	0.10325076
1457348_at	---	---	0.84296	-1.08496838	-0.1296845
1457528_at	solute carrier family 4, sodium bicarbonate cotransporter, membe	Slc4a7	0.84296	-1.08148484	-0.1461781
1460495_s_at	HtrA serine peptidase 2	Htra2	0.84298	1.06391218	0.08935985
1441696_at	---	---	0.84298	-1.08304176	-0.1365897
1423655_a_at	RIKEN cDNA 1500010J02 gene	1500010J02Rik	0.84298	1.05673187	0.07898743
1450903_at	RAD23b homolog (S. cerevisiae)	Rad23b	0.84298	-1.05650943	-0.0900312
1423936_at	potassium channel tetramerisation domain containing 5	Kctd5	0.84299	1.05829616	0.08053752
1451832_at	chemokine-like factor	Cklf	0.843	-1.07803854	-0.117333
1420562_at	secreted Ly6/Plaur domain containing 1	Slurp1	0.84301	1.09181142	0.11813045
1452114_s_at	insulin-like growth factor binding protein 5	Igfbp5	0.84302	-1.07725498	-0.1167442
1435880_at	ankyrin repeat domain 50	Ankrd50	0.84302	1.07337886	0.09826462
1453710_at	transmembrane protein 116	Tmem116	0.84303	1.09669246	0.12196011
1417376_a_at	cell adhesion molecule 1	Cadm1	0.84303	-1.06858684	-0.1037497
1427363_at	---	---	0.84304	1.09467173	0.11978922
1458060_at	Low density lipoprotein receptor adaptor protein 1	Ldlrap1	0.84306	-1.07186218	-0.1006998
1459398_at	Pellino 1	Peli1	0.84306	-1.06638627	-0.0994452
1441314_at	inner centromere protein	Incenp	0.84307	1.10198285	0.12925594
1455014_at	histidine triad nucleotide binding protein 3	Hint3	0.84307	-1.07768302	-0.1237313
1449360_at	colony stimulating factor 2 receptor, beta 2, low-affinity (granuloc	Csf2rb2	0.84308	-1.07517466	-0.1071194
1418132_a_at	ubiquitin family domain containing 1	Ubfd1	0.84312	-1.05658199	-0.079724
1430339_at	RIKEN cDNA 2610021K21 gene	2610021K21Rik	0.84313	1.09016823	0.11693179
1424780_a_at	receptor accessory protein 3	Reep3	0.84315	1.14458915	0.15535011
1427384_at	chromodomain helicase DNA binding protein 6	Chd6	0.84318	-1.0731072	-0.1083798
1440278_at	dynein light chain LC8-type 1	Dynll1	0.84318	-1.08407858	-0.1342269
1427178_at	transmembrane channel-like gene family 4	Tmc4	0.84318	1.07348774	0.09986691
1450484_a_at	cytidine monophosphate (UMP-CMP) kinase 2, mitochondrial	Cmpk2	0.84319	1.07491028	0.09929494
1438389_x_at	ATP citrate lyase	Acly	0.8432	-1.05442126	-0.0813178

1422376_at	vomeronasal 1 receptor 50	Vmn1r50	0.8432	1.08688383	0.10826334
1420008_s_at	WW, C2 and coiled-coil domain containing 1	Wwc1	0.8432	1.0693832	0.09179788
1440840_at	RIKEN cDNA D630004K10 gene	D630004K10Rik	0.84322	-1.07176437	-0.1000307
1455134_at	RIKEN cDNA D730040F13 gene	D730040F13Rik	0.84323	-1.06724738	-0.0982188
1456121_at	centrosomal protein 97	Cep97	0.84323	-1.08472795	-0.1314521
1429812_at	RIKEN cDNA 2610002D18 gene	2610002D18Rik	0.84324	1.05890264	0.08082263
1435166_at	contactin 2	Cntn2	0.84324	-1.06986813	-0.0980559
1442556_at	---	---	0.84325	-1.08280749	-0.1284484
1449973_a_at	proline, glutamic acid and leucine rich protein 1	Pelp1	0.84325	-1.07498886	-0.1365766
1456525_at	EST AA881470	AA881470	0.84326	-1.07943712	-0.1164585
1455550_x_at	serine incorporator 3	Serinc3	0.84326	-1.05658032	-0.0838027
1430417_s_at	neuralized homolog 4 (Drosophila)	Neurl4	0.84326	1.05387574	0.07562848
1435115_at	fibronectin type III domain containing 5	Fndc5	0.84327	1.08570554	0.11264089
1452163_at	E26 avian leukemia oncogene 1, 5' domain	Ets1	0.84327	1.11126468	0.13263376
1439123_at	---	---	0.84327	-1.07911309	-0.1130644
1451391_at	RIKEN cDNA 2700050L05 gene	2700050L05Rik	0.84327	1.07048528	0.09322207
1425359_at	mal, T-cell differentiation protein-like	Mall	0.84327	-1.07357109	-0.1047661
1428287_at	cullin 5	Cul5	0.84328	1.06034945	0.08381714
1422222_at	involucrin	Ivl	0.84329	-1.08144826	-0.1211808
1432650_at	RIKEN cDNA 2510019K15 gene	2510019K15Rik	0.84329	1.0793578	0.10872912
1420539_a_at	chordin-like 2	Chrdl2	0.84329	1.0935146	0.11932785
1451733_at	glucosaminyl (N-acetyl) transferase 2, I-branching enzyme	Gcnt2	0.8433	1.09170808	0.11750906
1451622_at	LMBR1 domain containing 1	Lmbrd1	0.8433	1.05990053	0.08366077
1440383_at	DNA cross-link repair 1B, PSO2 homolog (S. cerevisiae)	Dclre1b	0.8433	1.08210818	0.11079126
1418996_a_at	LYR motif containing 5	Lyrm5	0.8433	1.06421373	0.087333
1458799_at	RIKEN cDNA A330033J07 gene	A330033J07Rik	0.8433	-1.06862025	-0.0979005
1426681_at	unkempt homolog (Drosophila)	Unk	0.84331	1.06078433	0.08412219
1417560_at	sideroflexin 1	Sfxn1	0.84332	-1.05236339	-0.074872
1452690_at	KH-type splicing regulatory protein	Khsrp	0.84332	-1.07447107	-0.1410756
1440465_at	---	---	0.84332	-1.08201172	-0.1168851
1435291_at	LEM domain containing 3	Lemd3	0.84332	-1.05764346	-0.0833914
1420048_at	expressed sequence C78859	C78859	0.84333	-1.06641366	-0.0953717
1439227_at	---	---	0.84333	1.07384734	0.0973503
1444426_at	Cas scaffolding protein family member 4	Cass4	0.84333	1.0680937	0.09309956
1424229_at	dual-specificity tyrosine-(Y)-phosphorylation regulated kinase 3	Dyrk3	0.84333	1.05070174	0.07099589
1447596_at	---	---	0.84333	1.08272648	0.11139159
1454711_at	triple functional domain (PTPRF interacting)	Trio	0.84333	1.14109019	0.15171253
1443804_at	Protein phosphatase 2 (formerly 2A), regulatory subunit B (PR 52)	Ppp2r2c	0.84335	1.10596465	0.1297937
1448847_at	serine incorporator 3	Serinc3	0.84335	1.09816411	0.12306204
1455579_at	casein alpha s2-like A	Csn1s2a	0.84336	-1.06211803	-0.0884773
1452267_at	FLYWCH-type zinc finger 1	Flywch1	0.84337	-1.06955179	-0.0990721
1460722_at	sterol O-acyltransferase 2	Soat2	0.84337	1.08412108	0.10896286
1424582_at	acylglycerol kinase	Agk	0.84338	-1.06163073	-0.0899505
1444123_at	PR domain containing 2, with ZNF domain	Prdm2	0.84338	-1.07930007	-0.1132025
1448101_s_at	tripartite motif-containing 27	Trim27	0.84338	1.05244078	0.07197884
1421885_at	son of sevenless homolog 1 (Drosophila)	Sos1	0.84339	1.07949579	0.10631017
1442228_at	---	---	0.8434	-1.07407746	-0.1040333
1448925_at	twist homolog 2 (Drosophila)	Twist2	0.84341	-1.06992618	-0.0978081
1449981_a_at	N-acetyltransferase 2 (arylamine N-acetyltransferase)	Nat2	0.84341	1.08242084	0.10902859
1460485_at	RIKEN cDNA 8430434A19 gene	8430434A19Rik	0.84342	-1.07445266	-0.1254559
1448437_a_at	GTP binding protein 2	Gtpbp2	0.84343	-1.05903384	-0.0872084
1460505_at	RIKEN cDNA 8030442B05 gene	8030442B05Rik	0.84343	1.098869	0.12234671
1453023_at	ankyrin repeat and KH domain containing 1	Ankhd1	0.84345	1.0560406	0.07769963
1435064_a_at	transmembrane protein 27	Tmem27	0.84346	1.07330163	0.09535505
1446566_at	---	---	0.84346	1.08432704	0.11248715
1415868_at	chaperonin containing Tcp1, subunit 4 (delta)	Cct4	0.84347	1.09136431	0.11340608
1426576_at	sphingomyelin synthase 1	Sgms1	0.84347	-1.06659804	-0.1006713
1456762_at	GTPase, IMAP family member 6	Gimap6	0.84347	-1.0772876	-0.1188579
1441677_at	---	---	0.84348	1.09945864	0.1198554
1458468_at	Coenzyme Q10 homolog A (yeast)	Coq10a	0.84349	-1.07548035	-0.1125154
1434043_a_at	replication initiator 1	Repin1	0.84351	1.08362545	0.10857083
1420747_at	per-pentamer repeat gene	Ppnr	0.84352	1.06777076	0.0924809
1418863_at	GATA binding protein 4	Gata4	0.84355	1.08005468	0.10739121
1444880_at	---	---	0.84357	-1.07407806	-0.1037515
1416329_at	cytoplasmic FMR1 interacting protein 1	Cyfip1	0.84359	-1.0517504	-0.0739692
1448673_at	poliovirus receptor-related 3	Pvrl3	0.84361	-1.06499255	-0.1010357
1422972_s_at	K(lysine) acetyltransferase 2A	Kat2a	0.84362	-1.05412548	-0.0763104
1415710_at	COX18 cytochrome c oxidase assembly homolog (S. cerevisiae)	Cox18	0.84367	-1.05324258	-0.0753988
1453973_s_at	RIKEN cDNA 2900057H02 gene /// transmembrane protein 143	2900057H02Rik /// Tmem	0.84368	1.13290182	0.14558421
1427029_at	HtrA serine peptidase 3	Htra3	0.84372	-1.07922173	-0.1195467
1436524_at	RIKEN cDNA 4833438C02 gene /// hypothetical LOC100503392	4833438C02Rik /// LOC10	0.84372	1.1124069	0.13632369
1446100_at	Transformation related protein 53 binding protein 1	Trp53bp1	0.84372	-1.07570108	-0.1058362
1434804_at	exocyst complex component 6B	Exoc6b	0.84374	1.06855995	0.09262202
1444504_at	dehydrogenase/reductase (SDR family) member 7C	Dhrs7c	0.84377	1.08628648	0.11569913
1429746_at	EF-hand calcium binding domain 9	Efcab9	0.84377	-1.0814575	-0.1240181
1449898_at	septin 1	Sep-01	0.84381	1.11007187	0.12566145
1458287_at	---	---	0.84387	-1.08022061	-0.1273031
1418124_at	transmembrane protein 85	Tmem85	0.844	1.05216378	0.07273136
1443704_at	---	---	0.844	1.07805025	0.10628691
1444836_at	---	---	0.84401	-1.06054825	-0.084846
1441229_at	RIKEN cDNA D230019N24 gene	D230019N24Rik	0.84401	1.10928564	0.12956449
1419126_at	homeobox D9	Hoxd9	0.84401	1.11144563	0.12873238
1450001_a_at	Usher syndrome 1C homolog (human)	Ush1c	0.84402	-1.07811533	-0.1110418
1426098_a_at	calpastatin	Cast	0.84403	-1.06669155	-0.0995581
1435311_s_at	synapsin III	Syn3	0.84403	1.07519073	0.10054842
1447265_at	expressed sequence AI848615	AI848615	0.84404	-1.06066187	-0.0937639
1435484_at	solute carrier family 5 (inositol transporters), member 3	Slc5a3	0.84405	-1.07339416	-0.1220667
1438304_at	CXXC finger 4	Cxxc4	0.84405	-1.08080564	-0.1207985
1426731_at	desmin	Des	0.84405	-1.06136966	-0.086192
1449594_at	---	---	0.84407	-1.08188527	-0.1422659
1450686_at	paraoxonase 2	Pon2	0.84408	1.07543542	0.10152835
1437443_at	Predicted gene 6146	Gm6146	0.84412	-1.07520637	-0.1069038
1426505_at	ecotropic viral integration site 2b	Evi2b	0.84417	-1.07564592	-0.1139798
1451200_at	kinesin family member 1B	Kif1b	0.84419	-1.06118839	-0.0862624

1415766_at	SEC22 vesicle trafficking protein homolog B (S. cerevisiae)	Sec22b	0.84423	1.07834904	0.10216952
1432074_at	RIKEN cDNA 4930517E11 gene	4930517E11Rik	0.84424	1.10204289	0.12685352
1459992_x_at	ceroid-lipofuscinosis, neuronal 8	Cln8	0.84427	-1.0610772	-0.0867387
1450611_at	orosomucoid 3	Orm3	0.84429	1.08460596	0.11455526
1436297_a_at	glutamate receptor, ionotropic, N-methyl D-aspartate-associated	Grina	0.8443	-1.07219817	-0.108168
1448640_at	solute carrier family 14 (urea transporter), member 1	Slc14a1	0.84433	1.0826137	0.10893052
1440155_at	follistatin-like 4	Fstl4	0.84438	1.07525808	0.10461155
1435936_at	solute carrier family 13 (sodium-dependent citrate transporter), m	Slc13a5	0.84439	-1.06154519	-0.0866106
1458069_at	---	---	0.8444	-1.07726649	-0.1163396
1421731_a_at	flap structure specific endonuclease 1	Fen1	0.84456	1.05570627	0.07602649
1420197_at	Growth arrest and DNA-damage-inducible 45 beta	Gadd45b	0.84463	1.08600873	0.10732228
1422186_s_at	cytochrome b5 reductase 3	Cyb5r3	0.84466	1.05375091	0.0738533
1429087_at	RIKEN cDNA 1110054O05 gene	1110054O05Rik	0.84472	-1.06407801	-0.0898325
1436158_at	eukaryotic translation initiation factor 4E binding protein 2	Eif4ebp2	0.84483	-1.04760889	-0.0676306
1438400_at	RIKEN cDNA 4632411B12 gene	4632411B12Rik	0.84484	1.06532081	0.08635012
1432818_at	proline-rich coiled-coil 1	Prrc1	0.84484	-1.07772696	-0.1234948
1453114_at	nucleolar protein 9	Nol9	0.84484	-1.05198184	-0.0762353
1417617_at	ST6 (alpha-N-acetyl-neuraminyl-2,3-beta-galactosyl-1,3)-N-acety	St6galnac2	0.84485	1.07975971	0.10759946
1456165_at	RIKEN cDNA 4921521F21 gene	4921521F21Rik	0.84486	-1.07363041	-0.1108913
1434374_at	family with sequence similarity 168, member A	Fam168a	0.84486	-1.07479792	-0.1245235
1446671_at	---	---	0.84487	-1.06048975	-0.0903572
1456921_at	---	---	0.84487	-1.07551443	-0.109822
1452923_at	NADH dehydrogenase (ubiquinone) 1 alpha subcomplex, assembl	Ndufaf2	0.84487	1.06578028	0.0867899
1457470_at	---	---	0.84489	1.13853071	0.15554236
1457045_at	UDP-N-acetyl-alpha-D-galactosamine:polypeptide N-acetylgalact	Galnt13	0.84489	1.10481056	0.12038585
1452170_at	chondroitin polymerizing factor 2	Chpf2	0.8449	1.07464977	0.10334835
1445403_at	---	---	0.8449	-1.07941826	-0.1121419
1432476_at	RIKEN cDNA 1700040F17 gene	1700040F17Rik	0.84491	1.07126106	0.09757796
1420604_at	homeobox gene expressed in ES cells	Hesx1	0.84492	-1.07812717	-0.1099751
1416352_s_at	trans-2,3-enoyl-CoA reductase	Tecr	0.84495	1.04894707	0.06836805
1435000_at	G1 to S phase transition 1	Gspt1	0.84495	1.05638269	0.07761151
1450353_at	solute carrier family 25 (mitochondrial carrier, peroxisomal memb	Slc25a17	0.84498	1.1035259	0.12828893
1457595_at	---	---	0.84501	1.08551181	0.11266769
1423669_at	collagen, type I, alpha 1	Col1a1	0.84502	1.07269285	0.10037938
1436525_at	adaptor-related protein complex 3, sigma 2 subunit	Ap3s2	0.84505	-1.06254788	-0.0887971
1427660_x_at	predicted gene 10883 /// predicted gene 1420 /// predicted gene	Gm10883 /// Gm1420 ///	0.84505	-1.08109011	-0.1205042
1435763_at	TBC1 domain family, member 16	Tbc1d16	0.84507	1.07829536	0.10118155
1416083_at	zinc finger, AN1-type domain 5	Zfand5	0.84507	1.06278249	0.08283449
1449529_s_at	prolactin family 7, subfamily a, member 1	Prl7a1	0.84527	1.06759195	0.08908448
1460410_at	RIKEN cDNA 2310079F23 gene	2310079F23Rik	0.84532	1.06622224	0.09138691
1457403_at	RIKEN cDNA 9130409I23 gene	9130409I23Rik	0.84532	1.06504227	0.08901014
1426969_at	tripartite motif-containing 23	Trim23	0.84533	-1.05346808	-0.0756848
AFFX-TrpnX-5_at	---	---	0.84534	1.08182333	0.10811455
1417785_at	phospholipase A1 member A	Pla1a	0.84535	-1.06462931	-0.0927973
1455426_at	Eph receptor A3	Epha3	0.84535	1.05525341	0.07497934
1448541_at	kinesin light chain 1	Klc1	0.84537	1.0956018	0.11728054
1420281_at	---	---	0.84537	-1.04978981	-0.0716866
1418120_at	RNA binding motif protein 8a	Rbm8a	0.84537	-1.05564242	-0.0847978
1446478_at	thrombospondin, type I, domain containing 7B	Thsd7b	0.8454	-1.05989711	-0.0851387
1450360_at	phospholipase C, gamma 1	Plcg1	0.84541	1.08757844	0.11505198
1435938_at	cytoskeleton associated protein 2-like	Ckap2l	0.84543	1.0621151	0.08532385
1443611_at	---	---	0.84543	1.15824221	0.16083973
1443166_at	---	---	0.84549	-1.07855698	-0.1184944
1433593_at	yippee-like 5 (Drosophila)	Ypel5	0.84549	-1.06752793	-0.1044511
1452442_at	ubiquitin specific peptidase 13 (isopeptidase T-3)	Usp13	0.84551	-1.07609541	-0.1058221
1453111_a_at	solute carrier family 25, member 39	Slc25a39	0.84551	1.0573212	0.07754116
1421989_s_at	3'-phosphoadenosine 5'-phosphosulfate synthase 2	Papss2	0.84552	1.07025488	0.09717718
1450768_at	discs, large homolog 1 (Drosophila)	Dlg1	0.84554	1.06005634	0.08045306
1423914_at	RIKEN cDNA C630004H02 gene	C630004H02Rik	0.84554	1.06701109	0.09317861
1426987_at	RIKEN cDNA 5430417L22 gene	5430417L22Rik	0.84555	-1.08097324	-0.1257845
1441797_at	---	---	0.84558	-1.07525872	-0.1107008
1420755_a_at	Parkinson disease (autosomal recessive, juvenile) 2, parkin	Park2	0.8456	1.07527121	0.10324563
1454506_at	RIKEN cDNA 8430432A02 gene	8430432A02Rik	0.84561	1.08033965	0.10891283
1448355_at	protease, serine, 16 (thymus)	Prss16	0.84561	1.06830079	0.09473141
1426575_at	sphingomyelin synthase 1	Sgms1	0.84561	1.06286683	0.08750539
1455036_s_at	NADH dehydrogenase (ubiquinone) 1, subcomplex unknown, 2	Ndufc2	0.84563	-1.04908517	-0.0720177
1443818_at	---	---	0.84563	1.09181397	0.11935556
1443170_at	cyclin M1	Cnnm1	0.84563	1.07928968	0.10253698
1448973_at	sulfotransferase family 1D, member 1	Sult1d1	0.84564	1.06576337	0.08950359
1416033_at	transmembrane protein 109	Tmem109	0.84564	-1.08505168	-0.1895683
1421119_at	kinesin family member 21B	Kif21b	0.84565	1.08166306	0.10992741
1454795_at	Cobl-like 1	Cobll1	0.84566	-1.07133742	-0.1016385
1425760_a_at	phosphatidylinositol transfer protein, membrane-associated 1	Pitpnm1	0.84566	1.0812307	0.10799522
1423034_at	thioredoxin domain containing 17	Txndc17	0.84566	1.05603285	0.07536738
1416658_at	frizzled-related protein	Frzb	0.84566	1.08204192	0.10896052
1427279_at	CAP-GLY domain containing linker protein family, member 4	Clip4	0.84566	-1.07548779	-0.1063921
1446607_at	Microtubule-associated protein 7	Mtap7	0.84566	-1.07338492	-0.1061468
1444248_at	reticulocalbin 2	Rcn2	0.84567	1.11071863	0.13406112
1448447_at	vacuolar protein sorting 28 (yeast)	Vps28	0.84568	1.05278155	0.07231223
1421411_at	proline-serine-threonine phosphatase-interacting protein 2	Pstpip2	0.84568	1.09282792	0.11712513
1427803_at	---	---	0.84568	1.07713037	0.10455034
1431425_a_at	regulation of nuclear pre-mRNA domain containing 2	Rprd2	0.84568	-1.0754726	-0.1127718
1452567_at	granule cell antiserum positive 8	Gcap8	0.84569	1.06712713	0.0926666
1439899_at	UDP-N-acetyl-alpha-D-galactosamine:polypeptide N-acetylgalact	Galnt13	0.84569	1.0809219	0.10103222
1432979_at	GATA zinc finger domain containing 2B	Gatad2b	0.84569	-1.07654087	-0.1162619
1460700_at	signal transducer and activator of transcription 3	Stat3	0.8457	1.05565244	0.07713159
1427234_at	vang-like 1 (van gogh, Drosophila)	Vangl1	0.84571	1.06669879	0.08950972
1425737_at	RIKEN cDNA 1500002F19 gene	1500002F19Rik	0.84571	-1.08135529	-0.13862
1439280_at	predicted gene 13112	Gm13112	0.84572	-1.07112408	-0.1000742
1420732_at	RWD domain containing 3	Rwdd3	0.84572	1.10562062	0.12863192
1427258_at	tripartite motif-containing 24	Trim24	0.84572	-1.05511325	-0.0820802
1432497_at	coiled-coil domain containing 93	Ccdc93	0.84574	1.08308314	0.11290686
1430697_at	Alport syndrome, mental retardation, midface hypoplasia and elli	Ammecr1	0.84574	-1.08672365	-0.1914929
1452264_at	tensin like C1 domain-containing phosphatase	Tenc1	0.84575	1.07263951	0.09718277

1424472_at	nucleolar protein family 6 (RNA-associated)	Nol6	0.84576	1.06621398	0.08548016
1458884_at	Pleckstrin homology domain containing, family A (phosphoinositid	Plekha1	0.84576	1.07341153	0.10168853
1443584_at	RIKEN cDNA 1110028C15 gene	1110028C15Rik	0.84576	-1.06213095	-0.0897696
1420860_at	integrin alpha 9	Itga9	0.84576	-1.07296193	-0.101989
1446780_at	---	---	0.84576	1.06770532	0.09431103
1451663_a_at	tripartite motif-containing 3	Trim3	0.84577	1.06850887	0.09444715
1416782_s_at	PRA1 domain family 2	Praf2	0.84578	1.05671221	0.07713032
1459298_at	---	---	0.84578	1.08121502	0.11002815
1434994_at	death effector domain-containing	Dedd	0.84578	1.07764572	0.10062108
1459366_at	RGM domain family, member B	Rgmb	0.84579	1.08099265	0.10779211
1448874_a_at	SH2B adaptor protein 1	Sh2b1	0.84579	1.06141237	0.08418892
1425001_at	ring finger protein 146	Rnf146	0.8458	1.11377101	0.13392284
1428350_at	cytosolic thiouridylase subunit 2 homolog (S. pombe)	Ctu2	0.8458	1.05634863	0.07808788
1445748_at	---	---	0.8458	-1.08166596	-0.1323247
1450411_at	family with sequence similarity 122, member A	Fam122a	0.84581	-1.06780217	-0.0954369
1426703_at	cyclin G associated kinase	Gak	0.84583	1.07005873	0.09225327
1448302_at	potassium channel tetramerisation domain containing 20	Kctd20	0.84584	1.05118644	0.07200981
1450627_at	progressive ankylosis	Ank	0.84588	-1.06465119	-0.0921631
1439867_at	U box domain containing 5	Ubox5	0.84589	1.08462353	0.11084208
1439150_x_at	GH regulated TBC protein 1	Grtp1	0.8459	-1.06456423	-0.0996422
1446799_at	---	---	0.84591	-1.06731232	-0.0966431
1435788_at	ARP10 actin-related protein 10 homolog (S. cerevisiae)	Actr10	0.84591	1.06771774	0.09412939
1450282_at	fibroblast growth factor 4	Fgf4	0.84591	1.06940021	0.09135383
1431054_at	LSM6 homolog, U6 small nuclear RNA associated (S. cerevisiae)	Lsm6	0.84592	1.05815439	0.07945145
1432946_at	RIKEN cDNA 5230400M06 gene	5230400M06Rik	0.84593	1.0733892	0.10168726
1439000_at	acyl-CoA synthetase bubblegum family member 2	Acsbg2	0.84593	1.12553535	0.14381012
1431590_at	RIKEN cDNA 4930521C21 gene	4930521C21Rik	0.84594	-1.08752943	-0.1415626
1420063_at	---	---	0.84595	1.10834664	0.13254678
1437351_at	CXXC finger 4	Cxxc4	0.84595	1.11149466	0.13287885
1430282_at	RIKEN cDNA 5730437N04 gene	5730437N04Rik	0.84597	-1.07441946	-0.1056734
1456801_at	---	---	0.84597	1.08391776	0.10837394
1427827_at	---	---	0.84597	1.11155014	0.13268683
1456816_at	---	---	0.84598	1.18516034	0.18041391
1416519_at	predicted gene 13611 /// ribosomal protein L36	Gm13611 /// Rpl36	0.84598	1.03813572	0.05043935
1447248_at	solute carrier family 39 (zinc transporter), member 12	Slc39a12	0.84598	1.08308619	0.11039943
1430317_at	ubiquitin-conjugating enzyme E2, J2 homolog (yeast)	Ube2j2	0.84598	-1.06928989	-0.0970381
1419846_at	---	---	0.84599	-1.08613607	-0.143873
1445833_at	lipase maturation factor 1	Lmf1	0.84599	-1.08147677	-0.1255334
1417511_at	Ly1 antibody reactive clone	Lyar	0.846	1.04862994	0.06839438
1459368_at	---	---	0.84601	-1.08151431	-0.1390299
1433785_at	myelin-associated oligodendrocytic basic protein	Mobp	0.84601	-1.07898267	-0.1131661
1420203_at	---	---	0.84601	1.06150445	0.08156068
1457205_at	N-ethylmaleimide sensitive fusion protein	Nsf	0.84603	-1.08440303	-0.1252658
1460329_at	UDP-Gal:betaGlcNAc beta 1,4-galactosyltransferase, polypeptide	B4galt6	0.84604	-1.06583635	-0.107987
1458450_at	zinc finger RNA binding protein	Zfr	0.84604	1.08304003	0.11052871
1450929_at	zinc finger protein 57	Zfp57	0.84604	1.18558537	0.17679401
1448944_at	neuropilin 1	Nrp1	0.84605	1.10842359	0.12967683
1420331_at	C-type lectin domain family 4, member e	Clec4e	0.84605	-1.07195219	-0.1037817
1458995_at	Expressed sequence AA408296	AA408296	0.84606	-1.06936318	-0.1008791
1459987_s_at	chaperonin containing Tcp1, subunit 3 (gamma)	Cct3	0.84606	1.04147035	0.05780372
1453266_at	zinc finger and BTB domain containing 4	Zbtb4	0.84606	-1.07485747	-0.1056383
1435213_at	NHL repeat containing 1	Nhlrc1	0.84607	1.10959056	0.13144279
1440239_at	family with sequence similarity 65, member A	Fam65a	0.84607	1.08339136	0.1092896
1448764_a_at	fatty acid binding protein 1, liver	Fabp1	0.84607	1.08006627	0.10183994
1427311_at	bromodomain PHD finger transcription factor	Bptf	0.84607	-1.08045543	-0.1139874
1444581_at	RAN GTPase activating protein 1	Rangap1	0.84607	1.07600647	0.10188697
1435671_at	mirror-image polydactyly gene 1 homolog (human)	Mipol1	0.84607	-1.07867569	-0.1113226
1433671_at	RIKEN cDNA A130022J15 gene	A130022J15Rik	0.84608	1.06868644	0.09450414
1454937_at	RIKEN cDNA B630005N14 gene	B630005N14Rik	0.84608	-1.06699466	-0.0994992
1427220_a_at	seminal vesicle secretory protein 5	Svs5	0.84608	1.13394092	0.1506001
1435081_at	synaptophysin-like protein	Sypl	0.84608	-1.06406521	-0.0907023
1434707_at	SET binding factor 1	Sbf1	0.84608	1.05978691	0.08034492
1429288_x_at	syntaxin 18	Stx18	0.84608	1.07887973	0.10384533
1442861_at	---	---	0.84609	-1.06265281	-0.0883171
1427737_a_at	RIKEN cDNA 1200011M11 gene	1200011M11Rik	0.84609	1.05494871	0.07663178
1454497_at	RIKEN cDNA 5230401M06 gene	5230401M06Rik	0.84609	1.11996222	0.14000489
1422340_a_at	actin, gamma 2, smooth muscle, enteric	Actg2	0.84609	-1.08160492	-0.13777
1425022_at	ubiquitin specific peptidase 3	Usp3	0.84609	1.07130447	0.09415827
1458459_a_at	RIKEN cDNA E230029C05 gene	E230029C05Rik	0.84609	-1.07571829	-0.109844
1451615_at	carboxylesterase 4A	Ces4a	0.84609	-1.06856886	-0.09622
1443549_at	isoprenoid synthase domain containing	Ispd	0.8461	-1.08161196	-0.1259497
1416740_at	collagen, type V, alpha 1	Col5a1	0.8461	1.08917875	0.11350764
1457484_at	hypothetical D930050J11	D930050J11	0.8461	-1.07485992	-0.1046422
1433199_at	RIKEN cDNA B230114A03 gene	B230114A03Rik	0.8461	-1.07158852	-0.1128384
1431752_a_at	ubiquitin related modifier 1 homolog (S. cerevisiae)	Urm1	0.8461	1.06906971	0.08831602
1426596_a_at	survival motor neuron 1	Smn1	0.8461	1.0625641	0.08228745
1457841_at	hypothetical LOC100504701	LOC100504701	0.84611	-1.06796148	-0.0966472
1442472_at	Tetratricopeptide repeat domain 21A	Ttc21a	0.84611	-1.07432679	-0.1055269
1422757_at	solute carrier family 5 (neutral amino acid transporters, system A)	Slc5a4b	0.84611	-1.07698496	-0.1133425
1437302_at	adrenergic receptor, beta 2	Adrb2	0.84611	-1.07680942	-0.10697
1438129_at	WD repeat and SOCS box-containing 2	Wsb2	0.84611	-1.08064541	-0.1375416
1416440_at	CD164 antigen	Cd164	0.84611	-1.051074	-0.073805
1454499_at	RIKEN cDNA 4933431C10 gene	4933431C10Rik	0.84611	-1.07367305	-0.106787
1453253_a_at	RNA pseudouridylate synthase domain containing 1	Rpusd1	0.84611	-1.06190054	-0.0953458
1419585_at	retinitis pigmentosa 2 homolog (human)	Rp2h	0.84611	-1.08326708	-0.1310401
1459956_at	---	---	0.84612	1.14309554	0.15146612
1427745_x_at	predicted gene 4736 /// predicted gene 8882 /// proline-rich prot	Gm4736 /// Gm8882 /// I	0.84612	-1.0850204	-0.1324602
1437689_x_at	clusterin	Clu	0.84612	-1.07476113	-0.125892
1437979_at	Zinc finger, CCHC domain containing 2	Zcchc2	0.84612	-1.07028194	-0.1018828
1424517_at	coiled-coil domain containing 12	Ccdc12	0.84613	1.08533435	0.1063415
1446436_at	---	---	0.84613	1.07973586	0.10765844
1429228_at	RIKEN cDNA 4930534B04 gene	4930534B04Rik	0.84613	1.06973021	0.09480421
1440415_at	---	---	0.84614	1.11624951	0.13717001
1418284_at	vacuolar protein sorting 72 (yeast)	Vps72	0.84614	1.06665746	0.08660759

1448685_at	RIKEN cDNA 2900010M23 gene	2900010M23Rik	0.84615	1.068649	0.08863541
1451139_at	solute carrier family 39 (zinc transporter), member 4	Slc39a4	0.84615	1.05899932	0.08090585
1447716_x_at	transmembrane protein 229B	Tmem229b	0.84615	1.05687929	0.07591791
1448430_a_at	nascent polypeptide-associated complex alpha polypeptide	Naca	0.84615	-1.02856173	-0.040825
1441887_x_at	predicted gene 6377	Gm6377	0.84615	1.04968462	0.06882443
1420379_at	solute carrier organic anion transporter family, member 1a1	Slco1a1	0.84615	-1.06892651	-0.0973318
1416492_at	cyclin E1	Ccne1	0.84616	1.0685172	0.08752169
1424526_a_at	TDP-glucose 4,6-dehydratase	Tgds	0.84616	1.06278192	0.08756087
1438353_at	---	---	0.84616	-1.08478304	-0.1405574
1431109_at	proline rich 16	Prr16	0.84616	1.06227213	0.08587975
1453368_at	RIKEN cDNA 2310003H01 gene	2310003H01Rik	0.84617	1.07860917	0.10555935
1444366_at	---	---	0.84617	-1.0732414	-0.115363
1457556_at	plasma membrane Ca2+ pump interacting protein	LOC100216343	0.84618	-1.0691763	-0.1058562
1434321_at	ATR interacting protein /// three prime repair exonuclease 1	Atrip /// Trex1	0.84618	1.08955125	0.11349267
1445245_at	RIKEN cDNA B130065D12 gene	B130065D12Rik	0.8462	-1.09043053	-0.1619836
1457935_at	---	---	0.8462	-1.07538965	-0.1055703
1452326_at	ATPase, Ca++ transporting, type 2C, member 2	Atp2c2	0.8462	1.07311489	0.09594324
1450739_at	transducin (beta)-like 1X-linked receptor 1	Tbl1xr1	0.84621	-1.06358662	-0.0917367
1436851_at	protein kinase N1 /// prostaglandin E receptor 1 (subtype EP1)	Pkn1 /// Ptger1	0.84622	1.07396731	0.09699497
1416377_at	programmed cell death 7	Pdcd7	0.84622	1.05331327	0.07423014
1431584_at	RIKEN cDNA 1110028F18 gene	1110028F18Rik	0.84623	1.08766457	0.11373431
1446210_at	---	---	0.84627	-1.07830629	-0.1099287
1446440_at	---	---	0.84628	1.08168226	0.11109194
1434184_s_at	mitogen-activated protein kinase kinase kinase kinase 4	Map4k4	0.84631	-1.05570783	-0.0808449
1450947_at	RIKEN cDNA 2610528J11 gene	2610528J11Rik	0.84632	1.10274775	0.12575945
1416857_at	stromal cell derived factor 2	Sdf2	0.84632	1.05833034	0.08013911
1443418_at	---	---	0.84638	-1.07415776	-0.1084432
1419487_at	myosin binding protein H	Mybph	0.84654	1.08106457	0.10876363
1420922_at	ubiquitin specific peptidase 9, X chromosome	Usp9x	0.8466	-1.06827373	-0.1130814
1429459_at	sema domain, immunoglobulin domain (Ig), short basic domain, s	Sema3d	0.84661	1.06395151	0.08920719
1417252_at	5',3'-nucleotidase, cytosolic	Nt5c	0.84664	1.06070137	0.08133034
1419508_at	receptor (TNFRSF)-interacting serine-threonine kinase 1	Ripk1	0.84666	1.06814074	0.09456352
1417668_at	reticulon 4 interacting protein 1	Rtn4ip1	0.84668	1.07239783	0.0961881
1449439_at	Kruppel-like factor 7 (ubiquitous)	Klf7	0.84669	1.09499486	0.1174696
1443284_at	---	---	0.84671	1.08029502	0.10693851
1417215_at	RAB27b, member RAS oncogene family	Rab27b	0.84671	1.07131179	0.09410327
1427230_at	RIKEN cDNA B930041F14 gene	B930041F14Rik	0.84672	1.07150892	0.09315936
1445806_at	Component of oligomeric golgi complex 5	Cog5	0.84673	1.09069465	0.11475345
1425516_at	O-linked N-acetylglucosamine (GlcNAc) transferase (UDP-N-acety	Ogt	0.84673	1.09842728	0.11532563
1458501_at	vesicle-associated membrane protein, associated protein B and C	Vapb	0.84673	-1.07493097	-0.104545
1423775_s_at	protein regulator of cytokinesis 1	Prc1	0.84675	1.05264772	0.07297759
1444311_at	---	---	0.84675	1.08373272	0.11254947
1440996_at	---	---	0.84675	-1.08875745	-0.171817
1435006_s_at	ATP-binding cassette, sub-family B (MDR/TAP), member 7	Abcb7	0.84676	-1.05061298	-0.0724556
1421897_at	ELK1, member of ETS oncogene family	Elk1	0.84676	1.08186147	0.10625107
1455567_at	cyclin-dependent kinase 12 /// hypothetical LOC100502717	Cdk12 /// LOC100502717	0.84676	1.09746678	0.11925587
1442816_at	heparan sulfate (glucosamine) 3-O-sulfotransferase 3B1	Hs3st3b1	0.84677	1.0802973	0.10738304
1460095_at	GIY-YIG domain containing 2	Giyd2	0.84677	-1.07821099	-0.124758
1448751_at	adaptor-related protein complex 3, mu 2 subunit	Ap3m2	0.84678	-1.07907222	-0.1207969
1430855_at	collagen, type XX, alpha 1	Col20a1	0.84678	1.08311587	0.10950951
1429125_at	gametogenetin binding protein 1 /// zinc finger and BTB domain c	Ggnbp1 /// Zbtb9	0.84678	1.05788175	0.0808499
1424359_at	5-oxoprolinase (ATP-hydrolysing)	Oplah	0.84678	-1.07085771	-0.1038853
1456376_at	---	---	0.84678	1.07342517	0.09947307
1445193_x_at	DNA segment, Chr 8, ERATO Doi 575, expressed	D8Ertd575e	0.84678	1.08313738	0.10462344
1447965_at	expressed sequence C80012	C80012	0.84679	-1.06554945	-0.0921734
1448498_at	ribosomal protein S6 kinase, polypeptide 4	Rps6ka4	0.84679	1.06153447	0.08230173
1432422_at	RIKEN cDNA 1700063K16 gene	1700063K16Rik	0.84679	1.09552526	0.12196398
1437787_at	leucine rich repeat transmembrane neuronal 2	Lrrtm2	0.8468	1.06439578	0.08796482
1435513_at	5-hydroxytryptamine (serotonin) receptor 2C	Htr2c	0.8468	1.0647484	0.08955772
1455534_s_at	oxysterol binding protein-like 11	Osbpl11	0.8468	1.05933805	0.08230753
1457490_at	---	---	0.8468	-1.07453703	-0.1099506
1454449_at	Rab9 effector protein with kelch motifs	Rabepk	0.8468	-1.07719469	-0.1085294
1419378_a_at	FXYD domain-containing ion transport regulator 2	Fxyd2	0.8468	-1.07414191	-0.1045373
1419159_at	golgi autoantigen, golgin subfamily a, 3	Golga3	0.8468	1.06920468	0.09372528
1423514_at	glycerol kinase-like 1	Gykl1	0.84681	-1.07244375	-0.1022614
1450626_at	mannosidase, beta A, lysosomal	Manba	0.84681	-1.04751438	-0.067249
1441457_at	---	---	0.84681	1.08643913	0.11181389
1459507_at	---	---	0.84681	-1.06975613	-0.1008162
1457594_at	---	---	0.84681	1.10390871	0.12543441
1458445_at	---	---	0.84682	1.06617747	0.09229922
1457917_at	Lymphocyte protein tyrosine kinase	Lck	0.84682	1.12283431	0.14085166
1456731_x_at	polymerase (RNA) III (DNA directed) polypeptide K	Polr3k	0.84682	-1.06307969	-0.1049698
1438126_at	exocyst complex component 5	Exoc5	0.84682	1.06740371	0.09275142
1452798_s_at	mitogen-activated protein kinase 1 interacting protein 1	Mapk1ip1	0.84682	-1.05958257	-0.0879158
1443330_at	DNA segment, Chr 1, ERATO Doi 646, expressed	D1Ertd646e	0.84683	-1.08346836	-0.1287167
1422394_at	---	---	0.84683	1.10302617	0.12857281
1421007_at	collagen, type IV, alpha 6	Col4a6	0.84683	-1.07680074	-0.1084236
1421723_at	protocadherin beta 18	Pcdhb18	0.84683	-1.06951054	-0.1122269
1429354_at	thioesterase superfamily member 5	Them5	0.84683	1.07155938	0.09907633
1460628_at	essential meiotic endonuclease 1 homolog 2 (S. pombe)	Eme2	0.84683	1.09925649	0.12250514
1457013_at	Ral GTPase activating protein, alpha subunit 1	Ralgapa1	0.84683	1.08419353	0.11088456
1439687_at	RAB14, member RAS oncogene family	Rab14	0.84684	1.11527199	0.13460268
1455021_at	gamma-aminobutyric acid (GABA) B receptor, 1	Gabbr1	0.84684	-1.05375536	-0.0760138
1458126_at	RIKEN cDNA D030041H20 gene	D030041H20Rik	0.84684	-1.07490139	-0.1111029
1457703_at	calcium channel, voltage-dependent, alpha 2/delta subunit 4	Cacna2d4	0.84684	-1.0771931	-0.1109496
1429493_at	methyltransferase like 4	Mettl4	0.84684	-1.06250542	-0.0882443
1455149_at	SH3 domain containing ring finger 1	Sh3rf1	0.84684	-1.06709872	-0.0957132
1425846_a_at	calneuron 1	Caln1	0.84684	1.08161752	0.10984253
1428777_at	sprouty protein with EVH-1 domain 1, related sequence	Spred1	0.84684	1.06870992	0.09212753
1453556_x_at	CD99 antigen	Cd99	0.84684	-1.07625638	-0.1248494
1423757_x_at	insulin-like growth factor binding protein 4	Igfbp4	0.84685	1.06588048	0.09071631
1456509_at	RIKEN cDNA 1110032F04 gene	1110032F04Rik	0.84685	1.06558535	0.09139264
1451303_at	cDNA sequence BC002230	BC002230	0.84686	1.05427619	0.07593063
1440744_at	---	---	0.84686	-1.0679561	-0.0975593

1432634_at	RIKEN cDNA 4933429H19 gene	4933429H19Rik	0.84686	1.12241704	0.14015895
1426571_at	anoctamin 1, calcium activated chloride channel	Ano1	0.84686	1.07545723	0.10446226
1445818_at	RIKEN cDNA 4930544M13 gene	4930544M13Rik	0.84687	1.07173635	0.09616809
1457742_at	DNA segment, Chr 9, Wayne State University 74, expressed	D9Wsu74e	0.84687	1.07549	0.10389998
1425297_at	zinc finger protein 825	Zfp825	0.84687	1.08285874	0.10573177
1428166_at	congenital dyserythropoietic anemia, type I (human)	Cdan1	0.84687	1.07710809	0.10056403
1441269_at	---	---	0.84687	1.06825898	0.09489027
1429389_at	SET domain and mariner transposase fusion gene	Setmar	0.84688	-1.06086918	-0.0855882
1454221_a_at	kinesin family member 2C	Kif2c	0.84688	1.07446359	0.09578588
1443926_at	---	---	0.84688	1.05230915	0.07331849
1454730_at	transmembrane anterior posterior transformation 1	Tapt1	0.84688	1.08643549	0.11009872
1447294_at	F-box and WD-40 domain protein 11	Fbxw11	0.84689	-1.07500194	-0.1085119
1444144_at	---	---	0.84689	-1.07806673	-0.1125294
1456755_at	trafficking protein, kinesin binding 1	Trak1	0.8469	-1.07393641	-0.1057038
1453009_at	carboxypeptidase M	Cpm	0.84691	1.08049392	0.10749553
1438475_at	cDNA sequence AB124611	AB124611	0.84691	-1.07230872	-0.1014772
1420395_a_at	kinesin family member 9	Kif9	0.84691	1.08028869	0.1082288
1438991_x_at	protein phosphatase 2 (formerly 2A), regulatory subunit A (PR 65)	Ppp2r1a	0.84691	-1.04479305	-0.0656925
1460185_at	keratin 85	Krt85	0.84692	-1.07745662	-0.1222679
1440511_at	---	---	0.84692	-1.07638292	-0.1125409
1443969_at	insulin receptor substrate 2	Irs2	0.84692	-1.07334123	-0.1068546
1443471_at	zinc finger and BTB domain containing 20	Zbtb20	0.84693	1.07432659	0.10186122
1439850_at	cDNA sequence BC066028	BC066028	0.84693	1.06565832	0.09053937
1443775_x_at	zinc finger and AT hook domain containing	Zfat	0.84693	-1.07741279	-0.1201877
1443577_at	hypothetical LOC100503490	LOC100503490	0.84694	1.07515813	0.10198482
1446606_at	RIKEN cDNA 1700028E10 gene	1700028E10Rik	0.84694	1.08901331	0.11537679
1417793_at	immunity-related GTPase family M member 2	Irgm2	0.84695	1.09728472	0.12215335
1429753_at	neurexophilin 4	Nxph4	0.84696	-1.06065716	-0.0875343
1457744_at	DEAD (Asp-Glu-Ala-Asp) box polypeptide 46	Ddx46	0.84697	-1.0705941	-0.1272781
1422192_at	gap junction protein, alpha 5	Gja5	0.84697	1.08774067	0.11796452
1452945_at	zinc finger protein 157	Zfp157	0.84698	1.0573418	0.07966083
1458626_at	Nitric oxide synthase 1, neuronal	Nos1	0.84698	1.09911354	0.12534133
1425262_at	CCAAT/enhancer binding protein (C/EBP), gamma	Cebpg	0.84699	1.05365091	0.07519691
1442697_at	---	---	0.84699	1.10873598	0.12865568
1431157_at	RIKEN cDNA 1700025I17 gene	1700025I17Rik	0.847	-1.07076682	-0.1008397
1428904_at	AMME chromosomal region gene 1-like	Ammecr1l	0.84701	1.06464527	0.0877777
1440513_at	expressed sequence C80258	C80258	0.84701	1.08271658	0.11095003
1420791_x_at	spermatogenesis associated glutamate (E)-rich protein 4f	Speer4f	0.84702	-1.0652885	-0.0933814
1446761_at	DNA segment, Chr 8, ERATO Doi 56, expressed	D8Ertd56e	0.84702	1.08952172	0.11267656
1431967_at	RIKEN cDNA 1700006A11 gene	1700006A11Rik	0.84703	-1.06863114	-0.1124489
1436398_at	---	---	0.84705	-1.07457319	-0.104273
1452167_at	RIKEN cDNA 2810407C02 gene	2810407C02Rik	0.84705	-1.05433031	-0.0822171
1449681_at	Hepatoma-derived growth factor	Hdgf	0.84706	-1.08119552	-0.1333078
1450549_s_at	ELK4, member of ETS oncogene family	Elk4	0.84706	-1.07914001	-0.122005
1436148_at	Tenascin R	Tnr	0.84706	1.05321321	0.0740223
1436649_at	IKAROS family zinc finger 3	Ikzf3	0.84707	1.10709174	0.12673125
1421449_at	CUB and Sushi multiple domains 1	Csmd1	0.84708	1.07196528	0.09642249
1439408_a_at	mediator complex subunit 1	Med1	0.84708	1.08084949	0.10836833
1445105_at	---	---	0.8471	1.10909766	0.12949164
1456440_s_at	ST8 alpha-N-acetyl-neuraminide alpha-2,8-sialyltransferase 6	St8sia6	0.84714	1.08820268	0.11457866
1428967_at	insulin-like growth factor I receptor	Igf1r	0.84717	1.08392144	0.10714937
1443465_at	---	---	0.84719	-1.07189839	-0.1007391
1415756_a_at	SNAP-associated protein	Snapin	0.84729	1.05867369	0.08003157
1419987_at	---	---	0.84734	1.11472198	0.13506351
1445736_at	---	---	0.84735	1.10767875	0.12976904
1418362_at	zinc finger protein 42	Zfp42	0.84735	-1.0388871	-0.0551873
1448532_at	prolactin family8, subfamily a, member 9	Prl8a9	0.84737	-1.07348446	-0.1139526
1424079_x_at	ubiquitin related modifier 1 homolog (S. cerevisiae)	Urm1	0.84739	1.06625815	0.085982
1452889_at	phospholysine phosphohistidine inorganic pyrophosphate phosph	Lhpp	0.8474	-1.07509336	-0.1201821
1435444_at	activating transcription factor 6	Atf6	0.84749	-1.06155753	-0.0861877
1416582_a_at	BCL2-associated agonist of cell death	Bad	0.8475	1.06241276	0.08453594
1422104_at	visual system homeobox 1 homolog (zebrafish)	Vsx1	0.8475	1.08699991	0.11402185
1437573_at	---	---	0.84752	1.08362646	0.10909352
1447764_at	potassium voltage-gated channel, Shal-related family, member 2	Kcnd2	0.84752	-1.07146713	-0.1015926
1432682_at	RIKEN cDNA 5530400K19 gene	5530400K19Rik	0.84753	1.07580045	0.0995191
1425321_a_at	calmin	Clmn	0.84754	1.07195894	0.09798519
1446461_at	---	---	0.84757	1.08731435	0.1127041
1438049_at	RIKEN cDNA A430108E01 gene /// hypothetical LOC100504289	A430108E01Rik /// LOC10	0.84758	-1.06154309	-0.0948055
1420234_at	---	---	0.84766	1.08130829	0.10494638
1449860_at	HIG1 domain family, member 1B	Higd1b	0.84766	1.12366023	0.14174199
1459491_at	DNA segment, Chr 12, ERATO Doi 673, expressed	D12Ertd673e	0.84767	1.07864913	0.10921984
1452760_at	RIKEN cDNA 2510012J08 gene	2510012J08Rik	0.84768	1.06266148	0.08295901
1432852_at	phosphatase and actin regulator 1	Phactr1	0.84769	1.07960477	0.10606248
1421879_at	myotubularin related protein 1	Mtmr1	0.84773	-1.07100438	-0.1033253
1458846_at	---	---	0.84777	1.05608991	0.07850348
1453558_at	EF-hand calcium binding domain 10	Efcab10	0.84778	1.0753957	0.10340087
1447230_at	WD repeat domain 92	Wdr92	0.84779	-1.07428636	-0.1048746
1437059_at	SRY-box containing gene 21	Sox21	0.8478	1.06800023	0.09431861
1450440_at	glial cell line derived neurotrophic factor family receptor alpha 1	Gfra1	0.84781	1.08032543	0.10586212
1446524_at	---	---	0.84782	1.08165211	0.10615442
1417213_a_at	RNA binding motif protein 6	Rbm6	0.84782	-1.06052003	-0.0908016
1453031_at	transmembrane protein 174	Tmem174	0.84782	1.09159472	0.11637581
1426087_at	icos ligand	Icosl	0.84782	-1.07867423	-0.1201316
1450711_at	bromodomain containing 4	Brd4	0.84785	-1.05217454	-0.0763033
1459801_at	UDP-Gal:betaGlcNAc beta 1,3-galactosyltransferase, polypeptide	B3galt5	0.84786	1.08382819	0.1081693
1437963_at	RIKEN cDNA C530045E16 gene	C530045E16Rik	0.84786	-1.0769503	-0.1124365
1437859_x_at	eukaryotic translation initiation factor 5A	Eif5a	0.84787	-1.0410439	-0.0591832
1444328_at	clathrin, light polypeptide (Lca)	Clta	0.84788	1.08455031	0.11013713
1426118_a_at	translocase of outer mitochondrial membrane 40 homolog (yeast	Tomm40	0.8479	1.06664403	0.08571501
1428632_at	RIKEN cDNA 1810006K21 gene	1810006K21Rik	0.84791	-1.06742388	-0.098472
1424308_at	solute carrier family 24 (sodium/potassium/calcium exchanger), r	Slc24a3	0.84791	-1.07618735	-0.1182703
1415811_at	ubiquitin-like, containing PHD and RING finger domains, 1	Uhrf1	0.84792	-1.05144949	-0.0737309
1434988_x_at	aldehyde dehydrogenase 2, mitochondrial	Aldh2	0.84793	-1.07636234	-0.1076153
1428912_at	tubulin tyrosine ligase-like family, member 4	Ttll4	0.84794	-1.07543478	-0.1065234

1457598_at	Glutaredoxin 3	Glrx3	0.84795	-1.08106114	-0.145004
1451891_a_at	dysferlin	Dysf	0.84796	-1.07932197	-0.1264564
1415702_a_at	C-terminal binding protein 1	Ctbp1	0.84796	1.05330538	0.07305262
1459391_at	ATP-binding cassette, sub-family A (ABC1), member 5	Abca5	0.84797	1.07213867	0.09971257
1433345_s_at	glyceraldehyde-3-phosphate dehydrogenase pseudogene	4930448K20Rik	0.84798	-1.0762145	-0.1126446
1449028_at	ras homolog gene family, member U	Rhou	0.84798	-1.06872881	-0.1084616
1438407_at	dermatan sulfate epimerase-like	Dsel	0.84798	1.11567374	0.13589995
1456252_x_at	adaptor-related protein complex 1, sigma 2 subunit	Ap1s2	0.84798	1.06257955	0.0873642
1415725_at	RRN3 RNA polymerase I transcription factor homolog (yeast)	Rrn3	0.848	-1.06273113	-0.1054824
1422269_at	insulinoma-associated 2	Insm2	0.84801	-1.08078847	-0.1297993
1421959_s_at	adenylate cyclase 3	Adcy3	0.84801	1.0659092	0.09112237
1458358_at	pantothenate kinase 2	Pank2	0.84802	-1.06748497	-0.0961239
1450661_x_at	nuclear factor I/C	Nfic	0.84803	1.07786588	0.10492081
1432107_at	RIKEN cDNA 2310010M20 gene	2310010M20Rik	0.84804	1.06995211	0.09430977
1450543_at	myosin 1H	Myo1h	0.84805	1.12073623	0.13958263
1460658_at	adaptor protein complex AP-1, gamma 1 subunit	Ap1g1	0.84808	-1.06537889	-0.0999123
1436933_at	---	---	0.84839	1.14955553	0.16105236
1450602_at	vomeronasal 1 receptor 14	Vmn1r14	0.84839	-1.06531714	-0.0947831
1460508_at	mitochondrial ribosomal protein L1	Mrpl1	0.84843	1.09187828	0.11601408
1432758_at	RIKEN cDNA 2900011L18 gene	2900011L18Rik	0.84843	-1.08042356	-0.1230121
1436731_at	zinc finger protein 385B	Zfp385b	0.8487	1.07595653	0.10362493
1421549_at	protocadherin beta 15	Pcdhb15	0.8487	-1.06961043	-0.0988086
1419926_at	Thioredoxin-related transmembrane protein 3	Tmx3	0.84871	-1.07915191	-0.1271824
1432332_a_at	nudix (nucleoside diphosphate linked moiety X)-type motif 19	Nudt19	0.84873	1.05763119	0.08006914
1426359_at	zinc finger CCCH type containing 11A	Zc3h11a	0.84877	-1.069726	-0.126089
1445535_at	expressed sequence AA407107	AA407107	0.84877	-1.0773382	-0.1168959
1434271_at	glucosidase beta 2	Gba2	0.84878	1.06302547	0.08546441
1425352_at	REST corepressor 3	Rcor3	0.8488	1.07323457	0.09938107
1420674_at	phospholipase A2, group IIE	Pla2g2e	0.8488	1.09049471	0.11623532
1417830_at	structural maintenance of chromosomes 1A	Smc1a	0.84881	1.05532086	0.07499521
1428530_x_at	ribosomal protein S24	Rps24	0.84881	-1.03151737	-0.0456585
1417414_at	septin 3	Sep-03	0.84882	-1.0714186	-0.1082815
1445753_at	---	---	0.84883	-1.07282583	-0.1064447
1450485_at	SRY-box containing gene 3	Sox3	0.84883	1.07085518	0.09738303
1448503_at	myeloid cell leukemia sequence 1	Mcl1	0.84884	-1.04295881	-0.0627653
1438280_at	RIKEN cDNA 1700023L04 gene	1700023L04Rik	0.84893	1.07380751	0.10266472
1426895_at	zinc finger protein 191	Zfp191	0.84895	-1.0636192	-0.0952457
1429684_at	tocopherol (alpha) transfer protein-like	Ttpal	0.84896	-1.07624363	-0.118662
1453595_at	RIKEN cDNA 2900064B18 gene	2900064B18Rik	0.84897	1.07451983	0.10093026
1441489_at	Riken cDNA C130023C23 gene	C130023C23Rik	0.849	-1.06940644	-0.097944
1453116_at	zinc finger protein 689	Zfp689	0.84901	1.06392374	0.08662697
1424101_at	heterogeneous nuclear ribonucleoprotein L	Hnrnpl	0.84902	1.06297915	0.08152222
1446550_at	G1 to S phase transition 1	Gspt1	0.84906	-1.07647287	-0.1266887
1428468_at	RIKEN cDNA 3110043O21 gene	3110043O21Rik	0.84906	1.06790917	0.08963058
1455803_at	solute carrier organic anion transporter family, member 4a1	Slco4a1	0.84927	1.06454566	0.08768475
1459329_at	tetratricopeptide repeat domain 7	Ttc7	0.84929	-1.07378014	-0.1035407
1431409_at	RIKEN cDNA C030015A19 gene	C030015A19Rik	0.84933	-1.08447883	-0.1456696
1450469_at	differential display clone 8	Ddc8	0.84935	-1.07183248	-0.1095422
1440311_at	sorbin and SH3 domain containing 1	Sorbs1	0.84937	1.09115902	0.11666593
1427103_at	pleckstrin homology domain containing, family O member 2	Plekho2	0.84937	-1.06430223	-0.0919737
1433738_at	PAP associated domain containing 5	Papd5	0.8494	-1.05067875	-0.0734311
1442647_at	Yip1 domain family, member 1	Yipf1	0.84942	-1.07626576	-0.1168852
1447293_x_at	ARP1 actin-related protein 1 homolog B, centractin beta (yeast)	Actr1b	0.84942	1.13317289	0.14571172
1438555_x_at	mucin 4	Muc4	0.84944	1.07889079	0.09851094
1452721_a_at	coiled-coil domain containing 53	Ccdc53	0.84945	1.06795992	0.08879461
1434053_x_at	ATP synthase, H+ transporting, mitochondrial F1F0 complex, subu	Atp5k	0.84945	1.06336912	0.08222829
1459347_at	predicted gene 11190	Gm11190	0.84946	-1.07427644	-0.1067732
1435686_at	family with sequence similarity 149, member B	Fam149b	0.84947	1.0783925	0.1041175
1424900_at	solute carrier family 29 (nucleoside transporters), member 4	Slc29a4	0.84948	-1.06369243	-0.0902946
1438415_s_at	Yip1 domain family, member 2	Yipf2	0.8495	-1.06856717	-0.0997531
1446165_at	predicted gene 6211	Gm6211	0.84951	-1.06725509	-0.1008563
1420816_at	tyrosine 3-monooxygenase/tryptophan 5-monooxygenase activa	Ywhag	0.84952	-1.0534051	-0.0777589
1445712_at	---	---	0.84952	-1.07606	-0.1130273
1448947_at	RIKEN cDNA 2810004N23 gene	2810004N23Rik	0.84953	1.05950651	0.0793727
1438988_x_at	hematological and neurological expressed sequence 1	Hn1	0.84954	1.04917585	0.06880352
1457539_at	DNA segment, Chr 10, ERATO Doi 709, expressed	D10Ertd709e	0.84954	1.17064234	0.17258124
1459279_at	---	---	0.84955	1.07988574	0.1064348
1423834_s_at	golgi associated, gamma adaptin ear containing, ARF binding prot	Gga1	0.84957	1.05690724	0.0782918
1419656_at	solute carrier family 25, member 36	Slc25a36	0.84958	-1.05605941	-0.0820644
1431553_at	predicted gene 15270	Gm15270	0.84958	-1.07198384	-0.104158
1426991_at	RIKEN cDNA 1810048J11 gene	1810048J11Rik	0.84958	1.09873676	0.11781769
1446498_at	interleukin 20 receptor, alpha	Il20ra	0.84958	1.08748236	0.11398464
1453989_at	syntaxin binding protein 4	Stxbp4	0.8496	1.08042537	0.10886507
1439530_a_at	protein phosphatase 1, regulatory (inhibitor) subunit 13 like	Ppp1r13l	0.84961	1.07224668	0.09605422
1446190_at	doublecortin-like kinase 1	Dclk1	0.84962	1.09004296	0.11464387
1437140_at	RIKEN cDNA 4930412F15 gene	4930412F15Rik	0.84962	1.09794076	0.1228928
1452224_at	microrchidia 3	Morc3	0.84962	-1.05748776	-0.086425
1419636_at	RIKEN cDNA 4833420G17 gene	4833420G17Rik	0.84963	1.06838676	0.09191946
1459196_at	---	---	0.84963	1.07198505	0.09834374
1422981_at	DiGeorge syndrome critical region gene 8	Dgcr8	0.84963	-1.06877364	-0.1045892
1427502_at	a disintegrin and metallopeptidase domain 4	Adam4	0.84963	-1.07096988	-0.1033778
1455530_at	immunoglobulin heavy variable V14-2	Ighv14-2	0.84966	-1.07132473	-0.1067664
1443665_at	DENN/MADD domain containing 5B	Dennd5b	0.84966	1.09046378	0.11545927
1440992_at	RIKEN cDNA 3110052M02 gene	3110052M02Rik	0.84966	1.08291813	0.11111682
1460235_at	scavenger receptor class B, member 2	Scarb2	0.84967	1.06728303	0.08883085
1431139_at	chromodomain protein, Y chromosome-like 2	Cdyl2	0.84968	-1.07388284	-0.1053638
1426570_a_at	fyn-related kinase	Frk	0.84969	-1.05653639	-0.0794811
1428564_at	zinc finger protein 579	Zfp579	0.8497	1.06444876	0.08971722
1447937_a_at	RIKEN cDNA 4933409K07 gene /// predicted gene 10590 /// pred	4933409K07Rik /// Gm10	0.8497	-1.07824725	-0.115634
1418058_at	EGF, latrophilin seven transmembrane domain containing 1	Eltd1	0.84971	-1.08004631	-0.1225382
1428920_at	high mobility group 20A	Hmg20a	0.84971	1.0791108	0.10619564
1417497_at	ceruloplasmin	Cp	0.84971	1.07497013	0.10328844
1437131_x_at	mitochondrial ribosomal protein L11	Mrpl11	0.84971	-1.0509972	-0.0731566
1452708_a_at	Luc7 homolog (S. cerevisiae)-like	Luc7l	0.84971	-1.05783425	-0.0821913

1441283_at	---	---	0.84973	1.08706311	0.1098392
1435524_at	small nucleolar RNA host gene 8	Snhg8	0.84974	1.05837677	0.07702828
1443509_at	---	---	0.84974	1.13752587	0.15307359
1446438_at	ubiquitin protein ligase E3 component n-recognin 1	Ubr1	0.84974	-1.0746437	-0.1202025
1438527_at	predicted gene 5879 /// ribosomal protein L3	Gm5879 /// Rpl3	0.84974	1.06368407	0.08558259
1433503_at	prostaglandin reductase 2	Ptgr2	0.84976	-1.05914301	-0.0828981
1418002_at	HIG1 domain family, member 2A	Higd2a	0.84978	1.05931054	0.07914779
1460511_at	plakophilin 2	Pkp2	0.84978	1.07987861	0.10543223
1444662_at	phospholipase A2, group IVC (cytosolic, calcium-independent)	Pla2g4c	0.84982	1.0717254	0.09771059
1436730_at	chloride channel CLIC-like 1	Clcc1	0.84983	1.07781805	0.10547654
1433949_x_at	phospholipase A2, group IB, pancreas	Pla2g1b	0.84987	-1.06992795	-0.1006003
1433721_x_at	ribosomal protein S21	Rps21	0.84987	1.02854919	0.03987313
1436374_x_at	F11 receptor	F11r	0.8499	-1.07501562	-0.1062946
1442641_at	---	---	0.8499	-1.08056457	-0.1245382
1418952_at	taxilin beta	Txlnb	0.84996	-1.07097104	-0.0989523
1420792_at	RIKEN cDNA 4930433N12 gene	4930433N12Rik	0.84996	1.12223084	0.13671288
1419811_at	adenylate cyclase 9	Adcy9	0.84997	1.12759322	0.14381931
1459706_at	---	---	0.84998	1.11907347	0.13889406
1441424_at	---	---	0.84998	1.10962233	0.12836921
1429744_at	RIKEN cDNA 4931407G18 gene	4931407G18Rik	0.84999	1.0747933	0.10318726
1451495_at	WW domain containing adaptor with coiled-coil	Wac	0.84999	1.06459806	0.08470671
1419673_at	sparc/osteonectin, cwcv and kazal-like domains proteoglycan 1	Spock1	0.84999	-1.0733078	-0.1060163
1439849_at	predicted gene 12824	Gm12824	0.84999	-1.07119075	-0.1003625
1440681_at	cholinergic receptor, nicotinic, alpha polypeptide 7	Chrna7	0.85	-1.06453255	-0.0905312
1420863_at	dynactin 4	Dctn4	0.85	1.07752353	0.10422218
1447877_x_at	DNA methyltransferase (cytosine-5) 1	Dnmt1	0.85	1.06777967	0.08859345
1422944_a_at	diaphanous homolog 3 (Drosophila)	Diap3	0.85001	-1.05626039	-0.0817156
1417276_at	tubby-like protein 2	Tulp2	0.85002	1.07657986	0.10169836
1418355_at	nucleobindin 2	Nucb2	0.85003	1.11154809	0.13094146
1420201_at	---	---	0.85003	-1.06989789	-0.0977063
1448819_at	eukaryotic translation initiation factor 2, subunit 2 (beta)	Eif2s2	0.85003	-1.04718885	-0.0675813
1457503_at	---	---	0.85005	1.06896757	0.09527806
1422341_s_at	phospholipase A2, group XV	Pla2g15	0.85005	1.07042144	0.09561846
1422651_at	adiponectin, C1Q and collagen domain containing	Adipoq	0.85006	-1.07435141	-0.1118207
1443723_at	transient receptor potential cation channel, subfamily M, membe	Trpm3	0.85006	1.07117112	0.09846584
1416108_a_at	transmembrane emp24 domain containing 3	Tmed3	0.85008	1.07359796	0.09639914
1451811_at	calcium channel, voltage-dependent, gamma subunit 6	Cacng6	0.85008	-1.07293748	-0.1053447
1418724_at	complement component factor i	Cfi	0.85009	1.07979801	0.10868373
1460627_at	thrombospondin, type I, domain containing 7B	Thsd7b	0.85009	1.06287719	0.08547165
1453381_at	remodeling and spacing factor 1	Rsf1	0.85009	-1.06169171	-0.0864232
1418254_at	APAF1 interacting protein	Apip	0.85011	1.07468757	0.09345666
1456387_at	nucleolar protein 4	Nol4	0.85014	-1.0670967	-0.0944049
1448048_at	cDNA sequence BC016495	BC016495	0.85014	-1.07197414	-0.1011649
1424620_at	NOP16 nucleolar protein homolog (yeast)	Nop16	0.85015	1.05781786	0.07951268
1443891_at	RAD51-like 3 (S. cerevisiae)	Rad51l3	0.85016	1.0876176	0.11200782
1422656_at	RAS-like, family 2, locus 9, pseudogene	Rasl2-9-ps	0.85016	-1.07790824	-0.1096956
1418717_at	mitochondrial ribosomal protein S25	Mrps25	0.85016	1.07919324	0.10363811
1434526_at	epoxide hydrolase 4	Ephx4	0.85016	-1.07356182	-0.1107066
1460350_at	oxysterol binding protein	Osbp	0.85017	1.05346946	0.07484168
1459799_at	FERM, RhoGEF (Arhgef) and pleckstrin domain protein 1 (chondro	Farp1	0.85017	-1.0863307	-0.1433324
1434123_at	fucosyltransferase 11	Fut11	0.85018	1.07247689	0.09900161
1420459_at	ripply3 homolog (zebrafish)	Ripply3	0.85018	1.13458983	0.14782576
1416272_at	MAPK scaffold protein 1	Mapksp1	0.8502	1.05220778	0.07306443
1422282_at	tachykinin receptor 1	Tacr1	0.8502	1.06832486	0.09220897
1451472_at	glutamyl-tRNA synthetase 2 (mitochondrial)(putative)	Ears2	0.8502	1.08470281	0.11354658
1433595_at	solute carrier family 35 (UDP-glucuronic acid/UDP-N-acetylgalact	Slc35d1	0.8502	1.06255253	0.08619524
1459342_at	hypothetical protein C230029M16	C230029M16	0.85021	1.05892275	0.08253803
1418291_at	zinc finger protein 87	Zfp87	0.85021	1.06287774	0.08733145
1418651_at	spermatogenesis associated 6	Spata6	0.85021	-1.07261535	-0.1143692
1431668_at	capping protein (actin filament) muscle Z-line, beta	Capzb	0.85025	1.10509117	0.12793883
1424929_a_at	tripartite motif-containing 26	Trim26	0.85026	1.05773654	0.08076783
1443227_at	basic leucine zipper and W2 domains 2	Bzw2	0.85026	-1.07657388	-0.1162954
1424663_at	cDNA sequence BC017647	BC017647	0.85027	-1.06815597	-0.10872
1447186_at	solute carrier family 6, member 16	Slc6a16	0.85029	1.08245857	0.10708722
1418004_a_at	transmembrane protein 176B	Tmem176b	0.8503	1.0930712	0.11751686
1449318_at	tubulin, gamma 2	Tubg2	0.8503	1.09336677	0.11922452
1445643_at	---	---	0.8503	1.06774814	0.09448211
1418229_s_at	NFU1 iron-sulfur cluster scaffold homolog (S. cerevisiae)	Nfu1	0.85031	-1.05870334	-0.0944352
1431702_at	---	---	0.85031	1.09310642	0.11968534
1439930_at	---	---	0.85034	-1.07399132	-0.1050607
1441004_at	---	---	0.85034	1.09179599	0.11686233
1420806_at	fibroblast growth factor 16	Fgf16	0.85034	1.07488711	0.10320667
1428840_s_at	WD repeat domain 53	Wdr53	0.85034	1.05385607	0.0752295
1438801_at	dynamin 3	Dnm3	0.85036	1.08040966	0.10759698
1429519_at	fucose-1-phosphate guanylyltransferase	Fpgt	0.8504	-1.0678522	-0.0982844
1439190_at	forkhead-associated (FHA) phosphopeptide binding domain 1	Fhad1	0.85047	-1.07488962	-0.1088891
1444834_at	---	---	0.8505	-1.07433931	-0.1057307
1435077_at	additional sex combs like 1 (Drosophila)	Asxl1	0.85065	1.05741472	0.07826629
1417335_at	sulfotransferase family, cytosolic, 2B, member 1	Sult2b1	0.85066	1.08674791	0.11272514
1434213_x_at	NADH dehydrogenase (ubiquinone) Fe-S protein 8	Ndufs8	0.85067	1.0581202	0.07926202
1429540_at	cornifelin	Cnfn	0.85067	-1.08401321	-0.1429189
1445782_at	---	---	0.85068	-1.06235357	-0.0995306
1449396_at	amine oxidase, copper containing 3	Aoc3	0.85072	1.1543273	0.16271552
1427892_at	myosin IG	Myo1g	0.85072	1.06211811	0.08499132
1425893_a_at	fragile histidine triad gene	Fhit	0.85075	-1.07304921	-0.1021743
1446834_at	Cathepsin C	Ctsc	0.85075	-1.06399614	-0.0901407
1446343_at	---	---	0.85076	1.08220123	0.11074463
1452685_at	RIKEN cDNA 0610009O20 gene	0610009O20Rik	0.85078	1.07194816	0.09300568
1445109_at	WD repeat domain 4	Wdr4	0.85079	-1.0718395	-0.1024966
1458443_at	CREB regulated transcription coactivator 1	Crtc1	0.85079	1.10753498	0.12624877
1430889_a_at	thiopurine methyltransferase	Tpmt	0.8508	1.06719078	0.09129063
AFFX-r2-Bs-lys-5_	---	---	0.85081	1.19538103	0.18524492
1439643_at	---	---	0.85082	-1.07874431	-0.1169092
1436432_at	RIKEN cDNA B230343J05 gene	B230343J05Rik	0.85082	-1.05866215	-0.0843164

1435235_at	thioredoxin-like 1	Txnl1	0.85083	-1.05189067	-0.0749543
1427130_x_at	thymus, brain and testes associated	Tbata	0.85083	-1.07720018	-0.1221077
1447070_at	eukaryotic translation initiation factor 4H	Eif4h	0.85083	1.06055364	0.08279025
1419162_s_at	DnaJ (Hsp40) homolog, subfamily C, member 3	Dnajc3	0.85084	1.08911383	0.11429116
1439060_s_at	WD repeat domain, phosphoinositide interacting 1	Wipi1	0.85084	1.08533167	0.10949647
1459264_at	tripartite motif-containing 33	Trim33	0.85084	1.07240526	0.0963602
1427164_at	interleukin 13 receptor, alpha 1	Il13ra1	0.85085	-1.08294577	-0.1581071
1418094_s_at	carbonic anhydrase 4	Car4	0.85085	-1.07919651	-0.1273486
1424606_at	complexin 3	Cplx3	0.85085	1.0746047	0.10347159
1419242_at	RIKEN cDNA 9530002B09 gene	9530002B09Rik	0.85086	-1.08001593	-0.1228138
1445421_at	---	---	0.85086	-1.07009408	-0.1008978
1450448_at	stanniocalcin 1	Stc1	0.85086	1.07033948	0.09713759
1433921_s_at	DPH3 homolog (KTI11, S. cerevisiae)	Dph3	0.85086	1.05690544	0.07724986
1425792_a_at	RAR-related orphan receptor gamma	Rorc	0.85087	1.07511452	0.10301153
1430235_at	RIKEN cDNA 4933424G06 gene	4933424G06Rik	0.85088	1.0776745	0.10568506
1421168_at	ATP-binding cassette, sub-family G (WHITE), member 3	Abcg3	0.85088	1.1036243	0.1259641
1453552_at	RIKEN cDNA 2310014F07 gene	2310014F07Rik	0.85088	-1.07931394	-0.133287
1430441_at	RIKEN cDNA 4933438K21 gene	4933438K21Rik	0.85088	1.12591168	0.14245633
1428800_a_at	pseudouridylate synthase 7 homolog (S. cerevisiae)-like	Pus7l	0.85088	-1.05717046	-0.0818519
1450374_at	parathyroid hormone 2	Pth2	0.85089	1.07355461	0.09700467
1433558_at	disabled homolog 2 (Drosophila) interacting protein	Dab2ip	0.85089	1.0638008	0.08551308
1419574_at	zinc finger protein 292	Zfp292	0.85089	-1.05506279	-0.0824839
1433646_at	mitochondrial ribosomal protein S27	Mrps27	0.85089	1.05426116	0.07425769
1435347_at	staufen (RNA binding protein) homolog 1 (Drosophila)	Stau1	0.8509	1.0753036	0.1011156
1448599_s_at	migration and invasion inhibitory protein	Miip	0.8509	1.08152163	0.10676627
1423410_at	meiosis expressed gene 1	Meig1	0.8509	1.08693243	0.11141624
1456770_at	zinc finger protein 14	Zfp14	0.85091	1.0869151	0.11365836
1452301_at	aldehyde dehydrogenase 3 family, member B1	Aldh3b1	0.85091	1.07678261	0.10540122
1426017_a_at	RIKEN cDNA 0610011L14 gene	0610011L14Rik	0.85091	1.05660984	0.07914537
1453924_a_at	prostaglandin F receptor	Ptgfr	0.85091	-1.07640143	-0.1194693
1439540_at	membrane-associated ring finger (C3HC4) 2	Mar-02	0.85092	-1.07633172	-0.1162307
1437597_at	---	---	0.85092	1.07386045	0.09565944
1428356_at	oxysterol binding protein 2	Osbp2	0.85092	-1.07429402	-0.1061889
1458757_at	---	---	0.85092	-1.06689687	-0.0969005
1445507_at	---	---	0.85092	-1.0754791	-0.111329
1444995_at	Chromodomain protein, Y chromosome-like 2	Cdyl2	0.85092	-1.07179699	-0.1061799
1438791_at	RIKEN cDNA 1700016P04 gene	1700016P04Rik	0.85093	-1.07280236	-0.1077909
1449922_at	RIKEN cDNA 4933425L06 gene	4933425L06Rik	0.85093	1.07269751	0.09827911
1428213_at	non-SMC element 4 homolog A (S. cerevisiae)	Nsmce4a	0.85094	1.04894116	0.0678198
1426090_a_at	fer (fms/fps related) protein kinase, testis specific 2	Fert2	0.85094	-1.06228827	-0.0895494
1431742_at	RIKEN cDNA 1810053B23 gene	1810053B23Rik	0.85094	1.12828662	0.14388937
1448049_at	jumonji domain containing 1C	Jmjd1c	0.85095	1.11351245	0.13473373
1424686_at	HEAT repeat containing 6	Heatr6	0.85095	1.06468508	0.08622169
1421487_a_at	non-catalytic region of tyrosine kinase adaptor protein 1	Nck1	0.85096	1.06006242	0.08046983
1424212_at	RIKEN cDNA 9430023L20 gene	9430023L20Rik	0.85096	1.06974506	0.08831943
1455836_at	poly (A) polymerase alpha	Papola	0.85096	-1.06884197	-0.1161106
1418801_at	zinc finger with KRAB and SCAN domains 1	Zkscan1	0.85097	1.09001503	0.11007974
1441847_at	---	---	0.85098	-1.07908749	-0.1187404
1453086_at	RIKEN cDNA 6330408A02 gene	6330408A02Rik	0.85098	-1.07404788	-0.1067906
1431151_at	lipase, member O1 /// lipase, member O2 /// lipase, member O4	Lipo1 /// Lipo2 /// Lipo4	0.85098	1.07533663	0.10038529
1448012_at	RIKEN cDNA 3110053B16 gene	3110053B16Rik	0.85099	1.12258315	0.13931092
1428549_at	coiled-coil domain containing 3	Ccdc3	0.85099	1.0563195	0.07860776
1449554_at	transducin-like enhancer of split 3, homolog of Drosophila E(spl)	Tle3	0.85101	-1.06569974	-0.1018618
1418840_at	programmed cell death 4	Pdcd4	0.85101	-1.06332592	-0.0982749
1435017_at	CDGSH iron sulfur domain 3	Cisd3	0.85104	-1.048179	-0.0689295
1433108_at	predicted gene 6273	Gm6273	0.85104	1.07451007	0.10163516
1422987_at	netrin 1	Ntn1	0.85109	-1.07572249	-0.1128265
1439470_at	---	---	0.8511	-1.0677651	-0.1063834
1416520_x_at	predicted gene 13611 /// ribosomal protein L36	Gm13611 /// Rpl36	0.85113	1.04514137	0.0593347
1457945_at	---	---	0.85115	1.0956772	0.11892064
1427330_at	cysteinyl-tRNA synthetase	Cars	0.85119	-1.06708031	-0.0985434
1457630_at	---	---	0.85119	1.08821039	0.11377613
1424130_a_at	polymerase I and transcript release factor	Ptrf	0.8512	1.07239039	0.09329487
1448652_at	intraflagellar transport 88 homolog (Chlamydomonas)	Ift88	0.8512	1.06178327	0.08573866
1420894_at	transforming growth factor, beta receptor I	Tgfbr1	0.85122	1.16116643	0.16636932
1433847_at	family with sequence similarity 40, member B	Fam40b	0.85123	1.08066347	0.10440168
1454735_at	outer dense fiber of sperm tails 2	Odf2	0.85123	1.05187336	0.07082125
1415940_at	zinc finger, AN1-type domain 2A	Zfand2a	0.85124	-1.06489559	-0.0999832
1432992_at	RIKEN cDNA 4930403L11 gene	4930403L11Rik	0.85125	1.07348072	0.10205225
1458071_at	---	---	0.85125	-1.0804463	-0.1231959
1440079_at	RIKEN cDNA 3632454L22 gene	3632454L22Rik	0.85125	1.06091245	0.08475602
1426141_at	MAS-related GPR, member A1	Mrgpra1	0.85126	-1.07432111	-0.1105911
1459451_at	---	---	0.85128	1.07586168	0.10037131
1423522_at	nucleoplasmin 3 /// nucleoplasmin 3, pseudogene 1	Npm3 /// Npm3-ps1	0.8513	1.1096233	0.1215361
1432762_at	RIKEN cDNA 9530025H10 gene	9530025H10Rik	0.8513	1.08907197	0.11457529
1453525_at	RIKEN cDNA 9130413E14 gene	9130413E14Rik	0.85132	1.08438702	0.1120618
1421831_at	adenylate kinase 4	Ak4	0.85133	-1.07136563	-0.1011558
1437341_x_at	2',3'-cyclic nucleotide 3' phosphodiesterase	Cnp	0.85134	1.05361093	0.07351469
1447418_at	---	---	0.85136	-1.07362769	-0.1028086
1430577_at	HAUS augmin-like complex, subunit 2	Haus2	0.85149	1.0693157	0.09151719
1441608_at	calcium channel, voltage-dependent, alpha2/delta subunit 1	Cacna2d1	0.85149	-1.06935608	-0.0971967
1438462_x_at	KH domain containing, RNA binding, signal transduction associated	Khdrbs1	0.85149	-1.06040003	-0.0899902
1439700_at	a disintegrin and metallopeptidase domain 1b	Adam1b	0.85151	1.07415422	0.10042591
1428713_s_at	GINS complex subunit 2 (Psf2 homolog)	Gins2	0.85154	1.0548602	0.07562205
1424520_at	RIKEN cDNA 2010305A19 gene	2010305A19Rik	0.85157	1.05512897	0.07555982
1450345_at	ubiquilin 1	Ubqln1	0.85162	1.07220482	0.09990675
1415944_at	syndecan 1	Sdc1	0.85162	1.06582629	0.09158523
1439604_at	a disintegrin-like and metallopeptidase (reprolysin type) with thro	Adamts16	0.85163	-1.07224013	-0.1032683
1443434_s_at	plexin C1	Plxnc1	0.85167	1.07513246	0.10297143
1450399_at	presenilin 1	Psen1	0.85168	1.05717181	0.07805756
1451475_at	plexin D1	Plxnd1	0.85168	-1.07333186	-0.1079975
1459598_at	Hermansky-Pudlak syndrome 1 homolog (human)	Hps1	0.8517	-1.0678242	-0.0957075
1431095_a_at	hect domain and RLD 6	Herc6	0.85172	-1.06704642	-0.0992168
1445780_at	---	---	0.85174	-1.07297201	-0.1114491

1452930_at	dedicator of cyto-kinesis 3	Dock3	0.85174	1.07079601	0.09653008
1424976_at	ras homolog gene family, member V	Rhov	0.85175	-1.07482812	-0.1142307
1443709_at	---	---	0.85175	1.06390193	0.08828119
1427210_at	bromodomain adjacent to zinc finger domain, 2A	Baz2a	0.85176	-1.07351109	-0.1363962
1435228_at	cDNA sequence BC023829	BC023829	0.85177	1.05759498	0.08029128
1442204_at	Pannexin 2	Panx2	0.85177	-1.08348483	-0.141879
1423803_s_at	glioma tumor suppressor candidate region gene 2	Gltscr2	0.85177	1.04476364	0.06276621
1436972_at	G protein-coupled receptor 61	Gpr61	0.85179	-1.06243548	-0.0876217
1429174_at	WD repeat domain 34	Wdr34	0.8518	1.07231828	0.09363978
1456285_at	---	---	0.8518	-1.06228957	-0.0886741
1428479_at	nuclear factor of activated T-cells, cytoplasmic, calcineurin-depen	Nfatc1	0.8518	-1.06854355	-0.0958515
1456189_x_at	latent transforming growth factor beta binding protein 3	Ltbp3	0.8518	1.07145475	0.09867204
1453364_x_at	RIKEN cDNA 9130019O22 gene	9130019O22Rik	0.85181	-1.07145112	-0.0999509
1428828_at	RPA interacting protein	Rpain	0.85181	1.06135038	0.08302262
1437211_x_at	ELOVL family member 5, elongation of long chain fatty acids (yea	Elovl5	0.85181	-1.05144311	-0.076718
1438831_at	Cyclin-dependent kinase 12	Cdk12	0.85182	1.14309143	0.15222692
1418400_at	La ribonucleoprotein domain family, member 6	Larp6	0.85182	-1.07458559	-0.1051869
1440356_at	cytokine induced apoptosis inhibitor 1	Ciapin1	0.85183	-1.07431219	-0.1049964
1451795_at	target of myb1-like 2 (chicken)	Tom1l2	0.85183	-1.07304087	-0.1036486
1439312_at	---	---	0.85184	-1.07267049	-0.1025547
1434455_at	F-box protein 44	Fbxo44	0.85184	1.0738906	0.09764338
1416758_at	Rho GTPase activating protein 27	Arhgap27	0.85184	1.06166195	0.08583498
1449045_at	AFG3(ATPase family gene 3)-like 1 (yeast)	Afg3l1	0.85185	1.04995154	0.069577
1444761_at	---	---	0.85185	1.06703947	0.0903801
1440005_at	one cut domain, family member 2	Onecut2	0.85186	1.10680484	0.12673032
1448856_a_at	methionine sulfoxide reductase A	Msra	0.85186	1.08367453	0.10742874
1460182_at	sorting nexin 4	Snx4	0.85186	1.05598136	0.07540331
1454142_a_at	PWP1 homolog (S. cerevisiae)	Pwp1	0.85187	1.05037458	0.07085094
1457860_at	---	---	0.85187	1.07594751	0.10306278
1459626_at	---	---	0.85187	1.09727597	0.11991039
1449136_at	eosinophil peroxidase	Epx	0.85188	-1.07297905	-0.1060624
1432413_at	trafficking protein particle complex 9	Trappc9	0.85188	1.06878454	0.09580841
1454482_at	RIKEN cDNA 5830474E16 gene	5830474E16Rik	0.85188	1.07807729	0.1046423
1460146_at	---	---	0.85188	-1.05867211	-0.0917529
1424478_at	Bardet-Biedl syndrome 2 (human)	Bbs2	0.85188	-1.051207	-0.0740363
1437310_at	Bardet-Biedl syndrome 1 (human)	Bbs1	0.85189	1.07065165	0.09819861
1437091_at	amiloride-sensitive cation channel 4, pituitary	Accn4	0.85189	1.07745299	0.106239
1426615_s_at	N-myc downstream regulated gene 4	Ndrg4	0.8519	1.06339345	0.08755928
1439695_a_at	kinesin family member 20B	Kif20b	0.8519	-1.04897521	-0.0690146
1433587_at	RGM domain family, member B	Rgmb	0.8519	-1.08226076	-0.1225772
1454289_at	zinc fingers and homeoboxes 3	Zhx3	0.85191	-1.0779693	-0.1178231
1451884_a_at	LSM2 homolog, U6 small nuclear RNA associated (S. cerevisiae)	Lsm2	0.85191	1.0794737	0.09679111
1459624_at	---	---	0.85191	-1.07730481	-0.1263906
1458619_at	---	---	0.85191	-1.078631	-0.1148886
1447678_at	Hydroxysteroid (17-beta) dehydrogenase 14	Hsd17b14	0.85192	-1.05318244	-0.0776833
1419892_at	RIKEN cDNA 1110021J02 gene	1110021J02Rik	0.85192	-1.06353904	-0.0892307
1438374_x_at	amyloid beta (A4) precursor protein	App	0.85192	-1.06936022	-0.1098822
1449254_at	secreted phosphoprotein 1	Spp1	0.85194	-1.03920253	-0.0557917
1452050_at	calcium/calmodulin-dependent protein kinase ID	Camk1d	0.85194	-1.06350969	-0.0916695
1445214_at	---	---	0.85194	-1.0590238	-0.0846763
1460033_at	RIKEN cDNA A330023F24 gene	A330023F24Rik	0.85194	-1.07400776	-0.11428
1445793_at	---	---	0.85195	1.08524721	0.10969595
1434266_at	family with sequence similarity 185, member A	Fam185a	0.85195	1.06918629	0.09086656
1432152_at	RIKEN cDNA 2610109H07 gene	2610109H07Rik	0.85195	1.07266825	0.10105589
1449492_a_at	leukocyte cell-derived chemotaxin 2	Lect2	0.85196	1.07550841	0.10482569
1430345_at	RIKEN cDNA 5530402H23 gene	5530402H23Rik	0.85196	1.05853736	0.08086021
1434691_at	serine/arginine-rich splicing factor 2, interacting protein	Srsf2ip	0.85196	-1.06220911	-0.0960144
1445430_at	---	---	0.85197	1.08360146	0.10916722
1416248_at	NAD kinase	Nadk	0.85197	1.05799823	0.07787076
1458758_at	---	---	0.85197	1.08932101	0.11484643
1457319_at	RIKEN cDNA A130038J17 gene	A130038J17Rik	0.85197	1.08359533	0.10486646
1429814_at	pantothenate kinase 1	Pank1	0.85197	-1.07547684	-0.1067361
1419136_at	aldo-keto reductase family 1, member C18	Akr1c18	0.85197	-1.07161488	-0.1027059
1417097_at	mitochondrial trans-2-enoyl-CoA reductase	Mecr	0.85198	1.05923397	0.08213893
1415755_a_at	ubiquitin-conjugating enzyme E2 variant 1	Ube2v1	0.85199	1.05005716	0.06975358
1434550_at	RIKEN cDNA 3830406C13 gene	3830406C13Rik	0.85199	1.10381538	0.12705371
1424074_at	basic transcription factor 3-like 4	Btf3l4	0.85199	1.07671178	0.10053132
1458895_at	N(alpha)-acetyltransferase 11, NatA catalytic subunit	Naa11	0.852	1.08066099	0.10296735
1422771_at	MAD homolog 6 (Drosophila)	Smad6	0.852	1.10635478	0.12579126
1421397_a_at	leucine-rich and death domain containing	Lrdd	0.852	1.06528853	0.08671401
1416167_at	peroxiredoxin 4	Prdx4	0.852	1.05614745	0.07582666
1420011_s_at	X-box binding protein 1	Xbp1	0.852	1.05769283	0.07723023
1448651_at	nudix (nucleoside diphosphate linked moiety X)-type motif 5	Nudt5	0.85202	-1.04816333	-0.0681147
1442338_at	---	---	0.85203	1.07707147	0.10330338
1425251_at	prostaglandin E receptor 3 (subtype EP3)	Ptger3	0.85204	1.07501152	0.10056437
1449408_at	junction adhesion molecule 2	Jam2	0.85204	-1.05009709	-0.0722176
1416420_a_at	ribosomal protein L9	Rpl9	0.85204	-1.02460603	-0.0351077
1448284_a_at	NADH dehydrogenase (ubiquinone) 1, subcomplex unknown, 1	Ndufc1	0.85204	1.10328271	0.11688091
1424749_at	WD repeat and FYVE domain containing 1	Wdfy1	0.85205	-1.05288102	-0.0756953
1455188_at	Eph receptor B1	Ephb1	0.85205	-1.07324559	-0.1025727
1417783_at	amyotrophic lateral sclerosis 2 (juvenile) homolog (human)	Als2	0.85206	1.05774531	0.08046463
1438217_at	RNA binding protein, fox-1 homolog (C. elegans) 1	Rbfox1	0.85206	-1.06951034	-0.0979951
1426752_at	PHD finger protein 17	Phf17	0.85206	-1.05250259	-0.0783508
1428848_a_at	microtubule-actin crosslinking factor 1	Macf1	0.85208	-1.05932087	-0.092401
1438887_a_at	germ cell-less homolog 1 (Drosophila)	Gmcl1	0.85208	-1.06461337	-0.0981312
1418012_at	SH3-domain GRB2-like B1 (endophilin)	Sh3glb1	0.85208	-1.0614323	-0.087486
1420184_at	---	---	0.8521	1.06431132	0.08846701
1432387_at	plasma glutamate carboxypeptidase	Pgcp	0.8522	1.0813	0.10709648
1438244_at	nuclear factor I/B	Nfib	0.85223	-1.07769506	-0.1329066
1427441_a_at	succinate-Coenzyme A ligase, GDP-forming, beta subunit	Suclg2	0.85229	-1.05117326	-0.0737959
1425290_at	syntaxin 19	Stx19	0.85237	1.08726889	0.10633254
1440226_at	zinc finger protein 760	Zfp760	0.85237	-1.0624748	-0.0890944
1433985_at	abl-interactor 2	Abi2	0.85239	-1.05764579	-0.0846416
1425587_a_at	protein tyrosine phosphatase, receptor type, J	Ptprj	0.8524	1.09180939	0.11626152

1417485_at	integrin binding sialoprotein	Ibsp	0.85241	1.09561803	0.10893726
1439119_a_at	family with sequence similarity 120, member A	Fam120a	0.85241	-1.06668482	-0.1014196
1423662_at	ATPase, H+ transporting, lysosomal accessory protein 2	Atp6ap2	0.85241	-1.05520903	-0.0790345
1454881_s_at	uroplakin 3B	Upk3b	0.85242	-1.07749918	-0.1213246
1458087_at	SH3 and cysteine rich domain 3	Stac3	0.85242	1.11898327	0.1371289
1421795_s_at	killer cell lectin-like receptor subfamily C, member 2 /// killer cell l	Klrc2 /// Klrc3	0.85243	1.07482438	0.10099205
1450962_at	pyruvate dehydrogenase E1 alpha 2	Pdha2	0.85243	-1.07491192	-0.1081415
1425479_at	SET and MYND domain containing 5	Smyd5	0.85244	1.05682533	0.07808804
1432335_at	RIKEN cDNA 4930551O13 gene	4930551O13Rik	0.85244	1.09233113	0.11435724
1446582_at	DNA polymerase N	Poln	0.85244	1.06253421	0.0861226
1435168_at	zinc finger protein 710	Zfp710	0.85245	1.05573764	0.07584396
1422259_a_at	chemokine (C-C motif) receptor 5	Ccr5	0.85245	-1.07611382	-0.1211804
1446914_at	expressed sequence C80425	C80425	0.85245	-1.07141084	-0.1015795
1447624_s_at	storkhead box 2	Stox2	0.85245	-1.0706551	-0.1106521
1430179_at	RIKEN cDNA 4921507G05 gene	4921507G05Rik	0.85246	-1.08380472	-0.1363243
1424406_at	BCL2-like 13 (apoptosis facilitator)	Bcl2l13	0.85247	1.05893823	0.08127464
1417935_at	makorin, ring finger protein, 2	Mkrn2	0.85247	1.05223766	0.07312071
1429425_at	ring finger protein 139	Rnf139	0.85248	1.04719483	0.0664777
1440558_at	ATPase, class II, type 9B	Atp9b	0.85249	-1.07163452	-0.109281
1438479_at	zinc finger protein 213	Zfp213	0.85249	-1.0721117	-0.1014067
1457786_at	---	---	0.85249	-1.07437417	-0.1131262
1446053_at	---	---	0.8525	-1.07496223	-0.1094678
1420274_at	transporter 1, ATP-binding cassette, sub-family B (MDR/TAP)	Tap1	0.8525	1.07139235	0.09449846
1444441_at	Rap guanine nucleotide exchange factor (GEF)-like 1	Rapgefl1	0.85251	1.09789442	0.12223732
1437163_x_at	general transcription factor II H, polypeptide 4	Gtf2h4	0.85251	-1.05718278	-0.0907756
1419621_at	ankyrin repeat domain 2 (stretch responsive muscle)	Ankrd2	0.85251	1.07627007	0.09921479
1420975_at	bromodomain adjacent to zinc finger domain, 1B	Baz1b	0.85252	1.07646293	0.09679083
1442056_at	---	---	0.85252	-1.07409252	-0.1151115
1440702_at	expressed sequence AU022297	AU022297	0.85252	-1.07923767	-0.1297687
1423267_s_at	integrin alpha 5 (fibronectin receptor alpha)	Itga5	0.85252	1.05509059	0.07535986
1445855_at	gamma-glutamyl cyclotransferase	Ggct	0.85252	1.08938102	0.1120567
1427656_at	T-cell receptor beta, joining region	Tcrb-J	0.85252	-1.0772569	-0.1125574
1421579_at	homeobox A9	Hoxa9	0.85253	1.07337405	0.09858288
1428032_at	activin A receptor, type IC	Acvr1c	0.85253	1.07008721	0.09736396
1447765_at	WD repeat domain 48	Wdr48	0.85254	1.08540666	0.11069926
1416511_a_at	CDC42 effector protein (Rho GTPase binding) 4	Cdc42ep4	0.85254	1.06370694	0.08525718
1457943_at	---	---	0.85255	1.07335097	0.10143425
1460502_at	RIKEN cDNA 2010007E15 gene	2010007E15Rik	0.85256	-1.08103136	-0.1266265
1457687_at	B-cell leukemia/lymphoma 2	Bcl2	0.85256	-1.07440282	-0.1142652
1427259_at	tripartite motif-containing 24	Trim24	0.85256	-1.0687041	-0.0998077
1449861_at	NIMA (never in mitosis gene a)-related expressed kinase 4	Nek4	0.85256	1.06339668	0.0866887
1444668_at	amplified spermatogenic transcripts X encoded	Astx	0.85256	1.0787438	0.10715345
1440255_at	histone H4 transcription factor	Hinfp	0.85256	1.06437874	0.0853958
1423548_s_at	ERGIC and golgi 3	Ergic3	0.85257	1.06951034	0.0916385
1417712_at	eukaryotic translation initiation factor 2, subunit 2 (beta)	Eif2s2	0.85257	-1.03166141	-0.0452151
1457137_at	---	---	0.85257	1.07298912	0.10028095
1453029_s_at	ubiquitin protein ligase E3 component n-recognin 3	Ubr3	0.85257	1.05283682	0.07323703
1455702_at	DDB1 and CUL4 associated factor 5	Dcaf5	0.85259	1.08662018	0.11210448
1438820_at	ring finger protein 17	Rnf17	0.85259	-1.05173373	-0.0764657
1417891_at	splA/ryanodine receptor domain and SOCS box containing 3	Spsb3	0.8526	1.06078725	0.08215571
1445639_at	RIKEN cDNA 9130014G24 gene	9130014G24Rik	0.8526	1.08232477	0.10739565
1446613_at	---	---	0.8526	-1.0734819	-0.1104089
1436283_at	RIKEN cDNA D630042F21 gene	D630042F21Rik	0.85261	1.08695541	0.11281837
1453805_at	3-hydroxy-3-methylglutaryl-Coenzyme A lyase	Hmgcl	0.85263	-1.07945371	-0.1173347
1441692_at	---	---	0.85263	-1.07316099	-0.1093667
1418990_at	membrane-spanning 4-domains, subfamily A, member 4D	Ms4a4d	0.85264	1.07015891	0.09746813
1432750_at	zinc finger protein 711	Zfp711	0.85264	1.08661225	0.11216427
1444548_at	RIKEN cDNA 1600025M17 gene	1600025M17Rik	0.85265	-1.07505999	-0.1232697
1455455_at	glycosyltransferase 28 domain containing 2	Glt28d2	0.85265	1.08138782	0.1083633
1436218_at	leucine-rich repeat-containing G protein-coupled receptor 6	Lgr6	0.85266	-1.07286795	-0.1058255
1416571_at	TNF receptor associated factor 4	Traf4	0.85266	1.07041101	0.09800693
1437903_at	lysyl oxidase	Lox	0.85268	1.06415485	0.08818675
1430992_s_at	CDGSH iron sulfur domain 2	Cisd2	0.85297	-1.07027207	-0.1013263
1455913_x_at	transthyretin	Ttr	0.85297	-1.04726009	-0.0679039
1440505_at	phosphatidylinositol-specific phospholipase C, X domain containin	Plcxd1	0.853	-1.06876278	-0.1029654
1424146_at	G protein-coupled receptor 37-like 1	Gpr37l1	0.85301	1.08257652	0.11063683
1453097_a_at	upstream binding transcription factor, RNA polymerase I	Ubtf	0.85301	1.04812426	0.06726416
1446523_at	---	---	0.85302	1.05982076	0.08339208
1423624_at	Fanconi anemia, complementation group L	Fancl	0.85302	1.05951535	0.08143538
1439564_at	RIKEN cDNA 1700090G07 gene	1700090G07Rik	0.85303	-1.07527663	-0.1100825
1454934_at	protein phosphatase 1F (PP2C domain containing)	Ppm1f	0.85303	-1.05257915	-0.075223
1434008_at	sodium channel, type IV, beta	Scn4b	0.85303	-1.07732118	-0.114422
1451846_at	nebulette	Nebl	0.85304	1.09961613	0.11531219
1419886_at	DNA segment, Chr X, ERATO Doi 223, expressed	DXErtd223e	0.85304	-1.07331591	-0.1048827
1449483_at	polymerase (DNA directed), kappa	Polk	0.85305	-1.06402058	-0.0929816
1435885_s_at	intersectin 1 (SH3 domain protein 1A)	Itsn1	0.85305	1.05450854	0.07540602
1426353_at	signal transducer and activator of transcription 6	Stat6	0.85305	-1.05320915	-0.0776452
1444019_at	leucine rich repeat containing 19	Lrrc19	0.85305	-1.068339	-0.0964853
1447952_at	AU RNA binding protein/enoyl-coenzyme A hydratase	Auh	0.85305	1.06719332	0.09251834
1422517_a_at	zinc ribbon domain containing, 1	Znrd1	0.85307	1.05199317	0.07157907
1455606_at	NEDD4 binding protein 1	N4bp1	0.85307	1.05852214	0.0805701
1459037_at	predicted gene 1614	Gm1614	0.85309	-1.06783716	-0.0967352
1458686_at	---	---	0.85309	1.07112688	0.0964586
1458043_at	---	---	0.8531	1.10018661	0.12192236
1444334_at	Developing brain homeobox 2	Dbx2	0.85311	1.07413395	0.10217415
1458821_at	---	---	0.85311	1.10539398	0.12642253
1445525_at	kinesin family member 14	Kif14	0.85311	-1.07461099	-0.1053716
1417318_at	deleted in bladder cancer 1 (human)	Dbc1	0.85314	-1.06745772	-0.0947863
1434330_at	tubulin folding cofactor E-like	Tbcel	0.85315	1.08926859	0.11025846
1440009_at	olfactory receptor 78	Olfr78	0.85315	1.08810209	0.11158866
1448605_at	ras homolog gene family, member C	Rhoc	0.85316	1.07192346	0.09206717
1446204_at	---	---	0.85317	-1.06865492	-0.0989281
1438056_x_at	ATP-binding cassette, sub-family C (CFTR/MRP), member 5	Abcc5	0.85317	-1.05788128	-0.0816924
1420177_at	Immunoglobulin lambda-like polypeptide 1	Igll1	0.85317	1.08442897	0.10978723

1418812_a_at	BarH-like 1 (Drosophila)	Barhl1	0.85318	1.06934192	0.09616031
1425183_a_at	ribosomal protein L4	Rpl4	0.85318	-1.02265561	-0.0324947
1439218_at	---	---	0.85318	1.08475432	0.10977954
1431926_a_at	family with sequence similarity 134, member C	Fam134c	0.85319	1.07088731	0.09336014
1429345_at	tubulin, gamma complex associated protein 4	Tubgcp4	0.85319	1.08888252	0.11127327
1426815_s_at	SEC16 homolog A (S. cerevisiae)	Sec16a	0.8532	1.05292736	0.07335587
1449931_at	cytoplasmic polyadenylation element binding protein 4	Cpeb4	0.85321	1.10541938	0.12312973
1418464_at	matrilin 4	Matn4	0.85322	-1.07078233	-0.1001261
1458342_at	transmembrane protein 90a	Tmem90a	0.85322	1.06327039	0.08812127
1430624_at	predicted gene 15140 /// predicted gene 15144 /// predicted pse	Gm15140 /// Gm15144 //	0.85322	1.07767318	0.1016893
1431116_at	transmembrane protein 200A	Tmem200a	0.85323	-1.07939928	-0.1207919
1441133_at	---	---	0.85323	-1.06569841	-0.0922248
1416822_at	DiGeorge syndrome critical region gene 14	Dgcr14	0.85323	1.09224599	0.11405248
1425078_x_at	RIKEN cDNA C130026I21 gene /// sp110 nuclear body protein-like	C130026I21Rik /// LOC10	0.85324	-1.06434098	-0.0943185
1458880_at	---	---	0.85324	-1.07116921	-0.0997452
1435561_at	Ets2 repressor factor	Erf	0.85325	-1.06428647	-0.1159326
1447111_at	---	---	0.85325	-1.06533494	-0.0934741
1436040_at	small nucleolar RNA host gene 12	Snhg12	0.85325	1.05853692	0.079863
1435449_at	BCL2-like 11 (apoptosis facilitator)	Bcl2l11	0.85325	1.07727568	0.0995508
1417945_at	POU domain, class 5, transcription factor 1	Pou5f1	0.85325	-1.03401986	-0.049202
1426104_at	mitogen-activated protein kinase 14	Mapk14	0.85326	-1.08055705	-0.1158933
1425514_at	phosphatidylinositol 3-kinase, regulatory subunit, polypeptide 1 (p	Pik3r1	0.85326	-1.07655991	-0.1172665
1443247_at	---	---	0.85327	-1.07448441	-0.1110869
1450737_at	polymerase (RNA) III (DNA directed) polypeptide K	Polr3k	0.85328	-1.07418414	-0.1080834
1436877_at	leucine-rich repeats and calponin homology (CH) domain containi	Lrch2	0.85328	-1.07093219	-0.1117672
1438153_x_at	coenzyme Q10 homolog A (yeast)	Coq10a	0.85328	1.08634265	0.11242456
1433953_at	zinc finger protein 277	Zfp277	0.85329	-1.05115743	-0.0735358
1460371_at	heat shock protein 12B	Hspa12b	0.85329	1.07972048	0.10805259
1421951_at	LIM homeobox protein 1	Lhx1	0.85329	-1.07273189	-0.1038445
1438583_at	Endoplasmic reticulum (ER) to nucleus signalling 1	Ern1	0.85329	-1.07544888	-0.1116023
1445409_at	---	---	0.8533	1.07958325	0.10544925
1418478_at	LIM domain only 1	Lmo1	0.8533	1.07430261	0.10149815
1422547_at	RAN binding protein 1	Ranbp1	0.8533	1.03919134	0.05340505
1449455_at	hemopoietic cell kinase	Hck	0.85331	1.05349653	0.07388146
1416226_at	actin related protein 2/3 complex, subunit 1B	Arpc1b	0.85331	1.05644661	0.07751671
1456693_at	hypothetical LOC100504413	LOC100504413	0.85331	1.07269815	0.10069414
1425201_a_at	hydroxypyruvate isomerase homolog (E. coli)	Hyi	0.85331	1.08547689	0.11067589
1433096_at	RIKEN cDNA 4930473M17 gene	4930473M17Rik	0.85331	-1.07978029	-0.1305309
1429954_at	C-type lectin domain family 4, member a3	Clec4a3	0.85331	-1.06212551	-0.0887155
1440775_at	RIKEN cDNA 1700054K19 gene	1700054K19Rik	0.85332	1.08104447	0.10966401
1428865_at	BCL2-like 12 (proline rich)	Bcl2l12	0.85332	1.06386096	0.08912792
1430807_at	kelch-like 22 (Drosophila)	Klhl22	0.85332	-1.07342427	-0.1115266
1441814_s_at	RPA interacting protein	Rpain	0.85333	1.06831628	0.08990116
1416981_at	forkhead box O1	Foxo1	0.85333	1.06597032	0.08719006
1417387_at	mediator of RNA polymerase II transcription, subunit 31 homolog	Med31	0.85333	1.05991035	0.07892092
1449173_at	membrane protein, palmitoylated 2 (MAGUK p55 subfamily mem	Mpp2	0.85333	1.06426421	0.08975672
1417799_at	ATPase, H+ transporting, lysosomal V1 subunit G2	Atp6v1g2	0.85333	-1.07235149	-0.1011819
1449374_at	pipecolic acid oxidase	Pipox	0.85333	1.06358899	0.08524928
1431487_at	predicted gene 10817	Gm10817	0.85333	1.08104967	0.10584335
1427588_a_at	dystrobrevin alpha	Dtna	0.85334	1.08379453	0.10901802
1458118_at	---	---	0.85335	-1.06426422	-0.0942688
1439973_at	Crx opposite strand transcript 1	Crxos1	0.85335	-1.07790892	-0.1240154
1447633_x_at	---	---	0.85335	-1.05893411	-0.0871232
1427139_at	a disintegrin-like and metallopeptidase (reprolysin type) with thro	Adamts10	0.85335	1.07082776	0.09535589
1431341_at	---	---	0.85336	-1.07642078	-0.1175565
1426149_at	serine protease inhibitor 15	Spi15	0.85336	-1.07445346	-0.109032
1460345_at	axin interactor, dorsalization associated	Aida	0.85337	1.0693644	0.09582289
1419371_s_at	golgi SNAP receptor complex member 2	Gosr2	0.85337	1.05449802	0.07490852
1458156_at	RIKEN cDNA E230012J19 gene	E230012J19Rik	0.85337	1.07290153	0.09863997
1433444_at	3-hydroxy-3-methylglutaryl-Coenzyme A synthase 1	Hmgcs1	0.85337	-1.05478968	-0.0865992
1432213_at	RIKEN cDNA 9530047P18 gene	9530047P18Rik	0.85337	-1.07689127	-0.1252182
1435210_s_at	syntaphilin	Snph	0.85337	-1.07433929	-0.1099568
1442670_at	---	---	0.85337	-1.06399237	-0.0943966
1446199_at	regulator of G-protein signaling 20	Rgs20	0.85338	-1.07447604	-0.1120389
1449616_s_at	golgi autoantigen, golgin subfamily a, 3	Golga3	0.85338	1.06201874	0.085957
1455684_at	mitogen-activated protein kinase kinase kinase 9	Map3k9	0.85338	1.07719172	0.10456812
1432533_a_at	solute carrier family 35 (UDP-galactose transporter), member A2	Slc35a2	0.85338	1.06367908	0.08666557
1419307_at	tumor necrosis factor receptor superfamily, member 13c	Tnfrsf13c	0.85338	1.0676557	0.09439694
1443457_at	RIKEN cDNA A230055J12 gene	A230055J12Rik	0.85338	1.05744168	0.08038973
1446772_at	---	---	0.85339	1.07271419	0.09683353
1454172_at	BCL6 co-repressor-like 1	Bcorl1	0.8534	-1.0733506	-0.1069233
1456156_at	leptin receptor	Lepr	0.8534	1.06354197	0.0872818
1416797_at	non-catalytic region of tyrosine kinase adaptor protein 2	Nck2	0.8534	1.08504635	0.1102781
1453070_at	protocadherin 17	Pcdh17	0.8534	1.07405047	0.09441208
1418968_at	RB1-inducible coiled-coil 1	Rb1cc1	0.85341	1.07393417	0.09708562
1438052_at	RIKEN cDNA A130071D04 gene	A130071D04Rik	0.85341	-1.06756416	-0.0966718
1444197_at	Ras and Rab interactor-like	Rinl	0.85342	-1.07025625	-0.0985409
1421860_at	calsyntenin 1	Clstn1	0.85342	1.05696138	0.07801434
1429928_at	cancer/testis antigen 2	Ctag2	0.85342	-1.06945298	-0.1009518
1418443_at	exportin 1, CRM1 homolog (yeast)	Xpo1	0.85342	-1.04149773	-0.0592735
1422962_a_at	proteasome (prosome, macropain) subunit, beta type 8 (large mu	Psmb8	0.85342	1.07477142	0.10279245
1458573_at	---	---	0.85343	-1.07484042	-0.1073678
1431759_at	zinc finger, BED domain containing 4-like	4930529C04Rik	0.85343	-1.077522	-0.1172908
1423113_a_at	ubiquitin-conjugating enzyme E2D 3 (UBC4/5 homolog, yeast)	Ube2d3	0.85343	-1.04331624	-0.064059
1418148_at	abhydrolase domain containing 1	Abhd1	0.85343	1.06199772	0.08626434
1446547_at	RIKEN cDNA 9330175E14 gene	9330175E14Rik	0.85343	1.07922062	0.10364569
1431833_a_at	3-hydroxy-3-methylglutaryl-Coenzyme A synthase 2	Hmgcs2	0.85343	-1.08035598	-0.1283959
1442310_at	Phosphatidylinositol-5-phosphate 4-kinase, type II, alpha	Pip4k2a	0.85343	-1.06641681	-0.0950771
1419553_a_at	RAB geranylgeranyl transferase, b subunit	Rabggtb	0.85344	1.05170126	0.07094336
1453153_at	lines homolog (Drosophila)	Lins	0.85344	1.07694861	0.10084596
1430707_s_at	phosphatidylethanolamine binding protein 4	Pebp4	0.85345	-1.07325898	-0.1120506
1439016_x_at	small proline-rich protein 2A1 /// small proline-rich protein 2A2 //	Sprr2a1 /// Sprr2a2 /// Sp	0.85345	-1.05894338	-0.082719
1419703_at	collagen, type V, alpha 3	Col5a3	0.85345	-1.06004016	-0.0845594
1425063_at	Fc receptor-like 1	Fcrl1	0.85345	1.0641021	0.086292

1456977_at	Sterile alpha motif domain containing 11	Samd11	0.85345	1.08322464	0.10883887
1423791_at	IK cytokine	Ik	0.85345	1.05010622	0.06990157
1451506_at	myocyte enhancer factor 2C	Mef2c	0.85345	-1.07231897	-0.102372
1454842_a_at	UDP-GalNAc:betaGlcNAc beta 1,3-galactosaminyltransferase, pol	B3galnt2	0.85346	-1.07532722	-0.1377186
1444278_at	---	---	0.85346	1.0783817	0.10386009
1438945_x_at	gap junction protein, alpha 1	Gja1	0.85346	-1.05958485	-0.0986681
1431489_at	RIKEN cDNA 4933432K03 gene	4933432K03Rik	0.85347	1.06884869	0.09526556
1417068_a_at	protein tyrosine phosphatase, non-receptor type 1	Ptpn1	0.85347	1.05086257	0.06993471
1429029_at	sphingomyelin synthase 2	Sgms2	0.85347	-1.07591423	-0.1062122
1457708_at	methyl-CpG binding domain protein 4	Mbd4	0.85347	-1.07732	-0.1430414
1453399_at	cyclin T2	Ccnt2	0.85347	1.0919033	0.1108547
1458357_x_at	---	---	0.85347	-1.0664499	-0.0944325
1436558_at	TATA box binding protein (Tbp)-associated factor, RNA polymeras	Taf1d	0.85347	1.06737021	0.09113949
1451243_at	arginyl aminopeptidase (aminopeptidase B)	Rnpep	0.85347	1.06708506	0.09102811
1459558_at	---	---	0.85347	1.05901971	0.08258506
1435427_x_at	family with sequence similarity 193, member A	Fam193a	0.85348	-1.06602134	-0.0936441
1425936_a_at	ankyrin repeat and MYND domain containing 2	Ankmy2	0.85348	1.07574953	0.10072362
1427422_at	predicted gene 6484	Gm6484	0.85348	-1.07306277	-0.1039538
1419607_at	tumor necrosis factor	Tnf	0.85348	-1.07185863	-0.104916
1457031_at	fibronectin type III and SPRY domain containing 2	Fsd2	0.85349	-1.07752827	-0.1237529
1445556_at	---	---	0.85349	-1.07116119	-0.0996882
1434207_at	ring finger protein 169	Rnf169	0.8535	1.06435564	0.08771437
1449683_x_at	tubulin, beta 2a, pseudogene 2	Tubb2a-ps2	0.8535	-1.06369417	-0.0891746
1454717_at	ankyrin repeat domain 27 (VPS9 domain)	Ankrd27	0.8535	1.05686939	0.07690048
1427757_at	---	---	0.8535	1.12662985	0.14236665
1457977_at	MYST/Esa1-associated factor 6	Meaf6	0.85351	1.07738995	0.1017724
1436723_at	centromere protein I	Cenpi	0.85351	1.05425553	0.07446047
1441369_at	RIKEN cDNA C030017B01 gene	C030017B01Rik	0.85351	-1.06454022	-0.0986763
1417018_at	epidermal growth factor-containing fibulin-like extracellular matri	Efemp2	0.85351	-1.06801267	-0.098364
1435718_at	adaptor-related protein complex 3, sigma 2 subunit	Ap3s2	0.85351	1.06639196	0.08958554
1430419_at	RIKEN cDNA 2310031A07 gene	2310031A07Rik	0.85351	-1.07226864	-0.1073098
1449478_at	matrix metallopeptidase 7	Mmp7	0.85352	-1.06891152	-0.0976483
1458042_at	---	---	0.85352	-1.06490106	-0.093695
1424617_at	interferon-induced protein 35	Ifi35	0.85352	1.07057035	0.09575056
1419360_a_at	synovial sarcoma translocation, Chromosome 18	Ss18	0.85352	-1.04972545	-0.0718918
1426388_s_at	receptor-like tyrosine kinase	Ryk	0.85352	-1.05247842	-0.074698
1426231_at	vitrin	Vit	0.85352	1.08476349	0.10831467
1452281_at	son of sevenless homolog 2 (Drosophila)	Sos2	0.85352	-1.06203153	-0.0896018
1432770_at	RIKEN cDNA 0610040A22 gene	0610040A22Rik	0.85352	1.15622023	0.16477434
1418106_at	hairy/enhancer-of-split related with YRPW motif 2	Hey2	0.85352	1.10918943	0.13160528
1441081_a_at	RIKEN cDNA 1110038B12 gene	1110038B12Rik	0.85352	1.06702678	0.08430886
1422561_at	a disintegrin-like and metallopeptidase (reprolysin type) with thro	Adamts5	0.85353	-1.06817481	-0.098891
1434710_at	DEAH (Asp-Glu-Ala-His) box polypeptide 29	Dhx29	0.85353	1.06341435	0.08509837
1453372_at	DnaJ (Hsp40) homolog, subfamily C, member 1 /// hypothetical L	Dnajc1 /// LOC10050412€	0.85353	-1.07285845	-0.1079451
1435954_at	zinc finger protein 710	Zfp710	0.85353	1.071135	0.09495747
1435810_at	RIKEN cDNA 5730455O13 gene	5730455O13Rik	0.85353	1.06492242	0.0889965
1455844_at	predicted gene 6277	Gm6277	0.85354	-1.07379748	-0.1046548
1436812_at	fukutin related protein	Fkrp	0.85355	1.10742555	0.12135156
1458614_at	DCP1 decapping enzyme homolog b (S. cerevisiae)	Dcp1b	0.85355	-1.07370157	-0.1030168
1425470_at	---	---	0.85355	-1.05117134	-0.0723893
1421912_at	solute carrier family 23 (nucleobase transporters), member 1	Slc23a1	0.85355	-1.07672411	-0.1155354
1449244_at	cadherin 2	Cdh2	0.85355	1.07294225	0.10089072
1452770_at	vitamin K epoxide reductase complex, subunit 1	Vkorc1	0.85356	1.06852411	0.08879317
1438399_at	peroxisomal biogenesis factor 5-like	Pex5l	0.85356	1.07703935	0.10456029
1424814_a_at	BCL2-like 14 (apoptosis facilitator)	Bcl2l14	0.85356	1.06509997	0.0882945
1425215_at	free fatty acid receptor 2	Ffar2	0.85357	-1.07274587	-0.1090312
1455866_x_at	thioredoxin-like 1	Txnl1	0.85357	-1.06633066	-0.0929087
1448795_a_at	protein TBRG4-like /// transforming growth factor beta regulated	LOC100046166 /// Tbrg4	0.85357	1.04979165	0.06945184
1417917_at	calponin 1	Cnn1	0.85357	1.07818331	0.10499184
1421082_s_at	barrier to autointegration factor 1	Banf1	0.85357	1.04787675	0.0644339
1431493_at	RIKEN cDNA 9530046B11 gene	9530046B11Rik	0.85358	1.0794645	0.10455613
1429209_at	collagen, type XXIII, alpha 1	Col23a1	0.85358	1.09739711	0.12099642
1429881_at	Rho GTPase activating protein 15	Arhgap15	0.85358	1.0683282	0.09521043
1455574_at	Smg-7 homolog, nonsense mediated mRNA decay factor (C. eleg	Smg7	0.85359	-1.05411912	-0.0839231
1451113_a_at	IK cytokine	Ik	0.85359	-1.05584477	-0.087886
1451483_s_at	RIKEN cDNA 1700054N08 gene	1700054N08Rik	0.8536	1.06891081	0.09193901
1431992_at	RIKEN cDNA 4930568K20 gene	4930568K20Rik	0.85361	1.08791886	0.11321463
1456727_a_at	casein kinase 1, delta	Csnk1d	0.85361	1.04671707	0.06530745
1423182_at	tumor necrosis factor receptor superfamily, member 13b	Tnfrsf13b	0.85361	1.09764647	0.12074048
1455596_a_at	cDNA sequence BC029214	BC029214	0.85361	1.06664601	0.09129275
1443942_at	GA repeat binding protein, beta 2	Gabpb2	0.85362	1.08599876	0.11202981
1455840_at	Rap guanine nucleotide exchange factor (GEF) 5	Rapgef5	0.85362	-1.06670664	-0.1081467
1419662_at	osteoglycin	Ogn	0.85362	-1.07851966	-0.1349802
1425507_at	ADP-ribosylation factor related protein 1	Arfrp1	0.85362	1.05649869	0.07725225
1419819_s_at	SEC63-like (S. cerevisiae)	Sec63	0.85363	1.05586345	0.07790352
1428683_at	family with sequence similarity 173, member B	Fam173b	0.85363	1.094403	0.11643495
1422411_s_at	cDNA sequence BC151093 /// eosinophil-associated, ribonuclease	BC151093 /// Ear1 /// Ear	0.85363	-1.04823643	-0.0695032
1443049_at	Transmembrane protein 19	Tmem19	0.85364	1.09127554	0.11499829
1423699_at	non-SMC condensin II complex, subunit H2	Ncaph2	0.85364	1.05437474	0.07561579
1425238_at	40S ribosomal protein S20 pseudogene	Gm6607	0.85364	-1.06427099	-0.102713
1441948_x_at	zinc finger, AN1-type domain 3	Zfand3	0.85365	-1.055172	-0.0776427
1427705_a_at	nuclear factor of kappa light polypeptide gene enhancer in B-cells	Nfkb1	0.85365	-1.05806093	-0.0848659
1458154_at	---	---	0.85366	1.05633196	0.07819531
1436322_a_at	RIKEN cDNA 2810001A02 gene	2810001A02Rik	0.85366	-1.06207914	-0.0999689
1416088_a_at	ribosomal protein S15	Rps15	0.85367	1.04063319	0.05391788
1430433_at	RIKEN cDNA 4933406J08 gene	4933406J08Rik	0.85367	-1.07208287	-0.1004356
1460306_at	myocilin	Myoc	0.85367	-1.07834637	-0.1146412
1447356_at	cDNA sequence AB099516 /// HIG1 domain family, member 1C	AB099516 /// Higd1c	0.85368	-1.07590852	-0.1207077
1458172_at	malic enzyme 2, NAD(+)-dependent, mitochondrial	Me2	0.85369	-1.07074881	-0.1071731
1434328_at	ribosomal protein L15	Rpl15	0.85369	1.04737236	0.06640264
1444207_at	Alstrom syndrome 1 homolog (human)	Alms1	0.85369	-1.07245138	-0.1049819
1443196_at	---	---	0.85371	1.0740651	0.09870303
1426293_at	zinc finger protein 790	Zfp790	0.85371	1.07488122	0.10149965
1424726_at	transmembrane protein 150A	Tmem150a	0.85372	-1.0651187	-0.0915149

1448500_a_at	Lck interacting transmembrane adaptor 1	Lime1	0.85372	1.0816234	0.10441658
1436582_at	zinc finger, DHHC domain containing 15	Zdhhc15	0.85373	-1.064805	-0.0939409
1422139_at	plasminogen activator, urokinase	Plau	0.85373	1.07742095	0.10207175
1451880_at	predicted gene 9779	Gm9779	0.85375	1.10319023	0.12625519
1421753_a_at	LIM homeobox protein 3	Lhx3	0.85376	-1.07699238	-0.1177835
1429810_at	RPTOR independent companion of MTOR, complex 2	Rictor	0.85376	-1.07407729	-0.1053303
1423880_at	DNA segment, Chr 10, Wayne State University 52, expressed	D10Wsu52e	0.85376	1.07219206	0.09099998
1454981_at	DNA segment, Chr 4, ERATO Doi 22, expressed	D4Ertd22e	0.8538	1.0585511	0.07947567
1445718_at	---	---	0.85388	1.06888457	0.09534364
1429737_a_at	cardiolipin synthase 1	Crls1	0.85388	1.05688618	0.07798441
1452089_at	calcium channel, voltage-dependent, beta 4 subunit	Cacnb4	0.85392	-1.05853115	-0.0835743
1431158_at	---	---	0.85392	-1.07542587	-0.1195975
1455409_at	spire homolog 1 (Drosophila)	Spire1	0.85393	-1.06388217	-0.0982862
1456915_at	poly (A) polymerase beta (testis specific)	Papolb	0.85394	-1.07368805	-0.1050608
1416694_at	glycerol kinase 2	Gk2	0.85395	-1.06588308	-0.0938312
1430475_at	---	---	0.85395	1.06799845	0.09468417
1440104_at	RAN binding protein 2	Ranbp2	0.85397	-1.07737476	-0.1308779
1416542_at	PHD finger protein 1	Phf1	0.85398	1.06535184	0.09038279
1455715_at	hypothetical LOC100038746	LOC100038746	0.85399	1.09109225	0.11332259
1437742_at	RAB21, member RAS oncogene family	Rab21	0.85399	1.10946027	0.12686812
1426101_at	predicted gene 10997	Gm10997	0.854	-1.06883714	-0.0963493
1430369_at	erythrocyte protein band 4.1	Epb4.1	0.854	-1.07164969	-0.104411
1440623_at	G protein-coupled receptor 26	Gpr26	0.85401	-1.0721717	-0.1092508
1428438_s_at	LSM14 homolog A (SCD6, S. cerevisiae)	Lsm14a	0.85401	-1.04625267	-0.0662232
1448328_at	SH3-domain binding protein 2	Sh3bp2	0.85402	1.081795	0.10816426
1453640_at	RIKEN cDNA 2210019G11 gene	2210019G11Rik	0.85402	-1.06041517	-0.1096544
1423693_at	chymotrypsin-like elastase family, member 1	Cela1	0.85403	1.06910968	0.09455271
1451390_s_at	zinc finger, AN1 type domain 2B	Zfand2b	0.85404	1.07094137	0.0954282
1434407_at	SLIT-ROBO Rho GTPase activating protein 2	Srgap2	0.85404	-1.07015913	-0.1075082
1426946_at	importin 5	Ipo5	0.85404	-1.05231027	-0.0782699
1460111_at	myelin transcription factor 1-like	Myt1l	0.85405	-1.06059403	-0.0858481
1442748_at	BTB (POZ) domain containing 3	Btbd3	0.85405	-1.07728115	-0.1159726
1436910_at	RAS protein activator like 2	Rasal2	0.85405	-1.05684614	-0.0799012
1422900_at	meningioma expressed antigen 5 (hyaluronidase)	Mgea5	0.85405	1.06191405	0.08044519
1419613_at	collagen, type VII, alpha 1	Col7a1	0.85407	-1.06828526	-0.0961545
1425417_x_at	killer cell lectin-like receptor, subfamily A, member 8	Klra8	0.85407	1.06621751	0.08754141
1454805_at	Wilms' tumour 1-associating protein	Wtap	0.85407	-1.04846882	-0.0692691
1433218_at	RIKEN cDNA 4930566N20 gene	4930566N20Rik	0.85407	-1.07545513	-0.1080037
1440833_at	cyclin-dependent kinase 13	Cdk13	0.85407	-1.07456413	-0.1124148
1452755_at	---	---	0.85407	-1.06686154	-0.0973652
1445810_at	expressed sequence C85163	C85163	0.85408	-1.0678936	-0.097577
1443343_at	---	---	0.85408	-1.07078582	-0.0995991
1437490_x_at	UDP-N-acetylhexosamine pyrophosphorylase-like /// UDP-N-ace	LOC640502 /// Uap1	0.85408	-1.062244	-0.0872752
1460624_at	RIKEN cDNA 6330564D18 gene	6330564D18Rik	0.85408	1.06476432	0.08933787
1420252_at	---	---	0.85409	1.0758209	0.10342752
1442385_at	---	---	0.85409	1.07703257	0.10457785
1452522_at	stomatin	Stom	0.85409	-1.07908446	-0.1290404
1458746_at	---	---	0.85409	-1.06531672	-0.0921757
1457647_x_at	RIKEN cDNA 1600023N17 gene	1600023N17Rik	0.85409	-1.07662415	-0.1164454
1456738_s_at	brain protein 16	Brp16	0.8541	-1.04627074	-0.0707402
1430890_at	RIKEN cDNA 2210010C17 gene	2210010C17Rik	0.8541	1.08007644	0.10319917
1457721_at	peroxisome proliferator activated receptor alpha	Ppara	0.8541	-1.07376948	-0.1068946
1435627_x_at	MARCKS-like 1	Marcksl1	0.8541	-1.05739671	-0.0820669
1440980_at	potassium voltage-gated channel, subfamily H (eag-related), mer	Kcnh8	0.8541	1.06864717	0.09549044
1457586_at	---	---	0.85411	-1.07295003	-0.1060838
1415983_at	lymphocyte cytosolic protein 1	Lcp1	0.85411	-1.07271807	-0.1158513
1436494_x_at	TRM1 tRNA methyltransferase 1 homolog (S. cerevisiae)	Trmt1	0.85411	-1.0536695	-0.0810574
1449288_at	growth differentiation factor 3	Gdf3	0.85411	1.04773581	0.06617222
1454609_x_at	RIKEN cDNA 6430527G18 gene	6430527G18Rik	0.85411	-1.05184377	-0.0759417
1436514_at	glypican 4	Gpc4	0.85411	-1.05821952	-0.0824499
1450164_at	achaete-scute complex homolog 1 (Drosophila)	Ascl1	0.85412	1.09524357	0.12059525
1440933_at	RIKEN cDNA 4732460I02 gene	4732460I02Rik	0.85412	1.09676339	0.11966926
1452175_at	RIKEN cDNA 1810026J23 gene	1810026J23Rik	0.85412	1.07582166	0.09823393
1440964_s_at	core-binding factor, runt domain, alpha subunit 2, translocated to,	Cbfa2t3	0.85412	-1.06767191	-0.0978819
1439423_x_at	cDNA sequence U46068	U46068	0.85413	1.07342254	0.10221697
1440630_at	---	---	0.85413	-1.07702689	-0.1778258
1429080_at	M-phase phosphoprotein 10 (U3 small nucleolar ribonucleoprotei	Mphosph10	0.85413	-1.04802165	-0.0686867
1442437_at	---	---	0.85413	-1.05802057	-0.0822254
1448601_s_at	homeobox, msh-like 1	Msx1	0.85413	-1.07199587	-0.1006047
1444456_at	RIKEN cDNA 9030425P06 gene /// hypothetical LOC100504699	9030425P06Rik /// LOC1C	0.85413	1.08462161	0.10958556
1435260_at	thymoma viral proto-oncogene 3	Akt3	0.85413	-1.07221786	-0.1057718
1455180_at	GRINL1A complex locus	Gcom1	0.85414	1.08073403	0.10334247
1426047_a_at	protein tyrosine phosphatase, receptor type, R	Ptprr	0.85414	-1.07555052	-0.1251236
1441012_at	---	---	0.85414	-1.07855713	-0.1176866
1432483_at	AT rich interactive domain 2 (ARID, RFX-like)	Arid2	0.85415	1.07198126	0.09667884
1460464_at	RIKEN cDNA 2700089E24 gene	2700089E24Rik	0.85415	-1.06744572	-0.0953041
1443454_at	secretin receptor	Sctr	0.85415	-1.06765064	-0.0995104
1439177_at	---	---	0.85416	1.07842581	0.10415923
1460637_s_at	prefoldin 5	Pfdn5	0.85416	-1.04976821	-0.0732364
1443456_at	zinc finger pseudogene	3110048L19Rik	0.85417	-1.07067559	-0.1021315
1460093_at	---	---	0.85417	1.0691288	0.09359416
1458311_at	ubiquitin specific peptidase 36	Usp36	0.85417	-1.06906877	-0.0963809
1417044_at	leucine carboxyl methyltransferase 1	Lcmt1	0.85418	1.05649134	0.0767876
1435322_at	ankyrin repeat domain 40	Ankrd40	0.85418	1.05647318	0.0778223
1418657_at	INO80 complex subunit B	Ino80b	0.85419	1.0807267	0.1038811
1454562_at	predicted gene 10750	Gm10750	0.8542	1.10105344	0.12248741
1438181_x_at	TM2 domain containing 2	Tm2d2	0.85421	1.0710408	0.09305589
1423817_s_at	unconventional SNARE in the ER 1 homolog (S. cerevisiae)	Use1	0.85421	1.05769695	0.07710855
1427204_at	retinal degeneration 3	Rd3	0.85421	1.06619642	0.09042897
1437908_a_at	endoplasmic reticulum-golgi intermediate compartment (ERGIC) 1	Ergic1	0.85422	-1.05695577	-0.0861727
1431307_at	family with sequence similarity 184, member A	Fam184a	0.85423	1.07564231	0.10034798
1457316_at	microtubule-associated protein 6	Mtap6	0.85423	-1.0633484	-0.0968396
1421018_at	RIKEN cDNA 1110018J18 gene	1110018J18Rik	0.85423	1.06990047	0.09510992
1441482_at	---	---	0.85423	1.12835446	0.13812088

1447470_at	haloacid dehalogenase-like hydrolase domain containing 2	Hdhd2	0.85423	1.11654081	0.13435884
1460413_s_at	zinc finger, DHHC domain containing 1	Zdhhc1	0.85424	1.0822873	0.10770007
1436141_at	calcium binding protein 7 /// hypothetical LOC100503092	Cabp7 /// LOC100503092	0.85424	1.08109529	0.10542413
1426441_at	solute carrier family 11 (proton-coupled divalent metal ion transpo	Slc11a2	0.85426	1.06032888	0.08301374
1453374_at	zinc finger, AN1-type domain 5	Zfand5	0.85427	1.11092397	0.130392
1448469_at	nidogen 1	Nid1	0.85428	-1.06754069	-0.1206349
1419832_s_at	acid phosphatase, prostate	Acpp	0.85428	1.07832328	0.10495222
1451635_at	RIKEN cDNA C730048C13 gene /// RIKEN cDNA D630002G06 gen	C730048C13Rik /// D630C	0.85428	1.06268865	0.08538207
1449470_at	distal-less homeobox 1	Dlx1	0.85429	1.10610466	0.12547901
1452275_at	zinc finger, AN1-type domain 3	Zfand3	0.85429	1.07994809	0.10723219
1448198_a_at	NADH dehydrogenase (ubiquinone) 1 beta subcomplex 8	Ndufb8	0.85429	1.0571341	0.07525384
1444465_at	Ubiquitination factor E4B, UFD2 homolog (S. cerevisiae)	Ube4b	0.8543	-1.07721022	-0.1167223
1415847_at	lactate dehydrogenase C	Ldhc	0.85432	-1.07642968	-0.116166
1430583_at	neuron-glia-CAM-related cell adhesion molecule	Nrcam	0.85433	-1.07317843	-0.1109094
1456208_at	glycerol-3-phosphate acyltransferase 2, mitochondrial	Gpat2	0.85434	1.08715026	0.10947835
1459606_at	expressed sequence C79130	C79130	0.85434	1.0629303	0.0876602
1418293_at	interferon-induced protein with tetratricopeptide repeats 2	Ifit2	0.85437	-1.07250663	-0.1056507
1459138_at	---	---	0.85439	1.07175463	0.0985287
1429027_at	small nucleolar RNA, C/D box 123	Snord123	0.85441	1.07559018	0.10344951
1419874_x_at	zinc finger and BTB domain containing 16	Zbtb16	0.85445	1.13177241	0.14357761
1430150_at	DCP2 decapping enzyme homolog (S. cerevisiae)	Dcp2	0.85446	1.0601648	0.08344256
1416418_at	gamma-aminobutyric acid (GABA) A receptor-associated protein-	Gabarapl1	0.85446	1.08322805	0.10532977
1432581_at	RIKEN cDNA 3110054G05 gene	3110054G05Rik	0.85446	1.07457363	0.10048742
1457429_s_at	predicted gene 106	Gm106	0.85449	-1.06243962	-0.0970325
1435879_at	thymoma viral proto-oncogene 3	Akt3	0.85449	-1.07201228	-0.1061444
1456703_at	DNA segment, Chr 5, ERATO Doi 301, expressed	D5Ertd301e	0.85452	1.07603925	0.10485057
1444979_at	---	---	0.85457	1.08572386	0.11206358
1448074_at	Relaxin 1	Rln1	0.85458	-1.06799917	-0.0989952
1428569_at	RIKEN cDNA B230217C12 gene	B230217C12Rik	0.85458	1.08121979	0.10391966
1430945_at	RIKEN cDNA A930028C08 gene	A930028C08Rik	0.85462	1.07892918	0.10541148
1454503_at	RIKEN cDNA 9030201C23 gene	9030201C23Rik	0.85464	-1.07081349	-0.1061193
1428380_at	RIKEN cDNA 0610007C21 gene	0610007C21Rik	0.85464	1.06994164	0.08907717
1456601_x_at	FXYD domain-containing ion transport regulator 2	Fxyd2	0.85469	1.08014209	0.10689237
1455751_at	cullin associated and neddylation disassociated 1	Cand1	0.85485	1.06856345	0.09398439
1449880_s_at	bone gamma carboxyglutamate protein /// bone gamma-carbox	Bglap /// Bglap-rs1 /// Bg	0.85487	1.0707769	0.09543375
1440203_at	polycomb group ring finger 3	Pcgf3	0.85488	-1.0745835	-0.1061719
1418772_at	cDNA sequence BC016423	BC016423	0.8549	-1.06141998	-0.0926413
1417860_a_at	spondin 2, extracellular matrix protein	Spon2	0.85491	1.0927594	0.11639068
1438441_at	RIKEN cDNA A330033J07 gene	A330033J07Rik	0.85491	-1.07326153	-0.106651
1450479_x_at	protein tyrosine phosphatase, non-receptor type 12	Ptpn12	0.85491	-1.05794975	-0.0873701
1428907_at	RNA binding motif protein 25	Rbm25	0.85494	-1.04774532	-0.0674442
1424123_at	major facilitator superfamily domain containing 7C	Mfsd7c	0.85499	1.09015181	0.11066903
1433662_s_at	tissue inhibitor of metalloproteinase 2	Timp2	0.85499	-1.069189	-0.0995694
1430049_at	hyaluronoglucosaminidase 6	Hyal6	0.85502	1.10850987	0.12851311
1449748_at	expressed sequence AA517023	AA517023	0.85503	-1.06338004	-0.0905266
1459424_at	DNA segment, Chr 3, ERATO Doi 108, expressed	D3Ertd108e	0.85505	1.07961343	0.10346025
1426828_at	RIKEN cDNA 1300018I17 gene	1300018I17Rik	0.85506	1.07146557	0.09576879
1424258_at	polymerase (RNA) II (DNA directed) polypeptide D	Polr2d	0.85506	1.05013142	0.07001777
1416947_s_at	acetyl-Coenzyme A acyltransferase 1A /// acetyl-Coenzyme A ac	Acaa1a /// Acaa1b	0.85507	1.05333863	0.07271112
1427373_at	adhesion molecule with Ig like domain 1	Amigo1	0.85507	1.13986418	0.14904866
1423097_s_at	calpain 7	Capn7	0.85507	-1.05188517	-0.0736365
1443595_at	---	---	0.85508	1.06757442	0.09057141
1448864_at	SNF related kinase	Snrk	0.85509	-1.06102279	-0.0898909
1447883_x_at	microtubule-associated protein 1 light chain 3 alpha	Map1lc3a	0.85509	-1.05222229	-0.0792956
1428733_at	guanine nucleotide binding protein (G protein), gamma transducin	Gngt2	0.85509	1.09167157	0.11327123
1415980_at	ATP synthase, H+ transporting, mitochondrial F0 complex, subunit	Atp5g2	0.8551	1.03752736	0.0529224
1426944_at	F-box and WD-40 domain protein 8	Fbxw8	0.85511	-1.04827003	-0.0685464
1417812_a_at	laminin, beta 3	Lamb3	0.85511	1.07371796	0.1019568
1453729_a_at	60S ribosomal protein L37-like /// ribosomal protein L37	LOC100502825 /// Rpl37	0.85511	1.04079995	0.05267829
1419597_at	ectodysplasin-A	Eda	0.85511	1.0905614	0.11313509
1427555_at	myeloid/lymphoid or mixed-lineage leukemia 2	Mll2	0.85513	-1.07516971	-0.1123336
1415886_at	SH2 domain containing 3C	Sh2d3c	0.85514	1.09636197	0.11541978
1439480_at	predicted gene 10857	Gm10857	0.85515	1.09223517	0.11387967
1427790_at	a disintegrin and metallopeptidase domain 1a	Adam1a	0.85516	-1.07368603	-0.1108687
1444210_at	RIKEN cDNA 1600021P15 gene	1600021P15Rik	0.85516	-1.0794739	-0.1173277
1458503_at	B-cell CLL/lymphoma 7A	Bcl7a	0.85516	-1.05580824	-0.0803263
1449718_s_at	RIKEN cDNA 4930453N24 gene	4930453N24Rik	0.85517	-1.06011704	-0.08663
1453213_at	spermatogenesis associated 9	Spata9	0.85517	1.05764634	0.08048652
1436686_at	zinc finger protein 706	Zfp706	0.85517	-1.05805366	-0.0821152
1453826_at	par-3 partitioning defective 3 homolog B (C. elegans)	Pard3b	0.8552	-1.06902924	-0.1005326
1436424_at	RIKEN cDNA 1600020E01 gene	1600020E01Rik	0.85521	1.07205955	0.09240343
1443759_at	---	---	0.85522	1.06958045	0.0913655
1433843_at	HCLS1 binding protein 3	Hs1bp3	0.85522	1.06832721	0.09205281
1442722_at	---	---	0.85525	-1.07144089	-0.11113
1434506_at	AT rich interactive domain 2 (ARID, RFX-like)	Arid2	0.8553	1.0535309	0.07454854
1434351_at	nucleoporin 214	Nup214	0.85533	-1.05347936	-0.0764367
1456935_at	expressed sequence AI606473	AI606473	0.85536	1.09808912	0.11838856
1440266_at	RIKEN cDNA 5830428M24 gene	5830428M24Rik	0.85537	-1.07186968	-0.1009472
1437000_at	diacylglycerol kinase, theta	Dgkq	0.85538	1.07465053	0.09816365
1426071_at	TGF-beta1-induced anti-apoptotic factor 2	Tiaf2	0.85539	1.07823165	0.10380223
1446412_at	---	---	0.85539	-1.06039254	-0.0862554
1416553_at	stimulated by retinoic acid 13	Stra13	0.85539	1.05668978	0.07728851
1453068_at	hypothetical LOC100503505 /// PR domain containing 2, with ZNI	LOC100503505 /// Prdm2	0.85539	-1.06172438	-0.09612
1431241_at	coiled-coil-helix-coiled-coil-helix domain containing 3	Chchd3	0.85542	1.05777199	0.08059663
1445863_at	Von Willebrand factor A domain containing 3A	Vwa3a	0.85542	-1.07442383	-0.1123775
1430660_at	RIKEN cDNA 1500004A13 gene	1500004A13Rik	0.85542	-1.07011603	-0.1022022
1452647_a_at	DPH2 homolog (S. cerevisiae)	Dph2	0.85543	1.06921033	0.09250921
1457380_at	---	---	0.85544	-1.06884077	-0.0969903
1423056_at	neuron specific gene family member 1	Nsg1	0.85546	-1.07456631	-0.1162945
1437626_at	zinc finger protein 36, C3H type-like 2	Zfp36l2	0.85547	1.05462599	0.07512848
1415790_at	ubiquitin-like domain containing CTD phosphatase 1	Ublcp1	0.85551	1.05844899	0.08029536
1429301_at	---	---	0.85552	-1.06229055	-0.0966707
1437075_at	FERM domain containing 3	Frmd3	0.85571	1.0653519	0.08516759
1445540_at	---	---	0.85571	-1.07311146	-0.1075089

1449201_at	steroidogenic acute regulatory protein	Star	0.85574	-1.07371114	-0.1079166
1428057_a_at	AHNAK nucleoprotein (desmoyokin)	Ahnak	0.85575	-1.08336009	-0.1497742
1448887_x_at	fractured callus expressed transcript 1	Fxc1	0.85588	1.08294573	0.10000877
1453686_x_at	nephronophthisis 1 (juvenile) homolog (human)	Nphp1	0.85593	-1.06122715	-0.089035
1420476_a_at	nucleosome assembly protein 1-like 1	Nap1l1	0.85595	-1.04642731	-0.0679703
1442996_x_at	---	---	0.85597	-1.07344829	-0.104772
1451047_at	integral membrane protein 2A	Itm2a	0.85597	-1.05906562	-0.0904218
1434088_at	zinc finger with KRAB and SCAN domains 17	Zkscan17	0.85597	1.05614617	0.07615527
1425669_at	MOB1, Mps One Binder kinase activator-like 2B (yeast)	Mobkl2b	0.85598	1.08418805	0.10719417
1429529_at	tRNA splicing endonuclease 54 homolog (S. cerevisiae)	Tsen54	0.85599	1.06459462	0.08635335
1442416_at	hypothetical protein D930001B02	D930001B02	0.85599	-1.07616954	-0.113988
1436176_at	RIKEN cDNA 1500004A13 gene	1500004A13Rik	0.856	1.07533488	0.09940585
1416138_at	annexin A7	Anxa7	0.85601	-1.04849737	-0.0698977
1437207_at	ring finger protein 170	Rnf170	0.85602	-1.07155759	-0.1017764
1434428_at	RIKEN cDNA D330028D13 gene	D330028D13Rik	0.85602	1.07151542	0.09901816
1456441_at	coiled-coil domain containing 87	Ccdc87	0.85602	-1.07424857	-0.113231
1425917_at	histocompatibility 28	H28	0.85604	1.06396489	0.08882433
1435933_at	sodium channel, voltage-gated, type II, alpha 1	Scn2a1	0.85604	1.05622598	0.07713171
1429573_at	DMRT-like family C1a	Dmrtc1a	0.85605	-1.07688674	-0.1133427
1420565_at	homeobox A1	Hoxa1	0.85605	-1.07158586	-0.1080126
1421514_a_at	sex comb on midleg-like 2 (Drosophila)	Scml2	0.85606	-1.07394789	-0.1032158
1438912_at	hepatoma-derived growth factor, related protein 2	Hdgfrp2	0.85606	-1.06858454	-0.0999364
1432032_a_at	artemin	Artn	0.85606	1.08158101	0.1078634
1459125_at	RIKEN cDNA D030059C06 gene	D030059C06Rik	0.85606	1.07041018	0.09380966
1447095_at	expressed sequence C86942	C86942	0.85607	1.08036294	0.10671483
1446372_at	---	---	0.85607	-1.07157913	-0.1025126
1424682_at	GPN-loop GTPase 3	Gpn3	0.85608	1.0491819	0.06925751
1439229_at	pleckstrin homology domain containing, family A member 7	Plekha7	0.85608	-1.06654044	-0.0960861
1454425_at	RIKEN cDNA 5830495A06 gene	5830495A06Rik	0.85609	1.08328134	0.11150185
1460140_at	---	---	0.85609	1.07086666	0.09018253
1424519_at	mitochondrial GTPase 1 homolog (S. cerevisiae)	Mtg1	0.85611	1.05386848	0.0754066
1427879_at	RIKEN cDNA 1810031K17 gene	1810031K17Rik	0.85612	1.06183829	0.08435944
1443964_at	transmembrane inner ear	Tmie	0.85614	1.08128076	0.10694964
1450577_at	somatostatin receptor 3	Sstr3	0.85616	-1.07384448	-0.1078916
1446081_at	---	---	0.85631	1.0795277	0.10211649
1455133_s_at	expressed sequence AI848100	AI848100	0.85662	-1.05923196	-0.0880488
1434538_x_at	eukaryotic translation initiation factor 2B, subunit 2 beta	Eif2b2	0.85672	1.05284734	0.07146769
1428365_a_at	lon peptidase 1, mitochondrial	Lonp1	0.85677	1.05025165	0.06936111
1425949_at	solute carrier family 25, member 30	Slc25a30	0.8568	1.06957426	0.09569222
1456999_at	WD repeat domain, phosphoinositide interacting 2	Wipi2	0.85682	-1.07287748	-0.1228606
1420333_at	thioredoxin domain containing 8	Txndc8	0.85682	-1.07466197	-0.1104387
1446225_at	Ssu72 RNA polymerase II CTD phosphatase homolog (yeast)	Ssu72	0.85683	1.06581317	0.09067788
1445907_at	---	---	0.85684	-1.07241071	-0.1042582
1440941_at	hypothetical LOC552912	LOC552912	0.85684	-1.06754858	-0.1006317
1447999_x_at	glyceraldehyde-3-phosphate dehydrogenase /// glyceraldehyde-	Gapdh /// Gm2451 /// Gn	0.85684	-1.05035064	-0.0749576
1433080_at	RIKEN cDNA 4933436P19 gene	4933436P19Rik	0.85684	-1.07094331	-0.1029332
1458190_at	Rho GTPase activating protein 4	Arhgap4	0.85687	-1.07089009	-0.1004994
1453148_at	sema domain, immunoglobulin domain (Ig), short basic domain, s	Sema3d	0.85689	1.08293272	0.1081396
1428512_at	basic helix-loop-helix domain containing, class B9	Bhlhb9	0.8569	1.05021205	0.06991494
1431383_at	RIKEN cDNA 1110028F11 gene	1110028F11Rik	0.85692	1.07863316	0.10406968
1449705_x_at	minichromosome maintenance deficient 3 (S. cerevisiae)	Mcm3	0.85692	1.04689438	0.06538991
1435903_at	CD300A antigen	Cd300a	0.85692	-1.0725766	-0.1041567
1416164_at	fibulin 5	Fbln5	0.85708	1.07715748	0.1059388
1421935_at	ribosomal protein S20	Rps20	0.85708	1.08723152	0.11091055
1436669_at	RIKEN cDNA 1700019G17 gene	1700019G17Rik	0.85709	1.07663133	0.10347999
1449247_at	actin-related protein T2	Actrt2	0.8571	-1.07645745	-0.1256042
1448604_at	uridine-cytidine kinase 2	Uck2	0.8571	1.04756916	0.06575483
1457213_a_at	diacylglycerol kinase, eta	Dgkh	0.85711	-1.05635765	-0.0822723
1421500_at	steroid sulfatase	Sts	0.85711	1.07249853	0.09543467
1453833_a_at	ribonuclease H1	Rnaseh1	0.85715	1.0585536	0.07955466
1423583_at	feminization 1 homolog a (C. elegans)	Fem1a	0.85719	1.06941172	0.09392952
1433299_at	RIKEN cDNA 5830461L22 gene	5830461L22Rik	0.85721	-1.06338643	-0.0943106
1424039_at	transmembrane protein 66	Tmem66	0.85722	1.05146158	0.07186467
1427566_at	WAP, FS, Ig, KU, and NTR-containing protein 1	Wfikkn1	0.85725	1.06653035	0.09094599
1422095_a_at	cytidine monophosphate (UMP-CMP) kinase 2, mitochondrial	Cmpk2	0.85727	1.09024437	0.11452946
1440506_at	---	---	0.85728	-1.05778947	-0.0820978
1449036_at	ring finger protein 128	Rnf128	0.85729	-1.0709969	-0.1194823
1453955_a_at	family with sequence similarity 122, member C	Fam122c	0.8573	-1.07787382	-0.1252884
1451340_at	AT rich interactive domain 5A (MRF1-like)	Arid5a	0.85731	1.08210946	0.10332023
1436992_x_at	voltage-dependent anion channel 1	Vdac1	0.85732	-1.03933695	-0.0561066
1416854_at	solute carrier family 34 (sodium phosphate), member 2	Slc34a2	0.85732	1.07963903	0.10380347
1442319_at	ubiquitin specific peptidase 4 (proto-oncogene)	Usp4	0.85734	1.07020805	0.09741882
1438825_at	calmodulin 3	Calm3	0.85735	-1.06693121	-0.1077354
1459653_at	---	---	0.85737	-1.05450086	-0.0798923
1455189_at	tripartite motif-containing 33	Trim33	0.85739	-1.06064351	-0.0927463
1418186_at	glutathione S-transferase, theta 1	Gstt1	0.85739	1.07278015	0.0941669
1445683_at	---	---	0.85739	-1.07901551	-0.1554285
1415821_at	neuroplastin	Nptn	0.85739	-1.04845189	-0.0693022
1424366_at	dolichol kinase	Dolk	0.85739	1.07406561	0.09481875
1431227_at	---	---	0.8574	1.07376273	0.09494805
1426376_at	receptor accessory protein 5	Reep5	0.8574	1.06623344	0.09186622
1449471_at	potassium large conductance calcium-activated channel, subfami	Kcnmb4	0.8574	-1.07265422	-0.1035498
1419606_a_at	troponin T1, skeletal, slow	Tnnt1	0.8574	1.08596627	0.10523585
1416785_at	Kv channel-interacting protein 1	Kcnip1	0.8574	-1.07514119	-0.1054184
1419618_at	butyrobetaine (gamma), 2-oxoglutarate dioxygenase 1 (gamma-	Bbox1	0.85741	-1.07834956	-0.1408291
1433689_s_at	ribosomal protein S9	Rps9	0.85741	1.03377982	0.04609456
1457219_at	rho/rac guanine nucleotide exchange factor (GEF) 18	Arhgef18	0.85741	1.07946804	0.10565223
1420054_s_at	solute carrier family 35, member C2	Slc35c2	0.85742	1.07464008	0.0965989
1419063_at	UDP galactosyltransferase 8A	Ugt8a	0.85742	1.06927184	0.09617811
1423192_at	paraspeckle protein 1	Pspc1	0.85743	1.05142168	0.07057596
1432738_at	RIKEN cDNA 2810026P18 gene	2810026P18Rik	0.85743	-1.07406623	-0.1054072
1432669_at	RIKEN cDNA 9030420N05 gene	9030420N05Rik	0.85743	-1.0659568	-0.0923092
1454222_a_at	coiled-coil domain containing 13 /// coiled-coil domain-containing	Ccdc13 /// LOC10050286	0.85744	-1.06201717	-0.0873485
1417517_at	pleiomorphic adenoma gene-like 2	Plagl2	0.85744	1.06342776	0.08786973

1454241_at	RWD domain containing 1	Rwdd1	0.85744	1.07073567	0.09582662
1459222_x_at	---	---	0.85744	-1.0683013	-0.0957836
1422620_s_at	phosphatidic acid phosphatase type 2A	Ppap2a	0.85744	-1.05074041	-0.0714672
1432355_at	RIKEN cDNA 2210039B01 gene	2210039B01Rik	0.85744	-1.0713386	-0.1075591
1444510_at	---	---	0.85744	1.07138447	0.09906174
1422273_at	matrix metallopeptidase 1b (interstitial collagenase)	Mmp1b	0.85745	-1.07310179	-0.1129567
1444070_at	EST AA914427	AA914427	0.85745	1.08378002	0.10618964
1424991_s_at	thymidylate synthase /// thymidylate synthase, pseudogene	Tyms /// Tyms-ps	0.85745	1.04571774	0.06412936
1430000_at	RIKEN cDNA B230117O15 gene	B230117O15Rik	0.85745	1.09545717	0.11257292
1429638_at	Werner helicase interacting protein 1	Wrnip1	0.85746	-1.0700674	-0.1128116
1433036_at	RIKEN cDNA 4930564B12 gene	4930564B12Rik	0.85746	-1.08122962	-0.1335773
1430743_at	hypothetical LOC100502619	LOC100502619	0.85746	-1.07532386	-0.1155231
1421632_at	potassium intermediate/small conductance calcium-activated cha	Kcnn3	0.85746	1.07168401	0.09726168
1449574_a_at	cell division cycle 42 homolog (S. cerevisiae)	Cdc42	0.85747	-1.04800415	-0.0705241
1420924_at	tissue inhibitor of metalloproteinase 2	Timp2	0.85747	-1.07217048	-0.1039565
1460743_at	tigger transposable element derived 5	Tigd5	0.85747	1.0788777	0.10369472
1448267_at	syntaxin 5A	Stx5a	0.85747	1.06737032	0.09195857
1444819_at	expressed sequence C86090	C86090	0.85747	1.0727577	0.09915165
1430169_at	RIKEN cDNA 1700007K09 gene	1700007K09Rik	0.85747	1.09205877	0.11809331
1416674_at	protein tyrosine phosphatase, receptor type, U	Ptpru	0.85747	1.07615902	0.10263707
1441170_a_at	disabled homolog 2 (Drosophila) interacting protein	Dab2ip	0.85748	1.06737818	0.09387737
1454962_at	spire homolog 1 (Drosophila)	Spire1	0.85748	1.05866958	0.08154784
1443270_at	---	---	0.85748	-1.07506618	-0.1225343
1459028_at	---	---	0.85748	-1.06613197	-0.0930373
1425798_a_at	RecQ protein-like	Recql	0.85748	1.06041599	0.0835956
1438883_at	fibroblast growth factor 5	Fgf5	0.85748	1.08412513	0.10648427
1429117_at	TNFRSF1A-associated via death domain	Tradd	0.85749	1.06041214	0.08380575
1431130_at	RIKEN cDNA 2010110P09 gene	2010110P09Rik	0.85749	-1.07558514	-0.1132627
1424715_at	retinol saturase (all trans retinol 13,14 reductase)	Retsat	0.8575	1.08625722	0.10811345
1440827_x_at	SRY-box containing gene 5	Sox5	0.8575	-1.0671046	-0.1121441
1435446_a_at	choline phosphotransferase 1	Chpt1	0.8575	-1.05890302	-0.0831646
1428499_at	RIKEN cDNA 2810454L23 gene	2810454L23Rik	0.8575	1.07391301	0.09941392
1435454_a_at	cDNA sequence BC006779	BC006779	0.8575	1.08490327	0.10927635
1433071_at	RIKEN cDNA 6430605C03 gene	6430605C03Rik	0.85751	-1.04838959	-0.0734503
1447126_at	---	---	0.85751	-1.07053611	-0.0998703
1432112_at	RIKEN cDNA 4930589L23 gene	4930589L23Rik	0.85751	1.10774832	0.12593923
1417611_at	transmembrane protein 37	Tmem37	0.85752	1.06288006	0.08687405
1431203_at	serologically defined colon cancer antigen 8	Sdccag8	0.85752	-1.0718258	-0.1200764
1438383_x_at	protein phosphatase 2 (formerly 2A), regulatory subunit A (PR 65)	Ppp2r1a	0.85753	-1.04202472	-0.0613707
1455482_at	adaptor protein complex AP-2, alpha 2 subunit	Ap2a2	0.85754	-1.04942869	-0.072635
1450078_at	Nik related kinase	Nrk	0.85755	1.07609845	0.09762616
1417633_at	superoxide dismutase 3, extracellular	Sod3	0.85755	-1.0728446	-0.1069316
1424191_a_at	transmembrane protein 41a	Tmem41a	0.85756	1.05881006	0.08126617
1460283_at	Mediterranean fever	Mefv	0.85756	-1.06846927	-0.0968404
1459167_at	---	---	0.85757	-1.06756598	-0.0944806
1430217_at	leucine-rich repeats and guanylate kinase domain containing	Lrguk	0.85758	1.08565042	0.1090078
1430462_at	RIKEN cDNA 2310002L09 gene	2310002L09Rik	0.85758	-1.06163173	-0.0942265
1418127_a_at	apoptosis-inducing factor, mitochondrion-associated 1	Aifm1	0.85758	-1.05427152	-0.0809284
1432541_at	RIKEN cDNA 4930408O17 gene	4930408O17Rik	0.85759	-1.06421324	-0.0901366
1444297_at	serine (or cysteine) peptidase inhibitor, clade A, member 4, pseud	Serpina4-ps1	0.85759	1.09240304	0.1166599
1426036_a_at	phosphodiesterase 1C	Pde1c	0.8576	1.07166837	0.09736343
1448837_at	villin 1	Vil1	0.8576	1.09926829	0.12078206
1434118_at	mitochondrial ubiquitin ligase activator of NFKB 1	Mul1	0.8576	1.09320511	0.11298733
1428449_at	general transcription factor IIIC, polypeptide 2, beta	Gtf3c2	0.8576	-1.05884692	-0.0955254
1448208_at	MAD homolog 1 (Drosophila)	Smad1	0.85761	1.05811611	0.07895603
1445328_at	collagen, type IV, alpha 4	Col4a4	0.85761	1.07467954	0.10240341
1428408_a_at	extended synaptotagmin-like protein 2	Esyt2	0.85761	1.05630907	0.07761761
1459570_at	---	---	0.85761	1.05730468	0.07693028
1420779_at	four and a half LIM domains 3	Fhl3	0.85762	1.08044745	0.10586584
1431439_at	RIKEN cDNA 5830487J09 gene	5830487J09Rik	0.85762	-1.06308662	-0.0892972
1426133_a_at	MIT, microtubule interacting and transport, domain containing 1	Mitd1	0.85762	1.05845934	0.07967998
1435616_at	cytochrome P450, family 20, subfamily A, polypeptide 1	Cyp20a1	0.85762	-1.0578364	-0.0934726
1420946_at	alpha thalassemia/mental retardation syndrome X-linked homolo	Atrx	0.85762	-1.07549664	-0.1680338
1430520_at	copine VIII	Cpne8	0.85763	1.09640687	0.11947496
1431920_a_at	interleukin 34	Il34	0.85763	-1.07287342	-0.1082291
1430357_at	H3 histone, family 3B	H3f3b	0.85763	1.09439966	0.11557238
1418797_at	membrane-spanning 4-domains, subfamily A, member 8A	Ms4a8a	0.85763	-1.07263791	-0.104523
1444955_at	---	---	0.85763	-1.07395873	-0.1139211
1447848_at	epidermal growth factor receptor pathway substrate 15-like 1	Eps15l1	0.85763	-1.06679358	-0.1031919
1424555_at	RIKEN cDNA 9430015G10 gene	9430015G10Rik	0.85764	1.08008066	0.10132432
1422511_a_at	opioid growth factor receptor	Ogfr	0.85764	1.05710894	0.07667239
1442728_at	---	---	0.85764	-1.07323865	-0.1197133
1452307_at	CDK5 and Abl enzyme substrate 2	Cables2	0.85764	1.06639584	0.08871664
1441742_at	potassium voltage-gated channel, subfamily H (eag-related), mer	Kcnh5	0.85764	1.05522951	0.07620541
1452943_at	transmembrane protein 65	Tmem65	0.85764	-1.06676828	-0.0988649
1441691_at	---	---	0.85764	-1.08024255	-0.1426616
1427640_a_at	runt-related transcription factor 1; translocated to, 1 (cyclin D-rela	Runx1t1	0.85764	-1.07395034	-0.110447
1441511_at	potassium channel, subfamily T, member 1	Kcnt1	0.85764	1.08180951	0.10802338
1441865_at	---	---	0.85765	1.05912028	0.07685056
1426857_a_at	hydroxysteroid dehydrogenase like 2	Hsdl2	0.85765	-1.06036658	-0.0847174
1422309_a_at	RFad1, flavin adenine dinucleotide synthetase, homolog (yeast) /	Flad1 /// Lenep	0.85766	1.0728201	0.09907443
1418335_a_at	DPH1 homolog (S. cerevisiae) /// candidate tumor suppressor in c	Dph1 /// Ovca2	0.85766	-1.05832343	-0.0909308
1446898_at	---	---	0.85767	-1.07329825	-0.1154869
1451743_at	DNA segment, Chr 19, Wayne State University 162, expressed	D19Wsu162e	0.85768	1.08980645	0.10915915
1435065_x_at	vav 2 oncogene	Vav2	0.85768	1.08203463	0.10697826
1442409_at	DNA segment, Chr 9, Wayne State University 90, expressed	D9Wsu90e	0.85768	1.0941918	0.11743096
1418630_at	KH domain containing, RNA binding, signal transduction associate	Khdrbs1	0.85769	-1.07407635	-0.1073387
1418374_at	FXYD domain-containing ion transport regulator 3	Fxyd3	0.8577	1.06921718	0.0942238
1427226_at	epsin 2	Epn2	0.8577	1.05259977	0.07222135
1457961_at	---	---	0.8577	1.06980753	0.09028578
1418493_a_at	synuclein, alpha	Snca	0.8577	1.08381285	0.1086516
1444121_at	---	---	0.85771	1.06686068	0.09289409
1428617_at	host cell factor C2	Hcfc2	0.85771	-1.06434561	-0.0915451
1429770_at	protein geranylgeranyltransferase type I, beta subunit	Pggt1b	0.85772	-1.069703	-0.1005737

1415941_s_at	zinc finger, AN1-type domain 2A	Zfand2a	0.85772	-1.06944284	-0.1078006
1419007_at	zona pellucida glycoprotein 3	Zp3	0.85774	-1.06344224	-0.0959447
1425168_at	---	---	0.85775	-1.0561069	-0.0847407
1440957_at	defensin beta 29	Defb29	0.85775	-1.06587774	-0.0938355
1451098_at	chromatin modifying protein 1A	Chmp1a	0.85775	1.05995624	0.07902915
1435981_at	neuron navigator 2	Nav2	0.85776	1.06278291	0.08754816
1435730_at	RIKEN cDNA D930026N18 gene	D930026N18Rik	0.85777	1.1220769	0.1367939
1442944_at	expressed sequence C76555	C76555	0.85777	-1.07338378	-0.1213872
1422946_a_at	DNA methyltransferase (cytosine-5) 1	Dnmt1	0.85777	1.05316718	0.07268837
1430378_at	RIKEN cDNA 2900011G08 gene	2900011G08Rik	0.85778	-1.08001052	-0.1276215
1453934_at	RIKEN cDNA 4930535C22 gene	4930535C22Rik	0.85778	-1.06378489	-0.0893979
1439803_at	zinc finger protein 865	Zfp865	0.85779	1.07094111	0.09753997
1456532_at	platelet-derived growth factor, D polypeptide	Pdgfd	0.85788	-1.05514863	-0.0789503
1454157_a_at	phospholipase A2, group IID	Pla2g2d	0.85791	-1.07183784	-0.1096533
1456596_at	family with sequence similarity 70, member A	Fam70a	0.85791	1.09129068	0.10705626
1445216_at	---	---	0.85791	1.08762035	0.11032683
1445614_at	---	---	0.85791	-1.07172772	-0.1129888
1444349_at	---	---	0.85792	-1.07229386	-0.1131648
1418678_at	hyaluronan synthase 2	Has2	0.85792	1.10749378	0.1267044
1420071_at	---	---	0.85795	1.1161569	0.13388243
1450387_s_at	adenylate kinase 4	Ak4	0.85795	-1.0493825	-0.0716499
1441367_a_at	---	---	0.85795	1.08549027	0.11004
1455514_at	potassium voltage-gated channel, Shal-related family, member 1	Kcnd1	0.85796	1.06503656	0.08923184
1425445_a_at	claudin 18	Cldn18	0.85796	-1.07288607	-0.1041493
1441775_at	---	---	0.85797	-1.08197289	-0.1468938
1436994_a_at	histone cluster 1, H1c	Hist1h1c	0.85797	-1.07303801	-0.1328291
1427697_a_at	transformation related protein 73	Trp73	0.85798	-1.06535758	-0.0981925
1445443_at	---	---	0.85798	-1.06871887	-0.0959113
1416853_at	neurochondrin	Ncdn	0.85799	1.06388897	0.08682906
1440021_at	G protein-coupled receptor 20	Gpr20	0.85799	1.06826467	0.09274806
1424610_at	TruB pseudouridine (psi) synthase homolog 2 (E. coli)	Trub2	0.85799	-1.05486411	-0.0789817
1428373_at	inositol hexaphosphate kinase 2	Ip6k2	0.858	1.06982627	0.08997188
1418622_at	RAB2A, member RAS oncogene family	Rab2a	0.858	1.04681997	0.06531382
1446513_at	---	---	0.858	1.10083878	0.1181002
1444447_at	---	---	0.858	1.09463569	0.11609301
1434029_at	---	---	0.858	-1.04906222	-0.072165
1452277_at	arylsulfatase G	Arsg	0.85801	1.09188227	0.11364976
1455503_at	zinc finger protein 273 /// zinc finger protein 85, related sequence	Zfp273 /// Zfp85-rs1	0.85801	1.06274065	0.08754505
1457610_at	---	---	0.85801	-1.06871706	-0.1112935
1427862_at	Sec61 beta subunit	Sec61b	0.85802	-1.07246501	-0.1146434
1439996_at	TatD DNase domain containing 1	Tatdn1	0.85802	-1.07028519	-0.1011206
1446617_at	---	---	0.85802	1.0682313	0.09231245
1431914_at	phosphodiesterase 3A, cGMP inhibited	Pde3a	0.85802	-1.06533288	-0.0947757
1460441_at	zinc finger, X-linked, duplicated B	Zxdb	0.85803	-1.07500478	-0.1113333
1449766_at	Synaptotagmin VI	Syt6	0.85803	1.12030305	0.12350756
1425326_at	ATP citrate lyase	Acly	0.85803	1.05526761	0.07415774
1417241_at	archaelysin family metallopeptidase 2	Amz2	0.85803	1.0482173	0.06787693
1458245_at	sushi domain containing 1	Susd1	0.85804	1.07925694	0.10686684
1450281_a_at	thymus, brain and testes associated	Tbata	0.85804	-1.06211449	-0.0899268
1432490_a_at	phosphodiesterase 10A	Pde10a	0.85804	1.07686722	0.10274053
1416436_a_at	ubiquinol-cytochrome c reductase complex chaperone, CBP3 hom	Uqcc	0.85804	1.05745587	0.0798041
1446869_at	hypothetical LOC100503582	LOC100503582	0.85805	-1.07180273	-0.1085915
1432363_at	predicted gene 3336	Gm3336	0.85805	-1.0621689	-0.089543
1431956_at	RIKEN cDNA 5830453K13 gene	5830453K13Rik	0.85805	-1.07705903	-0.1154555
1450566_at	interleukin 3	Il3	0.85806	-1.07390651	-0.1062531
1435359_at	metastasis suppressor 1-like	Mtss1l	0.85806	1.07271678	0.10039254
1456923_at	transient receptor potential cation channel, subfamily M, membe	Trpm3	0.85806	-1.05706481	-0.0827868
1445154_at	---	---	0.85806	-1.07592282	-0.1095863
1436373_at	mitogen-activated protein kinase kinase kinase 10	Map3k10	0.85806	1.05553886	0.07789845
1421304_at	killer cell lectin-like receptor, subfamily A, member 2	Klra2	0.85806	1.0640488	0.08855865
1460138_at	---	---	0.85807	-1.06883407	-0.0967229
1426460_a_at	UDP-glucose pyrophosphorylase 2	Ugp2	0.85807	1.08352134	0.10602739
1440661_at	RIKEN cDNA A430010J10 gene	A430010J10Rik	0.85807	-1.06162508	-0.0908079
1451224_at	secretory carrier membrane protein 5	Scamp5	0.85807	1.05578683	0.07683926
1419086_at	fibroblast growth factor binding protein 1	Fgfbp1	0.85807	-1.06664759	-0.1137365
1425228_a_at	deoxyguanosine kinase	Dguok	0.85807	1.05903635	0.08064863
1451046_at	zinc finger protein, multitype 1	Zfpm1	0.85807	1.07322823	0.09676401
1430770_at	RIKEN cDNA 3110080E11 gene	3110080E11Rik	0.85808	-1.05246235	-0.0739556
1451689_a_at	SRY-box containing gene 10	Sox10	0.85808	1.06279353	0.08657743
1451936_a_at	thioredoxin reductase 2	Txnrd2	0.85808	1.06564908	0.09131915
1429840_at	RIKEN cDNA 4933439G12 gene	4933439G12Rik	0.85808	-1.05826541	-0.0856424
1445156_at	expressed sequence C81001	C81001	0.85808	1.08061453	0.10519667
1431966_at	ATP/GTP binding protein-like 3	Agbl3	0.85808	-1.04745598	-0.0693709
1421460_at	desmocollin 1	Dsc1	0.85808	-1.07325375	-0.10539
1419341_at	Eph receptor A8	Epha8	0.85809	-1.070613	-0.0995098
1440492_at	---	---	0.85809	-1.07112685	-0.1012176
1431516_at	RIKEN cDNA 4930565N06 gene	4930565N06Rik	0.85809	1.07222681	0.09934209
1438263_at	RIKEN cDNA 9430020K01 gene	9430020K01Rik	0.85809	1.08348695	0.10990904
1453061_at	elaC homolog 1 (E. coli)	Elac1	0.85809	-1.06359903	-0.0893803
1438717_a_at	oxysterol binding protein-like 6	Osbpl6	0.8581	1.06462659	0.08747657
1419142_at	cathepsin R	Ctsr	0.8581	1.08359026	0.10595153
1432291_at	RIKEN cDNA 0610033M10 gene	0610033M10Rik	0.8581	-1.06610366	-0.0954228
1448313_at	tripeptidyl peptidase I	Tpp1	0.8581	-1.04760964	-0.0673922
1435657_at	stonin 2	Ston2	0.8581	-1.06056345	-0.091217
1458272_at	---	---	0.8581	1.0844101	0.11053685
1420757_at	myogenic factor 5	Myf5	0.8581	1.05659053	0.07701221
1456359_at	peptidylprolyl isomerase domain and WD repeat containing 1	Ppwd1	0.8581	-1.04700053	-0.0668848
1421053_at	kinesin family member 1A	Kif1a	0.85811	1.09790048	0.11897767
1459106_at	---	---	0.85811	1.08286134	0.10814602
1459933_at	---	---	0.85811	1.05327229	0.073585
1421927_at	transducin (beta)-like 2	Tbl2	0.85811	1.06719915	0.09247604
1447672_x_at	protein serine kinase H1	Pskh1	0.85811	-1.06249999	-0.0926906
1443762_s_at	SET binding factor 2	Sbf2	0.85811	-1.0503551	-0.0715389
1459110_at	Myosin IXb	Myo9b	0.85812	1.09760338	0.11999911

1436263_at	myelin-associated oligodendrocytic basic protein	Mobp	0.85812	1.10046343	0.12021193
1451273_x_at	FtsJ methyltransferase domain containing 1	Ftsjd1	0.85812	1.08131098	0.10492366
1425890_at	lymphocyte antigen 6 complex, locus I	Ly6i	0.85812	-1.07642583	-0.1148245
1428158_at	AKT1 substrate 1 (proline-rich)	Akt1s1	0.85812	1.06572454	0.08648974
1422750_a_at	zinc finger, MYND domain containing 10	Zmynd10	0.85812	-1.07464727	-0.121653
1455549_at	SEC14 and spectrin domains 1	Sestd1	0.85813	-1.06867177	-0.1088219
1448649_at	glutamyl aminopeptidase	Enpep	0.85813	1.06666583	0.09013868
1435837_at	crystallin, alpha B	Cryab	0.85813	-1.06527879	-0.0943871
1449932_at	casein kinase 1, delta	Csnk1d	0.85813	1.06895205	0.08869329
1456385_x_at	UBX domain protein 10	Ubxn10	0.85813	1.08811056	0.11265768
1424110_a_at	non-metastatic cells 1, protein (NM23A) expressed in	Nme1	0.85813	1.05831692	0.07587011
1447228_at	ADP-ribosylation factor GTPase activating protein 2	Arfgap2	0.85813	-1.06158541	-0.0893052
1433238_at	RIKEN cDNA 4930448E06 gene	4930448E06Rik	0.85814	-1.06822835	-0.0972022
1436129_at	a disintegrin and metallopeptidase domain 6B	Adam6b	0.85814	1.07593965	0.1047666
1449152_at	cyclin-dependent kinase inhibitor 2B (p15, inhibits CDK4)	Cdkn2b	0.85814	1.0655421	0.09038539
1443629_at	neuron navigator 1	Nav1	0.85814	-1.07226216	-0.1145097
1435074_at	transmembrane protein 106B	Tmem106b	0.85814	-1.06013341	-0.0850716
1424439_at	RIKEN cDNA 1810065E05 gene	1810065E05Rik	0.85814	-1.06915193	-0.0983972
1434303_at	Ras association (RalGDS/AF-6) and pleckstrin homology domains	Raph1	0.85814	-1.06742671	-0.1122044
1443802_at	---	---	0.85814	-1.07008498	-0.1021088
1454385_at	RIKEN cDNA 1700018P08 gene	1700018P08Rik	0.85814	1.06832378	0.09502792
1451353_at	transmembrane 6 superfamily member 1	Tm6sf1	0.85815	-1.07489242	-0.111201
1443311_at	THAP domain containing 4	Thap4	0.85815	1.09386022	0.11459045
1436117_at	RIKEN cDNA A830010M20 gene	A830010M20Rik	0.85815	1.06770749	0.09348109
1436997_x_at	SH3-binding domain glutamic acid-rich protein like	Sh3bgrl	0.85816	-1.06259347	-0.0876313
1432895_at	RIKEN cDNA 9030409K20 gene	9030409K20Rik	0.85816	-1.07100537	-0.115266
1453923_at	coiled-coil domain containing 83	Ccdc83	0.85817	-1.06918554	-0.0968312
1439295_x_at	predicted gene 5595	Gm5595	0.85817	-1.0545716	-0.0776745
1421371_at	---	---	0.85818	-1.06922519	-0.1033005
1416876_at	parvin, gamma	Parvg	0.85818	-1.07197254	-0.1083212
1451327_a_at	transmembrane emp24 domain containing 1	Tmed1	0.85818	1.05022724	0.0699722
1417155_at	v-myc myelocytomatosis viral related oncogene, neuroblastoma	Mycn	0.85818	-1.04974344	-0.0730143
1427894_at	vasorin	Vasn	0.85818	1.07891906	0.10378477
1430190_at	coiled-coil domain containing 30	Ccdc30	0.85818	-1.06598063	-0.0938668
1451247_at	major facilitator superfamily domain containing 1	Mfsd1	0.85819	1.06174744	0.08273627
1450231_a_at	X-linked inhibitor of apoptosis	Xiap	0.8582	-1.06598978	-0.0934863
1422801_at	Ras-GTPase-activating protein SH3-domain binding protein 1	G3bp1	0.85821	1.04858058	0.06521631
1460002_at	tousled-like kinase 1	Tlk1	0.85821	1.06593911	0.09170171
1417822_at	DNA segment, Chr 17, human D6S56E 5	D17H6S56E-5	0.85822	1.0539582	0.07466218
1418973_at	basic leucine zipper nuclear factor 1	Blzf1	0.85823	-1.0560799	-0.0802166
1432002_at	RIKEN cDNA 2810471M01 gene	2810471M01Rik	0.85824	-1.07152109	-0.1181542
1441493_at	ELKS/RAB6-interacting/CAST family member 1	Erc1	0.85825	-1.0708176	-0.1018704
1445047_at	expressed sequence C79246	C79246	0.85826	1.08131103	0.10100284
1446188_at	centrosomal protein 97	Cep97	0.85827	-1.07085817	-0.1016049
1452375_at	aldehyde dehydrogenase 4 family, member A1	Aldh4a1	0.85827	1.05823458	0.07985563
1460702_at	TP53 regulated inhibitor of apoptosis 1	Triap1	0.85828	1.07085735	0.09095733
1416563_at	cytidine 5'-triphosphate synthase	Ctps	0.85828	1.04641715	0.06530968
1421902_at	mediator complex subunit 29	Med29	0.85828	1.11470059	0.13279685
1457129_at	hypothetical LOC100504796	LOC100504796	0.85829	1.09037766	0.11235453
1443192_at	Thymocyte selection associated	Themis	0.8583	1.0728714	0.10001333
1433584_at	predicted gene 5620 /// predicted gene 6682 /// tubulin, alpha 1A	Gm5620 /// Gm6682 /// T	0.8583	-1.0719387	-0.1087671
1437067_at	putative homeodomain transcription factor 2	Phtf2	0.8583	1.05418512	0.07491651
1417583_a_at	UTP3, small subunit (SSU) processome component, homolog (S. c	Utp3	0.8583	-1.04924157	-0.0719866
1455890_x_at	Small nuclear ribonucleoprotein N	Snrpn	0.8583	-1.06052959	-0.0885181
1454147_at	RIKEN cDNA 2810429I04 gene	2810429I04Rik	0.8583	1.0752623	0.09458887
1446240_at	---	---	0.8583	-1.06962987	-0.1013591
1440462_at	RIKEN cDNA B930068K11 gene	B930068K11Rik	0.8583	-1.07095036	-0.1011943
1438753_at	---	---	0.8583	-1.05922773	-0.0832045
1430903_at	RIKEN cDNA 4933416O17 gene	4933416O17Rik	0.85831	-1.06913342	-0.10088
1426674_at	eukaryotic translation initiation factor 3, subunit B	Eif3b	0.85831	1.04420248	0.06215643
1427501_at	RIKEN cDNA 4922501L14 gene	4922501L14Rik	0.85831	1.07966988	0.10472137
1454194_at	RIKEN cDNA 9330118I20 gene	9330118I20Rik	0.85832	1.07274729	0.09948466
1437312_at	bone morphogenetic protein receptor, type 1B	Bmpr1b	0.85832	-1.07713896	-0.1153204
1442149_at	TBC1 domain family, member 16	Tbc1d16	0.85832	-1.06365445	-0.0899892
1430731_at	keratin associated protein 2-4	Krtap2-4	0.85832	1.09190124	0.1157354
1423979_a_at	solute carrier family 25 (mitochondrial carrier, palmitoylcarnitine t	Slc25a29	0.85832	-1.07355019	-0.1180731
1443652_x_at	sprouty protein with EVH-1 domain 1, related sequence	Spred1	0.85832	-1.06936454	-0.1024026
1423299_at	thioredoxin-like 1	Txnl1	0.85833	1.05441396	0.0727387
1446068_at	adenosine kinase	Adk	0.85833	1.09629161	0.11522457
1433386_at	RIKEN cDNA 4933428C20 gene	4933428C20Rik	0.85833	1.07563937	0.10152869
1436503_at	cDNA sequence BC048546	BC048546	0.85834	-1.07401516	-0.1132647
1449692_at	protein kinase C, zeta	Prkcz	0.85834	1.09041945	0.11282322
1416400_at	pyrroline-5-carboxylate reductase-like	Pycrl	0.85834	1.04807564	0.06734496
1431620_at	hypothetical protein LOC100125594	LOC100125594	0.85835	1.07459663	0.10217873
1425932_a_at	CUGBP, Elav-like family member 1	Celf1	0.85835	-1.05445024	-0.0803466
1455164_at	Rho GTPase activating protein 31	Arhgap31	0.85835	1.07196387	0.09864429
1455536_at	RIKEN cDNA A630023A22 gene	A630023A22Rik	0.85836	1.08110884	0.10476774
1425355_at	acyl-CoA synthetase family member 2	Acsf2	0.85836	1.06299451	0.08247072
1448369_at	polymerase (DNA directed), alpha 2	Pola2	0.85836	1.06447394	0.0831587
1429078_a_at	COP9 (constitutive photomorphogenic) homolog, subunit 7a (Arab	Cops7a	0.85836	1.05778201	0.07739657
1421372_at	kallikrein 1-related pepidase b4	Klk1b4	0.85836	-1.06562211	-0.0996016
1450506_a_at	apoptosis enhancing nuclease	Aen	0.85836	1.05621177	0.07431746
1455322_at	tetratricopeptide repeat domain 21B	Ttc21b	0.85836	1.07530995	0.09867097
1423256_a_at	ATPase, H+ transporting, lysosomal V1 subunit G1	Atp6v1g1	0.85837	-1.05046451	-0.0761175
1435897_at	ribosomal protein L32	Rpl32	0.85837	1.03212999	0.04421409
1431582_at	eukaryotic translation initiation factor 6	Eif6	0.85838	-1.06071268	-0.0882978
1430142_at	transglutaminase 5	Tgm5	0.85838	1.0715631	0.09970093
1419332_at	EGF-like-domain, multiple 6	Egfl6	0.85839	-1.07929606	-0.1375544
1425849_at	cholinergic receptor, nicotinic, beta polypeptide 4	Chrnb4	0.85839	1.08089537	0.10323826
1443557_at	---	---	0.8584	-1.0674211	-0.0957443
1416146_at	heat shock protein 4	Hspa4	0.8584	-1.03665562	-0.0521336
1425288_at	sterile alpha motif domain containing 11	Samd11	0.8584	-1.07894917	-0.1497364
1446193_at	DNA segment, Chr 5, ERATO Doi 579, expressed	D5Ertd579e	0.8584	1.08113979	0.10344199
1452876_x_at	RIKEN cDNA 2610044O15 gene	2610044O15Rik	0.85841	-1.06961556	-0.109216

1459575_at	DNA segment, Chr 1, ERATO Doi 704, expressed	D1Ertd704e	0.85841	1.07484655	0.10175528
1444309_at	---	---	0.85842	-1.07940209	-0.1530726
1429159_at	inter-alpha (globulin) inhibitor H5	Itih5	0.85842	1.07406829	0.09930902
1455035_s_at	NOP56 ribonucleoprotein homolog (yeast)	Nop56	0.85843	1.04117427	0.05766398
1459120_at	erythrocyte protein band 4.1-like 5	Epb4.1l5	0.85844	-1.07334906	-0.1150231
1451921_a_at	nuclear factor of activated T-cells 5	Nfat5	0.85844	1.0693059	0.09520723
1446716_at	---	---	0.85844	-1.07087167	-0.1067775
1453901_at	Aldolase A, fructose-bisphosphate	Aldoa	0.85844	1.06281888	0.07626781
1460212_at	guanine nucleotide binding protein, alpha transducing 1	Gnat1	0.85844	1.08787791	0.11106601
1416860_s_at	inhibitor of growth family, member 1	Ing1	0.85845	-1.05005716	-0.0723856
1459711_at	sidekick homolog 1 (chicken)	Sdk1	0.85846	1.06475481	0.08995112
1422928_at	elastase, neutrophil expressed	Elane	0.85846	1.08503522	0.11165536
1435111_at	RIKEN cDNA 2310011E23 gene	2310011E23Rik	0.85846	-1.07045631	-0.1158563
1423515_at	sodium channel, voltage-gated, type VIII, alpha	Scn8a	0.85847	1.09242256	0.1163682
1440798_x_at	leucine rich repeat containing 6 (testis)	Lrrc6	0.85847	1.06754823	0.08882142
1423501_at	Max protein	Max	0.85847	-1.06857872	-0.1621131
1417800_at	poly (ADP-ribose) polymerase family, member 2	Parp2	0.85847	1.04623546	0.06477627
1441024_at	stromal interaction molecule 2	Stim2	0.85847	1.0717767	0.09878003
1454531_at	RIKEN cDNA C030043A13 gene	C030043A13Rik	0.85848	1.07650708	0.09572684
1420052_x_at	proteasome (prosome, macropain) subunit, beta type 1	Psmb1	0.85848	-1.05843555	-0.0847791
1440998_at	RIKEN cDNA 2310021P13 gene	2310021P13Rik	0.85848	1.06376161	0.08713201
1458095_at	---	---	0.85848	1.07587067	0.10283495
1448204_at	salvador homolog 1 (Drosophila)	Sav1	0.85849	1.05336116	0.07396703
1459540_at	---	---	0.8585	-1.07163343	-0.10292
1430683_at	dedicator of cytokinesis 6	Dock6	0.8585	-1.06879904	-0.099929
1457486_at	---	---	0.8585	-1.07223907	-0.1054436
1430979_a_at	peroxiredoxin 2	Prdx2	0.8585	1.06852001	0.09539408
1418452_at	guanine nucleotide binding protein (G protein), gamma 2	Gng2	0.85851	-1.07172539	-0.1079343
1444194_at	---	---	0.85852	-1.07148394	-0.1036468
1458193_at	peripheral myelin protein 2	Pmp2	0.85852	-1.07387072	-0.1187144
1438148_at	chemokine (C-X-C motif) ligand 3	Cxcl3	0.85852	1.05165	0.07215024
1433050_at	RIKEN cDNA 5730478J17 gene	5730478J17Rik	0.85852	-1.07167585	-0.1076987
1433564_at	diacylglycerol kinase, delta	Dgkd	0.85852	1.05450838	0.07426141
1456196_x_at	FK506 binding protein 1a	Fkbp1a	0.85853	-1.04338514	-0.0615157
1458198_at	Mitogen-activated protein kinase kinase kinase 12	Map3k12	0.85853	1.08305657	0.10469912
1424055_at	nuclear receptor coactivator 5	Ncoa5	0.85854	1.05702762	0.07808729
1429091_at	RIKEN cDNA 1600002K03 gene	1600002K03Rik	0.85854	1.06788295	0.09300754
1456979_at	---	---	0.85854	-1.07207364	-0.109601
1425617_at	DEAH (Asp-Glu-Ala-His) box polypeptide 9	Dhx9	0.85854	1.11364384	0.1228856
1417562_at	eukaryotic translation initiation factor 4E binding protein 1	Eif4ebp1	0.85855	1.04800283	0.06429313
1418365_at	cathepsin H	Ctsh	0.85855	1.07345045	0.10012534
1446796_at	---	---	0.85855	-1.07289044	-0.1045128
1454553_at	RIKEN cDNA 4930509E22 gene	4930509E22Rik	0.85856	1.07859467	0.10513158
1425197_at	protein tyrosine phosphatase, non-receptor type 2	Ptpn2	0.85856	1.05942849	0.08214635
1420668_a_at	Yip1 domain family, member 2	Yipf2	0.85857	-1.06180879	-0.0871193
1435674_at	Rho-related BTB domain containing 2	Rhobtb2	0.85858	1.06481042	0.08870019
1436045_at	testis specific 10	Tsga10	0.85858	1.07547115	0.09817212
1417063_at	complement component 1, q subcomponent, beta polypeptide	C1qb	0.85859	-1.0756503	-0.1154743
1450694_at	FK506 binding protein 2	Fkbp2	0.85859	1.05914008	0.0786227
1433054_at	integrin beta 2-like	Itgb2l	0.8586	-1.07832147	-0.140534
1426692_at	coiled-coil domain containing 97	Ccdc97	0.8586	1.0591656	0.07950643
1424938_at	six transmembrane epithelial antigen of the prostate 1	Steap1	0.85861	-1.06390162	-0.1119411
1436237_at	tetratricopeptide repeat domain 9	Ttc9	0.85861	1.17211016	0.17116691
1432838_at	---	---	0.85861	1.0677571	0.09318652
1419814_s_at	S100 calcium binding protein A1	S100a1	0.85861	-1.07215709	-0.1114509
1455782_at	sterile alpha motif domain containing 7	Samd7	0.85862	1.07063044	0.09146261
1423429_at	reproductive homeobox 5	Rhox5	0.85863	1.04646821	0.06539991
1420549_at	guanylate binding protein 1	Gbp1	0.85865	-1.07783784	-0.1409086
1456023_at	zinc finger protein 846	Zfp846	0.85866	-1.06474224	-0.0965405
1452777_a_at	negative regulator of ubiquitin-like proteins 1	Nub1	0.85869	1.05072537	0.06966918
1418600_at	Kruppel-like factor 1 (erythroid)	Klf1	0.8587	1.07189431	0.09943579
1428838_a_at	deoxycytidine kinase	Dck	0.85873	1.07070167	0.09028318
1455311_at	DiGeorge syndrome critical region gene 8	Dgcr8	0.85879	1.05243983	0.07100318
1427078_at	sorting nexin 19	Snx19	0.8588	1.06711038	0.08989447
1439116_at	---	---	0.85881	1.08799378	0.1112946
1426251_at	carboxypeptidase Z	Cpz	0.85885	-1.07531017	-0.1157825
1452347_at	myocyte enhancer factor 2A	Mef2a	0.85885	-1.0701215	-0.1300998
1425931_a_at	aryl hydrocarbon receptor nuclear translocator-like 2	Arntl2	0.85898	1.08552749	0.10841061
1423554_at	gamma-glutamyl carboxylase	Ggcx	0.85899	1.07823529	0.10424252
1439061_at	coiled-coil domain containing 84	Ccdc84	0.85913	1.06549791	0.08971878
1446996_at	---	---	0.8592	-1.06437703	-0.0926903
1439250_at	SLIT and NTRK-like family, member 3	Slitrk3	0.85921	1.07665301	0.10210324
1454776_at	euchromatic histone methyltransferase 1	Ehmt1	0.85925	1.05656101	0.07526731
1455096_at	fibronectin leucine rich transmembrane protein 2	Flrt2	0.85927	1.09198547	0.11253016
1447636_x_at	transmembrane and coiled-coil domains 4	Tmco4	0.85928	-1.06219028	-0.0897564
1444576_at	---	---	0.8593	1.08159499	0.10646387
1416429_a_at	catalase	Cat	0.85939	-1.0544413	-0.0784146
1443404_s_at	DNA segment, Chr 13, ERATO Doi 94, expressed	D13Ertd94e	0.85947	-1.04658334	-0.0670467
1434470_at	synaptotagmin XIII	Syt13	0.85947	1.09107496	0.11411331
1442799_x_at	NudC domain containing 3	Nudcd3	0.85948	1.06918151	0.09187627
1457793_a_at	Wolf-Hirschhorn syndrome candidate 1-like 1 (human)	Whsc1l1	0.8595	1.13099657	0.1413694
1430321_at	KRAB-A domain containing 1	Krba1	0.85951	1.05883928	0.0790821
1427335_at	RIKEN cDNA 6720456H20 gene	6720456H20Rik	0.85951	1.07215566	0.09808184
1438340_at	zinc finger protein 385C	Zfp385c	0.85951	-1.07157943	-0.1089814
1422093_at	wingless-related MMTV integration site 3A	Wnt3a	0.85951	1.09201501	0.11346741
1442419_at	---	---	0.85952	1.07400019	0.09881112
1455638_at	zinc finger protein 319	Zfp319	0.85953	1.06356231	0.08784896
1459173_at	protein tyrosine phosphatase, receptor type, D	Ptprd	0.85954	-1.06407237	-0.1024031
1420795_at	fibroblast growth factor 9	Fgf9	0.85955	-1.06749577	-0.0943134
1458260_at	---	---	0.85957	-1.07518085	-0.1098862
1420164_at	DNA segment, Chr 7, ERATO Doi 183, expressed	D7Ertd183e	0.85957	1.08890261	0.11064664
1459900_at	expressed sequence C79468	C79468	0.85957	-1.06636341	-0.0929295
1456335_at	predicted gene 106	Gm106	0.85958	1.12348426	0.13822895
1450871_a_at	branched chain aminotransferase 1, cytosolic	Bcat1	0.85959	-1.04611943	-0.0657053

1453449_at	RIKEN cDNA 1700058G18 gene	1700058G18Rik	0.85959	1.10585334	0.12360789
1424783_a_at	UDP glucuronosyltransferase 1 family, polypeptide A1 /// UDP gl	Ugt1a1 /// Ugt1a10 /// U	0.85959	1.06374854	0.08719732
1423216_a_at	family with sequence similarity 32, member A	Fam32a	0.8596	1.05399689	0.0744416
1417586_at	timeless homolog (Drosophila)	Timeless	0.85961	1.05209027	0.07006308
1428705_at	RIKEN cDNA 1700007K13 gene	1700007K13Rik	0.85961	1.0555942	0.07640252
1449313_at	kallikrein 1-related peptidase b5	Klk1b5	0.85965	-1.06778435	-0.0983949
1420347_at	palate, lung, and nasal epithelium associated	Plunc	0.85984	-1.06669254	-0.1010138
1419301_at	frizzled homolog 4 (Drosophila)	Fzd4	0.85997	1.08414371	0.10881803
1420143_at	ring finger and CCCH-type zinc finger domains 2	Rc3h2	0.85998	-1.07048452	-0.1000426
1415867_at	chaperonin containing Tcp1, subunit 4 (delta)	Cct4	0.85998	1.0355006	0.04940508
1443958_at	RIKEN cDNA 9030203C11 gene	9030203C11Rik	0.86001	-1.06515296	-0.095115
1459341_at	RIKEN cDNA 8030448K23 gene	8030448K23Rik	0.86003	-1.07439422	-0.1232281
1460032_at	---	---	0.86005	-1.07390795	-0.1369852
1432055_at	SAM and SH3 domain containing 1	Sash1	0.86006	1.14302828	0.15021713
1440219_at	---	---	0.86016	1.08378276	0.10748037
1421679_a_at	cyclin-dependent kinase inhibitor 1A (P21)	Cdkn1a	0.86028	-1.0630521	-0.1051301
1452823_at	glutathione S-transferase kappa 1	Gstk1	0.86035	1.06488216	0.08951942
1445916_at	---	---	0.86037	1.09004723	0.11162616
1449018_at	profilin 1	Pfn1	0.86042	1.04573251	0.05985103
1453831_at	transmembrane protein 138	Tmem138	0.86044	1.08214308	0.10563944
1420504_at	solute carrier family 6 (neurotransmitter transporter), member 14	Slc6a14	0.86045	1.05421106	0.07442452
1437100_x_at	proviral integration site 3	Pim3	0.86051	-1.04391657	-0.0625401
1448895_a_at	catenin (cadherin associated protein), alpha 2	Ctnna2	0.86064	-1.07281473	-0.1073286
1460142_at	DNA segment, Chr 13, ERATO Doi 324, expressed	D13Ertd324e	0.86065	1.07388531	0.10028044
1442589_at	---	---	0.86067	1.09123643	0.11396245
1420556_at	oxytocin	Oxt	0.86069	-1.05439805	-0.076424
1419793_at	DNA segment, Chr 5, ERATO Doi 615, expressed	D5Ertd615e	0.8607	-1.06573443	-0.0935378
1453538_at	cysteine-serine-rich nuclear protein 3	Csrnp3	0.86072	-1.06306971	-0.0900188
1425822_a_at	deltex 1 homolog (Drosophila)	Dtx1	0.86073	1.05415711	0.07408224
1442008_at	hypothetical LOC100502695	LOC100502695	0.86073	-1.07126564	-0.1040362
1450431_a_at	neural precursor cell expressed, developmentally down-regulated	Nedd4	0.86073	1.06771962	0.0835615
1450259_a_at	signal transducer and activator of transcription 5A	Stat5a	0.86074	1.08435219	0.10538228
1447969_at	minichromosome maintenance complex component 9	Mcm9	0.86074	1.10280473	0.12061676
1419872_at	colony stimulating factor 1 receptor	Csf1r	0.86076	1.08572818	0.10845072
1459313_at	---	---	0.86077	1.09398497	0.11441503
1445648_at	---	---	0.86079	1.06538791	0.09111568
1446261_at	DNA segment, Chr 1, ERATO Doi 507, expressed	D1Ertd507e	0.86083	-1.05973279	-0.0886614
1447773_x_at	RIKEN cDNA 6330409D20 gene	6330409D20Rik	0.86099	-1.07070505	-0.1158251
1458430_at	expressed sequence C87436	C87436	0.861	-1.06830707	-0.0997894
1424746_at	kinesin family member 1C	Kif1c	0.86102	-1.06586677	-0.1135032
1429636_at	RIKEN cDNA 1700010D01 gene /// predicted gene 5941	1700010D01Rik /// Gm59	0.86104	-1.07025753	-0.1019165
1440060_at	predicted pseudogene 5126	Gm5126	0.86104	1.07464516	0.10038598
1436566_at	Rab40b, member RAS oncogene family	Rab40b	0.86105	1.04563376	0.0626563
1418819_at	ADP-ribosylation factor-like 8B	Arl8b	0.86105	1.0561342	0.07583016
1455656_at	B and T lymphocyte associated	Btla	0.86105	1.0759301	0.10408297
1421493_a_at	regulator of G-protein signaling 20	Rgs20	0.86107	1.0738975	0.09701653
1450646_at	cytochrome P450, family 51	Cyp51	0.86107	1.05205443	0.07294933
1452706_a_at	family with sequence similarity 116, member B	Fam116b	0.86108	1.0577677	0.08063211
1450927_at	leucine-zipper-like transcriptional regulator, 1	Lztr1	0.86108	1.05762091	0.07973545
1437017_at	expressed sequence AI480653	AI480653	0.86121	1.06781745	0.08870397
1417075_at	family with sequence similarity 136, member A	Fam136a	0.86122	1.04467003	0.06285073
1432054_at	golgi autoantigen, golgin subfamily a, 1	Golga1	0.86126	-1.06941264	-0.1016516
1458845_at	DNA segment, Chr 4, ERATO Doi 264, expressed	D4Ertd264e	0.86126	-1.0701837	-0.1080185
1451437_at	zinc finger, DHHC domain containing 20	Zdhhc20	0.86128	-1.05455248	-0.0835979
1457029_at	protein phosphatase 4, regulatory subunit 1-like, pseudogene	Ppp4r1l-ps	0.86129	-1.07079772	-0.1084876
1450715_at	cytochrome P450, family 1, subfamily a, polypeptide 2	Cyp1a2	0.86129	1.08434674	0.11072721
1447395_at	defensin beta 21	Defb21	0.86133	1.06595035	0.09016702
1433419_at	RIKEN cDNA 4930405A07 gene	4930405A07Rik	0.86133	-1.06928378	-0.0971339
1446369_at	armadillo repeat containing 1	Armc1	0.86135	1.06910593	0.09613726
1457915_at	RIKEN cDNA 4833442J19 gene	4833442J19Rik	0.8616	1.06911736	0.09159514
1456825_at	---	---	0.86161	-1.06924085	-0.1062276
1416311_s_at	tubulin, alpha 3A /// tubulin, alpha 3B	Tuba3a /// Tuba3b	0.86162	-1.05415792	-0.0858173
1432137_at	RIKEN cDNA 4933434P08 gene	4933434P08Rik	0.86162	1.07032073	0.09457403
1421402_at	metastasis associated 3	Mta3	0.86163	1.07752881	0.09944328
1419681_a_at	prokineticin 2	Prok2	0.86163	-1.06640434	-0.0987789
1440172_s_at	RIKEN cDNA 1700113H08 gene	1700113H08Rik	0.86165	1.06716184	0.08714411
1459131_at	---	---	0.86165	-1.07286464	-0.1145039
1424827_a_at	casein kinase 1, alpha 1	Csnk1a1	0.86165	-1.04233625	-0.0621866
1431064_at	dipeptidylpeptidase 8	Dpp8	0.86165	1.06598612	0.09054515
1442157_at	---	---	0.86168	-1.06095622	-0.0873728
1416940_at	peptidylprolyl isomerase F (cyclophilin F)	Ppif	0.86169	1.04680098	0.06563416
1452665_at	tetratricopeptide repeat domain 27	Ttc27	0.86169	1.06341821	0.08104106
1419751_x_at	cDNA sequence AB056442	AB056442	0.86171	1.06447298	0.08103417
1438678_at	RIKEN cDNA 1500011K16 gene	1500011K16Rik	0.86172	1.07618909	0.09372356
1439332_at	DNA-damage-inducible transcript 4-like	Ddit4l	0.86174	1.06051108	0.08286149
1435292_at	TBC1 domain family, member 4	Tbc1d4	0.86175	1.10344275	0.11315404
1416824_at	RIKEN cDNA B230118H07 gene	B230118H07Rik	0.86176	1.07058937	0.0903487
1424636_at	coiled-coil domain containing 47	Ccdc47	0.86176	1.05733153	0.07864914
1433142_at	RIKEN cDNA 4921504P05 gene	4921504P05Rik	0.86178	-1.066903	-0.0950576
1442633_at	---	---	0.86179	1.07530459	0.09886451
1426326_at	zinc finger protein 91	Zfp91	0.86179	-1.05233671	-0.074404
1429026_at	hexamthylene bis-acetamide inducible 2	Hexim2	0.86184	1.06096749	0.08338473
1423159_at	dihydrolipoamide dehydrogenase	Dld	0.86185	-1.05100591	-0.0757948
1431490_at	RIKEN cDNA A930033C23 gene	A930033C23Rik	0.86185	-1.06822253	-0.1046626
1447685_x_at	E26 avian leukemia oncogene 2, 3' domain	Ets2	0.86187	-1.05812009	-0.0846576
1429341_at	ornithine decarboxylase antizyme 3	Oaz3	0.86187	1.0814686	0.10273723
1439193_at	paired related homeobox protein-like 1	Prrxl1	0.86189	-1.0672907	-0.0942462
1446984_at	---	---	0.8619	1.06511113	0.08747118
1423212_at	polyhomeotic-like 1 (Drosophila)	Phc1	0.86196	1.04096476	0.057371
1434185_at	acetyl-Coenzyme A carboxylase alpha	Acaca	0.86197	1.04983786	0.07003931
1451894_a_at	sodium channel, voltage-gated, type VIII, alpha	Scn8a	0.86199	1.09257447	0.11446994
1447498_at	ectodysplasin-A receptor	Edar	0.862	1.05490782	0.07683251
1426551_at	SID1 transmembrane family, member 1	Sidt1	0.86201	1.11877007	0.13314006
1441980_at	RIKEN cDNA C030007I09 gene	C030007I09Rik	0.86201	1.06349759	0.08556262

1441441_at	proline rich Gla (G-carboxyglutamic acid) 1	Prrg1	0.86201	1.07117441	0.09772604
1425368_a_at	numb gene homolog (Drosophila)	Numb	0.86203	1.0735576	0.09854785
1443183_at	HECT, UBA and WWE domain containing 1	Huwe1	0.86204	-1.06678256	-0.100095
1419222_at	thromboxane A2 receptor	Tbxa2r	0.86204	1.06272899	0.08757883
1437507_at	GATA zinc finger domain containing 2B	Gatad2b	0.86204	-1.0597001	-0.0919166
1457151_at	---	---	0.86205	1.08322822	0.10943637
1417333_at	RAS p21 protein activator 4	Rasa4	0.86208	1.07747553	0.09919876
1431404_at	transmembrane protein 56	Tmem56	0.86214	-1.07406411	-0.1130586
1442038_at	RNA binding motif protein 26	Rbm26	0.86217	-1.05393719	-0.0779669
1457280_at	---	---	0.86227	1.07749022	0.10341736
1439819_at	expressed sequence AU015263	AU015263	0.8623	-1.07427254	-0.1185546
1430832_at	RIKEN cDNA 4930426I24 gene	4930426I24Rik	0.8623	-1.0740369	-0.1192862
1443989_at	tripartite motif-containing 9	Trim9	0.8623	-1.05050886	-0.0714979
1436217_at	zinc finger protein 148	Zfp148	0.86231	1.13127633	0.14149221
1459585_at	growth arrest specific 6	Gas6	0.86231	1.06809154	0.09315299
1434521_at	regulatory factor X, 7	Rfx7	0.86233	-1.05344216	-0.0779541
1446474_at	---	---	0.86234	1.12322472	0.1358837
1459544_at	---	---	0.86235	-1.06320597	-0.0913605
1442494_at	expressed sequence C79242	C79242	0.86235	1.07843847	0.10490232
1434539_at	leucine rich repeat protein 3, neuronal	Lrrn3	0.86235	1.06733893	0.0922632
1441551_at	myopalladin	Mypn	0.86236	-1.06919358	-0.0977527
1459399_at	---	---	0.86236	1.0735199	0.09851477
1438798_at	RIKEN cDNA 4931406P16 gene	4931406P16Rik	0.86237	-1.06430501	-0.0986458
1447173_at	leucine rich repeat containing 31	Lrrc31	0.86238	-1.067775	-0.0969932
1430823_at	RIKEN cDNA 2700029L08 gene	2700029L08Rik	0.86238	-1.06963746	-0.0977903
1454068_at	RIKEN cDNA 4930470H14 gene	4930470H14Rik	0.86238	-1.07345271	-0.1107369
1447962_at	---	---	0.8624	-1.0699497	-0.1005445
1426621_a_at	protein phosphatase 2 (formerly 2A), regulatory subunit B (PR 52)	Ppp2r2b	0.8624	1.07246419	0.09649757
1448387_at	ring-box 1	Rbx1	0.8624	1.0462391	0.06138408
1437401_at	insulin-like growth factor 1	Igf1	0.86241	-1.07421597	-0.1383174
1441769_at	---	---	0.86241	-1.07588733	-0.1431186
1456897_at	---	---	0.86241	-1.06764776	-0.0957783
1418511_at	dermatopontin	Dpt	0.86241	-1.06553956	-0.0917062
1419769_at	CD22 antigen	Cd22	0.86242	1.07280508	0.09798112
1432166_at	RIKEN cDNA E130018N17 gene	E130018N17Rik	0.86242	1.08535107	0.10931723
1457406_at	---	---	0.86243	-1.06771709	-0.0977501
1457288_at	ankyrin 3, epithelial	Ank3	0.86243	-1.07077244	-0.1067635
1445656_at	---	---	0.86243	-1.06438812	-0.0905661
1439004_at	ribosomal protein S6 kinase, polypeptide 5	Rps6ka5	0.86245	1.12131093	0.13564698
1443721_x_at	strawberry notch homolog 2 (Drosophila)	Sbno2	0.86245	1.11140103	0.12825654
1442885_at	SREBF chaperone	Scap	0.86245	-1.07330775	-0.1045796
1459455_at	---	---	0.86246	-1.0690837	-0.0980434
1437240_at	phosphoglucomutase 2	Pgm2	0.86246	1.07893591	0.10522258
1418957_at	src homology three (SH3) and cysteine rich domain	Stac	0.86247	1.0774254	0.10222738
1447184_at	expressed sequence AU021760	AU021760	0.86249	-1.06573071	-0.10268
1417208_at	alpha-methylacyl-CoA racemase	Amacr	0.86249	1.08571306	0.10679013
1416194_at	cytochrome P450, family 4, subfamily b, polypeptide 1	Cyp4b1	0.86249	-1.07196527	-0.1056068
1459782_x_at	ATP-binding cassette, sub-family C (CFTR/MRP), member 5	Abcc5	0.86249	-1.06853176	-0.0961273
1434032_at	hypothetical LOC100503649	LOC100503649	0.8625	1.07108932	0.09575976
1440284_at	---	---	0.86251	-1.05063178	-0.0713219
1434409_at	armadillo repeat containing, X-linked 6	Armcx6	0.86251	-1.06872854	-0.0982782
1425770_at	RIKEN cDNA A730028G07 gene	A730028G07Rik	0.86251	-1.0756483	-0.1150713
1420552_s_at	testis specific gene A8	Tsga8	0.86251	1.07186965	0.09518865
1423816_at	CAAX box 1 homolog A (human) /// CAAX box 1 homolog B (huma	Cxx1a /// Cxx1b	0.86251	1.05734197	0.07814736
1443397_at	nuclear receptor coactivator 1	Ncoa1	0.86252	1.12835586	0.13714004
1421401_at	potassium large conductance calcium-activated channel, subfami	Kcnmb1	0.86253	-1.07077197	-0.106871
1421015_s_at	polymerase (DNA directed), epsilon 3 (p17 subunit)	Pole3	0.86253	1.0755052	0.09405815
1415904_at	lipoprotein lipase	Lpl	0.86254	1.08162279	0.1039075
1422859_a_at	ribosomal protein L23	Rpl23	0.86255	1.06537066	0.08394754
1439903_at	YjeF N-terminal domain containing 3	Yjefn3	0.86255	1.07984358	0.10572395
1451581_at	protein phosphatase 6, regulatory subunit 2	Ppp6r2	0.86256	-1.07265689	-0.1024747
1436486_x_at	whirlin	Whrn	0.86256	1.0646531	0.08922642
1448288_at	nuclear factor I/B	Nfib	0.86258	-1.06697243	-0.0970743
1457710_at	zinc fingers and homeoboxes 3	Zhx3	0.86258	-1.07127422	-0.1020766
1428186_at	potassium channel tetramerisation domain containing 6	Kctd6	0.86259	-1.05616599	-0.0837891
1423838_s_at	RIKEN cDNA 2400003C14 gene	2400003C14Rik	0.8626	1.04877696	0.06771179
1425829_a_at	STEAP family member 4	Steap4	0.86261	-1.07240641	-0.1127371
1451193_x_at	tetratricopeptide repeat domain 4	Ttc4	0.86261	1.04650165	0.0654774
1448269_a_at	kelch-like 13 (Drosophila)	Klhl13	0.86261	-1.0501007	-0.0745726
1454864_at	zinc finger protein 592	Zfp592	0.86262	1.07091332	0.09442202
1447372_at	aldehyde dehydrogenase 16 family, member A1	Aldh16a1	0.86262	1.07780837	0.09824161
1435391_at	RNA binding motif protein 33	Rbm33	0.86262	1.06847711	0.09017465
1434263_at	RIKEN cDNA F630110N24 gene	F630110N24Rik	0.86263	1.05658816	0.07926263
1420897_at	synaptosomal-associated protein 23	Snap23	0.86263	-1.05032496	-0.0709519
1445989_at	---	---	0.86263	1.0910369	0.10840553
1448342_at	mitogen-activated protein kinase 10	Mapk10	0.86263	-1.07185759	-0.1098122
1447501_at	---	---	0.86264	1.07270411	0.09455641
1431480_at	RIKEN cDNA 4833419E13 gene	4833419E13Rik	0.86264	-1.06120697	-0.0869605
1458322_x_at	RIKEN cDNA E230008N13 gene	E230008N13Rik	0.86264	1.07653508	0.09721589
1446971_at	DNA segment, Chr 10, Wayne State University 159, expressed	D10Wsu159e	0.86265	-1.06537689	-0.1035004
1444095_a_at	hypothetical LOC100048701	LOC100048701	0.86267	-1.06664796	-0.1005793
1457098_at	tubulin tyrosine ligase-like family, member 11	Ttll11	0.86268	-1.06504178	-0.0910009
1422516_a_at	fibroblast growth factor (acidic) intracellular binding protein	Fibp	0.86268	1.04927424	0.06870284
1423265_at	multiple inositol polyphosphate histidine phosphatase 1	Minpp1	0.86269	1.04826717	0.06722744
1433899_x_at	TSC22 domain family, member 1	Tsc22d1	0.86269	1.05213787	0.06981199
1449759_at	DNA segment, Chr 11, ERATO Doi 326, expressed	D11Ertd326e	0.86269	1.07338266	0.09974229
1456767_at	leucine rich repeat and fibronectin type III domain containing 3	Lrfn3	0.8627	-1.06526437	-0.0932704
1429184_at	GTPase, very large interferon inducible 1	Gvin1	0.86271	-1.07092391	-0.1009896
1456598_at	predicted gene 16367 /// predicted gene 16427 /// predicted ger	Gm16367 /// Gm16427 //	0.86271	-1.07490667	-0.1101566
1442411_at	---	---	0.86271	-1.06738956	-0.0962837
1418638_at	histocompatibility 2, class II, locus Mb1 /// histocompatibility 2, cl	H2-DMb1 /// H2-DMb2	0.86272	1.07209419	0.09756601
1442114_at	GPN-loop GTPase 1	Gpn1	0.86272	1.0605158	0.08368872
1429188_at	COX11 homolog, cytochrome c oxidase assembly protein (yeast)	Cox11	0.86273	-1.0482203	-0.0693513
1416458_at	ADP-ribosylation factor 2	Arf2	0.86273	1.06165541	0.08446428

1455768_at	Niemann Pick type C2	Npc2	0.86273	-1.07238118	-0.1081778
1429673_at	RIKEN cDNA 5830407E08 gene	5830407E08Rik	0.86274	1.06815498	0.09199949
1442801_x_at	transmembrane 7 superfamily member 3	Tm7sf3	0.86274	-1.07168756	-0.1116579
1422809_at	regulating synaptic membrane exocytosis 2	Rims2	0.86275	-1.06807633	-0.0995412
1453274_at	coiled-coil domain containing 103	Ccdc103	0.86275	-1.06993126	-0.1025816
1454197_a_at	coiled-coil domain containing 86	Ccdc86	0.86275	1.05248944	0.07237706
1436068_at	zinc finger and BTB domain containing 10	Zbtb10	0.86275	-1.06513231	-0.1032577
1451048_at	methionine aminopeptidase 2	Metap2	0.86277	-1.06115798	-0.0892743
1434397_at	zinc finger, DHHC domain containing 17	Zdhhc17	0.86277	1.07532319	0.09588348
1457750_at	DNA segment, Chr 16, ERATO Doi 780, expressed	D16Ertd780e	0.86277	-1.07558684	-0.1062185
1423312_at	trophoblast glycoprotein	Tpbg	0.86279	-1.06291299	-0.088478
1454365_at	RIKEN cDNA 4930554P06 gene	4930554P06Rik	0.86281	-1.06696957	-0.094323
1449966_s_at	calcium binding protein 39-like	Cab39l	0.86281	-1.05475397	-0.0793119
1439489_at	G protein-coupled receptor 120	Gpr120	0.86282	1.07192157	0.09862272
1452225_at	RIKEN cDNA 2010106G01 gene	2010106G01Rik	0.86283	-1.06700795	-0.1063646
1430123_a_at	aldo-keto reductase family 1, member A4 (aldehyde reductase)	Akr1a4	0.86286	1.04240894	0.057615
1445455_at	DNA segment, Chr 3, ERATO Doi 270, expressed	D3Ertd270e	0.86287	-1.0660195	-0.0944381
1429034_at	essential meiotic endonuclease 1 homolog 2 (S. pombe)	Eme2	0.86289	1.07661341	0.1022486
1460321_at	contactin 4	Cntn4	0.8629	-1.06733802	-0.1002854
1429585_s_at	myoneurin	Mynn	0.8629	1.06224026	0.08309635
1453151_at	solute carrier family 26, member 3	Slc26a3	0.86291	1.06576357	0.08789929
1422406_at	interferon alpha 9	Ifna9	0.86291	-1.07225023	-0.1153345
1418738_at	sodium channel, voltage-gated, type I, beta	Scn1b	0.86293	1.05961518	0.08276626
1454245_at	CUB and Sushi multiple domains 3	Csmd3	0.86295	-1.06719785	-0.0938921
1433677_at	splicing factor, suppressor of white-apricot homolog (Drosophila)	Sfswap	0.86297	-1.05018799	-0.0721336
1424948_x_at	histocompatibility 2, D region locus 1 /// histocompatibility 2, K1, I	H2-D1 /// H2-K1 /// LOC1	0.863	-1.06810556	-0.0977746
1424027_at	paxillin	Pxn	0.86309	1.06582926	0.08454085
1425600_a_at	phospholipase C, beta 1	Plcb1	0.8631	-1.05478161	-0.0780358
1431202_at	hect domain and RLD 3	Herc3	0.86311	-1.07321493	-0.1165326
1447447_s_at	serine/arginine repetitive matrix 1	Srrm1	0.86311	-1.04474387	-0.0644059
1428247_at	vacuolar protein sorting 26 homolog B (yeast)	Vps26b	0.86311	1.04876563	0.06809765
1419180_at	B-cell CLL/lymphoma 9-like	Bcl9l	0.86313	-1.06593595	-0.1050632
1440277_at	expressed sequence AU041474	AU041474	0.86314	1.07243023	0.09523461
1440185_x_at	SUMO1 activating enzyme subunit 1	Sae1	0.86314	1.06280609	0.08641419
1425028_a_at	tropomyosin 2, beta	Tpm2	0.86316	-1.06011722	-0.0909258
1452134_at	transmembrane protein 175	Tmem175	0.86318	1.08205589	0.10364471
1442903_at	RIKEN cDNA 4732423E21 gene	4732423E21Rik	0.86326	-1.06884011	-0.0986396
1432425_at	leucine rich repeat containing 67	Lrrc67	0.8633	-1.06345176	-0.1049189
1440265_at	Jun proto-oncogene related gene d	Jund	0.86337	1.08113334	0.10493825
1430883_at	RIKEN cDNA 4933402C05 gene	4933402C05Rik	0.86343	1.08688462	0.11017271
1446767_at	---	---	0.86345	1.06611175	0.09119586
1424280_at	motile sperm domain containing 1	Mospd1	0.86345	1.06848883	0.09212363
1456002_at	xeroderma pigmentosum, complementation group A	Xpa	0.86345	-1.0596925	-0.0881756
1437019_at	RIKEN cDNA 2200001I15 gene	2200001I15Rik	0.86345	-1.0479275	-0.0695323
1442007_at	zinc finger, MYM-type 5	Zmym5	0.8635	1.08763321	0.11025718
1440034_at	signal transducing adaptor molecule (SH3 domain and ITAM mot	Stam2	0.86351	-1.07052361	-0.0993375
1431880_at	RIKEN cDNA 3100003M19 gene	3100003M19Rik	0.86353	-1.07424777	-0.1197678
1422880_at	synaptophysin-like protein	Sypl	0.86367	-1.05101293	-0.079122
1450103_a_at	cytohesin 2	Cyth2	0.86369	1.05039386	0.06937453
1454471_at	RIKEN cDNA 3110062G12 gene	3110062G12Rik	0.86371	1.05796289	0.07868019
1436271_at	apolipoprotein L 7b /// apolipoprotein L 7e	Apol7b /// Apol7e	0.86372	-1.07030755	-0.106286
1434615_x_at	---	---	0.86374	-1.07057393	-0.0996612
1455752_a_at	transmembrane protein 134	Tmem134	0.86375	1.05778998	0.0762278
1454690_at	inhibitor of kappaB kinase gamma	Ikbkg	0.86375	1.07224565	0.09601079
1456558_s_at	expressed sequence C87977	C87977	0.86376	1.10297535	0.11650475
1427038_at	preproenkephalin	Penk	0.86376	-1.06544479	-0.0963611
1433095_at	predicted gene 13872	Gm13872	0.86377	-1.06537988	-0.0969908
1459089_at	predicted gene 11756 /// predicted gene 11757 /// predicted ger	Gm11756 /// Gm11757 //	0.86379	-1.06842981	-0.1027997
1418953_at	F-box protein 16	Fbxo16	0.86379	-1.07030329	-0.1115452
1443702_at	microtubule-associated protein 4	Mtap4	0.8638	-1.07212805	-0.1228909
1447288_at	---	---	0.8638	-1.06343137	-0.0903059
1434412_x_at	STIP1 homology and U-Box containing protein 1	Stub1	0.86381	1.0620505	0.08032882
1422764_at	microtubule-associated protein, RP/EB family, member 1	Mapre1	0.86381	-1.04640153	-0.065636
1431189_a_at	fumarylacetoacetate hydrolase domain containing 2A	Fahd2a	0.86381	-1.07267824	-0.1099998
1423750_a_at	splicing factor 1	Sf1	0.86381	-1.05405845	-0.0896026
1429621_at	cullin-associated and neddylation-dissociated 2 (putative)	Cand2	0.86382	1.05843557	0.0805324
1435881_at	poly(rC) binding protein 2	Pcbp2	0.86382	1.04578405	0.06436401
1453839_a_at	peptidase inhibitor 16	Pi16	0.86383	-1.06659757	-0.095762
1425374_at	2'-5' oligoadenylate synthetase 3	Oas3	0.86384	-1.06985799	-0.101962
1444502_at	PET112-like (yeast)	Pet112l	0.86384	-1.07151711	-0.1051365
1453314_x_at	RIKEN cDNA 2610039C10 gene	2610039C10Rik	0.86384	-1.04992006	-0.0733972
1427601_at	gap junction protein, delta 4	Gjd4	0.86384	1.07482224	0.10291521
1430487_at	AU RNA binding protein/enoyl-coenzyme A hydratase	Auh	0.86385	1.08172238	0.10394222
1423539_at	postmeiotic segregation increased 2 (S. cerevisiae)	Pms2	0.86386	1.05536583	0.07658236
1430649_at	brain and acute leukemia, cytoplasmic	Baalc	0.86387	-1.06320713	-0.0900347
1436472_at	schlafen 9	Slfn9	0.86388	-1.06840418	-0.0981551
1448686_at	interleukin 16	Il16	0.86388	1.06934399	0.09354335
1441870_s_at	polycystic kidney disease 2	Pkd2	0.86389	-1.05028241	-0.0730271
1449448_at	mediator of RNA polymerase II transcription, subunit 9 homolog (	Med9	0.8639	1.08161008	0.0990441
1437522_x_at	growth hormone	Gh	0.8639	1.06810365	0.09497939
AFFX-GapdhMur/	glyceraldehyde-3-phosphate dehydrogenase	Gapdh	0.86391	-1.02246128	-0.0323244
1454888_at	prefoldin 4	Pfdn4	0.86392	1.04506847	0.06310386
1438342_at	family with sequence similarity 113, member A	Fam113a	0.86393	-1.06661972	-0.094094
1441272_at	Matrin 3	Matr3	0.86393	1.07082713	0.09024182
1430746_at	RIKEN cDNA 1110050P16 gene	1110050P16Rik	0.86393	1.06954665	0.09527173
1438051_at	tetratricopeptide repeat domain 14	Ttc14	0.86393	-1.07593975	-0.1474452
1434004_at	deoxyhypusine synthase	Dhps	0.86393	1.05178876	0.07225717
1431058_at	HIV TAT specific factor 1	Htatsf1	0.86394	-1.05884911	-0.0828296
1450952_at	phospholamban	Pln	0.86395	1.086859	0.10949765
1420041_at	---	---	0.86395	-1.07052716	-0.1042522
1416752_at	LIM domain binding 3	Ldb3	0.86396	-1.07278736	-0.1029558
1447595_x_at	RIKEN cDNA 1810012K16 gene	1810012K16Rik	0.86396	-1.06584853	-0.0925817
1418545_at	WASP family 1	Wasf1	0.86396	-1.05698874	-0.0942701
1444514_at	RIKEN cDNA B930096F20 gene	B930096F20Rik	0.86396	-1.0681483	-0.1167256

1437128_a_at	RIKEN cDNA A630033E08 gene	A630033E08Rik	0.86397	-1.06816473	-0.0969337
1445004_a_at	PITPNM family member 3	Pitpnm3	0.86397	-1.06885144	-0.0961625
1443875_at	RIMS binding protein 3	Rimbp3	0.86398	-1.06559687	-0.0930672
1453491_at	pyridine nucleotide-disulphide oxidoreductase domain 2	Pyroxd2	0.86398	-1.06796721	-0.0996944
1433990_at	lipoma HMGIC fusion partner-like 3	Lhfpl3	0.86399	1.08807251	0.10798246
1459522_s_at	glycogenin	Gyg	0.864	1.06540023	0.08909834
1417128_at	pleckstrin homology domain containing, family O member 1	Plekho1	0.86401	1.07361078	0.09950441
1420921_at	CD151 antigen	Cd151	0.86401	-1.07367701	-0.1116229
1457580_at	chromodomain helicase DNA binding protein 8	Chd8	0.86401	1.0704785	0.09703738
1447826_x_at	intermediate filament family orphan 1	Iffo1	0.86401	1.05742656	0.07963085
1427328_a_at	CLIP associating protein 2	Clasp2	0.86401	1.08205797	0.100021
1431543_at	RIKEN cDNA 9130001E16 gene	9130001E16Rik	0.86401	1.06867202	0.09394441
1451009_at	ring finger protein 151	Rnf151	0.86401	1.06563952	0.08808056
1435356_at	F-box protein 42	Fbxo42	0.86404	1.05128072	0.06954292
1438050_x_at	predicted gene 9222	Gm9222	0.86405	-1.0753634	-0.1378487
1459949_at	---	---	0.86406	1.14190438	0.14889634
1418416_x_at	pregnancy-specific glycoprotein 23	Psg23	0.86406	-1.07346851	-0.1067654
1421009_at	radical S-adenosyl methionine domain containing 2	Rsad2	0.86407	1.08734584	0.11091779
1451032_at	four and a half LIM domains 4	Fhl4	0.86407	1.07178879	0.09418646
1445969_at	---	---	0.86407	-1.06962718	-0.0992605
1442994_at	---	---	0.86408	1.06917	0.08895726
1449306_at	heat shock factor 2	Hsf2	0.86408	-1.06839381	-0.1004775
1431942_at	WW domain-containing oxidoreductase	Wwox	0.86408	-1.06784034	-0.095591
1430985_at	RIKEN cDNA 1810027O10 gene	1810027O10Rik	0.86409	1.07592898	0.09147986
1444059_at	RIKEN cDNA 6330415G19 gene	6330415G19Rik	0.86409	-1.07269724	-0.128639
1441340_at	centrosomal protein 68	Cep68	0.8641	1.05742475	0.07956721
1419120_at	lymphoblastomic leukemia 1	Lyl1	0.8641	1.11717457	0.13141315
1427686_at	vomeronasal 2, receptor 88	Vmn2r88	0.86411	1.06190162	0.08223754
1442387_at	---	---	0.86411	-1.0738984	-0.1111543
1448625_at	golgi autoantigen, golgin subfamily a, 2	Golga2	0.86412	1.06868808	0.08891013
1418381_at	zinc finger protein 148	Zfp148	0.86412	-1.06137981	-0.0867788
1429939_at	protein phosphatase 6, regulatory subunit 3	Ppp6r3	0.86412	1.08133331	0.10500583
1448860_at	rad and gem related GTP binding protein 2	Rem2	0.86412	1.08447307	0.10826878
1439590_at	X Kell blood group precursor related family member 8 homolog	Xkr8	0.86414	1.07331092	0.1002043
1458779_at	RIKEN cDNA 8030445P17 gene	8030445P17Rik	0.86414	-1.05635206	-0.0811821
1459768_x_at	RAP1, GTP-GDP dissociation stimulator 1	Rap1gds1	0.86415	1.0688924	0.09246605
1443241_at	---	---	0.86415	1.0665071	0.09137344
1440876_at	---	---	0.86416	-1.06866895	-0.0992346
1435011_x_at	aldo-keto reductase family 1, member A4 (aldehyde reductase)	Akr1a4	0.86417	1.04314916	0.06054776
1420351_at	tumor necrosis factor receptor superfamily, member 4	Tnfrsf4	0.86417	1.0595015	0.08240678
1439885_at	homeobox C5	Hoxc5	0.86417	-1.06749262	-0.1011685
1419137_at	SH3/ankyrin domain gene 3	Shank3	0.86417	-1.05394507	-0.0760379
1459291_at	prostaglandin reductase 2	Ptgr2	0.86417	1.08742801	0.10877871
1419666_x_at	nuclear protein 1	Nupr1	0.86418	-1.06323139	-0.1189746
1435313_at	CD200 receptor 4	Cd200r4	0.86418	1.04771604	0.06604539
1458181_at	RIKEN cDNA 9030624J02 gene	9030624J02Rik	0.86419	-1.07095279	-0.1014894
1434031_at	zinc finger protein 692, pseudogene	Zfp692-ps	0.86419	1.06018775	0.08209293
1436561_at	suppressor of variegation 3-9 homolog 2 (Drosophila)	Suv39h2	0.86419	1.06549721	0.08912745
1455005_s_at	solute carrier family 5 (sodium/glucose cotransporter), member 2	Slc5a2	0.86419	-1.06376428	-0.0970973
1452967_at	gametocyte specific factor 1	Gtsf1	0.86419	1.06972481	0.09349479
1452977_at	zinc fingers and homeoboxes 3	Zhx3	0.8642	-1.06866733	-0.1095136
1452102_at	coatomer protein complex, subunit beta 2 (beta prime)	Copb2	0.8642	1.04900235	0.06693603
1446541_at	RIKEN cDNA 4930434E21 gene	4930434E21Rik	0.86421	1.08207154	0.10566724
1455589_at	RIKEN cDNA 1700100M05 gene	1700100M05Rik	0.86421	-1.06370872	-0.0999208
1436166_at	storkhead box 2	Stox2	0.86421	1.07208998	0.09791878
1435340_at	lysine (K)-specific demethylase 4A	Kdm4a	0.86422	1.06688561	0.08516685
1438682_at	phosphatidylinositol 3-kinase, regulatory subunit, polypeptide 1 (p	Pik3r1	0.86422	1.06540747	0.08929049
1416028_a_at	hematological and neurological expressed sequence 1	Hn1	0.86423	1.05409975	0.07241862
1442989_at	---	---	0.86423	-1.0707432	-0.1183309
1448408_at	Hermansky-Pudlak syndrome 1 homolog (human)	Hps1	0.86423	1.06481009	0.0868666
1440543_at	HEAT repeat containing 5A	Heatr5a	0.86425	-1.07191206	-0.1058829
1438994_at	hyaluronoglucosaminidase 5	Hyal5	0.86425	1.07573926	0.09940333
1456696_x_at	RMI1, RecQ mediated genome instability 1, homolog (S. cerevisia	Rmi1	0.86425	1.10905204	0.12420286
1418532_at	frizzled homolog 2 (Drosophila)	Fzd2	0.86426	1.06601503	0.09054604
1442444_at	---	---	0.86427	-1.06676244	-0.1048181
1432829_at	enolase 1, alpha non-neuron /// predicted gene 5506 /// alpha-e	Eno1 /// Gm5506 /// LOC	0.86429	-1.07414891	-0.122607
1445309_at	---	---	0.86429	-1.0655537	-0.0920487
1425225_at	Fc receptor, IgG, low affinity IV	Fcgr4	0.86429	-1.06561219	-0.091793
1435137_s_at	RIKEN cDNA 1200015M12 gene /// RIKEN cDNA A130040M12 ge	1200015M12Rik /// A130	0.86429	-1.07287654	-0.1121117
1456331_at	RIKEN cDNA 9130023H24 gene	9130023H24Rik	0.86429	-1.06587064	-0.0921644
1423745_at	synaptosomal-associated protein, 47	Snap47	0.86431	1.05312737	0.07335298
1419792_at	kinesin family member 20B	Kif20b	0.86431	-1.06066029	-0.0897706
1432266_at	enhancer trap locus 4	Etl4	0.86431	-1.07299923	-0.1060328
1446435_at	predicted gene 9947	Gm9947	0.86431	-1.07492431	-0.1083004
1451792_a_at	praja1, RING-H2 motif containing	Pja1	0.86431	-1.06424854	-0.0950754
1459951_at	ribosomal protein S6 kinase, polypeptide 1	Rps6kb1	0.86431	-1.07251355	-0.1196832
1444837_at	---	---	0.86431	1.07432248	0.0974318
1428770_at	DIP2 disco-interacting protein 2 homolog A (Drosophila)	Dip2a	0.86431	-1.05902503	-0.0840207
1460379_at	homeobox B4	Hoxb4	0.86432	-1.0710444	-0.105712
1435178_x_at	anaphase-promoting complex subunit 5	Anapc5	0.86432	1.04624531	0.06342399
1440503_at	---	---	0.86432	-1.07251191	-0.1131371
1419218_at	vang-like 2 (van gogh, Drosophila)	Vangl2	0.86432	1.06151963	0.08304922
1449652_at	NudC domain containing 1	Nudcd1	0.86433	1.06656079	0.09215191
1451392_at	ELMO/CED-12 domain containing 3	Elmod3	0.86433	1.06702862	0.09227815
1418405_at	hepatocyte growth factor activator	Hgfac	0.86433	-1.0708067	-0.1008038
1432886_at	RIKEN cDNA 5730488B01 gene	5730488B01Rik	0.86434	-1.07015087	-0.1027852
1417194_at	superoxide dismutase 2, mitochondrial	Sod2	0.86434	-1.05776665	-0.0821098
1438904_at	NHS-like 1	Nhsl1	0.86434	-1.05901997	-0.0852782
1453297_at	golgi autoantigen, golgin subfamily a, 7B	Golga7b	0.86434	1.07157571	0.09426891
1416202_at	prohibitin 2	Phb2	0.86435	1.0497381	0.06581089
1433215_at	---	---	0.86435	1.08877342	0.11245678
1457689_at	SET binding factor 2	Sbf2	0.86435	-1.06197067	-0.08989
1444852_at	---	---	0.86435	1.08614414	0.10903909
1425837_a_at	CCR4 carbon catabolite repression 4-like (S. cerevisiae)	Ccrn4l	0.86436	-1.05199001	-0.083578

1445226_at	cDNA sequence BC023969	BC023969	0.86436	-1.05764986	-0.0968791
1458137_at	---	---	0.86436	-1.07183174	-0.1117014
1441868_x_at	DC-STAMP domain containing 2	Dcst2	0.86436	1.08779276	0.10815377
1424855_at	oleoyl-ACP hydrolase	Olah	0.86437	-1.06893426	-0.0976625
1441028_at	DNA segment, Chr 2, ERATO Doi 210, expressed	D2Ertd210e	0.86437	1.07053336	0.09654776
1429438_at	BCL6 interacting corepressor	Bcor	0.86437	1.07091716	0.09130203
1454367_at	---	---	0.86437	-1.06875207	-0.0984867
1437335_x_at	polymerase (DNA-directed), delta interacting protein 3	Poldip3	0.86437	-1.04787837	-0.0723559
1455837_at	tripartite motif-containing 36	Trim36	0.86437	1.08033236	0.10144694
1420272_at	SAM domain and HD domain, 1	Samhd1	0.86438	1.08547188	0.10485098
1429699_at	3-oxoacyl-ACP synthase, mitochondrial	Oxsm	0.86438	1.0753675	0.10078975
1446312_at	---	---	0.86438	-1.06817357	-0.0985756
1439194_at	RIKEN cDNA C030048H21 gene	C030048H21Rik	0.86438	1.06964954	0.09656751
1459616_at	---	---	0.86438	-1.0657796	-0.0972819
1451766_at	3-hydroxy-3-methylglutaryl-Coenzyme A reductase	Hmgcr	0.86439	1.07219238	0.09894545
1445117_at	---	---	0.86439	-1.06784182	-0.1076231
1417270_at	WD repeat domain 12	Wdr12	0.8644	-1.06497949	-0.0979282
1417931_at	N-deacetylase/N-sulfotransferase (heparan glucosaminyl) 2	Ndst2	0.8644	1.05528015	0.07748648
1455066_s_at	melanoma inhibitory activity 3	Mia3	0.8644	1.05678322	0.07696626
1458900_at	---	---	0.86441	1.07121761	0.09715265
1435712_a_at	predicted gene 10260 /// ribosomal protein S18	Gm10260 /// Rps18	0.86441	1.02340618	0.03277491
1419069_at	RAB guanine nucleotide exchange factor (GEF) 1	Rabgef1	0.86441	-1.05923392	-0.086804
1420491_at	eukaryotic translation initiation factor 2, subunit 1 alpha	Eif2s1	0.86441	1.08573394	0.105876
1437692_x_at	annexin A2	Anxa2	0.86442	1.0781137	0.10419848
1457995_at	---	---	0.86442	-1.06782082	-0.1038912
1426103_a_at	estrogen receptor 2 (beta)	Esr2	0.86442	1.07128718	0.09678813
1426162_a_at	ribosomal protein L7	Rpl7	0.86442	-1.02576912	-0.0379265
1443075_at	---	---	0.86442	1.06279685	0.08509315
1426046_a_at	Rab geranylgeranyl transferase, a subunit	Rabggta	0.86443	-1.04806469	-0.0682121
1419295_at	cAMP responsive element binding protein 3-like 1	Creb3l1	0.86443	1.05688943	0.07810473
1444391_at	---	---	0.86443	-1.07084385	-0.110474
1425219_x_at	guanine nucleotide binding protein (G protein), gamma transducin	Gngt1	0.86445	1.09931729	0.11950032
1419838_s_at	polo-like kinase 4 (Drosophila)	Plk4	0.86445	-1.04676038	-0.0675704
1444800_at	---	---	0.86445	-1.07264459	-0.108932
1457190_at	---	---	0.86446	-1.06696976	-0.0973081
1450975_at	calcium channel, voltage-dependent, gamma subunit 4	Cacng4	0.86446	-1.06837113	-0.0966203
1433072_at	RIKEN cDNA 4933425E08 gene	4933425E08Rik	0.86446	1.1037655	0.12317822
1422695_at	tweety homolog 1 (Drosophila)	Ttyh1	0.86446	-1.06740218	-0.095308
1432451_at	RIKEN cDNA 4930445E18 gene	4930445E18Rik	0.86446	-1.06364499	-0.0930447
1415690_at	mitochondrial ribosomal protein L27	Mrpl27	0.86446	1.0457496	0.06377187
1440655_at	SH3-domain GRB2-like (endophilin) interacting protein 1	Sgip1	0.86447	1.07713092	0.09574425
1433526_at	kelch-like 8 (Drosophila)	Klhl8	0.86447	1.06251024	0.08514343
1421112_at	NK2 transcription factor related, locus 2 (Drosophila)	Nkx2-2	0.86447	-1.0643257	-0.09024
1429398_at	RIKEN cDNA 5730446D14 gene	5730446D14Rik	0.86448	-1.06360535	-0.0991618
1417287_at	histocompatibility 13	H13	0.86449	1.05487241	0.07394188
1436179_a_at	DnaJ (Hsp40) homolog, subfamily C, member 5	Dnajc5	0.86449	1.05838137	0.07731916
1419571_at	solute carrier family 28 (sodium-coupled nucleoside transporter),	Slc28a3	0.86449	-1.06718902	-0.0941068
1442680_at	---	---	0.86449	1.10218127	0.12026705
1457753_at	toll-like receptor 13	Tlr13	0.8645	1.07265995	0.09991014
1454960_at	MAD homolog 3 (Drosophila)	Smad3	0.8645	1.06357561	0.08543737
1449316_at	cytochrome P450, family 4, subfamily f, polypeptide 15	Cyp4f15	0.8645	1.06633904	0.09258091
1456136_at	Kinesin family member C1	Kifc1	0.8645	1.12804773	0.13570618
1444136_at	---	---	0.86451	1.0805876	0.1042265
1450158_at	rad and gem related GTP binding protein 1	Rem1	0.86452	1.07785803	0.10327261
1446560_at	protease, serine, 23	Prss23	0.86453	-1.05542958	-0.0782682
1415864_at	2,3-bisphosphoglycerate mutase	Bpgm	0.86454	1.05890914	0.08200634
1418150_at	myotubularin related protein 4	Mtmr4	0.86454	1.07776049	0.1004185
1438517_at	WW domain-containing oxidoreductase	Wwox	0.86455	1.07673829	0.10044089
1447648_at	RIKEN cDNA 1700021N20 gene	1700021N20Rik	0.86455	1.07320567	0.09612517
1429347_at	BCL2-like 14 (apoptosis facilitator)	Bcl2l14	0.86456	-1.07372618	-0.1135677
1453263_at	N(alpha)-acetyltransferase 35, NatC auxiliary subunit	Naa35	0.86456	-1.05285064	-0.077385
1417086_at	platelet-activating factor acetylhydrolase, isoform 1b, subunit 1	Pafah1b1	0.86458	1.05439337	0.07405854
1458977_at	Riken cDNA A530021J07 gene	A530021J07Rik	0.86458	1.04496072	0.0622705
1451952_at	prokineticin 2	Prok2	0.86459	1.0573538	0.07929838
1427392_at	Down syndrome cell adhesion molecule-like 1	Dscaml1	0.8646	-1.07237885	-0.1154957
1419008_at	neuropeptide Y receptor Y5	Npy5r	0.8646	-1.06797144	-0.097293
1453757_at	hect domain and RLD 6	Herc6	0.8646	1.07430428	0.0974587
1445248_at	---	---	0.86461	1.10590424	0.12365286
1433654_at	meningioma expressed antigen 5 (hyaluronidase)	Mgea5	0.86462	1.04512435	0.06364851
1444161_at	RIKEN cDNA A530020G20 gene	A530020G20Rik	0.86462	1.07460395	0.09859757
1421248_at	synapsin III	Syn3	0.86462	1.12063463	0.1335599
1434248_at	protein kinase C, eta	Prkch	0.86463	-1.0672836	-0.0964946
1443736_at	predicted gene 13648	Gm13648	0.86464	1.06930395	0.09305335
1459485_at	---	---	0.86464	1.09481962	0.11468706
1435635_at	protein-L-isoaspartate (D-aspartate) O-methyltransferase domain	Pcmtd1	0.86464	-1.06674337	-0.1063233
1417172_at	ubiquitin-conjugating enzyme E2L 6	Ube2l6	0.86464	-1.05936387	-0.0845425
1442873_at	fidgetin	Fign	0.86464	1.05785998	0.07905895
1432828_at	---	---	0.86465	-1.06557102	-0.2432183
1442581_at	kinase suppressor of ras 1	Ksr1	0.86465	1.11163475	0.12663454
1424538_at	ubiquitin-like 4	Ubl4	0.86466	1.06953973	0.08909146
1455498_at	G-protein-coupled receptor 50	Gpr50	0.86466	1.06489949	0.08855888
1417233_at	coiled-coil-helix-coiled-coil-helix domain containing 4	Chchd4	0.86466	1.04270501	0.06030166
1426395_s_at	eukaryotic translation initiation factor 3, subunit J	Eif3j	0.86467	-1.0520859	-0.0753449
1443298_at	F-box and WD-40 domain protein 13	Fbxw13	0.86467	-1.06342596	-0.0907374
1429548_at	testis expressed gene 22	Tex22	0.86467	1.0784465	0.09617976
1434823_x_at	myeloma overexpressed 2	Myeov2	0.86468	1.05834518	0.07552074
1433809_at	DEAD (Asp-Glu-Ala-Asp) box polypeptide 5	Ddx5	0.86468	1.10327081	0.1176252
1446300_at	Tripartite motif-containing 72	Trim72	0.86468	-1.06922964	-0.1071201
1442167_at	cytoplasmic FMR1 interacting protein 2	Cyfip2	0.86468	1.06264008	0.08401285
1417796_at	G protein pathway suppressor 2	Gps2	0.86469	1.08512177	0.10382362
1426634_at	solute carrier family 5 (sodium/glucose cotransporter), member 9	Slc5a9	0.86469	-1.06074339	-0.089162
1443377_at	a disintegrin and metallopeptidase domain 1a	Adam1a	0.86469	-1.06601125	-0.0986836
1445287_at	---	---	0.86469	-1.06628909	-0.0930275
1448940_at	tripartite motif-containing 21	Trim21	0.8647	1.06209603	0.08523273

1439183_at	alkaline ceramidase 1	Acer1	0.8647	-1.06079562	-0.0863985
1429798_s_at	CKLF-like MARVEL transmembrane domain containing 5	Cmtm5	0.8647	-1.06299793	-0.0927295
1425885_a_at	potassium voltage-gated channel, shaker-related subfamily, beta	Kcnab2	0.8647	1.09613479	0.11767158
1450057_at	SYS1 Golgi-localized integral membrane protein homolog (S. cere	Sys1	0.8647	1.05611298	0.07768421
1442367_at	ATPase, class VI, type 11C	Atp11c	0.86471	1.09910983	0.12013014
1430336_at	guanylate cyclase 2g	Gucy2g	0.86471	1.0797161	0.10216079
1450725_s_at	carbonic anhydrase 14	Car14	0.86471	-1.06615206	-0.096483
1430728_at	keratin associated protein 5-5	Krtap5-5	0.86471	-1.06935404	-0.1029276
1419132_at	toll-like receptor 2	Tlr2	0.86472	1.07141757	0.09362497
1447044_at	---	---	0.86472	1.08140807	0.10543583
1416317_a_at	STAM binding protein	Stambp	0.86472	-1.06206703	-0.0886132
1442491_at	Dipeptidylpeptidase 10	Dpp10	0.86472	1.05970978	0.08169625
1438124_at	vomeronasal type-2 receptor 26-like /// vomeronasal 2, receptor	LOC100505273 /// Vmn2	0.86472	1.05631389	0.07771251
1445338_at	---	---	0.86472	1.07233009	0.09858166
1442207_at	autophagy related 16 like 2 (S. cerevisiae)	Atg16l2	0.86473	1.10919147	0.12437502
1444878_at	---	---	0.86474	1.06260879	0.08713198
1458341_x_at	phosphodiesterase 5A, cGMP-specific	Pde5a	0.86474	1.06727185	0.09356736
1449275_at	family with sequence similarity 119, member A	Fam119a	0.86474	1.09040894	0.1068707
1433919_at	ankyrin repeat and SOCS box-containing 4	Asb4	0.86475	1.07966631	0.10212519
1424159_at	family with sequence similarity 134, member C	Fam134c	0.86475	1.06359434	0.08450322
1451897_a_at	neighbor of Brca1 gene 1	Nbr1	0.86476	1.05697579	0.0766912
1449834_at	MAGI family member, X-linked	Magix	0.86476	-1.06587044	-0.0925326
1426204_a_at	opioid receptor-like 1	Oprl1	0.86476	-1.06491807	-0.0915841
1439792_at	---	---	0.86477	1.07324468	0.0992378
1447572_at	RIKEN cDNA 1700052I22 gene	1700052I22Rik	0.86478	1.08554038	0.11029087
1454719_at	rhomboid domain containing 3	Rhbdd3	0.8648	1.06759302	0.08899938
1447741_x_at	acyl-Coenzyme A dehydrogenase family, member 10	Acad10	0.86481	-1.06157023	-0.0877739
1454856_x_at	ribosomal protein L35	Rpl35	0.86482	1.02788787	0.03872973
1427692_a_at	calcium/calmodulin-dependent serine protein kinase (MAGUK fa	Cask	0.86482	-1.06345656	-0.091778
1436211_at	THO complex 4	Thoc4	0.86485	1.06716266	0.08996994
1457912_at	RIKEN cDNA E130317F20 gene	E130317F20Rik	0.86486	1.07553085	0.10056436
1437218_at	fibronectin 1	Fn1	0.86487	1.12085926	0.1283314
1438687_at	lymphocyte transmembrane adaptor 1	Lax1	0.86487	1.08719935	0.10661571
1423130_a_at	serine/arginine-rich splicing factor 5	Srsf5	0.86487	-1.04647758	-0.0708155
1424921_at	bone marrow stromal cell antigen 2	Bst2	0.86488	-1.05353535	-0.0795764
1438882_at	---	---	0.86489	1.06720232	0.09258964
1446002_at	---	---	0.86489	-1.07054462	-0.1013001
1431196_at	ATPase, Ca++-sequestering	Atp2c1	0.8649	1.10045508	0.11727459
1422399_a_at	RAB23, member RAS oncogene family	Rab23	0.8649	-1.05722029	-0.0851595
1436075_at	secreted frizzled-related sequence protein 5	Sfrp5	0.86493	-1.06095684	-0.0860501
1450253_a_at	mitogen-activated protein kinase kinase kinase 4	Map3k4	0.86493	1.05507878	0.07631929
1425804_at	H6 homeobox 2	Hmx2	0.86494	1.1036874	0.12301541
1445000_at	---	---	0.86495	-1.07201771	-0.1112987
1458532_at	5-methyltetrahydrofolate-homocysteine methyltransferase	Mtr	0.86495	-1.06694139	-0.1080372
1432366_at	RIKEN cDNA 4930447K03 gene	4930447K03Rik	0.86495	-1.07131972	-0.1060434
1434493_at	RIKEN cDNA 1810022K09 gene	1810022K09Rik	0.86497	-1.05716449	-0.0819986
1427070_at	sorting nexin family member 21	Snx21	0.86497	1.06513399	0.09075092
1455520_at	protein phosphatase 2, regulatory subunit B (B56), gamma isofor	Ppp2r5c	0.86498	1.05144278	0.07158048
1426175_a_at	tryptase alpha/beta 1	Tpsab1	0.86499	1.0838324	0.10807941
1425953_at	---	---	0.86499	1.08345516	0.10813067
1442991_at	beta-1,3-glucuronyltransferase 2 (glucuronosyltransferase S)	B3gat2	0.865	1.06167638	0.08432369
1420033_s_at	protein phosphatase 2, regulatory subunit B, delta isoform	Ppp2r2d	0.86501	-1.04469386	-0.0634869
1444571_at	---	---	0.86501	-1.06649037	-0.1089307
1425454_a_at	interleukin 12a	Il12a	0.86504	1.09398135	0.11525859
1453131_at	CD300 antigen like family member G	Cd300lg	0.86509	1.08643973	0.11067222
1436739_at	angiotensin II receptor, type 1a	Agtr1a	0.86511	-1.07028262	-0.0987623
1449999_a_at	calcium channel, voltage-dependent, alpha2/delta subunit 1	Cacna2d1	0.86513	-1.07045084	-0.1030195
1445946_at	---	---	0.86513	-1.06617747	-0.0943287
1459945_at	predicted gene 9989	Gm9989	0.86514	1.06385236	0.08273317
1439851_at	---	---	0.86515	1.0752959	0.09994145
1453460_at	RIKEN cDNA 1700012I11 gene	1700012I11Rik	0.86515	1.08077837	0.10612147
1433081_at	RIKEN cDNA 4933407I18 gene	4933407I18Rik	0.86517	1.06800187	0.09310174
1442815_at	Peroxisomal biogenesis factor 19	Pex19	0.86518	-1.07321079	-0.1053715
1436563_at	RIKEN cDNA 4932441J04 gene	4932441J04Rik	0.8652	-1.06897751	-0.1014572
1459319_at	Polymerase (DNA directed), alpha 1	Pola1	0.8652	1.10827049	0.12351185
1427801_at	keratin associated protein 16-9	Krtap16-9	0.86521	-1.05852431	-0.0903705
1433237_at	RIKEN cDNA 3830422I06 gene	3830422I06Rik	0.86521	1.07197462	0.09819296
1442809_at	sodium channel, voltage-gated, type IX, alpha	Scn9a	0.86521	-1.05820094	-0.0873575
1444712_at	heat shock transcription factor family member 5	Hsf5	0.86523	-1.06376948	-0.093772
1444907_at	expressed sequence AU022138	AU022138	0.86525	-1.06212292	-0.0931722
1422434_a_at	RIKEN cDNA 2210010C04 gene	2210010C04Rik	0.86525	1.05519729	0.07607263
1424277_at	HAUS augmin-like complex, subunit 7	Haus7	0.86526	1.05484449	0.07484521
1418466_at	ring finger protein 141	Rnf141	0.86527	-1.0656189	-0.0946691
1427886_at	nuclear pore membrane protein 121	Pom121	0.86528	-1.05032377	-0.075176
1443530_at	---	---	0.86529	1.07138935	0.09709853
1450306_at	zona pellucida glycoprotein 1	Zp1	0.8653	-1.06923255	-0.1024614
1421265_a_at	RNA binding motif protein 38	Rbm38	0.8653	1.06259936	0.08066578
1443795_at	transcription factor 19	Tcf19	0.8653	-1.06740304	-0.0994762
1447772_at	pleckstrin homology domain-containing, family A (phosphoinositi	Plekha3	0.86531	1.0791305	0.10111873
1431008_at	histocompatibility 2, Q region locus 6 /// histocompatibility 2, Q re	H2-Q6 /// LOC68395	0.86531	-1.06760389	-0.1006219
1460007_at	thymocyte nuclear protein 1	Thyn1	0.86533	1.08699951	0.10878406
1426713_s_at	glutamyl-prolyl-tRNA synthetase	Eprs	0.86534	-1.06358253	-0.1396243
1448483_a_at	NADH dehydrogenase (ubiquinone) 1 beta subcomplex, 2	Ndufb2	0.86534	1.07825856	0.09280405
1457332_at	protein arginine N-methyltransferase 3	Prmt3	0.86535	-1.06976659	-0.108186
1420162_at	expressed sequence AA409749	AA409749	0.86535	1.08219025	0.10462735
1426724_at	calponin 3, acidic	Cnn3	0.86538	-1.04544722	-0.0669976
1449768_at	---	---	0.86541	-1.06495832	-0.0916764
1459449_at	---	---	0.86546	1.0646139	0.08762468
1445067_at	---	---	0.86549	-1.07073479	-0.1063213
1458141_at	---	---	0.8655	1.07532888	0.101808
1426111_x_at	interferon regulatory factor 3	Irf3	0.86553	1.04780865	0.06705723
1425103_at	angiotensin I converting enzyme (peptidyl-dipeptidase A) 2	Ace2	0.86554	-1.07042854	-0.0984147
1436427_at	PRP4 pre-mRNA processing factor 4 homolog B (yeast)	Prpf4b	0.86555	-1.05761887	-0.0812689
1459257_at	---	---	0.86555	-1.06619208	-0.0987648

1440641_at	vacuolar protein sorting 29 (S. pombe)	Vps29	0.86557	-1.07459343	-0.1485006
1445489_at	tocopherol (alpha) transfer protein-like	Ttpal	0.86557	1.07214159	0.09771819
1419824_a_at	aminoadipate-semialdehyde dehydrogenase	Aasdh	0.86557	1.05345208	0.0727065
1441714_at	predicted gene 6600	Gm6600	0.86558	1.05429324	0.07110383
1454099_at	RIKEN cDNA 4930593A02 gene	4930593A02Rik	0.86559	-1.06854334	-0.0978527
1427628_at	T-cell receptor beta, variable 8.2	Tcrb-V8.2	0.86559	1.08309258	0.1076344
1419349_a_at	cytochrome P450, family 2, subfamily d, polypeptide 9	Cyp2d9	0.8656	-1.06988145	-0.1078783
1438866_at	glutamate receptor ionotropic, NMDA3A	Grin3a	0.8656	1.07370918	0.09675223
1460483_at	RIKEN cDNA 2610034E01 gene	2610034E01Rik	0.8656	1.07529919	0.09705079
1447821_at	cut-like homeobox 2	Cux2	0.8656	1.07767867	0.09986292
1428619_at	family with sequence similarity 36, member A	Fam36a	0.8656	1.06629591	0.08518375
1458829_at	---	---	0.86562	-1.06886551	-0.0986347
1439047_s_at	RecQ protein-like	Recql	0.86562	-1.05109969	-0.076283
1419873_s_at	colony stimulating factor 1 receptor	Csf1r	0.86563	-1.06529471	-0.1044729
1460606_at	hydroxysteroid (17-beta) dehydrogenase 13	Hsd17b13	0.86563	-1.05498301	-0.0867776
1422529_s_at	calsequestrin 2	Casq2	0.86563	1.07092665	0.09575705
1418603_at	arginine vasopressin receptor 1A	Avpr1a	0.86564	-1.06553647	-0.0928779
1452069_a_at	THAP domain containing 7	Thap7	0.86564	1.07408165	0.09453147
1442625_at	---	---	0.86564	1.09352044	0.11497185
1417926_at	non-SMC condensin II complex, subunit G2	Ncapg2	0.86565	-1.05366558	-0.0805441
1444388_at	---	---	0.86566	-1.05691918	-0.0807669
1446803_at	---	---	0.86567	-1.07237765	-0.1052268
1449524_at	ectodysplasin-A	Eda	0.86568	1.06957065	0.09508167
1453518_at	PDZ domain containing 11	Pdzd11	0.86568	1.07236079	0.09485917
1420833_at	vesicle-associated membrane protein 2	Vamp2	0.86568	-1.07052329	-0.1216061
1442268_a_at	hypothetical LOC100503627	LOC100503627	0.86569	-1.05778948	-0.0825355
1452868_at	ubiquitin specific peptidase 24	Usp24	0.86569	-1.05327958	-0.0779262
1435384_at	ubiquitin-conjugating enzyme E2N	Ube2n	0.8657	1.0662764	0.08755421
1417602_at	period homolog 2 (Drosophila)	Per2	0.8657	-1.0613008	-0.0866908
1436782_at	seminal vesicle secretory protein 3A /// seminal vesicle secretory	Svs3a /// Svs3b	0.86571	-1.06958304	-0.1078843
1422485_at	MAD homolog 4 (Drosophila)	Smad4	0.86571	-1.04827756	-0.0719192
1433460_at	tetratricopeptide repeat domain 7B	Ttc7b	0.86571	-1.05052774	-0.0794941
1421319_at	prostaglandin F2 receptor negative regulator	Ptgfrn	0.86572	1.07172646	0.09490104
1418652_at	chemokine (C-X-C motif) ligand 9	Cxcl9	0.86573	1.06646088	0.09281539
1455896_a_at	potassium channel, subfamily K, member 1	Kcnk1	0.86574	1.06622828	0.09093267
1442196_at	RIKEN cDNA A930006K02 gene /// hypothetical LOC100503120	A930006K02Rik /// LOC10	0.86575	1.0956594	0.11733541
1417458_s_at	CDC28 protein kinase regulatory subunit 2	Cks2	0.86575	1.03972428	0.05566069
1446680_at	---	---	0.86575	1.0518019	0.0727904
1441262_at	---	---	0.86576	1.09570093	0.11467123
1436596_at	H2A histone family, member V	H2afv	0.86577	1.07386618	0.09403056
1416464_at	solute carrier family 4 (anion exchanger), member 1	Slc4a1	0.86577	1.06565711	0.08787444
1459031_at	tubulin tyrosine ligase-like family, member 4	Ttll4	0.86577	1.07356842	0.10053314
1440137_at	---	---	0.86579	1.08151858	0.10531224
1455575_at	eukaryotic translation initiation factor 4E binding protein 2	Eif4ebp2	0.86579	-1.06059638	-0.1051522
1432041_at	RIKEN cDNA 4930519A11 gene	4930519A11Rik	0.86579	1.07196257	0.09784253
1441993_at	adaptor-related protein complex 3, sigma 2 subunit	Ap3s2	0.86579	1.06377468	0.08712004
1440407_at	---	---	0.8658	1.0679957	0.09320337
1444983_at	---	---	0.8658	-1.06677877	-0.0941167
1444383_at	Ribosomal RNA processing 7 homolog A (S. cerevisiae)	Rrp7a	0.86581	1.07738474	0.10035452
1451799_at	coiled-coil domain containing 25	Ccdc25	0.86583	1.07070963	0.0956528
1447987_at	leucine zipper-EF-hand containing transmembrane protein 2	Letm2	0.86583	1.08934753	0.11201144
1453206_at	acyl-Coenzyme A dehydrogenase family, member 9	Acad9	0.86583	1.05104017	0.07128102
1446630_at	---	---	0.86584	1.1274542	0.13979066
1433825_at	neurotrophic tyrosine kinase, receptor, type 3	Ntrk3	0.86587	1.06792731	0.09312581
1445044_at	Prostaglandin I2 (prostacyclin) synthase	Ptgis	0.86587	1.09619056	0.11542637
1452598_at	GINS complex subunit 1 (Psf1 homolog)	Gins1	0.86588	1.04371568	0.06170758
1421475_at	membrane-spanning 4-domains, subfamily A, member 2	Ms4a2	0.86589	1.06424968	0.08948869
1439770_at	RIKEN cDNA 6430598A04 gene	6430598A04Rik	0.8659	-1.06593492	-0.0958661
1444626_at	hypothetical protein D930001B02	D930001B02	0.86592	-1.07416151	-0.1186971
1438011_at	phosphate cytidylyltransferase 1, choline, alpha isoform	Pcyt1a	0.86592	1.06433402	0.08573285
1420223_at	---	---	0.86592	-1.06527826	-0.0920749
1417794_at	zinc finger, MYM-type 3	Zmym3	0.86593	1.05452582	0.0757323
1454321_at	RIKEN cDNA 1500005C15 gene	1500005C15Rik	0.86594	-1.06166938	-0.087516
1428979_at	metal response element binding transcription factor 1	Mtf1	0.86595	-1.05788206	-0.0849526
1451753_at	plexin A2	Plxna2	0.86595	1.07352053	0.09817592
1437804_at	Insulin-like growth factor binding protein 7	Igfbp7	0.86596	-1.05735395	-0.0818051
1416695_at	translocator protein	Tspo	0.86597	1.05084376	0.07099383
1425795_a_at	mitogen-activated protein kinase kinase kinase 7	Map3k7	0.86597	-1.06044932	-0.1130642
1455297_at	spindlin family, member 2	Spin2	0.86597	-1.06937806	-0.1100163
1433992_at	shroom family member 2	Shroom2	0.86597	1.05087658	0.07049188
1441650_at	Rho GTPase activating protein 15	Arhgap15	0.86598	-1.05453645	-0.0778837
1452075_at	pseudouridylate synthase 10	Pus10	0.86598	1.05434979	0.07239289
1445104_at	RIKEN cDNA E230029C05 gene	E230029C05Rik	0.86598	1.06011621	0.08380362
1428334_at	osteopetrosis associated transmembrane protein 1	Ostm1	0.866	1.06010092	0.08085137
1422060_at	parvin, beta	Parvb	0.86601	-1.06159567	-0.1078917
1434471_at	cDNA sequence BC003331	BC003331	0.86601	1.0616407	0.08596956
1441611_at	zinc finger, DHHC domain containing 6	Zdhhc6	0.86602	-1.07300648	-0.1154651
1433714_at	sulfotransferase family 4A, member 1	Sult4a1	0.86602	1.04810833	0.06690138
1427914_a_at	transcription elongation factor B polypeptide 1-like /// transcriptio	LOC100045866 /// Tceb1	0.86602	-1.04359231	-0.0664233
1454373_x_at	hypothetical LOC100503298 /// hypothetical LOC100504128	LOC100503298 /// LOC10	0.86603	-1.05442554	-0.225855
1443774_at	zinc finger and AT hook domain containing	Zfat	0.86603	1.09015067	0.11114938
1418779_at	RCE1 homolog, prenyl protein peptidase (S. cerevisiae)	Rce1	0.86603	1.06096827	0.08097864
1428659_at	PHD finger protein 7	Phf7	0.86603	-1.05948018	-0.08829
1429862_at	phospholipase A2, group IVE	Pla2g4e	0.86603	1.10456324	0.12010217
1418686_at	2'-5' oligoadenylate synthetase 1C	Oas1c	0.86603	1.11759306	0.13320173
1458588_at	---	---	0.86603	1.07260685	0.09893914
1446656_at	---	---	0.86604	-1.06648881	-0.0989986
1451231_a_at	cullin 2	Cul2	0.86604	-1.04500166	-0.063849
1445672_at	---	---	0.86605	1.08175379	0.10552572
1456384_at	neuroligin 3	Nlgn3	0.86605	1.07482417	0.09900592
1425458_a_at	growth factor receptor bound protein 10	Grb10	0.86606	-1.04792997	-0.0719683
1424803_at	missing oocyte, meiosis regulator, homolog (Drosophila)	Mios	0.86606	1.07705675	0.09750444
1450378_at	TAP binding protein	Tapbp	0.86606	1.06394692	0.08793576
1455690_at	---	---	0.86606	-1.07443431	-0.118617

1438062_at	remodeling and spacing factor 1	Rsf1	0.86608	-1.05672195	-0.0830391
1452635_x_at	TATA box binding protein (Tbp)-associated factor, RNA polymeras	Taf1d	0.86608	-1.05283579	-0.0775199
1432339_at	RIKEN cDNA 4933432I03 gene	4933432I03Rik	0.8661	-1.07019543	-0.1082108
1423276_at	immunoglobulin-like domain containing receptor 1	Ildr1	0.86612	1.05333796	0.07496241
1422508_at	ATPase, H+ transporting, lysosomal V1 subunit A	Atp6v1a	0.86612	-1.04802986	-0.0712075
1418414_at	potassium voltage-gated channel, subfamily H (eag-related), mer	Kcnh1	0.86612	1.09958257	0.11908944
1428149_at	coronin 7	Coro7	0.86613	-1.05593364	-0.0819757
1451030_at	aldo-keto reductase family 1, member C21	Akr1c21	0.86613	-1.06696104	-0.0964634
1419454_x_at	protein inhibitor of activated STAT 2	Pias2	0.86615	-1.05435182	-0.0774106
1425972_a_at	zinc finger protein X-linked	Zfx	0.86615	1.05530219	0.07525558
1427320_at	coatomer protein complex, subunit gamma 2, antisense 2	Copg2as2	0.86615	-1.07128122	-0.1081988
1441092_at	RIKEN cDNA 9330159M07 gene	9330159M07Rik	0.86615	1.07333164	0.09834446
1450496_a_at	spindle and kinetochore associated complex subunit 1	Ska1	0.86616	-1.05334748	-0.0859196
1451784_x_at	histocompatibility 2, D region locus 1	H2-D1	0.86616	1.05074527	0.0708473
1437422_at	sema domain, seven thrombospondin repeats (type 1 and type 1-	Sema5a	0.86616	-1.05892438	-0.088086
1453204_at	RIKEN cDNA 4933431K14 gene	4933431K14Rik	0.86617	1.07489502	0.09997343
1456612_at	Interleukin 17D	Il17d	0.86617	1.14393454	0.14716862
1455985_x_at	serine hydroxymethyltransferase 2 (mitochondrial)	Shmt2	0.86617	1.05746216	0.07898193
1452321_at	bromodomain and WD repeat domain containing 1	Brwd1	0.86617	-1.06010676	-0.0897001
1416883_at	cleft lip and palate associated transmembrane protein 1	Clptm1	0.86617	1.04735819	0.0659252
1455685_at	microtubule associated monoxygenase, calponin and LIM domain	Mical2	0.86619	-1.07104	-0.1283415
1443204_at	---	---	0.8662	-1.07061405	-0.1059643
1455236_x_at	small EDRK-rich factor 2-like /// small EDRK-rich factor 2	LOC100503839 /// Serf2	0.86621	1.04480658	0.06065192
1459889_at	chloride channel calcium activated 3	Clca3	0.86621	-1.06068644	-0.0890143
1456838_at	leucine rich repeat and Ig domain containing 3	Lingo3	0.86621	1.06289389	0.08788074
1417787_at	dickkopf-like 1	Dkkl1	0.86621	1.07343215	0.09640231
1447427_at	Predicted gene 2717	Gm2717	0.86621	-1.06367253	-0.0948572
1441041_at	---	---	0.86621	-1.06443829	-0.0914989
1421099_at	basic helix-loop-helix family, member e41	Bhlhe41	0.86622	-1.07261134	-0.1222003
1418613_at	potassium inwardly-rectifying channel, subfamily J, member 1	Kcnj1	0.86622	1.09382446	0.11498544
1443491_at	---	---	0.86622	-1.07025324	-0.1068135
1445250_at	---	---	0.86622	-1.04724509	-0.0685224
1455387_at	nuclear fragile X mental retardation protein interacting protein 2	Nufip2	0.86623	-1.04575256	-0.0657832
1457008_at	cholinergic receptor, nicotinic, beta polypeptide 4	Chrnb4	0.86623	-1.07280292	-0.109396
1421611_at	hypothetical protein, MNCb-2457	AB041806	0.86623	-1.07073167	-0.1062596
1442654_at	RIKEN cDNA A530054K11 gene	A530054K11Rik	0.86623	1.05715532	0.07949194
1456007_at	ADP-ribosylation factor 3	Arf3	0.86624	-1.07121377	-0.1013911
1432395_at	RIKEN cDNA 1700039E15 gene	1700039E15Rik	0.86624	1.0914985	0.1147649
1457508_at	RIKEN cDNA C430003N24 gene	C430003N24Rik	0.86624	-1.06560178	-0.1010219
1427497_at	RIKEN cDNA 2610015P09 gene	2610015P09Rik	0.86624	-1.06744067	-0.107329
1450923_at	transforming growth factor, beta 2	Tgfb2	0.86624	-1.05611428	-0.0817833
1425006_a_at	vaccinia related kinase 1	Vrk1	0.86624	1.05067303	0.06857526
1422110_at	hairless	Hr	0.86625	-1.0633015	-0.0916603
1428335_a_at	Sec1 family domain containing 1	Scfd1	0.86625	-1.05165754	-0.0825198
1438960_at	---	---	0.86625	1.05903716	0.08175242
1441524_at	---	---	0.86625	1.06916568	0.09477712
1437863_at	butyrylcholinesterase	Bche	0.86625	1.06927741	0.09054891
1429901_at	Na+/K+ transporting ATPase interacting 2	Nkain2	0.86625	1.07731365	0.09413802
1428311_at	coiled-coil domain containing 6	Ccdc6	0.86626	1.05099601	0.06922566
1436584_at	sprouty homolog 2 (Drosophila)	Spry2	0.86626	-1.05578909	-0.0973182
1456381_x_at	myeloid cell leukemia sequence 1	Mcl1	0.86626	-1.05114207	-0.0792559
1459139_at	---	---	0.86626	-1.06781527	-0.0998884
1458310_at	RIKEN cDNA A230056J06 gene	A230056J06Rik	0.86627	-1.05625073	-0.0799294
1431464_a_at	phosphomannomutase 2	Pmm2	0.86627	-1.0699713	-0.1214119
1425920_at	CUE domain containing 1	Cuedc1	0.86627	-1.07176487	-0.1093051
1424029_at	TSPY-like 4	Tspyl4	0.86628	-1.05944748	-0.0926597
1439281_at	solute carrier family 26, member 8	Slc26a8	0.86628	-1.06786239	-0.0951971
1436858_at	muscleblind-like 2	Mbnl2	0.86628	1.06730204	0.09033454
1419256_at	spectrin beta 2	Spnb2	0.86628	-1.06171315	-0.0919943
1450114_at	kinase suppressor of ras 1	Ksr1	0.86629	-1.05996242	-0.0840501
1441528_at	tetratricopeptide repeat domain 15	Ttc15	0.86629	-1.06463642	-0.100134
1425630_at	transcriptional regulator, SIN3B (yeast)	Sin3b	0.86629	1.07260794	0.0973024
1423591_at	FGFR1 oncogene partner 2	Fgfr1op2	0.8663	-1.05162435	-0.0757284
1422332_at	interferon alpha 11 /// interferon alpha 2	Ifna11 /// Ifna2	0.8663	-1.06905705	-0.1093442
1449411_at	Down syndrome cell adhesion molecule	Dscam	0.8663	1.09626672	0.11736147
1436612_at	RIKEN cDNA 2410004I01 gene	2410004I01Rik	0.8663	1.07917626	0.10447874
1453886_a_at	solute carrier family 25 (mitochondrial carrier, phosphate carrier),	Slc25a26	0.8663	1.07072177	0.09373998
1426954_at	tripartite motif-containing 33	Trim33	0.8663	1.04752501	0.06557812
1435691_at	aminoglycoside phosphotransferase domain containing 1	Agphd1	0.86631	-1.06662384	-0.0974928
1439070_x_at	phosphatidylserine decarboxylase, pseudogene 1 /// phosphatidy	Pisd-ps1 /// Pisd-ps3	0.86631	-1.05765334	-0.097667
1434794_at	ras homolog gene family, member f	Rhof	0.86631	-1.05890481	-0.0836314
1421433_at	zinc finger homeodomain 4	Zfhx4	0.86632	1.06921897	0.09349899
1432012_a_at	NOL1/NOP2/Sun domain family member 6	Nsun6	0.86632	1.0694413	0.09417425
1451152_a_at	ATPase, Na+/K+ transporting, beta 1 polypeptide	Atp1b1	0.86633	1.05636158	0.07350589
1435498_at	ADP-ribosylation factor interacting protein 2	Arfip2	0.86634	1.06384648	0.08799584
1442651_at	---	---	0.86634	-1.06601339	-0.1008179
1427755_at	mast cell protease, pseudogene 1	Mcpt-ps1	0.86634	-1.06905107	-0.1022614
1440101_at	---	---	0.86635	1.06919818	0.09191798
1430011_at	RIKEN cDNA 2610317O13 gene	2610317O13Rik	0.86635	1.05692882	0.07975073
1434556_at	transmembrane protein 170B	Tmem170b	0.86635	-1.06198074	-0.0917833
1441066_at	---	---	0.86635	1.06535947	0.08751559
1417730_at	exostoses (multiple) 1	Ext1	0.86636	1.07538607	0.091279
1440207_at	lysophosphatidylcholine acyltransferase 4	Lpcat4	0.86636	1.06962734	0.08967711
1430680_a_at	RIKEN cDNA 5830417I10 gene /// gon-4-like (C.elegans)	5830417I10Rik /// Gon4l	0.86636	-1.05813834	-0.0846386
1442775_at	---	---	0.86637	1.09256987	0.11225946
1457268_at	DOT1-like, histone H3 methyltransferase (S. cerevisiae)	Dot1l	0.86637	1.05613828	0.07610175
1450355_a_at	capping protein (actin filament), gelsolin-like	Capg	0.86637	1.06458853	0.08518871
1429668_at	POU domain, class 4, transcription factor 1	Pou4f1	0.86638	-1.0651003	-0.0919085
1434803_a_at	syncollin	Sycn	0.86638	-1.06832497	-0.1032687
1442859_at	---	---	0.86638	1.09906797	0.11576928
1426321_at	T-box 4	Tbx4	0.86638	1.06615447	0.09240895
1423940_at	Yip1 interacting factor homolog A (S. cerevisiae)	Yif1a	0.86639	1.05661916	0.07539386
1435366_at	RIKEN cDNA D430042O09 gene	D430042O09Rik	0.86639	-1.07149025	-0.1190666
1419228_at	elaC homolog 1 (E. coli)	Elac1	0.86639	1.08584827	0.10574669

1419327_at	pyridoxal-dependent decarboxylase domain containing 1	Pdxdc1	0.8664	-1.07022578	-0.1079412
1451265_at	coiled-coil domain containing 115	Ccdc115	0.8664	1.05355138	0.07442582
1446314_at	glucosaminyl (N-acetyl) transferase family member 7	Gcnt7	0.86641	-1.06673284	-0.0937629
1454323_at	RIKEN cDNA 5830433D23 gene	5830433D23Rik	0.86641	-1.07123579	-0.1135239
1448097_at	---	---	0.86641	-1.05418161	-0.0851647
1458569_at	elongation factor RNA polymerase II 2	Ell2	0.86647	1.07125733	0.09421018
1445660_at	Transmembrane protein 209	Tmem209	0.86647	-1.07350757	-0.1153072
1444302_at	glucoside xylosyltransferase 2	Gxylt2	0.86647	-1.06683541	-0.0938753
1459732_at	---	---	0.86649	-1.07312356	-0.1092927
1452157_at	glutamyl-prolyl-tRNA synthetase	Eprs	0.86649	-1.04411556	-0.0625668
1443348_at	RIKEN cDNA D030028M11 gene	D030028M11Rik	0.86661	-1.06260946	-0.0876115
1433681_x_at	calpain 3	Capn3	0.86662	1.07290515	0.09314724
1428966_at	family with sequence similarity 103, member A1	Fam103a1	0.86664	-1.04385121	-0.0638489
1430012_at	RIKEN cDNA 1110050K14 gene	1110050K14Rik	0.86665	-1.0674097	-0.0941431
1432904_at	RIKEN cDNA 3110037C07 gene	3110037C07Rik	0.86667	-1.07187445	-0.1182098
1418078_at	proteaseome (prosome, macropain) 28 subunit, 3	Psme3	0.86667	-1.05608814	-0.1048939
1425896_a_at	fibrillin 1	Fbn1	0.86667	-1.06671541	-0.0955935
1456436_x_at	ribosomal protein S20	Rps20	0.86668	-1.0239089	-0.0354513
1456420_at	AT rich interactive domain 4A (RBP1-like)	Arid4a	0.8667	1.07140619	0.09624392
1432502_at	RIKEN cDNA 1700028N14 gene	1700028N14Rik	0.86671	1.09349155	0.11527545
1460195_at	mitochondrial ribosomal protein S11	Mrps11	0.86671	1.05144608	0.06970139
1422504_at	glycine receptor, beta subunit	Glrb	0.86671	1.05826936	0.08125562
1438370_x_at	downstream of Stk11	Dos	0.86671	1.08157161	0.10008938
1455041_at	oocyte maturation, beta	Omt2b	0.86671	1.06046322	0.08299641
1447219_at	---	---	0.86672	1.07231497	0.0984666
1460602_at	deleted in liver cancer 1	Dlc1	0.86673	-1.04903953	-0.0728062
1420332_x_at	late cornified envelope 1D	Lce1d	0.86674	1.0703406	0.09366606
1454826_at	zinc finger and BTB domain containing 11	Zbtb11	0.86675	-1.05334934	-0.0776983
1453780_at	hypothetical LOC100036525	LOC100036525	0.86675	1.0867148	0.10861945
1428929_s_at	solute carrier family 25 (mitochondrial carrier, phosphate carrier),	Slc25a26	0.86675	1.07875802	0.09993066
1446166_at	---	---	0.86676	-1.0669881	-0.0978724
1453586_at	ectonucleoside triphosphate diphosphohydrolase 1	Entpd1	0.86677	1.05794122	0.07895063
1426246_at	protein S (alpha)	Pros1	0.86678	-1.04722404	-0.0673435
1445151_at	Metal response element binding transcription factor 2	Mtf2	0.86678	-1.06462649	-0.0966215
1427398_at	mucin 4	Muc4	0.86679	1.08543216	0.10884607
1455873_a_at	vacuolar protein sorting 18 (yeast)	Vps18	0.8668	1.06906676	0.08989329
1429831_at	phosphoinositide-3-kinase adaptor protein 1	Pik3ap1	0.8668	1.12739379	0.13835919
1460458_at	cysteine-rich secretory protein LCCL domain containing 2	Crispld2	0.86681	-1.06894819	-0.1052092
1438513_at	TatD DNase domain containing 1	Tatdn1	0.86681	1.07647291	0.10205437
1425824_a_at	proprotein convertase subtilisin/kexin type 4	Pcsk4	0.86681	1.06216853	0.08602118
1433561_at	ArfGAP with coiled-coil, ankyrin repeat and PH domains 2	Acap2	0.86681	-1.04989248	-0.0722522
1440038_at	RIKEN cDNA B430007K19 gene /// hypothetical LOC100504050	B430007K19Rik /// LOC10	0.86681	1.06904288	0.09418028
1430875_a_at	PAK1 interacting protein 1	Pak1ip1	0.86682	1.04218837	0.05942206
1435815_at	leucine zipper, down-regulated in cancer 1	Ldoc1	0.86682	1.07705614	0.0998233
1454687_at	leucine rich repeat and fibronectin type III domain containing 5	Lrfn5	0.86683	1.07874909	0.09986801
1458285_at	Glutamate receptor, ionotropic, AMPA1 (alpha 1)	Gria1	0.86683	-1.06946006	-0.1104039
1448613_at	extracellular matrix protein 1	Ecm1	0.86683	1.06553926	0.08924851
1423100_at	FBJ osteosarcoma oncogene	Fos	0.86683	1.06560474	0.08914648
1443249_at	---	---	0.86684	1.07455398	0.10059816
1446110_at	DNA segment, Chr 3, ERATO Doi 711, expressed	D3Ertd711e	0.86685	-1.06693978	-0.0978133
1427911_at	transmembrane protein 173	Tmem173	0.86685	-1.05784719	-0.0832848
1444002_at	forkhead box L2	Foxl2	0.86685	-1.068854	-0.0989279
1444359_at	---	---	0.86685	1.09160215	0.11300775
1441253_at	---	---	0.86685	1.09242867	0.11067001
1458241_at	---	---	0.86686	1.0784264	0.10291566
1449351_s_at	platelet-derived growth factor, C polypeptide	Pdgfc	0.86687	-1.06449227	-0.1258145
1423078_a_at	sterol-C4-methyl oxidase-like	Sc4mol	0.86687	1.0466595	0.06572277
1416735_at	N-acylsphingosine amidohydrolase 1	Asah1	0.86687	-1.04805432	-0.0694577
1418324_at	feminization 1 homolog b (C. elegans)	Fem1b	0.86688	1.04840511	0.0660673
1429359_s_at	RNA binding protein gene with multiple splicing	Rbpms	0.86688	-1.05360966	-0.0776356
1419506_at	geranylgeranyl diphosphate synthase 1	Ggps1	0.86688	-1.06284091	-0.1078231
1442261_at	G protein-coupled receptor 150	Gpr150	0.86689	1.07038072	0.08965697
1442615_at	---	---	0.8669	-1.07090527	-0.1174593
1423679_at	RIKEN cDNA 2810432L12 gene	2810432L12Rik	0.8669	-1.06776339	-0.1023418
1455022_at	striatin, calmodulin binding protein	Strn	0.86691	-1.0563789	-0.0910181
1425412_at	NLR family, pyrin domain containing 3	Nlrp3	0.86691	-1.0656448	-0.0977193
1418873_at	sideroflexin 4	Sfxn4	0.86692	1.07214458	0.09699614
1453832_at	RIKEN cDNA 5330430P22 gene	5330430P22Rik	0.86692	1.06418972	0.08913605
1432788_at	RIKEN cDNA 6720420G18 gene	6720420G18Rik	0.86692	1.06281321	0.08368803
1453409_at	cell growth regulator with ring finger domain 1	Cgrrf1	0.86692	-1.06196652	-0.0897497
1458622_at	neurotrophic tyrosine kinase, receptor, type 2	Ntrk2	0.86693	-1.05851423	-0.0837207
1425853_s_at	prolactin receptor	Prlr	0.86693	-1.06317634	-0.0917475
1455443_at	ganglioside-induced differentiation-associated protein 1-like 1	Gdap1l1	0.86693	-1.07213598	-0.1099419
1445804_at	---	---	0.86693	-1.05188463	-0.0782521
1449866_at	synaptotagmin II	Syt2	0.86693	1.0684915	0.09419532
1444909_at	---	---	0.86693	1.06100662	0.08273398
1450576_a_at	splicing factor 3a, subunit 2	Sf3a2	0.86693	1.14141637	0.14098145
1441716_at	---	---	0.86694	-1.07515252	-0.1214444
1457511_at	regulator of G-protein signaling 12	Rgs12	0.86694	1.07429395	0.09879529
1453866_a_at	Kell blood group precursor (McLeod phenotype) homolog	Xk	0.86694	-1.07045046	-0.1022338
1440152_x_at	endothelial differentiation-related factor 1	Edf1	0.86695	1.06017555	0.07861248
1429194_at	tigger transposable element derived 2	Tigd2	0.86695	1.05338445	0.07442018
1418187_at	receptor (calcitonin) activity modifying protein 2	Ramp2	0.86695	1.08245389	0.10056408
1442736_at	Kelch-like 11 (Drosophila)	Klhl11	0.86696	1.07049241	0.09590248
1435801_at	fukutin	Fktn	0.86696	-1.05195178	-0.07457
1435695_a_at	gamma-glutamyl cyclotransferase	Ggct	0.86696	1.04923862	0.06839277
1424356_a_at	meteorin, glial cell differentiation regulator-like	Metrnl	0.86697	1.06782792	0.09413106
1446162_at	poly(A) binding protein, cytoplasmic 5	Pabpc5	0.86697	1.04707504	0.06480858
1439259_x_at	abhydrolase domain containing 4	Abhd4	0.86697	1.05765743	0.07517052
1434734_at	RAD54 homolog B (S. cerevisiae)	Rad54b	0.86697	1.06365362	0.08392299
1458254_at	proline-rich transmembrane protein 4	Prrt4	0.86698	-1.0608221	-0.0854854
1415991_a_at	kelch domain containing 3	Klhdc3	0.86698	1.0465249	0.06395553
1424043_at	peptidylprolyl isomerase (cyclophilin)-like 4	Ppil4	0.86698	1.05271408	0.07031595
1433924_at	paternally expressed 3	Peg3	0.86698	1.05177477	0.07192744

1452402_at	---	---	0.86698	1.11022382	0.12212653
1417885_at	microtubule-associated protein tau	Mapt	0.86699	1.07258746	0.09701223
1425510_at	MAP/microtubule affinity-regulating kinase 1	Mark1	0.86699	-1.067425	-0.1040958
1433840_a_at	RIKEN cDNA C330006K01 gene	C330006K01Rik	0.86699	1.05538791	0.07750294
1422480_at	sorting nexin 3	Snx3	0.86701	1.03914447	0.05483563
1433436_s_at	thiamine triphosphatase	Thtpa	0.86702	1.06185553	0.08354721
1429058_at	transmembrane protein 107	Tmem107	0.86703	1.05632369	0.07413013
1429518_at	Fas apoptotic inhibitory molecule 2	Faim2	0.86704	1.06771661	0.08900225
1418482_at	cytochrome b-561 domain containing 2	Cyb561d2	0.86704	1.06966072	0.08953498
1426079_at	CD247 antigen	Cd247	0.86707	1.0717634	0.09580957
1431053_at	M-phase phosphoprotein 9	Mphosph9	0.86711	-1.06070448	-0.086482
1432703_at	RIKEN cDNA 5730405A17 gene	5730405A17Rik	0.86712	-1.06593779	-0.0929489
1422473_at	phosphodiesterase 4B, cAMP specific	Pde4b	0.86713	-1.0748615	-0.1384365
1431253_s_at	TBC1 domain family, member 9	Tbc1d9	0.86716	1.07520114	0.10206596
1457513_at	family with sequence similarity 40, member A	Fam40a	0.86716	1.06697962	0.09296136
1417374_at	tubulin, alpha 4A	Tuba4a	0.8672	1.04608062	0.0637692
1460076_x_at	zinc finger protein 3	Zfp3	0.86721	1.07087815	0.09640848
1421924_at	solute carrier family 2 (facilitated glucose transporter), member 3	Slc2a3	0.86722	-1.05314203	-0.0843394
1425874_at	homeobox C13	Hoxc13	0.86723	-1.06482633	-0.0975014
1430777_a_at	golgi phosphoprotein 3	Golph3	0.86724	-1.05421841	-0.0842703
1431310_s_at	tubulin tyrosine ligase-like family, member 10	Ttll10	0.86725	1.05909041	0.07824021
1437562_at	RIKEN cDNA 4932422M17 gene	4932422M17Rik	0.86725	-1.06880747	-0.1030667
1433775_at	expressed sequence C77080	C77080	0.86726	1.0487291	0.06799303
1425391_a_at	oxysterol binding protein-like 5	Osbpl5	0.86727	-1.06422646	-0.0903467
1417965_at	pleckstrin homology domain containing, family A (phosphoinositi	Plekha1	0.86727	-1.05234172	-0.0779944
1457502_at	---	---	0.86731	-1.06504336	-0.0911336
1450705_at	RD RNA-binding protein	Rdbp	0.86734	1.05130154	0.06981142
1437448_s_at	catenin (cadherin associated protein), delta 1	Ctnnd1	0.86734	1.06605509	0.0826366
1423215_at	signal peptidase complex subunit 2 homolog (S. cerevisiae)	Spcs2	0.86735	1.05154471	0.06949495
1446966_at	Expressed sequence AU042651	AU042651	0.86736	-1.0703216	-0.1113612
1454244_at	minichromosome maintenance deficient 10 (S. cerevisiae)	Mcm10	0.86737	1.0805366	0.09982451
1419104_at	abhydrolase domain containing 6	Abhd6	0.86737	1.06901562	0.09301438
1445080_at	---	---	0.86738	-1.05859764	-0.0876687
1449243_a_at	ribosomal protein S19	Rps19	0.86739	1.0287631	0.03982857
1435600_s_at	cDNA sequence BC020535	BC020535	0.86739	1.06700164	0.09288156
1451025_at	ADP-ribosylation factor-like 1	Arl1	0.86739	-1.04368924	-0.0617431
1434901_at	zinc finger and BTB domain containing 2	Zbtb2	0.86741	-1.04959099	-0.0702916
1446500_at	---	---	0.86743	1.06137697	0.08393778
1431078_at	F-box protein 3	Fbxo3	0.86744	-1.07270946	-0.1418778
1435963_at	sema domain, seven thrombospondin repeats (type 1 and type 1-	Sema5b	0.86745	1.08929533	0.1071379
1424883_s_at	serine/arginine-rich splicing factor 7	Srsf7	0.86749	-1.03817634	-0.0549187
1447298_at	---	---	0.86753	1.09456964	0.11118915
1455065_x_at	glucosamine-6-phosphate deaminase 1 pseudogene /// glucosar	Gm8615 /// Gnpda1	0.86753	-1.04461969	-0.0636694
1446813_s_at	DNA segment, Chr 5, ERATO Doi 560, expressed	D5Ertd560e	0.86754	-1.06746921	-0.0961023
1429525_s_at	myosin IF	Myo1f	0.86755	-1.04558543	-0.0661956
1444771_at	ankyrin repeat and BTB (POZ) domain containing 2	Abtb2	0.86756	1.0571135	0.07753656
1420115_at	exosome component 8	Exosc8	0.86756	-1.06102388	-0.0966485
1444781_at	GPN-loop GTPase 1	Gpn1	0.86757	-1.05916685	-0.0844741
1429066_at	RIKEN cDNA 4930565B19 gene	4930565B19Rik	0.86758	1.0681115	0.08941266
1446290_at	mitotic spindle organizing protein 1	Mzt1	0.86758	-1.06557036	-0.0934008
1438038_at	RIKEN cDNA 4930402H24 gene	4930402H24Rik	0.86758	1.09028537	0.11077101
1460245_at	killer cell lectin-like receptor, subfamily D, member 1	Klrd1	0.86758	1.06150635	0.0842673
1458596_at	dynactin 4	Dctn4	0.86759	1.07386305	0.0993804
1424983_a_at	RIKEN cDNA 2700078E11 gene	2700078E11Rik	0.8676	1.05505978	0.07495388
1450526_at	T-cell leukemia, homeobox 1	Tlx1	0.8676	1.08004021	0.10302936
1423166_at	CD36 antigen	Cd36	0.8676	-1.05605518	-0.0800249
1439581_at	RIKEN cDNA 6330403L08 gene	6330403L08Rik	0.86761	1.0827151	0.10604617
1451701_x_at	claudin 3	Cldn3	0.86761	1.0750689	0.09663419
1455160_at	RIKEN cDNA 2610203C20 gene	2610203C20Rik	0.86762	-1.06403828	-0.1024345
1448811_at	mitochondrial ribosomal protein L2	Mrpl2	0.86762	1.0454336	0.06369775
1435001_at	phospholipase A2, activating protein	Plaa	0.86763	-1.04932455	-0.0722572
1424304_at	two pore segment channel 2	Tpcn2	0.86763	1.07633035	0.1001579
1451248_at	protein arginine N-methyltransferase 7	Prmt7	0.86763	1.0511048	0.07067957
1452614_at	BCLl2-like 15	Bcl2l15	0.86763	1.09707619	0.11467024
1450763_x_at	wingless-related MMTV integration site 3	Wnt3	0.86763	1.06953102	0.0906435
1449936_at	RIKEN cDNA 8430419L09 gene	8430419L09Rik	0.86764	1.06319054	0.0871418
1436930_x_at	predicted gene 6453 /// hydroxymethylbilane synthase	Gm6453 /// Hmbs	0.86764	-1.04857952	-0.0725915
1429338_a_at	nucleolar protein 9	Nol9	0.86764	-1.05421892	-0.0817907
1449980_a_at	gamma-aminobutyric acid (GABA) A receptor, subunit delta	Gabrd	0.86765	-1.06530389	-0.0947297
1426631_at	pseudouridylate synthase 7 homolog (S. cerevisiae)	Pus7	0.86765	-1.04953328	-0.0750372
1440233_at	RIKEN cDNA A930013B10 gene	A930013B10Rik	0.86766	-1.06811987	-0.0953263
1451933_a_at	cathepsin 7	Cts7	0.86767	1.07239059	0.09367549
1456338_at	solute carrier family 38, member 9	Slc38a9	0.86768	1.06071789	0.08344432
1443582_at	growth arrest-specific 2 like 2	Gas2l2	0.86768	1.06554852	0.09019703
1427666_a_at	T-cell receptor beta, joining region	Tcrb-J	0.86768	1.08063342	0.10424166
1458112_at	adenosine deaminase, RNA-specific, B2	Adarb2	0.86769	1.06597049	0.09209492
1454571_at	RIKEN cDNA A930036A04 gene	A930036A04Rik	0.86769	1.06273346	0.08765723
1416351_at	mitogen-activated protein kinase kinase 1	Map2k1	0.86769	1.05318177	0.07123972
1446224_at	HECT domain containing 2	Hectd2	0.86769	1.07894638	0.09898881
1423571_at	sphingosine-1-phosphate receptor 1	S1pr1	0.8677	1.08663669	0.10784723
1437400_at	neural precursor cell expressed, developmentally down-regulated	Nedd4l	0.8677	-1.06464774	-0.0974717
1446331_at	prostaglandin F receptor	Ptgfr	0.8677	-1.06288456	-0.0900975
1447104_at	---	---	0.86771	-1.06438391	-0.0925508
1443470_at	hypothetical LOC100303644	LOC100303644	0.86771	-1.0687778	-0.0969096
1459532_at	glutamate receptor, metabotropic 7	Grm7	0.86771	-1.06136199	-0.089593
1440298_at	triggering receptor expressed on myeloid cells-like 2	Treml2	0.86771	-1.06327683	-0.0926863
1424487_x_at	thioredoxin reductase 1	Txnrd1	0.86771	-1.05503764	-0.0793683
1423041_a_at	basic leucine zipper and W2 domains 1	Bzw1	0.86771	1.05636352	0.07250996
1421818_at	B-cell leukemia/lymphoma 6	Bcl6	0.86772	1.06884681	0.09494642
1418451_at	guanine nucleotide binding protein (G protein), gamma 2	Gng2	0.86772	-1.04762233	-0.0696974
1420420_at	hydroxyacid oxidase 1, liver	Hao1	0.86772	1.06691315	0.08598044
1448623_at	transmembrane protein 123	Tmem123	0.86772	1.05026128	0.06922114
1438576_x_at	---	---	0.86772	-1.06284209	-0.0956875
1446236_at	RIKEN cDNA C230072F16 gene	C230072F16Rik	0.86772	-1.06818137	-0.1006662

1441954_s_at	H1 histone family, member N, testis-specific	H1fnt	0.86772	1.06637678	0.08994618
1420994_at	UDP-GlcNAc:betaGal beta-1,3-N-acetylglucosaminyltransferase 5	B3gnt5	0.86772	-1.04948417	-0.0731011
1446196_at	high mobility group AT-hook 2	Hmga2	0.86772	1.08406781	0.10648935
1428211_at	prolyl 4-hydroxylase, transmembrane (endoplasmic reticulum)	P4htm	0.86773	1.06196337	0.08573736
1460368_at	membrane protein, palmitoylated 4 (MAGUK p55 subfamily mem	Mpp4	0.86773	-1.06746268	-0.1082534
1441425_at	---	---	0.86773	-1.0685856	-0.1079096
1446345_at	---	---	0.86774	1.06794923	0.0920163
1431586_at	RIKEN cDNA 4921529N20 gene	4921529N20Rik	0.86774	-1.07144869	-0.1252356
1458617_at	zinc finger, MYND-type containing 8	Zmynd8	0.86774	-1.06818689	-0.1012709
1422427_a_at	semenogelin I	Semg1	0.86774	-1.05517469	-0.0803575
1455182_at	kinesin family member 1B	Kif1b	0.86775	1.05978648	0.07926212
1424824_at	SLAIN motif family, member 1	Slain1	0.86775	-1.05710505	-0.0851548
1417578_a_at	GDP-mannose pyrophosphorylase A	Gmppa	0.86775	1.05211783	0.07285156
1431500_at	RIKEN cDNA 4933406J10 gene	4933406J10Rik	0.86775	-1.07186142	-0.1179393
1436367_at	protein tyrosine phosphatase, receptor type, B	Ptprb	0.86775	1.07283452	0.09698284
1433340_at	RIKEN cDNA 4930480K02 gene	4930480K02Rik	0.86775	1.08273659	0.10486807
1422195_s_at	T-box 15	Tbx15	0.86775	-1.06546603	-0.0940433
1430596_s_at	vestigial like 3 (Drosophila)	Vgll3	0.86776	-1.06922554	-0.1071045
1456913_at	---	---	0.86776	1.09431209	0.11493956
1426387_x_at	RIKEN cDNA C330019G07 gene /// phosphatidylserine decarboxy	C330019G07Rik /// Pisd /	0.86776	-1.04279087	-0.0607315
1459123_at	---	---	0.86776	-1.06203108	-0.093647
1431934_at	RIKEN cDNA 4930505O20 gene	4930505O20Rik	0.86777	-1.06634432	-0.0997884
1447171_at	---	---	0.86777	-1.06609042	-0.1075213
1439558_at	zinc finger protein 317	Zfp317	0.86777	-1.0713474	-0.1338672
1416984_at	mitochondrial ribosomal protein S18A	Mrps18a	0.86777	1.06134389	0.07852364
1433638_s_at	homeobox D8	Hoxd8	0.86777	-1.06968279	-0.1231556
1452861_at	RIKEN cDNA 2010300C02 gene	2010300C02Rik	0.86777	1.09472593	0.11443154
1428048_at	hypothetical LOC269472	LOC269472	0.86777	-1.0697649	-0.1009316
1432778_at	RIKEN cDNA 1700048O20 gene	1700048O20Rik	0.86777	1.21365228	0.18895834
1456984_at	sex comb on midleg-like 2 (Drosophila)	Scml2	0.86777	1.07059347	0.09667774
1446379_at	---	---	0.86778	1.06055888	0.08439496
1442772_at	---	---	0.86778	-1.06406625	-0.0904097
1449789_x_at	lymphocyte antigen 6 complex, locus G6C	Ly6g6c	0.86778	1.0665072	0.08939159
1424782_at	VDNA-damage regulated autophagy modulator 2	Dram2	0.86778	1.06781821	0.0897792
1441506_at	decorin	Dcn	0.86778	-1.06798815	-0.1169391
1437021_at	ADP-ribosylation factor-like 13B	Arl13b	0.86778	1.06105104	0.08163449
1426299_at	sorting nexin 20	Snx20	0.86778	1.05717967	0.07911058
1427162_a_at	ELK4, member of ETS oncogene family	Elk4	0.86778	1.07026958	0.09383037
1430711_at	RIKEN cDNA 4933416E03 gene	4933416E03Rik	0.86779	1.10401026	0.12036326
1429580_x_at	spermatogenesis associated 24	Spata24	0.86779	-1.06280133	-0.0941805
1431228_s_at	RIKEN cDNA 4930526I15 gene	4930526I15Rik	0.86779	1.06360904	0.08676996
1429373_x_at	CREB regulated transcription coactivator 2	Crtc2	0.86779	1.09917577	0.1140132
1436679_at	tectonin beta-propeller repeat containing 1	Tecpr1	0.86779	-1.06003696	-0.0852876
1449621_s_at	thrombospondin, type I, domain 1	Thsd1	0.86779	-1.06447384	-0.0995993
1421318_at	N-deacetylase/N-sulfotransferase (heparin glucosaminyl) 4	Ndst4	0.86779	1.07949811	0.10096869
1452117_a_at	FYN binding protein	Fyb	0.86779	1.13213262	0.13792797
1437549_at	RIKEN cDNA 2810408I11 gene	2810408I11Rik	0.86779	1.05599959	0.07763469
1424197_s_at	Fanconi anemia, complementation group E	Fance	0.8678	1.05486665	0.07549861
1422299_a_at	serine/threonine/tyrosine interacting-like 1	Styxl1	0.8678	1.0645197	0.08914884
1457522_at	SMEK homolog 1, suppressor of mek1 (Dictyostelium)	Smek1	0.8678	-1.06627114	-0.0948641
1416460_at	myo-inositol oxygenase	Miox	0.8678	-1.06940815	-0.0989881
1422568_at	nuclear distribution gene E-like homolog 1 (A. nidulans)	Ndel1	0.8678	1.04418652	0.06203907
1454737_at	dual specificity phosphatase 9	Dusp9	0.8678	1.07064372	0.08562279
1444838_at	predicted gene 9946	Gm9946	0.86781	1.06989139	0.09592222
1432271_a_at	DCN1, defective in cullin neddylation 1, domain containing 5 (S. ce	Dcun1d5	0.86781	-1.04631158	-0.0666855
1450087_a_at	nucleolar and coiled-body phosphoprotein 1	Nolc1	0.86781	1.0477757	0.0659431
1447386_at	---	---	0.86781	1.06514093	0.08988718
1430215_at	RIKEN cDNA 2610020H08 gene	2610020H08Rik	0.86781	1.06709921	0.09180577
1422829_at	dopamine receptor D4	Drd4	0.86781	1.07819985	0.10102917
1429208_at	RIKEN cDNA 5730408K05 gene	5730408K05Rik	0.86782	1.06511133	0.09017902
1437631_at	Kv channel interacting protein 4	Kcnip4	0.86782	1.05600127	0.07660183
1449982_at	interleukin 11	Il11	0.86782	1.09061847	0.11000496
1432688_at	RIKEN cDNA 4631422I05 gene	4631422I05Rik	0.86782	1.06745205	0.09300888
1448697_s_at	ribosomal protein L36A-like	Rpl36al	0.86782	1.03038593	0.04212522
1446659_at	hypothetical LOC100503230	LOC100503230	0.86782	-1.06361491	-0.0980855
1439379_x_at	protamine 1	Prm1	0.86782	1.06569628	0.08497886
1458960_at	---	---	0.86782	-1.07066578	-0.1074416
1446287_at	protease, serine, 41	Prss41	0.86782	1.06054452	0.08196504
1446032_at	---	---	0.86782	-1.05802824	-0.0814344
1429682_at	family with sequence similarity 46, member C	Fam46c	0.86782	1.08020258	0.1053294
1444317_at	protocadherin 15	Pcdh15	0.86782	1.07160042	0.08964775
1446492_at	transmembrane protein 132D	Tmem132d	0.86783	-1.06500631	-0.0924488
1458418_at	---	---	0.86783	1.07290221	0.09760443
1418270_at	a disintegrin-like and metallopeptidase (reprolysin type) with thro	Adamts8	0.86783	1.0694388	0.09302675
1448338_at	plasma glutamate carboxypeptidase	Pgcp	0.86783	-1.05577665	-0.0918382
1418397_at	zinc finger protein 275	Zfp275	0.86783	1.06279857	0.08432167
1425083_at	otoraplin	Otor	0.86783	-1.07109387	-0.1311754
1426064_at	cytochrome P450, family 3, subfamily a, polypeptide 44	Cyp3a44	0.86783	1.10139896	0.11525682
1424058_at	proline-rich coiled-coil 1	Prrc1	0.86783	1.05543717	0.07461525
1430511_at	unc-13 homolog C (C. elegans)	Unc13c	0.86783	1.07270269	0.09579947
1449284_at	RIKEN cDNA 1700008P20 gene	1700008P20Rik	0.86783	1.07414335	0.09722724
1454578_at	RIKEN cDNA 6030458A19 gene	6030458A19Rik	0.86784	-1.05689022	-0.0832522
1417732_at	annexin A8	Anxa8	0.86784	-1.06639578	-0.1001258
1423064_at	DNA methyltransferase 3A	Dnmt3a	0.86784	-1.04900366	-0.0745957
1445315_at	---	---	0.86784	-1.06656346	-0.0976445
1416121_at	lysyl oxidase	Lox	0.86784	-1.06827683	-0.098129
1427591_at	chloride channel 1	Clcn1	0.86784	-1.07057964	-0.1226606
1440504_at	coiled-coil domain containing 39	Ccdc39	0.86784	-1.0676095	-0.0947556
1423183_at	leucine-rich repeat LGI family, member 1	Lgi1	0.86784	1.06908475	0.09316895
1419373_at	ATPase, H+ transporting, lysosomal V1 subunit B1	Atp6v1b1	0.86784	-1.06988848	-0.109722
1419480_at	selectin, lymphocyte	Sell	0.86784	-1.07135934	-0.123532
1420769_at	DEXH (Asp-Glu-X-His) box polypeptide 58	Dhx58	0.86785	1.07961776	0.10168053
1436907_at	neuron navigator 1	Nav1	0.86785	-1.05336349	-0.0771534
1448509_at	family with sequence similarity 107, member B	Fam107b	0.86785	1.073509	0.09720626

1437817_at	synaptosomal-associated protein 91	Snap91	0.86785	-1.06772205	-0.0981894
1417590_at	cytochrome P450, family 27, subfamily a, polypeptide 1	Cyp27a1	0.86785	-1.06607729	-0.1018924
1451562_at	AIG2-like domain 1	A2ld1	0.86785	-1.06269056	-0.0911249
1450796_at	atonal homolog 7 (Drosophila)	Atoh7	0.86785	1.07353807	0.09746152
1419483_at	complement component 3a receptor 1	C3ar1	0.86785	-1.06812908	-0.10086
1439452_x_at	aspartyl aminopeptidase	Dnpep	0.86785	-1.04564174	-0.0663412
1459896_at	pogo transposable element with KRAB domain	Pogk	0.86785	-1.06507619	-0.0931221
1451747_a_at	autophagy-related 12 (yeast)	Atg12	0.86786	1.04898971	0.06854226
1428511_at	phosphorylase kinase, gamma 2 (testis)	Phkg2	0.86786	1.06443833	0.08403367
1428467_at	TAR DNA binding protein	Tardbp	0.86786	1.04937627	0.06931423
1426792_s_at	RUN and SH3 domain containing 2	Rusc2	0.86786	1.05147619	0.07122142
1438710_at	5-hydroxytryptamine (serotonin) receptor 1A	Htr1a	0.86786	1.06409411	0.08570253
1452005_at	dihydrolipoamide S-acetyltransferase (E2 component of pyruvate	Dlat	0.86786	1.05790325	0.07885137
1449989_at	mast cell protease 2	Mcpt2	0.86787	-1.06540516	-0.0921428
1447314_at	---	---	0.86787	-1.06644328	-0.1123539
1425604_at	v-crk sarcoma virus CT10 oncogene homolog (avian)-like	Crkl	0.86787	-1.0571568	-0.0810068
1430953_at	RIKEN cDNA 3200001G23 gene	3200001G23Rik	0.86787	1.04828606	0.06762329
1445966_at	hypothetical LOC100502594	LOC100502594	0.86787	1.06983796	0.09510707
1424412_at	opioid growth factor receptor-like 1	Ogfrl1	0.86787	1.07402919	0.09328538
1445498_at	---	---	0.86788	-1.06907531	-0.1004045
1453155_at	transmembrane protein 50A	Tmem50a	0.86788	-1.04657866	-0.0664719
1438557_x_at	aspartyl aminopeptidase	Dnpep	0.86788	-1.04391036	-0.0623814
1431978_at	RIKEN cDNA 8430437L04 gene	8430437L04Rik	0.86788	-1.06876829	-0.0981107
1422635_at	acetylcholinesterase	Ache	0.86788	1.07156334	0.09464935
1452732_at	aspartic peptidase, retroviral-like 1	Asprv1	0.86789	-1.0565755	-0.0808676
1445348_at	cell division cycle 40 homolog (yeast)	Cdc40	0.86789	-1.06326343	-0.0896809
1455683_a_at	TBC1 domain family, member 8	Tbc1d8	0.86789	-1.06655347	-0.0948097
1446180_at	laminin B1	Lamb1	0.86789	-1.07348915	-0.125531
1431604_a_at	coiled-coil domain containing 97	Ccdc97	0.8679	-1.06470665	-0.1069784
1443455_at	---	---	0.8679	1.06887723	0.09204957
1459699_at	---	---	0.8679	-1.06215987	-0.0871935
1439904_at	follistatin-like 5	Fstl5	0.8679	-1.05927603	-0.0857051
1437169_at	PDZ and LIM domain 1 (elfin)	Pdlim1	0.86791	1.09966849	0.11848355
1439942_at	prolyl endopeptidase	Prep	0.86791	-1.05973084	-0.0844222
1442232_at	RIKEN cDNA C530025M09 gene	C530025M09Rik	0.86791	1.14714196	0.14883897
1426106_a_at	synaptotagmin VI	Syt6	0.86791	1.08616782	0.10730185
1445438_at	DDHD domain containing 1	Ddhd1	0.86792	-1.06746042	-0.0975745
1460496_at	RIKEN cDNA 5430413K10 gene	5430413K10Rik	0.86792	-1.06581386	-0.0921157
1459674_at	---	---	0.86792	1.06622402	0.08793758
1442498_at	EST C78662	C78662	0.86792	-1.0731142	-0.121527
1434299_x_at	intraflagellar transport 27 homolog (Chlamydomonas)	Ift27	0.86792	1.0657375	0.0840214
1440747_at	coproporphyrinogen oxidase	Cpox	0.86792	1.06820633	0.09516988
1424603_at	sulfatase modifying factor 1	Sumf1	0.86793	1.06717288	0.088938
1449862_a_at	phosphatidylinositol 4-kinase type 2 beta	Pi4k2b	0.86793	-1.06382776	-0.1121255
1436450_at	DNA segment, Chr 11, Brigham & Women's Genetics 0517 expres	D11Bwg0517e	0.86793	-1.06658193	-0.1007225
1442742_at	ATPase, Ca++-sequestering	Atp2c1	0.86793	-1.07153602	-0.112464
1432394_a_at	apoptosis antagonizing transcription factor	Aatf	0.86793	-1.05167889	-0.0762123
1417265_s_at	coenzyme Q5 homolog, methyltransferase (yeast)	Coq5	0.86793	1.0579881	0.07831526
1443200_at	predicted gene 12541	Gm12541	0.86793	1.0787653	0.09936275
1422774_at	RIKEN cDNA 1700088E04 gene	1700088E04Rik	0.86794	1.06626269	0.089289
1418424_at	tumor necrosis factor alpha induced protein 6	Tnfaip6	0.86794	-1.05924725	-0.0894796
1429017_at	Smith-Magenis syndrome chromosome region, candidate 8 homo	Smcr8	0.86795	1.0651391	0.08596665
1453346_at	RIKEN cDNA 1700011L22 gene	1700011L22Rik	0.86795	-1.06622524	-0.1018175
1437037_x_at	staphylococcal nuclease and tudor domain containing 1	Snd1	0.86795	-1.06592806	-0.0987183
1423086_at	Niemann Pick type C1	Npc1	0.86796	1.08729637	0.10518212
1433827_at	ATPase, aminophospholipid transporter (APLT), class I, type 8A, m	Atp8a1	0.86796	1.06605547	0.09130113
1423953_at	CDK5 regulatory subunit associated protein 1-like 1	Cdkal1	0.86796	-1.05243038	-0.0745917
1455131_at	optic atrophy 3 (human)	Opa3	0.86796	1.05424683	0.07367155
1442730_at	Ariadne ubiquitin-conjugating enzyme E2 binding protein homolog	Arih1	0.86796	1.07070435	0.09679131
1427170_at	proteasome (prosome, macropain) subunit, alpha type, 8	Psma8	0.86797	-1.05144714	-0.0742844
1430579_at	TRAF2 and NCK interacting kinase	Tnik	0.86797	1.07562176	0.09974486
1443847_x_at	AF4/FMR2 family, member 2	Aff2	0.86797	1.05642669	0.07793218
1437546_at	DnaJ (Hsp40) homolog, subfamily C, member 14	Dnajc14	0.86797	-1.05189004	-0.0755936
1435047_at	RAB3C, member RAS oncogene family	Rab3c	0.86798	1.08141251	0.10487353
1419823_s_at	kinase suppressor of ras 1	Ksr1	0.86798	1.07676907	0.09647688
1435345_at	cerebral endothelial cell adhesion molecule	Cercam	0.86799	1.0663321	0.08768923
1442856_at	adrenomedullin 2	Adm2	0.868	-1.06308193	-0.1218754
1435239_at	glutamate receptor, ionotropic, AMPA1 (alpha 1)	Gria1	0.868	-1.05623313	-0.0850865
1435191_at	corneodesmosin	Cdsn	0.86801	1.05883167	0.07977361
1443960_at	asparaginase like 1	Asrgl1	0.86803	-1.06946052	-0.1116474
1425550_a_at	protein kinase, cAMP dependent regulatory, type I, alpha	Prkar1a	0.86803	1.04681826	0.06505201
1429549_at	collagen, type XXVII, alpha 1	Col27a1	0.86804	-1.06821095	-0.0994131
1420847_a_at	fibroblast growth factor receptor 2	Fgfr2	0.86804	1.07416359	0.09739483
1450554_at	defensin beta 2	Defb2	0.86804	1.07515042	0.09912045
1449593_at	---	---	0.86805	-1.05822375	-0.0832371
1437427_at	RIKEN cDNA 4933405O20 gene	4933405O20Rik	0.86805	-1.06932256	-0.1010246
1432942_at	RIKEN cDNA 5730408A14 gene	5730408A14Rik	0.86805	1.0643523	0.08841671
1438257_at	---	---	0.86805	1.09687552	0.11445545
1415842_at	MTOR associated protein, LST8 homolog (S. cerevisiae)	Mlst8	0.86805	1.05765023	0.07884325
1432299_at	RIKEN cDNA 4921513I08 gene	4921513I08Rik	0.86805	1.06997126	0.09392532
1441997_at	zinc finger protein 184 (Kruppel-like)	Zfp184	0.86805	1.06415106	0.08686525
1432288_at	RIKEN cDNA 5830468F06 gene	5830468F06Rik	0.86806	1.07777221	0.10240276
1434529_x_at	checkpoint with forkhead and ring finger domains	Chfr	0.86806	-1.05182143	-0.0758929
1437762_at	RAB39, member RAS oncogene family	Rab39	0.86807	1.08155421	0.10255511
1444554_at	---	---	0.86807	1.11500678	0.12683784
1421878_at	mitogen-activated protein kinase 9	Mapk9	0.86807	-1.05580751	-0.079621
1456177_x_at	zinc finger protein 706	Zfp706	0.86807	1.04638177	0.06395203
1434295_at	RAS guanyl releasing protein 1	Rasgrp1	0.86807	1.09516158	0.11402574
1437205_at	transcription factor 25 (basic helix-loop-helix)	Tcf25	0.86807	-1.05631354	-0.0828876
1420543_at	DnaJ (Hsp40) homolog, subfamily C, member 28	Dnajc28	0.86807	1.08248283	0.10489559
1446015_at	---	---	0.86807	-1.06481686	-0.09072
1458991_at	---	---	0.86807	1.07235534	0.0964101
1442549_at	muscleblind-like 3 (Drosophila)	Mbnl3	0.86808	-1.07184798	-0.119842
1443713_at	cell adhesion molecule with homology to L1CAM	Chl1	0.86808	1.05257629	0.07294836

1434044_at	replication initiator 1	Repin1	0.86809	1.06743908	0.08764868
1454597_at	RIKEN cDNA 4930423O20 gene	4930423O20Rik	0.86809	1.05558826	0.0747758
1442390_at	---	---	0.86809	-1.06912933	-0.1003124
1449753_at	Myosin IE	Myo1e	0.86809	-1.05103731	-0.0718765
1444319_at	E2F transcription factor 8	E2f8	0.8681	-1.06286838	-0.0984169
1449550_at	myosin IC	Myo1c	0.8681	1.08387927	0.10552587
1445450_x_at	Riken cDNA A530021J07 gene	A530021J07Rik	0.8681	1.04176365	0.05825768
1448852_at	regucalcin	Rgn	0.8681	1.10654305	0.12283153
1452468_s_at	methylmalonic aciduria (cobalamin deficiency) type B homolog (h	Mmab	0.86811	-1.05605705	-0.0836299
1431354_a_at	phenylalanine-tRNA synthetase 2 (mitochondrial)	Fars2	0.86811	1.0534121	0.07328259
1445093_at	---	---	0.86811	1.07403582	0.09620648
1434965_at	DNA segment, Chr 19, ERATO Doi 386, expressed	D19Ertd386e	0.86811	1.05821358	0.08031296
1445351_at	---	---	0.86811	1.07547169	0.09788148
1452430_s_at	serine/arginine-rich splicing factor 1	Srsf1	0.86812	-1.04827889	-0.0760232
1454786_at	RIKEN cDNA 5031439G07 gene	5031439G07Rik	0.86812	1.05020275	0.06846496
1453657_at	RIKEN cDNA 2310065F04 gene	2310065F04Rik	0.86812	1.103398	0.12069856
1444946_at	---	---	0.86812	-1.06686401	-0.0991451
1453957_a_at	insulin-like growth factor 2 mRNA binding protein 3	Igf2bp3	0.86812	-1.05551353	-0.0786051
1438493_at	phospholipase D family, member 6	Pld6	0.86813	1.07005482	0.0920243
1446682_at	---	---	0.86813	-1.0715742	-0.1179881
1456717_at	---	---	0.86813	-1.07116937	-0.1153082
1451537_at	chitinase 3-like 1	Chi3l1	0.86813	1.09976879	0.1188035
1459649_at	---	---	0.86813	1.09232212	0.10926393
1431099_at	homeobox D8	Hoxd8	0.86813	1.06364506	0.08872752
1426228_at	v-Ki-ras2 Kirsten rat sarcoma viral oncogene homolog	Kras	0.86814	-1.05369044	-0.0761471
1459879_at	RIKEN cDNA 4921513D23 gene	4921513D23Rik	0.86814	-1.06700013	-0.1463559
1429055_at	RIKEN cDNA 4930506M07 gene	4930506M07Rik	0.86814	-1.06395736	-0.0919581
1417255_at	intraflagellar transport 140 homolog (Chlamydomonas)	Ift140	0.86814	1.05393357	0.07406739
1444048_at	---	---	0.86815	-1.06816805	-0.1024467
1447709_at	RIKEN cDNA 2410021H03 gene	2410021H03Rik	0.86816	1.06368327	0.0881881
1421909_at	transcription factor 20	Tcf20	0.86817	1.05294316	0.07200497
1456959_at	RIKEN cDNA 1700034H15 gene	1700034H15Rik	0.86817	-1.06369587	-0.0891846
1425973_at	lysosomal trafficking regulator	Lyst	0.8682	-1.05917306	-0.0965534
1451052_at	component of oligomeric golgi complex 8 /// peptide deformylase	Cog8 /// Pdf	0.86822	1.05423776	0.07465861
1430675_at	RIKEN cDNA 2900055J20 gene	2900055J20Rik	0.86822	1.07302374	0.09508509
1416601_a_at	regulator of calcineurin 1	Rcan1	0.86824	1.0824254	0.10133764
1460159_at	myb-like, SWIRM and MPN domains 1	Mysm1	0.86825	-1.05945511	-0.0913832
1455382_at	ZW10 interactor	Zwint	0.86826	1.08718007	0.10583895
1442955_at	---	---	0.86826	-1.06327829	-0.0886774
1456855_at	---	---	0.86827	-1.05902457	-0.0879635
1437445_at	transient receptor potential cation channel, subfamily M, membe	Trpm1	0.86828	-1.06333996	-0.0973678
1454021_a_at	exosome component 10	Exosc10	0.8683	1.0420887	0.05946055
1442131_at	hypothetical LOC100503166	LOC100503166	0.8683	-1.06229732	-0.0877479
1424153_s_at	sal-like 4 (Drosophila)	Sall4	0.8683	-1.04944304	-0.0714978
1421874_a_at	mitochondrial ribosomal protein S23	Mrps23	0.8683	1.06576087	0.0834533
1429366_at	leucine rich repeat containing 34	Lrrc34	0.8683	1.04622181	0.06440823
1448628_at	secretogranin III	Scg3	0.86831	1.11639327	0.12949735
1420295_x_at	chloride channel 5	Clcn5	0.86831	-1.05346116	-0.080043
1421383_at	coiled-coil domain containing 82	Ccdc82	0.86831	1.09624382	0.11477502
1425884_at	ribosome production factor 2 homolog (S. cerevisiae)	Rpf2	0.86832	-1.0660961	-0.1334549
1441891_x_at	ELOVL family member 7, elongation of long chain fatty acids (yea	Elovl7	0.86832	-1.06894614	-0.1259392
1456627_at	Ubiquilin 2	Ubqln2	0.86832	1.08917755	0.10708673
1439428_x_at	GDP-mannose 4, 6-dehydratase	Gmds	0.86832	-1.07307353	-0.1356056
1417877_at	endonuclease/exonuclease/phosphatase family domain containi	Eepd1	0.86833	-1.06699802	-0.0994597
1439968_x_at	dysbindin (dystrobrevin binding protein 1) domain containing 2	Dbndd2	0.86833	1.07950821	0.10448869
1423344_at	erythropoietin receptor	Epor	0.86833	1.05614448	0.078132
1457700_at	cytochrome P450, family 4, subfamily f, polypeptide 39	Cyp4f39	0.86833	1.10548567	0.12110863
1437895_at	anoctamin 8	Ano8	0.86834	1.0646105	0.09023316
1452761_a_at	RNA binding motif, single stranded interacting protein	Rbms3	0.86834	-1.05876727	-0.0831857
1429650_at	serine/threonine kinase 40	Stk40	0.86834	-1.04877494	-0.0698467
1441726_at	---	---	0.86835	-1.06788601	-0.0953549
1460385_a_at	ring finger protein 112	Rnf112	0.86836	-1.06508847	-0.0952445
1444763_at	protein tyrosine phosphatase, receptor type, K	Ptprk	0.86836	1.08179662	0.10579302
1436999_at	phosphotyrosine interaction domain containing 1	Pid1	0.86836	1.07209145	0.09321276
1428112_at	mesencephalic astrocyte-derived neurotrophic factor	Manf	0.86837	1.0433404	0.06046243
1443327_at	RIKEN cDNA D130043K22 gene	D130043K22Rik	0.86837	-1.06471288	-0.0917444
1431654_at	RIKEN cDNA 1700112M01 gene	1700112M01Rik	0.86838	1.07728766	0.10167918
1440925_at	hypothetical LOC100503570	LOC100503570	0.86839	1.11910452	0.13188072
1439120_at	family with sequence similarity 120, member A	Fam120a	0.86839	-1.05653191	-0.0797579
1416006_at	midkine	Mdk	0.86839	1.04873233	0.0659963
1428418_s_at	family with sequence similarity 164, member A	Fam164a	0.8684	-1.05047944	-0.073848
1444882_at	---	---	0.8684	-1.06507561	-0.0969722
1441918_x_at	serine (or cysteine) peptidase inhibitor, clade B, member 6e	Serpinb6e	0.86841	-1.05666993	-0.0840818
1431371_at	RIKEN cDNA 9030411M13 gene	9030411M13Rik	0.86842	-1.05671207	-0.0847777
1444175_at	---	---	0.86842	-1.04505877	-0.0645039
1426280_at	expressed sequence AV249152	AV249152	0.86842	1.07334749	0.09941813
1458788_at	---	---	0.86842	-1.06555997	-0.095167
1416538_at	sialic acid acetylesterase	Siae	0.86843	1.06092264	0.08451023
1455259_a_at	UTP23, small subunit (SSU) processome component, homolog (ye	Utp23	0.86843	1.05705873	0.07913284
1437846_x_at	beta-site APP-cleaving enzyme 2	Bace2	0.86843	1.07753127	0.10209096
1427614_at	prolactin induced protein	Pip	0.86843	-1.06081943	-0.0856256
1440167_s_at	LIM domain containing preferred translocation partner in lipoma	Lpp	0.86844	-1.05858724	-0.0998453
1457229_at	G-protein coupled receptor 173	Gpr173	0.86845	-1.06365274	-0.0899881
1449107_at	nudix (nucleoside diphosphate linked moiety X)-type motif 4	Nudt4	0.86845	-1.04745095	-0.0689028
1457447_at	---	---	0.86845	1.09206887	0.11156783
1443021_at	5, 10-methenyltetrahydrofolate synthetase	Mthfs	0.86845	1.08355231	0.10620833
1415927_at	actin, alpha, cardiac muscle 1	Actc1	0.86845	-1.06649667	-0.0978528
1459021_at	Rab9 effector protein with kelch motifs	Rabepk	0.86846	-1.06481208	-0.0944631
1428291_at	exosome component 8	Exosc8	0.86846	1.04377944	0.06179215
1428625_a_at	RMI1, RecQ mediated genome instability 1, homolog (S. cerevisia	Rmi1	0.86846	-1.04953625	-0.069998
1456418_at	potassium inwardly-rectifying channel, subfamily J, member 13	Kcnj13	0.86846	1.07312571	0.09944476
1451406_a_at	proprotein convertase subtilisin/kexin type 5	Pcsk5	0.86846	-1.06115336	-0.0857427
1448585_at	general transcription factor II H, polypeptide 4	Gtf2h4	0.86847	1.05172158	0.06931381
1419175_a_at	butyrophilin, subfamily 1, member A1	Btn1a1	0.86847	1.13084606	0.14028035

1425084_at	GTPase, IMAP family member 7	Gimap7	0.86848	-1.06424701	-0.0911627
1453214_at	leucine rich repeat containing 15	Lrrc15	0.86848	1.06882875	0.09366051
1452404_at	phosphatase and actin regulator 2	Phactr2	0.86848	1.08824109	0.11180677
1429948_x_at	crystallin, gamma F	Crygf	0.86848	-1.06134504	-0.0907985
1435049_s_at	expressed sequence AI854703	AI854703	0.86848	-1.06651811	-0.0971837
1451035_a_at	aldo-keto reductase family 1, member A4 (aldehyde reductase)	Akr1a4	0.86849	1.04206395	0.05886182
1419400_at	microsomal triglyceride transfer protein	Mttp	0.86849	1.06686784	0.0924194
1460176_at	v-crk sarcoma virus CT10 oncogene homolog (avian)	Crk	0.86849	1.06003805	0.08378662
1427288_at	amyloid beta (A4) precursor protein-binding, family A, member 2	Apba2	0.8685	-1.06425079	-0.0908381
1447077_at	---	---	0.86851	-1.06581416	-0.099068
1425509_at	MAP/microtubule affinity-regulating kinase 1	Mark1	0.86851	1.07959211	0.10141784
1426056_at	phosphatidylinositol glycan anchor biosynthesis, class Q	Pigq	0.86851	1.06597561	0.09072223
1419028_at	cyclic AMP-regulated phosphoprotein, 21	Arpp21	0.86851	1.09656452	0.11400701
1457825_x_at	transcobalamin 2	Tcn2	0.86851	1.06513917	0.08790193
1429523_a_at	solute carrier family 39 (metal ion transporter), member 5	Slc39a5	0.86851	1.06442711	0.08915329
1448987_at	acyl-Coenzyme A dehydrogenase, long-chain	Acadl	0.86851	1.05417922	0.0733936
1417842_at	calcium modulating ligand	Caml	0.86851	1.05038449	0.07018027
1447618_at	RIKEN cDNA B630019K06 gene	B630019K06Rik	0.86852	1.05665609	0.07673454
1427553_at	predicted gene 16489	Gm16489	0.86852	1.10261715	0.11843944
1430518_at	RIKEN cDNA 5430402E10 gene	5430402E10Rik	0.86854	1.10670362	0.12164842
1443435_at	---	---	0.86854	-1.0594109	-0.0836937
1451769_s_at	protocadherin alpha 1 /// protocadherin alpha 10 /// protocadher	Pcdha1 /// Pcdha10 /// Pc	0.86855	1.06893053	0.09321333
1437793_at	---	---	0.86855	1.07206219	0.09470069
1426702_at	family with sequence similarity 160, member A2	Fam160a2	0.86855	-1.06861973	-0.1093007
1435968_at	protein tyrosine phosphatase, receptor type, N polypeptide 2	Ptprn2	0.86855	1.06848008	0.0948867
1441174_a_at	leishmanolysin-like (metallopeptidase M8 family)	Lmln	0.86856	1.13559098	0.14123072
1432836_at	RIKEN cDNA 4833410I11 gene	4833410I11Rik	0.86856	-1.06711759	-0.1154457
1444092_at	RIKEN cDNA 9430025M13 gene	9430025M13Rik	0.86857	-1.06196942	-0.0934014
1444982_at	RIKEN cDNA C230007H23 gene	C230007H23Rik	0.86858	1.08173934	0.10438913
1419240_at	testis expressed gene 14	Tex14	0.86858	1.06238081	0.08106545
1443638_at	hypothetical LOC100503244	LOC100503244	0.86859	-1.06462461	-0.0943099
1456907_at	chemokine (C-X-C motif) ligand 9	Cxcl9	0.8686	1.07237731	0.09547622
1445978_at	---	---	0.8686	1.06629428	0.08961126
1417529_at	RAB33A, member of RAS oncogene family	Rab33a	0.86861	1.06818828	0.09430199
1445798_at	discs, large homolog 1 (Drosophila)	Dlg1	0.86862	-1.0700568	-0.1333287
1450586_at	bradykinin receptor, beta 1	Bdkrb1	0.86864	1.06986842	0.09457061
1451271_a_at	acetyl-Coenzyme A acetyltransferase 1	Acat1	0.86865	-1.05672722	-0.0868156
1418581_a_at	LIM motif-containing protein kinase 2	Limk2	0.86867	1.05381936	0.0723756
1429880_at	RIKEN cDNA 4921531C22 gene	4921531C22Rik	0.86874	1.07085472	0.09608681
1452304_a_at	Rho guanine nucleotide exchange factor (GEF) 5	Arhgef5	0.86875	-1.06100531	-0.087903
1459059_at	RIKEN cDNA 2010308F09 gene	2010308F09Rik	0.86875	-1.06202293	-0.0893629
1432903_at	---	---	0.86876	-1.06914987	-0.104629
1437811_x_at	---	---	0.86878	1.09604752	0.1112068
1444974_at	expressed sequence AU023617	AU023617	0.86879	-1.06913355	-0.1123899
1454161_s_at	RIKEN cDNA 0610007P14 gene	0610007P14Rik	0.8688	1.0513628	0.0696981
1432663_at	RIKEN cDNA 1500005C15 gene	1500005C15Rik	0.86881	-1.06817791	-0.1048864
1451640_a_at	arginine/serine-rich coiled-coil 2	Rsrc2	0.86883	1.04919333	0.06837271
1452466_a_at	RNA binding motif protein 6	Rbm6	0.86886	-1.04790432	-0.067801
1443998_at	Ras association (RalGDS/AF-6) domain family member 2	Rassf2	0.86886	1.06356434	0.08781286
1445785_at	---	---	0.86887	1.06889235	0.09526101
1459134_at	---	---	0.86888	-1.06290742	-0.0900635
1432325_at	RIKEN cDNA 4930502M04 gene	4930502M04Rik	0.86889	-1.06396339	-0.0924355
1428400_at	RIKEN cDNA 2200002K05 gene	2200002K05Rik	0.8689	1.0642864	0.08674601
1443056_at	---	---	0.86891	-1.06272424	-0.0921543
1415960_at	myeloperoxidase	Mpo	0.86891	1.06654518	0.0913073
1455699_at	branched chain aminotransferase 1, cytosolic	Bcat1	0.86891	-1.05327866	-0.0761721
1441907_s_at	CD93 antigen	Cd93	0.86892	-1.06362787	-0.0924034
1416347_at	multiple endocrine neoplasia 1	Men1	0.86892	1.0517042	0.07261079
1440875_a_at	radical S-adenosyl methionine domain containing 1	Rsad1	0.86893	-1.06739852	-0.1016181
1434300_at	RIKEN cDNA 2610101N10 gene	2610101N10Rik	0.86895	-1.05148998	-0.081978
1422319_at	---	---	0.86895	-1.06805847	-0.0954836
1455196_s_at	expressed sequence AA987161	AA987161	0.86895	-1.04738686	-0.0677666
1447605_at	---	---	0.86896	-1.05014621	-0.0730795
1419733_at	GLI pathogenesis-related 1 like 2	Glipr1l2	0.86896	1.05897334	0.08263237
1430742_a_at	spermatogenesis associated 16	Spata16	0.86896	1.06867311	0.09329338
1425526_a_at	paired related homeobox 1	Prrx1	0.86896	1.14943851	0.14757239
1450482_a_at	paired-like homeodomain transcription factor 2	Pitx2	0.86897	1.12895197	0.13849924
1417411_at	nucleosome assembly protein 1-like 5	Nap1l5	0.86898	-1.06595971	-0.1014264
1435461_at	membrane associated guanylate kinase, WW and PDZ domain co	Magi3	0.86898	1.07315496	0.09168924
1449335_at	tissue inhibitor of metalloproteinase 3	Timp3	0.86899	-1.06946313	-0.1057766
1418283_at	claudin 4	Cldn4	0.86899	1.06045346	0.08020157
1429957_at	keratin associated protein 26-1	Krtap26-1	0.86899	-1.06735068	-0.0966813
1442002_at	TATA element modulatory factor 1	Tmf1	0.869	-1.06142414	-0.0901498
1457872_at	---	---	0.86902	-1.06826301	-0.113487
1430793_at	RIKEN cDNA 4931429I11 gene	4931429I11Rik	0.86902	1.0696675	0.09270412
1419775_at	---	---	0.86902	-1.06175888	-0.0867485
1442558_at	predicted gene 10421	Gm10421	0.86903	1.10475155	0.12072406
1433644_at	protein-O-mannosyltransferase 2	Pomt2	0.86903	1.05871672	0.08108199
1416333_at	docking protein 2	Dok2	0.86905	1.06413983	0.08514751
1434883_at	metadherin	Mtdh	0.86906	-1.04589823	-0.0673128
1435700_at	talin 2	Tln2	0.86907	1.07307471	0.09548899
1459537_at	---	---	0.86907	1.07904194	0.10274618
1430788_at	---	---	0.86909	-1.05622448	-0.0827866
1420672_at	potassium voltage-gated channel, Isk-related subfamily, membe	Kcne1	0.8691	1.05801512	0.08128807
1435376_at	DDHD domain containing 2	Ddhd2	0.8691	1.05256472	0.07173462
1416022_at	fatty acid binding protein 5, epidermal	Fabp5	0.86917	1.07430299	0.08923816
1425123_at	kelch-like 36 (Drosophila)	Klhl36	0.86926	1.07082949	0.09512313
1424139_at	RAS-related protein-1a	Rap1a	0.86926	-1.04632749	-0.0680338
1453063_at	clathrin, light polypeptide (Lcb)	Cltb	0.86929	1.05028938	0.06753151
1437135_at	F-box and WD-40 domain protein 10	Fbxw10	0.86929	1.06870808	0.09425855
1444874_at	ATP synthase, H+ transporting, mitochondrial F0 complex, subunit	Atp5g1	0.8693	1.06606374	0.08989583
1416339_a_at	protein kinase C substrate 80K-H	Prkcsh	0.86931	1.05215893	0.07070017
1415987_at	high density lipoprotein (HDL) binding protein	Hdlbp	0.86932	1.04523091	0.06282987
1452274_at	zinc finger, AN1-type domain 3	Zfand3	0.86933	1.05585365	0.07394387

1454185_at	RIKEN cDNA 4933436E23 gene	4933436E23Rik	0.86933	-1.06759474	-0.1013705
1426501_a_at	TRAF-interacting protein with forkhead-associated domain	Tifa	0.86936	-1.06552217	-0.092471
1447803_x_at	capping protein (actin filament), gelsolin-like	Capg	0.86936	-1.06233613	-0.0878433
1443016_at	---	---	0.86937	-1.06612262	-0.1101415
1431771_a_at	interleukin-1 receptor-associated kinase 1 binding protein 1	Irak1bp1	0.86938	1.05615724	0.07671536
1422147_a_at	phospholipase A2, group VI	Pla2g6	0.86938	-1.04566126	-0.0645111
1423998_at	general transcription factor IIIC, polypeptide 5	Gtf3c5	0.86938	1.05861325	0.07783602
1425086_a_at	SLAM family member 6	Slamf6	0.86938	1.08094201	0.10145119
1442152_at	RIKEN cDNA 4930513N10 gene	4930513N10Rik	0.86941	-1.0658935	-0.1030036
1447666_x_at	RIKEN cDNA 3200002M19 gene	3200002M19Rik	0.86942	-1.06492604	-0.113866
1445224_at	---	---	0.86942	-1.06645077	-0.0957708
1417609_at	ubiquitin-conjugating enzyme E2A, RAD6 homolog (S. cerevisiae)	Ube2a	0.86943	1.04918496	0.06891236
1457293_at	zinc finger and BTB domain containing 4	Zbtb4	0.86945	-1.06676305	-0.1033678
1422502_at	poly (ADP-ribose) polymerase family, member 1	Parp1	0.86946	1.03951451	0.05556073
1420904_at	interleukin 17 receptor A	Il17ra	0.86947	1.10132589	0.1159174
1432217_a_at	WD repeat domain 16	Wdr16	0.8695	1.09566237	0.11349285
1431122_at	armadillo repeat containing 9	Armc9	0.86951	1.07748488	0.10158237
1457005_at	mitochondrial methionyl-tRNA formyltransferase	Mtfmt	0.86954	1.05842285	0.08056897
1421599_at	heparan sulfate 6-O-sulfotransferase 3	Hs6st3	0.86954	-1.07086437	-0.111389
1457180_at	DIP2 disco-interacting protein 2 homolog C (Drosophila)	Dip2c	0.86955	-1.0695984	-0.112942
1437860_at	protein kinase C, epsilon	Prkce	0.86957	1.04602075	0.06256851
1418888_a_at	selenoprotein X 1	Sepx1	0.86958	1.04959745	0.06786413
1439023_at	DEAD (Asp-Glu-Ala-Asp) box polypeptide 55	Ddx55	0.86958	1.06060674	0.08448078
1430951_at	RIKEN cDNA 2810011L19 gene	2810011L19Rik	0.86959	1.06056715	0.08339987
1434102_at	nuclear factor I/B	Nfib	0.86959	-1.07110922	-0.1558678
1424041_s_at	complement component 1, s subcomponent	C1s	0.86959	1.08489247	0.10682765
1432834_at	carboxypeptidase B2 (plasma)	Cpb2	0.86959	1.07409569	0.09956027
1459317_at	ankyrin 2, brain	Ank2	0.86961	1.06612774	0.09159633
1440440_at	---	---	0.86963	-1.05616121	-0.0794688
1439275_s_at	RIKEN cDNA 9530010C24 gene	9530010C24Rik	0.86963	-1.07047242	-0.1064317
1425072_at	S-phase kinase-associated protein 2 (p45)	Skp2	0.86964	-1.05965095	-0.0886636
1421455_at	syntrophin, basic 1	Sntb1	0.86965	-1.06908073	-0.1264755
1438417_at	PWWP domain containing 2B	Pwwp2b	0.86965	-1.05295088	-0.0749751
1421240_at	endoplasmic reticulum (ER) to nucleus signalling 1	Ern1	0.86966	1.06355425	0.08692545
1421025_at	1-acylglycerol-3-phosphate O-acyltransferase 1 (lysophosphatidi	Agpat1	0.86967	-1.06202849	-0.1024533
1428884_at	transmembrane protein 57	Tmem57	0.86969	1.05056258	0.06952976
1451202_at	RIKEN cDNA C330007P06 gene	C330007P06Rik	0.86969	-1.05238685	-0.0817473
1455365_at	cadherin 8	Cdh8	0.86969	-1.05777499	-0.0817028
1422749_at	lymphocyte antigen 6 complex, locus G6C	Ly6g6c	0.8697	-1.06746326	-0.0956783
1417705_at	OTU domain, ubiquitin aldehyde binding 1	Otub1	0.86972	1.09778057	0.10833563
1457667_x_at	---	---	0.86972	1.05627315	0.07627873
1445746_at	Eukaryotic translation initiation factor 4H	Eif4h	0.86973	1.07741204	0.10057021
1460726_at	adenylosuccinate synthetase, non muscle	Adss	0.86974	1.05326031	0.06945411
1448621_a_at	sphingomyelin phosphodiesterase 1, acid lysosomal	Smpd1	0.86975	1.0569872	0.07891853
1439703_at	CD200 receptor 1	Cd200r1	0.86976	1.06056377	0.07946318
1443741_x_at	Wolf-Hirschhorn syndrome candidate 1 (human)	Whsc1	0.86976	-1.06996324	-0.1022449
1443341_at	---	---	0.86977	1.08245664	0.10601525
1424028_at	CTF8, chromosome transmission fidelity factor 8 homolog (S. cere	Chtf8	0.86977	1.04507386	0.06305223
1450872_s_at	lysosomal acid lipase A	Lipa	0.8698	1.11535762	0.12714038
1416927_at	transformation related protein 53 inducible nuclear protein 1	Trp53inp1	0.86983	-1.05761891	-0.0870346
1425542_a_at	protein phosphatase 2, regulatory subunit B (B56), gamma isoforr	Ppp2r5c	0.86983	-1.04329307	-0.0617826
1452504_s_at	chitobiase, di-N-acetyl-	Ctbs	0.86983	-1.05712336	-0.0866983
1457237_at	Muscleblind-like 2	Mbnl2	0.86984	1.05312572	0.07360744
1426822_at	ras homolog gene family, member T2	Rhot2	0.86984	1.06605625	0.0836284
1427495_at	sodium channel, voltage-gated, type VII, alpha	Scn7a	0.86985	1.09646399	0.11501777
1458810_at	---	---	0.86985	1.07275385	0.09588681
1417887_at	integrator complex subunit 5	Ints5	0.86985	1.06144673	0.08190342
1447399_at	---	---	0.86985	-1.05981328	-0.0865481
1416869_x_at	Lck interacting transmembrane adaptor 1	Lime1	0.86986	1.05805279	0.0812767
1423376_a_at	docking protein 4	Dok4	0.86986	1.06226337	0.08482308
1451580_a_at	transthyretin	Ttr	0.86986	1.06191186	0.08444465
1446404_at	---	---	0.86987	1.06652463	0.08828333
1423588_at	actin related protein 2/3 complex, subunit 4	Arpc4	0.86987	-1.04192776	-0.0606655
1428189_at	RIKEN cDNA 5730494M16 gene	5730494M16Rik	0.86988	1.05478031	0.0732997
1423470_at	polypyrimidine tract binding protein 2	Ptbp2	0.86988	-1.04919787	-0.0724294
1422262_a_at	LIM homeobox protein 6	Lhx6	0.86988	1.07406477	0.09766683
1433019_at	zinc finger and BTB domain containing 7C	Zbtb7c	0.86988	-1.05438845	-0.0828474
1417007_a_at	vacuolar protein sorting 4b (yeast)	Vps4b	0.86989	-1.04651746	-0.0712673
1421990_at	synaptotagmin I	Syt1	0.86989	-1.06456223	-0.0973163
1419707_at	keratin associated protein 14	Krtap14	0.8699	-1.06212632	-0.0937213
1424134_at	ring finger and SPRY domain containing 1	Rspry1	0.86991	-1.05379347	-0.0836023
1450266_at	sodium channel, voltage-gated, type X, alpha	Scn10a	0.86991	-1.05978611	-0.0850682
1451493_at	Nedd4 family interacting protein 1	Ndfip1	0.86994	1.04668764	0.06563676
1443142_at	beta-transducin repeat containing protein	Btrc	0.86994	1.07403049	0.09801901
1427898_at	ring finger protein (C3H2C3 type) 6	Rnf6	0.86998	-1.05139928	-0.0759695
1458010_at	---	---	0.86999	-1.06243265	-0.11599
1426997_at	thyroid hormone receptor alpha	Thra	0.86999	1.05519743	0.0751564
1445426_at	---	---	0.87	-1.05774783	-0.0857569
1454479_at	---	---	0.87	-1.06615982	-0.0930649
1444754_at	additional sex combs like 2 (Drosophila)	Asxl2	0.87001	-1.0614639	-0.095199
1439721_at	F-box and WD-40 domain protein 18	Fbxw18	0.87001	-1.06982281	-0.1101055
1444825_at	---	---	0.87001	-1.06773212	-0.1031101
1418982_at	CCAAT/enhancer binding protein (C/EBP), alpha	Cebpa	0.87002	-1.06394545	-0.0913779
1419267_at	nuclear transcription factor-Y beta	Nfyb	0.87002	1.0520084	0.06892832
1441734_at	calcium/calmodulin-dependent protein kinase II alpha	Camk2a	0.87004	-1.0574012	-0.0870649
1428388_at	tankyrase, TRF1-interacting ankyrin-related ADP-ribose polymera	Tnks2	0.87004	1.04967245	0.06600413
1459941_at	clavesin 1	Clvs1	0.87005	1.05603297	0.07813251
1446922_at	---	---	0.87005	1.05351862	0.07516797
1439228_at	zinc finger protein 407	Zfp407	0.87005	-1.06788993	-0.1110019
1417618_at	inter-alpha trypsin inhibitor, heavy chain 2	Itih2	0.87005	-1.06422184	-0.0909985
1418157_at	nuclear receptor subfamily 2, group F, member 1	Nr2f1	0.87005	1.0503139	0.06995686
1456828_at	RIKEN cDNA C030023E24 gene	C030023E24Rik	0.87006	1.04895465	0.06853616
1436706_at	membrane magnesium transporter 1	Mmgt1	0.87006	-1.05288619	-0.0747471
1416448_at	inosine triphosphatase (nucleoside triphosphate pyrophosphatase	Itpa	0.87006	1.04326904	0.06110328

1443844_at	reproductive homeobox 13	Rhox13	0.87006	1.06482012	0.08561138
1427009_at	laminin, alpha 5	Lama5	0.87007	-1.04929271	-0.073481
1416750_at	sigma non-opioid intracellular receptor 1	Sigmar1	0.87007	1.04944091	0.06773135
1442847_at	---	---	0.87008	-1.05450121	-0.0768585
1440055_at	---	---	0.87008	-1.0666933	-0.1071771
1457904_at	carbonic anhydrase 8	Car8	0.87008	-1.05924081	-0.1011757
1444619_x_at	proteasome (prosome, macropain) subunit, beta type 8 (large mu	Psmb8	0.87009	1.06871238	0.08980317
1459944_at	UTP11-like, U3 small nucleolar ribonucleoprotein, (yeast)	Utp11l	0.87009	-1.06777492	-0.0980301
1459816_x_at	polymerase (DNA directed), epsilon 3 (p17 subunit)	Pole3	0.8701	1.06522135	0.08518075
1418081_at	DnaJ (Hsp40) homolog, subfamily C, member 30	Dnajc30	0.87011	1.05463806	0.07416179
1443668_x_at	expressed sequence C79407	C79407	0.87011	-1.06405261	-0.093621
1446267_at	vomeronasal 2, receptor, pseudogene 129	Vmn2r-ps129	0.87012	1.05914423	0.08174628
1439502_at	tectonin beta-propeller repeat containing 2	Tecpr2	0.87013	1.0681936	0.09094939
1423937_at	potassium channel tetramerisation domain containing 5	Kctd5	0.87014	1.04683081	0.0653741
1457457_at	SLAIN motif family, member 2	Slain2	0.87015	-1.06894034	-0.1053527
1422523_at	silver	Si	0.87015	-1.05317469	-0.0791316
1456072_at	protein phosphatase 1, regulatory (inhibitor) subunit 9A	Ppp1r9a	0.87015	-1.05391554	-0.0763535
1447195_at	---	---	0.87016	1.1434987	0.14442813
1433532_a_at	myelin basic protein	Mbp	0.87016	1.08189211	0.10029322
1435641_at	mannoside acetylglucosaminyltransferase 4, isoenzyme A	Mgat4a	0.87018	-1.06158646	-0.0870304
1423643_at	DEAD (Asp-Glu-Ala-Asp) box polypeptide 39	Ddx39	0.87018	1.04003915	0.05551934
1426765_at	COMM domain containing 7	Commd7	0.87019	1.08172384	0.09822744
1449751_at	Solute carrier family 6 (neurotransmitter transporter, taurine), me	Slc6a6	0.8702	-1.0654526	-0.1192325
1438077_at	NLR family, pyrin domain containing 4A	Nlrp4a	0.8702	-1.0627868	-0.0978209
1432548_at	poly (ADP-ribose) polymerase family, member 14	Parp14	0.87021	1.10631889	0.12496911
1458916_at	---	---	0.87021	1.10300646	0.11741856
1424395_at	asparaginase like 1	Asrgl1	0.87022	1.0513358	0.07217611
1429247_at	annexin A6	Anxa6	0.87023	-1.06678657	-0.1060475
1439548_at	RAP2B, member of RAS oncogene family	Rap2b	0.87024	-1.068848	-0.116635
1416005_at	protease (prosome, macropain) 26S subunit, ATPase 1	Psmc1	0.87026	-1.04347369	-0.0621643
1425863_a_at	protein tyrosine phosphatase, receptor type, O	Ptpro	0.87027	1.0643697	0.08914969
1437783_x_at	canopy 2 homolog (zebrafish)	Cnpy2	0.87027	-1.05136747	-0.0917795
1441287_at	Mbt domain containing 1	Mbtd1	0.87027	-1.06730007	-0.0965134
1430022_at	SUMO1 activating enzyme subunit 1	Sae1	0.87027	1.06708496	0.09231969
1425826_a_at	sorbin and SH3 domain containing 1	Sorbs1	0.87027	1.08001264	0.09979515
1429866_at	AHNAK nucleoprotein (desmoyokin)	Ahnak	0.87027	1.06753979	0.09337307
1457171_at	---	---	0.87027	-1.05850601	-0.0824761
1433040_at	RIKEN cDNA 2900018E21 gene	2900018E21Rik	0.87027	-1.06421531	-0.0936897
1441660_at	ARP2 actin-related protein 2 homolog (yeast)	Actr2	0.87028	-1.06757466	-0.1005126
1427761_at	exoribonuclease 3	Eri3	0.8703	1.07181091	0.09428948
1422157_a_at	integrin beta 1 binding protein 1	Itgb1bp1	0.8703	1.06612653	0.08393181
1429365_at	RIKEN cDNA 1700016M24 gene	1700016M24Rik	0.87031	-1.06416331	-0.0964993
1434063_at	zinc finger protein 664	Zfp664	0.87031	1.0492586	0.0679652
1423268_at	integrin alpha 5 (fibronectin receptor alpha)	Itga5	0.87031	-1.0502286	-0.0748387
1419719_at	gamma-aminobutyric acid (GABA) A receptor, subunit beta 1	Gabrb1	0.87032	-1.06623537	-0.0937154
1421966_at	neural cell adhesion molecule 1	Ncam1	0.87033	-1.06716848	-0.0969445
1444025_at	potassium inwardly-rectifying channel, subfamily J, member 3	Kcnj3	0.87035	-1.06643292	-0.1110277
1447785_x_at	DNA segment, Chr 11, Wayne State University 99, expressed	D11Wsu99e	0.87035	-1.05899175	-0.0852804
1440435_at	kyphoscoliosis peptidase	Ky	0.87045	1.0892676	0.11004584
1454543_at	---	---	0.8705	-1.06550679	-0.1126856
1442490_at	RIKEN cDNA C130090I23 gene	C130090I23Rik	0.87055	-1.07121363	-0.1379383
1430437_a_at	RIKEN cDNA 1300018I17 gene	1300018I17Rik	0.87055	1.07269591	0.09564355
1452076_at	SPRY domain containing 4	Spryd4	0.87057	1.04740993	0.06630746
1450680_at	recombination activating gene 1	Rag1	0.87058	-1.05601665	-0.0847093
1424370_s_at	proteasome (prosome, macropain) inhibitor subunit 1	Psmf1	0.87058	1.06783347	0.08821868
1443150_at	---	---	0.87058	1.06973653	0.09442161
1439832_at	---	---	0.87059	1.18345215	0.16984219
1437555_at	BarH-like 2 (Drosophila)	Barhl2	0.87059	-1.0541151	-0.0785913
1425294_at	SLAM family member 8	Slamf8	0.87061	-1.06174318	-0.0907788
1429331_at	---	---	0.87061	-1.06274839	-0.0998923
1442465_s_at	spermatid perinuclear RNA binding protein	Strbp	0.87061	-1.04248107	-0.0604942
1440978_at	phosphatidylinositol glycan anchor biosynthesis, class M	Pigm	0.87062	-1.05859407	-0.082682
1445252_at	kringle containing transmembrane protein 1	Kremen1	0.87063	-1.06643311	-0.1003173
1454746_at	pleckstrin homology domain containing, family M (with RUN dom	Plekhm1	0.87064	-1.05015485	-0.0727483
1444777_at	Retinoic acid induced 14	Rai14	0.87065	1.10390761	0.11810663
1427297_at	mitochondrial ribosomal protein L9	Mrpl9	0.87065	-1.04331164	-0.0617516
1454457_at	RIKEN cDNA 4930439G18 gene	4930439G18Rik	0.87068	-1.06794308	-0.0985313
1417831_at	structural maintenance of chromosomes 1A	Smc1a	0.87068	1.06693128	0.08580083
1428714_at	progesterone receptor membrane component 2	Pgrmc2	0.87068	1.04641503	0.0648381
1440759_at	predicted gene 10672	Gm10672	0.87069	-1.06492339	-0.1006659
1442435_at	---	---	0.8707	1.06781257	0.0897776
1434095_at	unc-5 homolog A (C. elegans)	Unc5a	0.8707	1.06129235	0.08439063
1438164_x_at	flotillin 2	Flot2	0.87071	-1.04754538	-0.0678933
1443603_at	---	---	0.87071	-1.06916478	-0.1172365
1459287_at	---	---	0.87071	1.06371852	0.08860313
1415705_at	RIKEN cDNA 9130011J15 gene	9130011J15Rik	0.87071	-1.05295881	-0.0774093
1452946_a_at	raftlin family member 2	Rftn2	0.87071	-1.0701571	-0.0994557
1455701_at	Rho GTPase activating protein 33	Arhgap33	0.87073	1.06737672	0.09199178
1417986_at	Notch-regulated ankyrin repeat protein	Nrarp	0.87073	1.06622873	0.08622312
1438096_a_at	deoxythymidylate kinase	Dtymk	0.87074	1.04320535	0.05957966
1449372_at	DnaJ (Hsp40) homolog, subfamily C, member 3	Dnajc3	0.87074	1.06765023	0.08914277
1444474_at	ATG2 autophagy related 2 homolog B (S. cerevisiae)	Atg2b	0.87074	-1.06657172	-0.0998204
1439072_at	solute carrier family 1 (glial high affinity glutamate transporter), m	Slc1a3	0.87075	1.06565418	0.09102977
1427530_at	bicaudal D homolog 1 (Drosophila)	Bicd1	0.87075	-1.07076708	-0.1239938
1421254_a_at	sarcoglycan, gamma (dystrophin-associated glycoprotein)	Sgcg	0.87075	-1.06399398	-0.0916667
1433438_x_at	melanoma antigen	Mela	0.87075	1.06289469	0.08034616
1452409_at	glioma tumor suppressor candidate region gene 2	Gltscr2	0.87076	1.07033127	0.09063938
1451337_at	proteasome (prosome, macropain) inhibitor subunit 1	Psmf1	0.87076	1.04277422	0.06009287
1427917_s_at	single-stranded DNA binding protein 3	Ssbp3	0.87077	1.05344984	0.07310806
1430387_at	RIKEN cDNA 1810073O08 gene	1810073O08Rik	0.87077	1.08675701	0.10721446
1421956_at	recombination signal binding protein for immunoglobulin kappa J	Rbpjl	0.87077	-1.06253507	-0.0886413
1427738_at	RIKEN cDNA A230006K03 gene	A230006K03Rik	0.87078	-1.05094738	-0.0773861
1453712_a_at	mitogen-activated protein kinase kinase 5	Map2k5	0.87078	-1.05635512	-0.079784
1447903_x_at	adaptor-related protein complex 1, sigma 2 subunit	Ap1s2	0.87078	1.07667927	0.09677966

1437512_x_at	EBNA1 binding protein 2	Ebna1bp2	0.87078	-1.05882478	-0.0852603
1434612_s_at	sno, strawberry notch homolog 1 (Drosophila)	Sbno1	0.87078	1.05126912	0.06829172
1417538_at	solute carrier family 35 (CMP-sialic acid transporter), member 1	Slc35a1	0.87078	1.04663845	0.06522441
1447085_s_at	nuclear factor of activated T-cells, cytoplasmic, calcineurin-depen	Nfatc1	0.87079	-1.06313732	-0.0902874
1459831_s_at	transmembrane and coiled-coil domains 5	Tmco5	0.87079	1.05567293	0.0761048
1447808_s_at	solute carrier family 15 (H+/peptide transporter), member 2	Slc15a2	0.8708	-1.06537079	-0.0936673
1431090_at	Ssu72 RNA polymerase II CTD phosphatase homolog (yeast)	Ssu72	0.8708	1.08469999	0.10174184
1424253_at	family with sequence similarity 114, member A2	Fam114a2	0.8708	-1.04626668	-0.068115
1457003_at	glutamate receptor, ionotropic, NMDA2B (epsilon 2)	Grin2b	0.8708	-1.06003966	-0.0859466
1452964_at	tubulin tyrosine ligase-like family, member 11	Ttll11	0.87081	-1.06308197	-0.0924626
1429238_a_at	2-oxoglutarate and iron-dependent oxygenase domain containing	Ogfod2	0.87081	1.06182388	0.08525249
1431815_a_at	MMS19 (MET18 S. cerevisiae)	Mms19	0.87081	1.04820681	0.06742817
1460507_at	RIKEN cDNA 4930552P12 gene	4930552P12Rik	0.87082	1.1178219	0.12866121
1444627_at	---	---	0.87083	1.06133821	0.08339113
1429583_at	RIKEN cDNA 2900053A13 gene	2900053A13Rik	0.87083	1.07001472	0.09622849
1448721_at	DNA segment, Chr 1, ERATO Doi 622, expressed	D1Ertd622e	0.87083	1.07242829	0.09558985
1456722_at	chordin-like 1	Chrdl1	0.87083	-1.05605313	-0.0790245
1431796_at	RIKEN cDNA 2810430I11 gene	2810430I11Rik	0.87083	-1.06661512	-0.0938528
1444214_at	tubulin, beta 1	Tubb1	0.87084	1.06716346	0.09213258
1459053_at	ArfGAP with GTPase domain, ankyrin repeat and PH domain 1	Agap1	0.87084	1.06894076	0.09284332
1458223_at	transformed mouse 3T3 cell double minute 4	Mdm4	0.87084	1.10450667	0.11665523
1424006_at	alanyl-tRNA synthetase domain containing 1	Aarsd1	0.87084	1.0575215	0.07597644
1454267_a_at	protease, serine, 32	Prss32	0.87085	1.06503373	0.09065606
1423454_a_at	sema domain, transmembrane domain (TM), and cytoplasmic do	Sema6c	0.87085	1.05514141	0.07729679
1445126_at	---	---	0.87085	-1.0695787	-0.1134989
1448736_a_at	hypoxanthine guanine phosphoribosyl transferase	Hprt	0.87086	1.08594489	0.09500716
1444593_at	PH domain and leucine rich repeat protein phosphatase 2	Phlpp2	0.87088	1.08791586	0.10679121
1424841_s_at	ribokinase	Rbks	0.87088	1.06191749	0.08360175
1460426_at	phosphodiesterase 4D interacting protein (myomegalin)	Pde4dip	0.87089	-1.06098919	-0.0903204
1418933_at	solute carrier family 1 (high affinity aspartate/glutamate transpor	Slc1a6	0.87089	-1.06784975	-0.1194192
1431024_a_at	AT rich interactive domain 4B (RBP1-like)	Arid4b	0.8709	-1.06763538	-0.0999423
1460455_at	ubiquitin protein ligase E3 component n-recognin 3	Ubr3	0.87091	-1.05254906	-0.0757111
1434580_at	ectonucleotide pyrophosphatase/phosphodiesterase 4	Enpp4	0.87092	1.06132645	0.08151259
1416508_at	mediator of RNA polymerase II transcription, subunit 28 homolog	Med28	0.87094	1.04552214	0.06279428
1423309_at	trans-golgi network protein	Tgoln1	0.87094	-1.04936253	-0.072075
1428685_at	synaptonemal complex central element protein 1	Syce1	0.87095	1.0620689	0.0806434
1444467_at	expressed sequence C77534	C77534	0.87096	-1.0627052	-0.091333
1439255_s_at	G protein-coupled receptor 137B /// G protein-coupled receptor 1	Gpr137b /// Gpr137b-ps	0.87097	-1.0654953	-0.1333226
1427540_at	ZW10 interactor	Zwint	0.87098	1.0639411	0.08382324
1452818_at	transcription termination factor, RNA polymerase II	Ttf2	0.871	1.05696066	0.07621455
1450748_at	sphingomyelin phosphodiesterase 3, neutral	Smpd3	0.871	1.06839599	0.09287878
1420396_at	CD160 antigen	Cd160	0.87102	-1.0643119	-0.0908095
1453280_at	predicted gene 14781 /// melanoma antigen, family B, 5	Gm14781 /// Mageb5	0.87104	-1.06690275	-0.1003709
1460221_at	prostaglandin E synthase 3 (cytosolic)	Ptges3	0.87104	-1.03285233	-0.0491498
1424170_at	PHD finger protein 5A	Phf5a	0.87104	1.03873852	0.05405697
1444485_at	SHQ1 homolog (S. cerevisiae)	Shq1	0.87106	1.05542516	0.07728312
1443932_at	kelch domain containing 1	Klhdc1	0.87106	-1.0645231	-0.0936756
1444620_at	---	---	0.87107	1.04876826	0.06626062
1441087_at	RIKEN cDNA 2810011L19 gene	2810011L19Rik	0.87107	-1.0629422	-0.0915931
1427472_a_at	complement component 8, beta polypeptide	C8b	0.87107	1.07011848	0.09431809
1452426_x_at	---	---	0.87108	1.11572322	0.12469522
1453645_at	RIKEN cDNA 2700046A07 gene	2700046A07Rik	0.87108	-1.05914498	-0.0832633
1445710_x_at	double homeobox B-like /// predicted gene 10394	Duxbl /// Gm10394	0.87109	1.08492271	0.10297752
1439745_at	calcium channel, voltage-dependent, gamma subunit 7	Cacng7	0.87109	-1.06170123	-0.100295
1444360_at	---	---	0.87109	-1.06647015	-0.1155095
1447357_at	---	---	0.87109	-1.06528085	-0.0961588
1423746_at	thioredoxin domain containing 5	Txndc5	0.87109	-1.04293299	-0.0614254
1434313_at	coiled-coil domain containing 126	Ccdc126	0.8711	-1.05649201	-0.0795042
1437599_at	---	---	0.8711	1.07552748	0.10010953
1424303_at	DEP domain containing 7	Depdc7	0.87111	1.04766223	0.06693125
1424239_at	family with sequence similarity 65, member A	Fam65a	0.87111	1.08547976	0.10318078
1455945_at	zinc finger protein 58	Zfp58	0.87111	1.06775932	0.08739103
1445300_at	---	---	0.87112	-1.06740042	-0.096047
1426973_at	G protein-coupled receptor 153	Gpr153	0.87112	1.0735383	0.09562675
1459508_at	---	---	0.87113	-1.04033291	-0.0580998
1434846_at	DENN/MADD domain containing 4C	Dennd4c	0.87113	-1.0508424	-0.0729944
1440406_at	G patch domain containing 3	Gpatch3	0.87113	1.07872625	0.09760173
1448475_at	olfactomedin-like 3	Olfml3	0.87114	-1.06259396	-0.091903
1415695_at	proteasome (prosome, macropain) subunit, alpha type 1	Psma1	0.87114	-1.04048802	-0.0573401
1421344_a_at	ajuba	Jub	0.87116	-1.04342728	-0.0637334
1453176_a_at	RIKEN cDNA 4933404M02 gene	4933404M02Rik	0.87118	1.08106031	0.10012946
1424934_at	UDP glucuronosyltransferase 2 family, polypeptide B1	Ugt2b1	0.8712	1.07752805	0.0971965
1417490_at	cathepsin B	Ctsb	0.8712	1.04699919	0.06610373
1420725_at	trimethyllysine hydroxylase, epsilon	Tmlhe	0.87121	-1.06219741	-0.1021035
1426127_x_at	killer cell lectin-like receptor, subfamily A, member 18	Klra18	0.87121	1.09024089	0.10314837
1438031_at	RAS, guanyl releasing protein 3	Rasgrp3	0.87121	-1.06926653	-0.1063342
1419328_at	sema domain, immunoglobulin domain (Ig), TM domain, and sho	Sema4f	0.87121	-1.06210807	-0.0895855
1445973_at	expressed sequence C79461	C79461	0.87122	-1.06677998	-0.094106
1438047_at	zinc finger protein 384	Zfp384	0.87122	1.04245502	0.05964858
1427756_x_at	Immunoglobulin heavy chain 6 (heavy chain of IgM)	Igh-6	0.87123	-1.05590558	-0.0888734
1421321_a_at	neuroepithelial cell transforming gene 1	Net1	0.87123	1.05536438	0.07651053
1449942_a_at	integrin linked kinase	Ilk	0.87123	1.05589197	0.07495668
1457707_at	multiple C2 domains, transmembrane 2	Mctp2	0.87124	-1.05505698	-0.0779588
1453019_at	nuclear VCP-like	Nvl	0.87124	1.05418444	0.07373966
1456158_at	RIKEN cDNA 2610109H07 gene	2610109H07Rik	0.87125	-1.06845245	-0.1077822
1441276_at	protein tyrosine phosphatase, receptor type, K	Ptprk	0.87125	1.05418552	0.07429702
1458922_at	mitogen-activated protein kinase kinase kinase 9	Map3k9	0.87126	-1.06506487	-0.0919844
1425187_at	sel-1 suppressor of lin-12-like (C. elegans)	Sel1l	0.87126	1.06760247	0.08767968
1430758_x_at	breast carcinoma amplified sequence 1	Bcas1	0.87126	-1.06751435	-0.0948231
1450755_at	platelet-activating factor acetylhydrolase, isoform 1b, subunit 2	Pafah1b2	0.87126	1.05701329	0.07735224
1417470_at	apolipoprotein B mRNA editing enzyme, catalytic polypeptide 3	Apobec3	0.87126	1.04107154	0.05805072
1416836_at	low-density lipoprotein receptor-related protein 10	Lrp10	0.87126	1.0468745	0.06569536
1433819_s_at	1-acylglycerol-3-phosphate O-acyltransferase 3	Agpat3	0.87127	1.05536581	0.0776032
1431552_at	RIKEN cDNA 1810006J02 gene	1810006J02Rik	0.87128	1.07021473	0.08673351

1439636_at	NLR family, pyrin domain containing 9C	Nlrp9c	0.87128	1.0666264	0.08567224
1424751_at	activator of basal transcription 1	Abt1	0.87129	1.0493962	0.06733508
1445372_at	---	---	0.87129	-1.07023879	-0.1061144
1430155_at	RIKEN cDNA 4933427E11 gene	4933427E11Rik	0.8713	-1.05984717	-0.0844271
1457518_at	RIKEN cDNA C630004H02 gene	C630004H02Rik	0.8713	-1.06848598	-0.0982526
1454705_at	DNA segment, Chr 15, ERATO Doi 621, expressed	D15Ertd621e	0.8713	-1.04733036	-0.0680884
1444007_at	RIKEN cDNA A830082K12 gene	A830082K12Rik	0.87131	1.04801981	0.06637974
1419051_at	OVO homolog-like 1 (Drosophila)	Ovol1	0.87131	1.09717522	0.11518725
1428173_at	echinoderm microtubule associated protein like 2	Eml2	0.87132	-1.05131895	-0.0724138
1448788_at	CD200 antigen	Cd200	0.87133	1.06015401	0.08230215
1420828_s_at	14-3-3 protein theta-like /// tyrosine 3-monooxygenase/tryptoph	LOC100503129 /// Ywhaq	0.87134	1.03996937	0.05410394
1452393_at	AT-hook transcription factor	Akna	0.87135	1.05940667	0.0816893
1458624_at	RNA binding motif protein 24	Rbm24	0.87135	-1.05989219	-0.0870242
1446065_at	---	---	0.87149	1.05644274	0.07576442
1422942_at	galanin receptor 2	Galr2	0.87156	1.0913185	0.11024551
1431102_at	centrosomal protein 350	Cep350	0.8716	-1.0708246	-0.1179575
1433767_at	RIKEN cDNA 1110018G07 gene	1110018G07Rik	0.87174	1.05961462	0.07828035
1416609_at	cDNA sequence BC004004	BC004004	0.87175	1.04487278	0.06326865
1457196_at	---	---	0.87176	-1.06375225	-0.0895326
1454441_at	BCL2/adenovirus E1B interacting protein 2	Bnip2	0.87177	1.06421938	0.08809931
1459057_at	---	---	0.8718	-1.05587081	-0.080186
1434288_at	basic, immunoglobulin-like variable motif containing	Bivm	0.87181	1.06633738	0.08783373
1435531_at	ubiquitin specific peptidase 3	Usp3	0.87181	1.05812889	0.07855216
1421429_a_at	nephronectin	Npnt	0.87182	1.1108844	0.12495037
1434289_at	NOL1/NOP2/Sun domain family, member 5	Nsun5	0.87184	1.04841616	0.06801293
1443699_at	protein tyrosine phosphatase, receptor type, K	Ptprk	0.87185	1.11538672	0.12412231
1456811_at	CXXC finger 4	Cxxc4	0.87185	1.07119595	0.08841161
1456826_at	TAO kinase 2	Taok2	0.87186	-1.06843973	-0.1026302
1431643_at	spermatogenesis associated glutamate (E)-rich protein 6, pseudo	Speer6-ps1	0.87188	1.04541393	0.06194833
1441892_x_at	---	---	0.87189	-1.06877856	-0.1447124
1459877_x_at	Ring finger protein 185	Rnf185	0.87189	1.04836488	0.0677372
1417555_at	ATPase family, AAA domain containing 1	Atad1	0.87192	1.0650723	0.08848679
1430935_at	RIKEN cDNA 4933413G19 gene	4933413G19Rik	0.87192	-1.06482402	-0.100679
1458308_at	strawberry notch homolog 2 (Drosophila)	Sbno2	0.87197	-1.06411101	-0.0901606
1438515_at	zinc finger protein 207	Zfp207	0.872	-1.07075624	-0.1522888
1448457_at	keratin 71	Krt71	0.87201	-1.06279886	-0.0954674
1420454_at	retinoic acid induced 1	Rai1	0.87202	1.06210782	0.08642312
1455029_at	kinesin family member 21A	Kif21a	0.87202	1.08387283	0.10344338
1429028_at	dedicator of cytokinesis 11	Dock11	0.87203	-1.04936899	-0.0711605
1431560_at	dephospho-CoA kinase domain containing	Dcakd	0.87204	1.06625641	0.09082725
1452446_a_at	transmembrane and ubiquitin-like domain containing 2	Tmub2	0.87205	1.05192017	0.07127809
1428377_at	BTB (POZ) domain containing 11	Btbd11	0.87205	-1.06439475	-0.1071006
1457369_at	---	---	0.87205	-1.06617393	-0.1047947
1422398_at	histone cluster 1, H1e	Hist1h1e	0.87206	1.06933117	0.09184447
1422636_at	cyclin D binding myb-like transcription factor 1	Dmtf1	0.87207	-1.04778958	-0.0742653
1453150_at	proteasome (prosome, macropain) subunit, beta type, 11	Psmb11	0.87207	-1.06574642	-0.0959096
1457609_at	---	---	0.87209	-1.06778114	-0.0991254
1429558_a_at	La ribonucleoprotein domain family, member 7	Larp7	0.8721	1.04921566	0.06653031
1431201_at	solute carrier family 25, member 27	Slc25a27	0.87211	1.07251523	0.09758598
1426967_at	axin 1	Axin1	0.87211	1.06377554	0.084109
1441665_at	isoleucine-tRNA synthetase 2, mitochondrial	Iars2	0.87213	1.0603727	0.08443797
1440156_s_at	TOX high mobility group box family member 2	Tox2	0.87215	-1.06554436	-0.1135775
1429566_a_at	homeodomain interacting protein kinase 2	Hipk2	0.87215	1.05942028	0.08235659
1427901_at	mitochondrial ribosomal protein S18C	Mrps18c	0.87216	-1.04601968	-0.0687313
1455808_at	RIKEN cDNA 4922502D21 gene	4922502D21Rik	0.87216	1.05310703	0.07319941
1423513_at	UHRF1 (ICBP90) binding protein 1-like	Uhrf1bp1l	0.87218	1.05607445	0.07694241
1421116_a_at	reticulon 4	Rtn4	0.87219	-1.04171478	-0.0602455
1424694_at	RIKEN cDNA 2010011I20 gene	2010011I20Rik	0.87219	-1.06163985	-0.0890951
1451704_at	androgen binding protein delta	Abpd	0.87222	-1.06225365	-0.091378
1421381_a_at	collagen, type IX, alpha 1	Col9a1	0.87224	-1.06400217	-0.1034546
1443579_s_at	DEP domain containing 6 /// hypothetical LOC100505173	Depdc6 /// LOC10050517	0.87228	-1.05412783	-0.084093
1458227_at	---	---	0.8723	1.07325761	0.09648518
1429167_at	coiled-coil domain containing 112	Ccdc112	0.87231	-1.05926026	-0.0887421
1460022_at	---	---	0.87234	-1.06250566	-0.09062
1443261_at	RIKEN cDNA E130307A14 gene	E130307A14Rik	0.87235	-1.06502007	-0.0975965
1435965_at	CCR4-NOT transcription complex, subunit 3	Cnot3	0.87235	-1.04283125	-0.063567
1457441_at	early B-cell factor 1	Ebf1	0.87237	-1.06515595	-0.0940691
1425841_at	solute carrier family 26, member 7	Slc26a7	0.87237	-1.06706033	-0.1008633
1431399_at	a disintegrin-like and metallopeptidase (reprolysin type) with thro	Adamts9	0.87238	-1.06268018	-0.0903295
1455212_at	WD repeat domain 24	Wdr24	0.87238	1.05089887	0.07043266
1425627_x_at	glutathione S-transferase, mu 1	Gstm1	0.87238	1.05421761	0.07508427
1418696_at	tetratricopeptide repeat domain 36	Ttc36	0.87238	1.07431451	0.09657232
1452312_at	RIKEN cDNA 2810002D19 gene	2810002D19Rik	0.87238	1.07104632	0.09404655
1417880_at	glucose-6-phosphatase, catalytic	G6pc	0.87241	1.06134295	0.08440426
1420583_a_at	RAR-related orphan receptor alpha	Rora	0.87241	1.11824459	0.12789417
1426565_at	insulin-like growth factor I receptor	Igf1r	0.87242	-1.06071832	-0.0920469
1437551_at	family with sequence similarity 63, member A	Fam63a	0.87242	-1.06689801	-0.1139459
1417191_at	DnaJ (Hsp40) homolog, subfamily B, member 9	Dnajb9	0.87244	-1.04600016	-0.0651976
1459014_at	Solute carrier family 1 (glial high affinity glutamate transporter), n	Slc1a2	0.87245	1.0623646	0.08689728
1427653_at	---	---	0.87247	1.1102097	0.12360327
1421601_at	GS homeobox 2	Gsx2	0.87248	-1.06762762	-0.1074153
1450563_at	microsomal glutathione S-transferase 3	Mgst3	0.87248	-1.06755157	-0.1002744
1454484_at	RIKEN cDNA 4933424L07 gene	4933424L07Rik	0.8725	-1.06590692	-0.1004293
1443663_at	---	---	0.8725	-1.06023078	-0.086342
1421525_a_at	NLR family, apoptosis inhibitory protein 5	Naip5	0.87251	1.10249829	0.11596527
1427714_at	SET and MYND domain containing 1	Smyd1	0.87251	1.0595918	0.0797448
1441581_at	ankyrin repeat and SOCS box-containing 10	Asb10	0.87252	1.07414609	0.09905633
1451625_a_at	complement component 8, gamma polypeptide	C8g	0.87256	1.06927268	0.09185657
1446378_at	---	---	0.87261	-1.06689639	-0.1034087
1447734_x_at	aldolase A, fructose-bisphosphate	Aldoa	0.87262	-1.04921043	-0.0822453
1446269_at	high mobility group box transcription factor 1	Hbp1	0.87264	-1.06516028	-0.0953857
1442852_at	---	---	0.87264	-1.06795532	-0.1019712
1440622_at	---	---	0.87265	1.05995502	0.07974979
1437467_at	activated leukocyte cell adhesion molecule	Alcam	0.87265	-1.0568067	-0.0811554

1447870_x_at	RIKEN cDNA 1110002E22 gene	1110002E22Rik	0.87265	-1.06927066	-0.146762
1458005_at	---	---	0.87265	-1.06743561	-0.1164389
1441707_at	Proteasome (prosome, macropain) subunit, alpha type 3	Psma3	0.87266	1.09625734	0.11331033
1448479_at	proteasome (prosome, macropain) 26S subunit, non-ATPase, 3	Psmd3	0.87267	1.06609555	0.08071456
1441164_at	---	---	0.87267	1.08118204	0.10203922
1455373_at	piccolo (presynaptic cytomatrix protein)	Pclo	0.87267	-1.06185737	-0.0908239
1425380_at	RAS guanyl releasing protein 4	Rasgrp4	0.87268	1.06448453	0.08756524
1458053_at	abl-interactor 2	Abi2	0.87268	-1.06028946	-0.0874013
1447149_at	solute carrier family 9 (sodium/hydrogen exchanger), member 9	Slc9a9	0.87269	1.0708666	0.09481307
1428464_at	NADH dehydrogenase (ubiquinone) 1 alpha subcomplex, 3	Ndufa3	0.87269	1.05272777	0.06899486
1450270_at	protocadherin beta 11	Pcdhb11	0.87269	-1.06264551	-0.0883069
1460678_at	kelch domain containing 2	Klhdc2	0.87269	1.05307927	0.07244974
1429892_at	PLAC8-like 1	Plac8l1	0.87269	-1.0702015	-0.1022701
1417908_s_at	ubiquitin-conjugating enzyme E2L 3	Ube2l3	0.87269	1.04524205	0.06367468
1432982_at	RIKEN cDNA 4930456J16 gene	4930456J16Rik	0.8727	-1.06033433	-0.0907856
1458230_at	---	---	0.87271	-1.06671828	-0.0974283
1435218_at	RasGEF domain family, member 1A	Rasgef1a	0.87271	1.06813068	0.09178062
1452480_at	RIKEN cDNA 4930529M08 gene	4930529M08Rik	0.87272	1.0587234	0.07961386
1417710_at	methyltransferase like 9	Mettl9	0.87273	1.04383065	0.06036593
1440636_at	---	---	0.87273	1.06361266	0.08616767
1453817_at	ATP-binding cassette, sub-family A (ABC1), member 6	Abca6	0.87273	-1.0585772	-0.0873798
1432342_at	nicotinamide nucleotide adenylyltransferase 3	Nmnat3	0.87274	1.062106	0.08663214
1437977_at	Small glutamine-rich tetratricopeptide repeat (TPR)-containing, be	Sgtb	0.87274	1.08974586	0.10652151
1428209_at	brain expressed gene 4	Bex4	0.87274	1.04404118	0.06139128
1446465_at	ATPase, H+ transporting, lysosomal V1 subunit B2	Atp6v1b2	0.87274	1.0615657	0.08511694
1436295_at	hypocretin (orexin) receptor 1	Hcrtr1	0.87276	1.06376739	0.0883769
1420326_s_at	Crm, cramped-like (Drosophila)	Cramp1l	0.87276	-1.05101624	-0.0772109
1419196_at	hepcidin antimicrobial peptide	Hamp	0.87277	1.09705157	0.11516956
1457331_at	---	---	0.87277	-1.06479632	-0.0928068
1458403_at	TRAF2 and NCK interacting kinase	Tnik	0.87277	1.05756831	0.08014222
1452369_at	membrane associated guanylate kinase, WW and PDZ domain co	Magi1	0.87278	1.06807389	0.09445368
1449010_at	heat shock protein 4 like	Hspa4l	0.87279	1.06335543	0.08804478
1453275_at	RIKEN cDNA 2310002L13 gene	2310002L13Rik	0.8728	-1.06577308	-0.1275603
1425476_at	collagen, type IV, alpha 5	Col4a5	0.8728	-1.04570969	-0.0653761
1439630_x_at	suprabasin	Sbsn	0.8728	-1.06540981	-0.1050493
1447738_s_at	ankyrin repeat domain 13 family, member D	Ankrd13d	0.87281	1.05294971	0.07381865
1429452_x_at	phosphatidylserine decarboxylase, pseudogene 3	Pisd-ps3	0.87281	-1.05895645	-0.0894923
1419101_at	transcriptional regulator, SIN3A (yeast)	Sin3a	0.87284	1.05399936	0.07042221
1447722_at	---	---	0.87284	1.06130181	0.08374162
1426543_x_at	endonuclease domain containing 1	Endod1	0.87285	-1.05994772	-0.0850121
1442488_at	---	---	0.87286	1.09203081	0.10820492
1445135_at	---	---	0.87287	-1.06187083	-0.0883294
1451148_at	PTEN induced putative kinase 1	Pink1	0.87288	-1.0559283	-0.0807747
1440986_at	RNA polymerase II associated protein 1	Rpap1	0.87289	1.09018078	0.10748612
1452935_at	predicted gene 5918	Gm5918	0.8729	1.07432319	0.09459776
1445835_at	---	---	0.87291	1.06783205	0.09407547
1416086_at	protein-tyrosine sulfotransferase 2	Tpst2	0.87298	1.0516349	0.06934893
1432204_at	RIKEN cDNA 4930570B17 gene	4930570B17Rik	0.87299	1.06491085	0.09018067
1454600_at	RIKEN cDNA 9530086P17 gene	9530086P17Rik	0.87314	1.06572071	0.09058643
1433326_at	RIKEN cDNA 4930488N24 gene	4930488N24Rik	0.87346	1.06166706	0.08632641
1434996_at	solute carrier family 25 (mitochondrial carrier, Graves disease aut	Slc25a16	0.87348	-1.05298391	-0.0788915
1446711_at	RIKEN cDNA A130040G06 gene	A130040G06Rik	0.87348	-1.0653236	-0.0991083
1428192_at	kelch repeat and BTB (POZ) domain containing 7	Kbtbd7	0.87348	-1.05306414	-0.0755485
1433548_at	nitrogen permease regulator-like 3 (S. cerevisiae)	Nprl3	0.87348	1.05617483	0.07577356
1433556_at	ArfGAP with dual PH domains 1	Adap1	0.8735	-1.04651229	-0.0673626
1436399_s_at	Nik related kinase	Nrk	0.8735	1.07793795	0.09957853
1427419_x_at	chemokine (C-C motif) receptor 9	Ccr9	0.87351	1.06842554	0.09074841
1446843_at	---	---	0.87351	-1.06788684	-0.0968962
1430686_at	RIKEN cDNA 4833418N02 gene	4833418N02Rik	0.87352	-1.06401678	-0.0908182
1422610_s_at	insulin-like growth factor 2 mRNA binding protein 3	Igf2bp3	0.87353	1.05763596	0.07870089
1446756_at	growth hormone secretagogue receptor	Ghsr	0.87353	1.06181204	0.08600675
1420512_at	dickkopf homolog 2 (Xenopus laevis)	Dkk2	0.87353	-1.06034053	-0.0945456
1446457_at	DEAD (Asp-Glu-Ala-Asp) box polypeptide 58	Ddx58	0.87353	-1.06401055	-0.0910812
1457022_at	---	---	0.87354	-1.06460718	-0.098245
1420485_at	nucleolar protein 7	Nol7	0.87354	1.06805728	0.09154578
1440826_s_at	RIKEN cDNA 2610002I17	2610002I17Rik	0.87355	1.06788493	0.0875274
1457393_at	HEAT repeat containing 6	Heatr6	0.87355	-1.06606698	-0.0961787
1418736_at	UDP-GalNAc:betaGlcNAc beta 1,3-galactosaminyltransferase, pol	B3galnt1	0.87355	-1.05113165	-0.0728039
1417023_a_at	fatty acid binding protein 4, adipocyte	Fabp4	0.87355	1.05920022	0.07894332
1430085_at	RIKEN cDNA 4933412A08 gene	4933412A08Rik	0.87356	1.05855202	0.08164162
1460429_at	cell division cycle 5-like (S. pombe)	Cdc5l	0.87356	1.03692817	0.05177253
1418243_at	ficolin A	Fcna	0.87357	1.05843723	0.08136398
1424298_at	zinc finger protein 282	Zfp282	0.87357	1.06586914	0.09036986
1450848_at	death associated protein 3	Dap3	0.87357	1.04570081	0.06252635
1452091_a_at	RNA binding motif protein 28	Rbm28	0.87357	-1.04598602	-0.0658566
1448405_a_at	EP300 interacting inhibitor of differentiation 1	Eid1	0.87358	-1.05665695	-0.0989037
1453610_at	RIKEN cDNA 4931406B18 gene	4931406B18Rik	0.87358	-1.06498521	-0.099031
1428692_at	HD domain containing 3	Hddc3	0.87359	1.06164514	0.08416929
1448711_at	minichromosome maintenance deficient 3 (S. cerevisiae) associat	Mcm3ap	0.87359	1.04674241	0.06533839
1459490_at	---	---	0.87359	1.09335283	0.11123937
1442121_at	cyclic AMP-regulated phosphoprotein, 21	Arpp21	0.8736	1.06350821	0.08399301
1444337_at	---	---	0.87361	1.08279851	0.10564559
1430891_at	Dual specificity phosphatase 12	Dusp12	0.87362	-1.06884053	-0.1136041
1416043_at	nuclear autoantigenic sperm protein (histone-binding)	Nasp	0.87362	-1.05221392	-0.0890833
1457964_at	RIKEN cDNA 1810044D09 gene	1810044D09Rik	0.87363	1.08769751	0.10401991
1446480_at	hypothetical LOC100505026	LOC100505026	0.87364	1.05481873	0.07461621
1452658_at	KRR1, small subunit (SSU) processome component, homolog (yea	Krr1	0.87365	-1.05285581	-0.0782432
1420832_at	quiescin Q6 sulfhydryl oxidase 1	Qsox1	0.87365	-1.0541495	-0.0764383
1437950_at	family with sequence similarity 149, member A	Fam149a	0.87365	-1.0456946	-0.0665014
1421962_at	DnaJ (Hsp40) homolog, subfamily B, member 5	Dnajb5	0.87365	-1.05607058	-0.0828919
1454980_at	---	---	0.87365	1.04650655	0.06552905
1426411_a_at	spermatid perinuclear RNA binding protein	Strbp	0.87366	1.06381223	0.08068496
1450599_at	vomeronasal 1 receptor 53	Vmn1r53	0.87366	-1.06332567	-0.0918608
1416678_at	COP9 (constitutive photomorphogenic) homolog, subunit 3 (Arabi	Cops3	0.87366	-1.03772621	-0.0536974

1442826_at	---	---	0.87366	-1.06236081	-0.0885687
1450112_a_at	growth arrest specific 2	Gas2	0.87367	1.09195746	0.10881379
1420009_at	WW, C2 and coiled-coil domain containing 1	Wwc1	0.87367	1.06841796	0.09074057
1427303_at	ectonucleotide pyrophosphatase/phosphodiesterase 3	Enpp3	0.87368	1.05839253	0.08028421
1426894_s_at	family with sequence similarity 102, member A	Fam102a	0.8737	1.05178852	0.07170948
1435972_at	calpastatin	Cast	0.8737	-1.06384173	-0.110521
1435085_at	cAMP responsive element binding protein-like 2	Crebl2	0.8737	-1.0683918	-0.1227669
1448607_at	nicotinamide phosphoribosyltransferase	Nampt	0.87371	1.07920234	0.09603481
1443908_at	hypothetical LOC100503447	LOC100503447	0.87371	1.06763783	0.09148773
1434598_at	La ribonucleoprotein domain family, member 4B	Larp4b	0.87372	-1.04295727	-0.0619054
1421592_at	neural cell adhesion molecule 2	Ncam2	0.87372	1.07159178	0.09650195
1420696_at	sema domain, immunoglobulin domain (Ig), short basic domain, s	Sema3c	0.87373	1.07453245	0.09681378
1433045_at	RIKEN cDNA 4930535F04 gene	4930535F04Rik	0.87373	1.10008418	0.11514017
1416090_at	pyruvate dehydrogenase (lipoamide) beta	Pdhb	0.87373	1.04063583	0.05702767
1423283_at	phosphatidylinositol transfer protein, alpha	Pitpna	0.87374	1.04902049	0.06637737
1446238_at	RIKEN cDNA 4930431P03 gene	4930431P03Rik	0.87374	-1.06918794	-0.1197129
1458164_at	hypothetical LOC552913	LOC552913	0.87375	-1.06669628	-0.1094476
1422230_s_at	cytochrome P450, family 2, subfamily a, polypeptide 4 /// cytoch	Cyp2a4 /// Cyp2a5	0.87375	1.07315257	0.09469526
1444624_at	ubiquitin-associated protein 1	Ubap1	0.87375	-1.06442376	-0.0954423
1454806_at	family with sequence similarity 49, member A	Fam49a	0.87375	1.06275542	0.08654184
1452092_at	carbohydrate (N-acetylgalactosamine 4-sulfate 6-O) sulfotransfe	Chst15	0.87375	1.08757991	0.10674516
1457806_at	Family with sequence similarity 196, member A	Fam196a	0.87375	-1.06183545	-0.0879257
1442597_at	expressed sequence C79122	C79122	0.87375	1.06959962	0.09497144
1451128_s_at	kinesin family member 22	Kif22	0.87377	1.04899394	0.06648919
1447573_at	Solute carrier family 32 (GABA vesicular transporter), member 1	Slc32a1	0.87377	-1.06925966	-0.1261769
1445138_at	RIKEN cDNA A530083I20 gene	A530083I20Rik	0.87377	-1.0682631	-0.1044053
1444660_at	RIKEN cDNA 4932438A13 gene	4932438A13Rik	0.87378	1.07867152	0.10288609
1426278_at	interferon, alpha-inducible protein 27 like 2A	Ifi27l2a	0.87378	1.07221036	0.09406091
1442587_at	---	---	0.87378	1.0845526	0.10263796
1420458_at	tachykinin 4	Tac4	0.87379	1.06532708	0.08914667
1446745_at	predicted gene 5547	Gm5547	0.87379	-1.05891592	-0.0832872
1427489_at	integrin alpha 8	Itga8	0.87379	1.0625564	0.08672257
1437681_at	glutamate receptor, ionotropic, kainate 4	Grik4	0.87379	1.06462439	0.08720852
1415744_at	H2-K region expressed gene 2	H2-Ke2	0.8738	1.0437316	0.06124819
1427565_a_at	ATP-binding cassette, sub-family C (CFTR/MRP), member 5	Abcc5	0.8738	1.04943697	0.06835575
1447108_at	---	---	0.87381	-1.06731148	-0.10614
1421277_at	spectrin alpha 1	Spna1	0.87381	1.1179626	0.1303037
1444105_at	actin, alpha 2, smooth muscle, aorta	Acta2	0.87381	1.0788093	0.09569957
1437204_a_at	transcription factor 25 (basic helix-loop-helix)	Tcf25	0.87381	-1.0615852	-0.0878024
1451987_at	arrestin, beta 2	Arrb2	0.87381	1.08373465	0.09593636
1441505_at	---	---	0.87382	-1.05744236	-0.080762
1445607_at	DDB1 and CUL4 associated factor 17	Dcaf17	0.87382	1.06628747	0.09166054
1460322_at	carbohydrate (chondroitin 6/keratan) sulfotransferase 3	Chst3	0.87382	1.06856755	0.0928502
1426536_at	NMDA receptor-regulated gene 2	Narg2	0.87383	-1.05317257	-0.084316
1441112_at	zinc finger, BED domain containing 6	Zbed6	0.87383	-1.06645391	-0.1088053
1422761_at	transducin (beta)-like 1X-linked receptor 1	Tbl1xr1	0.87385	-1.06335856	-0.0944515
1421198_at	integrin alpha V	Itgav	0.87387	1.1253842	0.13188858
1420982_at	RNA binding motif protein 39	Rbm39	0.87391	-1.05937566	-0.0996869
1456978_s_at	DNA segment, Chr 19, ERATO Doi 652, expressed	D19Ertd652e	0.87391	1.06235744	0.08474986
1457682_at	Rho GTPase activating protein 42	Arhgap42	0.87393	-1.0549418	-0.0774556
1446516_at	B-cell CLL/lymphoma 7C /// hypothetical LOC100502737	Bcl7c /// LOC100502737	0.87394	1.06909223	0.09320357
1418919_at	shugoshin-like 1 (S. pombe)	Sgol1	0.87394	1.04246672	0.05989088
1449193_at	CD5 antigen-like	Cd5l	0.87394	-1.06527529	-0.0930374
1453044_at	RIKEN cDNA C030014I23 gene /// hypothetical LOC100504832	C030014I23Rik /// LOC10	0.87395	-1.05555025	-0.0795314
1424833_at	inositol 1,4,5-triphosphate receptor 2	Itpr2	0.87395	-1.06569225	-0.1160562
1439346_at	G protein-coupled receptor 135	Gpr135	0.87396	-1.06601142	-0.098823
1433296_at	RIKEN cDNA 2310045N14 gene	2310045N14Rik	0.87397	-1.05667864	-0.0809772
1458437_at	---	---	0.87398	1.07673311	0.09958364
1453144_at	phosphatidylserine decarboxylase, pseudogene 3	Pisd-ps3	0.87398	1.06225404	0.08583107
1430183_at	---	---	0.87399	-1.06776175	-0.1110471
1445999_at	DNA segment, Chr 19, ERATO Doi 132, expressed	D19Ertd132e	0.87399	-1.06414552	-0.1009337
1423077_at	sorting nexin 9	Snx9	0.87399	1.06922188	0.08587367
1434665_at	aspartylglucosaminidase	Aga	0.874	-1.05190988	-0.0785295
1447671_x_at	F-box protein 6	Fbxo6	0.874	1.06608485	0.08932048
1439815_at	HEAT repeat containing 5B	Heatr5b	0.87402	1.05852363	0.07685694
1418175_at	vitamin D receptor	Vdr	0.87403	1.1057204	0.12064082
1430989_a_at	RIKEN cDNA 1700020I14 gene	1700020I14Rik	0.87403	-1.05774953	-0.0829259
1454095_at	RIKEN cDNA 4930579H20 gene	4930579H20Rik	0.87404	1.04604984	0.06416272
1439310_at	RIKEN cDNA A630012P03 gene /// hypothetical protein LOC1005	A630012P03Rik /// LOC10	0.87407	-1.05197707	-0.0760711
1432893_at	RIKEN cDNA 9130011L11 gene	9130011L11Rik	0.87407	-1.06686805	-0.0943701
1459484_at	---	---	0.87407	-1.06248158	-0.088197
1441815_at	---	---	0.87408	-1.05785339	-0.0835756
1415783_at	vacuolar protein sorting 35	Vps35	0.87409	-1.04052782	-0.057686
1415780_a_at	armadillo repeat containing, X-linked 2	Armcx2	0.87409	1.04841876	0.06677966
1423531_a_at	heterogeneous nuclear ribonucleoprotein A1	Hnrnpa1	0.87409	-1.04173228	-0.0612285
1429160_at	mitochondrial translational initiation factor 3	Mtif3	0.8741	-1.05745431	-0.0811723
1452611_at	listerin E3 ubiquitin protein ligase 1	Ltn1	0.8741	1.06019715	0.07987579
1418165_at	intelectin 1 (galactofuranose binding)	Itln1	0.87411	1.06698224	0.08420085
1455868_a_at	tubulin, gamma complex associated protein 2	Tubgcp2	0.87412	1.04888492	0.06543643
1422996_at	acyl-CoA thioesterase 2	Acot2	0.87412	-1.06204719	-0.0932103
1459758_at	upstream transcription factor 1	Usf1	0.87412	1.07166175	0.09403255
1422916_at	fibroblast growth factor 21	Fgf21	0.87414	-1.06428055	-0.0936262
1457262_at	SMG1 homolog, phosphatidylinositol 3-kinase-related kinase (C.	Smg1	0.87415	-1.05380095	-0.1791662
1416605_at	NHP2 ribonucleoprotein homolog (yeast)	Nhp2	0.87415	1.05473334	0.06845003
1416838_at	methylmalonyl-Coenzyme A mutase	Mut	0.87416	1.04612742	0.06449194
1458968_at	expressed sequence C80283	C80283	0.87416	-1.06778379	-0.1257188
1425942_a_at	glycoprotein m6b	Gpm6b	0.87417	-1.06607764	-0.1029013
1431292_a_at	twinfilin, actin-binding protein, homolog 2 (Drosophila)	Twf2	0.87417	1.08490779	0.09582734
1448573_a_at	carcinoembryonic antigen-related cell adhesion molecule 10	Ceacam10	0.87417	-1.05770045	-0.0816678
1430911_at	DNA segment, Chr 19, ERATO Doi 652, expressed	D19Ertd652e	0.87419	-1.06234067	-0.1078026
1439282_at	RIKEN cDNA D930023I05 gene	D930023I05Rik	0.87419	-1.05966529	-0.0875557
1422707_at	phosphoinositide-3-kinase, catalytic, gamma polypeptide	Pik3cg	0.8742	-1.07018789	-0.1116047
1417519_at	pleiomorphic adenoma gene-like 2	Plagl2	0.8742	-1.05924334	-0.0838112
1446951_at	procollagen-proline, 2-oxoglutarate 4-dioxygenase (proline 4-hyd	P4ha3	0.8742	-1.0615923	-0.0953579

1420916_at	PRP40 pre-mRNA processing factor 40 homolog A (yeast)	Prpf40a	0.87421	-1.04892303	-0.079975
1427950_at	listerin E3 ubiquitin protein ligase 1	Ltn1	0.87421	-1.05526495	-0.0804664
1442623_at	Myocyte enhancer factor 2A	Mef2a	0.87421	-1.06450839	-0.095708
1443437_at	---	---	0.87422	-1.0648034	-0.0947069
1459771_x_at	alkaline ceramidase 3	Acer3	0.87423	-1.06307196	-0.0941997
1441729_at	LIM homeobox transcription factor 1 beta	Lmx1b	0.87423	-1.06298456	-0.088727
1459284_at	---	---	0.87424	-1.06407244	-0.0943172
1433828_at	ATPase, aminophospholipid transporter (APLT), class I, type 8A, m	Atp8a1	0.87424	-1.06058009	-0.089854
1432162_s_at	protein prenyltransferase alpha subunit repeat containing 1	Ptar1	0.87425	1.06675568	0.09141842
1420207_at	---	---	0.87426	-1.04681334	-0.0709503
1415809_at	trophoblast specific protein alpha	Tpbpa	0.87426	1.06037329	0.08431579
1447814_x_at	interleukin 16	Il16	0.87426	-1.06547455	-0.0973643
1444354_at	---	---	0.87426	-1.0656675	-0.1027541
1458683_at	signal-regulatory protein beta 1A	Sirpb1a	0.87427	1.06191987	0.08550023
1430212_at	zinc finger protein 712	Zfp712	0.87427	1.06693227	0.09165331
1444708_at	transmembrane protein 29	Tmem29	0.8743	1.04385152	0.06075728
1428146_s_at	acetyl-Coenzyme A acyltransferase 2 (mitochondrial 3-oxoacyl-Co	Acaa2	0.8743	1.05921758	0.07751409
1460161_at	DNA segment, Chr 5, ERATO Doi 422, expressed	D5Ertd422e	0.87449	-1.06036889	-0.0935962
1434648_a_at	cerebral cavernous malformation 2 homolog (human)	Ccm2	0.87451	-1.05515341	-0.0982685
1430316_at	isoprenoid synthase domain containing	Ispd	0.87452	1.06323288	0.08776244
1453730_at	sterile alpha motif domain containing 8	Samd8	0.87452	-1.06606302	-0.0942391
1438122_at	RIKEN cDNA 2900006K08 gene	2900006K08Rik	0.87453	1.07231986	0.09457891
1419406_a_at	B-cell CLL/lymphoma 11A (zinc finger protein)	Bcl11a	0.87454	1.06532996	0.09002475
1446836_at	---	---	0.87454	1.06213394	0.08420178
1457460_at	acyl-CoA synthetase short-chain family member 2	Acss2	0.87454	-1.06191586	-0.0876532
1453639_s_at	coiled-coil domain containing 163	Ccdc163	0.87456	-1.06351459	-0.0951613
1446583_at	---	---	0.87456	-1.06752878	-0.1058056
1451448_a_at	RIKEN cDNA 1110005A03 gene	1110005A03Rik	0.87456	1.06228907	0.08146777
1447798_at	RIKEN cDNA 1700123O21 gene	1700123O21Rik	0.87456	1.04748192	0.06646623
1416200_at	interleukin 33	Il33	0.87457	1.0607757	0.08391942
1453662_at	hypothetical LOC77413	LOC77413	0.87457	-1.06893176	-0.1170027
1449376_at	nicolin 1	Nicn1	0.87457	-1.0547405	-0.0791615
1457855_at	retinol binding protein 3, interstitial	Rbp3	0.87457	-1.0625936	-0.1222866
1419452_at	ubiquitin carboxyl-terminal esterase L5	Uchl5	0.87457	-1.04090055	-0.0582608
1448037_at	Zinc finger, CCHC domain containing 6	Zcchc6	0.87457	1.06306061	0.08647975
1420137_at	---	---	0.87458	-1.04867849	-0.0728648
1418921_at	cell adhesion molecule 3	Cadm3	0.87458	1.07685356	0.0973361
1448009_at	UDP-glucose glycoprotein glucosyltransferase 1	Uggt1	0.87459	1.05749325	0.07842321
1424247_at	ELKS/RAB6-interacting/CAST family member 1	Erc1	0.87459	1.04953916	0.06914607
1444978_at	---	---	0.8746	-1.05042549	-0.0749664
1442021_at	guanine nucleotide binding protein, alpha stimulating, olfactory ty	Gnal	0.87461	-1.0555131	-0.0810171
1447515_at	---	---	0.87462	1.06639369	0.09120114
1458219_at	breast carcinoma amplified sequence 2	Bcas2	0.87462	1.05673283	0.07743356
1449749_s_at	transcription factor B1, mitochondrial	Tfb1m	0.87462	1.05849104	0.07788243
1447971_at	RIKEN cDNA A230061C15 gene	A230061C15Rik	0.87464	1.06596053	0.08881961
1439753_x_at	sine oculis-related homeobox 4 homolog (Drosophila)	Six4	0.87465	-1.05990764	-0.1029204
1459593_x_at	protein phosphatase 1, regulatory (inhibitor) subunit 13 like	Ppp1r13l	0.87465	1.04644693	0.06492965
1433542_at	inositol polyphosphate-5-phosphatase F	Inpp5f	0.87466	1.06668138	0.08371741
1427340_at	regulator of G-protein signaling 6	Rgs6	0.87466	-1.05950977	-0.0859855
1444455_at	calcium channel, voltage-dependent, P/Q type, alpha 1A subunit	Cacna1a	0.87467	-1.06333041	-0.0956167
1418669_at	perlecan (heparan sulfate proteoglycan 2)	Hspg2	0.87467	-1.05537675	-0.1051078
1442866_at	RCSD domain containing 1	Rcsd1	0.87467	-1.06737319	-0.1021061
1448008_at	ankyrin repeat and KH domain containing 1	Ankhd1	0.87468	1.06294997	0.08542046
1427715_a_at	5'-nucleotidase, cytosolic IB	Nt5c1b	0.87468	-1.06459156	-0.1075805
1417307_at	dystrophin, muscular dystrophy	Dmd	0.87468	1.07855289	0.09213524
1448095_at	---	---	0.87468	-1.06252818	-0.1028174
1451240_a_at	glyoxalase 1	Glo1	0.87469	-1.04728846	-0.076791
1440631_at	---	---	0.87469	1.07513538	0.09838805
1442829_at	---	---	0.87469	1.06933926	0.09070063
1419501_at	polymerase (DNA directed), kappa	Polk	0.87469	-1.06569016	-0.103167
1456024_at	general transcription factor III C 1	Gtf3c1	0.8747	1.05574629	0.07790998
1451964_at	melanoma inhibitory activity 2	Mia2	0.8747	1.07257609	0.09407606
1439219_at	RIKEN cDNA A730082K24 gene	A730082K24Rik	0.87471	1.09740409	0.11191556
1427356_at	family with sequence similarity 89, member A	Fam89a	0.87471	1.06651639	0.09135608
1427115_at	myosin, heavy polypeptide 3, skeletal muscle, embryonic	Myh3	0.87472	-1.06494783	-0.1083495
1425216_at	free fatty acid receptor 2	Ffar2	0.87472	1.07342268	0.09716459
1456347_at	---	---	0.87472	1.11149513	0.12504072
1442729_at	---	---	0.87472	1.06516181	0.08862768
1433062_at	WD repeat domain 66	Wdr66	0.87472	1.06679125	0.09262639
1433635_at	WD repeat domain 18	Wdr18	0.87473	1.04992388	0.06674355
1433388_at	RIKEN cDNA 2900022M07 gene	2900022M07Rik	0.87473	1.07553081	0.09548566
1426310_at	zinc finger, DHHC domain containing 5	Zdhhc5	0.87473	1.051125	0.07033721
1449446_at	coiled-coil domain containing 59	Ccdc59	0.87474	-1.0509373	-0.0723353
1452878_at	protein kinase C, epsilon	Prkce	0.87474	-1.06422785	-0.1038267
1460072_at	---	---	0.87475	1.06298228	0.08793183
1452255_at	F-box protein 38	Fbxo38	0.87475	1.04776404	0.06644427
1438704_at	tripartite motif-containing 63	Trim63	0.87476	1.0638098	0.08361359
1441949_x_at	solute carrier family 39 (metal ion transporter), member 6	Slc39a6	0.87476	-1.06116933	-0.0991084
1432416_a_at	nucleophosmin 1	Npm1	0.87476	-1.02332916	-0.0340284
1444981_at	Immunoglobulin superfamily, DCC subclass, member 3	Igdcc3	0.87476	-1.06667346	-0.1060403
1424012_at	tetratricopeptide repeat domain 30A1	Ttc30a1	0.87476	-1.04601198	-0.0650655
1440372_at	---	---	0.87476	1.15390449	0.14872046
1458962_at	transmembrane emp24 protein transport domain containing 5	Tmed5	0.87477	1.06477078	0.08132816
1456283_at	neuropilin (NRP) and tolloid (TLL)-like 1	Neto1	0.87477	-1.06277567	-0.0964744
1421765_at	paired box gene 5	Pax5	0.87477	1.10885889	0.12317724
1426737_at	G1 to S phase transition 1	Gspt1	0.87477	-1.05543751	-0.0837105
1428890_at	fem-1 homolog c (C.elegans)	Fem1c	0.87478	-1.04371915	-0.0619421
1422869_at	c-mer proto-oncogene tyrosine kinase	Mertk	0.87478	1.067625	0.08881948
1456099_at	RIKEN cDNA D930017J03 gene	D930017J03Rik	0.87478	-1.06858289	-0.0964594
1456580_s_at	ATP synthase, H+ transporting, mitochondrial F1 complex, delta su	Atp5d	0.87478	1.0401416	0.05508378
1417378_at	cell adhesion molecule 1	Cadm1	0.87478	-1.04942792	-0.0728677
1451503_at	nucleolar protein 3 (apoptosis repressor with CARD domain)	Nol3	0.87478	1.06085285	0.08507428
1434818_at	hypothetical protein 9330159K06	9330159K06	0.87479	1.06812688	0.08999253
1440898_at	family with sequence similarity 179, member A	Fam179a	0.87479	-1.05317099	-0.0767547

1460297_at	nephrosis 2 homolog, podocin (human)	Nphs2	0.87479	-1.06059631	-0.0866774
1454632_at	transmembrane protein 229B	Tmem229b	0.87479	-1.0557005	-0.0800494
1434989_at	unc-80 homolog (C. elegans)	Unc80	0.8748	-1.06658929	-0.1132697
1438729_at	SRY-box containing gene 1	Sox1	0.8748	-1.06489918	-0.1106582
1432876_at	RIKEN cDNA 4632418H02 gene	4632418H02Rik	0.8748	-1.06642504	-0.1105879
1447663_at	---	---	0.8748	1.06807858	0.09233678
1426341_at	solute carrier family 1 (glial high affinity glutamate transporter), m	Slc1a3	0.8748	-1.06446927	-0.0949162
1440143_at	phosphatidylinositol glycan anchor biosynthesis, class Z	Pigz	0.87481	-1.06219222	-0.0905136
1451162_at	heat shock factor binding protein 1	Hsbp1	0.87481	1.04518407	0.0625853
1430124_x_at	aldo-keto reductase family 1, member A4 (aldehyde reductase)	Akr1a4	0.87481	1.04212124	0.05892987
1459948_at	---	---	0.87482	1.08115526	0.09866785
1442898_at	---	---	0.87482	1.06857813	0.09453698
1457807_at	Glucosamine-phosphate N-acetyltransferase 1	Gnpnat1	0.87482	1.09285534	0.11110098
1442963_at	Phospholipase C, gamma 1	Plcg1	0.87483	-1.06225688	-0.0947643
1449605_at	---	---	0.87483	1.09134049	0.11028285
1447642_x_at	dystrophia myotonica-containing WD repeat motif	Dmwd	0.87483	-1.06771254	-0.1067962
1446085_at	---	---	0.87483	-1.06207522	-0.1024323
1426561_a_at	nephronectin	Npnt	0.87483	1.07335782	0.09587331
1417202_s_at	ubiquitin-like modifier activating enzyme 3	Uba3	0.87483	-1.04550443	-0.0699461
1426319_at	platelet-derived growth factor, D polypeptide	Pdgfd	0.87483	1.06280769	0.0806323
1441022_at	ariadne ubiquitin-conjugating enzyme E2 binding protein homolog	Arih1	0.87485	1.07178274	0.09391037
1424283_at	jumping translocation breakpoint	Jtb	0.87485	1.04709631	0.06505317
1434661_at	synaptogyrin 1	Syngr1	0.87485	-1.05378615	-0.0767821
1441589_at	---	---	0.87485	1.06972355	0.08740755
1442388_at	RIKEN cDNA 2410137F16 gene	2410137F16Rik	0.87486	-1.06306301	-0.0926063
1430785_at	4short chain dehydrogenase/reductase family 9C, member 7	Sdr9c7	0.87486	1.06445907	0.08963835
1459878_a_at	RIKEN cDNA A430107O13 gene	A430107O13Rik	0.87487	-1.06064757	-0.0908182
1438859_x_at	ribosomal protein S29	Rps29	0.87488	-1.02514722	-0.0385544
1435151_a_at	ribosomal protein S3	Rps3	0.87488	-1.02440239	-0.0360321
1420601_at	glial cells missing homolog 1 (Drosophila)	Gcm1	0.87489	-1.06955216	-0.1174165
1422792_at	platelet-activating factor acetylhydrolase, isoform 1b, subunit 2	Pafah1b2	0.87489	1.0482073	0.06645867
1444154_at	NADH dehydrogenase (ubiquinone) 1 alpha subcomplex 11	Ndufa11	0.8749	-1.06041019	-0.0912284
1424836_a_at	CLIP associating protein 2	Clasp2	0.87491	1.04814241	0.06487735
1437219_at	---	---	0.87491	1.07324594	0.09434662
1439584_at	Zinc finger protein 526	Zfp526	0.87491	1.0751784	0.09701653
1420204_at	---	---	0.87492	-1.06457009	-0.0921427
1443116_at	proteasome (prosome, macropain) activator subunit 4	Psme4	0.87492	1.07215637	0.09412367
1446969_at	---	---	0.87492	1.12403533	0.13142678
1444268_at	cDNA sequence BC004004	BC004004	0.87493	1.07059202	0.09385774
1432700_at	RIKEN cDNA 2810047J09 gene	2810047J09Rik	0.87495	1.05796341	0.077829
1458893_at	---	---	0.87497	-1.06740999	-0.1222842
1438150_at	farnesyl diphosphate synthetase	Fdps	0.875	1.0629047	0.08204908
1433495_at	glycosyltransferase 25 domain containing 1	Glt25d1	0.87502	1.04278341	0.06015763
1433234_at	RIKEN cDNA 4930424E08 gene	4930424E08Rik	0.87522	-1.06491075	-0.0942097
1451797_at	Smg-5 homolog, nonsense mediated mRNA decay factor (C. eleg	Smg5	0.87525	-1.05762116	-0.0849015
1425930_a_at	MAX-like protein X	Mlx	0.87525	1.05583859	0.07287309
1442835_at	hypothetical LOC100503961	LOC100503961	0.87525	-1.06379015	-0.1003123
1449242_s_at	histidine-rich glycoprotein	Hrg	0.87525	1.05667867	0.07869724
1449642_at	---	---	0.87527	1.05866114	0.07753112
1441293_at	---	---	0.87529	-1.06355998	-0.1091578
1455165_at	RAR-related orphan receptor alpha	Rora	0.87531	-1.06072184	-0.0853882
1426664_x_at	solute carrier family 45, member 3	Slc45a3	0.87542	1.06222723	0.08417158
1421453_at	junctophilin 2	Jph2	0.87546	-1.06438395	-0.1012158
1448699_at	mitochondrial ribosomal protein L54	Mrpl54	0.87548	1.08356714	0.0939047
1458527_at	---	---	0.87549	1.10926157	0.12266823
1430975_at	RIKEN cDNA 8430416G17 gene	8430416G17Rik	0.87552	1.07431456	0.09629048
1443264_at	membrane-spanning 4-domains, subfamily A, member 2	Ms4a2	0.87552	-1.05894768	-0.0834461
1431585_at	RIKEN cDNA 1700034P13 gene	1700034P13Rik	0.87553	-1.06511499	-0.0998765
1454100_at	RIKEN cDNA 4930480E11 gene	4930480E11Rik	0.87553	1.07119476	0.09180827
1437325_x_at	aldehyde dehydrogenase 18 family, member A1	Aldh18a1	0.87554	-1.0471297	-0.0751879
1427719_s_at	keratin complex 2, basic, pseudogene 1 /// keratin 82	Krt2-ps1 /// Krt82	0.87554	1.05754833	0.08067516
1424034_at	RAR-related orphan receptor alpha	Rora	0.87554	1.0747588	0.0981326
1421970_a_at	glutamate receptor, ionotropic, AMPA2 (alpha 2)	Gria2	0.87555	1.06930406	0.09512495
1438997_at	---	---	0.87555	-1.06480504	-0.1059032
1426544_a_at	tetratricopeptide repeat domain 14	Ttc14	0.87555	-1.05050749	-0.0730132
1453396_at	WD repeat domain 38	Wdr38	0.87556	1.07611343	0.09802219
1448845_at	ribonuclease P 25 subunit (human)	Rpp25	0.87556	1.04919524	0.06571795
1429586_at	RIKEN cDNA 4930558N01 gene	4930558N01Rik	0.87557	1.09756224	0.11268296
1452192_at	nuclear assembly factor 1 homolog (S. cerevisiae)	Naf1	0.87558	-1.04406295	-0.0652589
1418785_at	mitogen-activated protein kinase 8 interacting protein 2	Mapk8ip2	0.87559	-1.05658633	-0.0895709
1434201_at	chordin-like 1	Chrdl1	0.87559	1.04970923	0.0691413
1452111_at	mitochondrial ribosomal protein S35	Mrps35	0.87559	1.0505	0.0671614
1424161_at	DEAD (Asp-Glu-Ala-Asp) box polypeptide 27	Ddx27	0.8756	1.04616081	0.06375792
1437036_at	Staphylococcal nuclease and tudor domain containing 1	Snd1	0.87561	-1.05632005	-0.0903199
1444032_at	---	---	0.87562	1.07092562	0.09296079
1450255_at	Rho GTPase activating protein 31	Arhgap31	0.87563	-1.06472776	-0.0904976
1433228_at	RIKEN cDNA 9430081G06 gene	9430081G06Rik	0.87569	-1.06610808	-0.0966899
1426578_s_at	SNAP-associated protein	Snapin	0.87571	-1.05076149	-0.0775067
1415824_at	stearoyl-Coenzyme A desaturase 2	Scd2	0.87572	1.05845018	0.07659892
1427741_x_at	NK2 transcription factor related, locus 3 (Drosophila)	Nkx2-3	0.87576	1.07746585	0.09985064
1416703_at	mitogen-activated protein kinase 14	Mapk14	0.8758	1.04473441	0.06158236
1458355_x_at	WD repeat domain 92	Wdr92	0.87582	-1.05084588	-0.0746949
1459772_at	Hermansky-Pudlak syndrome 3 homolog (human)	Hps3	0.87598	1.06301491	0.08705136
1426625_at	zinc finger protein 623	Zfp623	0.87599	1.04830066	0.06722505
1430163_at	RAB43, member RAS oncogene family	Rab43	0.8761	1.06465765	0.09006057
1442089_at	predicted gene 11563	Gm11563	0.87611	-1.05719825	-0.0805074
1443644_at	---	---	0.87612	-1.05470083	-0.0826718
1458608_at	---	---	0.87613	1.06633702	0.08894115
1428451_at	leucine rich repeat containing 47	Lrrc47	0.87613	1.04142866	0.05835619
1460386_a_at	solute carrier family 1 (neuronal/epithelial high affinity glutamate	Slc1a1	0.87613	1.07722562	0.09897189
1452993_at	RIKEN cDNA 5430416O09 gene	5430416O09Rik	0.87615	-1.06128602	-0.0862444
1443754_x_at	---	---	0.87616	-1.06403814	-0.09217
1448286_at	hydroxysteroid (17-beta) dehydrogenase 10	Hsd17b10	0.87617	1.05036634	0.06723626
1438516_at	Rap1 interacting factor 1 homolog (yeast)	Rif1	0.87618	-1.05416927	-0.0835308

1440491_at	solute carrier family 1 (glial high affinity glutamate transporter), m	Slc1a3	0.87618	-1.06146868	-0.0897194
1455147_at	---	---	0.87619	1.07303497	0.08932361
1423230_at	inositol polyphosphate-5-phosphatase E	Inpp5e	0.87619	-1.0604003	-0.0852369
1452592_at	microsomal glutathione S-transferase 2	Mgst2	0.87619	1.07358091	0.09829161
1435845_at	---	---	0.8762	1.06671958	0.08813705
1426553_at	DnaJ (Hsp40) homolog, subfamily C, member 14	Dnajc14	0.8762	1.06603467	0.08408197
1425963_at	calcium binding protein 7	Cabp7	0.8762	1.07499851	0.09627126
1427285_s_at	metastasis associated lung adenocarcinoma transcript 1 (non-cod	Malat1	0.87621	1.06984528	0.09084963
1452372_at	BSD domain containing 1	Bsdc1	0.87621	1.05361485	0.07320365
1425529_s_at	DNA segment, Chr 19, Wayne State University 162, expressed	D19Wsu162e	0.87622	1.07051796	0.08633246
1444643_at	---	---	0.87622	-1.06486255	-0.0989483
1430182_a_at	RIKEN cDNA 1700006J14 gene	1700006J14Rik	0.87622	1.07175864	0.09349103
1457782_at	talin 1	Tln1	0.87623	-1.06422462	-0.095601
1428371_at	tau tubulin kinase 2	Ttbk2	0.87624	1.07948763	0.09946171
1418220_at	forkhead box F2	Foxf2	0.87624	-1.06565156	-0.0969396
1429158_at	F-box protein 28	Fbxo28	0.87625	1.04400216	0.06175679
1436360_at	zinc finger and SCAN domain containing 22	Zscan22	0.87625	1.06144859	0.08102436
1435935_at	RIKEN cDNA 2410131K14 gene	2410131K14Rik	0.87626	1.06198099	0.08593369
1454081_at	---	---	0.87628	-1.06351193	-0.0945658
1455125_at	ArfGAP with coiled-coil, ankyrin repeat and PH domains 1	Acap1	0.87628	1.06247859	0.08536501
1422448_at	trefoil factor 2 (spasmolytic protein 1)	Tff2	0.8763	-1.06048868	-0.0855048
1439321_at	---	---	0.8763	-1.05931397	-0.0846061
1424305_at	immunoglobulin joining chain	Igj	0.8763	1.06483727	0.08981511
1455024_at	tousled-like kinase 1	Tlk1	0.8763	-1.04253202	-0.0603636
1422058_at	nodal	Nodal	0.87631	-1.04235189	-0.0629128
1447405_at	Predicted gene 941	Gm941	0.87631	1.05904922	0.08252075
1424679_at	mab-21-like 1 (C. elegans)	Mab21l1	0.87631	-1.06150292	-0.1011738
1449787_at	---	---	0.87633	-1.05693185	-0.0801148
1432337_at	Fanconi anemia, complementation group L	Fancl	0.87633	-1.06102508	-0.0947342
1459324_at	Fermitin family homolog 2 (Drosophila)	Fermt2	0.87634	1.0857337	0.10539255
1458941_at	zinc finger E-box binding homeobox 2	Zeb2	0.87635	-1.068653	-0.0968582
1417033_at	ubiquitin-conjugating enzyme E2G 2	Ube2g2	0.87636	1.06240346	0.08200476
1435398_at	syntaxin binding protein 5 (tomosyn)	Stxbp5	0.87637	1.06179484	0.08613991
1430058_at	stem-loop binding protein	Slbp	0.87639	1.10958894	0.12182791
1416604_at	cytochrome c-1	Cyc1	0.87639	1.03600412	0.04930551
1420257_at	ribosomal protein L7 pseudogene	1700073E17Rik	0.8764	-1.06666292	-0.0985509
1438503_x_at	endothelial differentiation-related factor 1	Edf1	0.87641	-1.04580223	-0.0732601
1445673_at	RIKEN cDNA 2900052N01 gene	2900052N01Rik	0.87642	1.06017512	0.08370831
1420829_a_at	14-3-3 protein theta-like /// tyrosine 3-monooxygenase/tryptoph	LOC100503129 /// Ywhaq	0.87642	1.03898249	0.05396271
1442001_at	protein kinase, AMP-activated, beta 2 non-catalytic subunit	Prkab2	0.87644	1.0624856	0.08565187
1457240_at	cyclin-dependent kinase 9 (CDC2-related kinase)	Cdk9	0.87646	1.06540483	0.09003641
1455590_at	NAD(P)H dehydrogenase, quinone 2	Nqo2	0.87657	1.06872659	0.08696116
1444494_at	kelch repeat and BTB (POZ) domain containing 10	Kbtbd10	0.8766	-1.05931706	-0.095892
1438137_at	---	---	0.87662	1.08261799	0.10355109
1430571_s_at	armadillo repeat containing 6	Armc6	0.87667	1.06914568	0.08589451
1447841_x_at	ST3 beta-galactoside alpha-2,3-sialyltransferase 6	St3gal6	0.8767	-1.0523128	-0.081333
1419815_at	methyltransferase 11 domain containing 1	Mett11d1	0.8767	1.05674859	0.077123
1451614_a_at	amelogenin X chromosome	Amelx	0.87674	1.06777039	0.09057413
1423874_at	WD repeat domain 33	Wdr33	0.87679	-1.06381635	-0.0929033
1423702_at	H1 histone family, member 0	H1f0	0.87684	1.04860737	0.06506615
1441688_at	RIKEN cDNA 6430537I21 gene	6430537I21Rik	0.87688	-1.06475667	-0.0980023
1457266_at	solute carrier family 38, member 6	Slc38a6	0.87691	1.0519803	0.07264393
1447697_at	---	---	0.87695	-1.06138352	-0.0862713
1426102_at	cytochrome P450, family 2, subfamily j, polypeptide 13	Cyp2j13	0.87697	1.08511458	0.10458984
1419207_at	zinc finger protein 37	Zfp37	0.87697	1.06242931	0.08121683
1446280_at	---	---	0.87698	-1.06085786	-0.0858528
1422106_a_at	splA/ryanodine receptor domain and SOCS box containing 2	Spsb2	0.87698	1.05254526	0.07358587
1418938_at	deiodinase, iodothyronine, type II	Dio2	0.87698	-1.0609605	-0.0872072
1418295_s_at	diacylglycerol O-acyltransferase 1	Dgat1	0.87706	1.04794544	0.06581012
1420342_at	ganglioside-induced differentiation-associated-protein 10	Gdap10	0.87708	1.09813942	0.1075217
1459445_at	---	---	0.87711	1.06850235	0.09130286
1432665_at	RIKEN cDNA 2210416J07 gene	2210416J07Rik	0.87712	1.07525967	0.09548671
1445725_at	predicted gene 10796	Gm10796	0.87713	1.06521037	0.09027482
1442205_at	---	---	0.87714	-1.06383027	-0.1079841
1455431_at	solute carrier family 5 (sodium/glucose cotransporter), member 1	Slc5a1	0.87716	-1.06354442	-0.1234283
1458494_at	---	---	0.87717	1.06684927	0.09213031
1421365_at	follistatin	Fst	0.87717	1.07667886	0.09786524
1418433_at	calcium binding protein 39	Cab39	0.87718	1.04527129	0.06362587
1456494_a_at	tripartite motif-containing 30A /// tripartite motif-containing 30D	Trim30a /// Trim30d	0.8772	-1.05982981	-0.0845065
1455274_at	predicted gene 14226	Gm14226	0.87721	-1.06114467	-0.0892457
1425268_a_at	platelet endothelial aggregation receptor 1	Pear1	0.87722	1.06923582	0.0924007
1446896_at	expressed sequence AU022186	AU022186	0.87722	1.07102713	0.09477937
1422791_at	platelet-activating factor acetylhydrolase, isoform 1b, subunit 2	Pafah1b2	0.87723	1.06480775	0.08425308
1423894_a_at	DALR anticodon binding domain containing 3	Dalrd3	0.87723	-1.04426362	-0.0630526
1458117_at	Ral GTPase activating protein, alpha subunit 1	Ralgapa1	0.87725	-1.06161745	-0.0934485
1419702_at	TATA box binding protein (Tbp)-associated factor, RNA polymeras	Taf1a	0.87725	1.08278907	0.09803351
1445003_at	---	---	0.87725	-1.06493827	-0.1065168
1421257_at	phosphatidylinositol glycan anchor biosynthesis, class B	Pigb	0.87725	-1.06561794	-0.0955942
1444045_at	---	---	0.87726	1.06498285	0.08822926
1456902_at	Special AT-rich sequence binding protein 1	Satb1	0.87726	-1.06427873	-0.0923786
1437143_a_at	thioredoxin-related transmembrane protein 1	Tmx1	0.87726	1.0568028	0.07405594
1431358_at	RIKEN cDNA 4930547N16 gene	4930547N16Rik	0.87726	1.05716213	0.07914311
1441564_at	---	---	0.87726	1.05232296	0.07108958
1416204_at	glycerol-3-phosphate dehydrogenase 1 (soluble)	Gpd1	0.87727	-1.06513677	-0.1055897
1429418_at	CDC14 cell division cycle 14 homolog B (S. cerevisiae)	Cdc14b	0.87729	-1.04896344	-0.0691614
1425819_at	zinc finger and BTB domain containing 7C	Zbtb7c	0.87729	1.07607989	0.09928992
1433125_at	RIKEN cDNA 5330422M15 gene	5330422M15Rik	0.87729	-1.06262213	-0.0886049
1451565_s_at	urocanase domain containing 1	Uroc1	0.8773	1.06956013	0.09399139
1428439_at	negative regulator of ubiquitin-like proteins 1	Nub1	0.8773	1.08666382	0.10548901
1457545_at	RIKEN cDNA 9530036O11Rik	9530036O11Rik	0.87731	-1.06160774	-0.0963482
1460189_at	DDB1 and CUL4 associated factor 11	Dcaf11	0.87732	1.0504992	0.0697559
1416809_at	cytochrome P450, family 3, subfamily a, polypeptide 11	Cyp3a11	0.87732	1.05311541	0.07444852
1433592_at	calmodulin 1	Calm1	0.87732	1.07147066	0.08829942
1420869_at	myeloid/lymphoid or mixed-lineage leukemia (trithorax homolog	Mllt10	0.87732	1.0656927	0.08296753

1458761_at	DDRGK domain containing 1	Ddrgk1	0.87732	1.05532764	0.07442645
1448549_a_at	dolichyl-phosphate (UDP-N-acetylglucosamine) acetylglucosamin	Dpagt1	0.87733	1.05432402	0.07263948
1416900_s_at	growth differentiation factor 1 /// LAG1 homolog, ceramide synth	Gdf1 /// Lass1	0.87733	1.05284714	0.07361598
1423814_at	DEAD (Asp-Glu-Ala-Asp) box polypeptide 41	Ddx41	0.87733	1.0483563	0.06773001
1439154_at	nucleoporin 98	Nup98	0.87734	1.05594624	0.07502927
1443855_at	potassium voltage gated channel, Shaw-related subfamily, meml	Kcnc1	0.87734	-1.06419244	-0.0961595
1449588_at	ATP-binding cassette, sub-family A (ABC1), member 4	Abca4	0.87734	-1.06347635	-0.1057485
1458034_at	---	---	0.87734	1.06783875	0.09110305
1446130_at	microRNA mir-1931	Mir1931	0.87735	-1.06001518	-0.0870595
1431496_at	RIKEN cDNA B230104F01 gene	B230104F01Rik	0.87735	1.06422712	0.08841103
1444526_at	Signal sequence receptor, alpha	Ssr1	0.87735	1.06582579	0.09077718
1425166_at	retinoblastoma-like 1 (p107)	Rbl1	0.87736	1.05273463	0.07372009
1431733_at	alpha-kinase 1	Alpk1	0.87736	-1.06076118	-0.0909347
1444717_at	---	---	0.87736	-1.05685882	-0.0850018
1418948_at	fascin homolog 3, actin-bundling protein, testicular (Strongylocent	Fscn3	0.87736	-1.068192	-0.1198779
1447502_at	---	---	0.87736	1.06911444	0.08738421
1447781_s_at	RNA (guanine-9-) methyltransferase domain containing 2	Rg9mtd2	0.87736	1.04909923	0.068432
1416734_at	muskelin 1, intracellular mediator containing kelch motifs	Mkln1	0.87736	1.06634131	0.09033602
1445340_at	MYC binding protein 2	Mycbp2	0.87736	-1.06518249	-0.0995775
1423324_at	pinin	Pnn	0.87737	-1.04357306	-0.0633164
1423039_a_at	basic leucine zipper and W2 domains 1	Bzw1	0.87737	-1.04739369	-0.0761512
1443354_at	---	---	0.87737	1.05747926	0.07820103
1422642_at	CDC42 effector protein (Rho GTPase binding) 3	Cdc42ep3	0.87738	1.06259258	0.0852259
1428317_at	FUN14 domain containing 2	Fundc2	0.87738	1.06278626	0.08729789
1451732_at	PRP4 pre-mRNA processing factor 4 homolog B (yeast)	Prpf4b	0.87738	1.06744981	0.08673689
1427186_a_at	myocyte enhancer factor 2A	Mef2a	0.87739	1.07506474	0.09795692
1450729_at	heparan sulfate 2-O-sulfotransferase 1	Hs2st1	0.87739	1.0461423	0.06323991
1416477_at	ubiquitin-conjugating enzyme E2D 2	Ube2d2	0.87739	-1.04302942	-0.0652123
1416641_at	ligase I, DNA, ATP-dependent	Lig1	0.87739	1.03988358	0.05607914
1416638_at	sal-like 2 (Drosophila)	Sall2	0.87739	1.10953629	0.12222697
1419546_at	ATPase, H+ transporting, lysosomal V1 subunit C1	Atp6v1c1	0.8774	-1.04551713	-0.068311
1418064_at	TCF3 (E2A) fusion partner	Tfpt	0.8774	1.0578192	0.07696891
1428315_at	EBNA1 binding protein 2	Ebna1bp2	0.8774	1.04271388	0.05945868
1449920_at	cytochrome P450, family 19, subfamily a, polypeptide 1	Cyp19a1	0.87741	-1.06130202	-0.0916656
1428510_at	latrophilin 1	Lphn1	0.87741	-1.04116993	-0.0583628
1428618_at	host cell factor C2	Hcfc2	0.87741	1.06057698	0.08458735
1440938_at	triggering receptor expressed on myeloid cells-like 2	Treml2	0.87741	1.0658242	0.08756937
1420381_a_at	ribosomal protein L31	Rpl31	0.87741	1.04535057	0.06350833
1448343_a_at	neighbor of Brca1 gene 1	Nbr1	0.87741	1.04251609	0.05971527
1443290_at	---	---	0.87741	1.07152399	0.09449418
1415823_at	stearoyl-Coenzyme A desaturase 2	Scd2	0.87742	1.04961388	0.06527685
1432092_a_at	GULP, engulfment adaptor PTB domain containing 1	Gulp1	0.87743	-1.0589516	-0.0839112
1431468_at	cysteine-rich secretory protein 4	Crisp4	0.87743	1.06677956	0.08844858
1457990_at	ankyrin repeat and sterile alpha motif domain containing 1B	Anks1b	0.87743	-1.06445432	-0.1076993
1430229_at	G protein-coupled receptor 137C	Gpr137c	0.87743	-1.06000478	-0.0846076
1460719_a_at	purinergic receptor P2X, ligand-gated ion channel, 1	P2rx1	0.87744	-1.06596426	-0.0959868
1419336_at	lipocalin 5	Lcn5	0.87744	-1.06378156	-0.0911184
1438270_at	expressed sequence AI846148	AI846148	0.87744	-1.05818387	-0.1085355
1457179_at	NACHT, LRR and PYD domains-containing protein 4E-like /// NLR f	LOC100503301 /// Nlrp4e	0.87744	-1.05659973	-0.1105417
1446212_at	---	---	0.87745	-1.05137697	-0.0738277
1447705_at	NSL1, MIND kinetochore complex component, homolog (S. cerevi	Nsl1	0.87745	-1.05633161	-0.0865073
1440711_at	RIKEN cDNA C630001G18 gene	C630001G18Rik	0.87745	-1.06334986	-0.0936769
1440993_at	expressed sequence AW111846	AW111846	0.87746	-1.06104298	-0.0878928
1424409_at	claudin 23	Cldn23	0.87746	1.06715788	0.08007234
1427152_at	glutamine and serine rich 1	Qser1	0.87747	-1.05111188	-0.0743134
1443478_at	expressed sequence C86695	C86695	0.87747	-1.06472281	-0.1071251
1447581_at	---	---	0.87748	1.09593838	0.11072681
1426392_a_at	ARP3 actin-related protein 3 homolog (yeast)	Actr3	0.8775	-1.0454676	-0.0724567
1425987_a_at	potassium large conductance calcium-activated channel, subfami	Kcnma1	0.8775	1.05564905	0.07700196
1454653_at	copine family member IX	Cpne9	0.87751	1.0680255	0.09031195
1458873_at	asp (abnormal spindle)-like, microcephaly associated (Drosophila	Aspm	0.87753	-1.06589371	-0.1105951
1432558_a_at	myelin and lymphocyte protein, T-cell differentiation protein	Mal	0.87755	-1.05935122	-0.0833039
1446713_at	---	---	0.87764	-1.05589901	-0.0802115
1436005_at	SURP and G patch domain containing 2	Sugp2	0.87766	-1.05968929	-0.0852582
1422725_at	male germ cell-associated kinase	Mak	0.87766	1.06739272	0.09105884
1426013_s_at	pleckstrin homology domain containing, family A (phosphoinositic	Plekha4	0.87767	-1.05502419	-0.1179351
1450500_at	U2AF homology motif (UHM) kinase 1	Uhmk1	0.87769	-1.06080515	-0.0909234
1448014_s_at	ubiquitin specific peptidase 24	Usp24	0.8777	-1.05292222	-0.078631
1440312_at	ELOVL family member 7, elongation of long chain fatty acids (yea	Elovl7	0.87774	-1.05897223	-0.0840726
1454326_at	RIKEN cDNA 1700017H01 gene	1700017H01Rik	0.87784	-1.06478568	-0.0941422
1419557_a_at	transmembrane protein 9	Tmem9	0.87784	1.0634946	0.08340502
1457243_at	Transmembrane protein 219	Tmem219	0.87789	1.08002481	0.10294357
1442534_at	---	---	0.87793	-1.05409262	-0.0770009
1451432_x_at	alkB, alkylation repair homolog 4 (E. coli)	Alkbh4	0.87804	1.05534089	0.07474061
1426727_s_at	protein phosphatase 1, regulatory subunit 10 pseudogene /// pro	Gm8801 /// Ppp1r10	0.87804	1.04991355	0.06684025
1449474_a_at	nasal embryonic LHRH factor	Nelf	0.87806	1.05639849	0.07738289
1458824_at	unc-45 homolog A (C. elegans)	Unc45a	0.87812	-1.05549053	-0.0791327
1429506_at	naked cuticle 1 homolog (Drosophila)	Nkd1	0.87825	1.07080328	0.09256134
1440662_at	---	---	0.87829	-1.06266853	-0.0881294
1436968_x_at	kelch-like 24 (Drosophila)	Klhl24	0.87829	-1.06527526	-0.1260797
1455771_at	benzodiazapine receptor associated protein 1	Bzrap1	0.8783	1.0679333	0.09074663
1455370_at	RIKEN cDNA A630023P12 gene	A630023P12Rik	0.87832	-1.06058264	-0.0878064
1416250_at	B-cell translocation gene 2, anti-proliferative	Btg2	0.87832	1.11745426	0.119527
1435206_at	solute carrier family 24 (sodium/potassium/calcium exchanger), r	Slc24a4	0.87833	1.07392674	0.0946285
1435947_at	Protein prenyltransferase alpha subunit repeat containing 1	Ptar1	0.87835	-1.04411389	-0.0638811
1417619_at	growth arrest and DNA-damage-inducible, gamma interacting pro	Gadd45gip1	0.87835	1.05976259	0.0748796
1433310_at	RIKEN cDNA D530031A16 gene	D530031A16Rik	0.87837	-1.06241146	-0.094634
1423004_at	vasoactive intestinal peptide receptor 1	Vipr1	0.87837	1.09459157	0.10829882
1436958_x_at	Tropomyosin 3, gamma	Tpm3	0.8784	1.0434534	0.06126308
1416100_at	eukaryotic translation initiation factor 3, subunit D	Eif3d	0.8784	1.03219869	0.04492726
1445815_at	frizzled homolog 8 (Drosophila)	Fzd8	0.8784	1.06594119	0.0876655
1457832_at	---	---	0.8784	-1.05969052	-0.098451
1426618_a_at	protein O-linked mannose beta1,2-N-acetylglucosaminyltransfera	Pomgnt1	0.87842	1.05617928	0.07560508
1437871_at	phosphoglucomutase 5	Pgm5	0.87843	-1.05892948	-0.0829094

1437746_at	leucine rich repeat transmembrane neuronal 1	Lrrtm1	0.87843	-1.0538461	-0.0846981
1421058_at	alcohol dehydrogenase 7 (class IV), mu or sigma polypeptide	Adh7	0.87843	1.08584746	0.10197004
1438470_at	suppressor of cytokine signaling 2	Socs2	0.87846	-1.05308014	-0.0779521
1428422_at	Fas associated factor family member 2	Faf2	0.87846	1.0514085	0.07068494
1437116_at	LETM1 domain containing 1	Letmd1	0.87846	-1.05954014	-0.1085201
1442830_at	nucleolar and spindle associated protein 1	Nusap1	0.87846	1.06249532	0.08683391
1453508_at	RIKEN cDNA 1700104B16 gene /// hypothetical protein LOC1005	1700104B16Rik /// LOC10	0.87846	-1.05690258	-0.0802088
1444638_at	titin	Ttn	0.87847	-1.0617284	-0.0869658
1443036_at	zinc finger protein 804A	Zfp804a	0.87847	1.0499522	0.07026934
1446844_at	coiled-coil-helix-coiled-coil-helix domain containing 7	Chchd7	0.87848	-1.05198881	-0.0885929
1434086_at	G protein-coupled receptor 107	Gpr107	0.87848	1.04762206	0.06673755
1431284_a_at	spinster homolog 1 (Drosophila)	Spns1	0.8785	1.04682717	0.0646767
1452228_at	TBC1 domain family, member 23	Tbc1d23	0.8785	1.0616185	0.08061426
1449047_at	2-hydroxyacyl-CoA lyase 1	Hacl1	0.8785	1.06855273	0.08843691
1449068_at	zinc finger protein 148	Zfp148	0.8785	-1.05462664	-0.0788248
1422351_at	olfactory receptor 480	Olfr480	0.87851	1.08157155	0.09821514
1441239_at	potassium channel tetramerisation domain containing 18	Kctd18	0.87852	1.06377065	0.0850497
1424336_at	phosphopantothenoylcysteine decarboxylase	Ppcdc	0.87853	-1.06094986	-0.0882052
1441131_at	---	---	0.87853	-1.06238013	-0.0908701
1424994_at	glycerate kinase	Glyctk	0.87853	-1.05870633	-0.0835259
1448517_at	translocase of inner mitochondrial membrane 22 homolog (yeast	Timm22	0.87854	1.05000767	0.06668543
1429982_at	ribosomal modification protein rimK-like family member B	Rimklb	0.87854	-1.05996103	-0.0895573
1456944_at	potassium voltage gated channel, Shaw-related subfamily, memb	Kcnc1	0.87854	-1.06447552	-0.0923283
1439497_at	ataxin 7-like 1	Atxn7l1	0.87855	1.06712722	0.09067031
1440928_at	RIKEN cDNA D630037F22 gene	D630037F22Rik	0.87855	-1.06513687	-0.1056651
1431239_at	non-POU-domain-containing, octamer binding protein	Nono	0.87855	-1.0641463	-0.1189505
1460230_at	synapsin II	Syn2	0.87855	-1.06723295	-0.1222608
1422874_at	proacrosin binding protein	Acrbp	0.87856	1.06092114	0.08450101
1428333_at	RIKEN cDNA 2900062L11 gene	2900062L11Rik	0.87856	-1.0435327	-0.0620411
1450145_at	ankyrin repeat domain 5	Ankrd5	0.87856	-1.0644588	-0.0989071
1424490_at	zinc finger protein 428	Zfp428	0.87857	-1.04741707	-0.0788895
1448931_at	coagulation factor II (thrombin) receptor-like 1	F2rl1	0.87858	1.05467402	0.06989319
1443770_x_at	---	---	0.87858	1.07027223	0.08969065
1419867_a_at	ankyrin repeat and KH domain containing 1	Ankhd1	0.87858	1.05467438	0.07175574
1430407_at	RIKEN cDNA 3110035C09 gene	3110035C09Rik	0.87858	1.06462159	0.08536033
1428778_at	Sfi1 homolog, spindle assembly associated (yeast)	Sfi1	0.87858	1.06787708	0.09133713
1425300_at	dihydroxyacetone kinase 2 homolog (yeast)	Dak	0.87859	-1.05852437	-0.1472412
1426159_x_at	T-cell receptor beta, joining region	Tcrb-J	0.87859	-1.05853903	-0.0843404
1424835_at	glutathione S-transferase, mu 4	Gstm4	0.8786	1.05527385	0.07751075
1442303_at	hypothetical LOC100504674	LOC100504674	0.8786	1.07832881	0.09879035
1439665_at	lysophosphatidic acid receptor 4	Lpar4	0.8786	1.06356874	0.08769861
1442505_at	---	---	0.87861	-1.0559449	-0.0791749
1420478_at	nucleosome assembly protein 1-like 1	Nap1l1	0.87861	1.0422744	0.05679874
1458457_at	---	---	0.87862	-1.06251827	-0.0929825
1437661_at	expressed sequence AU021092	AU021092	0.87862	-1.06231556	-0.0994437
1429545_at	ubiquitin-conjugating enzyme E2I	Ube2i	0.87862	-1.06510144	-0.117873
1448121_at	WW domain binding protein 2	Wbp2	0.87862	1.06839994	0.08285218
1445953_at	DNA segment, Chr 17, ERATO Doi 191, expressed	D17Ertd191e	0.87862	-1.0554618	-0.0782912
1440525_at	---	---	0.87863	-1.0567393	-0.1610586
1454954_at	WD repeat domain 61	Wdr61	0.87863	-1.06269766	-0.0895935
1419081_at	autophagy-related 10 (yeast)	Atg10	0.87863	-1.06079714	-0.0854782
1437277_x_at	transglutaminase 2, C polypeptide	Tgm2	0.87863	-1.04848857	-0.0687003
1425665_a_at	signal recognition particle 54A /// signal recognition particle 54B /	Srp54a /// Srp54b /// Srp	0.87863	-1.04828123	-0.0810625
1447850_x_at	zinc finger, AN1-type domain 3	Zfand3	0.87864	1.06011997	0.08383069
1424021_at	ADP-ribosylation factor-like 6 interacting protein 6	Arl6ip6	0.87865	1.04825112	0.06765915
1432324_at	RIKEN cDNA 4930432J09 gene	4930432J09Rik	0.87865	1.07905495	0.10100441
1422181_at	cyclic nucleotide gated channel alpha 2	Cnga2	0.87866	-1.06189701	-0.0933151
1434932_at	adenosine deaminase, RNA-specific, B1	Adarb1	0.87867	1.0551354	0.07683734
1460307_at	thymoma viral proto-oncogene 3	Akt3	0.87867	1.08465049	0.10340705
1442123_at	RIKEN cDNA 2010012P19 gene /// hypothetical LOC100503827	2010012P19Rik /// LOC10	0.8787	1.06148284	0.08445538
1445059_at	---	---	0.8787	-1.05869576	-0.0833425
1433861_at	coiled-coil and C2 domain containing 1B	Cc2d1b	0.87871	1.04330418	0.06082122
1431112_at	unc-51-like kinase 3 (C. elegans)	Ulk3	0.87872	-1.05602855	-0.0877003
1452543_a_at	secretoglobin, family 1A, member 1 (uteroglobin)	Scgb1a1	0.87872	-1.05350946	-0.0755967
1423812_s_at	vesicular, overexpressed in cancer, prosurvival protein 1	Vopp1	0.87873	-1.0631962	-0.0921824
1440900_at	G protein-coupled receptor 174	Gpr174	0.87873	-1.06052442	-0.0918629
1431520_at	RIKEN cDNA 4933406J09 gene	4933406J09Rik	0.87874	-1.06086579	-0.0910925
1421489_a_at	RIKEN cDNA 2010106E10 gene	2010106E10Rik	0.87874	-1.0481933	-0.0733251
1439976_at	asparagine-linked glycosylation 11 homolog (yeast, alpha-1,2-ma	Alg11	0.87875	1.06324623	0.08641675
1437348_at	F-box protein 28	Fbxo28	0.87876	1.04940014	0.06943633
1424640_at	ADP-ribosylation factor-like 8A	Arl8a	0.87876	1.0536023	0.07172408
1432608_at	nicotinamide nucleotide transhydrogenase	Nnt	0.87876	1.08884782	0.10653681
1441307_at	keratin associated protein 4-1	Krtap4-1	0.87877	1.06414022	0.08377314
1439908_at	zinc finger with KRAB and SCAN domains 1	Zkscan1	0.87877	1.06436859	0.08770548
1420406_at	paternally expressed 12	Peg12	0.87878	-1.06479471	-0.1393592
1455764_at	Vpr (HIV-1) binding protein	Vprbp	0.87878	-1.06159742	-0.1071709
1459307_at	---	---	0.87879	1.07111346	0.09323482
1436995_a_at	ribosomal protein L26	Rpl26	0.8788	1.02114253	0.02965751
1433600_at	adrenergic receptor, alpha 2a	Adra2a	0.8788	-1.06081975	-0.0889009
1427020_at	scavenger receptor class A, member 3	Scara3	0.8788	-1.0602885	-0.092529
1445077_at	---	---	0.8788	-1.06517397	-0.0962189
1442050_at	zinc finger protein 608	Zfp608	0.87881	1.05683611	0.07870642
1447393_at	---	---	0.87882	-1.05570866	-0.0898611
1437964_at	neurexophilin 2	Nxph2	0.87883	-1.05656619	-0.0880219
1445484_at	cytochrome c, somatic	Cycs	0.87884	1.08699876	0.1063333
1442551_at	---	---	0.87884	1.05454938	0.07225705
1417259_a_at	capping protein (actin filament) muscle Z-line, beta	Capzb	0.87884	-1.04199468	-0.0606476
1432888_at	RIKEN cDNA 4930455M05 gene	4930455M05Rik	0.87884	-1.05877474	-0.0835042
1421967_at	UDP-Gal:betaGlcNAc beta 1,4-galactosyltransferase, polypeptide	B4galt5	0.87886	-1.06018829	-0.084536
1422049_at	NK1 transcription factor related, locus 2 (Drosophila)	Nkx1-2	0.87887	1.07011986	0.09490999
1417317_s_at	INO80 homolog (S. cerevisiae) /// ribosomal protein L35A	Ino80 /// Rpl35a	0.87887	1.02485009	0.0345428
1454480_at	RIKEN cDNA 4933439N06 gene	4933439N06Rik	0.87887	-1.05796248	-0.0826747
1424808_at	laminin, alpha 4	Lama4	0.87888	-1.05936132	-0.0848681
1457634_at	thymosin beta 15b1 /// thymosin beta 15b2 /// thymosin beta 15	Tmsb15b1 /// Tmsb15b2	0.87888	1.04690401	0.06550645

1432076_at	RIKEN cDNA 4933430H16 gene	4933430H16Rik	0.87889	1.05294917	0.07350192
1425293_a_at	zinc finger, CCHC domain containing 18	Zcchc18	0.87891	1.0702402	0.0943986
1447315_at	---	---	0.87891	1.10773819	0.11855189
1448216_at	synaptogyrin 3	Syngr3	0.87894	1.09456666	0.11069529
1420243_at	zinc finger E-box binding homeobox 1	Zeb1	0.87895	-1.05682116	-0.0817602
1458211_at	---	---	0.87895	1.04706724	0.06476189
1439844_at	RIKEN cDNA 8430426J06 gene	8430426J06Rik	0.87896	1.06652345	0.09008431
1440464_at	ELAV (embryonic lethal, abnormal vision, Drosophila)-like 1 (Hu a	Elavl1	0.87897	-1.06000896	-0.0887001
1420267_at	RIKEN cDNA 2610201A13 gene	2610201A13Rik	0.87897	1.09426896	0.10632862
1460134_at	RIKEN cDNA 9330175E14 gene	9330175E14Rik	0.87897	1.11270731	0.12397171
1444259_at	expressed sequence AW495222	AW495222	0.87897	1.06693379	0.08841917
1415734_at	RAB7, member RAS oncogene family	Rab7	0.87897	1.04625428	0.06286138
1456391_at	tudor domain containing 5	Tdrd5	0.87899	1.05967379	0.07878511
1416128_at	predicted gene 5620 /// predicted gene 6682 /// tubulin, alpha 1/	Gm5620 /// Gm6682 /// T	0.87899	-1.05432871	-0.0936624
1450120_at	sodium channel, voltage-gated, type I, alpha	Scn1a	0.879	1.06527955	0.08933355
1415985_at	splicing factor 3b, subunit 3	Sf3b3	0.879	1.03974309	0.05559584
1425769_x_at	chemokine-like factor	Cklf	0.879	1.06464812	0.08550273
1451342_at	spondin 1, (f-spondin) extracellular matrix protein	Spon1	0.879	-1.06064211	-0.0881581
1454361_at	RIKEN cDNA 9230106L01 gene	9230106L01Rik	0.87901	1.07489935	0.09555099
1449485_at	receptor (TNFRSF)-interacting serine-threonine kinase 1	Ripk1	0.87902	-1.04789235	-0.0710455
1432098_a_at	olfactory receptor 701	Olfr701	0.87904	-1.05778691	-0.0844276
1432323_at	RIKEN cDNA 8430436N08 gene	8430436N08Rik	0.87909	1.071778	0.09325763
1450426_at	cholinergic receptor, nicotinic, alpha polypeptide 6	Chrna6	0.87919	-1.06298019	-0.0903566
1428990_at	RIKEN cDNA 2310047K21 gene	2310047K21Rik	0.87921	1.12006905	0.12937557
1426754_x_at	cytoskeleton-associated protein 4	Ckap4	0.87921	1.08152472	0.09311589
1460197_a_at	STEAP family member 4	Steap4	0.87922	-1.0605126	-0.0867117
1457882_at	Ewing's tumor-associated antigen 1	Etaa1	0.87923	-1.06012566	-0.0848655
1456073_s_at	pannexin 3	Panx3	0.87923	-1.05331039	-0.0816818
1423805_at	disabled homolog 2 (Drosophila)	Dab2	0.87923	-1.06401243	-0.0962979
1419532_at	interleukin 1 receptor, type II	Il1r2	0.87924	1.06588419	0.08809747
1433372_at	RIKEN cDNA 5330425B07 gene	5330425B07Rik	0.87925	-1.05945594	-0.0853881
1441657_at	---	---	0.87926	-1.06247131	-0.0941909
1459822_at	RAE1 RNA export 1 homolog (S. pombe)	Rae1	0.87928	1.08936252	0.1085712
1442067_at	---	---	0.87929	-1.06000046	-0.0974236
1427813_at	iduronate 2-sulfatase	Ids	0.8793	-1.05011371	-0.0707195
1435746_at	serine/arginine-rich protein specific kinase 2	Srpk2	0.87931	-1.06108846	-0.0954168
1420563_at	glutamate receptor, ionotropic, AMPA3 (alpha 3)	Gria3	0.87932	1.08098548	0.09993287
1432147_at	RIKEN cDNA 4930447F24 gene	4930447F24Rik	0.87933	1.06667172	0.09050922
1426994_at	PH domain and leucine rich repeat protein phosphatase 1	Phlpp1	0.87933	1.0531372	0.0743063
1458480_at	topoisomerase (DNA) II binding protein 1	Topbp1	0.87935	1.07149504	0.09513404
1451022_at	low density lipoprotein receptor-related protein 6	Lrp6	0.87935	-1.05504108	-0.0785686
1419029_at	ERO1-like (S. cerevisiae)	Ero1l	0.87936	1.05919175	0.07661027
1460081_at	synaptotagmin VII	Syt7	0.87936	-1.05833871	-0.092681
1441282_at	Ssu72 RNA polymerase II CTD phosphatase homolog (yeast)	Ssu72	0.87936	1.06766969	0.08734725
1460347_at	keratin 14	Krt14	0.87937	1.11813751	0.12644501
1427635_at	Kinesin family member 5A	Kif5a	0.87937	-1.04597821	-0.0679714
1443366_at	N-ethylmaleimide sensitive fusion protein attachment protein gar	Napg	0.87938	1.14797198	0.14373675
1417004_at	tumor susceptibility gene 101	Tsg101	0.87939	1.04668267	0.0631634
1447638_at	arylformamidase	Afmid	0.87939	-1.04805211	-0.06777
1451479_a_at	transmembrane protein 53	Tmem53	0.87939	-1.06165203	-0.0870168
1459633_at	---	---	0.8794	-1.0597365	-0.0852038
1449122_at	UBX domain protein 2B	Ubxn2b	0.8794	-1.05927668	-0.0952925
1436586_x_at	ribosomal protein S14	Rps14	0.87941	1.02513282	0.03442477
1447530_at	Factor 8-associated gene A	F8a	0.87941	-1.05669031	-0.0963819
1450494_x_at	carcinoembryonic antigen-related cell adhesion molecule 1	Ceacam1	0.87942	1.18922702	0.1663766
1458288_at	---	---	0.87942	-1.06270933	-0.0942453
1452033_at	GLE1 RNA export mediator (yeast)	Gle1	0.87943	1.0432934	0.06042151
1451601_a_at	spinster homolog 2 (Drosophila)	Spns2	0.87943	-1.05492972	-0.0787532
1456502_at	dual-specificity tyrosine-(Y)-phosphorylation regulated kinase 2	Dyrk2	0.87944	1.06460936	0.085227
1442364_at	Mitogen-activated protein kinase 14	Mapk14	0.87945	-1.05933661	-0.0972507
1434230_at	polymerase (DNA directed), beta	Polb	0.87948	-1.04420887	-0.0631227
1428070_at	synovial apoptosis inhibitor 1, synoviolin	Syvn1	0.87948	1.06169428	0.07877591
1437968_at	glutamate receptor, ionotropic, NMDA1 (zeta 1)	Grin1	0.87949	1.05867773	0.07876682
1419895_at	expressed sequence AA536748	AA536748	0.8795	-1.05668069	-0.0839601
1419100_at	serine (or cysteine) peptidase inhibitor, clade A, member 3N	Serpina3n	0.87951	1.06799009	0.09149072
1459010_at	---	---	0.87953	-1.0576524	-0.0864061
1451754_a_at	WD repeat domain 45	Wdr45	0.87953	-1.04743624	-0.0676863
1416690_at	GTP binding protein 2	Gtpbp2	0.87954	1.05370825	0.07443867
1457685_at	zinc finger protein 81	Zfp81	0.87955	-1.04600622	-0.0673296
1450366_at	harakiri, BCL2 interacting protein (contains only BH3 domain)	Hrk	0.87957	-1.05623876	-0.0797403
1421804_at	fatty acid binding protein 9, testis	Fabp9	0.87958	1.05702539	0.07583478
1430332_a_at	glucuronidase, beta	Gusb	0.87959	1.08356651	0.09982488
1449548_at	ephrin B2	Efnb2	0.87961	-1.06166894	-0.0907201
1444368_at	Cholinergic receptor, nicotinic, alpha polypeptide 3	Chrna3	0.87961	1.11736603	0.12405716
1426585_s_at	mitogen-activated protein kinase 1	Mapk1	0.87961	1.04794177	0.06440019
1419638_at	ephrin B2	Efnb2	0.87962	1.07302953	0.09319533
1453987_at	kelch-like 28 (Drosophila)	Klhl28	0.87962	-1.06217914	-0.0984918
1422931_at	fos-like antigen 2	Fosl2	0.87963	-1.05676105	-0.0824607
1446810_at	RIKEN cDNA A730062M13 gene	A730062M13Rik	0.87963	-1.06827022	-0.1469131
1423862_at	pleckstrin homology domain containing, family F (with FYVE dom	Plekhf2	0.87963	1.06591233	0.08266191
1449537_at	mutS homolog 5 (E. coli)	Msh5	0.87963	-1.05589142	-0.0786565
1438966_x_at	fibromodulin	Fmod	0.87963	1.06123903	0.08048497
1445333_at	---	---	0.87964	1.06188417	0.08484265
1416303_at	LPS-induced TN factor	Litaf	0.87964	1.04575798	0.06394355
1418088_a_at	syntaxin 8	Stx8	0.87964	1.05890755	0.07855591
1419121_at	thiopurine methyltransferase	Tpmt	0.87964	-1.05756341	-0.0820364
1446131_at	hypothetical LOC100503678	LOC100503678	0.87964	-1.06401381	-0.1029059
1420814_at	guanosine diphosphate (GDP) dissociation inhibitor 2	Gdi2	0.87965	-1.03761877	-0.0556733
1433885_at	IQ motif containing GTPase activating protein 2	Iqgap2	0.87965	1.05450608	0.07493659
1423018_at	potassium voltage-gated channel, shaker-related subfamily, men	Kcna3	0.87966	1.06018461	0.08403301
1444658_at	RIKEN cDNA 5830403L16 gene	5830403L16Rik	0.87966	-1.06738301	-0.1045309
1421427_at	collagen, type XXV, alpha 1	Col25a1	0.87967	-1.05412567	-0.0775275
1418154_at	NEDD4 binding protein 1	N4bp1	0.87967	-1.04680734	-0.0686677
1442252_at	kinesin family member 13A	Kif13a	0.87967	1.05602489	0.07816394

1449675_at	cyclin B1	Ccnb1	0.87967	-1.05518328	-0.0811479
1437212_at	zinc finger protein 420	Zfp420	0.87968	-1.06258955	-0.0907153
1428374_at	glucuronyl C5-epimerase	Glce	0.87968	-1.04678958	-0.0689872
1456576_x_at	CCR4-NOT transcription complex, subunit 2	Cnot2	0.87968	1.0499407	0.06588633
1430082_at	WD repeat domain 64	Wdr64	0.87969	-1.06373215	-0.097805
1424248_at	cyclic AMP-regulated phosphoprotein, 21	Arpp21	0.87969	1.06951513	0.08915652
1435940_at	doublecortin-like kinase 1	Dclk1	0.87969	-1.05975637	-0.0897563
1442516_at	DNA segment, Chr 2, ERATO Doi 623, expressed	D2Ertd623e	0.87969	1.05193156	0.07097378
1439845_at	heterochromatin protein 1, binding protein 3	Hp1bp3	0.8797	1.11847325	0.12736615
1459738_x_at	galactosidase, alpha	Gla	0.8797	1.06971882	0.08760731
1448758_at	nuclear receptor binding factor 2	Nrbf2	0.8797	-1.04304011	-0.0616482
1459018_at	DNA segment, Chr 10, ERATO Doi 447, expressed	D10Ertd447e	0.8797	1.0600712	0.07507775
1450722_at	nucleoporin 50	Nup50	0.8797	1.04155041	0.05794543
1460651_at	linker for activation of T cells	Lat	0.8797	1.05254204	0.07245929
1445325_at	---	---	0.87971	1.06591916	0.09018305
1417679_at	growth factor independent 1	Gfi1	0.87971	-1.05945742	-0.0996808
1441464_at	RIKEN cDNA A630035D09 gene	A630035D09Rik	0.87971	-1.06249506	-0.0944592
1446322_at	expressed sequence C78568	C78568	0.87972	-1.05521295	-0.0815943
1451042_a_at	myc induced nuclear antigen	Mina	0.87972	1.04987738	0.06678196
1452240_at	CUGBP, Elav-like family member 4	Celf4	0.87972	1.05631311	0.07862318
1426135_a_at	Parkinson disease (autosomal recessive, juvenile) 2, parkin	Park2	0.87972	1.09024417	0.10840107
1458160_at	---	---	0.87973	-1.059905	-0.1004978
1459498_at	---	---	0.87973	1.0671676	0.08925817
1434137_x_at	zymogen granule protein 16	Zg16	0.87973	1.07042376	0.09350466
1419440_at	tripartite motif-containing 54	Trim54	0.87974	1.07123734	0.09185693
1419541_at	protein phosphatase 1 regulatory subunit 2 pseudogene	4930403L05Rik	0.87974	1.06292485	0.08685608
1434562_at	microfibrillar-associated protein 3	Mfap3	0.87974	1.04925914	0.06719091
1436988_at	isthmin 1 homolog (zebrafish)	Ism1	0.87974	-1.06132661	-0.0907698
1449301_at	solute carrier family 7, (cationic amino acid transporter, y+ system	Slc7a13	0.87975	-1.05905387	-0.0937542
1449361_at	T-box 21	Tbx21	0.87975	1.06363127	0.08526553
1444320_at	DDHD domain containing 2	Ddhd2	0.87975	-1.06379674	-0.1264591
1430193_at	cancer susceptibility candidate 5	Casc5	0.87976	-1.04568455	-0.0648741
1438223_at	---	---	0.87976	-1.05885048	-0.0872084
1439468_at	---	---	0.87976	-1.06468265	-0.0942679
1446854_at	---	---	0.87976	-1.05898679	-0.09451
1416473_a_at	immunoglobulin superfamily, DCC subclass, member 4	Igdcc4	0.87976	1.04876549	0.06849753
1445729_at	Proteasome (prosome, macropain) 26S subunit, non-ATPase, 13	Psmd13	0.87977	1.10291714	0.11363831
1434017_at	zinc and ring finger 2	Znrf2	0.87977	1.04737829	0.06650675
1454598_at	RIKEN cDNA 4930425F17 gene	4930425F17Rik	0.87977	-1.06136942	-0.0947327
1455914_at	expressed sequence AI987944	AI987944	0.87977	-1.06150969	-0.1035665
1459730_at	---	---	0.87977	1.08133572	0.10013979
1444013_at	RIKEN cDNA 5930412G12 gene	5930412G12Rik	0.87978	-1.06053925	-0.0850261
1459292_at	---	---	0.87978	-1.05952994	-0.0848008
1442683_at	high mobility group 20A	Hmg20a	0.87978	-1.0579544	-0.0910724
1435736_x_at	intraflagellar transport 27 homolog (Chlamydomonas)	Ift27	0.87979	1.05750635	0.07724674
1420900_a_at	RAB18, member RAS oncogene family	Rab18	0.87979	1.06801609	0.08203911
1442585_at	RIKEN cDNA B230220N19 gene	B230220N19Rik	0.87979	-1.06279063	-0.0930435
1449225_a_at	mitogen-activated protein kinase 8 interacting protein 2	Mapk8ip2	0.87979	1.06757865	0.08775915
1427141_at	NDC80 homolog, kinetochore complex component pseudogene	2700099C18Rik	0.87979	-1.05979552	-0.1017385
1426669_at	calcineurin-like phosphoesterase domain containing 1	Cpped1	0.87979	-1.04673226	-0.0681384
1438716_at	tripartite motif-containing 30D	Trim30d	0.8798	-1.05776528	-0.0836443
1421227_at	granzyme D /// granzyme E	Gzmd /// Gzme	0.8798	1.05102969	0.06988678
1435848_at	RIKEN cDNA D430041D05 gene	D430041D05Rik	0.8798	-1.06138303	-0.0880507
1416479_a_at	transmembrane protein 14C	Tmem14c	0.8798	1.04728515	0.06490519
1445879_at	zinc finger protein 507	Zfp507	0.8798	-1.06518693	-0.1010833
1446299_at	---	---	0.87981	1.06795489	0.08921652
1438594_at	---	---	0.87982	-1.05871129	-0.0829202
1424390_at	nucleoporin like 1	Nupl1	0.87982	1.05963687	0.07451965
1453127_at	protein phosphatase 1J	Ppm1j	0.87982	1.05142076	0.06956609
1440267_at	peroxisomal biogenesis factor 1	Pex1	0.87983	1.07969868	0.10070257
1424908_at	mitochondrial methionyl-tRNA formyltransferase	Mtfmt	0.87983	1.05055199	0.07093806
1445036_at	DNA segment, Chr 11, ERATO Doi 518, expressed	D11Ertd518e	0.87983	1.08157828	0.09912146
1444804_at	DNA segment, Chr 17, ERATO Doi 96, expressed	D17Ertd96e	0.87985	1.05559495	0.07731237
1425965_at	ubiquitin C	Ubc	0.87985	1.08991281	0.10567888
1433178_at	RIKEN cDNA 4933427C19 gene	4933427C19Rik	0.87986	-1.05385443	-0.0776818
1456457_at	Solute carrier family 32 (GABA vesicular transporter), member 1	Slc32a1	0.87986	1.08142869	0.09921508
1432516_at	RIKEN cDNA 4930447F04 gene	4930447F04Rik	0.87987	-1.04902053	-0.0764787
1417715_a_at	glutamate oxaloacetate transaminase 2, mitochondrial	Got2	0.87988	1.04208067	0.05861135
1442311_at	Zinc finger RNA binding protein	Zfr	0.87989	1.06096498	0.08246614
1433820_a_at	RIKEN cDNA 1110012D08 gene	1110012D08Rik	0.87989	1.07731902	0.09518135
1421055_at	large tumor suppressor 2 /// exportin 4	Lats2 /// Xpo4	0.8799	-1.04497343	-0.0653584
1446578_at	---	---	0.87991	-1.06110451	-0.0857508
1460340_at	piwi-like homolog 1 (Drosophila)	Piwil1	0.87991	1.06552307	0.08927831
1426641_at	tribbles homolog 2 (Drosophila)	Trib2	0.87992	1.05959262	0.08277416
1445774_at	---	---	0.87994	-1.06253447	-0.0983767
1422503_s_at	poly (ADP-ribose) polymerase family, member 1	Parp1	0.87995	-1.03935038	-0.0567204
1424483_at	MOB1, Mps One Binder kinase activator-like 1B (yeast)	Mobkl1b	0.87996	1.06096871	0.07849759
1432392_at	kinesin family member 2C	Kif2c	0.87998	-1.06236581	-0.0912581
1450961_a_at	transcription elongation factor A (SII)-like 3 /// transcription elong	Tceal3 /// Tceal5 /// Tcea	0.87998	-1.06354427	-0.1300742
1460451_at	transmembrane protein 52	Tmem52	0.87998	1.05823387	0.08085518
1448869_a_at	mitochondrial ribosomal protein S16	Mrps16	0.87999	1.05475513	0.07014481
1440103_at	---	---	0.87999	-1.06030303	-0.0868281
1454961_at	synaptojanin 1	Synj1	0.88	1.050575	0.06849841
1429044_at	calmodulin regulated spectrin-associated protein 1-like 1	Camsap1l1	0.88	1.04882985	0.06765954
1449029_at	MAP kinase-interacting serine/threonine kinase 2	Mknk2	0.88	1.05917891	0.07587504
1422785_at	RAS p21 protein activator 2	Rasa2	0.88001	-1.05714449	-0.0991966
1438600_at	predicted gene 6531	Gm6531	0.88002	-1.06194244	-0.0872308
1445697_at	---	---	0.88002	1.06343244	0.0868807
1443333_at	hypothetical LOC100504740	LOC100504740	0.88002	1.07242956	0.08826336
1428144_at	leucine-rich repeats and WD repeat domain containing 1	Lrwd1	0.88002	1.04894617	0.0666329
1425722_at	interferon inducible GTPase 1B	Iigp1b	0.88003	1.07276432	0.09013512
1448138_at	protein phosphatase 2A, regulatory subunit B (PR 53)	Ppp2r4	0.88004	1.06475715	0.0798199
1454300_at	RIKEN cDNA 4933424C09 gene	4933424C09Rik	0.88004	-1.05752636	-0.0812673
1416363_at	FK506 binding protein 4 /// peptidyl-prolyl cis-trans isomerase FK	Fkbp4 /// LOC100048743	0.88005	-1.05457085	-0.0802665

1423738_at	oxidase assembly 1-like	Oxa1l	0.88005	1.07195358	0.09127089
1441596_at	---	---	0.88005	-1.06624954	-0.1118293
1441302_at	hypothetical LOC100502835	LOC100502835	0.88006	1.07023603	0.09346245
1450080_at	CAAX box 1 homolog C (human)	Cxx1c	0.88006	1.08015078	0.09873767
1459953_at	---	---	0.88006	-1.05442008	-0.0868178
1441938_x_at	CDK5 and Abl enzyme substrate 1	Cables1	0.88006	1.06814866	0.08722975
1429712_at	ethanol induced 1 /// predicted gene 14288	Etohi1 /// Gm14288	0.88007	-1.06387089	-0.0973914
1452863_at	RIKEN cDNA 1700003F12 gene	1700003F12Rik	0.88008	-1.05900703	-0.0827475
1455321_at	DDHD domain containing 1	Ddhd1	0.88008	1.08365762	0.10225603
1459567_at	expressed sequence C77905	C77905	0.88008	1.07482729	0.09714716
1450290_at	programmed cell death 1 ligand 2	Pdcd1lg2	0.88008	-1.0635009	-0.0980745
1450143_at	RAS guanyl releasing protein 1	Rasgrp1	0.88009	1.06774719	0.09170414
1455925_at	PR domain containing 8	Prdm8	0.88009	1.06236124	0.08519713
1449666_at	Attractin like 1	Atrnl1	0.88009	1.06621002	0.08966119
1416368_at	glutathione S-transferase, alpha 4	Gsta4	0.88009	1.03454178	0.04818874
1457199_at	---	---	0.88011	1.10758979	0.11902021
1455852_at	NSL1, MIND kinetochore complex component, homolog (S. cerevi	Nsl1	0.88011	1.05842399	0.08003855
1458169_at	hypothetical protein LOC675947	LOC675947	0.88011	-1.06055396	-0.0857618
1444228_s_at	hect (homologous to the E6-AP (UBE3A) carboxyl terminus) doma	Herc2	0.88011	1.06778289	0.08926711
1445281_a_at	RIKEN cDNA B230311B06 gene	B230311B06Rik	0.88011	1.05756654	0.07885121
1426842_at	YTH domain family 3	Ythdf3	0.88011	-1.05379825	-0.0891841
1458176_at	Period homolog 3 (Drosophila)	Per3	0.88011	1.06077321	0.08501463
1433139_at	---	---	0.88011	-1.06208399	-0.1195956
1453830_at	embryonic ectoderm development	Eed	0.88012	1.06470407	0.08625851
1459552_at	DNA segment, Chr 4, Brigham & Women's Genetics 0951 express	D4Bwg0951e	0.88012	-1.05497148	-0.0817745
1453627_at	RIKEN cDNA 2610020P09 gene	2610020P09Rik	0.88013	1.05231261	0.07212181
1443237_at	---	---	0.88013	1.05955604	0.07872303
1419794_at	---	---	0.88013	-1.06168719	-0.0873165
1451559_a_at	dehydrogenase/reductase (SDR family) member 4	Dhrs4	0.88013	1.05557833	0.07191995
1453870_at	RIKEN cDNA 1810018F18 gene	1810018F18Rik	0.88013	1.07943572	0.10038552
1431254_at	kelch repeat and BTB (POZ) domain containing 11	Kbtbd11	0.88014	1.06218283	0.08450538
1439575_at	transmembrane protein 232	Tmem232	0.88014	-1.05321381	-0.0753867
1439466_s_at	BUD31 homolog (yeast) /// protein BUD31 homolog	Bud31 /// LOC100045848	0.88014	1.04569951	0.06109646
1417019_a_at	cell division cycle 6 homolog (S. cerevisiae)	Cdc6	0.88014	1.04243331	0.05915441
1443515_at	RIKEN cDNA 1110032F04 gene	1110032F04Rik	0.88014	1.08394006	0.10370099
1427906_at	RIKEN cDNA 1110037F02 gene	1110037F02Rik	0.88014	1.05005939	0.06945161
1457306_at	---	---	0.88015	-1.0614123	-0.0881822
1434386_at	ATPase, Ca++-sequestering	Atp2c1	0.88015	-1.05050449	-0.0714307
1428126_a_at	ataxin 7-like 3B	Atxn7l3b	0.88015	-1.05398845	-0.0864272
1454094_at	RIKEN cDNA 4930471E19 gene	4930471E19Rik	0.88015	-1.05396508	-0.0798463
1439251_at	Iduronidase, alpha-L-	Idua	0.88015	1.08919431	0.10781617
1459563_x_at	---	---	0.88015	-1.05848308	-0.0829789
1430009_at	heat shock transcription factor, Y linked 2	Hsfy2	0.88015	1.08025056	0.09936783
1426480_at	Shwachman-Bodian-Diamond syndrome homolog (human)	Sbds	0.88015	1.04465266	0.06297399
1456480_at	furry homolog (Drosophila)	Fry	0.88016	1.07423979	0.0919655
1454741_s_at	transmembrane protein 164	Tmem164	0.88016	-1.05465863	-0.0791605
1416754_at	protein kinase, cAMP dependent regulatory, type I beta	Prkar1b	0.88016	-1.06207739	-0.0884439
1421373_at	cytochrome c oxidase subunit IV isoform 2	Cox4i2	0.88016	-1.06391172	-0.1211205
1441622_at	predicted gene 5485	Gm5485	0.88016	1.0645543	0.08609276
1457578_at	peroxisome proliferative activated receptor, gamma, coactivator	Ppargc1b	0.88017	-1.05206535	-0.0732309
1452921_at	ecotropic viral integration site 5 like /// EVI5-like protein-like	Evi5l /// LOC100503956	0.88017	-1.05406902	-0.0815179
1428937_at	ATPase, Ca++ transporting, plasma membrane 1	Atp2b1	0.88017	1.05563436	0.07181292
1430929_at	RIKEN cDNA 9430082L08 gene	9430082L08Rik	0.88017	1.05313768	0.07368156
1418710_at	CD59a antigen	Cd59a	0.88017	-1.05521122	-0.0817103
1427250_at	ATPase, Ca++ transporting, cardiac muscle, slow twitch 2	Atp2a2	0.88018	1.0452718	0.06076863
1429232_at	RIKEN cDNA 2610528B01 gene	2610528B01Rik	0.88018	-1.05774049	-0.1086592
1438060_at	neuronal PAS domain protein 3	Npas3	0.88018	1.06369719	0.08688477
1429797_at	atlastin GTPase 3	Atl3	0.88018	-1.06585798	-0.1094597
1435865_at	histone cluster 3, H2a /// tripartite motif-containing 17	Hist3h2a /// Trim17	0.88018	1.05694273	0.07389609
1416393_at	EMG1 nucleolar protein homolog (S. cerevisiae)	Emg1	0.88018	1.03428543	0.04848806
1453849_s_at	heterogeneous nuclear ribonucleoprotein A/B	Hnrnpab	0.88018	-1.0309444	-0.04445
1417204_at	KDEL (Lys-Asp-Glu-Leu) endoplasmic reticulum protein retention r	Kdelr2	0.88019	1.05459022	0.07023595
1445005_at	expressed sequence C79491	C79491	0.88019	-1.06008628	-0.0861474
1441177_at	---	---	0.88019	-1.05998303	-0.1229226
1434565_at	cell growth regulator with ring finger domain 1	Cgrrf1	0.88019	-1.06714166	-0.1326476
1440030_at	---	---	0.88019	-1.06082091	-0.0865577
1428148_s_at	coronin 7	Coro7	0.8802	1.06852447	0.08817007
1425008_a_at	interferon activated gene 203	Ifi203	0.88021	-1.05524558	-0.0790027
1433168_x_at	RIKEN cDNA 5830456J23 gene	5830456J23Rik	0.88021	-1.05772816	-0.0842876
1460675_at	immunoglobulin superfamily, member 8	Igsf8	0.88021	1.05107356	0.06880727
1452955_at	general transcription factor IIH, polypeptide 5	Gtf2h5	0.88021	-1.06309466	-0.1018318
1442523_at	predicted pseudogene 5124	Gm5124	0.88021	-1.05972899	-0.0883923
1438428_at	hypothetical protein LOC100504883	LOC100504883	0.88021	1.09921285	0.11369965
1443737_at	---	---	0.88021	1.0870662	0.10360703
1425876_a_at	glucuronyl C5-epimerase	Glce	0.88022	1.06195296	0.08513525
1449793_at	---	---	0.88022	-1.05944387	-0.0838792
1458773_at	---	---	0.88023	1.06435466	0.08684854
1445200_at	---	---	0.88023	-1.0592037	-0.0846172
1459054_at	castor homolog 1, zinc finger (Drosophila)	Casz1	0.88024	1.06505597	0.08721967
1429376_s_at	anaphase promoting complex subunit 10	Anapc10	0.88024	-1.04259717	-0.062677
1445057_at	---	---	0.88025	-1.05437646	-0.0800138
1433910_at	zinc finger, CCHC domain containing 6	Zcchc6	0.88026	1.05485216	0.07309926
1446731_at	Fanconi anemia, complementation group F	Fancf	0.88026	1.0676902	0.09148573
1440399_at	---	---	0.88027	1.04613165	0.06458276
1435491_at	zinc finger protein 669-like	LOC620419	0.88027	-1.06237291	-0.0955819
1441866_s_at	phosphatidylserine synthase 1	Ptdss1	0.88029	-1.04317649	-0.0622307
1435479_at	bone morphogenetic protein 7	Bmp7	0.88031	1.06532077	0.08767561
1452539_a_at	CD247 antigen	Cd247	0.88032	-1.04883935	-0.0710035
1452848_at	transmembrane protein 181A /// transmembrane protein 181B, p	Tmem181a /// Tmem181	0.88033	1.06500548	0.0846144
1458378_at	glutamate receptor ionotropic, NMDA3A	Grin3a	0.88033	-1.06160245	-0.0924117
1442545_at	---	---	0.88035	1.12650465	0.13058136
1429889_at	Fas apoptotic inhibitory molecule 3	Faim3	0.88036	-1.05942352	-0.0858581
1418275_a_at	E74-like factor 2	Elf2	0.88039	1.04871751	0.06570635
1422553_at	phosphatase and tensin homolog	Pten	0.8804	-1.05021116	-0.0872238

1430228_at	denticleless homolog (Drosophila)	Dtl	0.88042	1.05922303	0.08053627
1430353_at	GLIS family zinc finger 3	Glis3	0.88044	1.07157563	0.0912007
1430038_at	gephyrin	Gphn	0.88044	-1.06033191	-0.0962985
1436352_at	centrosomal protein 78	Cep78	0.88049	1.0488416	0.0680044
1451078_at	RIKEN cDNA 2510039O18 gene	2510039O18Rik	0.88056	-1.04278187	-0.0615938
1460671_at	glutathione peroxidase 1	Gpx1	0.88057	1.03456036	0.04765058
1431370_at	RIKEN cDNA 4930512B01 gene	4930512B01Rik	0.88058	1.06566306	0.08767172
1433463_at	RIKEN cDNA 0610010K06 gene	0610010K06Rik	0.88059	1.04225732	0.05938352
1449207_a_at	kinesin family member 20A	Kif20a	0.88059	1.04242536	0.05881111
1417088_at	zinc finger protein 346	Zfp346	0.88059	-1.04304754	-0.0617553
1426445_at	CTAGE family, member 5	Ctage5	0.88059	1.05358514	0.07063918
1444358_at	lysine (K)-specific demethylase 2A	Kdm2a	0.88059	-1.05952244	-0.0910623
1439449_at	special AT-rich sequence binding protein 1	Satb1	0.88059	1.0736116	0.09548232
1422733_at	four jointed box 1 (Drosophila)	Fjx1	0.8806	-1.05703912	-0.0876661
1445575_at	---	---	0.8806	1.04900004	0.0680716
1446012_at	---	---	0.8806	1.06810549	0.09097938
1438272_at	CUB and Sushi multiple domains 3	Csmd3	0.8806	-1.06446406	-0.1005685
1455991_at	cysteine conjugate-beta lyase 2	Ccbl2	0.88061	-1.04346667	-0.0617747
1424618_at	4-hydroxyphenylpyruvic acid dioxygenase	Hpd	0.88061	1.08376719	0.10198211
1426791_at	RUN and SH3 domain containing 2	Rusc2	0.88061	1.06373006	0.08161192
1444341_at	myosin binding protein C, slow-type	Mybpc1	0.88062	1.06804604	0.08959733
1456412_a_at	inositol polyphosphate 5-phosphatase K	Inpp5k	0.88062	1.05186114	0.07264711
1422626_at	matrix metallopeptidase 16	Mmp16	0.88062	-1.06382772	-0.0908843
1448841_at	pore forming protein-like	Pfpl	0.88063	1.09810818	0.11192066
1438499_at	RIKEN cDNA 1700029I15 gene	1700029I15Rik	0.88063	1.06752806	0.09076108
1426781_at	tRNA-yW synthesizing protein 1 homolog (S. cerevisiae)	Tyw1	0.88063	1.05574793	0.07725934
1420903_at	ST6 (alpha-N-acetyl-neuraminyl-2,3-beta-galactosyl-1,3)-N-acety	St6galnac3	0.88064	1.09238034	0.10797502
1434465_x_at	very low density lipoprotein receptor	Vldlr	0.88064	-1.04496143	-0.1948946
1420861_at	dynactin 4	Dctn4	0.88064	1.05514838	0.07328886
1450113_at	membrane protein, palmitoylated 5 (MAGUK p55 subfamily mem	Mpp5	0.88064	-1.05123618	-0.0769816
1438464_at	AT rich interactive domain 4A (RBP1-like)	Arid4a	0.88064	1.07482813	0.09319737
1421400_at	potassium large conductance calcium-activated channel, subfami	Kcnmb1	0.88064	-1.06592098	-0.1363814
1417634_at	superoxide dismutase 3, extracellular	Sod3	0.88064	-1.06053211	-0.0848127
1421612_a_at	H2A histone family, member Y2 /// H2A histone family, member	H2afy2 /// H2afy3	0.88064	-1.05019646	-0.0757938
1430672_at	RIKEN cDNA 5033418A18 gene	5033418A18Rik	0.88065	1.06576793	0.0877564
1445211_at	---	---	0.88065	1.0694549	0.09155452
1427205_x_at	coiled-coil domain containing 46	Ccdc46	0.88065	-1.06142312	-0.0878247
1424977_at	leucine rich repeat containing 67	Lrrc67	0.88065	1.05834129	0.07687847
1432915_at	RIKEN cDNA 5730407I07 gene	5730407I07Rik	0.88065	-1.06308059	-0.0995292
1448857_a_at	zinc finger, DHHC domain containing 12	Zdhhc12	0.88066	1.04056594	0.05731495
1419697_at	chemokine (C-X-C motif) ligand 11	Cxcl11	0.88066	1.07428464	0.0960348
1458726_at	expressed sequence C87033	C87033	0.88066	1.04624924	0.06458711
1444298_at	interaction protein for cytohesin exchange factors 1	Ipcef1	0.88066	-1.05340067	-0.083304
1445326_at	sidekick homolog 1 (chicken)	Sdk1	0.88067	1.06325473	0.08803902
1428233_at	cleavage and polyadenylation specific factor 6	Cpsf6	0.88067	-1.04580844	-0.0669701
1456111_at	family with sequence similarity 55, member D	Fam55d	0.88067	-1.06073783	-0.1059738
1445345_at	---	---	0.88068	1.06997201	0.09124518
1427383_at	Iroquois related homeobox 6 (Drosophila)	Irx6	0.88068	-1.06253718	-0.0879036
1459893_at	expressed sequence C76472	C76472	0.88068	1.0730063	0.09305999
1439391_at	family with sequence similarity 164, member C	Fam164c	0.88069	-1.06466119	-0.0906087
1422825_at	CART prepropeptide	Cartpt	0.88069	1.06947524	0.09184607
1432120_at	family with sequence similarity 82, member A1	Fam82a1	0.88069	1.06269735	0.0838473
1421929_at	Eph receptor A4	Epha4	0.88069	1.07719658	0.09286069
1455637_x_at	RIKEN cDNA B230307C23 gene	B230307C23Rik	0.8807	1.05455382	0.07425247
1440611_at	RIKEN cDNA 6230409E13 gene	6230409E13Rik	0.8807	-1.0561896	-0.0841123
1440472_at	sperm associated antigen 17, pseudogene	Spag17-ps	0.8807	1.08223256	0.09774587
1451384_at	jumonji domain containing 5	Jmjd5	0.88071	1.0576571	0.0786554
1430578_at	eukaryotic translation initiation factor 4 gamma, 3	Eif4g3	0.88072	1.06423221	0.08917641
1431701_a_at	PDZ domain containing 1	Pdzk1	0.88072	-1.05800002	-0.0846643
1425299_s_at	tRNA methyltransferase 11-2 homolog (S. cerevisiae)	Trmt112	0.88072	1.04466559	0.05922694
1426169_a_at	linker for activation of T cells family, member 2	Lat2	0.88073	-1.06099161	-0.0871988
1454789_x_at	PRP6 pre-mRNA splicing factor 6 homolog (yeast)	Prpf6	0.88073	1.08925701	0.10044612
1432656_at	RIKEN cDNA 3222402N08 gene	3222402N08Rik	0.88074	1.08761331	0.10460248
1427366_at	keratin associated protein 3-1	Krtap3-1	0.88074	-1.05075456	-0.0714976
1421628_at	interleukin 18 receptor 1	Il18r1	0.88075	1.05568601	0.07481881
1439733_at	hypothetical LOC100503882	LOC100503882	0.88076	-1.04242135	-0.0612639
1439106_at	zinc finger protein 462	Zfp462	0.88076	-1.04578512	-0.0710149
1459557_at	---	---	0.88079	1.07071602	0.09215801
1435961_at	N-acetyltransferase 14	Nat14	0.88079	-1.0617057	-0.1011136
1437951_at	DIS3 mitotic control homolog (S. cerevisiae)-like 2	Dis3l2	0.8808	1.06629046	0.08541154
1451600_s_at	carboxylesterase 3A /// carboxylesterase 3B	Ces3a /// Ces3b	0.88082	1.08095984	0.10183234
1432845_at	---	---	0.88085	-1.06122537	-0.0999885
1435355_at	nebulin	Neb	0.88089	1.05119789	0.07189123
1440271_at	---	---	0.88101	-1.06706396	-0.1111062
1428759_s_at	CWC25 spliceosome-associated protein homolog (S. cerevisiae)	Cwc25	0.88105	1.04938528	0.06825783
1422730_at	LIM domains containing 1	Limd1	0.88105	1.05714852	0.07906193
1450655_at	phosphatase and tensin homolog	Pten	0.88106	-1.04534908	-0.0710323
1456107_x_at	elongation factor Tu GTP binding domain containing 2	Eftud2	0.88108	-1.04591276	-0.0724338
1440049_at	---	---	0.88109	1.04469415	0.06012087
1450073_at	kinesin family member 3B	Kif3b	0.88112	-1.05609637	-0.0833241
1449350_at	odd-skipped related 1 (Drosophila)	Osr1	0.88129	-1.05867782	-0.0827243
1429142_at	RIKEN cDNA 1700012A03 gene	1700012A03Rik	0.88129	1.04075855	0.05707197
1436919_at	transformation related protein 53 inducible protein 11	Trp53i11	0.88129	1.06028606	0.07772246
1435685_x_at	ATP-binding cassette, sub-family C (CFTR/MRP), member 5	Abcc5	0.8813	1.06504707	0.08370644
1424151_at	aminoacyl tRNA synthetase complex-interacting multifunctional p	Aimp2	0.88131	1.04132824	0.05715711
1421551_s_at	interferon activated gene 202B	Ifi202b	0.88131	-1.05301961	-0.0771546
1448516_at	translin	Tsn	0.88131	-1.05341782	-0.0912974
1444139_at	DNA-damage-inducible transcript 4-like	Ddit4l	0.88131	1.05623255	0.07585439
1459029_at	P21 protein (Cdc42/Rac)-activated kinase 1	Pak1	0.88132	1.06274195	0.08625615
1430794_at	RIKEN cDNA 4933426K07 gene	4933426K07Rik	0.88132	-1.06280642	-0.1080633
1456349_x_at	SMT3 suppressor of mif two 3 homolog 1 (yeast)	Sumo1	0.88132	-1.03865802	-0.0556972
1448159_at	RAB7, member RAS oncogene family	Rab7	0.88133	1.05564645	0.07180455
1454520_at	RIKEN cDNA 5830415B17 gene	5830415B17Rik	0.88133	-1.05906302	-0.0860295
1420694_a_at	dachshund 1 (Drosophila)	Dach1	0.88133	1.05847986	0.08070008

1460344_at	pre-B-cell leukemia transcription factor interacting protein 1	Pbxip1	0.88133	-1.05507539	-0.0797432
1419085_at	Purkinje cell protein 2 (L7)	Pcp2	0.88134	-1.05934628	-0.0833752
1431181_a_at	Luc7 homolog (S. cerevisiae)-like	Luc7l	0.88134	1.04141033	0.05804713
1415860_at	karyopherin (importin) alpha 2	Kpna2	0.88135	-1.02874599	-0.0410403
1429352_at	molybdenum cofactor sulfurase	Mocos	0.88136	-1.05647774	-0.0820743
1450523_at	contactin 2	Cntn2	0.88136	-1.05566393	-0.0836736
1460255_at	tumor necrosis factor (ligand) superfamily, member 13b	Tnfsf13b	0.88136	-1.06030191	-0.0865539
1429124_s_at	REX1, RNA exonuclease 1 homolog (S. cerevisiae)	Rexo1	0.88138	1.04148638	0.05864078
1432309_at	RIKEN cDNA 9130410C08 gene	9130410C08Rik	0.88139	1.07741262	0.09919225
1430972_x_at	aquarius	Aqr	0.88139	-1.06304691	-0.0892329
1421338_at	E74-like factor 4 (ets domain transcription factor)	Elf4	0.88142	-1.05886784	-0.0832023
1448481_at	neuraminidase 1	Neu1	0.88143	1.0606095	0.08250247
1430322_at	ADP-ribosylation factor-like 13B	Arl13b	0.88145	1.11632946	0.1236442
1427295_at	solute carrier family 38, member 10	Slc38a10	0.88146	1.04999705	0.06932224
1451168_a_at	Rho GDP dissociation inhibitor (GDI) alpha	Arhgdia	0.88149	1.03476784	0.04907763
1446684_at	Ral GTPase activating protein, alpha subunit 1	Ralgapa1	0.88151	-1.05963124	-0.0998194
1442765_at	---	---	0.88155	1.06907195	0.09262175
1459230_at	RIKEN cDNA B430319G15 gene	B430319G15Rik	0.88162	-1.05558753	-0.0800996
1451023_at	hyperpolarization-activated, cyclic nucleotide-gated K+ 3	Hcn3	0.88163	-1.05413139	-0.0788285
1438567_at	von Willebrand factor A domain containing 2	Vwa2	0.88167	1.06291846	0.08471779
1433780_at	ubinuclein 1	Ubn1	0.88172	1.05232688	0.07312478
1448468_a_at	potassium voltage-gated channel, shaker-related subfamily, beta	Kcnab1	0.88174	-1.06230279	-0.100745
1416524_at	speckle-type POZ protein	Spop	0.88175	-1.04039727	-0.0577577
1444556_at	---	---	0.88179	-1.05937585	-0.0852677
1431497_at	RIKEN cDNA C330022C24 gene	C330022C24Rik	0.88182	-1.05848444	-0.0898733
1439344_at	---	---	0.88182	-1.05942733	-0.0838306
1449700_at	Immunoglobulin (CD79A) binding protein 1	Igbp1	0.88184	-1.05206494	-0.0743168
1439943_at	vacuolar protein sorting 54 (yeast)	Vps54	0.88184	-1.05317292	-0.0800743
1430795_at	RIKEN cDNA 5830407F19 gene	5830407F19Rik	0.88185	1.07142136	0.09269132
1443087_at	CDC23 (cell division cycle 23, yeast, homolog)	Cdc23	0.88187	-1.06600033	-0.1207584
1433968_a_at	multiple EGF-like-domains 9	Megf9	0.88187	1.05341137	0.07117978
1448308_at	adaptor-related protein complex 3, mu 1 subunit	Ap3m1	0.88194	1.04569836	0.06425148
1419208_at	mitogen-activated protein kinase kinase kinase 8	Map3k8	0.88195	-1.05684484	-0.0815433
1446401_at	Attractin like 1	Atrnl1	0.88197	1.05966153	0.08020557
1457798_at	RIKEN cDNA C130039O16 gene	C130039O16Rik	0.88198	1.06486704	0.08699354
1457553_at	---	---	0.88199	1.07290285	0.09499423
1436110_at	homer homolog 2 (Drosophila)	Homer2	0.882	-1.06196291	-0.0918701
1438433_at	WAS protein homolog associated with actin, golgi membranes an	Whamm	0.88201	-1.04804184	-0.1848105
1417626_at	phosphodiesterase 4D interacting protein (myomegalin)	Pde4dip	0.88202	1.06306693	0.08622561
1443626_at	---	---	0.88205	-1.05410512	-0.076554
1425731_at	ankyrin repeat domain 24	Ankrd24	0.88207	1.04543437	0.06371456
1424982_a_at	RIKEN cDNA 2700078E11 gene	2700078E11Rik	0.88207	1.04895429	0.06831149
1445304_at	mahogunin, ring finger 1	Mgrn1	0.88208	1.08306037	0.10169403
1442190_at	hypothetical LOC100503456	LOC100503456	0.88208	1.05802822	0.07495657
1457530_at	---	---	0.8821	-1.05931066	-0.0887558
1437990_x_at	hemoglobin Z, beta-like embryonic chain	Hbb-bh1	0.88211	1.05915868	0.08229537
1449978_at	zinc finger protein 2, Y linked	Zfy2	0.88211	1.07364096	0.09219778
1451912_a_at	fibroblast growth factor receptor-like 1	Fgfrl1	0.88211	1.06332718	0.08328839
1435371_x_at	carboxylesterase 1D	Ces1d	0.88211	1.05214776	0.07082096
1439383_x_at	protein phosphatase 2A, regulatory subunit B (PR 53)	Ppp2r4	0.88211	-1.06096619	-0.0899409
1416952_at	ATPase, H+ transporting, lysosomal V1 subunit D	Atp6v1d	0.88215	-1.04268467	-0.0604069
1436445_at	predicted gene 996	Gm996	0.88216	-1.06023092	-0.08618
1428892_at	peptidylprolyl isomerase (cyclophilin)-like 1	Ppil1	0.88216	1.03937292	0.05519552
1446248_at	solute carrier family 18 (vesicular monoamine), member 1	Slc18a1	0.88216	1.06962322	0.09068256
1450858_a_at	ubiquitin-conjugating enzyme E2D 3 (UBC4/5 homolog, yeast)	Ube2d3	0.88218	-1.04226366	-0.066106
1450769_s_at	StAR-related lipid transfer (START) domain containing 5	Stard5	0.88221	1.04963236	0.0698467
1424324_at	establishment of cohesion 1 homolog 1 (S. cerevisiae)	Esco1	0.88222	-1.04716123	-0.0666295
1440884_s_at	RIKEN cDNA A530047J11 gene	A530047J11Rik	0.88223	-1.05934552	-0.0955308
1422014_at	forkhead box P2	Foxp2	0.88224	-1.06128991	-0.0863239
1419528_at	sulfotransferase family 2A, dehydroepiandrosterone (DHEA)-pref	Sult2a2	0.88225	1.07540233	0.09567343
1455366_at	mitochondrial ribosomal protein L52	Mrpl52	0.88225	1.07191719	0.09406005
1428821_at	1-acylglycerol-3-phosphate O-acyltransferase 2 (lysophosphatidi	Agpat2	0.88226	1.05146436	0.0679956
1436257_at	synovial sarcoma translocation, Chromosome 18	Ss18	0.88227	-1.0647349	-0.0948471
1437881_at	RIKEN cDNA 6330416L07 gene	6330416L07Rik	0.88229	-1.06399937	-0.1070069
1455542_at	RIKEN cDNA C630043F03 gene	C630043F03Rik	0.88229	1.0966553	0.11125619
1456417_at	zinc finger protein of the cerebellum 4	Zic4	0.88233	1.07005331	0.09031451
1426588_at	tetraspanin 10	Tspan10	0.88234	1.06084936	0.08368333
1444507_at	ubiquitin specific peptidase 53	Usp53	0.88235	-1.05857794	-0.0908663
1436335_at	phospholipase C, eta 2	Plch2	0.88236	-1.0580167	-0.0879947
1434792_at	RIKEN cDNA 2010320M18 gene	2010320M18Rik	0.88239	1.06736397	0.08487679
1430315_at	RIKEN cDNA 1700010N08 gene	1700010N08Rik	0.8824	-1.05879187	-0.0876856
1460066_at	expressed sequence C80889	C80889	0.8824	-1.0586315	-0.0916744
1449536_at	potassium intermediate/small conductance calcium-activated cha	Kcnn1	0.88241	1.05918048	0.0824098
1442401_at	---	---	0.88241	-1.06301399	-0.1124701
1427736_a_at	chemokine (C-C motif) receptor-like 2	Ccrl2	0.88241	-1.05995298	-0.0857177
1426801_at	septin 8	Sep-08	0.88243	1.07519781	0.0904084
1445687_at	predicted gene 885	Gm885	0.88243	-1.06210581	-0.087548
1451419_at	splA/ryanodine receptor domain and SOCS box containing 4	Spsb4	0.88244	1.0940506	0.10889977
1450039_at	ubiquitin specific peptidase 9, X chromosome	Usp9x	0.88245	1.05384545	0.06943228
1447284_at	triggering receptor expressed on myeloid cells 1	Trem1	0.88245	-1.05859429	-0.082531
1431408_at	RIKEN cDNA 4930412B13 gene	4930412B13Rik	0.88245	1.08478564	0.10101698
1443708_at	cDNA sequence BC068281	BC068281	0.88247	1.08029846	0.09825679
1416559_at	ribosomal RNA processing 8, methyltransferase, homolog (yeast)	Rrp8	0.88248	1.04841912	0.06393939
1419220_at	xin actin-binding repeat containing 1	Xirp1	0.88249	-1.06054572	-0.0857804
1451836_at	transmembrane protein 150B	Tmem150b	0.88249	1.06098062	0.08425516
1417163_at	dual specificity phosphatase 10	Dusp10	0.8825	1.06003751	0.08392674
1427926_at	mastermind like 2 (Drosophila)	Maml2	0.8825	1.05758743	0.08045477
1440075_at	protein inhibitor of activated STAT 4	Pias4	0.8825	1.08575456	0.10315742
1455666_at	zinc finger protein 595	Zfp595	0.8825	1.05833289	0.08105299
1439790_at	serine (or cysteine) peptidase inhibitor, clade B, member 9	Serpinb9	0.8825	1.05670573	0.07916026
1434370_s_at	Fas-associated factor 1	Faf1	0.88251	-1.04458555	-0.0633723
1456879_at	RIKEN cDNA C130022K22 gene	C130022K22Rik	0.88251	1.05276906	0.07176906
1455750_at	Ral GTPase activating protein, alpha subunit 2 (catalytic)	Ralgapa2	0.88252	1.0854995	0.10039698
1458324_x_at	---	---	0.88252	-1.0597021	-0.1036466

1438508_at	---	---	0.88252	-1.06126255	-0.1214187
1460585_x_at	phosphatidylserine decarboxylase	Pisd	0.88253	1.04658724	0.0622667
1439136_at	---	---	0.88253	1.08954134	0.10376467
1420062_at	---	---	0.88253	-1.05788987	-0.0844804
1443443_at	---	---	0.88253	-1.06143639	-0.0939641
1456455_at	mortality factor 4 like 1	Morf4l1	0.88253	1.04781676	0.06560952
1444630_at	DNA segment, Chr 2, ERATO Doi 127, expressed	D2Ertd127e	0.88253	-1.06099965	-0.0993655
1421541_a_at	myocyte enhancer factor 2B	Mef2b	0.88253	-1.05380958	-0.0843607
1431385_a_at	membrane-bound transcription factor peptidase, site 1	Mbtps1	0.88255	-1.05439317	-0.0847161
1433105_at	RIKEN cDNA 5830438M01 gene	5830438M01Rik	0.88255	-1.06649461	-0.1028644
1439372_at	SMT3 suppressor of mif two 3 homolog 3 (yeast)	Sumo3	0.88255	1.06983119	0.09250328
1423093_at	inner centromere protein	Incenp	0.88255	-1.0453219	-0.0676487
1425618_at	DEAH (Asp-Glu-Ala-His) box polypeptide 9	Dhx9	0.88257	-1.05738638	-0.0864821
1425919_at	NADH dehydrogenase (ubiquinone) 1 alpha subcomplex, 12	Ndufa12	0.88257	1.06765829	0.08884425
1457986_at	---	---	0.88258	-1.06060657	-0.0869821
1449087_at	ring finger protein 141	Rnf141	0.88258	1.083307	0.0990106
1431237_at	nuclear receptor subfamily 2, group F, member 2	Nr2f2	0.88258	-1.05883141	-0.0839426
1427940_s_at	c-myc binding protein	Mycbp	0.88259	-1.05182348	-0.0752599
1445980_at	---	---	0.88259	-1.06157639	-0.0938973
1419833_s_at	ArfGAP with RhoGAP domain, ankyrin repeat and PH domain 3	Arap3	0.88259	1.07235189	0.09408454
1426663_s_at	solute carrier family 45, member 3	Slc45a3	0.8826	1.062734	0.08519307
1439786_at	growth factor receptor bound protein 2-associated protein 2	Gab2	0.8826	-1.06210144	-0.1002441
1454189_at	RIKEN cDNA 4930557J02 gene	4930557J02Rik	0.88261	1.08138513	0.10159944
1444547_at	ankyrin repeat domain 40	Ankrd40	0.88261	-1.05413313	-0.076719
1432967_at	RIKEN cDNA 4930456A14 gene	4930456A14Rik	0.88261	-1.06341133	-0.0927175
1423051_at	heterogeneous nuclear ribonucleoprotein U	Hnrnpu	0.88261	-1.03302633	-0.0483076
1433611_s_at	BUD31 homolog (yeast)	Bud31	0.88261	1.05122033	0.06678334
1423676_at	ATP synthase, H+ transporting, mitochondrial F0 complex, subunit	Atp5h	0.88262	1.03507365	0.04913
1442073_at	inositol polyphosphate-1-phosphatase	Inpp1	0.88262	-1.05675007	-0.0841182
1435455_at	intermediate filament family orphan 2	Iffo2	0.88262	1.05516972	0.07626164
1447927_at	guanylate-binding protein 10 /// macrophage activation 2 like	Gbp10 /// Mpa2l	0.88262	-1.05988769	-0.0843656
1451309_at	Rho GTPase activating protein 1	Arhgap1	0.88262	1.04962438	0.06906056
1439735_at	---	---	0.88263	-1.05383834	-0.076547
1443201_at	---	---	0.88263	-1.06145119	-0.0960185
1459713_s_at	anoctamin 1, calcium activated chloride channel	Ano1	0.88263	1.0800271	0.09785184
1452139_at	solute carrier family 35, member C1	Slc35c1	0.88263	1.06384672	0.08410835
1450761_s_at	regulating synaptic membrane exocytosis 2	Rims2	0.88264	1.05452524	0.07638897
1443308_at	unc-13 homolog B (C. elegans)	Unc13b	0.88264	1.06972189	0.09187027
1453891_at	polymerase I and transcript release factor	Ptrf	0.88264	1.07488708	0.09719117
1432745_at	RIKEN cDNA 9430092D12 gene	9430092D12Rik	0.88264	1.05899823	0.08160298
1444863_at	---	---	0.88265	1.06320943	0.08686054
1448768_at	myelin oligodendrocyte glycoprotein	Mog	0.88265	-1.05296105	-0.0783135
1449320_at	probasin	Pbsn	0.88265	1.05871796	0.07715633
1455561_at	carnosine dipeptidase 1 (metallopeptidase M20 family)	Cndp1	0.88266	-1.04953034	-0.0713106
1432197_at	RIKEN cDNA 4933417D19 gene	4933417D19Rik	0.88266	-1.0631029	-0.0975109
1451773_s_at	polymerase (RNA) III (DNA directed) polypeptide F	Polr3f	0.88266	-1.05123528	-0.0738753
1455617_at	LMBR1 domain containing 1	Lmbrd1	0.88266	1.06693247	0.08840998
1449552_at	zinc finger RNA binding protein	Zfr	0.88267	-1.01696806	-0.2175351
1457469_at	---	---	0.88267	-1.0629551	-0.0944213
1441931_x_at	glutathione synthetase	Gss	0.88267	1.05080706	0.06973869
1437970_at	HEAT repeat containing 2	Heatr2	0.88267	-1.05405639	-0.0841966
1448777_at	minichromosome maintenance deficient 2 mitotin (S. cerevisiae)	Mcm2	0.88268	1.03991082	0.05561065
1442336_at	---	---	0.88268	1.07974243	0.09860837
1421722_at	---	---	0.88268	1.06884766	0.08403018
1439135_at	---	---	0.88269	1.0703656	0.0920259
1453863_at	RIKEN cDNA 3300002P09 gene	3300002P09Rik	0.88269	1.08967884	0.10626094
1438512_at	cDNA sequence BC048679	BC048679	0.8827	-1.06113412	-0.0926923
1427560_at	sine oculis-related homeobox 5 homolog (Drosophila)	Six5	0.8827	1.06014244	0.08262719
1419755_at	antigen p97 (melanoma associated) identified by monoclonal ant	Mfi2	0.8827	1.06420763	0.08722535
1420914_at	solute carrier organic anion transporter family, member 2a1	Slco2a1	0.8827	-1.05933211	-0.0842018
1456700_x_at	myristoylated alanine rich protein kinase C substrate	Marcks	0.88271	1.04234311	0.05962806
1416203_at	aquaporin 1	Aqp1	0.88271	1.07707451	0.09715996
1443519_at	---	---	0.88271	-1.06324985	-0.1089064
1416380_at	Moloney leukemia virus 10	Mov10	0.88271	1.04655509	0.06498654
1429364_at	RIKEN cDNA 4930579G24 gene	4930579G24Rik	0.88271	1.05154072	0.07000109
1430444_at	RIKEN cDNA 0610006L08 gene	0610006L08Rik	0.88272	-1.06174725	-0.0971227
1449460_at	ankyrin repeat and SOCS box-containing 13	Asb13	0.88272	-1.05965909	-0.0872944
1458213_at	---	---	0.88272	1.07391797	0.09481181
1430523_s_at	immunoglobulin lambda chain, variable 1	Igl-V1	0.88272	-1.06211769	-0.087416
1433916_at	vesicle-associated membrane protein 3	Vamp3	0.88272	1.04731698	0.06372338
1431245_at	RIKEN cDNA 1700063H04 gene	1700063H04Rik	0.88273	1.07458473	0.09590405
1443526_at	---	---	0.88273	1.10883727	0.11768944
1417347_at	PYD and CARD domain containing	Pycard	0.88273	-1.06122415	-0.0880759
1422795_at	cullin 3	Cul3	0.88273	-1.04943419	-0.0771831
1442386_at	protein phosphatase 4, regulatory subunit 1-like, pseudogene	Ppp4r1l-ps	0.88273	1.06397052	0.08893681
1426535_at	protein O-glucosyltransferase 1	Poglut1	0.88274	1.04826938	0.06772958
1445194_at	connector enhancer of kinase suppressor of Ras 2	Cnksr2	0.88274	-1.05298563	-0.0757413
1416176_at	high mobility group box 1	Hmgb1	0.88274	-1.04100706	-0.0595045
1425203_at	DEAD (Asp-Glu-Ala-Asp) box polypeptide 19b	Ddx19b	0.88274	-1.06058208	-0.0939116
1426761_at	lysine (K)-specific demethylase 1A	Kdm1a	0.88275	1.04660682	0.0625978
1437252_at	GATS protein-like 2	Gatsl2	0.88275	-1.06161973	-0.0913334
1440858_at	cyclin-dependent kinase 12	Cdk12	0.88275	1.054972	0.07276826
1451388_a_at	ATPase, class VI, type 11B	Atp11b	0.88275	1.05998819	0.07394553
1452701_x_at	ubiquitin A-52 residue ribosomal protein fusion product 1	Uba52	0.88275	1.02443442	0.03431168
1450287_at	neuronal PAS domain protein 3	Npas3	0.88275	1.06955934	0.09047548
1437640_at	RIKEN cDNA 6430704M03 gene	6430704M03Rik	0.88276	1.06645861	0.08829399
1432203_at	transmembrane and coiled-coil domains 5B	Tmco5b	0.88276	1.05437417	0.07481582
1418694_at	potassium channel modulatory factor 1	Kcmf1	0.88276	1.05086944	0.06974138
1419507_at	keratin associated protein 15	Krtap15	0.88276	1.08324629	0.09964495
1425549_at	presenilin 1	Psen1	0.88277	-1.0632696	-0.1423681
1418574_a_at	split hand/foot malformation (ectrodactyly) type 1	Shfm1	0.88277	-1.0461043	-0.0703933
1457323_at	RIKEN cDNA C030030A07 gene	C030030A07Rik	0.88277	1.05879585	0.08205563
1451840_at	Kv channel interacting protein 4	Kcnip4	0.88277	-1.05047764	-0.0718637
1431430_s_at	tripartite motif-containing 59	Trim59	0.88278	-1.0579357	-0.1278231

1445042_at	---	---	0.88279	1.05961971	0.07997005
1422322_at	choroideremia-like	Chml	0.88279	-1.06364449	-0.1175816
1451563_at	EGF-like module containing, mucin-like, hormone receptor-like se	Emr4	0.8828	1.05712232	0.07892397
1421517_at	ST6 (alpha-N-acetyl-neuraminyl-2,3-beta-galactosyl-1,3)-N-acety	St6galnac1	0.88281	1.05607733	0.07717233
1416343_a_at	lysosomal-associated membrane protein 2	Lamp2	0.88282	-1.06261586	-0.0890944
1459297_at	---	---	0.88282	-1.06323079	-0.1040964
1424158_at	EH-domain containing 2	Ehd2	0.88282	1.08008325	0.09968766
1442622_at	---	---	0.88284	1.08021429	0.09796881
1442333_a_at	sodium channel, voltage-gated, type IX, alpha	Scn9a	0.88285	1.05795823	0.08011984
1449368_at	decorin	Dcn	0.88285	1.05920933	0.0818285
1453524_at	Kinesin family member 5B	Kif5b	0.88286	-1.06378871	-0.1109073
1422070_at	alcohol dehydrogenase 4 (class II), pi polypeptide	Adh4	0.88286	1.11737383	0.12431002
1447423_at	---	---	0.88286	1.06530551	0.08517942
1453631_at	syntaxin 8	Stx8	0.88287	1.07457892	0.09620471
1428901_at	DTW domain containing 2	Dtwd2	0.88287	-1.0536999	-0.0864745
1430358_at	beclin 1, autophagy related	Becn1	0.88288	-1.06173531	-0.0945404
1427015_at	NCK-associated protein 5-like	Nckap5l	0.88288	1.06781552	0.0864049
1449043_at	N-acetyl galactosaminidase, alpha	Naga	0.88289	-1.05658561	-0.0894411
1422047_at	cadherin 5	Cdh5	0.88289	-1.05891554	-0.0864928
1446481_at	---	---	0.88289	-1.06381738	-0.1098507
1450307_x_at	H2A histone family, member Y3	H2afy3	0.88289	-1.05444157	-0.0794202
1437728_at	alkB, alkylation repair homolog 5 (E. coli)	Alkbh5	0.88289	-1.04244482	-0.0630372
1428137_at	ADP-ribosylation factor-like 8B	Arl8b	0.8829	-1.05126868	-0.0840927
1421740_at	GNAS (guanine nucleotide binding protein, alpha stimulating) com	Gnas	0.8829	-1.05607787	-0.0790853
1446588_at	---	---	0.8829	-1.05645763	-0.0835179
1434709_at	neuron-glia-CAM-related cell adhesion molecule	Nrcam	0.8829	-1.05472436	-0.0820442
1431693_a_at	interleukin 17B	Il17b	0.88291	1.06408648	0.08235056
1456588_x_at	cytochrome c oxidase, subunit Vb	Cox5b	0.88291	1.03604439	0.04784286
1438522_at	POM121 membrane glycoprotein-like 12	Pom121l12	0.88292	-1.06229717	-0.0895958
1435856_x_at	SWI/SNF related, matrix associated, actin dependent regulator of	Smarcb1	0.88292	-1.04597683	-0.0736936
1450971_at	growth arrest and DNA-damage-inducible 45 beta	Gadd45b	0.88292	1.06957618	0.0840707
1447243_at	cDNA sequence BC040756	BC040756	0.88293	-1.0549544	-0.0792218
1425916_at	calpain 8	Capn8	0.88294	-1.05741251	-0.0852274
1440547_at	---	---	0.88295	1.0669452	0.08884468
1455307_at	family with sequence similarity 193, member A	Fam193a	0.88296	-1.05242731	-0.0806695
1427394_at	insulin-like growth factor 2, antisense	Igf2as	0.88296	1.07720375	0.09497885
1440358_at	Rho guanine nucleotide exchange factor (GEF) 15	Arhgef15	0.88297	-1.0517145	-0.0736878
1447473_at	---	---	0.88297	-1.05806436	-0.0867045
1430127_a_at	cyclin D2	Ccnd2	0.88299	-1.05724813	-0.0926548
1426347_at	RIKEN cDNA 2010321M09 gene	2010321M09Rik	0.88299	1.04720596	0.06474117
1442243_at	period homolog 3 (Drosophila)	Per3	0.88299	-1.05911561	-0.0859865
1423448_at	RAB11B, member RAS oncogene family	Rab11b	0.88299	1.04463335	0.06167728
1455000_at	G protein-coupled receptor 68	Gpr68	0.883	1.06626212	0.08849403
1447064_at	---	---	0.883	1.08421239	0.10141005
1434143_at	ATM interactor	Atmin	0.88301	1.04977394	0.06731488
1418481_at	protein kinase, membrane associated tyrosine/threonine 1	Pkmyt1	0.88301	1.05292574	0.07033945
1449131_s_at	CD1d1 antigen	Cd1d1	0.88301	-1.04987245	-0.0710097
1439507_at	TRAF type zinc finger domain containing 1	Trafd1	0.88301	-1.0603875	-0.0881138
1441370_at	Transmembrane and coiled coil domains 1	Tmcc1	0.88303	-1.0611736	-0.0887763
1456071_a_at	cytochrome c, somatic	Cycs	0.88303	-1.03921045	-0.0624543
1419965_at	---	---	0.88304	-1.06003388	-0.0898031
1415870_at	calumenin	Calu	0.88304	-1.04064866	-0.0581139
1427712_at	carcinoembryonic antigen-related cell adhesion molecule 1	Ceacam1	0.88305	1.10849092	0.11733569
1456750_at	RIKEN cDNA B230303O12 gene	B230303O12Rik	0.88306	1.06560871	0.08685626
1430167_a_at	RWD domain containing 3	Rwdd3	0.88306	-1.05911957	-0.0838106
1418978_at	SAP30 binding protein	Sap30bp	0.88306	1.05271673	0.07341321
1439949_at	Glycogen synthase kinase 3 beta	Gsk3b	0.88307	-1.05922577	-0.0839452
1452931_at	RIKEN cDNA 2810408M09 gene	2810408M09Rik	0.88307	-1.05301756	-0.0848993
1434933_at	RING CCCH (C3H) domains 1	Rc3h1	0.88307	-1.04625847	-0.0656124
1421107_at	serine/threonine kinase 4	Stk4	0.88307	-1.05437827	-0.0773883
1433817_at	1-acylglycerol-3-phosphate O-acyltransferase 3	Agpat3	0.88308	1.0488653	0.0687856
1419696_at	CD4 antigen	Cd4	0.88308	-1.05629846	-0.0835882
1446038_at	Chemokine (C-C motif) receptor 9	Ccr9	0.88308	1.08996268	0.10614874
1446938_at	expressed sequence AA408213	AA408213	0.88308	1.0635905	0.08597483
1458574_at	---	---	0.88309	-1.06044882	-0.0882761
1447197_at	expressed sequence C86544	C86544	0.88309	1.04655736	0.06424032
1434027_at	regulator of calcineurin 3	Rcan3	0.8831	-1.05267542	-0.0817562
1441143_at	RIKEN cDNA E130309D14 gene	E130309D14Rik	0.8831	1.0715441	0.09171533
1419009_at	actin-like 7a	Actl7a	0.88311	1.07813726	0.09797328
1455453_at	gem (nuclear organelle) associated protein 5	Gemin5	0.88311	1.05820518	0.07826287
1451684_a_at	bicaudal D homolog 1 (Drosophila)	Bicd1	0.88311	-1.06240406	-0.1130496
1458397_at	predicted gene 6297	Gm6297	0.88311	-1.05782351	-0.0841709
1426267_at	zinc finger and BTB domain containing 8 opposite strand	Zbtb8os	0.88312	1.06304417	0.08686679
1429940_at	cell division cycle 73, Paf1/RNA polymerase II complex componer	Cdc73	0.88312	-1.05409472	-0.077657
1439335_at	---	---	0.88313	1.06444078	0.08614285
1449662_at	---	---	0.88314	1.04939541	0.06912426
1455722_at	---	---	0.88316	-1.04971216	-0.0723663
1455086_at	Ubiquitin carboxyl-terminal esterase L5	Uchl5	0.88316	-1.06008962	-0.0870592
1452907_at	galactosylceramidase	Galc	0.88323	-1.06064772	-0.1121387
1454518_at	RIKEN cDNA 4930429L21 gene	4930429L21Rik	0.8833	-1.05411156	-0.0763817
1418841_s_at	cyclin-dependent kinase 11B	Cdk11b	0.88332	1.04583229	0.06159223
1436926_at	estrogen related receptor, beta	Esrrb	0.88333	1.04047104	0.05665074
1444659_at	---	---	0.88334	-1.05655708	-0.0806802
1457079_at	RIKEN cDNA 6720457D02 gene	6720457D02Rik	0.88334	-1.05707177	-0.0811231
1426688_at	succinate dehydrogenase complex, subunit A, flavoprotein (Fp)	Sdha	0.88335	1.03970822	0.05604844
1459501_at	expressed sequence AU022194	AU022194	0.88335	-1.06225456	-0.0902609
1445937_at	---	---	0.88336	-1.06106574	-0.0868517
1431161_at	Rho-guanine nucleotide exchange factor	Rgnef	0.88337	-1.05904723	-0.0837181
1434971_x_at	mitochondrial ribosomal protein L15	Mrpl15	0.88337	-1.03663489	-0.0548941
1421508_at	odd Oz/ten-m homolog 1 (Drosophila)	Odz1	0.88338	-1.06056585	-0.0877156
1445850_at	protein phosphatase 1, regulatory (inhibitor) subunit 12B	Ppp1r12b	0.88338	1.06606955	0.08427323
1424840_at	ribokinase	Rbks	0.88338	-1.05736125	-0.0867455
1434019_at	PDGFA associated protein 1	Pdap1	0.88338	1.0432093	0.05674762
1435787_at	protein phosphatase 1 (formerly 2C)-like	Ppm1l	0.88339	-1.05387386	-0.0796113

1433881_at	DnaJ (Hsp40) homolog, subfamily C, member 11	Dnajc11	0.88339	1.05207901	0.0717051
1447410_at	---	---	0.8834	1.06212232	0.0806554
1457500_at	DEP domain containing 5	Depdc5	0.8834	1.06764237	0.09048271
1422722_at	RIKEN cDNA 1700001K19 gene	1700001K19Rik	0.88341	-1.06122723	-0.0935416
1458867_at	EGF-like repeats and discoidin I-like domains 3	Edil3	0.88341	1.04695379	0.06590626
1422941_at	wingless-related MMTV integration site 16	Wnt16	0.88341	-1.05798941	-0.0824478
1423886_at	laminin, gamma 1	Lamc1	0.88342	-1.05586465	-0.0856338
1456055_x_at	polymerase (DNA directed), delta 1, catalytic subunit	Pold1	0.88343	1.05255411	0.06898423
1457764_at	RIKEN cDNA A530058O07 gene	A530058O07Rik	0.88343	-1.05837205	-0.0885673
1430310_at	tetraspanin 11	Tspan11	0.88344	-1.05873032	-0.0855675
1453869_at	predicted gene 5085	Gm5085	0.88344	1.06818285	0.08943426
1449865_at	sema domain, immunoglobulin domain (Ig), short basic domain, s	Sema3a	0.88345	1.08149878	0.0983765
1439555_at	rearranged L-myc fusion sequence	Rlf	0.88346	-1.04745557	-0.1987389
1417921_at	RIKEN cDNA 2610029G23 gene	2610029G23Rik	0.88348	-1.0381512	-0.054042
1416443_a_at	SUMO1 activating enzyme subunit 1	Sae1	0.88348	1.03697388	0.05114759
1425693_at	Braf transforming gene	Braf	0.88348	-1.05933414	-0.1025967
1429692_s_at	GTP cyclohydrolase 1	Gch1	0.88358	1.10470559	0.11822173
1445404_at	kinesin family member 27	Kif27	0.88358	1.0620092	0.08381499
1454164_at	RIKEN cDNA 4921523L03 gene	4921523L03Rik	0.88359	-1.05889099	-0.0855696
1425192_at	kelch-like 25 (Drosophila)	Klhl25	0.88359	1.05796814	0.07968244
1447255_at	RIKEN cDNA 2310015A10 gene	2310015A10Rik	0.8836	-1.06055837	-0.0938822
1439413_x_at	mortality factor 4 like 2	Morf4l2	0.88364	-1.03263195	-0.0484291
1445024_at	START domain containing 7	Stard7	0.88364	1.05507614	0.07733291
1429106_at	RIKEN cDNA 4921509J17 gene /// heat shock protein 4 like	4921509J17Rik /// Hspa4	0.88365	-1.05640144	-0.0800361
1456181_at	WD repeat domain 91	Wdr91	0.88365	1.06634069	0.08561147
1439626_at	tropomodulin 3	Tmod3	0.88367	1.04885546	0.06537483
1424244_at	RWD domain containing 4A	Rwdd4a	0.88368	1.04712258	0.06542627
1438201_at	pyruvate dehyrogenase phosphatase catalytic subunit 1	Pdp1	0.88368	-1.06230903	-0.1068054
1419047_at	pecanex homolog (Drosophila)	Pcnx	0.88371	1.05415527	0.07576176
1438654_x_at	monocyte to macrophage differentiation-associated 2	Mmd2	0.88371	1.05992466	0.08312099
1433882_at	CCR4-NOT transcription complex, subunit 10	Cnot10	0.88371	1.04306423	0.06032471
1455875_x_at	transmembrane 9 superfamily member 2	Tm9sf2	0.88372	-1.04309117	-0.065074
1436397_at	TRAF-interacting protein with forkhead-associated domain, family	Tifab	0.88373	1.06419316	0.08546211
1426523_a_at	glucosamine-6-phosphate deaminase 2	Gnpda2	0.88373	-1.04983209	-0.0714417
1423378_at	a disintegrin and metallopeptidase domain 23	Adam23	0.88373	-1.04290375	-0.0612897
1448175_at	EH-domain containing 1	Ehd1	0.88374	1.05190463	0.06733942
1459836_x_at	splicing factor 3b, subunit 2	Sf3b2	0.88374	-1.05046482	-0.0720772
1442421_at	---	---	0.88374	-1.06058881	-0.0863616
1445332_at	---	---	0.88375	-1.0572012	-0.083257
1452422_a_at	U2 small nuclear ribonucleoprotein B	Snrpb2	0.88375	-1.03735206	-0.0529844
1424482_at	Rho guanine nucleotide exchange factor (GEF7)	Arhgef7	0.88377	1.06885007	0.08988839
1419996_s_at	aryl hydrocarbon receptor nuclear translocator	Arnt	0.88377	-1.04512616	-0.0655806
1436157_at	cell division cycle and apoptosis regulator 1	Ccar1	0.88378	-1.04319646	-0.0663985
1422312_a_at	neurogenin 3	Neurog3	0.8838	1.07425805	0.09341856
1459331_at	surfeit gene 6	Surf6	0.88383	1.0945771	0.10911069
1432069_at	RIKEN cDNA 1700023G09 gene	1700023G09Rik	0.88384	1.0591336	0.08168254
1441074_at	---	---	0.88387	1.0901599	0.10658125
1455680_at	RIKEN cDNA 9630025H16 gene /// karyopherin (importin) alpha 4	9630025H16Rik /// Kpna4	0.88388	-1.04615707	-0.0700255
1434699_at	G2/M-phase specific E3 ubiquitin ligase	G2e3	0.88389	-1.04130378	-0.061245
1449827_at	aggrecan	Acan	0.8839	-1.05546612	-0.0823418
1439572_at	---	---	0.88391	1.07934084	0.09708103
1421084_at	retinoschisis (X-linked, juvenile) 1 (human)	Rs1	0.88391	1.062932	0.08236519
1452110_at	5-methyltetrahydrofolate-homocysteine methyltransferase redu	Mtrr	0.88392	-1.0470957	-0.0688917
1440368_at	---	---	0.88392	1.06292713	0.08546067
1440590_at	---	---	0.88393	-1.06449267	-0.1136835
1430291_at	dedicator of cytokinesis 5	Dock5	0.88411	-1.05490965	-0.0852879
1435468_at	UDP-GlcNAc:betaGal beta-1,3-N-acetylglucosaminyltransferase 9	B3gnt9-ps	0.88411	-1.06216262	-0.1094717
1427529_at	frizzled homolog 9 (Drosophila)	Fzd9	0.88411	1.05136704	0.07202924
1438268_at	ring finger and CCCH-type zinc finger domains 2	Rc3h2	0.88414	-1.05482548	-0.1265785
1433192_at	RIKEN cDNA A430105P17 gene	A430105P17Rik	0.88416	1.04912409	0.0661081
1429725_at	zinc finger homeobox 3	Zfhx3	0.88433	-1.05394866	-0.0764325
1439791_at	predicted gene 527	Gm527	0.88436	-1.0532105	-0.0754208
1453413_at	GNAS (guanine nucleotide binding protein, alpha stimulating) com	Gnas	0.88437	-1.05951151	-0.0837492
1450503_at	potassium inwardly-rectifying channel, subfamily J, member 2	Kcnj2	0.8844	1.06069639	0.0799177
1421346_a_at	solute carrier family 6 (neurotransmitter transporter, taurine), me	Slc6a6	0.88453	-1.05207033	-0.1126255
1448264_a_at	eukaryotic translation initiation factor 3, subunit I	Eif3i	0.88454	1.03578856	0.04966414
1460071_at	---	---	0.88455	-1.06088661	-0.0900333
1449375_at	carboxylesterase 2A	Ces2a	0.88456	1.06592203	0.08638572
1432861_at	RIKEN cDNA 2900046F13 gene	2900046F13Rik	0.88456	-1.05846056	-0.0888991
1429921_at	RIKEN cDNA 9530068E07 gene	9530068E07Rik	0.88456	-1.058446	-0.0825247
1422802_at	defensin, alpha, 3	Defa3	0.88457	1.04952298	0.06968676
1447819_x_at	collagen, type VIII, alpha 1	Col8a1	0.88458	-1.05779052	-0.1004906
1458856_at	---	---	0.88458	-1.0492737	-0.0717939
1417978_at	eukaryotic translation initiation factor 4E member 3	Eif4e3	0.88458	1.06027902	0.08310564
1420762_a_at	Y box protein 2	Ybx2	0.88459	1.06757203	0.08575926
1419902_at	---	---	0.88459	1.05859181	0.07752888
1439723_at	RIKEN cDNA 5730590G19 gene	5730590G19Rik	0.8846	-1.05483614	-0.0814798
1429997_at	RIKEN cDNA 4832441B07 gene	4832441B07Rik	0.88461	-1.05856308	-0.0843591
1437247_at	fos-like antigen 2	Fosl2	0.88461	-1.0601568	-0.1145844
1432169_at	RIKEN cDNA 4930523O13 gene	4930523O13Rik	0.88462	-1.06095836	-0.0873727
1452934_at	transmembrane channel-like gene family 5	Tmc5	0.88462	-1.06276108	-0.0976261
1453555_at	RIKEN cDNA 3830408D07 gene	3830408D07Rik	0.88462	-1.05751673	-0.1077033
1433136_at	RIKEN cDNA 4930432H08 gene	4930432H08Rik	0.88463	-1.0622151	-0.0879196
1441770_at	phosphoribosyl pyrophosphate amidotransferase	Ppat	0.88463	1.08989967	0.10497195
1442950_at	---	---	0.88463	-1.05919705	-0.0925937
1450731_s_at	tumor necrosis factor receptor superfamily, member 21	Tnfrsf21	0.88463	-1.05133434	-0.0870847
1435314_at	tryptophan hydroxylase 2	Tph2	0.88464	1.05206182	0.0720424
1444597_at	predicted gene 6590	Gm6590	0.88464	1.04659128	0.06384194
1441710_at	---	---	0.88464	-1.05841364	-0.0888091
1417053_at	prohibitin	Phb	0.88464	1.03619236	0.05123816
1419151_at	RIKEN cDNA 4930449I24 gene /// predicted gene 3402 /// predic	4930449I24Rik /// Gm340	0.88465	-1.05806363	-0.0890804
1460120_at	actin-binding LIM protein 1	Ablim1	0.88465	-1.04834336	-0.0682644
1437718_x_at	fibromodulin	Fmod	0.88465	-1.06434122	-0.1052591
1427385_s_at	actinin, alpha 1	Actn1	0.88466	-1.04467899	-0.1573466

1442327_at	RIKEN cDNA 2410141K09 gene /// zinc finger protein 640	2410141K09Rik /// Zfp64(	0.88467	1.06930508	0.08996845
1419045_at	solute carrier family 25 (mitochondrial carrier; phosphate carrier),	Slc25a23	0.88467	1.07148304	0.08836726
1443884_at	thyroid adenoma associated	Thada	0.88467	-1.05692118	-0.0944821
1459762_x_at	WD repeat domain 12	Wdr12	0.88467	-1.0508578	-0.0731226
1442602_at	---	---	0.88468	-1.06417442	-0.1175659
1440970_at	kalirin, RhoGEF kinase	Kalrn	0.88468	-1.06138181	-0.0885602
1416391_at	pentatricopeptide repeat domain 1	Ptcd1	0.88468	-1.05899389	-0.1092283
1416385_a_at	mannose-6-phosphate receptor, cation dependent	M6pr	0.88471	-1.04150766	-0.0593795
1449309_at	cytochrome P450, family 8, subfamily b, polypeptide 1	Cyp8b1	0.88471	-1.06237639	-0.0922317
1437283_at	transportin 2 (importin 3, karyopherin beta 2b)	Tnpo2	0.88471	1.06191487	0.07647091
1450302_at	RIKEN cDNA 4930470P17 gene	4930470P17Rik	0.88471	1.06335882	0.08616237
1459560_at	speckle-type POZ protein-like	Spopl	0.88471	-1.05980244	-0.0903148
1439981_at	RIKEN cDNA 5830405N20 gene	5830405N20Rik	0.88472	1.09215637	0.10657512
1448812_at	hippocalcin-like 1	Hpcal1	0.88472	1.04526848	0.06366503
1423860_at	prostaglandin D2 synthase (brain)	Ptgds	0.88473	1.06977748	0.09129523
1452698_at	Ts translation elongation factor, mitochondrial	Tsfm	0.88473	1.04270193	0.05878734
1431728_at	chromatin modifying protein 4C	Chmp4c	0.88473	-1.06349012	-0.1326473
1431301_at	proline rich 15	Prr15	0.88474	-1.05638258	-0.0807387
1418196_at	telomerase associated protein 1	Tep1	0.88474	-1.05081109	-0.0826715
1437336_x_at	prickle homolog 4 (Drosophila) /// translocase of outer mitochond	Prickle4 /// Tomm6	0.88475	1.04502047	0.05834377
1452115_a_at	polo-like kinase 4 (Drosophila)	Plk4	0.88475	-1.05489507	-0.0837283
1431372_at	serine/arginine-rich protein specific kinase 2	Srpk2	0.88475	1.09435526	0.10616807
1417372_a_at	pellino 1	Peli1	0.88475	-1.04563943	-0.0692533
1444523_s_at	ubiquitin-conjugating enzyme E2 variant 1	Ube2v1	0.88475	1.0401105	0.05559539
1431717_at	A kinase (PRKA) anchor protein 5	Akap5	0.88477	-1.05277383	-0.0806609
1430871_at	phospholipase C, beta 4	Plcb4	0.88477	1.08067762	0.09919551
1440834_at	solute carrier family 5 (sodium/glucose cotransporter), member 1	Slc5a10	0.88478	-1.05453163	-0.0790767
1436153_a_at	zinc finger, MYND domain containing 11	Zmynd11	0.88478	1.06982055	0.08478905
1429203_at	RIKEN cDNA 2410076I21 gene	2410076I21Rik	0.88479	1.07581885	0.09126862
1440887_at	RIKEN cDNA C330005M16 gene	C330005M16Rik	0.88479	1.07570391	0.09549993
1426481_at	kelch-like 22 (Drosophila)	Klhl22	0.88479	1.04055404	0.05712131
1432348_at	RIKEN cDNA 4930524O07 gene	4930524O07Rik	0.8848	-1.05554359	-0.0786314
1423569_at	glycine amidinotransferase (L-arginine:glycine amidinotransferas	Gatm	0.88482	1.07975277	0.09821314
1429070_at	nuclear apoptosis inducing factor 1	Naif1	0.88482	1.05990807	0.08088876
1438043_at	---	---	0.88483	1.07511698	0.09458697
1442184_at	---	---	0.88484	-1.05695904	-0.0865587
1429349_at	high-mobility group box 4	Hmgb4	0.88485	-1.05917454	-0.1136499
1424778_at	receptor accessory protein 3	Reep3	0.88485	-1.0499247	-0.071648
1459735_at	FK506 binding protein 15	Fkbp15	0.88486	-1.05803937	-0.0830419
1421004_at	centrosomal protein 110	Cep110	0.88486	-1.0566938	-0.0807851
1443488_at	---	---	0.88487	1.05072794	0.06939456
1417121_at	gamma-aminobutyric acid (GABA) A receptor, subunit alpha 6	Gabra6	0.88488	1.070276	0.09092675
1441720_at	RIKEN cDNA 4921530L18 gene	4921530L18Rik	0.88488	-1.06168498	-0.1012753
1451527_at	procollagen C-endopeptidase enhancer 2	Pcolce2	0.88488	1.06000592	0.07368773
1458637_x_at	---	---	0.88489	1.10638915	0.11647016
1431819_at	---	---	0.8849	-1.05800205	-0.08164
1424667_a_at	cut-like homeobox 1	Cux1	0.8849	1.07214264	0.08898635
1431704_a_at	Ral GEF with PH domain and SH3 binding motif 2	Ralgps2	0.8849	1.05926743	0.07967881
1455491_at	heterogeneous nuclear ribonucleoprotein H3	Hnrnph3	0.88491	1.04325467	0.05991784
1441075_at	nitric oxide synthase trafficker	Nostrin	0.88491	1.06626216	0.08768578
1458331_x_at	carboxylesterase 5A	Ces5a	0.88491	-1.05954161	-0.0874883
1459744_at	CD53 antigen	Cd53	0.88492	1.05367802	0.07522574
1423776_s_at	TBC1 domain family, member 22a	Tbc1d22a	0.88492	1.04448337	0.06133877
1416828_at	synaptosomal-associated protein 25	Snap25	0.88493	-1.05211294	-0.0760628
1453796_a_at	ERGIC and golgi 2	Ergic2	0.88494	-1.04734523	-0.0714243
1444807_at	---	---	0.88494	-1.05755899	-0.0815265
1453951_a_at	DNA segment, Chr 19, ERATO Doi 652, expressed	D19Ertd652e	0.88495	1.0775554	0.09379365
1446828_at	---	---	0.88495	1.05794564	0.08099771
1419464_at	WD repeat domain 86	Wdr86	0.88495	1.05657082	0.07534687
1418469_at	nuclear receptor interacting protein 1	Nrip1	0.88495	-1.06039735	-0.094062
1418962_at	NECAP endocytosis associated 2	Necap2	0.88495	1.04763493	0.06484099
1447829_x_at	TAF9B RNA polymerase II, TATA box binding protein (TBP)-associ	Taf9b	0.88495	1.0460451	0.06364815
1423199_at	bromodomain containing 3	Brd3	0.88495	-1.05512592	-0.0869014
1421952_at	calpain 6	Capn6	0.88496	1.07968438	0.09878752
1434880_at	ets variant gene 6 (TEL oncogene)	Etv6	0.88496	1.05327124	0.07373698
1453859_at	ubiquinol-cytochrome c reductase complex chaperone, CBP3 hom	Uqcc	0.88497	1.04980873	0.06807934
1439169_at	---	---	0.88498	1.09256938	0.10795476
1423822_a_at	transmembrane protein 168	Tmem168	0.88498	1.05882409	0.07809015
1442036_a_at	---	---	0.88499	-1.059035	-0.0846366
1420437_at	indoleamine 2,3-dioxygenase 1	Ido1	0.88499	-1.05018912	-0.0744856
1440397_at	calcium channel, voltage-dependent, alpha2/delta subunit 1	Cacna2d1	0.885	1.06012431	0.07462084
1452131_at	U2 small nuclear ribonucleoprotein B	Snrpb2	0.88501	1.0571631	0.08011103
1437782_at	contactin associated protein-like 2	Cntnap2	0.88502	-1.05627693	-0.0833116
1420872_at	guanylate cyclase 1, soluble, beta 3	Gucy1b3	0.88502	1.0966214	0.10989031
1423601_s_at	Treacher Collins Franceschetti syndrome 1, homolog	Tcof1	0.88502	-1.04759223	-0.0815862
1424593_at	ecdysoneless homolog (Drosophila)	Ecd	0.88502	-1.04921995	-0.0697489
1418087_at	ubiquitin fusion degradation 1 like	Ufd1l	0.88506	1.04720671	0.06582638
1431784_a_at	ribosome production factor 1 homolog (S. cerevisiae)	Rpf1	0.88507	1.04004062	0.05642395
1433489_s_at	fibroblast growth factor receptor 2	Fgfr2	0.88508	1.05425748	0.07591532
1453454_at	RIKEN cDNA 1700012E03 gene	1700012E03Rik	0.88509	-1.05905074	-0.0839582
1419473_a_at	cholecystokinin	Cck	0.8851	1.06267113	0.08379005
1460416_s_at	component of Sp100-rs /// predicted gene 7592 /// predicted pse	Csprs /// Gm7592 /// Gm	0.88513	-1.05916514	-0.0836846
1456783_at	zinc finger, DBF-type containing 2	Zdbf2	0.88513	1.05178218	0.06823555
1451730_at	zinc finger protein 62	Zfp62	0.88513	-1.05322349	-0.0819183
1458417_at	KDM3B lysine (K)-specific demethylase 3B	Kdm3b	0.88513	1.07506683	0.08943992
1434503_s_at	lysosomal-associated membrane protein 2	Lamp2	0.88514	-1.04298111	-0.0620309
1427811_at	thymidylate synthase	Tyms	0.88514	1.04830729	0.06723392
1457752_at	expressed sequence AU014876	AU014876	0.88515	-1.06142907	-0.0958595
1460712_s_at	adaptor protein complex AP-1, gamma 1 subunit	Ap1g1	0.88515	1.04319585	0.05964405
1451724_at	ankyrin repeat and MYND domain containing 2	Ankmy2	0.88515	1.05494272	0.07452193
1440107_at	---	---	0.88515	-1.05865768	-0.0839996
1428450_at	RIKEN cDNA 2610034B18 gene	2610034B18Rik	0.88515	-1.05417362	-0.0775963
1419039_at	cytochrome P450, family 2, subfamily d, polypeptide 22	Cyp2d22	0.88516	1.0585178	0.08087952
1421695_at	stereocilin	Strc	0.88516	-1.05795868	-0.0901539

1447890_at	reticulocalbin 1	Rcn1	0.88519	1.09047102	0.10442096
1423261_at	RIKEN cDNA 1500015O10 gene	1500015O10Rik	0.8852	-1.05920813	-0.0839914
1443275_at	---	---	0.88522	1.05836755	0.08099031
1419296_at	Rho GTPase activating protein 4	Arhgap4	0.88522	1.0787011	0.09785981
1448756_at	S100 calcium binding protein A9 (calgranulin B)	S100a9	0.88526	1.07399455	0.09232191
1458698_at	---	---	0.88527	-1.06233458	-0.0993961
1433902_at	kelch repeat and BTB (POZ) domain containing 8	Kbtbd8	0.88528	-1.04956073	-0.075107
1441675_at	---	---	0.8853	-1.05086569	-0.0725409
1446365_at	vesicle transport through interaction with t-SNAREs homolog 1A (	Vti1a	0.88531	-1.06082989	-0.1032317
1458636_at	ring finger protein 219	Rnf219	0.88532	-1.05781528	-0.0842323
1422808_s_at	dedicator of cyto-kinesis 2	Dock2	0.88533	-1.06037938	-0.0867207
1446098_at	---	---	0.88533	1.06224099	0.08476334
1443262_at	mitochondrial ribosomal protein S14	Mrps14	0.88533	-1.06286717	-0.1086787
1449918_at	CD209g antigen	Cd209g	0.88533	1.05911134	0.08058882
1449546_a_at	zinc finger protein 617	Zfp617	0.88534	-1.0478513	-0.0746074
1420605_at	metastasis associated gene 2	Mtag2	0.88535	1.0758889	0.09520248
1452691_at	RNA binding motif protein 17	Rbm17	0.88535	-1.03763912	-0.0541439
1438456_at	histocompatibility 13	H13	0.88536	-1.05904824	-0.0906237
1415796_at	DAZ associated protein 2	Dazap2	0.88536	1.04072492	0.05698809
1435015_at	zinc finger protein 787	Zfp787	0.88536	-1.05770217	-0.1126958
1440893_at	RIO kinase 1 (yeast)	Riok1	0.88537	-1.04645034	-0.066883
1446968_at	phosphatidylinositol binding clathrin assembly protein	Picalm	0.88537	-1.06250111	-0.1364196
1429950_at	unc-5 homolog C (C. elegans)-like	Unc5cl	0.88537	-1.06199838	-0.1038282
1433898_at	---	---	0.88538	1.05312771	0.07033229
1451632_a_at	Immunoglobulin heavy chain (gamma polypeptide)	Ighg	0.88538	1.07043508	0.08932848
1446083_at	---	---	0.88539	-1.06206749	-0.1155278
1441649_at	---	---	0.8854	1.07508895	0.09410247
1454388_at	RIKEN cDNA 2900046F13 gene	2900046F13Rik	0.8854	1.08010024	0.09763642
1429161_at	ARV1 homolog (yeast)	Arv1	0.8854	1.05453902	0.07340917
1437752_at	lin-28 homolog A (C. elegans)	Lin28a	0.88541	-1.04875295	-0.0893487
1418121_at	vaccinia related kinase 3	Vrk3	0.88542	1.04231124	0.05896036
1429413_at	carboxypeptidase M	Cpm	0.88542	-1.06213627	-0.0929335
1425256_a_at	DIX domain containing 1	Dixdc1	0.88543	1.0697762	0.08925546
1447995_x_at	small nucleolar RNA host gene (non-protein coding) 10	Snhg10	0.88543	1.06482727	0.0818503
1423313_at	phosphodiesterase 7A	Pde7a	0.88543	1.05044703	0.07037321
1424098_at	ELOVL family member 7, elongation of long chain fatty acids (yea	Elovl7	0.88545	-1.05841721	-0.0881742
1451345_at	methylthioadenosine phosphorylase	Mtap	0.88545	-1.04512104	-0.064856
1425485_at	myotubularin related protein 6	Mtmr6	0.88545	1.04751631	0.06599584
1449585_at	interleukin 1 receptor accessory protein	Il1rap	0.88545	-1.05939049	-0.0864949
1426522_at	hydroxyacyl-Coenzyme A dehydrogenase/3-ketoacyl-Coenzyme	Hadhb	0.88546	1.04289213	0.05999448
1430812_at	RIKEN cDNA 4930511A02 gene	4930511A02Rik	0.88546	1.05011573	0.06552197
1416769_s_at	ATPase, H+ transporting, lysosomal V0 subunit B	Atp6v0b	0.88547	1.04957335	0.06589272
1441126_at	---	---	0.88547	-1.05840913	-0.0920393
1430570_at	kynureninase (L-kynurenine hydrolase)	Kynu	0.88547	1.05488365	0.07588837
1431230_a_at	BTB (POZ) domain containing 9	Btbd9	0.88547	1.06690339	0.08385933
1451534_at	secretagogin, EF-hand calcium binding protein	Scgn	0.88548	1.06209969	0.08174447
1422854_at	src homology 2 domain-containing transforming protein C1	Shc1	0.88549	1.04734358	0.06355369
1451183_at	myosin XIX	Myo19	0.8855	1.05104748	0.07166735
1424033_at	serine/arginine-rich splicing factor 7	Srsf7	0.8855	-1.03857416	-0.05623
1448704_s_at	histocompatibility 47	H47	0.8855	1.04006273	0.05641776
1444517_at	---	---	0.8855	-1.05995044	-0.0922863
1452624_at	leucine rich repeat transmembrane neuronal 1	Lrrtm1	0.8855	1.05874959	0.08040023
1457517_at	RIKEN cDNA A730046G19 gene	A730046G19Rik	0.88551	-1.05911332	-0.0858172
1450781_at	high mobility group AT-hook 2	Hmga2	0.88551	-1.05700078	-0.0834361
1446831_at	DNA segment, Chr 5, ERATO Doi 521, expressed	D5Ertd521e	0.88552	-1.04478878	-0.0665785
1445465_at	expressed sequence N28178	N28178	0.88553	1.08233491	0.100128
1420461_at	macrophage stimulating 1 receptor (c-met-related tyrosine kinase	Mst1r	0.88553	1.06158573	0.08588159
1455301_at	WAS/WASL interacting protein family, member 3	Wipf3	0.88554	1.05338821	0.07488439
1446014_at	---	---	0.88554	1.10401338	0.11444865
1441793_at	Ring finger protein 39	Rnf39	0.88554	1.06743916	0.08959032
1446145_at	eukaryotic translation initiation factor 4 gamma, 3	Eif4g3	0.88554	1.0539623	0.07538472
1425328_at	family with sequence similarity 76, member A	Fam76a	0.88555	-1.04655177	-0.070873
1416180_a_at	radixin	Rdx	0.88556	-1.04484679	-0.0660601
1440487_at	deleted in colorectal carcinoma	Dcc	0.88556	1.06026709	0.07920336
1432630_at	tankyrase, TRF1-interacting ankyrin-related ADP-ribose polymera	Tnks	0.88557	1.06138337	0.08149064
1427318_s_at	myoferlin	Myof	0.88558	-1.05117731	-0.0799003
1415685_at	mitochondrial translational initiation factor 2	Mtif2	0.88558	-1.04294608	-0.0617624
1440381_at	RIO kinase 2 (yeast)	Riok2	0.8856	1.04880402	0.06601518
1435835_at	Meckel syndrome, type 1	Mks1	0.8856	1.05432405	0.0722464
1445173_at	cDNA sequence AK129341	AK129341	0.88562	-1.05789103	-0.0817836
1424333_at	RNA (guanine-9-) methyltransferase domain containing 1	Rg9mtd1	0.88562	-1.04985111	-0.0764695
1419672_at	sparc/osteonectin, cwcv and kazal-like domains proteoglycan 1	Spock1	0.88562	-1.05413382	-0.0780691
1423266_at	RIKEN cDNA 2810405K02 gene	2810405K02Rik	0.88563	1.04546145	0.06317159
1429099_at	double homeobox B-like /// predicted gene 10394	Duxbl /// Gm10394	0.88564	1.06002374	0.08311107
1423366_at	stearoyl-coenzyme A desaturase 3	Scd3	0.88564	1.06020769	0.0833769
1430117_a_at	zinc finger protein 64	Zfp64	0.88565	1.04664933	0.06470153
1429742_at	regulator of chromosome condensation (RCC1) and BTB (POZ) dor	Rcbtb2	0.88565	1.08148124	0.09807933
1451903_at	kynureninase (L-kynurenine hydrolase)	Kynu	0.88566	-1.05731562	-0.0847399
1427500_at	signal peptidase complex subunit 1 homolog (S. cerevisiae)	Spcs1	0.88567	-1.0561383	-0.0811241
1456224_x_at	cancer antigen 1	Cage1	0.88568	1.03779497	0.0532972
1433264_at	---	---	0.88568	1.06094468	0.08440056
1455718_at	spermatogenesis associated 21	Spata21	0.88568	1.07994544	0.09894641
1449273_at	cytoplasmic FMR1 interacting protein 2	Cyfip2	0.88569	1.05785419	0.07848097
1423222_at	CAP, adenylate cyclase-associated protein, 2 (yeast)	Cap2	0.8857	1.06124989	0.0848468
1444864_at	DNA segment, Chr 12, ERATO Doi 125, expressed	D12Ertd125e	0.8857	1.11934531	0.12496445
1442326_at	protocadherin 15	Pcdh15	0.8857	1.0449897	0.05941263
1436496_at	RIKEN cDNA 1700012O15 gene	1700012O15Rik	0.88571	-1.05779864	-0.0819299
1445517_at	---	---	0.88571	1.06563643	0.08664294
1430326_s_at	ubiquinol-cytochrome c reductase, complex III subunit VII	Uqcrq	0.88572	1.07515443	0.08494027
1440537_at	potassium channel, subfamily V, member 2	Kcnv2	0.88572	1.09407925	0.10497185
1441470_at	expressed sequence AV249152	AV249152	0.88574	-1.04395539	-0.0633749
1445891_at	cDNA sequence AF529169	AF529169	0.88574	1.06026856	0.08223295
1452566_at	---	---	0.88575	-1.05754099	-0.0813028
1457404_at	Nuclear factor of kappa light polypeptide gene enhancer in B-cells	Nfkbiz	0.88576	1.06229396	0.08606221

1449149_at	TNF receptor-associated factor 3	Traf3	0.88576	1.05833335	0.0800248
1447401_at	WW domain binding protein 11	Wbp11	0.88576	1.07511137	0.09565457
1433360_at	RIKEN cDNA 1700023B13 gene	1700023B13Rik	0.88578	-1.05248906	-0.0754389
1422668_at	serine (or cysteine) peptidase inhibitor, clade B, member 9b	Serpinb9b	0.88578	-1.05464052	-0.0864137
1435857_s_at	amyloid beta (A4) precursor-like protein 1	Aplp1	0.88579	-1.03961875	-0.0573601
1417702_a_at	histamine N-methyltransferase	Hnmt	0.88579	1.04998933	0.06746163
1452002_at	ADNP homeobox 2	Adnp2	0.88581	-1.04487567	-0.0661242
1423599_a_at	phosducin-like	Pdcl	0.88581	-1.04120546	-0.0600353
1419297_at	histocompatibility 2, O region alpha locus	H2-Oa	0.88583	1.065126	0.08621073
1460088_at	RIKEN cDNA 6430531B16 gene	6430531B16Rik	0.88584	1.07164637	0.09181628
1435591_at	expressed sequence AI426330	AI426330	0.88584	1.10497503	0.11418538
1446600_at	---	---	0.88587	1.07530186	0.09189052
1417478_a_at	protein phosphatase 2, regulatory subunit B'', gamma	Ppp2r3c	0.88587	1.04117419	0.05749984
1430503_at	RIKEN cDNA 6330522J23 gene	6330522J23Rik	0.88587	-1.05247723	-0.0751788
1446932_at	---	---	0.88588	-1.05626568	-0.0852634
1416634_at	family with sequence similarity 96, member A	Fam96a	0.88592	1.04181473	0.0580713
1433709_at	calcium activated nucleotidase 1	Cant1	0.88605	1.04717298	0.06563304
1457197_at	protein kinase, cAMP dependent, catalytic, beta	Prkacb	0.88606	1.06398539	0.08145753
1429905_at	LIM homeobox protein 9	Lhx9	0.8861	-1.04277334	-0.067945
1455112_at	apoptosis-inducing factor, mitochondrion-associated 2	Aifm2	0.88612	1.05931569	0.07780667
1417742_a_at	DNA methyltransferase 1-associated protein 1	Dmap1	0.88612	1.05286568	0.07013319
1458145_at	hypothetical LOC100502669	LOC100502669	0.88612	1.08192003	0.09754876
1428325_at	canopy 4 homolog (zebrafish)	Cnpy4	0.88613	1.04591653	0.06337663
1427120_at	zinc finger protein 26	Zfp26	0.88614	-1.04679128	-0.0674879
1433008_at	RIKEN cDNA 6330526H18 gene	6330526H18Rik	0.88614	1.05987892	0.0763649
1447797_x_at	PIH1 domain containing 1	Pih1d1	0.88615	1.06658036	0.08548212
1415874_at	sprouty homolog 1 (Drosophila)	Spry1	0.88615	1.06135904	0.07722306
1419460_at	ribonuclease P 14 subunit (human)	Rpp14	0.88615	1.0519724	0.07209553
1452232_at	UDP-N-acetyl-alpha-D-galactosamine: polypeptide N-acetylgalac	Galnt7	0.88616	1.06244069	0.0806617
1418842_at	hematopoietic cell specific Lyn substrate 1	Hcls1	0.88616	1.07680868	0.09608083
1458610_at	---	---	0.88617	-1.05844352	-0.0834926
1453652_at	RIKEN cDNA 4933400F21 gene	4933400F21Rik	0.88617	1.10644763	0.11617899
1435196_at	neurotrophic tyrosine kinase, receptor, type 2	Ntrk2	0.88618	1.06604249	0.08729567
1452806_at	family with sequence similarity 57, member B	Fam57b	0.88619	1.08899368	0.10431217
1425995_s_at	Wilms tumor 1 homolog	Wt1	0.88619	-1.0578446	-0.1105215
1415679_at	presenilin enhancer 2 homolog (C. elegans)	Psenen	0.88619	1.04523264	0.06112725
1435638_at	glycogen synthase kinase 3 alpha	Gsk3a	0.88619	-1.04357545	-0.0646421
1449582_at	caudal type homeobox 1	Cdx1	0.8862	1.06231248	0.08313636
1438629_x_at	granulin	Grn	0.8862	1.04110249	0.05690345
1447974_s_at	aquaporin 6	Aqp6	0.88621	-1.05572273	-0.0861093
1441158_at	---	---	0.88621	-1.06132714	-0.0862071
1444938_at	---	---	0.88621	-1.05739036	-0.0873869
1451292_at	Zinc finger protein 212	Zfp212	0.88621	1.04525404	0.06223068
1442881_at	---	---	0.88621	1.04975176	0.06940618
1447659_x_at	ATPase, H+ transporting, lysosomal V1 subunit H	Atp6v1h	0.88622	-1.0461702	-0.0704586
1450900_at	SMEK homolog 2, suppressor of mek1 (Dictyostelium)	Smek2	0.88622	-1.04532359	-0.0757597
1424462_at	ERGIC and golgi 2	Ergic2	0.88622	-1.04477709	-0.0643508
1416983_s_at	forkhead box O1	Foxo1	0.88622	1.04688488	0.06493892
1439751_at	Regulator of sex-limitation candidate 18	Rslcan18	0.88622	1.05617145	0.07860876
1457776_at	DNA segment, Chr 9, ERATO Doi 720, expressed	D9Ertd720e	0.88623	1.10904144	0.1174856
1420247_at	galactosidase, beta 1-like	Glb1l	0.88623	-1.05738831	-0.0836188
1429381_x_at	immunoglobulin heavy chain 2 (serum IgA) /// immunoglobulin h	Igh-2 /// Igh-VJ558 /// LC	0.88623	-1.05758091	-0.0829647
1445905_at	expressed sequence C79206	C79206	0.88623	-1.05613387	-0.0814337
1460299_at	motor neuron and pancreas homeobox 1	Mnx1	0.88624	-1.05845004	-0.0949649
1428991_at	HRAS-like suppressor	Hrasls	0.88624	1.09693196	0.10790635
1428232_at	cleavage and polyadenylation specific factor 6	Cpsf6	0.88624	-1.03959344	-0.0568181
1421125_at	spermatid perinuclear RNA binding protein	Strbp	0.88624	-1.05355406	-0.0758532
1438156_x_at	carnitine palmitoyltransferase 1a, liver	Cpt1a	0.88625	-1.05472371	-0.0830875
1435922_at	F-box and leucine-rich repeat protein 19	Fbxl19	0.88625	-1.04871945	-0.0851949
1439673_at	RIKEN cDNA E230008O15 gene	E230008O15Rik	0.88626	1.07421777	0.09410413
1436970_a_at	platelet derived growth factor receptor, beta polypeptide	Pdgfrb	0.88626	-1.06030548	-0.0851376
1424281_at	ubiquitin-associated protein 2	Ubap2	0.88626	1.04512358	0.06278912
1422428_at	acyl-CoA synthetase bubblegum family member 1	Acsbg1	0.88627	1.06753112	0.08784419
1454994_at	kelch-like 20 (Drosophila)	Klhl20	0.88627	1.05409447	0.07450662
1441508_at	predicted gene 10114	Gm10114	0.88628	1.06433262	0.08724718
1447913_x_at	A kinase (PRKA) anchor protein (yotiao) 9	Akap9	0.88628	1.05980529	0.08162129
1417516_at	DNA-damage inducible transcript 3	Ddit3	0.88628	-1.05146598	-0.1025301
1441026_at	poly (ADP-ribose) polymerase family, member 4	Parp4	0.88628	1.07792862	0.09708253
1445677_x_at	solute carrier family 35, member F2	Slc35f2	0.88629	1.07367195	0.09263807
1438402_at	family with sequence similarity 171, member A1	Fam171a1	0.88629	1.10184054	0.11256102
1450701_a_at	general transcription factor II H, polypeptide 2	Gtf2h2	0.8863	-1.04018574	-0.0570888
1445048_at	bromodomain adjacent to zinc finger domain, 2B	Baz2b	0.8863	1.06070763	0.08089614
1441233_at	RIKEN cDNA 7120451J01 gene /// hypothetical protein LOC10050	7120451J01Rik /// LOC10	0.8863	1.06225553	0.08223079
1451286_s_at	fusion, derived from t(12;16) malignant liposarcoma (human)	Fus	0.8863	-1.03840698	-0.0574883
1444645_at	---	---	0.8863	-1.04959551	-0.0707772
1441432_at	RIKEN cDNA C820005J03 gene	C820005J03Rik	0.8863	-1.06034055	-0.0965725
1432598_at	germ cell-less homolog 1 (Drosophila)	Gmcl1	0.8863	-1.06299467	-0.1094996
1419722_at	kallikrein related-peptidase 8	Klk8	0.88631	1.04848034	0.06816734
1454396_at	RIKEN cDNA 4632409D06 gene	4632409D06Rik	0.88631	1.09455047	0.10819373
1459472_at	---	---	0.88631	-1.05671994	-0.0821782
1442917_at	predicted gene 11627	Gm11627	0.88632	-1.05266007	-0.0746753
1438563_s_at	mitochondrial ribosomal protein S24	Mrps24	0.88632	-1.04454626	-0.0782258
1417658_at	protein TBRG4-like /// transforming growth factor beta regulated	LOC100046166 /// Tbrg4	0.88632	1.05250472	0.06877408
1431033_x_at	amylo-1,6-glucosidase, 4-alpha-glucanotransferase	Agl	0.88634	-1.05343837	-0.0803572
1417950_a_at	apolipoprotein A-II	Apoa2	0.88635	1.07459272	0.09355187
1423111_at	ATP synthase, H+ transporting, mitochondrial F1 complex, alpha s	Atp5a1	0.88636	1.03033713	0.04170687
1440181_at	predicted gene 1568	Gm1568	0.88637	1.06878301	0.08934483
1423089_at	tropomodulin 3	Tmod3	0.88638	-1.05093478	-0.078911
1421846_at	WD repeat and SOCS box-containing 2	Wsb2	0.88639	1.04970754	0.06506161
1439019_at	Fraser syndrome 1 homolog (human)	Fras1	0.88639	1.06342365	0.08597801
1454177_at	RIKEN cDNA 4930597G03 gene	4930597G03Rik	0.8864	-1.06048904	-0.0856088
1422367_at	olfactory receptor 70	Olfr70	0.88641	1.06914245	0.08920824
1443403_at	---	---	0.88641	1.08033507	0.10042739
1430626_at	zinc finger protein 398	Zfp398	0.88641	-1.06152274	-0.1084828

1416277_a_at	ribosomal protein, large, P1	Rplp1	0.88641	1.01842653	0.02620154
1443604_at	---	---	0.88642	-1.0484431	-0.0725411
1434874_x_at	ArfGAP with coiled-coil, ankyrin repeat and PH domains 1	Acap1	0.88642	-1.05358912	-0.0794872
1446347_at	---	---	0.88643	-1.05447414	-0.0822652
1451227_a_at	solute carrier family 10 (sodium/bile acid cotransporter family), m	Slc10a3	0.88643	1.06482199	0.08402644
1422772_at	core 1 synthase, glycoprotein-N-acetylgalactosamine 3-beta-gala	C1galt1	0.88643	1.10111661	0.113312
1455198_a_at	protein phosphatase 2 (formerly 2A), regulatory subunit B'', delta	Ppp2r3d	0.88643	-1.05084645	-0.0753989
1445943_at	---	---	0.88643	1.0661357	0.08790009
1459005_at	---	---	0.88644	1.07265286	0.0937498
1416254_a_at	vacuolar protein sorting 16 (yeast)	Vps16	0.88644	1.04380303	0.0604231
1459428_at	expressed sequence C76662	C76662	0.88644	-1.05851407	-0.0902431
1415934_at	COP9 (constitutive photomorphogenic) homolog, subunit 8 (Arabi	Cops8	0.88645	1.04187538	0.05872284
1452026_a_at	phospholipase A2, group XIIA	Pla2g12a	0.88646	1.06124318	0.07754515
1451177_at	DnaJ (Hsp40) homolog, subfamily B, member 4	Dnajb4	0.88646	1.07812095	0.09166147
1442869_at	docking protein 7	Dok7	0.88646	-1.05788419	-0.0843468
1442838_at	predicted gene 11691	Gm11691	0.88648	1.05618778	0.07800153
1422461_at	ATPase family, AAA domain containing 3A	Atad3a	0.88658	1.04136265	0.0576176
1425761_a_at	nuclear factor of activated T-cells, cytoplasmic, calcineurin-depen	Nfatc1	0.88661	1.06117642	0.08140253
1456061_at	GTPase, IMAP family member 8	Gimap8	0.88662	1.04989915	0.06737609
1454063_at	RIKEN cDNA 4933412E24 gene	4933412E24Rik	0.88662	1.06820151	0.08905843
1451481_s_at	organic solute transporter alpha	Osta	0.88663	-1.0601685	-0.0900953
1437506_at	a disintegrin-like and metallopeptidase (reprolysin type) with thro	Adamts6	0.88666	-1.05798796	-0.089191
1444345_at	---	---	0.88666	-1.05676719	-0.0816614
1428746_a_at	tRNA selenocysteine 1 associated protein 1	Trnau1ap	0.88667	1.04572553	0.06303383
1433286_at	RIKEN cDNA 4833417J16 gene	4833417J16Rik	0.88668	1.06244142	0.08288522
1433010_at	RIKEN cDNA 5530400N10 gene	5530400N10Rik	0.88669	1.0619726	0.08213506
1458832_at	---	---	0.88669	-1.0463441	-0.0729313
1439684_at	RIKEN cDNA 4930570G19 gene	4930570G19Rik	0.8867	-1.04911767	-0.0734133
1455983_at	cell division cycle associated 2	Cdca2	0.88671	1.0472252	0.06239703
1436870_s_at	actin filament associated protein 1-like 2	Afap1l2	0.88671	-1.06212238	-0.1401154
1459058_at	sialic acid acetylesterase	Siae	0.88672	-1.06019467	-0.0850208
1434862_at	fucosyltransferase 2	Fut2	0.88672	-1.05764254	-0.0856874
1455394_at	protein inhibitor of activated STAT 4	Pias4	0.88672	1.07378712	0.08631463
1432544_at	RIKEN cDNA 4930449E01 gene	4930449E01Rik	0.88673	-1.05869913	-0.0838419
1459922_at	expressed sequence AI646383	AI646383	0.88673	1.0603469	0.08443907
1460126_at	---	---	0.88674	1.0650622	0.0890845
1451436_at	sno, strawberry notch homolog 1 (Drosophila)	Sbno1	0.88675	-1.04196662	-0.0595985
1421981_at	potassium voltage gated channel, Shaw-related subfamily, memb	Kcnc3	0.88675	-1.05750877	-0.083458
1425338_at	phospholipase C, beta 4	Plcb4	0.88676	1.05696276	0.07798954
1436605_at	transketolase	Tkt	0.88677	1.06205826	0.08382123
1416536_at	melanoma associated antigen (mutated) 1	Mum1	0.88677	1.04104572	0.05728503
1427065_at	RIKEN cDNA 4933439F18 gene	4933439F18Rik	0.88678	1.04581755	0.06444842
1424742_at	cAMP responsive element binding protein 3	Creb3	0.88679	-1.05559614	-0.0806457
1429803_at	RIKEN cDNA 1700021F07 gene	1700021F07Rik	0.8868	-1.06351607	-0.0916996
1425357_a_at	gremlin 1	Grem1	0.88681	1.06189097	0.08248127
1440427_at	UV radiation resistance associated gene	Uvrag	0.88681	-1.06308964	-0.1130128
1436267_a_at	mechanistic target of rapamycin (serine/threonine kinase)	Mtor	0.88681	1.05224083	0.07205122
1416831_at	neuraminidase 1	Neu1	0.88682	1.05482793	0.07597515
1445809_at	---	---	0.88683	-1.06224179	-0.1015811
1431645_a_at	guanosine diphosphate (GDP) dissociation inhibitor 2	Gdi2	0.88683	-1.03986832	-0.0600874
1428152_a_at	ribosomal protein L18A	Rpl18a	0.88684	1.02307169	0.03209588
1430302_at	cannabinoid receptor interacting protein 1	Cnrip1	0.88684	-1.05847364	-0.0881603
1432698_at	RIKEN cDNA 2900024I21 gene	2900024I21Rik	0.88684	-1.05858655	-0.0853703
1450555_at	testis expressed gene 13	Tex13	0.88684	-1.04544739	-0.0661193
1449514_at	G protein-coupled receptor kinase 5	Grk5	0.88685	1.10740028	0.11528941
1431713_at	suppressor of fused homolog (Drosophila)	Sufu	0.88685	-1.05279448	-0.0745977
1456874_at	fibronectin leucine rich transmembrane protein 2	Flrt2	0.88686	1.05987944	0.08095762
1428935_at	calnexin	Canx	0.88686	-1.05183511	-0.0789091
1431591_s_at	predicted gene 9706 /// ISG15 ubiquitin-like modifier	Gm9706 /// Isg15	0.88687	1.05873412	0.08108561
1421793_at	fibroblast growth factor 11	Fgf11	0.88687	-1.0574505	-0.0835701
1424776_a_at	solute carrier family 25, member 28	Slc25a28	0.88687	1.05311536	0.06867652
1422211_a_at	UDP-GlcNAc:betaGal beta-1,3-N-acetylglucosaminyltransferase 3	B3gnt3	0.88687	1.05619671	0.07736577
1446077_at	---	---	0.88687	1.05745948	0.07649556
1430815_at	RIKEN cDNA 4930534B04 gene	4930534B04Rik	0.88687	-1.05869104	-0.0846465
1425667_at	acyl-CoA thioesterase 11	Acot11	0.88688	1.07438903	0.09313623
1432702_at	RIKEN cDNA 2810403G07 gene	2810403G07Rik	0.88688	-1.05761634	-0.0834974
1431380_at	RIKEN cDNA 5730409L17 gene	5730409L17Rik	0.88688	1.05505671	0.07729756
1443985_at	zinc finger protein 719	Zfp719	0.88689	-1.05840197	-0.0855544
1418817_at	chromatin modifying protein 1B	Chmp1b	0.88689	-1.0433341	-0.0620529
1460337_at	SH3-domain kinase binding protein 1	Sh3kbp1	0.8869	1.07299714	0.09238747
1433418_at	---	---	0.8869	-1.0599521	-0.0854981
1458857_at	---	---	0.88691	1.07130764	0.09140191
1430213_at	regulator of chromosome condensation (RCC1) and BTB (POZ) dor	Rcbtb1	0.88691	-1.06109728	-0.0971607
1459305_at	---	---	0.88692	-1.05558076	-0.0791853
1417494_a_at	ceruloplasmin	Cp	0.88692	1.07284589	0.09195719
1431121_at	angiomotin-like 1	Amotl1	0.88692	-1.0589516	-0.0900653
1458228_at	---	---	0.88693	1.05601599	0.0784793
1427779_a_at	CD4 antigen	Cd4	0.88693	-1.05582751	-0.0793033
1423165_a_at	metastasis-associated gene family, member 2	Mta2	0.88694	1.0530652	0.0690167
1435906_x_at	guanylate binding protein 2	Gbp2	0.88694	-1.04826916	-0.0725699
1449720_at	---	---	0.88694	-1.0621258	-0.1044757
1441388_at	Methyl-CpG binding domain protein 2	Mbd2	0.88694	-1.05683001	-0.0894124
1435808_at	RIKEN cDNA A230051G13 gene	A230051G13Rik	0.88694	1.06172133	0.08042693
1433612_at	adaptor-related protein complex 2, sigma 1 subunit	Ap2s1	0.88694	1.05745182	0.07311533
1436716_at	Protein phosphatase 1, regulatory (inhibitor) subunit 14B	Ppp1r14b	0.88695	-1.05446459	-0.0791972
1433206_at	RIKEN cDNA 5033430J17 gene	5033430J17Rik	0.88695	-1.06077991	-0.0884143
1418440_at	collagen, type VIII, alpha 1	Col8a1	0.88695	-1.06115466	-0.0968555
1446433_at	acyl-Coenzyme A binding domain containing 5	Acbd5	0.88695	1.06907222	0.09055973
1419213_at	N-acetyltransferase 6	Nat6	0.88695	1.05747531	0.07733858
1437801_at	mortality factor 4 like 1	Morf4l1	0.88697	1.08130851	0.09117611
1430364_at	activating transcription factor 7 interacting protein 2	Atf7ip2	0.88697	-1.05131148	-0.0773508
1434016_at	zinc and ring finger 2	Znrf2	0.88698	1.06144652	0.07541759
1442720_at	---	---	0.88698	-1.05900398	-0.0855358
1433022_at	RIKEN cDNA 2310068G24 gene	2310068G24Rik	0.88698	-1.04806531	-0.0735124

1459775_at	---	---	0.88698	1.0834017	0.0990776
1432223_at	RIKEN cDNA 4930552N02 gene	4930552N02Rik	0.88698	-1.05353738	-0.0799062
1434722_at	adenosine monophosphate deaminase 1	Ampd1	0.88698	1.06761302	0.08900369
1428793_at	solute carrier family 36 (proton/amino acid symporter), member 1	Slc36a1	0.88698	-1.0530704	-0.0747165
1435486_at	p21 protein (Cdc42/Rac)-activated kinase 3	Pak3	0.88698	-1.06144154	-0.0925633
1452525_a_at	neurofibromatosis 1	Nf1	0.88699	-1.05770171	-0.0816702
1419094_at	cytochrome P450, family 2. subfamily c, polypeptide 37	Cyp2c37	0.88699	1.06262086	0.08144811
1428747_at	tRNA selenocysteine 1 associated protein 1	Trnau1ap	0.887	1.05108376	0.06684126
1421230_a_at	Musashi homolog 2 (Drosophila)	Msi2	0.887	-1.05487173	-0.105098
1432006_at	adaptor protein complex AP-2, alpha 2 subunit	Ap2a2	0.88701	1.06061679	0.08465686
1440182_at	frizzled homolog 10 (Drosophila)	Fzd10	0.88701	1.0664434	0.08637662
1460450_at	AMME chromosomal region gene 1-like	Ammecr1l	0.88701	1.04838044	0.0663002
1441880_x_at	transmembrane protein 149	Tmem149	0.88701	1.07348433	0.09324542
1443703_at	CD28 antigen	Cd28	0.88702	1.06492785	0.07906392
1456946_at	SH3 domain containing ring finger 3	Sh3rf3	0.88702	-1.05798078	-0.0817405
1423610_at	methionine aminopeptidase 2	Metap2	0.88702	1.03621889	0.05124299
1455050_at	RIKEN cDNA E130203B14 gene	E130203B14Rik	0.88704	1.07747256	0.09455704
1424575_at	RAB, member of RAS oncogene family-like 5	Rabl5	0.88705	1.05978902	0.07995915
1441823_at	zinc finger, MIZ-type containing 1	Zmiz1	0.88705	-1.05890837	-0.082627
1434113_a_at	REX4, RNA exonuclease 4 homolog (S. cerevisiae)	Rexo4	0.88707	1.06067469	0.07441468
1425121_a_at	cDNA sequence BC006965	BC006965	0.88707	1.05011075	0.06908283
1419209_at	chemokine (C-X-C motif) ligand 1	Cxcl1	0.88707	-1.04907792	-0.0706084
1416013_at	phospholipase D family, member 3	Pld3	0.8871	1.05467172	0.07105954
1451758_at	laminin gamma 3	Lamc3	0.88711	-1.05957371	-0.0843892
1452469_a_at	smoothelin	Smtn	0.88712	1.05340565	0.07282217
1439392_x_at	protease (prosome, macropain) 26S subunit, ATPase 1	Psmc1	0.88714	1.07976521	0.09521247
1422258_at	cholinergic receptor, muscarinic 3, cardiac	Chrm3	0.88714	1.05756437	0.07876591
1454230_a_at	solute carrier family 25, member 27	Slc25a27	0.88715	1.08737893	0.10256209
1423666_s_at	60S ribosomal protein L5-like	LOC100503670	0.88716	1.0392939	0.05544626
1445732_at	---	---	0.88717	1.05556489	0.07737665
1437165_a_at	procollagen C-endopeptidase enhancer protein	Pcolce	0.88717	1.05303691	0.06765194
1435227_at	B-cell leukemia/lymphoma 11B	Bcl11b	0.88718	-1.05559501	-0.0781709
1450876_at	complement component factor h	Cfh	0.88719	1.03644401	0.05125182
1416084_at	zinc finger, AN1-type domain 5	Zfand5	0.88719	-1.04907866	-0.0815983
1437797_at	ATPase, Ca++ transporting, cardiac muscle, slow twitch 2	Atp2a2	0.8872	-1.0597609	-0.0901265
1441018_at	ubiquitin specific peptidase 24	Usp24	0.8872	-1.06245432	-0.0962042
1442687_at	RIKEN cDNA 1700064N11 gene	1700064N11Rik	0.88721	-1.06096023	-0.0911225
1433120_at	RIKEN cDNA 9530002O20 gene	9530002O20Rik	0.88721	-1.0525298	-0.0742277
1452560_a_at	nuclear transcription factor-Y alpha	Nfya	0.88724	-1.04355484	-0.0643294
1425089_at	potassium voltage gated channel, Shaw-related subfamily, memb	Kcnc4	0.8873	1.05848723	0.07791256
1436772_at	Glutamate receptor, ionotropic, AMPA4 (alpha 4)	Gria4	0.88741	1.04989224	0.06967653
1453566_at	RNA binding motif protein 33	Rbm33	0.88742	-1.05883956	-0.086952
1432014_at	RIKEN cDNA 6530409C15 gene	6530409C15Rik	0.88742	-1.05985914	-0.0896096
1417721_s_at	lysosomal-associated protein transmembrane 5	Laptm5	0.88742	-1.04959244	-0.1425095
1445110_at	---	---	0.88742	-1.06334007	-0.1063841
1447371_at	RIKEN cDNA 9430037G07 gene	9430037G07Rik	0.88743	1.06516958	0.08889809
1437786_at	N(alpha)-acetyltransferase 11, NatA catalytic subunit	Naa11	0.88744	1.05411492	0.07111151
1419410_at	basic leucine zipper transcription factor, ATF-like	Batf	0.88744	-1.05429469	-0.0799944
1457298_at	---	---	0.88746	1.08497544	0.10285366
1441626_at	---	---	0.88746	-1.05402142	-0.0821722
1436169_at	---	---	0.88746	1.06784002	0.0889889
1442565_at	protocadherin 10	Pcdh10	0.88747	-1.04518726	-0.0675978
1444054_at	---	---	0.88747	-1.05976395	-0.0840154
1443347_at	---	---	0.88748	1.0681274	0.08684201
1426336_at	calcium channel, voltage-dependent, gamma subunit 7	Cacng7	0.88748	-1.06142209	-0.0981565
1436220_at	zinc finger protein 287	Zfp287	0.88748	1.07356878	0.09133628
1449495_at	regenerating islet-derived 3 alpha	Reg3a	0.88748	1.06443844	0.08732441
1418074_at	ST6 (alpha-N-acetyl-neuraminyl-2,3-beta-galactosyl-1,3)-N-acety	St6galnac4	0.88749	1.07214713	0.09277263
1424758_s_at	serine (or cysteine) peptidase inhibitor, clade A (alpha-1 antiprote	Serpina10	0.88749	1.05967248	0.08228801
1451652_a_at	transmembrane protein 188	Tmem188	0.8875	-1.04148064	-0.0589556
1427859_at	immunoglobulin kappa chain variable 19 (V19)-15	Igk-V19-15	0.8875	1.07232236	0.09210328
1421667_at	neuromedin U receptor 1	Nmur1	0.8875	-1.05737443	-0.0881959
1443839_at	RIKEN cDNA 1700010H22 gene	1700010H22Rik	0.8875	-1.04683038	-0.0665276
1442040_at	transmembrane protein 125	Tmem125	0.88751	-1.05495464	-0.0801781
1447886_at	RIKEN cDNA 0610040B09 gene	0610040B09Rik	0.88751	1.13140085	0.1289479
1439188_at	cleavage and polyadenylation specific factor 6	Cpsf6	0.88751	1.10042272	0.11307883
1420684_at	acyl-Coenzyme A oxidase 3, pristanoyl	Acox3	0.88752	1.08154787	0.09886034
1449522_at	unc-5 homolog C (C. elegans)	Unc5c	0.88752	-1.05496077	-0.0802916
1429623_at	zinc finger protein 644	Zfp644	0.88752	-1.04415374	-0.0717592
1415905_at	regenerating islet-derived 1	Reg1	0.88753	-1.0583051	-0.0819698
1455273_at	zinc finger and BTB domain containing 6	Zbtb6	0.88754	-1.04743209	-0.0672094
1443372_at	predicted gene 2990	Gm2990	0.88754	1.06249606	0.08508784
1446349_at	zinc finger protein 78	Zfp78	0.88754	1.05627589	0.07566687
1427188_at	ariadne ubiquitin-conjugating enzyme E2 binding protein homolog	Arih1	0.88755	1.05379463	0.06953439
1427057_at	5'-nucleotidase domain containing 3	Nt5dc3	0.88755	1.04983469	0.06709312
1444023_at	ankyrin 2, brain	Ank2	0.88756	-1.0429558	-0.0609811
1444164_at	---	---	0.88756	-1.05835273	-0.0821532
1460684_at	transmembrane 7 superfamily member 2	Tm7sf2	0.88757	1.06339523	0.07958075
1418637_at	ets variant gene 3	Etv3	0.8876	-1.05163992	-0.0730779
1436260_at	---	---	0.8876	1.06677652	0.08735311
1416827_at	thromboxane A synthase 1, platelet	Tbxas1	0.88763	1.05852568	0.08065166
1429071_at	malic enzyme 3, NADP(+)-dependent, mitochondrial	Me3	0.88763	1.0732806	0.09334914
1428559_at	3-ketodihydrosphingosine reductase	Kdsr	0.88774	-1.04544002	-0.0658213
1433559_at	solute carrier family 45, member 4	Slc45a4	0.88775	1.04348432	0.06105195
1457342_at	IKAROS family zinc finger 4	Ikzf4	0.88775	-1.05846411	-0.0942786
1447430_at	---	---	0.88776	1.07456451	0.09123555
1454426_at	RIKEN cDNA 4930519E07 gene	4930519E07Rik	0.88776	-1.04866428	-0.0705122
1440293_at	RIKEN cDNA C230081A13 gene	C230081A13Rik	0.88776	1.06457819	0.08526324
1426325_at	kinesin family member 1C	Kif1c	0.88776	-1.06002419	-0.1046027
1453295_at	additional sex combs like 2 (Drosophila)	Asxl2	0.88776	1.08133083	0.09740799
1441182_at	bromodomain and WD repeat domain containing 3	Brwd3	0.88776	-1.05564017	-0.0789918
1438248_at	proprotein convertase subtilisin/kexin type 5	Pcsk5	0.88777	1.05394298	0.07572273
1428676_at	transmembrane serine protease 6	Tmprss6	0.88777	1.05137627	0.07227799
1439109_at	coiled-coil domain containing 68	Ccdc68	0.88778	1.07213441	0.09124134

1421949_a_at	coiled-coil domain containing 123	Ccdc123	0.88778	1.06450979	0.08250443
1448183_a_at	hypoxia inducible factor 1, alpha subunit	Hif1a	0.88778	-1.05171522	-0.0984676
1458225_at	FK506 binding protein 14	Fkbp14	0.88778	1.05682686	0.07854161
1432328_at	RIKEN cDNA 4930516K23 gene	4930516K23Rik	0.88779	1.0668958	0.08910134
1446383_at	---	---	0.88779	1.05606892	0.07731677
1425683_at	---	---	0.88779	-1.05317294	-0.0786853
1429670_a_at	centrosomal protein 97	Cep97	0.88779	1.05498304	0.07273671
1428950_s_at	nucleolar protein 8	Nol8	0.88779	-1.04163306	-0.059151
1445856_at	family with sequence similarity 178, member A	Fam178a	0.8878	1.06695554	0.08797157
1435190_at	cell adhesion molecule with homology to L1CAM	Chl1	0.8878	-1.05166934	-0.099262
1447086_at	---	---	0.8878	-1.0555958	-0.0781682
1421641_at	solute carrier family 6 (neurotransmitter transporter, noradrenalin	Slc6a2	0.8878	-1.05804314	-0.0856871
1458333_x_at	undifferentiated embryonic cell transcription factor 1	Utf1	0.88781	-1.05848795	-0.0822403
1428760_at	small nuclear RNA activating complex, polypeptide 3	Snapc3	0.88781	1.03902544	0.05494795
1432853_at	RIKEN cDNA 2900057C01 gene	2900057C01Rik	0.88781	-1.0572059	-0.0838397
1437429_at	ADAMTS-like 1	Adamtsl1	0.88781	1.06196047	0.08490583
1416450_at	TAF8 RNA polymerase II, TATA box binding protein (TBP)-associat	Taf8	0.88781	1.04762013	0.06674217
1424728_at	ribosomal RNA processing 36 homolog (S. cerevisiae)	Rrp36	0.88782	-1.04417031	-0.0640471
1445560_at	---	---	0.88782	-1.05520847	-0.0791831
1441219_at	---	---	0.88782	-1.05856424	-0.0839821
1418882_at	NLR family, pyrin domain containing 5	Nlrp5	0.88782	1.06760778	0.08674449
1442605_at	---	---	0.88783	1.0566094	0.07909437
1430831_at	LYR motif containing 1	Lyrm1	0.88783	-1.06014282	-0.0845095
1436195_at	cDNA sequence BC046404	BC046404	0.88783	1.07804442	0.09224194
1438872_at	tripartite motif family-like 1	Triml1	0.88783	-1.04092774	-0.058071
1457839_at	---	---	0.88784	1.06693051	0.08811961
1442621_at	expressed sequence C77798	C77798	0.88784	-1.05880737	-0.0976547
1430458_at	suppressor of Ty 6 homolog (S. cerevisiae)	Supt6h	0.88784	-1.04828985	-0.0719096
1418883_a_at	poly(A) binding protein, cytoplasmic 1	Pabpc1	0.88784	-1.02245364	-0.0331802
1458908_at	---	---	0.88784	-1.04876533	-0.0827688
1423257_at	cytochrome P450, family 4, subfamily a, polypeptide 14	Cyp4a14	0.88784	1.05565055	0.07666589
1452674_a_at	eukaryotic translation initiation factor 3, subunit K	Eif3k	0.88784	1.04627462	0.05905352
1456241_a_at	solute carrier family 38, member 10	Slc38a10	0.88784	1.052539	0.06928526
1453825_at	RIKEN cDNA 4933421O10 gene	4933421O10Rik	0.88784	1.05904859	0.08170922
1460187_at	secreted frizzled-related protein 1	Sfrp1	0.88785	-1.01137388	-0.2127756
1435624_at	RIKEN cDNA 9030409G11 gene	9030409G11Rik	0.88785	1.08196249	0.09978891
1438112_at	RIKEN cDNA 9430021M05 gene	9430021M05Rik	0.88785	-1.05544758	-0.0780806
1440448_at	---	---	0.88785	-1.05834424	-0.118203
1444684_at	RIKEN cDNA 4930539J05 gene	4930539J05Rik	0.88785	1.054274	0.07375428
1418706_at	solute carrier family 38, member 3	Slc38a3	0.88786	1.06546395	0.08349456
1460562_at	elongation factor Tu GTP binding domain containing 1	Eftud1	0.88786	1.04819855	0.06585338
1453195_at	serologically defined colon cancer antigen 3	Sdccag3	0.88786	1.04537969	0.06283423
1444464_at	---	---	0.88786	1.09410078	0.10657758
1431734_a_at	DnaJ (Hsp40) homolog, subfamily B, member 4	Dnajb4	0.88786	1.05924969	0.08080656
1437502_x_at	CD24a antigen	Cd24a	0.88786	1.10063608	0.10646383
1431353_at	poly(A) binding protein, cytoplasmic 4-like	Pabpc4l	0.88787	1.06484849	0.08691838
1422706_at	prostate transmembrane protein, androgen induced 1	Pmepa1	0.88787	1.04218594	0.05955571
1458432_at	---	---	0.88787	-1.04892182	-0.1066937
1458412_at	zinc finger protein 78	Zfp78	0.88787	1.08185779	0.09877514
1433266_at	RIKEN cDNA 2810416A17 gene	2810416A17Rik	0.88787	1.05622148	0.07701923
1440635_at	palladin, cytoskeletal associated protein	Palld	0.88787	1.06371894	0.08501138
1439921_at	predicted gene 3458	Gm3458	0.88788	-1.06056991	-0.0859614
1453562_a_at	NmrA-like family domain containing 1	Nmral1	0.88788	1.05630472	0.07297672
1450305_at	RIKEN cDNA 4930591A17 gene	4930591A17Rik	0.88788	-1.05881542	-0.087871
1450678_at	integrin beta 2	Itgb2	0.88788	-1.05955915	-0.0882632
1425439_a_at	solute carrier family 41, member 3	Slc41a3	0.88788	1.05362593	0.07237469
1437274_at	coatomer protein complex subunit alpha	Copa	0.88788	1.08139537	0.09924184
1432820_at	proteasome (prosome, macropain) 26S subunit, non-ATPase, 7	Psmd7	0.88789	1.06063274	0.0801679
1459919_a_at	MOB1, Mps One Binder kinase activator-like 2C (yeast)	Mobkl2c	0.8879	-1.05694372	-0.0829896
1434992_at	zinc finger RNA binding protein 2	Zfr2	0.8879	-1.05768186	-0.0826546
1451995_at	TAF11 RNA polymerase II, TATA box binding protein (TBP)-associa	Taf11	0.8879	1.04793754	0.06666965
1424811_at	camello-like 5	Cml5	0.8879	1.04778269	0.066541
1431059_x_at	HIV TAT specific factor 1	Htatsf1	0.8879	1.05937661	0.08098532
1448552_s_at	transmembrane protein 206	Tmem206	0.8879	1.05972117	0.08119985
1446356_at	---	---	0.8879	-1.05619376	-0.0804751
1422562_at	Ras-related associated with diabetes	Rrad	0.8879	-1.05869038	-0.0915855
1433527_at	iron responsive element binding protein 2	Ireb2	0.88791	-1.04185098	-0.0609375
1452188_at	mastermind like 1 (Drosophila)	Maml1	0.88791	1.05433289	0.07241788
1449496_at	RIKEN cDNA 2010109I03 gene	2010109I03Rik	0.88791	1.05517131	0.07635777
1434378_a_at	Max dimerization protein 4	Mxd4	0.88792	-1.05955547	-0.0873481
1433133_at	EDAR (ectodysplasin-A receptor)-associated death domain	Edaradd	0.88792	1.06403544	0.08532511
1425712_at	cDNA sequence BC025446	BC025446	0.88792	-1.04856043	-0.073667
1451606_at	RIKEN cDNA A530016L24 gene	A530016L24Rik	0.88792	1.06714597	0.08935422
1440463_at	RIKEN cDNA 5430414B19 gene	5430414B19Rik	0.88792	-1.05784311	-0.0815025
1439262_x_at	RIKEN cDNA 2310001A20 gene	2310001A20Rik	0.88792	-1.05379049	-0.0892285
1446235_at	adaptor-related protein complex 1 associated regulatory protein	Ap1ar	0.88792	-1.05764294	-0.0902573
1426125_a_at	caspase 9	Casp9	0.88792	1.07128542	0.08858585
1443271_at	---	---	0.88793	1.05371041	0.07105508
1448449_at	receptor-interacting serine-threonine kinase 3	Ripk3	0.88793	1.05253908	0.07005611
1455789_x_at	heat shock protein 8	Hspa8	0.88793	-1.02438234	-0.0370899
1440535_at	uroporphyrinogen III synthase	Uros	0.88793	-1.05249682	-0.0780736
1432508_at	RIKEN cDNA 1700113I22 gene	1700113I22Rik	0.88794	-1.06035835	-0.1283125
1440109_at	DNA segment, Chr 7, ERATO Doi 413, expressed	D7Ertd413e	0.88795	1.05900106	0.07616638
1457493_at	phosphatase and tensin homolog	Pten	0.88795	-1.06204746	-0.1025618
1435305_at	neurotrophic tyrosine kinase, receptor, type 2	Ntrk2	0.88795	-1.05628849	-0.0882211
1447414_at	---	---	0.88796	1.05731699	0.07881291
1433675_at	small nucleolar RNA host gene (non-protein coding) 1	Snhg1	0.88796	1.04809281	0.06303422
1437695_at	prokineticin receptor 2	Prokr2	0.88796	-1.05875077	-0.0835719
1450040_at	tissue inhibitor of metalloproteinase 2	Timp2	0.88796	-1.05797454	-0.0989648
1417279_at	inositol 1,4,5-triphosphate receptor 1	Itpr1	0.88796	1.05641599	0.07471512
1436591_at	V-set and immunoglobulin domain containing 10	Vsig10	0.88796	1.07008938	0.08663794
1453720_at	ring finger protein 157	Rnf157	0.88796	-1.05926095	-0.0876297
1433225_at	RIKEN cDNA 6330403N20 gene	6330403N20Rik	0.88797	1.06619955	0.08805313
1445619_at	---	---	0.88797	-1.05602027	-0.080187

1453585_at	RIKEN cDNA 1600010M07 gene	1600010M07Rik	0.88797	1.06723759	0.08793812
1449305_at	coagulation factor X	F10	0.88797	-1.06028182	-0.0877504
1431356_at	RIKEN cDNA 6430710C18 gene	6430710C18Rik	0.88797	1.04877384	0.06567635
1447845_s_at	vanin 1	Vnn1	0.88798	-1.05623834	-0.0906316
1429292_a_at	RIKEN cDNA 2310046K01 gene	2310046K01Rik	0.88798	-1.05019034	-0.0764193
1416925_at	karyopherin (importin) beta 1	Kpnb1	0.88798	-1.03993179	-0.0581994
1423321_at	myeloid-associated differentiation marker	Myadm	0.88798	1.06774266	0.07836827
1452205_x_at	predicted gene 6273 /// similar to T cell antigen receptor /// simil	Gm6273 /// LOC381765 /	0.88798	1.07168776	0.08838727
1437514_at	RIKEN cDNA B430306N03 gene	B430306N03Rik	0.88798	-1.0635244	-0.1125912
1457919_at	RIKEN cDNA D030054H15 gene	D030054H15Rik	0.88798	-1.05761914	-0.0863872
1421345_at	lecithin-retinol acyltransferase (phosphatidylcholine-retinol-O-acy	Lrat	0.88798	1.05177375	0.07236531
1452394_at	cysteinyl-tRNA synthetase	Cars	0.88798	-1.04196932	-0.0611581
1427897_s_at	suppressor of defective silencing 3 homolog (S. cerevisiae)	Suds3	0.88798	1.04205792	0.05752763
1443228_at	---	---	0.88799	-1.05536669	-0.0800416
1451015_at	transketolase	Tkt	0.88799	1.03608733	0.05103919
1433269_at	RIKEN cDNA 4930553M12 gene	4930553M12Rik	0.88799	-1.06011818	-0.1012443
1436864_at	---	---	0.88799	1.05817733	0.08125507
1460617_s_at	RAB6B, member RAS oncogene family	Rab6b	0.88799	-1.0582162	-0.0854051
1459149_at	---	---	0.88799	-1.05249291	-0.0796675
1456308_x_at	tripartite motif-containing 28	Trim28	0.88799	1.02833052	0.03852939
1455489_at	leucine rich repeat transmembrane neuronal 2	Lrrtm2	0.888	1.05989021	0.07864766
1433074_at	RIKEN cDNA 4931412I15 gene	4931412I15Rik	0.88801	1.06362382	0.0849968
1435055_a_at	target of myb1 homolog (chicken)	Tom1	0.88801	1.04246662	0.05858694
1441875_at	RIKEN cDNA 1700063J08 gene	1700063J08Rik	0.88801	-1.05151152	-0.0773276
1420365_a_at	heterogeneous nuclear ribonucleoprotein A2/B1	Hnrnpa2b1	0.88801	-1.02986701	-0.0424719
1416009_at	tetraspanin 3	Tspan3	0.88801	1.03973569	0.05570988
1426580_at	polo-like kinase 4 (Drosophila)	Plk4	0.88801	1.04951441	0.06690621
1450546_at	Alport syndrome, mental retardation, midface hypoplasia and ellip	Ammecr1	0.88802	-1.05621714	-0.0886544
1449808_at	---	---	0.88802	-1.04170806	-0.0590512
1439240_x_at	lin-7 homolog B (C. elegans)	Lin7b	0.88802	-1.052232	-0.080844
1454461_at	RIKEN cDNA 5830433M15 gene	5830433M15Rik	0.88803	1.09087564	0.10398145
1451011_at	zinc finger protein 358	Zfp358	0.88803	1.0559329	0.07548294
1420727_a_at	trimethyllysine hydroxylase, epsilon	Tmlhe	0.88803	-1.05308058	-0.0762196
1420595_at	distal-less homeobox 4	Dlx4	0.88803	1.09158602	0.10656332
1451494_at	WW domain containing adaptor with coiled-coil	Wac	0.88804	-1.05132646	-0.1137803
1444076_at	zinc finger protein 81	Zfp81	0.88804	1.08649694	0.09642411
1458644_at	coiled-coil domain containing 62	Ccdc62	0.88805	1.06745437	0.08651393
1433629_s_at	transmembrane protein 199	Tmem199	0.88805	1.0520836	0.0676956
1454966_at	integrin alpha 8	Itga8	0.88805	1.05958453	0.08175788
1450191_a_at	SRY-box containing gene 13	Sox13	0.88808	1.10057236	0.1042203
1450527_at	somatostatin receptor 1	Sstr1	0.88808	1.06564915	0.08666628
1458852_at	---	---	0.88808	1.06812955	0.0877763
1444186_at	alanine-glyoxylate aminotransferase 2-like 2	Agxt2l2	0.88809	-1.05900984	-0.084582
1416790_a_at	predicted gene 5806 /// thymine DNA glycosylase	Gm5806 /// Tdg	0.8881	-1.03992063	-0.060462
1429759_at	ribosomal protein S6 kinase polypeptide 6	Rps6ka6	0.88811	-1.0448325	-0.076013
1421173_at	interferon regulatory factor 4	Irf4	0.88812	1.06962445	0.08937203
1418984_at	InaD-like (Drosophila)	Inadl	0.88813	1.09201363	0.10466489
1444318_at	Coiled-coil-helix-coiled-coil-helix domain containing 7	Chchd7	0.88813	1.05996769	0.07766172
1449033_at	tumor necrosis factor receptor superfamily, member 11b (osteopr	Tnfrsf11b	0.88813	1.04125278	0.05568482
1451357_at	MPN domain containing	Mpnd	0.88814	1.03927203	0.05536978
1442749_at	Braf transforming gene	Braf	0.88814	-1.05840648	-0.0975567
1425668_a_at	ST3 beta-galactoside alpha-2,3-sialyltransferase 4	St3gal4	0.88814	-1.05729977	-0.0955052
1423178_at	abl-interactor 1	Abi1	0.88815	-1.05608702	-0.0894766
1426550_at	SID1 transmembrane family, member 1	Sidt1	0.88815	1.0699464	0.09111014
1457696_at	Rab interacting lysosomal protein	Rilp	0.88815	-1.05970451	-0.0864109
1448489_at	platelet-activating factor acetylhydrolase 2	Pafah2	0.88816	1.04799183	0.06545048
1426203_at	keratin associated protein 16-4	Krtap16-4	0.88816	1.06501379	0.08582943
1443482_at	predicted gene 1971	Gm1971	0.88816	-1.05832436	-0.086525
1443614_at	---	---	0.88816	-1.05529381	-0.0939946
1417901_a_at	islet cell autoantigen 1	Ica1	0.88817	1.05068593	0.06956778
1428314_at	PEST proteolytic signal containing nuclear protein	Pcnp	0.88817	-1.03800495	-0.0559078
1419117_at	solute carrier family 22 (organic cation transporter), member 2	Slc22a2	0.88819	-1.06002537	-0.0884255
1431415_a_at	TATA box binding protein-like 1	Tbpl1	0.88819	1.04384299	0.0598286
1452719_at	zinc finger, DHHC domain containing 24	Zdhhc24	0.8882	-1.05002969	-0.0755317
1457209_at	DDHD domain containing 2	Ddhd2	0.8882	-1.05989865	-0.0855975
1443911_at	---	---	0.88821	1.09302647	0.10495014
1436102_at	SEC22 vesicle trafficking protein homolog C (S. cerevisiae)	Sec22c	0.88822	-1.05237226	-0.0744868
1448221_at	HLA-B-associated transcript 1A	Bat1a	0.88822	1.03231508	0.04501306
1442715_at	---	---	0.88824	1.10040851	0.10756735
1433318_at	RIKEN cDNA 5430416B10 gene	5430416B10Rik	0.88824	-1.05882959	-0.1040578
1445051_at	---	---	0.88825	-1.04735903	-0.0671608
1432562_at	RIKEN cDNA 1110006G14 gene	1110006G14Rik	0.88825	1.06012828	0.08217367
1430225_at	exonuclease 1	Exo1	0.88825	1.06184048	0.08249221
1437898_at	RIKEN cDNA C920009B18 gene	C920009B18Rik	0.88826	1.05145219	0.07226253
1437641_at	Regulation of nuclear pre-mRNA domain containing 2	Rprd2	0.88826	-1.06007211	-0.1407596
1455711_at	deltex 4 homolog (Drosophila)	Dtx4	0.88827	1.05365926	0.07529045
1435301_at	RIKEN cDNA 1110004E09 gene	1110004E09Rik	0.88827	1.06375275	0.08150903
1451661_at	A kinase (PRKA) anchor protein 4	Akap4	0.88827	1.07170517	0.08991988
1459595_at	---	---	0.88827	1.07583407	0.09291572
1419344_at	t-complex-associated testis expressed 1	Tcte1	0.88828	1.06022657	0.08266532
1430435_at	AF4/FMR2 family, member 3	Aff3	0.88828	-1.05722722	-0.087629
1431264_at	---	---	0.88829	-1.04656419	-0.0723649
1457975_at	GTP binding protein 3	Gtpbp3	0.88829	-1.05767159	-0.0861168
1444020_at	---	---	0.88829	1.09061731	0.10362423
1428346_at	TRAF type zinc finger domain containing 1	Trafd1	0.88829	1.05828796	0.07565121
1438636_s_at	sushi domain containing 5	Susd5	0.88829	1.05309364	0.07384607
1456734_at	thioredoxin 1	Txn1	0.88829	1.08989882	0.1008179
1422877_at	protocadherin beta 12	Pcdhb12	0.8883	-1.05506684	-0.0857395
1417547_at	squamous cell carcinoma antigen recognized by T-cells 3	Sart3	0.8883	1.04036221	0.05672654
1416104_at	mannose-P-dolichol utilization defect 1	Mpdu1	0.88831	1.0537467	0.06962367
1449901_a_at	mitogen-activated protein kinase kinase kinase 6	Map3k6	0.88832	1.07621837	0.09348255
1446747_at	sperm flagellar 2	Spef2	0.88832	1.05945153	0.08062691
1446250_at	---	---	0.88832	1.05281047	0.07231807
1416455_a_at	crystallin, alpha B	Cryab	0.88832	1.05348608	0.07409582

1440131_at	---	---	0.88832	-1.05587787	-0.0802593
1448410_at	ubiquitination factor E4B, UFD2 homolog (S. cerevisiae)	Ube4b	0.88833	1.04370468	0.06170065
1428023_at	RIKEN cDNA 3110009E18 gene	3110009E18Rik	0.88833	1.07233592	0.08584703
1417837_at	pleckstrin homology-like domain, family A, member 2	Phlda2	0.88834	1.04319594	0.05857524
1420576_at	lysophosphatidic acid receptor 2	Lpar2	0.88834	-1.05051154	-0.0741406
1421618_at	myosin IF	Myo1f	0.88835	-1.03790364	-0.0548365
1422228_at	wingless-related MMTV integration site 8A	Wnt8a	0.88835	1.08332563	0.1016695
1429283_at	CDGSH iron sulfur domain 2	Cisd2	0.88835	-1.05352963	-0.0839825
1432588_at	RIKEN cDNA 5830468K08 gene	5830468K08Rik	0.88835	1.0614604	0.08189964
1423320_at	deoxyribonuclease 1-like 2	Dnase1l2	0.88835	-1.05703263	-0.0829625
1432807_at	RIKEN cDNA 5830426K05 gene	5830426K05Rik	0.88835	1.06085339	0.08161312
1458073_at	RIKEN cDNA 9330156P08 gene	9330156P08Rik	0.88835	-1.05894245	-0.0935985
1455075_at	phosphatidylinositol glycan anchor biosynthesis, class V	Pigv	0.88835	1.07188796	0.08974431
1437231_at	SLIT and NTRK-like family, member 6	Slitrk6	0.88835	1.06451596	0.07850033
1456542_s_at	glutaminyl-tRNA synthase (glutamine-hydrolyzing)-like 1	Qrsl1	0.88835	-1.04922909	-0.0810978
1435106_at	LIM and calponin homology domains 1	Limch1	0.88835	-1.05126524	-0.0735863
1421886_at	son of sevenless homolog 1 (Drosophila)	Sos1	0.88836	-1.05336116	-0.13292
1425955_at	caveolin 2	Cav2	0.88836	-1.05578537	-0.0807357
1429641_x_at	late cornified envelope 3B	Lce3b	0.88836	-1.0541254	-0.0814429
1450926_at	ATPase, H+ transporting, lysosomal V1 subunit G1	Atp6v1g1	0.88836	1.06719402	0.08812478
1427018_at	translin-associated factor X (Tsnax) interacting protein 1	Tsnaxip1	0.88836	-1.0598055	-0.0845289
1423770_at	transmembrane channel-like gene family 6	Tmc6	0.88837	1.0575156	0.07577767
1445612_at	---	---	0.88837	1.10708087	0.11528361
1449525_at	flavin containing monooxygenase 3	Fmo3	0.88838	-1.05656483	-0.08816
1455363_at	brain-specific angiogenesis inhibitor 1	Bai1	0.88838	1.06364924	0.08146717
1453478_at	hypothetical protein LOC100503762	LOC100503762	0.88839	1.10151812	0.10884026
1418794_at	CDP-diacylglycerol synthase (phosphatidate cytidylyltransferase)	Cds2	0.88839	1.05185932	0.06983172
1436481_at	ATPase, Cu++ transporting, beta polypeptide	Atp7b	0.88839	1.06860862	0.08863259
1419176_at	vacuolar protein sorting 37A (yeast)	Vps37a	0.88839	1.05742587	0.07602161
1431933_a_at	DCP1 decapping enzyme homolog A (S. cerevisiae)	Dcp1a	0.8884	1.12510542	0.12597662
1431541_at	predicted gene 9757	Gm9757	0.88841	-1.05924149	-0.0951965
1454397_at	RIKEN cDNA 4632418H02 gene	4632418H02Rik	0.88841	1.07040672	0.08809904
1440562_at	hypothetical LOC100504312	LOC100504312	0.88841	-1.06113812	-0.0915603
1459030_at	butyrobetaine (gamma), 2-oxoglutarate dioxygenase 1 (gamma-	Bbox1	0.88842	-1.05778373	-0.0813501
1416319_at	adenosine kinase	Adk	0.88843	-1.04578109	-0.067292
1458542_at	---	---	0.88844	-1.05564316	-0.0812011
1423639_at	histamine receptor H2	Hrh2	0.88845	-1.05632442	-0.0897729
1434076_at	WD repeat domain 37	Wdr37	0.88845	1.0422416	0.05905316
1440175_at	Epsin 2	Epn2	0.88846	1.05259086	0.07150753
1445964_at	---	---	0.88847	1.051442	0.07207132
1451895_a_at	24-dehydrocholesterol reductase	Dhcr24	0.88849	1.03930646	0.05520428
1418021_at	complement component 4B (Childo blood group)	C4b	0.88849	-1.05421972	-0.093266
1454165_at	RIKEN cDNA 1700025K04 gene	1700025K04Rik	0.88849	-1.05325803	-0.0786812
1427778_at	defensin beta 8	Defb8	0.8885	1.09854802	0.10645586
1451131_at	ADP-ribosylation factor-like 6 interacting protein 1	Arl6ip1	0.8885	1.04219886	0.05751817
1444404_at	ring finger protein 207	Rnf207	0.8885	1.08202393	0.09729829
1422921_at	pre-B lymphocyte gene 3	Vpreb3	0.8885	1.058829	0.07864198
1438055_at	retinoic acid receptor responder (tazarotene induced) 1	Rarres1	0.8885	1.05224722	0.07304743
1442402_at	SH3 domain containing ring finger 1	Sh3rf1	0.88851	-1.05835567	-0.0906944
1436292_a_at	ornithine decarboxylase antizyme 1	Oaz1	0.88852	-1.02697952	-0.0388705
1420079_at	vomeronasal 2, receptor 95 /// vomeronasal 2, receptor 96 /// vo	Vmn2r95 /// Vmn2r96 ///	0.88852	-1.04942247	-0.0847301
1415691_at	discs, large homolog 1 (Drosophila)	Dlg1	0.88852	1.04098487	0.05721309
1426477_at	RAS p21 protein activator 1	Rasa1	0.88852	-1.04405808	-0.062456
1439516_at	RIKEN cDNA 2610201A13 gene	2610201A13Rik	0.88852	1.04421379	0.06125524
1429542_at	RIKEN cDNA 1700007B14 gene	1700007B14Rik	0.88853	1.05235318	0.0696012
1425040_at	cytochrome b reductase 1	Cybrd1	0.88853	-1.05718387	-0.0891499
1436597_at	ankyrin repeat and KH domain containing 1	Ankhd1	0.88853	1.05468842	0.07298399
1420153_at	---	---	0.88853	-1.05117507	-0.0792747
1437636_at	RIKEN cDNA E030037K03 gene	E030037K03Rik	0.88854	1.07771307	0.0953406
1457940_at	---	---	0.88855	1.05931012	0.08155735
1418946_at	ST3 beta-galactoside alpha-2,3-sialyltransferase 1	St3gal1	0.88855	-1.05352769	-0.0754611
1449289_a_at	beta-2 microglobulin	B2m	0.88855	-1.0486522	-0.0709885
1451057_x_at	dynamin 2	Dnm2	0.88856	-1.03992254	-0.0566296
1416150_a_at	serine/arginine-rich splicing factor 3	Srsf3	0.88856	-1.03198458	-0.0470965
1444538_at	---	---	0.88857	-1.05398563	-0.0873661
1419566_at	fibronectin type 3 and ankyrin repeat domains 1	Fank1	0.88857	-1.05537319	-0.0809612
1416089_at	ribosomal protein S15	Rps15	0.88857	1.02656789	0.03586972
1440564_at	prokineticin receptor 2	Prokr2	0.88859	-1.06039095	-0.0945013
1421670_a_at	interleukin-1 receptor-associated kinase 4	Irak4	0.88859	1.05823013	0.07965691
1446094_at	---	---	0.8886	1.05386641	0.074773
1452292_at	adaptor-related protein complex 2, beta 1 subunit	Ap2b1	0.8886	1.03945043	0.0557819
1422224_at	predicted gene 9880 /// t-complex protein 10a /// t-complex prot	Gm9880 /// Tcp10a /// Tc	0.8886	-1.05674056	-0.0811179
1425252_a_at	mitotic arrest deficient 1-like 1	Mad1l1	0.88862	1.05368329	0.06904518
1443628_at	---	---	0.88862	-1.05948123	-0.0867377
1432638_at	BCL2-associated athanogene 5	Bag5	0.88863	1.06502826	0.08513914
1423073_at	cytidine monophosphate (UMP-CMP) kinase 1	Cmpk1	0.88863	1.05537423	0.06954865
1416663_at	NADH dehydrogenase (ubiquinone) 1 alpha subcomplex, 9	Ndufa9	0.88863	1.04051692	0.05589676
1456663_x_at	TM2 domain containing 2	Tm2d2	0.88864	1.05580582	0.07536276
1460192_at	oxysterol binding protein-like 1A	Osbpl1a	0.88864	1.0424221	0.05908797
1444691_at	protein kinase D3	Prkd3	0.88864	-1.0469099	-0.0674287
1433768_at	palladin, cytoskeletal associated protein	Palld	0.88865	-1.04967544	-0.0813775
1435525_at	potassium channel tetramerisation domain containing 17	Kctd17	0.88866	1.06130269	0.0769263
1420448_at	reproductive homeobox 2A	Rhox2a	0.88866	1.05575858	0.07813089
1432089_at	RIKEN cDNA 4930550C17 gene	4930550C17Rik	0.88868	-1.05340334	-0.0754105
1455802_x_at	anterior gradient 2 (Xenopus laevis)	Agr2	0.8887	1.04875235	0.06453204
1450683_at	transgelin 3	Tagln3	0.8887	1.05461953	0.07514722
1427344_s_at	RASD family, member 2	Rasd2	0.88872	1.07666859	0.09153604
1442560_at	---	---	0.88875	-1.05798021	-0.0855737
1417450_a_at	transforming, acidic coiled-coil containing protein 3	Tacc3	0.88876	1.04397385	0.05929197
1451771_at	two pore channel 1	Tpcn1	0.88877	-1.05831391	-0.1271093
1424559_at	RNA polymerase II associated protein 2	Rpap2	0.88888	1.04876843	0.06615639
1420752_at	deltex 3 homolog (Drosophila)	Dtx3	0.8889	1.04914453	0.06524175
1435212_at	purinergic receptor P2X, ligand-gated ion channel, 2	P2rx2	0.88892	1.06502951	0.08596203
1424629_at	breast cancer 1	Brca1	0.88892	1.05387223	0.06899663

1456920_at	---	---	0.88894	1.07267009	0.09238208
1455630_at	dynein, axonemal, light chain 1	Dnalc1	0.88894	1.05408434	0.07463963
1422810_at	zinc finger protein 191	Zfp191	0.88896	-1.05228685	-0.0907701
1421736_at	vomeronasal 1 receptor 56	Vmn1r56	0.88896	-1.06185141	-0.0929657
1416680_at	ubiquitin protein ligase E3A	Ube3a	0.88913	1.03650877	0.05136063
1415849_s_at	stathmin 1	Stmn1	0.88914	-1.02536654	-0.036358
1420151_at	hypothetical LOC100504636	LOC100504636	0.88915	1.04765189	0.06615194
1417224_a_at	CD2 antigen (cytoplasmic tail) binding protein 2	Cd2bp2	0.88916	-1.04809791	-0.0816479
1418789_at	syntrophin, gamma 2	Sntg2	0.88919	1.06431449	0.08638788
1426469_a_at	TATA box binding protein	Tbp	0.88922	-1.03791321	-0.0545084
1451253_at	PX domain containing serine/threonine kinase	Pxk	0.88922	1.06444677	0.08201973
1435385_at	teashirt zinc finger family member 2	Tshz2	0.88925	1.06143344	0.08354085
1429677_at	RIKEN cDNA 1700056E22 gene	1700056E22Rik	0.88926	-1.0542889	-0.0773604
1436459_at	---	---	0.88938	1.13784749	0.13524739
1444703_at	RIKEN cDNA 2810403D21 gene	2810403D21Rik	0.8894	-1.05498477	-0.0794975
1422781_at	toll-like receptor 3	Tlr3	0.8894	-1.04983166	-0.076011
1416589_at	secreted acidic cysteine rich glycoprotein	Sparc	0.88941	1.0488083	0.06329353
1460552_at	small nuclear ribonucleoprotein 200 (U5)	Snrnp200	0.88941	-1.04257237	-0.0700553
1439986_at	diacylglycerol kinase, iota	Dgki	0.88941	1.06048163	0.08263873
1428898_at	MON1 homolog A (yeast)	Mon1a	0.88942	1.04918811	0.06796739
1428143_a_at	patatin-like phospholipase domain containing 2	Pnpla2	0.88943	1.05164762	0.0681531
1444224_at	---	---	0.88943	-1.05808448	-0.0863287
1445478_at	---	---	0.88945	-1.0605135	-0.103835
1425887_at	transmembrane protein 114	Tmem114	0.88945	-1.05709771	-0.0808784
1451577_at	zinc finger and BTB domain containing 20	Zbtb20	0.88946	1.05225304	0.0725174
1457377_at	---	---	0.88948	1.06736887	0.08706162
1439318_at	microtubule associated serine/threonine kinase family member 4	Mast4	0.8895	-1.05521056	-0.0800773
1432623_at	suppressor of var1, 3-like 1 (S. cerevisiae)	Supv3l1	0.8895	1.05103609	0.06885587
1417105_at	trafficking protein particle complex 2-like	Trappc2l	0.88951	1.04966703	0.06409771
1431390_a_at	glutamate receptor, ionotropic, N-methyl D-aspartate-like 1A	Grinl1a	0.88951	1.03650284	0.05147576
1434895_s_at	protein phosphatase 1, regulatory (inhibitor) subunit 13B	Ppp1r13b	0.88951	1.04392033	0.06176763
1420679_a_at	androgen-induced 1	Aig1	0.88952	1.05179976	0.07285151
1436961_at	heat shock protein 12A	Hspa12a	0.88953	1.06006108	0.08267156
1416738_at	BRCA1 associated protein	Brap	0.88954	1.04722291	0.06524206
1455753_at	family with sequence similarity 163, member B	Fam163b	0.88954	-1.05874984	-0.0869325
1447985_s_at	ankyrin repeat and IBR domain containing 1	Ankib1	0.88955	-1.05502251	-0.0780421
1423685_at	alanyl-tRNA synthetase	Aars	0.88956	1.03265388	0.04622119
1453946_a_at	serologically defined colon cancer antigen 8	Sdccag8	0.88957	-1.06042463	-0.1008616
1436606_at	Zinc finger protein 498	Zfp498	0.88958	1.04750316	0.06526001
1434979_at	RIKEN cDNA 4933403F05 gene	4933403F05Rik	0.88959	1.05552858	0.07169976
1429978_at	antagonist of mitotic exit network 1 homolog (S. cerevisiae)	Amn1	0.88959	-1.03843273	-0.0563561
1441757_at	RIKEN cDNA 1190002F15 gene	1190002F15Rik	0.8896	-1.05742412	-0.0919141
1426410_at	pyruvate dehydrogenase kinase, isoenzyme 3	Pdk3	0.88962	1.04147471	0.05742478
1442066_at	RIKEN cDNA C530028O21 gene	C530028O21Rik	0.88962	1.07064766	0.08715204
1426917_s_at	secernin 3	Scrn3	0.88963	-1.05266199	-0.0817498
1458315_at	enhancer of polycomb homolog 1 (Drosophila)	Epc1	0.88963	1.08846695	0.09810106
1419901_at	predicted gene 11110	Gm11110	0.88965	1.05948213	0.08079116
1426302_at	transmembrane protease, serine 4	Tmprss4	0.88966	-1.05918752	-0.0973318
1441466_at	---	---	0.88972	-1.05325152	-0.0748996
1459736_at	---	---	0.88973	1.06953292	0.08800563
1425622_at	EGF-like repeats and discoidin I-like domains 3	Edil3	0.88974	-1.0571556	-0.0929033
1416910_at	DnaJ (Hsp40) homolog, subfamily C, member 15	Dnajc15	0.88995	1.05406432	0.06725801
1422085_at	T-box 19	Tbx19	0.88998	-1.05590547	-0.0808781
1452939_a_at	phosphatidylinositol transfer protein, cytoplasmic 1	Pitpnc1	0.89001	-1.05529575	-0.1010443
1429215_at	RIKEN cDNA 2310058N22 gene	2310058N22Rik	0.89002	-1.05693041	-0.0812295
1434154_at	potassium channel tetramerisation domain containing 13	Kctd13	0.89004	1.05443197	0.0725853
1420081_s_at	DNA segment, Chr 2, ERATO Doi 750, expressed	D2Ertd750e	0.8903	1.04898373	0.06597833
1444035_at	mesoderm development candidate 2	Mesdc2	0.89031	-1.06148848	-0.1220407
1438727_at	DDB1 and CUL4 associated factor 10	Dcaf10	0.89035	1.06509666	0.08277021
1457773_at	SLAM family member 6	Slamf6	0.89037	1.05945956	0.08150785
1458242_at	---	---	0.89039	1.05023555	0.06741116
1424922_a_at	bromodomain containing 4	Brd4	0.8904	-1.03919146	-0.1500423
1457091_at	RIKEN cDNA 4930526D03 gene	4930526D03Rik	0.89041	1.05522587	0.07462969
1429384_at	casein kinase 1, gamma 3	Csnk1g3	0.89041	-1.04376651	-0.062371
1457115_at	predicted gene 10643	Gm10643	0.89043	1.06732343	0.09073477
1438867_at	predicted gene 1070	Gm1070	0.89044	1.07223275	0.09260496
1452536_s_at	predicted gene 5571 /// immunoglobulin kappa chain variable 1 (	Gm5571 /// Igk-V1	0.89052	-1.05646808	-0.0822256
1450216_at	protocadherin beta 16	Pcdhb16	0.89052	1.09527819	0.10378989
1447589_at	---	---	0.89063	-1.05759198	-0.0833211
1428321_at	echinoderm microtubule associated protein like 1	Eml1	0.89068	1.06545924	0.08407913
1445930_at	fibronectin type III domain containing 7	Fndc7	0.89069	1.05513381	0.07348574
1421363_at	cytochrome P450, family 2, subfamily c, polypeptide 39	Cyp2c39	0.89069	-1.05703141	-0.0813434
1424601_at	DNA repair protein XRCC4-like /// X-ray repair complementing de	LOC100044170 /// Xrcc4	0.89073	1.05295136	0.07262832
1416134_at	amyloid beta (A4) precursor-like protein 1	Aplp1	0.89073	1.04491977	0.06110606
1436711_at	ARP5 actin-related protein 5 homolog (yeast)	Actr5	0.89075	1.05139119	0.06745701
1456118_at	methyltransferase like 2	Mettl2	0.89076	-1.04377729	-0.0677137
1443168_at	---	---	0.89076	1.06780698	0.08511618
1432744_at	RIKEN cDNA 4930443G03 gene	4930443G03Rik	0.89076	-1.05598847	-0.0795045
1422794_at	cullin 3	Cul3	0.89077	-1.0376669	-0.056092
1452316_at	integrator complex subunit 12	Ints12	0.89078	1.04332718	0.06095756
1440321_at	Microtubule associated serine/threonine kinase 2	Mast2	0.89078	-1.05518538	-0.0779738
1430472_at	armadillo repeat containing 1	Armc1	0.89078	1.08653562	0.0991959
1433576_at	methionine adenosyltransferase II, alpha	Mat2a	0.8908	-1.03894776	-0.0558889
1426650_at	myosin, heavy polypeptide 8, skeletal muscle, perinatal	Myh8	0.89083	1.05423071	0.07456031
1449629_s_at	---	---	0.89084	1.0622789	0.0796474
1441817_at	RIKEN cDNA 1700099I09 gene	1700099I09Rik	0.89085	-1.04385133	-0.065708
1439396_x_at	glycerol-3-phosphate dehydrogenase 1 (soluble)	Gpd1	0.89086	1.08258541	0.09935155
1434282_at	inhibitor of Bruton agammaglobulinemia tyrosine kinase	Ibtk	0.89086	-1.05044649	-0.076657
1417893_at	sideroflexin 3	Sfxn3	0.89086	-1.05083219	-0.0740222
1456125_a_at	dynein light chain LC8-type 1 /// predicted gene 6788	Dynll1 /// Gm6788	0.89087	-1.03349079	-0.0561656
1429363_at	vacuolar protein sorting 37A (yeast)	Vps37a	0.89088	-1.05110647	-0.0764196
1434769_at	BTB (POZ) domain containing 9	Btbd9	0.89088	1.05225781	0.07087305
1418508_a_at	growth factor receptor bound protein 2	Grb2	0.89088	-1.04670718	-0.0806421
1456821_at	---	---	0.89088	-1.05487568	-0.0816807

1455037_at	plexin A2	Plxna2	0.89089	1.07464123	0.09360018
1436065_at	chemokine (C-C motif) receptor 9	Ccr9	0.89089	-1.05622228	-0.0833957
1453911_at	---	---	0.89089	1.06423709	0.08446891
1427124_at	RIKEN cDNA 4932416H05 gene	4932416H05Rik	0.89089	-1.05922865	-0.1044844
1431445_at	family with sequence similarity 47, member A, pseudogene	Fam47a-ps	0.8909	-1.04411	-0.0670055
1431817_at	alcohol dehydrogenase 6 (class V), pseudogene 1	Adh6-ps1	0.8909	1.0535158	0.07449076
1445294_at	---	---	0.8909	1.05709331	0.07512839
1444150_at	erythrocyte protein band 4.1	Epb4.1	0.89091	-1.05515549	-0.0825321
1442429_at	---	---	0.89091	-1.05787891	-0.0911969
1422144_at	inhibin beta E	Inhbe	0.89091	-1.05693793	-0.0820377
1421580_at	contactin associated protein-like 1	Cntnap1	0.89092	1.06485891	0.08461169
1446400_at	olfactory receptor 558	Olfr558	0.89093	1.06603814	0.08621323
1455729_at	guanine nucleotide binding protein, alpha q polypeptide	Gnaq	0.89094	-1.04906678	-0.069753
1436026_at	zinc finger protein 703	Zfp703	0.89095	-1.05438501	-0.0779372
1428533_at	DNA segment, Chr 1, Brigham & Women's Genetics 0212 express	D1Bwg0212e	0.89095	1.03811527	0.05388867
1421513_at	tudor domain containing 1	Tdrd1	0.89095	-1.05885922	-0.0893105
1454982_at	ADP-ribosylation factor guanine nucleotide-exchange factor 2 (bre	Arfgef2	0.89096	1.04669466	0.06354801
1454111_at	RIKEN cDNA 4930404A10 gene	4930404A10Rik	0.89097	-1.05711986	-0.0916099
1443429_at	---	---	0.89097	-1.05601973	-0.0809955
1429683_at	tocopherol (alpha) transfer protein-like	Ttpal	0.89098	1.06265276	0.08296333
1419388_at	transmembrane 4 L six family member 20	Tm4sf20	0.89098	-1.05225402	-0.0828185
1453439_at	amelotin	Amtn	0.89099	1.07156341	0.08869029
1418901_at	CCAAT/enhancer binding protein (C/EBP), beta	Cebpb	0.891	-1.05091876	-0.1335731
1455007_s_at	glutamic pyruvate transaminase (alanine aminotransferase) 2	Gpt2	0.89102	-1.04860166	-0.0866035
1435034_at	RNA polymerase II associated protein 2	Rpap2	0.89102	-1.06040525	-0.0970794
1420506_a_at	syntaxin binding protein 1	Stxbp1	0.89102	-1.05912306	-0.0899235
1458618_at	---	---	0.89102	1.0733203	0.09031767
1454935_at	fat storage-inducing transmembrane protein 2	Fitm2	0.89104	1.05172112	0.07149957
1425484_at	thymocyte selection-associated high mobility group box	Tox	0.89104	1.07549543	0.0883793
1459794_at	---	---	0.89104	1.05707086	0.07883677
1451410_a_at	cysteine-rich protein 3	Crip3	0.89105	1.06536484	0.08620259
1426154_s_at	novel member of the major urinary protein (Mup) gene family ///	LOC100048884 /// Mup1	0.89105	1.05172717	0.07029449
1446979_at	Decapping enzyme, scavenger	Dcps	0.89106	-1.05880955	-0.0873418
1444727_at	RIKEN cDNA 2310042D19 gene	2310042D19Rik	0.89106	-1.05663788	-0.0849048
1435324_x_at	high mobility group box 1	Hmgb1	0.89107	-1.03009772	-0.0430305
1456534_at	Mannosidase, alpha, class 1A, member 2	Man1a2	0.89107	-1.05662864	-0.0827853
1429799_at	zinc finger protein 606	Zfp606	0.89107	-1.06036537	-0.1106877
1426547_at	group specific component	Gc	0.89108	1.05654159	0.07862102
1437421_at	RIKEN cDNA 6330509M05 gene	6330509M05Rik	0.89108	-1.05918604	-0.0883081
1436156_at	cell division cycle and apoptosis regulator 1	Ccar1	0.89108	-1.03817081	-0.0551307
1422527_at	histocompatibility 2, class II, locus DMa	H2-DMa	0.89108	1.05579836	0.07708995
1424295_at	developmental pluripotency-associated 3	Dppa3	0.89108	1.05165342	0.07229992
1434254_at	guanine nucleotide binding protein, alpha 11	Gna11	0.8911	1.04477878	0.06271763
1427248_at	Wolf-Hirschhorn syndrome candidate 2 (human)	Whsc2	0.8911	1.03723236	0.05242477
1458523_at	zinc finger, matrin-like	Zfml	0.8911	1.07848353	0.09688345
1427431_at	---	---	0.89111	-1.06130859	-0.1048272
1453535_at	olfactory receptor 78	Olfr78	0.89112	-1.05433108	-0.0817647
1420578_at	opticin	Optc	0.89112	-1.05781463	-0.0956616
1424230_at	exocyst complex component 6	Exoc6	0.89112	-1.05417348	-0.0925614
1423074_at	lectin, mannose-binding 2	Lman2	0.89112	1.04593634	0.06453002
1455237_at	ubiquitin specific peptidase 36	Usp36	0.89112	-1.04476874	-0.0686267
1416375_at	adaptor-related protein complex 3, mu 1 subunit	Ap3m1	0.89112	1.05067089	0.07098007
1442777_at	---	---	0.89113	1.05314367	0.0688619
1442984_at	expressed sequence C80060	C80060	0.89113	-1.05681696	-0.0897727
1443095_at	---	---	0.89113	-1.05477833	-0.0819275
1423976_at	RIKEN cDNA 4930453N24 gene	4930453N24Rik	0.89113	-1.03826676	-0.0556406
1449473_s_at	CD40 antigen	Cd40	0.89114	1.07788521	0.09494394
[truncated: 2,279,422 more chars]
